# Frequency and content analysis of adverse event reports in surgical centers: a cross-sectional study

**DOI:** 10.1590/0034-7167-2024-0082

**Published:** 2025-03-14

**Authors:** Ítalo Lennon Sales de Almeida, Ana Paula Agostinho Alencar, Vanessa de Brito Poveda, Rhanna Emanuela Fontenele Lima de Carvalho

**Affiliations:** I Universidade Estadual do Ceará. Fortaleza, Ceará, Brazil; II Universidade de São Paulo. São Paulo, São Paulo, Brazil

**Keywords:** Patient Safety, Notification, Adverse Events, Surgical Centers, Operative Surgical Procedures, Seguridad del Paciente, Notificación, Eventos Adversos, Centros Quirúrgicos, Procedimientos Quirúrgicos Operatorios

## Abstract

**Objectives:**

to identify the frequency of adverse events reported in surgical centers and analyze the content of the reports made.

**Methods:**

a cross-sectional study analyzed reports from January 2019 to March 2023 in eight hospitals, with a sample of 163 reports. The quantitative analysis considered variables such as type and degree of damage, while the qualitative analysis used similarity analysis in Iramuteq.

**Results:**

sixteen never events were identified, including pressure injuries (stages three and four), loss of biopsy material, incorrect surgical procedures, and unintentional retention of a foreign body. The qualitative analysis highlighted terms such as “failures in care”, “failure”, “surgical procedure”, and “pressure injury”.

**Conclusions:**

pressure injuries, burns, organ damage, and surgical site infection were the most frequent reports. There is underreporting of adverse events in surgical centers and limitations in the quality of records, including unspecified adverse events and lack of standardization in reporting.

## RESUMEN

**Objetivos:**

identificar la frecuencia de eventos adversos notificados en centros quirúrgicos y analizar el contenido de dichas notificaciones.

**Métodos:**

es un estudio transversal llevado a cabo en ocho hospitales con una muestra de 163 notificaciones, entre enero de 2019 y marzo de 2023. El análisis cuantitativo sopesó variables de clase y grado del daño, y el cualitativo utilizó el análisis de similitud, en Iramuteq.

**Resultados:**

se identificaron dieciséis “*never events*”, entre ellos lesiones por presión (fases tres y cuatro), pérdida de material de biopsia, procedimientos quirúrgicos incorrectos y retención involuntaria de cuerpo extraño. El análisis cualitativo puso de relieve términos como “errores asistenciales”, “errores”, “procedimiento quirúrgico” y “lesión por presión”.

**Conclusiones:**

lesiones por presión, quemaduras, lesión de órgano e infección de sitio quirúrgico fueron las notificaciones más comunes. Hay subnotificación de eventos adversos no especificados en los centros quirúrgicos y reticencias en la calidad y estandarización de los informes.

## INTRODUCTION

Harm caused by unsafe and therefore avoidable care can lead to death, disability and cause significant financial and economic impacts for healthcare institutions and healthcare systems. Therefore, this is a global challenge^(1)^. Harm in healthcare leads to a reduction in people’s trust in healthcare systems and in the work of professionals, who can also suffer psychological harm when involved in serious events with permanent harm or death to their patients^(1)^.

In Brazil, since the launch of the National Patient Safety Program (NPSC) in 2013, several actions have been implemented to improve care. This included the creation of Patient Safety Centers (PSC) and safety protocols in healthcare facilities. Other measures involved the participation of patients and family members in care, increasing society’s access to information on patient safety, producing and disseminating knowledge on the subject, and promoting the inclusion of the subject in training courses in the healthcare area^(2)^.

The second Global Patient Safety Challenge focuses on surgery safety, integrating actions proposed by the World Health Organization with essential protocols encouraged by the Brazilian Ministry of Health for health risk management. This approach is motivated by the incidence of adverse events, which reaches 16% in surgical centers, according to the WHO, and can reach 20%, according to Brazilian studies, in relation to surgical procedures performed^(3-6)^.

Reporting adverse events is what makes it possible to identify the moments in the provision of health care when most incidents occur. Therefore, notifications provide support for the development of risk management strategies to prevent the occurrence of failures in care^(7,8)^.

Notivisa (National Health Surveillance Notification System), developed by the National Health Surveillance Agency (Anvisa), is the national system that allows the notification of adverse events in health care. The system allows health professionals, managers and the general population to access it to report the occurrence of incidents^(9)^.

Thus, adverse events are defined as incidents that cause harm to patients during hospitalization or care in healthcare facilities or during the use of healthcare technologies. They include undesirable effects of medications in usual therapeutic doses, technical complaints of healthcare products, and surgical incidents, most of which could be prevented. Adverse events that cause serious harm and expose patients to the risk of death or generate irreparable damage are classified as never events, such as surgery on the wrong patient or on the wrong limb, loss of a biopsy specimen, retention of a foreign body in a patient after surgery, among others^(9)^.

The culture of reporting adverse events in Brazil is an important point of discussion, considering that healthcare institutions still encounter barriers and limitations in the act of reporting. The punitive culture and the fear of acknowledging errors are still realities that bring challenges to managers and leaders in healthcare institutions^(10,11)^.

In addition to the challenge of ensuring universal access to surgical procedures in the Brazilian health system^(12)^, it is important to highlight that adherence to surgical safety protocols is still incipient or presents significant flaws in their implementation. This highlights the need to develop improvement strategies to promote a more robust culture of surgical safety in health facilities^(13,14)^.

Therefore, the analysis of adverse events reported by surgical centers should be used as a way to support decision-making in the management of surgical care with a focus on improving the safety and quality of care offered to patients requiring surgical interventions.

In Brazil, the analysis of adverse events in surgical centers has focused on the review of medical records^(5-7)^, therefore, the content of the reports made in Notivisa is unknown and still requires analysis. Thus, the study may indicate which adverse events are most frequently reported, their characteristics and how reporting professionals report these events in the reporting process, contributing to the area’s knowledge regarding how the national reporting system for adverse events related to healthcare has been used and the strengths and weaknesses of the reporting process.

The characteristics of the reported events should indicate the direction that institutional protocols should follow in order to create prevention strategies. It is understood that by analyzing the content of the reports, it will be possible to devise strategies to improve their quality in reporting institutions.

## OBJECTIVES

To identify the frequency of adverse events reported in surgical centers and analyze the content of the reports made.

## METHODS

### Ethical aspects

The research project was assessed and approved by the Research Ethics Committee of the State University of Ceará. After the ethics approval, the Ceará State Health Department (SESA-CE) obtained permission to access the Notivisa data, and an alignment meeting was held with the Health Surveillance Coordination Office of SESA-CE (COVIS) to present the research project and to align the way in which the system’s database would be made available by the technical team responsible.

The database with all the data and variables available in Notivisa was then sent to the personal email of the researcher responsible for the study. However, in order to guarantee data confidentiality, a version of the database was created for data analysis that did not contain identifying data of the reporting professional and did not allow identification of the institution where the event occurred, since they were coded. For the presentation of the data, the names of the institutions were coded by letters (Hospital A, Hospital B, Hospital C, etc.).

### Study design, period and setting

Cross-sectional study conducted with data on adverse events reported in surgical centers from January 2019 to March 2023 in eight tertiary hospitals under state management, located in a state in the Northeast region of Brazil. The study followed the recommendations of the Strengthening the Reporting of Observational Studies in Epidemiology (STROBE) for reporting observational studies.

### Population or sample; inclusion and exclusion criteria

The reports were extracted from the Brazilian system for reporting adverse events in healthcare (Notivisa). The study sample consisted of reports of adverse events related to healthcare carried out by surgical centers in the institutions. No exclusion criteria were defined, and all reports identified were used. A total of 163 reports were detected during the period consulted (N = 163).

### Study protocol

The database of notifications made in Notivisa during the period analyzed contained 9,253 records of adverse events related to healthcare. These notifications were filtered by the variable “place of occurrence”, and notifications made in the surgical center were selected. From the variables available in the database, the following were selected for the study: type of incident, gender, patient age, degree of harm and hospitalization diagnosis.

To calculate the prevalence of adverse events in surgical centers, the number of surgeries performed by the institutions during the period analyzed by the study was used, using the Hospital Information System (SIH) as the source, through the DATASUS of the Ministry of Health, using the content “hospitalizations”, procedure group code 04 “surgical procedures” and the CNES of each establishment. The specialties served by the institutions were consulted through access to the National Registry of Health Establishments (CNES) via DATASUS and analysis of production in the SIH.

### Analysis of results and statistics

The data were analyzed by calculating the simple and relative frequencies of the variables. The free-access statistical program JASP 0.17.3.0 was used. The International Classification for Patient Safety of the World Health Organization (WHO)^(15)^ was used as a reference to classify the types of adverse events reported in the notifications. Never events, events that should never occur in health services, were classified using the Manual for Implementation of the Patient Safety Center in Health Services of ANVISA^(9)^ as a reference.

The records reported in the notifications were used for the qualitative analysis. The notification form of the Notivisa system has an area for the notifier to make a subjective report of the event that occurred, “report the type of incident that occurred”. Of the 163 notifications used in the study, 44 left this space blank, and 119 reports that were completed were used for the qualitative analysis.

The data were organized in a text file and analyzed in the RStudio software. R is a free programming language that offers several packages related to data quality assessment for observational health studies. The analysis of the domains identified in the research was performed using the R interface for multidimensional analysis of texts and questionnaires (IRaMuTeQ) V.0.7 alpha 2.

Initially, in the analysis of the content of the reports, figures were created using graph theory (similarity analysis) as a basis, as well as a word cloud, through which it is possible to identify the textual occurrences between words, helping to identify the structure of the reports based on the content of a textual corpus. Two textual analyses were performed: similarity analysis, which allows the identification of occurrences between words and its result indicates the connection between words, considering words with a frequency equal to or greater than 10; word cloud, to group the words and organize them graphically according to their relevance, with the largest ones being those with the highest frequency, considering words with any frequency.

## RESULTS

During the period analyzed, the eight hospitals performed 151,293 surgical procedures and 9,253 notifications in NOTIVISA, with 163 (1.76%) notifications of adverse events performed by the surgical center (Table 1), which represents a rate of eleven notifications for every 10,000 surgeries (0.0011%) and eighteen surgical notifications for every thousand notifications of adverse events performed (0.018%).

**Chart 1:** Characteristics of hospitals participating in the study, number of surgical procedures and reports of adverse events (January/2019 to March/2023)

	Surgical specialties	Surgeries performed^*^	Notifications in NOTIVISA	Surgical adverse event notifications
Hospital A	General, maxillofacial, neurosurgery, endocrinology, gastroenterology, gynecology, nephrology, urology, ophthalmology, oncology, trauma-orthopedics, otorhinolaryngology, plastic surgery and transplantation	41,559	3,885	48
Hospital B	General, gastroenterology, gynecology, nephrology, urology, oncology and thoracic	23,345	553	23
Hospital C	Cardiology, pulmonology, thoracic and transplant (adults and pediatrics)	20,818	327	1
Hospital D	General and neurosurgery	18,612	3,708	39
Hospital E	General and trauma-orthopedics	17,385	362	43
Hospital F	Pediatric surgery, cardiology, gastroenterology, nephrology, urology, neurosurgery, oncology, trauma-orthopedics, transplantation	14,213	59	4
Hospital G	General, otorhinolaryngology and trauma-orthopedics (adults and pediatrics)	7,952	111	4
Hospital H	General	7,409	248	1
Total		151,293	9,253	163

Source: CNES; SIH; NOTIVISA.

* SIH/DATASUS.

**Table 1:** Distribution of adverse events according to type of damage

Demage degree	F	%
None	17	10.4
light	93	57.1
Moderate	45	27.6
Severe	7	4.3
Death	1	0.6

*Source: Notivisa/ Anvisa/ Ministry of Health.*

The characteristics of the institutions participating in the study are described in Chart 1.

Regarding the degree of harm suffered, 57.1% of the notifications indicated events with mild harm (Table 1), which mainly affected males (95; 58.3%), adults aged between 18 and 65 years (92; 56.4%) or over 65 years (58; 35.6%). Diseases of the digestive system (22; 13.5%), injuries due to external causes (20; 12.3%), diseases of the circulatory system (19; 11.7%), respiratory system (14; 8.6%), neoplasms (13; 8%), disorders of the genitourinary system (12; 7.3%) and nervous system (10; 6.1%) were the main diagnoses of patients who suffered adverse events. One adverse event that resulted in death was reported (0.6%), which was related to postoperative hemorrhage.

In terms of identifying the type of adverse event reported, the most frequent was pressure injury, followed by burns, surgical site infection and organ damage. Twenty-one (12.9%) surgical suspensions were reported due to failures in preparing the patient for the procedure, lack of professionals or supplies, as well as overcrowding in the Post-Anesthesia Recovery Room (PACR). It should be noted that fifteen reports did not specify the type of adverse event on the notification form (Table 2).

**Table 2:** Classification of adverse events reported by surgical centers in the hospitals analyzed

Tipos de Incidentes	Especificação	F	%
Pressure injury (n = 59)	Pressure injury^+^	59	36.2
Incident / adverse event during surgical procedure (n = 17)	Organ damage	10	6.1
	Wrong procedure, wrong patient or in the wrong place^#^	4	2.4
	Involuntary opening of the surgical wound (dehiscence)	1	0.6
	Bleeding after surgery	1	0.6
	Retention of foreign body in patient^#^	1	0.6
Adverse event with patient (n = 17)	Patient burn	11	6.7
	Accidental endotracheal extubation	1	0.6
	Damage to the oral cavity during intubation	3	1.8
	Fall from stretcher	2	1.2
Healthcare-related infections (n = 10)	Surgical site infection	10	6.1
Incident / adverse event in administrative activities (n = 4)	Error in test results	1	0.6
	Wrong procedure on the surgical map	2	1.2
	Change of patient documents	1	0.6
Incident / adverse event in patient identification (n = 2)	Missing identification bracelet	1	0.6
	Wristband with another patient's name	1	0.6
Problem / adverse event related to the use of medicines (n = 1)	Wrong medication administered	1	0.6
Other incidents/adverse events (n =9)	Failure to identify hypoglycemia	1	0.6
	Problems with catheters or probes	8	4.9
Quality of service (n = 29)	Surgical suspension (including failure to prepare the patient, lack of professional or supplies and crowding in the Post Anesthesia Care Unit (PACU)	21	12.9
	Patient without intraoperative exam due to lack of supplies	1	0.6
	Sepsis protocol discontinued	1	0.6
	Loss of biopsy material^#^	2	1.2
	Crowded PACU	1	0.6
	Prolonged surgery time to disinfect equipment used in two surgeries	1	0.6
	Use of defective equipment in surgery (surgical focus)	1	0.6
	Prolonged surgery time due to short circuit	1	0.6
Event not specified		15	9.2
Total		163	100

*Source: Notivisa/ Anvisa/ Ministry of Health. #Never event. +Never event: stage 3 and 4 pressure injuries.*

Sixteen (9.8%) never events were identified, namely: stage three pressure injuries (6; 37.5%), stage four pressure injuries (3; 18.7%), loss of biopsy material (2; 12.5%), surgical procedure on wrong patient (2; 12.5%), wrong surgical procedure performed on patient (1; 6.2%), unintentional retention of foreign body in patient after surgery (1; 6.2%) and surgical procedure in wrong place (1; 6.2%) (Table 2).

Of the reports of pressure injuries, those classified as stage two (29; 49.2%) stood out, followed by those classified as stage one. The most frequent site of occurrence was the sacral region (23; 39.0%) (Table 3).

**Table 3:** Characteristics of reported perioperative positioning-related pressure injuries (n=59)

Training	F	%
1	21	35.6
2	29	49.2
3	6	10.1
4	3	5.1
Local		
Not informed	21	35.6
Sacral	23	39.0
Gluteal	5	8.4
Calcaneus	3	5.1
Trochanter	1	1.7
Malleolus	1	1.7
Auricular pavilion	1	1.7
Knee	1	1.7
Thorax	1	1.7
Occiput	1	1.7
Elbow	1	1.7
Total	59	100

*Source: Notivisa/ Anvisa/ Ministry of Health.*

**Figure 1:**
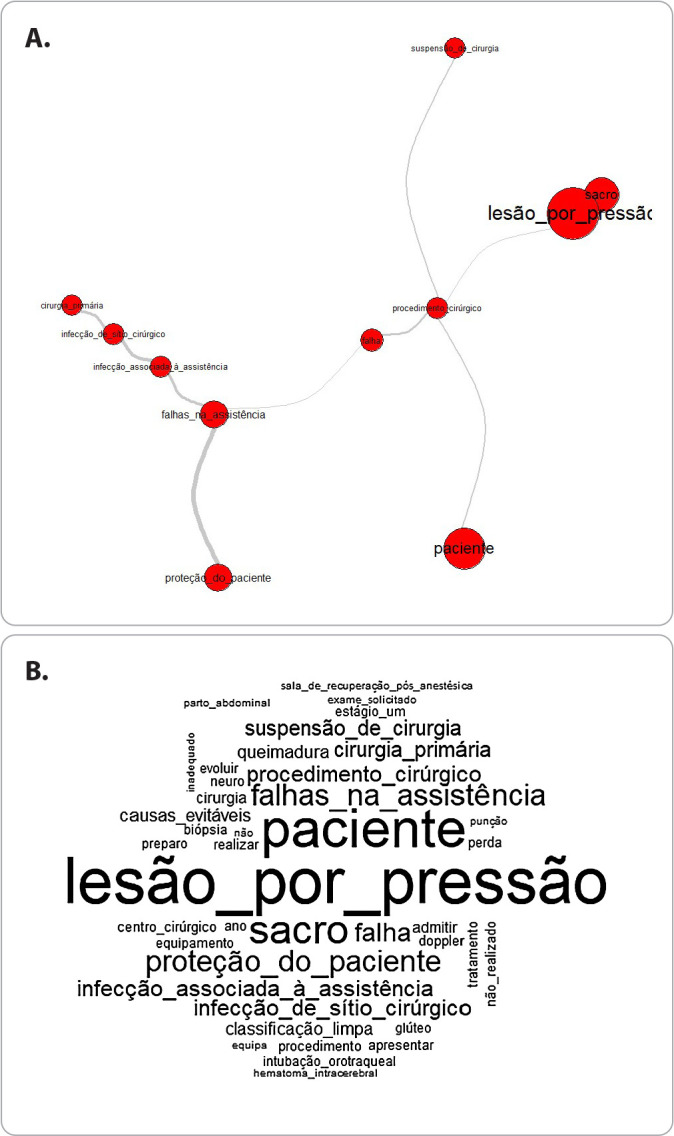
Similarity analysis.

In the similarity analysis conducted on the 119 reports, it was possible to observe that there are four prominent terms, “failures in care”, “failure”, “surgical procedure” and “pressure injury”, with which all other words or terms are connected (Figure 1).

*Fonte: Notivisa/Anvisa/ Ministério da Saúde.*

Additionally, the term “failures in care” is connected to the terms “care-associated infection”, “surgical site infection”, “primary surgery” and “patient protection”. The word “failure” is connected to the terms “failure in care” and “surgical procedure”. The term “surgical procedure” is connected to the words “failure” and “patients”, in addition to being connected to the term “suspension of surgery”. Finally, the term “pressure injury” is connected to the term “surgical procedure” and the word “sacrum”. The word cloud allowed the confirmation of the words and terms most frequently used in the notifications: pressure injury (f=39), patient (f=33), sacrum (f=23), surgery (f=16), care failures (f=15), patient protection (f=15), failure (f=14), surgical site infection (f=11), care-associated infection (f=11), surgery suspension (f=10), preventable causes (f=8) and burns (f=8).

## DISCUSSION

Reporting adverse events is a practice that allows healthcare institutions to identify and acknowledge situations that generate risk and harm to patients at the time of care. However, it still faces barriers and limitations to its full incorporation into the work routine of healthcare professionals^(16)^. Some types of adverse events face even more barriers to being reported, as suggested by the results of this study, with only eighteen surgical reports for every thousand reports, considering all those made by hospitals in the period analyzed. This data warns of a scenario of underreporting that limits the recognition of the real scenario of occurrence of adverse events in surgical centers.

In comparison with the results of other Brazilian studies, the prevalence of adverse events reported by surgical centers was lower in this study^(5,6)^. It is worth noting that the analyses cited here were performed taking into account investigations into medical records that detected adverse events reported by professional records. This shows that there is still a limited culture of reporting adverse events in surgical centers in official reporting systems, with patient records being the safest source for measuring the prevalence of incidents that resulted in harm to surgical patients.

On the other hand, the reports identified allowed us to verify weaknesses in processes, such as the adequate application of the Safe Surgery Checklist, which resulted in never events, such as the performance of incorrect procedures on incorrect patients, as well as failures in patient identification, among others.

Studies have shown that healthcare teams have limitations in the act of reporting, which range from understanding what adverse events are, identifying their occurrence in clinical practice, detecting near-misses and which situations are reportable^(17,18)^. In the present study, these limitations vary between institutions and sectors, considering that some made many more notifications than others and that a high number of records of other types of events that occurred in the hospital were also identified, although few specifically in the Surgical Center, which points to differences in the culture of inter-institutional notification and between sectors of the same institution.

The gaps in the process of reporting adverse events may contribute to the failure to recognize the problems experienced in the health sector, making it impossible for successful prevention actions to be adopted and implemented. In the present study, the most frequently reported adverse events were related to situations that could potentially be prevented, through the implementation of well-defined protocols or team training. Likewise, the only event that resulted in death and was associated with the occurrence of postoperative hemorrhage may have benefited from the existence of these protocols.

In this sense, an increased incidence of pressure injuries resulting from surgical positioning was observed, especially in surgeries lasting more than two hours, since they occur three times more frequently in these cases, which, in addition to the negative effect of the injury itself, cause additional problems for the patient and the health institution and are potentially preventable during surgical care. In agreement with the results found, the literature has indicated that injuries in early stages (one and two) are more prevalent, with the sacral region being the main site of occurrence, considering that the supine position in surgeries is the most prevalent^(19,20)^.

Another adverse event frequently observed was the occurrence of burns, which in most cases are related to aspects related to electrosurgery, resulting from maintenance failures, inadequate use or lack of training. Regular training of the surgical team in the handling of the electric scalpel, together with the creation and updating of specific protocols, can contribute to harm reduction. In this sense, the team must remain alert to audible alarms, avoiding inadvertent activations and ensuring the correct functioning of the electrosurgery systems^(21)^.

Another aspect that was related to the occurrence of adverse events was the appropriate indication and correct technique, since even elective surgical procedures are subject to events associated or not with the patient’s pre-existing clinical condition and that can lead to relevant complications that have an impact on the patient’s postoperative recovery, such as stroke, myocardial infarction, acute respiratory distress syndrome, acute kidney injury or acute intestinal injury are among the most common causes of morbidity and mortality in surgical patients. For their prevention, multidisciplinary patient-centered care protocols have beneficial effects in preventing surgical stress and early recovery in the postoperative period^(22)^.

In this sense, surgical site infection is the most common postoperative complication, and the most frequently reported adverse event related to care. Its occurrence has been related to the organizational safety culture and raises the need to evaluate surgical care indicators so that the risks of its occurrence can be assessed^(23)^.

In the context of improving surgical safety, the WHO Surgical Safety Checklist is the communication tool designed to improve processes and enhance teamwork in the surgical center, and is considered the gold standard for preventing adverse events such as those previously reported^(24)^. Therefore, its adoption and adherence is highly recommended and is strongly associated with reducing the occurrence of harm in the provision of care^(25)^. However, its adherence in the context of Brazilian health institutions is still incipient^(14,26)^.

This gap in implementation is particularly relevant when we consider the findings of the present study, which reveal that reporting professionals use the adverse event reporting system as a channel to report problems related to the quality of service or situations that generate potential risks to patients. These reports range from reports of surgery suspensions due to lack of professionals, material supplies or availability of beds in the PACU to other situations that require attention from management. In this context, safe and effective team communication is a determining factor in reducing adverse events, based on the appreciation and perception of attitudes and behavior of all professionals involved in the perioperative care of patients, with a view to promoting a culture of safety^(27)^. Regarding the content of the reports, there was evident variability in the way professionals described adverse events. Some reports presented brief reports and limited information about the occurrence. In contrast, other reports were more comprehensive, providing detailed details about the incident, its repercussions for the patient and the intervention measures implemented by the team. The literature indicates that professionals are unaware of the existence of report forms or instructions on how to report and use other means of recording adverse events that have occurred, such as nurses and nursing technicians who only use nursing notes to make this type of report^(28)^.

It is important to notice that some of the records analyzed in this study were not used in the qualitative analysis because they were not filled out in the space provided for the notifier’s report, which indicates a serious flaw in the notification process. The written report should support and complement the form with data that can be used in the root cause analysis of the event, and this support is necessary to elucidate the mechanisms of occurrence and what measures should be adopted for its prevention. It is worth noting that this study differs from other analyses of the notification process in that it uses data from the records made.

By using terms such as “failure in care” and “patient protection” in the description of the event at the time of notification, the notifiers demonstrate that they understand that the occurrences are related to errors in the care process and that these compromise the safety of the people receiving care, thus there is prior awareness, that is, an understanding of how adverse events affect work processes and the quality of surgical care. The similarity analysis shows that these terms stand out in the notifications, being prominent and related to the terms that define which adverse event is being reported.

It is worth noting that, given the COVID-19 pandemic, the act of notification has once again gained notoriety and has been discussed regarding its importance, as it allows the epidemiological scenario and factors associated with the occurrence of diseases and injuries to be highlighted^(16)^.

Additionally, the quality of the notification, considering complete and clear completion, should be one of the points to be worked on in creating an institutional culture of event notification. It is recommended that institutions, after implementing the event notification form, use this instrument in continuing education, training all professionals to complete the notification form, clarifying its relevance and exercising constant evaluation and monitoring of this activity by professionals^(17,28)^.

The performance of the Brazilian notification system is a substantial factor in analyzing the quality of notifications, and this has been pointed out in other types of notification, such as pharmacovigilance^(29)^. Improvements should be proposed to promote completeness of records, providing more data that can be used for root cause analysis and correct management of the system by reporting professionals.

### Study limitations

The study was limited by its cross-sectional design and access only to documented records of notifications without having approached the reporting professionals who could present their views on how they understand the reported events and the registration process in the system. Through this approach, improvements can be suggested and training strategies developed with the health team.

Another limiting factor was the small sample of detected notifications. This limits the generalization of the data to the population.

### Contributions to the field of nursing, health or public policy

The act of reporting is intrinsically associated with an organizational safety culture that supports professionals through non-punitive measures, open communication, present leadership, and the provision of quality reporting mechanisms. The study findings indicate that the culture of reporting adverse events in surgical centers still suffers from a strong limitation represented by the reduced number of reports identified, which hinders risk analysis and management.

The main contribution of the results found is related to the possibility of motivating managers to develop strategies aimed at encouraging reporting, training focused on the act of reporting and how to do it with quality. Additional studies are needed to propose training strategies and develop tools that facilitate reporting.

Besides that, the development of systems that meet prerequisites compatible with the needs of health services is an urgent demand. Define a minimum set of mandatory variables that must be included in all notifications and specific variables for surgical notifications, such as checking the use of the surgical safety checklist, the type of anesthesia used in the procedure, what measures were adopted by the surgical team upon detecting the adverse event, what aggravating and mitigating actions, must be included in the notification so that a broader overview can be drawn up regarding the occurrence of the reported event.

## CONCLUSIONS

Pressure injuries resulting from surgical positioning, burns caused by electric scalpels, organ damage and surgical site infection stood out as the main surgical adverse events reported. The patients who suffered the most events had injuries classified as mild, were male and were in the adult age range of 18 to 65 years. The diagnoses recorded at admission were mainly related to non-infectious chronic diseases linked to the digestive, circulatory and respiratory systems and neoplasms.

The prevalence of surgical notifications recorded in the system compared to the volume of general notifications and the number of surgeries performed points to a significant underreporting of incidents that occur during surgical care.

The adverse event notifications presented limitations in their records, such as unspecified adverse events, missing data and descriptions not filled out for more than a quarter of the notifications analyzed. In addition, there are differences between the reports recorded, with no standardization in the way the events were reported, which varied from excessively brief to detailed descriptions.

In short, the results indicate which adverse events reported by surgical centers are most prevalent and, therefore, deserve the attention of the team and health services for the adoption of specific measures and clinical protocols for their prevention. Some adverse events identified demonstrate inconsistency in the application of the surgical safety checklist.

Finally, strategies to encourage and improve reporting must be considered. Reporting more and better should be the goal of health institutions. A standard reporting model must be designed and replicated to professionals through training and a continuous learning process to ensure minimum data provided by reports. This will make it possible to conduct investigations of adverse events that point to associated factors and necessary points for improvement.

## Data Availability

https://doi.org/10.48331/scielodata.KUHHDE PDF
