# Supplementary material for: Frequency and content analysis of adverse event reports in surgical centers: a cross-sectional study
Source: Rev Bras Enferm. 2025 Mar 14;78(1):e20240082. doi: 10.1590/0034-7167-2024-0082 (PMC11913048; doi:10.1590/0034-7167-2024-0082)
Supplement: Supplementary file 1 [file 0034-7167-reben-78-01-e20240082-suppl01.pdf]

Data do envio	Estado	Municípi o	Nome fantasia do	Informe o tipo de incidente	Situação	Produto motivo	Tipo de Incidente / Evento	Mecanis mo do acidente	Tipo de dieta
18/05/2022	CE	FORTALE ZA	HOSPITA L ESTADU	Após quadro de	Enviada	Eventos Adversos Relaciona	Queda do paciente		
18/05/2022	CE	FORTALE ZA	HOSPITA L ESTADU	Houve troca das dietas	Enviada	Eventos Adversos Relaciona	Falha na identificaç ão do		
18/05/2022	CE	FORTALE ZA	HOSPITA L ESTADU	Perda de cateter de	Enviada	Eventos Adversos Relaciona	Falhas durante a assistênci		
14/06/2022	CE	FORTALE ZA	HOSPITA L ESTADU	Paciente teve a dieta	Enviada	Eventos Adversos Relaciona	Falhas na administra ção de		Enteral (oral, cateter
14/06/2022	CE	FORTALE ZA	HOSPITA L ESTADU	Identificad o membro	Enviada	Eventos Adversos Relaciona	Falhas envolvend o cateter		
14/06/2022	CE	FORTALE ZA	HOSPITA L ESTADU	Administr ação de medicame	Enviada	Eventos Adversos Relaciona	Falha na identificaç ão do		
14/06/2022	CE	FORTALE ZA	HOSPITA L ESTADU	PACIENT E RECEBE	Enviada	Eventos Adversos Relaciona	Falhas na administra ção de		Enteral (oral, cateter
14/06/2022	CE	FORTALE ZA	HOSPITA L ESTADU	PACIENT E SOFREU	Enviada	Eventos Adversos Relaciona	Queda do paciente		
17/06/2022	CE	FORTALE ZA	HOSPITA L ESTADU	Falha na administra ção de	Enviada	Eventos Adversos Relaciona	Falha na identificaç ão do		
17/06/2022	CE	FORTALE ZA	HOSPITA L ESTADU	Falha na instalação /	Enviada	Eventos Adversos Relaciona	Falhas na administra ção de		Parenteral (via intravenos
17/06/2022	CE	FORTALE ZA	HOSPITA L ESTADU	FLEBITE EM MSD, RETIRAD	Enviada	Eventos Adversos Relaciona	Falhas envolvend o cateter		
17/06/2022	CE	FORTALE ZA	HOSPITA L ESTADU	PERDA DE CATETE	Enviada	Eventos Adversos Relaciona	Falhas durante a assistênci		
17/06/2022	CE	FORTALE ZA	HOSPITA L ESTADU	PACIENT E SOFRE	Enviada	Eventos Adversos Relaciona	Falhas durante a assistênci		
08/07/2022	CE	FORTALE ZA	HOSPITA L ESTADU	PACIENT E APRESE	Enviada	Eventos Adversos Relaciona	Falhas durante a assistênci		
08/07/2022	CE	FORTALE ZA	HOSPITA L ESTADU	EVENTO ADVERS O SEM	Enviada	Eventos Adversos Relaciona	Falhas na administra ção de		Enteral (oral, cateter
29/07/2022	CE	FORTALE ZA	HOSPITA L ESTADU	Evento adverso com	Enviada	Eventos Adversos Relaciona	Queda do paciente		
29/07/2022	CE	FORTALE ZA	HOSPITA L ESTADU	Evento adverso moderado	Enviada	Eventos Adversos Relaciona	Queda do paciente		

18/08/2022	CE	FORTALE<br>ZA	HOSPITA<br>L<br>ESTADU	Evento<br>adverso<br>moderado	Enviada	Eventos<br>Adversos<br>Relaciona	Queda do<br>paciente		
18/08/2022	CE	FORTALE<br>ZA	HOSPITA<br>L<br>ESTADU	Evento<br>adverso<br>com	Enviada	Eventos<br>Adversos<br>Relaciona	Queda do<br>paciente		
19/08/2022	CE	FORTALE<br>ZA	HOSPITA<br>L<br>ESTADU	evento<br>adverso<br>moderado	Enviada	Eventos<br>Adversos<br>Relaciona	Queimadu<br>ra de<br>paciente		
19/08/2022	CE	FORTALE<br>ZA	HOSPITA<br>L<br>ESTADU	evento<br>sem<br>danos/	Enviada	Eventos<br>Adversos<br>Relaciona	Queda do<br>paciente		
19/08/2022	CE	FORTALE<br>ZA	HOSPITA<br>L<br>ESTADU	evento<br>sem<br>dano/	Enviada	Eventos<br>Adversos<br>Relaciona	Queda do<br>paciente		
19/08/2022	CE	FORTALE<br>ZA	HOSPITA<br>L<br>ESTADU	evento<br>adverso<br>moderado	Enviada	Eventos<br>Adversos<br>Relaciona	Queda do<br>paciente		
19/08/2022	CE	FORTALE<br>ZA	HOSPITA<br>L<br>ESTADU	Evento<br>adverso<br>sem dano	Enviada	Eventos<br>Adversos<br>Relaciona	Extubaçã<br>o<br>endotraqu		
30/08/2022	CE	FORTALE<br>ZA	HOSPITA<br>L<br>ESTADU	PACIENT<br>E<br>APRESE	Enviada	Eventos<br>Adversos<br>Relaciona	Falhas<br>envolvend<br>o cateter		
30/08/2022	CE	FORTALE<br>ZA	HOSPITA<br>L<br>ESTADU	PACIENT<br>E CAIU<br>NA	Enviada	Eventos<br>Adversos<br>Relaciona	Queda do<br>paciente		
30/08/2022	CE	FORTALE<br>ZA	HOSPITA<br>L<br>ESTADU	APÓS A<br>REALIZA<br>ÇÃO DE	Enviada	Eventos<br>Adversos<br>Relaciona	Queimadu<br>ra de<br>paciente		
30/08/2022	CE	FORTALE<br>ZA	HOSPITA<br>L<br>ESTADU	PACIENT<br>E VINDO	Enviada	Eventos<br>Adversos<br>Relaciona	Falhas<br>durante a<br>assistênci		
30/08/2022	CE	FORTALE<br>ZA	HOSPITA<br>L<br>ESTADU	DIETA<br>VIA<br>ORAL	Enviada	Eventos<br>Adversos<br>Relaciona	Falhas na<br>administra<br>ção de		Enteral<br>(oral,<br>cateter
30/08/2022	CE	FORTALE<br>ZA	HOSPITA<br>L<br>ESTADU	PACIENT<br>E<br>RECEBE	Enviada	Eventos<br>Adversos<br>Relaciona	Falhas na<br>administra<br>ção de		Enteral<br>(oral,<br>cateter
30/08/2022	CE	FORTALE<br>ZA	HOSPITA<br>L<br>ESTADU	NÃO FOI<br>ADMINIS<br>TRADA A	Enviada	Eventos<br>Adversos<br>Relaciona	Falhas na<br>administra<br>ção de		Enteral<br>(oral,<br>cateter
30/08/2022	CE	FORTALE<br>ZA	HOSPITA<br>L<br>ESTADU	PACIENT<br>E COM	Enviada	Eventos<br>Adversos<br>Relaciona	Falhas<br>durante a<br>assistênci		
30/08/2022	CE	FORTALE<br>ZA	HOSPITA<br>L<br>ESTADU	PACIENT<br>E no<br>SETOR	Enviada	Eventos<br>Adversos<br>Relaciona	Evasão<br>do<br>paciente		
30/08/2022	CE	FORTALE<br>ZA	HOSPITA<br>L<br>ESTADU	PACIENT<br>E<br>DESORIE	Enviada	Eventos<br>Adversos<br>Relaciona	Queda do<br>paciente		
30/08/2022	CE	FORTALE<br>ZA	HOSPITA<br>L<br>ESTADU	PACIENT<br>E<br>TRANSF	Enviada	Eventos<br>Adversos<br>Relaciona	Falhas<br>durante a<br>assistênci		

20/09/2022	CE	FORTALEZA	HOSPITAL ESTADU	SOLICITADO AUME	Enviada	Eventos Adversos Relaciona	Falhas na administração de		Enteral (oral, cateter
20/09/2022	CE	FORTALEZA	HOSPITAL ESTADU	Não houve a dispensaç	Enviada	Eventos Adversos Relaciona	Falhas na administração de		Enteral (oral, cateter
20/09/2022	CE	FORTALEZA	HOSPITAL ESTADU	Paciente recebeu a dieta	Enviada	Eventos Adversos Relaciona	Falhas na administração de		Enteral (oral, cateter
20/09/2022	CE	FORTALEZA	HOSPITAL ESTADU	PACIENTE SUBMETI	Enviada	Eventos Adversos Relaciona	Falhas durante procedim		
21/09/2022	CE	FORTALEZA	HOSPITAL ESTADU	PACIENTE CAIU DA	Enviada	Eventos Adversos Relaciona	Queda do paciente		
21/09/2022	CE	FORTALEZA	HOSPITAL ESTADU	PACIENTE ESCORR	Enviada	Eventos Adversos Relaciona	Queda do paciente		
21/09/2022	CE	FORTALEZA	HOSPITAL ESTADU	PRESCRITO DIETA	Enviada	Eventos Adversos Relaciona	Falhas na administração de		Enteral (oral, cateter
21/09/2022	CE	FORTALEZA	HOSPITAL ESTADU	PACIENTE APRESE	Enviada	Eventos Adversos Relaciona	Falhas durante procedim		
21/09/2022	CE	FORTALEZA	HOSPITAL ESTADU	PACIENTE APRESE	Enviada	Eventos Adversos Relaciona	Broncoaspiração		
21/09/2022	CE	FORTALEZA	HOSPITAL ESTADU	PACIENTE PARA A	Enviada	Eventos Adversos Relaciona	Falha na identificação do		
18/10/2022	CE	FORTALEZA	HOSPITAL ESTADU	PACIENTE	Enviada	Eventos Adversos Relaciona	Queda do paciente		
26/10/2022	CE	FORTALEZA	HOSPITAL ESTADU	PACIENTE APRESE	Enviada	Eventos Adversos Relaciona	Falhas envolvendo o cateter		
26/10/2022	CE	FORTALEZA	HOSPITAL ESTADU	Paciente retirou/puxou	Enviada	Eventos Adversos Relaciona	Falhas durante a assistênci		
26/10/2022	CE	FORTALEZA	HOSPITAL ESTADU	Paciente em jejum para	Enviada	Eventos Adversos Relaciona	Falhas na administração de		Enteral (oral, cateter
26/10/2022	CE	FORTALEZA	HOSPITAL ESTADU	Não administrado a	Enviada	Eventos Adversos Relaciona	Falhas na administração de		Enteral (oral, cateter
26/10/2022	CE	FORTALEZA	HOSPITAL ESTADU	Paciente com dieta prescrita	Enviada	Eventos Adversos Relaciona	Falhas na administração de		Enteral (oral, cateter
26/10/2022	CE	FORTALEZA	HOSPITAL ESTADU	PACIENTE APRESE	Enviada	Eventos Adversos Relaciona	Falhas envolvendo o cateter		
26/10/2022	CE	FORTALEZA	HOSPITAL ESTADU	Drenagem de hemotóra	Enviada	Eventos Adversos Relaciona	Falhas envolvendo o cateter		

26/10/2022	CE	FORTALE ZA	HOSPITA L ESTADU	Dieta não administra da às 9H.	Enviada	Eventos Adversos Relaciona	Falhas na administra ção de		Enteral (oral, cateter
27/10/2022	CE	FORTALE ZA	HOSPITA L ESTADU	Módulo compleme ntar	Enviada	Eventos Adversos Relaciona	Falhas na administra ção de		Enteral (oral, cateter
27/10/2022	CE	FORTALE ZA	HOSPITA L ESTADU	Paciente com membro	Enviada	Eventos Adversos Relaciona	Falhas durante a assistênci		
27/10/2022	CE	FORTALE ZA	HOSPITA L ESTADU	Atraso na instalação de	Enviada	Eventos Adversos Relaciona	Falhas durante a assistênci		
27/10/2022	CE	FORTALE ZA	HOSPITA L ESTADU	Atraso na reavaliaçã o do	Enviada	Eventos Adversos Relaciona	Falhas durante a assistênci		
27/10/2022	CE	FORTALE ZA	HOSPITA L ESTADU	Nutrição parenteral prescrita	Enviada	Eventos Adversos Relaciona	Falhas na administra ção de		Parenteral (via intravenos
27/10/2022	CE	FORTALE ZA	HOSPITA L ESTADU	Dieta não administra da as 9h,	Enviada	Eventos Adversos Relaciona	Falhas na administra ção de		Enteral (oral, cateter
27/10/2022	CE	FORTALE ZA	HOSPITA L ESTADU	Dieta não administra da as 9h,	Enviada	Eventos Adversos Relaciona	Falhas na administra ção de		Enteral (oral, cateter
27/10/2022	CE	FORTALE ZA	HOSPITA L ESTADU	Não foi ofertado a dieta às	Enviada	Eventos Adversos Relaciona	Falhas na administra ção de		Enteral (oral, cateter
27/10/2022	CE	FORTALE ZA	HOSPITA L ESTADU	PACIENT E HAVIA APRESE	Enviada	Eventos Adversos Relaciona	Falhas na administra ção de		Enteral (oral, cateter
28/10/2022	CE	FORTALE ZA	HOSPITA L ESTADU	Perda da funcionali dade do	Enviada	Eventos Adversos Relaciona	Falhas durante a assistênci		
28/10/2022	CE	FORTALE ZA	HOSPITA L ESTADU	Não administra do agua	Enviada	Eventos Adversos Relaciona	Falhas na administra ção de		Enteral (oral, cateter
28/10/2022	CE	FORTALE ZA	HOSPITA L ESTADU	Paciente recebeu duas	Enviada	Eventos Adversos Relaciona	Falhas durante a assistênci		
28/10/2022	CE	FORTALE ZA	HOSPITA L ESTADU	Paciente apresenta	Enviada	Eventos Adversos Relaciona	Falhas durante a assistênci		
31/10/2022	CE	FORTALE ZA	HOSPITA L ESTADU	FALHA NO PREPAR	Enviada	Eventos Adversos Relaciona	Falhas durante a assistênci		
07/11/2022	CE	FORTALE ZA	HOSPITA L ESTADU	Paciente apresenta AVP em	Enviada	Eventos Adversos Relaciona	Falhas envolvend o cateter		
07/11/2022	CE	FORTALE ZA	HOSPITA L ESTADU	Paciente apresenta flebite	Enviada	Eventos Adversos Relaciona	Falhas envolvend o cateter		
07/11/2022	CE	FORTALE ZA	HOSPITA L ESTADU	Paciente foi transferid	Enviada	Eventos Adversos Relaciona	Falhas durante a assistênci		

07/11/2022	CE	FORTALE ZA	HOSPITA L ESTADU	Paciente com CVC apresenta	Enviada	Eventos Adversos Relaciona	Falhas envolvend o cateter		
07/11/2022	CE	FORTALE ZA	HOSPITA L ESTADU	Não administra do dieta	Enviada	Eventos Adversos Relaciona	Falhas na administra ção de		Enteral (oral, cateter
07/11/2022	CE	FORTALE ZA	HOSPITA L ESTADU	Não administra do dieta	Enviada	Eventos Adversos Relaciona	Falhas na administra ção de		Enteral (oral, cateter
07/11/2022	CE	FORTALE ZA	HOSPITA L ESTADU	Paciente com CVP em MSE	Enviada	Eventos Adversos Relaciona	Falhas envolvend o cateter		
07/11/2022	CE	FORTALE ZA	HOSPITA L ESTADU	Acompan hante refere	Enviada	Eventos Adversos Relaciona	Queda do paciente		
07/11/2022	CE	FORTALE ZA	HOSPITA L ESTADU	Acompan hante relata	Enviada	Eventos Adversos Relaciona	Queda do paciente		
07/11/2022	CE	FORTALE ZA	HOSPITA L ESTADU	Paciente apresento u	Enviada	Eventos Adversos Relaciona	Falhas durante a assistênci		
07/11/2022	CE	FORTALE ZA	HOSPITA L ESTADU	Paciente apresenta edema,	Enviada	Eventos Adversos Relaciona	Falhas envolvend o cateter		
07/11/2022	CE	FORTALE ZA	HOSPITA L ESTADU	Paciente realizou tentativa	Enviada	Eventos Adversos Relaciona	Queda do paciente		
29/12/2022	CE	FORTALE ZA	HOSPITA L ESTADU	EVENTO ADVERS O LEVE -	Enviada	Eventos Adversos Relaciona	Queda do paciente		
29/12/2022	CE	FORTALE ZA	HOSPITA L ESTADU	EVENTO ADVERS O SEM	Enviada	Eventos Adversos Relaciona	Queda do paciente		
29/12/2022	CE	FORTALE ZA	HOSPITA L ESTADU	EVENTO ADVERS O SEM	Enviada	Eventos Adversos Relaciona	Falhas na administra ção de		Enteral (oral, cateter
29/12/2022	CE	FORTALE ZA	HOSPITA L ESTADU	EVENTO ADVERS O	Enviada	Eventos Adversos Relaciona	Falhas envolvend o cateter		
29/12/2022	CE	FORTALE ZA	HOSPITA L ESTADU	EVENTO ADVERS O COM	Enviada	Eventos Adversos Relaciona	Falhas envolvend o cateter		
20/01/2023	CE	FORTALE ZA	HOSPITA L ESTADU	Evento adverso moderado	Enviada	Eventos Adversos Relaciona	Queda do paciente		
24/01/2023	CE	FORTALE ZA	HOSPITA L ESTADU	Evento adverso sem dano	Enviada	Eventos Adversos Relaciona	Queda do paciente		
24/01/2023	CE	FORTALE ZA	HOSPITA L ESTADU	Evento adverso leve /	Enviada	Eventos Adversos Relaciona	Falhas envolvend o cateter		
24/01/2023	CE	FORTALE ZA	HOSPITA L ESTADU	Evento adverso leve /	Enviada	Eventos Adversos Relaciona	Falhas envolvend o cateter		

24/01/2023	CE	FORTALEZA	HOSPITAL ESTADU	Evento adverso com	Enviada	Eventos Adversos Relaciona	Falhas envolvendo cateter		
24/01/2023	CE	FORTALEZA	HOSPITAL ESTADU	Evento adverso com	Enviada	Eventos Adversos Relaciona	Falhas envolvendo cateter		
24/01/2023	CE	FORTALEZA	HOSPITAL ESTADU	Evento adverso leve /	Enviada	Eventos Adversos Relaciona	Falhas envolvendo cateter		
24/01/2023	CE	FORTALEZA	HOSPITAL ESTADU	Evento adverso leve /	Enviada	Eventos Adversos Relaciona	Queda do paciente		
24/01/2023	CE	FORTALEZA	HOSPITAL ESTADU	Evento adverso leve /	Enviada	Eventos Adversos Relaciona	Falhas envolvendo cateter		
17/02/2023	CE	FORTALEZA	HOSPITAL ESTADU	Evento adverso leve /	Enviada	Eventos Adversos Relaciona	Queda do paciente		
17/02/2023	CE	FORTALEZA	HOSPITAL ESTADU	Evento adverso leve /	Enviada	Eventos Adversos Relaciona	Falhas envolvendo cateter		
17/02/2023	CE	FORTALEZA	HOSPITAL ESTADU	Evento adverso sem dano	Enviada	Eventos Adversos Relaciona	Queda do paciente		
17/02/2023	CE	FORTALEZA	HOSPITAL ESTADU	Evento sem dano /	Enviada	Eventos Adversos Relaciona	Queda do paciente		
17/02/2023	CE	FORTALEZA	HOSPITAL ESTADU	Evento adverso moderado	Enviada	Eventos Adversos Relaciona	Lesão por pressão		
17/02/2023	CE	FORTALEZA	HOSPITAL ESTADU	Eventp adverso leve /	Enviada	Eventos Adversos Relaciona	Falhas envolvendo cateter		
17/02/2023	CE	FORTALEZA	HOSPITAL ESTADU	EVENTO SEM DANO /	Enviada	Eventos Adversos Relaciona	Falhas na administração de		Enteral (oral, cateter
17/02/2023	CE	FORTALEZA	HOSPITAL ESTADU	Evento adverso leve / AO	Enviada	Eventos Adversos Relaciona	Queimadura de paciente		
17/02/2023	CE	FORTALEZA	HOSPITAL ESTADU	Evento adverso leve /	Enviada	Eventos Adversos Relaciona	Falhas envolvendo cateter		
17/02/2023	CE	FORTALEZA	HOSPITAL ESTADU	Evento adverso moderado	Enviada	Eventos Adversos Relaciona	Queimadura de paciente		
20/03/2023	CE	FORTALEZA	HOSPITAL ESTADU	EVENTO ADVERSO LEVE /	Enviada	Eventos Adversos Relaciona	Falhas envolvendo cateter		
20/03/2023	CE	FORTALEZA	HOSPITAL ESTADU	EVENTO ADVERSO LEVE /	Enviada	Eventos Adversos Relaciona	Falhas envolvendo cateter		
20/03/2023	CE	FORTALEZA	HOSPITAL ESTADU	EVENTO ADVERSO LEVE /	Enviada	Eventos Adversos Relaciona	Lesão por pressão		

20/03/2023	CE	FORTALEZA	HOSPITAL ESTADU	EVENTO ADVERSO LEVE /	Enviada	Eventos Adversos Relaciona	Lesão por pressão		
20/03/2023	CE	FORTALEZA	HOSPITAL ESTADU	EVENTO ADVERSO LEVE /	Enviada	Eventos Adversos Relaciona	Falhas envolvendo cateter		
20/03/2023	CE	FORTALEZA	HOSPITAL ESTADU	EVENTO ADVERSO	Enviada	Eventos Adversos Relaciona	Queda do paciente		
20/03/2023	CE	FORTALEZA	HOSPITAL ESTADU	EVENTO ADVERSO	Enviada	Eventos Adversos Relaciona	Falhas envolvendo cateter		
21/05/2019	CE	FORTALEZA	HOSPITAL GERAL DR. CESAR	-	Enviada	Eventos Adversos Relacionados à	Lesão por pressão		
24/06/2019	CE	FORTALEZA	HOSPITAL GERAL DR. CESAR	-	Enviada	Eventos Adversos Relacionados à	Lesão por pressão		
24/06/2019	CE	FORTALEZA	HOSPITAL GERAL DR. CESAR	-	Enviada	Eventos Adversos Relacionados à	Lesão por pressão		
24/06/2019	CE	FORTALEZA	HOSPITAL GERAL DR. CESAR	-	Enviada	Eventos Adversos Relacionados à	Lesão por pressão		
02/07/2019	CE	FORTALEZA	HOSPITAL GERAL DR. CESAR	queda do RN pela mãe	Enviada	Eventos Adversos Relacionados à	Queda do paciente		
02/07/2019	CE	FORTALEZA	HOSPITAL GERAL DR. CESAR	RN caiu da cama com presenca	Enviada	Eventos Adversos Relacionados à	Queda do paciente		
02/07/2019	CE	FORTALEZA	HOSPITAL GERAL DR. CESAR	escorregou no pisp	Enviada	Eventos Adversos Relacionados à	Queda do paciente		
02/07/2019	CE	FORTALEZA	HOSPITAL GERAL DR. CESAR	lesão sacral	Enviada	Eventos Adversos Relacionados à	Lesão por pressão		
02/07/2019	CE	FORTALEZA	HOSPITAL GERAL DR. CESAR	admitido com lesão grau II e	Enviada	Eventos Adversos Relacionados à	Lesão por pressão		
03/07/2019	CE	FORTALEZA	HOSPITAL GERAL DR. CESAR	Paciente apresenta lesão por pressão	Enviada	Eventos Adversos Relacionados à	Lesão por pressão		
03/07/2019	CE	FORTALEZA	HOSPITAL GERAL DR. CESAR	lesão na sacra	Enviada	Eventos Adversos Relacionados à	Lesão por pressão		
03/07/2019	CE	FORTALEZA	HOSPITAL GERAL DR. CESAR	lesão sacral	Enviada	Eventos Adversos Relacionados à	Lesão por pressão		

31/08/2019	CE	FORTALEZA	HOSPITAL GERAL DR. CESAR	caiu do leito	Enviada	Eventos Adversos Relacionados à	Queda do paciente		
31/08/2019	CE	FORTALEZA	HOSPITAL GERAL DR. CESAR	ao levantar sozinha	Enviada	Eventos Adversos Relacionados à	Queda do paciente		
31/08/2019	CE	FORTALEZA	HOSPITAL GERAL DR. CESAR	sem acompanhante e desorienta	Enviada	Eventos Adversos Relacionados à	Queda do paciente		
31/08/2019	CE	FORTALEZA	HOSPITAL GERAL DR. CESAR	issional escorregou no piso molhado	Enviada	Eventos Adversos Relacionados à	Queda do paciente		
31/08/2019	CE	FORTALEZA	HOSPITAL GERAL DR. CESAR	lesaõ na sacra	Enviada	Eventos Adversos Relacionados à	Lesão por pressão		
31/08/2019	CE	FORTALEZA	HOSPITAL GERAL DR. CESAR	lesão na sacra	Enviada	Eventos Adversos Relacionados à	Lesão por pressão		
31/08/2019	CE	FORTALEZA	HOSPITAL GERAL DR. CESAR	evoluiu lesão por pressão	Enviada	Eventos Adversos Relacionados à	Lesão por pressão		
31/08/2019	CE	FORTALEZA	HOSPITAL GERAL DR. CESAR	observado lesão na admissao ndo fe	Enviada	Eventos Adversos Relacionados à	Lesão por pressão		
31/08/2019	CE	FORTALEZA	HOSPITAL GERAL DR. CESAR	na admissão observado lesão	Enviada	Eventos Adversos Relacionados à	Lesão por pressão		
31/08/2019	CE	FORTALEZA	HOSPITAL GERAL DR. CESAR	o acompanhante escorrego	Enviada	Eventos Adversos Relacionados à	Queda do paciente		
31/08/2019	CE	FORTALEZA	HOSPITAL GERAL DR. CESAR	escorregou na rampa	Enviada	Eventos Adversos Relacionados à	Queda do paciente		
31/08/2019	CE	FORTALEZA	HOSPITAL GERAL DR. CESAR	-	Enviada	Eventos Adversos Relacionados à	Queda do paciente		
31/08/2019	CE	FORTALEZA	HOSPITAL GERAL DR. CESAR	escorrgou do piso molhado	Enviada	Eventos Adversos Relacionados à	Queda do paciente		
31/08/2019	CE	FORTALEZA	HOSPITAL GERAL DR. CESAR	-	Enviada	Eventos Adversos Relacionados à	Queda do paciente		
31/08/2019	CE	FORTALEZA	HOSPITAL GERAL DR. CESAR	torce do pé por desnivel do piso	Enviada	Eventos Adversos Relacionados à	Queda do paciente		

31/08/2019	CE	FORTALEZA	HOSPITAL GERAL DR. CESAR	evoluiu ma internação c LP	Enviada	Eventos Adversos Relacionados à	Lesão por pressão		
31/08/2019	CE	FORTALEZA	HOSPITAL GERAL DR. CESAR	durante sua internação	Enviada	Eventos Adversos Relacionados à	Lesão por pressão		
31/08/2019	CE	FORTALEZA	HOSPITAL GERAL DR. CESAR	-	Enviada	Eventos Adversos Relacionados à	Lesão por pressão		
31/08/2019	CE	FORTALEZA	HOSPITAL GERAL DR. CESAR	evoluiu co, LP na imternação	Enviada	Eventos Adversos Relacionados à	Lesão por pressão		
31/08/2019	CE	FORTALEZA	HOSPITAL GERAL DR. CESAR	escorregou do leito , desorienta	Enviada	Eventos Adversos Relacionados à	Queda do paciente		
31/08/2019	CE	FORTALEZA	HOSPITAL GERAL DR. CESAR	deambulando e tropeçou	Enviada	Eventos Adversos Relacionados à	Queda do paciente		
31/08/2019	CE	FORTALEZA	HOSPITAL GERAL DR. CESAR	no termino do banho ao	Enviada	Eventos Adversos Relacionados à	Queda do paciente		
31/08/2019	CE	FORTALEZA	HOSPITAL GERAL DR. CESAR	mãe deambulando deixou	Enviada	Eventos Adversos Relacionados à	Queda do paciente		
07/10/2019	CE	FORTALEZA	HOSPITAL GERAL DR. CESAR	resistencia na infusao	Enviada	Eventos Adversos Relacionados à	Falhas envolvendo cateter venoso		
07/10/2019	CE	FORTALEZA	HOSPITAL GERAL DR. CESAR	resistencia de fluxo	Enviada	Eventos Adversos Relacionados à	Falhas envolvendo cateter venoso		
07/10/2019	CE	FORTALEZA	HOSPITAL GERAL DR. CESAR	exteriorização de cateter por falta	Enviada	Eventos Adversos Relacionados à	Falhas envolvendo cateter venoso		
07/10/2019	CE	FORTALEZA	HOSPITAL GERAL DR. CESAR	extravazamento pela lateral do	Enviada	Eventos Adversos Relacionados à	Falhas envolvendo cateter venoso		
07/10/2019	CE	FORTALEZA	HOSPITAL GERAL DR. CESAR	-	Enviada	Eventos Adversos Relacionados à	Falhas envolvendo cateter venoso		
09/10/2019	CE	FORTALEZA	HOSPITAL GERAL DR. CESAR	coleta em membro sinalizado com	Enviada	Eventos Adversos Relacionados à	Falhas ocorridas em laboratóri		
09/10/2019	CE	FORTALEZA	HOSPITAL GERAL DR. CESAR	-	Enviada	Eventos Adversos Relacionados à	Extubação endotraqueal		

09/10/2019	CE	FORTALEZA	HOSPITAL GERAL DR. CESAR	dialise com heparina nos	Enviada	Eventos Adversos Relacionados à	Falhas durante a assistência à saúde		
09/10/2019	CE	FORTALEZA	HOSPITAL GERAL DR. CESAR	-	Enviada	Eventos Adversos Relacionados à	Falhas durante a assistência à saúde		
19/10/2019	CE	FORTALEZA	HOSPITAL GERAL DR. CESAR	sonda vesical com balão	Enviada	Eventos Adversos Relacionados à	Falhas envolvendo sondas		
19/10/2019	CE	FORTALEZA	HOSPITAL GERAL DR. CESAR	extravazamento de contraste na	Enviada	Eventos Adversos Relacionados à	Falhas envolvendo cateter venoso		
19/10/2019	CE	FORTALEZA	HOSPITAL GERAL DR. CESAR	-	Enviada	Eventos Adversos Relacionados à	Falhas envolvendo cateter venoso		
19/10/2019	CE	FORTALEZA	HOSPITAL GERAL DR. CESAR	Nutrição parenteral com outras	Enviada	Eventos Adversos Relacionados à	Falhas durante a assistência à saúde		
19/10/2019	CE	FORTALEZA	HOSPITAL GERAL DR. CESAR	trocado o RN que seria o exame	Enviada	Eventos Adversos Relacionados à	Falhas ocorridas em laboratóri		
19/10/2019	CE	FORTALEZA	HOSPITAL GERAL DR. CESAR	preparo para colonoscc opia	Enviada	Eventos Adversos Relacionados à	Falhas durante a assistência à saúde		
19/10/2019	CE	FORTALEZA	HOSPITAL GERAL DR. CESAR	paciente agitado e extubou e	Enviada	Eventos Adversos Relacionados à	Extubação endotraqueal		
19/10/2019	CE	FORTALEZA	HOSPITAL GERAL DR. CESAR	paciente extubou pós moviment	Enviada	Eventos Adversos Relacionados à	Extubação endotraqueal		
19/10/2019	CE	FORTALEZA	HOSPITAL GERAL DR. CESAR	transfusão realizada com dados	Enviada	Eventos Adversos Relacionados à	Falha na documentação		
19/10/2019	CE	FORTALEZA	HOSPITAL GERAL DR. CESAR	pacientwe puxou o tubo endotraqu	Enviada	Eventos Adversos Relacionados à	Extubação endotraqueal		
19/10/2019	CE	FORTALEZA	HOSPITAL GERAL DR. CESAR	paciente ao tentar levar desequilib	Enviada	Eventos Adversos Relacionados à	Queda do paciente		
19/10/2019	CE	FORTALEZA	HOSPITAL GERAL DR. CESAR	admitido na UTI adulto com	Enviada	Eventos Adversos Relacionados à	Lesão por pressão		
19/10/2019	CE	FORTALEZA	HOSPITAL GERAL DR. CESAR	-	Enviada	Eventos Adversos Relacionados à	Lesão por pressão		

19/10/2019	CE	FORTALEZA	HOSPITAL GERAL DR. CESAR	lesão na região occipital	Enviada	Eventos Adversos Relacionados à	Lesão por pressão		
19/10/2019	CE	FORTALEZA	HOSPITAL GERAL DR. CESAR	admitido com LP	Enviada	Eventos Adversos Relacionados à	Lesão por pressão		
19/10/2019	CE	FORTALEZA	HOSPITAL GERAL DR. CESAR	exteriorização de PICC	Enviada	Eventos Adversos Relacionados à	Falhas envolvendo o cateter venoso		
19/10/2019	CE	FORTALEZA	HOSPITAL GERAL DR. CESAR	exteriorização de TQT	Enviada	Eventos Adversos Relacionados à	Falhas durante a assistência à saúde		
25/10/2019	CE	FORTALEZA	HOSPITAL GERAL DR. CESAR	uso inadequado do picc	Enviada	Eventos Adversos Relacionados à	Falhas envolvendo o cateter venoso		
25/10/2019	CE	FORTALEZA	HOSPITAL GERAL DR. CESAR	uso de grampeador e ocorreu	Enviada	Eventos Adversos Relacionados à	Falhas durante procedimento		
26/10/2019	CE	FORTALEZA	HOSPITAL GERAL DR. CESAR	instado NPT em single lumem e	Enviada	Eventos Adversos Relacionados à	Falhas durante a assistência à saúde		
11/01/2020	CE	FORTALEZA	HOSPITAL GERAL DR. CESAR	exame de TC de craneo enviado	Enviada	Eventos Adversos Relacionados à	Falha na identificação do paciente		
11/01/2020	CE	FORTALEZA	HOSPITAL GERAL DR. CESAR	perda de PICC por obstrução	Enviada	Eventos Adversos Relacionados à	Falhas envolvendo o cateter venoso		
11/01/2020	CE	FORTALEZA	HOSPITAL GERAL DR. CESAR	faltade bomba de infusão	Enviada	Eventos Adversos Relacionados à	Falhas durante a assistência à saúde		
11/01/2020	CE	FORTALEZA	HOSPITAL GERAL DR. CESAR	exteriorização de cateter PICC	Enviada	Eventos Adversos Relacionados à	Falhas envolvendo o cateter venoso		
11/01/2020	CE	FORTALEZA	HOSPITAL GERAL DR. CESAR	marcado cirurgia sem contactar	Enviada	Eventos Adversos Relacionados à	Falhas durante a assistência à saúde		
29/02/2020	CE	FORTALEZA	HOSPITAL GERAL DR. CESAR	descarte da bolsa de sangue	Enviada	Eventos Adversos Relacionados à	Falhas durante a assistência à saúde		
29/02/2020	CE	FORTALEZA	HOSPITAL GERAL DR. CESAR	paciente veio para UTI com DVA em	Enviada	Eventos Adversos Relacionados à	Falhas durante a assistência à saúde		
29/02/2020	CE	FORTALEZA	HOSPITAL GERAL DR. CESAR	amputação agendada porem o	Enviada	Eventos Adversos Relacionados à	Falhas durante a assistência à saúde		

29/02/2020	CE	FORTALEZA	HOSPITAL GERAL DR. CESAR	Perda do PICC por eteriorização e	Enviada	Eventos Adversos Relacionados à	Falhas envolvendo cateter venoso		
29/02/2020	CE	FORTALEZA	HOSPITAL GERAL DR. CESAR	queimadura no MMSD por uso	Enviada	Eventos Adversos Relacionados à	Queimadura de paciente		
29/02/2020	CE	FORTALEZA	HOSPITAL GERAL DR. CESAR	Perda da SNE acidentalmente ao	Enviada	Eventos Adversos Relacionados à	Falhas na administração de dietas		Enteral (oral, cateter oral ou
29/02/2020	CE	FORTALEZA	HOSPITAL GERAL DR. CESAR	Alteração da prescrição médica	Enviada	Eventos Adversos Relacionados à	Falhas na administração de dietas		Parenteral (via intravenosa)
29/02/2020	CE	FORTALEZA	HOSPITAL GERAL DR. CESAR	falha na comunicação envolvido	Enviada	Eventos Adversos Relacionados à	Falhas durante a assistência à saúde		
29/02/2020	CE	FORTALEZA	HOSPITAL GERAL DR. CESAR	Cirurgia prolongada a por curto no	Enviada	Eventos Adversos Relacionados à	Falhas durante a assistência à saúde		
29/02/2020	CE	FORTALEZA	HOSPITAL GERAL DR. CESAR	Extubação pós manuseio	Enviada	Eventos Adversos Relacionados à	Extubação endotraqueal		
29/02/2020	CE	FORTALEZA	HOSPITAL GERAL DR. CESAR	NA administração de hemocom	Enviada	Eventos Adversos Relacionados à	Falhas envolvendo cateter venoso		
29/02/2020	CE	FORTALEZA	HOSPITAL GERAL DR. CESAR	Cirurgião de plantão fez	Enviada	Eventos Adversos Relacionados à	Falhas durante a assistência à saúde		
29/02/2020	CE	FORTALEZA	HOSPITAL GERAL DR. CESAR	Paciente admitda da SRA com	Enviada	Eventos Adversos Relacionados à	Falhas envolvendo cateter venoso		
29/02/2020	CE	FORTALEZA	HOSPITAL GERAL DR. CESAR	Instalado ATB em BI porem sem	Enviada	Eventos Adversos Relacionados à	Falhas durante a assistência à saúde		
29/02/2020	CE	FORTALEZA	HOSPITAL GERAL DR. CESAR	Paciente admitido com sinais de	Enviada	Eventos Adversos Relacionados à	Falhas durante a assistência à saúde		
29/02/2020	CE	FORTALEZA	HOSPITAL GERAL DR. CESAR	Instalado NPT em bomba de	Enviada	Eventos Adversos Relacionados à	Falhas na administração de dietas		Parenteral (via intravenosa)
29/02/2020	CE	FORTALEZA	HOSPITAL GERAL DR. CESAR	Pte agendado e jejum de 12	Enviada	Eventos Adversos Relacionados à	Falhas durante a assistência à saúde		
06/03/2020	CE	FORTALEZA	HOSPITAL GERAL DR. CESAR	Perda dp PICC por exteriorização de	Enviada	Eventos Adversos Relacionados à	Falhas durante a assistência à saúde		

29/05/2020	CE	FORTALEZA	HOSPITAL GERAL DR. CESAR	Falah na alta ao entregar de RN a	Enviada	Eventos Adversos Relacionados à	Falha na identificação do paciente		
18/08/2020	CE	FORTALEZA	HOSPITAL GERAL DR. CESAR	Desaparecimento pós aborto do	Enviada	Eventos Adversos Relacionados à	Falhas durante a assistência à saúde		
18/08/2020	CE	FORTALEZA	HOSPITAL GERAL DR. CESAR	Ao levar a noite para ir banheiro	Enviada	Eventos Adversos Relacionados à	Queda do paciente		
18/08/2020	CE	FORTALEZA	HOSPITAL GERAL DR. CESAR	lesão em calcaneo e regiao sacra exp	Enviada	Eventos Adversos Relacionados à	Lesão por pressão		
18/08/2020	CE	FORTALEZA	HOSPITAL GERAL DR. CESAR	paciente puxou o TOT	Enviada	Eventos Adversos Relacionados à	Extubação endotraqueal		
18/08/2020	CE	FORTALEZA	HOSPITAL GERAL DR. CESAR	troca de curativo sem descrição	Enviada	Eventos Adversos Relacionados à	Lesão por pressão		
18/08/2020	CE	FORTALEZA	HOSPITAL GERAL DR. CESAR	IDENTIFICAÇÃO DA NPT NAO	Enviada	Eventos Adversos Relacionados à	Falha na identificação do paciente		
18/08/2020	CE	FORTALEZA	HOSPITAL GERAL DR. CESAR	PACIENTE CHEGOU AO CCG	Enviada	Eventos Adversos Relacionados à	Falha na identificação do paciente		
18/08/2020	CE	FORTALEZA	HOSPITAL GERAL DR. CESAR	desvio de amostra pós drenagem	Enviada	Eventos Adversos Relacionados à	Falhas ocorridas em laboratóri		
18/08/2020	CE	FORTALEZA	HOSPITAL GERAL DR. CESAR	Chão molhado ao redor da cama	Enviada	Eventos Adversos Relacionados à	Queda do paciente		
18/08/2020	CE	FORTALEZA	HOSPITAL GERAL DR. CESAR	durante procedimento de EDO	Enviada	Eventos Adversos Relacionados à	Falhas durante procedimento		
19/08/2020	CE	FORTALEZA	HOSPITAL GERAL DR. CESAR	Ao retornar do banheiro	Enviada	Eventos Adversos Relacionados à	Queda do paciente		
19/08/2020	CE	FORTALEZA	HOSPITAL GERAL DR. CESAR	lesão por pressão na região sacral	Enviada	Eventos Adversos Relacionados à	Lesão por pressão		
22/08/2020	CE	FORTALEZA	HOSPITAL GERAL DR. CESAR	Não foi prescrito portanto nem	Enviada	Eventos Adversos Relacionados à	Falhas na administração de dietas		Parenteral (via intravenosa)
22/08/2020	CE	FORTALEZA	HOSPITAL GERAL DR. CESAR	paciente evoui com lesão	Enviada	Eventos Adversos Relacionados à	Lesão por pressão		

22/08/2020	CE	FORTALEZA	HOSPITAL GERAL DR. CESAR	A NPT chegou a unidades quatro	Enviada	Eventos Adversos Relacionados à	Falhas na administração de dietas		Parenteral (via intravenosa)
22/08/2020	CE	FORTALEZA	HOSPITAL GERAL DR. CESAR	presença~ de LP em região sacra	Enviada	Eventos Adversos Relacionados à	Lesão por pressão		
22/08/2020	CE	FORTALEZA	HOSPITAL GERAL DR. CESAR	A NPT prescrita chegou quatro	Enviada	Eventos Adversos Relacionados à	Falhas na administração de dietas		Parenteral (via intravenosa)
22/08/2020	CE	FORTALEZA	HOSPITAL GERAL DR. CESAR	LESÃO POR PRESSÃO EM	Enviada	Eventos Adversos Relacionados à	Lesão por pressão		
22/08/2020	CE	FORTALEZA	HOSPITAL GERAL DR. CESAR	PACIENTE EXTUBOU	Enviada	Eventos Adversos Relacionados à	Extubação endotraqueal		
22/08/2020	CE	FORTALEZA	HOSPITAL GERAL DR. CESAR	Avaliado alguns dias pos admissão	Enviada	Eventos Adversos Relacionados à	Lesão por pressão		
22/08/2020	CE	FORTALEZA	HOSPITAL GERAL DR. CESAR	LP , ESTAGIO III EM REGIÃO	Enviada	Eventos Adversos Relacionados à	Lesão por pressão		
22/08/2020	CE	FORTALEZA	HOSPITAL GERAL DR. CESAR	REALIZADO DESBRIDAMENTO	Enviada	Eventos Adversos Relacionados à	Falhas durante a assistência à saúde		
22/08/2020	CE	FORTALEZA	HOSPITAL GERAL DR. CESAR	PACIENTE COM LPEM CALCAN	Enviada	Eventos Adversos Relacionados à	Lesão por pressão		
22/08/2020	CE	FORTALEZA	HOSPITAL GERAL DR. CESAR	PACIENTE FOI ADMITIDO COM	Enviada	Eventos Adversos Relacionados à	Lesão por pressão		
22/08/2020	CE	FORTALEZA	HOSPITAL GERAL DR. CESAR	apos realizar TC foi entregue	Enviada	Eventos Adversos Relacionados à	Falha na identificação do paciente		
22/08/2020	CE	FORTALEZA	HOSPITAL GERAL DR. CESAR	LP NAO CLASSIFICAVEL EM	Enviada	Eventos Adversos Relacionados à	Lesão por pressão		
22/08/2020	CE	FORTALEZA	HOSPITAL GERAL DR. CESAR	paciente coM LP estagio II em	Enviada	Eventos Adversos Relacionados à	Lesão por pressão		
22/08/2020	CE	FORTALEZA	HOSPITAL GERAL DR. CESAR	paciente com LP em regiao	Enviada	Eventos Adversos Relacionados à	Lesão por pressão		
22/08/2020	CE	FORTALEZA	HOSPITAL GERAL DR. CESAR	Paciente co LPem região sacra	Enviada	Eventos Adversos Relacionados à	Lesão por pressão		

22/08/2020	CE	FORTALEZA	HOSPITAL GERAL DR. CESAR	paciente LP com perda de epiderme	Enviada	Eventos Adversos Relacionados à	Lesão por pressão		
22/08/2020	CE	FORTALEZA	HOSPITAL GERAL DR. CESAR	Paciente apresenta LP estagioIII	Enviada	Eventos Adversos Relacionados à	Lesão por pressão		
22/08/2020	CE	FORTALEZA	HOSPITAL GERAL DR. CESAR	PAACIENTE CO LESÃO EM	Enviada	Eventos Adversos Relacionados à	Lesão por pressão		
22/08/2020	CE	FORTALEZA	HOSPITAL GERAL DR. CESAR	PAPaciente co LP ,ESTAGIO II em	Enviada	Eventos Adversos Relacionados à	Lesão por pressão		
22/08/2020	CE	FORTALEZA	HOSPITAL GERAL DR. CESAR	paciente co LP em região sacra	Enviada	Eventos Adversos Relacionados à	Lesão por pressão		
22/08/2020	CE	FORTALEZA	HOSPITAL GERAL DR. CESAR	LP EM ESTAGIO INDETER	Enviada	Eventos Adversos Relacionados à	Lesão por pressão		
22/08/2020	CE	FORTALEZA	HOSPITAL GERAL DR. CESAR	PACIENTE POR LP EM REGIÃO	Enviada	Eventos Adversos Relacionados à	Lesão por pressão		
22/08/2020	CE	FORTALEZA	HOSPITAL GERAL DR. CESAR	PACIENTE APRESENTA LP	Enviada	Eventos Adversos Relacionados à	Lesão por pressão		
22/08/2020	CE	FORTALEZA	HOSPITAL GERAL DR. CESAR	LP em região sacra e lesão em	Enviada	Eventos Adversos Relacionados à	Lesão por pressão		
22/08/2020	CE	FORTALEZA	HOSPITAL GERAL DR. CESAR	LP em região sacra, estagio II	Enviada	Eventos Adversos Relacionados à	Lesão por pressão		
22/08/2020	CE	FORTALEZA	HOSPITAL GERAL DR. CESAR	Paciente troca de roupa retira	Enviada	Eventos Adversos Relacionados à	Evasão do paciente		
22/08/2020	CE	FORTALEZA	HOSPITAL GERAL DR. CESAR	falta evolução multiprofissionais e	Enviada	Eventos Adversos Relacionados à	Falha na documentação		
22/08/2020	CE	FORTALEZA	HOSPITAL GERAL DR. CESAR	RN realizou PICC e a rotina, é	Enviada	Eventos Adversos Relacionados à	Falhas durante a assistência à saúde		
22/08/2020	CE	FORTALEZA	HOSPITAL GERAL DR. CESAR	procedimento invasivo no local	Enviada	Eventos Adversos Relacionados à	Falhas durante a assistência à saúde		
22/08/2020	CE	FORTALEZA	HOSPITAL GERAL DR. CESAR	falta de assinatura medica na	Enviada	Eventos Adversos Relacionados à	Falha na documentação		

22/08/2020	CE	FORTALEZA	HOSPITAL GERAL DR. CESAR	Instaldo NPT em PICC quando	Enviada	Eventos Adversos Relacionados à	Falhas durante a assistência à saúde		
22/08/2020	CE	FORTALEZA	HOSPITAL GERAL DR. CESAR	choque eletrico com leve queimadu	Enviada	Eventos Adversos Relacionados à	Falhas nas atividades		
22/08/2020	CE	FORTALEZA	HOSPITAL GERAL DR. CESAR	Administrado farmaco no rasto	Enviada	Eventos Adversos Relacionados à	Falhas durante a assistência à saúde		
22/08/2020	CE	FORTALEZA	HOSPITAL GERAL DR. CESAR	PACIENTE ENCAMINHADO	Enviada	Eventos Adversos Relacionados à	Falha na identificação do paciente		
25/08/2020	CE	FORTALEZA	HOSPITAL GERAL DR. CESAR	Na passagem de plantão	Enviada	Eventos Adversos Relacionados à	Extubação endotraqueal		
25/08/2020	CE	FORTALEZA	HOSPITAL GERAL DR. CESAR	Percebido as 6h que o paciente	Enviada	Eventos Adversos Relacionados à	Evasão do paciente		
07/09/2020	CE	FORTALEZA	HOSPITAL GERAL DR. CESAR	-	Enviada	Eventos Adversos Relacionados à	Falha na identificação do paciente		
07/09/2020	CE	FORTALEZA	HOSPITAL GERAL DR. CESAR	paciente foi so ao banheiro, sem	Enviada	Eventos Adversos Relacionados à	Queda do paciente		
07/09/2020	CE	FORTALEZA	HOSPITAL GERAL DR. CESAR	-	Enviada	Eventos Adversos Relacionados à	Queda do paciente		
07/09/2020	CE	FORTALEZA	HOSPITAL GERAL DR. CESAR	paciente extubação acindental	Enviada	Eventos Adversos Relacionados à	Falhas durante a assistência à saúde		
07/09/2020	CE	FORTALEZA	HOSPITAL GERAL DR. CESAR	foi ao banheiro sem comunicar	Enviada	Eventos Adversos Relacionados à	Queda do paciente		
07/09/2020	CE	FORTALEZA	HOSPITAL GERAL DR. CESAR	INDICADO TROCA	Enviada	Eventos Adversos Relacionados à	Falhas durante a assistência à saúde		
07/09/2020	CE	FORTALEZA	HOSPITAL GERAL DR. CESAR	-	Enviada	Eventos Adversos Relacionados à	Falhas durante a assistência à saúde		
07/09/2020	CE	FORTALEZA	HOSPITAL GERAL DR. CESAR	-	Enviada	Eventos Adversos Relacionados à	Falhas nas atividades		
07/09/2020	CE	FORTALEZA	HOSPITAL GERAL DR. CESAR	PRESCRIÇÃO DE NP CHEGAN	Enviada	Eventos Adversos Relacionados à	Falha na documentação		

07/09/2020	CE	FORTALEZA	HOSPITAL GERAL DR. CESAR	PERDEU ACESSO VENOSO-PICC	Enviada	Eventos Adversos Relacionados à	Falhas durante a assistência à saúde		
07/09/2020	CE	FORTALEZA	HOSPITAL GERAL DR. CESAR	No início do plantão noturno	Enviada	Eventos Adversos Relacionados à	Falhas envolvendo cateter venoso		
19/09/2020	CE	FORTALEZA	HOSPITAL GERAL DR. CESAR	perfuração de vias b	Enviada	Eventos Adversos Relacionados à	Falhas durante procedimento		
19/09/2020	CE	FORTALEZA	HOSPITAL GERAL DR. CESAR	-	Enviada	Eventos Adversos Relacionados à	Falhas durante procedimento		
19/09/2020	CE	FORTALEZA	HOSPITAL GERAL DR. CESAR	-	Enviada	Eventos Adversos Relacionados à	Falhas durante procedimento		
31/10/2020	CE	FORTALEZA	HOSPITAL GERAL DR. CESAR	dificuldade de punção pelo	Enviada	Eventos Adversos Relacionados à	Falhas envolvendo cateter venoso		
31/10/2020	CE	FORTALEZA	HOSPITAL GERAL DR. CESAR	paciente veio transferido de	Enviada	Eventos Adversos Relacionados à	Falhas durante a assistência à saúde		
31/10/2020	CE	FORTALEZA	HOSPITAL GERAL DR. CESAR	Observado lesão na pele do RN ao	Enviada	Eventos Adversos Relacionados à	Falhas envolvendo cateter venoso		
31/10/2020	CE	FORTALEZA	HOSPITAL GERAL DR. CESAR	EQUIPE DA ESTOMATERAPIA	Enviada	Eventos Adversos Relacionados à	Lesão por pressão		
31/10/2020	CE	FORTALEZA	HOSPITAL GERAL DR. CESAR	paciente em mesa cirurgica se	Enviada	Eventos Adversos Relacionados à	Queda do paciente		
31/10/2020	CE	FORTALEZA	HOSPITAL GERAL DR. CESAR	LP na regiao sacral e parietal	Enviada	Eventos Adversos Relacionados à	Lesão por pressão		
31/10/2020	CE	FORTALEZA	HOSPITAL GERAL DR. CESAR	paciente retirou tubo orotraquia	Enviada	Eventos Adversos Relacionados à	Extubação endotraqueal		
31/10/2020	CE	FORTALEZA	HOSPITAL GERAL DR. CESAR	Término da NPT antes do prescrito	Enviada	Eventos Adversos Relacionados à	Falhas na administração de dietas		Parenteral (via intravenosa)
31/10/2020	CE	FORTALEZA	HOSPITAL GERAL DR. CESAR	ruptura do balão no picc	Enviada	Eventos Adversos Relacionados à	Falhas durante a assistência à saúde		
31/10/2020	CE	FORTALEZA	HOSPITAL GERAL DR. CESAR	LP necrotizante no pé D RN	Enviada	Eventos Adversos Relacionados à	Lesão por pressão		

31/10/2020	CE	FORTALEZA	HOSPITAL GERAL DR. CESAR	paciente foi recebido no ccg	Enviada	Eventos Adversos Relacionados à	Falhas durante a assistência à saúde		
31/10/2020	CE	FORTALEZA	HOSPITAL GERAL DR. CESAR	Observado sinais e sintomas de flebite	Enviada	Eventos Adversos Relacionados à	Falhas envolvendo cateter venoso		
31/10/2020	CE	FORTALEZA	HOSPITAL GERAL DR. CESAR	FALHA NA ADMINISTRAÇÃO	Enviada	Eventos Adversos Relacionados à	Falhas durante a assistência à saúde		
31/10/2020	CE	FORTALEZA	HOSPITAL GERAL DR. CESAR	exames trocados	Enviada	Eventos Adversos Relacionados à	Falha na identificação do paciente		
31/10/2020	CE	FORTALEZA	HOSPITAL GERAL DR. CESAR	PACIENTE LEVANTOU-SE	Enviada	Eventos Adversos Relacionados à	Queda do paciente		
31/10/2020	CE	FORTALEZA	HOSPITAL GERAL DR. CESAR	Não foi enviado a prescrição medica	Enviada	Eventos Adversos Relacionados à	Falhas na administração de dietas		Parenteral (via intravenosa)
31/10/2020	CE	FORTALEZA	HOSPITAL GERAL DR. CESAR	PACIENTE COM LPP EM REGIÃO	Enviada	Eventos Adversos Relacionados à	Lesão por pressão		
31/10/2020	CE	FORTALEZA	HOSPITAL GERAL DR. CESAR	Perda do acesso venoso por	Enviada	Eventos Adversos Relacionados à	Falhas envolvendo cateter venoso		
31/10/2020	CE	FORTALEZA	HOSPITAL GERAL DR. CESAR	no sistema foi enviado o	Enviada	Eventos Adversos Relacionados à	Falhas nas atividades		
31/10/2020	CE	FORTALEZA	HOSPITAL GERAL DR. CESAR	lesão necrotizante comborda	Enviada	Eventos Adversos Relacionados à	Lesão por pressão		
31/10/2020	CE	FORTALEZA	HOSPITAL GERAL DR. CESAR	foi observado extravasa	Enviada	Eventos Adversos Relacionados à	Falhas envolvendo cateter venoso		
31/10/2020	CE	FORTALEZA	HOSPITAL GERAL DR. CESAR	admitido no sistema RN em	Enviada	Eventos Adversos Relacionados à	Falhas nas atividades		
31/10/2020	CE	FORTALEZA	HOSPITAL GERAL DR. CESAR	atraso na dieta do RN de uma hora	Enviada	Eventos Adversos Relacionados à	Falhas na administração de dietas		Enteral (oral, cateter oral ou
31/10/2020	CE	FORTALEZA	HOSPITAL GERAL DR. CESAR	No momento da intubação	Enviada	Eventos Adversos Relacionados à	Falhas durante a assistência à saúde		
31/10/2020	CE	FORTALEZA	HOSPITAL GERAL DR. CESAR	RN APRESENTA LESÃO	Enviada	Eventos Adversos Relacionados à	Falhas durante a assistência à saúde		

31/10/2020	CE	FORTALEZA	HOSPITAL GERAL DR. CESAR	lesão sacral com bordas	Enviada	Eventos Adversos Relacionados à	Lesão por pressão		
31/10/2020	CE	FORTALEZA	HOSPITAL GERAL DR. CESAR	PRESCRIÇAO DE DIETA DE RN	Enviada	Eventos Adversos Relacionados à	Falhas na administração de dietas		Enteral (oral, cateter oral ou
31/10/2020	CE	FORTALEZA	HOSPITAL GERAL DR. CESAR	PACIENTE ADMITIDO COM	Enviada	Eventos Adversos Relacionados à	Lesão por pressão		
31/10/2020	CE	FORTALEZA	HOSPITAL GERAL DR. CESAR	infusão de bomba diuretica	Enviada	Eventos Adversos Relacionados à	Lesão por pressão		
31/10/2020	CE	FORTALEZA	HOSPITAL GERAL DR. CESAR	RN APRESENTOU LESÃO	Enviada	Eventos Adversos Relacionados à	Falhas durante a assistência à saúde		
31/10/2020	CE	FORTALEZA	HOSPITAL GERAL DR. CESAR	realizado lavagem de cavidade	Enviada	Eventos Adversos Relacionados à	Falhas durante procedimento		
31/10/2020	CE	FORTALEZA	HOSPITAL GERAL DR. CESAR	LPP EM REGIÃO SACRAL E EM	Enviada	Eventos Adversos Relacionados à	Lesão por pressão		
31/10/2020	CE	FORTALEZA	HOSPITAL GERAL DR. CESAR	PRESCRIÇÃO AS 14HS E AS 21HS	Enviada	Eventos Adversos Relacionados à	Falhas na administração de dietas		Enteral (oral, cateter oral ou
31/10/2020	CE	FORTALEZA	HOSPITAL GERAL DR. CESAR	Residente da cirurgia puncionou	Enviada	Eventos Adversos Relacionados à	Falhas durante a assistência à saúde		
28/11/2020	CE	FORTALEZA	HOSPITAL GERAL DR. CESAR	agendados duas cirurgias com um	Enviada	Eventos Adversos Relacionados à	Falhas durante a assistência à saúde		
28/11/2020	CE	FORTALEZA	HOSPITAL GERAL DR. CESAR	alta foi entregue DNV trocada	Enviada	Eventos Adversos Relacionados à	Falhas nas atividades		
28/11/2020	CE	FORTALEZA	HOSPITAL GERAL DR. CESAR	Ao receber alta, percebeu	Enviada	Eventos Adversos Relacionados à	Falhas nas atividades		
28/11/2020	CE	FORTALEZA	HOSPITAL GERAL DR. CESAR	Relato da residente sobre o uso do	Enviada	Eventos Adversos Relacionados à	Falhas nas atividades		
28/11/2020	CE	FORTALEZA	HOSPITAL GERAL DR. CESAR	As10(dez) NPT dos RNs chegaram	Enviada	Eventos Adversos Relacionados à	Falhas na administração de dietas		Parenteral (via intravenosa)
30/11/2020	CE	FORTALEZA	HOSPITAL GERAL DR. CESAR	PRESCRIÇÃO DE NP CHEGAN	Enviada	Eventos Adversos Relacionados à	Falha na documentação		

30/11/2020	CE	FORTALEZA	HOSPITAL GERAL DR. CESAR	RN gemelar com identificac	Enviada	Eventos Adversos Relacionados à	Falha na identificação do paciente		
05/12/2020	CE	FORTALEZA	HOSPITAL GERAL DR. CESAR	Na extubação acidental do RN, o	Enviada	Eventos Adversos Relacionados à	Lesão por pressão		
05/12/2020	CE	FORTALEZA	HOSPITAL GERAL DR. CESAR	Pte apresentando hiposatura	Enviada	Eventos Adversos Relacionados à	Falhas durante a assistência à saúde		
05/12/2020	CE	FORTALEZA	HOSPITAL GERAL DR. CESAR	Pte agendado e preparado	Enviada	Eventos Adversos Relacionados à	Falhas durante a assistência à saúde		
05/12/2020	CE	FORTALEZA	HOSPITAL GERAL DR. CESAR	Paciente obesa, durante a intubação	Enviada	Eventos Adversos Relacionados à	Falhas durante a assistência à saúde		
05/12/2020	CE	FORTALEZA	HOSPITAL GERAL DR. CESAR	Paciente admitido na UTI sem	Enviada	Eventos Adversos Relacionados à	Falhas nas atividades		
05/12/2020	CE	FORTALEZA	HOSPITAL GERAL DR. CESAR	Paciente idoso, tentou levantar	Enviada	Eventos Adversos Relacionados à	Queda do paciente		
05/12/2020	CE	FORTALEZA	HOSPITAL GERAL DR. CESAR	Lesão região sacral e calcâneo	Enviada	Eventos Adversos Relacionados à	Lesão por pressão		
05/12/2020	CE	FORTALEZA	HOSPITAL GERAL DR. CESAR	Lesão por pressão sem	Enviada	Eventos Adversos Relacionados à	Falhas durante a assistência à saúde		
05/12/2020	CE	FORTALEZA	HOSPITAL GERAL DR. CESAR	RN prematuro extremo com dois	Enviada	Eventos Adversos Relacionados à	Lesão por pressão		
12/12/2020	CE	FORTALEZA	HOSPITAL GERAL DR. CESAR	Paciente com exames marcado	Enviada	Eventos Adversos Relacionados à	Falhas na administração de dietas		Enteral (oral, cateter oral ou
12/12/2020	CE	FORTALEZA	HOSPITAL GERAL DR. CESAR	Coletado sangue para exames	Enviada	Eventos Adversos Relacionados à	Falhas ocorridas em laboratóri		
12/12/2020	CE	FORTALEZA	HOSPITAL GERAL DR. CESAR	Averição da PA nos RN com	Enviada	Eventos Adversos Relacionados à	Falhas durante a assistência à saúde		
12/12/2020	CE	FORTALEZA	HOSPITAL GERAL DR. CESAR	Curativos na LPP na região do	Enviada	Eventos Adversos Relacionados à	Lesão por pressão		
16/12/2020	CE	FORTALEZA	HOSPITAL GERAL DR. CESAR	Paciente admitido procedente da UTI	Enviada	Eventos Adversos Relacionados à	Queda do paciente		

09/01/2021	CE	FORTALEZA	HOSPITAL GERAL DR. CESAR	lesão no MMSD por extravaza	Enviada	Eventos Adversos Relacionados à	Lesão por pressão		
09/01/2021	CE	FORTALEZA	HOSPITAL GERAL DR. CESAR	apresenta lpp em região occipital	Enviada	Eventos Adversos Relacionados à	Lesão por pressão		
09/01/2021	CE	FORTALEZA	HOSPITAL GERAL DR. CESAR	lesão em regiao frontal decorrent	Enviada	Eventos Adversos Relacionados à	Lesão por pressão		
09/01/2021	CE	FORTALEZA	HOSPITAL GERAL DR. CESAR	Lesão na região sacral e dorso do	Enviada	Eventos Adversos Relacionados à	Lesão por pressão		
09/01/2021	CE	FORTALEZA	HOSPITAL GERAL DR. CESAR	procedimento invasivo seria na	Enviada	Eventos Adversos Relacionados à	Falhas durante a assistência à saúde		
09/01/2021	CE	FORTALEZA	HOSPITAL GERAL DR. CESAR	Lesão na região do calcaneo E não	Enviada	Eventos Adversos Relacionados à	Lesão por pressão		
09/01/2021	CE	FORTALEZA	HOSPITAL GERAL DR. CESAR	lesão de queimadura em abdomem	Enviada	Eventos Adversos Relacionados à	Falhas durante a assistência à saúde		
09/01/2021	CE	FORTALEZA	HOSPITAL GERAL DR. CESAR	lpp em região sacra, estagio	Enviada	Eventos Adversos Relacionados à	Lesão por pressão		
09/01/2021	CE	FORTALEZA	HOSPITAL GERAL DR. CESAR	Lesão sacral não classificav	Enviada	Eventos Adversos Relacionados à	Lesão por pressão		
09/01/2021	CE	FORTALEZA	HOSPITAL GERAL DR. CESAR	Lesão na região sacral não	Enviada	Eventos Adversos Relacionados à	Lesão por pressão		
09/01/2021	CE	FORTALEZA	HOSPITAL GERAL DR. CESAR	utilizando bolsa coletora de urina	Enviada	Eventos Adversos Relacionados à	Falhas durante a assistência à saúde		
09/01/2021	CE	FORTALEZA	HOSPITAL GERAL DR. CESAR	Lesão na região sacral com	Enviada	Eventos Adversos Relacionados à	Lesão por pressão		
09/01/2021	CE	FORTALEZA	HOSPITAL GERAL DR. CESAR	lpp em região ociptal, presença	Enviada	Eventos Adversos Relacionados à	Lesão por pressão		
09/01/2021	CE	FORTALEZA	HOSPITAL GERAL DR. CESAR	paciente desorientado, puxou	Enviada	Eventos Adversos Relacionados à	Falhas durante a assistência à saúde		
09/01/2021	CE	FORTALEZA	HOSPITAL GERAL DR. CESAR	cateter central sem fixação a	Enviada	Eventos Adversos Relacionados à	Falhas envolvendo o cateter venoso		

09/01/2021	CE	FORTALEZA	HOSPITAL GERAL DR. CESAR	lesão por pressão decorrente do	Enviada	Eventos Adversos Relacionados à	Lesão por pressão		
09/01/2021	CE	FORTALEZA	HOSPITAL GERAL DR. CESAR	Lesão na região occipital	Enviada	Eventos Adversos Relacionados à	Lesão por pressão		
09/01/2021	CE	FORTALEZA	HOSPITAL GERAL DR. CESAR	paciente com seu RN AMAMEN	Enviada	Eventos Adversos Relacionados à	Queda do paciente		
23/01/2021	CE	FORTALEZA	HOSPITAL GERAL DR. CESAR	Puerpera caminhando com seu RN	Enviada	Eventos Adversos Relacionados à	Falhas durante a assistência à saúde		
23/01/2021	CE	FORTALEZA	HOSPITAL GERAL DR. CESAR	-	Enviada	Eventos Adversos Relacionados à	Falhas durante a assistência à saúde		
23/01/2021	CE	FORTALEZA	HOSPITAL GERAL DR. CESAR	Prescriçao em dose única e	Enviada	Eventos Adversos Relacionados à	Falhas durante a assistência à saúde		
23/01/2021	CE	FORTALEZA	HOSPITAL GERAL DR. CESAR	rito medicação em dose	Enviada	Eventos Adversos Relacionados à	Falhas durante a assistência à saúde		
23/01/2021	CE	FORTALEZA	HOSPITAL GERAL DR. CESAR	Desperdicio de leite ordenhado. A mãe	Enviada	Eventos Adversos Relacionados à	Falhas na administração de dietas		Enteral (oral, cateter oral ou
23/01/2021	CE	FORTALEZA	HOSPITAL GERAL DR. CESAR	Setor NEO colheu exame	Enviada	Eventos Adversos Relacionados à	Falhas durante a assistência à saúde		
23/01/2021	CE	FORTALEZA	HOSPITAL GERAL DR. CESAR	As 5:50 ocorreu extubação no	Enviada	Eventos Adversos Relacionados à	Extubaçao endotraqueal		
23/01/2021	CE	FORTALEZA	HOSPITAL GERAL DR. CESAR	Prescrito uma medicação sem	Enviada	Eventos Adversos Relacionados à	Falhas durante a assistência à saúde		
23/01/2021	CE	FORTALEZA	HOSPITAL GERAL DR. CESAR	Uso de clorexidina alcoolica	Enviada	Eventos Adversos Relacionados à	Lesão por pressão		
23/01/2021	CE	FORTALEZA	HOSPITAL GERAL DR. CESAR	Lesão sacral e calcaneo por	Enviada	Eventos Adversos Relacionados à	Lesão por pressão		
23/01/2021	CE	FORTALEZA	HOSPITAL GERAL DR. CESAR	-	Enviada	Eventos Adversos Relacionados à	Lesão por pressão		
23/01/2021	CE	FORTALEZA	HOSPITAL GERAL DR. CESAR	Apos realizar Raio X observado	Enviada	Eventos Adversos Relacionados à	Extubaçao endotraqueal		

23/01/2021	CE	FORTALEZA	HOSPITAL GERAL DR. CESAR	-	Enviada	Eventos Adversos Relacionados à	Extubação endotraqueal		
26/06/2021	CE	FORTALEZA	HOSPITAL GERAL DR. CESAR	paciente procedente da UPA	Enviada	Eventos Adversos Relacionados à	Falhas durante a assistência à saúde		
26/06/2021	CE	FORTALEZA	HOSPITAL GERAL DR. CESAR	Lesão por pressão na região	Em investigação	Eventos Adversos Relacionados à	Lesão por pressão		
26/06/2021	CE	FORTALEZA	HOSPITAL GERAL DR. CESAR	paciente veio tranferido de	Enviada	Eventos Adversos Relacionados à	Falhas durante a assistência à saúde		
26/06/2021	CE	FORTALEZA	HOSPITAL GERAL DR. CESAR	Colleta de exame de RN	Enviada	Eventos Adversos Relacionados à	Falhas ocorridas em laboratóri		
26/06/2021	CE	FORTALEZA	HOSPITAL GERAL DR. CESAR	paciente apresenta LPP em região	Enviada	Eventos Adversos Relacionados à	Falhas durante a assistência à saúde		
26/06/2021	CE	FORTALEZA	HOSPITAL GERAL DR. CESAR	LPP em região sacra estagio	Enviada	Eventos Adversos Relacionados à	Lesão por pressão		
26/06/2021	CE	FORTALEZA	HOSPITAL GERAL DR. CESAR	Não administrado medicaçã	Enviada	Eventos Adversos Relacionados à	Falhas durante a assistência à saúde		
26/06/2021	CE	FORTALEZA	HOSPITAL GERAL DR. CESAR	LPP em região sacra estagio III	Em investigação	Eventos Adversos Relacionados à	Lesão por pressão		
26/06/2021	CE	FORTALEZA	HOSPITAL GERAL DR. CESAR	LPP em região sacral e inter	Enviada	Eventos Adversos Relacionados à	Lesão por pressão		
26/06/2021	CE	FORTALEZA	HOSPITAL GERAL DR. CESAR	Não administrado medicaçã	Enviada	Eventos Adversos Relacionados à	Falhas durante a assistência à saúde		
26/06/2021	CE	FORTALEZA	HOSPITAL GERAL DR. CESAR	LPP EM estagio II, REGIÃO OCCIPTA	Enviada	Eventos Adversos Relacionados à	Lesão por pressão		
26/06/2021	CE	FORTALEZA	HOSPITAL GERAL DR. CESAR	lesão por pressão região glutea e	Em investigação	Eventos Adversos Relacionados à	Lesão por pressão		
26/06/2021	CE	FORTALEZA	HOSPITAL GERAL DR. CESAR	Colhido exames laboratoriais de RN	Enviada	Eventos Adversos Relacionados à	Falhas durante a assistência à saúde		
26/06/2021	CE	FORTALEZA	HOSPITAL GERAL DR. CESAR	lesão em região axilar decorrent	Enviada	Eventos Adversos Relacionados à	Falhas envolvendo cateter venoso		

26/06/2021	CE	FORTALEZA	HOSPITAL GERAL DR. CESAR	admitida com LPP, estagio IV REGIÃ	Em investigação	Eventos Adversos Relacionados à	Lesão por pressão		
26/06/2021	CE	FORTALEZA	HOSPITAL GERAL DR. CESAR	ADMITIDA com LPP EM REGIÃO	Em investigação	Eventos Adversos Relacionados à	Lesão por pressão		
26/06/2021	CE	FORTALEZA	HOSPITAL GERAL DR. CESAR	LPP em região sacra, estagio I	Enviada	Eventos Adversos Relacionados à	Lesão por pressão		
26/06/2021	CE	FORTALEZA	HOSPITAL GERAL DR. CESAR	PAPaciente desorientada no	Enviada	Eventos Adversos Relacionados à	Queda do paciente		
26/06/2021	CE	FORTALEZA	HOSPITAL GERAL DR. CESAR	Lesão por pressão na região	Em investigação	Eventos Adversos Relacionados à	Lesão por pressão		
26/06/2021	CE	FORTALEZA	HOSPITAL GERAL DR. CESAR	Admissão com LPP previa em	Em investigação	Eventos Adversos Relacionados à	Lesão por pressão		
26/06/2021	CE	FORTALEZA	HOSPITAL GERAL DR. CESAR	lesão por pressão glúteo com	Enviada	Eventos Adversos Relacionados à	Lesão por pressão		
26/06/2021	CE	FORTALEZA	HOSPITAL GERAL DR. CESAR	LPP EM REGIÃO GLUTEA, ESTAGIO	Enviada	Eventos Adversos Relacionados à	Lesão por pressão		
26/06/2021	CE	FORTALEZA	HOSPITAL GERAL DR. CESAR	LPP EM REGIÃO OCCIPTAL E	Em investigação	Eventos Adversos Relacionados à	Lesão por pressão		
26/06/2021	CE	FORTALEZA	HOSPITAL GERAL DR. CESAR	Lesão na região abdominal por uso	Enviada	Eventos Adversos Relacionados à	Queimadura de paciente		
26/06/2021	CE	FORTALEZA	HOSPITAL GERAL DR. CESAR	Exame agendado p paciente	Enviada	Eventos Adversos Relacionados à	Falhas durante a assistência à saúde		
26/06/2021	CE	FORTALEZA	HOSPITAL GERAL DR. CESAR	LPP em região sacra, estagio II	Enviada	Eventos Adversos Relacionados à	Lesão por pressão		
26/06/2021	CE	FORTALEZA	HOSPITAL GERAL DR. CESAR	Ao tentar deambular para ir ao	Enviada	Eventos Adversos Relacionados à	Queda do paciente		
26/06/2021	CE	FORTALEZA	HOSPITAL GERAL DR. CESAR	lpp em região sacral, estagil III	Em investigação	Eventos Adversos Relacionados à	Lesão por pressão		
26/06/2021	CE	FORTALEZA	HOSPITAL GERAL DR. CESAR	NPT prescrita e não foi manipulad	Enviada	Eventos Adversos Relacionados à	Falhas na administração de dietas		Parenteral (via intravenosa)

29/06/2021	CE	FORTALEZA	HOSPITAL GERAL DR. CESAR	Coleta de rotina pelo laboratóri	Enviada	Eventos Adversos Relacionados à	Falhas durante a assistência à saúde		
29/06/2021	CE	FORTALEZA	HOSPITAL GERAL DR. CESAR	Lesão em RN (região interna	Enviada	Eventos Adversos Relacionados à	Queimadura de paciente		
29/06/2021	CE	FORTALEZA	HOSPITAL GERAL DR. CESAR	Durante punção de acesso	Enviada	Eventos Adversos Relacionados à	Falhas durante a assistência à saúde		
29/06/2021	CE	FORTALEZA	HOSPITAL GERAL DR. CESAR	Puérpera ao visitar seu RN no médio	Enviada	Eventos Adversos Relacionados à	Queda do paciente		
29/06/2021	CE	FORTALEZA	HOSPITAL GERAL DR. CESAR	Pte de 54 anos com preparo para	Enviada	Eventos Adversos Relacionados à	Broncoaspiração		
03/08/2021	CE	FORTALEZA	HOSPITAL GERAL DR. CESAR	As 8h durante avaliação percebido	Enviada	Eventos Adversos Relacionados à	Extubação endotraqueal		
03/08/2021	CE	FORTALEZA	HOSPITAL GERAL DR. CESAR	PACIENTE 45 ANOS POLDE	Em retificação	Eventos Adversos Relacionados à	Falhas durante procedimento		
07/08/2021	CE	FORTALEZA	HOSPITAL GERAL DR. CESAR	Paciente 36 anos admitida na UTI	Em investigação	Eventos Adversos Relacionados à	Falhas envolvendo cateter venoso		
16/10/2021	CE	FORTALEZA	HOSPITAL GERAL DR. CESAR	VEIO ADMITIDA COM LPP	Enviada	Eventos Adversos Relacionados à	Lesão por pressão		
16/10/2021	CE	FORTALEZA	HOSPITAL GERAL DR. CESAR	PACIENTE RN APRESENTA	Enviada	Eventos Adversos Relacionados à	Falhas durante a assistência à saúde		
16/10/2021	CE	FORTALEZA	HOSPITAL GERAL DR. CESAR	LPP EM REGIÃO SACRA, ESTAGIO	Em investigação	Eventos Adversos Relacionados à	Lesão por pressão		
16/10/2021	CE	FORTALEZA	HOSPITAL GERAL DR. CESAR	LPP EM REGIÃO INTERGLUTEA	Enviada	Eventos Adversos Relacionados à	Lesão por pressão		
16/10/2021	CE	FORTALEZA	HOSPITAL GERAL DR. CESAR	LPP em região sacra e calcaneos	Enviada	Eventos Adversos Relacionados à	Lesão por pressão		
16/10/2021	CE	FORTALEZA	HOSPITAL GERAL DR. CESAR	-	Enviada	Eventos Adversos Relacionados à	Lesão por pressão		
16/10/2021	CE	FORTALEZA	HOSPITAL GERAL DR. CESAR	lpp em rgião posterior do torax	Enviada	Eventos Adversos Relacionados à	Lesão por pressão		

06/11/2021	CE	FORTALEZA	HOSPITAL GERAL DR. CESAR	NO MOMENTO DA IDENTIFI	Enviada	Eventos Adversos Relacionados à	Falha na identificação do paciente		
13/11/2021	CE	FORTALEZA	HOSPITAL GERAL DR. CESAR	paciente apresenta lpp em região	Enviada	Eventos Adversos Relacionados à	Lesão por pressão		
13/11/2021	CE	FORTALEZA	HOSPITAL GERAL DR. CESAR	Pte recebeu alta e ao chegar	Enviada	Eventos Adversos Relacionados à	Falhas durante a assistência à saúde		
13/11/2021	CE	FORTALEZA	HOSPITAL GERAL DR. CESAR	PACIENTE APRESENTA LPP	Enviada	Eventos Adversos Relacionados à	Lesão por pressão		
13/11/2021	CE	FORTALEZA	HOSPITAL GERAL DR. CESAR	Pte agitado mesmo sob	Enviada	Eventos Adversos Relacionados à	Extubação endotraqueal		
13/11/2021	CE	FORTALEZA	HOSPITAL GERAL DR. CESAR	Lesão extensa em braço esquerdo	Enviada	Eventos Adversos Relacionados à	Falhas envolvendo o cateter venoso		
13/11/2021	CE	FORTALEZA	HOSPITAL GERAL DR. CESAR	lesão escoriativa na região do	Enviada	Eventos Adversos Relacionados à	Falhas durante a assistência à saúde		
13/11/2021	CE	FORTALEZA	HOSPITAL GERAL DR. CESAR	paciente apresenta lppem região	Enviada	Eventos Adversos Relacionados à	Lesão por pressão		
13/11/2021	CE	FORTALEZA	HOSPITAL GERAL DR. CESAR	PACIENTE APRESENTA	Enviada	Eventos Adversos Relacionados à	Lesão por pressão		
13/11/2021	CE	FORTALEZA	HOSPITAL GERAL DR. CESAR	PACIENTE COM ACESSO VENOSO	Enviada	Eventos Adversos Relacionados à	Falhas durante a assistência à saúde		
13/11/2021	CE	FORTALEZA	HOSPITAL GERAL DR. CESAR	RN APRESENTANDO LPP	Em investigação	Eventos Adversos Relacionados à	Lesão por pressão		
16/11/2021	CE	FORTALEZA	HOSPITAL GERAL DR. CESAR	Pte com agendamento de CRICOTI	Enviada	Eventos Adversos Relacionados à	Falhas durante a assistência à saúde		
16/11/2021	CE	FORTALEZA	HOSPITAL GERAL DR. CESAR	Pte agitada sob sedoanalg	Enviada	Eventos Adversos Relacionados à	Extubação endotraqueal		
17/11/2021	CE	FORTALEZA	HOSPITAL GERAL DR. CESAR	Pte acamada, cabeceira	Em investigação	Eventos Adversos Relacionados à	Broncoaspiração		
30/11/2021	CE	FORTALEZA	HOSPITAL GERAL DR. CESAR	apresenta lpp estagio III em	Em investigação	Eventos Adversos Relacionados à	Lesão por pressão		

30/11/2021	CE	FORTALEZA	HOSPITAL GERAL DR. CESAR	lesão no dedo ocasionado pelo	Enviada	Eventos Adversos Relacionados à	Lesão por pressão		
30/11/2021	CE	FORTALEZA	HOSPITAL GERAL DR. CESAR	apresenta lpp em região occipital	Em investigação	Eventos Adversos Relacionados à	Lesão por pressão		
30/11/2021	CE	FORTALEZA	HOSPITAL GERAL DR. CESAR	Rn com lpp EM MSE com presença	Em investigação	Eventos Adversos Relacionados à	Lesão por pressão		
30/11/2021	CE	FORTALEZA	HOSPITAL GERAL DR. CESAR	lesão de hipo faringe pos	Enviada	Eventos Adversos Relacionados à	Lesão por pressão		
30/11/2021	CE	FORTALEZA	HOSPITAL GERAL DR. CESAR	lpp extensa em região	Enviada	Eventos Adversos Relacionados à	Lesão por pressão		
30/11/2021	CE	FORTALEZA	HOSPITAL GERAL DR. CESAR	-	Enviada	Eventos Adversos Relacionados à	Lesão por pressão		
30/11/2021	CE	FORTALEZA	HOSPITAL GERAL DR. CESAR	lesão em calcaneo esquerdoco	Em investigação	Eventos Adversos Relacionados à	Lesão por pressão		
30/11/2021	CE	FORTALEZA	HOSPITAL GERAL DR. CESAR	queimaduras em abdome inferior	Enviada	Eventos Adversos Relacionados à	Queimadura de paciente		
30/11/2021	CE	FORTALEZA	HOSPITAL GERAL DR. CESAR	lpp em região sacra , estagio III	Em investigação	Eventos Adversos Relacionados à	Lesão por pressão		
30/11/2021	CE	FORTALEZA	HOSPITAL GERAL DR. CESAR	PACIENTE RESTRITA AO	Enviada	Eventos Adversos Relacionados à	Queda do paciente		
04/01/2022	CE	FORTALEZA	HOSPITAL GERAL DR. CESAR	Pte admitida 11/11/21 procedent	Enviada	Eventos Adversos Relacionados à	Lesão por pressão		
04/01/2022	CE	FORTALEZA	HOSPITAL GERAL DR. CESAR	Gestante com HD de placenta	Enviada	Eventos Adversos Relacionados à	Falhas envolvendo o cateter venoso		
04/01/2022	CE	FORTALEZA	HOSPITAL GERAL DR. CESAR	Pte admitida por colelitiase	Enviada	Eventos Adversos Relacionados à	Falhas durante procedimento		
04/01/2022	CE	FORTALEZA	HOSPITAL GERAL DR. CESAR	Pte admitida com Sd colestatic	Enviada	Eventos Adversos Relacionados à	Falhas durante procedimento		
06/01/2022	CE	FORTALEZA	HOSPITAL GERAL DR. CESAR	Pte 20 anos procedente de UPA	Enviada	Eventos Adversos Relacionados à	Falhas durante procedimento		

06/01/2022	CE	FORTALEZA	HOSPITAL GERAL DR. CESAR	Pte evoluindo com LPP sacral na	Enviada	Eventos Adversos Relacionados à	Lesão por pressão		
06/01/2022	CE	FORTALEZA	HOSPITAL GERAL DR. CESAR	Pte apresentou LPP sacral	Enviada	Eventos Adversos Relacionados à	Lesão por pressão		
06/01/2022	CE	FORTALEZA	HOSPITAL GERAL DR. CESAR	Pte gestantede 39 semanas	Enviada	Eventos Adversos Relacionados à	Queda do paciente		
07/01/2022	CE	FORTALEZA	HOSPITAL GERAL DR. CESAR	Material biologico enviado ao	Enviada	Eventos Adversos Relacionados à	Falha na identificação do paciente		
07/01/2022	CE	FORTALEZA	HOSPITAL GERAL DR. CESAR	Residente da cirurgia questiona	Enviada	Eventos Adversos Relacionados à	Falhas durante a assistência à saúde		
07/01/2022	CE	FORTALEZA	HOSPITAL GERAL DR. CESAR	Pte admitido no sistema e	Enviada	Eventos Adversos Relacionados à	Falhas durante a assistência à saúde		
07/01/2022	CE	FORTALEZA	HOSPITAL GERAL DR. CESAR	Pte encaminhada ao centro	Enviada	Eventos Adversos Relacionados à	Falha na identificação do paciente		
07/01/2022	CE	FORTALEZA	HOSPITAL GERAL DR. CESAR	Na visita da equipe estomaterapia ao	Enviada	Eventos Adversos Relacionados à	Lesão por pressão		
07/01/2022	CE	FORTALEZA	HOSPITAL GERAL DR. CESAR	Na avaliação da equipe de	Enviada	Eventos Adversos Relacionados à	Lesão por pressão		
07/01/2022	CE	FORTALEZA	HOSPITAL GERAL DR. CESAR	RN com Hidrocafalia congenita	Enviada	Eventos Adversos Relacionados à	Falhas durante a assistência à saúde		
01/02/2022	CE	FORTALEZA	HOSPITAL GERAL DR. CESAR	Pte evoluiu com LPP no	Concluída	Eventos Adversos Relacionados à	Lesão por pressão		
05/03/2022	CE	FORTALEZA	HOSPITAL GERAL DR. CESAR	paciente apresenta multiplas lesões	Enviada	Eventos Adversos Relacionados à	Lesão por pressão		
05/03/2022	CE	FORTALEZA	HOSPITAL GERAL DR. CESAR	RN na internação e com restrição	Enviada	Eventos Adversos Relacionados à	Lesão por pressão		
05/03/2022	CE	FORTALEZA	HOSPITAL GERAL DR. CESAR	paciente apresenta lpp, estagio 3	Concluída	Eventos Adversos Relacionados à	Lesão por pressão		
05/03/2022	CE	FORTALEZA	HOSPITAL GERAL DR. CESAR	paciente com lpp em trocanter	Concluída	Eventos Adversos Relacionados à	Lesão por pressão		

05/03/2022	CE	FORTALEZA	HOSPITAL GERAL DR. CESAR	Pte admitido iriub=ndo de outro	Concluída	Eventos Adversos Relacionados à	Lesão por pressão			
05/03/2022	CE	FORTALEZA	HOSPITAL GERAL DR. CESAR	paciente ap'1resenta multiplas	Enviada	Eventos Adversos Relacionados à	Lesão por pressão			
05/03/2022	CE	FORTALEZA	HOSPITAL GERAL DR. CESAR	paciente sentada na cama	Enviada	Eventos Adversos Relacionados à	Queda do paciente			
05/03/2022	CE	FORTALEZA	HOSPITAL GERAL DR. CESAR	paciente no trans-operatorio de	Enviada	Eventos Adversos Relacionados à	Acidentes do paciente	Mecanismo térmico (sobreaquecimento		
05/03/2022	CE	FORTALEZA	HOSPITAL GERAL DR. CESAR	RN com acesso venoso por PICC	Enviada	Eventos Adversos Relacionados à	Falhas envolvendo o cateter venoso			
05/03/2022	CE	FORTALEZA	HOSPITAL GERAL DR. CESAR	RN veio transferido da sala de parto	Enviada	Eventos Adversos Relacionados à	Falhas durante a assistência à saúde			
05/03/2022	CE	FORTALEZA	HOSPITAL GERAL DR. CESAR	paciente com lpp, estagio III em	Concluída	Eventos Adversos Relacionados à	Lesão por pressão			
05/03/2022	CE	FORTALEZA	HOSPITAL GERAL DR. CESAR	paciente veio transferido da	Enviada	Eventos Adversos Relacionados à	Lesão por pressão			
05/03/2022	CE	FORTALEZA	HOSPITAL GERAL DR. CESAR	PACIENTE VEIO TRANSFERIDO	Enviada	Eventos Adversos Relacionados à	Lesão por pressão			
05/03/2022	CE	FORTALEZA	HOSPITAL GERAL DR. CESAR	PACIENTE APRESENTA LPP	Enviada	Eventos Adversos Relacionados à	Lesão por pressão			
05/03/2022	CE	FORTALEZA	HOSPITAL GERAL DR. CESAR	PACIENTE TQT, EM MV A 28%	Enviada	Eventos Adversos Relacionados à	Falhas durante a assistência à saúde			
05/03/2022	CE	FORTALEZA	HOSPITAL GERAL DR. CESAR	PACIENTE TOT EM VM, EVLUIU	Enviada	Eventos Adversos Relacionados à	Falhas durante a assistência à saúde			
05/03/2022	CE	FORTALEZA	HOSPITAL GERAL DR. CESAR	PACIENTE EVOLUI COM	Concluída	Eventos Adversos Relacionados à	Lesão por pressão			
05/03/2022	CE	FORTALEZA	HOSPITAL GERAL DR. CESAR	PACIENTE ADMITIDA	Concluída	Eventos Adversos Relacionados à	Lesão por pressão			
05/03/2022	CE	FORTALEZA	HOSPITAL GERAL DR. CESAR	PACIENTE PROCENTE DA	Enviada	Eventos Adversos Relacionados à	Lesão por pressão			

05/03/2022	CE	FORTALEZA	HOSPITAL GERAL DR. CESAR	PACIENTE COM LPP EM MALEOL	Enviada	Eventos Adversos Relacionados à	Lesão por pressão		
05/03/2022	CE	FORTALEZA	HOSPITAL GERAL DR. CESAR	RN EVOLUI COM LPP EM	Enviada	Eventos Adversos Relacionados à	Lesão por pressão		
05/03/2022	CE	FORTALEZA	HOSPITAL GERAL DR. CESAR	PACIENTE EVOLUI COM	Concluída	Eventos Adversos Relacionados à	Lesão por pressão		
05/03/2022	CE	FORTALEZA	HOSPITAL GERAL DR. CESAR	ADMITIDA COM LESOES ULCERO	Enviada	Eventos Adversos Relacionados à	Lesão por pressão		
05/03/2022	CE	FORTALEZA	HOSPITAL GERAL DR. CESAR	PACIENTE PROCEDENTE DE	Enviada	Eventos Adversos Relacionados à	Lesão por pressão		
05/03/2022	CE	FORTALEZA	HOSPITAL GERAL DR. CESAR	PACIENTE EVOLUI COM	Enviada	Eventos Adversos Relacionados à	Lesão por pressão		
05/03/2022	CE	FORTALEZA	HOSPITAL GERAL DR. CESAR	IDENTIFICAÇÃO DE ALERGIA	Enviada	Eventos Adversos Relacionados à	Falha na documentação		
07/03/2022	CE	FORTALEZA	HOSPITAL GERAL DR. CESAR	Pte admitida para realizar	Enviada	Eventos Adversos Relacionados à	Falhas durante procedimento		
07/03/2022	CE	FORTALEZA	HOSPITAL GERAL DR. CESAR	Pte 78 anos 1ºPO Histerosc	Enviada	Eventos Adversos Relacionados à	Broncoaspiração		
07/03/2022	CE	FORTALEZA	HOSPITAL GERAL DR. CESAR	-	Enviada	Eventos Adversos Relacionados à	Broncoaspiração		
07/03/2022	CE	FORTALEZA	HOSPITAL GERAL DR. CESAR	paciente ao levantar só , caiu	Enviada	Eventos Adversos Relacionados à	Queda do paciente		
07/03/2022	CE	FORTALEZA	HOSPITAL GERAL DR. CESAR	-	Enviada	Eventos Adversos Relacionados à	Queimadura de paciente		
07/03/2022	CE	FORTALEZA	HOSPITAL GERAL DR. CESAR	-	Enviada	Eventos Adversos Relacionados à	Queimadura de paciente		
07/03/2022	CE	FORTALEZA	HOSPITAL GERAL DR. CESAR	Paciente 31anos, pós datismo	Em investigação	Eventos Adversos Relacionados à	Falhas durante procedimento		
21/03/2022	CE	FORTALEZA	HOSPITAL GERAL DR. CESAR	Pte chegou 6:30 ao hospital	Enviada	Eventos Adversos Relacionados à	Falhas nas atividades		

21/03/2022	CE	FORTALEZA	HOSPITAL GERAL DR. CESAR	RN com SOG com 72h sem	Enviada	Eventos Adversos Relacionados à	Falhas envolvendo sondas		
21/03/2022	CE	FORTALEZA	HOSPITAL GERAL DR. CESAR	RN com inserção de PICC dia 14/02	Enviada	Eventos Adversos Relacionados à	Falhas envolvendo cateter venoso		
21/03/2022	CE	FORTALEZA	HOSPITAL GERAL DR. CESAR	RN com PICC obstruido por	Enviada	Eventos Adversos Relacionados à	Falhas envolvendo cateter venoso		
02/04/2022	CE	FORTALEZA	HOSPITAL GERAL DR. CESAR	paciente com lpp em trocanter	Enviada	Eventos Adversos Relacionados à	Lesão por pressão		
02/04/2022	CE	FORTALEZA	HOSPITAL GERAL DR. CESAR	LPP EM REGIÃO SACRA, ESTAGIO	Concluída	Eventos Adversos Relacionados à	Lesão por pressão		
02/04/2022	CE	FORTALEZA	HOSPITAL GERAL DR. CESAR	PACIENTE COM LPP EM TROCAN	Enviada	Eventos Adversos Relacionados à	Lesão por pressão		
02/04/2022	CE	FORTALEZA	HOSPITAL GERAL DR. CESAR	rn com lesão em região occiptal	Enviada	Eventos Adversos Relacionados à	Lesão por pressão		
02/04/2022	CE	FORTALEZA	HOSPITAL GERAL DR. CESAR	paciente lpp em região sacra,	Enviada	Eventos Adversos Relacionados à	Lesão por pressão		
02/04/2022	CE	FORTALEZA	HOSPITAL GERAL DR. CESAR	paciente restrita ao leito, lpp em	Enviada	Eventos Adversos Relacionados à	Falhas durante a assistência à saúde		
02/04/2022	CE	FORTALEZA	HOSPITAL GERAL DR. CESAR	paciente em uso de drogas	Enviada	Eventos Adversos Relacionados à	Falhas envolvendo cateter venoso		
02/04/2022	CE	FORTALEZA	HOSPITAL GERAL DR. CESAR	LP em maleolo externo e porção	Enviada	Eventos Adversos Relacionados à	Lesão por pressão		
02/04/2022	CE	FORTALEZA	HOSPITAL GERAL DR. CESAR	LPA em regiões sacra, estagio 2	Enviada	Eventos Adversos Relacionados à	Lesão por pressão		
02/04/2022	CE	FORTALEZA	HOSPITAL GERAL DR. CESAR	paciente com HD de penfego	Enviada	Eventos Adversos Relacionados à	Lesão por pressão		
02/04/2022	CE	FORTALEZA	HOSPITAL GERAL DR. CESAR	LPP NA REGIÃO SACRA, ESTAGIO	Enviada	Eventos Adversos Relacionados à	Lesão por pressão		
02/04/2022	CE	FORTALEZA	HOSPITAL GERAL DR. CESAR	LP na admissão em região	Enviada	Eventos Adversos Relacionados à	Lesão por pressão		

30/04/2022	CE	FORTALEZA	HOSPITAL GERAL DR. CESAR	leite do RN COM O NOME ERRADO	Enviada	Eventos Adversos Relacionados à	Falha na identificação do paciente		
30/04/2022	CE	FORTALEZA	HOSPITAL GERAL DR. CESAR	PAC. C/ CUIDADOS PALIATIV	Enviada	Eventos Adversos Relacionados à	Falhas nas atividades		
30/04/2022	CE	FORTALEZA	HOSPITAL GERAL DR. CESAR	Pte 25anos, gestante , sentada a	Enviada	Eventos Adversos Relacionados à	Queda do paciente		
30/04/2022	CE	FORTALEZA	HOSPITAL GERAL DR. CESAR	Pte caiu da cama ao colocar	Enviada	Eventos Adversos Relacionados à	Queda do paciente		
30/04/2022	CE	FORTALEZA	HOSPITAL GERAL DR. CESAR	Pte encaminhada para realizarTC	Enviada	Eventos Adversos Relacionados à	Falhas envolvendo o cateter venoso		
14/05/2022	CE	FORTALEZA	HOSPITAL GERAL DR. CESAR	PACIENTE COM LPP,ESTAGIO III	Enviada	Eventos Adversos Relacionados à	Lesão por pressão		
14/05/2022	CE	FORTALEZA	HOSPITAL GERAL DR. CESAR	Pte admitido de outro hospital	Enviada	Eventos Adversos Relacionados à	Lesão por pressão		
14/05/2022	CE	FORTALEZA	HOSPITAL GERAL DR. CESAR	Pte Marineide de Oliveira	Enviada	Eventos Adversos Relacionados à	Falha na identificação do paciente		
14/05/2022	CE	FORTALEZA	HOSPITAL GERAL DR. CESAR	Pte 41 anos admitido com	Enviada	Eventos Adversos Relacionados à	Falhas durante a assistência à saúde		
14/05/2022	CE	FORTALEZA	HOSPITAL GERAL DR. CESAR	Pte 30 anos, com historia	Enviada	Eventos Adversos Relacionados à	Falhas durante procedimento		
14/05/2022	CE	FORTALEZA	HOSPITAL GERAL DR. CESAR	Paciernte apresentou lpp em região	Enviada	Eventos Adversos Relacionados à	Lesão por pressão		
14/05/2022	CE	FORTALEZA	HOSPITAL GERAL DR. CESAR	RN APRESENTANDO LPP EM	Enviada	Eventos Adversos Relacionados à	Lesão por pressão		
14/05/2022	CE	FORTALEZA	HOSPITAL GERAL DR. CESAR	PACIENTE TENTOU LEVANTA	Enviada	Eventos Adversos Relacionados à	Queda do paciente		
14/05/2022	CE	FORTALEZA	HOSPITAL GERAL DR. CESAR	Pte 27anos c lesão por pressão	Enviada	Eventos Adversos Relacionados à	Lesão por pressão		
14/05/2022	CE	FORTALEZA	HOSPITAL GERAL DR. CESAR	RN COM LPP NO MMII ESQ	Enviada	Eventos Adversos Relacionados à	Lesão por pressão		

14/05/2022	CE	FORTALEZA	HOSPITAL GERAL DR. CESAR	PTE 78 ANOS APRESENTA	Enviada	Eventos Adversos Relacionados à	Lesão por pressão		
14/05/2022	CE	FORTALEZA	HOSPITAL GERAL DR. CESAR	Pte 48anos com LPP adquirida	Enviada	Eventos Adversos Relacionados à	Lesão por pressão		
14/05/2022	CE	FORTALEZA	HOSPITAL GERAL DR. CESAR	Paciente gestante em tto de anemia	Enviada	Eventos Adversos Relacionados à	Evasão do paciente		
14/05/2022	CE	FORTALEZA	HOSPITAL GERAL DR. CESAR	Pte 50 anos LPP na internação	Enviada	Eventos Adversos Relacionados à	Lesão por pressão		
14/05/2022	CE	FORTALEZA	HOSPITAL GERAL DR. CESAR	PTE 60 ANOS, APRESENTOU	Enviada	Eventos Adversos Relacionados à	Falhas envolvendo o cateter venoso		
14/05/2022	CE	FORTALEZA	HOSPITAL GERAL DR. CESAR	Paciente idosa, 78 anos por LPP em	Enviada	Eventos Adversos Relacionados à	Lesão por pressão		
14/05/2022	CE	FORTALEZA	HOSPITAL GERAL DR. CESAR	RN adquiriu LP estagio	Enviada	Eventos Adversos Relacionados à	Lesão por pressão		
14/05/2022	CE	FORTALEZA	HOSPITAL GERAL DR. CESAR	PACIENTE APRESENTA LP	Enviada	Eventos Adversos Relacionados à	Lesão por pressão		
16/05/2022	CE	FORTALEZA	HOSPITAL GERAL DR. CESAR	Pte 69 anos, admitido n	Em investigação	Eventos Adversos Relacionados à	Falhas durante a assistência à saúde		
24/05/2022	CE	FORTALEZA	HOSPITAL GERAL DR. CESAR	Pte 41anos admitido n	Enviada	Eventos Adversos Relacionados à	Falhas durante a assistência à saúde		
24/05/2022	CE	FORTALEZA	HOSPITAL GERAL DR. CESAR	RN sob sedação porem ativo ao	Enviada	Eventos Adversos Relacionados à	Extubação endotraqueal		
24/05/2022	CE	FORTALEZA	HOSPITAL GERAL DR. CESAR	RN gemelar com prescrição	Enviada	Eventos Adversos Relacionados à	Falhas durante a assistência à saúde		
24/05/2022	CE	FORTALEZA	HOSPITAL GERAL DR. CESAR	RN com PICC no inicio do plantão	Enviada	Eventos Adversos Relacionados à	Falhas envolvendo o cateter venoso		
24/05/2022	CE	FORTALEZA	HOSPITAL GERAL DR. CESAR	RN com PICC e na passagem	Enviada	Eventos Adversos Relacionados à	Falhas envolvendo o cateter venoso		
18/06/2022	CE	FORTALEZA	HOSPITAL GERAL DR. CESAR	Pte com LP em região sacra	Enviada	Eventos Adversos Relacionados à	Lesão por pressão		

18/06/2022	CE	FORTALEZA	HOSPITAL GERAL DR. CESAR	Pte apresentando lesão em fenda	Enviada	Eventos Adversos Relacionados à	Lesão por pressão		
18/06/2022	CE	FORTALEZA	HOSPITAL GERAL DR. CESAR	Paciente sentada no leito em	Enviada	Eventos Adversos Relacionados à	Queda do paciente		
18/06/2022	CE	FORTALEZA	HOSPITAL GERAL DR. CESAR	Pte sob sedoanalgesia comTOT	Enviada	Eventos Adversos Relacionados à	Extubação endotraqueal		
18/06/2022	CE	FORTALEZA	HOSPITAL GERAL DR. CESAR	Em região do trocânter direito	Enviada	Eventos Adversos Relacionados à	Lesão por pressão		
18/06/2022	CE	FORTALEZA	HOSPITAL GERAL DR. CESAR	paciente foi ao banheiro e	Enviada	Eventos Adversos Relacionados à	Queda do paciente		
18/06/2022	CE	FORTALEZA	HOSPITAL GERAL DR. CESAR	durante a troca de plantão foi	Enviada	Eventos Adversos Relacionados à	Falhas envolvendo o cateter venoso		
18/06/2022	CE	FORTALEZA	HOSPITAL GERAL DR. CESAR	RN em uso do picc e durante	Enviada	Eventos Adversos Relacionados à	Falhas envolvendo o cateter venoso		
18/06/2022	CE	FORTALEZA	HOSPITAL GERAL DR. CESAR	Pte com sintomas gripais, laboratori	Enviada	Eventos Adversos Relacionados à	Falhas ocorridas em laboratóri		
18/06/2022	CE	FORTALEZA	HOSPITAL GERAL DR. CESAR	recebo plantão com RN com	Enviada	Eventos Adversos Relacionados à	Falhas envolvendo o cateter venoso		
18/06/2022	CE	FORTALEZA	HOSPITAL GERAL DR. CESAR	RN 27 S, PARTO ABDOMINAL	Enviada	Eventos Adversos Relacionados à	Falhas durante procedimento		
05/07/2022	CE	FORTALEZA	HOSPITAL GERAL DR. CESAR	Paciente apresentando LP na	Enviada	Eventos Adversos Relacionados à	Lesão por pressão		
05/07/2022	CE	FORTALEZA	HOSPITAL GERAL DR. CESAR	Paciente idosa na internação	Enviada	Eventos Adversos Relacionados à	Lesão por pressão		
05/07/2022	CE	FORTALEZA	HOSPITAL GERAL DR. CESAR	Paciente na internação	Enviada	Eventos Adversos Relacionados à	Lesão por pressão		
05/07/2022	CE	FORTALEZA	HOSPITAL GERAL DR. CESAR	Pte na internação com presença	Enviada	Eventos Adversos Relacionados à	Lesão por pressão		
05/07/2022	CE	FORTALEZA	HOSPITAL GERAL DR. CESAR	Paciente apresenta múltiplas lesões	Enviada	Eventos Adversos Relacionados à	Lesão por pressão		

05/07/2022	CE	FORTALE ZA	HOSPITA L GERAL DR. CESAR	APRESE NTA LP EM REGIÃO	Enviada	Eventos Adversos Relaciona dos à	Lesão por pressão		
06/07/2022	CE	FORTALE ZA	HOSPITA L GERAL DR. CESAR	PACIENT E CAIU POR	Enviada	Eventos Adversos Relaciona dos à	Queda do paciente		
06/07/2022	CE	FORTALE ZA	HOSPITA L GERAL DR. CESAR	Pte 41 anos, não realizou o exame	Enviada	Eventos Adversos Relaciona dos à	Falhas durante a assistênci a à saúde		
06/07/2022	CE	FORTALE ZA	HOSPITA L GERAL DR. CESAR	RNT, 7º DDV, APRESE NTANDO	Enviada	Eventos Adversos Relaciona dos à	Falhas envolvend o cateter venoso		
06/07/2022	CE	FORTALE ZA	HOSPITA L GERAL DR. CESAR	PICC DE MSD PERDIDO POR	Enviada	Eventos Adversos Relaciona dos à	Falhas envolvend o cateter venoso		
06/07/2022	CE	FORTALE ZA	HOSPITA L GERAL DR. CESAR	PICC RESISTE NTE NO MSD	Enviada	Eventos Adversos Relaciona dos à	Falhas envolvend o cateter venoso		
06/07/2022	CE	FORTALE ZA	HOSPITA L GERAL DR. CESAR	PTE 62 ANOS COM EXAME	Enviada	Eventos Adversos Relaciona dos à	Falhas durante a assistênci a à saúde		
15/07/2022	CE	FORTALE ZA	HOSPITA L GERAL DR. CESAR	PACIENT E SOFRE QUEDA	Enviada	Eventos Adversos Relaciona dos à	Queda do paciente		
13/08/2022	CE	FORTALE ZA	HOSPITA L GERAL DR. CESAR	Paciente apresenta ndo LP sacra não	Enviada	Eventos Adversos Relaciona dos à	Lesão por pressão		
13/08/2022	CE	FORTALE ZA	HOSPITA L GERAL DR. CESAR	RN apresenta ndo LP tissular	Enviada	Eventos Adversos Relaciona dos à	Lesão por pressão		
13/08/2022	CE	FORTALE ZA	HOSPITA L GERAL DR. CESAR	Paciente ao retornar do	Enviada	Eventos Adversos Relaciona dos à	Queda do paciente		
13/08/2022	CE	FORTALE ZA	HOSPITA L GERAL DR. CESAR	Apresenta LP em estagio 2 em	Enviada	Eventos Adversos Relaciona dos à	Lesão por pressão		
13/08/2022	CE	FORTALE ZA	HOSPITA L GERAL DR. CESAR	PACIENT E ADMITID O	Concluída	Eventos Adversos Relaciona dos à	Lesão por pressão		
13/08/2022	CE	FORTALE ZA	HOSPITA L GERAL DR. CESAR	APRESE NTA FO EM	Enviada	Eventos Adversos Relaciona dos à	Lesão por pressão		
13/08/2022	CE	FORTALE ZA	HOSPITA L GERAL DR. CESAR	RN apresenta ndo lesão por	Concluída	Eventos Adversos Relaciona dos à	Lesão por pressão		

13/08/2022	CE	FORTALEZA	HOSPITAL GERAL DR. CESAR	PTE ADMITIDA APRESE	Enviada	Eventos Adversos Relacionados à	Lesão por pressão		
13/08/2022	CE	FORTALEZA	HOSPITAL GERAL DR. CESAR	RN SAIU DE ALTA SEM REALIZA	Enviada	Eventos Adversos Relacionados à	Falhas durante a assistência à saúde		
13/08/2022	CE	FORTALEZA	HOSPITAL GERAL DR. CESAR	RN SAIU DE ALTA HOSPITALAR	Enviada	Eventos Adversos Relacionados à	Falhas durante a assistência à saúde		
13/08/2022	CE	FORTALEZA	HOSPITAL GERAL DR. CESAR	PERDA DE CATETER PICC	Enviada	Eventos Adversos Relacionados à	Falhas envolvendo o cateter venoso		
13/08/2022	CE	FORTALEZA	HOSPITAL GERAL DR. CESAR	RN SAIU DE ALTA HOSPITALAR	Enviada	Eventos Adversos Relacionados à	Falhas ocorridas em laboratóri		
13/08/2022	CE	FORTALEZA	HOSPITAL GERAL DR. CESAR	Pte admitida na UTI pós	Enviada	Eventos Adversos Relacionados à	Falhas durante a assistência à saúde		
13/08/2022	CE	FORTALEZA	HOSPITAL GERAL DR. CESAR	FALTA DE INSTALAÇÃO DE	Enviada	Eventos Adversos Relacionados à	Falhas durante a assistência à saúde		
13/08/2022	CE	FORTALEZA	HOSPITAL GERAL DR. CESAR	Pte pos cesarea não foi feito dose	Enviada	Eventos Adversos Relacionados à	Falhas durante a assistência à saúde		
20/08/2022	CE	FORTALEZA	HOSPITAL GERAL DR. CESAR	Pte 96anos admitido com	Enviada	Eventos Adversos Relacionados à	Lesão por pressão		
20/08/2022	CE	FORTALEZA	HOSPITAL GERAL DR. CESAR	RN com lesão por pressão em	Enviada	Eventos Adversos Relacionados à	Lesão por pressão		
20/08/2022	CE	FORTALEZA	HOSPITAL GERAL DR. CESAR	PTE 21 ANOS POI DE PARTO	Enviada	Eventos Adversos Relacionados à	Falhas durante a assistência à saúde		
20/08/2022	CE	FORTALEZA	HOSPITAL GERAL DR. CESAR	PIIC apresenta obstrução	Enviada	Eventos Adversos Relacionados à	Falhas envolvendo o cateter venoso		
20/08/2022	CE	FORTALEZA	HOSPITAL GERAL DR. CESAR	Colhido material biologico do dreno	Enviada	Eventos Adversos Relacionados à	Falhas durante a assistência à saúde		
22/08/2022	CE	FORTALEZA	HOSPITAL GERAL DR. CESAR	RN EM BILIBERÇO APRESE	Enviada	Eventos Adversos Relacionados à	Queimadura de paciente		
22/08/2022	CE	FORTALEZA	HOSPITAL GERAL DR. CESAR	POI DE HERNIORRAFIA INGUINA	Enviada	Eventos Adversos Relacionados à	Extubaçao endotraqueal		

22/08/2022	CE	FORTALEZA	HOSPITAL GERAL DR. CESAR	MATERIAL BIOLÓGICO DO	Enviada	Eventos Adversos Relacionados à	Falhas durante a assistência à saúde		
10/09/2022	CE	FORTALEZA	HOSPITAL GERAL DR. CESAR	Pte 77anos ao tentar levantar	Enviada	Eventos Adversos Relacionados à	Queda do paciente		
10/09/2022	CE	FORTALEZA	HOSPITAL GERAL DR. CESAR	PTE LMA EM TRATAMENTO	Enviada	Eventos Adversos Relacionados à	Falhas envolvendo o cateter venoso		
10/09/2022	CE	FORTALEZA	HOSPITAL GERAL DR. CESAR	Pte apresentando lesão por	Enviada	Eventos Adversos Relacionados à	Lesão por pressão		
10/09/2022	CE	FORTALEZA	HOSPITAL GERAL DR. CESAR	Encaminhado da unidade a pte para	Enviada	Eventos Adversos Relacionados à	Falhas durante a assistência à saúde		
10/09/2022	CE	FORTALEZA	HOSPITAL GERAL DR. CESAR	RN com TOT ao realizar troca de	Enviada	Eventos Adversos Relacionados à	Lesão por pressão		
10/09/2022	CE	FORTALEZA	HOSPITAL GERAL DR. CESAR	RN com PICC em HV e ATB em	Enviada	Eventos Adversos Relacionados à	Falhas envolvendo o cateter venoso		
15/09/2022	CE	FORTALEZA	HOSPITAL GERAL DR. CESAR	Paciente ao retornar do	Enviada	Eventos Adversos Relacionados à	Queda do paciente		
01/10/2022	CE	FORTALEZA	HOSPITAL GERAL DR. CESAR	Paciente dependente de apoio des	Enviada	Eventos Adversos Relacionados à	Queda do paciente		
01/10/2022	CE	FORTALEZA	HOSPITAL GERAL DR. CESAR	Paciente 69 anos, relato que	Enviada	Eventos Adversos Relacionados à	Queda do paciente		
01/10/2022	CE	FORTALEZA	HOSPITAL GERAL DR. CESAR	PACIENTE RELATA QUE	Enviada	Eventos Adversos Relacionados à	Queda do paciente		
03/10/2022	CE	FORTALEZA	HOSPITAL GERAL DR. CESAR	Pte admitido com um nome	Enviada	Eventos Adversos Relacionados à	Falha na identificação do paciente		
08/10/2022	CE	FORTALEZA	HOSPITAL GERAL DR. CESAR	RN com LP em septo nasal por	Enviada	Eventos Adversos Relacionados à	Lesão por pressão		
08/10/2022	CE	FORTALEZA	HOSPITAL GERAL DR. CESAR	Pte admitido apresentando lesão	Concluída	Eventos Adversos Relacionados à	Lesão por pressão		
08/10/2022	CE	FORTALEZA	HOSPITAL GERAL DR. CESAR	PTE APRESENTA F.O EM	Enviada	Eventos Adversos Relacionados à	Lesão por pressão		

08/10/2022	CE	FORTALEZA	HOSPITAL GERAL DR. CESAR	RN Apresenta LP relacionad	Enviada	Eventos Adversos Relacionados à	Lesão por pressão		
08/10/2022	CE	FORTALEZA	HOSPITAL GERAL DR. CESAR	Pte na admissão apresenta	Enviada	Eventos Adversos Relacionados à	Lesão por pressão		
08/10/2022	CE	FORTALEZA	HOSPITAL GERAL DR. CESAR	Pte admitida e avaliada	Concluída	Eventos Adversos Relacionados à	Lesão por pressão		
08/10/2022	CE	FORTALEZA	HOSPITAL GERAL DR. CESAR	Pte apresentou na internação	Enviada	Eventos Adversos Relacionados à	Lesão por pressão		
08/10/2022	CE	FORTALEZA	HOSPITAL GERAL DR. CESAR	Pte apresentou LPP em	Enviada	Eventos Adversos Relacionados à	Lesão por pressão		
08/10/2022	CE	FORTALEZA	HOSPITAL GERAL DR. CESAR	Pte apresentou na internação	Enviada	Eventos Adversos Relacionados à	Lesão por pressão		
08/10/2022	CE	FORTALEZA	HOSPITAL GERAL DR. CESAR	Pte apresentando lesão por	Enviada	Eventos Adversos Relacionados à	Lesão por pressão		
08/10/2022	CE	FORTALEZA	HOSPITAL GERAL DR. CESAR	NPT acabou antes do tempo	Enviada	Eventos Adversos Relacionados à	Falhas na administração de dietas		Parenteral (via intravenosa)
08/10/2022	CE	FORTALEZA	HOSPITAL GERAL DR. CESAR	PTE ADMITIDO VINDO DA	Enviada	Eventos Adversos Relacionados à	Falhas durante a assistência à saúde		
08/10/2022	CE	FORTALEZA	HOSPITAL GERAL DR. CESAR	PTE APRESENTOU GLICEMI	Enviada	Eventos Adversos Relacionados à	Falhas durante a assistência à saúde		
08/10/2022	CE	FORTALEZA	HOSPITAL GERAL DR. CESAR	RN COM 9 DIAS DE NASCIDO	Enviada	Eventos Adversos Relacionados à	Falhas durante a assistência à saúde		
08/10/2022	CE	FORTALEZA	HOSPITAL GERAL DR. CESAR	MEDICO PLANTONISTA FEZ	Enviada	Eventos Adversos Relacionados à	Falhas durante a assistência à saúde		
08/10/2022	CE	FORTALEZA	HOSPITAL GERAL DR. CESAR	RN EM DIETA DE TRANSIC	Enviada	Eventos Adversos Relacionados à	Falhas na administração de dietas		Enteral (oral, cateter oral ou
10/10/2022	CE	FORTALEZA	HOSPITAL GERAL DR. CESAR	PTE 41ANOS EM VM, TRAQUE	Enviada	Eventos Adversos Relacionados à	Falhas durante a assistência à saúde		
22/10/2022	CE	FORTALEZA	HOSPITAL GERAL DR. CESAR	Paciente referiu dor no gluteo	Enviada	Eventos Adversos Relacionados à	Queda do paciente		

22/10/2022	CE	FORTALEZA	HOSPITAL GERAL DR. CESAR	RN com prescrição médica de LMO	Enviada	Eventos Adversos Relacionados à	Falhas na administração de dietas		Enteral (oral, cateter oral ou
22/10/2022	CE	FORTALEZA	HOSPITAL GERAL DR. CESAR	PTE GESTANTE G1P0, 26	Enviada	Eventos Adversos Relacionados à	Falhas durante a assistência à saúde		
05/11/2022	CE	FORTALEZA	HOSPITAL GERAL DR. CESAR	Pte 62 anos com LP região sacra não	Concluída	Eventos Adversos Relacionados à	Lesão por pressão		
05/11/2022	CE	FORTALEZA	HOSPITAL GERAL DR. CESAR	Pte 37 nos presenta LP região	Concluída	Eventos Adversos Relacionados à	Lesão por pressão		
05/11/2022	CE	FORTALEZA	HOSPITAL GERAL DR. CESAR	Pte presenta LP em trocanter	Enviada	Eventos Adversos Relacionados à	Lesão por pressão		
05/11/2022	CE	FORTALEZA	HOSPITAL GERAL DR. CESAR	Pte apresenta LP região	Enviada	Eventos Adversos Relacionados à	Lesão por pressão		
05/11/2022	CE	FORTALEZA	HOSPITAL GERAL DR. CESAR	RN apresentando LP não	Concluída	Eventos Adversos Relacionados à	Lesão por pressão		
05/11/2022	CE	FORTALEZA	HOSPITAL GERAL DR. CESAR	RN apresenta Lesão em pé	Enviada	Eventos Adversos Relacionados à	Lesão por pressão		
05/11/2022	CE	FORTALEZA	HOSPITAL GERAL DR. CESAR	Pte apresenta lesão (flictema)	Enviada	Eventos Adversos Relacionados à	Lesão por pressão		
05/11/2022	CE	FORTALEZA	HOSPITAL GERAL DR. CESAR	RN presenta LP em região	Enviada	Eventos Adversos Relacionados à	Lesão por pressão		
05/11/2022	CE	FORTALEZA	HOSPITAL GERAL DR. CESAR	Paciente apresenta lesão por pressão	Enviada	Eventos Adversos Relacionados à	Lesão por pressão		
05/11/2022	CE	FORTALEZA	HOSPITAL GERAL DR. CESAR	Pte apresenta LP estagio 2	Enviada	Eventos Adversos Relacionados à	Lesão por pressão		
05/11/2022	CE	FORTALEZA	HOSPITAL GERAL DR. CESAR	PTE APRESENTA LP EM	Enviada	Eventos Adversos Relacionados à	Lesão por pressão		
05/11/2022	CE	FORTALEZA	HOSPITAL GERAL DR. CESAR	RN apresenta LP em região	Enviada	Eventos Adversos Relacionados à	Lesão por pressão		
05/11/2022	CE	FORTALEZA	HOSPITAL GERAL DR. CESAR	Pte com LP em região sacra não	Concluída	Eventos Adversos Relacionados à	Lesão por pressão		

05/11/2022	CE	FORTALEZA	HOSPITAL GERAL DR. CESAR	Pte apresenta LP em região	Enviada	Eventos Adversos Relacionados à	Lesão por pressão		
09/11/2022	CE	FORTALEZA	HOSPITAL GERAL DR. CESAR	PTE 66ANOS COM LESÃO	Enviada	Eventos Adversos Relacionados à	Lesão por pressão		
09/11/2022	CE	FORTALEZA	HOSPITAL GERAL DR. CESAR	Pte apresentando deiscênci	Enviada	Eventos Adversos Relacionados à	Lesão por pressão		
09/11/2022	CE	FORTALEZA	HOSPITAL GERAL DR. CESAR	Pte admitido apresenta LP em	Enviada	Eventos Adversos Relacionados à	Lesão por pressão		
19/11/2022	CE	FORTALEZA	HOSPITAL GERAL DR. CESAR	Paciente algo desorientado	Enviada	Eventos Adversos Relacionados à	Queda do paciente		
19/11/2022	CE	FORTALEZA	HOSPITAL GERAL DR. CESAR	Pte apresenta lesão por pressão	Enviada	Eventos Adversos Relacionados à	Lesão por pressão		
19/11/2022	CE	FORTALEZA	HOSPITAL GERAL DR. CESAR	Pte admitido LP região sacra	Concluída	Eventos Adversos Relacionados à	Lesão por pressão		
19/11/2022	CE	FORTALEZA	HOSPITAL GERAL DR. CESAR	RN com LP em região parietal	Enviada	Eventos Adversos Relacionados à	Lesão por pressão		
10/12/2022	CE	FORTALEZA	HOSPITAL GERAL DR. CESAR	AS 09:55H, PACIENTE	Enviada	Eventos Adversos Relacionados à	Queda do paciente		
10/12/2022	CE	FORTALEZA	HOSPITAL GERAL DR. CESAR	Cama do leito 205 caiu com a	Enviada	Eventos Adversos Relacionados à	Queda do paciente		
10/12/2022	CE	FORTALEZA	HOSPITAL GERAL DR. CESAR	Admissão c LPP na região sacral e	Concluída	Eventos Adversos Relacionados à	Lesão por pressão		
10/12/2022	CE	FORTALEZA	HOSPITAL GERAL DR. CESAR	Transferência de uma pte da	Enviada	Eventos Adversos Relacionados à	Falha na identificação do paciente		
10/12/2022	CE	FORTALEZA	HOSPITAL GERAL DR. CESAR	RN ATIVO COM EXTUBA	Enviada	Eventos Adversos Relacionados à	Extubação endotraqueal		
10/12/2022	CE	FORTALEZA	HOSPITAL GERAL DR. CESAR	RN ENCONTRADO EM PCR	Enviada	Eventos Adversos Relacionados à	Extubação endotraqueal		
10/12/2022	CE	FORTALEZA	HOSPITAL GERAL DR. CESAR	RN SOB VMI PROLOGADO	Enviada	Eventos Adversos Relacionados à	Extubação endotraqueal		

10/12/2022	CE	FORTALEZA	HOSPITAL GERAL DR. CESAR	Pte 36 anos com HD pancreatt	Em retificação	Eventos Adversos Relacionados à	Lesão por pressão		
10/12/2022	CE	FORTALEZA	HOSPITAL GERAL DR. CESAR	PTE DIAGNOSTICADO EM	Enviada	Eventos Adversos Relacionados à	Falhas envolvendo cateter venoso		
14/01/2023	CE	FORTALEZA	HOSPITAL GERAL DR. CESAR	Paciente entrou em TPP, dilatação	Enviada	Eventos Adversos Relacionados à	Queda do paciente		
14/01/2023	CE	FORTALEZA	HOSPITAL GERAL DR. CESAR	paciente idoso, sentou-se ao vaso	Enviada	Eventos Adversos Relacionados à	Queda do paciente		
28/01/2023	CE	FORTALEZA	HOSPITAL GERAL DR. CESAR	Paciente com cateter implantad	Enviada	Eventos Adversos Relacionados à	Falhas envolvendo cateter venoso		
28/01/2023	CE	FORTALEZA	HOSPITAL GERAL DR. CESAR	LPP EM REGIÃO INFERIOR, PÉ	Concluída	Eventos Adversos Relacionados à	Lesão por pressão		
28/01/2023	CE	FORTALEZA	HOSPITAL GERAL DR. CESAR	PACIENTE ADMITIDO NA UTI	Concluída	Eventos Adversos Relacionados à	Lesão por pressão		
28/01/2023	CE	FORTALEZA	HOSPITAL GERAL DR. CESAR	PACIENTE EVOLUI COM	Enviada	Eventos Adversos Relacionados à	Lesão por pressão		
28/01/2023	CE	FORTALEZA	HOSPITAL GERAL DR. CESAR	PACIENTE ADMITIDA COM	Enviada	Eventos Adversos Relacionados à	Lesão por pressão		
28/01/2023	CE	FORTALEZA	HOSPITAL GERAL DR. CESAR	PACIENTE EVOLUIU COM	Enviada	Eventos Adversos Relacionados à	Lesão por pressão		
28/01/2023	CE	FORTALEZA	HOSPITAL GERAL DR. CESAR	PACIENTE FOI ADMITIDA COM	Concluída	Eventos Adversos Relacionados à	Lesão por pressão		
18/02/2023	CE	FORTALEZA	HOSPITAL GERAL DR. CESAR	RN apresenta lesão por dispositiv	Enviada	Eventos Adversos Relacionados à	Lesão por pressão		
18/02/2023	CE	FORTALEZA	HOSPITAL GERAL DR. CESAR	: Paciente apresenta LP em	Enviada	Eventos Adversos Relacionados à	Lesão por pressão		
18/02/2023	CE	FORTALEZA	HOSPITAL GERAL DR. CESAR	xPTE 24 ANOS EVOLUIU	Enviada	Eventos Adversos Relacionados à	Falhas durante a assistência à saúde		
18/02/2023	CE	FORTALEZA	HOSPITAL GERAL DR. CESAR	APOS AVALIAÇÃO DE EXAME	Enviada	Eventos Adversos Relacionados à	Falha na identificação do paciente		

18/02/2023	CE	FORTALEZA	HOSPITAL GERAL DR. CESAR	Paciente apresentando LP em	Concluída	Eventos Adversos Relacionados à	Lesão por pressão		
18/02/2023	CE	FORTALEZA	HOSPITAL GERAL DR. CESAR	PACIENTE COM AVP HA 48H	Enviada	Eventos Adversos Relacionados à	Falhas envolvendo o cateter venoso		
18/02/2023	CE	FORTALEZA	HOSPITAL GERAL DR. CESAR	Paciente apresentando lesão com	Enviada	Eventos Adversos Relacionados à	Lesão por pressão		
18/02/2023	CE	FORTALEZA	HOSPITAL GERAL DR. CESAR	Paciente apresentando lp estagio 2	Enviada	Eventos Adversos Relacionados à	Lesão por pressão		
18/02/2023	CE	FORTALEZA	HOSPITAL GERAL DR. CESAR	RN NO 8° PO DE COLOCACÃO DE	Enviada	Eventos Adversos Relacionados à	Falhas envolvendo o cateter venoso		
18/02/2023	CE	FORTALEZA	HOSPITAL GERAL DR. CESAR	RELATADO DA TEC DE	Enviada	Eventos Adversos Relacionados à	Falhas envolvendo o cateter venoso		
23/02/2023	CE	FORTALEZA	HOSPITAL GERAL DR. CESAR	RN COM RESISTENCIA DE ACESSO	Enviada	Eventos Adversos Relacionados à	Falhas envolvendo o cateter venoso		
28/02/2023	CE	FORTALEZA	HOSPITAL GERAL DR. CESAR	PTE 34 ANOS PARTO ABDOMI	Enviada	Eventos Adversos Relacionados à	Falhas durante procedimento		
28/02/2023	CE	FORTALEZA	HOSPITAL GERAL DR. CESAR	PACIENTE COM ALTO RISCO	Enviada	Eventos Adversos Relacionados à	Queda do paciente		
04/03/2023	CE	FORTALEZA	HOSPITAL GERAL DR. CESAR	RN com LP em maléolo esquerdo	Concluída	Eventos Adversos Relacionados à	Lesão por pressão		
04/03/2023	CE	FORTALEZA	HOSPITAL GERAL DR. CESAR	RN apresentando múltiplas	Enviada	Eventos Adversos Relacionados à	Lesão por pressão		
04/03/2023	CE	FORTALEZA	HOSPITAL GERAL DR. CESAR	Pte lesão em retirada de osteo	Concluída	Eventos Adversos Relacionados à	Lesão por pressão		
04/03/2023	CE	FORTALEZA	HOSPITAL GERAL DR. CESAR	Pte 74 anos evoluiu com LP	Enviada	Eventos Adversos Relacionados à	Lesão por pressão		
11/03/2023	CE	FORTALEZA	HOSPITAL GERAL DR. CESAR	APRESENTA LESÃO POR	Enviada	Eventos Adversos Relacionados à	Lesão por pressão		
11/03/2023	CE	FORTALEZA	HOSPITAL GERAL DR. CESAR	NA ADMISSÃO APRESE	Enviada	Eventos Adversos Relacionados à	Lesão por pressão		

11/03/2023	CE	FORTALEZA	HOSPITAL GERAL DR. CESAR	LESÃO EM REGIÃO SACRA	Enviada	Eventos Adversos Relacionados à	Lesão por pressão		
11/03/2023	CE	FORTALEZA	HOSPITAL GERAL DR. CESAR	PRESENTA LESÃO POR	Concluída	Eventos Adversos Relacionados à	Lesão por pressão		
18/03/2023	CE	FORTALEZA	HOSPITAL GERAL DR. CESAR	RN com Sindrome de Edwards	Enviada	Eventos Adversos Relacionados à	Extubaçao endotraqueal		
18/03/2023	CE	FORTALEZA	HOSPITAL GERAL DR. CESAR	RN com Sindrome de Edwards	Enviada	Eventos Adversos Relacionados à	Falhas envolvendo o cateter venoso		
18/03/2023	CE	FORTALEZA	HOSPITAL GERAL DR. CESAR	Pte 69 anos no momento da	Enviada	Eventos Adversos Relacionados à	Falhas durante a assistência à saúde		
23/03/2023	CE	FORTALEZA	HOSPITAL GERAL DR. CESAR	Pte 53 anos HD artrite septica e	Enviada	Eventos Adversos Relacionados à	Falhas ocorridas em laboratóri		
23/03/2023	CE	FORTALEZA	HOSPITAL GERAL DR. CESAR	PTE 40 ANOS POI COLEDO	Enviada	Eventos Adversos Relacionados à	Falhas durante a assistência à saúde		
27/03/2023	CE	FORTALEZA	HOSPITAL GERAL DR. CESAR	Paciente apresenta LP em região	Enviada	Eventos Adversos Relacionados à	Lesão por pressão		
27/03/2023	CE	FORTALEZA	HOSPITAL GERAL DR. CESAR	Pte 30 anos apresentou na	Enviada	Eventos Adversos Relacionados à	Lesão por pressão		
27/03/2023	CE	FORTALEZA	HOSPITAL GERAL DR. CESAR	REGISTRO DE LPP EM SACRA	Concluída	Eventos Adversos Relacionados à	Lesão por pressão		
27/03/2023	CE	FORTALEZA	HOSPITAL GERAL DR. CESAR	Pte com LP sacral estagio não	Enviada	Eventos Adversos Relacionados à	Lesão por pressão		
27/03/2023	CE	FORTALEZA	HOSPITAL GERAL DR. CESAR	LP EM REGIAO GLÚTEA ESTAGIO	Enviada	Eventos Adversos Relacionados à	Lesão por pressão		
27/03/2023	CE	FORTALEZA	HOSPITAL GERAL DR. CESAR	PTE NA INTERNAÇÃO EVOLUIU	Enviada	Eventos Adversos Relacionados à	Lesão por pressão		
27/03/2023	CE	FORTALEZA	HOSPITAL GERAL DR. CESAR	APRESENTA LP EM REGIÃO	Enviada	Eventos Adversos Relacionados à	Lesão por pressão		
27/03/2023	CE	FORTALEZA	HOSPITAL GERAL DR. CESAR	EM TEMPO: RN NO 7º DDV	Enviada	Eventos Adversos Relacionados à	Falhas na administração de dietas		Parenteral (via intravenosa)

27/03/2023	CE	FORTALEZA	HOSPITAL GERAL DR. CESAR	EM TEMPO: RN 18° DDV	Enviada	Eventos Adversos Relacionados à	Falhas na administração de dietas		Parenteral (via intravenosa)
12/02/2019	CE	FORTALEZA	HGF	-	Enviada	Eventos Adversos Relacionados à	Queda do paciente		
12/02/2019	CE	FORTALEZA	HGF	-	Enviada	Eventos Adversos Relacionados à	Queda do paciente		
12/02/2019	CE	FORTALEZA	HGF	-	Enviada	Eventos Adversos Relacionados à	Queda do paciente		
12/02/2019	CE	FORTALEZA	HGF	-	Enviada	Eventos Adversos Relacionados à	Falhas durante a assistência à saúde		
12/02/2019	CE	FORTALEZA	HGF	-	Enviada	Eventos Adversos Relacionados à	Queimadura de paciente		
12/02/2019	CE	FORTALEZA	HGF	-	Enviada	Eventos Adversos Relacionados à	Falhas durante a assistência à saúde		
12/02/2019	CE	FORTALEZA	HGF	-	Enviada	Eventos Adversos Relacionados à	Acidentes do paciente	Outro tipo de mecanismo de	
18/02/2019	CE	FORTALEZA	HGF	-	Enviada	Eventos Adversos Relacionados à	Queda do paciente		
19/02/2019	CE	FORTALEZA	HGF	-	Enviada	Eventos Adversos Relacionados à	Queda do paciente		
19/02/2019	CE	FORTALEZA	HGF	-	Enviada	Eventos Adversos Relacionados à	Queda do paciente		
19/02/2019	CE	FORTALEZA	HGF	-	Enviada	Eventos Adversos Relacionados à	Queda do paciente		
19/02/2019	CE	FORTALEZA	HGF	-	Enviada	Eventos Adversos Relacionados à	Queda do paciente		
20/02/2019	CE	FORTALEZA	HGF	-	Enviada	Eventos Adversos Relacionados à	Queda do paciente		
20/02/2019	CE	FORTALEZA	HGF	-	Enviada	Eventos Adversos Relacionados à	Queda do paciente		

22/02/2019	CE	FORTALEZA	HGF	-	Enviada	Eventos Adversos Relacionados à	Falhas durante a assistência à saúde		
13/03/2019	CE	FORTALEZA	HGF	-	Enviada	Eventos Adversos Relacionados à	Queda do paciente		
13/03/2019	CE	FORTALEZA	HGF	-	Enviada	Eventos Adversos Relacionados à	Queda do paciente		
13/03/2019	CE	FORTALEZA	HGF	-	Enviada	Eventos Adversos Relacionados à	Queda do paciente		
13/03/2019	CE	FORTALEZA	HGF	-	Enviada	Eventos Adversos Relacionados à	Falhas durante a assistência à saúde		
26/03/2019	CE	FORTALEZA	HGF	-	Enviada	Eventos Adversos Relacionados à	Falhas durante a assistência à saúde		
26/03/2019	CE	FORTALEZA	HGF	-	Enviada	Eventos Adversos Relacionados à	Falhas durante a assistência à saúde		
28/05/2019	CE	FORTALEZA	HGF	-	Enviada	Eventos Adversos Relacionados à	Queda do paciente		
28/05/2019	CE	FORTALEZA	HGF	-	Enviada	Eventos Adversos Relacionados à	Queda do paciente		
28/05/2019	CE	FORTALEZA	HGF	-	Enviada	Eventos Adversos Relacionados à	Queda do paciente		
28/05/2019	CE	FORTALEZA	HGF	-	Enviada	Eventos Adversos Relacionados à	Queda do paciente		
28/05/2019	CE	FORTALEZA	HGF	-	Enviada	Eventos Adversos Relacionados à	Falhas durante procedimento		
28/05/2019	CE	FORTALEZA	HGF	-	Enviada	Eventos Adversos Relacionados à	Queda do paciente		
28/05/2019	CE	FORTALEZA	HGF	-	Enviada	Eventos Adversos Relacionados à	Queda do paciente		
28/05/2019	CE	FORTALEZA	HGF	-	Enviada	Eventos Adversos Relacionados à	Falhas durante procedimento		

28/05/2019	CE	FORTALEZA	HGF	-	Enviada	Eventos Adversos Relacionados à	Queimadura de paciente		
28/05/2019	CE	FORTALEZA	HGF	-	Enviada	Eventos Adversos Relacionados à	Falhas durante a assistência à saúde		
28/05/2019	CE	FORTALEZA	HGF	-	Enviada	Eventos Adversos Relacionados à	Falhas durante procedimento		
28/05/2019	CE	FORTALEZA	HGF	-	Enviada	Eventos Adversos Relacionados à	Falhas durante a assistência à saúde		
31/05/2019	CE	FORTALEZA	HGF	-	Enviada	Eventos Adversos Relacionados à	Queda do paciente		
31/05/2019	CE	FORTALEZA	HGF	-	Enviada	Eventos Adversos Relacionados à	Queda do paciente		
31/05/2019	CE	FORTALEZA	HGF	-	Enviada	Eventos Adversos Relacionados à	Queda do paciente		
31/05/2019	CE	FORTALEZA	HGF	-	Enviada	Eventos Adversos Relacionados à	Queda do paciente		
31/05/2019	CE	FORTALEZA	HGF	-	Enviada	Eventos Adversos Relacionados à	Queda do paciente		
07/06/2019	CE	FORTALEZA	HGF	-	Enviada	Eventos Adversos Relacionados à	Queda do paciente		
07/06/2019	CE	FORTALEZA	HGF	-	Enviada	Eventos Adversos Relacionados à	Queda do paciente		
17/06/2019	CE	FORTALEZA	HGF	-	Enviada	Eventos Adversos Relacionados à	Queda do paciente		
26/06/2019	CE	FORTALEZA	HGF	-	Enviada	Eventos Adversos Relacionados à	Queda do paciente		
26/06/2019	CE	FORTALEZA	HGF	-	Enviada	Eventos Adversos Relacionados à	Queda do paciente		
01/07/2019	CE	FORTALEZA	HGF	-	Enviada	Eventos Adversos Relacionados à	Queda do paciente		

02/07/2019	CE	FORTALE ZA	HGF	-	Enviada	Eventos Adversos Relaciona dos à	Queda do paciente		
08/07/2019	CE	FORTALE ZA	HGF	-	Enviada	Eventos Adversos Relaciona dos à	Queda do paciente		
08/07/2019	CE	FORTALE ZA	HGF	-	Enviada	Eventos Adversos Relaciona dos à	Queda do paciente		
08/07/2019	CE	FORTALE ZA	HGF	-	Enviada	Eventos Adversos Relaciona dos à	Queda do paciente		
08/07/2019	CE	FORTALE ZA	HGF	-	Enviada	Eventos Adversos Relaciona dos à	Queda do paciente		
08/07/2019	CE	FORTALE ZA	HGF	-	Enviada	Eventos Adversos Relaciona dos à	Queda do paciente		
16/08/2019	CE	FORTALE ZA	HGF	-	Enviada	Eventos Adversos Relaciona dos à	Queda do paciente		
16/08/2019	CE	FORTALE ZA	HGF	-	Enviada	Eventos Adversos Relaciona dos à	Queda do paciente		
16/08/2019	CE	FORTALE ZA	HGF	-	Enviada	Eventos Adversos Relaciona dos à	Queda do paciente		
16/08/2019	CE	FORTALE ZA	HGF	-	Enviada	Eventos Adversos Relaciona dos à	Queda do paciente		
16/08/2019	CE	FORTALE ZA	HGF	-	Enviada	Eventos Adversos Relaciona dos à	Queda do paciente		
23/08/2019	CE	FORTALE ZA	HGF	-	Enviada	Eventos Adversos Relaciona dos à	Queda do paciente		
29/08/2019	CE	FORTALE ZA	HGF	-	Enviada	Eventos Adversos Relaciona dos à	Queda do paciente		
06/09/2019	CE	FORTALE ZA	HGF	EVENTO ADVERS O LEVE	Enviada	Eventos Adversos Relaciona dos à	Queda do paciente		
06/09/2019	CE	FORTALE ZA	HGF	-	Enviada	Eventos Adversos Relaciona dos à	Queda do paciente		

06/09/2019	CE	FORTALEZA	HGF	EVENTO ADVERSO LEVE	Enviada	Eventos Adversos Relacionados à	Queda do paciente		
06/09/2019	CE	FORTALEZA	HGF	EVENTO ADVERSO LEVE	Enviada	Eventos Adversos Relacionados à	Queda do paciente		
09/10/2019	CE	FORTALEZA	HGF	ERRO DE MEDICAÇAO	Enviada	Eventos Adversos Relacionados à	Falhas durante a assistência à saúde		
18/10/2019	CE	FORTALEZA	HGF	paciente apresentou queda do leito	Enviada	Eventos Adversos Relacionados à	Queda do paciente		
18/10/2019	CE	FORTALEZA	HGF	O PROCEDIMENTO COLOCA	Enviada	Eventos Adversos Relacionados à	Falha na documentação		
21/10/2019	CE	FORTALEZA	HGF	QUEDA NO BANHEIRO	Enviada	Eventos Adversos Relacionados à	Queda do paciente		
21/10/2019	CE	FORTALEZA	HGF	PRONTUARIO	Enviada	Eventos Adversos Relacionados à	Falha na documentação		
21/10/2019	CE	FORTALEZA	HGF	QUEDA	Enviada	Eventos Adversos Relacionados à	Queda do paciente		
21/10/2019	CE	FORTALEZA	HGF	QUEDA	Enviada	Eventos Adversos Relacionados à	Queda do paciente		
21/10/2019	CE	FORTALEZA	HGF	QUEDA BANHEIRO	Enviada	Eventos Adversos Relacionados à	Queda do paciente		
21/10/2019	CE	FORTALEZA	HGF	HIGIENIZAÇÃO DAS MÃOS	Enviada	Eventos Adversos Relacionados à	Falhas durante a assistência à saúde		
21/10/2019	CE	FORTALEZA	HGF	QUEIMADURA COM BISTURI	Enviada	Eventos Adversos Relacionados à	Queimadura de paciente		
21/10/2019	CE	FORTALEZA	HGF	QUEDA CADEIRA	Enviada	Eventos Adversos Relacionados à	Queda do paciente		
21/10/2019	CE	FORTALEZA	HGF	QUEDA	Enviada	Eventos Adversos Relacionados à	Queda do paciente		
27/01/2020	CE	FORTALEZA	HGF	EVENTO ADVERSO LEVE	Enviada	Eventos Adversos Relacionados à	Queda do paciente		

27/01/2020	CE	FORTALEZA	HGF	EVENTO ADVERSO LEVE	Enviada	Eventos Adversos Relacionados à	Queda do paciente		
27/01/2020	CE	FORTALEZA	HGF	EVENTO ADVERSO LEVE	Enviada	Eventos Adversos Relacionados à	Queda do paciente		
27/01/2020	CE	FORTALEZA	HGF	EVENTO ADVERSO LEVE	Enviada	Eventos Adversos Relacionados à	Queda do paciente		
27/01/2020	CE	FORTALEZA	HGF	EVENTO ADVERSO LEVE	Enviada	Eventos Adversos Relacionados à	Queda do paciente		
18/02/2020	CE	FORTALEZA	HGF	QUEDA	Enviada	Eventos Adversos Relacionados à	Queda do paciente		
18/02/2020	CE	FORTALEZA	HGF	QUEDA DA CAMA	Enviada	Eventos Adversos Relacionados à	Queda do paciente		
18/02/2020	CE	FORTALEZA	HGF	QUEDA BANHEIRO	Enviada	Eventos Adversos Relacionados à	Queda do paciente		
19/02/2020	CE	FORTALEZA	HGF	LESÃO NA REGIÃO SACRA	Enviada	Eventos Adversos Relacionados à	Lesão por pressão		
19/02/2020	CE	FORTALEZA	HGF	LESÃO DE PELE REGIÃO SACRA	Enviada	Eventos Adversos Relacionados à	Lesão por pressão		
19/02/2020	CE	FORTALEZA	HGF	LESÃO DE PELE REGIÃO SACRA	Enviada	Eventos Adversos Relacionados à	Lesão por pressão		
19/02/2020	CE	FORTALEZA	HGF	LESÃO REGIÃO SACRA	Enviada	Eventos Adversos Relacionados à	Lesão por pressão		
19/02/2020	CE	FORTALEZA	HGF	LESÃO DE PELE REGIÃO CALCÂN	Enviada	Eventos Adversos Relacionados à	Lesão por pressão		
19/02/2020	CE	FORTALEZA	HGF	LESÃO CALCÂNEO E	Enviada	Eventos Adversos Relacionados à	Lesão por pressão		
19/02/2020	CE	FORTALEZA	HGF	LESÃO REGIÃO SACRA	Enviada	Eventos Adversos Relacionados à	Lesão por pressão		
20/02/2020	CE	FORTALEZA	HGF	LESÃO REGIÃO SACRA	Enviada	Eventos Adversos Relacionados à	Lesão por pressão		

20/02/2020	CE	FORTALEZA	HGF	LESAO SACRA	Enviada	Eventos Adversos Relaciona dos à	Lesão por pressão		
20/02/2020	CE	FORTALEZA	HGF	LESÃO REGIÃO SACRA	Enviada	Eventos Adversos Relaciona dos à	Lesão por pressão		
20/02/2020	CE	FORTALEZA	HGF	LESÃO REGIÃO SACRA	Enviada	Eventos Adversos Relaciona dos à	Lesão por pressão		
20/02/2020	CE	FORTALEZA	HGF	LESAO REGIAO SACRA	Enviada	Eventos Adversos Relaciona dos à	Lesão por pressão		
20/02/2020	CE	FORTALEZA	HGF	LESÃO REGIÃO SACRA	Enviada	Eventos Adversos Relaciona dos à	Lesão por pressão		
20/02/2020	CE	FORTALEZA	HGF	LESÃO OCIPITAL	Enviada	Eventos Adversos Relaciona dos à	Lesão por pressão		
02/03/2020	CE	FORTALEZA	HGF	QUEDA DA MACA	Enviada	Eventos Adversos Relaciona dos à	Queda do paciente		
02/03/2020	CE	FORTALEZA	HGF	-	Enviada	Eventos Adversos Relaciona dos à	Queda do paciente		
02/03/2020	CE	FORTALEZA	HGF	QUEDA DA CAMA	Enviada	Eventos Adversos Relaciona dos à	Queda do paciente		
02/03/2020	CE	FORTALEZA	HGF	-	Enviada	Eventos Adversos Relaciona dos à	Queda do paciente		
03/03/2020	CE	FORTALEZA	HGF	queda	Enviada	Eventos Adversos Relaciona dos à	Queda do paciente		
03/03/2020	CE	FORTALEZA	HGF	queda	Enviada	Eventos Adversos Relaciona dos à	Queda do paciente		
03/03/2020	CE	FORTALEZA	HGF	QUEDA	Enviada	Eventos Adversos Relaciona dos à	Queda do paciente		
03/03/2020	CE	FORTALEZA	HGF	QUEDA	Enviada	Eventos Adversos Relaciona dos à	Queda do paciente		
03/03/2020	CE	FORTALEZA	HGF	QUEDA	Enviada	Eventos Adversos Relaciona dos à	Queda do paciente		

12/03/2020	CE	FORTALEZA	HGF	QUEDA	Enviada	Eventos Adversos Relacionados à	Queda do paciente		
12/03/2020	CE	FORTALEZA	HGF	QUEDA	Enviada	Eventos Adversos Relacionados à	Queda do paciente		
13/03/2020	CE	FORTALEZA	HGF	LESAO DE PELE	Enviada	Eventos Adversos Relacionados à	Lesão por pressão		
13/03/2020	CE	FORTALEZA	HGF	LESAO DE PELE	Enviada	Eventos Adversos Relacionados à	Lesão por pressão		
13/03/2020	CE	FORTALEZA	HGF	LESAO DE PELE	Enviada	Eventos Adversos Relacionados à	Lesão por pressão		
13/03/2020	CE	FORTALEZA	HGF	LESAO DE PELE	Enviada	Eventos Adversos Relacionados à	Lesão por pressão		
13/03/2020	CE	FORTALEZA	HGF	LESAO DE PELE	Enviada	Eventos Adversos Relacionados à	Lesão por pressão		
13/03/2020	CE	FORTALEZA	HGF	LESAO DE PELE	Enviada	Eventos Adversos Relacionados à	Lesão por pressão		
25/03/2020	CE	FORTALEZA	HGF	-	Enviada	Eventos Adversos Relacionados à	Falhas durante a assistência à saúde		
31/03/2020	CE	FORTALEZA	HGF	QUEDA	Enviada	Eventos Adversos Relacionados à	Queda do paciente		
31/03/2020	CE	FORTALEZA	HGF	MEDICACAO	Enviada	Eventos Adversos Relacionados à	Falhas durante a assistência à saúde		
31/03/2020	CE	FORTALEZA	HGF	-	Enviada	Eventos Adversos Relacionados à	Extubação endotraqueal		
31/03/2020	CE	FORTALEZA	HGF	TRANSPORTE	Enviada	Eventos Adversos Relacionados à	Falhas durante a assistência à saúde		
27/07/2020	CE	FORTALEZA	HGF	circunstância notificável	Enviada	Eventos Adversos Relacionados à	Falhas durante a assistência à saúde		
29/07/2020	CE	FORTALEZA	HGF	QUEDA	Enviada	Eventos Adversos Relacionados à	Queda do paciente		

30/07/2020	CE	FORTALEZA	HGF	circunstancia notificavel	Enviada	Eventos Adversos Relacionados à	Falha na documentação		
30/07/2020	CE	FORTALEZA	HGF	circunstancia notificável	Enviada	Eventos Adversos Relacionados à	Falhas durante a assistência à saúde		
30/07/2020	CE	FORTALEZA	HGF	circunstancia notificavel	Enviada	Eventos Adversos Relacionados à	Falha na identificação do paciente		
03/08/2020	CE	FORTALEZA	HGF	QUEDA	Enviada	Eventos Adversos Relacionados à	Queda do paciente		
04/08/2020	CE	FORTALEZA	HGF	QUEDA	Enviada	Eventos Adversos Relacionados à	Queda do paciente		
04/08/2020	CE	FORTALEZA	HGF	QUEDA	Enviada	Eventos Adversos Relacionados à	Queda do paciente		
04/08/2020	CE	FORTALEZA	HGF	QUEDA	Enviada	Eventos Adversos Relacionados à	Queda do paciente		
04/08/2020	CE	FORTALEZA	HGF	CAIU DA MACA	Enviada	Eventos Adversos Relacionados à	Queda do paciente		
04/08/2020	CE	FORTALEZA	HGF	ESCORREGOU DA MACA	Enviada	Eventos Adversos Relacionados à	Queda do paciente		
06/08/2020	CE	FORTALEZA	HGF	queda	Enviada	Eventos Adversos Relacionados à	Queda do paciente		
06/08/2020	CE	FORTALEZA	HGF	queda	Enviada	Eventos Adversos Relacionados à	Queda do paciente		
06/08/2020	CE	FORTALEZA	HGF	queda	Enviada	Eventos Adversos Relacionados à	Queda do paciente		
06/08/2020	CE	FORTALEZA	HGF	queda	Enviada	Eventos Adversos Relacionados à	Queda do paciente		
06/08/2020	CE	FORTALEZA	HGF	queda	Enviada	Eventos Adversos Relacionados à	Queda do paciente		
06/08/2020	CE	FORTALEZA	HGF	queda	Enviada	Eventos Adversos Relacionados à	Queda do paciente		

06/08/2020	CE	FORTALEZA	HGF	queda	Enviada	Eventos Adversos Relacionados à	Queda do paciente		
06/08/2020	CE	FORTALEZA	HGF	QUEDA	Enviada	Eventos Adversos Relacionados à	Queda do paciente		
06/08/2020	CE	FORTALEZA	HGF	QUEDA	Enviada	Eventos Adversos Relacionados à	Queda do paciente		
20/08/2020	CE	FORTALEZA	HGF	-	Enviada	Eventos Adversos Relacionados à	Lesão por pressão		
31/08/2020	CE	FORTALEZA	HGF	FALTA DE IDENTIFICAÇÃO	Enviada	Eventos Adversos Relacionados à	Falha na identificação do paciente		
31/08/2020	CE	FORTALEZA	HGF	ADMINISTRAÇÃO ERRADA DE	Enviada	Eventos Adversos Relacionados à	Falhas durante a assistência à saúde		
31/08/2020	CE	FORTALEZA	HGF	PACIENTE DESACOMPANHA	Enviada	Eventos Adversos Relacionados à	Queda do paciente		
31/08/2020	CE	FORTALEZA	HGF	PACIENTE DESACOMPANHA	Enviada	Eventos Adversos Relacionados à	Queda do paciente		
31/08/2020	CE	FORTALEZA	HGF	PACIENTE CAIU AO TENTAR	Enviada	Eventos Adversos Relacionados à	Queda do paciente		
31/08/2020	CE	FORTALEZA	HGF	ADMINISTRAÇÃO ERRADA DA	Enviada	Eventos Adversos Relacionados à	Falhas durante a assistência à saúde		
31/08/2020	CE	FORTALEZA	HGF	-	Enviada	Eventos Adversos Relacionados à	Lesão por pressão		
01/09/2020	CE	FORTALEZA	HGF	PACIENTE DESEQUILIBROU	Enviada	Eventos Adversos Relacionados à	Queda do paciente		
09/09/2020	CE	FORTALEZA	HGF	PACIENTE DESORIENTADA	Enviada	Eventos Adversos Relacionados à	Queda do paciente		
09/09/2020	CE	FORTALEZA	HGF	ACOMPANHANTE FOI AJUDAR	Enviada	Eventos Adversos Relacionados à	Queda do paciente		
09/09/2020	CE	FORTALEZA	HGF	FALHA NA ADMINISTRAÇÃO	Enviada	Eventos Adversos Relacionados à	Falhas durante a assistência à saúde		

05/10/2020	CE	FORTALEZA	HGF	ULCERA EM REGIÃO SACRA	Enviada	Eventos Adversos Relacionados à	Lesão por pressão		
06/10/2020	CE	FORTALEZA	HGF	PACIENTE CAIU DE LADO	Enviada	Eventos Adversos Relacionados à	Queda do paciente		
06/10/2020	CE	FORTALEZA	HGF	ULCERA EM REGIÃO SACRA	Enviada	Eventos Adversos Relacionados à	Lesão por pressão		
06/10/2020	CE	FORTALEZA	HGF	PACIENTE DESORIENTADO	Enviada	Eventos Adversos Relacionados à	Queda do paciente		
06/10/2020	CE	FORTALEZA	HGF	PACIENTE AO FORÇAR A	Enviada	Eventos Adversos Relacionados à	Queda do paciente		
06/10/2020	CE	FORTALEZA	HGF	ULCERA POR PRESSÃO	Enviada	Eventos Adversos Relacionados à	Lesão por pressão		
06/10/2020	CE	FORTALEZA	HGF	DURANTE O BANHO PACIENT	Enviada	Eventos Adversos Relacionados à	Queda do paciente		
06/10/2020	CE	FORTALEZA	HGF	EXTENSORES E CONECTORES	Enviada	Eventos Adversos Relacionados à	Falhas durante a assistência à saúde		
07/10/2020	CE	FORTALEZA	HGF	UPP ESTAGIO III	Enviada	Eventos Adversos Relacionados à	Lesão por pressão		
07/10/2020	CE	FORTALEZA	HGF	UPP ESTAGIO III	Enviada	Eventos Adversos Relacionados à	Lesão por pressão		
07/10/2020	CE	FORTALEZA	HGF	UPP ESTAGIO III	Enviada	Eventos Adversos Relacionados à	Lesão por pressão		
07/10/2020	CE	FORTALEZA	HGF	UPP ESTAGIO III	Enviada	Eventos Adversos Relacionados à	Lesão por pressão		
07/10/2020	CE	FORTALEZA	HGF	UPP ESTAGIO III	Enviada	Eventos Adversos Relacionados à	Lesão por pressão		
07/10/2020	CE	FORTALEZA	HGF	UPP LESAO III	Enviada	Eventos Adversos Relacionados à	Lesão por pressão		
07/10/2020	CE	FORTALEZA	HGF	UPP ESTAGIO III	Enviada	Eventos Adversos Relacionados à	Lesão por pressão		

07/10/2020	CE	FORTALE ZA	HGF	UPP ESTAGIO III	Enviada	Eventos Adversos Relaciona dos à	Lesão por pressão		
07/10/2020	CE	FORTALE ZA	HGF	UPP ESTAGIO III	Enviada	Eventos Adversos Relaciona dos à	Lesão por pressão		
07/10/2020	CE	FORTALE ZA	HGF	UPP ESTAGIO IV	Enviada	Eventos Adversos Relaciona dos à	Lesão por pressão		
07/10/2020	CE	FORTALE ZA	HGF	UPP ESTAGIO III	Enviada	Eventos Adversos Relaciona dos à	Lesão por pressão		
07/10/2020	CE	FORTALE ZA	HGF	UPP ESTÁGIO III	Enviada	Eventos Adversos Relaciona dos à	Lesão por pressão		
07/10/2020	CE	FORTALE ZA	HGF	UPP ESTÁGIO III	Enviada	Eventos Adversos Relaciona dos à	Lesão por pressão		
07/10/2020	CE	FORTALE ZA	HGF	UPP ESTÁGIO III	Enviada	Eventos Adversos Relaciona dos à	Lesão por pressão		
07/10/2020	CE	FORTALE ZA	HGF	UPP ESTÁGIO III	Enviada	Eventos Adversos Relaciona dos à	Lesão por pressão		
07/10/2020	CE	FORTALE ZA	HGF	UPP ESTÁGIO IV	Enviada	Eventos Adversos Relaciona dos à	Lesão por pressão		
07/10/2020	CE	FORTALE ZA	HGF	UPP ESTÁGIO IV	Enviada	Eventos Adversos Relaciona dos à	Lesão por pressão		
07/10/2020	CE	FORTALE ZA	HGF	UPP ESTÁGIO III	Enviada	Eventos Adversos Relaciona dos à	Lesão por pressão		
07/10/2020	CE	FORTALE ZA	HGF	UPP ESTÁGIO III	Enviada	Eventos Adversos Relaciona dos à	Lesão por pressão		
07/10/2020	CE	FORTALE ZA	HGF	PACIENT E AO TENTAR LEVANTA	Enviada	Eventos Adversos Relaciona dos à	Queda do paciente		
13/10/2020	CE	FORTALE ZA	HGF	PACIENT E AO TENTAR SENTAR-	Enviada	Eventos Adversos Relaciona dos à	Queda do paciente		
21/10/2020	CE	FORTALE ZA	HGF	UPP EM CALCÂN EO DIREITO	Enviada	Eventos Adversos Relaciona dos à	Lesão por pressão		

21/10/2020	CE	FORTALEZA	HGF	UPP ESTÁGIO IV EM CALCÂN	Enviada	Eventos Adversos Relacionados à	Lesão por pressão		
21/10/2020	CE	FORTALEZA	HGF	UPP SACRA ESTÁGIO IV	Enviada	Eventos Adversos Relacionados à	Lesão por pressão		
21/10/2020	CE	FORTALEZA	HGF	UPP SACRA ESTÁGIO III	Enviada	Eventos Adversos Relacionados à	Lesão por pressão		
21/10/2020	CE	FORTALEZA	HGF	UPP SACRA ESTÁGIO II	Enviada	Eventos Adversos Relacionados à	Lesão por pressão		
21/10/2020	CE	FORTALEZA	HGF	UPP SACRA ESTÁGIO IV	Enviada	Eventos Adversos Relacionados à	Lesão por pressão		
21/10/2020	CE	FORTALEZA	HGF	UPP SACRA ESTÁGIO IV	Enviada	Eventos Adversos Relacionados à	Lesão por pressão		
21/10/2020	CE	FORTALEZA	HGF	UPP SACRA ESTÁGIO IV	Enviada	Eventos Adversos Relacionados à	Lesão por pressão		
21/10/2020	CE	FORTALEZA	HGF	UPP SACRA ESTÁGIO IV	Enviada	Eventos Adversos Relacionados à	Lesão por pressão		
21/10/2020	CE	FORTALEZA	HGF	UPP SACRA ESTÁGIO IV	Enviada	Eventos Adversos Relacionados à	Lesão por pressão		
21/10/2020	CE	FORTALEZA	HGF	UPP SACRA ESTÁGIO IV	Enviada	Eventos Adversos Relacionados à	Lesão por pressão		
21/10/2020	CE	FORTALEZA	HGF	UPP SACRA ESTÁGIO IV	Enviada	Eventos Adversos Relacionados à	Lesão por pressão		
21/10/2020	CE	FORTALEZA	HGF	UPP SACRA ESTÁGIO IV	Enviada	Eventos Adversos Relacionados à	Lesão por pressão		
21/10/2020	CE	FORTALEZA	HGF	UPP SACRA ESTÁGIO IV	Enviada	Eventos Adversos Relacionados à	Lesão por pressão		
21/10/2020	CE	FORTALEZA	HGF	UPP SACRA ESTÁGIO IV	Enviada	Eventos Adversos Relacionados à	Lesão por pressão		
21/10/2020	CE	FORTALEZA	HGF	UPP SACRA ESTÁGIO IV	Enviada	Eventos Adversos Relacionados à	Lesão por pressão		

21/10/2020	CE	FORTALEZA	HGF	UPP SACRA ESTÁGIO III	Enviada	Eventos Adversos Relacionados à	Lesão por pressão		
21/10/2020	CE	FORTALEZA	HGF	UPP SACRA ESTÁGIO III	Enviada	Eventos Adversos Relacionados à	Lesão por pressão		
21/10/2020	CE	FORTALEZA	HGF	UPP SACRA ESTÁGIO III	Enviada	Eventos Adversos Relacionados à	Lesão por pressão		
21/10/2020	CE	FORTALEZA	HGF	UPP SACRA ESTÁGIO III	Enviada	Eventos Adversos Relacionados à	Lesão por pressão		
21/10/2020	CE	FORTALEZA	HGF	UPP SACRA ESTÁGIO III	Enviada	Eventos Adversos Relacionados à	Lesão por pressão		
21/10/2020	CE	FORTALEZA	HGF	UPP SACRA ESTÁGIO III	Enviada	Eventos Adversos Relacionados à	Lesão por pressão		
21/10/2020	CE	FORTALEZA	HGF	UPP SACRA ESTÁGIO III	Enviada	Eventos Adversos Relacionados à	Lesão por pressão		
21/10/2020	CE	FORTALEZA	HGF	UPP CALCANEO ESQUER	Enviada	Eventos Adversos Relacionados à	Lesão por pressão		
21/10/2020	CE	FORTALEZA	HGF	UPP TROCANTERES ESTÁGIO	Enviada	Eventos Adversos Relacionados à	Lesão por pressão		
21/10/2020	CE	FORTALEZA	HGF	UPP TROCANTERES ESTÁGIO	Enviada	Eventos Adversos Relacionados à	Lesão por pressão		
21/10/2020	CE	FORTALEZA	HGF	UPP ESTÁGIO IV EM REGIÃO	Enviada	Eventos Adversos Relacionados à	Lesão por pressão		
21/10/2020	CE	FORTALEZA	HGF	UPP ESTÁGIO IV EM REGIÃO	Enviada	Eventos Adversos Relacionados à	Lesão por pressão		
21/10/2020	CE	FORTALEZA	HGF	UPP ESTÁGIO IV EM REGIÃO	Enviada	Eventos Adversos Relacionados à	Lesão por pressão		
21/10/2020	CE	FORTALEZA	HGF	UPP ESTÁGIO IV EM REGIÃO	Enviada	Eventos Adversos Relacionados à	Lesão por pressão		
21/10/2020	CE	FORTALEZA	HGF	UPP ESTÁGIO IV EM REGIÃO	Enviada	Eventos Adversos Relacionados à	Lesão por pressão		

21/10/2020	CE	FORTALE ZA	HGF	UPP ESTÁGIO III EM REGIÃO	Enviada	Eventos Adversos Relaciona dos à	Lesão por pressão		
21/10/2020	CE	FORTALE ZA	HGF	UPP ESTÁGIO III EM REGIÃO	Enviada	Eventos Adversos Relaciona dos à	Lesão por pressão		
21/10/2020	CE	FORTALE ZA	HGF	UPP ESTÁGIO III EM REGIÃO	Enviada	Eventos Adversos Relaciona dos à	Lesão por pressão		
21/10/2020	CE	FORTALE ZA	HGF	UPP ESTÁGIO III EM REGIÃO	Enviada	Eventos Adversos Relaciona dos à	Lesão por pressão		
21/10/2020	CE	FORTALE ZA	HGF	UPP ESTÁGIO III EM REGIÃO	Enviada	Eventos Adversos Relaciona dos à	Lesão por pressão		
21/10/2020	CE	FORTALE ZA	HGF	UPP ESTÁGIO III EM REGIÃO	Enviada	Eventos Adversos Relaciona dos à	Lesão por pressão		
21/10/2020	CE	FORTALE ZA	HGF	UPP ESTÁGIO III EM REGIÃO	Enviada	Eventos Adversos Relaciona dos à	Lesão por pressão		
21/10/2020	CE	FORTALE ZA	HGF	UPP ESTÁGIO III EM REGIÃO	Enviada	Eventos Adversos Relaciona dos à	Lesão por pressão		
21/10/2020	CE	FORTALE ZA	HGF	UPP ESTÁGIO III EM REGIÃO	Enviada	Eventos Adversos Relaciona dos à	Lesão por pressão		
21/10/2020	CE	FORTALE ZA	HGF	UPP ESTÁGIO III EM REGIÃO	Enviada	Eventos Adversos Relaciona dos à	Lesão por pressão		
21/10/2020	CE	FORTALE ZA	HGF	UPP ESTÁGIO IV TROCAN	Enviada	Eventos Adversos Relaciona dos à	Lesão por pressão		
21/10/2020	CE	FORTALE ZA	HGF	UPP ESTÁGIO IV EM TROCAN	Enviada	Eventos Adversos Relaciona dos à	Lesão por pressão		
21/10/2020	CE	FORTALE ZA	HGF	UPP ESTÁGIO III EM TROCAN	Enviada	Eventos Adversos Relaciona dos à	Lesão por pressão		
21/10/2020	CE	FORTALE ZA	HGF	UPP ESTÁGIO III EM ORELHA	Enviada	Eventos Adversos Relaciona dos à	Lesão por pressão		
21/10/2020	CE	FORTALE ZA	HGF	UPP ESTÁGIO III EM ORELHA	Enviada	Eventos Adversos Relaciona dos à	Lesão por pressão		

21/10/2020	CE	FORTALEZA	HGF	UPP ESTÁGIO IV EM OREI HA	Enviada	Eventos Adversos Relacionados à	Lesão por pressão		
21/10/2020	CE	FORTALEZA	HGF	UPP ESTÁGIO III EM GLÚTEO	Enviada	Eventos Adversos Relacionados à	Lesão por pressão		
21/10/2020	CE	FORTALEZA	HGF	-	Enviada	Eventos Adversos Relacionados à	Lesão por pressão		
21/10/2020	CE	FORTALEZA	HGF	UPP ESTÁGIO III EM GLÚTEO	Enviada	Eventos Adversos Relacionados à	Lesão por pressão		
21/10/2020	CE	FORTALEZA	HGF	UPP ESTÁGIO III EM GLÚTEO	Enviada	Eventos Adversos Relacionados à	Lesão por pressão		
21/10/2020	CE	FORTALEZA	HGF	UPP ESTÁGIO III EM OCCIPTO	Enviada	Eventos Adversos Relacionados à	Lesão por pressão		
21/10/2020	CE	FORTALEZA	HGF	UPP ESTÁGIO III EM OCCIPTO	Enviada	Eventos Adversos Relacionados à	Lesão por pressão		
21/10/2020	CE	FORTALEZA	HGF	UPP ESTÁGIO III EM CALCAN	Enviada	Eventos Adversos Relacionados à	Lesão por pressão		
22/10/2020	CE	FORTALEZA	HGF	UPP SACRA IV	Enviada	Eventos Adversos Relacionados à	Lesão por pressão		
22/10/2020	CE	FORTALEZA	HGF	UPP SACRA IV	Enviada	Eventos Adversos Relacionados à	Lesão por pressão		
22/10/2020	CE	FORTALEZA	HGF	UPP SACRA IV	Enviada	Eventos Adversos Relacionados à	Lesão por pressão		
22/10/2020	CE	FORTALEZA	HGF	UPP SACRA IV	Enviada	Eventos Adversos Relacionados à	Lesão por pressão		
22/10/2020	CE	FORTALEZA	HGF	UPP SACRA IV	Enviada	Eventos Adversos Relacionados à	Lesão por pressão		
22/10/2020	CE	FORTALEZA	HGF	UPP SACRA IV	Enviada	Eventos Adversos Relacionados à	Lesão por pressão		
22/10/2020	CE	FORTALEZA	HGF	UPP SACRA IV	Enviada	Eventos Adversos Relacionados à	Lesão por pressão		

22/10/2020	CE	FORTALE ZA	HGF	UPP SACRA IV	Enviada	Eventos Adversos Relaciona dos à	Lesão por pressão		
22/10/2020	CE	FORTALE ZA	HGF	UPP SACRA IV	Enviada	Eventos Adversos Relaciona dos à	Lesão por pressão		
22/10/2020	CE	FORTALE ZA	HGF	UPP SACRA IV	Enviada	Eventos Adversos Relaciona dos à	Lesão por pressão		
22/10/2020	CE	FORTALE ZA	HGF	UPP SACRA IV	Enviada	Eventos Adversos Relaciona dos à	Lesão por pressão		
22/10/2020	CE	FORTALE ZA	HGF	UPP SACRA IV	Enviada	Eventos Adversos Relaciona dos à	Lesão por pressão		
22/10/2020	CE	FORTALE ZA	HGF	UPP SACRA III	Enviada	Eventos Adversos Relaciona dos à	Lesão por pressão		
22/10/2020	CE	FORTALE ZA	HGF	UPP SACRA III	Enviada	Eventos Adversos Relaciona dos à	Lesão por pressão		
22/10/2020	CE	FORTALE ZA	HGF	UPP SACRA III	Enviada	Eventos Adversos Relaciona dos à	Lesão por pressão		
22/10/2020	CE	FORTALE ZA	HGF	UPP SACRA III	Enviada	Eventos Adversos Relaciona dos à	Lesão por pressão		
22/10/2020	CE	FORTALE ZA	HGF	UPP SACRA III	Enviada	Eventos Adversos Relaciona dos à	Lesão por pressão		
22/10/2020	CE	FORTALE ZA	HGF	UPP SACRA III	Enviada	Eventos Adversos Relaciona dos à	Lesão por pressão		
22/10/2020	CE	FORTALE ZA	HGF	UPP SACRA III	Enviada	Eventos Adversos Relaciona dos à	Lesão por pressão		
22/10/2020	CE	FORTALE ZA	HGF	UPP SACRA III	Enviada	Eventos Adversos Relaciona dos à	Lesão por pressão		
22/10/2020	CE	FORTALE ZA	HGF	UPP SACRA III	Enviada	Eventos Adversos Relaciona dos à	Lesão por pressão		
22/10/2020	CE	FORTALE ZA	HGF	UPP SACRA III	Enviada	Eventos Adversos Relaciona dos à	Lesão por pressão		

22/10/2020	CE	FORTALEZA	HGF	UPP SACRA III	Enviada	Eventos Adversos Relacionados à	Lesão por pressão		
22/10/2020	CE	FORTALEZA	HGF	UPP SACRA III	Enviada	Eventos Adversos Relacionados à	Lesão por pressão		
22/10/2020	CE	FORTALEZA	HGF	UPP SACRA III	Enviada	Eventos Adversos Relacionados à	Lesão por pressão		
23/10/2020	CE	FORTALEZA	HGF	UPP EM REGIÃO SACRA	Enviada	Eventos Adversos Relacionados à	Lesão por pressão		
23/10/2020	CE	FORTALEZA	HGF	UPP EM REGIÃO SACRA	Enviada	Eventos Adversos Relacionados à	Lesão por pressão		
23/10/2020	CE	FORTALEZA	HGF	UPP ESTÁGIO III EM REGIÃO	Enviada	Eventos Adversos Relacionados à	Lesão por pressão		
23/10/2020	CE	FORTALEZA	HGF	UPP ESTÁGIO III EM REGIÃO	Enviada	Eventos Adversos Relacionados à	Lesão por pressão		
23/10/2020	CE	FORTALEZA	HGF	UPP ESTÁGIO III EM TROCAN	Enviada	Eventos Adversos Relacionados à	Lesão por pressão		
23/10/2020	CE	FORTALEZA	HGF	UPP ESTÁGIO III EM TROCAN	Enviada	Eventos Adversos Relacionados à	Lesão por pressão		
23/10/2020	CE	FORTALEZA	HGF	UPP ESTÁGIO IV EM REGIÃO	Enviada	Eventos Adversos Relacionados à	Lesão por pressão		
23/10/2020	CE	FORTALEZA	HGF	UPP ESTÁGIO III EM REGIÃO	Enviada	Eventos Adversos Relacionados à	Lesão por pressão		
23/10/2020	CE	FORTALEZA	HGF	UPP ESTÁGIO III EM REGIÃO	Enviada	Eventos Adversos Relacionados à	Lesão por pressão		
23/10/2020	CE	FORTALEZA	HGF	UPP ESTÁGIO III EM REGIÃO	Enviada	Eventos Adversos Relacionados à	Lesão por pressão		
23/10/2020	CE	FORTALEZA	HGF	UPP ESTÁGIO III EM REGIÃO	Enviada	Eventos Adversos Relacionados à	Lesão por pressão		
23/10/2020	CE	FORTALEZA	HGF	UPP ESTÁGIO IV EM REGIÃO	Enviada	Eventos Adversos Relacionados à	Lesão por pressão		

23/10/2020	CE	FORTALEZA	HGF	UPP ESTÁGIO IV EM GLÚTEO	Enviada	Eventos Adversos Relacionados à	Lesão por pressão		
23/10/2020	CE	FORTALEZA	HGF	UPP ESTÁGIO III EM GLÚTEO	Enviada	Eventos Adversos Relacionados à	Lesão por pressão		
23/10/2020	CE	FORTALEZA	HGF	UPP ESTÁGIO III EM GLÚTEO	Enviada	Eventos Adversos Relacionados à	Lesão por pressão		
23/10/2020	CE	FORTALEZA	HGF	UPP ESTÁGIO III EM GLÚTEO	Enviada	Eventos Adversos Relacionados à	Lesão por pressão		
23/10/2020	CE	FORTALEZA	HGF	UPP ESTÁGIO IV EM GLÚTEO	Enviada	Eventos Adversos Relacionados à	Lesão por pressão		
23/10/2020	CE	FORTALEZA	HGF	UPP ESTÁGIO III EM GLÚTEO	Enviada	Eventos Adversos Relacionados à	Lesão por pressão		
23/10/2020	CE	FORTALEZA	HGF	UPP ESTÁGIO IV EM GLÚTEO	Enviada	Eventos Adversos Relacionados à	Lesão por pressão		
23/10/2020	CE	FORTALEZA	HGF	UPP ESTÁGIO IV EM GLÚTEO	Enviada	Eventos Adversos Relacionados à	Lesão por pressão		
23/10/2020	CE	FORTALEZA	HGF	UPP ESTÁGIO IV EM GLÚTEO	Enviada	Eventos Adversos Relacionados à	Lesão por pressão		
23/10/2020	CE	FORTALEZA	HGF	UPP ESTÁGIO IV EM GLÚTEO	Enviada	Eventos Adversos Relacionados à	Lesão por pressão		
23/10/2020	CE	FORTALEZA	HGF	UPP ESTÁGIO III EM CALCAN	Enviada	Eventos Adversos Relacionados à	Lesão por pressão		
23/10/2020	CE	FORTALEZA	HGF	UPP ESTÁGIO III EM MALEOL	Enviada	Eventos Adversos Relacionados à	Lesão por pressão		
23/10/2020	CE	FORTALEZA	HGF	UPP ESTÁGIO III EM ORELHA	Enviada	Eventos Adversos Relacionados à	Lesão por pressão		
23/10/2020	CE	FORTALEZA	HGF	UPP ESTÁGIO III EM ORELHA	Enviada	Eventos Adversos Relacionados à	Lesão por pressão		
23/10/2020	CE	FORTALEZA	HGF	PACIENTE CONSCIENTE	Enviada	Eventos Adversos Relacionados à	Queda do paciente		

23/10/2020	CE	FORTALEZA	HGF	PACIENTE DORMIU SENTAD	Enviada	Eventos Adversos Relacionados à	Queda do paciente		
23/10/2020	CE	FORTALEZA	HGF	PACIENTE AGITADO	Enviada	Eventos Adversos Relacionados à	Queda do paciente		
27/10/2020	CE	FORTALEZA	HGF	PACIENTE ACOMPANHADO	Enviada	Eventos Adversos Relacionados à	Queda do paciente		
03/11/2020	CE	FORTALEZA	HGF	PACIENTE AGITADA CAIU	Enviada	Eventos Adversos Relacionados à	Queda do paciente		
06/11/2020	CE	FORTALEZA	HGF	TECNICA DE ENFERMAGEM	Enviada	Eventos Adversos Relacionados à	Falhas durante a assistência à saúde		
12/11/2020	CE	FORTALEZA	HGF	ULCERA EM SACRA	Enviada	Eventos Adversos Relacionados à	Lesão por pressão		
12/11/2020	CE	FORTALEZA	HGF	ULCERA EM SACRA	Enviada	Eventos Adversos Relacionados à	Lesão por pressão		
12/11/2020	CE	FORTALEZA	HGF	ULCERA EM GLUTEO ESQUER	Enviada	Eventos Adversos Relacionados à	Lesão por pressão		
12/11/2020	CE	FORTALEZA	HGF	ULCERA EM SACRA	Enviada	Eventos Adversos Relacionados à	Lesão por pressão		
12/11/2020	CE	FORTALEZA	HGF	ULCERA EM CALCANEOS	Enviada	Eventos Adversos Relacionados à	Lesão por pressão		
12/11/2020	CE	FORTALEZA	HGF	ULCERA EM SACRA	Enviada	Eventos Adversos Relacionados à	Lesão por pressão		
12/11/2020	CE	FORTALEZA	HGF	ULCERA OCCIPTAL	Enviada	Eventos Adversos Relacionados à	Lesão por pressão		
12/11/2020	CE	FORTALEZA	HGF	ULCERA EM SACRA	Enviada	Eventos Adversos Relacionados à	Lesão por pressão		
12/11/2020	CE	FORTALEZA	HGF	ULCERA EM SACRA	Enviada	Eventos Adversos Relacionados à	Lesão por pressão		
12/11/2020	CE	FORTALEZA	HGF	ULCERA EM SACRA	Enviada	Eventos Adversos Relacionados à	Lesão por pressão		

12/11/2020	CE	FORTALEZA	HGF	ULCERA EM SACRA	Enviada	Eventos Adversos Relacionados à	Lesão por pressão		
12/11/2020	CE	FORTALEZA	HGF	ULCERA EM SACRA	Enviada	Eventos Adversos Relacionados à	Lesão por pressão		
12/11/2020	CE	FORTALEZA	HGF	ULCERA EM SACRA	Enviada	Eventos Adversos Relacionados à	Lesão por pressão		
12/11/2020	CE	FORTALEZA	HGF	ULCERA EM OCCIPTAL	Enviada	Eventos Adversos Relacionados à	Lesão por pressão		
12/11/2020	CE	FORTALEZA	HGF	ULCERA EM CALCANEOS	Enviada	Eventos Adversos Relacionados à	Lesão por pressão		
12/11/2020	CE	FORTALEZA	HGF	ULCERA EM SACRA	Enviada	Eventos Adversos Relacionados à	Lesão por pressão		
12/11/2020	CE	FORTALEZA	HGF	ULCERA EM GLUTEO DIREITO	Enviada	Eventos Adversos Relacionados à	Lesão por pressão		
12/11/2020	CE	FORTALEZA	HGF	ULCERA EM SACRA	Enviada	Eventos Adversos Relacionados à	Lesão por pressão		
12/11/2020	CE	FORTALEZA	HGF	ULCERA EM SACRA	Enviada	Eventos Adversos Relacionados à	Lesão por pressão		
12/11/2020	CE	FORTALEZA	HGF	ULCERA EM SACRA	Enviada	Eventos Adversos Relacionados à	Lesão por pressão		
12/11/2020	CE	FORTALEZA	HGF	ULCERA EM GLUTEOS	Enviada	Eventos Adversos Relacionados à	Lesão por pressão		
12/11/2020	CE	FORTALEZA	HGF	ULCERA EM OCCIPTAL	Enviada	Eventos Adversos Relacionados à	Lesão por pressão		
12/11/2020	CE	FORTALEZA	HGF	ULCERA EM SACRA	Enviada	Eventos Adversos Relacionados à	Lesão por pressão		
12/11/2020	CE	FORTALEZA	HGF	ULCERA EM CALCANEOS	Enviada	Eventos Adversos Relacionados à	Lesão por pressão		
12/11/2020	CE	FORTALEZA	HGF	ULCERA EM MALEOLO	Enviada	Eventos Adversos Relacionados à	Lesão por pressão		

12/11/2020	CE	FORTALEZA	HGF	ULCERA EM SACRA	Enviada	Eventos Adversos Relacionados à	Lesão por pressão			
12/11/2020	CE	FORTALEZA	HGF	ULCERA EM SACRA	Enviada	Eventos Adversos Relacionados à	Lesão por pressão			
12/11/2020	CE	FORTALEZA	HGF	ULCERA EM SACRA	Enviada	Eventos Adversos Relacionados à	Lesão por pressão			
12/11/2020	CE	FORTALEZA	HGF	ULCERA EM SACRA	Enviada	Eventos Adversos Relacionados à	Lesão por pressão			
12/11/2020	CE	FORTALEZA	HGF	ULCERA EM ORELHA	Enviada	Eventos Adversos Relacionados à	Lesão por pressão			
12/11/2020	CE	FORTALEZA	HGF	UPP ESTÁGIO III EM SACRA	Enviada	Eventos Adversos Relacionados à	Lesão por pressão			
12/11/2020	CE	FORTALEZA	HGF	UPP EM SACRA	Enviada	Eventos Adversos Relacionados à	Lesão por pressão			
12/11/2020	CE	FORTALEZA	HGF	UPP EM GLUTEOS	Enviada	Eventos Adversos Relacionados à	Lesão por pressão			
12/11/2020	CE	FORTALEZA	HGF	UPP EM SACRA ESTAGIO IV	Enviada	Eventos Adversos Relacionados à	Lesão por pressão			
12/11/2020	CE	FORTALEZA	HGF	UPP EM SACRA	Enviada	Eventos Adversos Relacionados à	Lesão por pressão			
12/11/2020	CE	FORTALEZA	HGF	UPP SACRA ESTAGIO III	Enviada	Eventos Adversos Relacionados à	Lesão por pressão			
12/11/2020	CE	FORTALEZA	HGF	UPP EM OCCIPITAL ESTAGIO	Enviada	Eventos Adversos Relacionados à	Lesão por pressão			
12/11/2020	CE	FORTALEZA	HGF	UPP EM SACRO ESTÁGIO III	Enviada	Eventos Adversos Relacionados à	Lesão por pressão			
12/11/2020	CE	FORTALEZA	HGF	UPP EM SACRO ESTÁGIO IV	Enviada	Eventos Adversos Relacionados à	Lesão por pressão			
12/11/2020	CE	FORTALEZA	HGF	UPP EM GLUTEO ESTAGIO IV	Enviada	Eventos Adversos Relacionados à	Lesão por pressão			

12/11/2020	CE	FORTALEZA	HGF	UPP EM SACRA IV	Enviada	Eventos Adversos Relacionados à	Lesão por pressão		
12/11/2020	CE	FORTALEZA	HGF	UPP EM SACRO ESTÁGIO II	Enviada	Eventos Adversos Relacionados à	Lesão por pressão		
12/11/2020	CE	FORTALEZA	HGF	UPP EM OCCIPTAL ESTÁGIO	Enviada	Eventos Adversos Relacionados à	Lesão por pressão		
12/11/2020	CE	FORTALEZA	HGF	UPP EM GLUTEOS IV	Enviada	Eventos Adversos Relacionados à	Lesão por pressão		
12/11/2020	CE	FORTALEZA	HGF	UPP EM CALCANEOSESTÁGIO II	Enviada	Eventos Adversos Relacionados à	Lesão por pressão		
12/11/2020	CE	FORTALEZA	HGF	UPP EM SACRO ESTÁGIO III	Enviada	Eventos Adversos Relacionados à	Lesão por pressão		
12/11/2020	CE	FORTALEZA	HGF	UPP EM SACRO ESTÁGIO III	Enviada	Eventos Adversos Relacionados à	Lesão por pressão		
12/11/2020	CE	FORTALEZA	HGF	UPP EM CALCANEO DIREITO	Enviada	Eventos Adversos Relacionados à	Lesão por pressão		
12/11/2020	CE	FORTALEZA	HGF	UPP EM SACRO ESTÁGIO IV	Enviada	Eventos Adversos Relacionados à	Lesão por pressão		
12/11/2020	CE	FORTALEZA	HGF	UPP EM OCCIPTAL ESTÁGIO	Enviada	Eventos Adversos Relacionados à	Lesão por pressão		
12/11/2020	CE	FORTALEZA	HGF	UPP EM CALCANEO DIREITO	Enviada	Eventos Adversos Relacionados à	Lesão por pressão		
12/11/2020	CE	FORTALEZA	HGF	UPP EM SACRO ESTÁGIO IV	Enviada	Eventos Adversos Relacionados à	Lesão por pressão		
12/11/2020	CE	FORTALEZA	HGF	UPP EM CALCANEO ESQUER	Enviada	Eventos Adversos Relacionados à	Lesão por pressão		
12/11/2020	CE	FORTALEZA	HGF	UPP EM OCCIPTAL ESTÁGIO	Enviada	Eventos Adversos Relacionados à	Lesão por pressão		
12/11/2020	CE	FORTALEZA	HGF	UPP EM CALCANEOS ESTÁGIO	Enviada	Eventos Adversos Relacionados à	Lesão por pressão		

13/11/2020	CE	FORTALEZA	HGF	ULCERA EM SACRA	Enviada	Eventos Adversos Relacionados à	Lesão por pressão		
13/11/2020	CE	FORTALEZA	HGF	ULCERA EM SACRA	Enviada	Eventos Adversos Relacionados à	Lesão por pressão		
13/11/2020	CE	FORTALEZA	HGF	ULCERA EM CALCANEO	Enviada	Eventos Adversos Relacionados à	Lesão por pressão		
13/11/2020	CE	FORTALEZA	HGF	ULCERA EM SACRA	Enviada	Eventos Adversos Relacionados à	Lesão por pressão		
13/11/2020	CE	FORTALEZA	HGF	ULCERA EM ORELHA	Enviada	Eventos Adversos Relacionados à	Lesão por pressão		
13/11/2020	CE	FORTALEZA	HGF	ULCERA EM MALEOLO	Enviada	Eventos Adversos Relacionados à	Lesão por pressão		
13/11/2020	CE	FORTALEZA	HGF	ULCERA EM SACRA	Enviada	Eventos Adversos Relacionados à	Lesão por pressão		
13/11/2020	CE	FORTALEZA	HGF	ULCERA EM SACRA	Enviada	Eventos Adversos Relacionados à	Lesão por pressão		
13/11/2020	CE	FORTALEZA	HGF	ULCERA EM SACRA	Enviada	Eventos Adversos Relacionados à	Lesão por pressão		
13/11/2020	CE	FORTALEZA	HGF	ULCERA EM OCCIPTAL	Enviada	Eventos Adversos Relacionados à	Lesão por pressão		
13/11/2020	CE	FORTALEZA	HGF	ULCERA EM SACRA	Enviada	Eventos Adversos Relacionados à	Lesão por pressão		
13/11/2020	CE	FORTALEZA	HGF	ULCERA EM SACRA	Enviada	Eventos Adversos Relacionados à	Lesão por pressão		
13/11/2020	CE	FORTALEZA	HGF	ULCERA EM SACRA	Enviada	Eventos Adversos Relacionados à	Lesão por pressão		
13/11/2020	CE	FORTALEZA	HGF	ULCERA EM ORELHA	Enviada	Eventos Adversos Relacionados à	Lesão por pressão		
13/11/2020	CE	FORTALEZA	HGF	ULCERA EM OCCIPTAL	Enviada	Eventos Adversos Relacionados à	Lesão por pressão		

13/11/2020	CE	FORTALEZA	HGF	ULCERA EM SACRA	Enviada	Eventos Adversos Relacionados à	Lesão por pressão		
13/11/2020	CE	FORTALEZA	HGF	ULCERA EM CALCANEO	Enviada	Eventos Adversos Relacionados à	Lesão por pressão		
13/11/2020	CE	FORTALEZA	HGF	ULCERA EM TROCANTERES	Enviada	Eventos Adversos Relacionados à	Lesão por pressão		
13/11/2020	CE	FORTALEZA	HGF	ULCERA EM GLUTEOS	Enviada	Eventos Adversos Relacionados à	Lesão por pressão		
13/11/2020	CE	FORTALEZA	HGF	ULCERA EM OCCIPTAL	Enviada	Eventos Adversos Relacionados à	Lesão por pressão		
13/11/2020	CE	FORTALEZA	HGF	ULCERA EM GLUTEOS	Enviada	Eventos Adversos Relacionados à	Lesão por pressão		
13/11/2020	CE	FORTALEZA	HGF	ULCERA EM SACRA	Enviada	Eventos Adversos Relacionados à	Lesão por pressão		
13/11/2020	CE	FORTALEZA	HGF	ULCERA EM OCCIPTAL	Enviada	Eventos Adversos Relacionados à	Lesão por pressão		
13/11/2020	CE	FORTALEZA	HGF	ULCERA EM SACRA	Enviada	Eventos Adversos Relacionados à	Lesão por pressão		
13/11/2020	CE	FORTALEZA	HGF	ULCERA EM ORELHA	Enviada	Eventos Adversos Relacionados à	Lesão por pressão		
13/11/2020	CE	FORTALEZA	HGF	ULCERA EM PANTURRILHA	Enviada	Eventos Adversos Relacionados à	Lesão por pressão		
13/11/2020	CE	FORTALEZA	HGF	ULCERA EM SACRA	Enviada	Eventos Adversos Relacionados à	Lesão por pressão		
13/11/2020	CE	FORTALEZA	HGF	ULCERA EM GLUTEOS	Enviada	Eventos Adversos Relacionados à	Lesão por pressão		
13/11/2020	CE	FORTALEZA	HGF	ULCERA EM SACRA	Enviada	Eventos Adversos Relacionados à	Lesão por pressão		
13/11/2020	CE	FORTALEZA	HGF	ULCERA EM ORELHA	Enviada	Eventos Adversos Relacionados à	Lesão por pressão		

13/11/2020	CE	FORTALEZA	HGF	ULCERA EM CALCANEO	Enviada	Eventos Adversos Relacionados à	Lesão por pressão		
13/11/2020	CE	FORTALEZA	HGF	ULCERA EM CALCANEO	Enviada	Eventos Adversos Relacionados à	Lesão por pressão		
13/11/2020	CE	FORTALEZA	HGF	ULCERA EM SACRA	Enviada	Eventos Adversos Relacionados à	Lesão por pressão		
13/11/2020	CE	FORTALEZA	HGF	ULCERA EM SACRA	Enviada	Eventos Adversos Relacionados à	Lesão por pressão		
13/11/2020	CE	FORTALEZA	HGF	ULCERA EM OCCIPTAL	Enviada	Eventos Adversos Relacionados à	Lesão por pressão		
13/11/2020	CE	FORTALEZA	HGF	ULCERA EM TROCANTER	Enviada	Eventos Adversos Relacionados à	Lesão por pressão		
13/11/2020	CE	FORTALEZA	HGF	ULCERA EM MALEOLOS	Enviada	Eventos Adversos Relacionados à	Lesão por pressão		
13/11/2020	CE	FORTALEZA	HGF	ULCERA EM SACRA	Enviada	Eventos Adversos Relacionados à	Lesão por pressão		
13/11/2020	CE	FORTALEZA	HGF	ULCERA EM SACRA	Enviada	Eventos Adversos Relacionados à	Lesão por pressão		
13/11/2020	CE	FORTALEZA	HGF	ULCERA EM SACRA	Enviada	Eventos Adversos Relacionados à	Lesão por pressão		
13/11/2020	CE	FORTALEZA	HGF	ULCERA E, OCCIPTAL	Enviada	Eventos Adversos Relacionados à	Lesão por pressão		
13/11/2020	CE	FORTALEZA	HGF	ULCERA EM CALCANEO	Enviada	Eventos Adversos Relacionados à	Lesão por pressão		
13/11/2020	CE	FORTALEZA	HGF	ULCERA EM CALCANEOS	Enviada	Eventos Adversos Relacionados à	Lesão por pressão		
13/11/2020	CE	FORTALEZA	HGF	ULCERA EM MALEOLOS	Enviada	Eventos Adversos Relacionados à	Lesão por pressão		
13/11/2020	CE	FORTALEZA	HGF	ULCERA EM SACRA	Enviada	Eventos Adversos Relacionados à	Lesão por pressão		

13/11/2020	CE	FORTALEZA	HGF	ULCERA EM ORELHA	Enviada	Eventos Adversos Relacionados à	Lesão por pressão		
13/11/2020	CE	FORTALEZA	HGF	ULCERA EM SACRA	Enviada	Eventos Adversos Relacionados à	Lesão por pressão		
13/11/2020	CE	FORTALEZA	HGF	-	Enviada	Eventos Adversos Relacionados à	Lesão por pressão		
13/11/2020	CE	FORTALEZA	HGF	ULCERA EM TROCANTER	Enviada	Eventos Adversos Relacionados à	Lesão por pressão		
13/11/2020	CE	FORTALEZA	HGF	ULCERA EM CALCANEOS	Enviada	Eventos Adversos Relacionados à	Lesão por pressão		
13/11/2020	CE	FORTALEZA	HGF	ULCERA EM SACRA	Enviada	Eventos Adversos Relacionados à	Lesão por pressão		
13/11/2020	CE	FORTALEZA	HGF	ULCERA EM SACRA	Enviada	Eventos Adversos Relacionados à	Lesão por pressão		
13/11/2020	CE	FORTALEZA	HGF	ULCERA EM SACRA	Enviada	Eventos Adversos Relacionados à	Lesão por pressão		
13/11/2020	CE	FORTALEZA	HGF	ULCERA EM TROCANTER	Enviada	Eventos Adversos Relacionados à	Lesão por pressão		
13/11/2020	CE	FORTALEZA	HGF	ULCERA EM GLUTEOS	Enviada	Eventos Adversos Relacionados à	Lesão por pressão		
13/11/2020	CE	FORTALEZA	HGF	ULCERA EM CALCANEO	Enviada	Eventos Adversos Relacionados à	Lesão por pressão		
13/11/2020	CE	FORTALEZA	HGF	ULCERA EM GLUTEOS	Enviada	Eventos Adversos Relacionados à	Lesão por pressão		
13/11/2020	CE	FORTALEZA	HGF	ULCERA EM SACRA	Enviada	Eventos Adversos Relacionados à	Lesão por pressão		
13/11/2020	CE	FORTALEZA	HGF	ULCERA EM CALCANEOS	Enviada	Eventos Adversos Relacionados à	Lesão por pressão		
13/11/2020	CE	FORTALEZA	HGF	ULCERA EM SACRA	Enviada	Eventos Adversos Relacionados à	Lesão por pressão		

13/11/2020	CE	FORTALE ZA	HGF	ULCERA EM SACRA	Enviada	Eventos Adversos Relaciona dos à	Lesão por pressão		
13/11/2020	CE	FORTALE ZA	HGF	ULCERA EM CALCAN EO	Enviada	Eventos Adversos Relaciona dos à	Lesão por pressão		
17/11/2020	CE	FORTALE ZA	HGF	PACIENT E ESCORR EGOU	Enviada	Eventos Adversos Relaciona dos à	Queda do paciente		
17/11/2020	CE	FORTALE ZA	HGF	PACIENT E APRESE NTOU	Enviada	Eventos Adversos Relaciona dos à	Queda do paciente		
17/11/2020	CE	FORTALE ZA	HGF	ADMINIS TRADO TAZOCIN EM	Enviada	Eventos Adversos Relaciona dos à	Falhas durante a assistênci a à saúde		
23/11/2020	CE	FORTALE ZA	HGF	PACIENT E RELATO U HOJE	Enviada	Eventos Adversos Relaciona dos à	Queda do paciente		
25/11/2020	CE	FORTALE ZA	HGF	APOS SER AVALIAD A PELA	Enviada	Eventos Adversos Relaciona dos à	Queda do paciente		
07/12/2020	CE	FORTALE ZA	HGF	PACIENT E ACOMPA NHADO	Enviada	Eventos Adversos Relaciona dos à	Queda do paciente		
07/12/2020	CE	FORTALE ZA	HGF	PACIENT E J.B.G, 73 ANOS	Enviada	Eventos Adversos Relaciona dos à	Queda do paciente		
09/12/2020	CE	FORTALE ZA	HGF	UPP ESTÁGIO III EM MALÉOL	Enviada	Eventos Adversos Relaciona dos à	Lesão por pressão		
09/12/2020	CE	FORTALE ZA	HGF	UPP ESTÁGIO III EM SACRA	Enviada	Eventos Adversos Relaciona dos à	Lesão por pressão		
09/12/2020	CE	FORTALE ZA	HGF	UPP ESTÁGIO IV EM SACRA	Enviada	Eventos Adversos Relaciona dos à	Lesão por pressão		
09/12/2020	CE	FORTALE ZA	HGF	UPP ESTÁGIO IV EM GLUTEO	Enviada	Eventos Adversos Relaciona dos à	Lesão por pressão		
09/12/2020	CE	FORTALE ZA	HGF	UPP ESTÁGIO III EM TROCAN	Enviada	Eventos Adversos Relaciona dos à	Lesão por pressão		
09/12/2020	CE	FORTALE ZA	HGF	UPP ESTÁGIO III EM TROCAN	Enviada	Eventos Adversos Relaciona dos à	Lesão por pressão		

10/12/2020	CE	FORTALEZA	HGF	UPP EM SACRA GRAU III	Enviada	Eventos Adversos Relacionados à	Lesão por pressão		
10/12/2020	CE	FORTALEZA	HGF	UPP OCCIPTAL GRAU IV	Enviada	Eventos Adversos Relacionados à	Lesão por pressão		
10/12/2020	CE	FORTALEZA	HGF	UPP EM SACRA GRAU IV	Enviada	Eventos Adversos Relacionados à	Lesão por pressão		
10/12/2020	CE	FORTALEZA	HGF	UPP EM OCCIPTO GRAU III	Enviada	Eventos Adversos Relacionados à	Lesão por pressão		
10/12/2020	CE	FORTALEZA	HGF	UPP EM SACRA GRAU III	Enviada	Eventos Adversos Relacionados à	Lesão por pressão		
10/12/2020	CE	FORTALEZA	HGF	ULCERA EM GLÚTEOS	Enviada	Eventos Adversos Relacionados à	Lesão por pressão		
10/12/2020	CE	FORTALEZA	HGF	REGIÃO SACRA	Enviada	Eventos Adversos Relacionados à	Lesão por pressão		
10/12/2020	CE	FORTALEZA	HGF	ULCERA EM CALCANÊOS	Enviada	Eventos Adversos Relacionados à	Lesão por pressão		
10/12/2020	CE	FORTALEZA	HGF	ULCERA EM TROCANTERES	Enviada	Eventos Adversos Relacionados à	Lesão por pressão		
10/12/2020	CE	FORTALEZA	HGF	ULCERA EM TROCANTER	Enviada	Eventos Adversos Relacionados à	Lesão por pressão		
10/12/2020	CE	FORTALEZA	HGF	ULCERA EM SACRA	Enviada	Eventos Adversos Relacionados à	Lesão por pressão		
10/12/2020	CE	FORTALEZA	HGF	ULCERA EM SACRA	Enviada	Eventos Adversos Relacionados à	Lesão por pressão		
10/12/2020	CE	FORTALEZA	HGF	ULCERA EM SACRA	Enviada	Eventos Adversos Relacionados à	Lesão por pressão		
10/12/2020	CE	FORTALEZA	HGF	ULCERA EM SACRA	Enviada	Eventos Adversos Relacionados à	Lesão por pressão		
10/12/2020	CE	FORTALEZA	HGF	ULCERA EM SACRA	Enviada	Eventos Adversos Relacionados à	Lesão por pressão		

10/12/2020	CE	FORTALEZA	HGF	ULCERA EM OCCIPTAL	Enviada	Eventos Adversos Relacionados à	Lesão por pressão		
10/12/2020	CE	FORTALEZA	HGF	ULCERA EM GLUTEOS	Enviada	Eventos Adversos Relacionados à	Lesão por pressão		
10/12/2020	CE	FORTALEZA	HGF	ULCERA EM GLUTEO DIREITO	Enviada	Eventos Adversos Relacionados à	Lesão por pressão		
10/12/2020	CE	FORTALEZA	HGF	ULCERA EM SACRA	Enviada	Eventos Adversos Relacionados à	Lesão por pressão		
10/12/2020	CE	FORTALEZA	HGF	ULCERA EM GLUTEOS	Enviada	Eventos Adversos Relacionados à	Lesão por pressão		
10/12/2020	CE	FORTALEZA	HGF	ULCERA EM OCCIPTAL	Enviada	Eventos Adversos Relacionados à	Lesão por pressão		
10/12/2020	CE	FORTALEZA	HGF	ULCERA EM TROCANTER	Enviada	Eventos Adversos Relacionados à	Lesão por pressão		
10/12/2020	CE	FORTALEZA	HGF	ULCERA EM SACRA	Enviada	Eventos Adversos Relacionados à	Lesão por pressão		
10/12/2020	CE	FORTALEZA	HGF	ULCERA EM SACRA	Enviada	Eventos Adversos Relacionados à	Lesão por pressão		
10/12/2020	CE	FORTALEZA	HGF	ULCERA EM SACRA	Enviada	Eventos Adversos Relacionados à	Lesão por pressão		
10/12/2020	CE	FORTALEZA	HGF	ULCERA EM GLUTEOS	Enviada	Eventos Adversos Relacionados à	Lesão por pressão		
10/12/2020	CE	FORTALEZA	HGF	ULCERA EM SACRA	Enviada	Eventos Adversos Relacionados à	Lesão por pressão		
10/12/2020	CE	FORTALEZA	HGF	ULCERA EM TROCANTERES	Enviada	Eventos Adversos Relacionados à	Lesão por pressão		
10/12/2020	CE	FORTALEZA	HGF	ULCERA EM SACRA	Enviada	Eventos Adversos Relacionados à	Lesão por pressão		
10/12/2020	CE	FORTALEZA	HGF	ULCERA EM SACRA	Enviada	Eventos Adversos Relacionados à	Lesão por pressão		

10/12/2020	CE	FORTALEZA	HGF	ULCERA EM SACRA	Enviada	Eventos Adversos Relacionados à	Lesão por pressão		
10/12/2020	CE	FORTALEZA	HGF	ULCERA EM SACRA	Enviada	Eventos Adversos Relacionados à	Lesão por pressão		
10/12/2020	CE	FORTALEZA	HGF	ULCERA EM GLUTEOS	Enviada	Eventos Adversos Relacionados à	Lesão por pressão		
10/12/2020	CE	FORTALEZA	HGF	ULCERA EM MALEOLO	Enviada	Eventos Adversos Relacionados à	Lesão por pressão		
10/12/2020	CE	FORTALEZA	HGF	ULCERA EM SACRA	Enviada	Eventos Adversos Relacionados à	Lesão por pressão		
14/12/2020	CE	FORTALEZA	HGF	AS 23:40 CHEGAMOS AO LEITO	Enviada	Eventos Adversos Relacionados à	Queda do paciente		
28/12/2020	CE	FORTALEZA	HGF	ULCERA EM SACRA	Enviada	Eventos Adversos Relacionados à	Lesão por pressão		
28/12/2020	CE	FORTALEZA	HGF	ULCERA EM SACRA	Enviada	Eventos Adversos Relacionados à	Lesão por pressão		
28/12/2020	CE	FORTALEZA	HGF	ULCERA EM GLUTEO DIREITO	Enviada	Eventos Adversos Relacionados à	Lesão por pressão		
28/12/2020	CE	FORTALEZA	HGF	17/112020	Enviada	Eventos Adversos Relacionados à	Lesão por pressão		
28/12/2020	CE	FORTALEZA	HGF	ULCERA EM TROCANTER	Enviada	Eventos Adversos Relacionados à	Lesão por pressão		
28/12/2020	CE	FORTALEZA	HGF	UPP EM SACRA ESTÁGIO III	Enviada	Eventos Adversos Relacionados à	Lesão por pressão		
28/12/2020	CE	FORTALEZA	HGF	UPP EM SACRA ESTÁGIO III	Enviada	Eventos Adversos Relacionados à	Lesão por pressão		
28/12/2020	CE	FORTALEZA	HGF	UPP EM SACRA ESTÁGIO IV	Enviada	Eventos Adversos Relacionados à	Lesão por pressão		
28/12/2020	CE	FORTALEZA	HGF	UPP EM TROCANTERES ESTÁGIO	Enviada	Eventos Adversos Relacionados à	Lesão por pressão		

28/12/2020	CE	FORTALEZA	HGF	UPP EM CRISTA ILÍACA ESTÁGIO	Enviada	Eventos Adversos Relacionados à	Lesão por pressão		
28/12/2020	CE	FORTALEZA	HGF	UPP EM COTOVELOS ESTAGIO	Enviada	Eventos Adversos Relacionados à	Lesão por pressão		
28/12/2020	CE	FORTALEZA	HGF	UPP ESTÁGIO IV EM OMBRO	Enviada	Eventos Adversos Relacionados à	Lesão por pressão		
28/12/2020	CE	FORTALEZA	HGF	UPP EM SACRA ESTÁGIO III	Enviada	Eventos Adversos Relacionados à	Lesão por pressão		
28/12/2020	CE	FORTALEZA	HGF	UPP EM SACRA ESTAGIO IV	Enviada	Eventos Adversos Relacionados à	Lesão por pressão		
28/12/2020	CE	FORTALEZA	HGF	UPP EM SACRA ESTÁGIO III	Enviada	Eventos Adversos Relacionados à	Lesão por pressão		
28/12/2020	CE	FORTALEZA	HGF	UPP EM GLUTEOS ESTÁGIO	Enviada	Eventos Adversos Relacionados à	Lesão por pressão		
28/12/2020	CE	FORTALEZA	HGF	UPP EM SACRA IV	Enviada	Eventos Adversos Relacionados à	Lesão por pressão		
28/12/2020	CE	FORTALEZA	HGF	UPP EM ISQUI ESTÁGIO IV	Enviada	Eventos Adversos Relacionados à	Lesão por pressão		
28/12/2020	CE	FORTALEZA	HGF	UPP EM SACRA ESTÁGIO IV	Enviada	Eventos Adversos Relacionados à	Lesão por pressão		
28/12/2020	CE	FORTALEZA	HGF	UPP EM SACRA ESTÁGIO III	Enviada	Eventos Adversos Relacionados à	Lesão por pressão		
28/12/2020	CE	FORTALEZA	HGF	UPP EMPAVILHÃO AURICUL	Enviada	Eventos Adversos Relacionados à	Lesão por pressão		
28/12/2020	CE	FORTALEZA	HGF	UPP EM SACRA ESTAGIO III	Enviada	Eventos Adversos Relacionados à	Lesão por pressão		
28/12/2020	CE	FORTALEZA	HGF	UPP EM SACRA ESTÁGIO III	Enviada	Eventos Adversos Relacionados à	Lesão por pressão		
28/12/2020	CE	FORTALEZA	HGF	UPP EM SACRA ESTÁGIO III	Enviada	Eventos Adversos Relacionados à	Lesão por pressão		

28/12/2020	CE	FORTALE ZA	HGF	UPP EM SACRA ESTÁGIO III	Enviada	Eventos Adversos Relaciona dos à	Lesão por pressão		
28/12/2020	CE	FORTALE ZA	HGF	UPP EM DORSO ESTÁGIO III	Enviada	Eventos Adversos Relaciona dos à	Lesão por pressão		
28/12/2020	CE	FORTALE ZA	HGF	UPP SACRA ESTAGIO IV	Enviada	Eventos Adversos Relaciona dos à	Lesão por pressão		
28/12/2020	CE	FORTALE ZA	HGF	UPP EM SACRA ESTÁGIO IV	Enviada	Eventos Adversos Relaciona dos à	Lesão por pressão		
28/12/2020	CE	FORTALE ZA	HGF	UPP EM ÍSQUIO ESTAGIO IV	Enviada	Eventos Adversos Relaciona dos à	Lesão por pressão		
28/12/2020	CE	FORTALE ZA	HGF	UPP EM ISQUIO DIREITO IV	Enviada	Eventos Adversos Relaciona dos à	Lesão por pressão		
28/12/2020	CE	FORTALE ZA	HGF	UPP EM TROCAN TER ESTAGIO	Enviada	Eventos Adversos Relaciona dos à	Lesão por pressão		
28/12/2020	CE	FORTALE ZA	HGF	UPP EM COXA ESQUER DA	Enviada	Eventos Adversos Relaciona dos à	Lesão por pressão		
28/12/2020	CE	FORTALE ZA	HGF	UPP EM SACRA ESTAGIO III	Enviada	Eventos Adversos Relaciona dos à	Lesão por pressão		
28/12/2020	CE	FORTALE ZA	HGF	UPP EM SACRA ESTAGIO III	Enviada	Eventos Adversos Relaciona dos à	Lesão por pressão		
28/12/2020	CE	FORTALE ZA	HGF	UPP EM OCCIPTO ESTAGIO	Enviada	Eventos Adversos Relaciona dos à	Lesão por pressão		
28/12/2020	CE	FORTALE ZA	HGF	UPP EM SACRA III	Enviada	Eventos Adversos Relaciona dos à	Lesão por pressão		
28/12/2020	CE	FORTALE ZA	HGF	UPP EM TROCAN TERES ESTAGIO	Enviada	Eventos Adversos Relaciona dos à	Lesão por pressão		
28/12/2020	CE	FORTALE ZA	HGF	UPP EM SACRA ESTÁGIO III	Enviada	Eventos Adversos Relaciona dos à	Lesão por pressão		
28/12/2020	CE	FORTALE ZA	HGF	UPP EM CALCAN EOS ESTAGIO	Enviada	Eventos Adversos Relaciona dos à	Lesão por pressão		

28/12/2020	CE	FORTALEZA	HGF	UPP EM OCCIPTAL ESTAGIO	Enviada	Eventos Adversos Relacionados à	Lesão por pressão		
28/12/2020	CE	FORTALEZA	HGF	UPP EM SACRA ESTAGIO III	Enviada	Eventos Adversos Relacionados à	Lesão por pressão		
28/12/2020	CE	FORTALEZA	HGF	UPP EM TROCANTERES III	Enviada	Eventos Adversos Relacionados à	Lesão por pressão		
28/12/2020	CE	FORTALEZA	HGF	UPP EM CRISTA ILÍACA III	Enviada	Eventos Adversos Relacionados à	Lesão por pressão		
28/12/2020	CE	FORTALEZA	HGF	UPP EM COTOVELOS ESTAGIO	Enviada	Eventos Adversos Relacionados à	Lesão por pressão		
28/12/2020	CE	FORTALEZA	HGF	UPP EM OMBRO ESTÁGIO IV	Enviada	Eventos Adversos Relacionados à	Lesão por pressão		
28/12/2020	CE	FORTALEZA	HGF	UPP EM SACRA ESTAGIO IV	Enviada	Eventos Adversos Relacionados à	Lesão por pressão		
28/12/2020	CE	FORTALEZA	HGF	UPP EM CALCANEO DIREITO	Enviada	Eventos Adversos Relacionados à	Lesão por pressão		
28/12/2020	CE	FORTALEZA	HGF	UPP EM SACRA ESTAGIO IV	Enviada	Eventos Adversos Relacionados à	Lesão por pressão		
28/12/2020	CE	FORTALEZA	HGF	UPP EM SACRA IV	Enviada	Eventos Adversos Relacionados à	Lesão por pressão		
28/12/2020	CE	FORTALEZA	HGF	UPP EM GLUTEO DIREITO ESTAGIO	Enviada	Eventos Adversos Relacionados à	Lesão por pressão		
28/12/2020	CE	FORTALEZA	HGF	UPP EM SACRA IV	Enviada	Eventos Adversos Relacionados à	Lesão por pressão		
28/12/2020	CE	FORTALEZA	HGF	UPP EM SACRA ESTAGO III	Enviada	Eventos Adversos Relacionados à	Lesão por pressão		
28/12/2020	CE	FORTALEZA	HGF	UPP EM CALCANEOS III	Enviada	Eventos Adversos Relacionados à	Lesão por pressão		
28/12/2020	CE	FORTALEZA	HGF	UPP EM COTOVELOS GRAU III	Enviada	Eventos Adversos Relacionados à	Lesão por pressão		

28/12/2020	CE	FORTALEZA	HGF	UPP EM MALEOLOS ESTAGIO	Enviada	Eventos Adversos Relacionados à	Lesão por pressão		
28/12/2020	CE	FORTALEZA	HGF	UPP EM SACRA ESTAGIO IV	Enviada	Eventos Adversos Relacionados à	Lesão por pressão		
28/12/2020	CE	FORTALEZA	HGF	UPP EM GLUTEOS ESTAGIO	Enviada	Eventos Adversos Relacionados à	Lesão por pressão		
28/12/2020	CE	FORTALEZA	HGF	UPP EM REGIÃO OCCIPITAL	Enviada	Eventos Adversos Relacionados à	Lesão por pressão		
28/12/2020	CE	FORTALEZA	HGF	UPP EM PAVILHÃO AURICUL	Enviada	Eventos Adversos Relacionados à	Lesão por pressão		
28/12/2020	CE	FORTALEZA	HGF	UPP EM SACRA III	Enviada	Eventos Adversos Relacionados à	Lesão por pressão		
29/12/2020	CE	FORTALEZA	HGF	UPP EM GLUTEO DIREITO III	Enviada	Eventos Adversos Relacionados à	Lesão por pressão		
29/12/2020	CE	FORTALEZA	HGF	UPP EM SACRA ESTAGIO IV	Enviada	Eventos Adversos Relacionados à	Lesão por pressão		
29/12/2020	CE	FORTALEZA	HGF	UPP EM PAVILHÃO AURICUL	Enviada	Eventos Adversos Relacionados à	Lesão por pressão		
29/12/2020	CE	FORTALEZA	HGF	UPP EM PAVILHÃO AURICUL	Enviada	Eventos Adversos Relacionados à	Lesão por pressão		
29/12/2020	CE	FORTALEZA	HGF	UPP EM MALEOLO DIREITO	Enviada	Eventos Adversos Relacionados à	Lesão por pressão		
29/12/2020	CE	FORTALEZA	HGF	UPP EM OCCIPTO ESTAGIO	Enviada	Eventos Adversos Relacionados à	Lesão por pressão		
29/12/2020	CE	FORTALEZA	HGF	UPP EM PANTURRILHA ESTAGIO	Enviada	Eventos Adversos Relacionados à	Lesão por pressão		
29/12/2020	CE	FORTALEZA	HGF	UPP EM TROCANTER DIREITO	Enviada	Eventos Adversos Relacionados à	Lesão por pressão		
29/12/2020	CE	FORTALEZA	HGF	UPP EM GLUTEOS ESTAGIO	Enviada	Eventos Adversos Relacionados à	Lesão por pressão		

29/12/2020	CE	FORTALE ZA	HGF	UPP EM SACRA ESTAGIO IV	Enviada	Eventos Adversos Relaciona dos à	Lesão por pressão		
29/12/2020	CE	FORTALE ZA	HGF	UPP EM SACRA ESTAGIO III	Enviada	Eventos Adversos Relaciona dos à	Lesão por pressão		
29/12/2020	CE	FORTALE ZA	HGF	UPP EM PANTUR RILHA DIREITA	Enviada	Eventos Adversos Relaciona dos à	Lesão por pressão		
29/12/2020	CE	FORTALE ZA	HGF	UPP EM SACRA IV	Enviada	Eventos Adversos Relaciona dos à	Lesão por pressão		
29/12/2020	CE	FORTALE ZA	HGF	UPP EM GLUTEO S ESTAGIO	Enviada	Eventos Adversos Relaciona dos à	Lesão por pressão		
29/12/2020	CE	FORTALE ZA	HGF	UPP EM SACRA ESTAGIO III	Enviada	Eventos Adversos Relaciona dos à	Lesão por pressão		
29/12/2020	CE	FORTALE ZA	HGF	UPP EM SACRA IV	Enviada	Eventos Adversos Relaciona dos à	Lesão por pressão		
29/12/2020	CE	FORTALE ZA	HGF	UPP EM TROCAN TER DIREITO	Enviada	Eventos Adversos Relaciona dos à	Lesão por pressão		
29/12/2020	CE	FORTALE ZA	HGF	UPP EM SACRA ESTAGIO III	Enviada	Eventos Adversos Relaciona dos à	Lesão por pressão		
29/12/2020	CE	FORTALE ZA	HGF	UPP ESTAGIO IV EM SACRA	Enviada	Eventos Adversos Relaciona dos à	Lesão por pressão		
29/12/2020	CE	FORTALE ZA	HGF	UPP EM SACRA ESTAGIO IV	Enviada	Eventos Adversos Relaciona dos à	Lesão por pressão		
29/12/2020	CE	FORTALE ZA	HGF	UPP EM GLUTEO S ESTAGIO	Enviada	Eventos Adversos Relaciona dos à	Lesão por pressão		
29/12/2020	CE	FORTALE ZA	HGF	UPP EM SACRA ESTAGIO IV	Enviada	Eventos Adversos Relaciona dos à	Lesão por pressão		
29/12/2020	CE	FORTALE ZA	HGF	UPP EM PAVILHÃ O AURICUL	Enviada	Eventos Adversos Relaciona dos à	Lesão por pressão		
29/12/2020	CE	FORTALE ZA	HGF	UPP EM ESTAGIO III EM PAVILHÃ	Enviada	Eventos Adversos Relaciona dos à	Lesão por pressão		

29/12/2020	CE	FORTALE ZA	HGF	UPP EM SACRA III	Enviada	Eventos Adversos Relaciona dos à	Lesão por pressão		
29/12/2020	CE	FORTALE ZA	HGF	UPP EM TROCAN TER ESQUER	Enviada	Eventos Adversos Relaciona dos à	Lesão por pressão		
29/12/2020	CE	FORTALE ZA	HGF	UPP EM GLUTEO ESQUER DO	Enviada	Eventos Adversos Relaciona dos à	Lesão por pressão		
29/12/2020	CE	FORTALE ZA	HGF	UPP EM SACRA IV	Enviada	Eventos Adversos Relaciona dos à	Lesão por pressão		
29/12/2020	CE	FORTALE ZA	HGF	-	Enviada	Eventos Adversos Relaciona dos à	Lesão por pressão		
29/12/2020	CE	FORTALE ZA	HGF	UPP EM OCCCIPI TO ESTAGIO	Enviada	Eventos Adversos Relaciona dos à	Lesão por pressão		
29/12/2020	CE	FORTALE ZA	HGF	UPP EM SACRA ESTAGIO IV	Enviada	Eventos Adversos Relaciona dos à	Lesão por pressão		
29/12/2020	CE	FORTALE ZA	HGF	UPP EM SACRA ESTAGIO III	Enviada	Eventos Adversos Relaciona dos à	Lesão por pressão		
29/12/2020	CE	FORTALE ZA	HGF	UPP EM SACRA ESTAGIO III	Enviada	Eventos Adversos Relaciona dos à	Lesão por pressão		
29/12/2020	CE	FORTALE ZA	HGF	UPP EM GLUTEO DIREITO ESTAGIO	Enviada	Eventos Adversos Relaciona dos à	Lesão por pressão		
29/12/2020	CE	FORTALE ZA	HGF	UPP EM REGIÃO OCCIPTA L III	Enviada	Eventos Adversos Relaciona dos à	Lesão por pressão		
05/01/2021	CE	FORTALE ZA	HGF	As 1:30 acompanh ante relata	Enviada	Eventos Adversos Relaciona dos à	Queda do paciente		
05/01/2021	CE	FORTALE ZA	HGF	Segundo informaçã o colhida, acompanh	Enviada	Eventos Adversos Relaciona dos à	Queda do paciente		
05/01/2021	CE	FORTALE ZA	HGF	UPP EM SACRA IV	Enviada	Eventos Adversos Relaciona dos à	Lesão por pressão		
05/01/2021	CE	FORTALE ZA	HGF	UPP EM TROCAN TER DIREITO	Enviada	Eventos Adversos Relaciona dos à	Lesão por pressão		

05/01/2021	CE	FORTALE ZA	HGF	UPP EM SACRA GRAU III	Enviada	Eventos Adversos Relaciona dos à	Lesão por pressão		
05/01/2021	CE	FORTALE ZA	HGF	UPP EM SACRA III	Enviada	Eventos Adversos Relaciona dos à	Lesão por pressão		
05/01/2021	CE	FORTALE ZA	HGF	UPP EM SACRA ESTAGIO III	Enviada	Eventos Adversos Relaciona dos à	Lesão por pressão		
05/01/2021	CE	FORTALE ZA	HGF	UPP EM OCCIPTO III	Enviada	Eventos Adversos Relaciona dos à	Lesão por pressão		
05/01/2021	CE	FORTALE ZA	HGF	UPP EM INTERGL UTEO III	Enviada	Eventos Adversos Relaciona dos à	Lesão por pressão		
05/01/2021	CE	FORTALE ZA	HGF	UPP EM GLUTEO DIREITO GRAU II	Enviada	Eventos Adversos Relaciona dos à	Lesão por pressão		
05/01/2021	CE	FORTALE ZA	HGF	UPP EM CALCAN EO DIREITO	Enviada	Eventos Adversos Relaciona dos à	Lesão por pressão		
05/01/2021	CE	FORTALE ZA	HGF	UP SACRAL III	Enviada	Eventos Adversos Relaciona dos à	Lesão por pressão		
05/01/2021	CE	FORTALE ZA	HGF	UPP EM SACRA III	Enviada	Eventos Adversos Relaciona dos à	Lesão por pressão		
05/01/2021	CE	FORTALE ZA	HGF	UPP SACRAL III	Enviada	Eventos Adversos Relaciona dos à	Lesão por pressão		
05/01/2021	CE	FORTALE ZA	HGF	UPP EM SACRA IV	Enviada	Eventos Adversos Relaciona dos à	Lesão por pressão		
05/01/2021	CE	FORTALE ZA	HGF	UPP SACRAL III	Enviada	Eventos Adversos Relaciona dos à	Lesão por pressão		
05/01/2021	CE	FORTALE ZA	HGF	UPP EM ORELHA ESTAGIO III	Enviada	Eventos Adversos Relaciona dos à	Lesão por pressão		
05/01/2021	CE	FORTALE ZA	HGF	UPP EM GLUTEO S III	Enviada	Eventos Adversos Relaciona dos à	Lesão por pressão		
05/01/2021	CE	FORTALE ZA	HGF	UPP EM OCCIPTA L III	Enviada	Eventos Adversos Relaciona dos à	Lesão por pressão		

05/01/2021	CE	FORTALEZA	HGF	UPP EM PAVILHÃO AURICUL	Enviada	Eventos Adversos Relacionados à	Lesão por pressão		
06/01/2021	CE	FORTALEZA	HGF	UPP INTRAGLUTEO III	Enviada	Eventos Adversos Relacionados à	Lesão por pressão		
06/01/2021	CE	FORTALEZA	HGF	UPP EM OCCIPTAL III	Enviada	Eventos Adversos Relacionados à	Lesão por pressão		
06/01/2021	CE	FORTALEZA	HGF	UPP EM GLUTEO DIREITO III	Enviada	Eventos Adversos Relacionados à	Lesão por pressão		
06/01/2021	CE	FORTALEZA	HGF	UPP EM GLUTEO III	Enviada	Eventos Adversos Relacionados à	Lesão por pressão		
06/01/2021	CE	FORTALEZA	HGF	UPP EM TROCANTER DIREITO	Enviada	Eventos Adversos Relacionados à	Lesão por pressão		
06/01/2021	CE	FORTALEZA	HGF	UPP SACRAL ESTAGIO III	Enviada	Eventos Adversos Relacionados à	Lesão por pressão		
06/01/2021	CE	FORTALEZA	HGF	UPP EM DORSO III	Enviada	Eventos Adversos Relacionados à	Lesão por pressão		
06/01/2021	CE	FORTALEZA	HGF	UPP EM SACRO ESTAGIO III	Enviada	Eventos Adversos Relacionados à	Lesão por pressão		
06/01/2021	CE	FORTALEZA	HGF	UPP EM TROCANTERES	Enviada	Eventos Adversos Relacionados à	Lesão por pressão		
06/01/2021	CE	FORTALEZA	HGF	UPP SACRAL ESTAGIO III	Enviada	Eventos Adversos Relacionados à	Lesão por pressão		
06/01/2021	CE	FORTALEZA	HGF	UPP EM ISQUIO DIREITO IV	Enviada	Eventos Adversos Relacionados à	Lesão por pressão		
06/01/2021	CE	FORTALEZA	HGF	LPP SACRAL ESTÁGIO III	Enviada	Eventos Adversos Relacionados à	Lesão por pressão		
06/01/2021	CE	FORTALEZA	HGF	LPP MALÉOLO ESQUER	Enviada	Eventos Adversos Relacionados à	Lesão por pressão		
06/01/2021	CE	FORTALEZA	HGF	LPP SACRAL ESTÁGIO IV	Enviada	Eventos Adversos Relacionados à	Lesão por pressão		

06/01/2021	CE	FORTALEZA	HGF	LPP CALCANEO ESTÁGIO	Enviada	Eventos Adversos Relacionados à	Lesão por pressão		
06/01/2021	CE	FORTALEZA	HGF	LPP DORSO ESTÁGIO III	Enviada	Eventos Adversos Relacionados à	Lesão por pressão		
06/01/2021	CE	FORTALEZA	HGF	LPP SACRAL ESTÁGIO III	Enviada	Eventos Adversos Relacionados à	Lesão por pressão		
06/01/2021	CE	FORTALEZA	HGF	LPP CALCANEO III	Enviada	Eventos Adversos Relacionados à	Lesão por pressão		
06/01/2021	CE	FORTALEZA	HGF	LPP SACRAL ESTÁGIO III	Enviada	Eventos Adversos Relacionados à	Lesão por pressão		
11/01/2021	CE	FORTALEZA	HGF	A paciente estava apresenta	Enviada	Eventos Adversos Relacionados à	Queda do paciente		
14/01/2021	CE	FORTALEZA	HGF	paciente caiu no banheiro, escorrego	Enviada	Eventos Adversos Relacionados à	Queda do paciente		
18/01/2021	CE	FORTALEZA	HGF	AS 18:00 PACIENTE SAIU DO	Enviada	Eventos Adversos Relacionados à	Queda do paciente		
18/01/2021	CE	FORTALEZA	HGF	PACIENTE SEM ACOMPANHANTE	Enviada	Eventos Adversos Relacionados à	Queda do paciente		
18/01/2021	CE	FORTALEZA	HGF	PACIENTE FOI AO BANHEIR	Enviada	Eventos Adversos Relacionados à	Queda do paciente		
26/01/2021	CE	FORTALEZA	HGF	Paciente colocado em cadeira	Enviada	Eventos Adversos Relacionados à	Queda do paciente		
28/01/2021	CE	FORTALEZA	HGF	Paciente foi ao banheiro com a	Enviada	Eventos Adversos Relacionados à	Queda do paciente		
03/02/2021	CE	FORTALEZA	HGF	PACIENTE FOI SENTADO EM	Enviada	Eventos Adversos Relacionados à	Queda do paciente		
15/02/2021	CE	FORTALEZA	HGF	ANTIBIOTICOS PRESCRITOS E	Enviada	Eventos Adversos Relacionados à	Falhas durante a assistência à saúde		
15/02/2021	CE	FORTALEZA	HGF	PACIENTE AO TENTAR LEVANTA	Enviada	Eventos Adversos Relacionados à	Queda do paciente		

01/03/2021	CE	FORTALEZA	HGF	PACIENTE ESCORREGOU	Enviada	Eventos Adversos Relacionados à	Queda do paciente		
02/03/2021	CE	FORTALEZA	HGF	LESÃO EM SACRA	Concluída	Eventos Adversos Relacionados à	Lesão por pressão		
02/03/2021	CE	FORTALEZA	HGF	LESÃO EM SACRA	Concluída	Eventos Adversos Relacionados à	Lesão por pressão		
02/03/2021	CE	FORTALEZA	HGF	LESÃO EM LOMBAR	Concluída	Eventos Adversos Relacionados à	Lesão por pressão		
02/03/2021	CE	FORTALEZA	HGF	LESÃO EM SACRA	Concluída	Eventos Adversos Relacionados à	Lesão por pressão		
02/03/2021	CE	FORTALEZA	HGF	LESÃO EM SACRA	Concluída	Eventos Adversos Relacionados à	Lesão por pressão		
02/03/2021	CE	FORTALEZA	HGF	LESÃO EM CALCANEOS	Concluída	Eventos Adversos Relacionados à	Lesão por pressão		
02/03/2021	CE	FORTALEZA	HGF	LESÃO EM SACRA	Enviada	Eventos Adversos Relacionados à	Lesão por pressão		
02/03/2021	CE	FORTALEZA	HGF	LESÃO EM SACRA	Concluída	Eventos Adversos Relacionados à	Lesão por pressão		
02/03/2021	CE	FORTALEZA	HGF	LESÃO EM SACRA	Enviada	Eventos Adversos Relacionados à	Lesão por pressão		
02/03/2021	CE	FORTALEZA	HGF	LESÃO EM SACRA	Concluída	Eventos Adversos Relacionados à	Lesão por pressão		
02/03/2021	CE	FORTALEZA	HGF	LPP EM OCCIPTAL	Concluída	Eventos Adversos Relacionados à	Lesão por pressão		
02/03/2021	CE	FORTALEZA	HGF	LPP EM TROCANTER DIREITO	Concluída	Eventos Adversos Relacionados à	Lesão por pressão		
02/03/2021	CE	FORTALEZA	HGF	LPP EM SACRA	Concluída	Eventos Adversos Relacionados à	Lesão por pressão		
02/03/2021	CE	FORTALEZA	HGF	LPP EM SACRA	Concluída	Eventos Adversos Relacionados à	Lesão por pressão		

02/03/2021	CE	FORTALEZA	HGF	LPP EM INTERGLUTEO	Concluída	Eventos Adversos Relacionados à	Lesão por pressão		
02/03/2021	CE	FORTALEZA	HGF	LPP EM SACRA	Enviada	Eventos Adversos Relacionados à	Lesão por pressão		
02/03/2021	CE	FORTALEZA	HGF	LPP EM SACRA	Enviada	Eventos Adversos Relacionados à	Lesão por pressão		
02/03/2021	CE	FORTALEZA	HGF	LPP EM GLUTEOS	Enviada	Eventos Adversos Relacionados à	Lesão por pressão		
02/03/2021	CE	FORTALEZA	HGF	LPP EM SACRA	Concluída	Eventos Adversos Relacionados à	Lesão por pressão		
02/03/2021	CE	FORTALEZA	HGF	LPP EM CALCANEO ESQUER	Concluída	Eventos Adversos Relacionados à	Lesão por pressão		
02/03/2021	CE	FORTALEZA	HGF	LPP EM TROCANTER DIREITO	Concluída	Eventos Adversos Relacionados à	Lesão por pressão		
02/03/2021	CE	FORTALEZA	HGF	LPP EM SACRA	Concluída	Eventos Adversos Relacionados à	Lesão por pressão		
02/03/2021	CE	FORTALEZA	HGF	LPP EM SACRA	Enviada	Eventos Adversos Relacionados à	Lesão por pressão		
02/03/2021	CE	FORTALEZA	HGF	LPP EM GLUTEO DIREITO	Concluída	Eventos Adversos Relacionados à	Lesão por pressão		
03/03/2021	CE	FORTALEZA	HGF	LPP EM SACRA	Concluída	Eventos Adversos Relacionados à	Lesão por pressão		
03/03/2021	CE	FORTALEZA	HGF	LPP EM SACRA	Enviada	Eventos Adversos Relacionados à	Lesão por pressão		
03/03/2021	CE	FORTALEZA	HGF	LPP EM SACRA	Concluída	Eventos Adversos Relacionados à	Lesão por pressão		
03/03/2021	CE	FORTALEZA	HGF	LPP EM SACRA	Concluída	Eventos Adversos Relacionados à	Lesão por pressão		
03/03/2021	CE	FORTALEZA	HGF	LPP EM SACRA	Concluída	Eventos Adversos Relacionados à	Lesão por pressão		

03/03/2021	CE	FORTALEZA	HGF	LPP EM SACRA	Concluída	Eventos Adversos Relacionados à	Lesão por pressão		
03/03/2021	CE	FORTALEZA	HGF	LPP EM SACRA	Concluída	Eventos Adversos Relacionados à	Lesão por pressão		
03/03/2021	CE	FORTALEZA	HGF	LPP EM TROCANTER DIREITO	Concluída	Eventos Adversos Relacionados à	Lesão por pressão		
03/03/2021	CE	FORTALEZA	HGF	LPP EM MALEOLOS	Concluída	Eventos Adversos Relacionados à	Lesão por pressão		
03/03/2021	CE	FORTALEZA	HGF	LPP EM JOELHO DIREITO	Concluída	Eventos Adversos Relacionados à	Lesão por pressão		
03/03/2021	CE	FORTALEZA	HGF	LPP EM JOELHO ESQUERDO	Concluída	Eventos Adversos Relacionados à	Lesão por pressão		
03/03/2021	CE	FORTALEZA	HGF	LPP EM SACRA	Concluída	Eventos Adversos Relacionados à	Lesão por pressão		
03/03/2021	CE	FORTALEZA	HGF	LPP EM ISQUIO DIREITO	Concluída	Eventos Adversos Relacionados à	Lesão por pressão		
03/03/2021	CE	FORTALEZA	HGF	LPP EM GLUTEO DIREITO	Concluída	Eventos Adversos Relacionados à	Lesão por pressão		
03/03/2021	CE	FORTALEZA	HGF	LPP EM GLUTEO ESQUERDO	Concluída	Eventos Adversos Relacionados à	Lesão por pressão		
03/03/2021	CE	FORTALEZA	HGF	LPP EM ISQUIO ESQUERDO	Concluída	Eventos Adversos Relacionados à	Lesão por pressão		
03/03/2021	CE	FORTALEZA	HGF	LPP EM SACRA	Concluída	Eventos Adversos Relacionados à	Lesão por pressão		
03/03/2021	CE	FORTALEZA	HGF	LPP EM SACRA	Concluída	Eventos Adversos Relacionados à	Lesão por pressão		
03/03/2021	CE	FORTALEZA	HGF	LPP EM SACRA	Concluída	Eventos Adversos Relacionados à	Lesão por pressão		
03/03/2021	CE	FORTALEZA	HGF	LPP EM GLUTEO ESQUERDO	Concluída	Eventos Adversos Relacionados à	Lesão por pressão		

03/03/2021	CE	FORTALEZA	HGF	LPP EM TROCANTERES	Concluída	Eventos Adversos Relacionados à	Lesão por pressão		
03/03/2021	CE	FORTALEZA	HGF	LPP EM SACRA	Concluída	Eventos Adversos Relacionados à	Lesão por pressão		
03/03/2021	CE	FORTALEZA	HGF	LPP EM CALCANEO ESQUER	Concluída	Eventos Adversos Relacionados à	Lesão por pressão		
03/03/2021	CE	FORTALEZA	HGF	LPP EM SACRA	Concluída	Eventos Adversos Relacionados à	Lesão por pressão		
29/03/2021	CE	FORTALEZA	HGF	PACIENTE CITADO NÃO	Enviada	Eventos Adversos Relacionados à	Queda do paciente		
29/03/2021	CE	FORTALEZA	HGF	PACIENTE CITADO NÃO	Enviada	Eventos Adversos Relacionados à	Queda do paciente		
29/03/2021	CE	FORTALEZA	HGF	POR VOLTA DE 12:40 O	Enviada	Eventos Adversos Relacionados à	Queda do paciente		
29/03/2021	CE	FORTALEZA	HGF	PACIENTE INTERNADA	Enviada	Eventos Adversos Relacionados à	Falhas durante a assistência à saúde		
29/03/2021	CE	FORTALEZA	HGF	PACIENTE TRANSPORTADO	Enviada	Eventos Adversos Relacionados à	Falhas nas atividades		
01/04/2021	CE	FORTALEZA	HGF	Acompanhante ofereceu suco para	Enviada	Eventos Adversos Relacionados à	Broncoaspiração		
01/04/2021	CE	FORTALEZA	HGF	Técnica administra antibiótico	Enviada	Eventos Adversos Relacionados à	Falhas durante a assistência à saúde		
12/04/2021	CE	FORTALEZA	HGF	PACIENTE PERDEU EQUILIB	Enviada	Eventos Adversos Relacionados à	Queda do paciente		
23/04/2021	CE	FORTALEZA	HGF	LESÃO EM GLÚTEO ESQUER	Concluída	Eventos Adversos Relacionados à	Lesão por pressão		
23/04/2021	CE	FORTALEZA	HGF	LESÃO EM SACRA	Concluída	Eventos Adversos Relacionados à	Lesão por pressão		
23/04/2021	CE	FORTALEZA	HGF	LESÃO EM CALCÂNEOS	Concluída	Eventos Adversos Relacionados à	Lesão por pressão		

23/04/2021	CE	FORTALEZA	HGF	LESÃO EM ESCAPULA	Concluída	Eventos Adversos Relacionados à	Lesão por pressão		
23/04/2021	CE	FORTALEZA	HGF	LESÃO EM GLUTEO DIREITO	Concluída	Eventos Adversos Relacionados à	Lesão por pressão		
23/04/2021	CE	FORTALEZA	HGF	LESÃO EM SACRA	Concluída	Eventos Adversos Relacionados à	Lesão por pressão		
23/04/2021	CE	FORTALEZA	HGF	LESÃO EM INTERGLÚTEO	Concluída	Eventos Adversos Relacionados à	Lesão por pressão		
23/04/2021	CE	FORTALEZA	HGF	LESÃO EM SACRA	Concluída	Eventos Adversos Relacionados à	Lesão por pressão		
23/04/2021	CE	FORTALEZA	HGF	LESÃO EM SACRA	Concluída	Eventos Adversos Relacionados à	Lesão por pressão		
23/04/2021	CE	FORTALEZA	HGF	LESÃO EM SACRA	Concluída	Eventos Adversos Relacionados à	Lesão por pressão		
23/04/2021	CE	FORTALEZA	HGF	LESÃO EM SACRA	Concluída	Eventos Adversos Relacionados à	Lesão por pressão		
23/04/2021	CE	FORTALEZA	HGF	LESÃO EM SACRA	Concluída	Eventos Adversos Relacionados à	Lesão por pressão		
23/04/2021	CE	FORTALEZA	HGF	LESÃO EM INTERGLÚTEO	Concluída	Eventos Adversos Relacionados à	Lesão por pressão		
23/04/2021	CE	FORTALEZA	HGF	LESÃO EM ÍSQUIO DIREITO	Concluída	Eventos Adversos Relacionados à	Lesão por pressão		
23/04/2021	CE	FORTALEZA	HGF	LESÃO EM GLÚTEOS	Concluída	Eventos Adversos Relacionados à	Lesão por pressão		
23/04/2021	CE	FORTALEZA	HGF	LESÃO EM SACRA	Concluída	Eventos Adversos Relacionados à	Lesão por pressão		
23/04/2021	CE	FORTALEZA	HGF	LESÃO EM SACRA	Concluída	Eventos Adversos Relacionados à	Lesão por pressão		
23/04/2021	CE	FORTALEZA	HGF	LESÃO EM SACRA	Concluída	Eventos Adversos Relacionados à	Lesão por pressão		

23/04/2021	CE	FORTALEZA	HGF	LESÃO EM OCCIPTAL	Concluída	Eventos Adversos Relacionados à	Lesão por pressão		
23/04/2021	CE	FORTALEZA	HGF	LESÃO EM SACRA	Concluída	Eventos Adversos Relacionados à	Lesão por pressão		
23/04/2021	CE	FORTALEZA	HGF	LESÃO EM SACRA	Concluída	Eventos Adversos Relacionados à	Lesão por pressão		
23/04/2021	CE	FORTALEZA	HGF	LESÃO EM GLÚTEO DIREITO	Concluída	Eventos Adversos Relacionados à	Lesão por pressão		
23/04/2021	CE	FORTALEZA	HGF	LESÃO EM CALCÂNEOS	Concluída	Eventos Adversos Relacionados à	Lesão por pressão		
23/04/2021	CE	FORTALEZA	HGF	LESÃO EM SACRA	Concluída	Eventos Adversos Relacionados à	Lesão por pressão		
23/04/2021	CE	FORTALEZA	HGF	LESÃO EM SACRA	Concluída	Eventos Adversos Relacionados à	Lesão por pressão		
23/04/2021	CE	FORTALEZA	HGF	LESÃO EM GLÚTEOS	Concluída	Eventos Adversos Relacionados à	Lesão por pressão		
23/04/2021	CE	FORTALEZA	HGF	LESÃO EM SACRA	Concluída	Eventos Adversos Relacionados à	Lesão por pressão		
23/04/2021	CE	FORTALEZA	HGF	LESÃO EM GLÚTEOS	Concluída	Eventos Adversos Relacionados à	Lesão por pressão		
23/04/2021	CE	FORTALEZA	HGF	LESÃO EM SACRA	Concluída	Eventos Adversos Relacionados à	Lesão por pressão		
23/04/2021	CE	FORTALEZA	HGF	LESÃO EM CALCÂNEOS	Concluída	Eventos Adversos Relacionados à	Lesão por pressão		
23/04/2021	CE	FORTALEZA	HGF	LESÃO EM SACRA	Concluída	Eventos Adversos Relacionados à	Lesão por pressão		
23/04/2021	CE	FORTALEZA	HGF	LESÃO EM GLÚTEOS	Concluída	Eventos Adversos Relacionados à	Lesão por pressão		
23/04/2021	CE	FORTALEZA	HGF	LESÃO EM CALCÂNEOS	Concluída	Eventos Adversos Relacionados à	Lesão por pressão		

23/04/2021	CE	FORTALEZA	HGF	LESÃO EM SACRA	Concluída	Eventos Adversos Relacionados à	Lesão por pressão		
23/04/2021	CE	FORTALEZA	HGF	LESÃO EM SACRA	Concluída	Eventos Adversos Relacionados à	Lesão por pressão		
23/04/2021	CE	FORTALEZA	HGF	LESÃO EM ÍSQUIOS	Concluída	Eventos Adversos Relacionados à	Lesão por pressão		
23/04/2021	CE	FORTALEZA	HGF	LESÃO EM SACRA	Concluída	Eventos Adversos Relacionados à	Lesão por pressão		
23/04/2021	CE	FORTALEZA	HGF	LESÃO EM MALÉOLOS	Concluída	Eventos Adversos Relacionados à	Lesão por pressão		
23/04/2021	CE	FORTALEZA	HGF	LESÃO EM SACRA	Concluída	Eventos Adversos Relacionados à	Lesão por pressão		
23/04/2021	CE	FORTALEZA	HGF	LESÃO EM SACRA	Concluída	Eventos Adversos Relacionados à	Lesão por pressão		
23/04/2021	CE	FORTALEZA	HGF	LESÃO EM GLÚTEO ESQUER	Concluída	Eventos Adversos Relacionados à	Lesão por pressão		
23/04/2021	CE	FORTALEZA	HGF	LESÃO EM TROCANTER	Enviada	Eventos Adversos Relacionados à	Lesão por pressão		
23/04/2021	CE	FORTALEZA	HGF	LESÃO EM GLÚTEOS	Concluída	Eventos Adversos Relacionados à	Lesão por pressão		
23/04/2021	CE	FORTALEZA	HGF	LESÃO RETROAURICULAR	Concluída	Eventos Adversos Relacionados à	Lesão por pressão		
23/04/2021	CE	FORTALEZA	HGF	LESÃO EM CALCÂNEO	Concluída	Eventos Adversos Relacionados à	Lesão por pressão		
23/04/2021	CE	FORTALEZA	HGF	LESÃO EM SACRA	Concluída	Eventos Adversos Relacionados à	Lesão por pressão		
23/04/2021	CE	FORTALEZA	HGF	LESÃO EM SACRA	Concluída	Eventos Adversos Relacionados à	Lesão por pressão		
23/04/2021	CE	FORTALEZA	HGF	LESÃO EM GLÚTEOS	Concluída	Eventos Adversos Relacionados à	Lesão por pressão		

23/04/2021	CE	FORTALEZA	HGF	LESÃO EM TROCANTERES	Enviada	Eventos Adversos Relacionados à	Lesão por pressão		
23/04/2021	CE	FORTALEZA	HGF	LESÃO EM SACRA	Concluída	Eventos Adversos Relacionados à	Lesão por pressão		
23/04/2021	CE	FORTALEZA	HGF	LESÃO EM SACRA	Concluída	Eventos Adversos Relacionados à	Lesão por pressão		
23/04/2021	CE	FORTALEZA	HGF	LESÃO EM SACRA	Concluída	Eventos Adversos Relacionados à	Lesão por pressão		
23/04/2021	CE	FORTALEZA	HGF	LESÃO EM ISQUIOS	Concluída	Eventos Adversos Relacionados à	Lesão por pressão		
23/04/2021	CE	FORTALEZA	HGF	LESÃO EM SACRA	Concluída	Eventos Adversos Relacionados à	Lesão por pressão		
23/04/2021	CE	FORTALEZA	HGF	LESÃO EM OCCIPTAL	Concluída	Eventos Adversos Relacionados à	Lesão por pressão		
23/04/2021	CE	FORTALEZA	HGF	LESÃO EM TROCANTER	Concluída	Eventos Adversos Relacionados à	Lesão por pressão		
23/04/2021	CE	FORTALEZA	HGF	LESÃO EM CALCÂNEO	Concluída	Eventos Adversos Relacionados à	Lesão por pressão		
23/04/2021	CE	FORTALEZA	HGF	LESÃO EM SACRA	Concluída	Eventos Adversos Relacionados à	Lesão por pressão		
23/04/2021	CE	FORTALEZA	HGF	LESÃO EM CALCÂNEOS	Concluída	Eventos Adversos Relacionados à	Lesão por pressão		
23/04/2021	CE	FORTALEZA	HGF	LESÃO EM SACRA	Concluída	Eventos Adversos Relacionados à	Lesão por pressão		
23/04/2021	CE	FORTALEZA	HGF	LESÃO EM ORELHA	Concluída	Eventos Adversos Relacionados à	Lesão por pressão		
23/04/2021	CE	FORTALEZA	HGF	LESÃO EM SACRA	Concluída	Eventos Adversos Relacionados à	Lesão por pressão		
23/04/2021	CE	FORTALEZA	HGF	LESÃO EM SACRA	Concluída	Eventos Adversos Relacionados à	Lesão por pressão		

23/04/2021	CE	FORTALE ZA	HGF	LESÃO EM GLÚTEO S	Concluída	Eventos Adversos Relaciona dos à	Lesão por pressão		
23/04/2021	CE	FORTALE ZA	HGF	LESÃO EM SACRA	Concluída	Eventos Adversos Relaciona dos à	Lesão por pressão		
23/04/2021	CE	FORTALE ZA	HGF	LESÃO EM SACRA	Concluída	Eventos Adversos Relaciona dos à	Lesão por pressão		
23/04/2021	CE	FORTALE ZA	HGF	LESÃO EM GLÚTEO DIREITO	Concluída	Eventos Adversos Relaciona dos à	Lesão por pressão		
23/04/2021	CE	FORTALE ZA	HGF	LESÃO EM CALCÂN EOS	Concluída	Eventos Adversos Relaciona dos à	Lesão por pressão		
23/04/2021	CE	FORTALE ZA	HGF	LESÃO EM SACRA	Concluída	Eventos Adversos Relaciona dos à	Lesão por pressão		
23/04/2021	CE	FORTALE ZA	HGF	LESÃO EM SACRA	Concluída	Eventos Adversos Relaciona dos à	Lesão por pressão		
23/04/2021	CE	FORTALE ZA	HGF	LESÃO EM SACRA	Concluída	Eventos Adversos Relaciona dos à	Lesão por pressão		
23/04/2021	CE	FORTALE ZA	HGF	LESÃO EM SACRA	Concluída	Eventos Adversos Relaciona dos à	Lesão por pressão		
27/04/2021	CE	FORTALE ZA	HGF	foi administra do na referida	Enviada	Eventos Adversos Relaciona dos à	Falhas durante a assistênci a à saúde		
27/04/2021	CE	FORTALE ZA	HGF	paciente ao tentar lavantar sozinho	Enviada	Eventos Adversos Relaciona dos à	Queda do paciente		
27/04/2021	CE	FORTALE ZA	HGF	POR VOLTA DAS 24HS	Enviada	Eventos Adversos Relaciona dos à	Queda do paciente		
27/04/2021	CE	FORTALE ZA	HGF	PACIENT E REFERIA PRURIDO	Enviada	Eventos Adversos Relaciona dos à	Queda do paciente		
12/05/2021	CE	FORTALE ZA	HGF	ADMINIS TROU MEDICA MENTO	Enviada	Eventos Adversos Relaciona dos à	Falhas durante a assistênci a à saúde		
12/05/2021	CE	FORTALE ZA	HGF	Paciente desorienta do tentou descer	Enviada	Eventos Adversos Relaciona dos à	Queda do paciente		

14/05/2021	CE	FORTALEZA	HGF	PACIENTE CRÍTICO COM US	Enviada	Eventos Adversos Relacionados à	Falhas durante a assistência à saúde		
14/05/2021	CE	FORTALEZA	HGF	MEDICAÇÃO (ACICLOVIR	Enviada	Eventos Adversos Relacionados à	Falhas durante a assistência à saúde		
17/05/2021	CE	FORTALEZA	HGF	IDENTIFICADO PRESENCA DE	Enviada	Eventos Adversos Relacionados à	Falhas durante a assistência à saúde		
19/05/2021	CE	FORTALEZA	HGF	Ao banhar paciente é	Enviada	Eventos Adversos Relacionados à	Lesão por pressão		
25/05/2021	CE	FORTALEZA	HGF	PACIENTE ADMITIDA SEM	Enviada	Eventos Adversos Relacionados à	Falhas durante a assistência à saúde		
25/05/2021	CE	FORTALEZA	HGF	PACIENTE RENAL CRÔNICO COM	Enviada	Eventos Adversos Relacionados à	Falhas durante a assistência à saúde		
25/05/2021	CE	FORTALEZA	HGF	PACIENTE COM ALTERAÇÃO DE	Enviada	Eventos Adversos Relacionados à	Falhas durante a assistência à saúde		
25/05/2021	CE	FORTALEZA	HGF	PACIENTE INICIA DISFUNÇÃO	Enviada	Eventos Adversos Relacionados à	Falhas durante a assistência à saúde		
28/05/2021	CE	FORTALEZA	HGF	PACIENTE AGITADA	Enviada	Eventos Adversos Relacionados à	Queda do paciente		
31/05/2021	CE	FORTALEZA	HGF	Queda da paciente do leito que	Enviada	Eventos Adversos Relacionados à	Queda do paciente		
31/05/2021	CE	FORTALEZA	HGF	no momento do banho	Enviada	Eventos Adversos Relacionados à	Queda do paciente		
04/06/2021	CE	FORTALEZA	HGF	PACIENTE, AGITADA E EM	Enviada	Eventos Adversos Relacionados à	Queda do paciente		
09/06/2021	CE	FORTALEZA	HGF	PACIENTE NÃO AGUARDOU	Enviada	Eventos Adversos Relacionados à	Queda do paciente		
15/06/2021	CE	FORTALEZA	HGF	LPP EM REGIÃO SACRA	Concluída	Eventos Adversos Relacionados à	Lesão por pressão		
15/06/2021	CE	FORTALEZA	HGF	LPP EM REGIÃO SACRA	Concluída	Eventos Adversos Relacionados à	Lesão por pressão		

15/06/2021	CE	FORTALEZA	HGF	LPP EM REGIÃO SACRA	Concluída	Eventos Adversos Relacionados à	Lesão por pressão		
15/06/2021	CE	FORTALEZA	HGF	LPP EM REGIÃO SACRA	Concluída	Eventos Adversos Relacionados à	Lesão por pressão		
15/06/2021	CE	FORTALEZA	HGF	LPP EM REGIÃO SACRA	Concluída	Eventos Adversos Relacionados à	Lesão por pressão		
15/06/2021	CE	FORTALEZA	HGF	LPP EM REGIÃO SACRA	Concluída	Eventos Adversos Relacionados à	Lesão por pressão		
15/06/2021	CE	FORTALEZA	HGF	LPP EM TROCANTERES	Concluída	Eventos Adversos Relacionados à	Lesão por pressão		
15/06/2021	CE	FORTALEZA	HGF	LPP EM ISQUIOS	Concluída	Eventos Adversos Relacionados à	Lesão por pressão		
15/06/2021	CE	FORTALEZA	HGF	LPP EM MALEOLO ESQUER	Concluída	Eventos Adversos Relacionados à	Lesão por pressão		
15/06/2021	CE	FORTALEZA	HGF	LPP EM REGIÃO SACRA	Concluída	Eventos Adversos Relacionados à	Lesão por pressão		
15/06/2021	CE	FORTALEZA	HGF	LPP EM REGIÃO SACRA	Concluída	Eventos Adversos Relacionados à	Lesão por pressão		
15/06/2021	CE	FORTALEZA	HGF	LPP EM GLUTEO DIREITO	Concluída	Eventos Adversos Relacionados à	Lesão por pressão		
15/06/2021	CE	FORTALEZA	HGF	LPP EM REGIÃO SACRA	Concluída	Eventos Adversos Relacionados à	Lesão por pressão		
15/06/2021	CE	FORTALEZA	HGF	LPP EM GLUTEOS	Concluída	Eventos Adversos Relacionados à	Lesão por pressão		
15/06/2021	CE	FORTALEZA	HGF	LPP EM TROCANTER DIREITO	Concluída	Eventos Adversos Relacionados à	Lesão por pressão		
15/06/2021	CE	FORTALEZA	HGF	LPP EM CALCANEO ESQUER	Concluída	Eventos Adversos Relacionados à	Lesão por pressão		
15/06/2021	CE	FORTALEZA	HGF	LPP EM REGIÃO SACRA	Concluída	Eventos Adversos Relacionados à	Lesão por pressão		

15/06/2021	CE	FORTALEZA	HGF	LPP EM REGIÃO SACRA	Concluída	Eventos Adversos Relacionados à	Lesão por pressão		
15/06/2021	CE	FORTALEZA	HGF	LPP EM REGIÃO SACRA	Concluída	Eventos Adversos Relacionados à	Lesão por pressão		
15/06/2021	CE	FORTALEZA	HGF	LPP EM REGIÃO SACRA	Concluída	Eventos Adversos Relacionados à	Lesão por pressão		
15/06/2021	CE	FORTALEZA	HGF	LPP EM CALCANEO ESQUER	Concluída	Eventos Adversos Relacionados à	Lesão por pressão		
15/06/2021	CE	FORTALEZA	HGF	LPP EM MALEOLO DIREITO	Concluída	Eventos Adversos Relacionados à	Lesão por pressão		
15/06/2021	CE	FORTALEZA	HGF	LPP EM GLUTEOS	Concluída	Eventos Adversos Relacionados à	Lesão por pressão		
15/06/2021	CE	FORTALEZA	HGF	LPP EM REGIÃO SACRA	Concluída	Eventos Adversos Relacionados à	Lesão por pressão		
15/06/2021	CE	FORTALEZA	HGF	LPP EM REGIÃO SACRA	Concluída	Eventos Adversos Relacionados à	Lesão por pressão		
15/06/2021	CE	FORTALEZA	HGF	LPP EM REGIÃO SACRA	Concluída	Eventos Adversos Relacionados à	Lesão por pressão		
15/06/2021	CE	FORTALEZA	HGF	LPP EM REGIÃO SACRA	Concluída	Eventos Adversos Relacionados à	Lesão por pressão		
15/06/2021	CE	FORTALEZA	HGF	LPP EM REGIÃO SACRA	Concluída	Eventos Adversos Relacionados à	Lesão por pressão		
15/06/2021	CE	FORTALEZA	HGF	LPP EM REGIÃO SACRA	Concluída	Eventos Adversos Relacionados à	Lesão por pressão		
15/06/2021	CE	FORTALEZA	HGF	LPP EM JOELHOS	Concluída	Eventos Adversos Relacionados à	Lesão por pressão		
15/06/2021	CE	FORTALEZA	HGF	LPP EM REGIÃO SACRA	Concluída	Eventos Adversos Relacionados à	Lesão por pressão		
15/06/2021	CE	FORTALEZA	HGF	LPP EM GLUTEOS	Concluída	Eventos Adversos Relacionados à	Lesão por pressão		

15/06/2021	CE	FORTALEZA	HGF	LPP EM PAVILHÃO AURICUL	Concluída	Eventos Adversos Relacionados à	Lesão por pressão		
15/06/2021	CE	FORTALEZA	HGF	LPP EM PANTURRILHAS	Concluída	Eventos Adversos Relacionados à	Lesão por pressão		
15/06/2021	CE	FORTALEZA	HGF	LPP EM GLUTEOS	Concluída	Eventos Adversos Relacionados à	Lesão por pressão		
15/06/2021	CE	FORTALEZA	HGF	LPP EM CALCANEO ESQUER	Concluída	Eventos Adversos Relacionados à	Lesão por pressão		
15/06/2021	CE	FORTALEZA	HGF	LPP EM INTERGLUTEO	Concluída	Eventos Adversos Relacionados à	Lesão por pressão		
15/06/2021	CE	FORTALEZA	HGF	LPP EM REGIÃO SACRA	Concluída	Eventos Adversos Relacionados à	Lesão por pressão		
15/06/2021	CE	FORTALEZA	HGF	LPP EM REGIÃO SACRA	Concluída	Eventos Adversos Relacionados à	Lesão por pressão		
15/06/2021	CE	FORTALEZA	HGF	LPP EM REGIÃO SACRA	Concluída	Eventos Adversos Relacionados à	Lesão por pressão		
15/06/2021	CE	FORTALEZA	HGF	LPP EM REGIÃO SACRA	Concluída	Eventos Adversos Relacionados à	Lesão por pressão		
15/06/2021	CE	FORTALEZA	HGF	LPP EM REGIÃO SACRA	Concluída	Eventos Adversos Relacionados à	Lesão por pressão		
15/06/2021	CE	FORTALEZA	HGF	LPP EM REGIÃO SACRA	Concluída	Eventos Adversos Relacionados à	Lesão por pressão		
15/06/2021	CE	FORTALEZA	HGF	LPP EM REGIÃO SACRA	Concluída	Eventos Adversos Relacionados à	Lesão por pressão		
15/06/2021	CE	FORTALEZA	HGF	LPP EM REGIÃO SACRA	Concluída	Eventos Adversos Relacionados à	Lesão por pressão		
15/06/2021	CE	FORTALEZA	HGF	LPP EM REGIÃO SACRA	Concluída	Eventos Adversos Relacionados à	Lesão por pressão		
15/06/2021	CE	FORTALEZA	HGF	LPP EM REGIÃO SACRA	Concluída	Eventos Adversos Relacionados à	Lesão por pressão		

15/06/2021	CE	FORTALEZA	HGF	LPP EM GLUTEOS	Concluída	Eventos Adversos Relacionados à	Lesão por pressão		
15/06/2021	CE	FORTALEZA	HGF	LPP EM REGIÃO SACRA	Concluída	Eventos Adversos Relacionados à	Lesão por pressão		
15/06/2021	CE	FORTALEZA	HGF	LPP EM REGIÃO SACRA	Concluída	Eventos Adversos Relacionados à	Lesão por pressão		
15/06/2021	CE	FORTALEZA	HGF	LPP EM REGIÃO SACRA	Concluída	Eventos Adversos Relacionados à	Lesão por pressão		
15/06/2021	CE	FORTALEZA	HGF	LPP EM REGIÃO SACRA	Concluída	Eventos Adversos Relacionados à	Lesão por pressão		
15/06/2021	CE	FORTALEZA	HGF	LPP EM MALEOLO DIREITO	Concluída	Eventos Adversos Relacionados à	Lesão por pressão		
15/06/2021	CE	FORTALEZA	HGF	LPP EM ORELHA ESQUERDA	Concluída	Eventos Adversos Relacionados à	Lesão por pressão		
15/06/2021	CE	FORTALEZA	HGF	LPP EM REGIÃO SACRA	Concluída	Eventos Adversos Relacionados à	Lesão por pressão		
15/06/2021	CE	FORTALEZA	HGF	LPP EM REGIÃO SACRA	Concluída	Eventos Adversos Relacionados à	Lesão por pressão		
15/06/2021	CE	FORTALEZA	HGF	LPP EM GLUTEOS	Concluída	Eventos Adversos Relacionados à	Lesão por pressão		
17/06/2021	CE	FORTALEZA	HGF	ADMINISTRADO VANCOMICINA 1G	Enviada	Eventos Adversos Relacionados à	Falhas durante a assistência à saúde		
17/06/2021	CE	FORTALEZA	HGF	AO TENTAR LEVANTAR DO	Enviada	Eventos Adversos Relacionados à	Queda do paciente		
18/06/2021	CE	FORTALEZA	HGF	LPP em região sacra	Concluída	Eventos Adversos Relacionados à	Lesão por pressão		
18/06/2021	CE	FORTALEZA	HGF	LPP em calcâneo esquerdo	Concluída	Eventos Adversos Relacionados à	Lesão por pressão		
18/06/2021	CE	FORTALEZA	HGF	ADMINISTRADO MANITOL V.O	Enviada	Eventos Adversos Relacionados à	Falhas durante a assistência à saúde		

18/06/2021	CE	FORTALEZA	HGF	PACIENTE LEVANTOU DA	Enviada	Eventos Adversos Relacionados à	Queda do paciente		
23/06/2021	CE	FORTALEZA	HGF	LPP EM ESTÁGIO 3	Concluída	Eventos Adversos Relacionados à	Lesão por pressão		
23/06/2021	CE	FORTALEZA	HGF	PACIENTE APRESENTOU	Enviada	Eventos Adversos Relacionados à	Queda do paciente		
23/06/2021	CE	FORTALEZA	HGF	LPP	Concluída	Eventos Adversos Relacionados à	Lesão por pressão		
28/06/2021	CE	FORTALEZA	HGF	ADMINISTRADO CIPROFLOXACINO	Enviada	Eventos Adversos Relacionados à	Falhas durante a assistência à saúde		
28/06/2021	CE	FORTALEZA	HGF	NÃO ADMINISTRADO ATB	Enviada	Eventos Adversos Relacionados à	Falhas durante a assistência à saúde		
05/07/2021	CE	FORTALEZA	HGF	Foram administrados por sonda	Enviada	Eventos Adversos Relacionados à	Falhas durante a assistência à saúde		
08/07/2021	CE	FORTALEZA	HGF	Lesão por pressão estágio 3	Concluída	Eventos Adversos Relacionados à	Lesão por pressão		
08/07/2021	CE	FORTALEZA	HGF	LPP ESTAGIO 3 EM REGIÃO	Concluída	Eventos Adversos Relacionados à	Lesão por pressão		
08/07/2021	CE	FORTALEZA	HGF	LPP ESTÁGIO 3 EM ORELHA	Concluída	Eventos Adversos Relacionados à	Lesão por pressão		
08/07/2021	CE	FORTALEZA	HGF	Lesão por pressão em sacra	Concluída	Eventos Adversos Relacionados à	Lesão por pressão		
08/07/2021	CE	FORTALEZA	HGF	Lesão por pressão em	Concluída	Eventos Adversos Relacionados à	Lesão por pressão		
08/07/2021	CE	FORTALEZA	HGF	Paciente estava anteriormente com	Enviada	Eventos Adversos Relacionados à	Falhas durante a assistência à saúde		
08/07/2021	CE	FORTALEZA	HGF	LPP ESTÁGIO 4 EM REGIÃO	Concluída	Eventos Adversos Relacionados à	Lesão por pressão		
09/07/2021	CE	FORTALEZA	HGF	Lesão por pressão em	Concluída	Eventos Adversos Relacionados à	Lesão por pressão		

09/07/2021	CE	FORTALEZA	HGF	UPP ESTÁGIO 3 EM CALCÂN	Concluída	Eventos Adversos Relacionados à	Lesão por pressão		
12/07/2021	CE	FORTALEZA	HGF	LPP ESTÁGIO 3 EM REGIÃO	Concluída	Eventos Adversos Relacionados à	Lesão por pressão		
12/07/2021	CE	FORTALEZA	HGF	LPP ESTÁGIO 3 EM REGIÃO	Concluída	Eventos Adversos Relacionados à	Lesão por pressão		
12/07/2021	CE	FORTALEZA	HGF	LPP ESTÁGIO 3 EM REGIÃO	Concluída	Eventos Adversos Relacionados à	Lesão por pressão		
12/07/2021	CE	FORTALEZA	HGF	-	Concluída	Eventos Adversos Relacionados à	Lesão por pressão		
12/07/2021	CE	FORTALEZA	HGF	UPP EM SACRA ESTÁGIO 3	Concluída	Eventos Adversos Relacionados à	Lesão por pressão		
12/07/2021	CE	FORTALEZA	HGF	LPP ESTÁGIO 3 EM REGIÃO	Concluída	Eventos Adversos Relacionados à	Lesão por pressão		
12/07/2021	CE	FORTALEZA	HGF	LPP ESTÁGIO 4 EM REGIÃO	Concluída	Eventos Adversos Relacionados à	Lesão por pressão		
13/07/2021	CE	FORTALEZA	HGF	LPP ESTÁGIO 3 EM GLUTEO	Concluída	Eventos Adversos Relacionados à	Lesão por pressão		
13/07/2021	CE	FORTALEZA	HGF	LPP ESTÁGIO 4 (SACRA	Concluída	Eventos Adversos Relacionados à	Lesão por pressão		
13/07/2021	CE	FORTALEZA	HGF	Lesão por pressão em	Concluída	Eventos Adversos Relacionados à	Lesão por pressão		
13/07/2021	CE	FORTALEZA	HGF	Lesão por pressão em	Concluída	Eventos Adversos Relacionados à	Lesão por pressão		
13/07/2021	CE	FORTALEZA	HGF	LPP ESTÁGIO 4 EM REGIÃO	Concluída	Eventos Adversos Relacionados à	Lesão por pressão		
13/07/2021	CE	FORTALEZA	HGF	UPP ESTAGIO 3 EM PANTUR	Concluída	Eventos Adversos Relacionados à	Lesão por pressão		
13/07/2021	CE	FORTALEZA	HGF	UPP E SACRA ESTAGIO 4	Concluída	Eventos Adversos Relacionados à	Lesão por pressão		

13/07/2021	CE	FORTALE ZA	HGF	UPP ESTAGIO 4 EM SACRA	Concluída	Eventos Adversos Relaciona dos à	Lesão por pressão		
13/07/2021	CE	FORTALE ZA	HGF	esão por pressão Estágio 3 em	Concluída	Eventos Adversos Relaciona dos à	Lesão por pressão		
13/07/2021	CE	FORTALE ZA	HGF	LPP ESTÁGIO 3 EM REGIÃO	Concluída	Eventos Adversos Relaciona dos à	Lesão por pressão		
13/07/2021	CE	FORTALE ZA	HGF	LPP EM SACRA ESTAGIO 4	Concluída	Eventos Adversos Relaciona dos à	Lesão por pressão		
13/07/2021	CE	FORTALE ZA	HGF	LPP ESTÁGIO 4 EM REGIÃO	Concluída	Eventos Adversos Relaciona dos à	Lesão por pressão		
13/07/2021	CE	FORTALE ZA	HGF	LPP ESTÁGIO 4 EM REGIÃO	Concluída	Eventos Adversos Relaciona dos à	Lesão por pressão		
13/07/2021	CE	FORTALE ZA	HGF	UPP ESTAGIO 3 EM SACRA	Concluída	Eventos Adversos Relaciona dos à	Lesão por pressão		
13/07/2021	CE	FORTALE ZA	HGF	LPP ESTÁGIO 3 EM REGIÃO	Concluída	Eventos Adversos Relaciona dos à	Lesão por pressão		
13/07/2021	CE	FORTALE ZA	HGF	LPP ESTÁGIO 3 EM GLUTEO	Concluída	Eventos Adversos Relaciona dos à	Lesão por pressão		
13/07/2021	CE	FORTALE ZA	HGF	UPP EM SACRA ESTAGIO 3	Concluída	Eventos Adversos Relaciona dos à	Lesão por pressão		
13/07/2021	CE	FORTALE ZA	HGF	UPP EM SACRA ESTAGIO 3	Concluída	Eventos Adversos Relaciona dos à	Lesão por pressão		
13/07/2021	CE	FORTALE ZA	HGF	LPP EM SACRA ESTÁGIO 4	Concluída	Eventos Adversos Relaciona dos à	Lesão por pressão		
13/07/2021	CE	FORTALE ZA	HGF	LPP EM SACRA ESTÁGIO 4	Concluída	Eventos Adversos Relaciona dos à	Lesão por pressão		
13/07/2021	CE	FORTALE ZA	HGF	LESÃO POR PRESSÃ O EM	Concluída	Eventos Adversos Relaciona dos à	Lesão por pressão		
13/07/2021	CE	FORTALE ZA	HGF	LPP ESTÁGIO 3 EM CALCAN	Concluída	Eventos Adversos Relaciona dos à	Lesão por pressão		

13/07/2021	CE	FORTALEZA	HGF	UPP EM SACRA ESTAGIO 3	Concluída	Eventos Adversos Relacionados à	Lesão por pressão		
13/07/2021	CE	FORTALEZA	HGF	LPP ESTÁGIO 3 EM REGIÃO	Concluída	Eventos Adversos Relacionados à	Lesão por pressão		
13/07/2021	CE	FORTALEZA	HGF	UPP ESTRAGIO 3 EM SACRA	Concluída	Eventos Adversos Relacionados à	Lesão por pressão		
14/07/2021	CE	FORTALEZA	HGF	LPP ESTÁGIO 4 EM DORSO	Concluída	Eventos Adversos Relacionados à	Lesão por pressão		
14/07/2021	CE	FORTALEZA	HGF	PERCA ACIDENTAL DE SONDA	Enviada	Eventos Adversos Relacionados à	Falhas durante a assistência à saúde		
19/07/2021	CE	FORTALEZA	HGF	Paciente se desequilibrou ao	Enviada	Eventos Adversos Relacionados à	Queda do paciente		
19/07/2021	CE	FORTALEZA	HGF	Administrado Tramadol no	Enviada	Eventos Adversos Relacionados à	Falhas durante a assistência à saúde		
02/08/2021	CE	FORTALEZA	HGF	PACIENTE URINOU NO	Enviada	Eventos Adversos Relacionados à	Queda do paciente		
02/08/2021	CE	FORTALEZA	HGF	Paciente sem acompanhante	Enviada	Eventos Adversos Relacionados à	Queda do paciente		
02/08/2021	CE	FORTALEZA	HGF	REALIZADA TRÊS DOSES DE	Enviada	Eventos Adversos Relacionados à	Falhas durante a assistência à saúde		
02/08/2021	CE	FORTALEZA	HGF	ACOMPANHANTE PORTA ÁGUA	Enviada	Eventos Adversos Relacionados à	Acidentes do paciente	Exposição à substância química	
02/08/2021	CE	FORTALEZA	HGF	AO LEVANTAR DO LEITO	Enviada	Eventos Adversos Relacionados à	Queda do paciente		
02/08/2021	CE	FORTALEZA	HGF	Paciente em uso de MESNA	Enviada	Eventos Adversos Relacionados à	Falhas durante a assistência à saúde		
23/08/2021	CE	FORTALEZA	HGF	FLEBITE EM MEMBRO	Enviada	Eventos Adversos Relacionados à	Falhas envolvendo o cateter venoso		
23/08/2021	CE	FORTALEZA	HGF	observado flebite em MSD apresenta	Enviada	Eventos Adversos Relacionados à	Falhas envolvendo o cateter venoso		

23/08/2021	CE	FORTALEZA	HGF	PACIENTE APRESENTOU	Enviada	Eventos Adversos Relacionados à	Falhas envolvendo cateter venoso		
23/08/2021	CE	FORTALEZA	HGF	PACIENTE APRESENTOU	Enviada	Eventos Adversos Relacionados à	Falhas envolvendo cateter venoso		
23/08/2021	CE	FORTALEZA	HGF	PACIENTE APRESENTOU	Enviada	Eventos Adversos Relacionados à	Falhas envolvendo cateter venoso		
23/08/2021	CE	FORTALEZA	HGF	Flebite MSD	Enviada	Eventos Adversos Relacionados à	Falhas envolvendo cateter venoso		
23/08/2021	CE	FORTALEZA	HGF	O ACOMPANHANTE LEVA O	Enviada	Eventos Adversos Relacionados à	Queda do paciente		
23/08/2021	CE	FORTALEZA	HGF	QUEDA DA PROPRIA ALTURA	Enviada	Eventos Adversos Relacionados à	Queda do paciente		
23/08/2021	CE	FORTALEZA	HGF	SEGUNDO A PACIENTE FOI	Enviada	Eventos Adversos Relacionados à	Queda do paciente		
23/08/2021	CE	FORTALEZA	HGF	Paciente em uso de MESNA	Enviada	Eventos Adversos Relacionados à	Falhas durante a assistência à saúde		
23/08/2021	CE	FORTALEZA	HGF	AO LEVANTAR DO LEITO	Enviada	Eventos Adversos Relacionados à	Queda do paciente		
23/08/2021	CE	FORTALEZA	HGF	AGITADO E DESORIENTADO	Enviada	Eventos Adversos Relacionados à	Falhas envolvendo sondas		
23/08/2021	CE	FORTALEZA	HGF	SERTOR ESTAVA TENDO DETETIZ	Enviada	Eventos Adversos Relacionados à	Falhas durante a assistência à saúde		
23/08/2021	CE	FORTALEZA	HGF	PACIENTE SE LEVANTOU	Enviada	Eventos Adversos Relacionados à	Queda do paciente		
26/08/2021	CE	FORTALEZA	HGF	PACIENTE AGITADO	Enviada	Eventos Adversos Relacionados à	Queda do paciente		
26/08/2021	CE	FORTALEZA	HGF	HIPEREMIA E DOR EM MSD	Enviada	Eventos Adversos Relacionados à	Falhas envolvendo cateter venoso		
26/08/2021	CE	FORTALEZA	HGF	-	Enviada	Eventos Adversos Relacionados à	Falhas envolvendo cateter venoso		

26/08/2021	CE	FORTALEZA	HGF	DURANTE REALIZAÇÃO DO	Enviada	Eventos Adversos Relacionados à	Falhas durante a assistência à saúde		
31/08/2021	CE	FORTALEZA	HGF	APRESENTOU HIPEREMIA	Enviada	Eventos Adversos Relacionados à	Falhas envolvendo o cateter venoso		
31/08/2021	CE	FORTALEZA	HGF	Flebite MSD	Enviada	Eventos Adversos Relacionados à	Falhas envolvendo o cateter venoso		
31/08/2021	CE	FORTALEZA	HGF	APRESENTOU FLEBITE EM	Enviada	Eventos Adversos Relacionados à	Falhas envolvendo o cateter venoso		
31/08/2021	CE	FORTALEZA	HGF	FLEBITE EM MSE	Enviada	Eventos Adversos Relacionados à	Falhas envolvendo o cateter venoso		
31/08/2021	CE	FORTALEZA	HGF	Prescriçao de morfina 10mg	Enviada	Eventos Adversos Relacionados à	Falhas durante a assistência à saúde		
31/08/2021	CE	FORTALEZA	HGF	-	Enviada	Eventos Adversos Relacionados à	Falhas durante a assistência à saúde		
01/09/2021	CE	FORTALEZA	HGF	cresidente da gastro realizou	Enviada	Eventos Adversos Relacionados à	Falhas envolvendo o cateter venoso		
01/09/2021	CE	FORTALEZA	HGF	FALHA NA COMUNICAÇÃO	Enviada	Eventos Adversos Relacionados à	Broncoaspiração		
02/09/2021	CE	FORTALEZA	HGF	Paciente muito secretivo, contudo	Enviada	Eventos Adversos Relacionados à	Falhas durante a assistência à saúde		
06/09/2021	CE	FORTALEZA	HGF	ACOMPANHANTE CHAMOU PARA	Enviada	Eventos Adversos Relacionados à	Queda do paciente		
06/09/2021	CE	FORTALEZA	HGF	PACIENTE SONOLENTO	Enviada	Eventos Adversos Relacionados à	Falhas durante a assistência à saúde		
06/09/2021	CE	FORTALEZA	HGF	PACIENTE HIPERSECRETIVO	Enviada	Eventos Adversos Relacionados à	Falhas durante a assistência à saúde		
06/09/2021	CE	FORTALEZA	HGF	possibilidade de lesão em nervo	Enviada	Eventos Adversos Relacionados à	Falhas durante a assistência à saúde		
06/09/2021	CE	FORTALEZA	HGF	Ao realizar busca ativa do	Enviada	Eventos Adversos Relacionados à	Falhas durante a assistência à saúde		

06/09/2021	CE	FORTALEZA	HGF	Extubado pós pronacao por	Enviada	Eventos Adversos Relacionados à	Extubação endotraqueal		
06/09/2021	CE	FORTALEZA	HGF	Flebite em MSE	Enviada	Eventos Adversos Relacionados à	Falhas durante a assistência à saúde		
06/09/2021	CE	FORTALEZA	HGF	Flebite em msd	Enviada	Eventos Adversos Relacionados à	Falhas durante a assistência à saúde		
06/09/2021	CE	FORTALEZA	HGF	Paciente apresentou flebite em msd	Enviada	Eventos Adversos Relacionados à	Falhas durante a assistência à saúde		
06/09/2021	CE	FORTALEZA	HGF	Evento relacionado queda, enquanto	Enviada	Eventos Adversos Relacionados à	Queda do paciente		
13/09/2021	CE	FORTALEZA	HGF	Paciente com leucemia aguda	Enviada	Eventos Adversos Relacionados à	Falhas durante a assistência à saúde		
13/09/2021	CE	FORTALEZA	HGF	LPP EM CALCÂNEOS ESTÁGIO	Concluída	Eventos Adversos Relacionados à	Lesão por pressão		
13/09/2021	CE	FORTALEZA	HGF	LPP ESTÁGIO 3 EM GLÚTEO	Concluída	Eventos Adversos Relacionados à	Lesão por pressão		
13/09/2021	CE	FORTALEZA	HGF	LPP ESTÁGIO 3 EM SACRA	Concluída	Eventos Adversos Relacionados à	Lesão por pressão		
13/09/2021	CE	FORTALEZA	HGF	LPP EM GLÚTEOS ESTÁGIO	Concluída	Eventos Adversos Relacionados à	Lesão por pressão		
13/09/2021	CE	FORTALEZA	HGF	LPP EM SACRA ESTÁGIO 3	Concluída	Eventos Adversos Relacionados à	Lesão por pressão		
13/09/2021	CE	FORTALEZA	HGF	MSD HIPEREMIADO E COM	Enviada	Eventos Adversos Relacionados à	Falhas envolvendo cateter venoso		
13/09/2021	CE	FORTALEZA	HGF	LPP EM REGIÃO OCCIPITAL	Concluída	Eventos Adversos Relacionados à	Lesão por pressão		
13/09/2021	CE	FORTALEZA	HGF	LPP EM TROCÂNTER ESQUER	Concluída	Eventos Adversos Relacionados à	Lesão por pressão		
14/09/2021	CE	FORTALEZA	HGF	LPP EM REGIÃO SACRA ESTÁGIO	Concluída	Eventos Adversos Relacionados à	Lesão por pressão		

14/09/2021	CE	FORTALEZA	HGF	LPP EM REGIÃO SACRA ESTÁGIO	Concluída	Eventos Adversos Relacionados à	Lesão por pressão		
14/09/2021	CE	FORTALEZA	HGF	LPP EM REGIÃO SACRA ESTÁGIO	Concluída	Eventos Adversos Relacionados à	Lesão por pressão		
14/09/2021	CE	FORTALEZA	HGF	LPP EM MMII ESTÁGIO 3	Concluída	Eventos Adversos Relacionados à	Lesão por pressão		
14/09/2021	CE	FORTALEZA	HGF	LPP EM REGIÃO SACRA ESTÁGIO	Concluída	Eventos Adversos Relacionados à	Lesão por pressão		
14/09/2021	CE	FORTALEZA	HGF	LPP EM PAVILHÃO AURICUL	Concluída	Eventos Adversos Relacionados à	Lesão por pressão		
14/09/2021	CE	FORTALEZA	HGF	-	Concluída	Eventos Adversos Relacionados à	Lesão por pressão		
14/09/2021	CE	FORTALEZA	HGF	LPP EM REGIÃO SACRA ESTÁGIO	Concluída	Eventos Adversos Relacionados à	Lesão por pressão		
14/09/2021	CE	FORTALEZA	HGF	LPP EM CALCANEO ESQUER	Concluída	Eventos Adversos Relacionados à	Lesão por pressão		
14/09/2021	CE	FORTALEZA	HGF	LPP ESTÁGIO 2 EM GLÚTEO	Enviada	Eventos Adversos Relacionados à	Lesão por pressão		
14/09/2021	CE	FORTALEZA	HGF	LPP ESTÁGIO 1 EM GLÚTEO	Enviada	Eventos Adversos Relacionados à	Lesão por pressão		
14/09/2021	CE	FORTALEZA	HGF	LPP EM SACRA ESTÁGIO 2	Enviada	Eventos Adversos Relacionados à	Lesão por pressão		
14/09/2021	CE	FORTALEZA	HGF	LPP EM CALCÂNEO ESQUER	Enviada	Eventos Adversos Relacionados à	Lesão por pressão		
14/09/2021	CE	FORTALEZA	HGF	LPP EM REGIÃO SACRA ESTÁGIO	Enviada	Eventos Adversos Relacionados à	Lesão por pressão		
14/09/2021	CE	FORTALEZA	HGF	LPP EM MALÉOLO DIREITO	Enviada	Eventos Adversos Relacionados à	Lesão por pressão		
14/09/2021	CE	FORTALEZA	HGF	FACE LATERAL DO PÉ ESQUER	Enviada	Eventos Adversos Relacionados à	Lesão por pressão		

14/09/2021	CE	FORTALEZA	HGF	LPP EM CALCÂNEOS ESTÁGIO	Enviada	Eventos Adversos Relacionados à	Lesão por pressão		
14/09/2021	CE	FORTALEZA	HGF	LPP EM REGIÃO SACRA ESTÁGIO	Enviada	Eventos Adversos Relacionados à	Lesão por pressão		
14/09/2021	CE	FORTALEZA	HGF	LPP EM REGIÃO SACRA ESTÁGIO	Enviada	Eventos Adversos Relacionados à	Lesão por pressão		
14/09/2021	CE	FORTALEZA	HGF	LPP EM REGIÃO SACRA ESTÁGIO	Enviada	Eventos Adversos Relacionados à	Lesão por pressão		
14/09/2021	CE	FORTALEZA	HGF	LPP EM REGIÃO SACRA ESTÁGIO	Enviada	Eventos Adversos Relacionados à	Lesão por pressão		
14/09/2021	CE	FORTALEZA	HGF	LPP SACRA ESTÁGIO 2:	Enviada	Eventos Adversos Relacionados à	Lesão por pressão		
14/09/2021	CE	FORTALEZA	HGF	-	Enviada	Eventos Adversos Relacionados à	Lesão por pressão		
14/09/2021	CE	FORTALEZA	HGF	LPP EM ESCÁPULA ESTÁGIO	Enviada	Eventos Adversos Relacionados à	Lesão por pressão		
14/09/2021	CE	FORTALEZA	HGF	LPP EM REGIÃO SACRA ESTÁGIO	Enviada	Eventos Adversos Relacionados à	Lesão por pressão		
14/09/2021	CE	FORTALEZA	HGF	LPP EM REGIÃO SACRA ESTÁGIO	Enviada	Eventos Adversos Relacionados à	Lesão por pressão		
14/09/2021	CE	FORTALEZA	HGF	LPP EM GLÚTEO DIREITO ESTÁGIO	Enviada	Eventos Adversos Relacionados à	Lesão por pressão		
14/09/2021	CE	FORTALEZA	HGF	LPP EM GLÚTEOS ESTÁGIO	Enviada	Eventos Adversos Relacionados à	Lesão por pressão		
14/09/2021	CE	FORTALEZA	HGF	LPP EM TROCANTER DIREITO	Enviada	Eventos Adversos Relacionados à	Lesão por pressão		
14/09/2021	CE	FORTALEZA	HGF	LPP EM SACRA ESTÁGIO 1:	Enviada	Eventos Adversos Relacionados à	Lesão por pressão		
14/09/2021	CE	FORTALEZA	HGF	LPP EM REGIÃO SACRA ESTÁGIO	Enviada	Eventos Adversos Relacionados à	Lesão por pressão		

14/09/2021	CE	FORTALEZA	HGF	LPP ESTÁGIO 2 EM GLUTEO	Enviada	Eventos Adversos Relacionados à	Lesão por pressão		
14/09/2021	CE	FORTALEZA	HGF	LPP SACRA ESTÁGIO 2:	Enviada	Eventos Adversos Relacionados à	Lesão por pressão		
14/09/2021	CE	FORTALEZA	HGF	LPP EM SACRA ESTAGIO 1:	Enviada	Eventos Adversos Relacionados à	Lesão por pressão		
14/09/2021	CE	FORTALEZA	HGF	LPP ESTÁGIO 2 EM GLÚTEO	Enviada	Eventos Adversos Relacionados à	Lesão por pressão		
14/09/2021	CE	FORTALEZA	HGF	LPP ESTÁGIO 1 EM REGIÃO	Enviada	Eventos Adversos Relacionados à	Lesão por pressão		
14/09/2021	CE	FORTALEZA	HGF	LPP SACRA ESTÁGIO 1:	Enviada	Eventos Adversos Relacionados à	Lesão por pressão		
14/09/2021	CE	FORTALEZA	HGF	LPP SACRA ESTÁGIO 1:	Enviada	Eventos Adversos Relacionados à	Lesão por pressão		
14/09/2021	CE	FORTALEZA	HGF	LPP EM INTERGLÚTEO ESTÁGIO	Enviada	Eventos Adversos Relacionados à	Lesão por pressão		
14/09/2021	CE	FORTALEZA	HGF	LPP EM CALCÂNEO DIREITO	Enviada	Eventos Adversos Relacionados à	Lesão por pressão		
14/09/2021	CE	FORTALEZA	HGF	LPP EM SACRA ESTÁGIO 2:	Enviada	Eventos Adversos Relacionados à	Lesão por pressão		
14/09/2021	CE	FORTALEZA	HGF	LPP EM REGIÃO SACRA ESTÁGIO	Enviada	Eventos Adversos Relacionados à	Lesão por pressão		
16/09/2021	CE	FORTALEZA	HGF	LPP ESTÁGIO 2 TROCAN	Enviada	Eventos Adversos Relacionados à	Lesão por pressão		
16/09/2021	CE	FORTALEZA	HGF	LPP EM ESCÁPULAS ESTÁGIO	Enviada	Eventos Adversos Relacionados à	Lesão por pressão		
16/09/2021	CE	FORTALEZA	HGF	LPP EM REGIÃO SACRA ESTÁGIO	Enviada	Eventos Adversos Relacionados à	Lesão por pressão		
16/09/2021	CE	FORTALEZA	HGF	LPP EM REGIÃO SACRA ESTÁGIO	Enviada	Eventos Adversos Relacionados à	Lesão por pressão		

16/09/2021	CE	FORTALE ZA	HGF	LPP EM GLÚTEO ESQUER DO	Enviada	Eventos Adversos Relaciona dos à	Lesão por pressão		
16/09/2021	CE	FORTALE ZA	HGF	LPP EM REGIÃO SACRA ESTÁGIO	Enviada	Eventos Adversos Relaciona dos à	Lesão por pressão		
16/09/2021	CE	FORTALE ZA	HGF	LPP EM REGIÃO SACRA ESTÁGIO	Enviada	Eventos Adversos Relaciona dos à	Lesão por pressão		
16/09/2021	CE	FORTALE ZA	HGF	LPP EM REGIÃO SACRA ESTÁGIO	Enviada	Eventos Adversos Relaciona dos à	Lesão por pressão		
16/09/2021	CE	FORTALE ZA	HGF	LPP EM SACRA ESTÁGIO 2	Enviada	Eventos Adversos Relaciona dos à	Lesão por pressão		
16/09/2021	CE	FORTALE ZA	HGF	LPP EM GLÚTEO DIREITO ESTÁGIO	Enviada	Eventos Adversos Relaciona dos à	Lesão por pressão		
16/09/2021	CE	FORTALE ZA	HGF	LPP EM REGIÃO SACRA ESTÁGIO	Enviada	Eventos Adversos Relaciona dos à	Lesão por pressão		
16/09/2021	CE	FORTALE ZA	HGF	LPP ESTÁGIO 2 EM SACRA;	Enviada	Eventos Adversos Relaciona dos à	Lesão por pressão		
16/09/2021	CE	FORTALE ZA	HGF	LPP EM MALÉOL O ESQUER	Enviada	Eventos Adversos Relaciona dos à	Lesão por pressão		
16/09/2021	CE	FORTALE ZA	HGF	LPP EM REGIÃO SACRA ESTÁGIO	Enviada	Eventos Adversos Relaciona dos à	Lesão por pressão		
16/09/2021	CE	FORTALE ZA	HGF	LPP EM REGIÃO SACRA ESTÁGIO	Enviada	Eventos Adversos Relaciona dos à	Lesão por pressão		
16/09/2021	CE	FORTALE ZA	HGF	LPP EM GLÚTEO DIREITO ESTÁGIO	Enviada	Eventos Adversos Relaciona dos à	Lesão por pressão		
16/09/2021	CE	FORTALE ZA	HGF	LPP EM GLÚTEO S ESTÁGIO	Enviada	Eventos Adversos Relaciona dos à	Lesão por pressão		
16/09/2021	CE	FORTALE ZA	HGF	LPP SACRA ESTÁGIO 2	Enviada	Eventos Adversos Relaciona dos à	Lesão por pressão		
17/09/2021	CE	FORTALE ZA	HGF	LPP SACRA ESTÁGIO 2;	Enviada	Eventos Adversos Relaciona dos à	Lesão por pressão		

17/09/2021	CE	FORTALE ZA	HGF	LPP EM REGIÃO SACRA ESTÁGIO	Enviada	Eventos Adversos Relaciona dos à	Lesão por pressão		
17/09/2021	CE	FORTALE ZA	HGF	LPP EM ESCÁPUL AS ESTÁGIO	Enviada	Eventos Adversos Relaciona dos à	Lesão por pressão		
17/09/2021	CE	FORTALE ZA	HGF	LPP EM REGIÃO SACRA ESTÁGIO	Enviada	Eventos Adversos Relaciona dos à	Lesão por pressão		
17/09/2021	CE	FORTALE ZA	HGF	-	Enviada	Eventos Adversos Relaciona dos à	Lesão por pressão		
17/09/2021	CE	FORTALE ZA	HGF	LPP EM CALCÂN EOS D/E ESTÁGIO	Enviada	Eventos Adversos Relaciona dos à	Lesão por pressão		
17/09/2021	CE	FORTALE ZA	HGF	LPP EM GLÚTEO DIREITO ESTÁGIO	Enviada	Eventos Adversos Relaciona dos à	Lesão por pressão		
17/09/2021	CE	FORTALE ZA	HGF	-	Enviada	Eventos Adversos Relaciona dos à	Lesão por pressão		
17/09/2021	CE	FORTALE ZA	HGF	LPP EM COXA ESQUER DA	Enviada	Eventos Adversos Relaciona dos à	Lesão por pressão		
17/09/2021	CE	FORTALE ZA	HGF	LPP EM DORSO ESTÁGIO 2:	Enviada	Eventos Adversos Relaciona dos à	Lesão por pressão		
17/09/2021	CE	FORTALE ZA	HGF	LPP EM REGIÃO SACRA ESTÁGIO	Enviada	Eventos Adversos Relaciona dos à	Lesão por pressão		
17/09/2021	CE	FORTALE ZA	HGF	LPP EM CALCÂN EOS D/E ESTÁGIO	Enviada	Eventos Adversos Relaciona dos à	Lesão por pressão		
17/09/2021	CE	FORTALE ZA	HGF	LPP EM REGIÃO SACRA ESTÁGIO	Enviada	Eventos Adversos Relaciona dos à	Lesão por pressão		
17/09/2021	CE	FORTALE ZA	HGF	LPP EM LPP EM GLÚTEO S	Enviada	Eventos Adversos Relaciona dos à	Lesão por pressão		
17/09/2021	CE	FORTALE ZA	HGF	LPP EM REGIÃO SACRA ESTÁGIO	Enviada	Eventos Adversos Relaciona dos à	Lesão por pressão		
17/09/2021	CE	FORTALE ZA	HGF	LPP EM SACRA ESTÁGIO 2:	Enviada	Eventos Adversos Relaciona dos à	Lesão por pressão		

17/09/2021	CE	FORTALEZA	HGF	-	Enviada	Eventos Adversos Relacionados à	Lesão por pressão		
17/09/2021	CE	FORTALEZA	HGF	-	Enviada	Eventos Adversos Relacionados à	Lesão por pressão		
17/09/2021	CE	FORTALEZA	HGF	LPP EM GLÚTEOS ESTÁGIO	Enviada	Eventos Adversos Relacionados à	Lesão por pressão		
17/09/2021	CE	FORTALEZA	HGF	LPP EM REGIÃO SACRA ESTÁGIO	Enviada	Eventos Adversos Relacionados à	Lesão por pressão		
17/09/2021	CE	FORTALEZA	HGF	LPP EM REGIÃO SACRA ESTÁGIO	Enviada	Eventos Adversos Relacionados à	Lesão por pressão		
17/09/2021	CE	FORTALEZA	HGF	LPP EM REGIÃO SACRA ESTÁGIO	Enviada	Eventos Adversos Relacionados à	Lesão por pressão		
17/09/2021	CE	FORTALEZA	HGF	LPP EM REGIÃO SACRA ESTÁGIO	Enviada	Eventos Adversos Relacionados à	Lesão por pressão		
17/09/2021	CE	FORTALEZA	HGF	LPP EM REGIÃO SACRA ESTÁGIO	Enviada	Eventos Adversos Relacionados à	Lesão por pressão		
17/09/2021	CE	FORTALEZA	HGF	LPP EM REGIÃO SACRA ESTÁGIO	Enviada	Eventos Adversos Relacionados à	Lesão por pressão		
17/09/2021	CE	FORTALEZA	HGF	LPP EM SACRA ESTÁGIO 1:	Enviada	Eventos Adversos Relacionados à	Lesão por pressão		
17/09/2021	CE	FORTALEZA	HGF	LPP EM TROCANTER DIREITO	Enviada	Eventos Adversos Relacionados à	Lesão por pressão		
17/09/2021	CE	FORTALEZA	HGF	LPP EM SACRA ESTÁGIO 2:	Enviada	Eventos Adversos Relacionados à	Lesão por pressão		
17/09/2021	CE	FORTALEZA	HGF	LPP EM CALCÂNEO ESQUER	Enviada	Eventos Adversos Relacionados à	Lesão por pressão		
17/09/2021	CE	FORTALEZA	HGF	LPP EM DORSO ESTÁGIO 1:	Enviada	Eventos Adversos Relacionados à	Lesão por pressão		
17/09/2021	CE	FORTALEZA	HGF	LPP EM REGIÃO SACRA ESTÁGIO	Enviada	Eventos Adversos Relacionados à	Lesão por pressão		

20/09/2021	CE	FORTALEZA	HGF	PACIENTE TRANSPORTADO	Enviada	Eventos Adversos Relacionados à	Falhas durante a assistência à saúde		
21/09/2021	CE	FORTALEZA	HGF	LPP EM GLÚTEO DIREITO ESTÁGIO	Enviada	Eventos Adversos Relacionados à	Lesão por pressão		
21/09/2021	CE	FORTALEZA	HGF	LPP EM TROCANTER ESQUER	Enviada	Eventos Adversos Relacionados à	Lesão por pressão		
21/09/2021	CE	FORTALEZA	HGF	LPP EM SACRA ESTÁGIO 2	Enviada	Eventos Adversos Relacionados à	Lesão por pressão		
21/09/2021	CE	FORTALEZA	HGF	UPP EM SACRA	Enviada	Eventos Adversos Relacionados à	Lesão por pressão		
21/09/2021	CE	FORTALEZA	HGF	UPP EM GLUTEOS ESTAGIO	Enviada	Eventos Adversos Relacionados à	Lesão por pressão		
21/09/2021	CE	FORTALEZA	HGF	UPP EM TÓRAX POSTERIOR D	Enviada	Eventos Adversos Relacionados à	Lesão por pressão		
21/09/2021	CE	FORTALEZA	HGF	UPP EM SACRA ESTAGIO 2	Enviada	Eventos Adversos Relacionados à	Lesão por pressão		
22/09/2021	CE	FORTALEZA	HGF	upp em gluteo esquerdo	Enviada	Eventos Adversos Relacionados à	Lesão por pressão		
22/09/2021	CE	FORTALEZA	HGF	UPP EM SACRA ESTAGIO 2	Enviada	Eventos Adversos Relacionados à	Lesão por pressão		
22/09/2021	CE	FORTALEZA	HGF	UPP EM SACRA 2	Enviada	Eventos Adversos Relacionados à	Lesão por pressão		
22/09/2021	CE	FORTALEZA	HGF	UPP EM GLUTEOS	Enviada	Eventos Adversos Relacionados à	Lesão por pressão		
22/09/2021	CE	FORTALEZA	HGF	UPP EM SACRA 2	Enviada	Eventos Adversos Relacionados à	Lesão por pressão		
22/09/2021	CE	FORTALEZA	HGF	UPP EM SACRA	Enviada	Eventos Adversos Relacionados à	Lesão por pressão		
22/09/2021	CE	FORTALEZA	HGF	UPP EM SACRA	Enviada	Eventos Adversos Relacionados à	Lesão por pressão		

22/09/2021	CE	FORTALE ZA	HGF	UPP EM SACRA 1	Enviada	Eventos Adversos Relaciona dos à	Lesão por pressão		
22/09/2021	CE	FORTALE ZA	HGF	UPP EM SACRA	Enviada	Eventos Adversos Relaciona dos à	Lesão por pressão		
23/09/2021	CE	FORTALE ZA	HGF	Suspença o inadequad a de	Enviada	Eventos Adversos Relaciona dos à	Falhas durante a assistênci a à saúde		
23/09/2021	CE	FORTALE ZA	HGF	Paciente era pra ter realizado	Enviada	Eventos Adversos Relaciona dos à	Falhas durante a assistênci a à saúde		
23/09/2021	CE	FORTALE ZA	HGF	APÓS UTILIZAÇ ÃO DE DROGA	Enviada	Eventos Adversos Relaciona dos à	Falhas durante a assistênci a à saúde		
23/09/2021	CE	FORTALE ZA	HGF	LPP EM CALCÂN EOS ESTÁGIO	Enviada	Eventos Adversos Relaciona dos à	Lesão por pressão		
24/09/2021	CE	FORTALE ZA	HGF	Auxiliar de transporte arrancou	Enviada	Eventos Adversos Relaciona dos à	Falhas envolvend o sondas		
24/09/2021	CE	FORTALE ZA	HGF	PACIENT E TROPEÇ OU EM	Enviada	Eventos Adversos Relaciona dos à	Queda do paciente		
24/09/2021	CE	FORTALE ZA	HGF	Evento relacionad o queda, enquanto	Enviada	Eventos Adversos Relaciona dos à	Queda do paciente		
24/09/2021	CE	FORTALE ZA	HGF	paciente foi transporta da em	Enviada	Eventos Adversos Relaciona dos à	Falhas durante a assistênci a à saúde		
24/09/2021	CE	FORTALE ZA	HGF	APLICAÇ ÃO DE MEDICAÇ ÃO IM -	Enviada	Eventos Adversos Relaciona dos à	Falhas durante a assistênci a à saúde		
27/09/2021	CE	FORTALE ZA	HGF	LPP EM DORSO ESTÁGIO 2	Enviada	Eventos Adversos Relaciona dos à	Lesão por pressão		
27/09/2021	CE	FORTALE ZA	HGF	LPP EM REGIÃO SACRA ESTÁGIO	Enviada	Eventos Adversos Relaciona dos à	Lesão por pressão		
27/09/2021	CE	FORTALE ZA	HGF	LPP EM REGIÃO SACRA ESTÁGIO	Enviada	Eventos Adversos Relaciona dos à	Lesão por pressão		
27/09/2021	CE	FORTALE ZA	HGF	LPP EM REGIÃO SACRA ESTÁGIO	Enviada	Eventos Adversos Relaciona dos à	Lesão por pressão		

27/09/2021	CE	FORTALEZA	HGF	LPP EM SACRA ESTÁGIO 2	Enviada	Eventos Adversos Relacionados à	Lesão por pressão		
27/09/2021	CE	FORTALEZA	HGF	UPP EM SACRA	Enviada	Eventos Adversos Relacionados à	Lesão por pressão		
27/09/2021	CE	FORTALEZA	HGF	UPP EM SACRA 2	Enviada	Eventos Adversos Relacionados à	Lesão por pressão		
27/09/2021	CE	FORTALEZA	HGF	UPP EM SACRA	Enviada	Eventos Adversos Relacionados à	Lesão por pressão		
27/09/2021	CE	FORTALEZA	HGF	UPP EM SACRA	Enviada	Eventos Adversos Relacionados à	Lesão por pressão		
27/09/2021	CE	FORTALEZA	HGF	UPP EM SACRA ESTAGIO 1	Enviada	Eventos Adversos Relacionados à	Lesão por pressão		
27/09/2021	CE	FORTALEZA	HGF	UPP EM GLUTEOS	Enviada	Eventos Adversos Relacionados à	Lesão por pressão		
27/09/2021	CE	FORTALEZA	HGF	UPP EM SACRA	Enviada	Eventos Adversos Relacionados à	Lesão por pressão		
27/09/2021	CE	FORTALEZA	HGF	UPP EM SACRA	Enviada	Eventos Adversos Relacionados à	Lesão por pressão		
27/09/2021	CE	FORTALEZA	HGF	UPP EM DORSO 2	Enviada	Eventos Adversos Relacionados à	Lesão por pressão		
27/09/2021	CE	FORTALEZA	HGF	UPP EM SACRA	Enviada	Eventos Adversos Relacionados à	Lesão por pressão		
27/09/2021	CE	FORTALEZA	HGF	UPP EM SACRA	Enviada	Eventos Adversos Relacionados à	Lesão por pressão		
27/09/2021	CE	FORTALEZA	HGF	UPP EMD GLUTEO ESQUER	Enviada	Eventos Adversos Relacionados à	Lesão por pressão		
27/09/2021	CE	FORTALEZA	HGF	UPP EM SACRA 2	Enviada	Eventos Adversos Relacionados à	Lesão por pressão		
27/09/2021	CE	FORTALEZA	HGF	UPP EM SACRA	Enviada	Eventos Adversos Relacionados à	Lesão por pressão		

27/09/2021	CE	FORTALE ZA	HGF	UPP EM SACRA	Enviada	Eventos Adversos Relaciona dos à	Lesão por pressão		
27/09/2021	CE	FORTALE ZA	HGF	UPP EM SACRA	Enviada	Eventos Adversos Relaciona dos à	Lesão por pressão		
27/09/2021	CE	FORTALE ZA	HGF	UPP EM SACRA	Enviada	Eventos Adversos Relaciona dos à	Lesão por pressão		
27/09/2021	CE	FORTALE ZA	HGF	UPP EM OCCIPTA L	Concluída	Eventos Adversos Relaciona dos à	Lesão por pressão		
27/09/2021	CE	FORTALE ZA	HGF	UPP EM SACRA	Enviada	Eventos Adversos Relaciona dos à	Lesão por pressão		
27/09/2021	CE	FORTALE ZA	HGF	UPP EM SACRA	Enviada	Eventos Adversos Relaciona dos à	Lesão por pressão		
27/09/2021	CE	FORTALE ZA	HGF	UPP EM CALCAN EO ESQUER	Enviada	Eventos Adversos Relaciona dos à	Lesão por pressão		
27/09/2021	CE	FORTALE ZA	HGF	UPP EM GLUTEO S	Enviada	Eventos Adversos Relaciona dos à	Lesão por pressão		
27/09/2021	CE	FORTALE ZA	HGF	UPP EM CALCAN EOS	Enviada	Eventos Adversos Relaciona dos à	Lesão por pressão		
27/09/2021	CE	FORTALE ZA	HGF	UPP INTERGL UTEO	Enviada	Eventos Adversos Relaciona dos à	Lesão por pressão		
27/09/2021	CE	FORTALE ZA	HGF	UPP EM SACRA	Enviada	Eventos Adversos Relaciona dos à	Lesão por pressão		
27/09/2021	CE	FORTALE ZA	HGF	UPP EM OCCIPTO	Enviada	Eventos Adversos Relaciona dos à	Lesão por pressão		
27/09/2021	CE	FORTALE ZA	HGF	UPP EM SACRA	Concluída	Eventos Adversos Relaciona dos à	Lesão por pressão		
27/09/2021	CE	FORTALE ZA	HGF	UPP EM SACRA	Enviada	Eventos Adversos Relaciona dos à	Lesão por pressão		
27/09/2021	CE	FORTALE ZA	HGF	UPP EM SACRA	Enviada	Eventos Adversos Relaciona dos à	Lesão por pressão		

27/09/2021	CE	FORTALEZA	HGF	UPP EM SACRA	Enviada	Eventos Adversos Relacionados à	Lesão por pressão		
27/09/2021	CE	FORTALEZA	HGF	UPP EM SACRA	Enviada	Eventos Adversos Relacionados à	Lesão por pressão		
27/09/2021	CE	FORTALEZA	HGF	UPP EM SACRA	Enviada	Eventos Adversos Relacionados à	Lesão por pressão		
27/09/2021	CE	FORTALEZA	HGF	UPP INTERGLUTEO	Enviada	Eventos Adversos Relacionados à	Lesão por pressão		
27/09/2021	CE	FORTALEZA	HGF	UPP INTERGLUTEO	Enviada	Eventos Adversos Relacionados à	Lesão por pressão		
27/09/2021	CE	FORTALEZA	HGF	UPP EM SACRA	Enviada	Eventos Adversos Relacionados à	Lesão por pressão		
27/09/2021	CE	FORTALEZA	HGF	UPP INTERGLUTEO	Enviada	Eventos Adversos Relacionados à	Lesão por pressão		
27/09/2021	CE	FORTALEZA	HGF	UPP GLUTEO DIREITO	Enviada	Eventos Adversos Relacionados à	Lesão por pressão		
27/09/2021	CE	FORTALEZA	HGF	UPP EM GLUTEO ESQUERDO	Enviada	Eventos Adversos Relacionados à	Lesão por pressão		
27/09/2021	CE	FORTALEZA	HGF	LPP EM SACRA ESTÁGIO 2	Enviada	Eventos Adversos Relacionados à	Lesão por pressão		
27/09/2021	CE	FORTALEZA	HGF	LPP EM REGIÃO SACRA ESTÁGIO	Enviada	Eventos Adversos Relacionados à	Lesão por pressão		
27/09/2021	CE	FORTALEZA	HGF	UPP EM GLUTEOS	Enviada	Eventos Adversos Relacionados à	Lesão por pressão		
27/09/2021	CE	FORTALEZA	HGF	UPP EM GLUTEOS	Enviada	Eventos Adversos Relacionados à	Lesão por pressão		
27/09/2021	CE	FORTALEZA	HGF	LPP EM TROCÂNTER ESTÁGIO	Enviada	Eventos Adversos Relacionados à	Lesão por pressão		
27/09/2021	CE	FORTALEZA	HGF	UPP EM PAVILHÃO AURICUL	Enviada	Eventos Adversos Relacionados à	Lesão por pressão		

27/09/2021	CE	FORTALEZA	HGF	LPP EM TROCÂNTERES ESTÁGIO	Enviada	Eventos Adversos Relacionados à	Lesão por pressão		
27/09/2021	CE	FORTALEZA	HGF	LPP EM DORSO ESTÁGIO 2	Enviada	Eventos Adversos Relacionados à	Lesão por pressão		
27/09/2021	CE	FORTALEZA	HGF	-	Enviada	Eventos Adversos Relacionados à	Lesão por pressão		
27/09/2021	CE	FORTALEZA	HGF	LPP EM TROCÂNTER ESTÁGIO	Enviada	Eventos Adversos Relacionados à	Lesão por pressão		
27/09/2021	CE	FORTALEZA	HGF	UPP EM SACRA	Enviada	Eventos Adversos Relacionados à	Lesão por pressão		
27/09/2021	CE	FORTALEZA	HGF	LPP EM REGIÃO SACRA ESTÁGIO	Enviada	Eventos Adversos Relacionados à	Lesão por pressão		
27/09/2021	CE	FORTALEZA	HGF	-	Enviada	Eventos Adversos Relacionados à	Lesão por pressão		
27/09/2021	CE	FORTALEZA	HGF	-	Enviada	Eventos Adversos Relacionados à	Lesão por pressão		
27/09/2021	CE	FORTALEZA	HGF	UPP EM SACRA	Enviada	Eventos Adversos Relacionados à	Lesão por pressão		
27/09/2021	CE	FORTALEZA	HGF	UPP EM DORSO	Enviada	Eventos Adversos Relacionados à	Lesão por pressão		
27/09/2021	CE	FORTALEZA	HGF	LPP EM REGIÃO SACRA ESTÁGIO	Enviada	Eventos Adversos Relacionados à	Lesão por pressão		
27/09/2021	CE	FORTALEZA	HGF	UPP EM PANTURRILHA DIREITA	Enviada	Eventos Adversos Relacionados à	Lesão por pressão		
27/09/2021	CE	FORTALEZA	HGF	LPP EM REGIÃO SACRA ESTÁGIO	Enviada	Eventos Adversos Relacionados à	Lesão por pressão		
27/09/2021	CE	FORTALEZA	HGF	UPP EM COTOVELO DIREITO	Enviada	Eventos Adversos Relacionados à	Lesão por pressão		
27/09/2021	CE	FORTALEZA	HGF	UPP EM SACRA	Enviada	Eventos Adversos Relacionados à	Lesão por pressão		

27/09/2021	CE	FORTALEZA	HGF	LPP EM CALCÂNEO ESQUER	Enviada	Eventos Adversos Relacionados à	Lesão por pressão		
27/09/2021	CE	FORTALEZA	HGF	UPP EM CALCANEOS	Enviada	Eventos Adversos Relacionados à	Lesão por pressão		
27/09/2021	CE	FORTALEZA	HGF	LPP EM REGIÃO SACRA ESTÁGIO	Enviada	Eventos Adversos Relacionados à	Lesão por pressão		
27/09/2021	CE	FORTALEZA	HGF	UPP EM SACRA	Enviada	Eventos Adversos Relacionados à	Lesão por pressão		
27/09/2021	CE	FORTALEZA	HGF	LPP EM SACRA ESTÁGIO 1	Enviada	Eventos Adversos Relacionados à	Lesão por pressão		
27/09/2021	CE	FORTALEZA	HGF	UPP EM SACRA	Enviada	Eventos Adversos Relacionados à	Lesão por pressão		
27/09/2021	CE	FORTALEZA	HGF	LPP EM REGIÃO SACRA ESTÁGIO	Enviada	Eventos Adversos Relacionados à	Lesão por pressão		
27/09/2021	CE	FORTALEZA	HGF	UPP EM SACRA	Enviada	Eventos Adversos Relacionados à	Lesão por pressão		
27/09/2021	CE	FORTALEZA	HGF	LPP EM REGIÃO SACRA ESTÁGIO	Enviada	Eventos Adversos Relacionados à	Lesão por pressão		
27/09/2021	CE	FORTALEZA	HGF	LPP EM REGIÃO OCCIPITAL	Enviada	Eventos Adversos Relacionados à	Lesão por pressão		
27/09/2021	CE	FORTALEZA	HGF	LPP ESTÁGIO 2 EM CALCÂN	Enviada	Eventos Adversos Relacionados à	Lesão por pressão		
27/09/2021	CE	FORTALEZA	HGF	UPP EM GLUTEO ESQUERDO	Enviada	Eventos Adversos Relacionados à	Lesão por pressão		
27/09/2021	CE	FORTALEZA	HGF	LPP EM REGIÃO SACRA ESTÁGIO	Enviada	Eventos Adversos Relacionados à	Lesão por pressão		
27/09/2021	CE	FORTALEZA	HGF	UPP EM SACRA	Enviada	Eventos Adversos Relacionados à	Lesão por pressão		
27/09/2021	CE	FORTALEZA	HGF	LPP EM REGIÃO SACRA ESTÁGIO	Enviada	Eventos Adversos Relacionados à	Lesão por pressão		

27/09/2021	CE	FORTALEZA	HGF	UPP EM SACRA	Enviada	Eventos Adversos Relacionados à	Lesão por pressão		
27/09/2021	CE	FORTALEZA	HGF	UPP EM SACRA 2	Enviada	Eventos Adversos Relacionados à	Lesão por pressão		
27/09/2021	CE	FORTALEZA	HGF	UPP EM GLUTEO	Enviada	Eventos Adversos Relacionados à	Lesão por pressão		
27/09/2021	CE	FORTALEZA	HGF	LPP SACRA ESTÁGIO 1	Enviada	Eventos Adversos Relacionados à	Lesão por pressão		
27/09/2021	CE	FORTALEZA	HGF	UPP EM TROCANTER ESQUER	Enviada	Eventos Adversos Relacionados à	Lesão por pressão		
27/09/2021	CE	FORTALEZA	HGF	LPP SACRA ESTÁGIO 2	Enviada	Eventos Adversos Relacionados à	Lesão por pressão		
27/09/2021	CE	FORTALEZA	HGF	LPP EM REGIÃO SACRA ESTÁGIO	Enviada	Eventos Adversos Relacionados à	Lesão por pressão		
27/09/2021	CE	FORTALEZA	HGF	UPP EM SACRA	Enviada	Eventos Adversos Relacionados à	Lesão por pressão		
27/09/2021	CE	FORTALEZA	HGF	UPP EM SACRA	Enviada	Eventos Adversos Relacionados à	Lesão por pressão		
27/09/2021	CE	FORTALEZA	HGF	LPP EM DORSO ESTÁGIO 2	Enviada	Eventos Adversos Relacionados à	Lesão por pressão		
27/09/2021	CE	FORTALEZA	HGF	UPP EM SACRA	Enviada	Eventos Adversos Relacionados à	Lesão por pressão		
27/09/2021	CE	FORTALEZA	HGF	UPP EM SACRA	Enviada	Eventos Adversos Relacionados à	Lesão por pressão		
27/09/2021	CE	FORTALEZA	HGF	UPP EM TROCANTER DIREITO	Enviada	Eventos Adversos Relacionados à	Lesão por pressão		
27/09/2021	CE	FORTALEZA	HGF	UPP EM DORSO	Enviada	Eventos Adversos Relacionados à	Lesão por pressão		
27/09/2021	CE	FORTALEZA	HGF	UPP EM SACRA	Enviada	Eventos Adversos Relacionados à	Lesão por pressão		

27/09/2021	CE	FORTALEZA	HGF	UPP EM SACRA	Concluída	Eventos Adversos Relacionados à	Lesão por pressão		
27/09/2021	CE	FORTALEZA	HGF	UPP EM FLANCO DIREITO	Enviada	Eventos Adversos Relacionados à	Lesão por pressão		
27/09/2021	CE	FORTALEZA	HGF	UPP EM SACRA	Enviada	Eventos Adversos Relacionados à	Lesão por pressão		
27/09/2021	CE	FORTALEZA	HGF	UPP EM SACRA 2	Enviada	Eventos Adversos Relacionados à	Lesão por pressão		
27/09/2021	CE	FORTALEZA	HGF	UPP EM DORSO	Enviada	Eventos Adversos Relacionados à	Lesão por pressão		
27/09/2021	CE	FORTALEZA	HGF	UPP EM SACRA	Enviada	Eventos Adversos Relacionados à	Lesão por pressão		
27/09/2021	CE	FORTALEZA	HGF	UPP EM CALCANEO DIREITO	Concluída	Eventos Adversos Relacionados à	Lesão por pressão		
27/09/2021	CE	FORTALEZA	HGF	UPP EM SACRA	Enviada	Eventos Adversos Relacionados à	Lesão por pressão		
27/09/2021	CE	FORTALEZA	HGF	UPP EM SACRA	Enviada	Eventos Adversos Relacionados à	Lesão por pressão		
27/09/2021	CE	FORTALEZA	HGF	UPP EM GLUTEOS	Enviada	Eventos Adversos Relacionados à	Lesão por pressão		
27/09/2021	CE	FORTALEZA	HGF	UPP EM SACRA	Enviada	Eventos Adversos Relacionados à	Lesão por pressão		
27/09/2021	CE	FORTALEZA	HGF	LPP EM REGIÃO SACRA ESTÁGIO	Enviada	Eventos Adversos Relacionados à	Lesão por pressão		
27/09/2021	CE	FORTALEZA	HGF	LPP REGIÃO SACRA ESTÁGIO	Enviada	Eventos Adversos Relacionados à	Lesão por pressão		
27/09/2021	CE	FORTALEZA	HGF	LPP EM SACRA ESTÁGIO 2	Enviada	Eventos Adversos Relacionados à	Lesão por pressão		
27/09/2021	CE	FORTALEZA	HGF	LPP EM SACRA ESTÁGIO 2	Enviada	Eventos Adversos Relacionados à	Lesão por pressão		

27/09/2021	CE	FORTALE ZA	HGF	LPP EM REGIÃO SACRA ESTÁGIO	Enviada	Eventos Adversos Relaciona dos à	Lesão por pressão		
27/09/2021	CE	FORTALE ZA	HGF	LPP EM REGIÃO SACRA ESTÁGIO	Enviada	Eventos Adversos Relaciona dos à	Lesão por pressão		
27/09/2021	CE	FORTALE ZA	HGF	-	Enviada	Eventos Adversos Relaciona dos à	Lesão por pressão		
27/09/2021	CE	FORTALE ZA	HGF	LPP EM ISQUIOS ESTÁGIO 2	Enviada	Eventos Adversos Relaciona dos à	Lesão por pressão		
27/09/2021	CE	FORTALE ZA	HGF	LPP EM CALCÂN EO DIREITO	Enviada	Eventos Adversos Relaciona dos à	Lesão por pressão		
27/09/2021	CE	FORTALE ZA	HGF	LPP ESTÁGIO 2 EM MALÉOL	Enviada	Eventos Adversos Relaciona dos à	Lesão por pressão		
27/09/2021	CE	FORTALE ZA	HGF	LPP SACRA ESTÁGIO 1	Enviada	Eventos Adversos Relaciona dos à	Lesão por pressão		
27/09/2021	CE	FORTALE ZA	HGF	LESÃO EM REGIÃO SACRA	Concluída	Eventos Adversos Relaciona dos à	Lesão por pressão		
27/09/2021	CE	FORTALE ZA	HGF	LPP SACRA ESTÁGIO 2	Enviada	Eventos Adversos Relaciona dos à	Lesão por pressão		
27/09/2021	CE	FORTALE ZA	HGF	-	Enviada	Eventos Adversos Relaciona dos à	Lesão por pressão		
27/09/2021	CE	FORTALE ZA	HGF	LPP EM ESCÁPUL A ESQUER	Enviada	Eventos Adversos Relaciona dos à	Lesão por pressão		
27/09/2021	CE	FORTALE ZA	HGF	LPP EM REGIÃO SACRA ESTÁGIO	Enviada	Eventos Adversos Relaciona dos à	Lesão por pressão		
27/09/2021	CE	FORTALE ZA	HGF	LPP SACRA ESTÁGIO 2	Enviada	Eventos Adversos Relaciona dos à	Lesão por pressão		
27/09/2021	CE	FORTALE ZA	HGF	LPP EM GLÚTEO DIREITO ESTÁGIO	Enviada	Eventos Adversos Relaciona dos à	Lesão por pressão		
27/09/2021	CE	FORTALE ZA	HGF	LPP ESTÁGIO 2 EM REGIÃO	Enviada	Eventos Adversos Relaciona dos à	Lesão por pressão		

27/09/2021	CE	FORTALEZA	HGF	LPP EM CALCÂNEO ESQUER	Enviada	Eventos Adversos Relacionados à	Lesão por pressão		
27/09/2021	CE	FORTALEZA	HGF	LPP SACRA ESTÁGIO 2	Enviada	Eventos Adversos Relacionados à	Lesão por pressão		
27/09/2021	CE	FORTALEZA	HGF	LPP EM REGIÃO SACRA ESTÁGIO	Enviada	Eventos Adversos Relacionados à	Lesão por pressão		
27/09/2021	CE	FORTALEZA	HGF	LPP SACRA ESTÁGIO 2	Enviada	Eventos Adversos Relacionados à	Lesão por pressão		
27/09/2021	CE	FORTALEZA	HGF	LPP SACRA ESTÁGIO 2;	Enviada	Eventos Adversos Relacionados à	Lesão por pressão		
27/09/2021	CE	FORTALEZA	HGF	LPP EM SACRA ESTÁGIO 1	Enviada	Eventos Adversos Relacionados à	Lesão por pressão		
27/09/2021	CE	FORTALEZA	HGF	LPP EM REGIÃO SACRA ESTÁGIO	Enviada	Eventos Adversos Relacionados à	Lesão por pressão		
27/09/2021	CE	FORTALEZA	HGF	LPP EM GLÚTEO DIREITO ESTÁGIO	Enviada	Eventos Adversos Relacionados à	Lesão por pressão		
27/09/2021	CE	FORTALEZA	HGF	TROCANTER ESQUERDO	Enviada	Eventos Adversos Relacionados à	Lesão por pressão		
27/09/2021	CE	FORTALEZA	HGF	LPP SACRA ESTÁGIO 1	Enviada	Eventos Adversos Relacionados à	Lesão por pressão		
27/09/2021	CE	FORTALEZA	HGF	LPP ESTÁGIO 1 NA REGIÃO	Enviada	Eventos Adversos Relacionados à	Lesão por pressão		
27/09/2021	CE	FORTALEZA	HGF	LPP EM REGIÃO SACRA ESTÁGIO	Enviada	Eventos Adversos Relacionados à	Lesão por pressão		
27/09/2021	CE	FORTALEZA	HGF	LPP EM SACRA ESTÁGIO 2	Enviada	Eventos Adversos Relacionados à	Lesão por pressão		
27/09/2021	CE	FORTALEZA	HGF	LPP EM TROCANTER DIREITO	Enviada	Eventos Adversos Relacionados à	Lesão por pressão		
27/09/2021	CE	FORTALEZA	HGF	LPP EM SACRA ESTÁGIO 2	Enviada	Eventos Adversos Relacionados à	Lesão por pressão		

27/09/2021	CE	FORTALEZA	HGF	LPP SACRA ESTÁGIO 1	Enviada	Eventos Adversos Relacionados à	Lesão por pressão		
27/09/2021	CE	FORTALEZA	HGF	upp em lombar	Enviada	Eventos Adversos Relacionados à	Lesão por pressão		
27/09/2021	CE	FORTALEZA	HGF	UPP EM SACRA	Enviada	Eventos Adversos Relacionados à	Lesão por pressão		
27/09/2021	CE	FORTALEZA	HGF	UPP EM CALCANEOS	Enviada	Eventos Adversos Relacionados à	Lesão por pressão		
27/09/2021	CE	FORTALEZA	HGF	UPP EM SACRA	Enviada	Eventos Adversos Relacionados à	Lesão por pressão		
27/09/2021	CE	FORTALEZA	HGF	LESÃO EM DORSO ESTÁGIO	Enviada	Eventos Adversos Relacionados à	Lesão por pressão		
27/09/2021	CE	FORTALEZA	HGF	UPP EM SACRA	Enviada	Eventos Adversos Relacionados à	Lesão por pressão		
27/09/2021	CE	FORTALEZA	HGF	LPP ESTÁGIO 1 EM SACRA	Enviada	Eventos Adversos Relacionados à	Lesão por pressão		
27/09/2021	CE	FORTALEZA	HGF	UPP EM SACRA	Enviada	Eventos Adversos Relacionados à	Lesão por pressão		
27/09/2021	CE	FORTALEZA	HGF	LPP EM TROCANTER ESQUER	Enviada	Eventos Adversos Relacionados à	Lesão por pressão		
27/09/2021	CE	FORTALEZA	HGF	LPP EM GLÚTEOS ESTÁGIO	Enviada	Eventos Adversos Relacionados à	Lesão por pressão		
27/09/2021	CE	FORTALEZA	HGF	LPP ESTÁGIO 2 EM SACRA	Enviada	Eventos Adversos Relacionados à	Lesão por pressão		
27/09/2021	CE	FORTALEZA	HGF	SACRA ESTÁGIO 1	Enviada	Eventos Adversos Relacionados à	Lesão por pressão		
27/09/2021	CE	FORTALEZA	HGF	LPP SACRA ESTÁGIO 2	Enviada	Eventos Adversos Relacionados à	Lesão por pressão		
27/09/2021	CE	FORTALEZA	HGF	-	Enviada	Eventos Adversos Relacionados à	Lesão por pressão		

27/09/2021	CE	FORTALEZA	HGF	LPP EM REGIÃO SACRA ESTÁGIO	Enviada	Eventos Adversos Relacionados à	Lesão por pressão		
27/09/2021	CE	FORTALEZA	HGF	UPP EM JOELHO	Concluída	Eventos Adversos Relacionados à	Lesão por pressão		
27/09/2021	CE	FORTALEZA	HGF	LPP SACRA ESTÁGIO 1	Enviada	Eventos Adversos Relacionados à	Lesão por pressão		
27/09/2021	CE	FORTALEZA	HGF	UPP EM JOELHO	Concluída	Eventos Adversos Relacionados à	Lesão por pressão		
27/09/2021	CE	FORTALEZA	HGF	LPP ESTÁGIO 4 EM REGIÃO	Concluída	Eventos Adversos Relacionados à	Lesão por pressão		
27/09/2021	CE	FORTALEZA	HGF	-	Enviada	Eventos Adversos Relacionados à	Lesão por pressão		
27/09/2021	CE	FORTALEZA	HGF	UPP EM SACRA	Concluída	Eventos Adversos Relacionados à	Lesão por pressão		
27/09/2021	CE	FORTALEZA	HGF	UPP EM MUSCULO GRÁCIL	Concluída	Eventos Adversos Relacionados à	Lesão por pressão		
27/09/2021	CE	FORTALEZA	HGF	LPP ESTÁGIO 2 EM SACRA	Enviada	Eventos Adversos Relacionados à	Lesão por pressão		
27/09/2021	CE	FORTALEZA	HGF	UPP EM SACRA	Enviada	Eventos Adversos Relacionados à	Lesão por pressão		
27/09/2021	CE	FORTALEZA	HGF	UPP EM SACRA	Enviada	Eventos Adversos Relacionados à	Lesão por pressão		
27/09/2021	CE	FORTALEZA	HGF	UPP EM SACRA	Enviada	Eventos Adversos Relacionados à	Lesão por pressão		
27/09/2021	CE	FORTALEZA	HGF	UPP EM MALEOLOS	Concluída	Eventos Adversos Relacionados à	Lesão por pressão		
27/09/2021	CE	FORTALEZA	HGF	UPP EM MEBRO INFERIOR	Enviada	Eventos Adversos Relacionados à	Lesão por pressão		
27/09/2021	CE	FORTALEZA	HGF	LPP ESTÁGIO 2 EM SACRA	Enviada	Eventos Adversos Relacionados à	Lesão por pressão		

27/09/2021	CE	FORTALEZA	HGF	UPP EM SACRA	Enviada	Eventos Adversos Relacionados à	Lesão por pressão		
27/09/2021	CE	FORTALEZA	HGF	LPP SACRA ESTÁGIO 1	Enviada	Eventos Adversos Relacionados à	Lesão por pressão		
27/09/2021	CE	FORTALEZA	HGF	UPP EM TROCANTER ESQUER	Enviada	Eventos Adversos Relacionados à	Lesão por pressão		
27/09/2021	CE	FORTALEZA	HGF	UPP EM SACRA	Enviada	Eventos Adversos Relacionados à	Lesão por pressão		
27/09/2021	CE	FORTALEZA	HGF	LPP ESTÁGIO 2 EM REGIÃO	Enviada	Eventos Adversos Relacionados à	Lesão por pressão		
27/09/2021	CE	FORTALEZA	HGF	LPP EM GLÚTEOS ESTÁGIO	Enviada	Eventos Adversos Relacionados à	Lesão por pressão		
27/09/2021	CE	FORTALEZA	HGF	UPP EM SACRA	Concluída	Eventos Adversos Relacionados à	Lesão por pressão		
27/09/2021	CE	FORTALEZA	HGF	LPP EM SACRA ESTÁGIO 2	Enviada	Eventos Adversos Relacionados à	Lesão por pressão		
27/09/2021	CE	FORTALEZA	HGF	LPP EM GLÚTEO ESQUERDO	Enviada	Eventos Adversos Relacionados à	Lesão por pressão		
27/09/2021	CE	FORTALEZA	HGF	UPP EM SACRA	Enviada	Eventos Adversos Relacionados à	Lesão por pressão		
27/09/2021	CE	FORTALEZA	HGF	LPP EM SACRA ESTÁGIO 2	Enviada	Eventos Adversos Relacionados à	Lesão por pressão		
27/09/2021	CE	FORTALEZA	HGF	LPP ESTÁGIO 1 EM SACRA	Enviada	Eventos Adversos Relacionados à	Lesão por pressão		
27/09/2021	CE	FORTALEZA	HGF	LPP ESTÁGIO 1 EM SACRA	Enviada	Eventos Adversos Relacionados à	Lesão por pressão		
27/09/2021	CE	FORTALEZA	HGF	UPP EM SACRA	Enviada	Eventos Adversos Relacionados à	Lesão por pressão		
27/09/2021	CE	FORTALEZA	HGF	LPP ESTÁGIO 2 EM REGIÃO	Enviada	Eventos Adversos Relacionados à	Lesão por pressão		

27/09/2021	CE	FORTALEZA	HGF	UPP EM SACRA	Enviada	Eventos Adversos Relacionados à	Lesão por pressão		
27/09/2021	CE	FORTALEZA	HGF	UPP EM DORSO	Enviada	Eventos Adversos Relacionados à	Lesão por pressão		
27/09/2021	CE	FORTALEZA	HGF	LPP EM OUTRAS PARTES ESTÁGIO	Enviada	Eventos Adversos Relacionados à	Lesão por pressão		
27/09/2021	CE	FORTALEZA	HGF	UPP EM CALCANEOS	Enviada	Eventos Adversos Relacionados à	Lesão por pressão		
27/09/2021	CE	FORTALEZA	HGF	LPP EM CALCÂNEO DIREITO	Enviada	Eventos Adversos Relacionados à	Lesão por pressão		
27/09/2021	CE	FORTALEZA	HGF	UPP EM SACRA	Enviada	Eventos Adversos Relacionados à	Lesão por pressão		
27/09/2021	CE	FORTALEZA	HGF	LPP EM REGIÃO SACRA ESTÁGIO	Enviada	Eventos Adversos Relacionados à	Lesão por pressão		
27/09/2021	CE	FORTALEZA	HGF	UPP EM SACRA	Concluída	Eventos Adversos Relacionados à	Lesão por pressão		
27/09/2021	CE	FORTALEZA	HGF	UPP EM SACRA	Enviada	Eventos Adversos Relacionados à	Lesão por pressão		
27/09/2021	CE	FORTALEZA	HGF	LPP EM REGIÃO SACRA ESTÁGIO	Enviada	Eventos Adversos Relacionados à	Lesão por pressão		
27/09/2021	CE	FORTALEZA	HGF	UPP EM SACRA	Concluída	Eventos Adversos Relacionados à	Lesão por pressão		
27/09/2021	CE	FORTALEZA	HGF	UPP EM SACRA ESTÁGIO 2	Enviada	Eventos Adversos Relacionados à	Lesão por pressão		
27/09/2021	CE	FORTALEZA	HGF	UPP EM SACRA ESTÁGIO 1	Enviada	Eventos Adversos Relacionados à	Lesão por pressão		
27/09/2021	CE	FORTALEZA	HGF	HEMATURIA NÃO ESPECÍFICADA	Enviada	Eventos Adversos Relacionados à	Lesão por pressão		
27/09/2021	CE	FORTALEZA	HGF	UPP EM SACRA ESTÁGIO 2	Enviada	Eventos Adversos Relacionados à	Lesão por pressão		

27/09/2021	CE	FORTALE ZA	HGF	UPP EM SACRA ESTÁGIO 2	Enviada	Eventos Adversos Relaciona dos à	Lesão por pressão		
27/09/2021	CE	FORTALE ZA	HGF	UPP EM SACRA ESTÁGIO 1	Enviada	Eventos Adversos Relaciona dos à	Lesão por pressão		
27/09/2021	CE	FORTALE ZA	HGF	UPP EM SACRA ESTÁGIO 2	Enviada	Eventos Adversos Relaciona dos à	Lesão por pressão		
28/09/2021	CE	FORTALE ZA	HGF	LPP EM SACRA ESTÁGIO 2	Enviada	Eventos Adversos Relaciona dos à	Lesão por pressão		
28/09/2021	CE	FORTALE ZA	HGF	LPP EM SACRA ESTÁGIO 1	Enviada	Eventos Adversos Relaciona dos à	Lesão por pressão		
28/09/2021	CE	FORTALE ZA	HGF	LPP EM SACRA ESTÁGIO 1	Enviada	Eventos Adversos Relaciona dos à	Lesão por pressão		
28/09/2021	CE	FORTALE ZA	HGF	LPP EM MALEOL O DIREITO	Enviada	Eventos Adversos Relaciona dos à	Lesão por pressão		
28/09/2021	CE	FORTALE ZA	HGF	LPP EM BRAÇO ESQUER DO	Enviada	Eventos Adversos Relaciona dos à	Lesão por pressão		
28/09/2021	CE	FORTALE ZA	HGF	LPP EM SACRA ESTÁGIO 2	Enviada	Eventos Adversos Relaciona dos à	Lesão por pressão		
28/09/2021	CE	FORTALE ZA	HGF	LPP EM CALCÂN EO ESQUER	Concluída	Eventos Adversos Relaciona dos à	Lesão por pressão		
28/09/2021	CE	FORTALE ZA	HGF	LPP EM SACRA ESTÁGIO 2	Enviada	Eventos Adversos Relaciona dos à	Lesão por pressão		
28/09/2021	CE	FORTALE ZA	HGF	upp em sacra	Enviada	Eventos Adversos Relaciona dos à	Lesão por pressão		
28/09/2021	CE	FORTALE ZA	HGF	UPP DE CALCAN EOS	Enviada	Eventos Adversos Relaciona dos à	Lesão por pressão		
28/09/2021	CE	FORTALE ZA	HGF	UPP EM SACRA	Enviada	Eventos Adversos Relaciona dos à	Lesão por pressão		
28/09/2021	CE	FORTALE ZA	HGF	UPP EM SACRA	Enviada	Eventos Adversos Relaciona dos à	Lesão por pressão		

28/09/2021	CE	FORTALE ZA	HGF	UPP EM MMII	Enviada	Eventos Adversos Relaciona dos à	Lesão por pressão		
28/09/2021	CE	FORTALE ZA	HGF	LPP EM SACRA ESTÁGIO 2	Enviada	Eventos Adversos Relaciona dos à	Lesão por pressão		
28/09/2021	CE	FORTALE ZA	HGF	UPP EM MMII	Enviada	Eventos Adversos Relaciona dos à	Lesão por pressão		
28/09/2021	CE	FORTALE ZA	HGF	UPP EM SACRA	Enviada	Eventos Adversos Relaciona dos à	Lesão por pressão		
28/09/2021	CE	FORTALE ZA	HGF	LPP EM SACRA ESTÁGIO 2	Enviada	Eventos Adversos Relaciona dos à	Lesão por pressão		
28/09/2021	CE	FORTALE ZA	HGF	UPP EM SACRA	Enviada	Eventos Adversos Relaciona dos à	Lesão por pressão		
28/09/2021	CE	FORTALE ZA	HGF	UPP EM SACRA	Enviada	Eventos Adversos Relaciona dos à	Lesão por pressão		
28/09/2021	CE	FORTALE ZA	HGF	UPP EM GLUTEO S	Enviada	Eventos Adversos Relaciona dos à	Lesão por pressão		
28/09/2021	CE	FORTALE ZA	HGF	LPP EM SACRA ESTÁGIO 1	Enviada	Eventos Adversos Relaciona dos à	Lesão por pressão		
28/09/2021	CE	FORTALE ZA	HGF	UPP EM SACRA	Enviada	Eventos Adversos Relaciona dos à	Lesão por pressão		
28/09/2021	CE	FORTALE ZA	HGF	UPP EM SACRA	Enviada	Eventos Adversos Relaciona dos à	Lesão por pressão		
28/09/2021	CE	FORTALE ZA	HGF	LPP EM SACRA ESTÁGIO 2	Enviada	Eventos Adversos Relaciona dos à	Lesão por pressão		
28/09/2021	CE	FORTALE ZA	HGF	UPP EM DORSO	Enviada	Eventos Adversos Relaciona dos à	Lesão por pressão		
28/09/2021	CE	FORTALE ZA	HGF	UPP EM GLUTEO S	Concluída	Eventos Adversos Relaciona dos à	Lesão por pressão		
28/09/2021	CE	FORTALE ZA	HGF	LPP EM CALCÂN EO ESTÁGIO	Enviada	Eventos Adversos Relaciona dos à	Lesão por pressão		

28/09/2021	CE	FORTALEZA	HGF	UPP EM TROCANTERES	Enviada	Eventos Adversos Relacionados à	Lesão por pressão		
28/09/2021	CE	FORTALEZA	HGF	LPP EM SACRA ESTÁGIO 2	Enviada	Eventos Adversos Relacionados à	Lesão por pressão		
28/09/2021	CE	FORTALEZA	HGF	UPP EM SACRA	Enviada	Eventos Adversos Relacionados à	Lesão por pressão		
28/09/2021	CE	FORTALEZA	HGF	LPP EM DORSO ESTÁGIO 2	Enviada	Eventos Adversos Relacionados à	Lesão por pressão		
28/09/2021	CE	FORTALEZA	HGF	UPP EM GLUTEO DIREITO	Enviada	Eventos Adversos Relacionados à	Lesão por pressão		
28/09/2021	CE	FORTALEZA	HGF	LPP EM MALÉOLOS ESTÁGIO	Enviada	Eventos Adversos Relacionados à	Lesão por pressão		
28/09/2021	CE	FORTALEZA	HGF	UPP EM SACRA	Enviada	Eventos Adversos Relacionados à	Lesão por pressão		
28/09/2021	CE	FORTALEZA	HGF	LPP EM SACRA ESTÁGIO 2	Enviada	Eventos Adversos Relacionados à	Lesão por pressão		
28/09/2021	CE	FORTALEZA	HGF	UPP EM SACRA	Enviada	Eventos Adversos Relacionados à	Lesão por pressão		
28/09/2021	CE	FORTALEZA	HGF	UPP EM SACRA	Concluída	Eventos Adversos Relacionados à	Lesão por pressão		
28/09/2021	CE	FORTALEZA	HGF	UPP EM SACRA	Concluída	Eventos Adversos Relacionados à	Lesão por pressão		
28/09/2021	CE	FORTALEZA	HGF	UPP EM SACRA	Concluída	Eventos Adversos Relacionados à	Lesão por pressão		
28/09/2021	CE	FORTALEZA	HGF	UPP EM SACRA	Concluída	Eventos Adversos Relacionados à	Lesão por pressão		
28/09/2021	CE	FORTALEZA	HGF	UPP EM SACRA	Enviada	Eventos Adversos Relacionados à	Lesão por pressão		
28/09/2021	CE	FORTALEZA	HGF	UPP EM SACRA	Enviada	Eventos Adversos Relacionados à	Lesão por pressão		

28/09/2021	CE	FORTALE ZA	HGF	UPP EM SACRA	Enviada	Eventos Adversos Relaciona dos à	Lesão por pressão		
28/09/2021	CE	FORTALE ZA	HGF	UPP EM SACRA	Enviada	Eventos Adversos Relaciona dos à	Lesão por pressão		
28/09/2021	CE	FORTALE ZA	HGF	UPP EM SACRA	Enviada	Eventos Adversos Relaciona dos à	Lesão por pressão		
28/09/2021	CE	FORTALE ZA	HGF	UPP EM SACRA	Concluída	Eventos Adversos Relaciona dos à	Lesão por pressão		
28/09/2021	CE	FORTALE ZA	HGF	UPP EM SACRA	Concluída	Eventos Adversos Relaciona dos à	Lesão por pressão		
28/09/2021	CE	FORTALE ZA	HGF	UPP EM SACRA	Enviada	Eventos Adversos Relaciona dos à	Lesão por pressão		
28/09/2021	CE	FORTALE ZA	HGF	UPP EM CALCAN EO	Enviada	Eventos Adversos Relaciona dos à	Lesão por pressão		
28/09/2021	CE	FORTALE ZA	HGF	UPP EM SACRA	Enviada	Eventos Adversos Relaciona dos à	Lesão por pressão		
28/09/2021	CE	FORTALE ZA	HGF	UPP CALCAN EOS	Enviada	Eventos Adversos Relaciona dos à	Lesão por pressão		
28/09/2021	CE	FORTALE ZA	HGF	UPP EM SACRA	Enviada	Eventos Adversos Relaciona dos à	Lesão por pressão		
28/09/2021	CE	FORTALE ZA	HGF	UPP EM SACRA	Enviada	Eventos Adversos Relaciona dos à	Lesão por pressão		
28/09/2021	CE	FORTALE ZA	HGF	UPP EM SACRA	Enviada	Eventos Adversos Relaciona dos à	Lesão por pressão		
28/09/2021	CE	FORTALE ZA	HGF	UPP EM MMII	Enviada	Eventos Adversos Relaciona dos à	Lesão por pressão		
28/09/2021	CE	FORTALE ZA	HGF	UPP CALCAN EOS	Enviada	Eventos Adversos Relaciona dos à	Lesão por pressão		
28/09/2021	CE	FORTALE ZA	HGF	UPP EM OCCIPTO	Concluída	Eventos Adversos Relaciona dos à	Lesão por pressão		

28/09/2021	CE	FORTALEZA	HGF	UPP EM SACRA	Enviada	Eventos Adversos Relacionados à	Lesão por pressão		
28/09/2021	CE	FORTALEZA	HGF	UPP EM DORSO	Enviada	Eventos Adversos Relacionados à	Lesão por pressão		
28/09/2021	CE	FORTALEZA	HGF	LESÃO ARTERIAL NO MSE	Enviada	Eventos Adversos Relacionados à	Falhas durante a assistência à saúde		
28/09/2021	CE	FORTALEZA	HGF	UPP EM SACRA	Enviada	Eventos Adversos Relacionados à	Lesão por pressão		
28/09/2021	CE	FORTALEZA	HGF	UPP EM REGIÃO TIBIAL ESQUER	Enviada	Eventos Adversos Relacionados à	Lesão por pressão		
28/09/2021	CE	FORTALEZA	HGF	UPP EM PANTURRILHA DIREITA	Enviada	Eventos Adversos Relacionados à	Lesão por pressão		
28/09/2021	CE	FORTALEZA	HGF	UPP EM SACRA	Enviada	Eventos Adversos Relacionados à	Lesão por pressão		
28/09/2021	CE	FORTALEZA	HGF	Prescriçao entregue errada a	Enviada	Eventos Adversos Relacionados à	Falhas durante a assistência à saúde		
28/09/2021	CE	FORTALEZA	HGF	PERDA ACIDENTAL DE SNG	Enviada	Eventos Adversos Relacionados à	Falhas envolvendo sondas		
28/09/2021	CE	FORTALEZA	HGF	MEDICAMENTO PRESCRITO EM	Enviada	Eventos Adversos Relacionados à	Falhas durante a assistência à saúde		
28/09/2021	CE	FORTALEZA	HGF	APRESENTANDO ENRIGECIMENTO	Enviada	Eventos Adversos Relacionados à	Falhas envolvendo cateter venoso		
28/09/2021	CE	FORTALEZA	HGF	PACIENTE TROPEÇOU EM	Enviada	Eventos Adversos Relacionados à	Queda do paciente		
28/09/2021	CE	FORTALEZA	HGF	UPP EM SACRA	Enviada	Eventos Adversos Relacionados à	Lesão por pressão		
28/09/2021	CE	FORTALEZA	HGF	PRESENÇA DE HIPEREMIA	Enviada	Eventos Adversos Relacionados à	Falhas envolvendo cateter venoso		
28/09/2021	CE	FORTALEZA	HGF	APRESENTANDO HIPEREMIA	Enviada	Eventos Adversos Relacionados à	Falhas envolvendo cateter venoso		

28/09/2021	CE	FORTALEZA	HGF	FLEBITE EM MSD	Enviada	Eventos Adversos Relacionados à	Falhas envolvendo cateter venoso		
28/09/2021	CE	FORTALEZA	HGF	UPP EM DORSO	Concluída	Eventos Adversos Relacionados à	Lesão por pressão		
28/09/2021	CE	FORTALEZA	HGF	UPP EM PANTURRILHA	Concluída	Eventos Adversos Relacionados à	Lesão por pressão		
28/09/2021	CE	FORTALEZA	HGF	Flebite msd	Enviada	Eventos Adversos Relacionados à	Falhas envolvendo cateter venoso		
28/09/2021	CE	FORTALEZA	HGF	edema +hiperemia+ calor em MSE	Enviada	Eventos Adversos Relacionados à	Falhas envolvendo cateter venoso		
28/09/2021	CE	FORTALEZA	HGF	FLEBITE MSE	Enviada	Eventos Adversos Relacionados à	Falhas envolvendo cateter venoso		
28/09/2021	CE	FORTALEZA	HGF	Flebite msd	Enviada	Eventos Adversos Relacionados à	Falhas envolvendo cateter venoso		
28/09/2021	CE	FORTALEZA	HGF	FLEBITE MSD	Enviada	Eventos Adversos Relacionados à	Falhas envolvendo cateter venoso		
28/09/2021	CE	FORTALEZA	HGF	FLEBITE MSD	Enviada	Eventos Adversos Relacionados à	Falhas envolvendo cateter venoso		
28/09/2021	CE	FORTALEZA	HGF	MAQUEIRO FOI MUDAR PACIENT	Enviada	Eventos Adversos Relacionados à	Queda do paciente		
28/09/2021	CE	FORTALEZA	HGF	HEMATOMA MSD COM FLEBITE	Enviada	Eventos Adversos Relacionados à	Falhas envolvendo cateter venoso		
28/09/2021	CE	FORTALEZA	HGF	FLEBITE MSE	Enviada	Eventos Adversos Relacionados à	Falhas envolvendo cateter venoso		
28/09/2021	CE	FORTALEZA	HGF	APRESENTOU FLEBITE EM MSE	Enviada	Eventos Adversos Relacionados à	Falhas envolvendo cateter venoso		
28/09/2021	CE	FORTALEZA	HGF	-	Enviada	Eventos Adversos Relacionados à	Falhas envolvendo cateter venoso		
28/09/2021	CE	FORTALEZA	HGF	APRESENTOU FLEBITE EM	Enviada	Eventos Adversos Relacionados à	Falhas envolvendo cateter venoso		

28/09/2021	CE	FORTALEZA	HGF	PACIENTE ENCONTRAVA-	Enviada	Eventos Adversos Relacionados à	Queda do paciente		
28/09/2021	CE	FORTALEZA	HGF	PACIENTE APRESENTA-SE	Enviada	Eventos Adversos Relacionados à	Broncoaspiração		
28/09/2021	CE	FORTALEZA	HGF	PACIENTE FOI TOMAR BANHO	Enviada	Eventos Adversos Relacionados à	Queda do paciente		
28/09/2021	CE	FORTALEZA	HGF	APRESENTOU RUBOR, CALOR	Enviada	Eventos Adversos Relacionados à	Falhas envolvendo o cateter venoso		
04/10/2021	CE	FORTALEZA	HGF	Realizada cesariana às 8:15	Concluída	Eventos Adversos Relacionados à	Tromboembolismo venoso (TEV)		
04/10/2021	CE	FORTALEZA	HGF	Última hemodiálise dia 27.09	Enviada	Eventos Adversos Relacionados à	Falhas durante a assistência à saúde		
05/10/2021	CE	FORTALEZA	HGF	APRESENTOU FLEBITE EM MSD	Enviada	Eventos Adversos Relacionados à	Falhas envolvendo o cateter venoso		
14/10/2021	CE	FORTALEZA	HGF	FLEBITE EM MSD	Enviada	Eventos Adversos Relacionados à	Falhas envolvendo o cateter venoso		
21/10/2021	CE	FORTALEZA	HGF	FLEBITE MSD	Enviada	Eventos Adversos Relacionados à	Falhas envolvendo o cateter venoso		
21/10/2021	CE	FORTALEZA	HGF	Troca de administração da dieta	Enviada	Eventos Adversos Relacionados à	Falhas na administração de dietas		Enteral (oral, cateter oral ou
25/10/2021	CE	FORTALEZA	HGF	trocanter direito	Enviada	Eventos Adversos Relacionados à	Lesão por pressão		
26/10/2021	CE	FORTALEZA	HGF	Troca de bolsa de sangue. Paciente	Enviada	Eventos Adversos Relacionados à	Falha na identificação do paciente		
29/10/2021	CE	FORTALEZA	HGF	LPP EM MALÉOLO ESQUER	Enviada	Eventos Adversos Relacionados à	Lesão por pressão		
29/10/2021	CE	FORTALEZA	HGF	LPP EM SACRA ESTÁGIO 2	Enviada	Eventos Adversos Relacionados à	Lesão por pressão		
29/10/2021	CE	FORTALEZA	HGF	LPP SACRA ESTÁGIO 1	Enviada	Eventos Adversos Relacionados à	Lesão por pressão		

29/10/2021	CE	FORTALEZA	HGF	LPP SACRA ESTÁGIO 1	Enviada	Eventos Adversos Relacionados à	Lesão por pressão		
29/10/2021	CE	FORTALEZA	HGF	LPP EM CALCÂNEO	Enviada	Eventos Adversos Relacionados à	Lesão por pressão		
29/10/2021	CE	FORTALEZA	HGF	LPP EM SACRA ESTÁGIO	Enviada	Eventos Adversos Relacionados à	Lesão por pressão		
29/10/2021	CE	FORTALEZA	HGF	-	Enviada	Eventos Adversos Relacionados à	Lesão por pressão		
29/10/2021	CE	FORTALEZA	HGF	LPP EM SACRA ESTÁGIO 1	Enviada	Eventos Adversos Relacionados à	Lesão por pressão		
29/10/2021	CE	FORTALEZA	HGF	LPP SACRA ESTÁGIO 2	Enviada	Eventos Adversos Relacionados à	Lesão por pressão		
29/10/2021	CE	FORTALEZA	HGF	LPP SACRA ESTÁGIO 2	Enviada	Eventos Adversos Relacionados à	Lesão por pressão		
29/10/2021	CE	FORTALEZA	HGF	LPP SACRA ESTÁGIO 1	Enviada	Eventos Adversos Relacionados à	Lesão por pressão		
29/10/2021	CE	FORTALEZA	HGF	LPP SACRA ESTÁGIO 2	Enviada	Eventos Adversos Relacionados à	Lesão por pressão		
29/10/2021	CE	FORTALEZA	HGF	LPP SACRA ESTÁGIO 2	Enviada	Eventos Adversos Relacionados à	Lesão por pressão		
29/10/2021	CE	FORTALEZA	HGF	LPP SACRA ESTÁGIO 2	Enviada	Eventos Adversos Relacionados à	Lesão por pressão		
29/10/2021	CE	FORTALEZA	HGF	LPP SACRA ESTÁGIO 2	Enviada	Eventos Adversos Relacionados à	Lesão por pressão		
29/10/2021	CE	FORTALEZA	HGF	-	Enviada	Eventos Adversos Relacionados à	Lesão por pressão		
29/10/2021	CE	FORTALEZA	HGF	LPP SACRA ESTÁGIO 3	Concluída	Eventos Adversos Relacionados à	Lesão por pressão		
29/10/2021	CE	FORTALEZA	HGF	LPP SACRA ESTÁGIO 2	Enviada	Eventos Adversos Relacionados à	Lesão por pressão		

03/11/2021	CE	FORTALEZA	HGF	SACRA ESTÁGIO 2	Enviada	Eventos Adversos Relacionados à	Lesão por pressão		
03/11/2021	CE	FORTALEZA	HGF	-	Enviada	Eventos Adversos Relacionados à	Lesão por pressão		
04/11/2021	CE	FORTALEZA	HGF	LPP ESTÁGIO II EM GLÚTEO	Enviada	Eventos Adversos Relacionados à	Lesão por pressão		
04/11/2021	CE	FORTALEZA	HGF	LPP ESTÁGIO II EM CALCÂN	Enviada	Eventos Adversos Relacionados à	Lesão por pressão		
04/11/2021	CE	FORTALEZA	HGF	LPP ESTÁGIO II - TROCÂN	Enviada	Eventos Adversos Relacionados à	Lesão por pressão		
04/11/2021	CE	FORTALEZA	HGF	LPP ESTÁGIO II - DORSO	Enviada	Eventos Adversos Relacionados à	Lesão por pressão		
04/11/2021	CE	FORTALEZA	HGF	LPP SACRA	Enviada	Eventos Adversos Relacionados à	Lesão por pressão		
04/11/2021	CE	FORTALEZA	HGF	LPP SACRA	Enviada	Eventos Adversos Relacionados à	Lesão por pressão		
04/11/2021	CE	FORTALEZA	HGF	LPP SACRA	Concluída	Eventos Adversos Relacionados à	Lesão por pressão		
04/11/2021	CE	FORTALEZA	HGF	LPP SACRA	Enviada	Eventos Adversos Relacionados à	Lesão por pressão		
04/11/2021	CE	FORTALEZA	HGF	LPP SACRA	Enviada	Eventos Adversos Relacionados à	Lesão por pressão		
04/11/2021	CE	FORTALEZA	HGF	LPP GRAU III EM PANTUR	Concluída	Eventos Adversos Relacionados à	Lesão por pressão		
04/11/2021	CE	FORTALEZA	HGF	LPP SACRA	Enviada	Eventos Adversos Relacionados à	Lesão por pressão		
04/11/2021	CE	FORTALEZA	HGF	LPP SACRA	Enviada	Eventos Adversos Relacionados à	Lesão por pressão		
04/11/2021	CE	FORTALEZA	HGF	LPP EM GLÚTEO DIREITO	Enviada	Eventos Adversos Relacionados à	Lesão por pressão		

04/11/2021	CE	FORTALEZA	HGF	LPP ESTÁGIO II EM GLÚTEO	Enviada	Eventos Adversos Relacionados à	Lesão por pressão		
04/11/2021	CE	FORTALEZA	HGF	LPP SACRA	Enviada	Eventos Adversos Relacionados à	Lesão por pressão		
04/11/2021	CE	FORTALEZA	HGF	LPP GRAU III - INTERGL	Concluída	Eventos Adversos Relacionados à	Lesão por pressão		
04/11/2021	CE	FORTALEZA	HGF	LPP SACRA	Enviada	Eventos Adversos Relacionados à	Lesão por pressão		
04/11/2021	CE	FORTALEZA	HGF	LPP GRAU II - TÓRAX ANTERIO	Enviada	Eventos Adversos Relacionados à	Lesão por pressão		
04/11/2021	CE	FORTALEZA	HGF	LPP SACRA	Enviada	Eventos Adversos Relacionados à	Lesão por pressão		
04/11/2021	CE	FORTALEZA	HGF	CALCÂNEO DIREITO	Enviada	Eventos Adversos Relacionados à	Lesão por pressão		
04/11/2021	CE	FORTALEZA	HGF	LPP GRAU III - INTERGL	Concluída	Eventos Adversos Relacionados à	Lesão por pressão		
04/11/2021	CE	FORTALEZA	HGF	LPP SACRA	Enviada	Eventos Adversos Relacionados à	Lesão por pressão		
04/11/2021	CE	FORTALEZA	HGF	LPP SACRA	Enviada	Eventos Adversos Relacionados à	Lesão por pressão		
04/11/2021	CE	FORTALEZA	HGF	LPP GRAU II - REGIÃO SACRA	Enviada	Eventos Adversos Relacionados à	Lesão por pressão		
04/11/2021	CE	FORTALEZA	HGF	LPP SACRA	Enviada	Eventos Adversos Relacionados à	Lesão por pressão		
04/11/2021	CE	FORTALEZA	HGF	LPP SACRA	Enviada	Eventos Adversos Relacionados à	Lesão por pressão		
04/11/2021	CE	FORTALEZA	HGF	LPP GRAU I REGIÃO SACRA	Enviada	Eventos Adversos Relacionados à	Lesão por pressão		
04/11/2021	CE	FORTALEZA	HGF	LPP SACRA	Enviada	Eventos Adversos Relacionados à	Lesão por pressão		

04/11/2021	CE	FORTALE ZA	HGF	LPP SACRA	Concluída	Eventos Adversos Relaciona dos à	Lesão por pressão		
04/11/2021	CE	FORTALE ZA	HGF	LPP GRAU II - GLÚTEO DIREITO	Enviada	Eventos Adversos Relaciona dos à	Lesão por pressão		
04/11/2021	CE	FORTALE ZA	HGF	LPP SACRA	Concluída	Eventos Adversos Relaciona dos à	Lesão por pressão		
04/11/2021	CE	FORTALE ZA	HGF	LPP GRAU II - GLÚTEO A	Enviada	Eventos Adversos Relaciona dos à	Lesão por pressão		
04/11/2021	CE	FORTALE ZA	HGF	LPP SACRA	Concluída	Eventos Adversos Relaciona dos à	Lesão por pressão		
04/11/2021	CE	FORTALE ZA	HGF	LPP SACRA	Enviada	Eventos Adversos Relaciona dos à	Lesão por pressão		
04/11/2021	CE	FORTALE ZA	HGF	GLÚETO ESQUER DO	Enviada	Eventos Adversos Relaciona dos à	Lesão por pressão		
04/11/2021	CE	FORTALE ZA	HGF	LPP SACRA	Enviada	Eventos Adversos Relaciona dos à	Lesão por pressão		
04/11/2021	CE	FORTALE ZA	HGF	LPP GRAU II - GLUTEO À	Em retificação	Eventos Adversos Relaciona dos à	Lesão por pressão		
04/11/2021	CE	FORTALE ZA	HGF	LPP SACRA	Enviada	Eventos Adversos Relaciona dos à	Lesão por pressão		
04/11/2021	CE	FORTALE ZA	HGF	LPP GRAU III - SACRA	Concluída	Eventos Adversos Relaciona dos à	Lesão por pressão		
04/11/2021	CE	FORTALE ZA	HGF	LPP SACRA ESTÁGIO 4	Concluída	Eventos Adversos Relaciona dos à	Lesão por pressão		
04/11/2021	CE	FORTALE ZA	HGF	LLP GRAU II - REGIÃO SACRA	Enviada	Eventos Adversos Relaciona dos à	Lesão por pressão		
04/11/2021	CE	FORTALE ZA	HGF	LPP SACRA	Enviada	Eventos Adversos Relaciona dos à	Lesão por pressão		
04/11/2021	CE	FORTALE ZA	HGF	LPP GRAU II CALCÂN EO	Enviada	Eventos Adversos Relaciona dos à	Lesão por pressão		

04/11/2021	CE	FORTALEZA	HGF	LPP ESTÁGIO II - REGIÃO	Enviada	Eventos Adversos Relacionados à	Lesão por pressão		
04/11/2021	CE	FORTALEZA	HGF	LPP GRAU II - CALCÂNEO	Enviada	Eventos Adversos Relacionados à	Lesão por pressão		
04/11/2021	CE	FORTALEZA	HGF	LPP GRAU II - GLÚTEO ESQUER	Enviada	Eventos Adversos Relacionados à	Lesão por pressão		
04/11/2021	CE	FORTALEZA	HGF	LPP GRAU II - CALCÂNEO	Enviada	Eventos Adversos Relacionados à	Lesão por pressão		
04/11/2021	CE	FORTALEZA	HGF	LPP NAO ESPECIFICADO O LOCAL	Enviada	Eventos Adversos Relacionados à	Lesão por pressão		
04/11/2021	CE	FORTALEZA	HGF	LPP GRAU III - GLÚTEO	Concluída	Eventos Adversos Relacionados à	Lesão por pressão		
04/11/2021	CE	FORTALEZA	HGF	LPP GRAU II - GLÚTEO ESQUER	Enviada	Eventos Adversos Relacionados à	Lesão por pressão		
04/11/2021	CE	FORTALEZA	HGF	LPP GRAU II - CALCÂNEO	Enviada	Eventos Adversos Relacionados à	Lesão por pressão		
08/11/2021	CE	FORTALEZA	HGF	LPP GRAU II - GLÚTEO DIREITO	Enviada	Eventos Adversos Relacionados à	Lesão por pressão		
08/11/2021	CE	FORTALEZA	HGF	ESCAPULA ESQUERDA	Enviada	Eventos Adversos Relacionados à	Lesão por pressão		
08/11/2021	CE	FORTALEZA	HGF	LPP SACRA	Enviada	Eventos Adversos Relacionados à	Lesão por pressão		
08/11/2021	CE	FORTALEZA	HGF	LPP GRAU III EM REGIÃO	Concluída	Eventos Adversos Relacionados à	Lesão por pressão		
08/11/2021	CE	FORTALEZA	HGF	LPP SACRA	Enviada	Eventos Adversos Relacionados à	Lesão por pressão		
08/11/2021	CE	FORTALEZA	HGF	LPP GRAU II - SACRA	Enviada	Eventos Adversos Relacionados à	Lesão por pressão		
08/11/2021	CE	FORTALEZA	HGF	LPP CALCÂNEO ESQUER	Concluída	Eventos Adversos Relacionados à	Lesão por pressão		

08/11/2021	CE	FORTALEZA	HGF	LPP SACRA	Enviada	Eventos Adversos Relacionados à	Lesão por pressão		
08/11/2021	CE	FORTALEZA	HGF	LPP SACRA	Enviada	Eventos Adversos Relacionados à	Lesão por pressão		
08/11/2021	CE	FORTALEZA	HGF	LPP GRAU III EM REGIÃO	Concluída	Eventos Adversos Relacionados à	Lesão por pressão		
08/11/2021	CE	FORTALEZA	HGF	-	Enviada	Eventos Adversos Relacionados à	Lesão por pressão		
08/11/2021	CE	FORTALEZA	HGF	LPP EM INTEGLÚTEO	Enviada	Eventos Adversos Relacionados à	Lesão por pressão		
08/11/2021	CE	FORTALEZA	HGF	LPP GRAU II - SACRA	Enviada	Eventos Adversos Relacionados à	Lesão por pressão		
08/11/2021	CE	FORTALEZA	HGF	LPP SACRA	Enviada	Eventos Adversos Relacionados à	Lesão por pressão		
08/11/2021	CE	FORTALEZA	HGF	LPP SACRA	Concluída	Eventos Adversos Relacionados à	Lesão por pressão		
08/11/2021	CE	FORTALEZA	HGF	LPP GRAU I EM REGIÃO	Enviada	Eventos Adversos Relacionados à	Lesão por pressão		
08/11/2021	CE	FORTALEZA	HGF	LPP SACRA	Enviada	Eventos Adversos Relacionados à	Lesão por pressão		
08/11/2021	CE	FORTALEZA	HGF	LPP GRAU II EM GLÚTEO	Enviada	Eventos Adversos Relacionados à	Lesão por pressão		
08/11/2021	CE	FORTALEZA	HGF	LPP GRAU II EM REGIÃO	Enviada	Eventos Adversos Relacionados à	Lesão por pressão		
08/11/2021	CE	FORTALEZA	HGF	LPP SACRA	Enviada	Eventos Adversos Relacionados à	Lesão por pressão		
08/11/2021	CE	FORTALEZA	HGF	LPP SACRA	Concluída	Eventos Adversos Relacionados à	Lesão por pressão		
08/11/2021	CE	FORTALEZA	HGF	LPP SACRA	Enviada	Eventos Adversos Relacionados à	Lesão por pressão		

08/11/2021	CE	FORTALEZA	HGF	LPP EM ESCÁPULAS	Enviada	Eventos Adversos Relacionados à	Lesão por pressão		
08/11/2021	CE	FORTALEZA	HGF	LPP EM CALCÂNEO ESQUER	Enviada	Eventos Adversos Relacionados à	Lesão por pressão		
08/11/2021	CE	FORTALEZA	HGF	LPP SACRA	Enviada	Eventos Adversos Relacionados à	Lesão por pressão		
08/11/2021	CE	FORTALEZA	HGF	LPP SACRA	Enviada	Eventos Adversos Relacionados à	Lesão por pressão		
08/11/2021	CE	FORTALEZA	HGF	LPP GRAU II EM REGIÃO	Enviada	Eventos Adversos Relacionados à	Lesão por pressão		
08/11/2021	CE	FORTALEZA	HGF	PAVILHAO AURICULAR	Concluída	Eventos Adversos Relacionados à	Lesão por pressão		
08/11/2021	CE	FORTALEZA	HGF	LPP EM GLÚTEO ESQUERDO	Concluída	Eventos Adversos Relacionados à	Lesão por pressão		
08/11/2021	CE	FORTALEZA	HGF	LPP EM MALÉOLO ESQUER	Enviada	Eventos Adversos Relacionados à	Lesão por pressão		
08/11/2021	CE	FORTALEZA	HGF	LPP EM DORSO	Enviada	Eventos Adversos Relacionados à	Lesão por pressão		
08/11/2021	CE	FORTALEZA	HGF	LPP GRAU II EM REGIÃO	Enviada	Eventos Adversos Relacionados à	Lesão por pressão		
08/11/2021	CE	FORTALEZA	HGF	LPP EM PANTURRILHA ESQUER	Enviada	Eventos Adversos Relacionados à	Lesão por pressão		
08/11/2021	CE	FORTALEZA	HGF	LPP SACRA	Enviada	Eventos Adversos Relacionados à	Lesão por pressão		
08/11/2021	CE	FORTALEZA	HGF	LPP GRAU II EM REGIÃO	Enviada	Eventos Adversos Relacionados à	Lesão por pressão		
08/11/2021	CE	FORTALEZA	HGF	LPP SACRA	Enviada	Eventos Adversos Relacionados à	Lesão por pressão		
08/11/2021	CE	FORTALEZA	HGF	LPP GRAU II - GRAU II	Enviada	Eventos Adversos Relacionados à	Lesão por pressão		

08/11/2021	CE	FORTALEZA	HGF	LPP GRAU II EM REGIÃO	Enviada	Eventos Adversos Relacionados à	Lesão por pressão		
08/11/2021	CE	FORTALEZA	HGF	LPP EM DORSO	Enviada	Eventos Adversos Relacionados à	Lesão por pressão		
08/11/2021	CE	FORTALEZA	HGF	LPP SACRA	Concluída	Eventos Adversos Relacionados à	Lesão por pressão		
08/11/2021	CE	FORTALEZA	HGF	-	Enviada	Eventos Adversos Relacionados à	Lesão por pressão		
08/11/2021	CE	FORTALEZA	HGF	LPP EM CALCÂNEO DIREITO	Concluída	Eventos Adversos Relacionados à	Lesão por pressão		
08/11/2021	CE	FORTALEZA	HGF	LPP EM GLÚTEO DIREITO	Concluída	Eventos Adversos Relacionados à	Lesão por pressão		
08/11/2021	CE	FORTALEZA	HGF	-	Enviada	Eventos Adversos Relacionados à	Lesão por pressão		
08/11/2021	CE	FORTALEZA	HGF	LPP GRAU II EM REGIÃO	Enviada	Eventos Adversos Relacionados à	Lesão por pressão		
08/11/2021	CE	FORTALEZA	HGF	LPP SACRA	Enviada	Eventos Adversos Relacionados à	Lesão por pressão		
08/11/2021	CE	FORTALEZA	HGF	LPP GRAU II EM REGIÃO	Enviada	Eventos Adversos Relacionados à	Lesão por pressão		
08/11/2021	CE	FORTALEZA	HGF	LPP EM FACE INTERNA DO	Enviada	Eventos Adversos Relacionados à	Lesão por pressão		
08/11/2021	CE	FORTALEZA	HGF	LPP INTEGLÚTEO	Enviada	Eventos Adversos Relacionados à	Lesão por pressão		
08/11/2021	CE	FORTALEZA	HGF	LPP INTEGLÚTEO	Enviada	Eventos Adversos Relacionados à	Lesão por pressão		
08/11/2021	CE	FORTALEZA	HGF	LPP IV EM REGIÃO SACRAL	Concluída	Eventos Adversos Relacionados à	Lesão por pressão		
08/11/2021	CE	FORTALEZA	HGF	LPP SACRA	Enviada	Eventos Adversos Relacionados à	Lesão por pressão		

08/11/2021	CE	FORTALEZA	HGF	LPP IV EM GLÚTEO DIREITO	Concluída	Eventos Adversos Relacionados à	Lesão por pressão		
08/11/2021	CE	FORTALEZA	HGF	LPP SACRA	Enviada	Eventos Adversos Relacionados à	Lesão por pressão		
08/11/2021	CE	FORTALEZA	HGF	LPP SACRA	Enviada	Eventos Adversos Relacionados à	Lesão por pressão		
08/11/2021	CE	FORTALEZA	HGF	LPP GRAU IV EM GLÚTEO	Concluída	Eventos Adversos Relacionados à	Lesão por pressão		
08/11/2021	CE	FORTALEZA	HGF	LPP SACRA	Enviada	Eventos Adversos Relacionados à	Lesão por pressão		
08/11/2021	CE	FORTALEZA	HGF	LPP SACRA	Concluída	Eventos Adversos Relacionados à	Lesão por pressão		
08/11/2021	CE	FORTALEZA	HGF	LPP SACRA	Enviada	Eventos Adversos Relacionados à	Lesão por pressão		
08/11/2021	CE	FORTALEZA	HGF	-	Enviada	Eventos Adversos Relacionados à	Lesão por pressão		
08/11/2021	CE	FORTALEZA	HGF	LPP SACRA	Enviada	Eventos Adversos Relacionados à	Lesão por pressão		
08/11/2021	CE	FORTALEZA	HGF	LPP SACRA	Enviada	Eventos Adversos Relacionados à	Lesão por pressão		
08/11/2021	CE	FORTALEZA	HGF	LPP GRAU II EM REGIÃO	Enviada	Eventos Adversos Relacionados à	Lesão por pressão		
08/11/2021	CE	FORTALEZA	HGF	LPP SACRA	Enviada	Eventos Adversos Relacionados à	Lesão por pressão		
08/11/2021	CE	FORTALEZA	HGF	LPP SACRA	Enviada	Eventos Adversos Relacionados à	Lesão por pressão		
08/11/2021	CE	FORTALEZA	HGF	LPP SACRA	Enviada	Eventos Adversos Relacionados à	Lesão por pressão		
08/11/2021	CE	FORTALEZA	HGF	LPP GRAU II EM SACRA	Enviada	Eventos Adversos Relacionados à	Lesão por pressão		

08/11/2021	CE	FORTALEZA	HGF	LPP SACRA	Enviada	Eventos Adversos Relacionados à	Lesão por pressão		
08/11/2021	CE	FORTALEZA	HGF	LPP GRAU II EM REGIÃO	Enviada	Eventos Adversos Relacionados à	Lesão por pressão		
08/11/2021	CE	FORTALEZA	HGF	LPP SACRA ESTÁGIO 2	Enviada	Eventos Adversos Relacionados à	Lesão por pressão		
08/11/2021	CE	FORTALEZA	HGF	LPP EM TROCANTER	Enviada	Eventos Adversos Relacionados à	Lesão por pressão		
08/11/2021	CE	FORTALEZA	HGF	LPP GRAU III EM REGIÃO	Concluída	Eventos Adversos Relacionados à	Lesão por pressão		
08/11/2021	CE	FORTALEZA	HGF	CALCÂNEO DIREITO	Enviada	Eventos Adversos Relacionados à	Lesão por pressão		
08/11/2021	CE	FORTALEZA	HGF	TROCANTER DIREITO	Enviada	Eventos Adversos Relacionados à	Lesão por pressão		
08/11/2021	CE	FORTALEZA	HGF	LPP GRAU II EM REGIÃO	Enviada	Eventos Adversos Relacionados à	Lesão por pressão		
08/11/2021	CE	FORTALEZA	HGF	LPP GRAU II EM REGIÃO	Enviada	Eventos Adversos Relacionados à	Lesão por pressão		
08/11/2021	CE	FORTALEZA	HGF	LPP SACRA	Enviada	Eventos Adversos Relacionados à	Lesão por pressão		
08/11/2021	CE	FORTALEZA	HGF	LPP GRAU II EM REGIÃO	Enviada	Eventos Adversos Relacionados à	Lesão por pressão		
08/11/2021	CE	FORTALEZA	HGF	LPP SACRA ESTÁGIO 2	Enviada	Eventos Adversos Relacionados à	Lesão por pressão		
08/11/2021	CE	FORTALEZA	HGF	LPP GRAU II EM REGIÃO	Enviada	Eventos Adversos Relacionados à	Lesão por pressão		
08/11/2021	CE	FORTALEZA	HGF	LPP SACRA	Enviada	Eventos Adversos Relacionados à	Lesão por pressão		
08/11/2021	CE	FORTALEZA	HGF	LPP EM TROCANTER ESQUER	Enviada	Eventos Adversos Relacionados à	Lesão por pressão		

08/11/2021	CE	FORTALEZA	HGF	LPP SACRA	Enviada	Eventos Adversos Relacionados à	Lesão por pressão		
08/11/2021	CE	FORTALEZA	HGF	LPP GRAU I EM REGIÃO	Enviada	Eventos Adversos Relacionados à	Lesão por pressão		
08/11/2021	CE	FORTALEZA	HGF	LPP GRAU II EM REGIÃO	Enviada	Eventos Adversos Relacionados à	Lesão por pressão		
08/11/2021	CE	FORTALEZA	HGF	PACIENTE SENTIU DESCON	Enviada	Eventos Adversos Relacionados à	Falhas durante a assistência à saúde		
08/11/2021	CE	FORTALEZA	HGF	LPP ESTÁGIO I EM CALCÂN	Enviada	Eventos Adversos Relacionados à	Lesão por pressão		
08/11/2021	CE	FORTALEZA	HGF	Paciente foi ao banheiro sozinho e	Enviada	Eventos Adversos Relacionados à	Queda do paciente		
08/11/2021	CE	FORTALEZA	HGF	APÓS O TÉRMINO DO USO DA	Enviada	Eventos Adversos Relacionados à	Falhas durante a assistência à saúde		
08/11/2021	CE	FORTALEZA	HGF	FLEBITE EM MSD	Enviada	Eventos Adversos Relacionados à	Falhas envolvendo o cateter venoso		
08/11/2021	CE	FORTALEZA	HGF	FLEBITE EM MSD	Enviada	Eventos Adversos Relacionados à	Falhas envolvendo o cateter venoso		
08/11/2021	CE	FORTALEZA	HGF	FLEBITE EM MSD	Enviada	Eventos Adversos Relacionados à	Falhas envolvendo o cateter venoso		
08/11/2021	CE	FORTALEZA	HGF	QUEDA DA MACA	Enviada	Eventos Adversos Relacionados à	Queda do paciente		
08/11/2021	CE	FORTALEZA	HGF	QUEDA POR PROVÁVEL	Enviada	Eventos Adversos Relacionados à	Queda do paciente		
08/11/2021	CE	FORTALEZA	HGF	QUEDA AO TENTAR LEVANTA	Enviada	Eventos Adversos Relacionados à	Queda do paciente		
08/11/2021	CE	FORTALEZA	HGF	EDEMA E HIPEREMIA	Enviada	Eventos Adversos Relacionados à	Falhas envolvendo o cateter venoso		
08/11/2021	CE	FORTALEZA	HGF	FLEBITE EM MSD	Enviada	Eventos Adversos Relacionados à	Falhas envolvendo o cateter venoso		

08/11/2021	CE	FORTALEZA	HGF	EXTRAVASAMENTO DE DROGA	Enviada	Eventos Adversos Relacionados à	Falhas envolvendo o cateter venoso		
16/11/2021	CE	FORTALEZA	HGF	APRESENTOU DOR, RUBOR	Enviada	Eventos Adversos Relacionados à	Falhas envolvendo o cateter venoso		
16/11/2021	CE	FORTALEZA	HGF	PACIENTE RELATA QUE	Enviada	Eventos Adversos Relacionados à	Queda do paciente		
25/11/2021	CE	FORTALEZA	HGF	LPP SACRA	Enviada	Eventos Adversos Relacionados à	Lesão por pressão		
25/11/2021	CE	FORTALEZA	HGF	LPP SACRA	Enviada	Eventos Adversos Relacionados à	Lesão por pressão		
25/11/2021	CE	FORTALEZA	HGF	LPP EM GLÚTEO DIREITO	Enviada	Eventos Adversos Relacionados à	Lesão por pressão		
25/11/2021	CE	FORTALEZA	HGF	LPP SACRA	Enviada	Eventos Adversos Relacionados à	Lesão por pressão		
25/11/2021	CE	FORTALEZA	HGF	LPP SACRA	Enviada	Eventos Adversos Relacionados à	Lesão por pressão		
25/11/2021	CE	FORTALEZA	HGF	LPP SACRA	Enviada	Eventos Adversos Relacionados à	Lesão por pressão		
25/11/2021	CE	FORTALEZA	HGF	LPP SACRA	Enviada	Eventos Adversos Relacionados à	Lesão por pressão		
25/11/2021	CE	FORTALEZA	HGF	LPP SACRA	Enviada	Eventos Adversos Relacionados à	Lesão por pressão		
25/11/2021	CE	FORTALEZA	HGF	LPP SACRA	Concluída	Eventos Adversos Relacionados à	Lesão por pressão		
25/11/2021	CE	FORTALEZA	HGF	LPP SACRA	Enviada	Eventos Adversos Relacionados à	Lesão por pressão		
25/11/2021	CE	FORTALEZA	HGF	LPP SACRA	Concluída	Eventos Adversos Relacionados à	Lesão por pressão		
25/11/2021	CE	FORTALEZA	HGF	LPP SACRA	Enviada	Eventos Adversos Relacionados à	Lesão por pressão		

25/11/2021	CE	FORTALEZA	HGF	LPP SACRA	Enviada	Eventos Adversos Relaciona dos à	Lesão por pressão		
25/11/2021	CE	FORTALEZA	HGF	LPP SACRA	Enviada	Eventos Adversos Relaciona dos à	Lesão por pressão		
25/11/2021	CE	FORTALEZA	HGF	LPP SACRA	Enviada	Eventos Adversos Relaciona dos à	Lesão por pressão		
25/11/2021	CE	FORTALEZA	HGF	LPP SACRA	Enviada	Eventos Adversos Relaciona dos à	Lesão por pressão		
25/11/2021	CE	FORTALEZA	HGF	LPP SACRA	Concluída	Eventos Adversos Relaciona dos à	Lesão por pressão		
25/11/2021	CE	FORTALEZA	HGF	GLÚTEO DIREITO	Enviada	Eventos Adversos Relaciona dos à	Lesão por pressão		
25/11/2021	CE	FORTALEZA	HGF	LPP SACRA	Enviada	Eventos Adversos Relaciona dos à	Lesão por pressão		
25/11/2021	CE	FORTALEZA	HGF	LPP SACRA	Enviada	Eventos Adversos Relaciona dos à	Lesão por pressão		
25/11/2021	CE	FORTALEZA	HGF	TROCANTER DIREITO	Enviada	Eventos Adversos Relaciona dos à	Lesão por pressão		
25/11/2021	CE	FORTALEZA	HGF	TROCANTER DIREITO ESTÁGIO	Enviada	Eventos Adversos Relaciona dos à	Lesão por pressão		
25/11/2021	CE	FORTALEZA	HGF	LPP EM TROCÂNTER ESQUER	Concluída	Eventos Adversos Relaciona dos à	Lesão por pressão		
25/11/2021	CE	FORTALEZA	HGF	LPP SACRA	Concluída	Eventos Adversos Relaciona dos à	Lesão por pressão		
25/11/2021	CE	FORTALEZA	HGF	LPP SACRA	Enviada	Eventos Adversos Relaciona dos à	Lesão por pressão		
25/11/2021	CE	FORTALEZA	HGF	LPP SACRA	Enviada	Eventos Adversos Relaciona dos à	Lesão por pressão		
25/11/2021	CE	FORTALEZA	HGF	LPP SACRA	Enviada	Eventos Adversos Relaciona dos à	Lesão por pressão		

25/11/2021	CE	FORTALEZA	HGF	LPP SACRA	Enviada	Eventos Adversos Relacionados à	Lesão por pressão		
25/11/2021	CE	FORTALEZA	HGF	LPP SACRA	Enviada	Eventos Adversos Relacionados à	Lesão por pressão		
25/11/2021	CE	FORTALEZA	HGF	LPP SACRA	Concluída	Eventos Adversos Relacionados à	Lesão por pressão		
25/11/2021	CE	FORTALEZA	HGF	TROCANTER DIREITO	Enviada	Eventos Adversos Relacionados à	Lesão por pressão		
25/11/2021	CE	FORTALEZA	HGF	LPP SACRA	Enviada	Eventos Adversos Relacionados à	Lesão por pressão		
25/11/2021	CE	FORTALEZA	HGF	LPP SACRA	Enviada	Eventos Adversos Relacionados à	Lesão por pressão		
25/11/2021	CE	FORTALEZA	HGF	LPP SACRA	Enviada	Eventos Adversos Relacionados à	Lesão por pressão		
25/11/2021	CE	FORTALEZA	HGF	LPP SACRA	Enviada	Eventos Adversos Relacionados à	Lesão por pressão		
25/11/2021	CE	FORTALEZA	HGF	LPP SACRA	Enviada	Eventos Adversos Relacionados à	Lesão por pressão		
25/11/2021	CE	FORTALEZA	HGF	OUTROS- JOELHOS	Enviada	Eventos Adversos Relacionados à	Lesão por pressão		
25/11/2021	CE	FORTALEZA	HGF	LPP SACRA	Concluída	Eventos Adversos Relacionados à	Lesão por pressão		
25/11/2021	CE	FORTALEZA	HGF	-	Enviada	Eventos Adversos Relacionados à	Lesão por pressão		
25/11/2021	CE	FORTALEZA	HGF	-	Concluída	Eventos Adversos Relacionados à	Lesão por pressão		
25/11/2021	CE	FORTALEZA	HGF	LPP EM JOELHO DIREITO	Enviada	Eventos Adversos Relacionados à	Lesão por pressão		
25/11/2021	CE	FORTALEZA	HGF	ÍSQUIO DIREITO	Enviada	Eventos Adversos Relacionados à	Lesão por pressão		

25/11/2021	CE	FORTALE ZA	HGF	JOELHO E	Enviada	Eventos Adversos Relaciona dos à	Lesão por pressão		
25/11/2021	CE	FORTALE ZA	HGF	LPP SACRA	Enviada	Eventos Adversos Relaciona dos à	Lesão por pressão		
25/11/2021	CE	FORTALE ZA	HGF	LPP SACRA	Enviada	Eventos Adversos Relaciona dos à	Lesão por pressão		
25/11/2021	CE	FORTALE ZA	HGF	LPP EM GLÚTEO DIREITO	Enviada	Eventos Adversos Relaciona dos à	Lesão por pressão		
25/11/2021	CE	FORTALE ZA	HGF	LPP EM GLÚTEO DIREITO	Concluída	Eventos Adversos Relaciona dos à	Lesão por pressão		
25/11/2021	CE	FORTALE ZA	HGF	GLÚTEO ESQUER DO	Enviada	Eventos Adversos Relaciona dos à	Lesão por pressão		
25/11/2021	CE	FORTALE ZA	HGF	LPP SACRA	Concluída	Eventos Adversos Relaciona dos à	Lesão por pressão		
25/11/2021	CE	FORTALE ZA	HGF	TROCÂN TER DIREITO	Enviada	Eventos Adversos Relaciona dos à	Lesão por pressão		
26/11/2021	CE	FORTALE ZA	HGF	LPP SACRA	Enviada	Eventos Adversos Relaciona dos à	Lesão por pressão		
26/11/2021	CE	FORTALE ZA	HGF	LPP SACRA	Enviada	Eventos Adversos Relaciona dos à	Lesão por pressão		
26/11/2021	CE	FORTALE ZA	HGF	LPP SACRA	Enviada	Eventos Adversos Relaciona dos à	Lesão por pressão		
26/11/2021	CE	FORTALE ZA	HGF	-	Enviada	Eventos Adversos Relaciona dos à	Lesão por pressão		
26/11/2021	CE	FORTALE ZA	HGF	LPP SACRA	Enviada	Eventos Adversos Relaciona dos à	Lesão por pressão		
26/11/2021	CE	FORTALE ZA	HGF	LPP SACRA	Enviada	Eventos Adversos Relaciona dos à	Lesão por pressão		
26/11/2021	CE	FORTALE ZA	HGF	LPP SACRA	Concluída	Eventos Adversos Relaciona dos à	Lesão por pressão		

26/11/2021	CE	FORTALE ZA	HGF	LPP SACRA	Enviada	Eventos Adversos Relaciona dos à	Lesão por pressão		
26/11/2021	CE	FORTALE ZA	HGF	LPP SACRA	Enviada	Eventos Adversos Relaciona dos à	Lesão por pressão		
26/11/2021	CE	FORTALE ZA	HGF	LPP SACRA	Enviada	Eventos Adversos Relaciona dos à	Lesão por pressão		
26/11/2021	CE	FORTALE ZA	HGF	LPP EM TROCÂN TER DIREITO	Enviada	Eventos Adversos Relaciona dos à	Lesão por pressão		
26/11/2021	CE	FORTALE ZA	HGF	LPP SACRA	Concluída	Eventos Adversos Relaciona dos à	Lesão por pressão		
26/11/2021	CE	FORTALE ZA	HGF	LPP EM TROCÂN TER DIREITO	Enviada	Eventos Adversos Relaciona dos à	Lesão por pressão		
26/11/2021	CE	FORTALE ZA	HGF	LPP SACRA	Concluída	Eventos Adversos Relaciona dos à	Lesão por pressão		
26/11/2021	CE	FORTALE ZA	HGF	LPP SACRA	Enviada	Eventos Adversos Relaciona dos à	Lesão por pressão		
26/11/2021	CE	FORTALE ZA	HGF	LPP EM DORSO	Enviada	Eventos Adversos Relaciona dos à	Lesão por pressão		
26/11/2021	CE	FORTALE ZA	HGF	LPP EM CALCÂN EO DIREITO	Enviada	Eventos Adversos Relaciona dos à	Lesão por pressão		
26/11/2021	CE	FORTALE ZA	HGF	LPP SACRA	Enviada	Eventos Adversos Relaciona dos à	Lesão por pressão		
26/11/2021	CE	FORTALE ZA	HGF	LPP SACRA	Enviada	Eventos Adversos Relaciona dos à	Lesão por pressão		
26/11/2021	CE	FORTALE ZA	HGF	LPP SACRA	Enviada	Eventos Adversos Relaciona dos à	Lesão por pressão		
26/11/2021	CE	FORTALE ZA	HGF	MALEOL O ESQUER DO	Enviada	Eventos Adversos Relaciona dos à	Lesão por pressão		
26/11/2021	CE	FORTALE ZA	HGF	LPP SACRA	Enviada	Eventos Adversos Relaciona dos à	Lesão por pressão		

26/11/2021	CE	FORTALE ZA	HGF	LPP SACRA	Concluída	Eventos Adversos Relaciona dos à	Lesão por pressão		
26/11/2021	CE	FORTALE ZA	HGF	LPP SACRA	Enviada	Eventos Adversos Relaciona dos à	Lesão por pressão		
26/11/2021	CE	FORTALE ZA	HGF	LPP SACRA	Enviada	Eventos Adversos Relaciona dos à	Lesão por pressão		
26/11/2021	CE	FORTALE ZA	HGF	LPP SACRA	Enviada	Eventos Adversos Relaciona dos à	Lesão por pressão		
26/11/2021	CE	FORTALE ZA	HGF	LPP SACRA	Enviada	Eventos Adversos Relaciona dos à	Lesão por pressão		
26/11/2021	CE	FORTALE ZA	HGF	LPP SACRA	Enviada	Eventos Adversos Relaciona dos à	Lesão por pressão		
26/11/2021	CE	FORTALE ZA	HGF	TROCAN TER ESQUER DO	Enviada	Eventos Adversos Relaciona dos à	Lesão por pressão		
29/11/2021	CE	FORTALE ZA	HGF	LPP SACRA	Enviada	Eventos Adversos Relaciona dos à	Lesão por pressão		
29/11/2021	CE	FORTALE ZA	HGF	LPP SACRA	Enviada	Eventos Adversos Relaciona dos à	Lesão por pressão		
29/11/2021	CE	FORTALE ZA	HGF	LPP EM GLÚTEO DIREITO	Enviada	Eventos Adversos Relaciona dos à	Lesão por pressão		
29/11/2021	CE	FORTALE ZA	HGF	LPP SACRA	Concluída	Eventos Adversos Relaciona dos à	Lesão por pressão		
29/11/2021	CE	FORTALE ZA	HGF	LPP EM GLÚTEO ESQUER DO	Enviada	Eventos Adversos Relaciona dos à	Lesão por pressão		
29/11/2021	CE	FORTALE ZA	HGF	LPP SACRA	Enviada	Eventos Adversos Relaciona dos à	Lesão por pressão		
29/11/2021	CE	FORTALE ZA	HGF	LPP EM GLÚTEO DIREITO	Enviada	Eventos Adversos Relaciona dos à	Lesão por pressão		
29/11/2021	CE	FORTALE ZA	HGF	LPP SACRA	Enviada	Eventos Adversos Relaciona dos à	Lesão por pressão		

29/11/2021	CE	FORTALE ZA	HGF	LPP SACRA	Enviada	Eventos Adversos Relaciona dos à	Lesão por pressão		
29/11/2021	CE	FORTALE ZA	HGF	LPP EM GLÚTEO ESQUER DO	Enviada	Eventos Adversos Relaciona dos à	Lesão por pressão		
29/11/2021	CE	FORTALE ZA	HGF	LPP SACRA	Enviada	Eventos Adversos Relaciona dos à	Lesão por pressão		
29/11/2021	CE	FORTALE ZA	HGF	LPP SACRA	Enviada	Eventos Adversos Relaciona dos à	Lesão por pressão		
29/11/2021	CE	FORTALE ZA	HGF	LPP SACRA	Concluída	Eventos Adversos Relaciona dos à	Lesão por pressão		
29/11/2021	CE	FORTALE ZA	HGF	LPP SACRA	Enviada	Eventos Adversos Relaciona dos à	Lesão por pressão		
29/11/2021	CE	FORTALE ZA	HGF	LPP SACRA	Enviada	Eventos Adversos Relaciona dos à	Lesão por pressão		
29/11/2021	CE	FORTALE ZA	HGF	LPP EM GLÚTEO ESQUER DO	Enviada	Eventos Adversos Relaciona dos à	Lesão por pressão		
29/11/2021	CE	FORTALE ZA	HGF	LPP SACRA	Enviada	Eventos Adversos Relaciona dos à	Lesão por pressão		
29/11/2021	CE	FORTALE ZA	HGF	LPP SACRA	Enviada	Eventos Adversos Relaciona dos à	Lesão por pressão		
29/11/2021	CE	FORTALE ZA	HGF	LPP SACRA	Enviada	Eventos Adversos Relaciona dos à	Lesão por pressão		
29/11/2021	CE	FORTALE ZA	HGF	LPP SACRA	Enviada	Eventos Adversos Relaciona dos à	Lesão por pressão		
29/11/2021	CE	FORTALE ZA	HGF	LPP SACRA	Enviada	Eventos Adversos Relaciona dos à	Lesão por pressão		
29/11/2021	CE	FORTALE ZA	HGF	LPP SACRA	Enviada	Eventos Adversos Relaciona dos à	Lesão por pressão		
29/11/2021	CE	FORTALE ZA	HGF	LPP SACRA	Enviada	Eventos Adversos Relaciona dos à	Lesão por pressão		

29/11/2021	CE	FORTALEZA	HGF	LPP SACRA	Enviada	Eventos Adversos Relacionados à	Lesão por pressão		
29/11/2021	CE	FORTALEZA	HGF	-	Enviada	Eventos Adversos Relacionados à	Lesão por pressão		
29/11/2021	CE	FORTALEZA	HGF	LPP SACRA	Enviada	Eventos Adversos Relacionados à	Lesão por pressão		
29/11/2021	CE	FORTALEZA	HGF	LPP SACRA	Enviada	Eventos Adversos Relacionados à	Lesão por pressão		
29/11/2021	CE	FORTALEZA	HGF	LPP SACRA	Enviada	Eventos Adversos Relacionados à	Lesão por pressão		
29/11/2021	CE	FORTALEZA	HGF	lpp em trocânteres	Enviada	Eventos Adversos Relacionados à	Lesão por pressão		
29/11/2021	CE	FORTALEZA	HGF	LPP EM PANTURRILHA	Enviada	Eventos Adversos Relacionados à	Lesão por pressão		
30/11/2021	CE	FORTALEZA	HGF	LPP EM CALCÂNEO DIREITO	Enviada	Eventos Adversos Relacionados à	Lesão por pressão		
30/11/2021	CE	FORTALEZA	HGF	LPP SACRA	Enviada	Eventos Adversos Relacionados à	Lesão por pressão		
30/11/2021	CE	FORTALEZA	HGF	-	Enviada	Eventos Adversos Relacionados à	Lesão por pressão		
30/11/2021	CE	FORTALEZA	HGF	LPP SACRA	Enviada	Eventos Adversos Relacionados à	Lesão por pressão		
30/11/2021	CE	FORTALEZA	HGF	-	Enviada	Eventos Adversos Relacionados à	Lesão por pressão		
30/11/2021	CE	FORTALEZA	HGF	-	Enviada	Eventos Adversos Relacionados à	Lesão por pressão		
30/11/2021	CE	FORTALEZA	HGF	LPP SACRA	Enviada	Eventos Adversos Relacionados à	Lesão por pressão		
30/11/2021	CE	FORTALEZA	HGF	LPP EM REGIÃO SACRA	Enviada	Eventos Adversos Relacionados à	Lesão por pressão		

30/11/2021	CE	FORTALEZA	HGF	LPP SACRA	Concluída	Eventos Adversos Relacionados à	Lesão por pressão		
30/11/2021	CE	FORTALEZA	HGF	LPP EM REGIÃO SACRA	Concluída	Eventos Adversos Relacionados à	Lesão por pressão		
30/11/2021	CE	FORTALEZA	HGF	LPP EM REGIÃO GLUTEA DIREITA	Enviada	Eventos Adversos Relacionados à	Lesão por pressão		
30/11/2021	CE	FORTALEZA	HGF	LPP SACRA	Enviada	Eventos Adversos Relacionados à	Lesão por pressão		
30/11/2021	CE	FORTALEZA	HGF	-	Concluída	Eventos Adversos Relacionados à	Lesão por pressão		
30/11/2021	CE	FORTALEZA	HGF	-	Enviada	Eventos Adversos Relacionados à	Lesão por pressão		
30/11/2021	CE	FORTALEZA	HGF	LPP EM REGIÃO SACRA	Enviada	Eventos Adversos Relacionados à	Lesão por pressão		
30/11/2021	CE	FORTALEZA	HGF	LPP SACRA	Enviada	Eventos Adversos Relacionados à	Lesão por pressão		
30/11/2021	CE	FORTALEZA	HGF	LPP EM REGIÃO SACRA	Enviada	Eventos Adversos Relacionados à	Lesão por pressão		
30/11/2021	CE	FORTALEZA	HGF	LPP EM REGIAO SACRA	Enviada	Eventos Adversos Relacionados à	Lesão por pressão		
30/11/2021	CE	FORTALEZA	HGF	LPP EM REGIÃO SACRA	Enviada	Eventos Adversos Relacionados à	Lesão por pressão		
30/11/2021	CE	FORTALEZA	HGF	LPP EM REGIÃO SACRA	Enviada	Eventos Adversos Relacionados à	Lesão por pressão		
30/11/2021	CE	FORTALEZA	HGF	LPP SACRA	Enviada	Eventos Adversos Relacionados à	Lesão por pressão		
30/11/2021	CE	FORTALEZA	HGF	LPP EM REGIÃO CALCÂNEO	Enviada	Eventos Adversos Relacionados à	Lesão por pressão		
30/11/2021	CE	FORTALEZA	HGF	LPP EM CALCÂNEO DIREITO	Enviada	Eventos Adversos Relacionados à	Lesão por pressão		

30/11/2021	CE	FORTALEZA	HGF	LPP EM REGIÂO SACRA	Enviada	Eventos Adversos Relacionados à	Lesão por pressão		
30/11/2021	CE	FORTALEZA	HGF	LPP EM CALCÂNEO DIREITO	Enviada	Eventos Adversos Relacionados à	Lesão por pressão		
30/11/2021	CE	FORTALEZA	HGF	LPP EM PANTURRILHAS	Enviada	Eventos Adversos Relacionados à	Lesão por pressão		
30/11/2021	CE	FORTALEZA	HGF	LPP SACRA	Enviada	Eventos Adversos Relacionados à	Lesão por pressão		
30/11/2021	CE	FORTALEZA	HGF	LPP EM CALCÂNEO DIREITO	Enviada	Eventos Adversos Relacionados à	Lesão por pressão		
30/11/2021	CE	FORTALEZA	HGF	LPP EM CALCÂNEO DIREITO	Enviada	Eventos Adversos Relacionados à	Lesão por pressão		
30/11/2021	CE	FORTALEZA	HGF	LPP EM REGIÃO SACRA	Enviada	Eventos Adversos Relacionados à	Lesão por pressão		
30/11/2021	CE	FORTALEZA	HGF	LPP EM REGIÂO SACRA	Enviada	Eventos Adversos Relacionados à	Lesão por pressão		
30/11/2021	CE	FORTALEZA	HGF	LPP EM CALCÂNEO DIREITO	Enviada	Eventos Adversos Relacionados à	Lesão por pressão		
30/11/2021	CE	FORTALEZA	HGF	LPP EM REGIÃO SACRA	Enviada	Eventos Adversos Relacionados à	Lesão por pressão		
30/11/2021	CE	FORTALEZA	HGF	LPP SACRA	Enviada	Eventos Adversos Relacionados à	Lesão por pressão		
30/11/2021	CE	FORTALEZA	HGF	LPP SACRA	Concluída	Eventos Adversos Relacionados à	Lesão por pressão		
30/11/2021	CE	FORTALEZA	HGF	LPP SACRA	Concluída	Eventos Adversos Relacionados à	Lesão por pressão		
30/11/2021	CE	FORTALEZA	HGF	LPP EM GLUTEO ESQUERDO	Enviada	Eventos Adversos Relacionados à	Lesão por pressão		
30/11/2021	CE	FORTALEZA	HGF	LPP EM REGIÃO SACRA	Enviada	Eventos Adversos Relacionados à	Lesão por pressão		

30/11/2021	CE	FORTALE ZA	HGF	LPP EM REGIÃO SACRA	Enviada	Eventos Adversos Relaciona dos à	Lesão por pressão		
30/11/2021	CE	FORTALE ZA	HGF	LPP SACRA	Enviada	Eventos Adversos Relaciona dos à	Lesão por pressão		
30/11/2021	CE	FORTALE ZA	HGF	LPP EM REGIÃO SACRA	Enviada	Eventos Adversos Relaciona dos à	Lesão por pressão		
30/11/2021	CE	FORTALE ZA	HGF	LPP SACRA	Enviada	Eventos Adversos Relaciona dos à	Lesão por pressão		
30/11/2021	CE	FORTALE ZA	HGF	-	Enviada	Eventos Adversos Relaciona dos à	Lesão por pressão		
30/11/2021	CE	FORTALE ZA	HGF	LPP SACRA	Enviada	Eventos Adversos Relaciona dos à	Lesão por pressão		
30/11/2021	CE	FORTALE ZA	HGF	LPP EM REGIÃO SACRA	Enviada	Eventos Adversos Relaciona dos à	Lesão por pressão		
30/11/2021	CE	FORTALE ZA	HGF	LPP SACRA	Enviada	Eventos Adversos Relaciona dos à	Lesão por pressão		
30/11/2021	CE	FORTALE ZA	HGF	LPP EM COTOVE LOS	Enviada	Eventos Adversos Relaciona dos à	Lesão por pressão		
30/11/2021	CE	FORTALE ZA	HGF	LPP EM CALCÂN EO DIREITO	Enviada	Eventos Adversos Relaciona dos à	Lesão por pressão		
30/11/2021	CE	FORTALE ZA	HGF	ESCÁPUL AS	Enviada	Eventos Adversos Relaciona dos à	Lesão por pressão		
30/11/2021	CE	FORTALE ZA	HGF	LPP EM REGIÃO SACRA	Enviada	Eventos Adversos Relaciona dos à	Lesão por pressão		
30/11/2021	CE	FORTALE ZA	HGF	LPP EM REGIÃO SACRA	Enviada	Eventos Adversos Relaciona dos à	Lesão por pressão		
30/11/2021	CE	FORTALE ZA	HGF	LPP EM COTOVE LO ESQUER	Enviada	Eventos Adversos Relaciona dos à	Lesão por pressão		
30/11/2021	CE	FORTALE ZA	HGF	LPP EM REGIÃO SACRA	Enviada	Eventos Adversos Relaciona dos à	Lesão por pressão		

30/11/2021	CE	FORTALEZA	HGF	LPP SACRA ESTÁGIO 2	Enviada	Eventos Adversos Relacionados à	Lesão por pressão		
30/11/2021	CE	FORTALEZA	HGF	LPP SACRA ESTÁGIO 2	Enviada	Eventos Adversos Relacionados à	Lesão por pressão		
30/11/2021	CE	FORTALEZA	HGF	LPP EM GLÚTEO DIREITO	Enviada	Eventos Adversos Relacionados à	Lesão por pressão		
30/11/2021	CE	FORTALEZA	HGF	LPP SACRA ESTÁGIO 2	Enviada	Eventos Adversos Relacionados à	Lesão por pressão		
30/11/2021	CE	FORTALEZA	HGF	LPP EM REGIÃO SACRA	Concluída	Eventos Adversos Relacionados à	Lesão por pressão		
30/11/2021	CE	FORTALEZA	HGF	LPP EM REGIÃO SACRA	Enviada	Eventos Adversos Relacionados à	Lesão por pressão		
30/11/2021	CE	FORTALEZA	HGF	LPP EM REGIÃO SACRA	Enviada	Eventos Adversos Relacionados à	Lesão por pressão		
01/12/2021	CE	FORTALEZA	HGF	LPP SACRA	Enviada	Eventos Adversos Relacionados à	Lesão por pressão		
01/12/2021	CE	FORTALEZA	HGF	LPP SACRA	Enviada	Eventos Adversos Relacionados à	Lesão por pressão		
01/12/2021	CE	FORTALEZA	HGF	LPP SACRA	Enviada	Eventos Adversos Relacionados à	Lesão por pressão		
01/12/2021	CE	FORTALEZA	HGF	LPP SACRA	Enviada	Eventos Adversos Relacionados à	Lesão por pressão		
01/12/2021	CE	FORTALEZA	HGF	-	Enviada	Eventos Adversos Relacionados à	Lesão por pressão		
01/12/2021	CE	FORTALEZA	HGF	LPP SACRA	Enviada	Eventos Adversos Relacionados à	Lesão por pressão		
01/12/2021	CE	FORTALEZA	HGF	LPP SACRA	Enviada	Eventos Adversos Relacionados à	Lesão por pressão		
01/12/2021	CE	FORTALEZA	HGF	LPP SACRA	Enviada	Eventos Adversos Relacionados à	Lesão por pressão		

01/12/2021	CE	FORTALEZA	HGF	LPP SACRA	Enviada	Eventos Adversos Relacionados à	Lesão por pressão		
01/12/2021	CE	FORTALEZA	HGF	-	Enviada	Eventos Adversos Relacionados à	Lesão por pressão		
01/12/2021	CE	FORTALEZA	HGF	LPP EM TROCÂNTERES D	Enviada	Eventos Adversos Relacionados à	Lesão por pressão		
01/12/2021	CE	FORTALEZA	HGF	LPP SACRA	Enviada	Eventos Adversos Relacionados à	Lesão por pressão		
01/12/2021	CE	FORTALEZA	HGF	LPP SACRA	Enviada	Eventos Adversos Relacionados à	Lesão por pressão		
01/12/2021	CE	FORTALEZA	HGF	LPP SACRA	Enviada	Eventos Adversos Relacionados à	Lesão por pressão		
01/12/2021	CE	FORTALEZA	HGF	LPP SACRA	Enviada	Eventos Adversos Relacionados à	Lesão por pressão		
01/12/2021	CE	FORTALEZA	HGF	LPP EM GLÚTEO D e E	Enviada	Eventos Adversos Relacionados à	Lesão por pressão		
01/12/2021	CE	FORTALEZA	HGF	LPP SACRA	Enviada	Eventos Adversos Relacionados à	Lesão por pressão		
01/12/2021	CE	FORTALEZA	HGF	LPP EM GLÚTEO ESQUERDO	Enviada	Eventos Adversos Relacionados à	Lesão por pressão		
01/12/2021	CE	FORTALEZA	HGF	LPP SACRA	Enviada	Eventos Adversos Relacionados à	Lesão por pressão		
01/12/2021	CE	FORTALEZA	HGF	LPP SACRA	Enviada	Eventos Adversos Relacionados à	Lesão por pressão		
01/12/2021	CE	FORTALEZA	HGF	-	Enviada	Eventos Adversos Relacionados à	Lesão por pressão		
01/12/2021	CE	FORTALEZA	HGF	LPP SACRA	Enviada	Eventos Adversos Relacionados à	Lesão por pressão		
01/12/2021	CE	FORTALEZA	HGF	LPP SACRA	Enviada	Eventos Adversos Relacionados à	Lesão por pressão		

01/12/2021	CE	FORTALE ZA	HGF	-	Enviada	Eventos Adversos Relaciona dos à	Lesão por pressão		
01/12/2021	CE	FORTALE ZA	HGF	LPP SACRA	Enviada	Eventos Adversos Relaciona dos à	Lesão por pressão		
01/12/2021	CE	FORTALE ZA	HGF	LPP SACRA	Enviada	Eventos Adversos Relaciona dos à	Lesão por pressão		
01/12/2021	CE	FORTALE ZA	HGF	LPP SACRA	Concluída	Eventos Adversos Relaciona dos à	Lesão por pressão		
01/12/2021	CE	FORTALE ZA	HGF	LPP SACRA	Concluída	Eventos Adversos Relaciona dos à	Lesão por pressão		
01/12/2021	CE	FORTALE ZA	HGF	LPP SACRA	Enviada	Eventos Adversos Relaciona dos à	Lesão por pressão		
01/12/2021	CE	FORTALE ZA	HGF	-	Concluída	Eventos Adversos Relaciona dos à	Lesão por pressão		
01/12/2021	CE	FORTALE ZA	HGF	-	Concluída	Eventos Adversos Relaciona dos à	Lesão por pressão		
01/12/2021	CE	FORTALE ZA	HGF	LPP SACRA	Enviada	Eventos Adversos Relaciona dos à	Lesão por pressão		
01/12/2021	CE	FORTALE ZA	HGF	LPP SACRA	Enviada	Eventos Adversos Relaciona dos à	Lesão por pressão		
01/12/2021	CE	FORTALE ZA	HGF	LPP SACRA	Enviada	Eventos Adversos Relaciona dos à	Lesão por pressão		
01/12/2021	CE	FORTALE ZA	HGF	-	Enviada	Eventos Adversos Relaciona dos à	Lesão por pressão		
01/12/2021	CE	FORTALE ZA	HGF	LPP EM MAMA	Enviada	Eventos Adversos Relaciona dos à	Lesão por pressão		
02/12/2021	CE	FORTALE ZA	HGF	-	Enviada	Eventos Adversos Relaciona dos à	Lesão por pressão		
02/12/2021	CE	FORTALE ZA	HGF	-	Enviada	Eventos Adversos Relaciona dos à	Lesão por pressão		

02/12/2021	CE	FORTALE ZA	HGF	LPP SACRA	Concluída	Eventos Adversos Relaciona dos à	Lesão por pressão		
02/12/2021	CE	FORTALE ZA	HGF	-	Enviada	Eventos Adversos Relaciona dos à	Lesão por pressão		
02/12/2021	CE	FORTALE ZA	HGF	-	Enviada	Eventos Adversos Relaciona dos à	Lesão por pressão		
02/12/2021	CE	FORTALE ZA	HGF	LPP SACRA	Concluída	Eventos Adversos Relaciona dos à	Lesão por pressão		
02/12/2021	CE	FORTALE ZA	HGF	-	Enviada	Eventos Adversos Relaciona dos à	Lesão por pressão		
02/12/2021	CE	FORTALE ZA	HGF	LPP INTEGLÚ TEO	Enviada	Eventos Adversos Relaciona dos à	Lesão por pressão		
02/12/2021	CE	FORTALE ZA	HGF	LPP SACRA	Enviada	Eventos Adversos Relaciona dos à	Lesão por pressão		
02/12/2021	CE	FORTALE ZA	HGF	LPP SACRA	Enviada	Eventos Adversos Relaciona dos à	Lesão por pressão		
02/12/2021	CE	FORTALE ZA	HGF	LPP INTEGLÚ TEO	Enviada	Eventos Adversos Relaciona dos à	Lesão por pressão		
02/12/2021	CE	FORTALE ZA	HGF	-	Enviada	Eventos Adversos Relaciona dos à	Lesão por pressão		
02/12/2021	CE	FORTALE ZA	HGF	LPP EM TROCÂN TER DIREITO	Enviada	Eventos Adversos Relaciona dos à	Lesão por pressão		
02/12/2021	CE	FORTALE ZA	HGF	LPP EM GLÚTEO DIREITO	Enviada	Eventos Adversos Relaciona dos à	Lesão por pressão		
02/12/2021	CE	FORTALE ZA	HGF	LPP SACRA	Enviada	Eventos Adversos Relaciona dos à	Lesão por pressão		
02/12/2021	CE	FORTALE ZA	HGF	-	Concluída	Eventos Adversos Relaciona dos à	Lesão por pressão		
02/12/2021	CE	FORTALE ZA	HGF	-	Concluída	Eventos Adversos Relaciona dos à	Lesão por pressão		

02/12/2021	CE	FORTALEZA	HGF	LPP EM GLÚTEO	Concluída	Eventos Adversos Relacionados à	Lesão por pressão		
02/12/2021	CE	FORTALEZA	HGF	LPP SACRA	Enviada	Eventos Adversos Relacionados à	Lesão por pressão		
02/12/2021	CE	FORTALEZA	HGF	-	Enviada	Eventos Adversos Relacionados à	Lesão por pressão		
02/12/2021	CE	FORTALEZA	HGF	-	Enviada	Eventos Adversos Relacionados à	Lesão por pressão		
02/12/2021	CE	FORTALEZA	HGF	-	Enviada	Eventos Adversos Relacionados à	Lesão por pressão		
02/12/2021	CE	FORTALEZA	HGF	LPP SACRA	Enviada	Eventos Adversos Relacionados à	Lesão por pressão		
02/12/2021	CE	FORTALEZA	HGF	LPP SACRA	Enviada	Eventos Adversos Relacionados à	Lesão por pressão		
02/12/2021	CE	FORTALEZA	HGF	-	Enviada	Eventos Adversos Relacionados à	Lesão por pressão		
02/12/2021	CE	FORTALEZA	HGF	-	Enviada	Eventos Adversos Relacionados à	Lesão por pressão		
02/12/2021	CE	FORTALEZA	HGF	LPP SACRA	Enviada	Eventos Adversos Relacionados à	Lesão por pressão		
02/12/2021	CE	FORTALEZA	HGF	-	Enviada	Eventos Adversos Relacionados à	Lesão por pressão		
02/12/2021	CE	FORTALEZA	HGF	-	Enviada	Eventos Adversos Relacionados à	Lesão por pressão		
02/12/2021	CE	FORTALEZA	HGF	-	Enviada	Eventos Adversos Relacionados à	Lesão por pressão		
02/12/2021	CE	FORTALEZA	HGF	-	Enviada	Eventos Adversos Relacionados à	Lesão por pressão		
02/12/2021	CE	FORTALEZA	HGF	-	Enviada	Eventos Adversos Relacionados à	Lesão por pressão		

03/12/2021	CE	FORTALE ZA	HGF	LPP SACRA	Enviada	Eventos Adversos Relaciona dos à	Lesão por pressão		
03/12/2021	CE	FORTALE ZA	HGF	LPP SACRA	Enviada	Eventos Adversos Relaciona dos à	Lesão por pressão		
03/12/2021	CE	FORTALE ZA	HGF	LPP SACRA	Enviada	Eventos Adversos Relaciona dos à	Lesão por pressão		
03/12/2021	CE	FORTALE ZA	HGF	LPP SACRA	Enviada	Eventos Adversos Relaciona dos à	Lesão por pressão		
03/12/2021	CE	FORTALE ZA	HGF	LPP SACRA	Enviada	Eventos Adversos Relaciona dos à	Lesão por pressão		
03/12/2021	CE	FORTALE ZA	HGF	LPP SACRA	Enviada	Eventos Adversos Relaciona dos à	Lesão por pressão		
03/12/2021	CE	FORTALE ZA	HGF	LPP EM GLÚTEO DIREITO E	Enviada	Eventos Adversos Relaciona dos à	Lesão por pressão		
03/12/2021	CE	FORTALE ZA	HGF	PACIENT E CONSCIE NTE	Enviada	Eventos Adversos Relaciona dos à	Queda do paciente		
03/12/2021	CE	FORTALE ZA	HGF	Equipo com retorno sanguíneo	Enviada	Eventos Adversos Relaciona dos à	Falhas durante a assistênci a à saúde		
03/12/2021	CE	FORTALE ZA	HGF	Realizada troca de medicaçã o entre	Enviada	Eventos Adversos Relaciona dos à	Falhas durante a assistênci a à saúde		
03/12/2021	CE	FORTALE ZA	HGF	APRESE NTOU QUEDA DA	Enviada	Eventos Adversos Relaciona dos à	Queda do paciente		
09/12/2021	CE	FORTALE ZA	HGF	LPP EM REGIAO SACRA	Concluída	Eventos Adversos Relaciona dos à	Lesão por pressão		
09/12/2021	CE	FORTALE ZA	HGF	LPP EM REGIAO SACRA	Enviada	Eventos Adversos Relaciona dos à	Lesão por pressão		
09/12/2021	CE	FORTALE ZA	HGF	LPP EM REGIAO SACRA	Enviada	Eventos Adversos Relaciona dos à	Lesão por pressão		
09/12/2021	CE	FORTALE ZA	HGF	LPP EM REGIAO SACRA	Enviada	Eventos Adversos Relaciona dos à	Lesão por pressão		

09/12/2021	CE	FORTALE ZA	HGF	LPP EM REGIAO GLUTEA DIREITA	Enviada	Eventos Adversos Relaciona dos à	Lesão por pressão		
09/12/2021	CE	FORTALE ZA	HGF	LPP EM REGIAO SACRA	Enviada	Eventos Adversos Relaciona dos à	Lesão por pressão		
09/12/2021	CE	FORTALE ZA	HGF	LPP EM REGIAO SACRA	Enviada	Eventos Adversos Relaciona dos à	Lesão por pressão		
09/12/2021	CE	FORTALE ZA	HGF	LPP EM REGIAO SACRA	Enviada	Eventos Adversos Relaciona dos à	Lesão por pressão		
09/12/2021	CE	FORTALE ZA	HGF	LPP EM REGIAO GLUTEA	Concluída	Eventos Adversos Relaciona dos à	Lesão por pressão		
09/12/2021	CE	FORTALE ZA	HGF	LPP EM REGIAO SACRA	Enviada	Eventos Adversos Relaciona dos à	Lesão por pressão		
09/12/2021	CE	FORTALE ZA	HGF	LPP EM REGIAO SACRA	Enviada	Eventos Adversos Relaciona dos à	Lesão por pressão		
09/12/2021	CE	FORTALE ZA	HGF	LPP EM REGIAO GLUTEA	Enviada	Eventos Adversos Relaciona dos à	Lesão por pressão		
09/12/2021	CE	FORTALE ZA	HGF	LPP EM REGIAO AXILAR MEDIA	Enviada	Eventos Adversos Relaciona dos à	Lesão por pressão		
09/12/2021	CE	FORTALE ZA	HGF	LPP EM REGIAO SACRA	Enviada	Eventos Adversos Relaciona dos à	Lesão por pressão		
09/12/2021	CE	FORTALE ZA	HGF	LPP EM REGIAO SACRA	Concluída	Eventos Adversos Relaciona dos à	Lesão por pressão		
09/12/2021	CE	FORTALE ZA	HGF	LPP EM REGIAO SACRA	Enviada	Eventos Adversos Relaciona dos à	Lesão por pressão		
09/12/2021	CE	FORTALE ZA	HGF	LPP EM REGIAO SACRA	Enviada	Eventos Adversos Relaciona dos à	Lesão por pressão		
09/12/2021	CE	FORTALE ZA	HGF	LPP EM REGIAO SACRA	Enviada	Eventos Adversos Relaciona dos à	Lesão por pressão		
09/12/2021	CE	FORTALE ZA	HGF	LPP EM REGIAO DORSAL	Enviada	Eventos Adversos Relaciona dos à	Lesão por pressão		

09/12/2021	CE	FORTALEZA	HGF	LPP EM REGIAO SACRA	Enviada	Eventos Adversos Relacionados à	Lesão por pressão		
09/12/2021	CE	FORTALEZA	HGF	GLUTEO EM REGIAO DIREITA	Enviada	Eventos Adversos Relacionados à	Lesão por pressão		
09/12/2021	CE	FORTALEZA	HGF	LPP EM REGIAO SACRA	Enviada	Eventos Adversos Relacionados à	Lesão por pressão		
09/12/2021	CE	FORTALEZA	HGF	LPP EM REGIAO SACRA	Concluída	Eventos Adversos Relacionados à	Lesão por pressão		
09/12/2021	CE	FORTALEZA	HGF	LPP EM REGIAO SACRA	Enviada	Eventos Adversos Relacionados à	Lesão por pressão		
09/12/2021	CE	FORTALEZA	HGF	LPP EM REGIAO SACRA	Concluída	Eventos Adversos Relacionados à	Lesão por pressão		
09/12/2021	CE	FORTALEZA	HGF	LPP EM REGIAO SACRA	Enviada	Eventos Adversos Relacionados à	Lesão por pressão		
09/12/2021	CE	FORTALEZA	HGF	LPP EM REGIAO SACRA	Enviada	Eventos Adversos Relacionados à	Lesão por pressão		
09/12/2021	CE	FORTALEZA	HGF	LPP EM DORSO	Enviada	Eventos Adversos Relacionados à	Lesão por pressão		
09/12/2021	CE	FORTALEZA	HGF	LPP EM CALCANEOS	Enviada	Eventos Adversos Relacionados à	Lesão por pressão		
09/12/2021	CE	FORTALEZA	HGF	LPP EM REGIAO SACRA	Enviada	Eventos Adversos Relacionados à	Lesão por pressão		
09/12/2021	CE	FORTALEZA	HGF	LPP EM REGIAO SACRA	Concluída	Eventos Adversos Relacionados à	Lesão por pressão		
09/12/2021	CE	FORTALEZA	HGF	LPP EM REGIAO CALCANEA	Enviada	Eventos Adversos Relacionados à	Lesão por pressão		
09/12/2021	CE	FORTALEZA	HGF	LPP EM REGIAO SACRA	Concluída	Eventos Adversos Relacionados à	Lesão por pressão		
09/12/2021	CE	FORTALEZA	HGF	LPP EM REGIAO SACRA	Enviada	Eventos Adversos Relacionados à	Lesão por pressão		

09/12/2021	CE	FORTALEZA	HGF	LPP EM REGIAO SACRA	Enviada	Eventos Adversos Relacionados à	Lesão por pressão		
09/12/2021	CE	FORTALEZA	HGF	LPP EM REGIAO SACRA	Enviada	Eventos Adversos Relacionados à	Lesão por pressão		
09/12/2021	CE	FORTALEZA	HGF	LPP EM REGIAO GLUTEA	Enviada	Eventos Adversos Relacionados à	Lesão por pressão		
09/12/2021	CE	FORTALEZA	HGF	LPP EM REGIAO 1	Enviada	Eventos Adversos Relacionados à	Lesão por pressão		
09/12/2021	CE	FORTALEZA	HGF	LPP EM REGIAO SACRA	Concluída	Eventos Adversos Relacionados à	Lesão por pressão		
09/12/2021	CE	FORTALEZA	HGF	LPP EM REGIAO SACRA	Enviada	Eventos Adversos Relacionados à	Lesão por pressão		
09/12/2021	CE	FORTALEZA	HGF	LPP EM REGIAO ESCAPULAR	Enviada	Eventos Adversos Relacionados à	Lesão por pressão		
09/12/2021	CE	FORTALEZA	HGF	LPP EM TORNOZELO ESQUER	Enviada	Eventos Adversos Relacionados à	Lesão por pressão		
10/12/2021	CE	FORTALEZA	HGF	LPP EM REGIAO SACRA	Enviada	Eventos Adversos Relacionados à	Lesão por pressão		
10/12/2021	CE	FORTALEZA	HGF	LPP EM CALCANEOS	Enviada	Eventos Adversos Relacionados à	Lesão por pressão		
10/12/2021	CE	FORTALEZA	HGF	LPP EM REGIAO SACRA	Enviada	Eventos Adversos Relacionados à	Lesão por pressão		
10/12/2021	CE	FORTALEZA	HGF	LPP EM TROCANTER DIREITO	Enviada	Eventos Adversos Relacionados à	Lesão por pressão		
10/12/2021	CE	FORTALEZA	HGF	LPP EM PANTURILHA DIREITA	Enviada	Eventos Adversos Relacionados à	Lesão por pressão		
10/12/2021	CE	FORTALEZA	HGF	LPP EM MALEOLO DIREITO	Enviada	Eventos Adversos Relacionados à	Lesão por pressão		
10/12/2021	CE	FORTALEZA	HGF	LPP EM REGIAO SACRA	Enviada	Eventos Adversos Relacionados à	Lesão por pressão		

10/12/2021	CE	FORTALEZA	HGF	LPP EM REGIAO GLUTEA	Enviada	Eventos Adversos Relacionados à	Lesão por pressão		
10/12/2021	CE	FORTALEZA	HGF	LPP EM REGIAO GLUTEA	Enviada	Eventos Adversos Relacionados à	Lesão por pressão		
10/12/2021	CE	FORTALEZA	HGF	LPP EM REGIAO SACRA	Enviada	Eventos Adversos Relacionados à	Lesão por pressão		
10/12/2021	CE	FORTALEZA	HGF	LPP EM REGIAO GLUTEA	Enviada	Eventos Adversos Relacionados à	Lesão por pressão		
10/12/2021	CE	FORTALEZA	HGF	LPP EM REGIAO GLUTEA	Enviada	Eventos Adversos Relacionados à	Lesão por pressão		
10/12/2021	CE	FORTALEZA	HGF	LPP EM REGIAO SACRA	Enviada	Eventos Adversos Relacionados à	Lesão por pressão		
10/12/2021	CE	FORTALEZA	HGF	LPP EM REGIAO SACRA	Enviada	Eventos Adversos Relacionados à	Lesão por pressão		
10/12/2021	CE	FORTALEZA	HGF	LPP EM CALCANEOS	Enviada	Eventos Adversos Relacionados à	Lesão por pressão		
10/12/2021	CE	FORTALEZA	HGF	LPP EM REGIAO SACRA	Enviada	Eventos Adversos Relacionados à	Lesão por pressão		
10/12/2021	CE	FORTALEZA	HGF	LPP EM REGIAO SACRA	Enviada	Eventos Adversos Relacionados à	Lesão por pressão		
10/12/2021	CE	FORTALEZA	HGF	LPP EM REGIAO SACRA	Enviada	Eventos Adversos Relacionados à	Lesão por pressão		
10/12/2021	CE	FORTALEZA	HGF	LPP EM REGIAO SACRA	Enviada	Eventos Adversos Relacionados à	Lesão por pressão		
10/12/2021	CE	FORTALEZA	HGF	LPP EM REGIAO GLUTEA	Enviada	Eventos Adversos Relacionados à	Lesão por pressão		
10/12/2021	CE	FORTALEZA	HGF	LPP EM REGIAO SACRA	Concluída	Eventos Adversos Relacionados à	Lesão por pressão		
10/12/2021	CE	FORTALEZA	HGF	LPP EM REGIAO SACRA	Concluída	Eventos Adversos Relacionados à	Lesão por pressão		

10/12/2021	CE	FORTALE ZA	HGF	LPP EM REGIAO SACRA	Enviada	Eventos Adversos Relaciona dos à	Lesão por pressão		
10/12/2021	CE	FORTALE ZA	HGF	LPP EM REGIAO SACRA	Enviada	Eventos Adversos Relaciona dos à	Lesão por pressão		
10/12/2021	CE	FORTALE ZA	HGF	LPP EM REGIAO SACRA	Enviada	Eventos Adversos Relaciona dos à	Lesão por pressão		
14/12/2021	CE	FORTALE ZA	HGF	LPP EM REGIAO SACRA	Enviada	Eventos Adversos Relaciona dos à	Lesão por pressão		
14/12/2021	CE	FORTALE ZA	HGF	LPP EM REGIAO SACRA	Enviada	Eventos Adversos Relaciona dos à	Lesão por pressão		
14/12/2021	CE	FORTALE ZA	HGF	LPP EM REGIAO SACRA	Enviada	Eventos Adversos Relaciona dos à	Lesão por pressão		
14/12/2021	CE	FORTALE ZA	HGF	LPP EM REGIAO SACRA	Enviada	Eventos Adversos Relaciona dos à	Lesão por pressão		
14/12/2021	CE	FORTALE ZA	HGF	LPP EM REGIAO SACRA	Enviada	Eventos Adversos Relaciona dos à	Lesão por pressão		
14/12/2021	CE	FORTALE ZA	HGF	LPP EM REGIAO SACRA	Enviada	Eventos Adversos Relaciona dos à	Lesão por pressão		
14/12/2021	CE	FORTALE ZA	HGF	LPP EM REGIAO SACRA	Enviada	Eventos Adversos Relaciona dos à	Lesão por pressão		
14/12/2021	CE	FORTALE ZA	HGF	LPP EM REGIAO SACRA	Enviada	Eventos Adversos Relaciona dos à	Lesão por pressão		
14/12/2021	CE	FORTALE ZA	HGF	LPP EM REGIAO SACRA	Enviada	Eventos Adversos Relaciona dos à	Lesão por pressão		
14/12/2021	CE	FORTALE ZA	HGF	LPP EM REGIAO SACRA	Enviada	Eventos Adversos Relaciona dos à	Lesão por pressão		
14/12/2021	CE	FORTALE ZA	HGF	LPP EM REGIAO SACRA	Enviada	Eventos Adversos Relaciona dos à	Lesão por pressão		
14/12/2021	CE	FORTALE ZA	HGF	LPP EM REGIAO SACRA	Enviada	Eventos Adversos Relaciona dos à	Lesão por pressão		

14/12/2021	CE	FORTALEZA	HGF	Paciente que estava sentado	Enviada	Eventos Adversos Relacionados à	Queda do paciente		
14/12/2021	CE	FORTALEZA	HGF	Paciente estava finalizando o	Enviada	Eventos Adversos Relacionados à	Queda do paciente		
14/12/2021	CE	FORTALEZA	HGF	Apresentou flebite em msd, retirado	Enviada	Eventos Adversos Relacionados à	Falhas envolvendo o cateter venoso		
14/12/2021	CE	FORTALEZA	HGF	LPP EM REGIAO SACRA	Concluída	Eventos Adversos Relacionados à	Lesão por pressão		
14/12/2021	CE	FORTALEZA	HGF	LPP NA REGIAO TROCANTER	Enviada	Eventos Adversos Relacionados à	Lesão por pressão		
14/12/2021	CE	FORTALEZA	HGF	LPP EM CALCANEOS	Concluída	Eventos Adversos Relacionados à	Lesão por pressão		
14/12/2021	CE	FORTALEZA	HGF	intoxicação por morfina devido	Enviada	Eventos Adversos Relacionados à	Falhas durante a assistência à saúde		
14/12/2021	CE	FORTALEZA	HGF	DOR, HIPEREMIA E EDEMA	Enviada	Eventos Adversos Relacionados à	Falhas envolvendo o cateter venoso		
14/12/2021	CE	FORTALEZA	HGF	DOR, HIPEREMIA, EDEMA	Enviada	Eventos Adversos Relacionados à	Falhas envolvendo o cateter venoso		
14/12/2021	CE	FORTALEZA	HGF	PACIENTE TENTOU LEVANTA	Enviada	Eventos Adversos Relacionados à	Queda do paciente		
14/12/2021	CE	FORTALEZA	HGF	LPP EM REGIAO SACRA	Enviada	Eventos Adversos Relacionados à	Lesão por pressão		
14/12/2021	CE	FORTALEZA	HGF	Laudo de tomografia de	Enviada	Eventos Adversos Relacionados à	Falha na documentação		
14/12/2021	CE	FORTALEZA	HGF	LPP EM REGIAO SACRA	Enviada	Eventos Adversos Relacionados à	Lesão por pressão		
14/12/2021	CE	FORTALEZA	HGF	LPP EM REGIAO SACRA	Enviada	Eventos Adversos Relacionados à	Lesão por pressão		
14/12/2021	CE	FORTALEZA	HGF	Cliente desorientada levanta-	Enviada	Eventos Adversos Relacionados à	Queda do paciente		

15/12/2021	CE	FORTALE ZA	HGF	LPP EM REGIAO SACRA	Enviada	Eventos Adversos Relaciona dos à	Lesão por pressão		
15/12/2021	CE	FORTALE ZA	HGF	LPP EM REGIAO SACRA	Enviada	Eventos Adversos Relaciona dos à	Lesão por pressão		
15/12/2021	CE	FORTALE ZA	HGF	LPP EM REGIAO SACRA	Enviada	Eventos Adversos Relaciona dos à	Lesão por pressão		
15/12/2021	CE	FORTALE ZA	HGF	LPP EM REGIAO SACRA	Concluída	Eventos Adversos Relaciona dos à	Lesão por pressão		
15/12/2021	CE	FORTALE ZA	HGF	LPP EM REGIAO SACRA	Enviada	Eventos Adversos Relaciona dos à	Lesão por pressão		
15/12/2021	CE	FORTALE ZA	HGF	LPP EM REGIAO SACRA	Enviada	Eventos Adversos Relaciona dos à	Lesão por pressão		
15/12/2021	CE	FORTALE ZA	HGF	LPP SACRA	Enviada	Eventos Adversos Relaciona dos à	Lesão por pressão		
15/12/2021	CE	FORTALE ZA	HGF	LPP SACRA	Enviada	Eventos Adversos Relaciona dos à	Lesão por pressão		
15/12/2021	CE	FORTALE ZA	HGF	LPP SACRA	Enviada	Eventos Adversos Relaciona dos à	Lesão por pressão		
15/12/2021	CE	FORTALE ZA	HGF	LPP SACRA	Enviada	Eventos Adversos Relaciona dos à	Lesão por pressão		
15/12/2021	CE	FORTALE ZA	HGF	LPP SACRA	Enviada	Eventos Adversos Relaciona dos à	Lesão por pressão		
15/12/2021	CE	FORTALE ZA	HGF	LPP EM REGIAO SACRA	Enviada	Eventos Adversos Relaciona dos à	Lesão por pressão		
15/12/2021	CE	FORTALE ZA	HGF	LPP SACRA	Enviada	Eventos Adversos Relaciona dos à	Lesão por pressão		
15/12/2021	CE	FORTALE ZA	HGF	LPP EM REGIAO SACRA	Enviada	Eventos Adversos Relaciona dos à	Lesão por pressão		
15/12/2021	CE	FORTALE ZA	HGF	LPP EM REGIAO SACRA	Enviada	Eventos Adversos Relaciona dos à	Lesão por pressão		

15/12/2021	CE	FORTALE ZA	HGF	LPP EM REGIAO SACRA	Enviada	Eventos Adversos Relaciona dos à	Lesão por pressão		
15/12/2021	CE	FORTALE ZA	HGF	LPP EM REGIAO SACRA	Concluída	Eventos Adversos Relaciona dos à	Lesão por pressão		
15/12/2021	CE	FORTALE ZA	HGF	LPP EM GLÚTEO ESQUER DO	Enviada	Eventos Adversos Relaciona dos à	Lesão por pressão		
15/12/2021	CE	FORTALE ZA	HGF	LPP EM REGIAO SACRA	Enviada	Eventos Adversos Relaciona dos à	Lesão por pressão		
15/12/2021	CE	FORTALE ZA	HGF	LPP SACRA	Enviada	Eventos Adversos Relaciona dos à	Lesão por pressão		
15/12/2021	CE	FORTALE ZA	HGF	LPP SACRA	Enviada	Eventos Adversos Relaciona dos à	Lesão por pressão		
15/12/2021	CE	FORTALE ZA	HGF	LPP EM REGIÃO SACRA	Enviada	Eventos Adversos Relaciona dos à	Lesão por pressão		
15/12/2021	CE	FORTALE ZA	HGF	LPP EM TROCAN TER	Enviada	Eventos Adversos Relaciona dos à	Lesão por pressão		
15/12/2021	CE	FORTALE ZA	HGF	LPP SACRA	Enviada	Eventos Adversos Relaciona dos à	Lesão por pressão		
15/12/2021	CE	FORTALE ZA	HGF	LPP SACRA	Concluída	Eventos Adversos Relaciona dos à	Lesão por pressão		
15/12/2021	CE	FORTALE ZA	HGF	LPP EM REGIAO SACRA	Enviada	Eventos Adversos Relaciona dos à	Lesão por pressão		
15/12/2021	CE	FORTALE ZA	HGF	LPP EM TROCÂN TER DIREITO	Enviada	Eventos Adversos Relaciona dos à	Lesão por pressão		
15/12/2021	CE	FORTALE ZA	HGF	LPP EM REGIAO SACRA	Concluída	Eventos Adversos Relaciona dos à	Lesão por pressão		
15/12/2021	CE	FORTALE ZA	HGF	LPP SACRA	Enviada	Eventos Adversos Relaciona dos à	Lesão por pressão		
15/12/2021	CE	FORTALE ZA	HGF	LPP SACRA	Enviada	Eventos Adversos Relaciona dos à	Lesão por pressão		

15/12/2021	CE	FORTALEZA	HGF	LPP SACRA	Enviada	Eventos Adversos Relacionados à	Lesão por pressão		
15/12/2021	CE	FORTALEZA	HGF	LPP SACRA	Enviada	Eventos Adversos Relacionados à	Lesão por pressão		
15/12/2021	CE	FORTALEZA	HGF	LPP EM REGIAO SACRA	Enviada	Eventos Adversos Relacionados à	Lesão por pressão		
15/12/2021	CE	FORTALEZA	HGF	-	Enviada	Eventos Adversos Relacionados à	Lesão por pressão		
15/12/2021	CE	FORTALEZA	HGF	LPP EM REGIAO SACRA	Concluída	Eventos Adversos Relacionados à	Lesão por pressão		
15/12/2021	CE	FORTALEZA	HGF	LPP EM GLÚTEOS	Enviada	Eventos Adversos Relacionados à	Lesão por pressão		
15/12/2021	CE	FORTALEZA	HGF	LPP EM REGIÃO SACRA	Enviada	Eventos Adversos Relacionados à	Lesão por pressão		
15/12/2021	CE	FORTALEZA	HGF	LPP EM CALCÂNEO D e E	Enviada	Eventos Adversos Relacionados à	Lesão por pressão		
15/12/2021	CE	FORTALEZA	HGF	LPP EM REGIAO SACRA	Concluída	Eventos Adversos Relacionados à	Lesão por pressão		
15/12/2021	CE	FORTALEZA	HGF	LPP EM REGIAO SACRA	Enviada	Eventos Adversos Relacionados à	Lesão por pressão		
15/12/2021	CE	FORTALEZA	HGF	LPP SACRA	Enviada	Eventos Adversos Relacionados à	Lesão por pressão		
15/12/2021	CE	FORTALEZA	HGF	LPP EM GLUTEOS	Enviada	Eventos Adversos Relacionados à	Lesão por pressão		
15/12/2021	CE	FORTALEZA	HGF	LPP EM CALCÂNEO DIREITO	Enviada	Eventos Adversos Relacionados à	Lesão por pressão		
15/12/2021	CE	FORTALEZA	HGF	LPP SACRA	Enviada	Eventos Adversos Relacionados à	Lesão por pressão		
15/12/2021	CE	FORTALEZA	HGF	-	Enviada	Eventos Adversos Relacionados à	Lesão por pressão		

15/12/2021	CE	FORTALE ZA	HGF	LPP SACRA	Enviada	Eventos Adversos Relaciona dos à	Lesão por pressão		
15/12/2021	CE	FORTALE ZA	HGF	LPP SACRA	Enviada	Eventos Adversos Relaciona dos à	Lesão por pressão		
15/12/2021	CE	FORTALE ZA	HGF	-	Enviada	Eventos Adversos Relaciona dos à	Lesão por pressão		
15/12/2021	CE	FORTALE ZA	HGF	LPP EM REGIAO SACRA	Enviada	Eventos Adversos Relaciona dos à	Lesão por pressão		
15/12/2021	CE	FORTALE ZA	HGF	LPP SACRA	Enviada	Eventos Adversos Relaciona dos à	Lesão por pressão		
15/12/2021	CE	FORTALE ZA	HGF	LPP EM CALCÂN EO DIREITO	Enviada	Eventos Adversos Relaciona dos à	Lesão por pressão		
15/12/2021	CE	FORTALE ZA	HGF	LPP SACRA	Enviada	Eventos Adversos Relaciona dos à	Lesão por pressão		
15/12/2021	CE	FORTALE ZA	HGF	lpp em cotovelo esquerdo	Enviada	Eventos Adversos Relaciona dos à	Lesão por pressão		
15/12/2021	CE	FORTALE ZA	HGF	LPP GLUTEO S	Enviada	Eventos Adversos Relaciona dos à	Lesão por pressão		
15/12/2021	CE	FORTALE ZA	HGF	LPP EM TROCAN TER DIREITO	Enviada	Eventos Adversos Relaciona dos à	Lesão por pressão		
15/12/2021	CE	FORTALE ZA	HGF	LPP EM TROCAN TER DIREITO	Enviada	Eventos Adversos Relaciona dos à	Lesão por pressão		
15/12/2021	CE	FORTALE ZA	HGF	LPP EM TROCÂN TER DIREITO	Concluída	Eventos Adversos Relaciona dos à	Lesão por pressão		
15/12/2021	CE	FORTALE ZA	HGF	LPP SACRA	Concluída	Eventos Adversos Relaciona dos à	Lesão por pressão		
15/12/2021	CE	FORTALE ZA	HGF	LPP SACRA	Enviada	Eventos Adversos Relaciona dos à	Lesão por pressão		
15/12/2021	CE	FORTALE ZA	HGF	LPP EM REGIAO SACRA	Enviada	Eventos Adversos Relaciona dos à	Lesão por pressão		

15/12/2021	CE	FORTALE ZA	HGF	LPP SACRA	Enviada	Eventos Adversos Relaciona dos à	Lesão por pressão		
15/12/2021	CE	FORTALE ZA	HGF	LPP SACRA	Enviada	Eventos Adversos Relaciona dos à	Lesão por pressão		
15/12/2021	CE	FORTALE ZA	HGF	LPP EM REGIAO SACRA	Enviada	Eventos Adversos Relaciona dos à	Lesão por pressão		
15/12/2021	CE	FORTALE ZA	HGF	LPP EM REGIAO SACRA	Enviada	Eventos Adversos Relaciona dos à	Lesão por pressão		
15/12/2021	CE	FORTALE ZA	HGF	LPP SACRA	Enviada	Eventos Adversos Relaciona dos à	Lesão por pressão		
15/12/2021	CE	FORTALE ZA	HGF	LPP SACRA	Enviada	Eventos Adversos Relaciona dos à	Lesão por pressão		
15/12/2021	CE	FORTALE ZA	HGF	LPP EM REGIAO SACRA	Enviada	Eventos Adversos Relaciona dos à	Lesão por pressão		
15/12/2021	CE	FORTALE ZA	HGF	LPP EM REGIAO SACRA	Enviada	Eventos Adversos Relaciona dos à	Lesão por pressão		
16/12/2021	CE	FORTALE ZA	HGF	LPP EM REGIAO SACRA	Enviada	Eventos Adversos Relaciona dos à	Lesão por pressão		
16/12/2021	CE	FORTALE ZA	HGF	LPP SACRA	Enviada	Eventos Adversos Relaciona dos à	Lesão por pressão		
16/12/2021	CE	FORTALE ZA	HGF	LPP EM REGIAO SACRA	Enviada	Eventos Adversos Relaciona dos à	Lesão por pressão		
16/12/2021	CE	FORTALE ZA	HGF	LPP EM REGIAO GLUTEA	Enviada	Eventos Adversos Relaciona dos à	Lesão por pressão		
16/12/2021	CE	FORTALE ZA	HGF	LPP SACRA	Enviada	Eventos Adversos Relaciona dos à	Lesão por pressão		
16/12/2021	CE	FORTALE ZA	HGF	LPP EM REGIAO SACRA	Enviada	Eventos Adversos Relaciona dos à	Lesão por pressão		
16/12/2021	CE	FORTALE ZA	HGF	LPP SACRA	Enviada	Eventos Adversos Relaciona dos à	Lesão por pressão		

16/12/2021	CE	FORTALEZA	HGF	LPP EM REGIAO SACRA	Enviada	Eventos Adversos Relacionados à	Lesão por pressão		
16/12/2021	CE	FORTALEZA	HGF	LPP SACRA	Concluída	Eventos Adversos Relacionados à	Lesão por pressão		
16/12/2021	CE	FORTALEZA	HGF	LPP SACRA	Enviada	Eventos Adversos Relacionados à	Lesão por pressão		
16/12/2021	CE	FORTALEZA	HGF	LPP SACRA	Enviada	Eventos Adversos Relacionados à	Lesão por pressão		
16/12/2021	CE	FORTALEZA	HGF	LPP SACRA	Enviada	Eventos Adversos Relacionados à	Lesão por pressão		
16/12/2021	CE	FORTALEZA	HGF	LPP EM REGIAO SACRA	Concluída	Eventos Adversos Relacionados à	Lesão por pressão		
16/12/2021	CE	FORTALEZA	HGF	LPP SACRA	Concluída	Eventos Adversos Relacionados à	Lesão por pressão		
16/12/2021	CE	FORTALEZA	HGF	LPP EM REGIAO SACRA	Enviada	Eventos Adversos Relacionados à	Lesão por pressão		
16/12/2021	CE	FORTALEZA	HGF	LPP SACRA	Concluída	Eventos Adversos Relacionados à	Lesão por pressão		
16/12/2021	CE	FORTALEZA	HGF	LPP SACRA	Enviada	Eventos Adversos Relacionados à	Lesão por pressão		
16/12/2021	CE	FORTALEZA	HGF	LPP EM REGIAO GLUTEA	Concluída	Eventos Adversos Relacionados à	Lesão por pressão		
16/12/2021	CE	FORTALEZA	HGF	LPP SACRA	Enviada	Eventos Adversos Relacionados à	Lesão por pressão		
22/12/2021	CE	FORTALEZA	HGF	LPP EM REGIAO SACRA	Enviada	Eventos Adversos Relacionados à	Lesão por pressão		
22/12/2021	CE	FORTALEZA	HGF	LPP EM REGIAO SACRA	Enviada	Eventos Adversos Relacionados à	Lesão por pressão		
22/12/2021	CE	FORTALEZA	HGF	LPP SACRA	Enviada	Eventos Adversos Relacionados à	Lesão por pressão		

22/12/2021	CE	FORTALE ZA	HGF	LPP EM REGIAO SACRA	Concluída	Eventos Adversos Relaciona dos à	Lesão por pressão		
22/12/2021	CE	FORTALE ZA	HGF	LPP SACRA	Enviada	Eventos Adversos Relaciona dos à	Lesão por pressão		
22/12/2021	CE	FORTALE ZA	HGF	LPP EM REGIAO SACRA	Enviada	Eventos Adversos Relaciona dos à	Lesão por pressão		
22/12/2021	CE	FORTALE ZA	HGF	LPP SACRA	Enviada	Eventos Adversos Relaciona dos à	Lesão por pressão		
22/12/2021	CE	FORTALE ZA	HGF	LPP EM REGIAO SACRA	Enviada	Eventos Adversos Relaciona dos à	Lesão por pressão		
22/12/2021	CE	FORTALE ZA	HGF	LPP SACRA	Enviada	Eventos Adversos Relaciona dos à	Lesão por pressão		
22/12/2021	CE	FORTALE ZA	HGF	LPP SACRA	Concluída	Eventos Adversos Relaciona dos à	Lesão por pressão		
22/12/2021	CE	FORTALE ZA	HGF	LPP EM GLÚTEO ESQUER DO	Enviada	Eventos Adversos Relaciona dos à	Lesão por pressão		
22/12/2021	CE	FORTALE ZA	HGF	SACRA	Enviada	Eventos Adversos Relaciona dos à	Lesão por pressão		
22/12/2021	CE	FORTALE ZA	HGF	LPP SACRA	Concluída	Eventos Adversos Relaciona dos à	Lesão por pressão		
22/12/2021	CE	FORTALE ZA	HGF	LPP SACRA	Enviada	Eventos Adversos Relaciona dos à	Lesão por pressão		
22/12/2021	CE	FORTALE ZA	HGF	LPP EM GLUTEO DIREITO	Enviada	Eventos Adversos Relaciona dos à	Lesão por pressão		
22/12/2021	CE	FORTALE ZA	HGF	LPP EM COTOVE LOS	Enviada	Eventos Adversos Relaciona dos à	Lesão por pressão		
22/12/2021	CE	FORTALE ZA	HGF	LPP EM REGIAO SACRA	Enviada	Eventos Adversos Relaciona dos à	Lesão por pressão		
22/12/2021	CE	FORTALE ZA	HGF	LPP EM PANTURI LHA ESQUER	Enviada	Eventos Adversos Relaciona dos à	Lesão por pressão		

22/12/2021	CE	FORTALEZA	HGF	LPP EM REGIAO SACRA	Enviada	Eventos Adversos Relacionados à	Lesão por pressão		
22/12/2021	CE	FORTALEZA	HGF	LPP EM REGIAO GLUTEA	Enviada	Eventos Adversos Relacionados à	Lesão por pressão		
22/12/2021	CE	FORTALEZA	HGF	LPP EM REGIAO SACRA	Enviada	Eventos Adversos Relacionados à	Lesão por pressão		
22/12/2021	CE	FORTALEZA	HGF	LPP EM REGIAO GLUTEA	Enviada	Eventos Adversos Relacionados à	Lesão por pressão		
22/12/2021	CE	FORTALEZA	HGF	LPP EM REGIAO SACRA	Enviada	Eventos Adversos Relacionados à	Lesão por pressão		
22/12/2021	CE	FORTALEZA	HGF	LPP EM REGIAO GLUTEA	Enviada	Eventos Adversos Relacionados à	Lesão por pressão		
22/12/2021	CE	FORTALEZA	HGF	LPP OCCIPITAL	Enviada	Eventos Adversos Relacionados à	Lesão por pressão		
22/12/2021	CE	FORTALEZA	HGF	LPP SACRA	Concluída	Eventos Adversos Relacionados à	Lesão por pressão		
22/12/2021	CE	FORTALEZA	HGF	-	Enviada	Eventos Adversos Relacionados à	Lesão por pressão		
22/12/2021	CE	FORTALEZA	HGF	LPP EM TROCÂNTER DIREITO	Concluída	Eventos Adversos Relacionados à	Lesão por pressão		
22/12/2021	CE	FORTALEZA	HGF	LPP EM REGIAO SACRA	Enviada	Eventos Adversos Relacionados à	Lesão por pressão		
22/12/2021	CE	FORTALEZA	HGF	LPP EM DORSO	Concluída	Eventos Adversos Relacionados à	Lesão por pressão		
22/12/2021	CE	FORTALEZA	HGF	LPP SACRA	Enviada	Eventos Adversos Relacionados à	Lesão por pressão		
22/12/2021	CE	FORTALEZA	HGF	LPP NA REGIAO TROCANTER	Concluída	Eventos Adversos Relacionados à	Lesão por pressão		
22/12/2021	CE	FORTALEZA	HGF	LPP SACRA	Enviada	Eventos Adversos Relacionados à	Lesão por pressão		

22/12/2021	CE	FORTALEZA	HGF	LPP EM REGIAO DORSAL	Concluída	Eventos Adversos Relacionados à	Lesão por pressão		
22/12/2021	CE	FORTALEZA	HGF	LPP SACRA	Enviada	Eventos Adversos Relacionados à	Lesão por pressão		
22/12/2021	CE	FORTALEZA	HGF	LPP EM REGIAO SACRA	Enviada	Eventos Adversos Relacionados à	Lesão por pressão		
22/12/2021	CE	FORTALEZA	HGF	LPP EM GLÚTEOS	Enviada	Eventos Adversos Relacionados à	Lesão por pressão		
22/12/2021	CE	FORTALEZA	HGF	LPP EM MALÉOLO DIREITO	Enviada	Eventos Adversos Relacionados à	Lesão por pressão		
22/12/2021	CE	FORTALEZA	HGF	LPP SACRA	Concluída	Eventos Adversos Relacionados à	Lesão por pressão		
22/12/2021	CE	FORTALEZA	HGF	LPP EM REGIAO SACRA	Enviada	Eventos Adversos Relacionados à	Lesão por pressão		
22/12/2021	CE	FORTALEZA	HGF	LPP EM TROCANTER DIREITO	Enviada	Eventos Adversos Relacionados à	Lesão por pressão		
22/12/2021	CE	FORTALEZA	HGF	LPP EM REGIAO SACRA	Enviada	Eventos Adversos Relacionados à	Lesão por pressão		
22/12/2021	CE	FORTALEZA	HGF	LPP SACRA	Enviada	Eventos Adversos Relacionados à	Lesão por pressão		
22/12/2021	CE	FORTALEZA	HGF	LPP SACRA	Enviada	Eventos Adversos Relacionados à	Lesão por pressão		
22/12/2021	CE	FORTALEZA	HGF	LPP SACRA	Enviada	Eventos Adversos Relacionados à	Lesão por pressão		
22/12/2021	CE	FORTALEZA	HGF	LPP EM REGIAO SACRA	Enviada	Eventos Adversos Relacionados à	Lesão por pressão		
22/12/2021	CE	FORTALEZA	HGF	LPP EM REGIAO SACRA	Enviada	Eventos Adversos Relacionados à	Lesão por pressão		
22/12/2021	CE	FORTALEZA	HGF	LPP EM REGIAO SACRA	Concluída	Eventos Adversos Relacionados à	Lesão por pressão		

22/12/2021	CE	FORTALEZA	HGF	LPP EM REGIAO SACRA	Enviada	Eventos Adversos Relacionados à	Lesão por pressão		
22/12/2021	CE	FORTALEZA	HGF	LPP EM REGIAO SACRA	Enviada	Eventos Adversos Relacionados à	Lesão por pressão		
22/12/2021	CE	FORTALEZA	HGF	LPP EM COTOVELO	Enviada	Eventos Adversos Relacionados à	Lesão por pressão		
22/12/2021	CE	FORTALEZA	HGF	LPP EM REGIAO SACRA	Enviada	Eventos Adversos Relacionados à	Lesão por pressão		
22/12/2021	CE	FORTALEZA	HGF	LPP EM REGIAO SACRA	Enviada	Eventos Adversos Relacionados à	Lesão por pressão		
22/12/2021	CE	FORTALEZA	HGF	LPP EM COTOVELO DIREITO	Enviada	Eventos Adversos Relacionados à	Lesão por pressão		
23/12/2021	CE	FORTALEZA	HGF	LPP SACRA	Enviada	Eventos Adversos Relacionados à	Lesão por pressão		
23/12/2021	CE	FORTALEZA	HGF	LPP SACRA	Enviada	Eventos Adversos Relacionados à	Lesão por pressão		
23/12/2021	CE	FORTALEZA	HGF	LPP EM CALCANEO DIREITO	Enviada	Eventos Adversos Relacionados à	Lesão por pressão		
23/12/2021	CE	FORTALEZA	HGF	LPP EM DORSO	Enviada	Eventos Adversos Relacionados à	Lesão por pressão		
23/12/2021	CE	FORTALEZA	HGF	LPP SACRA	Enviada	Eventos Adversos Relacionados à	Lesão por pressão		
23/12/2021	CE	FORTALEZA	HGF	LPP EM ESCAPULA DIREITA	Enviada	Eventos Adversos Relacionados à	Lesão por pressão		
23/12/2021	CE	FORTALEZA	HGF	LPP SACRA	Enviada	Eventos Adversos Relacionados à	Lesão por pressão		
23/12/2021	CE	FORTALEZA	HGF	LPP EM REGIAO SACRA	Enviada	Eventos Adversos Relacionados à	Lesão por pressão		
23/12/2021	CE	FORTALEZA	HGF	LPP EM SACRA	Enviada	Eventos Adversos Relacionados à	Lesão por pressão		

23/12/2021	CE	FORTALEZA	HGF	LPP EM REGIAO SACRA	Enviada	Eventos Adversos Relacionados à	Lesão por pressão		
23/12/2021	CE	FORTALEZA	HGF	LPP SACRA	Enviada	Eventos Adversos Relacionados à	Lesão por pressão		
23/12/2021	CE	FORTALEZA	HGF	LPP SACRA	Enviada	Eventos Adversos Relacionados à	Lesão por pressão		
23/12/2021	CE	FORTALEZA	HGF	LPP SACRA	Enviada	Eventos Adversos Relacionados à	Lesão por pressão		
23/12/2021	CE	FORTALEZA	HGF	LPP EM SACRA	Enviada	Eventos Adversos Relacionados à	Lesão por pressão		
23/12/2021	CE	FORTALEZA	HGF	LPP EM SACRA	Enviada	Eventos Adversos Relacionados à	Lesão por pressão		
23/12/2021	CE	FORTALEZA	HGF	LPP SACRA	Concluída	Eventos Adversos Relacionados à	Lesão por pressão		
23/12/2021	CE	FORTALEZA	HGF	LPP SACRA	Enviada	Eventos Adversos Relacionados à	Lesão por pressão		
23/12/2021	CE	FORTALEZA	HGF	LPP SACRA	Enviada	Eventos Adversos Relacionados à	Lesão por pressão		
23/12/2021	CE	FORTALEZA	HGF	GLÚTEO DIREITO	Enviada	Eventos Adversos Relacionados à	Lesão por pressão		
23/12/2021	CE	FORTALEZA	HGF	LPP SACRA	Enviada	Eventos Adversos Relacionados à	Lesão por pressão		
23/12/2021	CE	FORTALEZA	HGF	LPP EM CALCÂNEO	Enviada	Eventos Adversos Relacionados à	Lesão por pressão		
23/12/2021	CE	FORTALEZA	HGF	LPP SACRA	Enviada	Eventos Adversos Relacionados à	Lesão por pressão		
23/12/2021	CE	FORTALEZA	HGF	LPP SACRA	Enviada	Eventos Adversos Relacionados à	Lesão por pressão		
23/12/2021	CE	FORTALEZA	HGF	LPP EM REGIAO GLUTEA	Enviada	Eventos Adversos Relacionados à	Lesão por pressão		

23/12/2021	CE	FORTALEZA	HGF	LPP EM REGIAO SACRA	Concluída	Eventos Adversos Relacionados à	Lesão por pressão		
23/12/2021	CE	FORTALEZA	HGF	LPP SACRA	Enviada	Eventos Adversos Relacionados à	Lesão por pressão		
23/12/2021	CE	FORTALEZA	HGF	LPP SACRA	Concluída	Eventos Adversos Relacionados à	Lesão por pressão		
23/12/2021	CE	FORTALEZA	HGF	LPP EM DORSO	Enviada	Eventos Adversos Relacionados à	Lesão por pressão		
27/12/2021	CE	FORTALEZA	HGF	LPP EM REGIAO SACRA	Enviada	Eventos Adversos Relacionados à	Lesão por pressão		
27/12/2021	CE	FORTALEZA	HGF	LPP EM REGIAO SACRA	Enviada	Eventos Adversos Relacionados à	Lesão por pressão		
27/12/2021	CE	FORTALEZA	HGF	LPP EM REGIAO OCCIPITAL	Concluída	Eventos Adversos Relacionados à	Lesão por pressão		
27/12/2021	CE	FORTALEZA	HGF	LPP EM REGIAO SACRA	Concluída	Eventos Adversos Relacionados à	Lesão por pressão		
27/12/2021	CE	FORTALEZA	HGF	LPP EM REGIAO SACRA	Enviada	Eventos Adversos Relacionados à	Lesão por pressão		
27/12/2021	CE	FORTALEZA	HGF	LPP EM REGIAO SACRA	Enviada	Eventos Adversos Relacionados à	Lesão por pressão		
27/12/2021	CE	FORTALEZA	HGF	LPP EM GLÚTEOS	Enviada	Eventos Adversos Relacionados à	Lesão por pressão		
27/12/2021	CE	FORTALEZA	HGF	LPP EM REGIAO SACRA	Enviada	Eventos Adversos Relacionados à	Lesão por pressão		
27/12/2021	CE	FORTALEZA	HGF	LPP EM SACRA	Enviada	Eventos Adversos Relacionados à	Lesão por pressão		
27/12/2021	CE	FORTALEZA	HGF	LPP EM REGIAO SACRA	Enviada	Eventos Adversos Relacionados à	Lesão por pressão		
27/12/2021	CE	FORTALEZA	HGF	LPP SACRA	Enviada	Eventos Adversos Relacionados à	Lesão por pressão		

27/12/2021	CE	FORTALEZA	HGF	LPP EM REGIAO SACRA	Enviada	Eventos Adversos Relacionados à	Lesão por pressão		
27/12/2021	CE	FORTALEZA	HGF	LPP EM REGIAO GLUTEA	Enviada	Eventos Adversos Relacionados à	Lesão por pressão		
27/12/2021	CE	FORTALEZA	HGF	LPP EM DORSO	Enviada	Eventos Adversos Relacionados à	Lesão por pressão		
27/12/2021	CE	FORTALEZA	HGF	LPP EM REGIAO SACRA	Enviada	Eventos Adversos Relacionados à	Lesão por pressão		
27/12/2021	CE	FORTALEZA	HGF	LPP SACRA	Enviada	Eventos Adversos Relacionados à	Lesão por pressão		
27/12/2021	CE	FORTALEZA	HGF	lpp em escápulas	Enviada	Eventos Adversos Relacionados à	Lesão por pressão		
27/12/2021	CE	FORTALEZA	HGF	LPP SACRA	Enviada	Eventos Adversos Relacionados à	Lesão por pressão		
27/12/2021	CE	FORTALEZA	HGF	LPP EM GLÚTEOS	Enviada	Eventos Adversos Relacionados à	Lesão por pressão		
27/12/2021	CE	FORTALEZA	HGF	LPP EM COTOVELO	Concluída	Eventos Adversos Relacionados à	Lesão por pressão		
27/12/2021	CE	FORTALEZA	HGF	LPP EM REGIAO SACRA	Enviada	Eventos Adversos Relacionados à	Lesão por pressão		
27/12/2021	CE	FORTALEZA	HGF	LPP EM REGIAO GLUTEA	Enviada	Eventos Adversos Relacionados à	Lesão por pressão		
27/12/2021	CE	FORTALEZA	HGF	LPP EM REGIAO SACRA	Concluída	Eventos Adversos Relacionados à	Lesão por pressão		
27/12/2021	CE	FORTALEZA	HGF	LPP EM CALCÂNEO DIREITO	Concluída	Eventos Adversos Relacionados à	Lesão por pressão		
27/12/2021	CE	FORTALEZA	HGF	LPP EM REGIAO SACRA	Enviada	Eventos Adversos Relacionados à	Lesão por pressão		
27/12/2021	CE	FORTALEZA	HGF	LPP EM REGIAO SACRA	Enviada	Eventos Adversos Relacionados à	Lesão por pressão		

27/12/2021	CE	FORTALEZA	HGF	LPP EM REGIAO SACRA	Enviada	Eventos Adversos Relacionados à	Lesão por pressão		
27/12/2021	CE	FORTALEZA	HGF	LPP EM REGIAO SACRA	Enviada	Eventos Adversos Relacionados à	Lesão por pressão		
27/12/2021	CE	FORTALEZA	HGF	LPP EM REGIAO SACRA	Concluída	Eventos Adversos Relacionados à	Lesão por pressão		
27/12/2021	CE	FORTALEZA	HGF	LPP EM REGIAO SACRA	Concluída	Eventos Adversos Relacionados à	Lesão por pressão		
27/12/2021	CE	FORTALEZA	HGF	LPP EM GLUTEO ESQUERDO	Concluída	Eventos Adversos Relacionados à	Lesão por pressão		
28/12/2021	CE	FORTALEZA	HGF	LPP EM COTOVELOS	Concluída	Eventos Adversos Relacionados à	Lesão por pressão		
28/12/2021	CE	FORTALEZA	HGF	LPP EM REGIAO SACRA	Enviada	Eventos Adversos Relacionados à	Lesão por pressão		
28/12/2021	CE	FORTALEZA	HGF	LPP EM CALCANEO ESQUER	Enviada	Eventos Adversos Relacionados à	Lesão por pressão		
28/12/2021	CE	FORTALEZA	HGF	LPP SACRA	Enviada	Eventos Adversos Relacionados à	Lesão por pressão		
28/12/2021	CE	FORTALEZA	HGF	LPP SACRA	Enviada	Eventos Adversos Relacionados à	Lesão por pressão		
28/12/2021	CE	FORTALEZA	HGF	LPP SACRA	Enviada	Eventos Adversos Relacionados à	Lesão por pressão		
28/12/2021	CE	FORTALEZA	HGF	LPP SACRA	Enviada	Eventos Adversos Relacionados à	Lesão por pressão		
28/12/2021	CE	FORTALEZA	HGF	LPP EM DORSO	Concluída	Eventos Adversos Relacionados à	Lesão por pressão		
28/12/2021	CE	FORTALEZA	HGF	LPP EM REGIAO SACRA	Enviada	Eventos Adversos Relacionados à	Lesão por pressão		
28/12/2021	CE	FORTALEZA	HGF	LPP EM REGIAO SACRA	Enviada	Eventos Adversos Relacionados à	Lesão por pressão		

28/12/2021	CE	FORTALEZA	HGF	LPP EM REGIAO SACRA	Enviada	Eventos Adversos Relacionados à	Lesão por pressão		
28/12/2021	CE	FORTALEZA	HGF	LPP EM DORSO	Enviada	Eventos Adversos Relacionados à	Lesão por pressão		
28/12/2021	CE	FORTALEZA	HGF	LPP EM REGIAO SACRA	Enviada	Eventos Adversos Relacionados à	Lesão por pressão		
28/12/2021	CE	FORTALEZA	HGF	LPP EM REGIAO SACRA	Enviada	Eventos Adversos Relacionados à	Lesão por pressão		
28/12/2021	CE	FORTALEZA	HGF	LPP EM REGIAO SACRA	Enviada	Eventos Adversos Relacionados à	Lesão por pressão		
28/12/2021	CE	FORTALEZA	HGF	LPP EM REGIAO SACRA	Concluída	Eventos Adversos Relacionados à	Lesão por pressão		
28/12/2021	CE	FORTALEZA	HGF	LPP EM REGIAO SACRA	Enviada	Eventos Adversos Relacionados à	Lesão por pressão		
28/12/2021	CE	FORTALEZA	HGF	LPP EM REGIAO GLUTEA	Enviada	Eventos Adversos Relacionados à	Lesão por pressão		
28/12/2021	CE	FORTALEZA	HGF	LPP EM DORSO	Enviada	Eventos Adversos Relacionados à	Lesão por pressão		
29/12/2021	CE	FORTALEZA	HGF	Foi chamado um paciente	Enviada	Eventos Adversos Relacionados à	Falha na identificação do paciente		
29/12/2021	CE	FORTALEZA	HGF	Paciente caiu do leito, acompanh	Enviada	Eventos Adversos Relacionados à	Queda do paciente		
29/12/2021	CE	FORTALEZA	HGF	DOSE ALTA DE DIAZEPAN (60 MG	Enviada	Eventos Adversos Relacionados à	Falhas durante a assistência à saúde		
29/12/2021	CE	FORTALEZA	HGF	PACIENTE RELATA QUE AO	Enviada	Eventos Adversos Relacionados à	Queda do paciente		
29/12/2021	CE	FORTALEZA	HGF	LPP EM TROCANTER DIREITO	Enviada	Eventos Adversos Relacionados à	Lesão por pressão		
29/12/2021	CE	FORTALEZA	HGF	LPP EM REGIAO SACRA	Enviada	Eventos Adversos Relacionados à	Lesão por pressão		

29/12/2021	CE	FORTALEZA	HGF	LPP EM TROCANTER DIREITO	Enviada	Eventos Adversos Relacionados à	Lesão por pressão		
29/12/2021	CE	FORTALEZA	HGF	LPP EM REGIAO SACRA	Enviada	Eventos Adversos Relacionados à	Lesão por pressão		
29/12/2021	CE	FORTALEZA	HGF	LPP EM TROCANTER DIREITO	Enviada	Eventos Adversos Relacionados à	Lesão por pressão		
29/12/2021	CE	FORTALEZA	HGF	LPP EM REGIAO SACRA	Enviada	Eventos Adversos Relacionados à	Lesão por pressão		
29/12/2021	CE	FORTALEZA	HGF	LPP EM REGIAO SACRA	Enviada	Eventos Adversos Relacionados à	Lesão por pressão		
29/12/2021	CE	FORTALEZA	HGF	LPP EM REGIAO SACRA	Enviada	Eventos Adversos Relacionados à	Lesão por pressão		
29/12/2021	CE	FORTALEZA	HGF	LPP EM CALCÂNEOS D e E	Enviada	Eventos Adversos Relacionados à	Lesão por pressão		
29/12/2021	CE	FORTALEZA	HGF	LPP EM REGIAO SACRA	Enviada	Eventos Adversos Relacionados à	Lesão por pressão		
29/12/2021	CE	FORTALEZA	HGF	LPP EM REGIAO DORSAL	Concluída	Eventos Adversos Relacionados à	Lesão por pressão		
30/12/2021	CE	FORTALEZA	HGF	LPP EM REGIAO SACRA	Concluída	Eventos Adversos Relacionados à	Lesão por pressão		
30/12/2021	CE	FORTALEZA	HGF	LPP EM REGIAO SACRA	Enviada	Eventos Adversos Relacionados à	Lesão por pressão		
30/12/2021	CE	FORTALEZA	HGF	LPP EM REGIAO CALCANEA	Enviada	Eventos Adversos Relacionados à	Lesão por pressão		
30/12/2021	CE	FORTALEZA	HGF	LPP EM REGIAO SACRA	Enviada	Eventos Adversos Relacionados à	Lesão por pressão		
30/12/2021	CE	FORTALEZA	HGF	LPP EM REGIAO SACRAL	Concluída	Eventos Adversos Relacionados à	Lesão por pressão		
30/12/2021	CE	FORTALEZA	HGF	LPP EM REGIAO SACRAL	Concluída	Eventos Adversos Relacionados à	Lesão por pressão		

30/12/2021	CE	FORTALE ZA	HGF	LPP EM REGIAO SACRAL	Enviada	Eventos Adversos Relaciona dos à	Lesão por pressão		
30/12/2021	CE	FORTALE ZA	HGF	LPP EM REGIAO SACRA	Enviada	Eventos Adversos Relaciona dos à	Lesão por pressão		
30/12/2021	CE	FORTALE ZA	HGF	LPP EM DORSO	Enviada	Eventos Adversos Relaciona dos à	Lesão por pressão		
30/12/2021	CE	FORTALE ZA	HGF	LPP EM REGIAO TROCAN TAREA	Concluída	Eventos Adversos Relaciona dos à	Lesão por pressão		
30/12/2021	CE	FORTALE ZA	HGF	LPP EM REGIAO SACRA	Enviada	Eventos Adversos Relaciona dos à	Lesão por pressão		
30/12/2021	CE	FORTALE ZA	HGF	LPP EM REGIAO SACRA	Enviada	Eventos Adversos Relaciona dos à	Lesão por pressão		
30/12/2021	CE	FORTALE ZA	HGF	LPP EM REGIAO SACRA	Enviada	Eventos Adversos Relaciona dos à	Lesão por pressão		
30/12/2021	CE	FORTALE ZA	HGF	LPP EM REGIAO SACRA	Enviada	Eventos Adversos Relaciona dos à	Lesão por pressão		
30/12/2021	CE	FORTALE ZA	HGF	LPP EM REGIAO GLUTEA	Enviada	Eventos Adversos Relaciona dos à	Lesão por pressão		
30/12/2021	CE	FORTALE ZA	HGF	LPP EM REGIAO SACRA	Enviada	Eventos Adversos Relaciona dos à	Lesão por pressão		
30/12/2021	CE	FORTALE ZA	HGF	LPP EM REGIAO SACRA	Enviada	Eventos Adversos Relaciona dos à	Lesão por pressão		
30/12/2021	CE	FORTALE ZA	HGF	LPP EM REGIAO SACRA	Concluída	Eventos Adversos Relaciona dos à	Lesão por pressão		
30/12/2021	CE	FORTALE ZA	HGF	LPP EM REGIAO SACRA	Concluída	Eventos Adversos Relaciona dos à	Lesão por pressão		
30/12/2021	CE	FORTALE ZA	HGF	LPP EM REGIAO SACRA	Enviada	Eventos Adversos Relaciona dos à	Lesão por pressão		
30/12/2021	CE	FORTALE ZA	HGF	LPP EM REGIAO SACRA	Concluída	Eventos Adversos Relaciona dos à	Lesão por pressão		

30/12/2021	CE	FORTALE ZA	HGF	LPP EM REGIAO SACRA	Enviada	Eventos Adversos Relaciona dos à	Lesão por pressão		
30/12/2021	CE	FORTALE ZA	HGF	LPP EM REGIAO SACRA	Enviada	Eventos Adversos Relaciona dos à	Lesão por pressão		
30/12/2021	CE	FORTALE ZA	HGF	LPP EM REGIAO SACRA	Enviada	Eventos Adversos Relaciona dos à	Lesão por pressão		
30/12/2021	CE	FORTALE ZA	HGF	LPP EM REGIAO SACRA	Enviada	Eventos Adversos Relaciona dos à	Lesão por pressão		
30/12/2021	CE	FORTALE ZA	HGF	LPP EM REGIAO SACRA	Enviada	Eventos Adversos Relaciona dos à	Lesão por pressão		
30/12/2021	CE	FORTALE ZA	HGF	LPP EM REGIAO SACRA	Enviada	Eventos Adversos Relaciona dos à	Lesão por pressão		
30/12/2021	CE	FORTALE ZA	HGF	LPP EM REGIAO SACRA	Enviada	Eventos Adversos Relaciona dos à	Lesão por pressão		
03/01/2022	CE	FORTALE ZA	HGF	LPP SACRA	Enviada	Eventos Adversos Relaciona dos à	Lesão por pressão		
03/01/2022	CE	FORTALE ZA	HGF	LPP SACRA	Enviada	Eventos Adversos Relaciona dos à	Lesão por pressão		
03/01/2022	CE	FORTALE ZA	HGF	LPP SACRA	Enviada	Eventos Adversos Relaciona dos à	Lesão por pressão		
03/01/2022	CE	FORTALE ZA	HGF	LPP SACRA	Enviada	Eventos Adversos Relaciona dos à	Lesão por pressão		
03/01/2022	CE	FORTALE ZA	HGF	LPP SACRA	Enviada	Eventos Adversos Relaciona dos à	Lesão por pressão		
03/01/2022	CE	FORTALE ZA	HGF	LPP SACRA	Enviada	Eventos Adversos Relaciona dos à	Lesão por pressão		
03/01/2022	CE	FORTALE ZA	HGF	LPP SACRA	Enviada	Eventos Adversos Relaciona dos à	Lesão por pressão		
03/01/2022	CE	FORTALE ZA	HGF	LPP SACRA	Enviada	Eventos Adversos Relaciona dos à	Lesão por pressão		

03/01/2022	CE	FORTALEZA	HGF	LPP EM DORSO	Enviada	Eventos Adversos Relacionados à	Lesão por pressão		
03/01/2022	CE	FORTALEZA	HGF	LPP SACRA	Enviada	Eventos Adversos Relacionados à	Lesão por pressão		
03/01/2022	CE	FORTALEZA	HGF	LPP SACRA	Enviada	Eventos Adversos Relacionados à	Lesão por pressão		
03/01/2022	CE	FORTALEZA	HGF	LPP SACRA	Enviada	Eventos Adversos Relacionados à	Lesão por pressão		
03/01/2022	CE	FORTALEZA	HGF	LPP SACRA	Enviada	Eventos Adversos Relacionados à	Lesão por pressão		
03/01/2022	CE	FORTALEZA	HGF	LPP SACRA	Enviada	Eventos Adversos Relacionados à	Lesão por pressão		
03/01/2022	CE	FORTALEZA	HGF	LPP SACRA	Enviada	Eventos Adversos Relacionados à	Lesão por pressão		
03/01/2022	CE	FORTALEZA	HGF	LP SACRA	Enviada	Eventos Adversos Relacionados à	Lesão por pressão		
03/01/2022	CE	FORTALEZA	HGF	LP SACRA	Enviada	Eventos Adversos Relacionados à	Lesão por pressão		
03/01/2022	CE	FORTALEZA	HGF	LPP SACRA	Enviada	Eventos Adversos Relacionados à	Lesão por pressão		
03/01/2022	CE	FORTALEZA	HGF	LPP EM GLÚTEO	Enviada	Eventos Adversos Relacionados à	Lesão por pressão		
03/01/2022	CE	FORTALEZA	HGF	LPP SACRA	Enviada	Eventos Adversos Relacionados à	Lesão por pressão		
03/01/2022	CE	FORTALEZA	HGF	LPP SACRA	Enviada	Eventos Adversos Relacionados à	Lesão por pressão		
03/01/2022	CE	FORTALEZA	HGF	LPP SACRA	Enviada	Eventos Adversos Relacionados à	Lesão por pressão		
03/01/2022	CE	FORTALEZA	HGF	-	Enviada	Eventos Adversos Relacionados à	Lesão por pressão		

03/01/2022	CE	FORTALEZA	HGF	LPP OCCIPTAL	Enviada	Eventos Adversos Relacionados à	Lesão por pressão		
03/01/2022	CE	FORTALEZA	HGF	LPP SACRA	Enviada	Eventos Adversos Relacionados à	Lesão por pressão		
03/01/2022	CE	FORTALEZA	HGF	LP TROCANTER	Enviada	Eventos Adversos Relacionados à	Lesão por pressão		
03/01/2022	CE	FORTALEZA	HGF	LPP EM DORSO	Enviada	Eventos Adversos Relacionados à	Lesão por pressão		
03/01/2022	CE	FORTALEZA	HGF	LPP SACRA	Enviada	Eventos Adversos Relacionados à	Lesão por pressão		
03/01/2022	CE	FORTALEZA	HGF	LPP SACRA	Enviada	Eventos Adversos Relacionados à	Lesão por pressão		
03/01/2022	CE	FORTALEZA	HGF	LPP SACRA	Enviada	Eventos Adversos Relacionados à	Lesão por pressão		
04/01/2022	CE	FORTALEZA	HGF	LPP SACRA	Enviada	Eventos Adversos Relacionados à	Lesão por pressão		
04/01/2022	CE	FORTALEZA	HGF	LPP SACRA	Enviada	Eventos Adversos Relacionados à	Lesão por pressão		
04/01/2022	CE	FORTALEZA	HGF	LPP SACRA	Enviada	Eventos Adversos Relacionados à	Lesão por pressão		
04/01/2022	CE	FORTALEZA	HGF	LPP SACRA	Enviada	Eventos Adversos Relacionados à	Lesão por pressão		
04/01/2022	CE	FORTALEZA	HGF	LPP SACRA	Enviada	Eventos Adversos Relacionados à	Lesão por pressão		
04/01/2022	CE	FORTALEZA	HGF	LPP SACRA	Enviada	Eventos Adversos Relacionados à	Lesão por pressão		
04/01/2022	CE	FORTALEZA	HGF	LPP EM CALCÂNEO DIREITO	Enviada	Eventos Adversos Relacionados à	Lesão por pressão		
04/01/2022	CE	FORTALEZA	HGF	LPP EM CALCÂNEO ESQUER	Enviada	Eventos Adversos Relacionados à	Lesão por pressão		

04/01/2022	CE	FORTALEZA	HGF	LPP EM CALCÂNEO ESQUER	Enviada	Eventos Adversos Relacionados à	Lesão por pressão		
04/01/2022	CE	FORTALEZA	HGF	-	Enviada	Eventos Adversos Relacionados à	Lesão por pressão		
04/01/2022	CE	FORTALEZA	HGF	LPP EM CALCÂNEO DIREITO	Enviada	Eventos Adversos Relacionados à	Lesão por pressão		
04/01/2022	CE	FORTALEZA	HGF	LPP SACRA	Enviada	Eventos Adversos Relacionados à	Lesão por pressão		
04/01/2022	CE	FORTALEZA	HGF	LPP SACRA	Enviada	Eventos Adversos Relacionados à	Lesão por pressão		
04/01/2022	CE	FORTALEZA	HGF	LPP SACRA	Enviada	Eventos Adversos Relacionados à	Lesão por pressão		
04/01/2022	CE	FORTALEZA	HGF	LPP SACRA	Enviada	Eventos Adversos Relacionados à	Lesão por pressão		
04/01/2022	CE	FORTALEZA	HGF	LPP EM TROCÂNTER DIREITO	Enviada	Eventos Adversos Relacionados à	Lesão por pressão		
04/01/2022	CE	FORTALEZA	HGF	LPP EM GLÚTEOS	Enviada	Eventos Adversos Relacionados à	Lesão por pressão		
04/01/2022	CE	FORTALEZA	HGF	LPP SACRA	Enviada	Eventos Adversos Relacionados à	Lesão por pressão		
04/01/2022	CE	FORTALEZA	HGF	LPP EM CALCÂNEO DIREITO	Enviada	Eventos Adversos Relacionados à	Lesão por pressão		
04/01/2022	CE	FORTALEZA	HGF	LPP SACRA	Enviada	Eventos Adversos Relacionados à	Lesão por pressão		
04/01/2022	CE	FORTALEZA	HGF	LPP SACRA	Enviada	Eventos Adversos Relacionados à	Lesão por pressão		
04/01/2022	CE	FORTALEZA	HGF	LPP SACRA	Enviada	Eventos Adversos Relacionados à	Lesão por pressão		
04/01/2022	CE	FORTALEZA	HGF	LPP SACRA	Enviada	Eventos Adversos Relacionados à	Lesão por pressão		

04/01/2022	CE	FORTALEZA	HGF	LPP SACRA	Enviada	Eventos Adversos Relacionados à	Lesão por pressão		
04/01/2022	CE	FORTALEZA	HGF	-	Enviada	Eventos Adversos Relacionados à	Lesão por pressão		
04/01/2022	CE	FORTALEZA	HGF	-	Enviada	Eventos Adversos Relacionados à	Lesão por pressão		
04/01/2022	CE	FORTALEZA	HGF	LPP SACRA	Enviada	Eventos Adversos Relacionados à	Lesão por pressão		
04/01/2022	CE	FORTALEZA	HGF	LPP SACRA	Enviada	Eventos Adversos Relacionados à	Lesão por pressão		
04/01/2022	CE	FORTALEZA	HGF	LPP SACRA	Enviada	Eventos Adversos Relacionados à	Lesão por pressão		
04/01/2022	CE	FORTALEZA	HGF	-	Enviada	Eventos Adversos Relacionados à	Lesão por pressão		
04/01/2022	CE	FORTALEZA	HGF	-	Enviada	Eventos Adversos Relacionados à	Lesão por pressão		
04/01/2022	CE	FORTALEZA	HGF	LPP EM GLÚTEOS	Enviada	Eventos Adversos Relacionados à	Lesão por pressão		
04/01/2022	CE	FORTALEZA	HGF	LPP SACRA	Enviada	Eventos Adversos Relacionados à	Lesão por pressão		
04/01/2022	CE	FORTALEZA	HGF	LPP EM TROCÂNTER DIREITO	Enviada	Eventos Adversos Relacionados à	Lesão por pressão		
04/01/2022	CE	FORTALEZA	HGF	LPP EM TROCÂNTER DIREITO	Enviada	Eventos Adversos Relacionados à	Lesão por pressão		
04/01/2022	CE	FORTALEZA	HGF	LPP INTEGLÚTEO	Enviada	Eventos Adversos Relacionados à	Lesão por pressão		
04/01/2022	CE	FORTALEZA	HGF	LPP SACRA	Enviada	Eventos Adversos Relacionados à	Lesão por pressão		
04/01/2022	CE	FORTALEZA	HGF	LPP SACRA	Enviada	Eventos Adversos Relacionados à	Lesão por pressão		

04/01/2022	CE	FORTALEZA	HGF	LPP COTOVELOS	Enviada	Eventos Adversos Relacionados à	Lesão por pressão		
05/01/2022	CE	FORTALEZA	HGF	LPP EM REGIAO SACRA	Enviada	Eventos Adversos Relacionados à	Lesão por pressão		
07/01/2022	CE	FORTALEZA	HGF	LPP SACRA	Enviada	Eventos Adversos Relacionados à	Lesão por pressão		
07/01/2022	CE	FORTALEZA	HGF	LPP SACRA	Enviada	Eventos Adversos Relacionados à	Lesão por pressão		
07/01/2022	CE	FORTALEZA	HGF	LPP SACRA	Enviada	Eventos Adversos Relacionados à	Lesão por pressão		
07/01/2022	CE	FORTALEZA	HGF	LESAO POR ADESIVO EM	Enviada	Eventos Adversos Relacionados à	Lesão por pressão		
07/01/2022	CE	FORTALEZA	HGF	LPP EM GLÚTEO DIREITO	Enviada	Eventos Adversos Relacionados à	Lesão por pressão		
07/01/2022	CE	FORTALEZA	HGF	LPP SACRA	Enviada	Eventos Adversos Relacionados à	Lesão por pressão		
07/01/2022	CE	FORTALEZA	HGF	LPP SACRA	Enviada	Eventos Adversos Relacionados à	Lesão por pressão		
07/01/2022	CE	FORTALEZA	HGF	LPP SACRA	Enviada	Eventos Adversos Relacionados à	Lesão por pressão		
07/01/2022	CE	FORTALEZA	HGF	LPP SACRA	Enviada	Eventos Adversos Relacionados à	Lesão por pressão		
07/01/2022	CE	FORTALEZA	HGF	LPP EM GLÚTEOS	Enviada	Eventos Adversos Relacionados à	Lesão por pressão		
07/01/2022	CE	FORTALEZA	HGF	LPP EM GLÚTEOS	Enviada	Eventos Adversos Relacionados à	Lesão por pressão		
07/01/2022	CE	FORTALEZA	HGF	LPP EM GLÚTEO ESQUERDO	Enviada	Eventos Adversos Relacionados à	Lesão por pressão		
07/01/2022	CE	FORTALEZA	HGF	LPP SACRA	Enviada	Eventos Adversos Relacionados à	Lesão por pressão		

07/01/2022	CE	FORTALE ZA	HGF	LPP SACRA	Enviada	Eventos Adversos Relaciona dos à	Lesão por pressão		
07/01/2022	CE	FORTALE ZA	HGF	LPP SACRA	Enviada	Eventos Adversos Relaciona dos à	Lesão por pressão		
07/01/2022	CE	FORTALE ZA	HGF	LPP SACRA	Enviada	Eventos Adversos Relaciona dos à	Lesão por pressão		
07/01/2022	CE	FORTALE ZA	HGF	TROCÂN TER DIREITO	Enviada	Eventos Adversos Relaciona dos à	Lesão por pressão		
07/01/2022	CE	FORTALE ZA	HGF	LPP EM CALCÂN EO D e E	Enviada	Eventos Adversos Relaciona dos à	Lesão por pressão		
07/01/2022	CE	FORTALE ZA	HGF	LPP SACRA	Enviada	Eventos Adversos Relaciona dos à	Lesão por pressão		
10/01/2022	CE	FORTALE ZA	HGF	LPP SACRA	Enviada	Eventos Adversos Relaciona dos à	Lesão por pressão		
10/01/2022	CE	FORTALE ZA	HGF	LPP SACRA	Enviada	Eventos Adversos Relaciona dos à	Lesão por pressão		
10/01/2022	CE	FORTALE ZA	HGF	LPP SACRA	Enviada	Eventos Adversos Relaciona dos à	Lesão por pressão		
10/01/2022	CE	FORTALE ZA	HGF	LPP SACRA	Enviada	Eventos Adversos Relaciona dos à	Lesão por pressão		
10/01/2022	CE	FORTALE ZA	HGF	LPP SACRA	Enviada	Eventos Adversos Relaciona dos à	Lesão por pressão		
10/01/2022	CE	FORTALE ZA	HGF	LPP SACRA	Enviada	Eventos Adversos Relaciona dos à	Lesão por pressão		
10/01/2022	CE	FORTALE ZA	HGF	LPP SACRA	Enviada	Eventos Adversos Relaciona dos à	Lesão por pressão		
11/01/2022	CE	FORTALE ZA	HGF	Administr ado NBZ-Atrovent + berotec	Enviada	Eventos Adversos Relaciona dos à	Falhas durante a assistênci a à saúde		
11/01/2022	CE	FORTALE ZA	HGF	Flebite em MSD	Enviada	Eventos Adversos Relaciona dos à	Falhas envolvend o cateter venoso		

11/01/2022	CE	FORTALEZA	HGF	Flebite em MSE	Enviada	Eventos Adversos Relacionados à	Falhas envolvendo cateter venoso		
27/01/2022	CE	FORTALEZA	HGF	Flebite em MSE	Enviada	Eventos Adversos Relacionados à	Falhas envolvendo cateter venoso		
27/01/2022	CE	FORTALEZA	HGF	FLEBITE MSD	Enviada	Eventos Adversos Relacionados à	Falhas envolvendo cateter venoso		
03/02/2022	CE	FORTALEZA	HGF	Paciente agitado, caiu do leito	Enviada	Eventos Adversos Relacionados à	Queda do paciente		
03/02/2022	CE	FORTALEZA	HGF	Paciente agitado passou por cima	Enviada	Eventos Adversos Relacionados à	Queda do paciente		
03/02/2022	CE	FORTALEZA	HGF	Paciente agitado e desorientado caiu	Enviada	Eventos Adversos Relacionados à	Queda do paciente		
03/02/2022	CE	FORTALEZA	HGF	DURANTE A VISITA DE	Enviada	Eventos Adversos Relacionados à	Falhas envolvendo cateter venoso		
03/02/2022	CE	FORTALEZA	HGF	PCT CAIU DA PROPRIA	Enviada	Eventos Adversos Relacionados à	Queda do paciente		
16/02/2022	CE	FORTALEZA	HGF	LPP EM REGIAO GLUTEA	Enviada	Eventos Adversos Relacionados à	Lesão por pressão		
16/02/2022	CE	FORTALEZA	HGF	LPP EM REGIAO SACRA	Enviada	Eventos Adversos Relacionados à	Lesão por pressão		
16/02/2022	CE	FORTALEZA	HGF	LPP EM REGIAO SACRA	Enviada	Eventos Adversos Relacionados à	Lesão por pressão		
16/02/2022	CE	FORTALEZA	HGF	LPP EM REGIAO SACRA	Enviada	Eventos Adversos Relacionados à	Lesão por pressão		
16/02/2022	CE	FORTALEZA	HGF	LPP EM REGIAO SACRA	Enviada	Eventos Adversos Relacionados à	Lesão por pressão		
16/02/2022	CE	FORTALEZA	HGF	LPP EM REGIAO SACRA	Enviada	Eventos Adversos Relacionados à	Lesão por pressão		
16/02/2022	CE	FORTALEZA	HGF	LPP EM REGIAO SACRA	Enviada	Eventos Adversos Relacionados à	Lesão por pressão		

16/02/2022	CE	FORTALE ZA	HGF	LPP EM REGIAO SACRA	Concluída	Eventos Adversos Relaciona dos à	Lesão por pressão		
16/02/2022	CE	FORTALE ZA	HGF	LPP EM REGIAO SACRA	Enviada	Eventos Adversos Relaciona dos à	Lesão por pressão		
18/02/2022	CE	FORTALE ZA	HGF	LPP EM REGIAO SACRA	Enviada	Eventos Adversos Relaciona dos à	Lesão por pressão		
18/02/2022	CE	FORTALE ZA	HGF	LPP EM REGIAO SACRA	Enviada	Eventos Adversos Relaciona dos à	Lesão por pressão		
18/02/2022	CE	FORTALE ZA	HGF	BITURI ELÉTRIC O SOLTOU	Enviada	Eventos Adversos Relaciona dos à	Queimadu ra de paciente		
21/02/2022	CE	FORTALE ZA	HGF	LPP EM REGIAO GLUTEA	Enviada	Eventos Adversos Relaciona dos à	Lesão por pressão		
21/02/2022	CE	FORTALE ZA	HGF	LPP EM TROCAN TER DIREITO	Enviada	Eventos Adversos Relaciona dos à	Lesão por pressão		
21/02/2022	CE	FORTALE ZA	HGF	LPP EM REGIAO SACRA	Concluída	Eventos Adversos Relaciona dos à	Lesão por pressão		
21/02/2022	CE	FORTALE ZA	HGF	LPP EM REGIAO SACRA	Enviada	Eventos Adversos Relaciona dos à	Lesão por pressão		
21/02/2022	CE	FORTALE ZA	HGF	LPP EM REGIAO GLUTEA	Enviada	Eventos Adversos Relaciona dos à	Lesão por pressão		
21/02/2022	CE	FORTALE ZA	HGF	-	Enviada	Eventos Adversos Relaciona dos à	Lesão por pressão		
21/02/2022	CE	FORTALE ZA	HGF	LPP EM REGIAO SACRA	Enviada	Eventos Adversos Relaciona dos à	Lesão por pressão		
21/02/2022	CE	FORTALE ZA	HGF	LPP EM REGIAO SACRA	Enviada	Eventos Adversos Relaciona dos à	Lesão por pressão		
21/02/2022	CE	FORTALE ZA	HGF	-	Enviada	Eventos Adversos Relaciona dos à	Lesão por pressão		
21/02/2022	CE	FORTALE ZA	HGF	LPP EM REGIAO SACRA	Enviada	Eventos Adversos Relaciona dos à	Lesão por pressão		

21/02/2022	CE	FORTALEZA	HGF	LPP EM REGIAO GLUTEA	Enviada	Eventos Adversos Relacionados à	Lesão por pressão		
21/02/2022	CE	FORTALEZA	HGF	LPP EM REGIAO SACRA	Enviada	Eventos Adversos Relacionados à	Lesão por pressão		
21/02/2022	CE	FORTALEZA	HGF	Administrado insulina em	Enviada	Eventos Adversos Relacionados à	Falhas durante a assistência à saúde		
21/02/2022	CE	FORTALEZA	HGF	-	Enviada	Eventos Adversos Relacionados à	Falhas durante a assistência à saúde		
21/02/2022	CE	FORTALEZA	HGF	LPP EM REGIAO SACRA	Enviada	Eventos Adversos Relacionados à	Lesão por pressão		
22/02/2022	CE	FORTALEZA	HGF	LPP EM REGIAO GLUTEA	Enviada	Eventos Adversos Relacionados à	Lesão por pressão		
22/02/2022	CE	FORTALEZA	HGF	LPP EM REGIAO SACRA	Enviada	Eventos Adversos Relacionados à	Lesão por pressão		
22/02/2022	CE	FORTALEZA	HGF	SACRA	Enviada	Eventos Adversos Relacionados à	Lesão por pressão		
22/02/2022	CE	FORTALEZA	HGF	LPP EM REGIAO GLUTEA	Enviada	Eventos Adversos Relacionados à	Lesão por pressão		
22/02/2022	CE	FORTALEZA	HGF	LPP EM REGIAO SACRA	Enviada	Eventos Adversos Relacionados à	Lesão por pressão		
22/02/2022	CE	FORTALEZA	HGF	LPP EM REGIAO SACRA	Enviada	Eventos Adversos Relacionados à	Lesão por pressão		
22/02/2022	CE	FORTALEZA	HGF	LPP EM CALCANEO ESQUER	Enviada	Eventos Adversos Relacionados à	Lesão por pressão		
22/02/2022	CE	FORTALEZA	HGF	LPP EM REGIAO GLUTEA	Enviada	Eventos Adversos Relacionados à	Lesão por pressão		
22/02/2022	CE	FORTALEZA	HGF	LPP EM GLUTEO ESQUERDO	Enviada	Eventos Adversos Relacionados à	Lesão por pressão		
22/02/2022	CE	FORTALEZA	HGF	LPP EM REGIAO SACRA	Enviada	Eventos Adversos Relacionados à	Lesão por pressão		

22/02/2022	CE	FORTALE ZA	HGF	LPP EM REGIAO SACRA	Enviada	Eventos Adversos Relaciona dos à	Lesão por pressão		
22/02/2022	CE	FORTALE ZA	HGF	LPP EM REGIAO SACRA	Enviada	Eventos Adversos Relaciona dos à	Lesão por pressão		
22/02/2022	CE	FORTALE ZA	HGF	LPP EM REGIAO SACRA	Enviada	Eventos Adversos Relaciona dos à	Lesão por pressão		
22/02/2022	CE	FORTALE ZA	HGF	LPP EM REGIAO SACRA	Enviada	Eventos Adversos Relaciona dos à	Lesão por pressão		
22/02/2022	CE	FORTALE ZA	HGF	LPP EM REGIAO SACRA	Enviada	Eventos Adversos Relaciona dos à	Lesão por pressão		
22/02/2022	CE	FORTALE ZA	HGF	LPP EM REGIAO SACRA	Enviada	Eventos Adversos Relaciona dos à	Lesão por pressão		
22/02/2022	CE	FORTALE ZA	HGF	LPP EM REGIAO SACRA	Enviada	Eventos Adversos Relaciona dos à	Lesão por pressão		
22/02/2022	CE	FORTALE ZA	HGF	LPP EM REGIAO SACRA	Enviada	Eventos Adversos Relaciona dos à	Lesão por pressão		
22/02/2022	CE	FORTALE ZA	HGF	LPP EM REGIAO SACRA	Enviada	Eventos Adversos Relaciona dos à	Lesão por pressão		
22/02/2022	CE	FORTALE ZA	HGF	LPP EM REGIAO SACRA	Enviada	Eventos Adversos Relaciona dos à	Lesão por pressão		
22/02/2022	CE	FORTALE ZA	HGF	LPP EM REGIAO SACRA	Enviada	Eventos Adversos Relaciona dos à	Lesão por pressão		
22/02/2022	CE	FORTALE ZA	HGF	LPP EM REGIAO SACRA	Enviada	Eventos Adversos Relaciona dos à	Lesão por pressão		
22/02/2022	CE	FORTALE ZA	HGF	LPP EM REGIAO SACRA	Enviada	Eventos Adversos Relaciona dos à	Lesão por pressão		
22/02/2022	CE	FORTALE ZA	HGF	LPP EM REGIAO SACRA	Enviada	Eventos Adversos Relaciona dos à	Lesão por pressão		
22/02/2022	CE	FORTALE ZA	HGF	LPP EM REGIAO SACRA	Enviada	Eventos Adversos Relaciona dos à	Lesão por pressão		

22/02/2022	CE	FORTALEZA	HGF	LPP EM REGIAO SACRA	Enviada	Eventos Adversos Relacionados à	Lesão por pressão		
22/02/2022	CE	FORTALEZA	HGF	LPP EM REGIAO SACRA	Enviada	Eventos Adversos Relacionados à	Lesão por pressão		
22/02/2022	CE	FORTALEZA	HGF	LPP EM REGIAO SACRA	Enviada	Eventos Adversos Relacionados à	Lesão por pressão		
22/02/2022	CE	FORTALEZA	HGF	LPP EM REGIAO SACRA	Enviada	Eventos Adversos Relacionados à	Lesão por pressão		
22/02/2022	CE	FORTALEZA	HGF	LPP EM DORSO	Enviada	Eventos Adversos Relacionados à	Lesão por pressão		
23/02/2022	CE	FORTALEZA	HGF	LPP EM REGIAO SACRA	Enviada	Eventos Adversos Relacionados à	Lesão por pressão		
23/02/2022	CE	FORTALEZA	HGF	LPP EM COTOVELO	Enviada	Eventos Adversos Relacionados à	Lesão por pressão		
23/02/2022	CE	FORTALEZA	HGF	LPP EM REGIAO SACRA	Enviada	Eventos Adversos Relacionados à	Lesão por pressão		
23/02/2022	CE	FORTALEZA	HGF	LPP EM TROCANTER DIREITO	Enviada	Eventos Adversos Relacionados à	Lesão por pressão		
23/02/2022	CE	FORTALEZA	HGF	LPP EM REGIAO SACRA	Enviada	Eventos Adversos Relacionados à	Lesão por pressão		
23/02/2022	CE	FORTALEZA	HGF	LPP EM REGIAO SACRA	Enviada	Eventos Adversos Relacionados à	Lesão por pressão		
23/02/2022	CE	FORTALEZA	HGF	LPP EM REGIAO SACRA	Enviada	Eventos Adversos Relacionados à	Lesão por pressão		
23/02/2022	CE	FORTALEZA	HGF	LPP EM REGIAO SACRA	Enviada	Eventos Adversos Relacionados à	Lesão por pressão		
23/02/2022	CE	FORTALEZA	HGF	LPP EM REGIAO SACRA	Enviada	Eventos Adversos Relacionados à	Lesão por pressão		
23/02/2022	CE	FORTALEZA	HGF	LPP EM REGIAO SACRA	Enviada	Eventos Adversos Relacionados à	Lesão por pressão		

23/02/2022	CE	FORTALEZA	HGF	LPP EM REGIAO SACRA	Enviada	Eventos Adversos Relacionados à	Lesão por pressão		
23/02/2022	CE	FORTALEZA	HGF	LPP EM REGIAO SACRA	Enviada	Eventos Adversos Relacionados à	Lesão por pressão		
23/02/2022	CE	FORTALEZA	HGF	LPP SACRA	Enviada	Eventos Adversos Relacionados à	Lesão por pressão		
24/02/2022	CE	FORTALEZA	HGF	LPP EM REGIAO SACRA	Enviada	Eventos Adversos Relacionados à	Lesão por pressão		
24/02/2022	CE	FORTALEZA	HGF	LPP EM REGIAO SACRA	Enviada	Eventos Adversos Relacionados à	Lesão por pressão		
24/02/2022	CE	FORTALEZA	HGF	LPP EM REGIAO SACRA	Enviada	Eventos Adversos Relacionados à	Lesão por pressão		
24/02/2022	CE	FORTALEZA	HGF	LPP EM REGIAO SACRA	Enviada	Eventos Adversos Relacionados à	Lesão por pressão		
25/02/2022	CE	FORTALEZA	HGF	LPP EM REGIAO SACRA	Enviada	Eventos Adversos Relacionados à	Lesão por pressão		
25/02/2022	CE	FORTALEZA	HGF	LPP EM REGIAO GLUTEA	Enviada	Eventos Adversos Relacionados à	Lesão por pressão		
25/02/2022	CE	FORTALEZA	HGF	LPP EM REGIAO SACRA	Enviada	Eventos Adversos Relacionados à	Lesão por pressão		
25/02/2022	CE	FORTALEZA	HGF	LPP EM REGIAO SACRA	Enviada	Eventos Adversos Relacionados à	Lesão por pressão		
25/02/2022	CE	FORTALEZA	HGF	LPP EM REGIAO SACRA	Enviada	Eventos Adversos Relacionados à	Lesão por pressão		
25/02/2022	CE	FORTALEZA	HGF	LPP EM REGIAO SACRA	Concluída	Eventos Adversos Relacionados à	Lesão por pressão		
25/02/2022	CE	FORTALEZA	HGF	LPP EM REGIAO SACRA	Enviada	Eventos Adversos Relacionados à	Lesão por pressão		
25/02/2022	CE	FORTALEZA	HGF	LPP EM REGIAO SACRA	Enviada	Eventos Adversos Relacionados à	Lesão por pressão		

25/02/2022	CE	FORTALEZA	HGF	LPP EM REGIAO SACRA	Enviada	Eventos Adversos Relacionados à	Lesão por pressão		
25/02/2022	CE	FORTALEZA	HGF	LPP EM REGIAO SACRA	Enviada	Eventos Adversos Relacionados à	Lesão por pressão		
25/02/2022	CE	FORTALEZA	HGF	LPP EM REGIAO SACRA	Enviada	Eventos Adversos Relacionados à	Lesão por pressão		
25/02/2022	CE	FORTALEZA	HGF	LPP EM TROCANTER ESQUER	Enviada	Eventos Adversos Relacionados à	Lesão por pressão		
25/02/2022	CE	FORTALEZA	HGF	LPP EM GLUTEO ESQUEDO	Enviada	Eventos Adversos Relacionados à	Lesão por pressão		
25/02/2022	CE	FORTALEZA	HGF	LPP EM REGIAO SACRA	Enviada	Eventos Adversos Relacionados à	Lesão por pressão		
25/02/2022	CE	FORTALEZA	HGF	LPP EM REGIAO GLUTEA	Enviada	Eventos Adversos Relacionados à	Lesão por pressão		
25/02/2022	CE	FORTALEZA	HGF	LPP EM REGIAO SACRA	Enviada	Eventos Adversos Relacionados à	Lesão por pressão		
25/02/2022	CE	FORTALEZA	HGF	LPP EM REGIAO DORSAL	Enviada	Eventos Adversos Relacionados à	Lesão por pressão		
25/02/2022	CE	FORTALEZA	HGF	LPP EM REGIAO CALCANEA	Enviada	Eventos Adversos Relacionados à	Lesão por pressão		
25/02/2022	CE	FORTALEZA	HGF	LPP EM REGIAO SACRA	Enviada	Eventos Adversos Relacionados à	Lesão por pressão		
25/02/2022	CE	FORTALEZA	HGF	LPP EM REGIAO SACRA	Enviada	Eventos Adversos Relacionados à	Lesão por pressão		
25/02/2022	CE	FORTALEZA	HGF	LPP EM REGIAO SACRA	Enviada	Eventos Adversos Relacionados à	Lesão por pressão		
25/02/2022	CE	FORTALEZA	HGF	LPP EM REGIAO SACRA	Enviada	Eventos Adversos Relacionados à	Lesão por pressão		
25/02/2022	CE	FORTALEZA	HGF	LPP EM REGIAO SACRA	Enviada	Eventos Adversos Relacionados à	Lesão por pressão		

25/02/2022	CE	FORTALEZA	HGF	LPP EM REGIAO SACRA	Enviada	Eventos Adversos Relacionados à	Lesão por pressão		
25/02/2022	CE	FORTALEZA	HGF	LPP EM REGIAO SACRA	Enviada	Eventos Adversos Relacionados à	Lesão por pressão		
25/02/2022	CE	FORTALEZA	HGF	LPP EM REGIAO SACRA	Enviada	Eventos Adversos Relacionados à	Lesão por pressão		
25/02/2022	CE	FORTALEZA	HGF	LPP EM REGIAO SACRA	Concluída	Eventos Adversos Relacionados à	Lesão por pressão		
25/02/2022	CE	FORTALEZA	HGF	LPP EM REGIAO SACRA	Enviada	Eventos Adversos Relacionados à	Lesão por pressão		
25/02/2022	CE	FORTALEZA	HGF	LPP EM REGIAO SACRA	Enviada	Eventos Adversos Relacionados à	Lesão por pressão		
25/02/2022	CE	FORTALEZA	HGF	LPP EM GLUTEO ESQUEDO	Enviada	Eventos Adversos Relacionados à	Lesão por pressão		
25/02/2022	CE	FORTALEZA	HGF	LPP EM GLUTEO DIREITO	Enviada	Eventos Adversos Relacionados à	Lesão por pressão		
25/02/2022	CE	FORTALEZA	HGF	LPP EM REGIAO SACRA	Enviada	Eventos Adversos Relacionados à	Lesão por pressão		
25/02/2022	CE	FORTALEZA	HGF	LPP EM GLUTEO DIREITO	Concluída	Eventos Adversos Relacionados à	Lesão por pressão		
25/02/2022	CE	FORTALEZA	HGF	LPP EM REGIAO SACRA	Enviada	Eventos Adversos Relacionados à	Lesão por pressão		
25/02/2022	CE	FORTALEZA	HGF	LPP EM REGIAO SACRA	Enviada	Eventos Adversos Relacionados à	Lesão por pressão		
25/02/2022	CE	FORTALEZA	HGF	LPP EM REGIAO SACRA	Enviada	Eventos Adversos Relacionados à	Lesão por pressão		
25/02/2022	CE	FORTALEZA	HGF	LPP EM REGIAO SACRA	Enviada	Eventos Adversos Relacionados à	Lesão por pressão		
28/02/2022	CE	FORTALEZA	HGF	Paciente relata que ao sentar na	Enviada	Eventos Adversos Relacionados à	Acidentes do paciente	Força contundente (esmaga	

28/02/2022	CE	FORTALEZA	HGF	APRESENTANDO RUBOR, DOR E	Enviada	Eventos Adversos Relacionados à	Falhas envolvendo o cateter venoso		
28/02/2022	CE	FORTALEZA	HGF	PACIENTE DESEQUILIBROU	Enviada	Eventos Adversos Relacionados à	Queda do paciente		
28/02/2022	CE	FORTALEZA	HGF	FLEBITE MSD , REALIZADO A	Enviada	Eventos Adversos Relacionados à	Falhas envolvendo o cateter venoso		
28/02/2022	CE	FORTALEZA	HGF	ADMINISTRADO MEDICAMENTO	Enviada	Eventos Adversos Relacionados à	Falhas durante a assistência à saúde		
28/02/2022	CE	FORTALEZA	HGF	FLEBITE EM MSD	Enviada	Eventos Adversos Relacionados à	Falhas envolvendo o cateter venoso		
28/02/2022	CE	FORTALEZA	HGF	VAZAO DE BICS INADEQUADA DE	Enviada	Eventos Adversos Relacionados à	Falhas durante a assistência à saúde		
28/02/2022	CE	FORTALEZA	HGF	Pct apresentou flebite no MSE	Enviada	Eventos Adversos Relacionados à	Falhas envolvendo o cateter venoso		
28/02/2022	CE	FORTALEZA	HGF	Pneumotorax em hemitórax direito	Enviada	Eventos Adversos Relacionados à	Falhas envolvendo o cateter venoso		
28/02/2022	CE	FORTALEZA	HGF	Medicamento prescrito com	Enviada	Eventos Adversos Relacionados à	Falhas durante a assistência à saúde		
28/02/2022	CE	FORTALEZA	HGF	Paciente retirou sonda nasoenter	Enviada	Eventos Adversos Relacionados à	Falhas envolvendo sondas		
28/02/2022	CE	FORTALEZA	HGF	PACIENTE ESTAVA REALIZA	Enviada	Eventos Adversos Relacionados à	Queda do paciente		
28/02/2022	CE	FORTALEZA	HGF	-	Enviada	Eventos Adversos Relacionados à	Queda do paciente		
09/03/2022	CE	FORTALEZA	HGF	Paciente em dupla coagulação em	Enviada	Eventos Adversos Relacionados à	Falhas durante a assistência à saúde		
09/03/2022	CE	FORTALEZA	HGF	PERCA DE QUIMIOTERÁPICO	Enviada	Eventos Adversos Relacionados à	Falhas durante a assistência à saúde		
09/03/2022	CE	FORTALEZA	HGF	Realizado medicação	Enviada	Eventos Adversos Relacionados à	Falhas durante a assistência à saúde		

11/03/2022	CE	FORTALEZA	HGF	PERCA ACIDENTAL DE D.V.E	Enviada	Eventos Adversos Relacionados à	Falhas durante a assistência à saúde		
30/03/2022	CE	FORTALEZA	HGF	EQUIPE DE ENFERMAGEM	Enviada	Eventos Adversos Relacionados à	Broncoaspiração		
30/03/2022	CE	FORTALEZA	HGF	Paciente hematológico apresento	Concluída	Eventos Adversos Relacionados à	Falhas durante a assistência à saúde		
30/03/2022	CE	FORTALEZA	HGF	PACIENTE CONSCIE	Enviada	Eventos Adversos Relacionados à	Queda do paciente		
31/03/2022	CE	FORTALEZA	HGF	A paciente relata que na	Enviada	Eventos Adversos Relacionados à	Queda do paciente		
31/03/2022	CE	FORTALEZA	HGF	Flebite MSE	Enviada	Eventos Adversos Relacionados à	Falhas envolvendo o cateter venoso		
31/03/2022	CE	FORTALEZA	HGF	Paciente em uso em uso de	Enviada	Eventos Adversos Relacionados à	Falhas durante a assistência à saúde		
31/03/2022	CE	FORTALEZA	HGF	PRESCRITO ALBUMINA PARA	Enviada	Eventos Adversos Relacionados à	Falhas durante a assistência à saúde		
31/03/2022	CE	FORTALEZA	HGF	Paciente caiu do leito por momento	Enviada	Eventos Adversos Relacionados à	Queda do paciente		
31/03/2022	CE	FORTALEZA	HGF	PACIENTE DESORIENTADO	Enviada	Eventos Adversos Relacionados à	Queda do paciente		
31/03/2022	CE	FORTALEZA	HGF	PACIENTE SOFRE QUEDA	Enviada	Eventos Adversos Relacionados à	Queda do paciente		
31/03/2022	CE	FORTALEZA	HGF	Flebite em MSE	Enviada	Eventos Adversos Relacionados à	Falhas envolvendo o cateter venoso		
31/03/2022	CE	FORTALEZA	HGF	Flebite MSE	Enviada	Eventos Adversos Relacionados à	Falhas envolvendo o cateter venoso		
31/03/2022	CE	FORTALEZA	HGF	Flebite em Picc	Enviada	Eventos Adversos Relacionados à	Falhas envolvendo o cateter venoso		
31/03/2022	CE	FORTALEZA	HGF	ADMINISTRADO 50 ML DE	Enviada	Eventos Adversos Relacionados à	Falhas durante a assistência à saúde		

07/04/2022	CE	FORTALEZA	HGF	Sofreu queda da própria altura ao	Enviada	Eventos Adversos Relacionados à	Queda do paciente		
07/04/2022	CE	FORTALEZA	HGF	AO REALIZAR O TRANSP	Enviada	Eventos Adversos Relacionados à	Falhas durante a assistência à saúde		
12/04/2022	CE	FORTALEZA	HGF	SACRA	Enviada	Eventos Adversos Relacionados à	Lesão por pressão		
12/04/2022	CE	FORTALEZA	HGF	CALCÂNEOS	Enviada	Eventos Adversos Relacionados à	Lesão por pressão		
12/04/2022	CE	FORTALEZA	HGF	SACRA	Concluída	Eventos Adversos Relacionados à	Lesão por pressão		
12/04/2022	CE	FORTALEZA	HGF	PAVILHAO AURICULAR	Enviada	Eventos Adversos Relacionados à	Lesão por pressão		
12/04/2022	CE	FORTALEZA	HGF	SACRA	Concluída	Eventos Adversos Relacionados à	Lesão por pressão		
12/04/2022	CE	FORTALEZA	HGF	SACRA	Enviada	Eventos Adversos Relacionados à	Lesão por pressão		
12/04/2022	CE	FORTALEZA	HGF	TROCÂNTER DIREITO	Enviada	Eventos Adversos Relacionados à	Lesão por pressão		
12/04/2022	CE	FORTALEZA	HGF	TROCANTER ESQUERDO	Enviada	Eventos Adversos Relacionados à	Lesão por pressão		
12/04/2022	CE	FORTALEZA	HGF	SACRA	Enviada	Eventos Adversos Relacionados à	Lesão por pressão		
12/04/2022	CE	FORTALEZA	HGF	PAVILHAO AURICULAR	Enviada	Eventos Adversos Relacionados à	Lesão por pressão		
12/04/2022	CE	FORTALEZA	HGF	TROCÂNTER DIREITO	Enviada	Eventos Adversos Relacionados à	Lesão por pressão		
12/04/2022	CE	FORTALEZA	HGF	INTERGLÚTEO	Enviada	Eventos Adversos Relacionados à	Lesão por pressão		
12/04/2022	CE	FORTALEZA	HGF	SACRA	Enviada	Eventos Adversos Relacionados à	Lesão por pressão		

12/04/2022	CE	FORTALEZA	HGF	SACRA	Concluída	Eventos Adversos Relacionados à	Lesão por pressão		
12/04/2022	CE	FORTALEZA	HGF	GLÚTEOS	Enviada	Eventos Adversos Relacionados à	Lesão por pressão		
12/04/2022	CE	FORTALEZA	HGF	DORSO	Enviada	Eventos Adversos Relacionados à	Lesão por pressão		
12/04/2022	CE	FORTALEZA	HGF	Flebite em MSD	Enviada	Eventos Adversos Relacionados à	Falhas envolvendo o cateter venoso		
12/04/2022	CE	FORTALEZA	HGF	MALEOLO ESQUERDO	Concluída	Eventos Adversos Relacionados à	Lesão por pressão		
12/04/2022	CE	FORTALEZA	HGF	SACRA	Concluída	Eventos Adversos Relacionados à	Lesão por pressão		
12/04/2022	CE	FORTALEZA	HGF	DORSO	Enviada	Eventos Adversos Relacionados à	Lesão por pressão		
12/04/2022	CE	FORTALEZA	HGF	Flebite em MSD	Enviada	Eventos Adversos Relacionados à	Falhas envolvendo o cateter venoso		
12/04/2022	CE	FORTALEZA	HGF	SACRA	Enviada	Eventos Adversos Relacionados à	Lesão por pressão		
12/04/2022	CE	FORTALEZA	HGF	SACRA	Enviada	Eventos Adversos Relacionados à	Lesão por pressão		
12/04/2022	CE	FORTALEZA	HGF	SACRA	Enviada	Eventos Adversos Relacionados à	Lesão por pressão		
12/04/2022	CE	FORTALEZA	HGF	SACRA	Enviada	Eventos Adversos Relacionados à	Lesão por pressão		
12/04/2022	CE	FORTALEZA	HGF	SACRA	Concluída	Eventos Adversos Relacionados à	Lesão por pressão		
12/04/2022	CE	FORTALEZA	HGF	SACRA	Enviada	Eventos Adversos Relacionados à	Lesão por pressão		
12/04/2022	CE	FORTALEZA	HGF	COTOVELO DIREITO	Enviada	Eventos Adversos Relacionados à	Lesão por pressão		

12/04/2022	CE	FORTALEZA	HGF	CALCÂNEO ESQUERDO	Enviada	Eventos Adversos Relacionados à	Lesão por pressão		
12/04/2022	CE	FORTALEZA	HGF	TROCÂNTER DIREITO	Enviada	Eventos Adversos Relacionados à	Lesão por pressão		
12/04/2022	CE	FORTALEZA	HGF	SACRA	Enviada	Eventos Adversos Relacionados à	Lesão por pressão		
12/04/2022	CE	FORTALEZA	HGF	GLÚTEO DIREITO	Concluída	Eventos Adversos Relacionados à	Lesão por pressão		
12/04/2022	CE	FORTALEZA	HGF	CALCÂNEOS	Enviada	Eventos Adversos Relacionados à	Lesão por pressão		
12/04/2022	CE	FORTALEZA	HGF	SACRA	Enviada	Eventos Adversos Relacionados à	Lesão por pressão		
12/04/2022	CE	FORTALEZA	HGF	SACRA	Enviada	Eventos Adversos Relacionados à	Lesão por pressão		
12/04/2022	CE	FORTALEZA	HGF	SACRA	Enviada	Eventos Adversos Relacionados à	Lesão por pressão		
12/04/2022	CE	FORTALEZA	HGF	SACRA	Enviada	Eventos Adversos Relacionados à	Lesão por pressão		
12/04/2022	CE	FORTALEZA	HGF	SACRA	Concluída	Eventos Adversos Relacionados à	Lesão por pressão		
12/04/2022	CE	FORTALEZA	HGF	GLÚTEOS	Concluída	Eventos Adversos Relacionados à	Lesão por pressão		
12/04/2022	CE	FORTALEZA	HGF	CALCÂNEO DIREITO	Concluída	Eventos Adversos Relacionados à	Lesão por pressão		
12/04/2022	CE	FORTALEZA	HGF	GLÚTEOS	Concluída	Eventos Adversos Relacionados à	Lesão por pressão		
12/04/2022	CE	FORTALEZA	HGF	CALCÂNEO ESQUERDO	Enviada	Eventos Adversos Relacionados à	Lesão por pressão		
12/04/2022	CE	FORTALEZA	HGF	Flebite msd	Enviada	Eventos Adversos Relacionados à	Falhas envolvendo o cateter venoso		

12/04/2022	CE	FORTALEZA	HGF	FLEBITE MSE	Enviada	Eventos Adversos Relacionados à	Falhas envolvendo cateter venoso		
13/04/2022	CE	FORTALEZA	HGF	GLÚTEO DIREITO	Concluída	Eventos Adversos Relacionados à	Lesão por pressão		
13/04/2022	CE	FORTALEZA	HGF	SACRA	Concluída	Eventos Adversos Relacionados à	Lesão por pressão		
13/04/2022	CE	FORTALEZA	HGF	SACRA	Concluída	Eventos Adversos Relacionados à	Lesão por pressão		
13/04/2022	CE	FORTALEZA	HGF	SACRA	Concluída	Eventos Adversos Relacionados à	Lesão por pressão		
13/04/2022	CE	FORTALEZA	HGF	SACRA	Enviada	Eventos Adversos Relacionados à	Lesão por pressão		
13/04/2022	CE	FORTALEZA	HGF	TROCANTER ESQUERDO	Enviada	Eventos Adversos Relacionados à	Lesão por pressão		
13/04/2022	CE	FORTALEZA	HGF	SACRA	Enviada	Eventos Adversos Relacionados à	Lesão por pressão		
13/04/2022	CE	FORTALEZA	HGF	SACRA	Enviada	Eventos Adversos Relacionados à	Lesão por pressão		
13/04/2022	CE	FORTALEZA	HGF	SACRA	Enviada	Eventos Adversos Relacionados à	Lesão por pressão		
13/04/2022	CE	FORTALEZA	HGF	SACRA	Enviada	Eventos Adversos Relacionados à	Lesão por pressão		
13/04/2022	CE	FORTALEZA	HGF	SACRA	Enviada	Eventos Adversos Relacionados à	Lesão por pressão		
13/04/2022	CE	FORTALEZA	HGF	SACRA	Enviada	Eventos Adversos Relacionados à	Lesão por pressão		
13/04/2022	CE	FORTALEZA	HGF	SACRA	Enviada	Eventos Adversos Relacionados à	Lesão por pressão		
13/04/2022	CE	FORTALEZA	HGF	GLÚTEOS	Enviada	Eventos Adversos Relacionados à	Lesão por pressão		

13/04/2022	CE	FORTALEZA	HGF	SACRA	Enviada	Eventos Adversos Relacionados à	Lesão por pressão		
13/04/2022	CE	FORTALEZA	HGF	SACRA	Enviada	Eventos Adversos Relacionados à	Lesão por pressão		
13/04/2022	CE	FORTALEZA	HGF	SACRA	Enviada	Eventos Adversos Relacionados à	Lesão por pressão		
13/04/2022	CE	FORTALEZA	HGF	COTOVELO DIREITO	Enviada	Eventos Adversos Relacionados à	Lesão por pressão		
13/04/2022	CE	FORTALEZA	HGF	SACRA	Enviada	Eventos Adversos Relacionados à	Lesão por pressão		
13/04/2022	CE	FORTALEZA	HGF	SACRA	Enviada	Eventos Adversos Relacionados à	Lesão por pressão		
13/04/2022	CE	FORTALEZA	HGF	SACRA	Enviada	Eventos Adversos Relacionados à	Lesão por pressão		
13/04/2022	CE	FORTALEZA	HGF	GLÚTEO ESQUERDO	Enviada	Eventos Adversos Relacionados à	Lesão por pressão		
13/04/2022	CE	FORTALEZA	HGF	SACRA	Enviada	Eventos Adversos Relacionados à	Lesão por pressão		
13/04/2022	CE	FORTALEZA	HGF	GLÚTEO DIREITO	Enviada	Eventos Adversos Relacionados à	Lesão por pressão		
13/04/2022	CE	FORTALEZA	HGF	GLÚTEO DIREITO	Enviada	Eventos Adversos Relacionados à	Lesão por pressão		
13/04/2022	CE	FORTALEZA	HGF	SACRA	Enviada	Eventos Adversos Relacionados à	Lesão por pressão		
13/04/2022	CE	FORTALEZA	HGF	GLÚTEO ESQUERDO	Enviada	Eventos Adversos Relacionados à	Lesão por pressão		
13/04/2022	CE	FORTALEZA	HGF	TROCÂNTER DIREITO	Concluída	Eventos Adversos Relacionados à	Lesão por pressão		
13/04/2022	CE	FORTALEZA	HGF	OCCIPTAL	Concluída	Eventos Adversos Relacionados à	Lesão por pressão		

13/04/2022	CE	FORTALEZA	HGF	SACRA	Enviada	Eventos Adversos Relacionados à	Lesão por pressão		
13/04/2022	CE	FORTALEZA	HGF	SACRA	Enviada	Eventos Adversos Relacionados à	Lesão por pressão		
13/04/2022	CE	FORTALEZA	HGF	GLÚTEO DIREITO	Enviada	Eventos Adversos Relacionados à	Lesão por pressão		
13/04/2022	CE	FORTALEZA	HGF	GLÚTEOS	Enviada	Eventos Adversos Relacionados à	Lesão por pressão		
13/04/2022	CE	FORTALEZA	HGF	SACRA	Concluída	Eventos Adversos Relacionados à	Lesão por pressão		
13/04/2022	CE	FORTALEZA	HGF	SACRA	Enviada	Eventos Adversos Relacionados à	Lesão por pressão		
13/04/2022	CE	FORTALEZA	HGF	ESCAPULA ESQUERDA	Enviada	Eventos Adversos Relacionados à	Lesão por pressão		
13/04/2022	CE	FORTALEZA	HGF	OUTRO LOCAL	Enviada	Eventos Adversos Relacionados à	Lesão por pressão		
13/04/2022	CE	FORTALEZA	HGF	TÓRAX	Enviada	Eventos Adversos Relacionados à	Lesão por pressão		
13/04/2022	CE	FORTALEZA	HGF	CALCANEO ESQUERDO	Enviada	Eventos Adversos Relacionados à	Lesão por pressão		
13/04/2022	CE	FORTALEZA	HGF	DORSO	Enviada	Eventos Adversos Relacionados à	Lesão por pressão		
13/04/2022	CE	FORTALEZA	HGF	GLÚTEOS	Enviada	Eventos Adversos Relacionados à	Lesão por pressão		
13/04/2022	CE	FORTALEZA	HGF	SACRA	Concluída	Eventos Adversos Relacionados à	Lesão por pressão		
13/04/2022	CE	FORTALEZA	HGF	SACRA	Enviada	Eventos Adversos Relacionados à	Lesão por pressão		
13/04/2022	CE	FORTALEZA	HGF	OUTRO LOCAL	Enviada	Eventos Adversos Relacionados à	Lesão por pressão		

13/04/2022	CE	FORTALEZA	HGF	GLÚTEO DIREITO	Enviada	Eventos Adversos Relacionados à	Lesão por pressão		
13/04/2022	CE	FORTALEZA	HGF	INTERGLÚTEOS	Enviada	Eventos Adversos Relacionados à	Lesão por pressão		
13/04/2022	CE	FORTALEZA	HGF	SACRA	Concluída	Eventos Adversos Relacionados à	Lesão por pressão		
13/04/2022	CE	FORTALEZA	HGF	GLÚTEOS	Enviada	Eventos Adversos Relacionados à	Lesão por pressão		
13/04/2022	CE	FORTALEZA	HGF	CALCÂNEO DIREITO	Enviada	Eventos Adversos Relacionados à	Lesão por pressão		
13/04/2022	CE	FORTALEZA	HGF	GLÚTEOS	Enviada	Eventos Adversos Relacionados à	Lesão por pressão		
13/04/2022	CE	FORTALEZA	HGF	GLÚTEOS	Enviada	Eventos Adversos Relacionados à	Lesão por pressão		
13/04/2022	CE	FORTALEZA	HGF	GLÚTEOS	Enviada	Eventos Adversos Relacionados à	Lesão por pressão		
13/04/2022	CE	FORTALEZA	HGF	GLÚTEO ESQUERDO	Enviada	Eventos Adversos Relacionados à	Lesão por pressão		
13/04/2022	CE	FORTALEZA	HGF	GLÚTEOS	Enviada	Eventos Adversos Relacionados à	Lesão por pressão		
13/04/2022	CE	FORTALEZA	HGF	DORSO	Enviada	Eventos Adversos Relacionados à	Lesão por pressão		
13/04/2022	CE	FORTALEZA	HGF	GLÚTEOS	Enviada	Eventos Adversos Relacionados à	Lesão por pressão		
13/04/2022	CE	FORTALEZA	HGF	SACRA	Enviada	Eventos Adversos Relacionados à	Lesão por pressão		
13/04/2022	CE	FORTALEZA	HGF	CALCANEO ESQUERDO	Enviada	Eventos Adversos Relacionados à	Lesão por pressão		
13/04/2022	CE	FORTALEZA	HGF	SACRA	Concluída	Eventos Adversos Relacionados à	Lesão por pressão		

13/04/2022	CE	FORTALEZA	HGF	SACRA	Enviada	Eventos Adversos Relacionados à	Lesão por pressão		
13/04/2022	CE	FORTALEZA	HGF	COTOVELO DIREITO	Enviada	Eventos Adversos Relacionados à	Lesão por pressão		
13/04/2022	CE	FORTALEZA	HGF	JOELHO DIREITO	Enviada	Eventos Adversos Relacionados à	Lesão por pressão		
13/04/2022	CE	FORTALEZA	HGF	SACRA	Concluída	Eventos Adversos Relacionados à	Lesão por pressão		
13/04/2022	CE	FORTALEZA	HGF	ESCÁPULA DIREITA	Enviada	Eventos Adversos Relacionados à	Lesão por pressão		
13/04/2022	CE	FORTALEZA	HGF	SACRA	Enviada	Eventos Adversos Relacionados à	Lesão por pressão		
13/04/2022	CE	FORTALEZA	HGF	CALCÂNEO DIREITO	Enviada	Eventos Adversos Relacionados à	Lesão por pressão		
13/04/2022	CE	FORTALEZA	HGF	SACRA	Enviada	Eventos Adversos Relacionados à	Lesão por pressão		
13/04/2022	CE	FORTALEZA	HGF	SACRA	Enviada	Eventos Adversos Relacionados à	Lesão por pressão		
13/04/2022	CE	FORTALEZA	HGF	SACRA	Enviada	Eventos Adversos Relacionados à	Lesão por pressão		
13/04/2022	CE	FORTALEZA	HGF	SACRA	Enviada	Eventos Adversos Relacionados à	Lesão por pressão		
13/04/2022	CE	FORTALEZA	HGF	SACRA	Enviada	Eventos Adversos Relacionados à	Lesão por pressão		
13/04/2022	CE	FORTALEZA	HGF	SACRA	Enviada	Eventos Adversos Relacionados à	Lesão por pressão		
13/04/2022	CE	FORTALEZA	HGF	GLÚTEOS	Enviada	Eventos Adversos Relacionados à	Lesão por pressão		
13/04/2022	CE	FORTALEZA	HGF	TROCÂNTER DIREITO	Enviada	Eventos Adversos Relacionados à	Lesão por pressão		

13/04/2022	CE	FORTALEZA	HGF	CALCÂNEO DIREITO	Enviada	Eventos Adversos Relacionados à	Lesão por pressão		
13/04/2022	CE	FORTALEZA	HGF	GLÚTEOS	Enviada	Eventos Adversos Relacionados à	Lesão por pressão		
13/04/2022	CE	FORTALEZA	HGF	SACRA	Enviada	Eventos Adversos Relacionados à	Lesão por pressão		
13/04/2022	CE	FORTALEZA	HGF	SACRA	Concluída	Eventos Adversos Relacionados à	Lesão por pressão		
13/04/2022	CE	FORTALEZA	HGF	SACRA	Enviada	Eventos Adversos Relacionados à	Lesão por pressão		
13/04/2022	CE	FORTALEZA	HGF	GLÚTEO DIREITO	Enviada	Eventos Adversos Relacionados à	Lesão por pressão		
13/04/2022	CE	FORTALEZA	HGF	SACRA	Enviada	Eventos Adversos Relacionados à	Lesão por pressão		
13/04/2022	CE	FORTALEZA	HGF	GLÚTEO ESQUERDO	Enviada	Eventos Adversos Relacionados à	Lesão por pressão		
13/04/2022	CE	FORTALEZA	HGF	SACRA	Concluída	Eventos Adversos Relacionados à	Lesão por pressão		
13/04/2022	CE	FORTALEZA	HGF	PANTURRILHA	Concluída	Eventos Adversos Relacionados à	Lesão por pressão		
13/04/2022	CE	FORTALEZA	HGF	TROCÂNTER DIREITO	Concluída	Eventos Adversos Relacionados à	Lesão por pressão		
13/04/2022	CE	FORTALEZA	HGF	SACRA	Enviada	Eventos Adversos Relacionados à	Lesão por pressão		
13/04/2022	CE	FORTALEZA	HGF	SACRA	Concluída	Eventos Adversos Relacionados à	Lesão por pressão		
13/04/2022	CE	FORTALEZA	HGF	SACRA	Enviada	Eventos Adversos Relacionados à	Lesão por pressão		
13/04/2022	CE	FORTALEZA	HGF	GLÚTEO DIREITO	Enviada	Eventos Adversos Relacionados à	Lesão por pressão		

13/04/2022	CE	FORTALEZA	HGF	SACRA	Enviada	Eventos Adversos Relacionados à	Lesão por pressão		
13/04/2022	CE	FORTALEZA	HGF	GLÚTEO ESQUERDO	Enviada	Eventos Adversos Relacionados à	Lesão por pressão		
13/04/2022	CE	FORTALEZA	HGF	SACRA	Enviada	Eventos Adversos Relacionados à	Lesão por pressão		
13/04/2022	CE	FORTALEZA	HGF	GLÚTEO DIREITO	Enviada	Eventos Adversos Relacionados à	Lesão por pressão		
13/04/2022	CE	FORTALEZA	HGF	GLÚTEO DIREITO	Enviada	Eventos Adversos Relacionados à	Lesão por pressão		
13/04/2022	CE	FORTALEZA	HGF	GLÚTEO ESQUERDO	Enviada	Eventos Adversos Relacionados à	Lesão por pressão		
13/04/2022	CE	FORTALEZA	HGF	SACRA	Enviada	Eventos Adversos Relacionados à	Lesão por pressão		
13/04/2022	CE	FORTALEZA	HGF	SACRA	Enviada	Eventos Adversos Relacionados à	Lesão por pressão		
13/04/2022	CE	FORTALEZA	HGF	SACRA	Enviada	Eventos Adversos Relacionados à	Lesão por pressão		
13/04/2022	CE	FORTALEZA	HGF	SACRA	Enviada	Eventos Adversos Relacionados à	Lesão por pressão		
13/04/2022	CE	FORTALEZA	HGF	SACRA	Enviada	Eventos Adversos Relacionados à	Lesão por pressão		
13/04/2022	CE	FORTALEZA	HGF	PANTURRILHA DIREITA	Enviada	Eventos Adversos Relacionados à	Lesão por pressão		
13/04/2022	CE	FORTALEZA	HGF	PANTURRILHA ESQUERDA	Enviada	Eventos Adversos Relacionados à	Lesão por pressão		
13/04/2022	CE	FORTALEZA	HGF	SACRA	Concluída	Eventos Adversos Relacionados à	Lesão por pressão		
13/04/2022	CE	FORTALEZA	HGF	SACRA	Enviada	Eventos Adversos Relacionados à	Lesão por pressão		

13/04/2022	CE	FORTALEZA	HGF	GLÚTEOS	Concluída	Eventos Adversos Relacionados à	Lesão por pressão		
13/04/2022	CE	FORTALEZA	HGF	SACRA	Concluída	Eventos Adversos Relacionados à	Lesão por pressão		
13/04/2022	CE	FORTALEZA	HGF	GLÚTEOS	Enviada	Eventos Adversos Relacionados à	Lesão por pressão		
13/04/2022	CE	FORTALEZA	HGF	CALCANEO ESQUERDO	Enviada	Eventos Adversos Relacionados à	Lesão por pressão		
13/04/2022	CE	FORTALEZA	HGF	COTOVELOS	Enviada	Eventos Adversos Relacionados à	Lesão por pressão		
13/04/2022	CE	FORTALEZA	HGF	PANTURRILHA DIREITA	Enviada	Eventos Adversos Relacionados à	Lesão por pressão		
13/04/2022	CE	FORTALEZA	HGF	COTOVELO DIREITO	Enviada	Eventos Adversos Relacionados à	Lesão por pressão		
13/04/2022	CE	FORTALEZA	HGF	SACRA	Enviada	Eventos Adversos Relacionados à	Lesão por pressão		
13/04/2022	CE	FORTALEZA	HGF	SACRA	Concluída	Eventos Adversos Relacionados à	Lesão por pressão		
13/04/2022	CE	FORTALEZA	HGF	CALCÂNEO DIREITO	Enviada	Eventos Adversos Relacionados à	Lesão por pressão		
13/04/2022	CE	FORTALEZA	HGF	SACRA	Enviada	Eventos Adversos Relacionados à	Lesão por pressão		
13/04/2022	CE	FORTALEZA	HGF	SACRA	Enviada	Eventos Adversos Relacionados à	Lesão por pressão		
13/04/2022	CE	FORTALEZA	HGF	SACRA	Enviada	Eventos Adversos Relacionados à	Lesão por pressão		
13/04/2022	CE	FORTALEZA	HGF	SACRA	Concluída	Eventos Adversos Relacionados à	Lesão por pressão		
13/04/2022	CE	FORTALEZA	HGF	CALCÂNEOS	Enviada	Eventos Adversos Relacionados à	Lesão por pressão		

13/04/2022	CE	FORTALEZA	HGF	SACRA	Enviada	Eventos Adversos Relacionados à	Lesão por pressão		
13/04/2022	CE	FORTALEZA	HGF	GLÚTEOS	Enviada	Eventos Adversos Relacionados à	Lesão por pressão		
13/04/2022	CE	FORTALEZA	HGF	CALCANEO ESQUERDO	Enviada	Eventos Adversos Relacionados à	Lesão por pressão		
13/04/2022	CE	FORTALEZA	HGF	SACRA	Enviada	Eventos Adversos Relacionados à	Lesão por pressão		
13/04/2022	CE	FORTALEZA	HGF	MALÉOLO DIREITO	Enviada	Eventos Adversos Relacionados à	Lesão por pressão		
13/04/2022	CE	FORTALEZA	HGF	SACRA	Enviada	Eventos Adversos Relacionados à	Lesão por pressão		
13/04/2022	CE	FORTALEZA	HGF	SACRA	Enviada	Eventos Adversos Relacionados à	Lesão por pressão		
13/04/2022	CE	FORTALEZA	HGF	TROCENTER ESQUERDO	Enviada	Eventos Adversos Relacionados à	Lesão por pressão		
13/04/2022	CE	FORTALEZA	HGF	GLÚTEOS	Enviada	Eventos Adversos Relacionados à	Lesão por pressão		
13/04/2022	CE	FORTALEZA	HGF	SACRA	Enviada	Eventos Adversos Relacionados à	Lesão por pressão		
13/04/2022	CE	FORTALEZA	HGF	SACRA	Enviada	Eventos Adversos Relacionados à	Lesão por pressão		
13/04/2022	CE	FORTALEZA	HGF	GLÚTEOS	Enviada	Eventos Adversos Relacionados à	Lesão por pressão		
13/04/2022	CE	FORTALEZA	HGF	SACRA	Enviada	Eventos Adversos Relacionados à	Lesão por pressão		
13/04/2022	CE	FORTALEZA	HGF	CALCÂNEOS	Enviada	Eventos Adversos Relacionados à	Lesão por pressão		
13/04/2022	CE	FORTALEZA	HGF	SACRA	Enviada	Eventos Adversos Relacionados à	Lesão por pressão		

13/04/2022	CE	FORTALEZA	HGF	SACRA	Concluída	Eventos Adversos Relacionados à	Lesão por pressão		
13/04/2022	CE	FORTALEZA	HGF	SACRA	Enviada	Eventos Adversos Relacionados à	Lesão por pressão		
13/04/2022	CE	FORTALEZA	HGF	SACRA	Enviada	Eventos Adversos Relacionados à	Lesão por pressão		
13/04/2022	CE	FORTALEZA	HGF	SACRA	Enviada	Eventos Adversos Relacionados à	Lesão por pressão		
13/04/2022	CE	FORTALEZA	HGF	CALCÂNEOS	Enviada	Eventos Adversos Relacionados à	Lesão por pressão		
13/04/2022	CE	FORTALEZA	HGF	SACRA	Enviada	Eventos Adversos Relacionados à	Lesão por pressão		
13/04/2022	CE	FORTALEZA	HGF	ORELHA DIREITA	Concluída	Eventos Adversos Relacionados à	Lesão por pressão		
13/04/2022	CE	FORTALEZA	HGF	SACRA	Concluída	Eventos Adversos Relacionados à	Lesão por pressão		
13/04/2022	CE	FORTALEZA	HGF	GLÚTEOS	Concluída	Eventos Adversos Relacionados à	Lesão por pressão		
13/04/2022	CE	FORTALEZA	HGF	TROCANTER ESQUERDO	Concluída	Eventos Adversos Relacionados à	Lesão por pressão		
13/04/2022	CE	FORTALEZA	HGF	SACRA	Enviada	Eventos Adversos Relacionados à	Lesão por pressão		
13/04/2022	CE	FORTALEZA	HGF	SACRA	Concluída	Eventos Adversos Relacionados à	Lesão por pressão		
13/04/2022	CE	FORTALEZA	HGF	SACRA	Enviada	Eventos Adversos Relacionados à	Lesão por pressão		
13/04/2022	CE	FORTALEZA	HGF	SACRA	Enviada	Eventos Adversos Relacionados à	Lesão por pressão		
13/04/2022	CE	FORTALEZA	HGF	SACRA	Enviada	Eventos Adversos Relacionados à	Lesão por pressão		

13/04/2022	CE	FORTALEZA	HGF	TROCANTER ESQUERDO	Enviada	Eventos Adversos Relacionados à	Lesão por pressão		
13/04/2022	CE	FORTALEZA	HGF	PAVILHAO AURICULAR	Enviada	Eventos Adversos Relacionados à	Lesão por pressão		
18/04/2022	CE	FORTALEZA	HGF	SACRA	Enviada	Eventos Adversos Relacionados à	Lesão por pressão		
18/04/2022	CE	FORTALEZA	HGF	SACRA	Enviada	Eventos Adversos Relacionados à	Lesão por pressão		
18/04/2022	CE	FORTALEZA	HGF	SACRA	Enviada	Eventos Adversos Relacionados à	Lesão por pressão		
18/04/2022	CE	FORTALEZA	HGF	SACRA	Enviada	Eventos Adversos Relacionados à	Lesão por pressão		
18/04/2022	CE	FORTALEZA	HGF	CALCÂNEOS	Enviada	Eventos Adversos Relacionados à	Lesão por pressão		
18/04/2022	CE	FORTALEZA	HGF	SACRA	Enviada	Eventos Adversos Relacionados à	Lesão por pressão		
18/04/2022	CE	FORTALEZA	HGF	SACRA	Enviada	Eventos Adversos Relacionados à	Lesão por pressão		
18/04/2022	CE	FORTALEZA	HGF	SACRA	Enviada	Eventos Adversos Relacionados à	Lesão por pressão		
18/04/2022	CE	FORTALEZA	HGF	SACRA	Enviada	Eventos Adversos Relacionados à	Lesão por pressão		
18/04/2022	CE	FORTALEZA	HGF	SACRA	Enviada	Eventos Adversos Relacionados à	Lesão por pressão		
18/04/2022	CE	FORTALEZA	HGF	SACRA	Enviada	Eventos Adversos Relacionados à	Lesão por pressão		
18/04/2022	CE	FORTALEZA	HGF	SACRA	Enviada	Eventos Adversos Relacionados à	Lesão por pressão		
18/04/2022	CE	FORTALEZA	HGF	GLÚTEO ESQUERDO	Enviada	Eventos Adversos Relacionados à	Lesão por pressão		

18/04/2022	CE	FORTALEZA	HGF	SACRA	Enviada	Eventos Adversos Relacionados à	Lesão por pressão		
18/04/2022	CE	FORTALEZA	HGF	GLÚTEOS	Enviada	Eventos Adversos Relacionados à	Lesão por pressão		
18/04/2022	CE	FORTALEZA	HGF	SACRA	Enviada	Eventos Adversos Relacionados à	Lesão por pressão		
18/04/2022	CE	FORTALEZA	HGF	MALÉOLO DIREITO	Enviada	Eventos Adversos Relacionados à	Lesão por pressão		
18/04/2022	CE	FORTALEZA	HGF	SACRA	Enviada	Eventos Adversos Relacionados à	Lesão por pressão		
18/04/2022	CE	FORTALEZA	HGF	SACRA	Enviada	Eventos Adversos Relacionados à	Lesão por pressão		
18/04/2022	CE	FORTALEZA	HGF	SACRA	Enviada	Eventos Adversos Relacionados à	Lesão por pressão		
18/04/2022	CE	FORTALEZA	HGF	SACRA	Concluída	Eventos Adversos Relacionados à	Lesão por pressão		
18/04/2022	CE	FORTALEZA	HGF	SACRA	Enviada	Eventos Adversos Relacionados à	Lesão por pressão		
18/04/2022	CE	FORTALEZA	HGF	GLÚTEOS	Enviada	Eventos Adversos Relacionados à	Lesão por pressão		
18/04/2022	CE	FORTALEZA	HGF	GLÚTEO DIREITO	Enviada	Eventos Adversos Relacionados à	Lesão por pressão		
18/04/2022	CE	FORTALEZA	HGF	SACRA	Enviada	Eventos Adversos Relacionados à	Lesão por pressão		
18/04/2022	CE	FORTALEZA	HGF	CALCÂNEO DIREITO	Enviada	Eventos Adversos Relacionados à	Lesão por pressão		
18/04/2022	CE	FORTALEZA	HGF	SACRA	Enviada	Eventos Adversos Relacionados à	Lesão por pressão		
18/04/2022	CE	FORTALEZA	HGF	SACRA	Enviada	Eventos Adversos Relacionados à	Lesão por pressão		

18/04/2022	CE	FORTALEZA	HGF	ESCÁPULA DIREITA	Enviada	Eventos Adversos Relacionados à	Lesão por pressão		
18/04/2022	CE	FORTALEZA	HGF	GLÚTEOS	Enviada	Eventos Adversos Relacionados à	Lesão por pressão		
18/04/2022	CE	FORTALEZA	HGF	SACRA	Enviada	Eventos Adversos Relacionados à	Lesão por pressão		
18/04/2022	CE	FORTALEZA	HGF	GLÚTEO ESQUERDO	Enviada	Eventos Adversos Relacionados à	Lesão por pressão		
18/04/2022	CE	FORTALEZA	HGF	SACRA	Enviada	Eventos Adversos Relacionados à	Lesão por pressão		
18/04/2022	CE	FORTALEZA	HGF	SACRA	Enviada	Eventos Adversos Relacionados à	Lesão por pressão		
18/04/2022	CE	FORTALEZA	HGF	GLÚTEOS	Concluída	Eventos Adversos Relacionados à	Lesão por pressão		
18/04/2022	CE	FORTALEZA	HGF	GLÚTEOS	Enviada	Eventos Adversos Relacionados à	Lesão por pressão		
18/04/2022	CE	FORTALEZA	HGF	SACRA	Enviada	Eventos Adversos Relacionados à	Lesão por pressão		
18/04/2022	CE	FORTALEZA	HGF	SACRA	Concluída	Eventos Adversos Relacionados à	Lesão por pressão		
18/04/2022	CE	FORTALEZA	HGF	SACRA	Concluída	Eventos Adversos Relacionados à	Lesão por pressão		
18/04/2022	CE	FORTALEZA	HGF	SACRA	Enviada	Eventos Adversos Relacionados à	Lesão por pressão		
18/04/2022	CE	FORTALEZA	HGF	GLÚTEOS	Enviada	Eventos Adversos Relacionados à	Lesão por pressão		
18/04/2022	CE	FORTALEZA	HGF	SACRA	Enviada	Eventos Adversos Relacionados à	Lesão por pressão		
18/04/2022	CE	FORTALEZA	HGF	SACRA	Enviada	Eventos Adversos Relacionados à	Lesão por pressão		

18/04/2022	CE	FORTALEZA	HGF	SACRA	Enviada	Eventos Adversos Relacionados à	Lesão por pressão		
18/04/2022	CE	FORTALEZA	HGF	CALCÂNEO DIREITO	Enviada	Eventos Adversos Relacionados à	Lesão por pressão		
18/04/2022	CE	FORTALEZA	HGF	SACRA	Enviada	Eventos Adversos Relacionados à	Lesão por pressão		
19/04/2022	CE	FORTALEZA	HGF	ESCÁPULA DIREITA	Enviada	Eventos Adversos Relacionados à	Lesão por pressão		
19/04/2022	CE	FORTALEZA	HGF	SACRA	Enviada	Eventos Adversos Relacionados à	Lesão por pressão		
19/04/2022	CE	FORTALEZA	HGF	SACRA	Enviada	Eventos Adversos Relacionados à	Lesão por pressão		
19/04/2022	CE	FORTALEZA	HGF	INTERGLÚTEO	Enviada	Eventos Adversos Relacionados à	Lesão por pressão		
19/04/2022	CE	FORTALEZA	HGF	SACRA	Enviada	Eventos Adversos Relacionados à	Lesão por pressão		
19/04/2022	CE	FORTALEZA	HGF	SACRA	Enviada	Eventos Adversos Relacionados à	Lesão por pressão		
19/04/2022	CE	FORTALEZA	HGF	SACRA	Enviada	Eventos Adversos Relacionados à	Lesão por pressão		
19/04/2022	CE	FORTALEZA	HGF	SACRA	Enviada	Eventos Adversos Relacionados à	Lesão por pressão		
19/04/2022	CE	FORTALEZA	HGF	INTERGLÚTEO	Concluída	Eventos Adversos Relacionados à	Lesão por pressão		
19/04/2022	CE	FORTALEZA	HGF	MALÉOLOS	Concluída	Eventos Adversos Relacionados à	Lesão por pressão		
19/04/2022	CE	FORTALEZA	HGF	GLÚTEOS	Enviada	Eventos Adversos Relacionados à	Lesão por pressão		
19/04/2022	CE	FORTALEZA	HGF	SACRA	Enviada	Eventos Adversos Relacionados à	Lesão por pressão		

19/04/2022	CE	FORTALEZA	HGF	SACRA	Enviada	Eventos Adversos Relacionados à	Lesão por pressão		
19/04/2022	CE	FORTALEZA	HGF	SACRA	Concluída	Eventos Adversos Relacionados à	Lesão por pressão		
19/04/2022	CE	FORTALEZA	HGF	SACRA	Enviada	Eventos Adversos Relacionados à	Lesão por pressão		
19/04/2022	CE	FORTALEZA	HGF	SACRA	Enviada	Eventos Adversos Relacionados à	Lesão por pressão		
19/04/2022	CE	FORTALEZA	HGF	SACRA	Enviada	Eventos Adversos Relacionados à	Lesão por pressão		
19/04/2022	CE	FORTALEZA	HGF	SACRA	Enviada	Eventos Adversos Relacionados à	Lesão por pressão		
19/04/2022	CE	FORTALEZA	HGF	SACRA	Enviada	Eventos Adversos Relacionados à	Lesão por pressão		
19/04/2022	CE	FORTALEZA	HGF	GLÚTEOS	Enviada	Eventos Adversos Relacionados à	Lesão por pressão		
19/04/2022	CE	FORTALEZA	HGF	SACRA	Enviada	Eventos Adversos Relacionados à	Lesão por pressão		
19/04/2022	CE	FORTALEZA	HGF	SACRA	Enviada	Eventos Adversos Relacionados à	Lesão por pressão		
19/04/2022	CE	FORTALEZA	HGF	SACRA	Enviada	Eventos Adversos Relacionados à	Lesão por pressão		
19/04/2022	CE	FORTALEZA	HGF	SACRA	Enviada	Eventos Adversos Relacionados à	Lesão por pressão		
19/04/2022	CE	FORTALEZA	HGF	INTERGLÚTEO	Enviada	Eventos Adversos Relacionados à	Lesão por pressão		
19/04/2022	CE	FORTALEZA	HGF	GLÚTEO DIREITO	Enviada	Eventos Adversos Relacionados à	Lesão por pressão		
19/04/2022	CE	FORTALEZA	HGF	SACRA	Enviada	Eventos Adversos Relacionados à	Lesão por pressão		

19/04/2022	CE	FORTALEZA	HGF	SACRA	Enviada	Eventos Adversos Relacionados à	Lesão por pressão		
19/04/2022	CE	FORTALEZA	HGF	SACRA	Enviada	Eventos Adversos Relacionados à	Lesão por pressão		
19/04/2022	CE	FORTALEZA	HGF	SACRA	Concluída	Eventos Adversos Relacionados à	Lesão por pressão		
19/04/2022	CE	FORTALEZA	HGF	SACRA	Enviada	Eventos Adversos Relacionados à	Lesão por pressão		
19/04/2022	CE	FORTALEZA	HGF	SACRA	Enviada	Eventos Adversos Relacionados à	Lesão por pressão		
19/04/2022	CE	FORTALEZA	HGF	CALCÂNEO DIREITO	Enviada	Eventos Adversos Relacionados à	Lesão por pressão		
19/04/2022	CE	FORTALEZA	HGF	OCCIPTAL	Enviada	Eventos Adversos Relacionados à	Lesão por pressão		
19/04/2022	CE	FORTALEZA	HGF	OCCIPTAL	Enviada	Eventos Adversos Relacionados à	Lesão por pressão		
19/04/2022	CE	FORTALEZA	HGF	SACRA	Enviada	Eventos Adversos Relacionados à	Lesão por pressão		
19/04/2022	CE	FORTALEZA	HGF	SACRA	Concluída	Eventos Adversos Relacionados à	Lesão por pressão		
19/04/2022	CE	FORTALEZA	HGF	GLÚTEOS	Concluída	Eventos Adversos Relacionados à	Lesão por pressão		
19/04/2022	CE	FORTALEZA	HGF	SACRA	Enviada	Eventos Adversos Relacionados à	Lesão por pressão		
19/04/2022	CE	FORTALEZA	HGF	SACRA	Concluída	Eventos Adversos Relacionados à	Lesão por pressão		
19/04/2022	CE	FORTALEZA	HGF	SACRA	Enviada	Eventos Adversos Relacionados à	Lesão por pressão		
19/04/2022	CE	FORTALEZA	HGF	SACRA	Enviada	Eventos Adversos Relacionados à	Lesão por pressão		

20/04/2022	CE	FORTALEZA	HGF	Queda da própria altura durante o	Enviada	Eventos Adversos Relacionados à	Queda do paciente		
20/04/2022	CE	FORTALEZA	HGF	Paciente desorientado, agitado	Enviada	Eventos Adversos Relacionados à	Queda do paciente		
20/04/2022	CE	FORTALEZA	HGF	Flebite em MSE (Maddox grau 2)	Enviada	Eventos Adversos Relacionados à	Falhas envolvendo o cateter venoso		
20/04/2022	CE	FORTALEZA	HGF	PACIENTE ESCORREGOU	Enviada	Eventos Adversos Relacionados à	Queda do paciente		
06/05/2022	CE	FORTALEZA	HGF	ACOMPANHANTE RELATA QUE O	Enviada	Eventos Adversos Relacionados à	Queda do paciente		
06/05/2022	CE	FORTALEZA	HGF	Flebite em MSE, Maddox Grau 3	Enviada	Eventos Adversos Relacionados à	Falhas envolvendo o cateter venoso		
06/05/2022	CE	FORTALEZA	HGF	Paciente sem acompanhante não	Enviada	Eventos Adversos Relacionados à	Queda do paciente		
09/05/2022	CE	FORTALEZA	HGF	RETIRADO O ACESSO E	Enviada	Eventos Adversos Relacionados à	Falhas envolvendo o cateter venoso		
09/05/2022	CE	FORTALEZA	HGF	Paciente com sinais de choque	Enviada	Eventos Adversos Relacionados à	Falhas durante a assistência à saúde		
09/05/2022	CE	FORTALEZA	HGF	ADMINISTRADO POLIMIXINA	Enviada	Eventos Adversos Relacionados à	Falhas durante a assistência à saúde		
09/05/2022	CE	FORTALEZA	HGF	APRESENTANDO DIFICULDADE DE	Enviada	Eventos Adversos Relacionados à	Falhas envolvendo o cateter venoso		
09/05/2022	CE	FORTALEZA	HGF	PACIENTE COM DIFICIL ACESSO	Enviada	Eventos Adversos Relacionados à	Falhas envolvendo o cateter venoso		
09/05/2022	CE	FORTALEZA	HGF	PACIENTE SEM ACOMPANHANTE	Enviada	Eventos Adversos Relacionados à	Queda do paciente		
09/05/2022	CE	FORTALEZA	HGF	Administrada água oxigenada por SNE	Enviada	Eventos Adversos Relacionados à	Acidentes do paciente	Exposição à substância química	
09/05/2022	CE	FORTALEZA	HGF	Durante o exame de tc de crânio, o	Enviada	Eventos Adversos Relacionados à	Acidentes do paciente	Outro tipo de mecanismo de	

09/05/2022	CE	FORTALEZA	HGF	PACIENTE PROVENIENTE DA	Enviada	Eventos Adversos Relacionados à	Falhas envolvendo o cateter venoso		
09/05/2022	CE	FORTALEZA	HGF	RETIRADO CVP E PUNCIONADO EM	Enviada	Eventos Adversos Relacionados à	Falhas envolvendo o cateter venoso		
09/05/2022	CE	FORTALEZA	HGF	A paciente tinha indicação	Enviada	Eventos Adversos Relacionados à	Falhas durante a assistência à saúde		
09/05/2022	CE	FORTALEZA	HGF	FLEBITE EM MSE.	Enviada	Eventos Adversos Relacionados à	Falhas envolvendo o cateter venoso		
09/05/2022	CE	FORTALEZA	HGF	FLEBITE EM MSE	Enviada	Eventos Adversos Relacionados à	Falhas envolvendo o cateter venoso		
09/05/2022	CE	FORTALEZA	HGF	Paciente advindo da emergênc	Enviada	Eventos Adversos Relacionados à	Falhas durante a assistência à saúde		
09/05/2022	CE	FORTALEZA	HGF	Paciente acamado tentou levantar	Enviada	Eventos Adversos Relacionados à	Queda do paciente		
09/05/2022	CE	FORTALEZA	HGF	A paciente estava em pé ao	Enviada	Eventos Adversos Relacionados à	Queda do paciente		
09/05/2022	CE	FORTALEZA	HGF	Paciente no andador com a	Enviada	Eventos Adversos Relacionados à	Queda do paciente		
09/05/2022	CE	FORTALEZA	HGF	FLEBITE EM MSD	Enviada	Eventos Adversos Relacionados à	Falhas envolvendo o cateter venoso		
09/05/2022	CE	FORTALEZA	HGF	Lesão bolhosa associada com	Enviada	Eventos Adversos Relacionados à	Falhas envolvendo o cateter venoso		
09/05/2022	CE	FORTALEZA	HGF	lpp em sacra	Enviada	Eventos Adversos Relacionados à	Lesão por pressão		
09/05/2022	CE	FORTALEZA	HGF	LPP EM DORSO	Enviada	Eventos Adversos Relacionados à	Lesão por pressão		
09/05/2022	CE	FORTALEZA	HGF	LPP INTEGLÚTEO/SACRA	Enviada	Eventos Adversos Relacionados à	Lesão por pressão		
09/05/2022	CE	FORTALEZA	HGF	LPP SACRA/GLÚTEO	Enviada	Eventos Adversos Relacionados à	Lesão por pressão		

09/05/2022	CE	FORTALEZA	HGF	LPP EM SACRA/GLÚTEO/INTERGUL	Enviada	Eventos Adversos Relacionados à	Lesão por pressão		
09/05/2022	CE	FORTALEZA	HGF	lpp em sacra	Enviada	Eventos Adversos Relacionados à	Lesão por pressão		
09/05/2022	CE	FORTALEZA	HGF	lpp em sacra	Enviada	Eventos Adversos Relacionados à	Lesão por pressão		
09/05/2022	CE	FORTALEZA	HGF	LPP SACRA/GLÚTEO E	Enviada	Eventos Adversos Relacionados à	Lesão por pressão		
09/05/2022	CE	FORTALEZA	HGF	lpp em sacra	Enviada	Eventos Adversos Relacionados à	Lesão por pressão		
09/05/2022	CE	FORTALEZA	HGF	lpp em sacra	Enviada	Eventos Adversos Relacionados à	Lesão por pressão		
09/05/2022	CE	FORTALEZA	HGF	lpp em trocanter	Enviada	Eventos Adversos Relacionados à	Lesão por pressão		
09/05/2022	CE	FORTALEZA	HGF	LPP EM SACRA/GLÚTEO	Concluída	Eventos Adversos Relacionados à	Lesão por pressão		
09/05/2022	CE	FORTALEZA	HGF	lpp em trocanter	Enviada	Eventos Adversos Relacionados à	Lesão por pressão		
01/06/2022	CE	FORTALEZA	HGF	Foi indicado no final da tarde	Enviada	Eventos Adversos Relacionados à	Falhas durante a assistência à saúde		
01/06/2022	CE	FORTALEZA	HGF	Flebite em MSD	Enviada	Eventos Adversos Relacionados à	Falhas envolvendo o cateter venoso		
01/06/2022	CE	FORTALEZA	HGF	FLEBITE EM MSE	Enviada	Eventos Adversos Relacionados à	Falhas envolvendo o cateter venoso		
01/06/2022	CE	FORTALEZA	HGF	Paciente refere queda da própria	Enviada	Eventos Adversos Relacionados à	Queda do paciente		
01/06/2022	CE	FORTALEZA	HGF	PROFISSIONAL RELATA MIÍASE	Enviada	Eventos Adversos Relacionados à	Falhas durante a assistência à saúde		
01/06/2022	CE	FORTALEZA	HGF	-	Enviada	Eventos Adversos Relacionados à	Falhas durante a assistência à saúde		

01/06/2022	CE	FORTALEZA	HGF	-	Enviada	Eventos Adversos Relacionados à	Falhas durante a assistência à saúde		
01/06/2022	CE	FORTALEZA	HGF	EM ISOLAMENTO DESORI	Enviada	Eventos Adversos Relacionados à	Queda do paciente		
01/06/2022	CE	FORTALEZA	HGF	AO SER CHAMADA PELA ACOMPA	Enviada	Eventos Adversos Relacionados à	Queda do paciente		
01/06/2022	CE	FORTALEZA	HGF	EM UMA UNIDADE RECEBE	Enviada	Eventos Adversos Relacionados à	Falhas durante a assistência à saúde		
01/06/2022	CE	FORTALEZA	HGF	Paciente agitada, levantou-se do	Enviada	Eventos Adversos Relacionados à	Queda do paciente		
01/06/2022	CE	FORTALEZA	HGF	Paciente encontra-se com ideações	Enviada	Eventos Adversos Relacionados à	Queda do paciente		
01/06/2022	CE	FORTALEZA	HGF	Enfermeira recebeu o	Enviada	Eventos Adversos Relacionados à	Falhas durante a assistência à saúde		
01/06/2022	CE	FORTALEZA	HGF	Paciente caiu da própria altura no	Enviada	Eventos Adversos Relacionados à	Queda do paciente		
01/06/2022	CE	FORTALEZA	HGF	Paciente foi extubado acidental	Enviada	Eventos Adversos Relacionados à	Extubação endotraqueal		
01/06/2022	CE	FORTALEZA	HGF	PACIENTE ENCNTRAVA	Enviada	Eventos Adversos Relacionados à	Queda do paciente		
01/06/2022	CE	FORTALEZA	HGF	PACIENTE DESORIENTADO	Enviada	Eventos Adversos Relacionados à	Queda do paciente		
01/06/2022	CE	FORTALEZA	HGF	Flebite em membro superior	Enviada	Eventos Adversos Relacionados à	Tromboembolismo venoso (TEV)		
02/06/2022	CE	FORTALEZA	HGF	Flebite em MSD	Enviada	Eventos Adversos Relacionados à	Falhas envolvendo o cateter venoso		
02/06/2022	CE	FORTALEZA	HGF	Dor, edema e hiperemia em MSD	Enviada	Eventos Adversos Relacionados à	Falhas envolvendo o cateter venoso		
02/06/2022	CE	FORTALEZA	HGF	Paciente apresenta terceiro episódio	Enviada	Eventos Adversos Relacionados à	Queda do paciente		

02/06/2022	CE	FORTALEZA	HGF	Paciente relata que ao ir subir na	Enviada	Eventos Adversos Relacionados à	Queda do paciente		
02/06/2022	CE	FORTALEZA	HGF	Paciente apresentou míiase ao ser	Enviada	Eventos Adversos Relacionados à	Falhas durante a assistência à saúde		
02/06/2022	CE	FORTALEZA	HGF	Paciente no momento da queda	Enviada	Eventos Adversos Relacionados à	Queda do paciente		
02/06/2022	CE	FORTALEZA	HGF	Paciente pediátrico caiu do berço	Enviada	Eventos Adversos Relacionados à	Queda do paciente		
02/06/2022	CE	FORTALEZA	HGF	Paciente pediátrica apresentou flebite	Enviada	Eventos Adversos Relacionados à	Falhas envolvendo cateter venoso		
02/06/2022	CE	FORTALEZA	HGF	A paciente levantou repentina	Enviada	Eventos Adversos Relacionados à	Queda do paciente		
02/06/2022	CE	FORTALEZA	HGF	Paciente consciente,algo orientado	Enviada	Eventos Adversos Relacionados à	Queda do paciente		
02/06/2022	CE	FORTALEZA	HGF	Paciente foi ao banheiro sem a	Enviada	Eventos Adversos Relacionados à	Queda do paciente		
20/06/2022	CE	FORTALEZA	HGF	Frasco de soro vazio abaixo do	Enviada	Eventos Adversos Relacionados à	Falhas durante a assistência à saúde		
21/06/2022	CE	FORTALEZA	HGF	LPP SACRA	Enviada	Eventos Adversos Relacionados à	Lesão por pressão		
21/06/2022	CE	FORTALEZA	HGF	LPP SACRA ESTÁGIO 2	Enviada	Eventos Adversos Relacionados à	Lesão por pressão		
21/06/2022	CE	FORTALEZA	HGF	LPP SACRA	Enviada	Eventos Adversos Relacionados à	Lesão por pressão		
21/06/2022	CE	FORTALEZA	HGF	LPP ESTÁGIO 1	Enviada	Eventos Adversos Relacionados à	Lesão por pressão		
21/06/2022	CE	FORTALEZA	HGF	LPP SACRA	Enviada	Eventos Adversos Relacionados à	Lesão por pressão		
21/06/2022	CE	FORTALEZA	HGF	LPP EM SACRA	Concluída	Eventos Adversos Relacionados à	Lesão por pressão		

21/06/2022	CE	FORTALE ZA	HGF	LPP EM CALCÂN EO ESQUER	Enviada	Eventos Adversos Relaciona dos à	Lesão por pressão		
21/06/2022	CE	FORTALE ZA	HGF	LPP SACRA	Enviada	Eventos Adversos Relaciona dos à	Lesão por pressão		
21/06/2022	CE	FORTALE ZA	HGF	lpp em glúteos est. 2	Enviada	Eventos Adversos Relaciona dos à	Lesão por pressão		
21/06/2022	CE	FORTALE ZA	HGF	LPP SACRA	Enviada	Eventos Adversos Relaciona dos à	Lesão por pressão		
21/06/2022	CE	FORTALE ZA	HGF	LPP EM GLÚTEO	Enviada	Eventos Adversos Relaciona dos à	Lesão por pressão		
22/06/2022	CE	FORTALE ZA	HGF	SACRA	Enviada	Eventos Adversos Relaciona dos à	Lesão por pressão		
22/06/2022	CE	FORTALE ZA	HGF	CALCÂN EOS	Enviada	Eventos Adversos Relaciona dos à	Lesão por pressão		
22/06/2022	CE	FORTALE ZA	HGF	SACRA	Enviada	Eventos Adversos Relaciona dos à	Lesão por pressão		
22/06/2022	CE	FORTALE ZA	HGF	SACRA	Enviada	Eventos Adversos Relaciona dos à	Lesão por pressão		
22/06/2022	CE	FORTALE ZA	HGF	SACRA	Enviada	Eventos Adversos Relaciona dos à	Lesão por pressão		
22/06/2022	CE	FORTALE ZA	HGF	SACRA	Enviada	Eventos Adversos Relaciona dos à	Lesão por pressão		
22/06/2022	CE	FORTALE ZA	HGF	SACRA	Enviada	Eventos Adversos Relaciona dos à	Lesão por pressão		
22/06/2022	CE	FORTALE ZA	HGF	SACRA	Enviada	Eventos Adversos Relaciona dos à	Lesão por pressão		
22/06/2022	CE	FORTALE ZA	HGF	SACRA	Concluída	Eventos Adversos Relaciona dos à	Lesão por pressão		
22/06/2022	CE	FORTALE ZA	HGF	SACRA	Concluída	Eventos Adversos Relaciona dos à	Lesão por pressão		

22/06/2022	CE	FORTALEZA	HGF	LPP EM CALCÂNEOS	Enviada	Eventos Adversos Relacionados à	Lesão por pressão		
22/06/2022	CE	FORTALEZA	HGF	LPP SACRA	Enviada	Eventos Adversos Relacionados à	Lesão por pressão		
22/06/2022	CE	FORTALEZA	HGF	SACRA	Enviada	Eventos Adversos Relacionados à	Lesão por pressão		
22/06/2022	CE	FORTALEZA	HGF	DORSO	Enviada	Eventos Adversos Relacionados à	Lesão por pressão		
22/06/2022	CE	FORTALEZA	HGF	PAVILHAO AURICULAR	Enviada	Eventos Adversos Relacionados à	Lesão por pressão		
22/06/2022	CE	FORTALEZA	HGF	SACRA	Enviada	Eventos Adversos Relacionados à	Lesão por pressão		
22/06/2022	CE	FORTALEZA	HGF	SACRA	Enviada	Eventos Adversos Relacionados à	Lesão por pressão		
22/06/2022	CE	FORTALEZA	HGF	LPP SACRA	Enviada	Eventos Adversos Relacionados à	Lesão por pressão		
22/06/2022	CE	FORTALEZA	HGF	2022.06.519066	Concluída	Eventos Adversos Relacionados à	Lesão por pressão		
22/06/2022	CE	FORTALEZA	HGF	LPP SACRA	Enviada	Eventos Adversos Relacionados à	Lesão por pressão		
22/06/2022	CE	FORTALEZA	HGF	TROCÂNTER DIREITO	Enviada	Eventos Adversos Relacionados à	Lesão por pressão		
22/06/2022	CE	FORTALEZA	HGF	SACRA	Enviada	Eventos Adversos Relacionados à	Lesão por pressão		
22/06/2022	CE	FORTALEZA	HGF	LPP SACRA	Enviada	Eventos Adversos Relacionados à	Lesão por pressão		
22/06/2022	CE	FORTALEZA	HGF	GLÚTEOS	Enviada	Eventos Adversos Relacionados à	Lesão por pressão		
22/06/2022	CE	FORTALEZA	HGF	LPP SACRA	Enviada	Eventos Adversos Relacionados à	Lesão por pressão		

22/06/2022	CE	FORTALEZA	HGF	SACRA	Enviada	Eventos Adversos Relacionados à	Lesão por pressão		
22/06/2022	CE	FORTALEZA	HGF	LPP EM TROCÂNTER DIREITO	Enviada	Eventos Adversos Relacionados à	Lesão por pressão		
22/06/2022	CE	FORTALEZA	HGF	SACRA	Enviada	Eventos Adversos Relacionados à	Lesão por pressão		
22/06/2022	CE	FORTALEZA	HGF	LPP SACRA	Enviada	Eventos Adversos Relacionados à	Lesão por pressão		
22/06/2022	CE	FORTALEZA	HGF	SACRA	Enviada	Eventos Adversos Relacionados à	Lesão por pressão		
22/06/2022	CE	FORTALEZA	HGF	SACRA	Concluída	Eventos Adversos Relacionados à	Lesão por pressão		
22/06/2022	CE	FORTALEZA	HGF	SACRA	Enviada	Eventos Adversos Relacionados à	Lesão por pressão		
22/06/2022	CE	FORTALEZA	HGF	LPP EM GLÚTEO DIREITO	Enviada	Eventos Adversos Relacionados à	Lesão por pressão		
22/06/2022	CE	FORTALEZA	HGF	MALEOLO ESQUERDO	Enviada	Eventos Adversos Relacionados à	Lesão por pressão		
22/06/2022	CE	FORTALEZA	HGF	LPP SACRA	Enviada	Eventos Adversos Relacionados à	Lesão por pressão		
22/06/2022	CE	FORTALEZA	HGF	LPP SACRA	Enviada	Eventos Adversos Relacionados à	Lesão por pressão		
22/06/2022	CE	FORTALEZA	HGF	CALCÂNEO DIREITO	Enviada	Eventos Adversos Relacionados à	Lesão por pressão		
22/06/2022	CE	FORTALEZA	HGF	LPP SACRA	Enviada	Eventos Adversos Relacionados à	Lesão por pressão		
22/06/2022	CE	FORTALEZA	HGF	LPP SACRA	Enviada	Eventos Adversos Relacionados à	Lesão por pressão		
22/06/2022	CE	FORTALEZA	HGF	LPP SACRA	Enviada	Eventos Adversos Relacionados à	Lesão por pressão		

22/06/2022	CE	FORTALEZA	HGF	LPP SACRA	Enviada	Eventos Adversos Relacionados à	Lesão por pressão		
22/06/2022	CE	FORTALEZA	HGF	LPP SACRA	Concluída	Eventos Adversos Relacionados à	Lesão por pressão		
22/06/2022	CE	FORTALEZA	HGF	LPP SACRA	Enviada	Eventos Adversos Relacionados à	Lesão por pressão		
22/06/2022	CE	FORTALEZA	HGF	LPP EM MALÉOLO	Enviada	Eventos Adversos Relacionados à	Lesão por pressão		
22/06/2022	CE	FORTALEZA	HGF	LPP SACRA	Concluída	Eventos Adversos Relacionados à	Lesão por pressão		
22/06/2022	CE	FORTALEZA	HGF	LPP EM SACRA	Enviada	Eventos Adversos Relacionados à	Lesão por pressão		
22/06/2022	CE	FORTALEZA	HGF	GLÚTEO ESQUERDO	Enviada	Eventos Adversos Relacionados à	Lesão por pressão		
22/06/2022	CE	FORTALEZA	HGF	LPP SACRA	Enviada	Eventos Adversos Relacionados à	Lesão por pressão		
22/06/2022	CE	FORTALEZA	HGF	LPP SACRA	Enviada	Eventos Adversos Relacionados à	Lesão por pressão		
22/06/2022	CE	FORTALEZA	HGF	LPP SACRA	Enviada	Eventos Adversos Relacionados à	Lesão por pressão		
22/06/2022	CE	FORTALEZA	HGF	LPP EM PAVILÃO AURICULAR D	Enviada	Eventos Adversos Relacionados à	Lesão por pressão		
22/06/2022	CE	FORTALEZA	HGF	LPP SACRA	Enviada	Eventos Adversos Relacionados à	Lesão por pressão		
22/06/2022	CE	FORTALEZA	HGF	LPP EM SACRA	Enviada	Eventos Adversos Relacionados à	Lesão por pressão		
22/06/2022	CE	FORTALEZA	HGF	LPP SACRA	Concluída	Eventos Adversos Relacionados à	Lesão por pressão		
22/06/2022	CE	FORTALEZA	HGF	LPP EM OCCIPITAL	Concluída	Eventos Adversos Relacionados à	Lesão por pressão		

22/06/2022	CE	FORTALEZA	HGF	LPP SACRA	Enviada	Eventos Adversos Relacionados à	Lesão por pressão		
22/06/2022	CE	FORTALEZA	HGF	LPP EM GLÚTEOS	Enviada	Eventos Adversos Relacionados à	Lesão por pressão		
22/06/2022	CE	FORTALEZA	HGF	LPP EM SACRA	Concluída	Eventos Adversos Relacionados à	Lesão por pressão		
22/06/2022	CE	FORTALEZA	HGF	LPP SACRA	Concluída	Eventos Adversos Relacionados à	Lesão por pressão		
22/06/2022	CE	FORTALEZA	HGF	LPP SACRA	Enviada	Eventos Adversos Relacionados à	Lesão por pressão		
22/06/2022	CE	FORTALEZA	HGF	CALCÂNEO E	Enviada	Eventos Adversos Relacionados à	Lesão por pressão		
22/06/2022	CE	FORTALEZA	HGF	LPP SACRA	Concluída	Eventos Adversos Relacionados à	Lesão por pressão		
22/06/2022	CE	FORTALEZA	HGF	LPP SACRA	Enviada	Eventos Adversos Relacionados à	Lesão por pressão		
22/06/2022	CE	FORTALEZA	HGF	LPP SACRA	Enviada	Eventos Adversos Relacionados à	Lesão por pressão		
22/06/2022	CE	FORTALEZA	HGF	LPP SACRA	Enviada	Eventos Adversos Relacionados à	Lesão por pressão		
22/06/2022	CE	FORTALEZA	HGF	LPP SACRA	Enviada	Eventos Adversos Relacionados à	Lesão por pressão		
22/06/2022	CE	FORTALEZA	HGF	LPP SACRA	Concluída	Eventos Adversos Relacionados à	Lesão por pressão		
22/06/2022	CE	FORTALEZA	HGF	LPP SACRA	Enviada	Eventos Adversos Relacionados à	Lesão por pressão		
22/06/2022	CE	FORTALEZA	HGF	LPP SACRA	Concluída	Eventos Adversos Relacionados à	Lesão por pressão		
22/06/2022	CE	FORTALEZA	HGF	LPP SACRA	Enviada	Eventos Adversos Relacionados à	Lesão por pressão		

22/06/2022	CE	FORTALE ZA	HGF	LPP SACRA	Enviada	Eventos Adversos Relaciona dos à	Lesão por pressão		
22/06/2022	CE	FORTALE ZA	HGF	LPP SACRA	Enviada	Eventos Adversos Relaciona dos à	Lesão por pressão		
22/06/2022	CE	FORTALE ZA	HGF	LPP SACRA	Enviada	Eventos Adversos Relaciona dos à	Lesão por pressão		
22/06/2022	CE	FORTALE ZA	HGF	LPP SACRA	Concluída	Eventos Adversos Relaciona dos à	Lesão por pressão		
22/06/2022	CE	FORTALE ZA	HGF	LPP SACRA	Enviada	Eventos Adversos Relaciona dos à	Lesão por pressão		
22/06/2022	CE	FORTALE ZA	HGF	LPP SACRA	Enviada	Eventos Adversos Relaciona dos à	Lesão por pressão		
22/06/2022	CE	FORTALE ZA	HGF	LPP SACRA	Enviada	Eventos Adversos Relaciona dos à	Lesão por pressão		
22/06/2022	CE	FORTALE ZA	HGF	LPP SACRA	Enviada	Eventos Adversos Relaciona dos à	Lesão por pressão		
22/06/2022	CE	FORTALE ZA	HGF	LPP SACRA	Enviada	Eventos Adversos Relaciona dos à	Lesão por pressão		
22/06/2022	CE	FORTALE ZA	HGF	LPP SACRA	Enviada	Eventos Adversos Relaciona dos à	Lesão por pressão		
22/06/2022	CE	FORTALE ZA	HGF	LPP SACRA	Enviada	Eventos Adversos Relaciona dos à	Lesão por pressão		
22/06/2022	CE	FORTALE ZA	HGF	LPP SACRA	Enviada	Eventos Adversos Relaciona dos à	Lesão por pressão		
22/06/2022	CE	FORTALE ZA	HGF	LPP SACRA	Concluída	Eventos Adversos Relaciona dos à	Lesão por pressão		
22/06/2022	CE	FORTALE ZA	HGF	LPP SACRA	Enviada	Eventos Adversos Relaciona dos à	Lesão por pressão		
22/06/2022	CE	FORTALE ZA	HGF	TROCAN TER E	Enviada	Eventos Adversos Relaciona dos à	Lesão por pressão		

22/06/2022	CE	FORTALEZA	HGF	LPP EM GLÚTEO DIREITO	Enviada	Eventos Adversos Relacionados à	Lesão por pressão		
22/06/2022	CE	FORTALEZA	HGF	LPP EM MALÉOLO DIREITO	Concluída	Eventos Adversos Relacionados à	Lesão por pressão		
22/06/2022	CE	FORTALEZA	HGF	LPP SACRA	Enviada	Eventos Adversos Relacionados à	Lesão por pressão		
22/06/2022	CE	FORTALEZA	HGF	LPP SACRA	Enviada	Eventos Adversos Relacionados à	Lesão por pressão		
22/06/2022	CE	FORTALEZA	HGF	LPP SACRA	Enviada	Eventos Adversos Relacionados à	Lesão por pressão		
22/06/2022	CE	FORTALEZA	HGF	LPP EM CALCÂNEO DIREITO	Enviada	Eventos Adversos Relacionados à	Lesão por pressão		
22/06/2022	CE	FORTALEZA	HGF	LPP SACRA	Enviada	Eventos Adversos Relacionados à	Lesão por pressão		
22/06/2022	CE	FORTALEZA	HGF	LPP SACRA	Enviada	Eventos Adversos Relacionados à	Lesão por pressão		
22/06/2022	CE	FORTALEZA	HGF	LPP SACRA	Enviada	Eventos Adversos Relacionados à	Lesão por pressão		
22/06/2022	CE	FORTALEZA	HGF	LPP SACRA	Enviada	Eventos Adversos Relacionados à	Lesão por pressão		
22/06/2022	CE	FORTALEZA	HGF	LPP SACRA	Enviada	Eventos Adversos Relacionados à	Lesão por pressão		
22/06/2022	CE	FORTALEZA	HGF	LPP SACRA	Enviada	Eventos Adversos Relacionados à	Lesão por pressão		
22/06/2022	CE	FORTALEZA	HGF	LPP EM PANTURRILHA	Enviada	Eventos Adversos Relacionados à	Lesão por pressão		
22/06/2022	CE	FORTALEZA	HGF	LPP SACRA	Enviada	Eventos Adversos Relacionados à	Lesão por pressão		
22/06/2022	CE	FORTALEZA	HGF	PAVILHAO AURICULAR	Enviada	Eventos Adversos Relacionados à	Lesão por pressão		

22/06/2022	CE	FORTALEZA	HGF	LPP SACRA	Enviada	Eventos Adversos Relacionados à	Lesão por pressão		
22/06/2022	CE	FORTALEZA	HGF	LPP EM CALCÂNEO ESQUER	Enviada	Eventos Adversos Relacionados à	Lesão por pressão		
22/06/2022	CE	FORTALEZA	HGF	LPP SACRA	Enviada	Eventos Adversos Relacionados à	Lesão por pressão		
01/07/2022	CE	FORTALEZA	HGF	LPP em escápula esquerda	Enviada	Eventos Adversos Relacionados à	Lesão por pressão		
01/07/2022	CE	FORTALEZA	HGF	LPP SACRA	Enviada	Eventos Adversos Relacionados à	Lesão por pressão		
01/07/2022	CE	FORTALEZA	HGF	LPP EM SACRA	Enviada	Eventos Adversos Relacionados à	Lesão por pressão		
01/07/2022	CE	FORTALEZA	HGF	LPP SACRA	Enviada	Eventos Adversos Relacionados à	Lesão por pressão		
01/07/2022	CE	FORTALEZA	HGF	LPP EM GLÚTEOS	Enviada	Eventos Adversos Relacionados à	Lesão por pressão		
05/07/2022	CE	FORTALEZA	HGF	LPP SACRA	Enviada	Eventos Adversos Relacionados à	Lesão por pressão		
05/07/2022	CE	FORTALEZA	HGF	SACRA	Concluída	Eventos Adversos Relacionados à	Lesão por pressão		
05/07/2022	CE	FORTALEZA	HGF	SACRA	Enviada	Eventos Adversos Relacionados à	Lesão por pressão		
05/07/2022	CE	FORTALEZA	HGF	SACRA	Enviada	Eventos Adversos Relacionados à	Lesão por pressão		
05/07/2022	CE	FORTALEZA	HGF	SACRA	Concluída	Eventos Adversos Relacionados à	Lesão por pressão		
05/07/2022	CE	FORTALEZA	HGF	SACRA	Enviada	Eventos Adversos Relacionados à	Lesão por pressão		
05/07/2022	CE	FORTALEZA	HGF	SACRA	Enviada	Eventos Adversos Relacionados à	Lesão por pressão		

05/07/2022	CE	FORTALEZA	HGF	SACRA	Enviada	Eventos Adversos Relacionados à	Lesão por pressão		
05/07/2022	CE	FORTALEZA	HGF	PAVILHAO AURICULAR	Enviada	Eventos Adversos Relacionados à	Lesão por pressão		
05/07/2022	CE	FORTALEZA	HGF	SACRA	Enviada	Eventos Adversos Relacionados à	Lesão por pressão		
05/07/2022	CE	FORTALEZA	HGF	SACRA	Enviada	Eventos Adversos Relacionados à	Lesão por pressão		
05/07/2022	CE	FORTALEZA	HGF	GLÚTEOS	Enviada	Eventos Adversos Relacionados à	Lesão por pressão		
05/07/2022	CE	FORTALEZA	HGF	sacra	Enviada	Eventos Adversos Relacionados à	Lesão por pressão		
05/07/2022	CE	FORTALEZA	HGF	sacra	Enviada	Eventos Adversos Relacionados à	Lesão por pressão		
05/07/2022	CE	FORTALEZA	HGF	sacra	Enviada	Eventos Adversos Relacionados à	Lesão por pressão		
05/07/2022	CE	FORTALEZA	HGF	sacra	Enviada	Eventos Adversos Relacionados à	Lesão por pressão		
05/07/2022	CE	FORTALEZA	HGF	SACRA	Enviada	Eventos Adversos Relacionados à	Lesão por pressão		
05/07/2022	CE	FORTALEZA	HGF	SACRA	Enviada	Eventos Adversos Relacionados à	Lesão por pressão		
05/07/2022	CE	FORTALEZA	HGF	SACRA	Enviada	Eventos Adversos Relacionados à	Lesão por pressão		
05/07/2022	CE	FORTALEZA	HGF	GLÚTEO ESQUERDO	Enviada	Eventos Adversos Relacionados à	Lesão por pressão		
05/07/2022	CE	FORTALEZA	HGF	SACRA	Concluída	Eventos Adversos Relacionados à	Lesão por pressão		
05/07/2022	CE	FORTALEZA	HGF	SACRA	Enviada	Eventos Adversos Relacionados à	Lesão por pressão		

05/07/2022	CE	FORTALEZA	HGF	SACRA	Enviada	Eventos Adversos Relacionados à	Lesão por pressão		
05/07/2022	CE	FORTALEZA	HGF	SACRA	Enviada	Eventos Adversos Relacionados à	Lesão por pressão		
05/07/2022	CE	FORTALEZA	HGF	GLÚTEOS	Enviada	Eventos Adversos Relacionados à	Lesão por pressão		
05/07/2022	CE	FORTALEZA	HGF	INTERGLÚTEO	Enviada	Eventos Adversos Relacionados à	Lesão por pressão		
05/07/2022	CE	FORTALEZA	HGF	SACRA	Enviada	Eventos Adversos Relacionados à	Lesão por pressão		
05/07/2022	CE	FORTALEZA	HGF	SACRA	Enviada	Eventos Adversos Relacionados à	Lesão por pressão		
05/07/2022	CE	FORTALEZA	HGF	GLÚTEO ESQUERDO	Enviada	Eventos Adversos Relacionados à	Lesão por pressão		
05/07/2022	CE	FORTALEZA	HGF	SACRA	Enviada	Eventos Adversos Relacionados à	Lesão por pressão		
05/07/2022	CE	FORTALEZA	HGF	SACRA	Enviada	Eventos Adversos Relacionados à	Lesão por pressão		
05/07/2022	CE	FORTALEZA	HGF	SACRA	Enviada	Eventos Adversos Relacionados à	Lesão por pressão		
05/07/2022	CE	FORTALEZA	HGF	SACRA	Enviada	Eventos Adversos Relacionados à	Lesão por pressão		
05/07/2022	CE	FORTALEZA	HGF	SACRA	Enviada	Eventos Adversos Relacionados à	Lesão por pressão		
05/07/2022	CE	FORTALEZA	HGF	GLÚTEOS	Enviada	Eventos Adversos Relacionados à	Lesão por pressão		
05/07/2022	CE	FORTALEZA	HGF	SACRA	Enviada	Eventos Adversos Relacionados à	Lesão por pressão		
05/07/2022	CE	FORTALEZA	HGF	GLÚTEO DIREITO	Enviada	Eventos Adversos Relacionados à	Lesão por pressão		

05/07/2022	CE	FORTALE ZA	HGF	SACRA	Concluída	Eventos Adversos Relaciona dos à	Lesão por pressão		
05/07/2022	CE	FORTALE ZA	HGF	SACRA	Enviada	Eventos Adversos Relaciona dos à	Lesão por pressão		
05/07/2022	CE	FORTALE ZA	HGF	SACRA	Concluída	Eventos Adversos Relaciona dos à	Lesão por pressão		
05/07/2022	CE	FORTALE ZA	HGF	SACRA	Enviada	Eventos Adversos Relaciona dos à	Lesão por pressão		
05/07/2022	CE	FORTALE ZA	HGF	SACRA	Enviada	Eventos Adversos Relaciona dos à	Lesão por pressão		
05/07/2022	CE	FORTALE ZA	HGF	SACRA	Concluída	Eventos Adversos Relaciona dos à	Lesão por pressão		
05/07/2022	CE	FORTALE ZA	HGF	SACRA	Enviada	Eventos Adversos Relaciona dos à	Lesão por pressão		
05/07/2022	CE	FORTALE ZA	HGF	SACRA	Concluída	Eventos Adversos Relaciona dos à	Lesão por pressão		
05/07/2022	CE	FORTALE ZA	HGF	SACRA	Enviada	Eventos Adversos Relaciona dos à	Lesão por pressão		
05/07/2022	CE	FORTALE ZA	HGF	SACRA	Enviada	Eventos Adversos Relaciona dos à	Lesão por pressão		
05/07/2022	CE	FORTALE ZA	HGF	SACRA	Enviada	Eventos Adversos Relaciona dos à	Lesão por pressão		
05/07/2022	CE	FORTALE ZA	HGF	SACRA	Enviada	Eventos Adversos Relaciona dos à	Lesão por pressão		
05/07/2022	CE	FORTALE ZA	HGF	SACRA	Enviada	Eventos Adversos Relaciona dos à	Lesão por pressão		
05/07/2022	CE	FORTALE ZA	HGF	SACRA	Enviada	Eventos Adversos Relaciona dos à	Lesão por pressão		
05/07/2022	CE	FORTALE ZA	HGF	SACRA	Enviada	Eventos Adversos Relaciona dos à	Lesão por pressão		

05/07/2022	CE	FORTALEZA	HGF	SACRA	Enviada	Eventos Adversos Relacionados à	Lesão por pressão		
05/07/2022	CE	FORTALEZA	HGF	SACRA	Enviada	Eventos Adversos Relacionados à	Lesão por pressão		
05/07/2022	CE	FORTALEZA	HGF	SACRA	Enviada	Eventos Adversos Relacionados à	Lesão por pressão		
05/07/2022	CE	FORTALEZA	HGF	SACRA	Enviada	Eventos Adversos Relacionados à	Lesão por pressão		
06/07/2022	CE	FORTALEZA	HGF	SACRA	Enviada	Eventos Adversos Relacionados à	Lesão por pressão		
06/07/2022	CE	FORTALEZA	HGF	GLÚTEO DIREITO	Enviada	Eventos Adversos Relacionados à	Lesão por pressão		
06/07/2022	CE	FORTALEZA	HGF	GLÚTEOS	Enviada	Eventos Adversos Relacionados à	Lesão por pressão		
06/07/2022	CE	FORTALEZA	HGF	TROCANTER ESQUERDO	Enviada	Eventos Adversos Relacionados à	Lesão por pressão		
06/07/2022	CE	FORTALEZA	HGF	SACRA	Concluída	Eventos Adversos Relacionados à	Lesão por pressão		
06/07/2022	CE	FORTALEZA	HGF	CALCANEO ESQUERDO	Enviada	Eventos Adversos Relacionados à	Lesão por pressão		
06/07/2022	CE	FORTALEZA	HGF	SACRA	Enviada	Eventos Adversos Relacionados à	Lesão por pressão		
06/07/2022	CE	FORTALEZA	HGF	GLÚTEO DIREITO	Enviada	Eventos Adversos Relacionados à	Lesão por pressão		
06/07/2022	CE	FORTALEZA	HGF	SACRA	Enviada	Eventos Adversos Relacionados à	Lesão por pressão		
06/07/2022	CE	FORTALEZA	HGF	SACRA	Enviada	Eventos Adversos Relacionados à	Lesão por pressão		
06/07/2022	CE	FORTALEZA	HGF	CALCÂNEO DIREITO	Enviada	Eventos Adversos Relacionados à	Lesão por pressão		

06/07/2022	CE	FORTALEZA	HGF	SACRA	Enviada	Eventos Adversos Relacionados à	Lesão por pressão		
06/07/2022	CE	FORTALEZA	HGF	SACRA	Enviada	Eventos Adversos Relacionados à	Lesão por pressão		
06/07/2022	CE	FORTALEZA	HGF	SACRA	Enviada	Eventos Adversos Relacionados à	Lesão por pressão		
06/07/2022	CE	FORTALEZA	HGF	GLÚTEOS	Enviada	Eventos Adversos Relacionados à	Lesão por pressão		
06/07/2022	CE	FORTALEZA	HGF	SACRA	Enviada	Eventos Adversos Relacionados à	Lesão por pressão		
06/07/2022	CE	FORTALEZA	HGF	SACRA	Enviada	Eventos Adversos Relacionados à	Lesão por pressão		
06/07/2022	CE	FORTALEZA	HGF	SACRA	Enviada	Eventos Adversos Relacionados à	Lesão por pressão		
06/07/2022	CE	FORTALEZA	HGF	SACRA	Enviada	Eventos Adversos Relacionados à	Lesão por pressão		
06/07/2022	CE	FORTALEZA	HGF	SACRA	Concluída	Eventos Adversos Relacionados à	Lesão por pressão		
06/07/2022	CE	FORTALEZA	HGF	SACRA	Enviada	Eventos Adversos Relacionados à	Lesão por pressão		
06/07/2022	CE	FORTALEZA	HGF	SACRA	Enviada	Eventos Adversos Relacionados à	Lesão por pressão		
06/07/2022	CE	FORTALEZA	HGF	SACRA	Enviada	Eventos Adversos Relacionados à	Lesão por pressão		
06/07/2022	CE	FORTALEZA	HGF	SACRA	Enviada	Eventos Adversos Relacionados à	Lesão por pressão		
07/07/2022	CE	FORTALEZA	HGF	SACRA	Enviada	Eventos Adversos Relacionados à	Lesão por pressão		
07/07/2022	CE	FORTALEZA	HGF	SACRA	Enviada	Eventos Adversos Relacionados à	Lesão por pressão		

07/07/2022	CE	FORTALEZA	HGF	SACRA	Enviada	Eventos Adversos Relacionados à	Lesão por pressão		
07/07/2022	CE	FORTALEZA	HGF	GLÚTEO	Enviada	Eventos Adversos Relacionados à	Lesão por pressão		
07/07/2022	CE	FORTALEZA	HGF	SACRA	Enviada	Eventos Adversos Relacionados à	Lesão por pressão		
07/07/2022	CE	FORTALEZA	HGF	sacra	Enviada	Eventos Adversos Relacionados à	Lesão por pressão		
07/07/2022	CE	FORTALEZA	HGF	glúteo esquerdo	Enviada	Eventos Adversos Relacionados à	Lesão por pressão		
07/07/2022	CE	FORTALEZA	HGF	trocânter esquerdo	Enviada	Eventos Adversos Relacionados à	Lesão por pressão		
07/07/2022	CE	FORTALEZA	HGF	sacra	Enviada	Eventos Adversos Relacionados à	Lesão por pressão		
07/07/2022	CE	FORTALEZA	HGF	sacra	Concluída	Eventos Adversos Relacionados à	Lesão por pressão		
07/07/2022	CE	FORTALEZA	HGF	sacra	Enviada	Eventos Adversos Relacionados à	Lesão por pressão		
07/07/2022	CE	FORTALEZA	HGF	sacra	Enviada	Eventos Adversos Relacionados à	Lesão por pressão		
07/07/2022	CE	FORTALEZA	HGF	interglúteo	Enviada	Eventos Adversos Relacionados à	Lesão por pressão		
07/07/2022	CE	FORTALEZA	HGF	sacra	Enviada	Eventos Adversos Relacionados à	Lesão por pressão		
07/07/2022	CE	FORTALEZA	HGF	sacra	Concluída	Eventos Adversos Relacionados à	Lesão por pressão		
07/07/2022	CE	FORTALEZA	HGF	sacra	Enviada	Eventos Adversos Relacionados à	Lesão por pressão		
07/07/2022	CE	FORTALEZA	HGF	glúteo direito	Enviada	Eventos Adversos Relacionados à	Lesão por pressão		

07/07/2022	CE	FORTALEZA	HGF	sacra	Enviada	Eventos Adversos Relacionados à	Lesão por pressão		
07/07/2022	CE	FORTALEZA	HGF	calcâneos	Enviada	Eventos Adversos Relacionados à	Lesão por pressão		
07/07/2022	CE	FORTALEZA	HGF	sacra	Enviada	Eventos Adversos Relacionados à	Lesão por pressão		
07/07/2022	CE	FORTALEZA	HGF	sacra	Enviada	Eventos Adversos Relacionados à	Lesão por pressão		
07/07/2022	CE	FORTALEZA	HGF	sacra	Enviada	Eventos Adversos Relacionados à	Lesão por pressão		
07/07/2022	CE	FORTALEZA	HGF	interglúteo	Enviada	Eventos Adversos Relacionados à	Lesão por pressão		
07/07/2022	CE	FORTALEZA	HGF	dorso	Enviada	Eventos Adversos Relacionados à	Lesão por pressão		
07/07/2022	CE	FORTALEZA	HGF	trocânteres	Enviada	Eventos Adversos Relacionados à	Lesão por pressão		
07/07/2022	CE	FORTALEZA	HGF	glúteo direito	Enviada	Eventos Adversos Relacionados à	Lesão por pressão		
07/07/2022	CE	FORTALEZA	HGF	dorso	Enviada	Eventos Adversos Relacionados à	Lesão por pressão		
07/07/2022	CE	FORTALEZA	HGF	sacra	Concluída	Eventos Adversos Relacionados à	Lesão por pressão		
07/07/2022	CE	FORTALEZA	HGF	sacra	Enviada	Eventos Adversos Relacionados à	Lesão por pressão		
07/07/2022	CE	FORTALEZA	HGF	sacra	Enviada	Eventos Adversos Relacionados à	Lesão por pressão		
07/07/2022	CE	FORTALEZA	HGF	sacra	Enviada	Eventos Adversos Relacionados à	Lesão por pressão		
07/07/2022	CE	FORTALEZA	HGF	sacra	Enviada	Eventos Adversos Relacionados à	Lesão por pressão		

07/07/2022	CE	FORTALEZA	HGF	sacra	Enviada	Eventos Adversos Relacionados à	Lesão por pressão		
07/07/2022	CE	FORTALEZA	HGF	sacra	Enviada	Eventos Adversos Relacionados à	Lesão por pressão		
07/07/2022	CE	FORTALEZA	HGF	sacra	Enviada	Eventos Adversos Relacionados à	Lesão por pressão		
07/07/2022	CE	FORTALEZA	HGF	sacra	Enviada	Eventos Adversos Relacionados à	Lesão por pressão		
07/07/2022	CE	FORTALEZA	HGF	sacra	Enviada	Eventos Adversos Relacionados à	Lesão por pressão		
07/07/2022	CE	FORTALEZA	HGF	sacra	Enviada	Eventos Adversos Relacionados à	Lesão por pressão		
07/07/2022	CE	FORTALEZA	HGF	sacra	Enviada	Eventos Adversos Relacionados à	Lesão por pressão		
07/07/2022	CE	FORTALEZA	HGF	SACRA	Enviada	Eventos Adversos Relacionados à	Lesão por pressão		
07/07/2022	CE	FORTALEZA	HGF	SACRA	Enviada	Eventos Adversos Relacionados à	Lesão por pressão		
07/07/2022	CE	FORTALEZA	HGF	SACRA	Enviada	Eventos Adversos Relacionados à	Lesão por pressão		
07/07/2022	CE	FORTALEZA	HGF	SACRA	Enviada	Eventos Adversos Relacionados à	Lesão por pressão		
07/07/2022	CE	FORTALEZA	HGF	SACRA	Enviada	Eventos Adversos Relacionados à	Lesão por pressão		
07/07/2022	CE	FORTALEZA	HGF	SACRA	Enviada	Eventos Adversos Relacionados à	Lesão por pressão		
07/07/2022	CE	FORTALEZA	HGF	SACRA	Enviada	Eventos Adversos Relacionados à	Lesão por pressão		
07/07/2022	CE	FORTALEZA	HGF	GLÚTEO DIREITO	Enviada	Eventos Adversos Relacionados à	Lesão por pressão		

07/07/2022	CE	FORTALEZA	HGF	INTERGLÚTEO	Enviada	Eventos Adversos Relacionados à	Lesão por pressão		
07/07/2022	CE	FORTALEZA	HGF	SACRA	Enviada	Eventos Adversos Relacionados à	Lesão por pressão		
07/07/2022	CE	FORTALEZA	HGF	SACRA	Enviada	Eventos Adversos Relacionados à	Lesão por pressão		
07/07/2022	CE	FORTALEZA	HGF	SACRA	Enviada	Eventos Adversos Relacionados à	Lesão por pressão		
07/07/2022	CE	FORTALEZA	HGF	OCCIPITAL	Enviada	Eventos Adversos Relacionados à	Lesão por pressão		
07/07/2022	CE	FORTALEZA	HGF	SACRA	Enviada	Eventos Adversos Relacionados à	Lesão por pressão		
07/07/2022	CE	FORTALEZA	HGF	SACRA	Enviada	Eventos Adversos Relacionados à	Lesão por pressão		
07/07/2022	CE	FORTALEZA	HGF	GLÚTEO DIREITO	Enviada	Eventos Adversos Relacionados à	Lesão por pressão		
07/07/2022	CE	FORTALEZA	HGF	SACRA	Enviada	Eventos Adversos Relacionados à	Lesão por pressão		
07/07/2022	CE	FORTALEZA	HGF	SACRA	Enviada	Eventos Adversos Relacionados à	Lesão por pressão		
07/07/2022	CE	FORTALEZA	HGF	SACRA	Enviada	Eventos Adversos Relacionados à	Lesão por pressão		
07/07/2022	CE	FORTALEZA	HGF	SACRA	Enviada	Eventos Adversos Relacionados à	Lesão por pressão		
14/07/2022	CE	FORTALEZA	HGF	Apos procedimento no setor de	Enviada	Eventos Adversos Relacionados à	Falhas durante a assistência à saúde		
18/07/2022	CE	FORTALEZA	HGF	Paciente evoluiu com sangrame	Enviada	Eventos Adversos Relacionados à	Falhas durante procedimento		
18/07/2022	CE	FORTALEZA	HGF	-	Enviada	Eventos Adversos Relacionados à	Falhas durante procedimento		

27/07/2022	CE	FORTALEZA	HGF	PUNCIONADO CATETER	Enviada	Eventos Adversos Relacionados à	Falhas durante a assistência à saúde		
27/07/2022	CE	FORTALEZA	HGF	PACIENTE DESORIENTADO	Enviada	Eventos Adversos Relacionados à	Falhas envolvendo sondas		
27/07/2022	CE	FORTALEZA	HGF	AO REALIZAR HIGIENE	Enviada	Eventos Adversos Relacionados à	Falhas durante a assistência à saúde		
27/07/2022	CE	FORTALEZA	HGF	Ao descer da cama, paciente	Enviada	Eventos Adversos Relacionados à	Queda do paciente		
28/07/2022	CE	FORTALEZA	HGF	SACRA	Enviada	Eventos Adversos Relacionados à	Lesão por pressão		
28/07/2022	CE	FORTALEZA	HGF	SACRA	Enviada	Eventos Adversos Relacionados à	Lesão por pressão		
28/07/2022	CE	FORTALEZA	HGF	SACRA	Enviada	Eventos Adversos Relacionados à	Lesão por pressão		
28/07/2022	CE	FORTALEZA	HGF	SACRA	Enviada	Eventos Adversos Relacionados à	Lesão por pressão		
28/07/2022	CE	FORTALEZA	HGF	GLÚTEO ESQUERDO	Enviada	Eventos Adversos Relacionados à	Lesão por pressão		
28/07/2022	CE	FORTALEZA	HGF	SACRA	Enviada	Eventos Adversos Relacionados à	Lesão por pressão		
28/07/2022	CE	FORTALEZA	HGF	SACRA	Enviada	Eventos Adversos Relacionados à	Lesão por pressão		
28/07/2022	CE	FORTALEZA	HGF	SACRA	Enviada	Eventos Adversos Relacionados à	Lesão por pressão		
28/07/2022	CE	FORTALEZA	HGF	SACRA	Enviada	Eventos Adversos Relacionados à	Lesão por pressão		
28/07/2022	CE	FORTALEZA	HGF	LPP EM GLÚTEO ESQUERDO	Enviada	Eventos Adversos Relacionados à	Lesão por pressão		
28/07/2022	CE	FORTALEZA	HGF	LPP SACRA	Concluída	Eventos Adversos Relacionados à	Lesão por pressão		

28/07/2022	CE	FORTALEZA	HGF	LPP SACRA	Enviada	Eventos Adversos Relacionados à	Lesão por pressão		
28/07/2022	CE	FORTALEZA	HGF	LPP SACRA	Enviada	Eventos Adversos Relacionados à	Lesão por pressão		
28/07/2022	CE	FORTALEZA	HGF	LPP SACRA	Enviada	Eventos Adversos Relacionados à	Lesão por pressão		
28/07/2022	CE	FORTALEZA	HGF	LPP SACRA	Enviada	Eventos Adversos Relacionados à	Lesão por pressão		
28/07/2022	CE	FORTALEZA	HGF	LPP SACRA	Enviada	Eventos Adversos Relacionados à	Lesão por pressão		
28/07/2022	CE	FORTALEZA	HGF	LPP SACRA	Enviada	Eventos Adversos Relacionados à	Lesão por pressão		
28/07/2022	CE	FORTALEZA	HGF	LPP SACRA	Enviada	Eventos Adversos Relacionados à	Lesão por pressão		
28/07/2022	CE	FORTALEZA	HGF	LPP EM CALCÂNEO DIREITO	Enviada	Eventos Adversos Relacionados à	Lesão por pressão		
28/07/2022	CE	FORTALEZA	HGF	LPP SACRA	Enviada	Eventos Adversos Relacionados à	Lesão por pressão		
28/07/2022	CE	FORTALEZA	HGF	SACRA	Enviada	Eventos Adversos Relacionados à	Lesão por pressão		
28/07/2022	CE	FORTALEZA	HGF	SACRA	Enviada	Eventos Adversos Relacionados à	Lesão por pressão		
28/07/2022	CE	FORTALEZA	HGF	ESCAPULA ESQUERDA	Enviada	Eventos Adversos Relacionados à	Lesão por pressão		
28/07/2022	CE	FORTALEZA	HGF	CALCÂNEO DIREITO	Enviada	Eventos Adversos Relacionados à	Lesão por pressão		
28/07/2022	CE	FORTALEZA	HGF	SACRA	Enviada	Eventos Adversos Relacionados à	Lesão por pressão		
28/07/2022	CE	FORTALEZA	HGF	SACRA	Enviada	Eventos Adversos Relacionados à	Lesão por pressão		

28/07/2022	CE	FORTALEZA	HGF	SACRA	Enviada	Eventos Adversos Relacionados à	Lesão por pressão		
28/07/2022	CE	FORTALEZA	HGF	SACRA	Enviada	Eventos Adversos Relacionados à	Lesão por pressão		
28/07/2022	CE	FORTALEZA	HGF	SACRA	Enviada	Eventos Adversos Relacionados à	Lesão por pressão		
28/07/2022	CE	FORTALEZA	HGF	SACRA	Enviada	Eventos Adversos Relacionados à	Lesão por pressão		
28/07/2022	CE	FORTALEZA	HGF	CALCÂNEO DIREITO	Enviada	Eventos Adversos Relacionados à	Lesão por pressão		
28/07/2022	CE	FORTALEZA	HGF	SACRA	Enviada	Eventos Adversos Relacionados à	Lesão por pressão		
28/07/2022	CE	FORTALEZA	HGF	CALCÂNEO DIREITO	Enviada	Eventos Adversos Relacionados à	Lesão por pressão		
28/07/2022	CE	FORTALEZA	HGF	SACRA	Enviada	Eventos Adversos Relacionados à	Lesão por pressão		
28/07/2022	CE	FORTALEZA	HGF	CALCÂNEO DIREITO	Enviada	Eventos Adversos Relacionados à	Lesão por pressão		
28/07/2022	CE	FORTALEZA	HGF	SACRA	Enviada	Eventos Adversos Relacionados à	Lesão por pressão		
28/07/2022	CE	FORTALEZA	HGF	GLÚTEO ESQUERDO	Enviada	Eventos Adversos Relacionados à	Lesão por pressão		
28/07/2022	CE	FORTALEZA	HGF	CALCÂNEO DIREITO	Enviada	Eventos Adversos Relacionados à	Lesão por pressão		
28/07/2022	CE	FORTALEZA	HGF	SACRA	Enviada	Eventos Adversos Relacionados à	Lesão por pressão		
28/07/2022	CE	FORTALEZA	HGF	SACRA	Enviada	Eventos Adversos Relacionados à	Lesão por pressão		
28/07/2022	CE	FORTALEZA	HGF	CALCÂNEO DIREITO	Enviada	Eventos Adversos Relacionados à	Lesão por pressão		

28/07/2022	CE	FORTALEZA	HGF	SACRA	Enviada	Eventos Adversos Relacionados à	Lesão por pressão		
28/07/2022	CE	FORTALEZA	HGF	SACRA	Enviada	Eventos Adversos Relacionados à	Lesão por pressão		
28/07/2022	CE	FORTALEZA	HGF	SACRA	Enviada	Eventos Adversos Relacionados à	Lesão por pressão		
28/07/2022	CE	FORTALEZA	HGF	SACRA	Concluída	Eventos Adversos Relacionados à	Lesão por pressão		
28/07/2022	CE	FORTALEZA	HGF	TROCÂNTER DIREITO	Enviada	Eventos Adversos Relacionados à	Lesão por pressão		
28/07/2022	CE	FORTALEZA	HGF	SACRA	Enviada	Eventos Adversos Relacionados à	Lesão por pressão		
28/07/2022	CE	FORTALEZA	HGF	SACRA	Enviada	Eventos Adversos Relacionados à	Lesão por pressão		
28/07/2022	CE	FORTALEZA	HGF	SACRA	Enviada	Eventos Adversos Relacionados à	Lesão por pressão		
28/07/2022	CE	FORTALEZA	HGF	PAVILHAO AURICULAR	Enviada	Eventos Adversos Relacionados à	Lesão por pressão		
28/07/2022	CE	FORTALEZA	HGF	SACRA	Enviada	Eventos Adversos Relacionados à	Lesão por pressão		
28/07/2022	CE	FORTALEZA	HGF	SACRA	Enviada	Eventos Adversos Relacionados à	Lesão por pressão		
28/07/2022	CE	FORTALEZA	HGF	SACRA	Enviada	Eventos Adversos Relacionados à	Lesão por pressão		
28/07/2022	CE	FORTALEZA	HGF	SACRA	Enviada	Eventos Adversos Relacionados à	Lesão por pressão		
28/07/2022	CE	FORTALEZA	HGF	INTERGLÚTEO	Enviada	Eventos Adversos Relacionados à	Lesão por pressão		
28/07/2022	CE	FORTALEZA	HGF	SACRA	Enviada	Eventos Adversos Relacionados à	Lesão por pressão		

28/07/2022	CE	FORTALEZA	HGF	Sacra	Enviada	Eventos Adversos Relacionados à	Lesão por pressão		
28/07/2022	CE	FORTALEZA	HGF	SACRA	Enviada	Eventos Adversos Relacionados à	Lesão por pressão		
28/07/2022	CE	FORTALEZA	HGF	SACRA	Enviada	Eventos Adversos Relacionados à	Lesão por pressão		
28/07/2022	CE	FORTALEZA	HGF	CALCÂNEO DIREITO	Enviada	Eventos Adversos Relacionados à	Lesão por pressão		
28/07/2022	CE	FORTALEZA	HGF	SACRA	Concluída	Eventos Adversos Relacionados à	Lesão por pressão		
28/07/2022	CE	FORTALEZA	HGF	SACRA	Enviada	Eventos Adversos Relacionados à	Lesão por pressão		
28/07/2022	CE	FORTALEZA	HGF	CALCÂNEO DIREITO	Enviada	Eventos Adversos Relacionados à	Lesão por pressão		
28/07/2022	CE	FORTALEZA	HGF	CALCÂNEO DIREITO	Enviada	Eventos Adversos Relacionados à	Lesão por pressão		
28/07/2022	CE	FORTALEZA	HGF	CALCÂNEO DIREITO	Concluída	Eventos Adversos Relacionados à	Lesão por pressão		
29/07/2022	CE	FORTALEZA	HGF	FOI ENCAMINHADA PARA	Enviada	Eventos Adversos Relacionados à	Queda do paciente		
29/07/2022	CE	FORTALEZA	HGF	Paciente em PCR, ia ser realizada	Enviada	Eventos Adversos Relacionados à	Falhas durante a assistência à saúde		
29/07/2022	CE	FORTALEZA	HGF	Infusão de fentanil mais	Enviada	Eventos Adversos Relacionados à	Falhas durante a assistência à saúde		
29/07/2022	CE	FORTALEZA	HGF	Paciente caiu da propria altura ao	Enviada	Eventos Adversos Relacionados à	Queda do paciente		
29/07/2022	CE	FORTALEZA	HGF	No banho, paciente fez apoio	Enviada	Eventos Adversos Relacionados à	Queda do paciente		
29/07/2022	CE	FORTALEZA	HGF	PACIENTE EM BIC DE AMIODA	Enviada	Eventos Adversos Relacionados à	Falhas durante a assistência à saúde		

29/07/2022	CE	FORTALEZA	HGF	FLEBITE E DEMORA PARA	Enviada	Eventos Adversos Relacionados à	Falhas envolvendo o cateter venoso		
29/07/2022	CE	FORTALEZA	HGF	ACOMPANHANTE RETIROU	Enviada	Eventos Adversos Relacionados à	Queda do paciente		
08/08/2022	CE	FORTALEZA	HGF	ADMINISTRADO DOSE DE	Enviada	Eventos Adversos Relacionados à	Falhas durante a assistência à saúde		
08/08/2022	CE	FORTALEZA	HGF	DILUIÇAO INCORRETA DE	Enviada	Eventos Adversos Relacionados à	Falhas durante a assistência à saúde		
08/08/2022	CE	FORTALEZA	HGF	Paciente ao se dirigir para	Enviada	Eventos Adversos Relacionados à	Queda do paciente		
08/08/2022	CE	FORTALEZA	HGF	Queda da própria altura ao levantar	Enviada	Eventos Adversos Relacionados à	Queda do paciente		
09/08/2022	CE	FORTALEZA	HGF	Paciente sofreu uma queda de	Enviada	Eventos Adversos Relacionados à	Queda do paciente		
29/08/2022	CE	FORTALEZA	HGF	Encontrado o paciente com	Enviada	Eventos Adversos Relacionados à	Falhas durante a assistência à saúde		
29/08/2022	CE	FORTALEZA	HGF	PACIENTE APRESENTOU	Enviada	Eventos Adversos Relacionados à	Queda do paciente		
29/08/2022	CE	FORTALEZA	HGF	Paciente passou mais de 6 horas	Enviada	Eventos Adversos Relacionados à	Falhas durante a assistência à saúde		
29/08/2022	CE	FORTALEZA	HGF	Paciente sofreu queda de própria	Enviada	Eventos Adversos Relacionados à	Queda do paciente		
29/08/2022	CE	FORTALEZA	HGF	FLEBITE EM MSD (ANTEBR	Enviada	Eventos Adversos Relacionados à	Falhas envolvendo o cateter venoso		
30/08/2022	CE	FORTALEZA	HGF	Paciente se encontrava sozinha	Enviada	Eventos Adversos Relacionados à	Queda do paciente		
30/08/2022	CE	FORTALEZA	HGF	Flebite em MSE, HIPEREMIA E	Enviada	Eventos Adversos Relacionados à	Falhas envolvendo o cateter venoso		
30/08/2022	CE	FORTALEZA	HGF	FLEBITE EM MSE , DOR E HIPEREM	Enviada	Eventos Adversos Relacionados à	Falhas envolvendo o cateter venoso		

30/08/2022	CE	FORTALE ZA	HGF	-	Enviada	Eventos Adversos Relaciona dos à	Falhas envolvend o cateter venoso		
30/08/2022	CE	FORTALE ZA	HGF	Flebite em MSE	Enviada	Eventos Adversos Relaciona dos à	Falhas envolvend o cateter venoso		
30/08/2022	CE	FORTALE ZA	HGF	-	Enviada	Eventos Adversos Relaciona dos à	Falhas envolvend o cateter venoso		
09/09/2022	CE	FORTALE ZA	HGF	EXAME LABORA TORIAL DE	Enviada	Eventos Adversos Relaciona dos à	Falha na identificaç ão do paciente		
09/09/2022	CE	FORTALE ZA	HGF	FALHA DA DISPENS ACAO	Enviada	Eventos Adversos Relaciona dos à	Falhas durante a assistênci a à saúde		
09/09/2022	CE	FORTALE ZA	HGF	Paciente intubado encontrad o pela	Enviada	Eventos Adversos Relaciona dos à	Falhas durante a assistênci a à saúde		
26/09/2022	CE	FORTALE ZA	HGF	Caiu do berço quando mãe foi	Enviada	Eventos Adversos Relaciona dos à	Queda do paciente		
26/09/2022	CE	FORTALE ZA	HGF	HIPEREM IA, CALOR E	Enviada	Eventos Adversos Relaciona dos à	Falhas envolvend o cateter venoso		
26/09/2022	CE	FORTALE ZA	HGF	Paciente escorrego u no banheiro	Enviada	Eventos Adversos Relaciona dos à	Queda do paciente		
26/09/2022	CE	FORTALE ZA	HGF	Paciente estava no banheiro e referiu	Enviada	Eventos Adversos Relaciona dos à	Queda do paciente		
26/09/2022	CE	FORTALE ZA	HGF	PACIENT E FOI AO BANHEIR	Enviada	Eventos Adversos Relaciona dos à	Queda do paciente		
26/09/2022	CE	FORTALE ZA	HGF	PACIENT E FOI AO BANHEIR	Enviada	Eventos Adversos Relaciona dos à	Queda do paciente		
26/09/2022	CE	FORTALE ZA	HGF	Fisioterap euta não atendeu aos	Enviada	Eventos Adversos Relaciona dos à	Falhas durante a assistênci a à saúde		
10/10/2022	CE	FORTALE ZA	HGF	OBSERV ADO PRESEN CA DE	Enviada	Eventos Adversos Relaciona dos à	Falhas envolvend o cateter venoso		
10/10/2022	CE	FORTALE ZA	HGF	LESÃO EM REGIÃO SACRAL	Enviada	Eventos Adversos Relaciona dos à	Lesão por pressão		

10/10/2022	CE	FORTALEZA	HGF	Escorregou em piso molhado. Não	Enviada	Eventos Adversos Relacionados à	Queda do paciente		
10/10/2022	CE	FORTALEZA	HGF	LESÃO REGIÃO SACRAL	Enviada	Eventos Adversos Relacionados à	Lesão por pressão		
10/10/2022	CE	FORTALEZA	HGF	Paciente foi ao banheiro realizar o	Enviada	Eventos Adversos Relacionados à	Queda do paciente		
10/10/2022	CE	FORTALEZA	HGF	LESÃO REGIÃO SACRAL	Enviada	Eventos Adversos Relacionados à	Lesão por pressão		
10/10/2022	CE	FORTALEZA	HGF	LESÃO EM REGIÃO SACRAL	Enviada	Eventos Adversos Relacionados à	Lesão por pressão		
10/10/2022	CE	FORTALEZA	HGF	PACIENTE EM QUIMIOTERAPIA	Enviada	Eventos Adversos Relacionados à	Falhas durante a assistência à saúde		
10/10/2022	CE	FORTALEZA	HGF	LESÃO EM REGIÃO SACRAL	Enviada	Eventos Adversos Relacionados à	Lesão por pressão		
10/10/2022	CE	FORTALEZA	HGF	LESÃO EM REGIÃO SACRAL	Enviada	Eventos Adversos Relacionados à	Lesão por pressão		
10/10/2022	CE	FORTALEZA	HGF	LESÃO EM REGIÃO TROCAN	Enviada	Eventos Adversos Relacionados à	Lesão por pressão		
10/10/2022	CE	FORTALEZA	HGF	Demora na Administração de	Enviada	Eventos Adversos Relacionados à	Falhas durante a assistência à saúde		
10/10/2022	CE	FORTALEZA	HGF	LESÃO EM PAVILHÃO	Enviada	Eventos Adversos Relacionados à	Lesão por pressão		
10/10/2022	CE	FORTALEZA	HGF	LESÃO EM REGIÃO SACRAL	Enviada	Eventos Adversos Relacionados à	Lesão por pressão		
10/10/2022	CE	FORTALEZA	HGF	FLEBITE EM REGIÃO ANTERIO	Enviada	Eventos Adversos Relacionados à	Falhas envolvendo cateter venoso		
10/10/2022	CE	FORTALEZA	HGF	LESÃO EM REGIÃO LOMBAR	Enviada	Eventos Adversos Relacionados à	Lesão por pressão		
10/10/2022	CE	FORTALEZA	HGF	LESÃO EM GLUTEO	Enviada	Eventos Adversos Relacionados à	Lesão por pressão		

10/10/2022	CE	FORTALEZA	HGF	LESÃO EM MSE	Enviada	Eventos Adversos Relacionados à	Lesão por pressão		
10/10/2022	CE	FORTALEZA	HGF	LESÃO EM REGIÃO TROCAN	Enviada	Eventos Adversos Relacionados à	Lesão por pressão		
10/10/2022	CE	FORTALEZA	HGF	PACIENTE PROVENIENTE DA	Enviada	Eventos Adversos Relacionados à	Queda do paciente		
10/10/2022	CE	FORTALEZA	HGF	LESÃO EM MID	Enviada	Eventos Adversos Relacionados à	Lesão por pressão		
10/10/2022	CE	FORTALEZA	HGF	LESÃO EM REGIÃO SACRAL	Enviada	Eventos Adversos Relacionados à	Lesão por pressão		
10/10/2022	CE	FORTALEZA	HGF	Paciente desorientada cai do leito na	Enviada	Eventos Adversos Relacionados à	Queda do paciente		
10/10/2022	CE	FORTALEZA	HGF	LESÃO EM GLUTEO D	Enviada	Eventos Adversos Relacionados à	Lesão por pressão		
10/10/2022	CE	FORTALEZA	HGF	LESÃO EM REGIÃO SACRAL	Enviada	Eventos Adversos Relacionados à	Lesão por pressão		
10/10/2022	CE	FORTALEZA	HGF	Acompanhante relata que	Enviada	Eventos Adversos Relacionados à	Queda do paciente		
10/10/2022	CE	FORTALEZA	HGF	LESÃO EM REGIÃO SACRAL	Enviada	Eventos Adversos Relacionados à	Lesão por pressão		
10/10/2022	CE	FORTALEZA	HGF	LESÃO EM REGIÃO SACRAL	Enviada	Eventos Adversos Relacionados à	Lesão por pressão		
10/10/2022	CE	FORTALEZA	HGF	LESÃO EM DORSO	Enviada	Eventos Adversos Relacionados à	Lesão por pressão		
10/10/2022	CE	FORTALEZA	HGF	LESÃO EM REGIÃO SACRAL	Concluída	Eventos Adversos Relacionados à	Lesão por pressão		
10/10/2022	CE	FORTALEZA	HGF	PACIENTE APRESENTA	Enviada	Eventos Adversos Relacionados à	Falhas envolvendo o cateter venoso		
10/10/2022	CE	FORTALEZA	HGF	-	Enviada	Eventos Adversos Relacionados à	Falhas envolvendo o cateter venoso		

10/10/2022	CE	FORTALEZA	HGF	RETIRADA TRAUMÁTICA DE	Enviada	Eventos Adversos Relacionados à	Falhas durante a assistência à saúde		
10/10/2022	CE	FORTALEZA	HGF	Paciente apresentou flebite devido	Enviada	Eventos Adversos Relacionados à	Falhas envolvendo cateter venoso		
10/10/2022	CE	FORTALEZA	HGF	FALHA DE CONDUTA DE	Enviada	Eventos Adversos Relacionados à	Falhas durante a assistência à saúde		
10/10/2022	CE	FORTALEZA	HGF	HIPEREMIA, CALOR E	Enviada	Eventos Adversos Relacionados à	Falhas envolvendo cateter venoso		
10/10/2022	CE	FORTALEZA	HGF	RETIRADO PELO MEDICO(SEGUND	Enviada	Eventos Adversos Relacionados à	Queda do paciente		
10/10/2022	CE	FORTALEZA	HGF	LESÃO EM REGIÃO SACRAL	Enviada	Eventos Adversos Relacionados à	Lesão por pressão		
10/10/2022	CE	FORTALEZA	HGF	Falta de respeito ao fluxo e com a	Enviada	Eventos Adversos Relacionados à	Falhas nas atividades		
10/10/2022	CE	FORTALEZA	HGF	LESÃO EM REGIÃO SACRA	Enviada	Eventos Adversos Relacionados à	Lesão por pressão		
10/10/2022	CE	FORTALEZA	HGF	LESÃO EM REGIÃO SACRAL	Enviada	Eventos Adversos Relacionados à	Lesão por pressão		
10/10/2022	CE	FORTALEZA	HGF	SOLICITADO RAIO-X , PORÉM	Enviada	Eventos Adversos Relacionados à	Falhas na administração de dietas		Enteral (oral, cateter oral ou
10/10/2022	CE	FORTALEZA	HGF	LESÃO EM TROCANTER	Enviada	Eventos Adversos Relacionados à	Lesão por pressão		
10/10/2022	CE	FORTALEZA	HGF	LESÃO EM REGIÃO SACRAL	Concluída	Eventos Adversos Relacionados à	Lesão por pressão		
10/10/2022	CE	FORTALEZA	HGF	LESÃO EM REGIÃO SACRAL	Enviada	Eventos Adversos Relacionados à	Lesão por pressão		
10/10/2022	CE	FORTALEZA	HGF	ACOMPANHANTE DE PACIENT	Enviada	Eventos Adversos Relacionados à	Falha na identificação do paciente		
10/10/2022	CE	FORTALEZA	HGF	LESÃO EM REGIÃO SACRAL	Enviada	Eventos Adversos Relacionados à	Lesão por pressão		

10/10/2022	CE	FORTALEZA	HGF	LESÃO EM REGIÃO DORSAL	Enviada	Eventos Adversos Relacionados à	Lesão por pressão		
10/10/2022	CE	FORTALEZA	HGF	LESÃO EM REGIÃO SACRAL	Enviada	Eventos Adversos Relacionados à	Lesão por pressão		
10/10/2022	CE	FORTALEZA	HGF	LESÃO EM REGIÃO INTERGL	Enviada	Eventos Adversos Relacionados à	Lesão por pressão		
10/10/2022	CE	FORTALEZA	HGF	LESÃO EM REGIÃO SACRAL	Enviada	Eventos Adversos Relacionados à	Lesão por pressão		
10/10/2022	CE	FORTALEZA	HGF	LESÃO EM GLUTEO ESQUER	Enviada	Eventos Adversos Relacionados à	Lesão por pressão		
10/10/2022	CE	FORTALEZA	HGF	LESÃO EM REGIÃO SACRAL	Enviada	Eventos Adversos Relacionados à	Lesão por pressão		
10/10/2022	CE	FORTALEZA	HGF	LESÃO EM REGIÃO SACRAL	Enviada	Eventos Adversos Relacionados à	Lesão por pressão		
10/10/2022	CE	FORTALEZA	HGF	LESÃO EM REGIÃO SACRAL	Enviada	Eventos Adversos Relacionados à	Lesão por pressão		
10/10/2022	CE	FORTALEZA	HGF	Flebite em MSD, realizo retirada	Enviada	Eventos Adversos Relacionados à	Falhas envolvendo o cateter venoso		
10/10/2022	CE	FORTALEZA	HGF	Flebite em MID, apresentando	Enviada	Eventos Adversos Relacionados à	Falhas envolvendo o cateter venoso		
10/10/2022	CE	FORTALEZA	HGF	Caiu da própria altura pela	Enviada	Eventos Adversos Relacionados à	Queda do paciente		
10/10/2022	CE	FORTALEZA	HGF	LESÃO EM REGIÃO SACRAL	Enviada	Eventos Adversos Relacionados à	Lesão por pressão		
10/10/2022	CE	FORTALEZA	HGF	LESÃO EM REGIÃO SACRAL	Enviada	Eventos Adversos Relacionados à	Lesão por pressão		
10/10/2022	CE	FORTALEZA	HGF	LESÃO EM REGIÃO DORSAL	Enviada	Eventos Adversos Relacionados à	Lesão por pressão		
10/10/2022	CE	FORTALEZA	HGF	LESÃO EM FIBULA ESQUER	Enviada	Eventos Adversos Relacionados à	Lesão por pressão		

10/10/2022	CE	FORTALEZA	HGF	LESÃO EM REGIÃO SACRAL	Enviada	Eventos Adversos Relacionados à	Lesão por pressão		
10/10/2022	CE	FORTALEZA	HGF	LESÃO EM REGIÃO SACRAL	Enviada	Eventos Adversos Relacionados à	Lesão por pressão		
10/10/2022	CE	FORTALEZA	HGF	LESÃO EM REGIÃO SACRAL	Enviada	Eventos Adversos Relacionados à	Lesão por pressão		
10/10/2022	CE	FORTALEZA	HGF	LESÃO EM GLUTEO DIREITO	Enviada	Eventos Adversos Relacionados à	Lesão por pressão		
10/10/2022	CE	FORTALEZA	HGF	Paciente encontrava-se na cadeira	Enviada	Eventos Adversos Relacionados à	Queda do paciente		
10/10/2022	CE	FORTALEZA	HGF	LESÃO EM REGIÃO TROCAN	Enviada	Eventos Adversos Relacionados à	Lesão por pressão		
10/10/2022	CE	FORTALEZA	HGF	LESÃO EM REGIÃO SACRAL	Enviada	Eventos Adversos Relacionados à	Lesão por pressão		
10/10/2022	CE	FORTALEZA	HGF	LESÃO EM REGIÃO SACRAL	Enviada	Eventos Adversos Relacionados à	Lesão por pressão		
10/10/2022	CE	FORTALEZA	HGF	LESÃO EM REGIÃO SACRAL	Enviada	Eventos Adversos Relacionados à	Lesão por pressão		
10/10/2022	CE	FORTALEZA	HGF	LESÃO EM REGIÃO SACRAL	Enviada	Eventos Adversos Relacionados à	Lesão por pressão		
10/10/2022	CE	FORTALEZA	HGF	LESÃO EM REGIÃO SACRAL	Enviada	Eventos Adversos Relacionados à	Lesão por pressão		
10/10/2022	CE	FORTALEZA	HGF	LESÃO EM REGIÃO SACRAL	Enviada	Eventos Adversos Relacionados à	Lesão por pressão		
10/10/2022	CE	FORTALEZA	HGF	LESÃO EM REGIÃO SACRAL	Enviada	Eventos Adversos Relacionados à	Lesão por pressão		
10/10/2022	CE	FORTALEZA	HGF	LESÃO EM, REGIÃO SACRAL	Enviada	Eventos Adversos Relacionados à	Lesão por pressão		
10/10/2022	CE	FORTALEZA	HGF	LESÃO EM REGIÃO SACRAL	Enviada	Eventos Adversos Relacionados à	Lesão por pressão		

10/10/2022	CE	FORTALEZA	HGF	LESÃO EM REGIÃO SACRAL	Enviada	Eventos Adversos Relacionados à	Lesão por pressão		
10/10/2022	CE	FORTALEZA	HGF	LESÃO EM REGIÃO SACRAL	Enviada	Eventos Adversos Relacionados à	Lesão por pressão		
10/10/2022	CE	FORTALEZA	HGF	Flebite - infiltração em MSD	Enviada	Eventos Adversos Relacionados à	Falhas envolvendo o cateter venoso		
10/10/2022	CE	FORTALEZA	HGF	LESÃO EM REGIÃO SACRAL	Enviada	Eventos Adversos Relacionados à	Lesão por pressão		
10/10/2022	CE	FORTALEZA	HGF	LESÃO EM REGIÃO SACRAL	Enviada	Eventos Adversos Relacionados à	Lesão por pressão		
10/10/2022	CE	FORTALEZA	HGF	LESÃO EM REGIÃO SACRAL	Enviada	Eventos Adversos Relacionados à	Lesão por pressão		
10/10/2022	CE	FORTALEZA	HGF	Paciente perdeu radioterapia pois	Enviada	Eventos Adversos Relacionados à	Falhas durante a assistência à saúde		
10/10/2022	CE	FORTALEZA	HGF	LESÃO EM REGIÃO SACRAL	Enviada	Eventos Adversos Relacionados à	Lesão por pressão		
10/10/2022	CE	FORTALEZA	HGF	LESÃO EM REGIÃO SACRAL	Enviada	Eventos Adversos Relacionados à	Lesão por pressão		
10/10/2022	CE	FORTALEZA	HGF	LESÃO EM REGIÃO SACRAL	Enviada	Eventos Adversos Relacionados à	Lesão por pressão		
10/10/2022	CE	FORTALEZA	HGF	LESÃO EM REGIÃO SACRAL	Enviada	Eventos Adversos Relacionados à	Lesão por pressão		
10/10/2022	CE	FORTALEZA	HGF	LESÃO EM GLUTEO	Enviada	Eventos Adversos Relacionados à	Lesão por pressão		
10/10/2022	CE	FORTALEZA	HGF	LESÃO EM REGIÃO SACRAL	Enviada	Eventos Adversos Relacionados à	Lesão por pressão		
10/10/2022	CE	FORTALEZA	HGF	LESÃO EM REGIÃO INTERGL	Enviada	Eventos Adversos Relacionados à	Lesão por pressão		
10/10/2022	CE	FORTALEZA	HGF	LESÃO EM GLUTEO D	Enviada	Eventos Adversos Relacionados à	Lesão por pressão		

10/10/2022	CE	FORTALEZA	HGF	LESÃO EM REGIÃO SACRAL	Enviada	Eventos Adversos Relacionados à	Lesão por pressão		
10/10/2022	CE	FORTALEZA	HGF	LESÃO EM REGIÃO SACRAL	Enviada	Eventos Adversos Relacionados à	Lesão por pressão		
10/10/2022	CE	FORTALEZA	HGF	LESÃO EM REGIÃO INTERGL	Enviada	Eventos Adversos Relacionados à	Lesão por pressão		
10/10/2022	CE	FORTALEZA	HGF	LESÃO EM TROCANTER	Enviada	Eventos Adversos Relacionados à	Lesão por pressão		
10/10/2022	CE	FORTALEZA	HGF	LESÃO EM TROCANTER	Enviada	Eventos Adversos Relacionados à	Lesão por pressão		
10/10/2022	CE	FORTALEZA	HGF	LESÃO EM MALEOLO	Enviada	Eventos Adversos Relacionados à	Lesão por pressão		
10/10/2022	CE	FORTALEZA	HGF	LESÃO EM MALEOLO	Enviada	Eventos Adversos Relacionados à	Lesão por pressão		
10/10/2022	CE	FORTALEZA	HGF	LESÃO EM GLUTEO	Enviada	Eventos Adversos Relacionados à	Lesão por pressão		
10/10/2022	CE	FORTALEZA	HGF	Paciente desorientado, sem contenção	Enviada	Eventos Adversos Relacionados à	Queda do paciente		
10/10/2022	CE	FORTALEZA	HGF	APRESENTA HIPEREMIA	Enviada	Eventos Adversos Relacionados à	Falhas envolvendo o cateter venoso		
10/10/2022	CE	FORTALEZA	HGF	FLEBITE MSD	Enviada	Eventos Adversos Relacionados à	Falhas envolvendo o cateter venoso		
10/10/2022	CE	FORTALEZA	HGF	PACIENTE , DADO O SEU	Enviada	Eventos Adversos Relacionados à	Queda do paciente		
10/10/2022	CE	FORTALEZA	HGF	FLEBITE EM MSE	Enviada	Eventos Adversos Relacionados à	Falhas envolvendo o cateter venoso		
11/10/2022	CE	FORTALEZA	HGF	estava no banheiro sentado se	Enviada	Eventos Adversos Relacionados à	Queda do paciente		
11/10/2022	CE	FORTALEZA	HGF	-	Enviada	Eventos Adversos Relacionados à	Queda do paciente		

11/10/2022	CE	FORTALE ZA	HGF	Decanula çao acidental	Enviada	Eventos Adversos Relaciona dos à	Extubaça o endotraqu eal		
11/10/2022	CE	FORTALE ZA	HGF	Extravasa mento de QUIMIOT ERÁPICO	Enviada	Eventos Adversos Relaciona dos à	Falhas envolvend o cateter venoso		
11/10/2022	CE	FORTALE ZA	HGF	Extravasa mento a medicaçã o	Enviada	Eventos Adversos Relaciona dos à	Falhas envolvend o cateter venoso		
13/10/2022	CE	FORTALE ZA	HGF	PACIENT E COM ACOMPA NHANTE	Enviada	Eventos Adversos Relaciona dos à	Queda do paciente		
13/10/2022	CE	FORTALE ZA	HGF	PROFISS IONALO RESPON SÁVEL	Enviada	Eventos Adversos Relaciona dos à	Falhas durante a assistênci a à saúde		
13/10/2022	CE	FORTALE ZA	HGF	ACOMPA NHANTE LEVOU O	Enviada	Eventos Adversos Relaciona dos à	Queda do paciente		
13/10/2022	CE	FORTALE ZA	HGF	APRESE NTA HIPEREM IA E	Enviada	Eventos Adversos Relaciona dos à	Falhas envolvend o cateter venoso		
13/10/2022	CE	FORTALE ZA	HGF	Maca não cabe a paciente assim	Enviada	Eventos Adversos Relaciona dos à	Falhas durante a assistênci a à saúde		
13/10/2022	CE	FORTALE ZA	HGF	Caiu durante brincadeir a com	Enviada	Eventos Adversos Relaciona dos à	Queda do paciente		
13/10/2022	CE	FORTALE ZA	HGF	Flebite MSE	Enviada	Eventos Adversos Relaciona dos à	Falhas envolvend o cateter venoso		
14/10/2022	CE	FORTALE ZA	HGF	Paciente broncoasp irou após apresenta	Enviada	Eventos Adversos Relaciona dos à	Broncoas piração		
14/10/2022	CE	FORTALE ZA	HGF	Lesão por pressão estágio 2	Enviada	Eventos Adversos Relaciona dos à	Lesão por pressão		
14/10/2022	CE	FORTALE ZA	HGF	Flebite em MSE	Enviada	Eventos Adversos Relaciona dos à	Falhas envolvend o cateter venoso		
14/10/2022	CE	FORTALE ZA	HGF	EDEMA, HIPEREM IA E DOR	Enviada	Eventos Adversos Relaciona dos à	Falhas envolvend o cateter venoso		
14/10/2022	CE	FORTALE ZA	HGF	Paciente admitido no setor sem	Enviada	Eventos Adversos Relaciona dos à	Falhas durante a assistênci a à saúde		

14/10/2022	CE	FORTALE ZA	HGF	PACIENT E SEM ACOMPA NHANTE	Enviada	Eventos Adversos Relaciona dos à	Queda do paciente		
14/10/2022	CE	FORTALE ZA	HGF	Sitio de inserção de hipodemó	Enviada	Eventos Adversos Relaciona dos à	Falhas envolvend o cateter venoso		
14/10/2022	CE	FORTALE ZA	HGF	INFILTRA ÇÃO + HIPEREM IA	Enviada	Eventos Adversos Relaciona dos à	Falhas envolvend o cateter venoso		
14/10/2022	CE	FORTALE ZA	HGF	Lesão interglúte a	Enviada	Eventos Adversos Relaciona dos à	Lesão por pressão		
20/10/2022	CE	FORTALE ZA	HGF	EVENTO ADVERS O RELACIO	Enviada	Eventos Adversos Relaciona dos à	Falhas durante a assistênci a à saúde		
20/10/2022	CE	FORTALE ZA	HGF	Paciente estaria clinicame nte	Enviada	Eventos Adversos Relaciona dos à	Falhas durante a assistênci a à saúde		
20/10/2022	CE	FORTALE ZA	HGF	Paciente estava estável, quando	Enviada	Eventos Adversos Relaciona dos à	Falhas durante a assistênci a à saúde		
20/10/2022	CE	FORTALE ZA	HGF	Paciente apresento u parada cardíaca	Enviada	Eventos Adversos Relaciona dos à	Falhas durante a assistênci a à saúde		
25/10/2022	CE	FORTALE ZA	HGF	LESÃO EM REGIÃO SACRAL	Enviada	Eventos Adversos Relaciona dos à	Lesão por pressão		
25/10/2022	CE	FORTALE ZA	HGF	LESÃO EM REGIAO DE	Em retificação	Eventos Adversos Relaciona dos à	Lesão por pressão		
25/10/2022	CE	FORTALE ZA	HGF	LESÃO EM REGIÃO SACRAL	Enviada	Eventos Adversos Relaciona dos à	Lesão por pressão		
25/10/2022	CE	FORTALE ZA	HGF	LESÃO EM REGIÃO SACRAL	Enviada	Eventos Adversos Relaciona dos à	Lesão por pressão		
25/10/2022	CE	FORTALE ZA	HGF	LESÃO EM REGIÃO INTERGL	Concluída	Eventos Adversos Relaciona dos à	Lesão por pressão		
25/10/2022	CE	FORTALE ZA	HGF	LESÃO EM REGIÃO SACRAL	Enviada	Eventos Adversos Relaciona dos à	Lesão por pressão		
25/10/2022	CE	FORTALE ZA	HGF	LESÃO EM GLUTEO	Enviada	Eventos Adversos Relaciona dos à	Lesão por pressão		

25/10/2022	CE	FORTALE ZA	HGF	LESÃO EM GLUTEO	Enviada	Eventos Adversos Relaciona dos à	Lesão por pressão		
25/10/2022	CE	FORTALE ZA	HGF	LESÃO EM GLUTEO DIREITO	Enviada	Eventos Adversos Relaciona dos à	Lesão por pressão		
25/10/2022	CE	FORTALE ZA	HGF	LESÃO EM REGIÃO SACRAL	Enviada	Eventos Adversos Relaciona dos à	Lesão por pressão		
25/10/2022	CE	FORTALE ZA	HGF	LESÃO EM GLUTEO ESQUER	Enviada	Eventos Adversos Relaciona dos à	Lesão por pressão		
25/10/2022	CE	FORTALE ZA	HGF	LESÃO EM REGIÃO SACRAL	Enviada	Eventos Adversos Relaciona dos à	Lesão por pressão		
25/10/2022	CE	FORTALE ZA	HGF	LESÃO EM REGIÃO SACRAL	Enviada	Eventos Adversos Relaciona dos à	Lesão por pressão		
25/10/2022	CE	FORTALE ZA	HGF	LESÃO EM REGIÃO INTERGL	Enviada	Eventos Adversos Relaciona dos à	Lesão por pressão		
25/10/2022	CE	FORTALE ZA	HGF	LESÃO EM TROCAN TER	Enviada	Eventos Adversos Relaciona dos à	Lesão por pressão		
25/10/2022	CE	FORTALE ZA	HGF	LESÃO EM GLUTEO DIREITO	Concluída	Eventos Adversos Relaciona dos à	Lesão por pressão		
25/10/2022	CE	FORTALE ZA	HGF	LESÃO EM REGIÃO SACRAL	Enviada	Eventos Adversos Relaciona dos à	Lesão por pressão		
25/10/2022	CE	FORTALE ZA	HGF	LESÃO EM GLUTEO ESQUER	Enviada	Eventos Adversos Relaciona dos à	Lesão por pressão		
25/10/2022	CE	FORTALE ZA	HGF	LESÃO EM REGIÃO SACRAL	Enviada	Eventos Adversos Relaciona dos à	Lesão por pressão		
25/10/2022	CE	FORTALE ZA	HGF	LESÃO EM REGIÃO SACRAL	Enviada	Eventos Adversos Relaciona dos à	Lesão por pressão		
25/10/2022	CE	FORTALE ZA	HGF	LESÃO EM CALCAN EO D	Enviada	Eventos Adversos Relaciona dos à	Lesão por pressão		
25/10/2022	CE	FORTALE ZA	HGF	LESÃO EM CALCAN EO	Enviada	Eventos Adversos Relaciona dos à	Lesão por pressão		

25/10/2022	CE	FORTALEZA	HGF	LESÃO EM REGIÃO SACRAL	Enviada	Eventos Adversos Relacionados à	Lesão por pressão		
25/10/2022	CE	FORTALEZA	HGF	LESÃO EM REGIÃO SACRAL	Enviada	Eventos Adversos Relacionados à	Lesão por pressão		
25/10/2022	CE	FORTALEZA	HGF	LESÃO EM REGIÃO SACRAL	Enviada	Eventos Adversos Relacionados à	Lesão por pressão		
25/10/2022	CE	FORTALEZA	HGF	LESÃO EM REGIÃO SACRAL	Enviada	Eventos Adversos Relacionados à	Lesão por pressão		
25/10/2022	CE	FORTALEZA	HGF	LESÃO EM REGIÃO SACRAL	Enviada	Eventos Adversos Relacionados à	Lesão por pressão		
25/10/2022	CE	FORTALEZA	HGF	LESÃO EM REGIÃO SACRAL	Enviada	Eventos Adversos Relacionados à	Lesão por pressão		
25/10/2022	CE	FORTALEZA	HGF	LESÃO EM TROCANTER	Enviada	Eventos Adversos Relacionados à	Lesão por pressão		
25/10/2022	CE	FORTALEZA	HGF	LESÃO EM REGIÃO SACRAL	Concluída	Eventos Adversos Relacionados à	Lesão por pressão		
25/10/2022	CE	FORTALEZA	HGF	LESÃO EM ESCAPULA	Enviada	Eventos Adversos Relacionados à	Lesão por pressão		
25/10/2022	CE	FORTALEZA	HGF	LESÃO EM ESCAPULA	Enviada	Eventos Adversos Relacionados à	Lesão por pressão		
25/10/2022	CE	FORTALEZA	HGF	LESÃO EM TROCANTER	Enviada	Eventos Adversos Relacionados à	Lesão por pressão		
25/10/2022	CE	FORTALEZA	HGF	LESÃO EM CALCANEO	Enviada	Eventos Adversos Relacionados à	Lesão por pressão		
25/10/2022	CE	FORTALEZA	HGF	LESÃO EM REGIÃO SACRAL	Enviada	Eventos Adversos Relacionados à	Lesão por pressão		
25/10/2022	CE	FORTALEZA	HGF	LESÃO EM REGIÃO SACRAL	Enviada	Eventos Adversos Relacionados à	Lesão por pressão		
25/10/2022	CE	FORTALEZA	HGF	LESÃO EM REGIÃO SACRAL	Enviada	Eventos Adversos Relacionados à	Lesão por pressão		

25/10/2022	CE	FORTALEZA	HGF	LESÃO EM REGIÃO INTERGL	Enviada	Eventos Adversos Relacionados à	Lesão por pressão		
25/10/2022	CE	FORTALEZA	HGF	LESÃO EM REGIÃO SACRAL	Enviada	Eventos Adversos Relacionados à	Lesão por pressão		
25/10/2022	CE	FORTALEZA	HGF	LESÃO EM REGIÃO SACRAL	Enviada	Eventos Adversos Relacionados à	Lesão por pressão		
25/10/2022	CE	FORTALEZA	HGF	LESÃO EM REGIÃO SACRAL	Enviada	Eventos Adversos Relacionados à	Lesão por pressão		
25/10/2022	CE	FORTALEZA	HGF	LESÃO EM REGIÃO SACRAL	Enviada	Eventos Adversos Relacionados à	Lesão por pressão		
25/10/2022	CE	FORTALEZA	HGF	LESÃO EM REGIÃO SACRAL	Enviada	Eventos Adversos Relacionados à	Lesão por pressão		
25/10/2022	CE	FORTALEZA	HGF	LESÃO EM REGIÃO SACRAL	Enviada	Eventos Adversos Relacionados à	Lesão por pressão		
25/10/2022	CE	FORTALEZA	HGF	LESÃO EM REGIÃO INTERGL	Enviada	Eventos Adversos Relacionados à	Lesão por pressão		
25/10/2022	CE	FORTALEZA	HGF	LESÃO EM REGIÃO SACRAL	Enviada	Eventos Adversos Relacionados à	Lesão por pressão		
25/10/2022	CE	FORTALEZA	HGF	LESÃO EM REGIÃO INTERGL	Enviada	Eventos Adversos Relacionados à	Lesão por pressão		
25/10/2022	CE	FORTALEZA	HGF	LESÃO EM REGIÃO SACRAL	Enviada	Eventos Adversos Relacionados à	Lesão por pressão		
25/10/2022	CE	FORTALEZA	HGF	LESÃO EM REGIÃO SACRAL	Enviada	Eventos Adversos Relacionados à	Lesão por pressão		
25/10/2022	CE	FORTALEZA	HGF	LESÃO EM REGIÃO SACRAL	Enviada	Eventos Adversos Relacionados à	Lesão por pressão		
25/10/2022	CE	FORTALEZA	HGF	LESÃO EM REGIÃO SACRAL	Enviada	Eventos Adversos Relacionados à	Lesão por pressão		
25/10/2022	CE	FORTALEZA	HGF	LESÃO EM REGIÃO SACRAL	Enviada	Eventos Adversos Relacionados à	Lesão por pressão		

25/10/2022	CE	FORTALEZA	HGF	LESÃO EM TROCANTER	Enviada	Eventos Adversos Relacionados à	Lesão por pressão		
25/10/2022	CE	FORTALEZA	HGF	LESÃO EM TROCANTER	Enviada	Eventos Adversos Relacionados à	Lesão por pressão		
25/10/2022	CE	FORTALEZA	HGF	LESÃO EM REGIÃO SACRAL	Enviada	Eventos Adversos Relacionados à	Lesão por pressão		
25/10/2022	CE	FORTALEZA	HGF	LESÃO EM REGIÃO SACRAL	Enviada	Eventos Adversos Relacionados à	Lesão por pressão		
25/10/2022	CE	FORTALEZA	HGF	LESÃO EM REGIÃO SACRAL	Enviada	Eventos Adversos Relacionados à	Lesão por pressão		
25/10/2022	CE	FORTALEZA	HGF	LESÃO EM CALCANEO	Concluída	Eventos Adversos Relacionados à	Lesão por pressão		
25/10/2022	CE	FORTALEZA	HGF	LESÃO EM MALEOLO	Enviada	Eventos Adversos Relacionados à	Lesão por pressão		
25/10/2022	CE	FORTALEZA	HGF	LESÃO EM REGIÃO SACRAL	Enviada	Eventos Adversos Relacionados à	Lesão por pressão		
25/10/2022	CE	FORTALEZA	HGF	LESÃO EM REGIÃO SACRAL	Enviada	Eventos Adversos Relacionados à	Lesão por pressão		
25/10/2022	CE	FORTALEZA	HGF	LESÃO EM REGIÃO SACRAL	Enviada	Eventos Adversos Relacionados à	Lesão por pressão		
25/10/2022	CE	FORTALEZA	HGF	LESÃO EM REGIÃO SACRAL	Enviada	Eventos Adversos Relacionados à	Lesão por pressão		
25/10/2022	CE	FORTALEZA	HGF	LESÃO EM REGIÃO SACRAL	Enviada	Eventos Adversos Relacionados à	Lesão por pressão		
25/10/2022	CE	FORTALEZA	HGF	LESÃO EM REGIAO SACRAL	Enviada	Eventos Adversos Relacionados à	Lesão por pressão		
25/10/2022	CE	FORTALEZA	HGF	LESÃO EM REGIAO ESCAPUL	Concluída	Eventos Adversos Relacionados à	Lesão por pressão		
25/10/2022	CE	FORTALEZA	HGF	LESÃO EM REGIÃO ESCAPUL	Enviada	Eventos Adversos Relacionados à	Lesão por pressão		

25/10/2022	CE	FORTALEZA	HGF	LESÃO EM REGIÃO SACRAL	Enviada	Eventos Adversos Relacionados à	Lesão por pressão		
25/10/2022	CE	FORTALEZA	HGF	LESÃO EM REGIÃO SACRAL	Enviada	Eventos Adversos Relacionados à	Lesão por pressão		
25/10/2022	CE	FORTALEZA	HGF	LESÃO EM REGIÃO SACRAL	Enviada	Eventos Adversos Relacionados à	Lesão por pressão		
25/10/2022	CE	FORTALEZA	HGF	LESÃO EM REGIAO SACRAL	Enviada	Eventos Adversos Relacionados à	Lesão por pressão		
25/10/2022	CE	FORTALEZA	HGF	LESÃO EM REGIÃO SACRAL	Enviada	Eventos Adversos Relacionados à	Lesão por pressão		
25/10/2022	CE	FORTALEZA	HGF	LESÃO EM REGIÃO SACRAL	Enviada	Eventos Adversos Relacionados à	Lesão por pressão		
25/10/2022	CE	FORTALEZA	HGF	LESÃO EM REGIÃO SACRAL	Enviada	Eventos Adversos Relacionados à	Lesão por pressão		
25/10/2022	CE	FORTALEZA	HGF	LESÃO EM REGIÃO SACRAL	Enviada	Eventos Adversos Relacionados à	Lesão por pressão		
26/10/2022	CE	FORTALEZA	HGF	LESÃO EM TROCANTER	Enviada	Eventos Adversos Relacionados à	Lesão por pressão		
26/10/2022	CE	FORTALEZA	HGF	LESÃO EM REGIÃO SACRAL	Enviada	Eventos Adversos Relacionados à	Lesão por pressão		
26/10/2022	CE	FORTALEZA	HGF	LESÃO EM REGIÃO SACRAL	Enviada	Eventos Adversos Relacionados à	Lesão por pressão		
26/10/2022	CE	FORTALEZA	HGF	LESÃO EM REGIÃO SACRAL	Enviada	Eventos Adversos Relacionados à	Lesão por pressão		
26/10/2022	CE	FORTALEZA	HGF	LESÃO EM TROCANTER	Enviada	Eventos Adversos Relacionados à	Lesão por pressão		
26/10/2022	CE	FORTALEZA	HGF	LESÃO EM TROCANTER	Enviada	Eventos Adversos Relacionados à	Lesão por pressão		
26/10/2022	CE	FORTALEZA	HGF	LESÃO EM REGIÃO SACRAL	Enviada	Eventos Adversos Relacionados à	Lesão por pressão		

26/10/2022	CE	FORTALEZA	HGF	LESÃO EM REGIÃO SACRAL	Enviada	Eventos Adversos Relacionados à	Lesão por pressão		
26/10/2022	CE	FORTALEZA	HGF	LESÃO EM REGIÃO SACRAL	Enviada	Eventos Adversos Relacionados à	Lesão por pressão		
26/10/2022	CE	FORTALEZA	HGF	LESÃO EM GLUTEO ESQUER	Enviada	Eventos Adversos Relacionados à	Lesão por pressão		
26/10/2022	CE	FORTALEZA	HGF	LESÃO EM REGIÃO SACRAL	Enviada	Eventos Adversos Relacionados à	Lesão por pressão		
26/10/2022	CE	FORTALEZA	HGF	LESÃO EM REGIÃO INTERGL	Enviada	Eventos Adversos Relacionados à	Lesão por pressão		
26/10/2022	CE	FORTALEZA	HGF	-	Enviada	Eventos Adversos Relacionados à	Lesão por pressão		
26/10/2022	CE	FORTALEZA	HGF	LESÃO EM GLUTEO ESQUER	Enviada	Eventos Adversos Relacionados à	Lesão por pressão		
26/10/2022	CE	FORTALEZA	HGF	LESÃO EM REGIÃO INTERGL	Enviada	Eventos Adversos Relacionados à	Lesão por pressão		
26/10/2022	CE	FORTALEZA	HGF	LESÃO EM REGIÃO SACRAL	Enviada	Eventos Adversos Relacionados à	Lesão por pressão		
26/10/2022	CE	FORTALEZA	HGF	LESÃO EM REGIÃO SACRAL	Enviada	Eventos Adversos Relacionados à	Lesão por pressão		
26/10/2022	CE	FORTALEZA	HGF	LESÃO EM REGIÃO SACRAL	Enviada	Eventos Adversos Relacionados à	Lesão por pressão		
26/10/2022	CE	FORTALEZA	HGF	LESÃO EM REGIÃO SACRAL	Enviada	Eventos Adversos Relacionados à	Lesão por pressão		
26/10/2022	CE	FORTALEZA	HGF	LESÃO EM REGIÃO SACRAL	Enviada	Eventos Adversos Relacionados à	Lesão por pressão		
26/10/2022	CE	FORTALEZA	HGF	LESÃO EM MALEOLO	Enviada	Eventos Adversos Relacionados à	Lesão por pressão		
26/10/2022	CE	FORTALEZA	HGF	LESÃO EM REGIÃO SACRAL	Enviada	Eventos Adversos Relacionados à	Lesão por pressão		

26/10/2022	CE	FORTALEZA	HGF	LESÃO EM GLUTEO ESQUER	Enviada	Eventos Adversos Relacionados à	Lesão por pressão		
26/10/2022	CE	FORTALEZA	HGF	LESÃO EM REGIAO SACRAL	Enviada	Eventos Adversos Relacionados à	Lesão por pressão		
26/10/2022	CE	FORTALEZA	HGF	LESÃO EM REGIÃO SACRAL	Enviada	Eventos Adversos Relacionados à	Lesão por pressão		
26/10/2022	CE	FORTALEZA	HGF	LESÃO EM GLUTEO ESQUER	Enviada	Eventos Adversos Relacionados à	Lesão por pressão		
26/10/2022	CE	FORTALEZA	HGF	LESÃO EM REGIÃO SACRAL	Enviada	Eventos Adversos Relacionados à	Lesão por pressão		
26/10/2022	CE	FORTALEZA	HGF	LESÃO EM REGIÃO SACRAL	Enviada	Eventos Adversos Relacionados à	Lesão por pressão		
26/10/2022	CE	FORTALEZA	HGF	LESÃO EM REGIAO SACRAL	Enviada	Eventos Adversos Relacionados à	Lesão por pressão		
26/10/2022	CE	FORTALEZA	HGF	LESÃO EM GLUTEO DIREITO	Enviada	Eventos Adversos Relacionados à	Lesão por pressão		
26/10/2022	CE	FORTALEZA	HGF	LESÃO EM GLUTEO DIREITO	Enviada	Eventos Adversos Relacionados à	Lesão por pressão		
26/10/2022	CE	FORTALEZA	HGF	LESÃO EM MALEOLO	Enviada	Eventos Adversos Relacionados à	Lesão por pressão		
26/10/2022	CE	FORTALEZA	HGF	LESÃO EM GLUTEO DIREITO	Enviada	Eventos Adversos Relacionados à	Lesão por pressão		
26/10/2022	CE	FORTALEZA	HGF	LESÃO EM REGIÃO SACRAL	Enviada	Eventos Adversos Relacionados à	Lesão por pressão		
26/10/2022	CE	FORTALEZA	HGF	LESÃO EM REGIÃO SACRAL	Enviada	Eventos Adversos Relacionados à	Lesão por pressão		
26/10/2022	CE	FORTALEZA	HGF	LESÃO EM REGIÃO SACRAL	Enviada	Eventos Adversos Relacionados à	Lesão por pressão		
26/10/2022	CE	FORTALEZA	HGF	LESÃO EM REGIÃO SACRAL	Enviada	Eventos Adversos Relacionados à	Lesão por pressão		

26/10/2022	CE	FORTALE ZA	HGF	LESAO EM REGIAO SACRAL	Enviada	Eventos Adversos Relaciona dos à	Lesão por pressão		
26/10/2022	CE	FORTALE ZA	HGF	LESÃO EM REGIÃO SACRAL	Concluída	Eventos Adversos Relaciona dos à	Lesão por pressão		
26/10/2022	CE	FORTALE ZA	HGF	LESÃO EM REGIÃO SACRAL	Enviada	Eventos Adversos Relaciona dos à	Lesão por pressão		
26/10/2022	CE	FORTALE ZA	HGF	LESÃO EM REGIÃO SACRAL	Enviada	Eventos Adversos Relaciona dos à	Lesão por pressão		
26/10/2022	CE	FORTALE ZA	HGF	LESÃO EM REGIÃO SACRAL	Enviada	Eventos Adversos Relaciona dos à	Lesão por pressão		
26/10/2022	CE	FORTALE ZA	HGF	LESÃO EM REGIÃO SACRAL	Em retificação	Eventos Adversos Relaciona dos à	Lesão por pressão		
28/10/2022	CE	FORTALE ZA	HGF	LESÃO EM REGIÃO SACRAL	Concluída	Eventos Adversos Relaciona dos à	Lesão por pressão		
28/10/2022	CE	FORTALE ZA	HGF	LESÃO EM REGIÃO SACRAL	Enviada	Eventos Adversos Relaciona dos à	Lesão por pressão		
28/10/2022	CE	FORTALE ZA	HGF	LESÃO EM CALCAN EO E	Enviada	Eventos Adversos Relaciona dos à	Lesão por pressão		
28/10/2022	CE	FORTALE ZA	HGF	LESÃO EM REGIÃO SACRAL	Enviada	Eventos Adversos Relaciona dos à	Lesão por pressão		
28/10/2022	CE	FORTALE ZA	HGF	LESÃO EM REGIÃO SACRAL	Enviada	Eventos Adversos Relaciona dos à	Lesão por pressão		
28/10/2022	CE	FORTALE ZA	HGF	LESÃO EM REGIÃO SACRAL	Enviada	Eventos Adversos Relaciona dos à	Lesão por pressão		
28/10/2022	CE	FORTALE ZA	HGF	LESÃO EM REGIÃO SACRAL	Enviada	Eventos Adversos Relaciona dos à	Lesão por pressão		
28/10/2022	CE	FORTALE ZA	HGF	LESÃO EM REGIÃO SACRAL	Concluída	Eventos Adversos Relaciona dos à	Lesão por pressão		
28/10/2022	CE	FORTALE ZA	HGF	LESÃO EM REGIÃO SACRAL	Enviada	Eventos Adversos Relaciona dos à	Lesão por pressão		

28/10/2022	CE	FORTALEZA	HGF	LESÃO EM REGIÃO SACRAL	Enviada	Eventos Adversos Relacionados à	Lesão por pressão		
28/10/2022	CE	FORTALEZA	HGF	LESÃO EM REGIÃO INTERGL	Enviada	Eventos Adversos Relacionados à	Lesão por pressão		
28/10/2022	CE	FORTALEZA	HGF	LESÃO EM REGIÃO SACRAL	Enviada	Eventos Adversos Relacionados à	Lesão por pressão		
28/10/2022	CE	FORTALEZA	HGF	LESÃO EM GLUTEO DIREITO	Enviada	Eventos Adversos Relacionados à	Lesão por pressão		
28/10/2022	CE	FORTALEZA	HGF	LESÃO EM REGIÃO SACRAL	Enviada	Eventos Adversos Relacionados à	Lesão por pressão		
09/11/2022	CE	FORTALEZA	HGF	LESÃO EM GLUTEO ESQUER	Enviada	Eventos Adversos Relacionados à	Lesão por pressão		
09/11/2022	CE	FORTALEZA	HGF	LESÃO EM SACRA	Concluída	Eventos Adversos Relacionados à	Lesão por pressão		
09/11/2022	CE	FORTALEZA	HGF	LESÃO EM SACRA	Enviada	Eventos Adversos Relacionados à	Lesão por pressão		
09/11/2022	CE	FORTALEZA	HGF	LESÃO EM SACRA	Enviada	Eventos Adversos Relacionados à	Lesão por pressão		
09/11/2022	CE	FORTALEZA	HGF	LESÃO EM SACRA	Enviada	Eventos Adversos Relacionados à	Lesão por pressão		
09/11/2022	CE	FORTALEZA	HGF	LESÃO EM SACRA	Enviada	Eventos Adversos Relacionados à	Lesão por pressão		
09/11/2022	CE	FORTALEZA	HGF	LESÃO EM SACRA	Enviada	Eventos Adversos Relacionados à	Lesão por pressão		
09/11/2022	CE	FORTALEZA	HGF	LESÃO EM SACRA	Enviada	Eventos Adversos Relacionados à	Lesão por pressão		
09/11/2022	CE	FORTALEZA	HGF	LESAO EM SACRA	Enviada	Eventos Adversos Relacionados à	Lesão por pressão		
09/11/2022	CE	FORTALEZA	HGF	LESÃO EM GLUTEO D	Enviada	Eventos Adversos Relacionados à	Lesão por pressão		

09/11/2022	CE	FORTALEZA	HGF	LESÃO EM SACRA	Enviada	Eventos Adversos Relacionados à	Lesão por pressão		
09/11/2022	CE	FORTALEZA	HGF	LESAO EM TROCANTER	Enviada	Eventos Adversos Relacionados à	Lesão por pressão		
09/11/2022	CE	FORTALEZA	HGF	LESÃO EM SACRA	Enviada	Eventos Adversos Relacionados à	Lesão por pressão		
09/11/2022	CE	FORTALEZA	HGF	LESÃO EM SACRA	Enviada	Eventos Adversos Relacionados à	Lesão por pressão		
09/11/2022	CE	FORTALEZA	HGF	LESÃO EM SACRA	Enviada	Eventos Adversos Relacionados à	Lesão por pressão		
10/11/2022	CE	FORTALEZA	HGF	LESAO EM SACRA	Enviada	Eventos Adversos Relacionados à	Lesão por pressão		
10/11/2022	CE	FORTALEZA	HGF	LESÃO EM SACRA	Enviada	Eventos Adversos Relacionados à	Lesão por pressão		
10/11/2022	CE	FORTALEZA	HGF	LESÃO EM SACRA	Enviada	Eventos Adversos Relacionados à	Lesão por pressão		
10/11/2022	CE	FORTALEZA	HGF	LESÃO EM SACRA	Enviada	Eventos Adversos Relacionados à	Lesão por pressão		
10/11/2022	CE	FORTALEZA	HGF	LESÃO EM SACRA	Enviada	Eventos Adversos Relacionados à	Lesão por pressão		
10/11/2022	CE	FORTALEZA	HGF	LESÃO EM SACRA	Enviada	Eventos Adversos Relacionados à	Lesão por pressão		
10/11/2022	CE	FORTALEZA	HGF	LESAO EM GLUTEO DIREITO	Enviada	Eventos Adversos Relacionados à	Lesão por pressão		
10/11/2022	CE	FORTALEZA	HGF	LESAO EM GLUTEO ESQUER	Enviada	Eventos Adversos Relacionados à	Lesão por pressão		
10/11/2022	CE	FORTALEZA	HGF	LESAO EM GLUTEO ESQUER	Enviada	Eventos Adversos Relacionados à	Lesão por pressão		
10/11/2022	CE	FORTALEZA	HGF	LESAO EM GLUTEO ESQUER	Enviada	Eventos Adversos Relacionados à	Lesão por pressão		

10/11/2022	CE	FORTALEZA	HGF	LESÃO OCCIPTAL	Enviada	Eventos Adversos Relacionados à	Lesão por pressão		
10/11/2022	CE	FORTALEZA	HGF	LESAO EM SACRA	Enviada	Eventos Adversos Relacionados à	Lesão por pressão		
10/11/2022	CE	FORTALEZA	HGF	LESAO EM REGIÃO INTERGI	Enviada	Eventos Adversos Relacionados à	Lesão por pressão		
10/11/2022	CE	FORTALEZA	HGF	LESAO EM CALCANEO	Enviada	Eventos Adversos Relacionados à	Lesão por pressão		
10/11/2022	CE	FORTALEZA	HGF	LESÃO EM SACRA	Enviada	Eventos Adversos Relacionados à	Lesão por pressão		
01/12/2022	CE	FORTALEZA	HGF	Lesao inflamatória no paciente	Enviada	Eventos Adversos Relacionados à	Falhas envolvendo cateter venoso		
05/12/2022	CE	FORTALEZA	HGF	Paciente apresentou flebite em	Enviada	Eventos Adversos Relacionados à	Falhas envolvendo cateter venoso		
05/12/2022	CE	FORTALEZA	HGF	Paciente apresentou estravasa	Enviada	Eventos Adversos Relacionados à	Falhas envolvendo cateter venoso		
05/12/2022	CE	FORTALEZA	HGF	Paciente apresenta desorienta	Enviada	Eventos Adversos Relacionados à	Queda do paciente		
05/12/2022	CE	FORTALEZA	HGF	PACIENTE ALGO ORIENTADO, SEM	Enviada	Eventos Adversos Relacionados à	Queda do paciente		
05/12/2022	CE	FORTALEZA	HGF	Paciente arrancou sonda nasogástri	Enviada	Eventos Adversos Relacionados à	Falhas envolvendo sondas		
05/12/2022	CE	FORTALEZA	HGF	procedimento de raio x não foi	Enviada	Eventos Adversos Relacionados à	Falhas durante a assistência à saúde		
06/12/2022	CE	FORTALEZA	HGF	AS 13:20H DURANTE A	Enviada	Eventos Adversos Relacionados à	Falhas envolvendo cateter venoso		
06/12/2022	CE	FORTALEZA	HGF	OBSTRUÇÃO DE SNE, TENTAD	Enviada	Eventos Adversos Relacionados à	Falhas envolvendo sondas		
06/12/2022	CE	FORTALEZA	HGF	PACIENTE DESORIENTADA	Enviada	Eventos Adversos Relacionados à	Falhas envolvendo sondas		

06/12/2022	CE	FORTALEZA	HGF	Paciente caiu de joelhos quando	Enviada	Eventos Adversos Relacionados à	Queda do paciente		
06/12/2022	CE	FORTALEZA	HGF	Paciente retirou Sonda nasoenter	Enviada	Eventos Adversos Relacionados à	Falhas envolvendo sondas		
06/12/2022	CE	FORTALEZA	HGF	Paciente com Sonda nasogastri	Enviada	Eventos Adversos Relacionados à	Falhas envolvendo sondas		
06/12/2022	CE	FORTALEZA	HGF	Paciente puxou a sonda nasoenter	Enviada	Eventos Adversos Relacionados à	Falhas envolvendo sondas		
06/12/2022	CE	FORTALEZA	HGF	Fixação soltou e sonda deslocou	Enviada	Eventos Adversos Relacionados à	Falhas envolvendo sondas		
06/12/2022	CE	FORTALEZA	HGF	Paciente com sonda nasoenter	Enviada	Eventos Adversos Relacionados à	Falhas envolvendo sondas		
06/12/2022	CE	FORTALEZA	HGF	Flebite em membro superior	Enviada	Eventos Adversos Relacionados à	Falhas envolvendo cateter venoso		
06/12/2022	CE	FORTALEZA	HGF	PACIENTE RETIROU SONDA	Enviada	Eventos Adversos Relacionados à	Falhas envolvendo sondas		
06/12/2022	CE	FORTALEZA	HGF	QUEDA DA PRÓPRIA ALTURA	Enviada	Eventos Adversos Relacionados à	Queda do paciente		
06/12/2022	CE	FORTALEZA	HGF	PACIENTE RETIROU SONDA	Enviada	Eventos Adversos Relacionados à	Falhas envolvendo sondas		
06/12/2022	CE	FORTALEZA	HGF	PRESENÇA DE FLICTENA EM	Enviada	Eventos Adversos Relacionados à	Lesão por pressão		
06/12/2022	CE	FORTALEZA	HGF	HIPEREMIA EM CALCANEO	Enviada	Eventos Adversos Relacionados à	Lesão por pressão		
06/12/2022	CE	FORTALEZA	HGF	MEMBRO SUPERIOR	Enviada	Eventos Adversos Relacionados à	Falhas envolvendo cateter venoso		
06/12/2022	CE	FORTALEZA	HGF	OBSTRUÇÃO DE SONDA NASOEN	Enviada	Eventos Adversos Relacionados à	Falhas envolvendo sondas		
06/12/2022	CE	FORTALEZA	HGF	OBSTRUÇÃO DE SONDA NASOEN	Enviada	Eventos Adversos Relacionados à	Falhas envolvendo sondas		

06/12/2022	CE	FORTALEZA	HGF	HOUVE OBSTRUÇÃO DE SONDA	Enviada	Eventos Adversos Relacionados à	Falhas envolvendo sondas		
06/12/2022	CE	FORTALEZA	HGF	PERDA DE SONDA POR	Enviada	Eventos Adversos Relacionados à	Falhas envolvendo sondas		
06/12/2022	CE	FORTALEZA	HGF	Paciente com dor, hiperemia e rubor	Enviada	Eventos Adversos Relacionados à	Falhas envolvendo cateter venoso		
06/12/2022	CE	FORTALEZA	HGF	Paciente ao levantar-se ficou	Enviada	Eventos Adversos Relacionados à	Queda do paciente		
06/12/2022	CE	FORTALEZA	HGF	Flebite em acesso venoso	Enviada	Eventos Adversos Relacionados à	Falhas envolvendo cateter venoso		
06/12/2022	CE	FORTALEZA	HGF	PACIENTE APRESENTOU	Enviada	Eventos Adversos Relacionados à	Falhas envolvendo cateter venoso		
06/12/2022	CE	FORTALEZA	HGF	Paciente agitado, retirou a sonda	Enviada	Eventos Adversos Relacionados à	Falhas envolvendo sondas		
06/12/2022	CE	FORTALEZA	HGF	PACIENTE AGITADO	Enviada	Eventos Adversos Relacionados à	Falhas envolvendo sondas		
06/12/2022	CE	FORTALEZA	HGF	PACIENTE RETIROU SONDA	Enviada	Eventos Adversos Relacionados à	Falhas envolvendo sondas		
06/12/2022	CE	FORTALEZA	HGF	PACIENTE RETIROU SONDA	Enviada	Eventos Adversos Relacionados à	Falhas envolvendo sondas		
06/12/2022	CE	FORTALEZA	HGF	Flebite em membro superior	Enviada	Eventos Adversos Relacionados à	Falhas envolvendo cateter venoso		
06/12/2022	CE	FORTALEZA	HGF	Paciente com edema, dor e	Enviada	Eventos Adversos Relacionados à	Falhas envolvendo cateter venoso		
06/12/2022	CE	FORTALEZA	HGF	Broncoaspiraçao após episódio	Enviada	Eventos Adversos Relacionados à	Broncoaspiração		
06/12/2022	CE	FORTALEZA	HGF	Hipertensa,calor e dor	Enviada	Eventos Adversos Relacionados à	Falhas envolvendo cateter venoso		
06/12/2022	CE	FORTALEZA	HGF	PACIENTE RETIROU SONDA	Enviada	Eventos Adversos Relacionados à	Falhas envolvendo sondas		

06/12/2022	CE	FORTALE ZA	HGF	PACIENT E APRESE NTOU	Enviada	Eventos Adversos Relaciona dos à	Falhas envolvend o cateter venoso		
06/12/2022	CE	FORTALE ZA	HGF	Sonda nasoenter al exteriorizo	Enviada	Eventos Adversos Relaciona dos à	Falhas envolvend o sondas		
06/12/2022	CE	FORTALE ZA	HGF	Paciente caiu da própria altura	Enviada	Eventos Adversos Relaciona dos à	Queda do paciente		
06/12/2022	CE	FORTALE ZA	HGF	Quimioter apia veio com o invólucro	Enviada	Eventos Adversos Relaciona dos à	Falha na identificaç ão do paciente		
06/12/2022	CE	FORTALE ZA	HGF	Flebite em AVP/MID com	Enviada	Eventos Adversos Relaciona dos à	Falhas envolvend o cateter venoso		
06/12/2022	CE	FORTALE ZA	HGF	AVP/MSD apresenta ndo	Enviada	Eventos Adversos Relaciona dos à	Falhas envolvend o cateter venoso		
06/12/2022	CE	FORTALE ZA	HGF	AVP/MSD (03/10), apresenta ndo	Enviada	Eventos Adversos Relaciona dos à	Falhas envolvend o cateter venoso		
07/12/2022	CE	FORTALE ZA	HGF	paciente caiu ao se dirigir ao	Enviada	Eventos Adversos Relaciona dos à	Queda do paciente		
07/12/2022	CE	FORTALE ZA	HGF	PACIENT E AO CAMINHA R PARA	Enviada	Eventos Adversos Relaciona dos à	Queda do paciente		
07/12/2022	CE	FORTALE ZA	HGF	DURANT E O EXAME FÍSICO	Enviada	Eventos Adversos Relaciona dos à	Lesão por pressão		
07/12/2022	CE	FORTALE ZA	HGF	Paciente sofreu queda do leito ao	Enviada	Eventos Adversos Relaciona dos à	Queda do paciente		
07/12/2022	CE	FORTALE ZA	HGF	Foi escutado um grito ao	Enviada	Eventos Adversos Relaciona dos à	Queda do paciente		
07/12/2022	CE	FORTALE ZA	HGF	Paciente foi sem acompanh ante para	Enviada	Eventos Adversos Relaciona dos à	Queda do paciente		
07/12/2022	CE	FORTALE ZA	HGF	Paciente admitida com LPP sacral	Enviada	Eventos Adversos Relaciona dos à	Lesão por pressão		
07/12/2022	CE	FORTALE ZA	HGF	Paciente foi ao banheiro levantou	Enviada	Eventos Adversos Relaciona dos à	Queda do paciente		

08/12/2022	CE	FORTALEZA	HGF	Pacientes conciente, a tentativa	Enviada	Eventos Adversos Relacionados à	Queda do paciente		
08/12/2022	CE	FORTALEZA	HGF	PACIENTE ACAMADO POR	Enviada	Eventos Adversos Relacionados à	Lesão por pressão		
12/12/2022	CE	FORTALEZA	HGF	Perda de sonda nasoenteral	Enviada	Eventos Adversos Relacionados à	Falhas envolvendo sondas		
12/12/2022	CE	FORTALEZA	HGF	PACIENTE APRESENTOU	Enviada	Eventos Adversos Relacionados à	Queda do paciente		
12/12/2022	CE	FORTALEZA	HGF	Membro superior direito com	Enviada	Eventos Adversos Relacionados à	Falhas envolvendo cateter venoso		
12/12/2022	CE	FORTALEZA	HGF	Paciente apresentando flebite	Enviada	Eventos Adversos Relacionados à	Falhas envolvendo cateter venoso		
12/12/2022	CE	FORTALEZA	HGF	LESÃO POR PRESSÃO	Enviada	Eventos Adversos Relacionados à	Lesão por pressão		
12/12/2022	CE	FORTALEZA	HGF	Obstruído Sondanasoenteral quando	Enviada	Eventos Adversos Relacionados à	Falhas envolvendo sondas		
12/12/2022	CE	FORTALEZA	HGF	Paciente apresentou exterioriza	Enviada	Eventos Adversos Relacionados à	Falhas envolvendo sondas		
12/12/2022	CE	FORTALEZA	HGF	sondanasoenteral deslocada	Enviada	Eventos Adversos Relacionados à	Falhas envolvendo sondas		
12/12/2022	CE	FORTALEZA	HGF	Paciente puxou Sondanasoenteral	Enviada	Eventos Adversos Relacionados à	Falhas envolvendo sondas		
13/12/2022	CE	FORTALEZA	HGF	LPP ESTÁGIO 1 (LEVE HIPEREM	Enviada	Eventos Adversos Relacionados à	Lesão por pressão		
13/12/2022	CE	FORTALEZA	HGF	Paciente no banheiro com sua	Enviada	Eventos Adversos Relacionados à	Queda do paciente		
20/12/2022	CE	FORTALEZA	HGF	Flebite em membro superior	Enviada	Eventos Adversos Relacionados à	Falhas envolvendo cateter venoso		
20/12/2022	CE	FORTALEZA	HGF	Flebite em membro superior	Enviada	Eventos Adversos Relacionados à	Falhas envolvendo cateter venoso		

20/12/2022	CE	FORTALEZA	HGF	perda de sonda nasoenteral	Enviada	Eventos Adversos Relacionados à	Falhas envolvendo sondas		
20/12/2022	CE	FORTALEZA	HGF	perda de sonda nasoenteral	Enviada	Eventos Adversos Relacionados à	Falhas envolvendo sondas		
20/12/2022	CE	FORTALEZA	HGF	perda de sonda nasoenteral	Enviada	Eventos Adversos Relacionados à	Falhas envolvendo sondas		
20/12/2022	CE	FORTALEZA	HGF	Flebite Membro superior direito	Enviada	Eventos Adversos Relacionados à	Falhas envolvendo cateter venoso		
20/12/2022	CE	FORTALEZA	HGF	Flebite MSD, apresentando	Enviada	Eventos Adversos Relacionados à	Falhas envolvendo cateter venoso		
20/12/2022	CE	FORTALEZA	HGF	LESÃO POR PRESSÃO	Enviada	Eventos Adversos Relacionados à	Lesão por pressão		
20/12/2022	CE	FORTALEZA	HGF	PACIENTE APRESENTANDO	Enviada	Eventos Adversos Relacionados à	Lesão por pressão		
20/12/2022	CE	FORTALEZA	HGF	PACIENTE COM PRESENCA DE	Enviada	Eventos Adversos Relacionados à	Falhas envolvendo cateter venoso		
20/12/2022	CE	FORTALEZA	HGF	Flebite em msd data do acesso	Enviada	Eventos Adversos Relacionados à	Falhas envolvendo cateter venoso		
20/12/2022	CE	FORTALEZA	HGF	PACIENTE INQUIETA	Enviada	Eventos Adversos Relacionados à	Lesão por pressão		
20/12/2022	CE	FORTALEZA	HGF	Paciente agitada, retirou sonda	Enviada	Eventos Adversos Relacionados à	Falhas envolvendo sondas		
20/12/2022	CE	FORTALEZA	HGF	Paciente puxou a SNG por conta	Enviada	Eventos Adversos Relacionados à	Falhas envolvendo sondas		
20/12/2022	CE	FORTALEZA	HGF	Paciente deu entrada na	Enviada	Eventos Adversos Relacionados à	Lesão por pressão		
20/12/2022	CE	FORTALEZA	HGF	PERDA DE AVP, APRESENTANDO	Enviada	Eventos Adversos Relacionados à	Falhas envolvendo cateter venoso		
20/12/2022	CE	FORTALEZA	HGF	Paciente apresentando flebite	Enviada	Eventos Adversos Relacionados à	Falhas envolvendo cateter venoso		

20/12/2022	CE	FORTALEZA	HGF	Paciente retirou SNE	Enviada	Eventos Adversos Relacionados à	Falhas envolvendo sondas		
21/12/2022	CE	FORTALEZA	HGF	Paciente apresenta hiperemia	Enviada	Eventos Adversos Relacionados à	Falhas envolvendo cateter venoso		
21/12/2022	CE	FORTALEZA	HGF	OBSTRUÇÃO DE SNE	Enviada	Eventos Adversos Relacionados à	Falhas envolvendo sondas		
21/12/2022	CE	FORTALEZA	HGF	Paciente retirou sne	Enviada	Eventos Adversos Relacionados à	Falhas envolvendo sondas		
23/12/2022	CE	FORTALEZA	HGF	PACIENTE COM MSE, MAIS	Enviada	Eventos Adversos Relacionados à	Falhas envolvendo cateter venoso		
23/12/2022	CE	FORTALEZA	HGF	Durante o banho do paciente foi	Enviada	Eventos Adversos Relacionados à	Lesão por pressão		
23/12/2022	CE	FORTALEZA	HGF	LESÃO POR PRESSÃO	Enviada	Eventos Adversos Relacionados à	Lesão por pressão		
23/12/2022	CE	FORTALEZA	HGF	LESÃO POR PRESSÃO	Enviada	Eventos Adversos Relacionados à	Lesão por pressão		
23/12/2022	CE	FORTALEZA	HGF	Paciente retirou sne.	Enviada	Eventos Adversos Relacionados à	Falhas envolvendo sondas		
23/12/2022	CE	FORTALEZA	HGF	PACT,CAIU NO BANHEIRO NAO	Enviada	Eventos Adversos Relacionados à	Queda do paciente		
23/12/2022	CE	FORTALEZA	HGF	ACOMPANHANTE RELATA QUE	Enviada	Eventos Adversos Relacionados à	Queda do paciente		
26/12/2022	CE	FORTALEZA	HGF	Paciente foi ao banheiro com o	Enviada	Eventos Adversos Relacionados à	Queda do paciente		
26/12/2022	CE	FORTALEZA	HGF	PACIENTE TEVE FLEBITE NO	Enviada	Eventos Adversos Relacionados à	Falhas envolvendo cateter venoso		
26/12/2022	CE	FORTALEZA	HGF	Paciente sofre queda da própria	Enviada	Eventos Adversos Relacionados à	Queda do paciente		
26/12/2022	CE	FORTALEZA	HGF	paciente apresentou uma sincope e	Enviada	Eventos Adversos Relacionados à	Queda do paciente		

29/12/2022	CE	FORTALEZA	HGF	Administração do medicamento	Concluída	Eventos Adversos Relacionados à	Falhas durante a assistência à saúde		
02/01/2023	CE	FORTALEZA	HGF	PACIENTE REFERIU DOR NO	Enviada	Eventos Adversos Relacionados à	Falhas envolvendo cateter venoso		
02/01/2023	CE	FORTALEZA	HGF	LESÃO POR PRESSÃO EM	Enviada	Eventos Adversos Relacionados à	Lesão por pressão		
02/01/2023	CE	FORTALEZA	HGF	LESÃO POR PRESSÃO	Enviada	Eventos Adversos Relacionados à	Lesão por pressão		
02/01/2023	CE	FORTALEZA	HGF	Após término de infusão	Enviada	Eventos Adversos Relacionados à	Falhas envolvendo cateter venoso		
02/01/2023	CE	FORTALEZA	HGF	FLEBITE MEMBRO SUPERIO	Enviada	Eventos Adversos Relacionados à	Falhas envolvendo cateter venoso		
03/01/2023	CE	FORTALEZA	HGF	APRESENTANDO LP ESTÁGIO	Enviada	Eventos Adversos Relacionados à	Lesão por pressão		
03/01/2023	CE	FORTALEZA	HGF	Paciente estava dormindo quando	Enviada	Eventos Adversos Relacionados à	Queda do paciente		
03/01/2023	CE	FORTALEZA	HGF	PERDA DE SONDA NASOEN	Enviada	Eventos Adversos Relacionados à	Falhas envolvendo sondas		
03/01/2023	CE	FORTALEZA	HGF	Flebite em membro superior	Enviada	Eventos Adversos Relacionados à	Falhas envolvendo cateter venoso		
03/01/2023	CE	FORTALEZA	HGF	Paciente deu entrada na	Enviada	Eventos Adversos Relacionados à	Lesão por pressão		
10/01/2023	CE	FORTALEZA	HGF	Decanulou acidentalmente	Enviada	Eventos Adversos Relacionados à	Extubação endotraqueal		
10/01/2023	CE	FORTALEZA	HGF	Por volta das 22h o paciente queixa-se	Em retificação	Eventos Adversos Relacionados à	Falhas envolvendo cateter venoso		
19/01/2023	CE	FORTALEZA	HGF	Nutrição Parenteral instalada no	Enviada	Eventos Adversos Relacionados à	Falhas durante a assistência à saúde		
19/01/2023	CE	FORTALEZA	HGF	Alta da sala de recuperação com	Enviada	Eventos Adversos Relacionados à	Falhas durante a assistência à saúde		

19/01/2023	CE	FORTALE ZA	HGF	Paciente sem acompanh ante foi	Enviada	Eventos Adversos Relaciona dos à	Queda do paciente		
19/01/2023	CE	FORTALE ZA	HGF	-	Enviada	Eventos Adversos Relaciona dos à	Queda do paciente		
23/01/2023	CE	FORTALE ZA	HGF	LESÃO EM REGIÇÃO	Enviada	Eventos Adversos Relaciona dos à	Lesão por pressão		
23/01/2023	CE	FORTALE ZA	HGF	lesão em glúteo	Enviada	Eventos Adversos Relaciona dos à	Lesão por pressão		
23/01/2023	CE	FORTALE ZA	HGF	LESÃO EM GLÚTEO	Enviada	Eventos Adversos Relaciona dos à	Lesão por pressão		
23/01/2023	CE	FORTALE ZA	HGF	lesão em sacra	Enviada	Eventos Adversos Relaciona dos à	Lesão por pressão		
23/01/2023	CE	FORTALE ZA	HGF	LESÃO EM SACRA	Enviada	Eventos Adversos Relaciona dos à	Lesão por pressão		
23/01/2023	CE	FORTALE ZA	HGF	lesão em sacra	Enviada	Eventos Adversos Relaciona dos à	Lesão por pressão		
31/01/2023	CE	FORTALE ZA	HGF	O PACIENT E PUXOU	Enviada	Eventos Adversos Relaciona dos à	Falhas envolvend o sondas		
31/01/2023	CE	FORTALE ZA	HGF	Paciente apresenta ndo flebite	Enviada	Eventos Adversos Relaciona dos à	Falhas envolvend o cateter venoso		
31/01/2023	CE	FORTALE ZA	HGF	LESÃO POR PRESSÃ O NA	Enviada	Eventos Adversos Relaciona dos à	Lesão por pressão		
31/01/2023	CE	FORTALE ZA	HGF	PACIENT E ADMITID O DA	Enviada	Eventos Adversos Relaciona dos à	Falhas envolvend o sondas		
31/01/2023	CE	FORTALE ZA	HGF	Suporte com bombas de	Enviada	Eventos Adversos Relaciona dos à	Falhas envolvend o sondas		
31/01/2023	CE	FORTALE ZA	HGF	Paciente chamou a equipe por volta	Enviada	Eventos Adversos Relaciona dos à	Lesão por pressão		
31/01/2023	CE	FORTALE ZA	HGF	Paciente retirou sne	Enviada	Eventos Adversos Relaciona dos à	Falhas envolvend o sondas		

31/01/2023	CE	FORTALEZA	HGF	APRESENTA FLEBITE EM MSE	Enviada	Eventos Adversos Relacionados à	Falhas envolvendo cateter venoso		
31/01/2023	CE	FORTALEZA	HGF	PACIENTE APRESENTOU	Enviada	Eventos Adversos Relacionados à	Falhas envolvendo cateter venoso		
03/02/2023	CE	FORTALEZA	HGF	SACRO-GLÚTEO	Enviada	Eventos Adversos Relacionados à	Lesão por pressão		
07/02/2023	CE	FORTALEZA	HGF	Paciente idoso caiu da própria	Enviada	Eventos Adversos Relacionados à	Queda do paciente		
07/02/2023	CE	FORTALEZA	HGF	Paciente com hemiparesia à	Enviada	Eventos Adversos Relacionados à	Queda do paciente		
07/02/2023	CE	FORTALEZA	HGF	Flebite química em MSD	Enviada	Eventos Adversos Relacionados à	Falhas envolvendo cateter venoso		
07/02/2023	CE	FORTALEZA	HGF	PACIENTE EM LEITO DE UCE	Enviada	Eventos Adversos Relacionados à	Extubação endotraqueal		
10/02/2023	CE	FORTALEZA	HGF	Flebite em MSE	Enviada	Eventos Adversos Relacionados à	Falhas envolvendo cateter venoso		
10/02/2023	CE	FORTALEZA	HGF	GLÚTEO ESQUERDO	Enviada	Eventos Adversos Relacionados à	Lesão por pressão		
10/02/2023	CE	FORTALEZA	HGF	EXTRAVASAMENTO DE QUIMIOT	Enviada	Eventos Adversos Relacionados à	Falhas envolvendo cateter venoso		
10/02/2023	CE	FORTALEZA	HGF	Paciente caiu ao levantar da cama	Enviada	Eventos Adversos Relacionados à	Queda do paciente		
10/02/2023	CE	FORTALEZA	HGF	sacra	Enviada	Eventos Adversos Relacionados à	Lesão por pressão		
10/02/2023	CE	FORTALEZA	HGF	SACRA	Enviada	Eventos Adversos Relacionados à	Lesão por pressão		
10/02/2023	CE	FORTALEZA	HGF	FLEBITE EM MSD	Enviada	Eventos Adversos Relacionados à	Falhas envolvendo cateter venoso		
13/02/2023	CE	FORTALEZA	HGF	Flebite em MSE	Enviada	Eventos Adversos Relacionados à	Falhas envolvendo cateter venoso		

13/02/2023	CE	FORTALEZA	HGF	Flebite em MSD	Enviada	Eventos Adversos Relacionados à	Falhas envolvendo cateter venoso		
13/02/2023	CE	FORTALEZA	HGF	Extravasamento de sangue/hemocompo	Enviada	Eventos Adversos Relacionados à	Falhas envolvendo cateter venoso		
13/02/2023	CE	FORTALEZA	HGF	hiperemia em sacra	Enviada	Eventos Adversos Relacionados à	Lesão por pressão		
13/02/2023	CE	FORTALEZA	HGF	lesao necrótica em MSE relacionad	Enviada	Eventos Adversos Relacionados à	Lesão por pressão		
13/02/2023	CE	FORTALEZA	HGF	PANTURRILHA DIREITA	Enviada	Eventos Adversos Relacionados à	Lesão por pressão		
13/02/2023	CE	FORTALEZA	HGF	Flebite em MSE	Enviada	Eventos Adversos Relacionados à	Falhas envolvendo cateter venoso		
13/02/2023	CE	FORTALEZA	HGF	MSE	Enviada	Eventos Adversos Relacionados à	Falhas envolvendo cateter venoso		
13/02/2023	CE	FORTALEZA	HGF	SACRA	Enviada	Eventos Adversos Relacionados à	Lesão por pressão		
13/02/2023	CE	FORTALEZA	HGF	Flebite em MSE	Enviada	Eventos Adversos Relacionados à	Falhas envolvendo cateter venoso		
13/02/2023	CE	FORTALEZA	HGF	MSE	Enviada	Eventos Adversos Relacionados à	Falhas envolvendo cateter venoso		
13/02/2023	CE	FORTALEZA	HGF	MSE	Enviada	Eventos Adversos Relacionados à	Falhas envolvendo cateter venoso		
13/02/2023	CE	FORTALEZA	HGF	Não especificado do local	Enviada	Eventos Adversos Relacionados à	Falhas envolvendo cateter venoso		
22/02/2023	CE	FORTALEZA	HGF	Paciente caiu do leito ao tentar	Enviada	Eventos Adversos Relacionados à	Queda do paciente		
22/02/2023	CE	FORTALEZA	HGF	Flebite em MSE	Enviada	Eventos Adversos Relacionados à	Falhas envolvendo cateter venoso		
22/02/2023	CE	FORTALEZA	HGF	Flebite em MSE	Enviada	Eventos Adversos Relacionados à	Falhas envolvendo cateter venoso		

22/02/2023	CE	FORTALEZA	HGF	Paciente apresentou síncope ao ir ao	Enviada	Eventos Adversos Relacionados à	Queda do paciente		
22/02/2023	CE	FORTALEZA	HGF	sacra	Enviada	Eventos Adversos Relacionados à	Lesão por pressão		
22/02/2023	CE	FORTALEZA	HGF	PACIENTE CAIU DA PRÓPRIA	Enviada	Eventos Adversos Relacionados à	Queda do paciente		
22/02/2023	CE	FORTALEZA	HGF	PACIENTE SOFREU QUEDA	Enviada	Eventos Adversos Relacionados à	Queda do paciente		
22/02/2023	CE	FORTALEZA	HGF	Flebite em MSE	Enviada	Eventos Adversos Relacionados à	Falhas envolvendo o cateter venoso		
22/02/2023	CE	FORTALEZA	HGF	Flebite em MSD	Enviada	Eventos Adversos Relacionados à	Falhas envolvendo o cateter venoso		
22/02/2023	CE	FORTALEZA	HGF	Flebite em MSE	Enviada	Eventos Adversos Relacionados à	Falhas envolvendo o cateter venoso		
22/02/2023	CE	FORTALEZA	HGF	Flebite em MSD	Enviada	Eventos Adversos Relacionados à	Falhas envolvendo o cateter venoso		
22/02/2023	CE	FORTALEZA	HGF	Flebite em MSD	Enviada	Eventos Adversos Relacionados à	Falhas envolvendo o cateter venoso		
22/02/2023	CE	FORTALEZA	HGF	PACIENTE CAIU DA CAMA	Enviada	Eventos Adversos Relacionados à	Queda do paciente		
22/02/2023	CE	FORTALEZA	HGF	SACRA	Enviada	Eventos Adversos Relacionados à	Lesão por pressão		
23/02/2023	CE	FORTALEZA	HGF	INTERGLÚTEA	Enviada	Eventos Adversos Relacionados à	Lesão por pressão		
23/02/2023	CE	FORTALEZA	HGF	SACRA	Enviada	Eventos Adversos Relacionados à	Lesão por pressão		
23/02/2023	CE	FORTALEZA	HGF	SACRA	Enviada	Eventos Adversos Relacionados à	Lesão por pressão		
23/02/2023	CE	FORTALEZA	HGF	SACRA	Enviada	Eventos Adversos Relacionados à	Lesão por pressão		

23/02/2023	CE	FORTALEZA	HGF	SACROGLÚTEA	Enviada	Eventos Adversos Relacionados à	Lesão por pressão		
23/02/2023	CE	FORTALEZA	HGF	SACRA	Enviada	Eventos Adversos Relacionados à	Lesão por pressão		
23/02/2023	CE	FORTALEZA	HGF	SACROGLÚTEA	Enviada	Eventos Adversos Relacionados à	Lesão por pressão		
23/02/2023	CE	FORTALEZA	HGF	SACRA	Enviada	Eventos Adversos Relacionados à	Lesão por pressão		
23/02/2023	CE	FORTALEZA	HGF	INTERGLÚTEA	Enviada	Eventos Adversos Relacionados à	Lesão por pressão		
23/02/2023	CE	FORTALEZA	HGF	GLÚTEO	Enviada	Eventos Adversos Relacionados à	Lesão por pressão		
23/02/2023	CE	FORTALEZA	HGF	GLÚTEO	Enviada	Eventos Adversos Relacionados à	Lesão por pressão		
23/02/2023	CE	FORTALEZA	HGF	SACRA	Enviada	Eventos Adversos Relacionados à	Lesão por pressão		
23/02/2023	CE	FORTALEZA	HGF	SACRA	Enviada	Eventos Adversos Relacionados à	Lesão por pressão		
23/02/2023	CE	FORTALEZA	HGF	ESCÁPULA	Enviada	Eventos Adversos Relacionados à	Lesão por pressão		
23/02/2023	CE	FORTALEZA	HGF	MSE	Enviada	Eventos Adversos Relacionados à	Lesão por pressão		
23/02/2023	CE	FORTALEZA	HGF	SACRA	Enviada	Eventos Adversos Relacionados à	Lesão por pressão		
23/02/2023	CE	FORTALEZA	HGF	SACRA	Enviada	Eventos Adversos Relacionados à	Lesão por pressão		
23/02/2023	CE	FORTALEZA	HGF	SACRA	Enviada	Eventos Adversos Relacionados à	Lesão por pressão		
23/02/2023	CE	FORTALEZA	HGF	SACRA	Enviada	Eventos Adversos Relacionados à	Lesão por pressão		

23/02/2023	CE	FORTALEZA	HGF	SACRA	Enviada	Eventos Adversos Relacionados à	Lesão por pressão		
23/02/2023	CE	FORTALEZA	HGF	GLÚTEO	Enviada	Eventos Adversos Relacionados à	Lesão por pressão		
27/02/2023	CE	FORTALEZA	HGF	GLÚTEO	Enviada	Eventos Adversos Relacionados à	Lesão por pressão		
27/02/2023	CE	FORTALEZA	HGF	GLÚTEO	Enviada	Eventos Adversos Relacionados à	Lesão por pressão		
27/02/2023	CE	FORTALEZA	HGF	SACRA	Enviada	Eventos Adversos Relacionados à	Lesão por pressão		
27/02/2023	CE	FORTALEZA	HGF	SACRA	Enviada	Eventos Adversos Relacionados à	Lesão por pressão		
27/02/2023	CE	FORTALEZA	HGF	SACRA	Enviada	Eventos Adversos Relacionados à	Lesão por pressão		
27/02/2023	CE	FORTALEZA	HGF	GLÚTEO	Enviada	Eventos Adversos Relacionados à	Lesão por pressão		
27/02/2023	CE	FORTALEZA	HGF	GLÚTEO	Enviada	Eventos Adversos Relacionados à	Lesão por pressão		
27/02/2023	CE	FORTALEZA	HGF	INTERGLÚTEA	Enviada	Eventos Adversos Relacionados à	Lesão por pressão		
27/02/2023	CE	FORTALEZA	HGF	SACRA	Enviada	Eventos Adversos Relacionados à	Lesão por pressão		
27/02/2023	CE	FORTALEZA	HGF	SACRA	Enviada	Eventos Adversos Relacionados à	Lesão por pressão		
27/02/2023	CE	FORTALEZA	HGF	SACRA	Enviada	Eventos Adversos Relacionados à	Lesão por pressão		
27/02/2023	CE	FORTALEZA	HGF	SACRA	Enviada	Eventos Adversos Relacionados à	Lesão por pressão		
27/02/2023	CE	FORTALEZA	HGF	GLÚTEO	Enviada	Eventos Adversos Relacionados à	Lesão por pressão		

27/02/2023	CE	FORTALE ZA	HGF	SACRA	Enviada	Eventos Adversos Relaciona dos à	Lesão por pressão		
27/02/2023	CE	FORTALE ZA	HGF	SACRA	Enviada	Eventos Adversos Relaciona dos à	Lesão por pressão		
28/02/2023	CE	FORTALE ZA	HGF	GLÚTEO	Enviada	Eventos Adversos Relaciona dos à	Lesão por pressão		
03/03/2023	CE	FORTALE ZA	HGF	Má condução da assistênci	Em retificação	Eventos Adversos Relaciona dos à	Falhas durante a assistênci a à saúde		
06/03/2023	CE	FORTALE ZA	HGF	LESÃO POR PRESSÃ O EM	Enviada	Eventos Adversos Relaciona dos à	Lesão por pressão		
06/03/2023	CE	FORTALE ZA	HGF	LESÃO POR PRESSÃ O EM	Enviada	Eventos Adversos Relaciona dos à	Lesão por pressão		
06/03/2023	CE	FORTALE ZA	HGF	LESÃO POR PRESSÃ O EM	Enviada	Eventos Adversos Relaciona dos à	Lesão por pressão		
06/03/2023	CE	FORTALE ZA	HGF	LESÃO POR PRESSÃ O EM	Enviada	Eventos Adversos Relaciona dos à	Lesão por pressão		
06/03/2023	CE	FORTALE ZA	HGF	LESÃO POR PRESSÃ O EM	Enviada	Eventos Adversos Relaciona dos à	Lesão por pressão		
06/03/2023	CE	FORTALE ZA	HGF	LESÃO POR PRESSÃ O EM	Enviada	Eventos Adversos Relaciona dos à	Lesão por pressão		
06/03/2023	CE	FORTALE ZA	HGF	LESÃO POR PRESSÃ O EM	Enviada	Eventos Adversos Relaciona dos à	Lesão por pressão		
06/03/2023	CE	FORTALE ZA	HGF	LESÃO POR PRESSÃ O EM	Enviada	Eventos Adversos Relaciona dos à	Lesão por pressão		
06/03/2023	CE	FORTALE ZA	HGF	LESÃO POR PRESSÃ O EM	Enviada	Eventos Adversos Relaciona dos à	Lesão por pressão		
06/03/2023	CE	FORTALE ZA	HGF	LESÃO POR PRESSÃ O EM	Enviada	Eventos Adversos Relaciona dos à	Lesão por pressão		
06/03/2023	CE	FORTALE ZA	HGF	-	Enviada	Eventos Adversos Relaciona dos à	Falhas envolvend o cateter venoso		

06/03/2023	CE	FORTALEZA	HGF	LESÃO POR PRESSÃO EM	Enviada	Eventos Adversos Relacionados à	Lesão por pressão		
06/03/2023	CE	FORTALEZA	HGF	LESÃO POR PRESSÃO EM	Enviada	Eventos Adversos Relacionados à	Lesão por pressão		
06/03/2023	CE	FORTALEZA	HGF	LESÃO POR PRESSÃO EM	Enviada	Eventos Adversos Relacionados à	Lesão por pressão		
06/03/2023	CE	FORTALEZA	HGF	LESÃO POR PRESSÃO EM	Enviada	Eventos Adversos Relacionados à	Lesão por pressão		
06/03/2023	CE	FORTALEZA	HGF	LESÃO POR PRESSÃO EM	Enviada	Eventos Adversos Relacionados à	Lesão por pressão		
06/03/2023	CE	FORTALEZA	HGF	Administração oral de ácido trans-	Enviada	Eventos Adversos Relacionados à	Falhas durante a assistência à saúde		
06/03/2023	CE	FORTALEZA	HGF	LESÃO POR PRESSÃO EM	Em retificação	Eventos Adversos Relacionados à	Lesão por pressão		
06/03/2023	CE	FORTALEZA	HGF	Flebite em MSE	Enviada	Eventos Adversos Relacionados à	Falhas envolvendo o cateter venoso		
06/03/2023	CE	FORTALEZA	HGF	LESÃO POR PRESSÃO EM	Enviada	Eventos Adversos Relacionados à	Lesão por pressão		
06/03/2023	CE	FORTALEZA	HGF	LESÃO POR PRESSÃO EM	Enviada	Eventos Adversos Relacionados à	Lesão por pressão		
06/03/2023	CE	FORTALEZA	HGF	LESÃO POR PRESSÃO EM	Enviada	Eventos Adversos Relacionados à	Lesão por pressão		
06/03/2023	CE	FORTALEZA	HGF	paciente teve procedimento	Enviada	Eventos Adversos Relacionados à	Falhas nas atividades		
06/03/2023	CE	FORTALEZA	HGF	LESÃO POR PRESSÃO EM	Enviada	Eventos Adversos Relacionados à	Lesão por pressão		
06/03/2023	CE	FORTALEZA	HGF	sacra	Enviada	Eventos Adversos Relacionados à	Lesão por pressão		
06/03/2023	CE	FORTALEZA	HGF	LESÃO POR PRESSÃO EM	Enviada	Eventos Adversos Relacionados à	Lesão por pressão		

06/03/2023	CE	FORTALEZA	HGF	rompimento de flictena	Enviada	Eventos Adversos Relacionados à	Lesão por pressão		
06/03/2023	CE	FORTALEZA	HGF	LESÃO POR PRESSÃO EM	Enviada	Eventos Adversos Relacionados à	Lesão por pressão		
06/03/2023	CE	FORTALEZA	HGF	LESÃO POR PRESSÃO EM	Em retificação	Eventos Adversos Relacionados à	Lesão por pressão		
06/03/2023	CE	FORTALEZA	HGF	LESÃO POR PRESSÃO EM	Enviada	Eventos Adversos Relacionados à	Lesão por pressão		
06/03/2023	CE	FORTALEZA	HGF	LESÃO POR PRESSÃO EM	Enviada	Eventos Adversos Relacionados à	Lesão por pressão		
06/03/2023	CE	FORTALEZA	HGF	LESÃO POR PRESSÃO EM	Enviada	Eventos Adversos Relacionados à	Lesão por pressão		
06/03/2023	CE	FORTALEZA	HGF	-	Enviada	Eventos Adversos Relacionados à	Falhas envolvendo cateter venoso		
06/03/2023	CE	FORTALEZA	HGF	LESÃO POR PRESSÃO EM	Enviada	Eventos Adversos Relacionados à	Lesão por pressão		
06/03/2023	CE	FORTALEZA	HGF	LESÃO POR PRESSÃO EM	Enviada	Eventos Adversos Relacionados à	Lesão por pressão		
06/03/2023	CE	FORTALEZA	HGF	Flebite em MSD	Enviada	Eventos Adversos Relacionados à	Falhas envolvendo cateter venoso		
06/03/2023	CE	FORTALEZA	HGF	LESÃO POR PRESSÃO EM	Enviada	Eventos Adversos Relacionados à	Lesão por pressão		
07/03/2023	CE	FORTALEZA	HGF	LESÃO EM REGIÃO SACRA	Enviada	Eventos Adversos Relacionados à	Lesão por pressão		
07/03/2023	CE	FORTALEZA	HGF	LESÃO EM REGIÃO SACRA	Enviada	Eventos Adversos Relacionados à	Lesão por pressão		
07/03/2023	CE	FORTALEZA	HGF	LESÃO EM REGIÃO SACRA	Enviada	Eventos Adversos Relacionados à	Lesão por pressão		
07/03/2023	CE	FORTALEZA	HGF	LESÃO EM REGIÃO SACRA	Enviada	Eventos Adversos Relacionados à	Lesão por pressão		

07/03/2023	CE	FORTALEZA	HGF	LESÃO EM REGIÃO INTERGI	Enviada	Eventos Adversos Relacionados à	Lesão por pressão		
07/03/2023	CE	FORTALEZA	HGF	LESÃO EM REGIÃO SACRA	Enviada	Eventos Adversos Relacionados à	Lesão por pressão		
07/03/2023	CE	FORTALEZA	HGF	LESÃO EM REGIÃO SACRA	Enviada	Eventos Adversos Relacionados à	Lesão por pressão		
07/03/2023	CE	FORTALEZA	HGF	LESÃO EM REGIÃO SACRA	Enviada	Eventos Adversos Relacionados à	Lesão por pressão		
07/03/2023	CE	FORTALEZA	HGF	LESÃO EM REGIÃO SACRA	Enviada	Eventos Adversos Relacionados à	Lesão por pressão		
07/03/2023	CE	FORTALEZA	HGF	LESÃO EM REGIÃO SACRA	Enviada	Eventos Adversos Relacionados à	Lesão por pressão		
07/03/2023	CE	FORTALEZA	HGF	LESÃO EM REGIÃO SACRA	Enviada	Eventos Adversos Relacionados à	Lesão por pressão		
07/03/2023	CE	FORTALEZA	HGF	LESÃO EM REGIÃO SACRAL	Enviada	Eventos Adversos Relacionados à	Lesão por pressão		
07/03/2023	CE	FORTALEZA	HGF	LESÃO EM REGIÃO SACRA	Enviada	Eventos Adversos Relacionados à	Lesão por pressão		
07/03/2023	CE	FORTALEZA	HGF	LESÃO EM REGIÃO SACRA	Enviada	Eventos Adversos Relacionados à	Lesão por pressão		
07/03/2023	CE	FORTALEZA	HGF	LESÃO EM REGIÃO SACRA	Enviada	Eventos Adversos Relacionados à	Lesão por pressão		
07/03/2023	CE	FORTALEZA	HGF	Flebite em MSE	Enviada	Eventos Adversos Relacionados à	Falhas envolvendo o cateter venoso		
07/03/2023	CE	FORTALEZA	HGF	LESÃO EM REGIÃO SACRA	Enviada	Eventos Adversos Relacionados à	Lesão por pressão		
07/03/2023	CE	FORTALEZA	HGF	Flebite em MSE	Enviada	Eventos Adversos Relacionados à	Falhas envolvendo o cateter venoso		
07/03/2023	CE	FORTALEZA	HGF	LESÃO EM REGIÃO SACRA	Enviada	Eventos Adversos Relacionados à	Lesão por pressão		

07/03/2023	CE	FORTALEZA	HGF	Flebite em MSD	Enviada	Eventos Adversos Relacionados à	Falhas envolvendo o cateter venoso		
07/03/2023	CE	FORTALEZA	HGF	LESÃO EM REGIÃO SACRA	Enviada	Eventos Adversos Relacionados à	Lesão por pressão		
07/03/2023	CE	FORTALEZA	HGF	LESÃO EM REGIÃO SACRA	Enviada	Eventos Adversos Relacionados à	Lesão por pressão		
07/03/2023	CE	FORTALEZA	HGF	LESÃO EM REGIÃO SACRA	Enviada	Eventos Adversos Relacionados à	Lesão por pressão		
07/03/2023	CE	FORTALEZA	HGF	NÃO VERIFICADO SSVV	Enviada	Eventos Adversos Relacionados à	Falhas nas atividades		
07/03/2023	CE	FORTALEZA	HGF	Paciente apresentou rash cutâneo	Enviada	Eventos Adversos Relacionados à	Falhas durante a assistência à saúde		
07/03/2023	CE	FORTALEZA	HGF	Paciente apresentou disnpeia	Enviada	Eventos Adversos Relacionados à	Falhas durante a assistência à saúde		
07/03/2023	CE	FORTALEZA	HGF	LESÃO EM REGIÃO GLUTEO	Enviada	Eventos Adversos Relacionados à	Lesão por pressão		
07/03/2023	CE	FORTALEZA	HGF	LESÃO EM REGIÃO SACRA	Enviada	Eventos Adversos Relacionados à	Lesão por pressão		
07/03/2023	CE	FORTALEZA	HGF	Paciente em uso de Vancomici	Enviada	Eventos Adversos Relacionados à	Falhas durante a assistência à saúde		
07/03/2023	CE	FORTALEZA	HGF	Paciente apresentou hipotermi	Enviada	Eventos Adversos Relacionados à	Falhas durante a assistência à saúde		
07/03/2023	CE	FORTALEZA	HGF	LESÃO EM REGIÃO SACRA	Enviada	Eventos Adversos Relacionados à	Lesão por pressão		
07/03/2023	CE	FORTALEZA	HGF	LESÃO EM REGIÃO SACRA	Enviada	Eventos Adversos Relacionados à	Lesão por pressão		
07/03/2023	CE	FORTALEZA	HGF	LESÃO EM REGIÃO SACRA	Enviada	Eventos Adversos Relacionados à	Lesão por pressão		
07/03/2023	CE	FORTALEZA	HGF	LESÃO EM REGIÃO SACRA	Enviada	Eventos Adversos Relacionados à	Lesão por pressão		

07/03/2023	CE	FORTALEZA	HGF	LESÃO EM REGIÃO SACRA	Enviada	Eventos Adversos Relacionados à	Lesão por pressão		
07/03/2023	CE	FORTALEZA	HGF	LESÃO EM REGIÃO SACRA	Enviada	Eventos Adversos Relacionados à	Lesão por pressão		
07/03/2023	CE	FORTALEZA	HGF	LESÃO EM REGIÃO SACRA	Enviada	Eventos Adversos Relacionados à	Lesão por pressão		
07/03/2023	CE	FORTALEZA	HGF	LESÃO EM REGIÃO SACRA	Enviada	Eventos Adversos Relacionados à	Lesão por pressão		
07/03/2023	CE	FORTALEZA	HGF	LESÃO EM REGIÃO EM	Enviada	Eventos Adversos Relacionados à	Lesão por pressão		
07/03/2023	CE	FORTALEZA	HGF	GLÚTEO DIREITO	Enviada	Eventos Adversos Relacionados à	Lesão por pressão		
07/03/2023	CE	FORTALEZA	HGF	LESÃO EM REGIÃO SACRA	Enviada	Eventos Adversos Relacionados à	Lesão por pressão		
07/03/2023	CE	FORTALEZA	HGF	LESÃO EM REGIÃO SACRA	Em retificação	Eventos Adversos Relacionados à	Lesão por pressão		
07/03/2023	CE	FORTALEZA	HGF	LESÃO EM REGIÃO SACRA	Enviada	Eventos Adversos Relacionados à	Lesão por pressão		
07/03/2023	CE	FORTALEZA	HGF	LESÃO EM REGIÃO SACRA	Enviada	Eventos Adversos Relacionados à	Lesão por pressão		
07/03/2023	CE	FORTALEZA	HGF	LESÃO EM REGIÃO SACRA	Enviada	Eventos Adversos Relacionados à	Lesão por pressão		
07/03/2023	CE	FORTALEZA	HGF	LESÃO EM REGIÃO OCCIPIT	Enviada	Eventos Adversos Relacionados à	Lesão por pressão		
07/03/2023	CE	FORTALEZA	HGF	LESÃO EM REGIÃO GLUTEO	Em retificação	Eventos Adversos Relacionados à	Lesão por pressão		
07/03/2023	CE	FORTALEZA	HGF	PACIENTE AGUARDANDO	Enviada	Eventos Adversos Relacionados à	Falhas durante a assistência à saúde		
07/03/2023	CE	FORTALEZA	HGF	LESÃO EM REGIÃO SACRA	Enviada	Eventos Adversos Relacionados à	Lesão por pressão		

07/03/2023	CE	FORTALEZA	HGF	LESÃO EM REGIÃO SACRA	Enviada	Eventos Adversos Relacionados à	Lesão por pressão		
07/03/2023	CE	FORTALEZA	HGF	LESÃO EM REGIÃO DE	Enviada	Eventos Adversos Relacionados à	Lesão por pressão		
07/03/2023	CE	FORTALEZA	HGF	-	Enviada	Eventos Adversos Relacionados à	Falhas envolvendo o cateter venoso		
07/03/2023	CE	FORTALEZA	HGF	Nao verificada FR, sendo	Enviada	Eventos Adversos Relacionados à	Falhas durante a assistência à saúde		
07/03/2023	CE	FORTALEZA	HGF	-	Enviada	Eventos Adversos Relacionados à	Falhas envolvendo sondas		
07/03/2023	CE	FORTALEZA	HGF	-	Enviada	Eventos Adversos Relacionados à	Falhas envolvendo sondas		
07/03/2023	CE	FORTALEZA	HGF	LESÃO EM NARINA DIREITA	Enviada	Eventos Adversos Relacionados à	Lesão por pressão		
07/03/2023	CE	FORTALEZA	HGF	PACIENTE TRACIONOU	Enviada	Eventos Adversos Relacionados à	Falhas envolvendo sondas		
07/03/2023	CE	FORTALEZA	HGF	PACIENTE EM CONTENÇÃO	Enviada	Eventos Adversos Relacionados à	Falhas envolvendo sondas		
08/03/2023	CE	FORTALEZA	HGF	LESÃO EM REGIÃO SACRA	Enviada	Eventos Adversos Relacionados à	Lesão por pressão		
08/03/2023	CE	FORTALEZA	HGF	LESÃO EM REGIÃO SACRA	Concluída	Eventos Adversos Relacionados à	Lesão por pressão		
08/03/2023	CE	FORTALEZA	HGF	LESÃO EM REGIÃO SACRA	Enviada	Eventos Adversos Relacionados à	Lesão por pressão		
08/03/2023	CE	FORTALEZA	HGF	LESÃO EM REGIÃO SACRA	Concluída	Eventos Adversos Relacionados à	Lesão por pressão		
08/03/2023	CE	FORTALEZA	HGF	LESÃO EM REGIÃO SACRA	Enviada	Eventos Adversos Relacionados à	Lesão por pressão		
08/03/2023	CE	FORTALEZA	HGF	LESÃO EM REGIÃO SACRA	Em análise	Eventos Adversos Relacionados à	Lesão por pressão		

08/03/2023	CE	FORTALEZA	HGF	LESÃO EM REGIÃO SACRA	Em análise	Eventos Adversos Relacionados à	Lesão por pressão		
08/03/2023	CE	FORTALEZA	HGF	LESÃO EM REGIÃO INTERGL	Em análise	Eventos Adversos Relacionados à	Lesão por pressão		
08/03/2023	CE	FORTALEZA	HGF	LESÃO EM REGIÃO DE	Em análise	Eventos Adversos Relacionados à	Lesão por pressão		
08/03/2023	CE	FORTALEZA	HGF	LESÃO EM REGIÃO DE	Em análise	Eventos Adversos Relacionados à	Lesão por pressão		
08/03/2023	CE	FORTALEZA	HGF	LESÃO EM REGIÃO DE	Em análise	Eventos Adversos Relacionados à	Lesão por pressão		
08/03/2023	CE	FORTALEZA	HGF	LESÃO EM REGIÃO DORSO	Em análise	Eventos Adversos Relacionados à	Lesão por pressão		
08/03/2023	CE	FORTALEZA	HGF	LESÃO EM REGIÃO SACRA	Em análise	Eventos Adversos Relacionados à	Lesão por pressão		
08/03/2023	CE	FORTALEZA	HGF	LESÃO EM REGIÃO SACRA	Concluída	Eventos Adversos Relacionados à	Lesão por pressão		
08/03/2023	CE	FORTALEZA	HGF	LESÃO EM REGIÃO SACRA	Em análise	Eventos Adversos Relacionados à	Lesão por pressão		
08/03/2023	CE	FORTALEZA	HGF	LESÃO EM REGIÃO SACRA	Em análise	Eventos Adversos Relacionados à	Lesão por pressão		
08/03/2023	CE	FORTALEZA	HGF	LESÃO EM REGIÃO SACRA	Concluída	Eventos Adversos Relacionados à	Lesão por pressão		
08/03/2023	CE	FORTALEZA	HGF	LESÃO EM REGIÃO INTERGL	Em análise	Eventos Adversos Relacionados à	Lesão por pressão		
08/03/2023	CE	FORTALEZA	HGF	LESÃO EM REGIÃO SACRA	Concluída	Eventos Adversos Relacionados à	Lesão por pressão		
08/03/2023	CE	FORTALEZA	HGF	LESÃO EM REGIÃO SACRA	Em análise	Eventos Adversos Relacionados à	Lesão por pressão		
08/03/2023	CE	FORTALEZA	HGF	LESÃO EM REGIÃO SACRA	Em análise	Eventos Adversos Relacionados à	Lesão por pressão		

08/03/2023	CE	FORTALE ZA	HGF	LESÃO EM REGIÃO SACRA	Em análise	Eventos Adversos Relaciona dos à	Lesão por pressão		
08/03/2023	CE	FORTALE ZA	HGF	LESÃO EM REGIÃO GLUTEA	Em análise	Eventos Adversos Relaciona dos à	Lesão por pressão		
08/03/2023	CE	FORTALE ZA	HGF	LESÃO EM REGIÃO SACRA	Em análise	Eventos Adversos Relaciona dos à	Lesão por pressão		
08/03/2023	CE	FORTALE ZA	HGF	LESÃO EM REGIÃO SACRA	Enviada	Eventos Adversos Relaciona dos à	Lesão por pressão		
08/03/2023	CE	FORTALE ZA	HGF	LESÃO EM REGIÃO SACRA	Em análise	Eventos Adversos Relaciona dos à	Lesão por pressão		
08/03/2023	CE	FORTALE ZA	HGF	LESÃO EM REGIÃO SACRA	Em análise	Eventos Adversos Relaciona dos à	Lesão por pressão		
08/03/2023	CE	FORTALE ZA	HGF	LESÃO EM REGIÃO SACRA	Em análise	Eventos Adversos Relaciona dos à	Lesão por pressão		
08/03/2023	CE	FORTALE ZA	HGF	LESÃO EM REGIÃO SACRA	Em análise	Eventos Adversos Relaciona dos à	Lesão por pressão		
08/03/2023	CE	FORTALE ZA	HGF	LESÃO EM REGIÃO SACRA	Em análise	Eventos Adversos Relaciona dos à	Lesão por pressão		
08/03/2023	CE	FORTALE ZA	HGF	LESÃO EM REGIÃO SACRA	Em análise	Eventos Adversos Relaciona dos à	Lesão por pressão		
08/03/2023	CE	FORTALE ZA	HGF	LESÃO EM REGIÃO SACRA	Em análise	Eventos Adversos Relaciona dos à	Lesão por pressão		
08/03/2023	CE	FORTALE ZA	HGF	LESÃO EM REGIÃO GLUTEO	Em análise	Eventos Adversos Relaciona dos à	Lesão por pressão		
08/03/2023	CE	FORTALE ZA	HGF	LESÃO EM REGIÃO SACRA	Em análise	Eventos Adversos Relaciona dos à	Lesão por pressão		
08/03/2023	CE	FORTALE ZA	HGF	LESÃO EM REGIÃO SACRA	Em análise	Eventos Adversos Relaciona dos à	Lesão por pressão		
08/03/2023	CE	FORTALE ZA	HGF	LESÃO EM REGIÃO SACRA	Em análise	Eventos Adversos Relaciona dos à	Lesão por pressão		

08/03/2023	CE	FORTALEZA	HGF	LESÃO EM REGIÃO SACRA	Em análise	Eventos Adversos Relacionados à	Lesão por pressão		
08/03/2023	CE	FORTALEZA	HGF	LESÃO EM REGIÃO SACRA	Em análise	Eventos Adversos Relacionados à	Lesão por pressão		
08/03/2023	CE	FORTALEZA	HGF	LESÃO EM REGIÃO SACRA	Em análise	Eventos Adversos Relacionados à	Lesão por pressão		
08/03/2023	CE	FORTALEZA	HGF	LESÃO EM REGIÃO DE	Em análise	Eventos Adversos Relacionados à	Lesão por pressão		
17/03/2023	CE	FORTALEZA	HGF	LESÃO EM REGIÃO DORSAL	Enviada	Eventos Adversos Relacionados à	Lesão por pressão		
17/03/2023	CE	FORTALEZA	HGF	LESÃO EM REGIÃO SACRA	Enviada	Eventos Adversos Relacionados à	Lesão por pressão		
17/03/2023	CE	FORTALEZA	HGF	LESÃO EM REGIÃO SACRA	Enviada	Eventos Adversos Relacionados à	Lesão por pressão		
17/03/2023	CE	FORTALEZA	HGF	LESÃO EM REGIÃO INTERGL	Enviada	Eventos Adversos Relacionados à	Lesão por pressão		
17/03/2023	CE	FORTALEZA	HGF	LESÃO EM MALEOLO	Enviada	Eventos Adversos Relacionados à	Lesão por pressão		
17/03/2023	CE	FORTALEZA	HGF	LESÃO EM REGIÃO SACRA	Enviada	Eventos Adversos Relacionados à	Lesão por pressão		
17/03/2023	CE	FORTALEZA	HGF	LESÃO EM REGIÃO SACRA	Enviada	Eventos Adversos Relacionados à	Lesão por pressão		
17/03/2023	CE	FORTALEZA	HGF	LESÃO EM REGIÃO SACRA	Enviada	Eventos Adversos Relacionados à	Lesão por pressão		
17/03/2023	CE	FORTALEZA	HGF	LESÃO EM REGIÃO SACRA	Enviada	Eventos Adversos Relacionados à	Lesão por pressão		
17/03/2023	CE	FORTALEZA	HGF	LESÃO EM REGIÃO SACRA	Enviada	Eventos Adversos Relacionados à	Lesão por pressão		
17/03/2023	CE	FORTALEZA	HGF	LESÃO EM REGIÃO SACRA	Enviada	Eventos Adversos Relacionados à	Lesão por pressão		

17/03/2023	CE	FORTALEZA	HGF	LESÃO EM REGIÃO SACRA	Enviada	Eventos Adversos Relacionados à	Lesão por pressão		
17/03/2023	CE	FORTALEZA	HGF	LESÃO EM REGIÃO SACRA	Enviada	Eventos Adversos Relacionados à	Lesão por pressão		
17/03/2023	CE	FORTALEZA	HGF	LESÃO EM REGIÃO SACRA	Enviada	Eventos Adversos Relacionados à	Lesão por pressão		
17/03/2023	CE	FORTALEZA	HGF	LESÃO EM REGIÃO SACRA	Enviada	Eventos Adversos Relacionados à	Lesão por pressão		
17/03/2023	CE	FORTALEZA	HGF	LESÃO EM REGIÃO SACRA	Enviada	Eventos Adversos Relacionados à	Lesão por pressão		
20/03/2023	CE	FORTALEZA	HGF	LESÃO EM REGIÃO SACRA	Enviada	Eventos Adversos Relacionados à	Lesão por pressão		
20/03/2023	CE	FORTALEZA	HGF	LESÃO EM REGIÃO SACRA	Enviada	Eventos Adversos Relacionados à	Lesão por pressão		
20/03/2023	CE	FORTALEZA	HGF	LESÃO EM REGIÃO SACRA	Enviada	Eventos Adversos Relacionados à	Lesão por pressão		
20/03/2023	CE	FORTALEZA	HGF	LESÃO EM REGIÃO SACRA	Enviada	Eventos Adversos Relacionados à	Lesão por pressão		
20/03/2023	CE	FORTALEZA	HGF	LESÃO EM REGIÃO SACRA	Enviada	Eventos Adversos Relacionados à	Lesão por pressão		
20/03/2023	CE	FORTALEZA	HGF	LESÃO EM REGIÃO SACRA	Enviada	Eventos Adversos Relacionados à	Lesão por pressão		
20/03/2023	CE	FORTALEZA	HGF	LESÃO EM REGIÃO SACRA	Enviada	Eventos Adversos Relacionados à	Lesão por pressão		
20/03/2023	CE	FORTALEZA	HGF	LESÃO EM REGIÃO SACRA	Enviada	Eventos Adversos Relacionados à	Lesão por pressão		
20/03/2023	CE	FORTALEZA	HGF	LESÃO EM TROCANTER	Enviada	Eventos Adversos Relacionados à	Lesão por pressão		
20/03/2023	CE	FORTALEZA	HGF	LESÃO EM TROCANTER	Enviada	Eventos Adversos Relacionados à	Lesão por pressão		

20/03/2023	CE	FORTALEZA	HGF	LESÃO EM REGIÃO DE	Enviada	Eventos Adversos Relacionados à	Lesão por pressão		
20/03/2023	CE	FORTALEZA	HGF	LESÃO EM REGIÃO SACRA	Enviada	Eventos Adversos Relacionados à	Lesão por pressão		
20/03/2023	CE	FORTALEZA	HGF	LESÃO EM GLÚTEO DIREITO	Enviada	Eventos Adversos Relacionados à	Lesão por pressão		
20/03/2023	CE	FORTALEZA	HGF	LESÃO EM REGIÃO SACRA	Enviada	Eventos Adversos Relacionados à	Lesão por pressão		
20/03/2023	CE	FORTALEZA	HGF	LESÃO EM REGIÃO SACRA	Enviada	Eventos Adversos Relacionados à	Lesão por pressão		
20/03/2023	CE	FORTALEZA	HGF	LESÃO EM REGIÃO SACRA	Enviada	Eventos Adversos Relacionados à	Lesão por pressão		
20/03/2023	CE	FORTALEZA	HGF	LESÃO EM GLÚTEO	Enviada	Eventos Adversos Relacionados à	Lesão por pressão		
20/03/2023	CE	FORTALEZA	HGF	LESÃO EM TROCANTER	Enviada	Eventos Adversos Relacionados à	Lesão por pressão		
20/03/2023	CE	FORTALEZA	HGF	LESÃO EM REGIÃO DE	Enviada	Eventos Adversos Relacionados à	Lesão por pressão		
20/03/2023	CE	FORTALEZA	HGF	LESÃO EM REGIÃO SACRA	Enviada	Eventos Adversos Relacionados à	Lesão por pressão		
20/03/2023	CE	FORTALEZA	HGF	LESÃO EM REGIÃO DE	Enviada	Eventos Adversos Relacionados à	Lesão por pressão		
20/03/2023	CE	FORTALEZA	HGF	LESÃO EM REGIÃO SACRA	Enviada	Eventos Adversos Relacionados à	Lesão por pressão		
20/03/2023	CE	FORTALEZA	HGF	LESÃO EM REGIÃO SACRA	Enviada	Eventos Adversos Relacionados à	Lesão por pressão		
20/03/2023	CE	FORTALEZA	HGF	LESÃO EM REGIÃO SACRA	Enviada	Eventos Adversos Relacionados à	Lesão por pressão		
20/03/2023	CE	FORTALEZA	HGF	LESÃO EM CALCÂNEO	Enviada	Eventos Adversos Relacionados à	Lesão por pressão		

20/03/2023	CE	FORTALE ZA	HGF	LESÃO EM REGIÃO SACRA	Enviada	Eventos Adversos Relaciona dos à	Lesão por pressão		
20/03/2023	CE	FORTALE ZA	HGF	LESÃO EM REGIÃO SACRA	Enviada	Eventos Adversos Relaciona dos à	Lesão por pressão		
20/03/2023	CE	FORTALE ZA	HGF	LESÃO EM REGIÃO SACRA	Enviada	Eventos Adversos Relaciona dos à	Lesão por pressão		
20/03/2023	CE	FORTALE ZA	HGF	LESÃO EM REGIÃO SACRA	Enviada	Eventos Adversos Relaciona dos à	Lesão por pressão		
21/03/2023	CE	FORTALE ZA	HGF	LESÃO EM REGIÃO SACRA	Enviada	Eventos Adversos Relaciona dos à	Lesão por pressão		
21/03/2023	CE	FORTALE ZA	HGF	LESÃO EM REGIÃO DE	Enviada	Eventos Adversos Relaciona dos à	Lesão por pressão		
22/03/2023	CE	FORTALE ZA	HGF	MSE	Enviada	Eventos Adversos Relaciona dos à	Falhas envolvend o cateter venoso		
22/03/2023	CE	FORTALE ZA	HGF	PACIENT E EM CUIDADO S	Enviada	Eventos Adversos Relaciona dos à	Falhas durante a assistênci a à saúde		
22/03/2023	CE	FORTALE ZA	HGF	MSE	Enviada	Eventos Adversos Relaciona dos à	Falhas envolvend o cateter venoso		
23/03/2023	CE	FORTALE ZA	HGF	LESÃO EM REGIÃO SACRA	Enviada	Eventos Adversos Relaciona dos à	Lesão por pressão		
23/03/2023	CE	FORTALE ZA	HGF	LESÃO EM REGIÃO SACRA	Enviada	Eventos Adversos Relaciona dos à	Lesão por pressão		
23/03/2023	CE	FORTALE ZA	HGF	LESÃO EM REGIÃO SACRA	Enviada	Eventos Adversos Relaciona dos à	Lesão por pressão		
23/03/2023	CE	FORTALE ZA	HGF	LESÃO EM REGIÃO SACRA	Enviada	Eventos Adversos Relaciona dos à	Lesão por pressão		
23/03/2023	CE	FORTALE ZA	HGF	LESÃO EM REGIÃO SACRAL	Enviada	Eventos Adversos Relaciona dos à	Lesão por pressão		
23/03/2023	CE	FORTALE ZA	HGF	LESÃO EM REGIÃO SACRA	Enviada	Eventos Adversos Relaciona dos à	Lesão por pressão		

23/03/2023	CE	FORTALEZA	HGF	LESÃO EM REGIÃO SACRA	Concluída	Eventos Adversos Relacionados à	Lesão por pressão		
23/03/2023	CE	FORTALEZA	HGF	LESÃO EM REGIÃO SACRA	Concluída	Eventos Adversos Relacionados à	Lesão por pressão		
23/03/2023	CE	FORTALEZA	HGF	LESÃO EM REGIÃO SACRA	Enviada	Eventos Adversos Relacionados à	Lesão por pressão		
23/03/2023	CE	FORTALEZA	HGF	LESÃO EM REGIÃO GLÚTEA	Enviada	Eventos Adversos Relacionados à	Lesão por pressão		
23/03/2023	CE	FORTALEZA	HGF	LESÃO EM REGIÃO SACRA	Enviada	Eventos Adversos Relacionados à	Lesão por pressão		
23/03/2023	CE	FORTALEZA	HGF	LESÃO EM REGIÃO GLÚTEA	Enviada	Eventos Adversos Relacionados à	Lesão por pressão		
23/03/2023	CE	FORTALEZA	HGF	LESÃO EM REGIÃO GLÚTEA	Enviada	Eventos Adversos Relacionados à	Lesão por pressão		
23/03/2023	CE	FORTALEZA	HGF	LESÃO EM REGIÃO SACRA	Enviada	Eventos Adversos Relacionados à	Lesão por pressão		
23/03/2023	CE	FORTALEZA	HGF	LESÃO EM REGIÃO SACRA	Enviada	Eventos Adversos Relacionados à	Lesão por pressão		
23/03/2023	CE	FORTALEZA	HGF	LESÃO EM CALCÂNEO	Enviada	Eventos Adversos Relacionados à	Lesão por pressão		
23/03/2023	CE	FORTALEZA	HGF	LESÃO EM REGIÃO SACRA	Concluída	Eventos Adversos Relacionados à	Lesão por pressão		
23/03/2023	CE	FORTALEZA	HGF	LESÃO EM CALCÂNEO	Concluída	Eventos Adversos Relacionados à	Lesão por pressão		
23/03/2023	CE	FORTALEZA	HGF	LESÃO EM REGIÃO SACRA	Enviada	Eventos Adversos Relacionados à	Lesão por pressão		
23/03/2023	CE	FORTALEZA	HGF	LESÃO EM REGIÃO SACRA	Enviada	Eventos Adversos Relacionados à	Lesão por pressão		
23/03/2023	CE	FORTALEZA	HGF	LESÃO EM REGIÃO SACRA	Enviada	Eventos Adversos Relacionados à	Lesão por pressão		

23/03/2023	CE	FORTALE ZA	HGF	LESÃO EM REGIÃO GLÚTEA	Concluída	Eventos Adversos Relaciona dos à	Lesão por pressão		
23/03/2023	CE	FORTALE ZA	HGF	LESÃO EM REGIÃO SACRA	Enviada	Eventos Adversos Relaciona dos à	Lesão por pressão		
23/03/2023	CE	FORTALE ZA	HGF	LESÃO EM REGIÃO SACRAL	Enviada	Eventos Adversos Relaciona dos à	Lesão por pressão		
23/03/2023	CE	FORTALE ZA	HGF	LESÃO EM REGIÃO SACRAL	Enviada	Eventos Adversos Relaciona dos à	Lesão por pressão		
23/03/2023	CE	FORTALE ZA	HGF	LESÃO EM REGIÃO SACRA	Enviada	Eventos Adversos Relaciona dos à	Lesão por pressão		
23/03/2023	CE	FORTALE ZA	HGF	LESÃO EM REGIÃO SACRA	Enviada	Eventos Adversos Relaciona dos à	Lesão por pressão		
23/03/2023	CE	FORTALE ZA	HGF	LESÃO EM CALCÂN EO	Enviada	Eventos Adversos Relaciona dos à	Lesão por pressão		
23/03/2023	CE	FORTALE ZA	HGF	LESÃO EM REGIÃO TROCAN	Enviada	Eventos Adversos Relaciona dos à	Lesão por pressão		
23/03/2023	CE	FORTALE ZA	HGF	LESÃO EM REGIÃO SACRA	Enviada	Eventos Adversos Relaciona dos à	Lesão por pressão		
23/03/2023	CE	FORTALE ZA	HGF	LESÃO EM REGIÃO ESCAPUL	Enviada	Eventos Adversos Relaciona dos à	Lesão por pressão		
23/03/2023	CE	FORTALE ZA	HGF	LESÃO EM REGIÃO DORSAL	Enviada	Eventos Adversos Relaciona dos à	Lesão por pressão		
23/03/2023	CE	FORTALE ZA	HGF	LESÃO EM REGIÃO SACRA	Enviada	Eventos Adversos Relaciona dos à	Lesão por pressão		
23/03/2023	CE	FORTALE ZA	HGF	LESÃO EM REGIÃO SACRA	Enviada	Eventos Adversos Relaciona dos à	Lesão por pressão		
23/03/2023	CE	FORTALE ZA	HGF	LESÃO EM CALCÂN EO	Enviada	Eventos Adversos Relaciona dos à	Lesão por pressão		
23/03/2023	CE	FORTALE ZA	HGF	LESÃO EM REGIÃO SACRA	Enviada	Eventos Adversos Relaciona dos à	Lesão por pressão		

23/03/2023	CE	FORTALE ZA	HGF	LESÃO EM REGIÃO SACRA	Enviada	Eventos Adversos Relaciona dos à	Lesão por pressão		
23/03/2023	CE	FORTALE ZA	HGF	LESÃO EM REGIÃO SACRA	Enviada	Eventos Adversos Relaciona dos à	Lesão por pressão		
23/03/2023	CE	FORTALE ZA	HGF	LESÃO EM REGIÃO SACRA	Enviada	Eventos Adversos Relaciona dos à	Lesão por pressão		
23/03/2023	CE	FORTALE ZA	HGF	LESÃO EM REGIÃO SACRA	Enviada	Eventos Adversos Relaciona dos à	Lesão por pressão		
23/03/2023	CE	FORTALE ZA	HGF	LESÃO EM REGIÃO SACRA	Enviada	Eventos Adversos Relaciona dos à	Lesão por pressão		
23/03/2023	CE	FORTALE ZA	HGF	LESÃO EM REGIÃO SACRA	Enviada	Eventos Adversos Relaciona dos à	Lesão por pressão		
23/03/2023	CE	FORTALE ZA	HGF	LESÃO EM REGIÃO SACRA	Enviada	Eventos Adversos Relaciona dos à	Lesão por pressão		
23/03/2023	CE	FORTALE ZA	HGF	LESÃO EM REGIÃO SACRA	Enviada	Eventos Adversos Relaciona dos à	Lesão por pressão		
23/03/2023	CE	FORTALE ZA	HGF	LESÃO EM REGIÃO GLÚTEA	Enviada	Eventos Adversos Relaciona dos à	Lesão por pressão		
23/03/2023	CE	FORTALE ZA	HGF	LESÃO EM REGIÃO SACRA	Enviada	Eventos Adversos Relaciona dos à	Lesão por pressão		
23/03/2023	CE	FORTALE ZA	HGF	LESÃO EM REGIÃO SACRA	Enviada	Eventos Adversos Relaciona dos à	Lesão por pressão		
23/03/2023	CE	FORTALE ZA	HGF	LESÃO EM REGIÃO SACRA	Enviada	Eventos Adversos Relaciona dos à	Lesão por pressão		
23/03/2023	CE	FORTALE ZA	HGF	LESÃO EM REGIÃO SACRA	Enviada	Eventos Adversos Relaciona dos à	Lesão por pressão		
23/03/2023	CE	FORTALE ZA	HGF	LESÃO EM REGIÃO SACRA	Enviada	Eventos Adversos Relaciona dos à	Lesão por pressão		
23/03/2023	CE	FORTALE ZA	HGF	LESÃO EM REGIÃO SACRA	Enviada	Eventos Adversos Relaciona dos à	Lesão por pressão		

23/03/2023	CE	FORTALEZA	HGF	LESÃO EM REGIÃO SACRA	Enviada	Eventos Adversos Relacionados à	Lesão por pressão		
23/03/2023	CE	FORTALEZA	HGF	LESÃO EM REGIÃO SACRA	Enviada	Eventos Adversos Relacionados à	Lesão por pressão		
23/03/2023	CE	FORTALEZA	HGF	LESÃO EM REGIÃO SACRA	Enviada	Eventos Adversos Relacionados à	Lesão por pressão		
23/03/2023	CE	FORTALEZA	HGF	LESÃO EM REGIÃO SACRA	Enviada	Eventos Adversos Relacionados à	Lesão por pressão		
23/03/2023	CE	FORTALEZA	HGF	LESÃO EM TROCANTER	Enviada	Eventos Adversos Relacionados à	Lesão por pressão		
23/03/2023	CE	FORTALEZA	HGF	LESÃO EM REGIÃO DORSAL	Enviada	Eventos Adversos Relacionados à	Lesão por pressão		
23/03/2023	CE	FORTALEZA	HGF	LESÃO EM REGIÃO SACRA	Enviada	Eventos Adversos Relacionados à	Lesão por pressão		
23/03/2023	CE	FORTALEZA	HGF	LESÃO EM REGIÃO SACRA	Enviada	Eventos Adversos Relacionados à	Lesão por pressão		
23/03/2023	CE	FORTALEZA	HGF	LESÃO EM REIÃO SACRA	Enviada	Eventos Adversos Relacionados à	Lesão por pressão		
23/03/2023	CE	FORTALEZA	HGF	LESÃO EM TROCANTER	Enviada	Eventos Adversos Relacionados à	Lesão por pressão		
23/03/2023	CE	FORTALEZA	HGF	LESÃO EM REGIÃO SACRA	Enviada	Eventos Adversos Relacionados à	Lesão por pressão		
23/03/2023	CE	FORTALEZA	HGF	LESÃO EM REGIÃO SACRA	Enviada	Eventos Adversos Relacionados à	Lesão por pressão		
23/03/2023	CE	FORTALEZA	HGF	LESÃO EM CLACÂNEO	Enviada	Eventos Adversos Relacionados à	Lesão por pressão		
23/03/2023	CE	FORTALEZA	HGF	LESÃO EM TROCANTER	Em retificação	Eventos Adversos Relacionados à	Lesão por pressão		
23/03/2023	CE	FORTALEZA	HGF	LESÃO EM TROCANTER	Enviada	Eventos Adversos Relacionados à	Lesão por pressão		

23/03/2023	CE	FORTALEZA	HGF	LESÃO EM REGIÃO INTEGLÚ	Enviada	Eventos Adversos Relacionados à	Lesão por pressão		
23/03/2023	CE	FORTALEZA	HGF	LESÃO EM REGIÃO GLÚTEA	Em retificação	Eventos Adversos Relacionados à	Lesão por pressão		
23/03/2023	CE	FORTALEZA	HGF	LESÃO EM REGIÃO INTERGI	Enviada	Eventos Adversos Relacionados à	Lesão por pressão		
23/03/2023	CE	FORTALEZA	HGF	LESÃO EM CALCÂNEO	Enviada	Eventos Adversos Relacionados à	Lesão por pressão		
23/03/2023	CE	FORTALEZA	HGF	LESÃO EM CLACÂNEO	Enviada	Eventos Adversos Relacionados à	Lesão por pressão		
23/03/2023	CE	FORTALEZA	HGF	LESÃO EM REGIÃO SACRA	Enviada	Eventos Adversos Relacionados à	Lesão por pressão		
23/03/2023	CE	FORTALEZA	HGF	LESÃO EM GLÚTEO DIREITO	Enviada	Eventos Adversos Relacionados à	Lesão por pressão		
23/03/2023	CE	FORTALEZA	HGF	LESÃO EM REGIÃO INTERGI	Enviada	Eventos Adversos Relacionados à	Lesão por pressão		
23/03/2023	CE	FORTALEZA	HGF	LESÃO EM REGIÃO SACRA	Enviada	Eventos Adversos Relacionados à	Lesão por pressão		
23/03/2023	CE	FORTALEZA	HGF	LESÃO EM REGIÃO INTERGI	Enviada	Eventos Adversos Relacionados à	Lesão por pressão		
23/03/2023	CE	FORTALEZA	HGF	LESÃO EM REGIÃO SACRA	Enviada	Eventos Adversos Relacionados à	Lesão por pressão		
23/03/2023	CE	FORTALEZA	HGF	LESÃO EM CALCÂNEO	Enviada	Eventos Adversos Relacionados à	Lesão por pressão		
23/03/2023	CE	FORTALEZA	HGF	LESÃO EM REGIÃO DORSAL	Enviada	Eventos Adversos Relacionados à	Lesão por pressão		
23/03/2023	CE	FORTALEZA	HGF	LESÃO EM REGIÃO SACRA	Enviada	Eventos Adversos Relacionados à	Lesão por pressão		
23/03/2023	CE	FORTALEZA	HGF	LESÃO EM TROCANTER	Enviada	Eventos Adversos Relacionados à	Lesão por pressão		

23/03/2023	CE	FORTALEZA	HGF	LESÃO EM REGIÃO SACRA	Enviada	Eventos Adversos Relacionados à	Lesão por pressão		
23/03/2023	CE	FORTALEZA	HGF	LESÃO EM CLACÂNEO	Enviada	Eventos Adversos Relacionados à	Lesão por pressão		
23/03/2023	CE	FORTALEZA	HGF	LESÃO EM REGIÃO SACRA	Enviada	Eventos Adversos Relacionados à	Lesão por pressão		
23/03/2023	CE	FORTALEZA	HGF	LESÃO EM REGIÃO SACRA	Enviada	Eventos Adversos Relacionados à	Lesão por pressão		
23/03/2023	CE	FORTALEZA	HGF	LESÃO EM GLÚTEO DIREITO	Enviada	Eventos Adversos Relacionados à	Lesão por pressão		
23/03/2023	CE	FORTALEZA	HGF	LESÃO EM REGIÃO SACRA	Enviada	Eventos Adversos Relacionados à	Lesão por pressão		
23/03/2023	CE	FORTALEZA	HGF	LESÃO EM REGIÃO SACRA	Enviada	Eventos Adversos Relacionados à	Lesão por pressão		
23/03/2023	CE	FORTALEZA	HGF	LESÃO EM REGIÃO SACRA	Enviada	Eventos Adversos Relacionados à	Lesão por pressão		
23/03/2023	CE	FORTALEZA	HGF	LESÃO EM REGIÃO SACRA	Enviada	Eventos Adversos Relacionados à	Lesão por pressão		
23/03/2023	CE	FORTALEZA	HGF	LESÃO EM REGIÃO SACRA	Enviada	Eventos Adversos Relacionados à	Lesão por pressão		
23/03/2023	CE	FORTALEZA	HGF	LESÃO EM REGIÃO SACRA	Enviada	Eventos Adversos Relacionados à	Lesão por pressão		
23/03/2023	CE	FORTALEZA	HGF	LESÃO EM REGIÃO SACRA	Enviada	Eventos Adversos Relacionados à	Lesão por pressão		
23/03/2023	CE	FORTALEZA	HGF	LESÃO EM REGIÃO SACRA	Enviada	Eventos Adversos Relacionados à	Lesão por pressão		
23/03/2023	CE	FORTALEZA	HGF	LESÃO EM REGIÃO SACRA	Concluída	Eventos Adversos Relacionados à	Lesão por pressão		
23/03/2023	CE	FORTALEZA	HGF	LESÃO EM REGIÃO SACRA	Enviada	Eventos Adversos Relacionados à	Lesão por pressão		

23/03/2023	CE	FORTALEZA	HGF	LESÃO EM REGIÃO SACRA	Enviada	Eventos Adversos Relacionados à	Lesão por pressão		
23/03/2023	CE	FORTALEZA	HGF	LESÃO EM REGIÃO INTERGL	Enviada	Eventos Adversos Relacionados à	Lesão por pressão		
23/03/2023	CE	FORTALEZA	HGF	LESÃO EM REGIÃO SACRA	Enviada	Eventos Adversos Relacionados à	Lesão por pressão		
23/03/2023	CE	FORTALEZA	HGF	LESÃO EM CALCÂNEOS	Enviada	Eventos Adversos Relacionados à	Lesão por pressão		
23/03/2023	CE	FORTALEZA	HGF	LESÃO EM REGIÃO SACRA	Enviada	Eventos Adversos Relacionados à	Lesão por pressão		
23/03/2023	CE	FORTALEZA	HGF	LESÃO EM REGIÃO SACRA	Enviada	Eventos Adversos Relacionados à	Lesão por pressão		
23/03/2023	CE	FORTALEZA	HGF	LESÃO EM REGIÃO SACRA	Enviada	Eventos Adversos Relacionados à	Lesão por pressão		
23/03/2023	CE	FORTALEZA	HGF	LESÃO EM REGIÃO SACRA	Enviada	Eventos Adversos Relacionados à	Lesão por pressão		
23/03/2023	CE	FORTALEZA	HGF	LESÃO EM REGIÃO SACRA	Enviada	Eventos Adversos Relacionados à	Lesão por pressão		
27/03/2023	CE	FORTALEZA	HGF	INTERGLÚTEA	Enviada	Eventos Adversos Relacionados à	Lesão por pressão		
27/03/2023	CE	FORTALEZA	HGF	SACRA	Enviada	Eventos Adversos Relacionados à	Lesão por pressão		
27/03/2023	CE	FORTALEZA	HGF	-	Enviada	Eventos Adversos Relacionados à	Falhas envolvendo cateter venoso		
27/03/2023	CE	FORTALEZA	HGF	SACRA	Enviada	Eventos Adversos Relacionados à	Lesão por pressão		
27/03/2023	CE	FORTALEZA	HGF	-	Enviada	Eventos Adversos Relacionados à	Queda do paciente		
27/03/2023	CE	FORTALEZA	HGF	DIETA ENTERAL ADMINIS	Enviada	Eventos Adversos Relacionados à	Falhas na administração de dietas		Enteral (oral, cateter oral ou

27/03/2023	CE	FORTALEZA	HGF	ÍLIO ESQUERDO	Enviada	Eventos Adversos Relacionados à	Lesão por pressão		
27/03/2023	CE	FORTALEZA	HGF	PACIENTE EVOLUIU BEM	Enviada	Eventos Adversos Relacionados à	Extubaçao endotraqueal		
27/03/2023	CE	FORTALEZA	HGF	PACIENTE COLOCADO NO	Enviada	Eventos Adversos Relacionados à	Queda do paciente		
27/03/2023	CE	FORTALEZA	HGF	PACIENTE EM DESMAME DE	Enviada	Eventos Adversos Relacionados à	Extubaçao endotraqueal		
27/03/2023	CE	FORTALEZA	HGF	paciente apresentando dermatite	Enviada	Eventos Adversos Relacionados à	Falhas durante a assistência à saúde		
27/03/2023	CE	FORTALEZA	HGF	SACRA	Enviada	Eventos Adversos Relacionados à	Lesão por pressão		
27/03/2023	CE	FORTALEZA	HGF	MSE	Enviada	Eventos Adversos Relacionados à	Falhas envolvendo o cateter venoso		
27/03/2023	CE	FORTALEZA	HGF	AO LEVANTAR-SE E TENTAR	Enviada	Eventos Adversos Relacionados à	Queda do paciente		
27/03/2023	CE	FORTALEZA	HGF	-	Enviada	Eventos Adversos Relacionados à	Queda do paciente		
27/03/2023	CE	FORTALEZA	HGF	MSE	Enviada	Eventos Adversos Relacionados à	Falhas envolvendo o cateter venoso		
27/03/2023	CE	FORTALEZA	HGF	-	Enviada	Eventos Adversos Relacionados à	Falhas envolvendo o cateter venoso		
28/03/2023	CE	FORTALEZA	HGF	Flebite em MSD	Enviada	Eventos Adversos Relacionados à	Falhas envolvendo o cateter venoso		
28/03/2023	CE	FORTALEZA	HGF	-	Enviada	Eventos Adversos Relacionados à	Queda do paciente		
30/03/2023	CE	FORTALEZA	HGF	PACIENTE EM USO DE UMIDIFIC	Concluída	Eventos Adversos Relacionados à	Falhas durante a assistência à saúde		
30/03/2023	CE	FORTALEZA	HGF	PACIENTE APRESENTANDO	Enviada	Eventos Adversos Relacionados à	Falhas envolvendo o sondas		

30/03/2023	CE	FORTALEZA	HGF	O NÚMERO DO PRONTU	Enviada	Eventos Adversos Relacionados à	Falha na identificação do paciente		
13/06/2022	CE	FORTALEZA	HOSPITAL GERAL DR. WALDEM	queda	Enviada	Eventos Adversos Relacionados à	Falhas durante a assistência à saúde		
14/06/2022	CE	FORTALEZA	HOSPITAL GERAL DR. WALDEM	Perda de sonda	Enviada	Eventos Adversos Relacionados à	Falhas envolvendo sondas		
14/06/2022	CE	FORTALEZA	HOSPITAL GERAL DR. WALDEM	Perda de sonda	Enviada	Eventos Adversos Relacionados à	Falhas envolvendo sondas		
14/06/2022	CE	FORTALEZA	HOSPITAL GERAL DR. WALDEM	Perda de acesso periférico	Enviada	Eventos Adversos Relacionados à	Falhas envolvendo cateter venoso		
14/06/2022	CE	FORTALEZA	HOSPITAL GERAL DR. WALDEM	Perda de sonda	Enviada	Eventos Adversos Relacionados à	Falhas envolvendo sondas		
14/06/2022	CE	FORTALEZA	HOSPITAL GERAL DR. WALDEM	Perda de sonda	Enviada	Eventos Adversos Relacionados à	Falhas envolvendo sondas		
14/06/2022	CE	FORTALEZA	HOSPITAL GERAL DR. WALDEM	Perda de sonda	Enviada	Eventos Adversos Relacionados à	Falhas envolvendo sondas		
14/06/2022	CE	FORTALEZA	HOSPITAL GERAL DR. WALDEM	Perda de PICC	Enviada	Eventos Adversos Relacionados à	Falhas envolvendo cateter venoso		
14/06/2022	CE	FORTALEZA	HOSPITAL GERAL DR. WALDEM	-	Enviada	Eventos Adversos Relacionados à	Extubação endotraqueal		
14/06/2022	CE	FORTALEZA	HOSPITAL GERAL DR. WALDEM	Lesão por pressão	Enviada	Eventos Adversos Relacionados à	Lesão por pressão		
14/06/2022	CE	FORTALEZA	HOSPITAL GERAL DR. WALDEM	Lesão por pressão	Enviada	Eventos Adversos Relacionados à	Lesão por pressão		
14/06/2022	CE	FORTALEZA	HOSPITAL GERAL DR. WALDEM	Lesão por pressão	Enviada	Eventos Adversos Relacionados à	Lesão por pressão		
14/06/2022	CE	FORTALEZA	HOSPITAL GERAL DR. WALDEM	-	Enviada	Eventos Adversos Relacionados à	Lesão por pressão		
14/06/2022	CE	FORTALEZA	HOSPITAL GERAL DR. WALDEM	Queda da própria altura, paciente	Enviada	Eventos Adversos Relacionados à	Queda do paciente		
14/06/2022	CE	FORTALEZA	HOSPITAL GERAL DR. WALDEM	Perda de sonda	Enviada	Eventos Adversos Relacionados à	Falhas envolvendo sondas		

14/06/2022	CE	FORTALEZA	HOSPITAL GERAL DR. WALDEM	Flebite	Enviada	Eventos Adversos Relacionados à	Falhas durante a assistência à saúde		
14/06/2022	CE	FORTALEZA	HOSPITAL GERAL DR. WALDEM	Lesão por pressão	Enviada	Eventos Adversos Relacionados à	Lesão por pressão		
14/06/2022	CE	FORTALEZA	HOSPITAL GERAL DR. WALDEM	Perda de PICC	Enviada	Eventos Adversos Relacionados à	Falhas envolvendo sondas		
14/06/2022	CE	FORTALEZA	HOSPITAL GERAL DR. WALDEM	Perda de sonda	Enviada	Eventos Adversos Relacionados à	Falhas envolvendo sondas		
14/06/2022	CE	FORTALEZA	HOSPITAL GERAL DR. WALDEM	Queda da própria altura, paciente	Enviada	Eventos Adversos Relacionados à	Queda do paciente		
14/06/2022	CE	FORTALEZA	HOSPITAL GERAL DR. WALDEM	Perda de SNG sem fixação	Enviada	Eventos Adversos Relacionados à	Falhas envolvendo sondas		
14/06/2022	CE	FORTALEZA	HOSPITAL GERAL DR. WALDEM	Perda de sonda	Enviada	Eventos Adversos Relacionados à	Falhas envolvendo sondas		
14/06/2022	CE	FORTALEZA	HOSPITAL GERAL DR. WALDEM	Saída não planejada de cânula	Enviada	Eventos Adversos Relacionados à	Falhas envolvendo sondas		
14/06/2022	CE	FORTALEZA	HOSPITAL GERAL DR. WALDEM	Perda de sonda por obstrução	Enviada	Eventos Adversos Relacionados à	Falhas envolvendo sondas		
14/06/2022	CE	FORTALEZA	HOSPITAL GERAL DR. WALDEM	Perda de sonda por obstrução	Enviada	Eventos Adversos Relacionados à	Falhas envolvendo sondas		
14/06/2022	CE	FORTALEZA	HOSPITAL GERAL DR. WALDEM	Lesão por pressão por	Enviada	Eventos Adversos Relacionados à	Lesão por pressão		
14/06/2022	CE	FORTALEZA	HOSPITAL GERAL DR. WALDEM	Perda de PICC	Enviada	Eventos Adversos Relacionados à	Falhas envolvendo sondas		
14/06/2022	CE	FORTALEZA	HOSPITAL GERAL DR. WALDEM	Perda de acesso central	Enviada	Eventos Adversos Relacionados à	Falhas envolvendo sondas		
14/06/2022	CE	FORTALEZA	HOSPITAL GERAL DR. WALDEM	Queda de cadeira	Enviada	Eventos Adversos Relacionados à	Queda do paciente		
14/06/2022	CE	FORTALEZA	HOSPITAL GERAL DR. WALDEM	Lesão por pressão	Enviada	Eventos Adversos Relacionados à	Lesão por pressão		
14/06/2022	CE	FORTALEZA	HOSPITAL GERAL DR. WALDEM	Perda de sonda por obstrução	Enviada	Eventos Adversos Relacionados à	Falhas envolvendo sondas		

14/06/2022	CE	FORTALEZA	HOSPITAL GERAL DR. WALDEM	Perda de sonda	Enviada	Eventos Adversos Relacionados à	Falhas envolvendo sondas		
14/06/2022	CE	FORTALEZA	HOSPITAL GERAL DR. WALDEM	Saída não planejada de cânula	Enviada	Eventos Adversos Relacionados à	Falhas envolvendo sondas		
14/06/2022	CE	FORTALEZA	HOSPITAL GERAL DR. WALDEM	Perda de sonda, paciente desorienta	Enviada	Eventos Adversos Relacionados à	Falhas envolvendo sondas		
14/06/2022	CE	FORTALEZA	HOSPITAL GERAL DR. WALDEM	Saída não planejada do	Enviada	Eventos Adversos Relacionados à	Falhas envolvendo sondas		
14/06/2022	CE	FORTALEZA	HOSPITAL GERAL DR. WALDEM	Perda de sonda, paciente agitado e	Enviada	Eventos Adversos Relacionados à	Falhas envolvendo sondas		
14/06/2022	CE	FORTALEZA	HOSPITAL GERAL DR. WALDEM	Perda de sonda, paciente agitado	Enviada	Eventos Adversos Relacionados à	Falhas envolvendo sondas		
14/06/2022	CE	FORTALEZA	HOSPITAL GERAL DR. WALDEM	Perda de sonda por obstrução	Enviada	Eventos Adversos Relacionados à	Falhas envolvendo sondas		
14/06/2022	CE	FORTALEZA	HOSPITAL GERAL DR. WALDEM	Flebite	Enviada	Eventos Adversos Relacionados à	Falhas envolvendo cateter venoso		
14/06/2022	CE	FORTALEZA	HOSPITAL GERAL DR. WALDEM	Perda de sonda	Enviada	Eventos Adversos Relacionados à	Falhas envolvendo sondas		
14/06/2022	CE	FORTALEZA	HOSPITAL GERAL DR. WALDEM	Saída não planejada de cânula	Enviada	Eventos Adversos Relacionados à	Falhas envolvendo sondas		
14/06/2022	CE	FORTALEZA	HOSPITAL GERAL DR. WALDEM	Flebite	Enviada	Eventos Adversos Relacionados à	Falhas envolvendo cateter venoso		
14/06/2022	CE	FORTALEZA	HOSPITAL GERAL DR. WALDEM	Queda da própria altura	Enviada	Eventos Adversos Relacionados à	Queda do paciente		
14/06/2022	CE	FORTALEZA	HOSPITAL GERAL DR. WALDEM	Perda de sonda, paciente agitado	Enviada	Eventos Adversos Relacionados à	Falhas envolvendo sondas		
14/06/2022	CE	FORTALEZA	HOSPITAL GERAL DR. WALDEM	Perda de sonda por obstrução	Enviada	Eventos Adversos Relacionados à	Falhas envolvendo sondas		
14/06/2022	CE	FORTALEZA	HOSPITAL GERAL DR. WALDEM	Perda de sonda, paciente agitado	Enviada	Eventos Adversos Relacionados à	Falhas envolvendo sondas		
14/06/2022	CE	FORTALEZA	HOSPITAL GERAL DR. WALDEM	Flebite em PICC	Enviada	Eventos Adversos Relacionados à	Falhas envolvendo cateter venoso		

14/06/2022	CE	FORTALEZA	HOSPITAL GERAL DR. WALDEM	Lesão por pressão	Enviada	Eventos Adversos Relacionados à	Lesão por pressão		
14/06/2022	CE	FORTALEZA	HOSPITAL GERAL DR. WALDEM	Queda da própria altura, paciente	Enviada	Eventos Adversos Relacionados à	Queda do paciente		
14/06/2022	CE	FORTALEZA	HOSPITAL GERAL DR. WALDEM	Perda de sonda, paciente sacou	Enviada	Eventos Adversos Relacionados à	Falhas envolvendo sondas		
14/06/2022	CE	FORTALEZA	HOSPITAL GERAL DR. WALDEM	Perda de sonda, paciente agitado	Enviada	Eventos Adversos Relacionados à	Falhas envolvendo sondas		
14/06/2022	CE	FORTALEZA	HOSPITAL GERAL DR. WALDEM	Queda	Enviada	Eventos Adversos Relacionados à	Queda do paciente		
14/06/2022	CE	FORTALEZA	HOSPITAL GERAL DR. WALDEM	Lesão por pressão	Enviada	Eventos Adversos Relacionados à	Lesão por pressão		
14/06/2022	CE	FORTALEZA	HOSPITAL GERAL DR. WALDEM	Lesão por pressão	Enviada	Eventos Adversos Relacionados à	Lesão por pressão		
14/06/2022	CE	FORTALEZA	HOSPITAL GERAL DR. WALDEM	Perda de acesso venoso central	Enviada	Eventos Adversos Relacionados à	Falhas envolvendo sondas		
14/06/2022	CE	FORTALEZA	HOSPITAL GERAL DR. WALDEM	Saída não planejada de cânula	Enviada	Eventos Adversos Relacionados à	Falhas envolvendo sondas		
14/06/2022	CE	FORTALEZA	HOSPITAL GERAL DR. WALDEM	Queda	Enviada	Eventos Adversos Relacionados à	Queda do paciente		
14/06/2022	CE	FORTALEZA	HOSPITAL GERAL DR. WALDEM	Perda de sonda por obstrução	Enviada	Eventos Adversos Relacionados à	Falhas envolvendo sondas		
15/06/2022	CE	FORTALEZA	HOSPITAL GERAL DR. WALDEM	Perda de sonda, paciente agitado	Enviada	Eventos Adversos Relacionados à	Falhas envolvendo sondas		
15/06/2022	CE	FORTALEZA	HOSPITAL GERAL DR. WALDEM	Perda de sonda, paciente agitado	Enviada	Eventos Adversos Relacionados à	Falhas envolvendo sondas		
15/06/2022	CE	FORTALEZA	HOSPITAL GERAL DR. WALDEM	Perda de sonda, paciente agitado	Enviada	Eventos Adversos Relacionados à	Falhas envolvendo sondas		
15/06/2022	CE	FORTALEZA	HOSPITAL GERAL DR. WALDEM	Lesão por pressão	Enviada	Eventos Adversos Relacionados à	Lesão por pressão		
15/06/2022	CE	FORTALEZA	HOSPITAL GERAL DR. WALDEM	Queda do leito	Enviada	Eventos Adversos Relacionados à	Queda do paciente		

15/06/2022	CE	FORTALEZA	HOSPITAL GERAL DR. WALDEM	Queda da própria altura	Enviada	Eventos Adversos Relacionados à	Queda do paciente		
15/06/2022	CE	FORTALEZA	HOSPITAL GERAL DR. WALDEM	Perda de sonda, paciente agitado	Enviada	Eventos Adversos Relacionados à	Falhas envolvendo sondas		
15/06/2022	CE	FORTALEZA	HOSPITAL GERAL DR. WALDEM	Perda de sonda por obstrução	Enviada	Eventos Adversos Relacionados à	Falhas envolvendo sondas		
15/06/2022	CE	FORTALEZA	HOSPITAL GERAL DR. WALDEM	Perda de sonda, paciente sacou	Enviada	Eventos Adversos Relacionados à	Falhas envolvendo sondas		
17/06/2022	CE	FORTALEZA	HOSPITAL GERAL DR. WALDEM	-	Enviada	Eventos Adversos Relacionados à	Falhas envolvendo cateter venoso		
17/06/2022	CE	FORTALEZA	HOSPITAL GERAL DR. WALDEM	Retirada equivocada do catéter	Enviada	Eventos Adversos Relacionados à	Falhas envolvendo sondas		
17/06/2022	CE	FORTALEZA	HOSPITAL GERAL DR. WALDEM	Perda de PICC	Enviada	Eventos Adversos Relacionados à	Falhas envolvendo sondas		
17/06/2022	CE	FORTALEZA	HOSPITAL GERAL DR. WALDEM	Perda de PICC	Enviada	Eventos Adversos Relacionados à	Falhas envolvendo sondas		
17/06/2022	CE	FORTALEZA	HOSPITAL GERAL DR. WALDEM	Perda de sonda durante ida ao	Enviada	Eventos Adversos Relacionados à	Falhas envolvendo sondas		
17/06/2022	CE	FORTALEZA	HOSPITAL GERAL DR. WALDEM	Queda do paciente de berço com	Enviada	Eventos Adversos Relacionados à	Queda do paciente		
17/06/2022	CE	FORTALEZA	HOSPITAL GERAL DR. WALDEM	Flebite em MSE	Enviada	Eventos Adversos Relacionados à	Falhas envolvendo cateter venoso		
17/06/2022	CE	FORTALEZA	HOSPITAL GERAL DR. WALDEM	Queda da própria altura	Enviada	Eventos Adversos Relacionados à	Queda do paciente		
17/06/2022	CE	FORTALEZA	HOSPITAL GERAL DR. WALDEM	Paciente sem SOG	Enviada	Eventos Adversos Relacionados à	Falhas envolvendo sondas		
17/06/2022	CE	FORTALEZA	HOSPITAL GERAL DR. WALDEM	Paciente sem SOG	Enviada	Eventos Adversos Relacionados à	Falhas envolvendo sondas		
17/06/2022	CE	FORTALEZA	HOSPITAL GERAL DR. WALDEM	RN agitado	Enviada	Eventos Adversos Relacionados à	Falhas envolvendo sondas		
20/06/2022	CE	FORTALEZA	HOSPITAL GERAL DR. WALDEM	69232 lesão por pressão	Enviada	Eventos Adversos Relacionados à	Lesão por pressão		

20/06/2022	CE	FORTALEZA	HOSPITAL GERAL DR. WALDEM	69238 lesão por pressão	Enviada	Eventos Adversos Relacionados à	Lesão por pressão		
20/06/2022	CE	FORTALEZA	HOSPITAL GERAL DR. WALDEM	69353 lesão de pele por alergia à	Enviada	Eventos Adversos Relacionados à	Lesão por pressão		
20/06/2022	CE	FORTALEZA	HOSPITAL GERAL DR. WALDEM	69472 flebite em msd	Enviada	Eventos Adversos Relacionados à	Falhas envolvendo o cateter venoso		
20/06/2022	CE	FORTALEZA	HOSPITAL GERAL DR. WALDEM	69233 LPP interglúteo	Enviada	Eventos Adversos Relacionados à	Lesão por pressão		
20/06/2022	CE	FORTALEZA	HOSPITAL GERAL DR. WALDEM	69235 LPP sacra	Enviada	Eventos Adversos Relacionados à	Lesão por pressão		
20/06/2022	CE	FORTALEZA	HOSPITAL GERAL DR. WALDEM	69786 lesão por pressão mse	Enviada	Eventos Adversos Relacionados à	Lesão por pressão		
20/06/2022	CE	FORTALEZA	HOSPITAL GERAL DR. WALDEM	69592 saída não planejada de cânula	Enviada	Eventos Adversos Relacionados à	Falhas envolvendo sondas		
20/06/2022	CE	FORTALEZA	HOSPITAL GERAL DR. WALDEM	69979 lpp sacra	Enviada	Eventos Adversos Relacionados à	Lesão por pressão		
20/06/2022	CE	FORTALEZA	HOSPITAL GERAL DR. WALDEM	69641 lpp sacra	Enviada	Eventos Adversos Relacionados à	Lesão por pressão		
20/06/2022	CE	FORTALEZA	HOSPITAL GERAL DR. WALDEM	69677 não realização da troca	Enviada	Eventos Adversos Relacionados à	Falhas durante a assistência à saúde		
20/06/2022	CE	FORTALEZA	HOSPITAL GERAL DR. WALDEM	69683 pct mesmo com contenção	Enviada	Eventos Adversos Relacionados à	Falhas envolvendo sondas		
20/06/2022	CE	FORTALEZA	HOSPITAL GERAL DR. WALDEM	69684 sne obstruiu	Enviada	Eventos Adversos Relacionados à	Falhas envolvendo sondas		
20/06/2022	CE	FORTALEZA	HOSPITAL GERAL DR. WALDEM	69241	Enviada	Eventos Adversos Relacionados à	Lesão por pressão		
20/06/2022	CE	FORTALEZA	HOSPITAL GERAL DR. WALDEM	68025 saída não planejada de cânula	Enviada	Eventos Adversos Relacionados à	Falhas envolvendo sondas		
20/06/2022	CE	FORTALEZA	HOSPITAL GERAL DR. WALDEM	68026 perda de picc	Enviada	Eventos Adversos Relacionados à	Falhas envolvendo sondas		
20/06/2022	CE	FORTALEZA	HOSPITAL GERAL DR. WALDEM	68054 saída não planejada do	Enviada	Eventos Adversos Relacionados à	Falhas envolvendo sondas		

20/06/2022	CE	FORTALEZA	HOSPITAL GERAL DR. WALDEM	68055 perda de sonda por	Enviada	Eventos Adversos Relacionados à	Falhas envolvendo sondas		
20/06/2022	CE	FORTALEZA	HOSPITAL GERAL DR. WALDEM	68084 flebite grau 2	Enviada	Eventos Adversos Relacionados à	Falhas envolvendo cateter venoso		
20/06/2022	CE	FORTALEZA	HOSPITAL GERAL DR. WALDEM	68146 deitado na cama sofreu	Enviada	Eventos Adversos Relacionados à	Queda do paciente		
20/06/2022	CE	FORTALEZA	HOSPITAL GERAL DR. WALDEM	68193 flebite	Enviada	Eventos Adversos Relacionados à	Falhas envolvendo cateter venoso		
20/06/2022	CE	FORTALEZA	HOSPITAL GERAL DR. WALDEM	68229 lpp sacra, trocanter e	Enviada	Eventos Adversos Relacionados à	Lesão por pressão		
20/06/2022	CE	FORTALEZA	HOSPITAL GERAL DR. WALDEM	68298 lpp em mmii	Enviada	Eventos Adversos Relacionados à	Lesão por pressão		
20/06/2022	CE	FORTALEZA	HOSPITAL GERAL DR. WALDEM	68511 sonda rasgada	Enviada	Eventos Adversos Relacionados à	Falhas envolvendo sondas		
20/06/2022	CE	FORTALEZA	HOSPITAL GERAL DR. WALDEM	68512 sonda rasgada	Enviada	Eventos Adversos Relacionados à	Falhas envolvendo sondas		
20/06/2022	CE	FORTALEZA	HOSPITAL GERAL DR. WALDEM	68513 sonda rasgada	Enviada	Eventos Adversos Relacionados à	Falhas envolvendo sondas		
20/06/2022	CE	FORTALEZA	HOSPITAL GERAL DR. WALDEM	68605 sonda rasgada	Enviada	Eventos Adversos Relacionados à	Falhas envolvendo sondas		
20/06/2022	CE	FORTALEZA	HOSPITAL GERAL DR. WALDEM	68667 perda de picc	Enviada	Eventos Adversos Relacionados à	Falhas envolvendo sondas		
21/06/2022	CE	FORTALEZA	HOSPITAL GERAL DR. WALDEM	68681 flebite em msd	Enviada	Eventos Adversos Relacionados à	Falhas envolvendo cateter venoso		
21/06/2022	CE	FORTALEZA	HOSPITAL GERAL DR. WALDEM	68682 sacou sne	Enviada	Eventos Adversos Relacionados à	Falhas envolvendo sondas		
21/06/2022	CE	FORTALEZA	HOSPITAL GERAL DR. WALDEM	68852 queda no banheiro ao	Enviada	Eventos Adversos Relacionados à	Queda do paciente		
21/06/2022	CE	FORTALEZA	HOSPITAL GERAL DR. WALDEM	68945 pct restrito ao leito com vício	Enviada	Eventos Adversos Relacionados à	Lesão por pressão		
21/06/2022	CE	FORTALEZA	HOSPITAL GERAL DR. WALDEM	68949 troca de sne por mal uso	Enviada	Eventos Adversos Relacionados à	Falhas envolvendo sondas		

22/06/2022	CE	FORTALEZA	HOSPITAL GERAL DR. WALDEM	67064 saída não planejada de cânula	Enviada	Eventos Adversos Relacionados à	Falhas envolvendo sondas		
22/06/2022	CE	FORTALEZA	HOSPITAL GERAL DR. WALDEM	67123 criança caiu do berço	Enviada	Eventos Adversos Relacionados à	Queda do paciente		
22/06/2022	CE	FORTALEZA	HOSPITAL GERAL DR. WALDEM	67250 flebite medicamentos	Enviada	Eventos Adversos Relacionados à	Falhas envolvendo cateter venoso		
22/06/2022	CE	FORTALEZA	HOSPITAL GERAL DR. WALDEM	67273 paciente idoso arrancou	Enviada	Eventos Adversos Relacionados à	Falhas envolvendo sondas		
22/06/2022	CE	FORTALEZA	HOSPITAL GERAL DR. WALDEM	67322 pct removeu sne mesmo	Enviada	Eventos Adversos Relacionados à	Falhas envolvendo sondas		
22/06/2022	CE	FORTALEZA	HOSPITAL GERAL DR. WALDEM	67406 perda de sne por fixação	Enviada	Eventos Adversos Relacionados à	Falhas envolvendo sondas		
22/06/2022	CE	FORTALEZA	HOSPITAL GERAL DR. WALDEM	67407 perda de sne por fixação	Enviada	Eventos Adversos Relacionados à	Falhas envolvendo sondas		
22/06/2022	CE	FORTALEZA	HOSPITAL GERAL DR. WALDEM	67446 queda da cama	Enviada	Eventos Adversos Relacionados à	Queda do paciente		
22/06/2022	CE	FORTALEZA	HOSPITAL GERAL DR. WALDEM	67450 queda do pct que tentou	Enviada	Eventos Adversos Relacionados à	Queda do paciente		
22/06/2022	CE	FORTALEZA	HOSPITAL GERAL DR. WALDEM	67497 perda de sne por pct	Enviada	Eventos Adversos Relacionados à	Falhas envolvendo sondas		
22/06/2022	CE	FORTALEZA	HOSPITAL GERAL DR. WALDEM	67573 lpp sacra	Enviada	Eventos Adversos Relacionados à	Lesão por pressão		
22/06/2022	CE	FORTALEZA	HOSPITAL GERAL DR. WALDEM	67692 furo em cateter impedindo	Enviada	Eventos Adversos Relacionados à	Falhas envolvendo cateter venoso		
22/06/2022	CE	FORTALEZA	HOSPITAL GERAL DR. WALDEM	67734 perda cvc por manuseio	Enviada	Eventos Adversos Relacionados à	Falhas envolvendo cateter venoso		
22/06/2022	CE	FORTALEZA	HOSPITAL GERAL DR. WALDEM	67740 pct sacou sne	Enviada	Eventos Adversos Relacionados à	Falhas envolvendo sondas		
22/06/2022	CE	FORTALEZA	HOSPITAL GERAL DR. WALDEM	67753 perda de sne por obstrução	Enviada	Eventos Adversos Relacionados à	Falhas envolvendo sondas		
22/06/2022	CE	FORTALEZA	HOSPITAL GERAL DR. WALDEM	67888 perda de picc	Enviada	Eventos Adversos Relacionados à	Falhas envolvendo sondas		

22/06/2022	CE	FORTALEZA	HOSPITAL GERAL DR. WALDEM	65897 pct caiu do leito	Enviada	Eventos Adversos Relacionados à	Queda do paciente		
22/06/2022	CE	FORTALEZA	HOSPITAL GERAL DR. WALDEM	65911 pct sacou sne	Enviada	Eventos Adversos Relacionados à	Falhas envolvendo sondas		
22/06/2022	CE	FORTALEZA	HOSPITAL GERAL DR. WALDEM	65937lpp mie	Enviada	Eventos Adversos Relacionados à	Lesão por pressão		
22/06/2022	CE	FORTALEZA	HOSPITAL GERAL DR. WALDEM	66039 lesão pelo oxímetro	Enviada	Eventos Adversos Relacionados à	Lesão por pressão		
22/06/2022	CE	FORTALEZA	HOSPITAL GERAL DR. WALDEM	66083 sne obstruída	Enviada	Eventos Adversos Relacionados à	Falhas envolvendo sondas		
29/06/2022	CE	FORTALEZA	HOSPITAL GERAL DR. WALDEM	66198 lpp sacra, pct idosa, magra e	Enviada	Eventos Adversos Relacionados à	Lesão por pressão		
29/06/2022	CE	FORTALEZA	HOSPITAL GERAL DR. WALDEM	66266 queda ao calçar a sandália	Enviada	Eventos Adversos Relacionados à	Queda do paciente		
29/06/2022	CE	FORTALEZA	HOSPITAL GERAL DR. WALDEM	66296 queda da própria altura e	Enviada	Eventos Adversos Relacionados à	Queda do paciente		
29/06/2022	CE	FORTALEZA	HOSPITAL GERAL DR. WALDEM	66297 flebite em avp em msd	Enviada	Eventos Adversos Relacionados à	Falhas envolvendo cateter venoso		
29/06/2022	CE	FORTALEZA	HOSPITAL GERAL DR. WALDEM	66301 pct retirou sne de forma	Enviada	Eventos Adversos Relacionados à	Falhas envolvendo sondas		
29/06/2022	CE	FORTALEZA	HOSPITAL GERAL DR. WALDEM	66302 paciente sacou sne	Enviada	Eventos Adversos Relacionados à	Falhas envolvendo sondas		
29/06/2022	CE	FORTALEZA	HOSPITAL GERAL DR. WALDEM	66370 pct sacou sne	Enviada	Eventos Adversos Relacionados à	Falhas envolvendo sondas		
29/06/2022	CE	FORTALEZA	HOSPITAL GERAL DR. WALDEM	66402 pct sacou sonda	Enviada	Eventos Adversos Relacionados à	Falhas envolvendo sondas		
29/06/2022	CE	FORTALEZA	HOSPITAL GERAL DR. WALDEM	66403 flebite em msd	Enviada	Eventos Adversos Relacionados à	Falhas envolvendo cateter venoso		
29/06/2022	CE	FORTALEZA	HOSPITAL GERAL DR. WALDEM	66404 perda de sne	Enviada	Eventos Adversos Relacionados à	Falhas envolvendo sondas		
29/06/2022	CE	FORTALEZA	HOSPITAL GERAL DR. WALDEM	66429 queda do leito	Enviada	Eventos Adversos Relacionados à	Queda do paciente		

29/06/2022	CE	FORTALEZA	HOSPITAL GERAL DR. WALDEM	66457 perda de tqt	Enviada	Eventos Adversos Relacionados à	Falhas envolvendo sondas		
29/06/2022	CE	FORTALEZA	HOSPITAL GERAL DR. WALDEM	66473 pct sacou sne	Enviada	Eventos Adversos Relacionados à	Falhas envolvendo sondas		
29/06/2022	CE	FORTALEZA	HOSPITAL GERAL DR. WALDEM	66490 perda de picc	Enviada	Eventos Adversos Relacionados à	Falhas envolvendo sondas		
29/06/2022	CE	FORTALEZA	HOSPITAL GERAL DR. WALDEM	66522 lpp eritema mie	Enviada	Eventos Adversos Relacionados à	Lesão por pressão		
29/06/2022	CE	FORTALEZA	HOSPITAL GERAL DR. WALDEM	66524 lpp em mie	Enviada	Eventos Adversos Relacionados à	Lesão por pressão		
29/06/2022	CE	FORTALEZA	HOSPITAL GERAL DR. WALDEM	66536 perda de cateter umbilical	Enviada	Eventos Adversos Relacionados à	Falhas envolvendo sondas		
30/06/2022	CE	FORTALEZA	HOSPITAL GERAL DR. WALDEM	66628 pct sacou sne	Enviada	Eventos Adversos Relacionados à	Falhas envolvendo sondas		
30/06/2022	CE	FORTALEZA	HOSPITAL GERAL DR. WALDEM	66639 lpp sacral	Enviada	Eventos Adversos Relacionados à	Lesão por pressão		
30/06/2022	CE	FORTALEZA	HOSPITAL GERAL DR. WALDEM	66743 picc tracionado durante	Enviada	Eventos Adversos Relacionados à	Falhas envolvendo sondas		
30/06/2022	CE	FORTALEZA	HOSPITAL GERAL DR. WALDEM	66747 sonda vesical obstruída	Enviada	Eventos Adversos Relacionados à	Falhas envolvendo sondas		
30/06/2022	CE	FORTALEZA	HOSPITAL GERAL DR. WALDEM	66809 rn extubou-se acidentam	Enviada	Eventos Adversos Relacionados à	Falhas envolvendo sondas		
30/06/2022	CE	FORTALEZA	HOSPITAL GERAL DR. WALDEM	66810 perda de picc	Enviada	Eventos Adversos Relacionados à	Falhas envolvendo sondas		
30/06/2022	CE	FORTALEZA	HOSPITAL GERAL DR. WALDEM	66888 rn extubou-se acidental	Enviada	Eventos Adversos Relacionados à	Falhas envolvendo sondas		
30/06/2022	CE	FORTALEZA	HOSPITAL GERAL DR. WALDEM	66927 perda acidental de sne	Enviada	Eventos Adversos Relacionados à	Falhas envolvendo sondas		
30/06/2022	CE	FORTALEZA	HOSPITAL GERAL DR. WALDEM	66942 pct sacou sne mesmo	Enviada	Eventos Adversos Relacionados à	Falhas envolvendo sondas		
30/06/2022	CE	FORTALEZA	HOSPITAL GERAL DR. WALDEM	64661 pct escorregou no banheiro	Enviada	Eventos Adversos Relacionados à	Queda do paciente		

30/06/2022	CE	FORTALEZA	HOSPITAL GERAL DR. WALDEM	64733 lesão por uso de oxímetro	Enviada	Eventos Adversos Relacionados à	Lesão por pressão		
30/06/2022	CE	FORTALEZA	HOSPITAL GERAL DR. WALDEM	64798 sne exteriorizada	Enviada	Eventos Adversos Relacionados à	Falhas envolvendo sondas		
30/06/2022	CE	FORTALEZA	HOSPITAL GERAL DR. WALDEM	64856 perda de picc	Enviada	Eventos Adversos Relacionados à	Falhas envolvendo sondas		
07/07/2022	CE	FORTALEZA	HOSPITAL GERAL DR. WALDEM	64876 - pct sacou sne mesmo	Enviada	Eventos Adversos Relacionados à	Falhas envolvendo sondas		
07/07/2022	CE	FORTALEZA	HOSPITAL GERAL DR. WALDEM	64933 - pct sacou sne	Enviada	Eventos Adversos Relacionados à	Falhas envolvendo sondas		
07/07/2022	CE	FORTALEZA	HOSPITAL GERAL DR. WALDEM	64947 - perda de picc	Enviada	Eventos Adversos Relacionados à	Falhas envolvendo sondas		
07/07/2022	CE	FORTALEZA	HOSPITAL GERAL DR. WALDEM	64959 - perda de avp durante	Enviada	Eventos Adversos Relacionados à	Falhas envolvendo sondas		
07/07/2022	CE	FORTALEZA	HOSPITAL GERAL DR. WALDEM	64960 - lpp sacral	Enviada	Eventos Adversos Relacionados à	Lesão por pressão		
07/07/2022	CE	FORTALEZA	HOSPITAL GERAL DR. WALDEM	64966 - perda de acesso central	Enviada	Eventos Adversos Relacionados à	Falhas envolvendo sondas		
07/07/2022	CE	FORTALEZA	HOSPITAL GERAL DR. WALDEM	65014 - queda da escada de apoio	Enviada	Eventos Adversos Relacionados à	Queda do paciente		
07/07/2022	CE	FORTALEZA	HOSPITAL GERAL DR. WALDEM	65099 - pct sacou sne	Enviada	Eventos Adversos Relacionados à	Falhas envolvendo sondas		
07/07/2022	CE	FORTALEZA	HOSPITAL GERAL DR. WALDEM	65091 - queda	Enviada	Eventos Adversos Relacionados à	Queda do paciente		
07/07/2022	CE	FORTALEZA	HOSPITAL GERAL DR. WALDEM	65111 - perda de picc	Enviada	Eventos Adversos Relacionados à	Falhas envolvendo sondas		
07/07/2022	CE	FORTALEZA	HOSPITAL GERAL DR. WALDEM	65112 - perda de picc	Enviada	Eventos Adversos Relacionados à	Falhas envolvendo sondas		
07/07/2022	CE	FORTALEZA	HOSPITAL GERAL DR. WALDEM	65161 - pct psiquiátrica agitada	Enviada	Eventos Adversos Relacionados à	Queda do paciente		
07/07/2022	CE	FORTALEZA	HOSPITAL GERAL DR. WALDEM	65119 - lpp região glútea	Enviada	Eventos Adversos Relacionados à	Lesão por pressão		

07/07/2022	CE	FORTALEZA	HOSPITAL GERAL DR. WALDEM	65295 - sne esteriorizada	Enviada	Eventos Adversos Relacionados à	Falhas envolvendo sondas		
07/07/2022	CE	FORTALEZA	HOSPITAL GERAL DR. WALDEM	65337 - lpp em orelha direita	Enviada	Eventos Adversos Relacionados à	Lesão por pressão		
07/07/2022	CE	FORTALEZA	HOSPITAL GERAL DR. WALDEM	65513 - lpp sacra	Enviada	Eventos Adversos Relacionados à	Lesão por pressão		
07/07/2022	CE	FORTALEZA	HOSPITAL GERAL DR. WALDEM	65398 - flebite	Enviada	Eventos Adversos Relacionados à	Falhas envolvendo cateter venoso		
07/07/2022	CE	FORTALEZA	HOSPITAL GERAL DR. WALDEM	65397 - flebite	Enviada	Eventos Adversos Relacionados à	Falhas envolvendo cateter venoso		
11/07/2022	CE	FORTALEZA	HOSPITAL GERAL DR. WALDEM	63523 - perda de sng	Enviada	Eventos Adversos Relacionados à	Falhas envolvendo sondas		
11/07/2022	CE	FORTALEZA	HOSPITAL GERAL DR. WALDEM	63582 - flebite	Enviada	Eventos Adversos Relacionados à	Falhas envolvendo cateter venoso		
11/07/2022	CE	FORTALEZA	HOSPITAL GERAL DR. WALDEM	63583 - flebite	Enviada	Eventos Adversos Relacionados à	Falhas envolvendo cateter venoso		
11/07/2022	CE	FORTALEZA	HOSPITAL GERAL DR. WALDEM	63705 - remoção acidental de ott	Enviada	Eventos Adversos Relacionados à	Falhas envolvendo sondas		
11/07/2022	CE	FORTALEZA	HOSPITAL GERAL DR. WALDEM	63820 - pct desorientado	Enviada	Eventos Adversos Relacionados à	Queda do paciente		
11/07/2022	CE	FORTALEZA	HOSPITAL GERAL DR. WALDEM	64005 - lpp por uso prolongad	Enviada	Eventos Adversos Relacionados à	Lesão por pressão		
11/07/2022	CE	FORTALEZA	HOSPITAL GERAL DR. WALDEM	64186 - perda acidental de	Enviada	Eventos Adversos Relacionados à	Falhas envolvendo sondas		
11/07/2022	CE	FORTALEZA	HOSPITAL GERAL DR. WALDEM	64195 - pct sacou svd	Enviada	Eventos Adversos Relacionados à	Falhas envolvendo sondas		
11/07/2022	CE	FORTALEZA	HOSPITAL GERAL DR. WALDEM	64213 - flebite	Enviada	Eventos Adversos Relacionados à	Falhas envolvendo cateter venoso		
11/07/2022	CE	FORTALEZA	HOSPITAL GERAL DR. WALDEM	64214 - flebite	Enviada	Eventos Adversos Relacionados à	Falhas envolvendo cateter venoso		
11/07/2022	CE	FORTALEZA	HOSPITAL GERAL DR. WALDEM	64215 - lpp em região sacral	Enviada	Eventos Adversos Relacionados à	Lesão por pressão		

11/07/2022	CE	FORTALEZA	HOSPITAL GERAL DR. WALDEM	64461 - pct escorregou no	Enviada	Eventos Adversos Relacionados à	Queda do paciente		
11/07/2022	CE	FORTALEZA	HOSPITAL GERAL DR. WALDEM	64462 - pct escorregou no	Enviada	Eventos Adversos Relacionados à	Queda do paciente		
11/07/2022	CE	FORTALEZA	HOSPITAL GERAL DR. WALDEM	64556 - queda pct no banheiro	Enviada	Eventos Adversos Relacionados à	Queda do paciente		
11/07/2022	CE	FORTALEZA	HOSPITAL GERAL DR. WALDEM	64619 - pct sacou sonda	Enviada	Eventos Adversos Relacionados à	Falhas envolvendo sondas		
12/07/2022	CE	FORTALEZA	HOSPITAL GERAL DR. WALDEM	62404 - hiperemia em região	Enviada	Eventos Adversos Relacionados à	Lesão por pressão		
12/07/2022	CE	FORTALEZA	HOSPITAL GERAL DR. WALDEM	62406 - lpp infraglútea estágio I	Enviada	Eventos Adversos Relacionados à	Lesão por pressão		
12/07/2022	CE	FORTALEZA	HOSPITAL GERAL DR. WALDEM	62408 - lpp evoluindo em	Enviada	Eventos Adversos Relacionados à	Lesão por pressão		
12/07/2022	CE	FORTALEZA	HOSPITAL GERAL DR. WALDEM	62471 - lpp em dorso estágio II	Enviada	Eventos Adversos Relacionados à	Lesão por pressão		
12/07/2022	CE	FORTALEZA	HOSPITAL GERAL DR. WALDEM	62605 - lpp abdominal	Enviada	Eventos Adversos Relacionados à	Lesão por pressão		
12/07/2022	CE	FORTALEZA	HOSPITAL GERAL DR. WALDEM	62606 - lpp região tórax	Enviada	Eventos Adversos Relacionados à	Lesão por pressão		
12/07/2022	CE	FORTALEZA	HOSPITAL GERAL DR. WALDEM	62616 - lpp em calcâneo estágio	Enviada	Eventos Adversos Relacionados à	Lesão por pressão		
12/07/2022	CE	FORTALEZA	HOSPITAL GERAL DR. WALDEM	62621 - lpp em abdômen estágio	Enviada	Eventos Adversos Relacionados à	Lesão por pressão		
12/07/2022	CE	FORTALEZA	HOSPITAL GERAL DR. WALDEM	62639 - lpp em abdômen estágio	Enviada	Eventos Adversos Relacionados à	Lesão por pressão		
12/07/2022	CE	FORTALEZA	HOSPITAL GERAL DR. WALDEM	62758 - coleta do pct errado	Enviada	Eventos Adversos Relacionados à	Falhas ocorridas em laboratóri		
12/07/2022	CE	FORTALEZA	HOSPITAL GERAL DR. WALDEM	62917 - flebite	Enviada	Eventos Adversos Relacionados à	Falhas envolvendo cateter venoso		
12/07/2022	CE	FORTALEZA	HOSPITAL GERAL DR. WALDEM	62925 - lpp em região sacra	Enviada	Eventos Adversos Relacionados à	Lesão por pressão		

12/07/2022	CE	FORTALEZA	HOSPITAL GERAL DR. WALDEM	62935 - perda de picc	Enviada	Eventos Adversos Relacionados à	Falhas envolvendo sondas		
12/07/2022	CE	FORTALEZA	HOSPITAL GERAL DR. WALDEM	62964 - lpp em mmii direito	Enviada	Eventos Adversos Relacionados à	Lesão por pressão		
12/07/2022	CE	FORTALEZA	HOSPITAL GERAL DR. WALDEM	62966 - lpp em região sacra	Enviada	Eventos Adversos Relacionados à	Lesão por pressão		
12/07/2022	CE	FORTALEZA	HOSPITAL GERAL DR. WALDEM	63010 - lpp em região sacra	Enviada	Eventos Adversos Relacionados à	Lesão por pressão		
12/07/2022	CE	FORTALEZA	HOSPITAL GERAL DR. WALDEM	63029 - lpp em região sacra	Enviada	Eventos Adversos Relacionados à	Lesão por pressão		
12/07/2022	CE	FORTALEZA	HOSPITAL GERAL DR. WALDEM	63044 - lpp sacra e trocânter	Enviada	Eventos Adversos Relacionados à	Lesão por pressão		
12/07/2022	CE	FORTALEZA	HOSPITAL GERAL DR. WALDEM	63183 - lpp interglúteo estágio	Enviada	Eventos Adversos Relacionados à	Lesão por pressão		
12/07/2022	CE	FORTALEZA	HOSPITAL GERAL DR. WALDEM	63239 - lpp em região sacra	Enviada	Eventos Adversos Relacionados à	Lesão por pressão		
12/07/2022	CE	FORTALEZA	HOSPITAL GERAL DR. WALDEM	63249 - sne obstruída	Enviada	Eventos Adversos Relacionados à	Falhas envolvendo sondas		
12/07/2022	CE	FORTALEZA	HOSPITAL GERAL DR. WALDEM	63253 - perda de sne	Enviada	Eventos Adversos Relacionados à	Falhas envolvendo sondas		
12/07/2022	CE	FORTALEZA	HOSPITAL GERAL DR. WALDEM	63275 - sne obstruída	Enviada	Eventos Adversos Relacionados à	Falhas envolvendo sondas		
12/07/2022	CE	FORTALEZA	HOSPITAL GERAL DR. WALDEM	63294 - lpp em tórax, axila	Enviada	Eventos Adversos Relacionados à	Lesão por pressão		
12/07/2022	CE	FORTALEZA	HOSPITAL GERAL DR. WALDEM	63296 - lesão próxima ao	Enviada	Eventos Adversos Relacionados à	Falhas envolvendo cateter venoso		
10/08/2022	CE	FORTALEZA	HOSPITAL GERAL DR. WALDEM	61221	Enviada	Eventos Adversos Relacionados à	Lesão por pressão		
10/08/2022	CE	FORTALEZA	HOSPITAL GERAL DR. WALDEM	61302	Enviada	Eventos Adversos Relacionados à	Lesão por pressão		
10/08/2022	CE	FORTALEZA	HOSPITAL GERAL DR. WALDEM	60092	Enviada	Eventos Adversos Relacionados à	Lesão por pressão		

10/08/2022	CE	FORTALEZA	HOSPITAL GERAL DR. WALDEM	60124	Enviada	Eventos Adversos Relacionados à	Lesão por pressão		
10/08/2022	CE	FORTALEZA	HOSPITAL GERAL DR. WALDEM	58991	Enviada	Eventos Adversos Relacionados à	Falhas envolvendo sondas		
10/08/2022	CE	FORTALEZA	HOSPITAL GERAL DR. WALDEM	59197	Enviada	Eventos Adversos Relacionados à	Queda do paciente		
13/10/2022	CE	FORTALEZA	HOSPITAL GERAL DR. WALDEM	78449	Enviada	Eventos Adversos Relacionados à	Queda do paciente		
13/10/2022	CE	FORTALEZA	HOSPITAL GERAL DR. WALDEM	78575	Enviada	Eventos Adversos Relacionados à	Queda do paciente		
13/10/2022	CE	FORTALEZA	HOSPITAL GERAL DR. WALDEM	78583 - atraso na realização de	Enviada	Eventos Adversos Relacionados à	Extubação endotraqueal		
13/10/2022	CE	FORTALEZA	HOSPITAL GERAL DR. WALDEM	78697	Enviada	Eventos Adversos Relacionados à	Queda do paciente		
13/10/2022	CE	FORTALEZA	HOSPITAL GERAL DR. WALDEM	78666	Enviada	Eventos Adversos Relacionados à	Falhas envolvendo sondas		
13/10/2022	CE	FORTALEZA	HOSPITAL GERAL DR. WALDEM	78665	Enviada	Eventos Adversos Relacionados à	Falhas envolvendo sondas		
13/10/2022	CE	FORTALEZA	HOSPITAL GERAL DR. WALDEM	78657	Enviada	Eventos Adversos Relacionados à	Lesão por pressão		
13/10/2022	CE	FORTALEZA	HOSPITAL GERAL DR. WALDEM	78655 - Perda de PICC	Enviada	Eventos Adversos Relacionados à	Falhas envolvendo sondas		
13/10/2022	CE	FORTALEZA	HOSPITAL GERAL DR. WALDEM	78830	Enviada	Eventos Adversos Relacionados à	Falhas envolvendo sondas		
13/10/2022	CE	FORTALEZA	HOSPITAL GERAL DR. WALDEM	78937	Enviada	Eventos Adversos Relacionados à	Falhas envolvendo sondas		
13/10/2022	CE	FORTALEZA	HOSPITAL GERAL DR. WALDEM	78938	Enviada	Eventos Adversos Relacionados à	Falhas envolvendo sondas		
13/10/2022	CE	FORTALEZA	HOSPITAL GERAL DR. WALDEM	-	Enviada	Eventos Adversos Relacionados à	Falhas envolvendo sondas		
13/10/2022	CE	FORTALEZA	HOSPITAL GERAL DR. WALDEM	78939	Enviada	Eventos Adversos Relacionados à	Falhas envolvendo sondas		

13/10/2022	CE	FORTALEZA	HOSPITAL GERAL DR. WALDEM	78940	Enviada	Eventos Adversos Relacionados à	Falhas envolvendo sondas		
13/10/2022	CE	FORTALEZA	HOSPITAL GERAL DR. WALDEM	78941	Enviada	Eventos Adversos Relacionados à	Falhas envolvendo sondas		
13/10/2022	CE	FORTALEZA	HOSPITAL GERAL DR. WALDEM	78942	Enviada	Eventos Adversos Relacionados à	Falhas envolvendo sondas		
13/10/2022	CE	FORTALEZA	HOSPITAL GERAL DR. WALDEM	79675	Enviada	Eventos Adversos Relacionados à	Falhas envolvendo sondas		
13/10/2022	CE	FORTALEZA	HOSPITAL GERAL DR. WALDEM	79679	Enviada	Eventos Adversos Relacionados à	Falhas envolvendo sondas		
13/10/2022	CE	FORTALEZA	HOSPITAL GERAL DR. WALDEM	79680	Enviada	Eventos Adversos Relacionados à	Falhas envolvendo sondas		
13/10/2022	CE	FORTALEZA	HOSPITAL GERAL DR. WALDEM	79717	Enviada	Eventos Adversos Relacionados à	Falhas envolvendo cateter venoso		
13/10/2022	CE	FORTALEZA	HOSPITAL GERAL DR. WALDEM	79678	Enviada	Eventos Adversos Relacionados à	Falhas envolvendo sondas		
26/10/2022	CE	FORTALEZA	HOSPITAL GERAL DR. WALDEM	66238 perda de picc	Enviada	Eventos Adversos Relacionados à	Falhas envolvendo sondas		
06/06/2019	CE	FORTALEZA	HIAS - HOSPITAL INFANTIL	Lesao por possível extravasa	Enviada	Eventos Adversos Relacionados à	Falhas durante a assistência à saúde		
24/10/2019	CE	FORTALEZA	HIAS - HOSPITAL INFANTIL	Paciente L A.D.L, idade de 1 ano e	Enviada	Eventos Adversos Relacionados à	Queda do paciente		
02/01/2020	CE	FORTALEZA	HIAS - HOSPITAL INFANTIL	Paciente com histórico de má	Enviada	Eventos Adversos Relacionados à	Falhas durante a assistência à saúde		
31/12/2020	CE	FORTALEZA	HIAS - HOSPITAL INFANTIL	PACIENTE REALIZOU	Enviada	Eventos Adversos Relacionados à	Falhas durante a assistência à saúde		
31/12/2020	CE	FORTALEZA	HIAS - HOSPITAL INFANTIL	enviado pelicula de raio x de outro	Enviada	Eventos Adversos Relacionados à	Falha na identificação do paciente		
31/12/2020	CE	FORTALEZA	HIAS - HOSPITAL INFANTIL	FOI ENTREGUE NA UNIDADE	Enviada	Eventos Adversos Relacionados à	Falha na identificação do paciente		

31/12/2020	CE	FORTALEZA	HIAS - HOSPITAL INFANTIL	durante inserção do cateter	Enviada	Eventos Adversos Relacionados à	Falhas durante procedimento		
28/12/2021	CE	FORTALEZA	hospital infantil albert sabin	Houve troca de paciente durante a	Enviada	Eventos Adversos Relacionados à	Falhas ocorridas em laboratóri		
28/12/2021	CE	FORTALEZA	hospital infantil albert sabin	Houve troca de paciente durante a	Enviada	Eventos Adversos Relacionados à	Falhas ocorridas em laboratóri		
28/12/2021	CE	FORTALEZA	hospital infantil albert sabin	Lesão por pressão estágio 1	Enviada	Eventos Adversos Relacionados à	Lesão por pressão		
28/12/2021	CE	FORTALEZA	hospital infantil albert sabin	Lesão por pressão não	Enviada	Eventos Adversos Relacionados à	Lesão por pressão		
28/12/2021	CE	FORTALEZA	hospital infantil albert sabin	Lesão por pressão em	Enviada	Eventos Adversos Relacionados à	Lesão por pressão		
15/02/2022	CE	FORTALEZA	hospital infantil albert sabin	Sobrenome incorreto na ficha	Enviada	Eventos Adversos Relacionados à	Falhas nas atividades		
15/03/2022	CE	FORTALEZA	hospital infantil albert sabin	Identificada lesão por pressão	Enviada	Eventos Adversos Relacionados à	Lesão por pressão		
14/04/2022	CE	FORTALEZA	hospital infantil albert sabin	Lesão por pressão identificad	Enviada	Eventos Adversos Relacionados à	Lesão por pressão		
15/06/2022	CE	FORTALEZA	hospital infantil albert sabin	Queda do leito.	Enviada	Eventos Adversos Relacionados à	Queda do paciente		
15/07/2022	CE	FORTALEZA	hospital infantil albert sabin	Presença de eritema na região	Enviada	Eventos Adversos Relacionados à	Lesão por pressão		
12/08/2022	CE	FORTALEZA	hospital infantil albert sabin	lesão por pressão não classificáv	Enviada	Eventos Adversos Relacionados à	Lesão por pressão		
08/09/2022	CE	FORTALEZA	hospital infantil albert sabin	Laudo de tomografia de crânio	Enviada	Eventos Adversos Relacionados à	Falha na documentação		
11/10/2022	CE	FORTALEZA	hospital infantil albert sabin	Lesão por pressão em	Enviada	Eventos Adversos Relacionados à	Lesão por pressão		
22/11/2022	CE	FORTALEZA	HIAS - HOSPITAL INFANTIL	As fezes do paciente nao	Enviada	Eventos Adversos Relacionados à	Falhas durante a assistência à saúde		

14/12/2022	CE	FORTALEZA	HIAS - HOSPITAL INFANTIL	PACIENTE DEAMBULANDO	Concluída	Eventos Adversos Relacionados à	Falhas no cuidado / proteção do		
14/12/2022	CE	FORTALEZA	HIAS - HOSPITAL INFANTIL	Prontuario incompleto	Enviada	Eventos Adversos Relacionados à	Falhas nas atividades		
15/12/2022	CE	FORTALEZA	hospital infantil albert sabin	lesão de pele	Enviada	Eventos Adversos Relacionados à	Lesão por pressão		
15/12/2022	CE	FORTALEZA	hospital infantil albert sabin	-	Enviada	Eventos Adversos Relacionados à	Falhas durante a assistência à saúde		
16/12/2022	CE	FORTALEZA	HIAS - HOSPITAL INFANTIL	-	Enviada	Eventos Adversos Relacionados à	Lesão por pressão		
19/12/2022	CE	FORTALEZA	HIAS - HOSPITAL INFANTIL	Solicitado raio X DE DIVERSAS	Enviada	Eventos Adversos Relacionados à	Falhas durante a assistência à saúde		
20/12/2022	CE	FORTALEZA	HIAS - HOSPITAL INFANTIL	Prescrito dieta laxativa para	Enviada	Eventos Adversos Relacionados à	Falhas na administração de dietas		Enteral (oral, cateter oral ou
20/12/2022	CE	FORTALEZA	HIAS - HOSPITAL INFANTIL	Dectetado LP nas seguintes regiões	Enviada	Eventos Adversos Relacionados à	Lesão por pressão		
20/12/2022	CE	FORTALEZA	HIAS - HOSPITAL INFANTIL	Profissional administrou	Enviada	Eventos Adversos Relacionados à	Falhas durante a assistência à saúde		
26/12/2022	CE	FORTALEZA	HIAS - HOSPITAL INFANTIL	Paciente com quadro de	Enviada	Eventos Adversos Relacionados à	Broncoaspiração		
27/12/2022	CE	FORTALEZA	HIAS - HOSPITAL INFANTIL	Demora no resultado, comparec	Enviada	Eventos Adversos Relacionados à	Falhas ocorridas em laboratóri		
27/12/2022	CE	FORTALEZA	HIAS - HOSPITAL INFANTIL	PACIENTE EM ESTADO GRAVE	Enviada	Eventos Adversos Relacionados à	Falhas envolvendo o cateter venoso		
29/12/2022	CE	FORTALEZA	HIAS - HOSPITAL INFANTIL	Durante a realização de RX de	Enviada	Eventos Adversos Relacionados à	Acidentes do paciente	Força contundente (esmaga	
29/12/2022	CE	FORTALEZA	HIAS - HOSPITAL INFANTIL	PACIENTE COM LESÃO DE EPEL	Enviada	Eventos Adversos Relacionados à	Lesão por pressão		
29/12/2022	CE	FORTALEZA	HIAS - HOSPITAL INFANTIL	RECEBEMOS PACIENTE	Enviada	Eventos Adversos Relacionados à	Falhas nas atividades		

29/12/2022	CE	FORTALEZA	HIAS - HOSPITAL INFANTIL	Ao exame físico observado	Enviada	Eventos Adversos Relacionados à	Lesão por pressão		
29/12/2022	CE	FORTALEZA	HIAS - HOSPITAL INFANTIL	LESAO DE PELE POR DISPOSI	Enviada	Eventos Adversos Relacionados à	Falhas durante a assistência à saúde		
09/01/2023	CE	FORTALEZA	HIAS - HOSPITAL INFANTIL	PACIENTE RPOVENIENTE DA	Enviada	Eventos Adversos Relacionados à	Lesão por pressão		
09/01/2023	CE	FORTALEZA	HIAS - HOSPITAL INFANTIL	PACIENTE INTERNOU NO	Enviada	Eventos Adversos Relacionados à	Falhas nas atividades		
10/01/2023	CE	FORTALEZA	HIAS - HOSPITAL INFANTIL	PACIENTE COM CITOACIDOSE	Enviada	Eventos Adversos Relacionados à	Falhas durante a assistência à saúde		
10/01/2023	CE	FORTALEZA	HIAS - HOSPITAL INFANTIL	PACIENTE APRESENTAA	Enviada	Eventos Adversos Relacionados à	Falhas ocorridas em laboratóri		
10/01/2023	CE	FORTALEZA	HIAS - HOSPITAL INFANTIL	PACIENTE COLHEU PCR	Enviada	Eventos Adversos Relacionados à	Falhas ocorridas em laboratóri		
11/01/2023	CE	FORTALEZA	HIAS - HOSPITAL INFANTIL	NO DIA 21/11/2022 ENFERM	Enviada	Eventos Adversos Relacionados à	Falhas durante a assistência à saúde		
11/01/2023	CE	FORTALEZA	HIAS - HOSPITAL INFANTIL	PICC OBSTRUÍDO	Enviada	Eventos Adversos Relacionados à	Falhas envolvendo o cateter venoso		
12/01/2023	CE	FORTALEZA	HIAS - HOSPITAL INFANTIL	PACIENTE ADMITIDA NA	Enviada	Eventos Adversos Relacionados à	Falhas nas atividades		
16/01/2023	CE	FORTALEZA	HIAS - HOSPITAL INFANTIL	PACIENTE COLETOU PCR P/	Enviada	Eventos Adversos Relacionados à	Falhas nas atividades		
16/01/2023	CE	FORTALEZA	HIAS - HOSPITAL INFANTIL	EM 04/01/23 FOI SOLICITA	Enviada	Eventos Adversos Relacionados à	Falhas durante a assistência à saúde		
16/01/2023	CE	FORTALEZA	HIAS - HOSPITAL INFANTIL	LATENTE, 1M, SETOR REANIMA	Enviada	Eventos Adversos Relacionados à	Lesão por pressão		
18/01/2023	CE	FORTALEZA	HIAS - HOSPITAL INFANTIL	No dia 04/01 foi solicitado coleta do	Enviada	Eventos Adversos Relacionados à	Falhas ocorridas em laboratóri		
18/01/2023	CE	FORTALEZA	HIAS - HOSPITAL INFANTIL	IDENTIFICADO DISCORDANCIA	Enviada	Eventos Adversos Relacionados à	Falha na identificação do paciente		

18/01/2023	CE	FORTALEZA	HIAS - HOSPITAL INFANTIL	PACIENTE EM DIETAA ZERO	Enviada	Eventos Adversos Relacionados à	Falhas durante a assistência à saúde		
20/01/2023	CE	FORTALEZA	HIAS - HOSPITAL INFANTIL	AZUL DE METILENO 1% DISPENS	Enviada	Eventos Adversos Relacionados à	Falhas durante a assistência à saúde		
20/01/2023	CE	FORTALEZA	HIAS - HOSPITAL INFANTIL	RN ADMITIDO E ABERTO	Enviada	Eventos Adversos Relacionados à	Falhas nas atividades		
30/01/2023	CE	FORTALEZA	HIAS - HOSPITAL INFANTIL	Paciente oriundo do centro ciurgico	Enviada	Eventos Adversos Relacionados à	Lesão por pressão		
10/02/2023	CE	FORTALEZA	HIAS - HOSPITAL INFANTIL	Recebido visita do escritório de	Enviada	Eventos Adversos Relacionados à	Falhas nas atividades		
10/02/2023	CE	FORTALEZA	HIAS - HOSPITAL INFANTIL	FARMACIA DISPENSOU	Enviada	Eventos Adversos Relacionados à	Falhas durante a assistência à saúde		
10/02/2023	CE	FORTALEZA	HIAS - HOSPITAL INFANTIL	Laudo UCM REALIZADO É	Enviada	Eventos Adversos Relacionados à	Falhas ocorridas em laboratóri		
17/02/2023	CE	FORTALEZA	HIAS - HOSPITAL INFANTIL	AO EXAME FISÍCO OBSERV	Enviada	Eventos Adversos Relacionados à	Lesão por pressão		
17/02/2023	CE	FORTALEZA	HIAS - HOSPITAL INFANTIL	Dispensado o lifazul pela farmacia	Enviada	Eventos Adversos Relacionados à	Falhas durante a assistência à saúde		
17/02/2023	CE	FORTALEZA	HIAS - HOSPITAL INFANTIL	Identificado o que a data de nasciento	Enviada	Eventos Adversos Relacionados à	Falhas nas atividades		
17/02/2023	CE	FORTALEZA	HIAS - HOSPITAL INFANTIL	PACIENTE ENCAMINHADA	Enviada	Eventos Adversos Relacionados à	Falhas envolvendo o cateter venoso		
17/02/2023	CE	FORTALEZA	HIAS - HOSPITAL INFANTIL	PACIENTE REALIZOU RX	Enviada	Eventos Adversos Relacionados à	Falhas nas atividades		
17/02/2023	CE	FORTALEZA	HIAS - HOSPITAL INFANTIL	Prescrito um frasco de heparina	Enviada	Eventos Adversos Relacionados à	Falhas durante a assistência à saúde		
23/02/2023	CE	FORTALEZA	HIAS - HOSPITAL INFANTIL	Recebo paciente sem pulseira	Enviada	Eventos Adversos Relacionados à	Falha na identificação do paciente		
23/02/2023	CE	FORTALEZA	HIAS - HOSPITAL INFANTIL	Resultado de exame de	Enviada	Eventos Adversos Relacionados à	Falhas durante a assistência à saúde		

23/02/2023	CE	FORTALEZA	HIAS - HOSPITAL INFANTIL	-	Enviada	Eventos Adversos Relacionados à	Falhas durante a assistência à saúde		
06/03/2023	CE	FORTALEZA	HIAS - HOSPITAL INFANTIL	IDENTIFICADO DIVERGENCIA EM	Enviada	Eventos Adversos Relacionados à	Falhas durante a assistência à saúde		
08/03/2023	CE	FORTALEZA	HIAS - HOSPITAL INFANTIL	Foi prescrito o medicame	Em análise	Eventos Adversos Relacionados à	Falhas durante a assistência à saúde		
09/03/2023	CE	FORTALEZA	HIAS - HOSPITAL INFANTIL	Evento adverso flagrado de	Enviada	Eventos Adversos Relacionados à	Queda do paciente		
13/03/2023	CE	FORTALEZA	HIAS - HOSPITAL INFANTIL	Queimadura por oximetro, dispositiv	Enviada	Eventos Adversos Relacionados à	Lesão por pressão		
14/03/2023	CE	FORTALEZA	HIAS - HOSPITAL INFANTIL	Identificad p lesão em região	Enviada	Eventos Adversos Relacionados à	Lesão por pressão		
14/03/2023	CE	FORTALEZA	HIAS - HOSPITAL INFANTIL	Paciente com leãi em MID por	Enviada	Eventos Adversos Relacionados à	Lesão por pressão		
23/03/2023	CE	FORTALEZA	HIAS - HOSPITAL INFANTIL	Paciente voltou da traqueostomia	Concluída	Eventos Adversos Relacionados à	Falhas durante procedimento		
19/06/2019	CE	FORTALEZA	HOSPITAL E MATERNIDADE JOSE MARTINI	-	Enviada	Eventos Adversos Relacionados à Assistência à	Queda do paciente		
16/06/2021	CE	FORTALEZA	HOSPITAL E MATERNIDADE JOSE MARTINI	Troca de amostra na coleta	Enviada	Eventos Adversos Relacionados à Assistência à	Falhas ocorridas em laboratórios clínicos		
13/07/2021	CE	FORTALEZA	HOSPITAL E MATERNIDADE JOSE MARTINI	Extravasamento de medicamento	Enviada	Eventos Adversos Relacionados à Assistência à	Falhas durante a assistência à saúde		
29/07/2021	CE	FORTALEZA	HOSPITAL E MATERNIDADE JOSE MARTINI	Paciente ao levantar da cama apresentou	Enviada	Eventos Adversos Relacionados à Assistência à	Queda do paciente		
15/01/2019	CE	FORTALEZA	Hospital de Messejana Dr. Carlos	-	Em retificação	Eventos Adversos Relacionados à Assistênci	Queda do paciente		

12/02/2019	CE	FORTALEZA	Hospital de Messejana Dr. Carlos	-	Enviada	Eventos Adversos Relacionados à Assistênci	Queda do paciente		
16/09/2019	CE	FORTALEZA	Hospital de Messejana Dr. Carlos	-	Enviada	Eventos Adversos Relacionados à Assistênci	Queda do paciente		
16/09/2019	CE	FORTALEZA	Hospital de Messejana Dr. Carlos	apresentou sincope enquanto estava sentada	Enviada	Eventos Adversos Relacionados à Assistênci	Queda do paciente		
20/09/2019	CE	FORTALEZA	Hospital de Messejana Dr. Carlos	paciente ao levantar-se da cadeira	Enviada	Eventos Adversos Relacionados à Assistênci	Queda do paciente		
23/09/2019	CE	FORTALEZA	Hospital de Messejana Dr. Carlos	-	Enviada	Eventos Adversos Relacionados à Assistênci	Queda do paciente		
01/10/2019	CE	FORTALEZA	Hospital de Messejana Dr. Carlos	queda	Enviada	Eventos Adversos Relacionados à Assistênci	Queda do paciente		
01/10/2019	CE	FORTALEZA	Hospital de Messejana Dr. Carlos	-	Enviada	Eventos Adversos Relacionados à Assistênci	Queda do paciente		
04/02/2020	CE	FORTALEZA	Hospital de Messejana Dr. Carlos	-	Enviada	Eventos Adversos Relacionados à Assistênci	Queda do paciente		
04/02/2020	CE	FORTALEZA	Hospital de Messejana Dr. Carlos	-	Enviada	Eventos Adversos Relacionados à Assistênci	Queda do paciente		
04/02/2020	CE	FORTALEZA	Hospital de Messejana Dr. Carlos	-	Enviada	Eventos Adversos Relacionados à Assistênci	Queda do paciente		
10/02/2020	CE	FORTALEZA	Hospital de Messejana Dr. Carlos	lesão por pressão	Enviada	Eventos Adversos Relacionados à Assistênci	Lesão por pressão		
19/02/2020	CE	FORTALEZA	Hospital de Messejana Dr. Carlos	lesão por pressão	Enviada	Eventos Adversos Relacionados à Assistênci	Lesão por pressão		

19/02/2020	CE	FORTALEZA	Hospital de Messejana Dr. Carlos	queda	Enviada	Eventos Adversos Relacionados à Assistênci	Queda do paciente		
20/02/2020	CE	FORTALEZA	Hospital de Messejana Dr. Carlos	lesão por pressão	Enviada	Eventos Adversos Relacionados à Assistênci	Lesão por pressão		
20/02/2020	CE	FORTALEZA	Hospital de Messejana Dr. Carlos	lesão por pressão	Enviada	Eventos Adversos Relacionados à Assistênci	Lesão por pressão		
20/02/2020	CE	FORTALEZA	Hospital de Messejana Dr. Carlos	lesão por pressão	Enviada	Eventos Adversos Relacionados à Assistênci	Lesão por pressão		
03/03/2020	CE	FORTALEZA	Hospital de Messejana Dr. Carlos	queda	Enviada	Eventos Adversos Relacionados à Assistênci	Queda do paciente		
03/03/2020	CE	FORTALEZA	Hospital de Messejana Dr. Carlos	queda	Enviada	Eventos Adversos Relacionados à Assistênci	Queda do paciente		
03/03/2020	CE	FORTALEZA	Hospital de Messejana Dr. Carlos	queda	Enviada	Eventos Adversos Relacionados à Assistênci	Queda do paciente		
04/03/2020	CE	FORTALEZA	Hospital de Messejana Dr. Carlos	lesão por pressão	Enviada	Eventos Adversos Relacionados à Assistênci	Lesão por pressão		
04/03/2020	CE	FORTALEZA	Hospital de Messejana Dr. Carlos	lesão por pressão	Enviada	Eventos Adversos Relacionados à Assistênci	Lesão por pressão		
04/03/2020	CE	FORTALEZA	Hospital de Messejana Dr. Carlos	lesão por pressão	Enviada	Eventos Adversos Relacionados à Assistênci	Lesão por pressão		
09/03/2020	CE	FORTALEZA	Hospital de Messejana Dr. Carlos	-	Enviada	Eventos Adversos Relacionados à Assistênci	Queda do paciente		
12/03/2020	CE	FORTALEZA	Hospital de Messejana Dr. Carlos	queda	Enviada	Eventos Adversos Relacionados à Assistênci	Queda do paciente		

12/08/2020	CE	FORTALEZA	Hospital de Messejana Dr. Carlos	lesão por pressão	Enviada	Eventos Adversos Relacionados à Assistênci	Lesão por pressão		
21/09/2020	CE	FORTALEZA	Hospital de Messejana Dr. Carlos	Paciente transportado para realizar ECO em	Enviada	Eventos Adversos Relacionados à Assistênci	Falhas durante a assistência à saúde		
25/09/2020	CE	FORTALEZA	Hospital de Messejana Dr. Carlos	PACIENTE PUXOU A SNE, AGITADA	Enviada	Eventos Adversos Relacionados à Assistênci	Falhas envolvendo sondas		
06/10/2020	CE	FORTALEZA	Hospital de Messejana Dr. Carlos	Paciente ao levantar-se do leito	Enviada	Eventos Adversos Relacionados à Assistênci	Queda do paciente		
07/10/2020	CE	FORTALEZA	Hospital de Messejana Dr. Carlos	Paciente hipersecretiva, ao tentar se levantar	Enviada	Eventos Adversos Relacionados à Assistênci	Queda do paciente		
07/10/2020	CE	FORTALEZA	Hospital de Messejana Dr. Carlos	Encontrava-se sentado em cadeira	Enviada	Eventos Adversos Relacionados à Assistênci	Queda do paciente		
28/10/2020	CE	FORTALEZA	Hospital de Messejana Dr. Carlos	realizado RX, porém sem identificac	Enviada	Eventos Adversos Relacionados à Assistênci	Falhas nas atividades administra		
28/10/2020	CE	FORTALEZA	Hospital de Messejana Dr. Carlos	Paciente transportado para realizar ECO em	Enviada	Eventos Adversos Relacionados à Assistênci	Falhas durante a assistência à saúde		
29/10/2020	CE	FORTALEZA	Hospital de Messejana Dr. Carlos	PACIENTE PUXOU A SNE, AGITADA	Enviada	Eventos Adversos Relacionados à Assistênci	Falhas envolvendo sondas		
29/10/2020	CE	FORTALEZA	Hospital de Messejana Dr. Carlos	Paciente procedente da UTI apresentando	Enviada	Eventos Adversos Relacionados à Assistênci	Lesão por pressão		
29/10/2020	CE	FORTALEZA	Hospital de Messejana Dr. Carlos	Perda acidental do cateter PAI	Enviada	Eventos Adversos Relacionados à Assistênci	Acidentes do paciente	Outro tipo de mecanismo de lesão	
29/10/2020	CE	FORTALEZA	Hospital de Messejana Dr. Carlos	Paciente procedente da UTI informado como	Enviada	Eventos Adversos Relacionados à Assistênci	Lesão por pressão		

30/10/2020	CE	FORTALEZA	Hospital de Messejana Dr. Carlos	Realizado RX porém, películas de RX	Enviada	Eventos Adversos Relacionados à Assistênci	Falha na identificação do paciente		
30/10/2020	CE	FORTALEZA	Hospital de Messejana Dr. Carlos	realizado raio-x, porém sem identificac	Enviada	Eventos Adversos Relacionados à Assistênci	Falha na identificação do paciente		
30/10/2020	CE	FORTALEZA	Hospital de Messejana Dr. Carlos	PELICULA DE RADIOLOGIA VEIO	Enviada	Eventos Adversos Relacionados à Assistênci	Falha na identificação do paciente		
30/10/2020	CE	FORTALEZA	Hospital de Messejana Dr. Carlos	TROCA NA IDENTIFICAÇÃO DAS	Enviada	Eventos Adversos Relacionados à Assistênci	Falha na identificação do paciente		
03/11/2020	CE	FORTALEZA	Hospital de Messejana Dr. Carlos	Segundo descrição da notificação	Enviada	Eventos Adversos Relacionados à Assistênci	Lesão por pressão		
03/11/2020	CE	FORTALEZA	Hospital de Messejana Dr. Carlos	Segundo descrição da notificação, o	Enviada	Eventos Adversos Relacionados à Assistênci	Lesão por pressão		
03/11/2020	CE	FORTALEZA	Hospital de Messejana Dr. Carlos	Paciente procedente da emergência	Enviada	Eventos Adversos Relacionados à Assistênci	Lesão por pressão		
03/11/2020	CE	FORTALEZA	Hospital de Messejana Dr. Carlos	Falta de identificação do nome do paciente	Enviada	Eventos Adversos Relacionados à Assistênci	Falha na identificação do paciente		
03/11/2020	CE	FORTALEZA	Hospital de Messejana Dr. Carlos	Falta de identificação do nome do paciente	Enviada	Eventos Adversos Relacionados à Assistênci	Falha na identificação do paciente		
03/11/2020	CE	FORTALEZA	Hospital de Messejana Dr. Carlos	Paciente admitido em determinada	Enviada	Eventos Adversos Relacionados à Assistênci	Lesão por pressão		
04/11/2020	CE	FORTALEZA	Hospital de Messejana Dr. Carlos	Paciente procedente da emergência	Enviada	Eventos Adversos Relacionados à Assistênci	Lesão por pressão		
04/11/2020	CE	FORTALEZA	Hospital de Messejana Dr. Carlos	Equipo de dieta por SOG protegido, foi	Enviada	Eventos Adversos Relacionados à Assistênci	Falhas na administração de dietas		Enteral (oral, cateter oral ou nasal

05/11/2020	CE	FORTALEZA	Hospital de Messejana Dr. Carlos	queda	Enviada	Eventos Adversos Relacionados à Assistênci	Queda do paciente		
05/11/2020	CE	FORTALEZA	Hospital de Messejana Dr. Carlos	-	Enviada	Eventos Adversos Relacionados à Assistênci	Queda do paciente		
05/11/2020	CE	FORTALEZA	Hospital de Messejana Dr. Carlos	-	Enviada	Eventos Adversos Relacionados à Assistênci	Queda do paciente		
05/11/2020	CE	FORTALEZA	Hospital de Messejana Dr. Carlos	Queda	Enviada	Eventos Adversos Relacionados à Assistênci	Queda do paciente		
05/11/2020	CE	FORTALEZA	Hospital de Messejana Dr. Carlos	-	Enviada	Eventos Adversos Relacionados à Assistênci	Queda do paciente		
09/11/2020	CE	FORTALEZA	Hospital de Messejana Dr. Carlos	PACIENTE DO DETRMINADA UNIDADE	Enviada	Eventos Adversos Relacionados à Assistênci	Lesão por pressão		
10/11/2020	CE	FORTALEZA	Hospital de Messejana Dr. Carlos	Paciente admitida na UTI procedente da	Enviada	Eventos Adversos Relacionados à Assistênci	Lesão por pressão		
10/11/2020	CE	FORTALEZA	Hospital de Messejana Dr. Carlos	PACIENTE ADMITIDO NA EMERGÊ	Enviada	Eventos Adversos Relacionados à Assistênci	Lesão por pressão		
10/11/2020	CE	FORTALEZA	Hospital de Messejana Dr. Carlos	Paciente admitido na Unidade de	Enviada	Eventos Adversos Relacionados à Assistênci	Lesão por pressão		
11/11/2020	CE	FORTALEZA	Hospital de Messejana Dr. Carlos	Paciente admitido da UNIDADE DE	Enviada	Eventos Adversos Relacionados à Assistênci	Lesão por pressão		
11/11/2020	CE	FORTALEZA	Hospital de Messejana Dr. Carlos	PACIENTE ADMITIDO COM LESAO	Enviada	Eventos Adversos Relacionados à Assistênci	Lesão por pressão		
11/11/2020	CE	FORTALEZA	Hospital de Messejana Dr. Carlos	Paciente admitido apresentando lesão por	Enviada	Eventos Adversos Relacionados à Assistênci	Lesão por pressão		

11/11/2020	CE	FORTALEZA	Hospital de Messejana Dr. Carlos	Paciente admitida na emergência	Enviada	Eventos Adversos Relacionados à Assistênci	Lesão por pressão		
11/11/2020	CE	FORTALEZA	Hospital de Messejana Dr. Carlos	Paciente apresentando lesão por pressão	Enviada	Eventos Adversos Relacionados à Assistênci	Lesão por pressão		
11/11/2020	CE	FORTALEZA	Hospital de Messejana Dr. Carlos	PACIENTE ADMITIDA APRESE	Enviada	Eventos Adversos Relacionados à Assistênci	Lesão por pressão		
11/11/2020	CE	FORTALEZA	Hospital de Messejana Dr. Carlos	PELE INTEGRA COM DISCRETA	Enviada	Eventos Adversos Relacionados à Assistênci	Lesão por pressão		
11/11/2020	CE	FORTALEZA	Hospital de Messejana Dr. Carlos	Na admissão da paciente em	Enviada	Eventos Adversos Relacionados à Assistênci	Lesão por pressão		
16/11/2020	CE	FORTALEZA	Hospital de Messejana Dr. Carlos	ao levantar-se do leito paciente	Enviada	Eventos Adversos Relacionados à Assistênci	Queda do paciente		
16/11/2020	CE	FORTALEZA	Hospital de Messejana Dr. Carlos	Paciente admitida na UTI com LP em	Enviada	Eventos Adversos Relacionados à Assistênci	Lesão por pressão		
23/11/2020	CE	FORTALEZA	Hospital de Messejana Dr. Carlos	Lesão em região sacral, com bordas	Enviada	Eventos Adversos Relacionados à Assistênci	Lesão por pressão		
23/11/2020	CE	FORTALEZA	Hospital de Messejana Dr. Carlos	Paciente procedente da Emergência	Enviada	Eventos Adversos Relacionados à Assistênci	Lesão por pressão		
23/11/2020	CE	FORTALEZA	Hospital de Messejana Dr. Carlos	Segundo descrição da notificação	Enviada	Eventos Adversos Relacionados à Assistênci	Lesão por pressão		
24/11/2020	CE	FORTALEZA	Hospital de Messejana Dr. Carlos	PACIENTE ESTAVA EM PÉ PRÓXIM	Enviada	Eventos Adversos Relacionados à Assistênci	Queda do paciente		
26/11/2020	CE	FORTALEZA	Hospital de Messejana Dr. Carlos	paciente sofreu queda ao ser transporta	Enviada	Eventos Adversos Relacionados à Assistênci	Queda do paciente		

27/11/2020	CE	FORTALEZA	Hospital de Messejana Dr. Carlos	Jarra de umidificação do respirador vazia	Enviada	Eventos Adversos Relacionados à Assistênci	Falhas durante a assistência à saúde		
27/11/2020	CE	FORTALEZA	Hospital de Messejana Dr. Carlos	Jarra de umidificação do respirador vazia	Enviada	Eventos Adversos Relacionados à Assistênci	Falhas durante a assistência à saúde		
27/11/2020	CE	FORTALEZA	Hospital de Messejana Dr. Carlos	Jarra de umidificação do respirador vazia	Enviada	Eventos Adversos Relacionados à Assistênci	Falhas durante a assistência à saúde		
27/11/2020	CE	FORTALEZA	Hospital de Messejana Dr. Carlos	CANULA DE TRAQUEOSTOMO	Enviada	Eventos Adversos Relacionados à Assistênci	Falhas durante a assistência à saúde		
30/11/2020	CE	FORTALEZA	Hospital de Messejana Dr. Carlos	PACIENTE ADMITIDO COM PRESEN	Enviada	Eventos Adversos Relacionados à Assistênci	Lesão por pressão		
01/12/2020	CE	FORTALEZA	Hospital de Messejana Dr. Carlos	PACIENTE ENCONTRAVA-SE NO	Enviada	Eventos Adversos Relacionados à Assistênci	Queda do paciente		
01/12/2020	CE	FORTALEZA	Hospital de Messejana Dr. Carlos	IDENTIFICADO DURANTE RENOVA	Enviada	Eventos Adversos Relacionados à Assistênci	Lesão por pressão		
01/12/2020	CE	FORTALEZA	Hospital de Messejana Dr. Carlos	OBSERVADO LESÃO ISQUÊMICA EM	Enviada	Eventos Adversos Relacionados à Assistênci	Lesão por pressão		
02/12/2020	CE	FORTALEZA	Hospital de Messejana Dr. Carlos	APRESENTA LESÃO EM OCCIPIT	Enviada	Eventos Adversos Relacionados à Assistênci	Lesão por pressão		
03/12/2020	CE	FORTALEZA	Hospital de Messejana Dr. Carlos	PACIENTE ESTAVA DORMINDO EM	Enviada	Eventos Adversos Relacionados à Assistênci	Queda do paciente		
03/12/2020	CE	FORTALEZA	Hospital de Messejana Dr. Carlos	paciente sem acompanhante, caiu ao tentar	Enviada	Eventos Adversos Relacionados à Assistênci	Queda do paciente		
03/12/2020	CE	FORTALEZA	Hospital de Messejana Dr. Carlos	Lesão por pressão em região	Enviada	Eventos Adversos Relacionados à Assistênci	Lesão por pressão		

07/12/2020	CE	FORTALEZA	Hospital de Messejana Dr. Carlos	Paciente admitida apresentando lesão por	Enviada	Eventos Adversos Relacionados à Assistênci	Lesão por pressão		
07/12/2020	CE	FORTALEZA	Hospital de Messejana Dr. Carlos	Paciente com lesão por pressão em sacra	Enviada	Eventos Adversos Relacionados à Assistênci	Lesão por pressão		
07/12/2020	CE	FORTALEZA	Hospital de Messejana Dr. Carlos	Paciente admitida apresentando lesão por	Enviada	Eventos Adversos Relacionados à Assistênci	Lesão por pressão		
07/12/2020	CE	FORTALEZA	Hospital de Messejana Dr. Carlos	LESÃO NA SACRA EXTENSA	Enviada	Eventos Adversos Relacionados à Assistênci	Lesão por pressão		
07/12/2020	CE	FORTALEZA	Hospital de Messejana Dr. Carlos	Paciente com insuficiência cardíaca	Enviada	Eventos Adversos Relacionados à Assistênci	Lesão por pressão		
15/12/2020	CE	FORTALEZA	Hospital de Messejana Dr. Carlos	PACIENTE ATIVO,CAI DO LEITO	Enviada	Eventos Adversos Relacionados à Assistênci	Queda do paciente		
16/12/2020	CE	FORTALEZA	Hospital de Messejana Dr. Carlos	PACIENTE ESTAVA SENTADO NO	Enviada	Eventos Adversos Relacionados à Assistênci	Queda do paciente		
21/01/2021	CE	FORTALEZA	Hospital de Messejana Dr. Carlos	paciente encontrava-se realizando higiene	Enviada	Eventos Adversos Relacionados à Assistênci	Queda do paciente		
28/01/2021	CE	FORTALEZA	Hospital de Messejana Dr. Carlos	Paciente ao retornar do banho em	Enviada	Eventos Adversos Relacionados à Assistênci	Queda do paciente		
02/02/2021	CE	FORTALEZA	Hospital de Messejana Dr. Carlos	PACIENTE AGITADO E ALUCINA	Enviada	Eventos Adversos Relacionados à Assistênci	Queda do paciente		
02/02/2021	CE	FORTALEZA	Hospital de Messejana Dr. Carlos	Durante realização de procedimento de	Enviada	Eventos Adversos Relacionados à Assistênci	Falhas durante a assistência à saúde		
04/02/2021	CE	FORTALEZA	Hospital de Messejana Dr. Carlos	Violação do protocolo de higienizac	Enviada	Eventos Adversos Relacionados à Assistênci	Falhas durante a assistência à saúde		

04/02/2021	CE	FORTALEZA	Hospital de Messejana Dr. Carlos	Suco de laranja com presença de	Enviada	Eventos Adversos Relacionados à Assistênci	Falhas na administração de dietas		Enteral (oral, cateter oral ou nasal
05/02/2021	CE	FORTALEZA	Hospital de Messejana Dr. Carlos	PACIENTE TENTOU SE LEVANTA	Enviada	Eventos Adversos Relacionados à Assistênci	Queda do paciente		
15/02/2021	CE	FORTALEZA	Hospital de Messejana Dr. Carlos	PACIENTE FOI ENCAMINHADO PELA	Enviada	Eventos Adversos Relacionados à Assistênci	Queda do paciente		
16/02/2021	CE	FORTALEZA	Hospital de Messejana Dr. Carlos	AS 18:30 MÃE DA CRIANÇA DEIXOU GRADE	Enviada	Eventos Adversos Relacionados à Assistênci	Queda do paciente		
16/02/2021	CE	FORTALEZA	Hospital de Messejana Dr. Carlos	PACIENTE ESCORREGOU EM	Enviada	Eventos Adversos Relacionados à Assistênci	Queda do paciente		
16/02/2021	CE	FORTALEZA	Hospital de Messejana Dr. Carlos	Paciente acamado, com amputação de MIE	Enviada	Eventos Adversos Relacionados à Assistênci	Queda do paciente		
08/03/2021	CE	FORTALEZA	Hospital de Messejana Dr. Carlos	Paciente acamado, com amputação de MIE	Enviada	Eventos Adversos Relacionados à Assistênci	Falhas durante a assistência à saúde		
08/03/2021	CE	FORTALEZA	Hospital de Messejana Dr. Carlos	Lesão em septo nasal por uso do de CPAP	Concluída	Eventos Adversos Relacionados à Assistênci	Lesão por pressão		
23/03/2021	CE	FORTALEZA	Hospital de Messejana Dr. Carlos	AO SAIR DO BANHEIRO O PACIENT	Enviada	Eventos Adversos Relacionados à Assistênci	Queda do paciente		
01/04/2021	CE	FORTALEZA	Hospital de Messejana Dr. Carlos	SENTIU TONTURA, SENTOU-SE NO	Enviada	Eventos Adversos Relacionados à Assistênci	Queda do paciente		
01/04/2021	CE	FORTALEZA	Hospital de Messejana Dr. Carlos	Fluxo de atendimento do pacientes com LP	Concluída	Eventos Adversos Relacionados à Assistênci	Lesão por pressão		
01/04/2021	CE	FORTALEZA	Hospital de Messejana Dr. Carlos	Paciente iniciou hiperemia em região	Enviada	Eventos Adversos Relacionados à Assistênci	Lesão por pressão		

01/04/2021	CE	FORTALEZA	Hospital de Messejana Dr. Carlos	Paciente apresentou hiperemia	Enviada	Eventos Adversos Relacionados à Assistênci	Lesão por pressão		
05/04/2021	CE	FORTALEZA	Hospital de Messejana Dr. Carlos	Lesão por dispositivo causada	Enviada	Eventos Adversos Relacionados à Assistênci	Lesão por pressão		
05/04/2021	CE	FORTALEZA	Hospital de Messejana Dr. Carlos	Lesão estágio 2 em sacra	Enviada	Eventos Adversos Relacionados à Assistênci	Lesão por pressão		
05/04/2021	CE	FORTALEZA	Hospital de Messejana Dr. Carlos	apresentando lp nao classificavel em	Enviada	Eventos Adversos Relacionados à Assistênci	Lesão por pressão		
06/04/2021	CE	FORTALEZA	Hospital de Messejana Dr. Carlos	HIPEREMIA NÃO BRANQUEÁVEL EM	Enviada	Eventos Adversos Relacionados à Assistênci	Lesão por pressão		
06/04/2021	CE	FORTALEZA	Hospital de Messejana Dr. Carlos	Lesão por pressão estágio 2, aplicado	Enviada	Eventos Adversos Relacionados à Assistênci	Lesão por pressão		
12/04/2021	CE	FORTALEZA	Hospital de Messejana Dr. Carlos	IDENTIFICADO LP EM OCCIPIT	Enviada	Eventos Adversos Relacionados à Assistênci	Lesão por pressão		
12/04/2021	CE	FORTALEZA	Hospital de Messejana Dr. Carlos	PACIENTE COM PEQUENA LESÃO EM	Enviada	Eventos Adversos Relacionados à Assistênci	Lesão por pressão		
13/04/2021	CE	FORTALEZA	Hospital de Messejana Dr. Carlos	Paciente desorientado, durante a plantão	Em retificação	Eventos Adversos Relacionados à Assistênci	Queda do paciente		
14/04/2021	CE	FORTALEZA	Hospital de Messejana Dr. Carlos	Lesão por pressão estágio 1	Enviada	Eventos Adversos Relacionados à Assistênci	Lesão por pressão		
16/04/2021	CE	FORTALEZA	Hospital de Messejana Dr. Carlos	Lesão por pressão estágio 2	Enviada	Eventos Adversos Relacionados à Assistênci	Lesão por pressão		
29/04/2021	CE	FORTALEZA	Hospital de Messejana Dr. Carlos	LPP região sacra estagio 2.	Enviada	Eventos Adversos Relacionados à Assistênci	Lesão por pressão		

29/04/2021	CE	FORTALEZA	Hospital de Messejana Dr. Carlos	LP EM REGIÃO SACRA NAO CLASSIFI	Enviada	Eventos Adversos Relacionados à Assistênci	Lesão por pressão		
30/04/2021	CE	FORTALEZA	Hospital de Messejana Dr. Carlos	Paciente foi ao banheiro sozinho sem	Em retificação	Eventos Adversos Relacionados à Assistênci	Queda do paciente		
04/05/2021	CE	FORTALEZA	Hospital de Messejana Dr. Carlos	Paciente adimita na UTI procedente da	Enviada	Eventos Adversos Relacionados à Assistênci	Lesão por pressão		
04/05/2021	CE	FORTALEZA	Hospital de Messejana Dr. Carlos	PACIENTE APRESENTANDO PERDA	Enviada	Eventos Adversos Relacionados à Assistênci	Lesão por pressão		
06/05/2021	CE	FORTALEZA	Hospital de Messejana Dr. Carlos	SÃO EM REGIÃO SACRA NÃO CLASSIFI	Enviada	Eventos Adversos Relacionados à Assistênci	Lesão por pressão		
06/05/2021	CE	FORTALEZA	Hospital de Messejana Dr. Carlos	Lesão sacro glúteo	Enviada	Eventos Adversos Relacionados à Assistênci	Lesão por pressão		
12/05/2021	CE	FORTALEZA	Hospital de Messejana Dr. Carlos	Escorregou ao tentar sair do leito	Enviada	Eventos Adversos Relacionados à Assistênci	Queda do paciente		
12/05/2021	CE	FORTALEZA	Hospital de Messejana Dr. Carlos	LP estágio 2 em sacra, pequenas	Enviada	Eventos Adversos Relacionados à Assistênci	Lesão por pressão		
13/05/2021	CE	FORTALEZA	Hospital de Messejana Dr. Carlos	Paciente solicitou ida ao banheiro ao	Enviada	Eventos Adversos Relacionados à Assistênci	Queda do paciente		
17/05/2021	CE	FORTALEZA	Hospital de Messejana Dr. Carlos	LP EM REGIÃO SACRA ESTÁGIO 2. LP	Enviada	Eventos Adversos Relacionados à Assistênci	Lesão por pressão		
17/05/2021	CE	FORTALEZA	Hospital de Messejana Dr. Carlos	PACIENTE COM COVID (+), RESTRIT	Enviada	Eventos Adversos Relacionados à Assistênci	Lesão por pressão		
17/05/2021	CE	FORTALEZA	Hospital de Messejana Dr. Carlos	LP EM TROCANTER D (PEQUENAS	Enviada	Eventos Adversos Relacionados à Assistênci	Lesão por pressão		

17/05/2021	CE	FORTALEZA	Hospital de Messejana Dr. Carlos	PEQUENA PERDA EPIDÉRMICA NA	Enviada	Eventos Adversos Relacionados à Assistênci	Lesão por pressão		
17/05/2021	CE	FORTALEZA	Hospital de Messejana Dr. Carlos	paciente admitido na UCP proveniente da	Enviada	Eventos Adversos Relacionados à Assistênci	Lesão por pressão		
18/05/2021	CE	FORTALEZA	Hospital de Messejana Dr. Carlos	Paciente desorientada, com função renal	Enviada	Eventos Adversos Relacionados à Assistênci	Queda do paciente		
18/05/2021	CE	FORTALEZA	Hospital de Messejana Dr. Carlos	Sofreu queda ao tentar levantar-se	Enviada	Eventos Adversos Relacionados à Assistênci	Queda do paciente		
21/05/2021	CE	FORTALEZA	Hospital de Messejana Dr. Carlos	PACIENTE ADMITIDO NA SPCR	Enviada	Eventos Adversos Relacionados à Assistênci	Lesão por pressão		
21/05/2021	CE	FORTALEZA	Hospital de Messejana Dr. Carlos	Lp Sacra não classificável/ trocanter	Enviada	Eventos Adversos Relacionados à Assistênci	Lesão por pressão		
21/05/2021	CE	FORTALEZA	Hospital de Messejana Dr. Carlos	IDENTIFICADO LP ESTÁGIO 2	Enviada	Eventos Adversos Relacionados à Assistênci	Lesão por pressão		
21/05/2021	CE	FORTALEZA	Hospital de Messejana Dr. Carlos	Paciente admitido na UCP proveniente da	Enviada	Eventos Adversos Relacionados à Assistênci	Lesão por pressão		
21/05/2021	CE	FORTALEZA	Hospital de Messejana Dr. Carlos	Paciente diabética com lesão em região de	Em investigação	Eventos Adversos Relacionados à Assistênci	Lesão por pressão		
21/05/2021	CE	FORTALEZA	Hospital de Messejana Dr. Carlos	LP EM SACRA EXTENSA BORDAS	Enviada	Eventos Adversos Relacionados à Assistênci	Lesão por pressão		
24/05/2021	CE	FORTALEZA	Hospital de Messejana Dr. Carlos	Paciente ao ir ao banheiro tropeçou na porta	Enviada	Eventos Adversos Relacionados à Assistênci	Queda do paciente		
25/05/2021	CE	FORTALEZA	Hospital de Messejana Dr. Carlos	PACIENTE ADMITIDA APRESE	Enviada	Eventos Adversos Relacionados à Assistênci	Lesão por pressão		

25/05/2021	CE	FORTALEZA	Hospital de Messejana Dr. Carlos	Lesão Identificada e notificada.	Enviada	Eventos Adversos Relacionados à Assistênci	Lesão por pressão			
25/05/2021	CE	FORTALEZA	Hospital de Messejana Dr. Carlos	LESÃO POR QUEIMADURA, PROVÁV	Enviada	Eventos Adversos Relacionados à Assistênci	Queimadura de paciente			
26/05/2021	CE	FORTALEZA	Hospital de Messejana Dr. Carlos	LP EM REGIÃO SACRA ESTAGIO II DE	Enviada	Eventos Adversos Relacionados à Assistênci	Lesão por pressão			
26/05/2021	CE	FORTALEZA	Hospital de Messejana Dr. Carlos	Lesão regiao interglutea e glutea	Enviada	Eventos Adversos Relacionados à Assistênci	Lesão por pressão			
26/05/2021	CE	FORTALEZA	Hospital de Messejana Dr. Carlos	LP ESTÁGIO 2 EM PANTURRILHA E	Enviada	Eventos Adversos Relacionados à Assistênci	Lesão por pressão			
26/05/2021	CE	FORTALEZA	Hospital de Messejana Dr. Carlos	PACIENTE ADMITIDA COM LESÃO	Enviada	Eventos Adversos Relacionados à Assistênci	Lesão por pressão			
26/05/2021	CE	FORTALEZA	Hospital de Messejana Dr. Carlos	Paciente com lesão tissular profunda	Enviada	Eventos Adversos Relacionados à Assistênci	Lesão por pressão			
27/05/2021	CE	FORTALEZA	Hospital de Messejana Dr. Carlos	Paciente encontrava-se sentada em	Enviada	Eventos Adversos Relacionados à Assistênci	Queda do paciente			
04/06/2021	CE	FORTALEZA	Hospital de Messejana Dr. Carlos	Ao sentar no leito paciente teveperda de	Enviada	Eventos Adversos Relacionados à Assistênci	Queda do paciente			
04/06/2021	CE	FORTALEZA	Hospital de Messejana Dr. Carlos	Paciente orientada, porém pouco colaborati	Enviada	Eventos Adversos Relacionados à Assistênci	Queda do paciente			
08/06/2021	CE	FORTALEZA	Hospital de Messejana Dr. Carlos	Paciente após realização de clister foi	Enviada	Eventos Adversos Relacionados à Assistênci	Queda do paciente			
10/06/2021	CE	FORTALEZA	Hospital de Messejana Dr. Carlos	Paciente tentou levantar-se do leito	Enviada	Eventos Adversos Relacionados à Assistênci	Queda do paciente			

10/06/2021	CE	FORTALEZA	Hospital de Messejana Dr. Carlos	PACIENTE APRESENTA NA REGIÃO	Enviada	Eventos Adversos Relacionados à Assistênci	Lesão por pressão		
10/06/2021	CE	FORTALEZA	Hospital de Messejana Dr. Carlos	Paciente encontrava-se sentado na	Enviada	Eventos Adversos Relacionados à Assistênci	Queda do paciente		
14/06/2021	CE	FORTALEZA	Hospital de Messejana Dr. Carlos	APRESENTA LESÃO NÃO CLASSIFI	Em investigação	Eventos Adversos Relacionados à Assistênci	Lesão por pressão		
14/06/2021	CE	FORTALEZA	Hospital de Messejana Dr. Carlos	LESÃO ESTAGIO 2 EM REGIÃO SACRA	Enviada	Eventos Adversos Relacionados à Assistênci	Lesão por pressão		
15/06/2021	CE	FORTALEZA	Hospital de Messejana Dr. Carlos	Paciente apesar de todas sa orientação	Enviada	Eventos Adversos Relacionados à Assistênci	Queda do paciente		
17/06/2021	CE	FORTALEZA	Hospital de Messejana Dr. Carlos	Avaliação de enfermagem pois adimissão	Em investigação	Eventos Adversos Relacionados à Assistênci	Lesão por pressão		
22/06/2021	CE	FORTALEZA	Hospital de Messejana Dr. Carlos	lesão em calcaneo direito formação de bolha	Enviada	Eventos Adversos Relacionados à Assistênci	Lesão por pressão		
29/06/2021	CE	FORTALEZA	Hospital de Messejana Dr. Carlos	LESÃO GRAU 2, TENDO ROMPIMENTO	Enviada	Eventos Adversos Relacionados à Assistênci	Lesão por pressão		
29/06/2021	CE	FORTALEZA	Hospital de Messejana Dr. Carlos	LESÃO GRAL 2, TENDO ROMPIMENTO	Enviada	Eventos Adversos Relacionados à Assistênci	Lesão por pressão		
30/06/2021	CE	FORTALEZA	Hospital de Messejana Dr. Carlos	apresenta LPP em sacra não classificável com	Enviada	Eventos Adversos Relacionados à Assistênci	Lesão por pressão		
30/06/2021	CE	FORTALEZA	Hospital de Messejana Dr. Carlos	LPP em sacra e glúteos extensa, est. 2	Enviada	Eventos Adversos Relacionados à Assistênci	Lesão por pressão		
05/07/2021	CE	FORTALEZA	Hospital de Messejana Dr. Carlos	APRESENTANDO LESÃO ESTÁGIO 4 EM	Em investigação	Eventos Adversos Relacionados à Assistênci	Lesão por pressão		

05/07/2021	CE	FORTALEZA	Hospital de Messejana Dr. Carlos	LP EM ORIFÍCIO NASAL DIREITO E BOLHA	Enviada	Eventos Adversos Relacionados à Assistênci	Lesão por pressão		
05/07/2021	CE	FORTALEZA	Hospital de Messejana Dr. Carlos	lp sacra estagio ii	Enviada	Eventos Adversos Relacionados à Assistênci	Lesão por pressão		
05/07/2021	CE	FORTALEZA	Hospital de Messejana Dr. Carlos	LPP em sacra e glúteos extensa, est. 2	Enviada	Eventos Adversos Relacionados à Assistênci	Lesão por pressão		
07/07/2021	CE	FORTALEZA	Hospital de Messejana Dr. Carlos	LTP EM REGIÃO SACRA E ESTÁGIO	Enviada	Eventos Adversos Relacionados à Assistênci	Lesão por pressão		
07/07/2021	CE	FORTALEZA	Hospital de Messejana Dr. Carlos	Lesão em sacra com placa e tecidos	Enviada	Eventos Adversos Relacionados à Assistênci	Lesão por pressão		
13/07/2021	CE	FORTALEZA	Hospital de Messejana Dr. Carlos	LP em região sacra: Ñ Classificavel por	Enviada	Eventos Adversos Relacionados à Assistênci	Lesão por pressão		
13/07/2021	CE	FORTALEZA	Hospital de Messejana Dr. Carlos	PACIENTE EMAGRECIDO, COM	Enviada	Eventos Adversos Relacionados à Assistênci	Lesão por pressão		
13/07/2021	CE	FORTALEZA	Hospital de Messejana Dr. Carlos	LP NA REGIÃO SACRA ESTAGIO	Em investigação	Eventos Adversos Relacionados à Assistênci	Lesão por pressão		
15/07/2021	CE	FORTALEZA	Hospital de Messejana Dr. Carlos	Paciente apresentou queda ao levantar-	Enviada	Eventos Adversos Relacionados à Assistênci	Queda do paciente		
16/07/2021	CE	FORTALEZA	Hospital de Messejana Dr. Carlos	LP NÃO CLASSIFICAVEL EM REGIÃO	Enviada	Eventos Adversos Relacionados à Assistênci	Lesão por pressão		
16/07/2021	CE	FORTALEZA	Hospital de Messejana Dr. Carlos	LESÃO EM REGIÃO SACRAL COM	Enviada	Eventos Adversos Relacionados à Assistênci	Lesão por pressão		
16/07/2021	CE	FORTALEZA	Hospital de Messejana Dr. Carlos	OCCIPITAL (TISSULAR PROFUN	Em investigação	Eventos Adversos Relacionados à Assistênci	Lesão por pressão		

20/07/2021	CE	FORTALEZA	Hospital de Messejana Dr. Carlos	Paciente apresentando hipotensão	Enviada	Eventos Adversos Relacionados à Assistênci	Queda do paciente		
09/08/2021	CE	FORTALEZA	Hospital de Messejana Dr. Carlos	Paciente tentou levantar-se do leito	Enviada	Eventos Adversos Relacionados à Assistênci	Queda do paciente		
30/08/2021	CE	FORTALEZA	Hospital de Messejana Dr. Carlos	Administração de dieta por sonda orogástric	Enviada	Eventos Adversos Relacionados à Assistênci	Falhas na administração de dietas		Enteral (oral, cateter oral ou nasal
02/09/2021	CE	FORTALEZA	Hospital de Messejana Dr. Carlos	Paciente desorientado, agitado, tentou	Enviada	Eventos Adversos Relacionados à Assistênci	Queda do paciente		
08/09/2021	CE	FORTALEZA	Hospital de Messejana Dr. Carlos	Paciente tentou levantar-se da cama	Enviada	Eventos Adversos Relacionados à Assistênci	Queda do paciente		
08/09/2021	CE	FORTALEZA	Hospital de Messejana Dr. Carlos	Paciente foi ao banheiro com a acompanh	Enviada	Eventos Adversos Relacionados à Assistênci	Queda do paciente		
08/09/2021	CE	FORTALEZA	Hospital de Messejana Dr. Carlos	Paciente sem acompanhante, levantou-	Enviada	Eventos Adversos Relacionados à Assistênci	Queda do paciente		
10/09/2021	CE	FORTALEZA	Hospital de Messejana Dr. Carlos	Paciente caiu da cadeira de banho	Enviada	Eventos Adversos Relacionados à Assistênci	Queda do paciente		
10/09/2021	CE	FORTALEZA	Hospital de Messejana Dr. Carlos	Paciente sentado na poltrona, levantou-	Enviada	Eventos Adversos Relacionados à Assistênci	Queda do paciente		
23/09/2021	CE	FORTALEZA	Hospital de Messejana Dr. Carlos	Paciente admitido com o número de	Enviada	Eventos Adversos Relacionados à Assistênci	Falha na identificação do paciente		
23/09/2021	CE	FORTALEZA	Hospital de Messejana Dr. Carlos	Pulseira presente, porém com identificac	Enviada	Eventos Adversos Relacionados à Assistênci	Falha na identificação do paciente		
28/09/2021	CE	FORTALEZA	Hospital de Messejana Dr. Carlos	Ao realizar Rx de paciente, técnico	Enviada	Eventos Adversos Relacionados à Assistênci	Falha na identificação do paciente		

05/11/2021	CE	FORTALEZA	Hospital de Messejana Dr. Carlos	Paciente encaminhada ao banheiro, pela	Enviada	Eventos Adversos Relacionados à Assistênci	Queda do paciente		
09/11/2021	CE	FORTALEZA	Hospital de Messejana Dr. Carlos	Paciente após saída da acompanhante	Enviada	Eventos Adversos Relacionados à Assistênci	Queda do paciente		
11/11/2021	CE	FORTALEZA	Hospital de Messejana Dr. Carlos	Paciente levantou-se sozinho do leito	Enviada	Eventos Adversos Relacionados à Assistênci	Queda do paciente		
22/11/2021	CE	FORTALEZA	Hospital de Messejana Dr. Carlos	Paciente ao deambular sem sua bengala	Enviada	Eventos Adversos Relacionados à Assistênci	Queda do paciente		
22/11/2021	CE	FORTALEZA	Hospital de Messejana Dr. Carlos	SOFREU QUEDA DA PROPRIA ALTURA	Enviada	Eventos Adversos Relacionados à Assistênci	Queda do paciente		
09/12/2021	CE	FORTALEZA	Hospital de Messejana Dr. Carlos	Caiu ao tentar levantar-se sozinho	Enviada	Eventos Adversos Relacionados à Assistênci	Queda do paciente		
22/12/2021	CE	FORTALEZA	Hospital de Messejana Dr. Carlos	Enquanto dormia paciente caiu do leito	Enviada	Eventos Adversos Relacionados à Assistênci	Queda do paciente		
22/12/2021	CE	FORTALEZA	Hospital de Messejana Dr. Carlos	Paciente tentou levantar-se do leito	Enviada	Eventos Adversos Relacionados à Assistênci	Queda do paciente		
22/12/2021	CE	FORTALEZA	Hospital de Messejana Dr. Carlos	Ao levantar-se para ir ao banheiro	Enviada	Eventos Adversos Relacionados à Assistênci	Queda do paciente		
22/12/2021	CE	FORTALEZA	Hospital de Messejana Dr. Carlos	Quebra de barreira de precaução	Enviada	Eventos Adversos Relacionados à Assistênci	Falhas durante a assistência à saúde		
27/12/2021	CE	FORTALEZA	Hospital de Messejana Dr. Carlos	Paciente foi ao banheiro com apoio do	Enviada	Eventos Adversos Relacionados à Assistênci	Queda do paciente		
27/12/2021	CE	FORTALEZA	Hospital de Messejana Dr. Carlos	Foram nomeados dois RX com o mesmo	Enviada	Eventos Adversos Relacionados à Assistênci	Falha na identificação do paciente		

27/12/2021	CE	FORTALEZA	Hospital de Messejana Dr. Carlos	PACIENTE CAIU ENQUANTO DORMIA	Enviada	Eventos Adversos Relacionados à Assistênci	Queda do paciente		
27/12/2021	CE	FORTALEZA	Hospital de Messejana Dr. Carlos	Acompanhante encaminhou paciente	Enviada	Eventos Adversos Relacionados à Assistênci	Queda do paciente		
03/01/2022	CE	FORTALEZA	Hospital de Messejana Dr. Carlos	Paciente levantou-se para urinar beira	Enviada	Eventos Adversos Relacionados à Assistênci	Queda do paciente		
03/01/2022	CE	FORTALEZA	Hospital de Messejana Dr. Carlos	Paciente levantou-se do leito, desequilib	Enviada	Eventos Adversos Relacionados à Assistênci	Queda do paciente		
21/01/2022	CE	FORTALEZA	Hospital de Messejana Dr. Carlos	Paciente encaminhou-se ao banheiro e	Enviada	Eventos Adversos Relacionados à Assistênci	Queda do paciente		
21/02/2022	CE	FORTALEZA	Hospital de Messejana Dr. Carlos	Na tentativa de sair do leito para cadeira	Enviada	Eventos Adversos Relacionados à Assistênci	Queda do paciente		
21/02/2022	CE	FORTALEZA	Hospital de Messejana Dr. Carlos	Síncope no banheiro. Apresentou	Enviada	Eventos Adversos Relacionados à Assistênci	Queda do paciente		
22/02/2022	CE	FORTALEZA	Hospital de Messejana Dr. Carlos	PACIENTE RESTRITA AO LEITO	Enviada	Eventos Adversos Relacionados à Assistênci	Queda do paciente		
22/02/2022	CE	FORTALEZA	Hospital de Messejana Dr. Carlos	PACIENTE DEFICIENTE FÍSICO	Enviada	Eventos Adversos Relacionados à Assistênci	Queda do paciente		
28/02/2022	CE	FORTALEZA	Hospital de Messejana Dr. Carlos	PACIENTE RELATOU QUE FOI AO	Enviada	Eventos Adversos Relacionados à Assistênci	Queda do paciente		
04/03/2022	CE	FORTALEZA	Hospital de Messejana Dr. Carlos	Lesão plana com necrose.	Enviada	Eventos Adversos Relacionados à Assistênci	Lesão por pressão		
17/03/2022	CE	FORTALEZA	Hospital de Messejana Dr. Carlos	Pulseiras de identificação não estavam	Enviada	Eventos Adversos Relacionados à Assistênci	Falha na identificação do paciente		

30/03/2022	CE	FORTALEZA	Hospital de Messejana Dr. Carlos	paciente relata que ao tentar levantar	Enviada	Eventos Adversos Relacionados à Assistênci	Queda do paciente		
30/03/2022	CE	FORTALEZA	Hospital de Messejana Dr. Carlos	PACIENTE LEVANTOU DO LEITO	Enviada	Eventos Adversos Relacionados à Assistênci	Queda do paciente		
12/04/2022	CE	FORTALEZA	Hospital de Messejana Dr. Carlos	PACIENTE TRANSFERIDA DO	Enviada	Eventos Adversos Relacionados à Assistênci	Falha na identificação do paciente		
25/04/2022	CE	FORTALEZA	Hospital de Messejana Dr. Carlos	evento adverso	Enviada	Eventos Adversos Relacionados à Assistênci	Falhas durante a assistência à saúde		
13/05/2022	CE	FORTALEZA	Hospital de Messejana Dr. Carlos	CRIANÇA AGITADA NA HORA	Enviada	Eventos Adversos Relacionados à Assistênci	Queda do paciente		
24/05/2022	CE	FORTALEZA	Hospital de Messejana Dr. Carlos	As 03:45h paciente estava no leito	Concluída	Eventos Adversos Relacionados à Assistênci	Queda do paciente		
25/05/2022	CE	FORTALEZA	Hospital de Messejana Dr. Carlos	QUEDA POR DESEQUILÍBRIO	Enviada	Eventos Adversos Relacionados à Assistênci	Queda do paciente		
06/06/2022	CE	FORTALEZA	Hospital de Messejana Dr. Carlos	ADMITIDA DA SPCR COM ISQUEMI	Enviada	Eventos Adversos Relacionados à Assistênci	Lesão por pressão		
07/06/2022	CE	FORTALEZA	Hospital de Messejana Dr. Carlos	PACIENTE ADMITIDO ÀS 14:00H	Enviada	Eventos Adversos Relacionados à Assistênci	Falha na identificação do paciente		
20/06/2022	CE	FORTALEZA	Hospital de Messejana Dr. Carlos	TROCA DE PACIENTES A SER	Enviada	Eventos Adversos Relacionados à Assistênci	Falha na identificação do paciente		
20/06/2022	CE	FORTALEZA	Hospital de Messejana Dr. Carlos	PACIENTE CONVULSIONOU CAUSAN	Enviada	Eventos Adversos Relacionados à Assistênci	Queda do paciente		
22/06/2022	CE	FORTALEZA	Hospital de Messejana Dr. Carlos	PACIENTE SOFREU PCR DURANT	Concluída	Eventos Adversos Relacionados à Assistênci	Falhas durante a assistência à saúde		

06/07/2022	CE	FORTALEZA	Hospital de Messejana Dr. Carlos	lesão por pressão em região sacra	Enviada	Eventos Adversos Relacionados à Assistênci	Lesão por pressão		
06/07/2022	CE	FORTALEZA	Hospital de Messejana Dr. Carlos	LESÃO POR PRESSÃO REGIÃO	Enviada	Eventos Adversos Relacionados à Assistênci	Lesão por pressão		
06/07/2022	CE	FORTALEZA	Hospital de Messejana Dr. Carlos	lesão por pressão sacra	Enviada	Eventos Adversos Relacionados à Assistênci	Lesão por pressão		
07/07/2022	CE	FORTALEZA	Hospital de Messejana Dr. Carlos	ACOMPANHANTE RELATA QUE AO IR AO	Enviada	Eventos Adversos Relacionados à Assistênci	Queda do paciente		
07/07/2022	CE	FORTALEZA	Hospital de Messejana Dr. Carlos	PACIENTE CONVULSIONOU CAUSAN	Enviada	Eventos Adversos Relacionados à Assistênci	Queda do paciente		
07/07/2022	CE	FORTALEZA	Hospital de Messejana Dr. Carlos	PACIENTE SEM PULSEIRA DE IDENTIFI	Enviada	Eventos Adversos Relacionados à Assistênci	Falha na identificação do paciente		
07/07/2022	CE	FORTALEZA	Hospital de Messejana Dr. Carlos	PACIENTE SEM PULSEIRA DE IDENTIFI	Enviada	Eventos Adversos Relacionados à Assistênci	Falha na identificação do paciente		
07/07/2022	CE	FORTALEZA	Hospital de Messejana Dr. Carlos	PACIENTE SEM PULSEIRA DE IDENTIFI	Enviada	Eventos Adversos Relacionados à Assistênci	Falha na identificação do paciente		
13/07/2022	CE	FORTALEZA	Hospital de Messejana Dr. Carlos	PERDA PARCIAL DA ESPESSURA	Enviada	Eventos Adversos Relacionados à Assistênci	Lesão por pressão		
15/07/2022	CE	FORTALEZA	Hospital de Messejana Dr. Carlos	LESÃO TISSULAR EM REGIAO SACRA	Enviada	Eventos Adversos Relacionados à Assistênci	Lesão por pressão		
15/07/2022	CE	FORTALEZA	Hospital de Messejana Dr. Carlos	APRESENTA LP EM REGIÃO SACRA	Enviada	Eventos Adversos Relacionados à Assistênci	Lesão por pressão		
15/07/2022	CE	FORTALEZA	Hospital de Messejana Dr. Carlos	LP EM SACRA ESTAGIO 2	Enviada	Eventos Adversos Relacionados à Assistênci	Lesão por pressão		

15/07/2022	CE	FORTALEZA	Hospital de Messejana Dr. Carlos	UMA LEVE LESAO TISSULAR	Enviada	Eventos Adversos Relacionados à Assistênci	Lesão por pressão		
28/07/2022	CE	FORTALEZA	Hospital de Messejana Dr. Carlos	PACIENTE APRESENTOU LESÃO	Enviada	Eventos Adversos Relacionados à Assistênci	Lesão por pressão		
29/07/2022	CE	FORTALEZA	Hospital de Messejana Dr. Carlos	PACIENTE DEU ENTRADA NA UNIDADE	Enviada	Eventos Adversos Relacionados à Assistênci	Lesão por pressão		
29/07/2022	CE	FORTALEZA	Hospital de Messejana Dr. Carlos	PACIENTE ADMITIDO NO RISCO	Enviada	Eventos Adversos Relacionados à Assistênci	Lesão por pressão		
29/07/2022	CE	FORTALEZA	Hospital de Messejana Dr. Carlos	Paciente grave aguardando transporte	Enviada	Eventos Adversos Relacionados à Assistênci	Falhas nas atividades administra		
02/08/2022	CE	FORTALEZA	Hospital de Messejana Dr. Carlos	PACIENTE SEM ACOMPANHANTE,	Enviada	Eventos Adversos Relacionados à Assistênci	Queda do paciente		
29/08/2022	CE	FORTALEZA	Hospital de Messejana Dr. Carlos	PACIENTE APRESENTANDO TOSSE,	Enviada	Eventos Adversos Relacionados à Assistênci	Queda do paciente		
30/08/2022	CE	FORTALEZA	Hospital de Messejana Dr. Carlos	ACOMPANHANTE VEIO CHAMAR A	Enviada	Eventos Adversos Relacionados à Assistênci	Queda do paciente		
30/08/2022	CE	FORTALEZA	Hospital de Messejana Dr. Carlos	PACIENTE APRESENTANDO TOSSE,	Enviada	Eventos Adversos Relacionados à Assistênci	Queda do paciente		
30/08/2022	CE	FORTALEZA	Hospital de Messejana Dr. Carlos	PACIENTE PERDEU O EQUILIB	Enviada	Eventos Adversos Relacionados à Assistênci	Queda do paciente		
30/08/2022	CE	FORTALEZA	Hospital de Messejana Dr. Carlos	Paciente escorregou do leito	Enviada	Eventos Adversos Relacionados à Assistênci	Queda do paciente		
30/08/2022	CE	FORTALEZA	Hospital de Messejana Dr. Carlos	PACIENTE LEVOU QUEDA DO LEITO	Enviada	Eventos Adversos Relacionados à Assistênci	Queda do paciente		

30/08/2022	CE	FORTALEZA	Hospital de Messejana Dr. Carlos	-	Enviada	Eventos Adversos Relacionados à Assistênci	Queda do paciente		
15/09/2022	CE	FORTALEZA	Hospital de Messejana Dr. Carlos	Paciente com tosse persistente em pé	Enviada	Eventos Adversos Relacionados à Assistênci	Queda do paciente		
15/09/2022	CE	FORTALEZA	Hospital de Messejana Dr. Carlos	Lesão de pele estágio II	Enviada	Eventos Adversos Relacionados à Assistênci	Lesão por pressão		
15/09/2022	CE	FORTALEZA	Hospital de Messejana Dr. Carlos	PACIENTE ANA ALICE MENDES, 88	Concluída	Eventos Adversos Relacionados à Assistênci	Lesão por pressão		
15/09/2022	CE	FORTALEZA	Hospital de Messejana Dr. Carlos	PERDA DE EPIDERME E LESÃO	Concluída	Eventos Adversos Relacionados à Assistênci	Lesão por pressão		
15/09/2022	CE	FORTALEZA	Hospital de Messejana Dr. Carlos	-	Enviada	Eventos Adversos Relacionados à Assistênci	Lesão por pressão		
15/09/2022	CE	FORTALEZA	Hospital de Messejana Dr. Carlos	Apresenta hiperemia em sacra na	Enviada	Eventos Adversos Relacionados à Assistênci	Lesão por pressão		
15/09/2022	CE	FORTALEZA	Hospital de Messejana Dr. Carlos	LESÃO SUPERFICIAL	Enviada	Eventos Adversos Relacionados à Assistênci	Lesão por pressão		
15/09/2022	CE	FORTALEZA	Hospital de Messejana Dr. Carlos	LESÃO TISSULAR EXTENSA NÃO	Concluída	Eventos Adversos Relacionados à Assistênci	Lesão por pressão		
15/09/2022	CE	FORTALEZA	Hospital de Messejana Dr. Carlos	Pact proveniente da SPCR com	Concluída	Eventos Adversos Relacionados à Assistênci	Lesão por pressão		
20/09/2022	CE	FORTALEZA	Hospital de Messejana Dr. Carlos	QUEDA DENTRO DO BANHEIRO NO	Enviada	Eventos Adversos Relacionados à Assistênci	Queda do paciente		
20/09/2022	CE	FORTALEZA	Hospital de Messejana Dr. Carlos	PACIENTE APRESENTOU HIPOTEN	Enviada	Eventos Adversos Relacionados à Assistênci	Queda do paciente		

22/09/2022	CE	FORTALE ZA	Hospital de Messejan a Dr. Carlos	CADEIRA DE PLASTIC O	Enviada	Eventos Adversos Relaciona dos à Assistênci	Queda do paciente		
22/09/2022	CE	FORTALE ZA	Hospital de Messejan a Dr. Carlos	FAMILIAR EPOUSA VANAÁR EADAEN FERMARI	Enviada	Eventos Adversos Relaciona dos à Assistênci	Queda do paciente		
22/09/2022	CE	FORTALE ZA	Hospital de Messejan a Dr. Carlos	PACIENT E,CAIU DA PRÓPRIA ALTURA	Enviada	Eventos Adversos Relaciona dos à Assistênci	Queda do paciente		
28/09/2022	CE	FORTALE ZA	Hospital de Messejan a Dr. Carlos	Paciente sem acompanh ante estava	Enviada	Eventos Adversos Relaciona dos à Assistênci	Queda do paciente		
18/10/2022	CE	FORTALE ZA	Hospital de Messejan a Dr. Carlos	PACIENT E FOI AO BANHO ESCORR	Enviada	Eventos Adversos Relaciona dos à Assistênci	Queda do paciente		
18/10/2022	CE	FORTALE ZA	Hospital de Messejan a Dr. Carlos	DURANT EOPLAN TÃONOT URNOOP ACIENTE	Enviada	Eventos Adversos Relaciona dos à Assistênci	Queda do paciente		
18/10/2022	CE	FORTALE ZA	Hospital de Messejan a Dr. Carlos	PACIENT E RELATA QUE ESTAVA	Enviada	Eventos Adversos Relaciona dos à Assistênci	Queda do paciente		
18/10/2022	CE	FORTALE ZA	Hospital de Messejan a Dr. Carlos	ACOMPA NHANTE S RELATA M QUE	Enviada	Eventos Adversos Relaciona dos à Assistênci	Queda do paciente		
18/10/2022	CE	FORTALE ZA	Hospital de Messejan a Dr. Carlos	PACIENT E FOI AO BANHEIR O SEM	Enviada	Eventos Adversos Relaciona dos à Assistênci	Queda do paciente		
19/10/2022	CE	FORTALE ZA	Hospital de Messejan a Dr. Carlos	SOU INTERPE LADO PELA ACOMPA	Enviada	Eventos Adversos Relaciona dos à Assistênci	Queda do paciente		
19/10/2022	CE	FORTALE ZA	Hospital de Messejan a Dr. Carlos	PACIENT E AO TENTAR IR LEVANTA	Enviada	Eventos Adversos Relaciona dos à Assistênci	Queda do paciente		
25/10/2022	CE	FORTALE ZA	Hospital de Messejan a Dr. Carlos	PACIENT E AGITADA , DESORIE	Enviada	Eventos Adversos Relaciona dos à Assistênci	Queda do paciente		

31/10/2022	CE	FORTALEZA	Hospital de Messejana Dr. Carlos	lesão por fricção	Enviada	Eventos Adversos Relacionados à Assistênci	Lesão por pressão		
03/11/2022	CE	FORTALEZA	Hospital de Messejana Dr. Carlos	PACIENTE DESORIENTADO TEM	Enviada	Eventos Adversos Relacionados à Assistênci	Queda do paciente		
03/11/2022	CE	FORTALEZA	Hospital de Messejana Dr. Carlos	PACIENTE ESTAVA SENTADA, FOI	Enviada	Eventos Adversos Relacionados à Assistênci	Queda do paciente		
11/11/2022	CE	FORTALEZA	Hospital de Messejana Dr. Carlos	PACIENTE CAI DA PRÓPRIA MACA DURANT	Enviada	Eventos Adversos Relacionados à Assistênci	Queda do paciente		
11/11/2022	CE	FORTALEZA	Hospital de Messejana Dr. Carlos	PACIENTE CONSCIENTE E ORIENTA	Enviada	Eventos Adversos Relacionados à Assistênci	Queda do paciente		
11/11/2022	CE	FORTALEZA	Hospital de Messejana Dr. Carlos	PACIENTE AO TENTAR SE LEVANTA	Enviada	Eventos Adversos Relacionados à Assistênci	Queda do paciente		
11/11/2022	CE	FORTALEZA	Hospital de Messejana Dr. Carlos	PACIENTE CAIU DA POLTRONA (	Enviada	Eventos Adversos Relacionados à Assistênci	Queda do paciente		
11/11/2022	CE	FORTALEZA	Hospital de Messejana Dr. Carlos	-	Enviada	Eventos Adversos Relacionados à Assistênci	Queda do paciente		
17/11/2022	CE	FORTALEZA	Hospital de Messejana Dr. Carlos	PACIENTE APRESENTA MEMBRO	Enviada	Eventos Adversos Relacionados à Assistênci	Queda do paciente		
17/11/2022	CE	FORTALEZA	Hospital de Messejana Dr. Carlos	PACIENTE APÓS REALIZAR ESFORÇ	Enviada	Eventos Adversos Relacionados à Assistênci	Queda do paciente		
17/11/2022	CE	FORTALEZA	Hospital de Messejana Dr. Carlos	PACIENTE COM NÁUSEAS, REFERIU	Enviada	Eventos Adversos Relacionados à Assistênci	Queda do paciente		
24/11/2022	CE	FORTALEZA	Hospital de Messejana Dr. Carlos	PACIENTE CAIU DE JOELHOS	Enviada	Eventos Adversos Relacionados à Assistênci	Queda do paciente		

02/12/2022	CE	FORTALEZA	Hospital de Messejana Dr. Carlos	PACIENTE LEVANTOU-SE DO	Enviada	Eventos Adversos Relacionados à Assistênci	Queda do paciente		
02/12/2022	CE	FORTALEZA	Hospital de Messejana Dr. Carlos	PACIENTE AO SAIR DO BANHEIRO	Enviada	Eventos Adversos Relacionados à Assistênci	Queda do paciente		
02/12/2022	CE	FORTALEZA	Hospital de Messejana Dr. Carlos	AS 07:20 HORAS PACIENTE SOFRE	Enviada	Eventos Adversos Relacionados à Assistênci	Queda do paciente		
02/12/2022	CE	FORTALEZA	Hospital de Messejana Dr. Carlos	PACIENTE REFERIU TER IDO AO	Enviada	Eventos Adversos Relacionados à Assistênci	Queda do paciente		
12/12/2022	CE	FORTALEZA	Hospital de Messejana Dr. Carlos	-	Enviada	Eventos Adversos Relacionados à Assistênci	Lesão por pressão		
12/12/2022	CE	FORTALEZA	Hospital de Messejana Dr. Carlos	-	Concluída	Eventos Adversos Relacionados à Assistênci	Lesão por pressão		
12/12/2022	CE	FORTALEZA	Hospital de Messejana Dr. Carlos	LP não classificável	Enviada	Eventos Adversos Relacionados à Assistênci	Lesão por pressão		
12/12/2022	CE	FORTALEZA	Hospital de Messejana Dr. Carlos	-	Enviada	Eventos Adversos Relacionados à Assistênci	Falhas envolvendo o cateter venoso		
12/12/2022	CE	FORTALEZA	Hospital de Messejana Dr. Carlos	-	Enviada	Eventos Adversos Relacionados à Assistênci	Lesão por pressão		
22/12/2022	CE	FORTALEZA	Hospital de Messejana Dr. Carlos	LPP EM SACRA ESTÁGIO 2, EM USO DE	Enviada	Eventos Adversos Relacionados à Assistênci	Lesão por pressão		
22/12/2022	CE	FORTALEZA	Hospital de Messejana Dr. Carlos	Lesão por pressão não classificáv	Enviada	Eventos Adversos Relacionados à Assistênci	Lesão por pressão		
23/12/2022	CE	FORTALEZA	Hospital de Messejana Dr. Carlos	#FALHA NA COMUNICAÇÃO em	Enviada	Eventos Adversos Relacionados à Assistênci	Falhas durante a assistência à saúde		

23/12/2022	CE	FORTALEZA	Hospital de Messejana Dr. Carlos	RECEBO PCTE DA UNIDADE D, APRESE	Enviada	Eventos Adversos Relacionados à Assistênci	Lesão por pressão		
23/12/2022	CE	FORTALEZA	Hospital de Messejana Dr. Carlos	flebite	Enviada	Eventos Adversos Relacionados à Assistênci	Falhas envolvendo cateter venoso		
27/12/2022	CE	FORTALEZA	Hospital de Messejana Dr. Carlos	PACIENTE LUCILENE DA SILVA	Enviada	Eventos Adversos Relacionados à Assistênci	Lesão por pressão		
27/01/2023	CE	FORTALEZA	Hospital de Messejana Dr. Carlos	PACIENTE PELA MANHÃ REFERE QUE	Enviada	Eventos Adversos Relacionados à Assistênci	Queda do paciente		
27/01/2023	CE	FORTALEZA	Hospital de Messejana Dr. Carlos	PACIENTE SOFREU QUEDA DA	Enviada	Eventos Adversos Relacionados à Assistênci	Queda do paciente		
27/01/2023	CE	FORTALEZA	Hospital de Messejana Dr. Carlos	PACIENTE CAMINHANDO NA	Enviada	Eventos Adversos Relacionados à Assistênci	Queda do paciente		
27/01/2023	CE	FORTALEZA	Hospital de Messejana Dr. Carlos	PACIENTE FOI AO BANHEIR	Enviada	Eventos Adversos Relacionados à Assistênci	Queda do paciente		
27/01/2023	CE	FORTALEZA	Hospital de Messejana Dr. Carlos	PACIENTE ESTAVA COLHILANDO	Enviada	Eventos Adversos Relacionados à Assistênci	Queda do paciente		
30/01/2023	CE	FORTALEZA	Hospital de Messejana Dr. Carlos	PACIENTE RELATOU TER SOFRIDO	Enviada	Eventos Adversos Relacionados à Assistênci	Queda do paciente		
31/01/2023	CE	FORTALEZA	Hospital de Messejana Dr. Carlos	-	Enviada	Eventos Adversos Relacionados à Assistênci	Lesão por pressão		
31/01/2023	CE	FORTALEZA	Hospital de Messejana Dr. Carlos	NO PERÍODO DA MADRUGADA	Enviada	Eventos Adversos Relacionados à Assistênci	Queda do paciente		
31/01/2023	CE	FORTALEZA	Hospital de Messejana Dr. Carlos	PACIENTE DURANTE VISITA DE	Enviada	Eventos Adversos Relacionados à Assistênci	Queda do paciente		

06/02/2023	CE	FORTALEZA	Hospital de Messejana Dr. Carlos	PACIENTE ÀS 22:20 HORAS , SOFREU	Enviada	Eventos Adversos Relacionados à Assistênci	Queda do paciente		
15/02/2023	CE	FORTALEZA	Hospital de Messejana Dr. Carlos	PACIENTE LEVOU QUEDA DA PROPRIA	Enviada	Eventos Adversos Relacionados à Assistênci	Queda do paciente		
15/02/2023	CE	FORTALEZA	Hospital de Messejana Dr. Carlos	PACIENTE AO SEGUIR ANDANDO PARA	Enviada	Eventos Adversos Relacionados à Assistênci	Queda do paciente		
15/02/2023	CE	FORTALEZA	Hospital de Messejana Dr. Carlos	PACIENTE ESTAVA NA POLTRO	Enviada	Eventos Adversos Relacionados à Assistênci	Queda do paciente		
23/02/2023	CE	FORTALEZA	Hospital de Messejana Dr. Carlos	PICC ESTAVA PINÇADO NO QUAL	Enviada	Eventos Adversos Relacionados à Assistênci	Falhas envolvendo o cateter venoso		
23/02/2023	CE	FORTALEZA	Hospital de Messejana Dr. Carlos	PACIENTE COM LESÃO EM SACRA	Enviada	Eventos Adversos Relacionados à Assistênci	Lesão por pressão		
23/02/2023	CE	FORTALEZA	Hospital de Messejana Dr. Carlos	DURANTE A COLETA DE GASOME	Enviada	Eventos Adversos Relacionados à Assistênci	Falhas envolvendo o cateter venoso		
27/02/2023	CE	FORTALEZA	Hospital de Messejana Dr. Carlos	O LEITO NÃO ESTAVA COM AS RODAS	Enviada	Eventos Adversos Relacionados à Assistênci	Queda do paciente		
27/02/2023	CE	FORTALEZA	Hospital de Messejana Dr. Carlos	POR VOLTA DE 06H PACIENTE	Enviada	Eventos Adversos Relacionados à Assistênci	Queda do paciente		
27/02/2023	CE	FORTALEZA	Hospital de Messejana Dr. Carlos	PACIENTE SOFREU QUEDA DA	Enviada	Eventos Adversos Relacionados à Assistênci	Queda do paciente		
03/03/2023	CE	FORTALEZA	Hospital de Messejana Dr. Carlos	POR VOLTA DAS 14:25H PACIENT	Enviada	Eventos Adversos Relacionados à Assistênci	Queda do paciente		
03/03/2023	CE	FORTALEZA	Hospital de Messejana Dr. Carlos	-	Enviada	Eventos Adversos Relacionados à Assistênci	Queda do paciente		

09/03/2023	CE	FORTALEZA	Hospital de Messejana Dr. Carlos	POR VOLTA DAS 13H, FOMOS	Enviada	Eventos Adversos Relacionados à Assistênci	Queda do paciente		
09/03/2023	CE	FORTALEZA	Hospital de Messejana Dr. Carlos	ENCONTRAMOS O PACIENTE NO	Enviada	Eventos Adversos Relacionados à Assistênci	Queda do paciente		
28/03/2019	CE	JUAZEIRO DO NORTE	HOSPITAL REGIONA	PERDA DE SONDA	Enviada	Eventos Adversos Relaciona	Outro		
28/03/2019	CE	JUAZEIRO DO NORTE	HOSPITAL REGIONA	perda de sonda nasoenter	Enviada	Eventos Adversos Relaciona	Outro		
28/03/2019	CE	JUAZEIRO DO NORTE	HOSPITAL REGIONA	perda de acesso venoso	Enviada	Eventos Adversos Relaciona	Outro		
28/03/2019	CE	JUAZEIRO DO NORTE	HOSPITAL REGIONA	-	Enviada	Eventos Adversos Relaciona	Lesão por pressão		
28/03/2019	CE	JUAZEIRO DO NORTE	HOSPITAL REGIONA	-	Enviada	Eventos Adversos Relaciona	Lesão por pressão		
28/03/2019	CE	JUAZEIRO DO NORTE	HOSPITAL REGIONA	-	Enviada	Eventos Adversos Relaciona	Lesão por pressão		
28/03/2019	CE	JUAZEIRO DO NORTE	HOSPITAL REGIONA	-	Enviada	Eventos Adversos Relaciona	Lesão por pressão		
28/03/2019	CE	JUAZEIRO DO NORTE	HOSPITAL REGIONA	-	Enviada	Eventos Adversos Relaciona	Lesão por pressão		
28/03/2019	CE	JUAZEIRO DO NORTE	HOSPITAL REGIONA	-	Enviada	Eventos Adversos Relaciona	Lesão por pressão		
28/03/2019	CE	JUAZEIRO DO NORTE	HOSPITAL REGIONA	-	Enviada	Eventos Adversos Relaciona	Queda do paciente		
28/03/2019	CE	JUAZEIRO DO NORTE	HOSPITAL REGIONA	perda de dreno	Enviada	Eventos Adversos Relaciona	Outro		
28/03/2019	CE	JUAZEIRO DO NORTE	HOSPITAL REGIONA	-	Enviada	Eventos Adversos Relaciona	Lesão por pressão		
28/03/2019	CE	JUAZEIRO DO NORTE	HOSPITAL REGIONA	-	Enviada	Eventos Adversos Relaciona	Lesão por pressão		
28/03/2019	CE	JUAZEIRO DO NORTE	HOSPITAL REGIONA	-	Enviada	Eventos Adversos Relaciona	Lesão por pressão		
28/03/2019	CE	JUAZEIRO DO NORTE	HOSPITAL REGIONA	perda de sonda nasoenter	Enviada	Eventos Adversos Relaciona	Outro		
28/03/2019	CE	JUAZEIRO DO NORTE	HOSPITAL REGIONA	-	Enviada	Eventos Adversos Relaciona	Queda do paciente		
01/04/2019	CE	JUAZEIRO DO NORTE	HOSPITAL REGIONA	perda de dreno	Enviada	Eventos Adversos Relaciona	Outro		
01/04/2019	CE	JUAZEIRO DO NORTE	HOSPITAL REGIONA	perda de sonda nasoenter	Enviada	Eventos Adversos Relaciona	Outro		
01/04/2019	CE	JUAZEIRO DO NORTE	HOSPITAL REGIONA	-	Enviada	Eventos Adversos Relaciona	Lesão por pressão		

01/04/2019	CE	JUAZEIR O DO NORTE	HOSPITA L REGIONA	-	Enviada	Eventos Adversos Relaciona	Lesão por pressão		
01/04/2019	CE	JUAZEIR O DO NORTE	HOSPITA L REGIONA	-	Enviada	Eventos Adversos Relaciona	Lesão por pressão		
01/04/2019	CE	JUAZEIR O DO NORTE	HOSPITA L REGIONA	-	Enviada	Eventos Adversos Relaciona	Lesão por pressão		
01/04/2019	CE	JUAZEIR O DO NORTE	HOSPITA L REGIONA	-	Enviada	Eventos Adversos Relaciona	Lesão por pressão		
01/04/2019	CE	JUAZEIR O DO NORTE	HOSPITA L REGIONA	-	Enviada	Eventos Adversos Relaciona	Lesão por pressão		
01/04/2019	CE	JUAZEIR O DO NORTE	HOSPITA L REGIONA	perda de dreno	Enviada	Eventos Adversos Relaciona	Outro		
01/04/2019	CE	JUAZEIR O DO NORTE	HOSPITA L REGIONA	PERDA DE SONDA	Enviada	Eventos Adversos Relaciona	Outro		
04/04/2019	CE	JUAZEIR O DO NORTE	HOSPITA L REGIONA	PERDA DE CATETE	Enviada	Eventos Adversos Relaciona	Outro		
04/04/2019	CE	JUAZEIR O DO NORTE	HOSPITA L REGIONA	-	Enviada	Eventos Adversos Relaciona	Lesão por pressão		
04/04/2019	CE	JUAZEIR O DO NORTE	HOSPITA L REGIONA	-	Enviada	Eventos Adversos Relaciona	Lesão por pressão		
04/04/2019	CE	JUAZEIR O DO NORTE	HOSPITA L REGIONA	PERDA DE SONDA	Enviada	Eventos Adversos Relaciona	Outro		
04/04/2019	CE	JUAZEIR O DO NORTE	HOSPITA L REGIONA	PERDA DE SONDA	Enviada	Eventos Adversos Relaciona	Outro		
04/04/2019	CE	JUAZEIR O DO NORTE	HOSPITA L REGIONA	PERDA DE ACESSO	Enviada	Eventos Adversos Relaciona	Outro		
04/04/2019	CE	JUAZEIR O DO NORTE	HOSPITA L REGIONA	-	Enviada	Eventos Adversos Relaciona	Lesão por pressão		
04/04/2019	CE	JUAZEIR O DO NORTE	HOSPITA L REGIONA	-	Enviada	Eventos Adversos Relaciona	Lesão por pressão		
04/04/2019	CE	JUAZEIR O DO NORTE	HOSPITA L REGIONA	PERDA DE ACESSO	Enviada	Eventos Adversos Relaciona	Outro		
04/04/2019	CE	JUAZEIR O DO NORTE	HOSPITA L REGIONA	-	Enviada	Eventos Adversos Relaciona	Queda do paciente		
04/04/2019	CE	JUAZEIR O DO NORTE	HOSPITA L REGIONA	-	Enviada	Eventos Adversos Relaciona	Lesão por pressão		
04/04/2019	CE	JUAZEIR O DO NORTE	HOSPITA L REGIONA	PERDA DE SONDA	Enviada	Eventos Adversos Relaciona	Outro		
19/05/2020	CE	JUAZEIR O DO NORTE	HOSPITA L REGIONA	INCIDEN CIA DE LPP	Enviada	Eventos Adversos Relaciona	Lesão por pressão		
19/05/2020	CE	JUAZEIR O DO NORTE	HOSPITA L REGIONA	INCIDEN CIA DE LPP	Enviada	Eventos Adversos Relaciona	Lesão por pressão		
21/05/2020	CE	JUAZEIR O DO NORTE	HOSPITA L REGIONA	INCIDEN CIA DE LPP	Enviada	Eventos Adversos Relaciona	Lesão por pressão		

21/05/2020	CE	JUAZEIR O DO NORTE	HOSPITA L REGIONA	PERDA DE SNE - REMOÇÃ	Enviada	Eventos Adversos Relaciona	Falhas envolvend o sondas		
21/05/2020	CE	JUAZEIR O DO NORTE	HOSPITA L REGIONA	PERDA DE SNE - REMOÇÃ	Enviada	Eventos Adversos Relaciona	Falhas envolvend o sondas		
21/05/2020	CE	JUAZEIR O DO NORTE	HOSPITA L REGIONA	PERDA DE SNE - REMOÇÃ	Enviada	Eventos Adversos Relaciona	Falhas envolvend o sondas		
21/05/2020	CE	JUAZEIR O DO NORTE	HOSPITA L REGIONA	INCIDEN CIA DE FLEBITE	Enviada	Eventos Adversos Relaciona	Falhas envolvend o cateter		
21/05/2020	CE	JUAZEIR O DO NORTE	HOSPITA L REGIONA	PERDA DE SONDA	Enviada	Eventos Adversos Relaciona	Falhas envolvend o sondas		
01/11/2021	CE	JUAZEIR O DO NORTE	HOSPITA L REGIONA	-	Enviada	Eventos Adversos Relaciona	Queda do paciente		
01/11/2021	CE	JUAZEIR O DO NORTE	HOSPITA L REGIONA	-	Enviada	Eventos Adversos Relaciona	Falhas envolvend o sondas		
01/11/2021	CE	JUAZEIR O DO NORTE	HOSPITA L REGIONA	-	Enviada	Eventos Adversos Relaciona	Falhas ocorridas em		
01/11/2021	CE	JUAZEIR O DO NORTE	HOSPITA L REGIONA	INTERGL ÚTEA	Enviada	Eventos Adversos Relaciona	Lesão por pressão		
01/11/2021	CE	JUAZEIR O DO NORTE	HOSPITA L REGIONA	-	Enviada	Eventos Adversos Relaciona	Falhas na administra ção de		Enteral (oral, cateter
01/11/2021	CE	JUAZEIR O DO NORTE	HOSPITA L REGIONA	-	Enviada	Eventos Adversos Relaciona	Falhas na administra ção de		
21/01/2022	CE	JUAZEIR O DO NORTE	HOSPITA L REGIONA	lesão por dispositiv o - lesão	Enviada	Eventos Adversos Relaciona	Lesão por pressão		
21/01/2022	CE	JUAZEIR O DO NORTE	HOSPITA L REGIONA	SNE - REMOÇÃ O PELO	Enviada	Eventos Adversos Relaciona	Falhas envolvend o sondas		
21/01/2022	CE	JUAZEIR O DO NORTE	HOSPITA L REGIONA	LESÃO POR PRESSÃ	Enviada	Eventos Adversos Relaciona	Lesão por pressão		
21/01/2022	CE	JUAZEIR O DO NORTE	HOSPITA L REGIONA	PACIENT E CONSCIE	Enviada	Eventos Adversos Relaciona	Queda do paciente		
21/01/2022	CE	JUAZEIR O DO NORTE	HOSPITA L REGIONA	PACIENT E CONSCIE	Enviada	Eventos Adversos Relaciona	Queda do paciente		
21/01/2022	CE	JUAZEIR O DO NORTE	HOSPITA L REGIONA	NOTIFIC O INCIDÊN	Enviada	Eventos Adversos Relaciona	Falhas envolvend o cateter		
21/01/2022	CE	JUAZEIR O DO NORTE	HOSPITA L REGIONA	NOTIFIC O OCORRE	Enviada	Eventos Adversos Relaciona	Queda do paciente		
21/01/2022	CE	JUAZEIR O DO NORTE	HOSPITA L REGIONA	LPP SACRAL GRAU 2	Enviada	Eventos Adversos Relaciona	Lesão por pressão		
21/01/2022	CE	JUAZEIR O DO NORTE	HOSPITA L REGIONA	LESÃO POR PRESSÃ	Enviada	Eventos Adversos Relaciona	Lesão por pressão		
21/01/2022	CE	JUAZEIR O DO NORTE	HOSPITA L REGIONA	NÃO ADMINIS TRADO	Enviada	Eventos Adversos Relaciona	Falhas na administra ção de		Enteral (oral, cateter
04/02/2022	CE	JUAZEIR O DO NORTE	HOSPITA L REGIONA	lp sacral estágio 1	Enviada	Eventos Adversos Relaciona	Lesão por pressão		

17/02/2022	CE	JUAZEIR O DO NORTE	HOSPITA L REGIONA	INCIDEN CIA DE LESÃO	Enviada	Eventos Adversos Relaciona	Lesão por pressão		
17/02/2022	CE	JUAZEIR O DO NORTE	HOSPITA L REGIONA	PACIENT E APRESE	Enviada	Eventos Adversos Relaciona	Queda do paciente		
17/02/2022	CE	JUAZEIR O DO NORTE	HOSPITA L REGIONA	INCIDEN CIA DE LESÃO	Enviada	Eventos Adversos Relaciona	Lesão por pressão		
24/02/2022	CE	JUAZEIR O DO NORTE	HOSPITA L REGIONA	FOI ORIENTA DO	Enviada	Eventos Adversos Relaciona	Falhas na administra ção de		Enteral (oral, cateter
24/02/2022	CE	JUAZEIR O DO NORTE	HOSPITA L REGIONA	LESÃO POR PRESSÃ	Concluída	Eventos Adversos Relaciona	Lesão por pressão		
24/02/2022	CE	JUAZEIR O DO NORTE	HOSPITA L REGIONA	INCIDEN CIA DE LESÃO	Enviada	Eventos Adversos Relaciona	Lesão por pressão		
25/02/2022	CE	JUAZEIR O DO NORTE	HOSPITA L REGIONA	HIGIENIZ AÇÃO DO	Enviada	Eventos Adversos Relaciona	Falhas durante a assistênci		
31/03/2022	CE	JUAZEIR O DO NORTE	HOSPITA L REGIONA	DEMORA NA COLETA	Enviada	Eventos Adversos Relaciona	Falhas durante a assistênci		
31/03/2022	CE	JUAZEIR O DO NORTE	HOSPITA L REGIONA	TEMPO DE JEJUM	Enviada	Eventos Adversos Relaciona	Falhas na administra ção de		Enteral (oral, cateter
30/04/2022	CE	JUAZEIR O DO NORTE	HOSPITA L REGIONA	perda de sne por obstrução	Enviada	Eventos Adversos Relaciona	Falhas envolvend o sondas		
30/04/2022	CE	JUAZEIR O DO NORTE	HOSPITA L REGIONA	TEMPO DE JEJUM	Enviada	Eventos Adversos Relaciona	Falhas na administra ção de		Enteral (oral, cateter
30/04/2022	CE	JUAZEIR O DO NORTE	HOSPITA L REGIONA	-	Enviada	Eventos Adversos Relaciona	Falhas na administra ção de		
30/04/2022	CE	JUAZEIR O DO NORTE	HOSPITA L REGIONA	FALHA NA CONDUC	Enviada	Eventos Adversos Relaciona	Falhas durante a assistênci		
30/04/2022	CE	JUAZEIR O DO NORTE	HOSPITA L REGIONA	LESÃO SACRAL ESTÁGIO	Enviada	Eventos Adversos Relaciona	Lesão por pressão		
30/04/2022	CE	JUAZEIR O DO NORTE	HOSPITA L REGIONA	LP SACRAL ESTÁGIO	Enviada	Eventos Adversos Relaciona	Lesão por pressão		
30/04/2022	CE	JUAZEIR O DO NORTE	HOSPITA L REGIONA	LP EM TROCAN TER	Enviada	Eventos Adversos Relaciona	Lesão por pressão		
30/04/2022	CE	JUAZEIR O DO NORTE	HOSPITA L REGIONA	-	Enviada	Eventos Adversos Relaciona	Lesão por pressão		
30/06/2022	CE	JUAZEIR O DO NORTE	HOSPITA L REGIONA	PACIENT E EM DIETA	Enviada	Eventos Adversos Relaciona	Falhas na administra ção de		Enteral (oral, cateter
30/06/2022	CE	JUAZEIR O DO NORTE	HOSPITA L REGIONA	PACIENT E EM DIETA	Enviada	Eventos Adversos Relaciona	Falhas na administra ção de		Enteral (oral, cateter
30/06/2022	CE	JUAZEIR O DO NORTE	HOSPITA L REGIONA	HIPOTEN SÃO POSTUR	Enviada	Eventos Adversos Relaciona	Queda do paciente		
30/06/2022	CE	JUAZEIR O DO NORTE	HOSPITA L REGIONA	CAIU AO TENTAR DESCER	Enviada	Eventos Adversos Relaciona	Queda do paciente		
30/06/2022	CE	JUAZEIR O DO NORTE	HOSPITA L REGIONA	MACA DESNCAI CHOU	Enviada	Eventos Adversos Relaciona	Queda do paciente		

30/06/2022	CE	JUAZEIRO DO NORTE	HOSPITAL REGIONA	AO TENTAR PASSAR	Enviada	Eventos Adversos Relaciona	Queda do paciente		
30/06/2022	CE	JUAZEIRO DO NORTE	HOSPITAL REGIONA	PACIENTE LEVANT	Enviada	Eventos Adversos Relaciona	Queda do paciente		
30/06/2022	CE	JUAZEIRO DO NORTE	HOSPITAL REGIONA	MÁ FIXAÇÃO -	Enviada	Eventos Adversos Relaciona	Falhas envolvendo sondas		
30/06/2022	CE	JUAZEIRO DO NORTE	HOSPITAL REGIONA	REMOÇÃO PELO PACIENT	Enviada	Eventos Adversos Relaciona	Falhas envolvendo sondas		
30/06/2022	CE	JUAZEIRO DO NORTE	HOSPITAL REGIONA	SNE - REMOÇÃO PELO	Enviada	Eventos Adversos Relaciona	Falhas envolvendo sondas		
29/07/2022	CE	JUAZEIRO DO NORTE	HOSPITAL REGIONA	-	Enviada	Eventos Adversos Relaciona	Lesão por pressão		
29/07/2022	CE	JUAZEIRO DO NORTE	HOSPITAL REGIONA	LPP CALACÂNEOS	Enviada	Eventos Adversos Relaciona	Lesão por pressão		
29/07/2022	CE	JUAZEIRO DO NORTE	HOSPITAL REGIONA	LESÃO POR PRESSÃ	Enviada	Eventos Adversos Relaciona	Lesão por pressão		
29/07/2022	CE	JUAZEIRO DO NORTE	HOSPITAL REGIONA	SAÍDA NÃO PLANEJA	Enviada	Eventos Adversos Relaciona	Extubação endotraqu		
29/07/2022	CE	JUAZEIRO DO NORTE	HOSPITAL REGIONA	SNE - REMOÇÃO PELO	Enviada	Eventos Adversos Relaciona	Falhas envolvendo sondas		
29/07/2022	CE	JUAZEIRO DO NORTE	HOSPITAL REGIONA	SNE- REMOÇÃO	Enviada	Eventos Adversos Relaciona	Falhas envolvendo sondas		
29/07/2022	CE	JUAZEIRO DO NORTE	HOSPITAL REGIONA	SNE - REMOÇÃO PELO	Enviada	Eventos Adversos Relaciona	Falhas envolvendo sondas		
31/08/2022	CE	JUAZEIRO DO NORTE	HOSPITAL REGIONA	QUEDA DA PROPRIA	Enviada	Eventos Adversos Relaciona	Queda do paciente		
31/08/2022	CE	JUAZEIRO DO NORTE	HOSPITAL REGIONA	INCIDENCIA DE LP	Enviada	Eventos Adversos Relaciona	Lesão por pressão		
31/08/2022	CE	JUAZEIRO DO NORTE	HOSPITAL REGIONA	EMISSÃO DE RESULTA	Enviada	Eventos Adversos Relaciona	Falhas ocorridas em		
31/08/2022	CE	JUAZEIRO DO NORTE	HOSPITAL REGIONA	LPP ESTÁGIO II EM	Enviada	Eventos Adversos Relaciona	Lesão por pressão		
31/08/2022	CE	JUAZEIRO DO NORTE	HOSPITAL REGIONA	LPP SACRAL E	Enviada	Eventos Adversos Relaciona	Lesão por pressão		
31/08/2022	CE	JUAZEIRO DO NORTE	HOSPITAL REGIONA	ATRASO NO RESULTA	Enviada	Eventos Adversos Relaciona	Falhas ocorridas em		
31/08/2022	CE	JUAZEIRO DO NORTE	HOSPITAL REGIONA	FLEBITE	Enviada	Eventos Adversos Relaciona	Falhas envolvendo cateter		
31/08/2022	CE	JUAZEIRO DO NORTE	HOSPITAL REGIONA	CAMA QUEBROU COM	Enviada	Eventos Adversos Relaciona	Queda do paciente		
31/08/2022	CE	JUAZEIRO DO NORTE	HOSPITAL REGIONA	FLEBITE	Enviada	Eventos Adversos Relaciona	Falhas envolvendo cateter		
31/08/2022	CE	JUAZEIRO DO NORTE	HOSPITAL REGIONA	SAÍDA NÃO PLANEJA	Enviada	Eventos Adversos Relaciona	Extubação endotraqu		

31/08/2022	CE	JUAZEIR O DO NORTE	HOSPITA L REGIONA	LP ESTAGIO 01 EM	Enviada	Eventos Adversos Relaciona	Lesão por pressão		
31/08/2022	CE	JUAZEIR O DO NORTE	HOSPITA L REGIONA	SNE - OBSTRU ÇÃO	Enviada	Eventos Adversos Relaciona	Falhas envolvend o sondas		
31/08/2022	CE	JUAZEIR O DO NORTE	HOSPITA L REGIONA	INCIDEN CIA DE LP	Enviada	Eventos Adversos Relaciona	Lesão por pressão		
31/08/2022	CE	JUAZEIR O DO NORTE	HOSPITA L REGIONA	SNE - REMOÇÃ O PELO	Enviada	Eventos Adversos Relaciona	Falhas envolvend o sondas		
31/08/2022	CE	JUAZEIR O DO NORTE	HOSPITA L REGIONA	QUEDA DO PACIENT	Enviada	Eventos Adversos Relaciona	Queda do paciente		
16/09/2022	CE	JUAZEIR O DO NORTE	HOSPITA L REGIONA	SAÍDA NÃO PLANEJA	Enviada	Eventos Adversos Relaciona	Extubaçã o endotraqu		
16/09/2022	CE	JUAZEIR O DO NORTE	HOSPITA L REGIONA	QUEDA DO PACIENT	Enviada	Eventos Adversos Relaciona	Queda do paciente		
26/09/2022	CE	JUAZEIR O DO NORTE	HOSPITA L REGIONA	EXAMES PROGRA MADOS	Enviada	Eventos Adversos Relaciona	Falhas durante a assistênci		
26/09/2022	CE	JUAZEIR O DO NORTE	HOSPITA L REGIONA	PROFISS IONAL RETIROU	Enviada	Eventos Adversos Relaciona	Falhas durante a assistênci		
26/09/2022	CE	JUAZEIR O DO NORTE	HOSPITA L REGIONA	PERDA DE SONDA	Enviada	Eventos Adversos Relaciona	Falhas envolvend o sondas		
26/09/2022	CE	JUAZEIR O DO NORTE	HOSPITA L REGIONA	FALHA NA COMUNI	Enviada	Eventos Adversos Relaciona	Falhas nas atividades		
27/09/2022	CE	JUAZEIR O DO NORTE	HOSPITA L REGIONA	LESÃO POR DISPOSI	Enviada	Eventos Adversos Relaciona	Lesão por pressão		
27/09/2022	CE	JUAZEIR O DO NORTE	HOSPITA L REGIONA	-	Enviada	Eventos Adversos Relaciona	Falhas envolvend o sondas		
27/09/2022	CE	JUAZEIR O DO NORTE	HOSPITA L REGIONA	INCIDEN CIA DE LP	Enviada	Eventos Adversos Relaciona	Lesão por pressão		
27/09/2022	CE	JUAZEIR O DO NORTE	HOSPITA L REGIONA	INCIDEN CIA DE LP	Enviada	Eventos Adversos Relaciona	Lesão por pressão		
27/09/2022	CE	JUAZEIR O DO NORTE	HOSPITA L REGIONA	FALHA NO SEGUIME	Enviada	Eventos Adversos Relaciona	Falhas durante a assistênci		
27/09/2022	CE	JUAZEIR O DO NORTE	HOSPITA L REGIONA	REMOÇA O PELO PACIENT	Enviada	Eventos Adversos Relaciona	Falhas envolvend o sondas		
27/09/2022	CE	JUAZEIR O DO NORTE	HOSPITA L REGIONA	PACIENT E APRESE	Enviada	Eventos Adversos Relaciona	Falhas envolvend o cateter		
27/09/2022	CE	JUAZEIR O DO NORTE	HOSPITA L REGIONA	FALHA EM EXAME	Enviada	Eventos Adversos Relaciona	Falhas nas atividades		
27/09/2022	CE	JUAZEIR O DO NORTE	HOSPITA L REGIONA	REMOÇA O PELO PACIENT	Enviada	Eventos Adversos Relaciona	Falhas envolvend o sondas		
28/09/2022	CE	JUAZEIR O DO NORTE	HOSPITA L REGIONA	FALHA NA COMUNI	Enviada	Eventos Adversos Relaciona	Falhas nas atividades		
28/09/2022	CE	JUAZEIR O DO NORTE	HOSPITA L REGIONA	PACIENT E EM PCR	Enviada	Eventos Adversos Relaciona	Falhas durante a assistênci		

28/09/2022	CE	JUAZEIRO DO NORTE	HOSPITAL REGIONA	FALHA NA REALIZA	Enviada	Eventos Adversos Relaciona	Falhas durante a assistênci		
28/09/2022	CE	JUAZEIRO DO NORTE	HOSPITAL REGIONA	PNEUMOTORAX NÃO	Enviada	Eventos Adversos Relaciona	Falhas durante a assistênci		
28/09/2022	CE	JUAZEIRO DO NORTE	HOSPITAL REGIONA	QUEDA DO PACIENT	Enviada	Eventos Adversos Relaciona	Queda do paciente		
28/09/2022	CE	JUAZEIRO DO NORTE	HOSPITAL REGIONA	LPP SACRAL E1 E LPP	Enviada	Eventos Adversos Relaciona	Lesão por pressão		
28/09/2022	CE	JUAZEIRO DO NORTE	HOSPITAL REGIONA	LESÃO POR PRESSÃ	Enviada	Eventos Adversos Relaciona	Lesão por pressão		
28/09/2022	CE	JUAZEIRO DO NORTE	HOSPITAL REGIONA	FALHA NA REALIZA	Enviada	Eventos Adversos Relaciona	Falhas durante a assistênci		
28/09/2022	CE	JUAZEIRO DO NORTE	HOSPITAL REGIONA	LESÃO POR PRESSÃ	Enviada	Eventos Adversos Relaciona	Lesão por pressão		
28/09/2022	CE	JUAZEIRO DO NORTE	HOSPITAL REGIONA	SAÍDA NÃO PLANEJA	Enviada	Eventos Adversos Relaciona	Extubação endotraqu		
28/09/2022	CE	JUAZEIRO DO NORTE	HOSPITAL REGIONA	PRODUTO DISPENS	Enviada	Eventos Adversos Relaciona	Falhas durante a assistênci		
28/09/2022	CE	JUAZEIRO DO NORTE	HOSPITAL REGIONA	DIFICULDADE COM	Enviada	Eventos Adversos Relaciona	Falhas nas atividades		
28/09/2022	CE	JUAZEIRO DO NORTE	HOSPITAL REGIONA	EQUIPAMENTO DEFEITU	Enviada	Eventos Adversos Relaciona	Falhas durante a assistênci		
28/09/2022	CE	JUAZEIRO DO NORTE	HOSPITAL REGIONA	PERDA DE SNE - REMOÇÃ	Enviada	Eventos Adversos Relaciona	Falhas envolvendo sondas		
28/09/2022	CE	JUAZEIRO DO NORTE	HOSPITAL REGIONA	SNE - REMOÇÃO PELO	Enviada	Eventos Adversos Relaciona	Falhas envolvendo sondas		
28/09/2022	CE	JUAZEIRO DO NORTE	HOSPITAL REGIONA	FLEBITE	Enviada	Eventos Adversos Relaciona	Falhas envolvendo cateter		
28/09/2022	CE	JUAZEIRO DO NORTE	HOSPITAL REGIONA	PERDA DE SONDA	Enviada	Eventos Adversos Relaciona	Falhas envolvendo sondas		
28/09/2022	CE	JUAZEIRO DO NORTE	HOSPITAL REGIONA	SNE-REMOÇÃO PELO	Enviada	Eventos Adversos Relaciona	Falhas envolvendo sondas		
28/09/2022	CE	JUAZEIRO DO NORTE	HOSPITAL REGIONA	LESÃO POR PRESSÃ	Enviada	Eventos Adversos Relaciona	Lesão por pressão		
28/09/2022	CE	JUAZEIRO DO NORTE	HOSPITAL REGIONA	DOPPLER PROGRA	Enviada	Eventos Adversos Relaciona	Falhas durante a assistênci		
29/09/2022	CE	JUAZEIRO DO NORTE	HOSPITAL REGIONA	DOPPLER PROGRA	Enviada	Eventos Adversos Relaciona	Falhas durante a assistênci		
29/09/2022	CE	JUAZEIRO DO NORTE	HOSPITAL REGIONA	INCIDENCIA DE LP	Enviada	Eventos Adversos Relaciona	Lesão por pressão		
29/09/2022	CE	JUAZEIRO DO NORTE	HOSPITAL REGIONA	FALHA NA HIGIENIZ	Enviada	Eventos Adversos Relaciona	Falhas durante a assistênci		
29/09/2022	CE	JUAZEIRO DO NORTE	HOSPITAL REGIONA	MEDICO ORTOPEDISTA	Enviada	Eventos Adversos Relaciona	Falhas durante a assistênci		

29/09/2022	CE	JUAZEIR O DO NORTE	HOSPITA L REGIONA	NECESSI DADE DE	Enviada	Eventos Adversos Relaciona	Extubaça o endotraqu		
29/09/2022	CE	JUAZEIR O DO NORTE	HOSPITA L REGIONA	EXAME SOLICITA DO E	Enviada	Eventos Adversos Relaciona	Falhas durante a assistênci		
29/09/2022	CE	JUAZEIR O DO NORTE	HOSPITA L REGIONA	EXAME SOLICITA DO E	Enviada	Eventos Adversos Relaciona	Falhas durante a assistênci		
29/09/2022	CE	JUAZEIR O DO NORTE	HOSPITA L REGIONA	EXAME SOLICITA DO DE	Enviada	Eventos Adversos Relaciona	Falhas durante a assistênci		
29/09/2022	CE	JUAZEIR O DO NORTE	HOSPITA L REGIONA	PACIENT E ENCAMIN	Enviada	Eventos Adversos Relaciona	Falhas durante a assistênci		
29/09/2022	CE	JUAZEIR O DO NORTE	HOSPITA L REGIONA	ACESSO VENOSO PERIFÉRI	Enviada	Eventos Adversos Relaciona	Falhas envolvend o cateter		
29/09/2022	CE	JUAZEIR O DO NORTE	HOSPITA L REGIONA	PERDA DE SNE POR	Enviada	Eventos Adversos Relaciona	Falhas envolvend o sondas		
29/09/2022	CE	JUAZEIR O DO NORTE	HOSPITA L REGIONA	MEDICA MENTO MPP	Enviada	Eventos Adversos Relaciona	Falhas durante a assistênci		
29/09/2022	CE	JUAZEIR O DO NORTE	HOSPITA L REGIONA	MEDICAÇ ÃO ADMINIS	Enviada	Eventos Adversos Relaciona	Falhas durante a assistênci		
29/09/2022	CE	JUAZEIR O DO NORTE	HOSPITA L REGIONA	ACESSO VENOSO PERIFÉRI	Enviada	Eventos Adversos Relaciona	Falhas envolvend o cateter		
29/09/2022	CE	JUAZEIR O DO NORTE	HOSPITA L REGIONA	INCIDEN CIA DE FLEBITE	Enviada	Eventos Adversos Relaciona	Falhas envolvend o cateter		
29/09/2022	CE	JUAZEIR O DO NORTE	HOSPITA L REGIONA	PERDA DE SNE POR	Enviada	Eventos Adversos Relaciona	Falhas envolvend o sondas		
29/09/2022	CE	JUAZEIR O DO NORTE	HOSPITA L REGIONA	PERDA DE SNE POR	Enviada	Eventos Adversos Relaciona	Falhas envolvend o sondas		
29/09/2022	CE	JUAZEIR O DO NORTE	HOSPITA L REGIONA	PERDA DE SNE DURANT	Enviada	Eventos Adversos Relaciona	Falhas envolvend o sondas		
29/09/2022	CE	JUAZEIR O DO NORTE	HOSPITA L REGIONA	INCIDEN CIA DE FLEBITE	Enviada	Eventos Adversos Relaciona	Falhas envolvend o cateter		
29/09/2022	CE	JUAZEIR O DO NORTE	HOSPITA L REGIONA	INCIDEN CIA DE LP	Enviada	Eventos Adversos Relaciona	Lesão por pressão		
29/09/2022	CE	JUAZEIR O DO NORTE	HOSPITA L REGIONA	EXAME SOLICTA DO E	Enviada	Eventos Adversos Relaciona	Falhas durante a assistênci		
29/09/2022	CE	JUAZEIR O DO NORTE	HOSPITA L REGIONA	EXAME DOPPLE R	Enviada	Eventos Adversos Relaciona	Falhas durante a assistênci		
29/09/2022	CE	JUAZEIR O DO NORTE	HOSPITA L REGIONA	LESÃO POR DISPOSI	Enviada	Eventos Adversos Relaciona	Lesão por pressão		
30/09/2022	CE	JUAZEIR O DO NORTE	HOSPITA L REGIONA	SUSPEN SÃO DE CIRURGI	Enviada	Eventos Adversos Relaciona	Falhas durante a assistênci		
30/09/2022	CE	JUAZEIR O DO NORTE	HOSPITA L REGIONA	EXAME NÃO REALIZA	Enviada	Eventos Adversos Relaciona	Falhas durante a assistênci		
30/09/2022	CE	JUAZEIR O DO NORTE	HOSPITA L REGIONA	INCIDEN CIA DE LP	Enviada	Eventos Adversos Relaciona	Lesão por pressão		

30/09/2022	CE	JUAZEIR O DO NORTE	HOSPITA L REGIONA	INCIDEN CIA DE LP	Enviada	Eventos Adversos Relaciona	Lesão por pressão		
30/09/2022	CE	JUAZEIR O DO NORTE	HOSPITA L REGIONA	ATRASO NA ADMINIS	Enviada	Eventos Adversos Relaciona	Falhas durante a assistênci		
30/09/2022	CE	JUAZEIR O DO NORTE	HOSPITA L REGIONA	SRPA SUPERL OTADA	Enviada	Eventos Adversos Relaciona	Falhas nas atividades		
30/09/2022	CE	JUAZEIR O DO NORTE	HOSPITA L REGIONA	DOPPLE R PROGRA	Enviada	Eventos Adversos Relaciona	Falhas durante a assistênci		
18/10/2022	CE	JUAZEIR O DO NORTE	HOSPITA L REGIONA	PACIENT E APRESE	Enviada	Eventos Adversos Relaciona	Falhas durante a assistênci		
18/10/2022	CE	JUAZEIR O DO NORTE	HOSPITA L REGIONA	FALHA NA DISPONI	Enviada	Eventos Adversos Relaciona	Falhas durante a assistênci		
18/10/2022	CE	JUAZEIR O DO NORTE	HOSPITA L REGIONA	REMANE JAMENT O DE	Enviada	Eventos Adversos Relaciona	Falhas durante a assistênci		
18/10/2022	CE	JUAZEIR O DO NORTE	HOSPITA L REGIONA	SUSPEN SÃO DE	Enviada	Eventos Adversos Relaciona	Falhas durante a assistênci		
18/10/2022	CE	JUAZEIR O DO NORTE	HOSPITA L REGIONA	LPP SACRAL EST 1	Enviada	Eventos Adversos Relaciona	Lesão por pressão		
18/10/2022	CE	JUAZEIR O DO NORTE	HOSPITA L REGIONA	AUSENCI A DE JANTAR	Enviada	Eventos Adversos Relaciona	Falhas na administra ção de		Enteral (oral, cateter
18/10/2022	CE	JUAZEIR O DO NORTE	HOSPITA L REGIONA	FALHA NA REAVALI	Enviada	Eventos Adversos Relaciona	Falhas durante a assistênci		
18/10/2022	CE	JUAZEIR O DO NORTE	HOSPITA L REGIONA	ISC DE CIRURGI A	Enviada	Eventos Adversos Relaciona	Falhas durante a assistênci		
18/10/2022	CE	JUAZEIR O DO NORTE	HOSPITA L REGIONA	PERDA DE AMOSTR	Enviada	Eventos Adversos Relaciona	Falhas ocorridas em		
18/10/2022	CE	JUAZEIR O DO NORTE	HOSPITA L REGIONA	LP E2 NO MESMO	Enviada	Eventos Adversos Relaciona	Lesão por pressão		
18/10/2022	CE	JUAZEIR O DO NORTE	HOSPITA L REGIONA	LP E2 NO MESMO	Enviada	Eventos Adversos Relaciona	Lesão por pressão		
18/10/2022	CE	JUAZEIR O DO NORTE	HOSPITA L REGIONA	ERRO DE MEDICA	Enviada	Eventos Adversos Relaciona	Falhas durante a assistênci		
18/10/2022	CE	JUAZEIR O DO NORTE	HOSPITA L REGIONA	FALHA NA COMUNI	Enviada	Eventos Adversos Relaciona	Falhas durante a assistênci		
18/10/2022	CE	JUAZEIR O DO NORTE	HOSPITA L REGIONA	FALHAS NA ASSISTE	Enviada	Eventos Adversos Relaciona	Falhas envolvend o cateter		
18/10/2022	CE	JUAZEIR O DO NORTE	HOSPITA L REGIONA	EXAMES DE IMAGEM	Enviada	Eventos Adversos Relaciona	Falhas durante a assistênci		
18/10/2022	CE	JUAZEIR O DO NORTE	HOSPITA L REGIONA	SNE - REMOÇÃ O PELO	Enviada	Eventos Adversos Relaciona	Falhas envolvend o sondas		
18/10/2022	CE	JUAZEIR O DO NORTE	HOSPITA L REGIONA	SNE - REMOÇÃ O PELO	Enviada	Eventos Adversos Relaciona	Falhas envolvend o sondas		
18/10/2022	CE	JUAZEIR O DO NORTE	HOSPITA L REGIONA	SNE - REMOÇÃ O PELO	Enviada	Eventos Adversos Relaciona	Falhas envolvend o sondas		

18/10/2022	CE	JUAZEIR O DO NORTE	HOSPITA L REGIONA	FALHA NA REALIZA	Enviada	Eventos Adversos Relaciona	Falhas durante a assistênci		
18/10/2022	CE	JUAZEIR O DO NORTE	HOSPITA L REGIONA	FALHA NO PROCEDI	Enviada	Eventos Adversos Relaciona	Falhas durante a assistênci		
18/10/2022	CE	JUAZEIR O DO NORTE	HOSPITA L REGIONA	FALHAS NA ASSISTE	Enviada	Eventos Adversos Relaciona	Falhas envolvend o cateter		
27/10/2022	CE	JUAZEIR O DO NORTE	HOSPITA L REGIONA	ERRO DE MEDICA	Enviada	Eventos Adversos Relaciona	Falhas durante a assistênci		
27/10/2022	CE	JUAZEIR O DO NORTE	HOSPITA L REGIONA	PACIENT E COM LESÃO	Enviada	Eventos Adversos Relaciona	Falhas durante a assistênci		
27/10/2022	CE	JUAZEIR O DO NORTE	HOSPITA L REGIONA	FALHA EM EXAME	Enviada	Eventos Adversos Relaciona	Falhas durante a assistênci		
27/10/2022	CE	JUAZEIR O DO NORTE	HOSPITA L REGIONA	FALHA EM EXAME	Enviada	Eventos Adversos Relaciona	Falhas durante a assistênci		
27/10/2022	CE	JUAZEIR O DO NORTE	HOSPITA L REGIONA	LESÃO POR PRESSÃ	Enviada	Eventos Adversos Relaciona	Lesão por pressão		
27/10/2022	CE	JUAZEIR O DO NORTE	HOSPITA L REGIONA	FALHAS NA ASSISTE	Enviada	Eventos Adversos Relaciona	Falhas envolvend o cateter		
27/10/2022	CE	JUAZEIR O DO NORTE	HOSPITA L REGIONA	FALHAS NA ASSISTE	Enviada	Eventos Adversos Relaciona	Falhas envolvend o sondas		
27/10/2022	CE	JUAZEIR O DO NORTE	HOSPITA L REGIONA	ERRO DE MEDICA	Enviada	Eventos Adversos Relaciona	Falhas durante a assistênci		
27/10/2022	CE	JUAZEIR O DO NORTE	HOSPITA L REGIONA	FALHA EM EXAME	Enviada	Eventos Adversos Relaciona	Falhas durante a assistênci		
27/10/2022	CE	JUAZEIR O DO NORTE	HOSPITA L REGIONA	FALHAS NA ASSISTE	Enviada	Eventos Adversos Relaciona	Falhas durante a assistênci		
27/10/2022	CE	JUAZEIR O DO NORTE	HOSPITA L REGIONA	FALHAS NA ASSISTE	Enviada	Eventos Adversos Relaciona	Falhas envolvend o sondas		
28/10/2022	CE	JUAZEIR O DO NORTE	HOSPITA L REGIONA	ERROS DE DIETA -	Enviada	Eventos Adversos Relaciona	Falhas durante a assistênci		
28/10/2022	CE	JUAZEIR O DO NORTE	HOSPITA L REGIONA	FALHAS NA ASSISTE	Enviada	Eventos Adversos Relaciona	Lesão por pressão		
28/10/2022	CE	JUAZEIR O DO NORTE	HOSPITA L REGIONA	FALHAS NA ASSISTE	Enviada	Eventos Adversos Relaciona	Falhas envolvend o sondas		
28/10/2022	CE	JUAZEIR O DO NORTE	HOSPITA L REGIONA	FALHAS NA ASSISTE	Enviada	Eventos Adversos Relaciona	Falhas durante a assistênci		
28/10/2022	CE	JUAZEIR O DO NORTE	HOSPITA L REGIONA	FALHAS NA ASSISTE	Enviada	Eventos Adversos Relaciona	Falhas durante a assistênci		
28/10/2022	CE	JUAZEIR O DO NORTE	HOSPITA L REGIONA	FALHA EM EXAME	Enviada	Eventos Adversos Relaciona	Falhas durante a assistênci		
28/10/2022	CE	JUAZEIR O DO NORTE	HOSPITA L REGIONA	FALHA EM EXAME	Enviada	Eventos Adversos Relaciona	Falhas durante a assistênci		
28/10/2022	CE	JUAZEIR O DO NORTE	HOSPITA L REGIONA	FALHAS NA ASSISTE	Enviada	Eventos Adversos Relaciona	Falhas envolvend o cateter		

28/10/2022	CE	JUAZEIR O DO NORTE	HOSPITA L REGIONA	FALHAS NA ASSISTE	Enviada	Eventos Adversos Relaciona	Lesão por pressão		
28/10/2022	CE	JUAZEIR O DO NORTE	HOSPITA L REGIONA	FALHAS NA ASSISTE	Enviada	Eventos Adversos Relaciona	Falhas envolvend o sondas		
28/10/2022	CE	JUAZEIR O DO NORTE	HOSPITA L REGIONA	FALHAS NA ASSISTE	Enviada	Eventos Adversos Relaciona	Queda do paciente		
28/10/2022	CE	JUAZEIR O DO NORTE	HOSPITA L REGIONA	ERROS DE DIETA -	Enviada	Eventos Adversos Relaciona	Falhas durante a assistênci		
28/10/2022	CE	JUAZEIR O DO NORTE	HOSPITA L REGIONA	FALHA NA ADMINIS	Enviada	Eventos Adversos Relaciona	Falhas durante a assistênci		
28/10/2022	CE	JUAZEIR O DO NORTE	HOSPITA L REGIONA	FALHAS NA ASSISTE	Enviada	Eventos Adversos Relaciona	Falhas durante a assistênci		
28/10/2022	CE	JUAZEIR O DO NORTE	HOSPITA L REGIONA	-	Enviada	Eventos Adversos Relaciona	Falhas durante a assistênci		
28/10/2022	CE	JUAZEIR O DO NORTE	HOSPITA L REGIONA	FALHAS NA ASSISTE	Enviada	Eventos Adversos Relaciona	Falhas durante a assistênci		
28/10/2022	CE	JUAZEIR O DO NORTE	HOSPITA L REGIONA	FALHAS NA ASSISTE	Enviada	Eventos Adversos Relaciona	Falhas envolvend o sondas		
28/10/2022	CE	JUAZEIR O DO NORTE	HOSPITA L REGIONA	FALHAS NA ASSISTE	Enviada	Eventos Adversos Relaciona	Falhas envolvend o sondas		
28/10/2022	CE	JUAZEIR O DO NORTE	HOSPITA L REGIONA	ERRO DE MEDICA	Enviada	Eventos Adversos Relaciona	Falhas durante a assistênci		
28/10/2022	CE	JUAZEIR O DO NORTE	HOSPITA L REGIONA	ERROS DE DIETA -	Enviada	Eventos Adversos Relaciona	Falhas na administra ção de		Enteral (oral, cateter
28/10/2022	CE	JUAZEIR O DO NORTE	HOSPITA L REGIONA	FALHAS NA ASSISTE	Enviada	Eventos Adversos Relaciona	Falhas envolvend o sondas		
28/10/2022	CE	JUAZEIR O DO NORTE	HOSPITA L REGIONA	FALHAS NA ASSISTE	Enviada	Eventos Adversos Relaciona	Falhas envolvend o sondas		
28/10/2022	CE	JUAZEIR O DO NORTE	HOSPITA L REGIONA	FALHAS NA ASSISTE	Enviada	Eventos Adversos Relaciona	Falhas envolvend o sondas		
28/10/2022	CE	JUAZEIR O DO NORTE	HOSPITA L REGIONA	FALHAS NA ASSISTE	Enviada	Eventos Adversos Relaciona	Falhas envolvend o sondas		
28/10/2022	CE	JUAZEIR O DO NORTE	HOSPITA L REGIONA	FALHA EM EXAME	Enviada	Eventos Adversos Relaciona	Falhas durante a assistênci		
09/11/2022	CE	JUAZEIR O DO NORTE	HOSPITA L REGIONA	FALHAS NA ASSISTE	Enviada	Eventos Adversos Relaciona	Falhas envolvend o sondas		
09/11/2022	CE	JUAZEIR O DO NORTE	HOSPITA L REGIONA	PERDA DE ACESSO	Enviada	Eventos Adversos Relaciona	Falhas envolvend o sondas		
23/11/2022	CE	JUAZEIR O DO NORTE	HOSPITA L REGIONA	FALHAS NA ASSISTE	Enviada	Eventos Adversos Relaciona	Falhas envolvend o sondas		
24/11/2022	CE	JUAZEIR O DO NORTE	HOSPITA L REGIONA	FALHAS NA ASSISTE	Enviada	Eventos Adversos Relaciona	Lesão por pressão		
24/11/2022	CE	JUAZEIR O DO NORTE	HOSPITA L REGIONA	FALHA NO PROCEDI	Enviada	Eventos Adversos Relaciona	Falhas durante a assistênci		

24/11/2022	CE	JUAZEIR O DO NORTE	HOSPITA L REGIONA	FALHA EM EXAME	Enviada	Eventos Adversos Relaciona	Falhas durante a assistênci		
25/11/2022	CE	JUAZEIR O DO NORTE	HOSPITA L REGIONA	FALHAS NA ASSISTE	Enviada	Eventos Adversos Relaciona	Falhas durante a assistênci		
25/11/2022	CE	JUAZEIR O DO NORTE	HOSPITA L REGIONA	FALHA EM EXAME	Enviada	Eventos Adversos Relaciona	Falhas durante a assistênci		
25/11/2022	CE	JUAZEIR O DO NORTE	HOSPITA L REGIONA	FALHAS NA ASSISTE	Enviada	Eventos Adversos Relaciona	Falhas durante a assistênci		
29/11/2022	CE	JUAZEIR O DO NORTE	HOSPITA L REGIONA	FALHAS NA ASSISTE	Enviada	Eventos Adversos Relaciona	Falhas envolvend o sondas		
29/11/2022	CE	JUAZEIR O DO NORTE	HOSPITA L REGIONA	FALHAS NA ASSISTE	Enviada	Eventos Adversos Relaciona	Lesão por pressão		
29/11/2022	CE	JUAZEIR O DO NORTE	HOSPITA L REGIONA	FALHAS NA ASSISTE	Enviada	Eventos Adversos Relaciona	Lesão por pressão		
29/11/2022	CE	JUAZEIR O DO NORTE	HOSPITA L REGIONA	FALHAS NA ASSISTE	Enviada	Eventos Adversos Relaciona	Falhas durante a assistênci		
30/11/2022	CE	JUAZEIR O DO NORTE	HOSPITA L REGIONA	FALHAS NA ASSISTE	Enviada	Eventos Adversos Relaciona	Lesão por pressão		
30/11/2022	CE	JUAZEIR O DO NORTE	HOSPITA L REGIONA	FALHA NO PROCEDI	Enviada	Eventos Adversos Relaciona	Queimadu ra de paciente		
30/11/2022	CE	JUAZEIR O DO NORTE	HOSPITA L REGIONA	FALHAS NA ASSISTE	Enviada	Eventos Adversos Relaciona	Falhas durante a assistênci		
30/11/2022	CE	JUAZEIR O DO NORTE	HOSPITA L REGIONA	FALHAS NA ASSISTE	Enviada	Eventos Adversos Relaciona	Falhas envolvend o cateter		
30/11/2022	CE	JUAZEIR O DO NORTE	HOSPITA L REGIONA	FALHAS NA ASSISTE	Enviada	Eventos Adversos Relaciona	Queda do paciente		
30/11/2022	CE	JUAZEIR O DO NORTE	HOSPITA L REGIONA	ERROS DE DIETA -	Enviada	Eventos Adversos Relaciona	Falhas na administra ção de		Enteral (oral, cateter
30/11/2022	CE	JUAZEIR O DO NORTE	HOSPITA L REGIONA	FALHAS NA ASSISTE	Enviada	Eventos Adversos Relaciona	Lesão por pressão		
07/12/2022	CE	JUAZEIR O DO NORTE	HOSPITA L REGIONA	ERRO DE MEDICA	Enviada	Eventos Adversos Relaciona	Falhas durante a assistênci		
07/12/2022	CE	JUAZEIR O DO NORTE	HOSPITA L REGIONA	FALHAS NA ASSISTE	Enviada	Eventos Adversos Relaciona	Falhas durante a assistênci		
07/12/2022	CE	JUAZEIR O DO NORTE	HOSPITA L REGIONA	ERROS DE DIETA -	Enviada	Eventos Adversos Relaciona	Falhas na administra ção de		Enteral (oral, cateter
07/12/2022	CE	JUAZEIR O DO NORTE	HOSPITA L REGIONA	FALHAS NA ASSISTE	Enviada	Eventos Adversos Relaciona	Falhas durante a assistênci		
07/12/2022	CE	JUAZEIR O DO NORTE	HOSPITA L REGIONA	FALHAS NA ASSISTE	Enviada	Eventos Adversos Relaciona	Falhas durante a assistênci		
07/12/2022	CE	JUAZEIR O DO NORTE	HOSPITA L REGIONA	FALHA NO PROCEDI	Enviada	Eventos Adversos Relaciona	Falhas durante a assistênci		
07/12/2022	CE	JUAZEIR O DO NORTE	HOSPITA L REGIONA	FALHA NO PROCEDI	Enviada	Eventos Adversos Relaciona	Falhas durante a assistênci		

07/12/2022	CE	JUAZEIR O DO NORTE	HOSPITA L REGIONA	FALHA NO PROCEDI	Enviada	Eventos Adversos Relaciona	Falhas durante a assistênci		
07/12/2022	CE	JUAZEIR O DO NORTE	HOSPITA L REGIONA	FALHAS NA ASSISTE	Enviada	Eventos Adversos Relaciona	Falhas durante a assistênci		
14/12/2022	CE	JUAZEIR O DO NORTE	HOSPITA L REGIONA	FALHA NA ADMINIS	Enviada	Eventos Adversos Relaciona	Falhas durante a assistênci		
14/12/2022	CE	JUAZEIR O DO NORTE	HOSPITA L REGIONA	FALHA NA ADMINIS	Enviada	Eventos Adversos Relaciona	Falhas nas atividades		
14/12/2022	CE	JUAZEIR O DO NORTE	HOSPITA L REGIONA	FALHA NA ADMINIS	Enviada	Eventos Adversos Relaciona	Falhas durante a assistênci		
14/12/2022	CE	JUAZEIR O DO NORTE	HOSPITA L REGIONA	FALHAS NA ASSISTE	Enviada	Eventos Adversos Relaciona	Falhas durante a assistênci		
14/12/2022	CE	JUAZEIR O DO NORTE	HOSPITA L REGIONA	FALHA NA ADMINIS	Enviada	Eventos Adversos Relaciona	Falhas nas atividades		
14/12/2022	CE	JUAZEIR O DO NORTE	HOSPITA L REGIONA	FALHAS NA ASSISTE	Enviada	Eventos Adversos Relaciona	Falhas durante a assistênci		
14/12/2022	CE	JUAZEIR O DO NORTE	HOSPITA L REGIONA	FALHAS NA ASSISTE	Enviada	Eventos Adversos Relaciona	Lesão por pressão		
14/12/2022	CE	JUAZEIR O DO NORTE	HOSPITA L REGIONA	FALHAS NA ASSISTE	Enviada	Eventos Adversos Relaciona	Queimadu ra de paciente		
15/12/2022	CE	JUAZEIR O DO NORTE	HOSPITA L REGIONA	ERRO DE MEDICA	Enviada	Eventos Adversos Relaciona	Falhas durante a assistênci		
15/12/2022	CE	JUAZEIR O DO NORTE	HOSPITA L REGIONA	FALHA NA ADMINIS	Enviada	Eventos Adversos Relaciona	Falhas durante a assistênci		
15/12/2022	CE	JUAZEIR O DO NORTE	HOSPITA L REGIONA	FALHAS NA ASSISTE	Enviada	Eventos Adversos Relaciona	Lesão por pressão		
15/12/2022	CE	JUAZEIR O DO NORTE	HOSPITA L REGIONA	FALHAS NA ASSISTE	Enviada	Eventos Adversos Relaciona	Falhas durante a assistênci		
15/12/2022	CE	JUAZEIR O DO NORTE	HOSPITA L REGIONA	FALHAS NA ASSISTE	Enviada	Eventos Adversos Relaciona	Falhas durante a assistênci		
15/12/2022	CE	JUAZEIR O DO NORTE	HOSPITA L REGIONA	FALHAS NA ASSISTE	Enviada	Eventos Adversos Relaciona	Falhas durante a assistênci		
17/01/2023	CE	JUAZEIR O DO NORTE	HOSPITA L REGIONA	FALHAS NA ASSISTE	Enviada	Eventos Adversos Relaciona	Queda do paciente		
17/01/2023	CE	JUAZEIR O DO NORTE	HOSPITA L REGIONA	FALHAS NA ASSISTE	Enviada	Eventos Adversos Relaciona	Falhas durante a assistênci		
17/01/2023	CE	JUAZEIR O DO NORTE	HOSPITA L REGIONA	FALHA EM EXAME	Enviada	Eventos Adversos Relaciona	Falhas durante a assistênci		
17/01/2023	CE	JUAZEIR O DO NORTE	HOSPITA L REGIONA	FALHA EM EXAME	Enviada	Eventos Adversos Relaciona	Falhas durante a assistênci		
17/01/2023	CE	JUAZEIR O DO NORTE	HOSPITA L REGIONA	FALHAS NA ASSISTE	Enviada	Eventos Adversos Relaciona	Falhas durante a assistênci		
17/01/2023	CE	JUAZEIR O DO NORTE	HOSPITA L REGIONA	FALHAS NA ASSISTE	Enviada	Eventos Adversos Relaciona	Falhas durante a assistênci		

18/01/2023	CE	JUAZEIR O DO NORTE	HOSPITA L REGIONA	PROBLE MAS DE GESTÃO	Enviada	Eventos Adversos Relaciona	Falhas nas atividades		
18/01/2023	CE	JUAZEIR O DO NORTE	HOSPITA L REGIONA	FALHAS NA ASSISTE	Enviada	Eventos Adversos Relaciona	Falhas durante a assistênci		
18/01/2023	CE	JUAZEIR O DO NORTE	HOSPITA L REGIONA	FALHAS NA ASSISTE	Enviada	Eventos Adversos Relaciona	Falhas durante a assistênci		
18/01/2023	CE	JUAZEIR O DO NORTE	HOSPITA L REGIONA	FALHAS NA ASSISTE	Enviada	Eventos Adversos Relaciona	Falhas durante a assistênci		
18/01/2023	CE	JUAZEIR O DO NORTE	HOSPITA L REGIONA	FALHAS NA ASSISTE	Enviada	Eventos Adversos Relaciona	Falhas durante a assistênci		
18/01/2023	CE	JUAZEIR O DO NORTE	HOSPITA L REGIONA	FALHAS NA ASSISTE	Enviada	Eventos Adversos Relaciona	Falhas durante a assistênci		
18/01/2023	CE	JUAZEIR O DO NORTE	HOSPITA L REGIONA	FALHAS NA ASSISTE	Enviada	Eventos Adversos Relaciona	Falhas durante a assistênci		
18/01/2023	CE	JUAZEIR O DO NORTE	HOSPITA L REGIONA	FALHAS NA ASSISTE	Enviada	Eventos Adversos Relaciona	Falhas durante a assistênci		
18/01/2023	CE	JUAZEIR O DO NORTE	HOSPITA L REGIONA	FALHAS NA ASSISTE	Enviada	Eventos Adversos Relaciona	Falhas durante a assistênci		
18/01/2023	CE	JUAZEIR O DO NORTE	HOSPITA L REGIONA	FALHAS NA ASSISTE	Enviada	Eventos Adversos Relaciona	Falhas durante a assistênci		
18/01/2023	CE	JUAZEIR O DO NORTE	HOSPITA L REGIONA	FALHAS NA ASSISTE	Enviada	Eventos Adversos Relaciona	Falhas durante a assistênci		
18/01/2023	CE	JUAZEIR O DO NORTE	HOSPITA L REGIONA	FALHAS NA ASSISTE	Enviada	Eventos Adversos Relaciona	Falhas durante a assistênci		
18/01/2023	CE	JUAZEIR O DO NORTE	HOSPITA L REGIONA	FALHAS NA ASSISTE	Enviada	Eventos Adversos Relaciona	Falhas durante a assistênci		
18/01/2023	CE	JUAZEIR O DO NORTE	HOSPITA L REGIONA	FALHAS NA ASSISTE	Enviada	Eventos Adversos Relaciona	Falhas durante a assistênci		
19/01/2023	CE	JUAZEIR O DO NORTE	HOSPITA L REGIONA	FALHAS NA ASSISTE	Enviada	Eventos Adversos Relaciona	Falhas durante a assistênci		
19/01/2023	CE	JUAZEIR O DO NORTE	HOSPITA L REGIONA	FALHAS NA ASSISTE	Enviada	Eventos Adversos Relaciona	Falhas durante a assistênci		
19/01/2023	CE	JUAZEIR O DO NORTE	HOSPITA L REGIONA	FALHAS NA ASSISTE	Enviada	Eventos Adversos Relaciona	Falhas durante a assistênci		
19/01/2023	CE	JUAZEIR O DO NORTE	HOSPITA L REGIONA	FALHAS NA ASSISTE	Enviada	Eventos Adversos Relaciona	Falhas durante a assistênci		
16/02/2023	CE	JUAZEIR O DO NORTE	HOSPITA L REGIONA	FALHAS NA ASSISTE	Enviada	Eventos Adversos Relaciona	Falhas durante a assistênci		
16/02/2023	CE	JUAZEIR O DO NORTE	HOSPITA L REGIONA	FALHAS NA ASSISTE	Enviada	Eventos Adversos Relaciona	Falhas durante a assistênci		
16/02/2023	CE	JUAZEIR O DO NORTE	HOSPITA L REGIONA	FALHAS NA ASSISTE	Enviada	Eventos Adversos Relaciona	Falhas durante a assistênci		
16/02/2023	CE	JUAZEIR O DO NORTE	HOSPITA L REGIONA	FALHAS NA ASSISTE	Enviada	Eventos Adversos Relaciona	Falhas durante a assistênci		

16/02/2023	CE	JUAZEIR O DO NORTE	HOSPITA L REGIONA	FALHAS NA ASSISTE	Enviada	Eventos Adversos Relaciona	Falhas durante a assistênci		
16/02/2023	CE	JUAZEIR O DO NORTE	HOSPITA L REGIONA	FALHAS NA ASSISTE	Enviada	Eventos Adversos Relaciona	Falhas envolvend o sondas		
16/02/2023	CE	JUAZEIR O DO NORTE	HOSPITA L REGIONA	FALHA EM EXAME	Enviada	Eventos Adversos Relaciona	Falhas na assistênci a		
16/02/2023	CE	JUAZEIR O DO NORTE	HOSPITA L REGIONA	PROBLE MAS DE GESTÃO	Enviada	Eventos Adversos Relaciona	Falhas durante a assistênci		
16/02/2023	CE	JUAZEIR O DO NORTE	HOSPITA L REGIONA	PROBLE MAS DE GESTÃO	Enviada	Eventos Adversos Relaciona	Falhas na assistênci a		
16/02/2023	CE	JUAZEIR O DO NORTE	HOSPITA L REGIONA	FALHAS NA ASSISTE	Enviada	Eventos Adversos Relaciona	Falhas durante a assistênci		
16/02/2023	CE	JUAZEIR O DO NORTE	HOSPITA L REGIONA	FALHAS NA ASSISTE	Enviada	Eventos Adversos Relaciona	Falhas durante a assistênci		
16/02/2023	CE	JUAZEIR O DO NORTE	HOSPITA L REGIONA	FALHA NA ADMINIS	Enviada	Eventos Adversos Relaciona	Falhas durante a assistênci		
16/02/2023	CE	JUAZEIR O DO NORTE	HOSPITA L REGIONA	PROBLE MAS DE GESTÃO	Enviada	Eventos Adversos Relaciona	Falhas durante a assistênci		
16/02/2023	CE	JUAZEIR O DO NORTE	HOSPITA L REGIONA	FALHAS NA ASSISTE	Enviada	Eventos Adversos Relaciona	Falhas durante a assistênci		
16/02/2023	CE	JUAZEIR O DO NORTE	HOSPITA L REGIONA	FALHA NA ADMINIS	Enviada	Eventos Adversos Relaciona	Falhas nas atividades		
16/02/2023	CE	JUAZEIR O DO NORTE	HOSPITA L REGIONA	FALHA NA ADMINIS	Enviada	Eventos Adversos Relaciona	Falhas nas atividades		
16/02/2023	CE	JUAZEIR O DO NORTE	HOSPITA L REGIONA	FALHAS NA ASSISTE	Enviada	Eventos Adversos Relaciona	Falhas envolvend o sondas		
16/02/2023	CE	JUAZEIR O DO NORTE	HOSPITA L REGIONA	FALHAS NA ASSISTE	Enviada	Eventos Adversos Relaciona	Falhas envolvend o sondas		
16/02/2023	CE	JUAZEIR O DO NORTE	HOSPITA L REGIONA	ERROS DE DIETA -	Enviada	Eventos Adversos Relaciona	Falhas na administra ção de		Enteral (oral, cateter
16/02/2023	CE	JUAZEIR O DO NORTE	HOSPITA L REGIONA	FALHAS NA ASSISTE	Enviada	Eventos Adversos Relaciona	Falhas envolvend o sondas		
16/02/2023	CE	JUAZEIR O DO NORTE	HOSPITA L REGIONA	FALHAS NA ASSISTE	Enviada	Eventos Adversos Relaciona	Falhas nas atividades		
16/02/2023	CE	JUAZEIR O DO NORTE	HOSPITA L REGIONA	FALHA NO PROCEDI	Enviada	Eventos Adversos Relaciona	Falhas nas atividades		
16/02/2023	CE	JUAZEIR O DO NORTE	HOSPITA L REGIONA	PROBLE MAS DE GESTÃO	Enviada	Eventos Adversos Relaciona	Falhas nas atividades		
21/03/2023	CE	JUAZEIR O DO NORTE	HOSPITA L REGIONA	FALHAS NA ASSISTE	Enviada	Eventos Adversos Relaciona	Lesão por pressão		
21/03/2023	CE	JUAZEIR O DO NORTE	HOSPITA L REGIONA	FALHAS NA ASSISTE	Enviada	Eventos Adversos Relaciona	Falhas durante a assistênci		
21/03/2023	CE	JUAZEIR O DO NORTE	HOSPITA L REGIONA	FALHA NO PROCEDI	Enviada	Eventos Adversos Relaciona	Falhas durante a assistênci		

21/03/2023	CE	JUAZEIR O DO NORTE	HOSPITA L REGIONA	FALHAS NA ASSISTE	Enviada	Eventos Adversos Relaciona	Falhas envolvend o sondas		
21/03/2023	CE	JUAZEIR O DO NORTE	HOSPITA L REGIONA	FALHAS NA ASSISTE	Enviada	Eventos Adversos Relaciona	Falhas durante a assistênci		
21/03/2023	CE	JUAZEIR O DO NORTE	HOSPITA L REGIONA	FALHA NA ADMINIS	Enviada	Eventos Adversos Relaciona	Falhas durante a assistênci		
21/03/2023	CE	JUAZEIR O DO NORTE	HOSPITA L REGIONA	FALHAS NA ASSISTE	Enviada	Eventos Adversos Relaciona	Falhas nas atividades		
21/03/2023	CE	JUAZEIR O DO NORTE	HOSPITA L REGIONA	FALHAS NA ASSISTE	Enviada	Eventos Adversos Relaciona	Falhas envolvend o cateter		
21/03/2023	CE	JUAZEIR O DO NORTE	HOSPITA L REGIONA	PROBLE MA RELACIO	Enviada	Eventos Adversos Relaciona	Falhas durante a assistênci		
21/03/2023	CE	JUAZEIR O DO NORTE	HOSPITA L REGIONA	FALHAS NA ASSISTE	Enviada	Eventos Adversos Relaciona	Falhas envolvend o cateter		
21/03/2023	CE	JUAZEIR O DO NORTE	HOSPITA L REGIONA	FALHAS NA ASSISTE	Enviada	Eventos Adversos Relaciona	Lesão por pressão		
21/03/2023	CE	JUAZEIR O DO NORTE	HOSPITA L REGIONA	FALHA NA ADMINIS	Enviada	Eventos Adversos Relaciona	Falhas durante a assistênci		
21/03/2023	CE	JUAZEIR O DO NORTE	HOSPITA L REGIONA	FALHAS NA ASSISTE	Enviada	Eventos Adversos Relaciona	Falhas durante a assistênci		
21/03/2023	CE	JUAZEIR O DO NORTE	HOSPITA L REGIONA	FALHA NA ADMINIS	Enviada	Eventos Adversos Relaciona	Falhas durante a assistênci		
21/03/2023	CE	JUAZEIR O DO NORTE	HOSPITA L REGIONA	FALHAS NA ASSISTE	Enviada	Eventos Adversos Relaciona	Acidentes do paciente	Outro tipo de mecanism	
21/03/2023	CE	JUAZEIR O DO NORTE	HOSPITA L REGIONA	FALHAS NA ASSISTE	Enviada	Eventos Adversos Relaciona	Falhas durante a assistênci		
22/03/2023	CE	JUAZEIR O DO NORTE	HOSPITA L REGIONA	FALHAS NA ASSISTE	Enviada	Eventos Adversos Relaciona	Falhas durante a assistênci		
22/03/2023	CE	JUAZEIR O DO NORTE	HOSPITA L REGIONA	FALHA NO PROCEDI	Enviada	Eventos Adversos Relaciona	Falhas durante a assistênci		
22/03/2023	CE	JUAZEIR O DO NORTE	HOSPITA L REGIONA	FALHAS NA ASSISTE	Enviada	Eventos Adversos Relaciona	Falhas durante a assistênci		
22/03/2023	CE	JUAZEIR O DO NORTE	HOSPITA L REGIONA	FALHAS NA ASSISTE	Enviada	Eventos Adversos Relaciona	Falhas envolvend o cateter		
22/03/2023	CE	JUAZEIR O DO NORTE	HOSPITA L REGIONA	FALHAS NA ASSISTE	Enviada	Eventos Adversos Relaciona	Falhas envolvend o cateter		
22/03/2023	CE	JUAZEIR O DO NORTE	HOSPITA L REGIONA	FALHAS NA ASSISTE	Enviada	Eventos Adversos Relaciona	Falhas na assistênci a		
22/03/2023	CE	JUAZEIR O DO NORTE	HOSPITA L REGIONA	FALHAS NA ASSISTE	Enviada	Eventos Adversos Relaciona	Falhas envolvend o cateter		
23/03/2023	CE	JUAZEIR O DO NORTE	HOSPITA L REGIONA	FALHA NA ADMINIS	Enviada	Eventos Adversos Relaciona	Falhas durante a assistênci		
23/03/2023	CE	JUAZEIR O DO NORTE	HOSPITA L REGIONA	ERRO DE MEDICA	Enviada	Eventos Adversos Relaciona	Falhas durante a assistênci		

23/03/2023	CE	JUAZEIRO DO NORTE	HOSPITAL REGIONA	FALHA NO PROCEDI	Enviada	Eventos Adversos Relaciona	Falhas durante a assistênci		
23/03/2023	CE	JUAZEIRO DO NORTE	HOSPITAL REGIONA	FALHAS NA ASSISTE	Enviada	Eventos Adversos Relaciona	Falhas envolvendo cateter		
23/03/2023	CE	JUAZEIRO DO NORTE	HOSPITAL REGIONA	FALHAS NA ASSISTE	Enviada	Eventos Adversos Relaciona	Falhas durante a assistênci		
23/03/2023	CE	JUAZEIRO DO NORTE	HOSPITAL REGIONA	FALHA EM EXAME	Enviada	Eventos Adversos Relaciona	Falhas na assistência		
23/03/2023	CE	JUAZEIRO DO NORTE	HOSPITAL REGIONA	FALHA NO PROCEDI	Enviada	Eventos Adversos Relaciona	Falhas durante a assistênci		
23/03/2023	CE	JUAZEIRO DO NORTE	HOSPITAL REGIONA	FALHA NO PROCEDI	Enviada	Eventos Adversos Relaciona	Falhas durante a assistênci		
23/03/2023	CE	JUAZEIRO DO NORTE	HOSPITAL REGIONA	FALHA NO PROCEDI	Enviada	Eventos Adversos Relaciona	Falhas durante a assistênci		
23/03/2023	CE	JUAZEIRO DO NORTE	HOSPITAL REGIONA	FALHAS NA ASSISTE	Enviada	Eventos Adversos Relaciona	Falhas na administração de		Enteral (oral, cateter
23/03/2023	CE	JUAZEIRO DO NORTE	HOSPITAL REGIONA	FALHAS NA ASSISTE	Enviada	Eventos Adversos Relaciona	Lesão por pressão		
23/03/2023	CE	JUAZEIRO DO NORTE	HOSPITAL REGIONA	FALHAS NA ASSISTE	Enviada	Eventos Adversos Relaciona	Lesão por pressão		
23/03/2023	CE	JUAZEIRO DO NORTE	HOSPITAL REGIONA	FALHAS NA ASSISTE	Enviada	Eventos Adversos Relaciona	Falhas durante a assistênci		
23/03/2023	CE	JUAZEIRO DO NORTE	HOSPITAL REGIONA	FALHAS NA ASSISTE	Enviada	Eventos Adversos Relaciona	Lesão por pressão		
23/03/2023	CE	JUAZEIRO DO NORTE	HOSPITAL REGIONA	FALHA NO PROCEDI	Enviada	Eventos Adversos Relaciona	Falhas durante a assistênci		
29/03/2019	CE	SOBRAL	HOSPITAL REGIONA	-	Enviada	Eventos Adversos Relaciona	Lesão por pressão		
29/03/2019	CE	SOBRAL	HOSPITAL REGIONA	-	Enviada	Eventos Adversos Relaciona	Lesão por pressão		
29/03/2019	CE	SOBRAL	HOSPITAL REGIONA	-	Enviada	Eventos Adversos Relaciona	Lesão por pressão		
29/03/2019	CE	SOBRAL	HOSPITAL REGIONA	-	Enviada	Eventos Adversos Relaciona	Lesão por pressão		
29/03/2019	CE	SOBRAL	HOSPITAL REGIONA	-	Enviada	Eventos Adversos Relaciona	Lesão por pressão		
29/03/2019	CE	SOBRAL	HOSPITAL REGIONA	-	Enviada	Eventos Adversos Relaciona	Lesão por pressão		
29/03/2019	CE	SOBRAL	HOSPITAL REGIONA	-	Enviada	Eventos Adversos Relaciona	Lesão por pressão		
16/04/2019	CE	SOBRAL	HOSPITAL REGIONA	-	Enviada	Eventos Adversos Relaciona	Lesão por pressão		

16/04/2019	CE	SOBRAL	HOSPITA L REGIONA	-	Enviada	Eventos Adversos Relaciona	Lesão por pressão		
16/04/2019	CE	SOBRAL	HOSPITA L REGIONA	-	Enviada	Eventos Adversos Relaciona	Lesão por pressão		
16/04/2019	CE	SOBRAL	HOSPITA L REGIONA	-	Enviada	Eventos Adversos Relaciona	Lesão por pressão		
16/04/2019	CE	SOBRAL	HOSPITA L REGIONA	-	Enviada	Eventos Adversos Relaciona	Lesão por pressão		
16/04/2019	CE	SOBRAL	HOSPITA L REGIONA	-	Enviada	Eventos Adversos Relaciona	Lesão por pressão		
16/04/2019	CE	SOBRAL	HOSPITA L REGIONA	-	Enviada	Eventos Adversos Relaciona	Lesão por pressão		
16/04/2019	CE	SOBRAL	HOSPITA L REGIONA	-	Enviada	Eventos Adversos Relaciona	Lesão por pressão		
16/04/2019	CE	SOBRAL	HOSPITA L REGIONA	-	Enviada	Eventos Adversos Relaciona	Lesão por pressão		
16/04/2019	CE	SOBRAL	HOSPITA L REGIONA	-	Enviada	Eventos Adversos Relaciona	Lesão por pressão		
16/04/2019	CE	SOBRAL	HOSPITA L REGIONA	-	Enviada	Eventos Adversos Relaciona	Lesão por pressão		
16/04/2019	CE	SOBRAL	HOSPITA L REGIONA	-	Enviada	Eventos Adversos Relaciona	Lesão por pressão		
16/04/2019	CE	SOBRAL	HOSPITA L REGIONA	-	Enviada	Eventos Adversos Relaciona	Lesão por pressão		
16/04/2019	CE	SOBRAL	HOSPITA L REGIONA	-	Enviada	Eventos Adversos Relaciona	Lesão por pressão		
16/04/2019	CE	SOBRAL	HOSPITA L REGIONA	-	Enviada	Eventos Adversos Relaciona	Lesão por pressão		
16/04/2019	CE	SOBRAL	HOSPITA L REGIONA	-	Enviada	Eventos Adversos Relaciona	Lesão por pressão		
16/04/2019	CE	SOBRAL	HOSPITA L REGIONA	-	Enviada	Eventos Adversos Relaciona	Lesão por pressão		
16/04/2019	CE	SOBRAL	HOSPITA L REGIONA	-	Enviada	Eventos Adversos Relaciona	Lesão por pressão		
16/04/2019	CE	SOBRAL	HOSPITA L REGIONA	-	Enviada	Eventos Adversos Relaciona	Lesão por pressão		
16/04/2019	CE	SOBRAL	HOSPITA L REGIONA	-	Enviada	Eventos Adversos Relaciona	Lesão por pressão		

16/04/2019	CE	SOBRAL	HOSPITA L REGIONA	-	Enviada	Eventos Adversos Relaciona	Lesão por pressão		
16/04/2019	CE	SOBRAL	HOSPITA L REGIONA	-	Enviada	Eventos Adversos Relaciona	Lesão por pressão		
16/04/2019	CE	SOBRAL	HOSPITA L REGIONA	-	Enviada	Eventos Adversos Relaciona	Lesão por pressão		
16/04/2019	CE	SOBRAL	HOSPITA L REGIONA	-	Enviada	Eventos Adversos Relaciona	Lesão por pressão		
16/04/2019	CE	SOBRAL	HOSPITA L REGIONA	-	Enviada	Eventos Adversos Relaciona	Lesão por pressão		
16/04/2019	CE	SOBRAL	HOSPITA L REGIONA	-	Enviada	Eventos Adversos Relaciona	Lesão por pressão		
17/04/2019	CE	SOBRAL	HOSPITA L REGIONA	-	Enviada	Eventos Adversos Relaciona	Lesão por pressão		
17/04/2019	CE	SOBRAL	HOSPITA L REGIONA	-	Enviada	Eventos Adversos Relaciona	Lesão por pressão		
17/04/2019	CE	SOBRAL	HOSPITA L REGIONA	-	Enviada	Eventos Adversos Relaciona	Lesão por pressão		
17/04/2019	CE	SOBRAL	HOSPITA L REGIONA	-	Enviada	Eventos Adversos Relaciona	Lesão por pressão		
17/04/2019	CE	SOBRAL	HOSPITA L REGIONA	-	Enviada	Eventos Adversos Relaciona	Lesão por pressão		
17/04/2019	CE	SOBRAL	HOSPITA L REGIONA	-	Enviada	Eventos Adversos Relaciona	Lesão por pressão		
17/04/2019	CE	SOBRAL	HOSPITA L REGIONA	-	Enviada	Eventos Adversos Relaciona	Lesão por pressão		
17/04/2019	CE	SOBRAL	HOSPITA L REGIONA	-	Enviada	Eventos Adversos Relaciona	Lesão por pressão		
17/04/2019	CE	SOBRAL	HOSPITA L REGIONA	-	Enviada	Eventos Adversos Relaciona	Lesão por pressão		
17/04/2019	CE	SOBRAL	HOSPITA L REGIONA	-	Enviada	Eventos Adversos Relaciona	Lesão por pressão		
17/04/2019	CE	SOBRAL	HOSPITA L REGIONA	-	Enviada	Eventos Adversos Relaciona	Lesão por pressão		
17/04/2019	CE	SOBRAL	HOSPITA L REGIONA	-	Enviada	Eventos Adversos Relaciona	Lesão por pressão		
17/04/2019	CE	SOBRAL	HOSPITA L REGIONA	-	Enviada	Eventos Adversos Relaciona	Lesão por pressão		

17/04/2019	CE	SOBRAL	HOSPITA L REGIONA	-	Enviada	Eventos Adversos Relaciona	Lesão por pressão		
17/04/2019	CE	SOBRAL	HOSPITA L REGIONA	-	Enviada	Eventos Adversos Relaciona	Lesão por pressão		
17/04/2019	CE	SOBRAL	HOSPITA L REGIONA	-	Enviada	Eventos Adversos Relaciona	Lesão por pressão		
17/04/2019	CE	SOBRAL	HOSPITA L REGIONA	-	Enviada	Eventos Adversos Relaciona	Lesão por pressão		
17/04/2019	CE	SOBRAL	HOSPITA L REGIONA	-	Enviada	Eventos Adversos Relaciona	Lesão por pressão		
17/04/2019	CE	SOBRAL	HOSPITA L REGIONA	-	Enviada	Eventos Adversos Relaciona	Lesão por pressão		
17/04/2019	CE	SOBRAL	HOSPITA L REGIONA	-	Enviada	Eventos Adversos Relaciona	Lesão por pressão		
17/04/2019	CE	SOBRAL	HOSPITA L REGIONA	-	Enviada	Eventos Adversos Relaciona	Lesão por pressão		
17/04/2019	CE	SOBRAL	HOSPITA L REGIONA	-	Enviada	Eventos Adversos Relaciona	Lesão por pressão		
17/04/2019	CE	SOBRAL	HOSPITA L REGIONA	-	Enviada	Eventos Adversos Relaciona	Lesão por pressão		
17/04/2019	CE	SOBRAL	HOSPITA L REGIONA	-	Enviada	Eventos Adversos Relaciona	Lesão por pressão		
17/04/2019	CE	SOBRAL	HOSPITA L REGIONA	-	Enviada	Eventos Adversos Relaciona	Lesão por pressão		
17/04/2019	CE	SOBRAL	HOSPITA L REGIONA	-	Enviada	Eventos Adversos Relaciona	Lesão por pressão		
17/04/2019	CE	SOBRAL	HOSPITA L REGIONA	-	Enviada	Eventos Adversos Relaciona	Lesão por pressão		
17/04/2019	CE	SOBRAL	HOSPITA L REGIONA	-	Enviada	Eventos Adversos Relaciona	Lesão por pressão		
17/04/2019	CE	SOBRAL	HOSPITA L REGIONA	-	Enviada	Eventos Adversos Relaciona	Lesão por pressão		
17/04/2019	CE	SOBRAL	HOSPITA L REGIONA	-	Enviada	Eventos Adversos Relaciona	Lesão por pressão		
17/04/2019	CE	SOBRAL	HOSPITA L REGIONA	-	Enviada	Eventos Adversos Relaciona	Lesão por pressão		
17/04/2019	CE	SOBRAL	HOSPITA L REGIONA	-	Enviada	Eventos Adversos Relaciona	Falhas durante a assistênci		

17/04/2019	CE	SOBRAL	HOSPITA L REGIONA	-	Enviada	Eventos Adversos Relaciona	Falhas durante a assistênci		
17/04/2019	CE	SOBRAL	HOSPITA L REGIONA	-	Enviada	Eventos Adversos Relaciona	Falhas durante a assistênci		
17/04/2019	CE	SOBRAL	HOSPITA L REGIONA	-	Enviada	Eventos Adversos Relaciona	Falhas durante a assistênci		
17/04/2019	CE	SOBRAL	HOSPITA L REGIONA	-	Enviada	Eventos Adversos Relaciona	Falhas durante a assistênci		
17/04/2019	CE	SOBRAL	HOSPITA L REGIONA	-	Enviada	Eventos Adversos Relaciona	Falhas durante a assistênci		
17/04/2019	CE	SOBRAL	HOSPITA L REGIONA	-	Enviada	Eventos Adversos Relaciona	Falhas durante a assistênci		
17/04/2019	CE	SOBRAL	HOSPITA L REGIONA	-	Enviada	Eventos Adversos Relaciona	Falhas durante a assistênci		
17/04/2019	CE	SOBRAL	HOSPITA L REGIONA	-	Enviada	Eventos Adversos Relaciona	Falhas durante a assistênci		
17/04/2019	CE	SOBRAL	HOSPITA L REGIONA	-	Enviada	Eventos Adversos Relaciona	Falhas durante a assistênci		
17/04/2019	CE	SOBRAL	HOSPITA L REGIONA	-	Enviada	Eventos Adversos Relaciona	Falhas durante a assistênci		
17/04/2019	CE	SOBRAL	HOSPITA L REGIONA	-	Enviada	Eventos Adversos Relaciona	Falhas durante a assistênci		
17/04/2019	CE	SOBRAL	HOSPITA L REGIONA	-	Enviada	Eventos Adversos Relaciona	Falhas durante a assistênci		
17/04/2019	CE	SOBRAL	HOSPITA L REGIONA	-	Enviada	Eventos Adversos Relaciona	Falhas durante a assistênci		
17/04/2019	CE	SOBRAL	HOSPITA L REGIONA	-	Enviada	Eventos Adversos Relaciona	Falhas durante a assistênci		
17/04/2019	CE	SOBRAL	HOSPITA L REGIONA	-	Enviada	Eventos Adversos Relaciona	Falhas durante a assistênci		
17/04/2019	CE	SOBRAL	HOSPITA L REGIONA	-	Enviada	Eventos Adversos Relaciona	Falhas durante a assistênci		
17/04/2019	CE	SOBRAL	HOSPITA L REGIONA	-	Enviada	Eventos Adversos Relaciona	Falhas durante a assistênci		
17/04/2019	CE	SOBRAL	HOSPITA L REGIONA	-	Enviada	Eventos Adversos Relaciona	Falhas durante a assistênci		
17/04/2019	CE	SOBRAL	HOSPITA L REGIONA	-	Enviada	Eventos Adversos Relaciona	Falhas durante a assistênci		

17/04/2019	CE	SOBRAL	HOSPITA L REGIONA	-	Enviada	Eventos Adversos Relaciona	Falhas durante a assistênci		
17/04/2019	CE	SOBRAL	HOSPITA L REGIONA	-	Enviada	Eventos Adversos Relaciona	Falhas durante a assistênci		
17/04/2019	CE	SOBRAL	HOSPITA L REGIONA	-	Enviada	Eventos Adversos Relaciona	Falhas durante a assistênci		
17/04/2019	CE	SOBRAL	HOSPITA L REGIONA	-	Enviada	Eventos Adversos Relaciona	Falhas durante a assistênci		
17/04/2019	CE	SOBRAL	HOSPITA L REGIONA	-	Enviada	Eventos Adversos Relaciona	Falhas durante a assistênci		
17/04/2019	CE	SOBRAL	HOSPITA L REGIONA	-	Enviada	Eventos Adversos Relaciona	Falhas durante a assistênci		
17/04/2019	CE	SOBRAL	HOSPITA L REGIONA	-	Enviada	Eventos Adversos Relaciona	Falhas durante a assistênci		
17/04/2019	CE	SOBRAL	HOSPITA L REGIONA	-	Enviada	Eventos Adversos Relaciona	Falhas durante a assistênci		
17/04/2019	CE	SOBRAL	HOSPITA L REGIONA	-	Enviada	Eventos Adversos Relaciona	Falhas durante a assistênci		
17/04/2019	CE	SOBRAL	HOSPITA L REGIONA	-	Enviada	Eventos Adversos Relaciona	Falhas durante a assistênci		
17/04/2019	CE	SOBRAL	HOSPITA L REGIONA	-	Enviada	Eventos Adversos Relaciona	Falhas durante a assistênci		
17/04/2019	CE	SOBRAL	HOSPITA L REGIONA	-	Enviada	Eventos Adversos Relaciona	Falhas durante a assistênci		
17/04/2019	CE	SOBRAL	HOSPITA L REGIONA	-	Enviada	Eventos Adversos Relaciona	Falhas durante a assistênci		
17/04/2019	CE	SOBRAL	HOSPITA L REGIONA	-	Enviada	Eventos Adversos Relaciona	Falhas durante a assistênci		
17/04/2019	CE	SOBRAL	HOSPITA L REGIONA	-	Enviada	Eventos Adversos Relaciona	Falhas durante a assistênci		
17/04/2019	CE	SOBRAL	HOSPITA L REGIONA	-	Enviada	Eventos Adversos Relaciona	Falhas durante a assistênci		
17/04/2019	CE	SOBRAL	HOSPITA L REGIONA	-	Enviada	Eventos Adversos Relaciona	Falhas durante a assistênci		
17/04/2019	CE	SOBRAL	HOSPITA L REGIONA	-	Enviada	Eventos Adversos Relaciona	Falhas durante a assistênci		
17/04/2019	CE	SOBRAL	HOSPITA L REGIONA	-	Enviada	Eventos Adversos Relaciona	Falhas durante a assistênci		

17/04/2019	CE	SOBRAL	HOSPITA L REGIONA	-	Enviada	Eventos Adversos Relaciona	Falhas durante a assistênci		
17/04/2019	CE	SOBRAL	HOSPITA L REGIONA	-	Enviada	Eventos Adversos Relaciona	Falhas durante a assistênci		
17/04/2019	CE	SOBRAL	HOSPITA L REGIONA	-	Enviada	Eventos Adversos Relaciona	Falhas durante a assistênci		
17/04/2019	CE	SOBRAL	HOSPITA L REGIONA	-	Enviada	Eventos Adversos Relaciona	Falhas durante a assistênci		
17/04/2019	CE	SOBRAL	HOSPITA L REGIONA	-	Enviada	Eventos Adversos Relaciona	Falhas durante a assistênci		
17/04/2019	CE	SOBRAL	HOSPITA L REGIONA	-	Enviada	Eventos Adversos Relaciona	Falhas durante a assistênci		
17/04/2019	CE	SOBRAL	HOSPITA L REGIONA	-	Enviada	Eventos Adversos Relaciona	Falhas durante a assistênci		
17/04/2019	CE	SOBRAL	HOSPITA L REGIONA	-	Enviada	Eventos Adversos Relaciona	Falhas durante a assistênci		
17/04/2019	CE	SOBRAL	HOSPITA L REGIONA	-	Enviada	Eventos Adversos Relaciona	Falhas durante a assistênci		
17/04/2019	CE	SOBRAL	HOSPITA L REGIONA	-	Enviada	Eventos Adversos Relaciona	Falhas durante a assistênci		
17/04/2019	CE	SOBRAL	HOSPITA L REGIONA	-	Enviada	Eventos Adversos Relaciona	Falhas durante a assistênci		
17/04/2019	CE	SOBRAL	HOSPITA L REGIONA	-	Enviada	Eventos Adversos Relaciona	Falhas durante a assistênci		
17/04/2019	CE	SOBRAL	HOSPITA L REGIONA	-	Enviada	Eventos Adversos Relaciona	Falhas durante a assistênci		
17/04/2019	CE	SOBRAL	HOSPITA L REGIONA	-	Enviada	Eventos Adversos Relaciona	Falhas durante a assistênci		
17/04/2019	CE	SOBRAL	HOSPITA L REGIONA	-	Enviada	Eventos Adversos Relaciona	Falhas ocorridas em		
17/04/2019	CE	SOBRAL	HOSPITA L REGIONA	-	Enviada	Eventos Adversos Relaciona	Falhas durante a assistênci		
17/04/2019	CE	SOBRAL	HOSPITA L REGIONA	-	Enviada	Eventos Adversos Relaciona	Falhas durante a assistênci		
17/04/2019	CE	SOBRAL	HOSPITA L REGIONA	-	Enviada	Eventos Adversos Relaciona	Falhas durante a assistênci		
17/04/2019	CE	SOBRAL	HOSPITA L REGIONA	-	Enviada	Eventos Adversos Relaciona	Falhas durante a assistênci		

18/04/2019	CE	SOBRAL	HOSPITA L REGIONA	-	Enviada	Eventos Adversos Relaciona	Falhas na administra ção de		Enteral (oral, cateter
18/04/2019	CE	SOBRAL	HOSPITA L REGIONA	-	Enviada	Eventos Adversos Relaciona	Falhas na administra ção de		Enteral (oral, cateter
18/04/2019	CE	SOBRAL	HOSPITA L REGIONA	-	Enviada	Eventos Adversos Relaciona	Falhas na administra ção de		Enteral (oral, cateter
18/04/2019	CE	SOBRAL	HOSPITA L REGIONA	-	Enviada	Eventos Adversos Relaciona	Falhas na administra ção de		Enteral (oral, cateter
18/04/2019	CE	SOBRAL	HOSPITA L REGIONA	-	Enviada	Eventos Adversos Relaciona	Falhas na administra ção de		Enteral (oral, cateter
18/04/2019	CE	SOBRAL	HOSPITA L REGIONA	-	Enviada	Eventos Adversos Relaciona	Falhas na administra ção de		Enteral (oral, cateter
18/04/2019	CE	SOBRAL	HOSPITA L REGIONA	-	Enviada	Eventos Adversos Relaciona	Falhas na administra ção de		Parenteral (via intravenos
18/04/2019	CE	SOBRAL	HOSPITA L REGIONA	-	Enviada	Eventos Adversos Relaciona	Falhas na administra ção de		Enteral (oral, cateter
18/04/2019	CE	SOBRAL	HOSPITA L REGIONA	-	Enviada	Eventos Adversos Relaciona	Falhas na administra ção de		Enteral (oral, cateter
18/04/2019	CE	SOBRAL	HOSPITA L REGIONA	-	Enviada	Eventos Adversos Relaciona	Falhas na administra ção de		Enteral (oral, cateter
18/04/2019	CE	SOBRAL	HOSPITA L REGIONA	-	Enviada	Eventos Adversos Relaciona	Falhas na administra ção de		Enteral (oral, cateter
18/04/2019	CE	SOBRAL	HOSPITA L REGIONA	-	Enviada	Eventos Adversos Relaciona	Falhas na administra ção de		Enteral (oral, cateter
18/04/2019	CE	SOBRAL	HOSPITA L REGIONA	-	Enviada	Eventos Adversos Relaciona	Falhas na administra ção de		Enteral (oral, cateter
18/04/2019	CE	SOBRAL	HOSPITA L REGIONA	-	Enviada	Eventos Adversos Relaciona	Falhas na administra ção de		Enteral (oral, cateter
18/04/2019	CE	SOBRAL	HOSPITA L REGIONA	-	Enviada	Eventos Adversos Relaciona	Falhas na administra ção de		Enteral (oral, cateter
18/04/2019	CE	SOBRAL	HOSPITA L REGIONA	-	Enviada	Eventos Adversos Relaciona	Falhas na administra ção de		Enteral (oral, cateter
18/04/2019	CE	SOBRAL	HOSPITA L REGIONA	-	Enviada	Eventos Adversos Relaciona	Falhas na administra ção de		Enteral (oral, cateter
18/04/2019	CE	SOBRAL	HOSPITA L REGIONA	-	Enviada	Eventos Adversos Relaciona	Falhas na administra ção de		Enteral (oral, cateter
18/04/2019	CE	SOBRAL	HOSPITA L REGIONA	-	Enviada	Eventos Adversos Relaciona	Falhas na administra ção de		Enteral (oral, cateter

18/04/2019	CE	SOBRAL	HOSPITA L REGIONA	-	Enviada	Eventos Adversos Relaciona	Falhas na administra ção de		Enteral (oral, cateter
18/04/2019	CE	SOBRAL	HOSPITA L REGIONA	-	Enviada	Eventos Adversos Relaciona	Falhas na administra ção de		Enteral (oral, cateter
18/04/2019	CE	SOBRAL	HOSPITA L REGIONA	-	Enviada	Eventos Adversos Relaciona	Falhas na administra ção de		Enteral (oral, cateter
18/04/2019	CE	SOBRAL	HOSPITA L REGIONA	-	Enviada	Eventos Adversos Relaciona	Falhas na administra ção de		Enteral (oral, cateter
18/04/2019	CE	SOBRAL	HOSPITA L REGIONA	-	Enviada	Eventos Adversos Relaciona	Falhas na administra ção de		Enteral (oral, cateter
18/04/2019	CE	SOBRAL	HOSPITA L REGIONA	-	Enviada	Eventos Adversos Relaciona	Falhas na administra ção de		Enteral (oral, cateter
18/04/2019	CE	SOBRAL	HOSPITA L REGIONA	-	Enviada	Eventos Adversos Relaciona	Falhas na administra ção de		Enteral (oral, cateter
18/04/2019	CE	SOBRAL	HOSPITA L REGIONA	-	Enviada	Eventos Adversos Relaciona	Falhas na administra ção de		Enteral (oral, cateter
18/04/2019	CE	SOBRAL	HOSPITA L REGIONA	-	Enviada	Eventos Adversos Relaciona	Falhas na administra ção de		Enteral (oral, cateter
18/04/2019	CE	SOBRAL	HOSPITA L REGIONA	-	Enviada	Eventos Adversos Relaciona	Falhas na administra ção de		Enteral (oral, cateter
18/04/2019	CE	SOBRAL	HOSPITA L REGIONA	-	Enviada	Eventos Adversos Relaciona	Falhas na administra ção de		Enteral (oral, cateter
18/04/2019	CE	SOBRAL	HOSPITA L REGIONA	-	Enviada	Eventos Adversos Relaciona	Falhas na administra ção de		Enteral (oral, cateter
18/04/2019	CE	SOBRAL	HOSPITA L REGIONA	-	Enviada	Eventos Adversos Relaciona	Falhas na administra ção de		Enteral (oral, cateter
18/04/2019	CE	SOBRAL	HOSPITA L REGIONA	-	Enviada	Eventos Adversos Relaciona	Falhas na administra ção de		Enteral (oral, cateter
18/04/2019	CE	SOBRAL	HOSPITA L REGIONA	-	Enviada	Eventos Adversos Relaciona	Falhas na administra ção de		Enteral (oral, cateter
18/04/2019	CE	SOBRAL	HOSPITA L REGIONA	-	Enviada	Eventos Adversos Relaciona	Falhas na administra ção de		Enteral (oral, cateter
18/04/2019	CE	SOBRAL	HOSPITA L REGIONA	-	Enviada	Eventos Adversos Relaciona	Falhas na administra ção de		Enteral (oral, cateter
18/04/2019	CE	SOBRAL	HOSPITA L REGIONA	-	Enviada	Eventos Adversos Relaciona	Falhas na administra ção de		Enteral (oral, cateter
22/04/2019	CE	SOBRAL	HOSPITA L REGIONA	-	Enviada	Eventos Adversos Relaciona	Falhas na administra ção de		Enteral (oral, cateter

22/04/2019	CE	SOBRAL	HOSPITA L REGIONA	-	Enviada	Eventos Adversos Relaciona	Falhas na administra ção de		Enteral (oral, cateter
22/04/2019	CE	SOBRAL	HOSPITA L REGIONA	-	Enviada	Eventos Adversos Relaciona	Falhas na administra ção de		Enteral (oral, cateter
22/04/2019	CE	SOBRAL	HOSPITA L REGIONA	-	Enviada	Eventos Adversos Relaciona	Falhas na administra ção de		Enteral (oral, cateter
22/04/2019	CE	SOBRAL	HOSPITA L REGIONA	-	Enviada	Eventos Adversos Relaciona	Falhas na administra ção de		Enteral (oral, cateter
22/04/2019	CE	SOBRAL	HOSPITA L REGIONA	-	Enviada	Eventos Adversos Relaciona	Falhas na administra ção de		Enteral (oral, cateter
22/04/2019	CE	SOBRAL	HOSPITA L REGIONA	-	Enviada	Eventos Adversos Relaciona	Queda do paciente		
22/04/2019	CE	SOBRAL	HOSPITA L REGIONA	-	Enviada	Eventos Adversos Relaciona	Queda do paciente		
22/04/2019	CE	SOBRAL	HOSPITA L REGIONA	-	Enviada	Eventos Adversos Relaciona	Queda do paciente		
22/04/2019	CE	SOBRAL	HOSPITA L REGIONA	-	Enviada	Eventos Adversos Relaciona	Queda do paciente		
22/04/2019	CE	SOBRAL	HOSPITA L REGIONA	-	Enviada	Eventos Adversos Relaciona	Queda do paciente		
22/04/2019	CE	SOBRAL	HOSPITA L REGIONA	-	Enviada	Eventos Adversos Relaciona	Queda do paciente		
22/04/2019	CE	SOBRAL	HOSPITA L REGIONA	-	Enviada	Eventos Adversos Relaciona	Queda do paciente		
22/04/2019	CE	SOBRAL	HOSPITA L REGIONA	-	Enviada	Eventos Adversos Relaciona	Queda do paciente		
22/04/2019	CE	SOBRAL	HOSPITA L REGIONA	-	Enviada	Eventos Adversos Relaciona	Queda do paciente		
22/04/2019	CE	SOBRAL	HOSPITA L REGIONA	-	Enviada	Eventos Adversos Relaciona	Queda do paciente		
22/04/2019	CE	SOBRAL	HOSPITA L REGIONA	-	Enviada	Eventos Adversos Relaciona	Queda do paciente		
12/07/2019	CE	SOBRAL	HOSPITA L REGIONA	-	Enviada	Eventos Adversos Relaciona	Lesão por pressão		
12/07/2019	CE	SOBRAL	HOSPITA L REGIONA	-	Enviada	Eventos Adversos Relaciona	Lesão por pressão		
12/07/2019	CE	SOBRAL	HOSPITA L REGIONA	-	Enviada	Eventos Adversos Relaciona	Lesão por pressão		

12/07/2019	CE	SOBRAL	HOSPITAL REGIONA	-	Enviada	Eventos Adversos Relaciona	Lesão por pressão		
12/07/2019	CE	SOBRAL	HOSPITAL REGIONA	-	Enviada	Eventos Adversos Relaciona	Lesão por pressão		
12/07/2019	CE	SOBRAL	HOSPITAL REGIONA	-	Enviada	Eventos Adversos Relaciona	Lesão por pressão		
12/07/2019	CE	SOBRAL	HOSPITAL REGIONA	-	Enviada	Eventos Adversos Relaciona	Lesão por pressão		
12/07/2019	CE	SOBRAL	HOSPITAL REGIONA	-	Enviada	Eventos Adversos Relaciona	Lesão por pressão		
12/07/2019	CE	SOBRAL	HOSPITAL REGIONA	-	Enviada	Eventos Adversos Relaciona	Lesão por pressão		
12/07/2019	CE	SOBRAL	HOSPITAL REGIONA	-	Enviada	Eventos Adversos Relaciona	Lesão por pressão		
12/07/2019	CE	SOBRAL	HOSPITAL REGIONA	-	Enviada	Eventos Adversos Relaciona	Lesão por pressão		
12/07/2019	CE	SOBRAL	HOSPITAL REGIONA	-	Enviada	Eventos Adversos Relaciona	Lesão por pressão		
12/07/2019	CE	SOBRAL	HOSPITAL REGIONA	-	Enviada	Eventos Adversos Relaciona	Lesão por pressão		
12/07/2019	CE	SOBRAL	HOSPITAL REGIONA	-	Enviada	Eventos Adversos Relaciona	Lesão por pressão		
12/07/2019	CE	SOBRAL	HOSPITAL REGIONA	-	Enviada	Eventos Adversos Relaciona	Lesão por pressão		
12/07/2019	CE	SOBRAL	HOSPITAL REGIONA	-	Enviada	Eventos Adversos Relaciona	Lesão por pressão		
12/07/2019	CE	SOBRAL	HOSPITAL REGIONA	-	Enviada	Eventos Adversos Relaciona	Lesão por pressão		
12/07/2019	CE	SOBRAL	HOSPITAL REGIONA	-	Enviada	Eventos Adversos Relaciona	Lesão por pressão		
12/07/2019	CE	SOBRAL	HOSPITAL REGIONA	-	Enviada	Eventos Adversos Relaciona	Lesão por pressão		
12/07/2019	CE	SOBRAL	HOSPITAL REGIONA	-	Enviada	Eventos Adversos Relaciona	Lesão por pressão		
12/07/2019	CE	SOBRAL	HOSPITAL REGIONA	-	Enviada	Eventos Adversos Relaciona	Lesão por pressão		
12/07/2019	CE	SOBRAL	HOSPITAL REGIONA	-	Enviada	Eventos Adversos Relaciona	Lesão por pressão		

12/07/2019	CE	SOBRAL	HOSPITA L REGIONA	-	Enviada	Eventos Adversos Relaciona	Lesão por pressão		
12/07/2019	CE	SOBRAL	HOSPITA L REGIONA	-	Enviada	Eventos Adversos Relaciona	Lesão por pressão		
12/07/2019	CE	SOBRAL	HOSPITA L REGIONA	-	Enviada	Eventos Adversos Relaciona	Lesão por pressão		
12/07/2019	CE	SOBRAL	HOSPITA L REGIONA	-	Enviada	Eventos Adversos Relaciona	Lesão por pressão		
12/07/2019	CE	SOBRAL	HOSPITA L REGIONA	-	Enviada	Eventos Adversos Relaciona	Lesão por pressão		
12/07/2019	CE	SOBRAL	HOSPITA L REGIONA	-	Enviada	Eventos Adversos Relaciona	Lesão por pressão		
12/07/2019	CE	SOBRAL	HOSPITA L REGIONA	-	Enviada	Eventos Adversos Relaciona	Lesão por pressão		
12/07/2019	CE	SOBRAL	HOSPITA L REGIONA	-	Enviada	Eventos Adversos Relaciona	Lesão por pressão		
15/07/2019	CE	SOBRAL	HOSPITA L REGIONA	-	Enviada	Eventos Adversos Relaciona	Lesão por pressão		
15/07/2019	CE	SOBRAL	HOSPITA L REGIONA	-	Enviada	Eventos Adversos Relaciona	Lesão por pressão		
15/07/2019	CE	SOBRAL	HOSPITA L REGIONA	-	Enviada	Eventos Adversos Relaciona	Lesão por pressão		
15/07/2019	CE	SOBRAL	HOSPITA L REGIONA	-	Enviada	Eventos Adversos Relaciona	Lesão por pressão		
15/07/2019	CE	SOBRAL	HOSPITA L REGIONA	-	Enviada	Eventos Adversos Relaciona	Lesão por pressão		
15/07/2019	CE	SOBRAL	HOSPITA L REGIONA	-	Enviada	Eventos Adversos Relaciona	Lesão por pressão		
15/07/2019	CE	SOBRAL	HOSPITA L REGIONA	-	Enviada	Eventos Adversos Relaciona	Lesão por pressão		
15/07/2019	CE	SOBRAL	HOSPITA L REGIONA	-	Enviada	Eventos Adversos Relaciona	Lesão por pressão		
15/07/2019	CE	SOBRAL	HOSPITA L REGIONA	-	Enviada	Eventos Adversos Relaciona	Lesão por pressão		
15/07/2019	CE	SOBRAL	HOSPITA L REGIONA	-	Enviada	Eventos Adversos Relaciona	Lesão por pressão		
15/07/2019	CE	SOBRAL	HOSPITA L REGIONA	-	Enviada	Eventos Adversos Relaciona	Lesão por pressão		

15/07/2019	CE	SOBRAL	HOSPITA L REGIONA	-	Enviada	Eventos Adversos Relaciona	Lesão por pressão		
15/07/2019	CE	SOBRAL	HOSPITA L REGIONA	-	Enviada	Eventos Adversos Relaciona	Lesão por pressão		
15/07/2019	CE	SOBRAL	HOSPITA L REGIONA	-	Enviada	Eventos Adversos Relaciona	Lesão por pressão		
15/07/2019	CE	SOBRAL	HOSPITA L REGIONA	-	Enviada	Eventos Adversos Relaciona	Lesão por pressão		
15/07/2019	CE	SOBRAL	HOSPITA L REGIONA	-	Enviada	Eventos Adversos Relaciona	Lesão por pressão		
15/07/2019	CE	SOBRAL	HOSPITA L REGIONA	-	Enviada	Eventos Adversos Relaciona	Lesão por pressão		
15/07/2019	CE	SOBRAL	HOSPITA L REGIONA	-	Enviada	Eventos Adversos Relaciona	Lesão por pressão		
15/07/2019	CE	SOBRAL	HOSPITA L REGIONA	-	Enviada	Eventos Adversos Relaciona	Lesão por pressão		
15/07/2019	CE	SOBRAL	HOSPITA L REGIONA	-	Enviada	Eventos Adversos Relaciona	Lesão por pressão		
15/07/2019	CE	SOBRAL	HOSPITA L REGIONA	-	Enviada	Eventos Adversos Relaciona	Lesão por pressão		
15/07/2019	CE	SOBRAL	HOSPITA L REGIONA	-	Enviada	Eventos Adversos Relaciona	Lesão por pressão		
15/07/2019	CE	SOBRAL	HOSPITA L REGIONA	-	Enviada	Eventos Adversos Relaciona	Lesão por pressão		
15/07/2019	CE	SOBRAL	HOSPITA L REGIONA	-	Enviada	Eventos Adversos Relaciona	Lesão por pressão		
15/07/2019	CE	SOBRAL	HOSPITA L REGIONA	-	Enviada	Eventos Adversos Relaciona	Lesão por pressão		
15/07/2019	CE	SOBRAL	HOSPITA L REGIONA	-	Enviada	Eventos Adversos Relaciona	Lesão por pressão		
15/07/2019	CE	SOBRAL	HOSPITA L REGIONA	-	Enviada	Eventos Adversos Relaciona	Lesão por pressão		
15/07/2019	CE	SOBRAL	HOSPITA L REGIONA	-	Enviada	Eventos Adversos Relaciona	Lesão por pressão		
15/07/2019	CE	SOBRAL	HOSPITA L REGIONA	-	Enviada	Eventos Adversos Relaciona	Lesão por pressão		
15/07/2019	CE	SOBRAL	HOSPITA L REGIONA	-	Enviada	Eventos Adversos Relaciona	Lesão por pressão		

15/07/2019	CE	SOBRAL	HOSPITA L REGIONA	-	Enviada	Eventos Adversos Relaciona	Lesão por pressão		
15/07/2019	CE	SOBRAL	HOSPITA L REGIONA	-	Enviada	Eventos Adversos Relaciona	Lesão por pressão		
15/07/2019	CE	SOBRAL	HOSPITA L REGIONA	-	Enviada	Eventos Adversos Relaciona	Lesão por pressão		
15/07/2019	CE	SOBRAL	HOSPITA L REGIONA	-	Enviada	Eventos Adversos Relaciona	Lesão por pressão		
15/07/2019	CE	SOBRAL	HOSPITA L REGIONA	-	Enviada	Eventos Adversos Relaciona	Lesão por pressão		
15/07/2019	CE	SOBRAL	HOSPITA L REGIONA	-	Enviada	Eventos Adversos Relaciona	Lesão por pressão		
15/07/2019	CE	SOBRAL	HOSPITA L REGIONA	-	Enviada	Eventos Adversos Relaciona	Lesão por pressão		
15/07/2019	CE	SOBRAL	HOSPITA L REGIONA	-	Enviada	Eventos Adversos Relaciona	Lesão por pressão		
15/07/2019	CE	SOBRAL	HOSPITA L REGIONA	-	Enviada	Eventos Adversos Relaciona	Lesão por pressão		
15/07/2019	CE	SOBRAL	HOSPITA L REGIONA	-	Enviada	Eventos Adversos Relaciona	Lesão por pressão		
15/07/2019	CE	SOBRAL	HOSPITA L REGIONA	-	Enviada	Eventos Adversos Relaciona	Lesão por pressão		
15/07/2019	CE	SOBRAL	HOSPITA L REGIONA	-	Enviada	Eventos Adversos Relaciona	Lesão por pressão		
15/07/2019	CE	SOBRAL	HOSPITA L REGIONA	-	Enviada	Eventos Adversos Relaciona	Lesão por pressão		
15/07/2019	CE	SOBRAL	HOSPITA L REGIONA	-	Enviada	Eventos Adversos Relaciona	Lesão por pressão		
15/07/2019	CE	SOBRAL	HOSPITA L REGIONA	-	Enviada	Eventos Adversos Relaciona	Lesão por pressão		
15/07/2019	CE	SOBRAL	HOSPITA L REGIONA	-	Enviada	Eventos Adversos Relaciona	Lesão por pressão		
15/07/2019	CE	SOBRAL	HOSPITA L REGIONA	-	Enviada	Eventos Adversos Relaciona	Lesão por pressão		
15/07/2019	CE	SOBRAL	HOSPITA L REGIONA	-	Enviada	Eventos Adversos Relaciona	Lesão por pressão		
15/07/2019	CE	SOBRAL	HOSPITA L REGIONA	-	Enviada	Eventos Adversos Relaciona	Lesão por pressão		

15/07/2019	CE	SOBRAL	HOSPITA L REGIONA	-	Enviada	Eventos Adversos Relaciona	Lesão por pressão		
15/07/2019	CE	SOBRAL	HOSPITA L REGIONA	-	Enviada	Eventos Adversos Relaciona	Lesão por pressão		
15/07/2019	CE	SOBRAL	HOSPITA L REGIONA	-	Enviada	Eventos Adversos Relaciona	Lesão por pressão		
15/07/2019	CE	SOBRAL	HOSPITA L REGIONA	-	Enviada	Eventos Adversos Relaciona	Lesão por pressão		
15/07/2019	CE	SOBRAL	HOSPITA L REGIONA	-	Enviada	Eventos Adversos Relaciona	Lesão por pressão		
01/08/2019	CE	SOBRAL	HOSPITA L REGIONA	-	Enviada	Eventos Adversos Relaciona	Lesão por pressão		
05/08/2019	CE	SOBRAL	HOSPITA L REGIONA	-	Enviada	Eventos Adversos Relaciona	Lesão por pressão		
05/08/2019	CE	SOBRAL	HOSPITA L REGIONA	-	Enviada	Eventos Adversos Relaciona	Lesão por pressão		
05/08/2019	CE	SOBRAL	HOSPITA L REGIONA	-	Enviada	Eventos Adversos Relaciona	Lesão por pressão		
05/08/2019	CE	SOBRAL	HOSPITA L REGIONA	-	Enviada	Eventos Adversos Relaciona	Lesão por pressão		
05/08/2019	CE	SOBRAL	HOSPITA L REGIONA	-	Enviada	Eventos Adversos Relaciona	Lesão por pressão		
05/08/2019	CE	SOBRAL	HOSPITA L REGIONA	-	Enviada	Eventos Adversos Relaciona	Lesão por pressão		
05/08/2019	CE	SOBRAL	HOSPITA L REGIONA	-	Enviada	Eventos Adversos Relaciona	Lesão por pressão		
05/08/2019	CE	SOBRAL	HOSPITA L REGIONA	-	Enviada	Eventos Adversos Relaciona	Lesão por pressão		
05/08/2019	CE	SOBRAL	HOSPITA L REGIONA	-	Enviada	Eventos Adversos Relaciona	Lesão por pressão		
05/08/2019	CE	SOBRAL	HOSPITA L REGIONA	-	Enviada	Eventos Adversos Relaciona	Lesão por pressão		
05/08/2019	CE	SOBRAL	HOSPITA L REGIONA	-	Enviada	Eventos Adversos Relaciona	Lesão por pressão		
05/08/2019	CE	SOBRAL	HOSPITA L REGIONA	-	Enviada	Eventos Adversos Relaciona	Lesão por pressão		
05/08/2019	CE	SOBRAL	HOSPITA L REGIONA	-	Enviada	Eventos Adversos Relaciona	Lesão por pressão		

05/08/2019	CE	SOBRAL	HOSPITA L REGIONA	-	Enviada	Eventos Adversos Relaciona	Lesão por pressão		
05/08/2019	CE	SOBRAL	HOSPITA L REGIONA	-	Enviada	Eventos Adversos Relaciona	Lesão por pressão		
05/08/2019	CE	SOBRAL	HOSPITA L REGIONA	-	Enviada	Eventos Adversos Relaciona	Lesão por pressão		
05/08/2019	CE	SOBRAL	HOSPITA L REGIONA	-	Enviada	Eventos Adversos Relaciona	Lesão por pressão		
05/08/2019	CE	SOBRAL	HOSPITA L REGIONA	-	Enviada	Eventos Adversos Relaciona	Lesão por pressão		
05/08/2019	CE	SOBRAL	HOSPITA L REGIONA	-	Enviada	Eventos Adversos Relaciona	Lesão por pressão		
05/08/2019	CE	SOBRAL	HOSPITA L REGIONA	-	Enviada	Eventos Adversos Relaciona	Lesão por pressão		
05/08/2019	CE	SOBRAL	HOSPITA L REGIONA	FLEBITE	Enviada	Eventos Adversos Relaciona	Falhas durante a assistênci		
05/08/2019	CE	SOBRAL	HOSPITA L REGIONA	FLEBITE	Enviada	Eventos Adversos Relaciona	Falhas durante a assistênci		
05/08/2019	CE	SOBRAL	HOSPITA L REGIONA	FLEBITE	Enviada	Eventos Adversos Relaciona	Falhas durante a assistênci		
05/08/2019	CE	SOBRAL	HOSPITA L REGIONA	FLEBITE	Enviada	Eventos Adversos Relaciona	Falhas durante a assistênci		
05/08/2019	CE	SOBRAL	HOSPITA L REGIONA	FLEBITE	Enviada	Eventos Adversos Relaciona	Falhas durante a assistênci		
05/08/2019	CE	SOBRAL	HOSPITA L REGIONA	FLEBITE	Enviada	Eventos Adversos Relaciona	Falhas durante a assistênci		
05/08/2019	CE	SOBRAL	HOSPITA L REGIONA	FLEBITE	Enviada	Eventos Adversos Relaciona	Falhas durante a assistênci		
05/08/2019	CE	SOBRAL	HOSPITA L REGIONA	FLEBITE	Enviada	Eventos Adversos Relaciona	Falhas durante a assistênci		
05/08/2019	CE	SOBRAL	HOSPITA L REGIONA	FLEBITE	Enviada	Eventos Adversos Relaciona	Falhas durante a assistênci		
05/08/2019	CE	SOBRAL	HOSPITA L REGIONA	FLEBITE	Enviada	Eventos Adversos Relaciona	Falhas durante a assistênci		
05/08/2019	CE	SOBRAL	HOSPITA L REGIONA	FLEBITE	Enviada	Eventos Adversos Relaciona	Falhas durante a assistênci		
05/08/2019	CE	SOBRAL	HOSPITA L REGIONA	DIETA RELACIO NADA A	Enviada	Eventos Adversos Relaciona	Falhas na administra ção de		Enteral (oral, cateter

05/08/2019	CE	SOBRAL	HOSPITAL REGIONA	Dispositivo em uso fora do	Enviada	Eventos Adversos Relaciona	Falhas durante a assistênci		
05/08/2019	CE	SOBRAL	HOSPITAL REGIONA	Dispositivo em uso fora do	Enviada	Eventos Adversos Relaciona	Falhas durante a assistênci		
05/08/2019	CE	SOBRAL	HOSPITAL REGIONA	Dispositivo em uso fora do	Enviada	Eventos Adversos Relaciona	Falhas durante a assistênci		
05/08/2019	CE	SOBRAL	HOSPITAL REGIONA	-	Enviada	Eventos Adversos Relaciona	Falhas durante a assistênci		
05/08/2019	CE	SOBRAL	HOSPITAL REGIONA	Perda de acesso venoso	Enviada	Eventos Adversos Relaciona	Falhas durante a assistênci		
05/08/2019	CE	SOBRAL	HOSPITAL REGIONA	Não entregue como	Enviada	Eventos Adversos Relaciona	Falhas na administração de		Enteral (oral, cateter
05/08/2019	CE	SOBRAL	HOSPITAL REGIONA	Não entregue como	Enviada	Eventos Adversos Relaciona	Falhas na administração de		Enteral (oral, cateter
05/08/2019	CE	SOBRAL	HOSPITAL REGIONA	Fora do prazo após	Enviada	Eventos Adversos Relaciona	Falhas na administração de		Enteral (oral, cateter
16/08/2019	CE	SOBRAL	HOSPITAL REGIONA	Perda de acesso venoso	Enviada	Eventos Adversos Relaciona	Falhas durante a assistênci		
16/08/2019	CE	SOBRAL	HOSPITAL REGIONA	Perda de acesso venoso	Enviada	Eventos Adversos Relaciona	Falhas durante a assistênci		
16/08/2019	CE	SOBRAL	HOSPITAL REGIONA	Perda de acesso venoso	Enviada	Eventos Adversos Relaciona	Falhas durante a assistênci		
16/08/2019	CE	SOBRAL	HOSPITAL REGIONA	Perda de acesso venoso	Enviada	Eventos Adversos Relaciona	Falhas durante a assistênci		
16/08/2019	CE	SOBRAL	HOSPITAL REGIONA	Perda de acesso venoso	Enviada	Eventos Adversos Relaciona	Falhas durante a assistênci		
16/08/2019	CE	SOBRAL	HOSPITAL REGIONA	Perda de acesso venoso	Enviada	Eventos Adversos Relaciona	Falhas durante a assistênci		
16/08/2019	CE	SOBRAL	HOSPITAL REGIONA	Perda de acesso venoso	Enviada	Eventos Adversos Relaciona	Falhas durante a assistênci		
16/08/2019	CE	SOBRAL	HOSPITAL REGIONA	Perda de acesso venoso	Enviada	Eventos Adversos Relaciona	Falhas durante a assistênci		
16/08/2019	CE	SOBRAL	HOSPITAL REGIONA	Perda de acesso venoso	Enviada	Eventos Adversos Relaciona	Falhas durante a assistênci		
16/08/2019	CE	SOBRAL	HOSPITAL REGIONA	Perda de acesso venoso	Enviada	Eventos Adversos Relaciona	Falhas durante a assistênci		
16/08/2019	CE	SOBRAL	HOSPITAL REGIONA	Perda de acesso venoso	Enviada	Eventos Adversos Relaciona	Falhas durante a assistênci		

16/08/2019	CE	SOBRAL	HOSPITA L REGIONA	Perda de acesso venoso	Enviada	Eventos Adversos Relaciona	Falhas durante a assistênci		
16/08/2019	CE	SOBRAL	HOSPITA L REGIONA	Perda de acesso venoso	Enviada	Eventos Adversos Relaciona	Falhas durante a assistênci		
16/08/2019	CE	SOBRAL	HOSPITA L REGIONA	Perda de acesso venoso	Enviada	Eventos Adversos Relaciona	Falhas durante a assistênci		
16/08/2019	CE	SOBRAL	HOSPITA L REGIONA	Perda de acesso venoso	Enviada	Eventos Adversos Relaciona	Falhas durante a assistênci		
16/08/2019	CE	SOBRAL	HOSPITA L REGIONA	Perda de acesso venoso	Enviada	Eventos Adversos Relaciona	Falhas durante a assistênci		
16/08/2019	CE	SOBRAL	HOSPITA L REGIONA	Perda de acesso central	Enviada	Eventos Adversos Relaciona	Falhas durante a assistênci		
16/08/2019	CE	SOBRAL	HOSPITA L REGIONA	Perda de acesso central	Enviada	Eventos Adversos Relaciona	Falhas durante a assistênci		
16/08/2019	CE	SOBRAL	HOSPITA L REGIONA	Perda de acesso central	Enviada	Eventos Adversos Relaciona	Falhas durante a assistênci		
16/08/2019	CE	SOBRAL	HOSPITA L REGIONA	-	Enviada	Eventos Adversos Relaciona	Falhas durante a assistênci		
16/08/2019	CE	SOBRAL	HOSPITA L REGIONA	Perda de acesso central	Enviada	Eventos Adversos Relaciona	Falhas durante a assistênci		
16/08/2019	CE	SOBRAL	HOSPITA L REGIONA	Perda de acesso central	Enviada	Eventos Adversos Relaciona	Falhas durante a assistênci		
16/08/2019	CE	SOBRAL	HOSPITA L REGIONA	Preparo inadequad o do	Enviada	Eventos Adversos Relaciona	Falhas durante a assistênci		
17/08/2019	CE	SOBRAL	HOSPITA L REGIONA	Preparo inadequad o do	Enviada	Eventos Adversos Relaciona	Falhas durante a assistênci		
17/08/2019	CE	SOBRAL	HOSPITA L REGIONA	Preparo inadequad o do	Enviada	Eventos Adversos Relaciona	Falhas durante a assistênci		
17/08/2019	CE	SOBRAL	HOSPITA L REGIONA	-	Enviada	Eventos Adversos Relaciona	Falhas durante a assistênci		
17/08/2019	CE	SOBRAL	HOSPITA L REGIONA	-	Enviada	Eventos Adversos Relaciona	Falhas durante a assistênci		
17/08/2019	CE	SOBRAL	HOSPITA L REGIONA	Preparo inadequad o do	Enviada	Eventos Adversos Relaciona	Falhas durante a assistênci		
17/08/2019	CE	SOBRAL	HOSPITA L REGIONA	Preparo inadequad o do	Enviada	Eventos Adversos Relaciona	Falhas durante a assistênci		
17/08/2019	CE	SOBRAL	HOSPITA L REGIONA	Preparo inadequad o do	Enviada	Eventos Adversos Relaciona	Falhas durante a assistênci		

17/08/2019	CE	SOBRAL	HOSPITA L REGIONA	Preparo inadequad o do	Enviada	Eventos Adversos Relaciona	Falhas durante a assistênci		
17/08/2019	CE	SOBRAL	HOSPITA L REGIONA	Preparo inadequad o do	Enviada	Eventos Adversos Relaciona	Falhas durante a assistênci		
17/08/2019	CE	SOBRAL	HOSPITA L REGIONA	Não entregue.	Enviada	Eventos Adversos Relaciona	Falhas na administra ção de		Enteral (oral, cateter
17/08/2019	CE	SOBRAL	HOSPITA L REGIONA	-	Enviada	Eventos Adversos Relaciona	Falhas na administra ção de		Enteral (oral, cateter
17/08/2019	CE	SOBRAL	HOSPITA L REGIONA	Não foi entregue	Enviada	Eventos Adversos Relaciona	Falhas na administra ção de		Enteral (oral, cateter
17/08/2019	CE	SOBRAL	HOSPITA L REGIONA	Não feito solicitaçã o dos	Enviada	Eventos Adversos Relaciona	Falhas durante a assistênci		
17/08/2019	CE	SOBRAL	HOSPITA L REGIONA	Solicitaçã o de exames	Enviada	Eventos Adversos Relaciona	Falhas durante a assistênci		
17/08/2019	CE	SOBRAL	HOSPITA L REGIONA	Suspensã o de cirurgia	Enviada	Eventos Adversos Relaciona	Falhas durante a assistênci		
17/08/2019	CE	SOBRAL	HOSPITA L REGIONA	Suspensã o de cirurgia	Enviada	Eventos Adversos Relaciona	Falhas durante a assistênci		
17/08/2019	CE	SOBRAL	HOSPITA L REGIONA	Solicitaçã o de exame	Enviada	Eventos Adversos Relaciona	Falhas durante a assistênci		
17/08/2019	CE	SOBRAL	HOSPITA L REGIONA	Não realização	Enviada	Eventos Adversos Relaciona	Falhas durante a assistênci		
17/08/2019	CE	SOBRAL	HOSPITA L REGIONA	Não foi entregue	Enviada	Eventos Adversos Relaciona	Falhas na administra ção de		Enteral (oral, cateter
17/08/2019	CE	SOBRAL	HOSPITA L REGIONA	Adiament o de cirurgia	Enviada	Eventos Adversos Relaciona	Falhas durante a assistênci		
17/08/2019	CE	SOBRAL	HOSPITA L REGIONA	Dieta incomplet a de	Enviada	Eventos Adversos Relaciona	Falhas na administra ção de		Enteral (oral, cateter
17/08/2019	CE	SOBRAL	HOSPITA L REGIONA	-	Enviada	Eventos Adversos Relaciona	Falhas na administra ção de		Enteral (oral, cateter
17/08/2019	CE	SOBRAL	HOSPITA L REGIONA	Dieta entregue atrasada	Enviada	Eventos Adversos Relaciona	Falhas na administra ção de		Enteral (oral, cateter
17/08/2019	CE	SOBRAL	HOSPITA L REGIONA	Não entregue	Enviada	Eventos Adversos Relaciona	Falhas na administra ção de		Enteral (oral, cateter
17/08/2019	CE	SOBRAL	HOSPITA L REGIONA	Não foi entregue	Enviada	Eventos Adversos Relaciona	Falhas na administra ção de		Enteral (oral, cateter
17/08/2019	CE	SOBRAL	HOSPITA L REGIONA	Não entregue	Enviada	Eventos Adversos Relaciona	Falhas na administra ção de		Enteral (oral, cateter

17/08/2019	CE	SOBRAL	HOSPITA L REGIONA	Dieta admnistrada depois	Enviada	Eventos Adversos Relaciona	Falhas na administração de		Enteral (oral, cateter
17/08/2019	CE	SOBRAL	HOSPITA L REGIONA	Não entregue	Enviada	Eventos Adversos Relaciona	Falhas na administração de		Enteral (oral, cateter
17/08/2019	CE	SOBRAL	HOSPITA L REGIONA	Não entregue	Enviada	Eventos Adversos Relaciona	Falhas na administração de		Enteral (oral, cateter
17/08/2019	CE	SOBRAL	HOSPITA L REGIONA	-	Enviada	Eventos Adversos Relaciona	Falhas na administração de		Enteral (oral, cateter
17/08/2019	CE	SOBRAL	HOSPITA L REGIONA	-	Enviada	Eventos Adversos Relaciona	Falhas na administração de		Enteral (oral, cateter
17/08/2019	CE	SOBRAL	HOSPITA L REGIONA	-	Enviada	Eventos Adversos Relaciona	Queda do paciente		
17/08/2019	CE	SOBRAL	HOSPITA L REGIONA	-	Enviada	Eventos Adversos Relaciona	Queda do paciente		
17/08/2019	CE	SOBRAL	HOSPITA L REGIONA	-	Enviada	Eventos Adversos Relaciona	Queda do paciente		
17/08/2019	CE	SOBRAL	HOSPITA L REGIONA	-	Enviada	Eventos Adversos Relaciona	Queda do paciente		
17/08/2019	CE	SOBRAL	HOSPITA L REGIONA	-	Enviada	Eventos Adversos Relaciona	Queda do paciente		
17/08/2019	CE	SOBRAL	HOSPITA L REGIONA	-	Enviada	Eventos Adversos Relaciona	Queda do paciente		
17/08/2019	CE	SOBRAL	HOSPITA L REGIONA	-	Enviada	Eventos Adversos Relaciona	Queda do paciente		
17/08/2019	CE	SOBRAL	HOSPITA L REGIONA	-	Enviada	Eventos Adversos Relaciona	Queda do paciente		
17/08/2019	CE	SOBRAL	HOSPITA L REGIONA	-	Enviada	Eventos Adversos Relaciona	Queda do paciente		
17/08/2019	CE	SOBRAL	HOSPITA L REGIONA	-	Enviada	Eventos Adversos Relaciona	Queda do paciente		
17/08/2019	CE	SOBRAL	HOSPITA L REGIONA	-	Enviada	Eventos Adversos Relaciona	Queda do paciente		
17/08/2019	CE	SOBRAL	HOSPITA L REGIONA	-	Enviada	Eventos Adversos Relaciona	Queda do paciente		
17/08/2019	CE	SOBRAL	HOSPITA L REGIONA	-	Enviada	Eventos Adversos Relaciona	Queda do paciente		
17/08/2019	CE	SOBRAL	HOSPITA L REGIONA	Queimadura no pé por	Enviada	Eventos Adversos Relaciona	Queimadura de paciente		

19/08/2019	CE	SOBRAL	HOSPITA L REGIONA	-	Enviada	Eventos Adversos Relaciona	Queda do paciente		
19/08/2019	CE	SOBRAL	HOSPITA L REGIONA	Sofreu queda do colo da	Enviada	Eventos Adversos Relaciona	Queda do paciente		
19/08/2019	CE	SOBRAL	HOSPITA L REGIONA	-	Enviada	Eventos Adversos Relaciona	Queda do paciente		
19/08/2019	CE	SOBRAL	HOSPITA L REGIONA	Queimadu ra relacionad	Enviada	Eventos Adversos Relaciona	Queimadu ra de paciente		
19/08/2019	CE	SOBRAL	HOSPITA L REGIONA	velocidad e de infusão	Enviada	Eventos Adversos Relaciona	Falhas na administra ção de		Enteral (oral, cateter
19/08/2019	CE	SOBRAL	HOSPITA L REGIONA	-	Enviada	Eventos Adversos Relaciona	Falhas na administra ção de		Enteral (oral, cateter
19/08/2019	CE	SOBRAL	HOSPITA L REGIONA	DIETA EM BIC ADMINIS	Enviada	Eventos Adversos Relaciona	Falhas na administra ção de		Parenteral (via intravenos
19/08/2019	CE	SOBRAL	HOSPITA L REGIONA	dieta administra da em	Enviada	Eventos Adversos Relaciona	Falhas na administra ção de		Enteral (oral, cateter
19/08/2019	CE	SOBRAL	HOSPITA L REGIONA	-	Enviada	Eventos Adversos Relaciona	Falhas na administra ção de		Enteral (oral, cateter
19/08/2019	CE	SOBRAL	HOSPITA L REGIONA	-	Enviada	Eventos Adversos Relaciona	Falhas na administra ção de		Parenteral (via intravenos
19/08/2019	CE	SOBRAL	HOSPITA L REGIONA	-	Enviada	Eventos Adversos Relaciona	Falhas na administra ção de		Parenteral (via intravenos
19/08/2019	CE	SOBRAL	HOSPITA L REGIONA	-	Enviada	Eventos Adversos Relaciona	Falhas na administra ção de		Parenteral (via intravenos
19/08/2019	CE	SOBRAL	HOSPITA L REGIONA	dispositiv o em uso fora do	Enviada	Eventos Adversos Relaciona	Falhas durante a assistênci		
19/08/2019	CE	SOBRAL	HOSPITA L REGIONA	dispositiv o em uso fora do	Enviada	Eventos Adversos Relaciona	Falhas durante a assistênci		
19/08/2019	CE	SOBRAL	HOSPITA L REGIONA	dispositiv o em uso fora do	Enviada	Eventos Adversos Relaciona	Falhas durante a assistênci		
19/08/2019	CE	SOBRAL	HOSPITA L REGIONA	saída não planejada de dreno	Enviada	Eventos Adversos Relaciona	Falhas durante a assistênci		
19/08/2019	CE	SOBRAL	HOSPITA L REGIONA	FRASCO DE EXAME	Enviada	Eventos Adversos Relaciona	Falha na identificaç ão do		
19/08/2019	CE	SOBRAL	HOSPITA L REGIONA	-	Enviada	Eventos Adversos Relaciona	Falhas ocorridas em		
19/08/2019	CE	SOBRAL	HOSPITA L REGIONA	-	Enviada	Eventos Adversos Relaciona	Falhas na administra ção de		Enteral (oral, cateter

19/08/2019	CE	SOBRAL	HOSPITAL REGIONA	-	Enviada	Eventos Adversos Relaciona	Falhas durante a assistênci		
10/03/2020	CE	SOBRAL	HOSPITAL REGIONA	-	Enviada	Eventos Adversos Relaciona	Lesão por pressão		
10/03/2020	CE	SOBRAL	HOSPITAL REGIONA	-	Enviada	Eventos Adversos Relaciona	Lesão por pressão		
10/03/2020	CE	SOBRAL	HOSPITAL REGIONA	-	Enviada	Eventos Adversos Relaciona	Lesão por pressão		
10/03/2020	CE	SOBRAL	HOSPITAL REGIONA	-	Enviada	Eventos Adversos Relaciona	Lesão por pressão		
10/03/2020	CE	SOBRAL	HOSPITAL REGIONA	-	Enviada	Eventos Adversos Relaciona	Lesão por pressão		
10/03/2020	CE	SOBRAL	HOSPITAL REGIONA	-	Enviada	Eventos Adversos Relaciona	Lesão por pressão		
10/03/2020	CE	SOBRAL	HOSPITAL REGIONA	-	Enviada	Eventos Adversos Relaciona	Lesão por pressão		
10/03/2020	CE	SOBRAL	HOSPITAL REGIONA	-	Enviada	Eventos Adversos Relaciona	Lesão por pressão		
10/03/2020	CE	SOBRAL	HOSPITAL REGIONA	-	Enviada	Eventos Adversos Relaciona	Lesão por pressão		
10/03/2020	CE	SOBRAL	HOSPITAL REGIONA	-	Enviada	Eventos Adversos Relaciona	Lesão por pressão		
10/03/2020	CE	SOBRAL	HOSPITAL REGIONA	-	Enviada	Eventos Adversos Relaciona	Lesão por pressão		
10/03/2020	CE	SOBRAL	HOSPITAL REGIONA	-	Enviada	Eventos Adversos Relaciona	Lesão por pressão		
10/03/2020	CE	SOBRAL	HOSPITAL REGIONA	-	Enviada	Eventos Adversos Relaciona	Lesão por pressão		
10/03/2020	CE	SOBRAL	HOSPITAL REGIONA	-	Enviada	Eventos Adversos Relaciona	Lesão por pressão		
11/03/2020	CE	SOBRAL	HOSPITAL REGIONA	-	Enviada	Eventos Adversos Relaciona	Lesão por pressão		
11/03/2020	CE	SOBRAL	HOSPITAL REGIONA	-	Enviada	Eventos Adversos Relaciona	Lesão por pressão		
11/03/2020	CE	SOBRAL	HOSPITAL REGIONA	-	Enviada	Eventos Adversos Relaciona	Lesão por pressão		
11/03/2020	CE	SOBRAL	HOSPITAL REGIONA	-	Enviada	Eventos Adversos Relaciona	Lesão por pressão		

11/03/2020	CE	SOBRAL	HOSPITAL REGIONA	-	Enviada	Eventos Adversos Relaciona	Lesão por pressão		
11/03/2020	CE	SOBRAL	HOSPITAL REGIONA	-	Enviada	Eventos Adversos Relaciona	Lesão por pressão		
11/03/2020	CE	SOBRAL	HOSPITAL REGIONA	-	Enviada	Eventos Adversos Relaciona	Lesão por pressão		
11/03/2020	CE	SOBRAL	HOSPITAL REGIONA	-	Enviada	Eventos Adversos Relaciona	Lesão por pressão		
11/03/2020	CE	SOBRAL	HOSPITAL REGIONA	-	Enviada	Eventos Adversos Relaciona	Lesão por pressão		
11/03/2020	CE	SOBRAL	HOSPITAL REGIONA	-	Enviada	Eventos Adversos Relaciona	Lesão por pressão		
11/03/2020	CE	SOBRAL	HOSPITAL REGIONA	-	Enviada	Eventos Adversos Relaciona	Lesão por pressão		
11/03/2020	CE	SOBRAL	HOSPITAL REGIONA	-	Enviada	Eventos Adversos Relaciona	Lesão por pressão		
11/03/2020	CE	SOBRAL	HOSPITAL REGIONA	-	Enviada	Eventos Adversos Relaciona	Lesão por pressão		
11/03/2020	CE	SOBRAL	HOSPITAL REGIONA	-	Enviada	Eventos Adversos Relaciona	Lesão por pressão		
11/03/2020	CE	SOBRAL	HOSPITAL REGIONA	-	Enviada	Eventos Adversos Relaciona	Lesão por pressão		
11/03/2020	CE	SOBRAL	HOSPITAL REGIONA	-	Enviada	Eventos Adversos Relaciona	Lesão por pressão		
11/03/2020	CE	SOBRAL	HOSPITAL REGIONA	-	Enviada	Eventos Adversos Relaciona	Lesão por pressão		
11/03/2020	CE	SOBRAL	HOSPITAL REGIONA	-	Enviada	Eventos Adversos Relaciona	Lesão por pressão		
11/03/2020	CE	SOBRAL	HOSPITAL REGIONA	-	Enviada	Eventos Adversos Relaciona	Lesão por pressão		
11/03/2020	CE	SOBRAL	HOSPITAL REGIONA	-	Enviada	Eventos Adversos Relaciona	Lesão por pressão		
11/03/2020	CE	SOBRAL	HOSPITAL REGIONA	-	Enviada	Eventos Adversos Relaciona	Lesão por pressão		
11/03/2020	CE	SOBRAL	HOSPITAL REGIONA	-	Enviada	Eventos Adversos Relaciona	Lesão por pressão		
11/03/2020	CE	SOBRAL	HOSPITAL REGIONA	-	Enviada	Eventos Adversos Relaciona	Lesão por pressão		

11/03/2020	CE	SOBRAL	HOSPITA L REGIONA	-	Enviada	Eventos Adversos Relaciona	Lesão por pressão		
11/03/2020	CE	SOBRAL	HOSPITA L REGIONA	-	Enviada	Eventos Adversos Relaciona	Lesão por pressão		
11/03/2020	CE	SOBRAL	HOSPITA L REGIONA	-	Enviada	Eventos Adversos Relaciona	Lesão por pressão		
11/03/2020	CE	SOBRAL	HOSPITA L REGIONA	-	Enviada	Eventos Adversos Relaciona	Lesão por pressão		
11/03/2020	CE	SOBRAL	HOSPITA L REGIONA	-	Enviada	Eventos Adversos Relaciona	Lesão por pressão		
11/03/2020	CE	SOBRAL	HOSPITA L REGIONA	-	Enviada	Eventos Adversos Relaciona	Lesão por pressão		
11/03/2020	CE	SOBRAL	HOSPITA L REGIONA	-	Enviada	Eventos Adversos Relaciona	Lesão por pressão		
11/03/2020	CE	SOBRAL	HOSPITA L REGIONA	-	Enviada	Eventos Adversos Relaciona	Lesão por pressão		
11/03/2020	CE	SOBRAL	HOSPITA L REGIONA	-	Enviada	Eventos Adversos Relaciona	Lesão por pressão		
11/03/2020	CE	SOBRAL	HOSPITA L REGIONA	-	Enviada	Eventos Adversos Relaciona	Lesão por pressão		
11/03/2020	CE	SOBRAL	HOSPITA L REGIONA	-	Enviada	Eventos Adversos Relaciona	Lesão por pressão		
11/03/2020	CE	SOBRAL	HOSPITA L REGIONA	-	Enviada	Eventos Adversos Relaciona	Lesão por pressão		
11/03/2020	CE	SOBRAL	HOSPITA L REGIONA	-	Enviada	Eventos Adversos Relaciona	Lesão por pressão		
11/03/2020	CE	SOBRAL	HOSPITA L REGIONA	-	Enviada	Eventos Adversos Relaciona	Lesão por pressão		
11/03/2020	CE	SOBRAL	HOSPITA L REGIONA	-	Enviada	Eventos Adversos Relaciona	Lesão por pressão		
11/03/2020	CE	SOBRAL	HOSPITA L REGIONA	-	Enviada	Eventos Adversos Relaciona	Lesão por pressão		
11/03/2020	CE	SOBRAL	HOSPITA L REGIONA	-	Enviada	Eventos Adversos Relaciona	Lesão por pressão		
11/03/2020	CE	SOBRAL	HOSPITA L REGIONA	-	Enviada	Eventos Adversos Relaciona	Lesão por pressão		
11/03/2020	CE	SOBRAL	HOSPITA L REGIONA	-	Enviada	Eventos Adversos Relaciona	Lesão por pressão		

11/03/2020	CE	SOBRAL	HOSPITA L REGIONA	-	Enviada	Eventos Adversos Relaciona	Lesão por pressão		
11/03/2020	CE	SOBRAL	HOSPITA L REGIONA	-	Enviada	Eventos Adversos Relaciona	Lesão por pressão		
11/03/2020	CE	SOBRAL	HOSPITA L REGIONA	-	Enviada	Eventos Adversos Relaciona	Lesão por pressão		
11/03/2020	CE	SOBRAL	HOSPITA L REGIONA	-	Enviada	Eventos Adversos Relaciona	Lesão por pressão		
11/03/2020	CE	SOBRAL	HOSPITA L REGIONA	-	Enviada	Eventos Adversos Relaciona	Lesão por pressão		
11/03/2020	CE	SOBRAL	HOSPITA L REGIONA	-	Enviada	Eventos Adversos Relaciona	Lesão por pressão		
11/03/2020	CE	SOBRAL	HOSPITA L REGIONA	-	Enviada	Eventos Adversos Relaciona	Lesão por pressão		
11/03/2020	CE	SOBRAL	HOSPITA L REGIONA	-	Enviada	Eventos Adversos Relaciona	Lesão por pressão		
11/03/2020	CE	SOBRAL	HOSPITA L REGIONA	-	Enviada	Eventos Adversos Relaciona	Lesão por pressão		
11/03/2020	CE	SOBRAL	HOSPITA L REGIONA	-	Enviada	Eventos Adversos Relaciona	Lesão por pressão		
11/03/2020	CE	SOBRAL	HOSPITA L REGIONA	-	Enviada	Eventos Adversos Relaciona	Lesão por pressão		
11/03/2020	CE	SOBRAL	HOSPITA L REGIONA	-	Enviada	Eventos Adversos Relaciona	Lesão por pressão		
11/03/2020	CE	SOBRAL	HOSPITA L REGIONA	-	Enviada	Eventos Adversos Relaciona	Lesão por pressão		
11/03/2020	CE	SOBRAL	HOSPITA L REGIONA	-	Enviada	Eventos Adversos Relaciona	Lesão por pressão		
11/03/2020	CE	SOBRAL	HOSPITA L REGIONA	-	Enviada	Eventos Adversos Relaciona	Lesão por pressão		
11/03/2020	CE	SOBRAL	HOSPITA L REGIONA	-	Enviada	Eventos Adversos Relaciona	Lesão por pressão		
11/03/2020	CE	SOBRAL	HOSPITA L REGIONA	-	Enviada	Eventos Adversos Relaciona	Lesão por pressão		
11/03/2020	CE	SOBRAL	HOSPITA L REGIONA	-	Enviada	Eventos Adversos Relaciona	Lesão por pressão		
11/03/2020	CE	SOBRAL	HOSPITA L REGIONA	-	Enviada	Eventos Adversos Relaciona	Lesão por pressão		

11/03/2020	CE	SOBRAL	HOSPITA L REGIONA	-	Enviada	Eventos Adversos Relaciona	Lesão por pressão		
11/03/2020	CE	SOBRAL	HOSPITA L REGIONA	-	Enviada	Eventos Adversos Relaciona	Lesão por pressão		
11/03/2020	CE	SOBRAL	HOSPITA L REGIONA	-	Enviada	Eventos Adversos Relaciona	Lesão por pressão		
11/03/2020	CE	SOBRAL	HOSPITA L REGIONA	-	Enviada	Eventos Adversos Relaciona	Lesão por pressão		
11/03/2020	CE	SOBRAL	HOSPITA L REGIONA	-	Enviada	Eventos Adversos Relaciona	Lesão por pressão		
11/03/2020	CE	SOBRAL	HOSPITA L REGIONA	-	Enviada	Eventos Adversos Relaciona	Lesão por pressão		
11/03/2020	CE	SOBRAL	HOSPITA L REGIONA	-	Enviada	Eventos Adversos Relaciona	Lesão por pressão		
11/03/2020	CE	SOBRAL	HOSPITA L REGIONA	-	Enviada	Eventos Adversos Relaciona	Lesão por pressão		
11/03/2020	CE	SOBRAL	HOSPITA L REGIONA	-	Enviada	Eventos Adversos Relaciona	Lesão por pressão		
11/03/2020	CE	SOBRAL	HOSPITA L REGIONA	-	Enviada	Eventos Adversos Relaciona	Lesão por pressão		
11/03/2020	CE	SOBRAL	HOSPITA L REGIONA	-	Enviada	Eventos Adversos Relaciona	Lesão por pressão		
11/03/2020	CE	SOBRAL	HOSPITA L REGIONA	-	Enviada	Eventos Adversos Relaciona	Lesão por pressão		
11/03/2020	CE	SOBRAL	HOSPITA L REGIONA	-	Enviada	Eventos Adversos Relaciona	Lesão por pressão		
11/03/2020	CE	SOBRAL	HOSPITA L REGIONA	-	Enviada	Eventos Adversos Relaciona	Lesão por pressão		
11/03/2020	CE	SOBRAL	HOSPITA L REGIONA	-	Enviada	Eventos Adversos Relaciona	Lesão por pressão		
11/03/2020	CE	SOBRAL	HOSPITA L REGIONA	-	Enviada	Eventos Adversos Relaciona	Lesão por pressão		
11/03/2020	CE	SOBRAL	HOSPITA L REGIONA	-	Enviada	Eventos Adversos Relaciona	Lesão por pressão		
11/03/2020	CE	SOBRAL	HOSPITA L REGIONA	-	Enviada	Eventos Adversos Relaciona	Lesão por pressão		
11/03/2020	CE	SOBRAL	HOSPITA L REGIONA	-	Enviada	Eventos Adversos Relaciona	Lesão por pressão		

11/03/2020	CE	SOBRAL	HOSPITA L REGIONA	-	Enviada	Eventos Adversos Relaciona	Lesão por pressão		
11/03/2020	CE	SOBRAL	HOSPITA L REGIONA	-	Enviada	Eventos Adversos Relaciona	Lesão por pressão		
11/03/2020	CE	SOBRAL	HOSPITA L REGIONA	-	Enviada	Eventos Adversos Relaciona	Lesão por pressão		
11/03/2020	CE	SOBRAL	HOSPITA L REGIONA	-	Enviada	Eventos Adversos Relaciona	Lesão por pressão		
11/03/2020	CE	SOBRAL	HOSPITA L REGIONA	-	Enviada	Eventos Adversos Relaciona	Lesão por pressão		
11/03/2020	CE	SOBRAL	HOSPITA L REGIONA	-	Enviada	Eventos Adversos Relaciona	Lesão por pressão		
11/03/2020	CE	SOBRAL	HOSPITA L REGIONA	-	Enviada	Eventos Adversos Relaciona	Lesão por pressão		
23/03/2020	CE	SOBRAL	HOSPITA L REGIONA	-	Enviada	Eventos Adversos Relaciona	Falhas envolvend o cateter		
23/03/2020	CE	SOBRAL	HOSPITA L REGIONA	-	Enviada	Eventos Adversos Relaciona	Falhas durante a assistênci		
23/03/2020	CE	SOBRAL	HOSPITA L REGIONA	-	Enviada	Eventos Adversos Relaciona	Falhas envolvend o cateter		
23/03/2020	CE	SOBRAL	HOSPITA L REGIONA	-	Enviada	Eventos Adversos Relaciona	Falhas durante a assistênci		
23/03/2020	CE	SOBRAL	HOSPITA L REGIONA	-	Enviada	Eventos Adversos Relaciona	Falhas durante a assistênci		
26/03/2020	CE	SOBRAL	HOSPITA L REGIONA	perda de acesso central	Enviada	Eventos Adversos Relaciona	Falhas envolvend o cateter		
26/03/2020	CE	SOBRAL	HOSPITA L REGIONA	-	Enviada	Eventos Adversos Relaciona	Falhas na administra ção de		Enteral (oral, cateter
26/03/2020	CE	SOBRAL	HOSPITA L REGIONA	-	Enviada	Eventos Adversos Relaciona	Falhas durante a assistênci		
26/03/2020	CE	SOBRAL	HOSPITA L REGIONA	-	Enviada	Eventos Adversos Relaciona	Falhas envolvend o cateter		
26/03/2020	CE	SOBRAL	HOSPITA L REGIONA	-	Enviada	Eventos Adversos Relaciona	Falhas envolvend o cateter		
26/03/2020	CE	SOBRAL	HOSPITA L REGIONA	Preparo inadequad o do	Enviada	Eventos Adversos Relaciona	Falhas durante a assistênci		
26/03/2020	CE	SOBRAL	HOSPITA L REGIONA	Perda de acesso venoso	Enviada	Eventos Adversos Relaciona	Falhas envolvend o cateter		

26/03/2020	CE	SOBRAL	HOSPITAL REGIONA	Adiamento de cirurgia.	Enviada	Eventos Adversos Relaciona	Falhas durante a assistênci		
26/03/2020	CE	SOBRAL	HOSPITAL REGIONA	-	Enviada	Eventos Adversos Relaciona	Falhas envolvendo cateter		
26/03/2020	CE	SOBRAL	HOSPITAL REGIONA	Falha na prescrição/solicitaçã	Enviada	Eventos Adversos Relaciona	Falhas durante a assistênci		
26/03/2020	CE	SOBRAL	HOSPITAL REGIONA	Preparo inadequado do	Enviada	Eventos Adversos Relaciona	Falhas durante a assistênci		
26/03/2020	CE	SOBRAL	HOSPITAL REGIONA	-	Enviada	Eventos Adversos Relaciona	Lesão por pressão		
26/03/2020	CE	SOBRAL	HOSPITAL REGIONA	-	Enviada	Eventos Adversos Relaciona	Queda do paciente		
26/03/2020	CE	SOBRAL	HOSPITAL REGIONA	-	Enviada	Eventos Adversos Relaciona	Queda do paciente		
26/03/2020	CE	SOBRAL	HOSPITAL REGIONA	-	Enviada	Eventos Adversos Relaciona	Falhas na administração de		Enteral (oral, cateter
26/03/2020	CE	SOBRAL	HOSPITAL REGIONA	Preparo inadequado do	Enviada	Eventos Adversos Relaciona	Falhas durante a assistênci		
26/03/2020	CE	SOBRAL	HOSPITAL REGIONA	-	Enviada	Eventos Adversos Relaciona	Falhas envolvendo cateter		
26/03/2020	CE	SOBRAL	HOSPITAL REGIONA	Preparo inadequado do	Enviada	Eventos Adversos Relaciona	Falhas durante a assistênci		
26/03/2020	CE	SOBRAL	HOSPITAL REGIONA	-	Enviada	Eventos Adversos Relaciona	Queda do paciente		
26/03/2020	CE	SOBRAL	HOSPITAL REGIONA	-	Enviada	Eventos Adversos Relaciona	Falhas na administração de		Enteral (oral, cateter
26/03/2020	CE	SOBRAL	HOSPITAL REGIONA	-	Enviada	Eventos Adversos Relaciona	Falhas na administração de		Enteral (oral, cateter
26/03/2020	CE	SOBRAL	HOSPITAL REGIONA	Perda de acesso venoso	Enviada	Eventos Adversos Relaciona	Falhas envolvendo cateter		
26/03/2020	CE	SOBRAL	HOSPITAL REGIONA	Ausência de identificaç	Enviada	Eventos Adversos Relaciona	Falha na documentação		
26/03/2020	CE	SOBRAL	HOSPITAL REGIONA	-	Enviada	Eventos Adversos Relaciona	Queda do paciente		
26/03/2020	CE	SOBRAL	HOSPITAL REGIONA	Preparo inadequado do	Enviada	Eventos Adversos Relaciona	Falhas durante a assistênci		
26/03/2020	CE	SOBRAL	HOSPITAL REGIONA	Preparo inadequado do	Enviada	Eventos Adversos Relaciona	Falhas durante a assistênci		

26/03/2020	CE	SOBRAL	HOSPITA L REGIONA	-	Enviada	Eventos Adversos Relaciona	Lesão por pressão		
26/03/2020	CE	SOBRAL	HOSPITA L REGIONA	Remaneja mento	Enviada	Eventos Adversos Relaciona	Falhas durante a assistênci		
26/03/2020	CE	SOBRAL	HOSPITA L REGIONA	-	Enviada	Eventos Adversos Relaciona	Falhas ocorridas em		
26/03/2020	CE	SOBRAL	HOSPITA L REGIONA	-	Enviada	Eventos Adversos Relaciona	Falhas na administra ção de		Enteral (oral, cateter
26/03/2020	CE	SOBRAL	HOSPITA L REGIONA	-	Enviada	Eventos Adversos Relaciona	Falhas na administra ção de		Enteral (oral, cateter
26/03/2020	CE	SOBRAL	HOSPITA L REGIONA	Preparo inadequad o do	Enviada	Eventos Adversos Relaciona	Falhas durante a assistênci		
26/03/2020	CE	SOBRAL	HOSPITA L REGIONA	-	Enviada	Eventos Adversos Relaciona	Queda do paciente		
26/03/2020	CE	SOBRAL	HOSPITA L REGIONA	Perda de acesso venoso	Enviada	Eventos Adversos Relaciona	Falhas envolvend o cateter		
26/03/2020	CE	SOBRAL	HOSPITA L REGIONA	-	Enviada	Eventos Adversos Relaciona	Queda do paciente		
26/03/2020	CE	SOBRAL	HOSPITA L REGIONA	Perda de acesso central	Enviada	Eventos Adversos Relaciona	Falhas envolvend o cateter		
26/03/2020	CE	SOBRAL	HOSPITA L REGIONA	Prontuário com ausência	Enviada	Eventos Adversos Relaciona	Falha na document ação		
26/03/2020	CE	SOBRAL	HOSPITA L REGIONA	-	Enviada	Eventos Adversos Relaciona	Falha na identificaç ão do		
26/03/2020	CE	SOBRAL	HOSPITA L REGIONA	-	Enviada	Eventos Adversos Relaciona	Falha na identificaç ão do		
26/03/2020	CE	SOBRAL	HOSPITA L REGIONA	Perda de acesso venoso	Enviada	Eventos Adversos Relaciona	Falhas envolvend o cateter		
26/03/2020	CE	SOBRAL	HOSPITA L REGIONA	-	Enviada	Eventos Adversos Relaciona	Queda do paciente		
26/03/2020	CE	SOBRAL	HOSPITA L REGIONA	Perda de acesso central	Enviada	Eventos Adversos Relaciona	Falhas envolvend o cateter		
26/03/2020	CE	SOBRAL	HOSPITA L REGIONA	Preparo inadequad o do	Enviada	Eventos Adversos Relaciona	Falhas durante a assistênci		
26/03/2020	CE	SOBRAL	HOSPITA L REGIONA	Preparo inadequad o do	Enviada	Eventos Adversos Relaciona	Falhas durante a assistênci		
26/03/2020	CE	SOBRAL	HOSPITA L REGIONA	Perda de acesso venoso	Enviada	Eventos Adversos Relaciona	Falhas envolvend o cateter		

26/03/2020	CE	SOBRAL	HOSPITA L REGIONA	Perda de acesso central	Enviada	Eventos Adversos Relaciona	Falhas envolvend o cateter		
26/03/2020	CE	SOBRAL	HOSPITA L REGIONA	Preparo inadequad o do	Enviada	Eventos Adversos Relaciona	Falhas durante a assistênci		
26/03/2020	CE	SOBRAL	HOSPITA L REGIONA	-	Enviada	Eventos Adversos Relaciona	Queda do paciente		
26/03/2020	CE	SOBRAL	HOSPITA L REGIONA	-	Enviada	Eventos Adversos Relaciona	Falhas envolvend o cateter		
26/03/2020	CE	SOBRAL	HOSPITA L REGIONA	Preparo inadequad o do	Enviada	Eventos Adversos Relaciona	Falhas durante a assistênci		
26/03/2020	CE	SOBRAL	HOSPITA L REGIONA	Preparo inadequad o do	Enviada	Eventos Adversos Relaciona	Falhas durante a assistênci		
26/03/2020	CE	SOBRAL	HOSPITA L REGIONA	Preparo inadequad o do	Enviada	Eventos Adversos Relaciona	Falhas durante a assistênci		
26/03/2020	CE	SOBRAL	HOSPITA L REGIONA	Perda de acesso central	Enviada	Eventos Adversos Relaciona	Falhas envolvend o cateter		
26/03/2020	CE	SOBRAL	HOSPITA L REGIONA	Preparo inadequad o do	Enviada	Eventos Adversos Relaciona	Falhas durante a assistênci		
26/03/2020	CE	SOBRAL	HOSPITA L REGIONA	Preparo inadequad o do	Enviada	Eventos Adversos Relaciona	Falhas durante a assistênci		
26/03/2020	CE	SOBRAL	HOSPITA L REGIONA	-	Enviada	Eventos Adversos Relaciona	Falhas envolvend o cateter		
26/03/2020	CE	SOBRAL	HOSPITA L REGIONA	Preparo inadequad o do	Enviada	Eventos Adversos Relaciona	Falhas durante a assistênci		
26/03/2020	CE	SOBRAL	HOSPITA L REGIONA	-	Enviada	Eventos Adversos Relaciona	Queda do paciente		
26/03/2020	CE	SOBRAL	HOSPITA L REGIONA	Preparo inadequad o do	Enviada	Eventos Adversos Relaciona	Falhas durante a assistênci		
26/03/2020	CE	SOBRAL	HOSPITA L REGIONA	Preparo inadequad o do	Enviada	Eventos Adversos Relaciona	Falhas durante a assistênci		
26/03/2020	CE	SOBRAL	HOSPITA L REGIONA	Preparo inadequad o do	Enviada	Eventos Adversos Relaciona	Falhas durante a assistênci		
26/03/2020	CE	SOBRAL	HOSPITA L REGIONA	-	Enviada	Eventos Adversos Relaciona	Queda do paciente		
26/03/2020	CE	SOBRAL	HOSPITA L REGIONA	Preparo inadequad o do	Enviada	Eventos Adversos Relaciona	Falhas durante a assistênci		
30/03/2020	CE	SOBRAL	HOSPITA L REGIONA	Preparo inadequad o do	Enviada	Eventos Adversos Relaciona	Falhas durante a assistênci		

30/03/2020	CE	SOBRAL	HOSPITAL REGIONA	Preparo inadequado do	Enviada	Eventos Adversos Relaciona	Falhas durante a assistênci		
30/03/2020	CE	SOBRAL	HOSPITAL REGIONA	-	Enviada	Eventos Adversos Relaciona	Falhas na administração de		Enteral (oral, cateter
30/03/2020	CE	SOBRAL	HOSPITAL REGIONA	-	Enviada	Eventos Adversos Relaciona	Falhas na administração de		Enteral (oral, cateter
30/03/2020	CE	SOBRAL	HOSPITAL REGIONA	-	Enviada	Eventos Adversos Relaciona	Falhas envolvendo cateter		
30/03/2020	CE	SOBRAL	HOSPITAL REGIONA	-	Enviada	Eventos Adversos Relaciona	Queda do paciente		
30/03/2020	CE	SOBRAL	HOSPITAL REGIONA	-	Enviada	Eventos Adversos Relaciona	Lesão por pressão		
30/03/2020	CE	SOBRAL	HOSPITAL REGIONA	Preparo inadequado do	Enviada	Eventos Adversos Relaciona	Falhas durante a assistênci		
30/03/2020	CE	SOBRAL	HOSPITAL REGIONA	Preparo inadequado do	Enviada	Eventos Adversos Relaciona	Falhas durante a assistênci		
30/03/2020	CE	SOBRAL	HOSPITAL REGIONA	Preparo inadequado do	Enviada	Eventos Adversos Relaciona	Falhas durante a assistênci		
30/03/2020	CE	SOBRAL	HOSPITAL REGIONA	-	Enviada	Eventos Adversos Relaciona	Falhas na administração de		Enteral (oral, cateter
30/03/2020	CE	SOBRAL	HOSPITAL REGIONA	Perda de acesso central	Enviada	Eventos Adversos Relaciona	Falhas envolvendo cateter		
30/03/2020	CE	SOBRAL	HOSPITAL REGIONA	Preparo inadequado do	Enviada	Eventos Adversos Relaciona	Falhas durante a assistênci		
30/03/2020	CE	SOBRAL	HOSPITAL REGIONA	Preparo inadequado do	Enviada	Eventos Adversos Relaciona	Falhas durante a assistênci		
30/03/2020	CE	SOBRAL	HOSPITAL REGIONA	Perda de acesso venoso	Enviada	Eventos Adversos Relaciona	Falhas envolvendo cateter		
30/03/2020	CE	SOBRAL	HOSPITAL REGIONA	-	Enviada	Eventos Adversos Relaciona	Falhas envolvendo cateter		
30/03/2020	CE	SOBRAL	HOSPITAL REGIONA	-	Enviada	Eventos Adversos Relaciona	Falhas envolvendo cateter		
30/03/2020	CE	SOBRAL	HOSPITAL REGIONA	-	Enviada	Eventos Adversos Relaciona	Falhas envolvendo cateter		
30/03/2020	CE	SOBRAL	HOSPITAL REGIONA	-	Enviada	Eventos Adversos Relaciona	Lesão por pressão		
30/03/2020	CE	SOBRAL	HOSPITAL REGIONA	-	Enviada	Eventos Adversos Relaciona	Lesão por pressão		

30/03/2020	CE	SOBRAL	HOSPITA L REGIONA	-	Enviada	Eventos Adversos Relaciona	Lesão por pressão		
30/03/2020	CE	SOBRAL	HOSPITA L REGIONA	-	Enviada	Eventos Adversos Relaciona	Lesão por pressão		
30/03/2020	CE	SOBRAL	HOSPITA L REGIONA	-	Enviada	Eventos Adversos Relaciona	Lesão por pressão		
30/03/2020	CE	SOBRAL	HOSPITA L REGIONA	-	Enviada	Eventos Adversos Relaciona	Lesão por pressão		
30/03/2020	CE	SOBRAL	HOSPITA L REGIONA	-	Enviada	Eventos Adversos Relaciona	Lesão por pressão		
30/03/2020	CE	SOBRAL	HOSPITA L REGIONA	-	Enviada	Eventos Adversos Relaciona	Lesão por pressão		
30/03/2020	CE	SOBRAL	HOSPITA L REGIONA	-	Enviada	Eventos Adversos Relaciona	Lesão por pressão		
30/03/2020	CE	SOBRAL	HOSPITA L REGIONA	-	Enviada	Eventos Adversos Relaciona	Lesão por pressão		
30/03/2020	CE	SOBRAL	HOSPITA L REGIONA	-	Enviada	Eventos Adversos Relaciona	Lesão por pressão		
30/03/2020	CE	SOBRAL	HOSPITA L REGIONA	-	Enviada	Eventos Adversos Relaciona	Lesão por pressão		
30/03/2020	CE	SOBRAL	HOSPITA L REGIONA	-	Enviada	Eventos Adversos Relaciona	Lesão por pressão		
30/03/2020	CE	SOBRAL	HOSPITA L REGIONA	-	Enviada	Eventos Adversos Relaciona	Lesão por pressão		
30/03/2020	CE	SOBRAL	HOSPITA L REGIONA	-	Enviada	Eventos Adversos Relaciona	Lesão por pressão		
30/03/2020	CE	SOBRAL	HOSPITA L REGIONA	-	Enviada	Eventos Adversos Relaciona	Lesão por pressão		
30/03/2020	CE	SOBRAL	HOSPITA L REGIONA	-	Enviada	Eventos Adversos Relaciona	Lesão por pressão		
30/03/2020	CE	SOBRAL	HOSPITA L REGIONA	-	Enviada	Eventos Adversos Relaciona	Lesão por pressão		
30/03/2020	CE	SOBRAL	HOSPITA L REGIONA	-	Enviada	Eventos Adversos Relaciona	Lesão por pressão		
30/03/2020	CE	SOBRAL	HOSPITA L REGIONA	-	Enviada	Eventos Adversos Relaciona	Lesão por pressão		
30/03/2020	CE	SOBRAL	HOSPITA L REGIONA	-	Enviada	Eventos Adversos Relaciona	Lesão por pressão		

30/03/2020	CE	SOBRAL	HOSPITA L REGIONA	-	Enviada	Eventos Adversos Relaciona	Lesão por pressão		
30/03/2020	CE	SOBRAL	HOSPITA L REGIONA	-	Enviada	Eventos Adversos Relaciona	Lesão por pressão		
30/03/2020	CE	SOBRAL	HOSPITA L REGIONA	-	Enviada	Eventos Adversos Relaciona	Lesão por pressão		
30/03/2020	CE	SOBRAL	HOSPITA L REGIONA	-	Enviada	Eventos Adversos Relaciona	Lesão por pressão		
30/03/2020	CE	SOBRAL	HOSPITA L REGIONA	-	Enviada	Eventos Adversos Relaciona	Lesão por pressão		
30/03/2020	CE	SOBRAL	HOSPITA L REGIONA	-	Enviada	Eventos Adversos Relaciona	Lesão por pressão		
30/03/2020	CE	SOBRAL	HOSPITA L REGIONA	-	Enviada	Eventos Adversos Relaciona	Lesão por pressão		
30/03/2020	CE	SOBRAL	HOSPITA L REGIONA	-	Enviada	Eventos Adversos Relaciona	Lesão por pressão		
30/03/2020	CE	SOBRAL	HOSPITA L REGIONA	-	Enviada	Eventos Adversos Relaciona	Lesão por pressão		
30/03/2020	CE	SOBRAL	HOSPITA L REGIONA	-	Enviada	Eventos Adversos Relaciona	Lesão por pressão		
30/03/2020	CE	SOBRAL	HOSPITA L REGIONA	-	Enviada	Eventos Adversos Relaciona	Lesão por pressão		
30/03/2020	CE	SOBRAL	HOSPITA L REGIONA	-	Enviada	Eventos Adversos Relaciona	Lesão por pressão		
30/03/2020	CE	SOBRAL	HOSPITA L REGIONA	-	Enviada	Eventos Adversos Relaciona	Lesão por pressão		
30/03/2020	CE	SOBRAL	HOSPITA L REGIONA	-	Enviada	Eventos Adversos Relaciona	Lesão por pressão		
30/03/2020	CE	SOBRAL	HOSPITA L REGIONA	-	Enviada	Eventos Adversos Relaciona	Lesão por pressão		
30/03/2020	CE	SOBRAL	HOSPITA L REGIONA	-	Enviada	Eventos Adversos Relaciona	Lesão por pressão		
30/03/2020	CE	SOBRAL	HOSPITA L REGIONA	-	Enviada	Eventos Adversos Relaciona	Lesão por pressão		
30/03/2020	CE	SOBRAL	HOSPITA L REGIONA	-	Enviada	Eventos Adversos Relaciona	Lesão por pressão		
30/03/2020	CE	SOBRAL	HOSPITA L REGIONA	-	Enviada	Eventos Adversos Relaciona	Lesão por pressão		

30/03/2020	CE	SOBRAL	HOSPITA L REGIONA	-	Enviada	Eventos Adversos Relaciona	Lesão por pressão		
30/03/2020	CE	SOBRAL	HOSPITA L REGIONA	-	Enviada	Eventos Adversos Relaciona	Lesão por pressão		
30/03/2020	CE	SOBRAL	HOSPITA L REGIONA	-	Enviada	Eventos Adversos Relaciona	Lesão por pressão		
02/04/2020	CE	SOBRAL	HOSPITA L REGIONA	-	Enviada	Eventos Adversos Relaciona	Falhas envolvend o cateter		
02/04/2020	CE	SOBRAL	HOSPITA L REGIONA	Preparo inadequad o do	Enviada	Eventos Adversos Relaciona	Falhas durante a assistênci		
02/04/2020	CE	SOBRAL	HOSPITA L REGIONA	-	Enviada	Eventos Adversos Relaciona	Falhas envolvend o cateter		
02/04/2020	CE	SOBRAL	HOSPITA L REGIONA	Perda de acesso central	Enviada	Eventos Adversos Relaciona	Falhas envolvend o cateter		
02/04/2020	CE	SOBRAL	HOSPITA L REGIONA	-	Enviada	Eventos Adversos Relaciona	Falhas envolvend o cateter		
02/04/2020	CE	SOBRAL	HOSPITA L REGIONA	Perda de acesso venoso	Enviada	Eventos Adversos Relaciona	Falhas envolvend o cateter		
02/04/2020	CE	SOBRAL	HOSPITA L REGIONA	Perda de acesso venoso	Enviada	Eventos Adversos Relaciona	Falhas envolvend o cateter		
02/04/2020	CE	SOBRAL	HOSPITA L REGIONA	Perda de acesso venoso	Enviada	Eventos Adversos Relaciona	Falhas envolvend o cateter		
02/04/2020	CE	SOBRAL	HOSPITA L REGIONA	Perda de acesso venoso	Enviada	Eventos Adversos Relaciona	Falhas envolvend o cateter		
02/04/2020	CE	SOBRAL	HOSPITA L REGIONA	Preparo inadequad o do	Enviada	Eventos Adversos Relaciona	Falhas durante a assistênci		
02/04/2020	CE	SOBRAL	HOSPITA L REGIONA	Preparo inadequad o do	Enviada	Eventos Adversos Relaciona	Falhas durante a assistênci		
03/04/2020	CE	SOBRAL	HOSPITA L REGIONA	Perda de acesso central	Enviada	Eventos Adversos Relaciona	Falhas envolvend o cateter		
03/04/2020	CE	SOBRAL	HOSPITA L REGIONA	Preparo inadequad o do	Enviada	Eventos Adversos Relaciona	Falhas durante a assistênci		
03/04/2020	CE	SOBRAL	HOSPITA L REGIONA	Perda de acesso venoso	Enviada	Eventos Adversos Relaciona	Falhas envolvend o cateter		
03/04/2020	CE	SOBRAL	HOSPITA L REGIONA	Perda de acesso venoso	Enviada	Eventos Adversos Relaciona	Falhas envolvend o cateter		
03/04/2020	CE	SOBRAL	HOSPITA L REGIONA	-	Enviada	Eventos Adversos Relaciona	Falhas envolvend o cateter		

03/04/2020	CE	SOBRAL	HOSPITA L REGIONA	-	Enviada	Eventos Adversos Relaciona	Falhas envolvend o cateter		
03/04/2020	CE	SOBRAL	HOSPITA L REGIONA	Preparo inadequad o do	Enviada	Eventos Adversos Relaciona	Falhas durante a assistênci		
03/04/2020	CE	SOBRAL	HOSPITA L REGIONA	Preparo inadequad o do	Enviada	Eventos Adversos Relaciona	Falhas durante a assistênci		
03/04/2020	CE	SOBRAL	HOSPITA L REGIONA	Saída não planejada	Enviada	Eventos Adversos Relaciona	Falhas durante a assistênci		
03/04/2020	CE	SOBRAL	HOSPITA L REGIONA	Saída não planejada	Enviada	Eventos Adversos Relaciona	Falhas durante a assistênci		
03/04/2020	CE	SOBRAL	HOSPITA L REGIONA	-	Enviada	Eventos Adversos Relaciona	Falhas envolvend o cateter		
03/04/2020	CE	SOBRAL	HOSPITA L REGIONA	Perda de acesso central	Enviada	Eventos Adversos Relaciona	Falhas envolvend o cateter		
03/04/2020	CE	SOBRAL	HOSPITA L REGIONA	-	Enviada	Eventos Adversos Relaciona	Falhas envolvend o cateter		
03/04/2020	CE	SOBRAL	HOSPITA L REGIONA	Preparo inadequad o do	Enviada	Eventos Adversos Relaciona	Falhas durante a assistênci		
03/04/2020	CE	SOBRAL	HOSPITA L REGIONA	-	Enviada	Eventos Adversos Relaciona	Falhas envolvend o cateter		
03/04/2020	CE	SOBRAL	HOSPITA L REGIONA	Perda de acesso venoso	Enviada	Eventos Adversos Relaciona	Falhas envolvend o cateter		
03/04/2020	CE	SOBRAL	HOSPITA L REGIONA	Dispositiv o em uso fora do	Enviada	Eventos Adversos Relaciona	Falhas durante a assistênci		
03/04/2020	CE	SOBRAL	HOSPITA L REGIONA	Perda de acesso venoso	Enviada	Eventos Adversos Relaciona	Falhas envolvend o cateter		
03/04/2020	CE	SOBRAL	HOSPITA L REGIONA	-	Enviada	Eventos Adversos Relaciona	Falhas envolvend o cateter		
03/04/2020	CE	SOBRAL	HOSPITA L REGIONA	Preparo inadequad o do	Enviada	Eventos Adversos Relaciona	Falhas durante a assistênci		
03/04/2020	CE	SOBRAL	HOSPITA L REGIONA	-	Enviada	Eventos Adversos Relaciona	Falhas envolvend o cateter		
03/04/2020	CE	SOBRAL	HOSPITA L REGIONA	Dispositiv o em uso fora do	Enviada	Eventos Adversos Relaciona	Falhas durante a assistênci		
03/04/2020	CE	SOBRAL	HOSPITA L REGIONA	Saída não planejada	Enviada	Eventos Adversos Relaciona	Falhas durante a assistênci		
03/04/2020	CE	SOBRAL	HOSPITA L REGIONA	Preparo inadequad o do	Enviada	Eventos Adversos Relaciona	Falhas durante a assistênci		

03/04/2020	CE	SOBRAL	HOSPITA L REGIONA	Perda de acesso venoso	Enviada	Eventos Adversos Relaciona	Falhas envolvend o cateter		
03/04/2020	CE	SOBRAL	HOSPITA L REGIONA	Preparo inadequad o do	Enviada	Eventos Adversos Relaciona	Falhas durante a assistênci		
03/04/2020	CE	SOBRAL	HOSPITA L REGIONA	Perda de acesso venoso	Enviada	Eventos Adversos Relaciona	Falhas envolvend o cateter		
13/05/2020	CE	SOBRAL	HOSPITA L REGIONA	-	Enviada	Eventos Adversos Relaciona	Lesão por pressão		
13/05/2020	CE	SOBRAL	HOSPITA L REGIONA	-	Enviada	Eventos Adversos Relaciona	Lesão por pressão		
13/05/2020	CE	SOBRAL	HOSPITA L REGIONA	-	Enviada	Eventos Adversos Relaciona	Lesão por pressão		
14/05/2020	CE	SOBRAL	HOSPITA L REGIONA	-	Enviada	Eventos Adversos Relaciona	Lesão por pressão		
14/05/2020	CE	SOBRAL	HOSPITA L REGIONA	-	Enviada	Eventos Adversos Relaciona	Lesão por pressão		
14/05/2020	CE	SOBRAL	HOSPITA L REGIONA	-	Enviada	Eventos Adversos Relaciona	Lesão por pressão		
14/05/2020	CE	SOBRAL	HOSPITA L REGIONA	-	Enviada	Eventos Adversos Relaciona	Lesão por pressão		
14/05/2020	CE	SOBRAL	HOSPITA L REGIONA	-	Enviada	Eventos Adversos Relaciona	Lesão por pressão		
14/05/2020	CE	SOBRAL	HOSPITA L REGIONA	-	Enviada	Eventos Adversos Relaciona	Lesão por pressão		
14/05/2020	CE	SOBRAL	HOSPITA L REGIONA	-	Enviada	Eventos Adversos Relaciona	Lesão por pressão		
14/05/2020	CE	SOBRAL	HOSPITA L REGIONA	-	Enviada	Eventos Adversos Relaciona	Lesão por pressão		
14/05/2020	CE	SOBRAL	HOSPITA L REGIONA	-	Enviada	Eventos Adversos Relaciona	Lesão por pressão		
14/05/2020	CE	SOBRAL	HOSPITA L REGIONA	-	Enviada	Eventos Adversos Relaciona	Lesão por pressão		
14/05/2020	CE	SOBRAL	HOSPITA L REGIONA	-	Enviada	Eventos Adversos Relaciona	Lesão por pressão		
14/05/2020	CE	SOBRAL	HOSPITA L REGIONA	-	Enviada	Eventos Adversos Relaciona	Lesão por pressão		
14/05/2020	CE	SOBRAL	HOSPITA L REGIONA	-	Enviada	Eventos Adversos Relaciona	Lesão por pressão		

14/05/2020	CE	SOBRAL	HOSPITA L REGIONA	-	Enviada	Eventos Adversos Relaciona	Lesão por pressão			
14/05/2020	CE	SOBRAL	HOSPITA L REGIONA	-	Enviada	Eventos Adversos Relaciona	Lesão por pressão			
14/05/2020	CE	SOBRAL	HOSPITA L REGIONA	-	Enviada	Eventos Adversos Relaciona	Lesão por pressão			
14/05/2020	CE	SOBRAL	HOSPITA L REGIONA	-	Enviada	Eventos Adversos Relaciona	Lesão por pressão			
14/05/2020	CE	SOBRAL	HOSPITA L REGIONA	-	Enviada	Eventos Adversos Relaciona	Lesão por pressão			
14/05/2020	CE	SOBRAL	HOSPITA L REGIONA	-	Enviada	Eventos Adversos Relaciona	Lesão por pressão			
14/05/2020	CE	SOBRAL	HOSPITA L REGIONA	-	Enviada	Eventos Adversos Relaciona	Lesão por pressão			
14/05/2020	CE	SOBRAL	HOSPITA L REGIONA	-	Enviada	Eventos Adversos Relaciona	Lesão por pressão			
14/05/2020	CE	SOBRAL	HOSPITA L REGIONA	-	Enviada	Eventos Adversos Relaciona	Lesão por pressão			
14/05/2020	CE	SOBRAL	HOSPITA L REGIONA	-	Enviada	Eventos Adversos Relaciona	Lesão por pressão			
14/05/2020	CE	SOBRAL	HOSPITA L REGIONA	-	Enviada	Eventos Adversos Relaciona	Lesão por pressão			
14/05/2020	CE	SOBRAL	HOSPITA L REGIONA	-	Enviada	Eventos Adversos Relaciona	Lesão por pressão			
14/05/2020	CE	SOBRAL	HOSPITA L REGIONA	-	Enviada	Eventos Adversos Relaciona	Lesão por pressão			
14/05/2020	CE	SOBRAL	HOSPITA L REGIONA	-	Enviada	Eventos Adversos Relaciona	Lesão por pressão			
14/05/2020	CE	SOBRAL	HOSPITA L REGIONA	-	Enviada	Eventos Adversos Relaciona	Lesão por pressão			
14/05/2020	CE	SOBRAL	HOSPITA L REGIONA	-	Enviada	Eventos Adversos Relaciona	Lesão por pressão			
14/05/2020	CE	SOBRAL	HOSPITA L REGIONA	-	Enviada	Eventos Adversos Relaciona	Lesão por pressão			
14/05/2020	CE	SOBRAL	HOSPITA L REGIONA	-	Enviada	Eventos Adversos Relaciona	Lesão por pressão			
14/05/2020	CE	SOBRAL	HOSPITA L REGIONA	-	Enviada	Eventos Adversos Relaciona	Lesão por pressão			

14/05/2020	CE	SOBRAL	HOSPITA<br>L<br>REGIONA	-	Enviada	Eventos<br>Adversos<br>Relaciona	Lesão<br>por<br>pressão		
14/05/2020	CE	SOBRAL	HOSPITA<br>L<br>REGIONA	-	Enviada	Eventos<br>Adversos<br>Relaciona	Lesão<br>por<br>pressão		
14/05/2020	CE	SOBRAL	HOSPITA<br>L<br>REGIONA	-	Enviada	Eventos<br>Adversos<br>Relaciona	Lesão<br>por<br>pressão		
14/05/2020	CE	SOBRAL	HOSPITA<br>L<br>REGIONA	-	Enviada	Eventos<br>Adversos<br>Relaciona	Lesão<br>por<br>pressão		
26/05/2020	CE	SOBRAL	HOSPITA<br>L<br>REGIONA	-	Enviada	Eventos<br>Adversos<br>Relaciona	Lesão<br>por<br>pressão		
26/05/2020	CE	SOBRAL	HOSPITA<br>L<br>REGIONA	-	Enviada	Eventos<br>Adversos<br>Relaciona	Lesão<br>por<br>pressão		
26/05/2020	CE	SOBRAL	HOSPITA<br>L<br>REGIONA	Dispositiv<br>o em uso<br>fora do	Enviada	Eventos<br>Adversos<br>Relaciona	Falhas<br>durante a<br>assistênci		
26/05/2020	CE	SOBRAL	HOSPITA<br>L<br>REGIONA	Dispositiv<br>o em uso<br>fora do	Enviada	Eventos<br>Adversos<br>Relaciona	Falhas<br>durante a<br>assistênci		
26/05/2020	CE	SOBRAL	HOSPITA<br>L<br>REGIONA	Dispositiv<br>o em uso<br>fora do	Enviada	Eventos<br>Adversos<br>Relaciona	Falhas<br>durante a<br>assistênci		
26/05/2020	CE	SOBRAL	HOSPITA<br>L<br>REGIONA	-	Enviada	Eventos<br>Adversos<br>Relaciona	Falhas<br>durante a<br>assistênci		
26/05/2020	CE	SOBRAL	HOSPITA<br>L<br>REGIONA	-	Enviada	Eventos<br>Adversos<br>Relaciona	Falhas<br>durante a<br>assistênci		
26/05/2020	CE	SOBRAL	HOSPITA<br>L<br>REGIONA	-	Enviada	Eventos<br>Adversos<br>Relaciona	Falhas<br>durante a<br>assistênci		
26/05/2020	CE	SOBRAL	HOSPITA<br>L<br>REGIONA	-	Enviada	Eventos<br>Adversos<br>Relaciona	Falhas<br>durante a<br>assistênci		
26/05/2020	CE	SOBRAL	HOSPITA<br>L<br>REGIONA	-	Enviada	Eventos<br>Adversos<br>Relaciona	Falhas<br>durante a<br>assistênci		
26/05/2020	CE	SOBRAL	HOSPITA<br>L<br>REGIONA	-	Enviada	Eventos<br>Adversos<br>Relaciona	Falhas<br>durante a<br>assistênci		
26/05/2020	CE	SOBRAL	HOSPITA<br>L<br>REGIONA	Perda de<br>acesso<br>venoso	Enviada	Eventos<br>Adversos<br>Relaciona	Falhas<br>durante a<br>assistênci		
26/05/2020	CE	SOBRAL	HOSPITA<br>L<br>REGIONA	Perda de<br>acesso<br>venoso	Enviada	Eventos<br>Adversos<br>Relaciona	Falhas<br>durante a<br>assistênci		
26/05/2020	CE	SOBRAL	HOSPITA<br>L<br>REGIONA	Perda de<br>acesso<br>venoso	Enviada	Eventos<br>Adversos<br>Relaciona	Falhas<br>durante a<br>assistênci		
26/05/2020	CE	SOBRAL	HOSPITA<br>L<br>REGIONA	Perda de<br>acesso<br>venoso	Enviada	Eventos<br>Adversos<br>Relaciona	Falhas<br>durante a<br>assistênci		

26/05/2020	CE	SOBRAL	HOSPITA L REGIONA	Perda de acesso venoso	Enviada	Eventos Adversos Relaciona	Falhas durante a assistênci		
26/05/2020	CE	SOBRAL	HOSPITA L REGIONA	Perda de acesso venoso	Enviada	Eventos Adversos Relaciona	Falhas durante a assistênci		
26/05/2020	CE	SOBRAL	HOSPITA L REGIONA	-	Enviada	Eventos Adversos Relaciona	Falhas durante a assistênci		
24/06/2020	CE	SOBRAL	HOSPITA L REGIONA	Perda de acesso venoso	Enviada	Eventos Adversos Relaciona	Falhas durante a assistênci		
24/06/2020	CE	SOBRAL	HOSPITA L REGIONA	Perda de acesso venoso	Enviada	Eventos Adversos Relaciona	Falhas durante a assistênci		
24/06/2020	CE	SOBRAL	HOSPITA L REGIONA	Perda de acesso venoso	Enviada	Eventos Adversos Relaciona	Falhas durante a assistênci		
24/06/2020	CE	SOBRAL	HOSPITA L REGIONA	-	Enviada	Eventos Adversos Relaciona	Falhas durante a assistênci		
24/06/2020	CE	SOBRAL	HOSPITA L REGIONA	-	Enviada	Eventos Adversos Relaciona	Falhas durante a assistênci		
24/06/2020	CE	SOBRAL	HOSPITA L REGIONA	-	Enviada	Eventos Adversos Relaciona	Falhas durante a assistênci		
24/06/2020	CE	SOBRAL	HOSPITA L REGIONA	-	Enviada	Eventos Adversos Relaciona	Falhas durante a assistênci		
24/06/2020	CE	SOBRAL	HOSPITA L REGIONA	-	Enviada	Eventos Adversos Relaciona	Falhas durante a assistênci		
24/06/2020	CE	SOBRAL	HOSPITA L REGIONA	-	Enviada	Eventos Adversos Relaciona	Falhas durante a assistênci		
24/06/2020	CE	SOBRAL	HOSPITA L REGIONA	-	Enviada	Eventos Adversos Relaciona	Falhas durante a assistênci		
24/06/2020	CE	SOBRAL	HOSPITA L REGIONA	-	Enviada	Eventos Adversos Relaciona	Falhas durante a assistênci		
24/06/2020	CE	SOBRAL	HOSPITA L REGIONA	-	Enviada	Eventos Adversos Relaciona	Falhas durante a assistênci		
30/07/2020	CE	SOBRAL	HOSPITA L REGIONA	-	Enviada	Eventos Adversos Relaciona	Falhas na administra ção de		Enteral (oral, cateter
30/07/2020	CE	SOBRAL	HOSPITA L REGIONA	-	Enviada	Eventos Adversos Relaciona	Falhas na administra ção de		Enteral (oral, cateter
30/07/2020	CE	SOBRAL	HOSPITA L REGIONA	-	Enviada	Eventos Adversos Relaciona	Falhas na administra ção de		Enteral (oral, cateter
30/07/2020	CE	SOBRAL	HOSPITA L REGIONA	-	Enviada	Eventos Adversos Relaciona	Falhas na administra ção de		Enteral (oral, cateter

30/07/2020	CE	SOBRAL	HOSPITA L REGIONA	-	Enviada	Eventos Adversos Relaciona	Falhas na administra ção de			Enteral (oral, cateter
30/07/2020	CE	SOBRAL	HOSPITA L REGIONA	-	Enviada	Eventos Adversos Relaciona	Falhas na administra ção de			Enteral (oral, cateter
30/07/2020	CE	SOBRAL	HOSPITA L REGIONA	-	Enviada	Eventos Adversos Relaciona	Falhas na administra ção de			Enteral (oral, cateter
29/10/2020	CE	SOBRAL	HOSPITA L REGIONA	-	Enviada	Eventos Adversos Relaciona	Lesão por pressão			
29/10/2020	CE	SOBRAL	HOSPITA L REGIONA	-	Enviada	Eventos Adversos Relaciona	Lesão por pressão			
29/10/2020	CE	SOBRAL	HOSPITA L REGIONA	-	Enviada	Eventos Adversos Relaciona	Lesão por pressão			
29/10/2020	CE	SOBRAL	HOSPITA L REGIONA	-	Enviada	Eventos Adversos Relaciona	Lesão por pressão			
29/10/2020	CE	SOBRAL	HOSPITA L REGIONA	-	Enviada	Eventos Adversos Relaciona	Lesão por pressão			
29/10/2020	CE	SOBRAL	HOSPITA L REGIONA	-	Enviada	Eventos Adversos Relaciona	Lesão por pressão			
29/10/2020	CE	SOBRAL	HOSPITA L REGIONA	-	Enviada	Eventos Adversos Relaciona	Lesão por pressão			
29/10/2020	CE	SOBRAL	HOSPITA L REGIONA	-	Enviada	Eventos Adversos Relaciona	Lesão por pressão			
29/10/2020	CE	SOBRAL	HOSPITA L REGIONA	-	Enviada	Eventos Adversos Relaciona	Lesão por pressão			
29/10/2020	CE	SOBRAL	HOSPITA L REGIONA	-	Enviada	Eventos Adversos Relaciona	Lesão por pressão			
29/10/2020	CE	SOBRAL	HOSPITA L REGIONA	-	Enviada	Eventos Adversos Relaciona	Lesão por pressão			
29/10/2020	CE	SOBRAL	HOSPITA L REGIONA	-	Enviada	Eventos Adversos Relaciona	Lesão por pressão			
29/10/2020	CE	SOBRAL	HOSPITA L REGIONA	-	Enviada	Eventos Adversos Relaciona	Lesão por pressão			
29/10/2020	CE	SOBRAL	HOSPITA L REGIONA	-	Enviada	Eventos Adversos Relaciona	Lesão por pressão			
29/10/2020	CE	SOBRAL	HOSPITA L REGIONA	-	Enviada	Eventos Adversos Relaciona	Lesão por pressão			
29/10/2020	CE	SOBRAL	HOSPITA L REGIONA	-	Enviada	Eventos Adversos Relaciona	Lesão por pressão			

29/10/2020	CE	SOBRAL	HOSPITA L REGIONA	-	Enviada	Eventos Adversos Relaciona	Lesão por pressão		
30/10/2020	CE	SOBRAL	HOSPITA L REGIONA	-	Enviada	Eventos Adversos Relaciona	Lesão por pressão		
30/10/2020	CE	SOBRAL	HOSPITA L REGIONA	-	Enviada	Eventos Adversos Relaciona	Lesão por pressão		
30/10/2020	CE	SOBRAL	HOSPITA L REGIONA	-	Enviada	Eventos Adversos Relaciona	Lesão por pressão		
30/10/2020	CE	SOBRAL	HOSPITA L REGIONA	-	Enviada	Eventos Adversos Relaciona	Lesão por pressão		
30/10/2020	CE	SOBRAL	HOSPITA L REGIONA	-	Enviada	Eventos Adversos Relaciona	Lesão por pressão		
30/10/2020	CE	SOBRAL	HOSPITA L REGIONA	-	Enviada	Eventos Adversos Relaciona	Lesão por pressão		
30/10/2020	CE	SOBRAL	HOSPITA L REGIONA	-	Enviada	Eventos Adversos Relaciona	Lesão por pressão		
30/10/2020	CE	SOBRAL	HOSPITA L REGIONA	-	Enviada	Eventos Adversos Relaciona	Lesão por pressão		
30/10/2020	CE	SOBRAL	HOSPITA L REGIONA	-	Enviada	Eventos Adversos Relaciona	Lesão por pressão		
30/10/2020	CE	SOBRAL	HOSPITA L REGIONA	-	Enviada	Eventos Adversos Relaciona	Lesão por pressão		
30/10/2020	CE	SOBRAL	HOSPITA L REGIONA	-	Enviada	Eventos Adversos Relaciona	Lesão por pressão		
30/10/2020	CE	SOBRAL	HOSPITA L REGIONA	-	Enviada	Eventos Adversos Relaciona	Lesão por pressão		
30/10/2020	CE	SOBRAL	HOSPITA L REGIONA	-	Enviada	Eventos Adversos Relaciona	Lesão por pressão		
05/11/2020	CE	SOBRAL	HOSPITA L REGIONA	-	Enviada	Eventos Adversos Relaciona	Lesão por pressão		
05/11/2020	CE	SOBRAL	HOSPITA L REGIONA	-	Enviada	Eventos Adversos Relaciona	Lesão por pressão		
05/11/2020	CE	SOBRAL	HOSPITA L REGIONA	-	Enviada	Eventos Adversos Relaciona	Lesão por pressão		
05/11/2020	CE	SOBRAL	HOSPITA L REGIONA	-	Enviada	Eventos Adversos Relaciona	Lesão por pressão		
05/11/2020	CE	SOBRAL	HOSPITA L REGIONA	-	Enviada	Eventos Adversos Relaciona	Lesão por pressão		

05/11/2020	CE	SOBRAL	HOSPITA L REGIONA	-	Enviada	Eventos Adversos Relaciona	Lesão por pressão		
05/11/2020	CE	SOBRAL	HOSPITA L REGIONA	-	Enviada	Eventos Adversos Relaciona	Lesão por pressão		
05/11/2020	CE	SOBRAL	HOSPITA L REGIONA	-	Enviada	Eventos Adversos Relaciona	Lesão por pressão		
05/11/2020	CE	SOBRAL	HOSPITA L REGIONA	-	Enviada	Eventos Adversos Relaciona	Lesão por pressão		
05/11/2020	CE	SOBRAL	HOSPITA L REGIONA	-	Enviada	Eventos Adversos Relaciona	Lesão por pressão		
06/11/2020	CE	SOBRAL	HOSPITA L REGIONA	-	Enviada	Eventos Adversos Relaciona	Lesão por pressão		
06/11/2020	CE	SOBRAL	HOSPITA L REGIONA	-	Enviada	Eventos Adversos Relaciona	Lesão por pressão		
06/11/2020	CE	SOBRAL	HOSPITA L REGIONA	-	Enviada	Eventos Adversos Relaciona	Lesão por pressão		
06/11/2020	CE	SOBRAL	HOSPITA L REGIONA	-	Enviada	Eventos Adversos Relaciona	Lesão por pressão		
06/11/2020	CE	SOBRAL	HOSPITA L REGIONA	-	Enviada	Eventos Adversos Relaciona	Lesão por pressão		
06/11/2020	CE	SOBRAL	HOSPITA L REGIONA	-	Enviada	Eventos Adversos Relaciona	Lesão por pressão		
06/11/2020	CE	SOBRAL	HOSPITA L REGIONA	-	Enviada	Eventos Adversos Relaciona	Lesão por pressão		
06/11/2020	CE	SOBRAL	HOSPITA L REGIONA	-	Enviada	Eventos Adversos Relaciona	Lesão por pressão		
06/11/2020	CE	SOBRAL	HOSPITA L REGIONA	-	Enviada	Eventos Adversos Relaciona	Lesão por pressão		
06/11/2020	CE	SOBRAL	HOSPITA L REGIONA	-	Enviada	Eventos Adversos Relaciona	Lesão por pressão		
06/11/2020	CE	SOBRAL	HOSPITA L REGIONA	-	Enviada	Eventos Adversos Relaciona	Lesão por pressão		
09/11/2020	CE	SOBRAL	HOSPITA L REGIONA	-	Enviada	Eventos Adversos Relaciona	Lesão por pressão		
09/11/2020	CE	SOBRAL	HOSPITA L REGIONA	-	Enviada	Eventos Adversos Relaciona	Lesão por pressão		
09/11/2020	CE	SOBRAL	HOSPITA L REGIONA	-	Enviada	Eventos Adversos Relaciona	Lesão por pressão		

09/11/2020	CE	SOBRAL	HOSPITAL REGIONA	-	Enviada	Eventos Adversos Relaciona	Lesão por pressão		
09/11/2020	CE	SOBRAL	HOSPITAL REGIONA	-	Enviada	Eventos Adversos Relaciona	Lesão por pressão		
09/11/2020	CE	SOBRAL	HOSPITAL REGIONA	-	Enviada	Eventos Adversos Relaciona	Lesão por pressão		
09/11/2020	CE	SOBRAL	HOSPITAL REGIONA	-	Enviada	Eventos Adversos Relaciona	Lesão por pressão		
09/11/2020	CE	SOBRAL	HOSPITAL REGIONA	-	Enviada	Eventos Adversos Relaciona	Lesão por pressão		
09/11/2020	CE	SOBRAL	HOSPITAL REGIONA	-	Enviada	Eventos Adversos Relaciona	Lesão por pressão		
09/11/2020	CE	SOBRAL	HOSPITAL REGIONA	-	Enviada	Eventos Adversos Relaciona	Lesão por pressão		
09/11/2020	CE	SOBRAL	HOSPITAL REGIONA	-	Enviada	Eventos Adversos Relaciona	Lesão por pressão		
09/11/2020	CE	SOBRAL	HOSPITAL REGIONA	-	Enviada	Eventos Adversos Relaciona	Lesão por pressão		
09/11/2020	CE	SOBRAL	HOSPITAL REGIONA	-	Enviada	Eventos Adversos Relaciona	Lesão por pressão		
09/11/2020	CE	SOBRAL	HOSPITAL REGIONA	-	Enviada	Eventos Adversos Relaciona	Lesão por pressão		
09/11/2020	CE	SOBRAL	HOSPITAL REGIONA	-	Enviada	Eventos Adversos Relaciona	Lesão por pressão		
09/11/2020	CE	SOBRAL	HOSPITAL REGIONA	-	Enviada	Eventos Adversos Relaciona	Lesão por pressão		
09/11/2020	CE	SOBRAL	HOSPITAL REGIONA	-	Enviada	Eventos Adversos Relaciona	Lesão por pressão		
09/11/2020	CE	SOBRAL	HOSPITAL REGIONA	-	Enviada	Eventos Adversos Relaciona	Lesão por pressão		
09/11/2020	CE	SOBRAL	HOSPITAL REGIONA	-	Enviada	Eventos Adversos Relaciona	Lesão por pressão		
09/11/2020	CE	SOBRAL	HOSPITAL REGIONA	-	Enviada	Eventos Adversos Relaciona	Lesão por pressão		
09/11/2020	CE	SOBRAL	HOSPITAL REGIONA	-	Enviada	Eventos Adversos Relaciona	Lesão por pressão		
09/11/2020	CE	SOBRAL	HOSPITAL REGIONA	-	Enviada	Eventos Adversos Relaciona	Lesão por pressão		

09/11/2020	CE	SOBRAL	HOSPITA L REGIONA	-	Enviada	Eventos Adversos Relaciona	Lesão por pressão		
09/11/2020	CE	SOBRAL	HOSPITA L REGIONA	-	Enviada	Eventos Adversos Relaciona	Lesão por pressão		
09/11/2020	CE	SOBRAL	HOSPITA L REGIONA	-	Enviada	Eventos Adversos Relaciona	Lesão por pressão		
09/11/2020	CE	SOBRAL	HOSPITA L REGIONA	-	Enviada	Eventos Adversos Relaciona	Lesão por pressão		
09/11/2020	CE	SOBRAL	HOSPITA L REGIONA	-	Enviada	Eventos Adversos Relaciona	Lesão por pressão		
09/11/2020	CE	SOBRAL	HOSPITA L REGIONA	-	Enviada	Eventos Adversos Relaciona	Lesão por pressão		
09/11/2020	CE	SOBRAL	HOSPITA L REGIONA	-	Enviada	Eventos Adversos Relaciona	Lesão por pressão		
09/11/2020	CE	SOBRAL	HOSPITA L REGIONA	-	Enviada	Eventos Adversos Relaciona	Lesão por pressão		
09/11/2020	CE	SOBRAL	HOSPITA L REGIONA	-	Enviada	Eventos Adversos Relaciona	Lesão por pressão		
09/11/2020	CE	SOBRAL	HOSPITA L REGIONA	-	Enviada	Eventos Adversos Relaciona	Lesão por pressão		
09/11/2020	CE	SOBRAL	HOSPITA L REGIONA	-	Enviada	Eventos Adversos Relaciona	Lesão por pressão		
09/11/2020	CE	SOBRAL	HOSPITA L REGIONA	-	Enviada	Eventos Adversos Relaciona	Lesão por pressão		
09/11/2020	CE	SOBRAL	HOSPITA L REGIONA	-	Enviada	Eventos Adversos Relaciona	Lesão por pressão		
09/11/2020	CE	SOBRAL	HOSPITA L REGIONA	-	Enviada	Eventos Adversos Relaciona	Lesão por pressão		
09/11/2020	CE	SOBRAL	HOSPITA L REGIONA	-	Enviada	Eventos Adversos Relaciona	Lesão por pressão		
09/11/2020	CE	SOBRAL	HOSPITA L REGIONA	-	Enviada	Eventos Adversos Relaciona	Lesão por pressão		
09/11/2020	CE	SOBRAL	HOSPITA L REGIONA	-	Enviada	Eventos Adversos Relaciona	Lesão por pressão		
09/11/2020	CE	SOBRAL	HOSPITA L REGIONA	-	Enviada	Eventos Adversos Relaciona	Lesão por pressão		
10/11/2020	CE	SOBRAL	HOSPITA L REGIONA	-	Enviada	Eventos Adversos Relaciona	Falhas na administra ção de		Enteral (oral, cateter

10/11/2020	CE	SOBRAL	HOSPITA L REGIONA	-	Enviada	Eventos Adversos Relaciona	Falhas na administra ção de		Enteral (oral, cateter
10/11/2020	CE	SOBRAL	HOSPITA L REGIONA	-	Enviada	Eventos Adversos Relaciona	Falhas na administra ção de		Enteral (oral, cateter
10/11/2020	CE	SOBRAL	HOSPITA L REGIONA	-	Enviada	Eventos Adversos Relaciona	Falhas na administra ção de		Enteral (oral, cateter
10/11/2020	CE	SOBRAL	HOSPITA L REGIONA	-	Enviada	Eventos Adversos Relaciona	Falhas na administra ção de		Enteral (oral, cateter
10/11/2020	CE	SOBRAL	HOSPITA L REGIONA	-	Enviada	Eventos Adversos Relaciona	Falhas na administra ção de		Enteral (oral, cateter
10/11/2020	CE	SOBRAL	HOSPITA L REGIONA	-	Enviada	Eventos Adversos Relaciona	Falhas na administra ção de		Enteral (oral, cateter
10/11/2020	CE	SOBRAL	HOSPITA L REGIONA	-	Enviada	Eventos Adversos Relaciona	Falhas na administra ção de		Enteral (oral, cateter
10/11/2020	CE	SOBRAL	HOSPITA L REGIONA	-	Enviada	Eventos Adversos Relaciona	Falhas na administra ção de		Enteral (oral, cateter
10/11/2020	CE	SOBRAL	HOSPITA L REGIONA	-	Enviada	Eventos Adversos Relaciona	Falhas na administra ção de		Enteral (oral, cateter
10/11/2020	CE	SOBRAL	HOSPITA L REGIONA	-	Enviada	Eventos Adversos Relaciona	Falhas na administra ção de		Enteral (oral, cateter
10/11/2020	CE	SOBRAL	HOSPITA L REGIONA	-	Enviada	Eventos Adversos Relaciona	Falhas na administra ção de		Enteral (oral, cateter
10/11/2020	CE	SOBRAL	HOSPITA L REGIONA	-	Enviada	Eventos Adversos Relaciona	Falhas na administra ção de		Enteral (oral, cateter
10/11/2020	CE	SOBRAL	HOSPITA L REGIONA	-	Enviada	Eventos Adversos Relaciona	Falhas na administra ção de		Enteral (oral, cateter
10/11/2020	CE	SOBRAL	HOSPITA L REGIONA	-	Enviada	Eventos Adversos Relaciona	Falha na identificaç ão do		
10/11/2020	CE	SOBRAL	HOSPITA L REGIONA	-	Enviada	Eventos Adversos Relaciona	Falha na identificaç ão do		
10/11/2020	CE	SOBRAL	HOSPITA L REGIONA	-	Enviada	Eventos Adversos Relaciona	Falha na identificaç ão do		
10/11/2020	CE	SOBRAL	HOSPITA L REGIONA	-	Enviada	Eventos Adversos Relaciona	Falha na identificaç ão do		
10/11/2020	CE	SOBRAL	HOSPITA L REGIONA	-	Enviada	Eventos Adversos Relaciona	Falhas ocorridas em		
10/11/2020	CE	SOBRAL	HOSPITA L REGIONA	-	Enviada	Eventos Adversos Relaciona	Falhas ocorridas em		

10/11/2020	CE	SOBRAL	HOSPITA L REGIONA	-	Enviada	Eventos Adversos Relaciona	Falhas ocorridas em		
10/11/2020	CE	SOBRAL	HOSPITA L REGIONA	-	Enviada	Eventos Adversos Relaciona	Falhas ocorridas em		
10/11/2020	CE	SOBRAL	HOSPITA L REGIONA	-	Enviada	Eventos Adversos Relaciona	Falhas ocorridas em		
10/11/2020	CE	SOBRAL	HOSPITA L REGIONA	-	Enviada	Eventos Adversos Relaciona	Falhas ocorridas em		
10/11/2020	CE	SOBRAL	HOSPITA L REGIONA	-	Enviada	Eventos Adversos Relaciona	Falhas ocorridas em		
10/11/2020	CE	SOBRAL	HOSPITA L REGIONA	-	Enviada	Eventos Adversos Relaciona	Falhas ocorridas em		
10/11/2020	CE	SOBRAL	HOSPITA L REGIONA	-	Enviada	Eventos Adversos Relaciona	Falhas ocorridas em		
10/11/2020	CE	SOBRAL	HOSPITA L REGIONA	-	Enviada	Eventos Adversos Relaciona	Falhas ocorridas em		
10/11/2020	CE	SOBRAL	HOSPITA L REGIONA	-	Enviada	Eventos Adversos Relaciona	Falhas ocorridas em		
10/11/2020	CE	SOBRAL	HOSPITA L REGIONA	-	Enviada	Eventos Adversos Relaciona	Falhas ocorridas em		
10/11/2020	CE	SOBRAL	HOSPITA L REGIONA	-	Enviada	Eventos Adversos Relaciona	Falhas ocorridas em		
11/11/2020	CE	SOBRAL	HOSPITA L REGIONA	-	Enviada	Eventos Adversos Relaciona	Lesão por pressão		
11/11/2020	CE	SOBRAL	HOSPITA L REGIONA	-	Enviada	Eventos Adversos Relaciona	Lesão por pressão		
11/11/2020	CE	SOBRAL	HOSPITA L REGIONA	-	Enviada	Eventos Adversos Relaciona	Lesão por pressão		
11/11/2020	CE	SOBRAL	HOSPITA L REGIONA	-	Enviada	Eventos Adversos Relaciona	Lesão por pressão		
11/11/2020	CE	SOBRAL	HOSPITA L REGIONA	-	Enviada	Eventos Adversos Relaciona	Lesão por pressão		
11/11/2020	CE	SOBRAL	HOSPITA L REGIONA	-	Enviada	Eventos Adversos Relaciona	Lesão por pressão		
11/11/2020	CE	SOBRAL	HOSPITA L REGIONA	-	Enviada	Eventos Adversos Relaciona	Lesão por pressão		
11/11/2020	CE	SOBRAL	HOSPITA L REGIONA	-	Enviada	Eventos Adversos Relaciona	Lesão por pressão		

11/11/2020	CE	SOBRAL	HOSPITA L REGIONA	-	Enviada	Eventos Adversos Relaciona	Lesão por pressão		
11/11/2020	CE	SOBRAL	HOSPITA L REGIONA	-	Enviada	Eventos Adversos Relaciona	Lesão por pressão		
11/11/2020	CE	SOBRAL	HOSPITA L REGIONA	-	Enviada	Eventos Adversos Relaciona	Lesão por pressão		
11/11/2020	CE	SOBRAL	HOSPITA L REGIONA	-	Enviada	Eventos Adversos Relaciona	Lesão por pressão		
11/11/2020	CE	SOBRAL	HOSPITA L REGIONA	-	Enviada	Eventos Adversos Relaciona	Lesão por pressão		
11/11/2020	CE	SOBRAL	HOSPITA L REGIONA	-	Enviada	Eventos Adversos Relaciona	Falhas durante a assistênci		
11/11/2020	CE	SOBRAL	HOSPITA L REGIONA	-	Enviada	Eventos Adversos Relaciona	Falhas durante a assistênci		
11/11/2020	CE	SOBRAL	HOSPITA L REGIONA	-	Enviada	Eventos Adversos Relaciona	Falhas durante a assistênci		
11/11/2020	CE	SOBRAL	HOSPITA L REGIONA	-	Enviada	Eventos Adversos Relaciona	Falhas nas atividades		
11/11/2020	CE	SOBRAL	HOSPITA L REGIONA	-	Enviada	Eventos Adversos Relaciona	Falhas nas atividades		
11/11/2020	CE	SOBRAL	HOSPITA L REGIONA	-	Enviada	Eventos Adversos Relaciona	Falhas nas atividades		
11/11/2020	CE	SOBRAL	HOSPITA L REGIONA	-	Enviada	Eventos Adversos Relaciona	Falhas durante a assistênci		
11/11/2020	CE	SOBRAL	HOSPITA L REGIONA	-	Enviada	Eventos Adversos Relaciona	Falhas durante a assistênci		
11/11/2020	CE	SOBRAL	HOSPITA L REGIONA	-	Enviada	Eventos Adversos Relaciona	Falhas durante a assistênci		
11/11/2020	CE	SOBRAL	HOSPITA L REGIONA	-	Enviada	Eventos Adversos Relaciona	Falhas durante a assistênci		
11/11/2020	CE	SOBRAL	HOSPITA L REGIONA	-	Enviada	Eventos Adversos Relaciona	Falhas durante a assistênci		
11/11/2020	CE	SOBRAL	HOSPITA L REGIONA	-	Enviada	Eventos Adversos Relaciona	Falhas durante a assistênci		
11/11/2020	CE	SOBRAL	HOSPITA L REGIONA	-	Enviada	Eventos Adversos Relaciona	Falhas durante a assistênci		
11/11/2020	CE	SOBRAL	HOSPITA L REGIONA	-	Enviada	Eventos Adversos Relaciona	Falhas durante a assistênci		

11/11/2020	CE	SOBRAL	HOSPITA L REGIONA	-	Enviada	Eventos Adversos Relaciona	Falhas durante a assistênci		
11/11/2020	CE	SOBRAL	HOSPITA L REGIONA	-	Enviada	Eventos Adversos Relaciona	Falhas durante a assistênci		
11/11/2020	CE	SOBRAL	HOSPITA L REGIONA	-	Enviada	Eventos Adversos Relaciona	Falhas durante a assistênci		
11/11/2020	CE	SOBRAL	HOSPITA L REGIONA	-	Enviada	Eventos Adversos Relaciona	Falhas durante a assistênci		
11/11/2020	CE	SOBRAL	HOSPITA L REGIONA	-	Enviada	Eventos Adversos Relaciona	Falhas envolvend o cateter		
11/11/2020	CE	SOBRAL	HOSPITA L REGIONA	-	Enviada	Eventos Adversos Relaciona	Falhas envolvend o cateter		
11/11/2020	CE	SOBRAL	HOSPITA L REGIONA	-	Enviada	Eventos Adversos Relaciona	Falhas envolvend o cateter		
11/11/2020	CE	SOBRAL	HOSPITA L REGIONA	-	Enviada	Eventos Adversos Relaciona	Falhas envolvend o cateter		
11/11/2020	CE	SOBRAL	HOSPITA L REGIONA	-	Enviada	Eventos Adversos Relaciona	Falhas envolvend o cateter		
11/11/2020	CE	SOBRAL	HOSPITA L REGIONA	-	Enviada	Eventos Adversos Relaciona	Falhas envolvend o cateter		
11/11/2020	CE	SOBRAL	HOSPITA L REGIONA	-	Enviada	Eventos Adversos Relaciona	Falhas envolvend o cateter		
11/11/2020	CE	SOBRAL	HOSPITA L REGIONA	-	Enviada	Eventos Adversos Relaciona	Falhas envolvend o cateter		
11/11/2020	CE	SOBRAL	HOSPITA L REGIONA	-	Enviada	Eventos Adversos Relaciona	Falhas envolvend o cateter		
11/11/2020	CE	SOBRAL	HOSPITA L REGIONA	-	Enviada	Eventos Adversos Relaciona	Falhas envolvend o cateter		
11/11/2020	CE	SOBRAL	HOSPITA L REGIONA	-	Enviada	Eventos Adversos Relaciona	Falhas envolvend o cateter		
11/11/2020	CE	SOBRAL	HOSPITA L REGIONA	-	Enviada	Eventos Adversos Relaciona	Falhas envolvend o cateter		
11/11/2020	CE	SOBRAL	HOSPITA L REGIONA	-	Enviada	Eventos Adversos Relaciona	Falhas envolvend o cateter		
11/11/2020	CE	SOBRAL	HOSPITA L REGIONA	-	Enviada	Eventos Adversos Relaciona	Falhas envolvend o cateter		
11/11/2020	CE	SOBRAL	HOSPITA L REGIONA	-	Enviada	Eventos Adversos Relaciona	Falhas envolvend o cateter		

11/11/2020	CE	SOBRAL	HOSPITA L REGIONA	-	Enviada	Eventos Adversos Relaciona	Falhas envolvend o cateter		
11/11/2020	CE	SOBRAL	HOSPITA L REGIONA	-	Enviada	Eventos Adversos Relaciona	Falhas envolvend o cateter		
11/11/2020	CE	SOBRAL	HOSPITA L REGIONA	-	Enviada	Eventos Adversos Relaciona	Falhas envolvend o cateter		
11/11/2020	CE	SOBRAL	HOSPITA L REGIONA	-	Enviada	Eventos Adversos Relaciona	Falhas envolvend o cateter		
11/11/2020	CE	SOBRAL	HOSPITA L REGIONA	-	Enviada	Eventos Adversos Relaciona	Queda do paciente		
11/11/2020	CE	SOBRAL	HOSPITA L REGIONA	-	Enviada	Eventos Adversos Relaciona	Queda do paciente		
11/11/2020	CE	SOBRAL	HOSPITA L REGIONA	-	Enviada	Eventos Adversos Relaciona	Queda do paciente		
11/11/2020	CE	SOBRAL	HOSPITA L REGIONA	-	Enviada	Eventos Adversos Relaciona	Falhas ocorridas em		
11/11/2020	CE	SOBRAL	HOSPITA L REGIONA	-	Enviada	Eventos Adversos Relaciona	Queda do paciente		
11/11/2020	CE	SOBRAL	HOSPITA L REGIONA	-	Enviada	Eventos Adversos Relaciona	Falhas ocorridas em		
11/11/2020	CE	SOBRAL	HOSPITA L REGIONA	-	Enviada	Eventos Adversos Relaciona	Queda do paciente		
11/11/2020	CE	SOBRAL	HOSPITA L REGIONA	-	Enviada	Eventos Adversos Relaciona	Falhas ocorridas em		
11/11/2020	CE	SOBRAL	HOSPITA L REGIONA	-	Enviada	Eventos Adversos Relaciona	Queda do paciente		
11/11/2020	CE	SOBRAL	HOSPITA L REGIONA	-	Enviada	Eventos Adversos Relaciona	Falhas ocorridas em		
11/11/2020	CE	SOBRAL	HOSPITA L REGIONA	-	Enviada	Eventos Adversos Relaciona	Queda do paciente		
11/11/2020	CE	SOBRAL	HOSPITA L REGIONA	-	Enviada	Eventos Adversos Relaciona	Queda do paciente		
11/11/2020	CE	SOBRAL	HOSPITA L REGIONA	-	Enviada	Eventos Adversos Relaciona	Falhas ocorridas em		
11/11/2020	CE	SOBRAL	HOSPITA L REGIONA	-	Enviada	Eventos Adversos Relaciona	Falhas ocorridas em		
11/11/2020	CE	SOBRAL	HOSPITA L REGIONA	-	Enviada	Eventos Adversos Relaciona	Queda do paciente		

11/11/2020	CE	SOBRAL	HOSPITA L REGIONA	-	Enviada	Eventos Adversos Relaciona	Falhas ocorridas em		
11/11/2020	CE	SOBRAL	HOSPITA L REGIONA	-	Enviada	Eventos Adversos Relaciona	Falhas ocorridas em		
11/11/2020	CE	SOBRAL	HOSPITA L REGIONA	-	Enviada	Eventos Adversos Relaciona	Queda do paciente		
11/11/2020	CE	SOBRAL	HOSPITA L REGIONA	-	Enviada	Eventos Adversos Relaciona	Falhas ocorridas em		
11/11/2020	CE	SOBRAL	HOSPITA L REGIONA	-	Enviada	Eventos Adversos Relaciona	Queda do paciente		
11/11/2020	CE	SOBRAL	HOSPITA L REGIONA	-	Enviada	Eventos Adversos Relaciona	Falhas nas atividades		
11/11/2020	CE	SOBRAL	HOSPITA L REGIONA	-	Enviada	Eventos Adversos Relaciona	Falha na document ação		
11/11/2020	CE	SOBRAL	HOSPITA L REGIONA	-	Enviada	Eventos Adversos Relaciona	Queda do paciente		
11/11/2020	CE	SOBRAL	HOSPITA L REGIONA	-	Enviada	Eventos Adversos Relaciona	Queda do paciente		
11/11/2020	CE	SOBRAL	HOSPITA L REGIONA	-	Enviada	Eventos Adversos Relaciona	Falhas nas atividades		
11/11/2020	CE	SOBRAL	HOSPITA L REGIONA	-	Enviada	Eventos Adversos Relaciona	Queda do paciente		
11/11/2020	CE	SOBRAL	HOSPITA L REGIONA	-	Enviada	Eventos Adversos Relaciona	Falhas nas atividades		
11/11/2020	CE	SOBRAL	HOSPITA L REGIONA	-	Enviada	Eventos Adversos Relaciona	Falhas nas atividades		
11/11/2020	CE	SOBRAL	HOSPITA L REGIONA	-	Enviada	Eventos Adversos Relaciona	Falhas nas atividades		
11/11/2020	CE	SOBRAL	HOSPITA L REGIONA	-	Enviada	Eventos Adversos Relaciona	Queda do paciente		
11/11/2020	CE	SOBRAL	HOSPITA L REGIONA	-	Enviada	Eventos Adversos Relaciona	Falhas durante a assistênci		
11/11/2020	CE	SOBRAL	HOSPITA L REGIONA	-	Enviada	Eventos Adversos Relaciona	Falhas nas atividades		
11/11/2020	CE	SOBRAL	HOSPITA L REGIONA	-	Enviada	Eventos Adversos Relaciona	Falhas nas atividades		
11/11/2020	CE	SOBRAL	HOSPITA L REGIONA	-	Enviada	Eventos Adversos Relaciona	Queda do paciente		

11/11/2020	CE	SOBRAL	HOSPITAL REGIONA	-	Enviada	Eventos Adversos Relaciona	Falhas durante a assistênci		
11/11/2020	CE	SOBRAL	HOSPITAL REGIONA	-	Enviada	Eventos Adversos Relaciona	Queda do paciente		
11/11/2020	CE	SOBRAL	HOSPITAL REGIONA	-	Enviada	Eventos Adversos Relaciona	Falhas durante a assistênci		
11/11/2020	CE	SOBRAL	HOSPITAL REGIONA	-	Enviada	Eventos Adversos Relaciona	Falhas ocorridas em		
11/11/2020	CE	SOBRAL	HOSPITAL REGIONA	-	Enviada	Eventos Adversos Relaciona	Queda do paciente		
11/11/2020	CE	SOBRAL	HOSPITAL REGIONA	-	Enviada	Eventos Adversos Relaciona	Falhas durante a assistênci		
11/11/2020	CE	SOBRAL	HOSPITAL REGIONA	-	Enviada	Eventos Adversos Relaciona	Queda do paciente		
11/11/2020	CE	SOBRAL	HOSPITAL REGIONA	-	Enviada	Eventos Adversos Relaciona	Falhas durante a assistênci		
11/11/2020	CE	SOBRAL	HOSPITAL REGIONA	-	Enviada	Eventos Adversos Relaciona	Queda do paciente		
12/11/2020	CE	SOBRAL	HOSPITAL REGIONA	-	Enviada	Eventos Adversos Relaciona	Queda do paciente		
12/11/2020	CE	SOBRAL	HOSPITAL REGIONA	-	Enviada	Eventos Adversos Relaciona	Queda do paciente		
12/11/2020	CE	SOBRAL	HOSPITAL REGIONA	-	Enviada	Eventos Adversos Relaciona	Queda do paciente		
12/11/2020	CE	SOBRAL	HOSPITAL REGIONA	-	Enviada	Eventos Adversos Relaciona	Queda do paciente		
12/11/2020	CE	SOBRAL	HOSPITAL REGIONA	-	Enviada	Eventos Adversos Relaciona	Queda do paciente		
12/11/2020	CE	SOBRAL	HOSPITAL REGIONA	-	Enviada	Eventos Adversos Relaciona	Queda do paciente		
12/11/2020	CE	SOBRAL	HOSPITAL REGIONA	-	Enviada	Eventos Adversos Relaciona	Queda do paciente		
12/11/2020	CE	SOBRAL	HOSPITAL REGIONA	-	Enviada	Eventos Adversos Relaciona	Queda do paciente		
12/11/2020	CE	SOBRAL	HOSPITAL REGIONA	-	Enviada	Eventos Adversos Relaciona	Falhas durante a assistênci		
12/11/2020	CE	SOBRAL	HOSPITAL REGIONA	-	Enviada	Eventos Adversos Relaciona	Falhas durante a assistênci		

12/11/2020	CE	SOBRAL	HOSPITA L REGIONA	-	Enviada	Eventos Adversos Relaciona	Falhas durante a assistênci		
12/11/2020	CE	SOBRAL	HOSPITA L REGIONA	-	Enviada	Eventos Adversos Relaciona	Falhas durante a assistênci		
12/11/2020	CE	SOBRAL	HOSPITA L REGIONA	-	Enviada	Eventos Adversos Relaciona	Falhas durante a assistênci		
12/11/2020	CE	SOBRAL	HOSPITA L REGIONA	-	Enviada	Eventos Adversos Relaciona	Falhas durante a assistênci		
12/11/2020	CE	SOBRAL	HOSPITA L REGIONA	-	Enviada	Eventos Adversos Relaciona	Falhas durante a assistênci		
12/11/2020	CE	SOBRAL	HOSPITA L REGIONA	-	Enviada	Eventos Adversos Relaciona	Falhas durante a assistênci		
12/11/2020	CE	SOBRAL	HOSPITA L REGIONA	-	Enviada	Eventos Adversos Relaciona	Falhas durante a assistênci		
12/11/2020	CE	SOBRAL	HOSPITA L REGIONA	-	Enviada	Eventos Adversos Relaciona	Queda do paciente		
12/11/2020	CE	SOBRAL	HOSPITA L REGIONA	-	Enviada	Eventos Adversos Relaciona	Falhas durante a assistênci		
12/11/2020	CE	SOBRAL	HOSPITA L REGIONA	-	Enviada	Eventos Adversos Relaciona	Falhas durante a assistênci		
12/11/2020	CE	SOBRAL	HOSPITA L REGIONA	-	Enviada	Eventos Adversos Relaciona	Queda do paciente		
12/11/2020	CE	SOBRAL	HOSPITA L REGIONA	-	Enviada	Eventos Adversos Relaciona	Queda do paciente		
12/11/2020	CE	SOBRAL	HOSPITA L REGIONA	-	Enviada	Eventos Adversos Relaciona	Queda do paciente		
12/11/2020	CE	SOBRAL	HOSPITA L REGIONA	-	Enviada	Eventos Adversos Relaciona	Queda do paciente		
12/11/2020	CE	SOBRAL	HOSPITA L REGIONA	-	Enviada	Eventos Adversos Relaciona	Queda do paciente		
12/11/2020	CE	SOBRAL	HOSPITA L REGIONA	-	Enviada	Eventos Adversos Relaciona	Queda do paciente		
12/11/2020	CE	SOBRAL	HOSPITA L REGIONA	-	Enviada	Eventos Adversos Relaciona	Queda do paciente		
12/11/2020	CE	SOBRAL	HOSPITA L REGIONA	-	Enviada	Eventos Adversos Relaciona	Queda do paciente		
12/11/2020	CE	SOBRAL	HOSPITA L REGIONA	-	Enviada	Eventos Adversos Relaciona	Queda do paciente		

12/11/2020	CE	SOBRAL	HOSPITA L REGIONA	-	Enviada	Eventos Adversos Relaciona	Queda do paciente		
12/11/2020	CE	SOBRAL	HOSPITA L REGIONA	-	Enviada	Eventos Adversos Relaciona	Falhas durante a assistênci		
12/11/2020	CE	SOBRAL	HOSPITA L REGIONA	-	Enviada	Eventos Adversos Relaciona	Falhas na administra ção de		Enteral (oral, cateter
12/11/2020	CE	SOBRAL	HOSPITA L REGIONA	-	Enviada	Eventos Adversos Relaciona	Falhas na administra ção de		Enteral (oral, cateter
12/11/2020	CE	SOBRAL	HOSPITA L REGIONA	-	Enviada	Eventos Adversos Relaciona	Falhas durante a assistênci		
12/11/2020	CE	SOBRAL	HOSPITA L REGIONA	-	Enviada	Eventos Adversos Relaciona	Falhas durante a assistênci		
12/11/2020	CE	SOBRAL	HOSPITA L REGIONA	-	Enviada	Eventos Adversos Relaciona	Falhas na administra ção de		Enteral (oral, cateter
12/11/2020	CE	SOBRAL	HOSPITA L REGIONA	-	Enviada	Eventos Adversos Relaciona	Falhas durante a assistênci		
12/11/2020	CE	SOBRAL	HOSPITA L REGIONA	-	Enviada	Eventos Adversos Relaciona	Falhas na administra ção de		Enteral (oral, cateter
13/11/2020	CE	SOBRAL	HOSPITA L REGIONA	-	Enviada	Eventos Adversos Relaciona	Falhas na administra ção de		Enteral (oral, cateter
13/11/2020	CE	SOBRAL	HOSPITA L REGIONA	-	Enviada	Eventos Adversos Relaciona	Falhas na administra ção de		Enteral (oral, cateter
13/11/2020	CE	SOBRAL	HOSPITA L REGIONA	-	Enviada	Eventos Adversos Relaciona	Falhas na administra ção de		Enteral (oral, cateter
13/11/2020	CE	SOBRAL	HOSPITA L REGIONA	-	Enviada	Eventos Adversos Relaciona	Falhas na administra ção de		Enteral (oral, cateter
13/11/2020	CE	SOBRAL	HOSPITA L REGIONA	-	Enviada	Eventos Adversos Relaciona	Falhas na administra ção de		Enteral (oral, cateter
13/11/2020	CE	SOBRAL	HOSPITA L REGIONA	-	Enviada	Eventos Adversos Relaciona	Falhas na administra ção de		Enteral (oral, cateter
13/11/2020	CE	SOBRAL	HOSPITA L REGIONA	-	Enviada	Eventos Adversos Relaciona	Falhas na administra ção de		Enteral (oral, cateter
13/11/2020	CE	SOBRAL	HOSPITA L REGIONA	-	Enviada	Eventos Adversos Relaciona	Falhas na administra ção de		Enteral (oral, cateter
13/11/2020	CE	SOBRAL	HOSPITA L REGIONA	-	Enviada	Eventos Adversos Relaciona	Falhas na administra ção de		Enteral (oral, cateter
13/11/2020	CE	SOBRAL	HOSPITA L REGIONA	-	Enviada	Eventos Adversos Relaciona	Falhas na administra ção de		Enteral (oral, cateter

13/11/2020	CE	SOBRAL	HOSPITA L REGIONA	-	Enviada	Eventos Adversos Relaciona	Falhas na administra ção de		Enteral (oral, cateter
13/11/2020	CE	SOBRAL	HOSPITA L REGIONA	-	Enviada	Eventos Adversos Relaciona	Falhas na administra ção de		Enteral (oral, cateter
13/11/2020	CE	SOBRAL	HOSPITA L REGIONA	-	Enviada	Eventos Adversos Relaciona	Falhas na administra ção de		Enteral (oral, cateter
13/11/2020	CE	SOBRAL	HOSPITA L REGIONA	-	Enviada	Eventos Adversos Relaciona	Falhas na administra ção de		Enteral (oral, cateter
13/11/2020	CE	SOBRAL	HOSPITA L REGIONA	-	Enviada	Eventos Adversos Relaciona	Falhas na administra ção de		Enteral (oral, cateter
13/11/2020	CE	SOBRAL	HOSPITA L REGIONA	-	Enviada	Eventos Adversos Relaciona	Falhas na administra ção de		Enteral (oral, cateter
13/11/2020	CE	SOBRAL	HOSPITA L REGIONA	-	Enviada	Eventos Adversos Relaciona	Falhas na administra ção de		Enteral (oral, cateter
13/11/2020	CE	SOBRAL	HOSPITA L REGIONA	-	Enviada	Eventos Adversos Relaciona	Falhas na administra ção de		Enteral (oral, cateter
16/11/2020	CE	SOBRAL	HOSPITA L REGIONA	-	Enviada	Eventos Adversos Relaciona	Falhas na administra ção de		Enteral (oral, cateter
16/11/2020	CE	SOBRAL	HOSPITA L REGIONA	-	Enviada	Eventos Adversos Relaciona	Falhas na administra ção de		Enteral (oral, cateter
16/11/2020	CE	SOBRAL	HOSPITA L REGIONA	-	Enviada	Eventos Adversos Relaciona	Falhas na administra ção de		Enteral (oral, cateter
16/11/2020	CE	SOBRAL	HOSPITA L REGIONA	-	Enviada	Eventos Adversos Relaciona	Falha na identificaç ão do		
16/11/2020	CE	SOBRAL	HOSPITA L REGIONA	-	Enviada	Eventos Adversos Relaciona	Falhas na administra ção de		Enteral (oral, cateter
16/11/2020	CE	SOBRAL	HOSPITA L REGIONA	-	Enviada	Eventos Adversos Relaciona	Falhas na administra ção de		Enteral (oral, cateter
16/11/2020	CE	SOBRAL	HOSPITA L REGIONA	-	Enviada	Eventos Adversos Relaciona	Falhas na administra ção de		Enteral (oral, cateter
16/11/2020	CE	SOBRAL	HOSPITA L REGIONA	-	Enviada	Eventos Adversos Relaciona	Falhas na administra ção de		Enteral (oral, cateter
16/11/2020	CE	SOBRAL	HOSPITA L REGIONA	-	Enviada	Eventos Adversos Relaciona	Falhas na administra ção de		Enteral (oral, cateter
16/11/2020	CE	SOBRAL	HOSPITA L REGIONA	-	Enviada	Eventos Adversos Relaciona	Falhas na administra ção de		Enteral (oral, cateter
16/11/2020	CE	SOBRAL	HOSPITA L REGIONA	-	Enviada	Eventos Adversos Relaciona	Falhas na administra ção de		Enteral (oral, cateter

19/11/2020	CE	SOBRAL	HOSPITA L REGIONA	-	Enviada	Eventos Adversos Relaciona	Falhas nas atividades		
19/11/2020	CE	SOBRAL	HOSPITA L REGIONA	-	Enviada	Eventos Adversos Relaciona	Falhas nas atividades		
19/11/2020	CE	SOBRAL	HOSPITA L REGIONA	-	Enviada	Eventos Adversos Relaciona	Falhas nas atividades		
19/11/2020	CE	SOBRAL	HOSPITA L REGIONA	-	Enviada	Eventos Adversos Relaciona	Falhas nas atividades		
19/11/2020	CE	SOBRAL	HOSPITA L REGIONA	-	Enviada	Eventos Adversos Relaciona	Falhas nas atividades		
19/11/2020	CE	SOBRAL	HOSPITA L REGIONA	-	Enviada	Eventos Adversos Relaciona	Falhas nas atividades		
19/11/2020	CE	SOBRAL	HOSPITA L REGIONA	-	Enviada	Eventos Adversos Relaciona	Falhas nas atividades		
19/11/2020	CE	SOBRAL	HOSPITA L REGIONA	-	Enviada	Eventos Adversos Relaciona	Falhas nas atividades		
19/11/2020	CE	SOBRAL	HOSPITA L REGIONA	-	Enviada	Eventos Adversos Relaciona	Falhas nas atividades		
20/11/2020	CE	SOBRAL	HOSPITA L REGIONA	-	Enviada	Eventos Adversos Relaciona	Falhas nas atividades		
20/11/2020	CE	SOBRAL	HOSPITA L REGIONA	-	Enviada	Eventos Adversos Relaciona	Falhas nas atividades		
20/11/2020	CE	SOBRAL	HOSPITA L REGIONA	-	Enviada	Eventos Adversos Relaciona	Falhas nas atividades		
20/11/2020	CE	SOBRAL	HOSPITA L REGIONA	-	Enviada	Eventos Adversos Relaciona	Falhas nas atividades		
20/11/2020	CE	SOBRAL	HOSPITA L REGIONA	-	Enviada	Eventos Adversos Relaciona	Falhas nas atividades		
20/11/2020	CE	SOBRAL	HOSPITA L REGIONA	-	Enviada	Eventos Adversos Relaciona	Falhas nas atividades		
20/11/2020	CE	SOBRAL	HOSPITA L REGIONA	-	Enviada	Eventos Adversos Relaciona	Falhas nas atividades		
20/11/2020	CE	SOBRAL	HOSPITA L REGIONA	-	Enviada	Eventos Adversos Relaciona	Falhas nas atividades		
20/11/2020	CE	SOBRAL	HOSPITA L REGIONA	-	Enviada	Eventos Adversos Relaciona	Falhas nas atividades		
20/11/2020	CE	SOBRAL	HOSPITA L REGIONA	-	Enviada	Eventos Adversos Relaciona	Falhas nas atividades		

20/11/2020	CE	SOBRAL	HOSPITA L REGIONA	-	Enviada	Eventos Adversos Relaciona	Falhas nas atividades		
20/11/2020	CE	SOBRAL	HOSPITA L REGIONA	-	Enviada	Eventos Adversos Relaciona	Falhas nas atividades		
20/11/2020	CE	SOBRAL	HOSPITA L REGIONA	-	Enviada	Eventos Adversos Relaciona	Falhas nas atividades		
20/11/2020	CE	SOBRAL	HOSPITA L REGIONA	-	Enviada	Eventos Adversos Relaciona	Falhas nas atividades		
20/11/2020	CE	SOBRAL	HOSPITA L REGIONA	-	Enviada	Eventos Adversos Relaciona	Falhas nas atividades		
20/11/2020	CE	SOBRAL	HOSPITA L REGIONA	-	Enviada	Eventos Adversos Relaciona	Falhas nas atividades		
20/11/2020	CE	SOBRAL	HOSPITA L REGIONA	-	Enviada	Eventos Adversos Relaciona	Falhas nas atividades		
20/11/2020	CE	SOBRAL	HOSPITA L REGIONA	-	Enviada	Eventos Adversos Relaciona	Falhas nas atividades		
20/11/2020	CE	SOBRAL	HOSPITA L REGIONA	-	Enviada	Eventos Adversos Relaciona	Falhas nas atividades		
20/11/2020	CE	SOBRAL	HOSPITA L REGIONA	-	Enviada	Eventos Adversos Relaciona	Falhas ocorridas em		
20/11/2020	CE	SOBRAL	HOSPITA L REGIONA	-	Enviada	Eventos Adversos Relaciona	Falhas nas atividades		
20/11/2020	CE	SOBRAL	HOSPITA L REGIONA	-	Enviada	Eventos Adversos Relaciona	Falhas nas atividades		
20/11/2020	CE	SOBRAL	HOSPITA L REGIONA	-	Enviada	Eventos Adversos Relaciona	Falhas durante a assistênci		
20/11/2020	CE	SOBRAL	HOSPITA L REGIONA	-	Enviada	Eventos Adversos Relaciona	Falhas durante a assistênci		
20/11/2020	CE	SOBRAL	HOSPITA L REGIONA	-	Enviada	Eventos Adversos Relaciona	Falhas nas atividades		
30/11/2020	CE	SOBRAL	HOSPITA L REGIONA	-	Enviada	Eventos Adversos Relaciona	Lesão por pressão		
30/11/2020	CE	SOBRAL	HOSPITA L REGIONA	-	Enviada	Eventos Adversos Relaciona	Lesão por pressão		
30/11/2020	CE	SOBRAL	HOSPITA L REGIONA	-	Enviada	Eventos Adversos Relaciona	Lesão por pressão		
30/11/2020	CE	SOBRAL	HOSPITA L REGIONA	-	Enviada	Eventos Adversos Relaciona	Lesão por pressão		

30/11/2020	CE	SOBRAL	HOSPITA L REGIONA	-	Enviada	Eventos Adversos Relaciona	Lesão por pressão		
30/11/2020	CE	SOBRAL	HOSPITA L REGIONA	-	Enviada	Eventos Adversos Relaciona	Lesão por pressão		
30/11/2020	CE	SOBRAL	HOSPITA L REGIONA	-	Enviada	Eventos Adversos Relaciona	Lesão por pressão		
30/11/2020	CE	SOBRAL	HOSPITA L REGIONA	-	Enviada	Eventos Adversos Relaciona	Lesão por pressão		
30/11/2020	CE	SOBRAL	HOSPITA L REGIONA	-	Enviada	Eventos Adversos Relaciona	Lesão por pressão		
30/11/2020	CE	SOBRAL	HOSPITA L REGIONA	-	Enviada	Eventos Adversos Relaciona	Lesão por pressão		
30/11/2020	CE	SOBRAL	HOSPITA L REGIONA	-	Enviada	Eventos Adversos Relaciona	Lesão por pressão		
30/11/2020	CE	SOBRAL	HOSPITA L REGIONA	-	Enviada	Eventos Adversos Relaciona	Lesão por pressão		
30/11/2020	CE	SOBRAL	HOSPITA L REGIONA	-	Enviada	Eventos Adversos Relaciona	Lesão por pressão		
30/11/2020	CE	SOBRAL	HOSPITA L REGIONA	-	Enviada	Eventos Adversos Relaciona	Lesão por pressão		
30/11/2020	CE	SOBRAL	HOSPITA L REGIONA	-	Enviada	Eventos Adversos Relaciona	Lesão por pressão		
30/11/2020	CE	SOBRAL	HOSPITA L REGIONA	-	Enviada	Eventos Adversos Relaciona	Lesão por pressão		
30/11/2020	CE	SOBRAL	HOSPITA L REGIONA	-	Enviada	Eventos Adversos Relaciona	Lesão por pressão		
30/11/2020	CE	SOBRAL	HOSPITA L REGIONA	-	Enviada	Eventos Adversos Relaciona	Lesão por pressão		
30/11/2020	CE	SOBRAL	HOSPITA L REGIONA	-	Enviada	Eventos Adversos Relaciona	Lesão por pressão		
30/11/2020	CE	SOBRAL	HOSPITA L REGIONA	-	Enviada	Eventos Adversos Relaciona	Lesão por pressão		
30/11/2020	CE	SOBRAL	HOSPITA L REGIONA	-	Enviada	Eventos Adversos Relaciona	Lesão por pressão		
30/11/2020	CE	SOBRAL	HOSPITA L REGIONA	-	Enviada	Eventos Adversos Relaciona	Lesão por pressão		
30/11/2020	CE	SOBRAL	HOSPITA L REGIONA	-	Enviada	Eventos Adversos Relaciona	Lesão por pressão		

30/11/2020	CE	SOBRAL	HOSPITA L REGIONA	-	Enviada	Eventos Adversos Relaciona	Lesão por pressão		
30/11/2020	CE	SOBRAL	HOSPITA L REGIONA	-	Enviada	Eventos Adversos Relaciona	Lesão por pressão		
30/11/2020	CE	SOBRAL	HOSPITA L REGIONA	-	Enviada	Eventos Adversos Relaciona	Lesão por pressão		
30/11/2020	CE	SOBRAL	HOSPITA L REGIONA	-	Enviada	Eventos Adversos Relaciona	Lesão por pressão		
30/11/2020	CE	SOBRAL	HOSPITA L REGIONA	-	Enviada	Eventos Adversos Relaciona	Lesão por pressão		
30/11/2020	CE	SOBRAL	HOSPITA L REGIONA	-	Enviada	Eventos Adversos Relaciona	Lesão por pressão		
30/11/2020	CE	SOBRAL	HOSPITA L REGIONA	-	Enviada	Eventos Adversos Relaciona	Lesão por pressão		
30/11/2020	CE	SOBRAL	HOSPITA L REGIONA	-	Enviada	Eventos Adversos Relaciona	Lesão por pressão		
30/11/2020	CE	SOBRAL	HOSPITA L REGIONA	-	Enviada	Eventos Adversos Relaciona	Lesão por pressão		
30/11/2020	CE	SOBRAL	HOSPITA L REGIONA	-	Enviada	Eventos Adversos Relaciona	Lesão por pressão		
30/11/2020	CE	SOBRAL	HOSPITA L REGIONA	-	Enviada	Eventos Adversos Relaciona	Lesão por pressão		
30/11/2020	CE	SOBRAL	HOSPITA L REGIONA	-	Enviada	Eventos Adversos Relaciona	Lesão por pressão		
30/11/2020	CE	SOBRAL	HOSPITA L REGIONA	-	Enviada	Eventos Adversos Relaciona	Lesão por pressão		
30/11/2020	CE	SOBRAL	HOSPITA L REGIONA	-	Enviada	Eventos Adversos Relaciona	Lesão por pressão		
30/11/2020	CE	SOBRAL	HOSPITA L REGIONA	-	Enviada	Eventos Adversos Relaciona	Lesão por pressão		
30/11/2020	CE	SOBRAL	HOSPITA L REGIONA	-	Enviada	Eventos Adversos Relaciona	Lesão por pressão		
01/12/2020	CE	SOBRAL	HOSPITA L REGIONA	-	Enviada	Eventos Adversos Relaciona	Lesão por pressão		
01/12/2020	CE	SOBRAL	HOSPITA L REGIONA	-	Enviada	Eventos Adversos Relaciona	Lesão por pressão		
01/12/2020	CE	SOBRAL	HOSPITA L REGIONA	-	Enviada	Eventos Adversos Relaciona	Lesão por pressão		

01/12/2020	CE	SOBRAL	HOSPITA L REGIONA	-	Enviada	Eventos Adversos Relaciona	Lesão por pressão		
01/12/2020	CE	SOBRAL	HOSPITA L REGIONA	-	Enviada	Eventos Adversos Relaciona	Lesão por pressão		
01/12/2020	CE	SOBRAL	HOSPITA L REGIONA	-	Enviada	Eventos Adversos Relaciona	Falhas nas atividades		
01/12/2020	CE	SOBRAL	HOSPITA L REGIONA	-	Enviada	Eventos Adversos Relaciona	Falhas durante a assistênci		
01/12/2020	CE	SOBRAL	HOSPITA L REGIONA	-	Enviada	Eventos Adversos Relaciona	Falhas envolvend o cateter		
01/12/2020	CE	SOBRAL	HOSPITA L REGIONA	-	Enviada	Eventos Adversos Relaciona	Falhas envolvend o cateter		
04/12/2020	CE	SOBRAL	HOSPITA L REGIONA	-	Enviada	Eventos Adversos Relaciona	Falhas envolvend o cateter		
04/12/2020	CE	SOBRAL	HOSPITA L REGIONA	-	Enviada	Eventos Adversos Relaciona	Falhas envolvend o cateter		
04/12/2020	CE	SOBRAL	HOSPITA L REGIONA	-	Enviada	Eventos Adversos Relaciona	Falhas envolvend o cateter		
04/12/2020	CE	SOBRAL	HOSPITA L REGIONA	-	Enviada	Eventos Adversos Relaciona	Falhas envolvend o cateter		
04/12/2020	CE	SOBRAL	HOSPITA L REGIONA	-	Enviada	Eventos Adversos Relaciona	Falhas envolvend o cateter		
04/12/2020	CE	SOBRAL	HOSPITA L REGIONA	-	Enviada	Eventos Adversos Relaciona	Falhas envolvend o cateter		
04/12/2020	CE	SOBRAL	HOSPITA L REGIONA	-	Enviada	Eventos Adversos Relaciona	Falhas envolvend o cateter		
04/12/2020	CE	SOBRAL	HOSPITA L REGIONA	-	Enviada	Eventos Adversos Relaciona	Falhas envolvend o cateter		
04/12/2020	CE	SOBRAL	HOSPITA L REGIONA	-	Enviada	Eventos Adversos Relaciona	Falhas envolvend o cateter		
04/12/2020	CE	SOBRAL	HOSPITA L REGIONA	-	Enviada	Eventos Adversos Relaciona	Falhas envolvend o cateter		
04/12/2020	CE	SOBRAL	HOSPITA L REGIONA	-	Enviada	Eventos Adversos Relaciona	Falhas durante a assistênci		
04/12/2020	CE	SOBRAL	HOSPITA L REGIONA	-	Enviada	Eventos Adversos Relaciona	Falhas nas atividades		
04/12/2020	CE	SOBRAL	HOSPITA L REGIONA	-	Enviada	Eventos Adversos Relaciona	Falhas envolvend o cateter		

04/12/2020	CE	SOBRAL	HOSPITA L REGIONA	-	Enviada	Eventos Adversos Relaciona	Falhas envolvend o cateter		
04/12/2020	CE	SOBRAL	HOSPITA L REGIONA	-	Enviada	Eventos Adversos Relaciona	Falhas envolvend o cateter		
04/12/2020	CE	SOBRAL	HOSPITA L REGIONA	-	Enviada	Eventos Adversos Relaciona	Falhas envolvend o cateter		
04/12/2020	CE	SOBRAL	HOSPITA L REGIONA	-	Enviada	Eventos Adversos Relaciona	Queda do paciente		
04/12/2020	CE	SOBRAL	HOSPITA L REGIONA	-	Enviada	Eventos Adversos Relaciona	Queda do paciente		
07/12/2020	CE	SOBRAL	HOSPITA L REGIONA	-	Enviada	Eventos Adversos Relaciona	Queda do paciente		
07/12/2020	CE	SOBRAL	HOSPITA L REGIONA	-	Enviada	Eventos Adversos Relaciona	Queda do paciente		
07/12/2020	CE	SOBRAL	HOSPITA L REGIONA	-	Enviada	Eventos Adversos Relaciona	Queda do paciente		
07/12/2020	CE	SOBRAL	HOSPITA L REGIONA	-	Enviada	Eventos Adversos Relaciona	Queda do paciente		
07/12/2020	CE	SOBRAL	HOSPITA L REGIONA	-	Enviada	Eventos Adversos Relaciona	Queda do paciente		
07/12/2020	CE	SOBRAL	HOSPITA L REGIONA	-	Enviada	Eventos Adversos Relaciona	Queda do paciente		
07/12/2020	CE	SOBRAL	HOSPITA L REGIONA	-	Enviada	Eventos Adversos Relaciona	Falhas durante a assistênci		
07/12/2020	CE	SOBRAL	HOSPITA L REGIONA	-	Enviada	Eventos Adversos Relaciona	Falhas na administra ção de		Enteral (oral, cateter
07/12/2020	CE	SOBRAL	HOSPITA L REGIONA	-	Enviada	Eventos Adversos Relaciona	Falhas na administra ção de		Enteral (oral, cateter
07/12/2020	CE	SOBRAL	HOSPITA L REGIONA	-	Enviada	Eventos Adversos Relaciona	Falhas na administra ção de		Enteral (oral, cateter
07/12/2020	CE	SOBRAL	HOSPITA L REGIONA	-	Enviada	Eventos Adversos Relaciona	Falhas na administra ção de		Enteral (oral, cateter
10/12/2020	CE	SOBRAL	HOSPITA L REGIONA	-	Enviada	Eventos Adversos Relaciona	Falhas na administra ção de		Enteral (oral, cateter
10/12/2020	CE	SOBRAL	HOSPITA L REGIONA	-	Enviada	Eventos Adversos Relaciona	Falhas na administra ção de		Enteral (oral, cateter
10/12/2020	CE	SOBRAL	HOSPITA L REGIONA	-	Enviada	Eventos Adversos Relaciona	Falhas na administra ção de		Enteral (oral, cateter

10/12/2020	CE	SOBRAL	HOSPITA L REGIONA	-	Enviada	Eventos Adversos Relaciona	Falhas na administra ção de		Enteral (oral, cateter
10/12/2020	CE	SOBRAL	HOSPITA L REGIONA	-	Enviada	Eventos Adversos Relaciona	Falhas na administra ção de		Enteral (oral, cateter
10/12/2020	CE	SOBRAL	HOSPITA L REGIONA	-	Enviada	Eventos Adversos Relaciona	Falhas na administra ção de		Enteral (oral, cateter
10/12/2020	CE	SOBRAL	HOSPITA L REGIONA	-	Enviada	Eventos Adversos Relaciona	Falhas na administra ção de		Enteral (oral, cateter
10/12/2020	CE	SOBRAL	HOSPITA L REGIONA	-	Enviada	Eventos Adversos Relaciona	Falhas nas atividades		
10/12/2020	CE	SOBRAL	HOSPITA L REGIONA	-	Enviada	Eventos Adversos Relaciona	Falhas nas atividades		
10/12/2020	CE	SOBRAL	HOSPITA L REGIONA	-	Enviada	Eventos Adversos Relaciona	Falhas nas atividades		
10/12/2020	CE	SOBRAL	HOSPITA L REGIONA	-	Enviada	Eventos Adversos Relaciona	Falhas nas atividades		
10/12/2020	CE	SOBRAL	HOSPITA L REGIONA	-	Enviada	Eventos Adversos Relaciona	Falhas durante a assistênci		
10/12/2020	CE	SOBRAL	HOSPITA L REGIONA	-	Enviada	Eventos Adversos Relaciona	Falhas durante a assistênci		
10/12/2020	CE	SOBRAL	HOSPITA L REGIONA	-	Enviada	Eventos Adversos Relaciona	Falhas durante a assistênci		
10/12/2020	CE	SOBRAL	HOSPITA L REGIONA	-	Enviada	Eventos Adversos Relaciona	Falhas durante a assistênci		
10/12/2020	CE	SOBRAL	HOSPITA L REGIONA	-	Enviada	Eventos Adversos Relaciona	Falhas durante a assistênci		
10/12/2020	CE	SOBRAL	HOSPITA L REGIONA	-	Enviada	Eventos Adversos Relaciona	Falhas durante a assistênci		
14/12/2020	CE	SOBRAL	HOSPITA L REGIONA	-	Enviada	Eventos Adversos Relaciona	Falhas durante a assistênci		
14/12/2020	CE	SOBRAL	HOSPITA L REGIONA	-	Enviada	Eventos Adversos Relaciona	Falhas durante a assistênci		
14/12/2020	CE	SOBRAL	HOSPITA L REGIONA	-	Enviada	Eventos Adversos Relaciona	Falhas nas atividades		
14/12/2020	CE	SOBRAL	HOSPITA L REGIONA	-	Enviada	Eventos Adversos Relaciona	Falhas durante a assistênci		
14/12/2020	CE	SOBRAL	HOSPITA L REGIONA	-	Enviada	Eventos Adversos Relaciona	Falhas durante a assistênci		

14/12/2020	CE	SOBRAL	HOSPITA L REGIONA	-	Enviada	Eventos Adversos Relaciona	Falhas durante a assistênci		
14/12/2020	CE	SOBRAL	HOSPITA L REGIONA	-	Enviada	Eventos Adversos Relaciona	Falhas durante a assistênci		
14/12/2020	CE	SOBRAL	HOSPITA L REGIONA	-	Enviada	Eventos Adversos Relaciona	Falhas durante a assistênci		
14/12/2020	CE	SOBRAL	HOSPITA L REGIONA	-	Enviada	Eventos Adversos Relaciona	Falhas durante a assistênci		
14/12/2020	CE	SOBRAL	HOSPITA L REGIONA	-	Enviada	Eventos Adversos Relaciona	Falhas durante a assistênci		
14/12/2020	CE	SOBRAL	HOSPITA L REGIONA	-	Enviada	Eventos Adversos Relaciona	Falhas durante a assistênci		
14/12/2020	CE	SOBRAL	HOSPITA L REGIONA	-	Enviada	Eventos Adversos Relaciona	Falhas durante a assistênci		
14/12/2020	CE	SOBRAL	HOSPITA L REGIONA	-	Enviada	Eventos Adversos Relaciona	Falhas durante a assistênci		
14/12/2020	CE	SOBRAL	HOSPITA L REGIONA	-	Enviada	Eventos Adversos Relaciona	Falhas durante a assistênci		
14/12/2020	CE	SOBRAL	HOSPITA L REGIONA	-	Enviada	Eventos Adversos Relaciona	Falhas durante a assistênci		
14/12/2020	CE	SOBRAL	HOSPITA L REGIONA	-	Enviada	Eventos Adversos Relaciona	Falhas durante a assistênci		
14/12/2020	CE	SOBRAL	HOSPITA L REGIONA	-	Enviada	Eventos Adversos Relaciona	Falhas durante a assistênci		
14/12/2020	CE	SOBRAL	HOSPITA L REGIONA	-	Enviada	Eventos Adversos Relaciona	Falhas durante a assistênci		
14/12/2020	CE	SOBRAL	HOSPITA L REGIONA	-	Enviada	Eventos Adversos Relaciona	Falhas durante a assistênci		
14/12/2020	CE	SOBRAL	HOSPITA L REGIONA	-	Enviada	Eventos Adversos Relaciona	Falhas durante a assistênci		
14/12/2020	CE	SOBRAL	HOSPITA L REGIONA	-	Enviada	Eventos Adversos Relaciona	Falhas durante a assistênci		
14/12/2020	CE	SOBRAL	HOSPITA L REGIONA	-	Enviada	Eventos Adversos Relaciona	Falhas durante a assistênci		
14/12/2020	CE	SOBRAL	HOSPITA L REGIONA	-	Enviada	Eventos Adversos Relaciona	Falhas durante a assistênci		
14/12/2020	CE	SOBRAL	HOSPITA L REGIONA	-	Enviada	Eventos Adversos Relaciona	Falhas durante a assistênci		

14/12/2020	CE	SOBRAL	HOSPITA L REGIONA	-	Enviada	Eventos Adversos Relaciona	Falhas durante a assistênci		
14/12/2020	CE	SOBRAL	HOSPITA L REGIONA	-	Enviada	Eventos Adversos Relaciona	Falhas durante a assistênci		
14/12/2020	CE	SOBRAL	HOSPITA L REGIONA	-	Enviada	Eventos Adversos Relaciona	Falhas durante a assistênci		
14/12/2020	CE	SOBRAL	HOSPITA L REGIONA	-	Enviada	Eventos Adversos Relaciona	Falhas durante a assistênci		
14/12/2020	CE	SOBRAL	HOSPITA L REGIONA	-	Enviada	Eventos Adversos Relaciona	Falhas durante a assistênci		
14/12/2020	CE	SOBRAL	HOSPITA L REGIONA	-	Enviada	Eventos Adversos Relaciona	Falhas durante a assistênci		
14/12/2020	CE	SOBRAL	HOSPITA L REGIONA	-	Enviada	Eventos Adversos Relaciona	Falhas durante a assistênci		
14/12/2020	CE	SOBRAL	HOSPITA L REGIONA	-	Enviada	Eventos Adversos Relaciona	Falhas durante a assistênci		
14/12/2020	CE	SOBRAL	HOSPITA L REGIONA	-	Enviada	Eventos Adversos Relaciona	Falhas durante a assistênci		
14/12/2020	CE	SOBRAL	HOSPITA L REGIONA	-	Enviada	Eventos Adversos Relaciona	Falhas durante a assistênci		
14/12/2020	CE	SOBRAL	HOSPITA L REGIONA	-	Enviada	Eventos Adversos Relaciona	Falhas durante a assistênci		
14/12/2020	CE	SOBRAL	HOSPITA L REGIONA	-	Enviada	Eventos Adversos Relaciona	Falhas durante a assistênci		
14/12/2020	CE	SOBRAL	HOSPITA L REGIONA	-	Enviada	Eventos Adversos Relaciona	Falhas durante a assistênci		
22/12/2020	CE	SOBRAL	HOSPITA L REGIONA	-	Enviada	Eventos Adversos Relaciona	Lesão por pressão		
22/12/2020	CE	SOBRAL	HOSPITA L REGIONA	-	Enviada	Eventos Adversos Relaciona	Lesão por pressão		
22/12/2020	CE	SOBRAL	HOSPITA L REGIONA	-	Enviada	Eventos Adversos Relaciona	Lesão por pressão		
22/12/2020	CE	SOBRAL	HOSPITA L REGIONA	-	Enviada	Eventos Adversos Relaciona	Lesão por pressão		
22/12/2020	CE	SOBRAL	HOSPITA L REGIONA	-	Enviada	Eventos Adversos Relaciona	Lesão por pressão		
22/12/2020	CE	SOBRAL	HOSPITA L REGIONA	-	Enviada	Eventos Adversos Relaciona	Lesão por pressão		

22/12/2020	CE	SOBRAL	HOSPITA L REGIONA	-	Enviada	Eventos Adversos Relaciona	Lesão por pressão		
22/12/2020	CE	SOBRAL	HOSPITA L REGIONA	-	Enviada	Eventos Adversos Relaciona	Lesão por pressão		
22/12/2020	CE	SOBRAL	HOSPITA L REGIONA	-	Enviada	Eventos Adversos Relaciona	Lesão por pressão		
22/12/2020	CE	SOBRAL	HOSPITA L REGIONA	-	Enviada	Eventos Adversos Relaciona	Lesão por pressão		
22/12/2020	CE	SOBRAL	HOSPITA L REGIONA	-	Enviada	Eventos Adversos Relaciona	Lesão por pressão		
22/12/2020	CE	SOBRAL	HOSPITA L REGIONA	-	Enviada	Eventos Adversos Relaciona	Lesão por pressão		
22/12/2020	CE	SOBRAL	HOSPITA L REGIONA	-	Enviada	Eventos Adversos Relaciona	Lesão por pressão		
22/12/2020	CE	SOBRAL	HOSPITA L REGIONA	-	Enviada	Eventos Adversos Relaciona	Lesão por pressão		
22/12/2020	CE	SOBRAL	HOSPITA L REGIONA	-	Enviada	Eventos Adversos Relaciona	Falhas envolvend o cateter		
22/12/2020	CE	SOBRAL	HOSPITA L REGIONA	-	Enviada	Eventos Adversos Relaciona	Falhas envolvend o cateter		
22/12/2020	CE	SOBRAL	HOSPITA L REGIONA	-	Enviada	Eventos Adversos Relaciona	Falhas envolvend o cateter		
22/12/2020	CE	SOBRAL	HOSPITA L REGIONA	-	Enviada	Eventos Adversos Relaciona	Falhas envolvend o cateter		
22/12/2020	CE	SOBRAL	HOSPITA L REGIONA	-	Enviada	Eventos Adversos Relaciona	Falhas envolvend o cateter		
22/12/2020	CE	SOBRAL	HOSPITA L REGIONA	-	Enviada	Eventos Adversos Relaciona	Falhas durante a assistênci		
22/12/2020	CE	SOBRAL	HOSPITA L REGIONA	-	Enviada	Eventos Adversos Relaciona	Queda do paciente		
22/12/2020	CE	SOBRAL	HOSPITA L REGIONA	-	Enviada	Eventos Adversos Relaciona	Queda do paciente		
22/12/2020	CE	SOBRAL	HOSPITA L REGIONA	-	Enviada	Eventos Adversos Relaciona	Falhas na administra ção de		Enteral (oral, cateter
23/12/2020	CE	SOBRAL	HOSPITA L REGIONA	-	Enviada	Eventos Adversos Relaciona	Falhas na administra ção de		Enteral (oral, cateter
23/12/2020	CE	SOBRAL	HOSPITA L REGIONA	-	Enviada	Eventos Adversos Relaciona	Falhas na administra ção de		Enteral (oral, cateter

23/12/2020	CE	SOBRAL	HOSPITA L REGIONA	-	Enviada	Eventos Adversos Relaciona	Falhas na administra ção de		Enteral (oral, cateter
23/12/2020	CE	SOBRAL	HOSPITA L REGIONA	-	Enviada	Eventos Adversos Relaciona	Falhas na administra ção de		Enteral (oral, cateter
23/12/2020	CE	SOBRAL	HOSPITA L REGIONA	-	Enviada	Eventos Adversos Relaciona	Falhas na administra ção de		Enteral (oral, cateter
23/12/2020	CE	SOBRAL	HOSPITA L REGIONA	-	Enviada	Eventos Adversos Relaciona	Falhas nas atividades		
23/12/2020	CE	SOBRAL	HOSPITA L REGIONA	-	Enviada	Eventos Adversos Relaciona	Falhas nas atividades		
23/12/2020	CE	SOBRAL	HOSPITA L REGIONA	-	Enviada	Eventos Adversos Relaciona	Falhas nas atividades		
23/12/2020	CE	SOBRAL	HOSPITA L REGIONA	-	Enviada	Eventos Adversos Relaciona	Falhas nas atividades		
23/12/2020	CE	SOBRAL	HOSPITA L REGIONA	-	Enviada	Eventos Adversos Relaciona	Falhas nas atividades		
23/12/2020	CE	SOBRAL	HOSPITA L REGIONA	-	Enviada	Eventos Adversos Relaciona	Falhas nas atividades		
23/12/2020	CE	SOBRAL	HOSPITA L REGIONA	-	Enviada	Eventos Adversos Relaciona	Falhas nas atividades		
23/12/2020	CE	SOBRAL	HOSPITA L REGIONA	-	Enviada	Eventos Adversos Relaciona	Falhas nas atividades		
23/12/2020	CE	SOBRAL	HOSPITA L REGIONA	-	Enviada	Eventos Adversos Relaciona	Falhas nas atividades		
23/12/2020	CE	SOBRAL	HOSPITA L REGIONA	-	Enviada	Eventos Adversos Relaciona	Falhas nas atividades		
28/12/2020	CE	SOBRAL	HOSPITA L REGIONA	-	Enviada	Eventos Adversos Relaciona	Falhas durante a assistênci		
28/12/2020	CE	SOBRAL	HOSPITA L REGIONA	-	Enviada	Eventos Adversos Relaciona	Falhas durante a assistênci		
28/12/2020	CE	SOBRAL	HOSPITA L REGIONA	-	Enviada	Eventos Adversos Relaciona	Falhas durante a assistênci		
28/12/2020	CE	SOBRAL	HOSPITA L REGIONA	-	Enviada	Eventos Adversos Relaciona	Falhas durante a assistênci		
28/12/2020	CE	SOBRAL	HOSPITA L REGIONA	-	Enviada	Eventos Adversos Relaciona	Falhas durante a assistênci		
28/12/2020	CE	SOBRAL	HOSPITA L REGIONA	-	Enviada	Eventos Adversos Relaciona	Falhas durante a assistênci		

28/12/2020	CE	SOBRAL	HOSPITA L REGIONA	-	Enviada	Eventos Adversos Relaciona	Falhas durante a assistênci		
28/12/2020	CE	SOBRAL	HOSPITA L REGIONA	-	Enviada	Eventos Adversos Relaciona	Falhas durante a assistênci		
28/12/2020	CE	SOBRAL	HOSPITA L REGIONA	-	Enviada	Eventos Adversos Relaciona	Falhas durante a assistênci		
28/12/2020	CE	SOBRAL	HOSPITA L REGIONA	-	Enviada	Eventos Adversos Relaciona	Falhas durante a assistênci		
28/12/2020	CE	SOBRAL	HOSPITA L REGIONA	-	Enviada	Eventos Adversos Relaciona	Falhas durante a assistênci		
28/12/2020	CE	SOBRAL	HOSPITA L REGIONA	-	Enviada	Eventos Adversos Relaciona	Falhas ocorridas em		
28/12/2020	CE	SOBRAL	HOSPITA L REGIONA	-	Enviada	Eventos Adversos Relaciona	Falhas durante a assistênci		
28/12/2020	CE	SOBRAL	HOSPITA L REGIONA	-	Enviada	Eventos Adversos Relaciona	Falhas durante a assistênci		
28/12/2020	CE	SOBRAL	HOSPITA L REGIONA	-	Enviada	Eventos Adversos Relaciona	Falhas durante a assistênci		
28/12/2020	CE	SOBRAL	HOSPITA L REGIONA	-	Enviada	Eventos Adversos Relaciona	Falhas ocorridas em		
28/12/2020	CE	SOBRAL	HOSPITA L REGIONA	-	Enviada	Eventos Adversos Relaciona	Falhas durante a assistênci		
28/12/2020	CE	SOBRAL	HOSPITA L REGIONA	-	Enviada	Eventos Adversos Relaciona	Falhas durante a assistênci		
28/12/2020	CE	SOBRAL	HOSPITA L REGIONA	-	Enviada	Eventos Adversos Relaciona	Falhas ocorridas em		
28/12/2020	CE	SOBRAL	HOSPITA L REGIONA	-	Enviada	Eventos Adversos Relaciona	Falhas durante a assistênci		
28/12/2020	CE	SOBRAL	HOSPITA L REGIONA	-	Enviada	Eventos Adversos Relaciona	Falhas durante a assistênci		
28/12/2020	CE	SOBRAL	HOSPITA L REGIONA	-	Enviada	Eventos Adversos Relaciona	Falhas durante a assistênci		
28/12/2020	CE	SOBRAL	HOSPITA L REGIONA	-	Enviada	Eventos Adversos Relaciona	Falhas nas atividades		
28/12/2020	CE	SOBRAL	HOSPITA L REGIONA	-	Enviada	Eventos Adversos Relaciona	Falhas durante a assistênci		
28/12/2020	CE	SOBRAL	HOSPITA L REGIONA	-	Enviada	Eventos Adversos Relaciona	Falhas durante a assistênci		

11/01/2021	CE	SOBRAL	HOSPITA L REGIONA	-	Enviada	Eventos Adversos Relaciona	Falhas envolvend o cateter		
11/01/2021	CE	SOBRAL	HOSPITA L REGIONA	-	Enviada	Eventos Adversos Relaciona	Falhas envolvend o cateter		
11/01/2021	CE	SOBRAL	HOSPITA L REGIONA	-	Enviada	Eventos Adversos Relaciona	Falhas durante a assistênci		
11/01/2021	CE	SOBRAL	HOSPITA L REGIONA	-	Enviada	Eventos Adversos Relaciona	Falhas nas atividades		
11/01/2021	CE	SOBRAL	HOSPITA L REGIONA	-	Enviada	Eventos Adversos Relaciona	Falhas durante a assistênci		
11/01/2021	CE	SOBRAL	HOSPITA L REGIONA	-	Enviada	Eventos Adversos Relaciona	Falhas durante a assistênci		
11/01/2021	CE	SOBRAL	HOSPITA L REGIONA	-	Enviada	Eventos Adversos Relaciona	Falhas nas atividades		
11/01/2021	CE	SOBRAL	HOSPITA L REGIONA	-	Enviada	Eventos Adversos Relaciona	Falhas na administra ção de		Enteral (oral, cateter
11/01/2021	CE	SOBRAL	HOSPITA L REGIONA	-	Enviada	Eventos Adversos Relaciona	Falhas na administra ção de		Parenteral (via intravenos
11/01/2021	CE	SOBRAL	HOSPITA L REGIONA	-	Enviada	Eventos Adversos Relaciona	Falhas na administra ção de		Enteral (oral, cateter
11/01/2021	CE	SOBRAL	HOSPITA L REGIONA	-	Enviada	Eventos Adversos Relaciona	Falhas na administra ção de		Enteral (oral, cateter
11/01/2021	CE	SOBRAL	HOSPITA L REGIONA	-	Enviada	Eventos Adversos Relaciona	Falhas na administra ção de		Enteral (oral, cateter
11/01/2021	CE	SOBRAL	HOSPITA L REGIONA	-	Enviada	Eventos Adversos Relaciona	Falhas na administra ção de		Enteral (oral, cateter
11/01/2021	CE	SOBRAL	HOSPITA L REGIONA	-	Enviada	Eventos Adversos Relaciona	Queda do paciente		
11/01/2021	CE	SOBRAL	HOSPITA L REGIONA	-	Enviada	Eventos Adversos Relaciona	Falhas envolvend o cateter		
11/01/2021	CE	SOBRAL	HOSPITA L REGIONA	-	Enviada	Eventos Adversos Relaciona	Falhas envolvend o cateter		
11/01/2021	CE	SOBRAL	HOSPITA L REGIONA	-	Enviada	Eventos Adversos Relaciona	Lesão por pressão		
11/01/2021	CE	SOBRAL	HOSPITA L REGIONA	-	Enviada	Eventos Adversos Relaciona	Lesão por pressão		
11/01/2021	CE	SOBRAL	HOSPITA L REGIONA	-	Enviada	Eventos Adversos Relaciona	Lesão por pressão		

11/01/2021	CE	SOBRAL	HOSPITA L REGIONA	-	Enviada	Eventos Adversos Relaciona	Lesão por pressão		
11/01/2021	CE	SOBRAL	HOSPITA L REGIONA	-	Enviada	Eventos Adversos Relaciona	Lesão por pressão		
11/01/2021	CE	SOBRAL	HOSPITA L REGIONA	-	Enviada	Eventos Adversos Relaciona	Lesão por pressão		
11/01/2021	CE	SOBRAL	HOSPITA L REGIONA	-	Enviada	Eventos Adversos Relaciona	Lesão por pressão		
11/01/2021	CE	SOBRAL	HOSPITA L REGIONA	-	Enviada	Eventos Adversos Relaciona	Lesão por pressão		
11/01/2021	CE	SOBRAL	HOSPITA L REGIONA	-	Enviada	Eventos Adversos Relaciona	Lesão por pressão		
12/01/2021	CE	SOBRAL	HOSPITA L REGIONA	-	Enviada	Eventos Adversos Relaciona	Lesão por pressão		
12/01/2021	CE	SOBRAL	HOSPITA L REGIONA	-	Enviada	Eventos Adversos Relaciona	Lesão por pressão		
12/01/2021	CE	SOBRAL	HOSPITA L REGIONA	-	Enviada	Eventos Adversos Relaciona	Lesão por pressão		
28/01/2021	CE	SOBRAL	HOSPITA L REGIONA	-	Enviada	Eventos Adversos Relaciona	Falhas durante a assistênci		
28/01/2021	CE	SOBRAL	HOSPITA L REGIONA	-	Enviada	Eventos Adversos Relaciona	Falhas envolvend o cateter		
28/01/2021	CE	SOBRAL	HOSPITA L REGIONA	-	Enviada	Eventos Adversos Relaciona	Falhas envolvend o cateter		
28/01/2021	CE	SOBRAL	HOSPITA L REGIONA	-	Enviada	Eventos Adversos Relaciona	Falhas envolvend o cateter		
28/01/2021	CE	SOBRAL	HOSPITA L REGIONA	-	Enviada	Eventos Adversos Relaciona	Lesão por pressão		
28/01/2021	CE	SOBRAL	HOSPITA L REGIONA	-	Enviada	Eventos Adversos Relaciona	Lesão por pressão		
28/01/2021	CE	SOBRAL	HOSPITA L REGIONA	-	Enviada	Eventos Adversos Relaciona	Lesão por pressão		
28/01/2021	CE	SOBRAL	HOSPITA L REGIONA	-	Enviada	Eventos Adversos Relaciona	Lesão por pressão		
28/01/2021	CE	SOBRAL	HOSPITA L REGIONA	-	Enviada	Eventos Adversos Relaciona	Lesão por pressão		
28/01/2021	CE	SOBRAL	HOSPITA L REGIONA	-	Enviada	Eventos Adversos Relaciona	Lesão por pressão		

28/01/2021	CE	SOBRAL	HOSPITA L REGIONA	-	Enviada	Eventos Adversos Relaciona	Lesão por pressão		
28/01/2021	CE	SOBRAL	HOSPITA L REGIONA	-	Enviada	Eventos Adversos Relaciona	Lesão por pressão		
28/01/2021	CE	SOBRAL	HOSPITA L REGIONA	-	Enviada	Eventos Adversos Relaciona	Lesão por pressão		
28/01/2021	CE	SOBRAL	HOSPITA L REGIONA	-	Enviada	Eventos Adversos Relaciona	Lesão por pressão		
28/01/2021	CE	SOBRAL	HOSPITA L REGIONA	-	Enviada	Eventos Adversos Relaciona	Lesão por pressão		
28/01/2021	CE	SOBRAL	HOSPITA L REGIONA	-	Enviada	Eventos Adversos Relaciona	Lesão por pressão		
28/01/2021	CE	SOBRAL	HOSPITA L REGIONA	-	Enviada	Eventos Adversos Relaciona	Lesão por pressão		
28/01/2021	CE	SOBRAL	HOSPITA L REGIONA	-	Enviada	Eventos Adversos Relaciona	Lesão por pressão		
28/01/2021	CE	SOBRAL	HOSPITA L REGIONA	-	Enviada	Eventos Adversos Relaciona	Lesão por pressão		
28/01/2021	CE	SOBRAL	HOSPITA L REGIONA	-	Enviada	Eventos Adversos Relaciona	Lesão por pressão		
28/01/2021	CE	SOBRAL	HOSPITA L REGIONA	-	Enviada	Eventos Adversos Relaciona	Lesão por pressão		
28/01/2021	CE	SOBRAL	HOSPITA L REGIONA	-	Enviada	Eventos Adversos Relaciona	Lesão por pressão		
28/01/2021	CE	SOBRAL	HOSPITA L REGIONA	-	Enviada	Eventos Adversos Relaciona	Lesão por pressão		
28/01/2021	CE	SOBRAL	HOSPITA L REGIONA	-	Enviada	Eventos Adversos Relaciona	Lesão por pressão		
28/01/2021	CE	SOBRAL	HOSPITA L REGIONA	-	Enviada	Eventos Adversos Relaciona	Lesão por pressão		
28/01/2021	CE	SOBRAL	HOSPITA L REGIONA	-	Enviada	Eventos Adversos Relaciona	Falhas envolvend o cateter		
28/01/2021	CE	SOBRAL	HOSPITA L REGIONA	-	Enviada	Eventos Adversos Relaciona	Falhas envolvend o cateter		
28/01/2021	CE	SOBRAL	HOSPITA L REGIONA	-	Enviada	Eventos Adversos Relaciona	Falhas na administra ção de		Enteral (oral, cateter
28/01/2021	CE	SOBRAL	HOSPITA L REGIONA	-	Enviada	Eventos Adversos Relaciona	Falhas na administra ção de		Enteral (oral, cateter

28/01/2021	CE	SOBRAL	HOSPITA L REGIONA	-	Enviada	Eventos Adversos Relaciona	Falhas na administra ção de		Enteral (oral, cateter
28/01/2021	CE	SOBRAL	HOSPITA L REGIONA	-	Enviada	Eventos Adversos Relaciona	Falhas na administra ção de		Enteral (oral, cateter
28/01/2021	CE	SOBRAL	HOSPITA L REGIONA	-	Enviada	Eventos Adversos Relaciona	Falhas durante a assistênci		
29/01/2021	CE	SOBRAL	HOSPITA L REGIONA	-	Enviada	Eventos Adversos Relaciona	Falhas na administra ção de		Enteral (oral, cateter
29/01/2021	CE	SOBRAL	HOSPITA L REGIONA	-	Enviada	Eventos Adversos Relaciona	Falhas nas atividades		
29/01/2021	CE	SOBRAL	HOSPITA L REGIONA	-	Enviada	Eventos Adversos Relaciona	Falhas nas atividades		
29/01/2021	CE	SOBRAL	HOSPITA L REGIONA	-	Enviada	Eventos Adversos Relaciona	Falhas nas atividades		
29/01/2021	CE	SOBRAL	HOSPITA L REGIONA	-	Enviada	Eventos Adversos Relaciona	Falhas durante a assistênci		
18/02/2021	CE	SOBRAL	HOSPITA L REGIONA	-	Enviada	Eventos Adversos Relaciona	Falhas envolvend o cateter		
18/02/2021	CE	SOBRAL	HOSPITA L REGIONA	-	Enviada	Eventos Adversos Relaciona	Falhas envolvend o cateter		
18/02/2021	CE	SOBRAL	HOSPITA L REGIONA	-	Enviada	Eventos Adversos Relaciona	Falhas durante a assistênci		
18/02/2021	CE	SOBRAL	HOSPITA L REGIONA	-	Enviada	Eventos Adversos Relaciona	Falhas durante a assistênci		
18/02/2021	CE	SOBRAL	HOSPITA L REGIONA	-	Enviada	Eventos Adversos Relaciona	Lesão por pressão		
18/02/2021	CE	SOBRAL	HOSPITA L REGIONA	-	Enviada	Eventos Adversos Relaciona	Falhas envolvend o cateter		
18/02/2021	CE	SOBRAL	HOSPITA L REGIONA	-	Enviada	Eventos Adversos Relaciona	Falhas envolvend o cateter		
18/02/2021	CE	SOBRAL	HOSPITA L REGIONA	-	Enviada	Eventos Adversos Relaciona	Falhas na administra ção de		Enteral (oral, cateter
18/02/2021	CE	SOBRAL	HOSPITA L REGIONA	-	Enviada	Eventos Adversos Relaciona	Lesão por pressão		
18/02/2021	CE	SOBRAL	HOSPITA L REGIONA	-	Enviada	Eventos Adversos Relaciona	Lesão por pressão		
18/02/2021	CE	SOBRAL	HOSPITA L REGIONA	-	Enviada	Eventos Adversos Relaciona	Falhas nas atividades		

18/02/2021	CE	SOBRAL	HOSPITA L REGIONA	-	Enviada	Eventos Adversos Relaciona	Falhas na administra ção de		Enteral (oral, cateter
18/02/2021	CE	SOBRAL	HOSPITA L REGIONA	-	Enviada	Eventos Adversos Relaciona	Falhas na administra ção de		Enteral (oral, cateter
18/02/2021	CE	SOBRAL	HOSPITA L REGIONA	-	Enviada	Eventos Adversos Relaciona	Lesão por pressão		
18/02/2021	CE	SOBRAL	HOSPITA L REGIONA	-	Enviada	Eventos Adversos Relaciona	Falha na document ação		
18/02/2021	CE	SOBRAL	HOSPITA L REGIONA	-	Enviada	Eventos Adversos Relaciona	Falha na document ação		
18/02/2021	CE	SOBRAL	HOSPITA L REGIONA	-	Enviada	Eventos Adversos Relaciona	Falha na document ação		
18/02/2021	CE	SOBRAL	HOSPITA L REGIONA	-	Enviada	Eventos Adversos Relaciona	Lesão por pressão		
18/02/2021	CE	SOBRAL	HOSPITA L REGIONA	-	Enviada	Eventos Adversos Relaciona	Lesão por pressão		
23/02/2021	CE	SOBRAL	HOSPITA L REGIONA	-	Enviada	Eventos Adversos Relaciona	Falhas durante a assistênci		
23/02/2021	CE	SOBRAL	HOSPITA L REGIONA	-	Enviada	Eventos Adversos Relaciona	Queda do paciente		
23/02/2021	CE	SOBRAL	HOSPITA L REGIONA	-	Enviada	Eventos Adversos Relaciona	Falhas na administra ção de		Enteral (oral, cateter
23/02/2021	CE	SOBRAL	HOSPITA L REGIONA	-	Enviada	Eventos Adversos Relaciona	Lesão por pressão		
23/02/2021	CE	SOBRAL	HOSPITA L REGIONA	-	Enviada	Eventos Adversos Relaciona	Lesão por pressão		
23/02/2021	CE	SOBRAL	HOSPITA L REGIONA	-	Enviada	Eventos Adversos Relaciona	Lesão por pressão		
23/02/2021	CE	SOBRAL	HOSPITA L REGIONA	-	Enviada	Eventos Adversos Relaciona	Falhas durante a assistênci		
23/02/2021	CE	SOBRAL	HOSPITA L REGIONA	-	Enviada	Eventos Adversos Relaciona	Queda do paciente		
23/02/2021	CE	SOBRAL	HOSPITA L REGIONA	-	Enviada	Eventos Adversos Relaciona	Queda do paciente		
23/02/2021	CE	SOBRAL	HOSPITA L REGIONA	-	Enviada	Eventos Adversos Relaciona	Falhas na administra ção de		Enteral (oral, cateter
02/03/2021	CE	SOBRAL	HOSPITA L REGIONA	-	Enviada	Eventos Adversos Relaciona	Falhas durante a assistênci		

02/03/2021	CE	SOBRAL	HOSPITA L REGIONA	-	Enviada	Eventos Adversos Relaciona	Lesão por pressão		
02/03/2021	CE	SOBRAL	HOSPITA L REGIONA	-	Enviada	Eventos Adversos Relaciona	Lesão por pressão		
02/03/2021	CE	SOBRAL	HOSPITA L REGIONA	-	Enviada	Eventos Adversos Relaciona	Falhas envolvend o cateter		
02/03/2021	CE	SOBRAL	HOSPITA L REGIONA	-	Enviada	Eventos Adversos Relaciona	Falhas durante a assistênci		
02/03/2021	CE	SOBRAL	HOSPITA L REGIONA	-	Enviada	Eventos Adversos Relaciona	Falhas na administra ção de		Enteral (oral, cateter
02/03/2021	CE	SOBRAL	HOSPITA L REGIONA	-	Enviada	Eventos Adversos Relaciona	Falhas na administra ção de		Enteral (oral, cateter
02/03/2021	CE	SOBRAL	HOSPITA L REGIONA	-	Enviada	Eventos Adversos Relaciona	Falhas na administra ção de		Enteral (oral, cateter
02/03/2021	CE	SOBRAL	HOSPITA L REGIONA	-	Enviada	Eventos Adversos Relaciona	Falhas na administra ção de		Enteral (oral, cateter
02/03/2021	CE	SOBRAL	HOSPITA L REGIONA	-	Enviada	Eventos Adversos Relaciona	Falhas na administra ção de		Enteral (oral, cateter
02/03/2021	CE	SOBRAL	HOSPITA L REGIONA	-	Enviada	Eventos Adversos Relaciona	Lesão por pressão		
02/03/2021	CE	SOBRAL	HOSPITA L REGIONA	-	Enviada	Eventos Adversos Relaciona	Queda do paciente		
02/03/2021	CE	SOBRAL	HOSPITA L REGIONA	-	Enviada	Eventos Adversos Relaciona	Queda do paciente		
02/03/2021	CE	SOBRAL	HOSPITA L REGIONA	-	Enviada	Eventos Adversos Relaciona	Falhas na administra ção de		Enteral (oral, cateter
02/03/2021	CE	SOBRAL	HOSPITA L REGIONA	-	Enviada	Eventos Adversos Relaciona	Falhas na administra ção de		Enteral (oral, cateter
03/03/2021	CE	SOBRAL	HOSPITA L REGIONA	-	Enviada	Eventos Adversos Relaciona	Lesão por pressão		
03/03/2021	CE	SOBRAL	HOSPITA L REGIONA	-	Enviada	Eventos Adversos Relaciona	Lesão por pressão		
03/03/2021	CE	SOBRAL	HOSPITA L REGIONA	-	Enviada	Eventos Adversos Relaciona	Falhas envolvend o cateter		
03/03/2021	CE	SOBRAL	HOSPITA L REGIONA	-	Enviada	Eventos Adversos Relaciona	Queda do paciente		
03/03/2021	CE	SOBRAL	HOSPITA L REGIONA	-	Enviada	Eventos Adversos Relaciona	Falhas durante a assistênci		

03/03/2021	CE	SOBRAL	HOSPITA<br>L<br>REGIONA	-	Enviada	Eventos<br>Adversos<br>Relaciona	Lesão<br>por<br>pressão		
03/03/2021	CE	SOBRAL	HOSPITA<br>L<br>REGIONA	-	Enviada	Eventos<br>Adversos<br>Relaciona	Lesão<br>por<br>pressão		
03/03/2021	CE	SOBRAL	HOSPITA<br>L<br>REGIONA	-	Enviada	Eventos<br>Adversos<br>Relaciona	Queda do<br>paciente		
03/03/2021	CE	SOBRAL	HOSPITA<br>L<br>REGIONA	-	Enviada	Eventos<br>Adversos<br>Relaciona	Falhas<br>durante a<br>assistênci		
03/03/2021	CE	SOBRAL	HOSPITA<br>L<br>REGIONA	-	Enviada	Eventos<br>Adversos<br>Relaciona	Lesão<br>por<br>pressão		
03/03/2021	CE	SOBRAL	HOSPITA<br>L<br>REGIONA	-	Enviada	Eventos<br>Adversos<br>Relaciona	Lesão<br>por<br>pressão		
03/03/2021	CE	SOBRAL	HOSPITA<br>L<br>REGIONA	-	Enviada	Eventos<br>Adversos<br>Relaciona	Lesão<br>por<br>pressão		
05/03/2021	CE	SOBRAL	HOSPITA<br>L<br>REGIONA	-	Enviada	Eventos<br>Adversos<br>Relaciona	Falhas<br>envolvend<br>o cateter		
05/03/2021	CE	SOBRAL	HOSPITA<br>L<br>REGIONA	-	Enviada	Eventos<br>Adversos<br>Relaciona	Falhas<br>envolvend<br>o cateter		
05/03/2021	CE	SOBRAL	HOSPITA<br>L<br>REGIONA	-	Enviada	Eventos<br>Adversos<br>Relaciona	Lesão<br>por<br>pressão		
05/03/2021	CE	SOBRAL	HOSPITA<br>L<br>REGIONA	-	Enviada	Eventos<br>Adversos<br>Relaciona	Lesão<br>por<br>pressão		
05/03/2021	CE	SOBRAL	HOSPITA<br>L<br>REGIONA	-	Enviada	Eventos<br>Adversos<br>Relaciona	Lesão<br>por<br>pressão		
05/03/2021	CE	SOBRAL	HOSPITA<br>L<br>REGIONA	-	Enviada	Eventos<br>Adversos<br>Relaciona	Lesão<br>por<br>pressão		
05/03/2021	CE	SOBRAL	HOSPITA<br>L<br>REGIONA	-	Enviada	Eventos<br>Adversos<br>Relaciona	Lesão<br>por<br>pressão		
05/03/2021	CE	SOBRAL	HOSPITA<br>L<br>REGIONA	-	Enviada	Eventos<br>Adversos<br>Relaciona	Lesão<br>por<br>pressão		
05/03/2021	CE	SOBRAL	HOSPITA<br>L<br>REGIONA	-	Enviada	Eventos<br>Adversos<br>Relaciona	Lesão<br>por<br>pressão		
05/03/2021	CE	SOBRAL	HOSPITA<br>L<br>REGIONA	-	Enviada	Eventos<br>Adversos<br>Relaciona	Lesão<br>por<br>pressão		
05/03/2021	CE	SOBRAL	HOSPITA<br>L<br>REGIONA	-	Enviada	Eventos<br>Adversos<br>Relaciona	Lesão<br>por<br>pressão		
05/03/2021	CE	SOBRAL	HOSPITA<br>L<br>REGIONA	-	Enviada	Eventos<br>Adversos<br>Relaciona	Lesão<br>por<br>pressão		

05/03/2021	CE	SOBRAL	HOSPITA L REGIONA	-	Enviada	Eventos Adversos Relaciona	Lesão por pressão		
05/03/2021	CE	SOBRAL	HOSPITA L REGIONA	-	Enviada	Eventos Adversos Relaciona	Lesão por pressão		
05/03/2021	CE	SOBRAL	HOSPITA L REGIONA	-	Enviada	Eventos Adversos Relaciona	Lesão por pressão		
05/03/2021	CE	SOBRAL	HOSPITA L REGIONA	-	Enviada	Eventos Adversos Relaciona	Falhas envolvend o cateter		
05/03/2021	CE	SOBRAL	HOSPITA L REGIONA	-	Enviada	Eventos Adversos Relaciona	Falhas envolvend o cateter		
05/03/2021	CE	SOBRAL	HOSPITA L REGIONA	-	Enviada	Eventos Adversos Relaciona	Queda do paciente		
05/03/2021	CE	SOBRAL	HOSPITA L REGIONA	-	Enviada	Eventos Adversos Relaciona	Queda do paciente		
08/03/2021	CE	SOBRAL	HOSPITA L REGIONA	-	Enviada	Eventos Adversos Relaciona	Queda do paciente		
08/03/2021	CE	SOBRAL	HOSPITA L REGIONA	-	Enviada	Eventos Adversos Relaciona	Queda do paciente		
08/03/2021	CE	SOBRAL	HOSPITA L REGIONA	-	Enviada	Eventos Adversos Relaciona	Queda do paciente		
08/03/2021	CE	SOBRAL	HOSPITA L REGIONA	-	Enviada	Eventos Adversos Relaciona	Falhas na administra ção de		Enteral (oral, cateter
08/03/2021	CE	SOBRAL	HOSPITA L REGIONA	-	Enviada	Eventos Adversos Relaciona	Falhas na administra ção de		Enteral (oral, cateter
08/03/2021	CE	SOBRAL	HOSPITA L REGIONA	-	Enviada	Eventos Adversos Relaciona	Falhas na administra ção de		
08/03/2021	CE	SOBRAL	HOSPITA L REGIONA	-	Enviada	Eventos Adversos Relaciona	Falhas nas atividades		
16/03/2021	CE	SOBRAL	HOSPITA L REGIONA	-	Enviada	Eventos Adversos Relaciona	Falha na document ação		
16/03/2021	CE	SOBRAL	HOSPITA L REGIONA	-	Enviada	Eventos Adversos Relaciona	Falha na identificaç ão do		
16/03/2021	CE	SOBRAL	HOSPITA L REGIONA	-	Enviada	Eventos Adversos Relaciona	Falha na identificaç ão do		
16/03/2021	CE	SOBRAL	HOSPITA L REGIONA	-	Enviada	Eventos Adversos Relaciona	Falha na document ação		
16/03/2021	CE	SOBRAL	HOSPITA L REGIONA	-	Enviada	Eventos Adversos Relaciona	Falhas durante a assistênci		

16/03/2021	CE	SOBRAL	HOSPITA L REGIONA	-	Enviada	Eventos Adversos Relaciona	Falhas durante a assistênci		
16/03/2021	CE	SOBRAL	HOSPITA L REGIONA	-	Enviada	Eventos Adversos Relaciona	Falhas durante a assistênci		
16/03/2021	CE	SOBRAL	HOSPITA L REGIONA	-	Enviada	Eventos Adversos Relaciona	Falhas durante a assistênci		
16/03/2021	CE	SOBRAL	HOSPITA L REGIONA	-	Enviada	Eventos Adversos Relaciona	Falhas ocorridas em		
16/03/2021	CE	SOBRAL	HOSPITA L REGIONA	-	Enviada	Eventos Adversos Relaciona	Falhas durante a assistênci		
12/04/2021	CE	SOBRAL	HOSPITA L REGIONA	-	Enviada	Eventos Adversos Relaciona	Lesão por pressão		
12/04/2021	CE	SOBRAL	HOSPITA L REGIONA	-	Enviada	Eventos Adversos Relaciona	Lesão por pressão		
12/04/2021	CE	SOBRAL	HOSPITA L REGIONA	-	Enviada	Eventos Adversos Relaciona	Lesão por pressão		
12/04/2021	CE	SOBRAL	HOSPITA L REGIONA	-	Enviada	Eventos Adversos Relaciona	Lesão por pressão		
12/04/2021	CE	SOBRAL	HOSPITA L REGIONA	-	Enviada	Eventos Adversos Relaciona	Lesão por pressão		
12/04/2021	CE	SOBRAL	HOSPITA L REGIONA	-	Enviada	Eventos Adversos Relaciona	Lesão por pressão		
12/04/2021	CE	SOBRAL	HOSPITA L REGIONA	-	Enviada	Eventos Adversos Relaciona	Lesão por pressão		
12/04/2021	CE	SOBRAL	HOSPITA L REGIONA	-	Enviada	Eventos Adversos Relaciona	Lesão por pressão		
12/04/2021	CE	SOBRAL	HOSPITA L REGIONA	-	Enviada	Eventos Adversos Relaciona	Lesão por pressão		
12/04/2021	CE	SOBRAL	HOSPITA L REGIONA	-	Enviada	Eventos Adversos Relaciona	Lesão por pressão		
12/04/2021	CE	SOBRAL	HOSPITA L REGIONA	-	Enviada	Eventos Adversos Relaciona	Lesão por pressão		
12/04/2021	CE	SOBRAL	HOSPITA L REGIONA	-	Enviada	Eventos Adversos Relaciona	Lesão por pressão		
12/04/2021	CE	SOBRAL	HOSPITA L REGIONA	-	Enviada	Eventos Adversos Relaciona	Lesão por pressão		
12/04/2021	CE	SOBRAL	HOSPITA L REGIONA	-	Enviada	Eventos Adversos Relaciona	Lesão por pressão		

12/04/2021	CE	SOBRAL	HOSPITA L REGIONA	-	Enviada	Eventos Adversos Relaciona	Lesão por pressão		
12/04/2021	CE	SOBRAL	HOSPITA L REGIONA	-	Enviada	Eventos Adversos Relaciona	Lesão por pressão		
12/04/2021	CE	SOBRAL	HOSPITA L REGIONA	-	Enviada	Eventos Adversos Relaciona	Lesão por pressão		
12/04/2021	CE	SOBRAL	HOSPITA L REGIONA	-	Enviada	Eventos Adversos Relaciona	Lesão por pressão		
12/04/2021	CE	SOBRAL	HOSPITA L REGIONA	-	Enviada	Eventos Adversos Relaciona	Lesão por pressão		
12/04/2021	CE	SOBRAL	HOSPITA L REGIONA	-	Enviada	Eventos Adversos Relaciona	Lesão por pressão		
12/04/2021	CE	SOBRAL	HOSPITA L REGIONA	-	Enviada	Eventos Adversos Relaciona	Lesão por pressão		
12/04/2021	CE	SOBRAL	HOSPITA L REGIONA	-	Enviada	Eventos Adversos Relaciona	Falhas envolvend o cateter		
13/04/2021	CE	SOBRAL	HOSPITA L REGIONA	-	Enviada	Eventos Adversos Relaciona	Falhas envolvend o cateter		
13/04/2021	CE	SOBRAL	HOSPITA L REGIONA	-	Enviada	Eventos Adversos Relaciona	Falhas envolvend o cateter		
13/04/2021	CE	SOBRAL	HOSPITA L REGIONA	-	Enviada	Eventos Adversos Relaciona	Falhas envolvend o cateter		
13/04/2021	CE	SOBRAL	HOSPITA L REGIONA	Perda de acesso central	Enviada	Eventos Adversos Relaciona	Falhas envolvend o cateter		
13/04/2021	CE	SOBRAL	HOSPITA L REGIONA	Perda de acesso central	Enviada	Eventos Adversos Relaciona	Falhas envolvend o cateter		
13/04/2021	CE	SOBRAL	HOSPITA L REGIONA	Perda de acesso venoso	Enviada	Eventos Adversos Relaciona	Falhas envolvend o cateter		
13/04/2021	CE	SOBRAL	HOSPITA L REGIONA	perda de acesso venoso	Enviada	Eventos Adversos Relaciona	Falhas envolvend o cateter		
13/04/2021	CE	SOBRAL	HOSPITA L REGIONA	-	Enviada	Eventos Adversos Relaciona	Queda do paciente		
13/04/2021	CE	SOBRAL	HOSPITA L REGIONA	-	Enviada	Eventos Adversos Relaciona	Queda do paciente		
13/04/2021	CE	SOBRAL	HOSPITA L REGIONA	-	Enviada	Eventos Adversos Relaciona	Falhas na administra ção de		Enteral (oral, cateter
13/04/2021	CE	SOBRAL	HOSPITA L REGIONA	-	Enviada	Eventos Adversos Relaciona	Falhas na administra ção de		Enteral (oral, cateter

13/04/2021	CE	SOBRAL	HOSPITA L REGIONA	-	Enviada	Eventos Adversos Relaciona	Falhas na administra ção de			Enteral (oral, cateter
13/04/2021	CE	SOBRAL	HOSPITA L REGIONA	-	Enviada	Eventos Adversos Relaciona	Falhas na administra ção de			Enteral (oral, cateter
13/04/2021	CE	SOBRAL	HOSPITA L REGIONA	-	Enviada	Eventos Adversos Relaciona	Falhas na administra ção de			Enteral (oral, cateter
13/04/2021	CE	SOBRAL	HOSPITA L REGIONA	-	Em retificação	Eventos Adversos Relaciona	Falha na document ação			
13/04/2021	CE	SOBRAL	HOSPITA L REGIONA	-	Enviada	Eventos Adversos Relaciona	Falhas durante a assistênci			
13/04/2021	CE	SOBRAL	HOSPITA L REGIONA	-	Enviada	Eventos Adversos Relaciona	Falhas durante a assistênci			
13/04/2021	CE	SOBRAL	HOSPITA L REGIONA	-	Enviada	Eventos Adversos Relaciona	Falhas durante a assistênci			
13/04/2021	CE	SOBRAL	HOSPITA L REGIONA	-	Enviada	Eventos Adversos Relaciona	Falhas durante a assistênci			
13/04/2021	CE	SOBRAL	HOSPITA L REGIONA	-	Enviada	Eventos Adversos Relaciona	Falhas durante a assistênci			
13/04/2021	CE	SOBRAL	HOSPITA L REGIONA	-	Enviada	Eventos Adversos Relaciona	Falhas durante a assistênci			
27/05/2021	CE	SOBRAL	HOSPITA L REGIONA	-	Enviada	Eventos Adversos Relaciona	Falhas envolvend o cateter			
31/05/2021	CE	SOBRAL	HOSPITA L REGIONA	-	Enviada	Eventos Adversos Relaciona	Lesão por pressão			
31/05/2021	CE	SOBRAL	HOSPITA L REGIONA	-	Enviada	Eventos Adversos Relaciona	Lesão por pressão			
02/06/2021	CE	SOBRAL	HOSPITA L REGIONA	-	Enviada	Eventos Adversos Relaciona	Lesão por pressão			
02/06/2021	CE	SOBRAL	HOSPITA L REGIONA	-	Enviada	Eventos Adversos Relaciona	Lesão por pressão			
02/06/2021	CE	SOBRAL	HOSPITA L REGIONA	-	Enviada	Eventos Adversos Relaciona	Falhas envolvend o cateter			
02/06/2021	CE	SOBRAL	HOSPITA L REGIONA	-	Enviada	Eventos Adversos Relaciona	Lesão por pressão			
02/06/2021	CE	SOBRAL	HOSPITA L REGIONA	-	Enviada	Eventos Adversos Relaciona	Lesão por pressão			
02/06/2021	CE	SOBRAL	HOSPITA L REGIONA	-	Enviada	Eventos Adversos Relaciona	Lesão por pressão			

02/06/2021	CE	SOBRAL	HOSPITA L REGIONA	-	Enviada	Eventos Adversos Relaciona	Falhas na administra ção de		Enteral (oral, cateter
02/06/2021	CE	SOBRAL	HOSPITA L REGIONA	-	Enviada	Eventos Adversos Relaciona	Lesão por pressão		
02/06/2021	CE	SOBRAL	HOSPITA L REGIONA	-	Enviada	Eventos Adversos Relaciona	Falhas envolvend o cateter		
02/06/2021	CE	SOBRAL	HOSPITA L REGIONA	-	Enviada	Eventos Adversos Relaciona	Lesão por pressão		
02/06/2021	CE	SOBRAL	HOSPITA L REGIONA	-	Enviada	Eventos Adversos Relaciona	Falhas envolvend o cateter		
02/06/2021	CE	SOBRAL	HOSPITA L REGIONA	-	Enviada	Eventos Adversos Relaciona	Lesão por pressão		
17/06/2021	CE	SOBRAL	HOSPITA L REGIONA	-	Enviada	Eventos Adversos Relaciona	Lesão por pressão		
18/06/2021	CE	SOBRAL	HOSPITA L REGIONA	-	Enviada	Eventos Adversos Relaciona	Lesão por pressão		
18/06/2021	CE	SOBRAL	HOSPITA L REGIONA	-	Enviada	Eventos Adversos Relaciona	Falhas durante a assistênci		
18/06/2021	CE	SOBRAL	HOSPITA L REGIONA	-	Enviada	Eventos Adversos Relaciona	Lesão por pressão		
21/06/2021	CE	SOBRAL	HOSPITA L REGIONA	-	Enviada	Eventos Adversos Relaciona	Falha na identificaç ão do		
21/06/2021	CE	SOBRAL	HOSPITA L REGIONA	-	Enviada	Eventos Adversos Relaciona	Lesão por pressão		
21/06/2021	CE	SOBRAL	HOSPITA L REGIONA	-	Enviada	Eventos Adversos Relaciona	Falhas envolvend o cateter		
21/06/2021	CE	SOBRAL	HOSPITA L REGIONA	-	Enviada	Eventos Adversos Relaciona	Falhas envolvend o cateter		
21/06/2021	CE	SOBRAL	HOSPITA L REGIONA	-	Enviada	Eventos Adversos Relaciona	Falhas durante a assistênci		
21/06/2021	CE	SOBRAL	HOSPITA L REGIONA	-	Enviada	Eventos Adversos Relaciona	Falhas durante a assistênci		
21/06/2021	CE	SOBRAL	HOSPITA L REGIONA	-	Enviada	Eventos Adversos Relaciona	Falhas durante a assistênci		
21/06/2021	CE	SOBRAL	HOSPITA L REGIONA	-	Enviada	Eventos Adversos Relaciona	Lesão por pressão		
21/06/2021	CE	SOBRAL	HOSPITA L REGIONA	-	Enviada	Eventos Adversos Relaciona	Lesão por pressão		

21/06/2021	CE	SOBRAL	HOSPITA L REGIONA	-	Enviada	Eventos Adversos Relaciona	Lesão por pressão		
21/06/2021	CE	SOBRAL	HOSPITA L REGIONA	-	Enviada	Eventos Adversos Relaciona	Queda do paciente		
21/06/2021	CE	SOBRAL	HOSPITA L REGIONA	-	Enviada	Eventos Adversos Relaciona	Falhas envolvend o cateter		
21/06/2021	CE	SOBRAL	HOSPITA L REGIONA	-	Enviada	Eventos Adversos Relaciona	Lesão por pressão		
21/06/2021	CE	SOBRAL	HOSPITA L REGIONA	-	Enviada	Eventos Adversos Relaciona	Lesão por pressão		
21/06/2021	CE	SOBRAL	HOSPITA L REGIONA	-	Enviada	Eventos Adversos Relaciona	Queda do paciente		
25/06/2021	CE	SOBRAL	HOSPITA L REGIONA	-	Enviada	Eventos Adversos Relaciona	Lesão por pressão		
25/06/2021	CE	SOBRAL	HOSPITA L REGIONA	-	Enviada	Eventos Adversos Relaciona	Falhas envolvend o cateter		
25/06/2021	CE	SOBRAL	HOSPITA L REGIONA	-	Enviada	Eventos Adversos Relaciona	Falhas na administra ção de		Enteral (oral, cateter
25/06/2021	CE	SOBRAL	HOSPITA L REGIONA	-	Enviada	Eventos Adversos Relaciona	Lesão por pressão		
25/06/2021	CE	SOBRAL	HOSPITA L REGIONA	-	Enviada	Eventos Adversos Relaciona	Lesão por pressão		
25/06/2021	CE	SOBRAL	HOSPITA L REGIONA	-	Enviada	Eventos Adversos Relaciona	Lesão por pressão		
25/06/2021	CE	SOBRAL	HOSPITA L REGIONA	-	Enviada	Eventos Adversos Relaciona	Lesão por pressão		
25/06/2021	CE	SOBRAL	HOSPITA L REGIONA	-	Enviada	Eventos Adversos Relaciona	Lesão por pressão		
25/06/2021	CE	SOBRAL	HOSPITA L REGIONA	-	Enviada	Eventos Adversos Relaciona	Lesão por pressão		
25/06/2021	CE	SOBRAL	HOSPITA L REGIONA	-	Enviada	Eventos Adversos Relaciona	Lesão por pressão		
28/06/2021	CE	SOBRAL	HOSPITA L REGIONA	-	Enviada	Eventos Adversos Relaciona	Lesão por pressão		
28/06/2021	CE	SOBRAL	HOSPITA L REGIONA	-	Enviada	Eventos Adversos Relaciona	Lesão por pressão		
28/06/2021	CE	SOBRAL	HOSPITA L REGIONA	-	Enviada	Eventos Adversos Relaciona	Lesão por pressão		

28/06/2021	CE	SOBRAL	HOSPITA L REGIONA	-	Enviada	Eventos Adversos Relaciona	Lesão por pressão		
28/06/2021	CE	SOBRAL	HOSPITA L REGIONA	-	Enviada	Eventos Adversos Relaciona	Lesão por pressão		
28/06/2021	CE	SOBRAL	HOSPITA L REGIONA	-	Enviada	Eventos Adversos Relaciona	Lesão por pressão		
28/06/2021	CE	SOBRAL	HOSPITA L REGIONA	-	Enviada	Eventos Adversos Relaciona	Lesão por pressão		
28/06/2021	CE	SOBRAL	HOSPITA L REGIONA	-	Enviada	Eventos Adversos Relaciona	Lesão por pressão		
28/06/2021	CE	SOBRAL	HOSPITA L REGIONA	-	Enviada	Eventos Adversos Relaciona	Lesão por pressão		
28/06/2021	CE	SOBRAL	HOSPITA L REGIONA	-	Enviada	Eventos Adversos Relaciona	Lesão por pressão		
28/06/2021	CE	SOBRAL	HOSPITA L REGIONA	-	Enviada	Eventos Adversos Relaciona	Lesão por pressão		
28/06/2021	CE	SOBRAL	HOSPITA L REGIONA	-	Enviada	Eventos Adversos Relaciona	Lesão por pressão		
28/06/2021	CE	SOBRAL	HOSPITA L REGIONA	-	Enviada	Eventos Adversos Relaciona	Lesão por pressão		
28/06/2021	CE	SOBRAL	HOSPITA L REGIONA	-	Enviada	Eventos Adversos Relaciona	Lesão por pressão		
28/06/2021	CE	SOBRAL	HOSPITA L REGIONA	-	Enviada	Eventos Adversos Relaciona	Lesão por pressão		
28/06/2021	CE	SOBRAL	HOSPITA L REGIONA	-	Enviada	Eventos Adversos Relaciona	Lesão por pressão		
28/06/2021	CE	SOBRAL	HOSPITA L REGIONA	-	Enviada	Eventos Adversos Relaciona	Lesão por pressão		
28/06/2021	CE	SOBRAL	HOSPITA L REGIONA	-	Enviada	Eventos Adversos Relaciona	Lesão por pressão		
29/06/2021	CE	SOBRAL	HOSPITA L REGIONA	-	Enviada	Eventos Adversos Relaciona	Lesão por pressão		
29/06/2021	CE	SOBRAL	HOSPITA L REGIONA	-	Enviada	Eventos Adversos Relaciona	Lesão por pressão		
29/06/2021	CE	SOBRAL	HOSPITA L REGIONA	-	Enviada	Eventos Adversos Relaciona	Lesão por pressão		
29/06/2021	CE	SOBRAL	HOSPITA L REGIONA	-	Enviada	Eventos Adversos Relaciona	Lesão por pressão		

29/06/2021	CE	SOBRAL	HOSPITA L REGIONA	-	Enviada	Eventos Adversos Relaciona	Lesão por pressão		
29/06/2021	CE	SOBRAL	HOSPITA L REGIONA	-	Enviada	Eventos Adversos Relaciona	Lesão por pressão		
29/06/2021	CE	SOBRAL	HOSPITA L REGIONA	-	Enviada	Eventos Adversos Relaciona	Lesão por pressão		
29/06/2021	CE	SOBRAL	HOSPITA L REGIONA	-	Enviada	Eventos Adversos Relaciona	Lesão por pressão		
29/06/2021	CE	SOBRAL	HOSPITA L REGIONA	-	Enviada	Eventos Adversos Relaciona	Lesão por pressão		
29/06/2021	CE	SOBRAL	HOSPITA L REGIONA	-	Enviada	Eventos Adversos Relaciona	Lesão por pressão		
29/06/2021	CE	SOBRAL	HOSPITA L REGIONA	-	Enviada	Eventos Adversos Relaciona	Lesão por pressão		
29/06/2021	CE	SOBRAL	HOSPITA L REGIONA	-	Enviada	Eventos Adversos Relaciona	Lesão por pressão		
29/06/2021	CE	SOBRAL	HOSPITA L REGIONA	-	Enviada	Eventos Adversos Relaciona	Lesão por pressão		
29/06/2021	CE	SOBRAL	HOSPITA L REGIONA	-	Enviada	Eventos Adversos Relaciona	Lesão por pressão		
29/06/2021	CE	SOBRAL	HOSPITA L REGIONA	-	Enviada	Eventos Adversos Relaciona	Lesão por pressão		
29/06/2021	CE	SOBRAL	HOSPITA L REGIONA	-	Enviada	Eventos Adversos Relaciona	Lesão por pressão		
29/06/2021	CE	SOBRAL	HOSPITA L REGIONA	-	Enviada	Eventos Adversos Relaciona	Lesão por pressão		
29/06/2021	CE	SOBRAL	HOSPITA L REGIONA	-	Enviada	Eventos Adversos Relaciona	Lesão por pressão		
29/06/2021	CE	SOBRAL	HOSPITA L REGIONA	-	Enviada	Eventos Adversos Relaciona	Lesão por pressão		
29/06/2021	CE	SOBRAL	HOSPITA L REGIONA	-	Enviada	Eventos Adversos Relaciona	Lesão por pressão		
29/06/2021	CE	SOBRAL	HOSPITA L REGIONA	-	Enviada	Eventos Adversos Relaciona	Falhas nas atividades		
29/06/2021	CE	SOBRAL	HOSPITA L REGIONA	-	Enviada	Eventos Adversos Relaciona	Falhas durante a assistênci		
29/06/2021	CE	SOBRAL	HOSPITA L REGIONA	-	Enviada	Eventos Adversos Relaciona	Falhas durante a assistênci		

29/06/2021	CE	SOBRAL	HOSPITA L REGIONA	-	Enviada	Eventos Adversos Relaciona	Falhas envolvend o cateter		
01/07/2021	CE	SOBRAL	HOSPITA L REGIONA	-	Enviada	Eventos Adversos Relaciona	Falhas durante a assistênci		
01/07/2021	CE	SOBRAL	HOSPITA L REGIONA	-	Enviada	Eventos Adversos Relaciona	Falhas durante a assistênci		
01/07/2021	CE	SOBRAL	HOSPITA L REGIONA	-	Enviada	Eventos Adversos Relaciona	Falhas envolvend o cateter		
01/07/2021	CE	SOBRAL	HOSPITA L REGIONA	-	Enviada	Eventos Adversos Relaciona	Falhas durante a assistênci		
01/07/2021	CE	SOBRAL	HOSPITA L REGIONA	-	Enviada	Eventos Adversos Relaciona	Falhas durante a assistênci		
01/07/2021	CE	SOBRAL	HOSPITA L REGIONA	-	Enviada	Eventos Adversos Relaciona	Falhas durante a assistênci		
01/07/2021	CE	SOBRAL	HOSPITA L REGIONA	-	Enviada	Eventos Adversos Relaciona	Falhas envolvend o cateter		
01/07/2021	CE	SOBRAL	HOSPITA L REGIONA	-	Enviada	Eventos Adversos Relaciona	Falhas envolvend o cateter		
01/07/2021	CE	SOBRAL	HOSPITA L REGIONA	-	Enviada	Eventos Adversos Relaciona	Falhas envolvend o cateter		
01/07/2021	CE	SOBRAL	HOSPITA L REGIONA	-	Enviada	Eventos Adversos Relaciona	Falhas nas atividades		
01/07/2021	CE	SOBRAL	HOSPITA L REGIONA	-	Enviada	Eventos Adversos Relaciona	Falhas na administra ção de		Enteral (oral, cateter
01/07/2021	CE	SOBRAL	HOSPITA L REGIONA	-	Enviada	Eventos Adversos Relaciona	Falhas envolvend o cateter		
01/07/2021	CE	SOBRAL	HOSPITA L REGIONA	-	Enviada	Eventos Adversos Relaciona	Falhas nas atividades		
01/07/2021	CE	SOBRAL	HOSPITA L REGIONA	-	Enviada	Eventos Adversos Relaciona	Falhas envolvend o cateter		
01/07/2021	CE	SOBRAL	HOSPITA L REGIONA	-	Enviada	Eventos Adversos Relaciona	Falhas envolvend o cateter		
01/07/2021	CE	SOBRAL	HOSPITA L REGIONA	-	Enviada	Eventos Adversos Relaciona	Falhas durante a assistênci		
01/07/2021	CE	SOBRAL	HOSPITA L REGIONA	-	Enviada	Eventos Adversos Relaciona	Falhas durante a assistênci		
01/07/2021	CE	SOBRAL	HOSPITA L REGIONA	-	Enviada	Eventos Adversos Relaciona	Falhas durante a assistênci		

01/07/2021	CE	SOBRAL	HOSPITA L REGIONA	-	Enviada	Eventos Adversos Relaciona	Falhas durante a assistênci		
01/07/2021	CE	SOBRAL	HOSPITA L REGIONA	-	Enviada	Eventos Adversos Relaciona	Falhas na administra ção de		Enteral (oral, cateter
01/07/2021	CE	SOBRAL	HOSPITA L REGIONA	-	Enviada	Eventos Adversos Relaciona	Falhas envolvend o cateter		
01/07/2021	CE	SOBRAL	HOSPITA L REGIONA	-	Enviada	Eventos Adversos Relaciona	Falhas durante a assistênci		
01/07/2021	CE	SOBRAL	HOSPITA L REGIONA	-	Enviada	Eventos Adversos Relaciona	Falhas na administra ção de		Enteral (oral, cateter
01/07/2021	CE	SOBRAL	HOSPITA L REGIONA	-	Enviada	Eventos Adversos Relaciona	Falhas durante a assistênci		
01/07/2021	CE	SOBRAL	HOSPITA L REGIONA	-	Enviada	Eventos Adversos Relaciona	Falhas na administra ção de		Enteral (oral, cateter
01/07/2021	CE	SOBRAL	HOSPITA L REGIONA	-	Enviada	Eventos Adversos Relaciona	Falhas envolvend o cateter		
01/07/2021	CE	SOBRAL	HOSPITA L REGIONA	-	Enviada	Eventos Adversos Relaciona	Falhas envolvend o cateter		
01/07/2021	CE	SOBRAL	HOSPITA L REGIONA	-	Enviada	Eventos Adversos Relaciona	Queda do paciente		
01/07/2021	CE	SOBRAL	HOSPITA L REGIONA	-	Enviada	Eventos Adversos Relaciona	Queda do paciente		
01/07/2021	CE	SOBRAL	HOSPITA L REGIONA	-	Enviada	Eventos Adversos Relaciona	Falhas envolvend o cateter		
01/07/2021	CE	SOBRAL	HOSPITA L REGIONA	-	Enviada	Eventos Adversos Relaciona	Falhas na administra ção de		Enteral (oral, cateter
01/07/2021	CE	SOBRAL	HOSPITA L REGIONA	-	Enviada	Eventos Adversos Relaciona	Falhas na administra ção de		Enteral (oral, cateter
01/07/2021	CE	SOBRAL	HOSPITA L REGIONA	-	Enviada	Eventos Adversos Relaciona	Falhas na administra ção de		Enteral (oral, cateter
01/07/2021	CE	SOBRAL	HOSPITA L REGIONA	-	Enviada	Eventos Adversos Relaciona	Falhas durante a assistênci		
01/07/2021	CE	SOBRAL	HOSPITA L REGIONA	-	Enviada	Eventos Adversos Relaciona	Queda do paciente		
01/07/2021	CE	SOBRAL	HOSPITA L REGIONA	-	Enviada	Eventos Adversos Relaciona	Queda do paciente		
01/07/2021	CE	SOBRAL	HOSPITA L REGIONA	-	Enviada	Eventos Adversos Relaciona	Falhas envolvend o cateter		

01/07/2021	CE	SOBRAL	HOSPITA L REGIONA	-	Enviada	Eventos Adversos Relaciona	Falhas envolvend o cateter		
01/07/2021	CE	SOBRAL	HOSPITA L REGIONA	-	Enviada	Eventos Adversos Relaciona	Falhas durante a assistênci		
05/07/2021	CE	SOBRAL	HOSPITA L REGIONA	-	Enviada	Eventos Adversos Relaciona	Lesão por pressão		
05/07/2021	CE	SOBRAL	HOSPITA L REGIONA	-	Enviada	Eventos Adversos Relaciona	Lesão por pressão		
05/07/2021	CE	SOBRAL	HOSPITA L REGIONA	-	Enviada	Eventos Adversos Relaciona	Lesão por pressão		
05/07/2021	CE	SOBRAL	HOSPITA L REGIONA	-	Enviada	Eventos Adversos Relaciona	Lesão por pressão		
05/07/2021	CE	SOBRAL	HOSPITA L REGIONA	-	Enviada	Eventos Adversos Relaciona	Lesão por pressão		
07/07/2021	CE	SOBRAL	HOSPITA L REGIONA	-	Enviada	Eventos Adversos Relaciona	Lesão por pressão		
07/07/2021	CE	SOBRAL	HOSPITA L REGIONA	-	Enviada	Eventos Adversos Relaciona	Lesão por pressão		
07/07/2021	CE	SOBRAL	HOSPITA L REGIONA	-	Enviada	Eventos Adversos Relaciona	Falhas envolvend o cateter		
07/07/2021	CE	SOBRAL	HOSPITA L REGIONA	-	Enviada	Eventos Adversos Relaciona	Falhas envolvend o cateter		
07/07/2021	CE	SOBRAL	HOSPITA L REGIONA	-	Enviada	Eventos Adversos Relaciona	Falhas durante a assistênci		
07/07/2021	CE	SOBRAL	HOSPITA L REGIONA	-	Enviada	Eventos Adversos Relaciona	Falhas na administra ção de		Enteral (oral, cateter
07/07/2021	CE	SOBRAL	HOSPITA L REGIONA	-	Enviada	Eventos Adversos Relaciona	Falhas envolvend o cateter		
07/07/2021	CE	SOBRAL	HOSPITA L REGIONA	-	Enviada	Eventos Adversos Relaciona	Falhas nas atividades		
07/07/2021	CE	SOBRAL	HOSPITA L REGIONA	-	Enviada	Eventos Adversos Relaciona	Falhas durante a assistênci		
07/07/2021	CE	SOBRAL	HOSPITA L REGIONA	-	Enviada	Eventos Adversos Relaciona	Falhas durante a assistênci		
07/07/2021	CE	SOBRAL	HOSPITA L REGIONA	-	Enviada	Eventos Adversos Relaciona	Falhas na administra ção de		Enteral (oral, cateter
07/07/2021	CE	SOBRAL	HOSPITA L REGIONA	-	Enviada	Eventos Adversos Relaciona	Queda do paciente		

07/07/2021	CE	SOBRAL	HOSPITA L REGIONA	-	Enviada	Eventos Adversos Relaciona	Falhas envolvend o cateter		
07/07/2021	CE	SOBRAL	HOSPITA L REGIONA	-	Enviada	Eventos Adversos Relaciona	Falhas envolvend o cateter		
07/07/2021	CE	SOBRAL	HOSPITA L REGIONA	-	Enviada	Eventos Adversos Relaciona	Falhas envolvend o cateter		
07/07/2021	CE	SOBRAL	HOSPITA L REGIONA	-	Enviada	Eventos Adversos Relaciona	Falhas durante a assistênci		
07/07/2021	CE	SOBRAL	HOSPITA L REGIONA	-	Enviada	Eventos Adversos Relaciona	Falhas envolvend o cateter		
07/07/2021	CE	SOBRAL	HOSPITA L REGIONA	-	Enviada	Eventos Adversos Relaciona	Falhas durante a assistênci		
07/07/2021	CE	SOBRAL	HOSPITA L REGIONA	-	Enviada	Eventos Adversos Relaciona	Falhas durante a assistênci		
08/07/2021	CE	SOBRAL	HOSPITA L REGIONA	-	Enviada	Eventos Adversos Relaciona	Falha na identificaç ão do		
08/07/2021	CE	SOBRAL	HOSPITA L REGIONA	-	Enviada	Eventos Adversos Relaciona	Falhas durante a assistênci		
08/07/2021	CE	SOBRAL	HOSPITA L REGIONA	-	Enviada	Eventos Adversos Relaciona	Falhas durante a assistênci		
08/07/2021	CE	SOBRAL	HOSPITA L REGIONA	-	Enviada	Eventos Adversos Relaciona	Falhas envolvend o cateter		
08/07/2021	CE	SOBRAL	HOSPITA L REGIONA	-	Enviada	Eventos Adversos Relaciona	Falhas envolvend o cateter		
08/07/2021	CE	SOBRAL	HOSPITA L REGIONA	-	Enviada	Eventos Adversos Relaciona	Queimadu ra de paciente		
08/07/2021	CE	SOBRAL	HOSPITA L REGIONA	-	Enviada	Eventos Adversos Relaciona	Falhas na administra ção de		Enteral (oral, cateter
08/07/2021	CE	SOBRAL	HOSPITA L REGIONA	-	Enviada	Eventos Adversos Relaciona	Queda do paciente		
08/07/2021	CE	SOBRAL	HOSPITA L REGIONA	-	Enviada	Eventos Adversos Relaciona	Falhas envolvend o cateter		
08/07/2021	CE	SOBRAL	HOSPITA L REGIONA	-	Enviada	Eventos Adversos Relaciona	Falhas envolvend o cateter		
08/07/2021	CE	SOBRAL	HOSPITA L REGIONA	-	Enviada	Eventos Adversos Relaciona	Falha na identificaç ão do		
08/07/2021	CE	SOBRAL	HOSPITA L REGIONA	-	Enviada	Eventos Adversos Relaciona	Falhas envolvend o cateter		

08/07/2021	CE	SOBRAL	HOSPITA L REGIONA	-	Enviada	Eventos Adversos Relaciona	Queda do paciente		
08/07/2021	CE	SOBRAL	HOSPITA L REGIONA	-	Enviada	Eventos Adversos Relaciona	Falhas durante a assistênci		
08/07/2021	CE	SOBRAL	HOSPITA L REGIONA	-	Enviada	Eventos Adversos Relaciona	Falhas na administra ção de		Enteral (oral, cateter
08/07/2021	CE	SOBRAL	HOSPITA L REGIONA	-	Enviada	Eventos Adversos Relaciona	Falhas durante a assistênci		
13/07/2021	CE	SOBRAL	HOSPITA L REGIONA	-	Enviada	Eventos Adversos Relaciona	Lesão por pressão		
13/07/2021	CE	SOBRAL	HOSPITA L REGIONA	-	Enviada	Eventos Adversos Relaciona	Lesão por pressão		
13/07/2021	CE	SOBRAL	HOSPITA L REGIONA	-	Enviada	Eventos Adversos Relaciona	Lesão por pressão		
13/07/2021	CE	SOBRAL	HOSPITA L REGIONA	-	Enviada	Eventos Adversos Relaciona	Lesão por pressão		
13/07/2021	CE	SOBRAL	HOSPITA L REGIONA	-	Enviada	Eventos Adversos Relaciona	Lesão por pressão		
13/07/2021	CE	SOBRAL	HOSPITA L REGIONA	-	Enviada	Eventos Adversos Relaciona	Lesão por pressão		
13/07/2021	CE	SOBRAL	HOSPITA L REGIONA	-	Enviada	Eventos Adversos Relaciona	Lesão por pressão		
13/07/2021	CE	SOBRAL	HOSPITA L REGIONA	-	Enviada	Eventos Adversos Relaciona	Lesão por pressão		
13/07/2021	CE	SOBRAL	HOSPITA L REGIONA	-	Enviada	Eventos Adversos Relaciona	Lesão por pressão		
13/07/2021	CE	SOBRAL	HOSPITA L REGIONA	-	Enviada	Eventos Adversos Relaciona	Lesão por pressão		
13/07/2021	CE	SOBRAL	HOSPITA L REGIONA	-	Enviada	Eventos Adversos Relaciona	Lesão por pressão		
13/07/2021	CE	SOBRAL	HOSPITA L REGIONA	-	Enviada	Eventos Adversos Relaciona	Lesão por pressão		
13/07/2021	CE	SOBRAL	HOSPITA L REGIONA	-	Enviada	Eventos Adversos Relaciona	Lesão por pressão		
13/07/2021	CE	SOBRAL	HOSPITA L REGIONA	-	Enviada	Eventos Adversos Relaciona	Lesão por pressão		
13/07/2021	CE	SOBRAL	HOSPITA L REGIONA	-	Enviada	Eventos Adversos Relaciona	Lesão por pressão		

13/07/2021	CE	SOBRAL	HOSPITA L REGIONA	-	Enviada	Eventos Adversos Relaciona	Lesão por pressão		
13/07/2021	CE	SOBRAL	HOSPITA L REGIONA	-	Enviada	Eventos Adversos Relaciona	Lesão por pressão		
13/07/2021	CE	SOBRAL	HOSPITA L REGIONA	-	Enviada	Eventos Adversos Relaciona	Lesão por pressão		
14/07/2021	CE	SOBRAL	HOSPITA L REGIONA	-	Enviada	Eventos Adversos Relaciona	Lesão por pressão		
14/07/2021	CE	SOBRAL	HOSPITA L REGIONA	-	Enviada	Eventos Adversos Relaciona	Lesão por pressão		
14/07/2021	CE	SOBRAL	HOSPITA L REGIONA	-	Enviada	Eventos Adversos Relaciona	Lesão por pressão		
14/07/2021	CE	SOBRAL	HOSPITA L REGIONA	-	Enviada	Eventos Adversos Relaciona	Lesão por pressão		
14/07/2021	CE	SOBRAL	HOSPITA L REGIONA	-	Enviada	Eventos Adversos Relaciona	Lesão por pressão		
14/07/2021	CE	SOBRAL	HOSPITA L REGIONA	-	Enviada	Eventos Adversos Relaciona	Lesão por pressão		
14/07/2021	CE	SOBRAL	HOSPITA L REGIONA	-	Enviada	Eventos Adversos Relaciona	Lesão por pressão		
14/07/2021	CE	SOBRAL	HOSPITA L REGIONA	-	Enviada	Eventos Adversos Relaciona	Lesão por pressão		
14/07/2021	CE	SOBRAL	HOSPITA L REGIONA	-	Enviada	Eventos Adversos Relaciona	Lesão por pressão		
14/07/2021	CE	SOBRAL	HOSPITA L REGIONA	-	Enviada	Eventos Adversos Relaciona	Lesão por pressão		
14/07/2021	CE	SOBRAL	HOSPITA L REGIONA	-	Enviada	Eventos Adversos Relaciona	Falhas na administra ção de		Enteral (oral, cateter
14/07/2021	CE	SOBRAL	HOSPITA L REGIONA	-	Enviada	Eventos Adversos Relaciona	Falhas durante a assistênci		
14/07/2021	CE	SOBRAL	HOSPITA L REGIONA	-	Enviada	Eventos Adversos Relaciona	Falhas envolvend o cateter		
15/07/2021	CE	SOBRAL	HOSPITA L REGIONA	-	Enviada	Eventos Adversos Relaciona	Falhas na administra ção de		Enteral (oral, cateter
15/07/2021	CE	SOBRAL	HOSPITA L REGIONA	-	Enviada	Eventos Adversos Relaciona	Falhas envolvend o cateter		
15/07/2021	CE	SOBRAL	HOSPITA L REGIONA	-	Enviada	Eventos Adversos Relaciona	Falhas envolvend o cateter		

15/07/2021	CE	SOBRAL	HOSPITA L REGIONA	-	Enviada	Eventos Adversos Relaciona	Falhas durante a assistênci		
15/07/2021	CE	SOBRAL	HOSPITA L REGIONA	-	Enviada	Eventos Adversos Relaciona	Falhas na administra ção de		Enteral (oral, cateter
15/07/2021	CE	SOBRAL	HOSPITA L REGIONA	-	Enviada	Eventos Adversos Relaciona	Falhas na administra ção de		Enteral (oral, cateter
15/07/2021	CE	SOBRAL	HOSPITA L REGIONA	-	Enviada	Eventos Adversos Relaciona	Falhas durante a assistênci		
15/07/2021	CE	SOBRAL	HOSPITA L REGIONA	-	Enviada	Eventos Adversos Relaciona	Falhas na administra ção de		Enteral (oral, cateter
15/07/2021	CE	SOBRAL	HOSPITA L REGIONA	-	Enviada	Eventos Adversos Relaciona	Falhas na administra ção de		Enteral (oral, cateter
15/07/2021	CE	SOBRAL	HOSPITA L REGIONA	-	Enviada	Eventos Adversos Relaciona	Falhas na administra ção de		Enteral (oral, cateter
15/07/2021	CE	SOBRAL	HOSPITA L REGIONA	-	Enviada	Eventos Adversos Relaciona	Falhas envolvend o cateter		
15/07/2021	CE	SOBRAL	HOSPITA L REGIONA	-	Enviada	Eventos Adversos Relaciona	Falhas durante a assistênci		
15/07/2021	CE	SOBRAL	HOSPITA L REGIONA	-	Enviada	Eventos Adversos Relaciona	Falhas envolvend o cateter		
15/07/2021	CE	SOBRAL	HOSPITA L REGIONA	-	Enviada	Eventos Adversos Relaciona	Falhas durante a assistênci		
15/07/2021	CE	SOBRAL	HOSPITA L REGIONA	-	Enviada	Eventos Adversos Relaciona	Falhas envolvend o cateter		
15/07/2021	CE	SOBRAL	HOSPITA L REGIONA	-	Enviada	Eventos Adversos Relaciona	Falhas envolvend o cateter		
15/07/2021	CE	SOBRAL	HOSPITA L REGIONA	-	Enviada	Eventos Adversos Relaciona	Falhas envolvend o cateter		
15/07/2021	CE	SOBRAL	HOSPITA L REGIONA	-	Enviada	Eventos Adversos Relaciona	Falhas envolvend o cateter		
15/07/2021	CE	SOBRAL	HOSPITA L REGIONA	-	Enviada	Eventos Adversos Relaciona	Falhas envolvend o cateter		
15/07/2021	CE	SOBRAL	HOSPITA L REGIONA	-	Enviada	Eventos Adversos Relaciona	Falhas envolvend o cateter		
15/07/2021	CE	SOBRAL	HOSPITA L REGIONA	-	Enviada	Eventos Adversos Relaciona	Falhas envolvend o cateter		
15/07/2021	CE	SOBRAL	HOSPITA L REGIONA	-	Enviada	Eventos Adversos Relaciona	Falhas durante a assistênci		

15/07/2021	CE	SOBRAL	HOSPITA L REGIONA	-	Enviada	Eventos Adversos Relaciona	Falhas durante a assistênci		
15/07/2021	CE	SOBRAL	HOSPITA L REGIONA	-	Enviada	Eventos Adversos Relaciona	Queda do paciente		
15/07/2021	CE	SOBRAL	HOSPITA L REGIONA	-	Enviada	Eventos Adversos Relaciona	Queda do paciente		
15/07/2021	CE	SOBRAL	HOSPITA L REGIONA	-	Enviada	Eventos Adversos Relaciona	Falhas na administra ção de		Enteral (oral, cateter
15/07/2021	CE	SOBRAL	HOSPITA L REGIONA	-	Enviada	Eventos Adversos Relaciona	Falhas durante a assistênci		
15/07/2021	CE	SOBRAL	HOSPITA L REGIONA	-	Enviada	Eventos Adversos Relaciona	Falhas durante a assistênci		
15/07/2021	CE	SOBRAL	HOSPITA L REGIONA	-	Enviada	Eventos Adversos Relaciona	Falhas na administra ção de		Enteral (oral, cateter
15/07/2021	CE	SOBRAL	HOSPITA L REGIONA	-	Enviada	Eventos Adversos Relaciona	Falhas durante a assistênci		
15/07/2021	CE	SOBRAL	HOSPITA L REGIONA	-	Enviada	Eventos Adversos Relaciona	Falhas durante procedim		
15/07/2021	CE	SOBRAL	HOSPITA L REGIONA	-	Enviada	Eventos Adversos Relaciona	Falhas na administra ção de		Enteral (oral, cateter
15/07/2021	CE	SOBRAL	HOSPITA L REGIONA	-	Enviada	Eventos Adversos Relaciona	Falhas na administra ção de		Enteral (oral, cateter
16/07/2021	CE	SOBRAL	HOSPITA L REGIONA	-	Enviada	Eventos Adversos Relaciona	Falhas durante a assistênci		
16/07/2021	CE	SOBRAL	HOSPITA L REGIONA	-	Enviada	Eventos Adversos Relaciona	Falhas durante a assistênci		
16/07/2021	CE	SOBRAL	HOSPITA L REGIONA	-	Enviada	Eventos Adversos Relaciona	Falhas envolvend o cateter		
16/07/2021	CE	SOBRAL	HOSPITA L REGIONA	-	Enviada	Eventos Adversos Relaciona	Falhas na administra ção de		Enteral (oral, cateter
16/07/2021	CE	SOBRAL	HOSPITA L REGIONA	-	Enviada	Eventos Adversos Relaciona	Falhas durante a assistênci		
16/07/2021	CE	SOBRAL	HOSPITA L REGIONA	-	Enviada	Eventos Adversos Relaciona	Falhas durante a assistênci		
16/07/2021	CE	SOBRAL	HOSPITA L REGIONA	-	Enviada	Eventos Adversos Relaciona	Falhas envolvend o cateter		
16/07/2021	CE	SOBRAL	HOSPITA L REGIONA	-	Enviada	Eventos Adversos Relaciona	Queda do paciente		

16/07/2021	CE	SOBRAL	HOSPITA L REGIONA	-	Enviada	Eventos Adversos Relaciona	Falhas envolvend o cateter		
16/07/2021	CE	SOBRAL	HOSPITA L REGIONA	-	Enviada	Eventos Adversos Relaciona	Falhas durante a assistênci		
16/07/2021	CE	SOBRAL	HOSPITA L REGIONA	-	Enviada	Eventos Adversos Relaciona	Falhas durante a assistênci		
16/07/2021	CE	SOBRAL	HOSPITA L REGIONA	-	Enviada	Eventos Adversos Relaciona	Falhas durante a assistênci		
16/07/2021	CE	SOBRAL	HOSPITA L REGIONA	-	Enviada	Eventos Adversos Relaciona	Falhas na administra ção de		Enteral (oral, cateter
16/07/2021	CE	SOBRAL	HOSPITA L REGIONA	-	Enviada	Eventos Adversos Relaciona	Queda do paciente		
16/07/2021	CE	SOBRAL	HOSPITA L REGIONA	-	Enviada	Eventos Adversos Relaciona	Queda do paciente		
16/07/2021	CE	SOBRAL	HOSPITA L REGIONA	-	Enviada	Eventos Adversos Relaciona	Falhas durante a assistênci		
16/07/2021	CE	SOBRAL	HOSPITA L REGIONA	-	Enviada	Eventos Adversos Relaciona	Falhas nas atividades		
16/07/2021	CE	SOBRAL	HOSPITA L REGIONA	-	Enviada	Eventos Adversos Relaciona	Falhas na administra ção de		Enteral (oral, cateter
16/07/2021	CE	SOBRAL	HOSPITA L REGIONA	-	Enviada	Eventos Adversos Relaciona	Falhas durante a assistênci		
16/07/2021	CE	SOBRAL	HOSPITA L REGIONA	-	Enviada	Eventos Adversos Relaciona	Falhas na administra ção de		Enteral (oral, cateter
16/07/2021	CE	SOBRAL	HOSPITA L REGIONA	-	Enviada	Eventos Adversos Relaciona	Falhas na administra ção de		Enteral (oral, cateter
16/07/2021	CE	SOBRAL	HOSPITA L REGIONA	-	Enviada	Eventos Adversos Relaciona	Falhas durante a assistênci		
16/07/2021	CE	SOBRAL	HOSPITA L REGIONA	-	Enviada	Eventos Adversos Relaciona	Queda do paciente		
16/07/2021	CE	SOBRAL	HOSPITA L REGIONA	-	Enviada	Eventos Adversos Relaciona	Queda do paciente		
16/07/2021	CE	SOBRAL	HOSPITA L REGIONA	-	Enviada	Eventos Adversos Relaciona	Falhas envolvend o cateter		
19/07/2021	CE	SOBRAL	HOSPITA L REGIONA	-	Enviada	Eventos Adversos Relaciona	Queda do paciente		
19/07/2021	CE	SOBRAL	HOSPITA L REGIONA	-	Enviada	Eventos Adversos Relaciona	Falhas durante a assistênci		

06/08/2021	CE	SOBRAL	HOSPITA L REGIONA	-	Enviada	Eventos Adversos Relaciona	Falhas na administra ção de		Enteral (oral, cateter
06/08/2021	CE	SOBRAL	HOSPITA L REGIONA	-	Enviada	Eventos Adversos Relaciona	Falhas durante a assistênci		
06/08/2021	CE	SOBRAL	HOSPITA L REGIONA	-	Enviada	Eventos Adversos Relaciona	Falhas durante a assistênci		
06/08/2021	CE	SOBRAL	HOSPITA L REGIONA	-	Enviada	Eventos Adversos Relaciona	Lesão por pressão		
06/08/2021	CE	SOBRAL	HOSPITA L REGIONA	-	Enviada	Eventos Adversos Relaciona	Lesão por pressão		
06/08/2021	CE	SOBRAL	HOSPITA L REGIONA	-	Enviada	Eventos Adversos Relaciona	Queda do paciente		
06/08/2021	CE	SOBRAL	HOSPITA L REGIONA	-	Enviada	Eventos Adversos Relaciona	Falhas envolvend o cateter		
06/08/2021	CE	SOBRAL	HOSPITA L REGIONA	-	Enviada	Eventos Adversos Relaciona	Falhas durante a assistênci		
06/08/2021	CE	SOBRAL	HOSPITA L REGIONA	-	Enviada	Eventos Adversos Relaciona	Falhas na administra ção de		Enteral (oral, cateter
06/08/2021	CE	SOBRAL	HOSPITA L REGIONA	-	Enviada	Eventos Adversos Relaciona	Lesão por pressão		
06/08/2021	CE	SOBRAL	HOSPITA L REGIONA	-	Enviada	Eventos Adversos Relaciona	Queda do paciente		
06/08/2021	CE	SOBRAL	HOSPITA L REGIONA	-	Enviada	Eventos Adversos Relaciona	Lesão por pressão		
06/08/2021	CE	SOBRAL	HOSPITA L REGIONA	-	Enviada	Eventos Adversos Relaciona	Falhas na administra ção de		Enteral (oral, cateter
06/08/2021	CE	SOBRAL	HOSPITA L REGIONA	-	Enviada	Eventos Adversos Relaciona	Lesão por pressão		
06/08/2021	CE	SOBRAL	HOSPITA L REGIONA	-	Enviada	Eventos Adversos Relaciona	Falhas envolvend o cateter		
06/08/2021	CE	SOBRAL	HOSPITA L REGIONA	-	Enviada	Eventos Adversos Relaciona	Lesão por pressão		
06/08/2021	CE	SOBRAL	HOSPITA L REGIONA	-	Enviada	Eventos Adversos Relaciona	Falhas na administra ção de		Enteral (oral, cateter
06/08/2021	CE	SOBRAL	HOSPITA L REGIONA	-	Enviada	Eventos Adversos Relaciona	Queda do paciente		
06/08/2021	CE	SOBRAL	HOSPITA L REGIONA	-	Enviada	Eventos Adversos Relaciona	Queda do paciente		

13/08/2021	CE	SOBRAL	HOSPITAL REGIONA	-	Enviada	Eventos Adversos Relaciona	Lesão por pressão		
13/08/2021	CE	SOBRAL	HOSPITAL REGIONA	-	Enviada	Eventos Adversos Relaciona	Falhas durante a assistênci		
13/08/2021	CE	SOBRAL	HOSPITAL REGIONA	-	Enviada	Eventos Adversos Relaciona	Falhas envolvendo cateter		
13/08/2021	CE	SOBRAL	HOSPITAL REGIONA	-	Enviada	Eventos Adversos Relaciona	Lesão por pressão		
13/08/2021	CE	SOBRAL	HOSPITAL REGIONA	-	Enviada	Eventos Adversos Relaciona	Lesão por pressão		
13/08/2021	CE	SOBRAL	HOSPITAL REGIONA	-	Enviada	Eventos Adversos Relaciona	Lesão por pressão		
13/08/2021	CE	SOBRAL	HOSPITAL REGIONA	-	Enviada	Eventos Adversos Relaciona	Queimadura de paciente		
13/08/2021	CE	SOBRAL	HOSPITAL REGIONA	-	Enviada	Eventos Adversos Relaciona	Falhas durante a assistênci		
13/08/2021	CE	SOBRAL	HOSPITAL REGIONA	-	Enviada	Eventos Adversos Relaciona	Falhas durante a assistênci		
13/08/2021	CE	SOBRAL	HOSPITAL REGIONA	-	Enviada	Eventos Adversos Relaciona	Falhas durante a assistênci		
13/08/2021	CE	SOBRAL	HOSPITAL REGIONA	-	Enviada	Eventos Adversos Relaciona	Lesão por pressão		
13/08/2021	CE	SOBRAL	HOSPITAL REGIONA	-	Enviada	Eventos Adversos Relaciona	Lesão por pressão		
13/08/2021	CE	SOBRAL	HOSPITAL REGIONA	-	Enviada	Eventos Adversos Relaciona	Lesão por pressão		
13/08/2021	CE	SOBRAL	HOSPITAL REGIONA	-	Enviada	Eventos Adversos Relaciona	Lesão por pressão		
13/08/2021	CE	SOBRAL	HOSPITAL REGIONA	-	Enviada	Eventos Adversos Relaciona	Falhas na administração de		Enteral (oral, cateter
13/08/2021	CE	SOBRAL	HOSPITAL REGIONA	-	Enviada	Eventos Adversos Relaciona	Queda do paciente		
13/08/2021	CE	SOBRAL	HOSPITAL REGIONA	-	Enviada	Eventos Adversos Relaciona	Falhas envolvendo cateter		
13/08/2021	CE	SOBRAL	HOSPITAL REGIONA	-	Enviada	Eventos Adversos Relaciona	Falhas envolvendo cateter		
13/08/2021	CE	SOBRAL	HOSPITAL REGIONA	-	Enviada	Eventos Adversos Relaciona	Falhas durante a assistênci		

13/08/2021	CE	SOBRAL	HOSPITAL REGIONA	-	Enviada	Eventos Adversos Relaciona	Falhas durante a assistênci		
13/08/2021	CE	SOBRAL	HOSPITAL REGIONA	-	Enviada	Eventos Adversos Relaciona	Queda do paciente		
13/08/2021	CE	SOBRAL	HOSPITAL REGIONA	-	Enviada	Eventos Adversos Relaciona	Lesão por pressão		
13/08/2021	CE	SOBRAL	HOSPITAL REGIONA	-	Enviada	Eventos Adversos Relaciona	Lesão por pressão		
13/08/2021	CE	SOBRAL	HOSPITAL REGIONA	-	Enviada	Eventos Adversos Relaciona	Lesão por pressão		
13/08/2021	CE	SOBRAL	HOSPITAL REGIONA	-	Enviada	Eventos Adversos Relaciona	Falhas envolvendo cateter		
13/08/2021	CE	SOBRAL	HOSPITAL REGIONA	-	Enviada	Eventos Adversos Relaciona	Falhas envolvendo cateter		
13/08/2021	CE	SOBRAL	HOSPITAL REGIONA	-	Enviada	Eventos Adversos Relaciona	Falhas durante a assistênci		
13/08/2021	CE	SOBRAL	HOSPITAL REGIONA	-	Enviada	Eventos Adversos Relaciona	Falhas durante a assistênci		
16/08/2021	CE	SOBRAL	HOSPITAL REGIONA	-	Enviada	Eventos Adversos Relaciona	Falhas durante a assistênci		
16/08/2021	CE	SOBRAL	HOSPITAL REGIONA	-	Enviada	Eventos Adversos Relaciona	Falhas durante a assistênci		
16/08/2021	CE	SOBRAL	HOSPITAL REGIONA	-	Enviada	Eventos Adversos Relaciona	Falhas durante a assistênci		
16/08/2021	CE	SOBRAL	HOSPITAL REGIONA	-	Enviada	Eventos Adversos Relaciona	Falhas durante a assistênci		
16/08/2021	CE	SOBRAL	HOSPITAL REGIONA	-	Enviada	Eventos Adversos Relaciona	Falhas durante a assistênci		
16/08/2021	CE	SOBRAL	HOSPITAL REGIONA	-	Enviada	Eventos Adversos Relaciona	Falhas durante a assistênci		
16/08/2021	CE	SOBRAL	HOSPITAL REGIONA	-	Enviada	Eventos Adversos Relaciona	Lesão por pressão		
16/08/2021	CE	SOBRAL	HOSPITAL REGIONA	-	Enviada	Eventos Adversos Relaciona	Lesão por pressão		
17/08/2021	CE	SOBRAL	HOSPITAL REGIONA	-	Enviada	Eventos Adversos Relaciona	Falhas durante a assistênci		
17/08/2021	CE	SOBRAL	HOSPITAL REGIONA	-	Enviada	Eventos Adversos Relaciona	Queda do paciente		

17/08/2021	CE	SOBRAL	HOSPITA L REGIONA	-	Enviada	Eventos Adversos Relaciona	Falhas durante a assistênci		
17/08/2021	CE	SOBRAL	HOSPITA L REGIONA	-	Enviada	Eventos Adversos Relaciona	Falhas envolvend o cateter		
17/08/2021	CE	SOBRAL	HOSPITA L REGIONA	-	Enviada	Eventos Adversos Relaciona	Falhas durante a assistênci		
17/08/2021	CE	SOBRAL	HOSPITA L REGIONA	-	Enviada	Eventos Adversos Relaciona	Falhas envolvend o cateter		
25/08/2021	CE	SOBRAL	HOSPITA L REGIONA	-	Enviada	Eventos Adversos Relaciona	Lesão por pressão		
25/08/2021	CE	SOBRAL	HOSPITA L REGIONA	-	Enviada	Eventos Adversos Relaciona	Lesão por pressão		
25/08/2021	CE	SOBRAL	HOSPITA L REGIONA	-	Enviada	Eventos Adversos Relaciona	Lesão por pressão		
25/08/2021	CE	SOBRAL	HOSPITA L REGIONA	-	Enviada	Eventos Adversos Relaciona	Lesão por pressão		
25/08/2021	CE	SOBRAL	HOSPITA L REGIONA	-	Enviada	Eventos Adversos Relaciona	Lesão por pressão		
25/08/2021	CE	SOBRAL	HOSPITA L REGIONA	-	Enviada	Eventos Adversos Relaciona	Lesão por pressão		
25/08/2021	CE	SOBRAL	HOSPITA L REGIONA	-	Enviada	Eventos Adversos Relaciona	Lesão por pressão		
25/08/2021	CE	SOBRAL	HOSPITA L REGIONA	-	Enviada	Eventos Adversos Relaciona	Falhas envolvend o cateter		
25/08/2021	CE	SOBRAL	HOSPITA L REGIONA	-	Enviada	Eventos Adversos Relaciona	Falhas envolvend o cateter		
25/08/2021	CE	SOBRAL	HOSPITA L REGIONA	-	Enviada	Eventos Adversos Relaciona	Queda do paciente		
25/08/2021	CE	SOBRAL	HOSPITA L REGIONA	-	Enviada	Eventos Adversos Relaciona	Queda do paciente		
26/08/2021	CE	SOBRAL	HOSPITA L REGIONA	-	Enviada	Eventos Adversos Relaciona	Falhas durante a assistênci		
26/08/2021	CE	SOBRAL	HOSPITA L REGIONA	-	Enviada	Eventos Adversos Relaciona	Falhas durante a assistênci		
26/08/2021	CE	SOBRAL	HOSPITA L REGIONA	-	Enviada	Eventos Adversos Relaciona	Falhas durante a assistênci		
26/08/2021	CE	SOBRAL	HOSPITA L REGIONA	-	Enviada	Eventos Adversos Relaciona	Falhas envolvend o cateter		

26/08/2021	CE	SOBRAL	HOSPITA L REGIONA	-	Enviada	Eventos Adversos Relaciona	Queda do paciente		
26/08/2021	CE	SOBRAL	HOSPITA L REGIONA	-	Enviada	Eventos Adversos Relaciona	Falhas envolvend o cateter		
02/09/2021	CE	SOBRAL	HOSPITA L REGIONA	-	Enviada	Eventos Adversos Relaciona	Lesão por pressão		
02/09/2021	CE	SOBRAL	HOSPITA L REGIONA	-	Enviada	Eventos Adversos Relaciona	Lesão por pressão		
02/09/2021	CE	SOBRAL	HOSPITA L REGIONA	-	Enviada	Eventos Adversos Relaciona	Lesão por pressão		
02/09/2021	CE	SOBRAL	HOSPITA L REGIONA	-	Enviada	Eventos Adversos Relaciona	Lesão por pressão		
02/09/2021	CE	SOBRAL	HOSPITA L REGIONA	-	Enviada	Eventos Adversos Relaciona	Lesão por pressão		
06/10/2021	CE	SOBRAL	HOSPITA L REGIONA	-	Enviada	Eventos Adversos Relaciona	Queda do paciente		
06/10/2021	CE	SOBRAL	HOSPITA L REGIONA	-	Enviada	Eventos Adversos Relaciona	Queda do paciente		
06/10/2021	CE	SOBRAL	HOSPITA L REGIONA	-	Enviada	Eventos Adversos Relaciona	Falhas durante a assistênci		
06/10/2021	CE	SOBRAL	HOSPITA L REGIONA	-	Enviada	Eventos Adversos Relaciona	Falhas envolvend o cateter		
06/10/2021	CE	SOBRAL	HOSPITA L REGIONA	-	Enviada	Eventos Adversos Relaciona	Falhas envolvend o cateter		
06/10/2021	CE	SOBRAL	HOSPITA L REGIONA	-	Enviada	Eventos Adversos Relaciona	Falhas durante a assistênci		
06/10/2021	CE	SOBRAL	HOSPITA L REGIONA	-	Enviada	Eventos Adversos Relaciona	Falhas envolvend o cateter		
06/10/2021	CE	SOBRAL	HOSPITA L REGIONA	-	Enviada	Eventos Adversos Relaciona	Lesão por pressão		
06/10/2021	CE	SOBRAL	HOSPITA L REGIONA	-	Enviada	Eventos Adversos Relaciona	Queda do paciente		
06/10/2021	CE	SOBRAL	HOSPITA L REGIONA	-	Enviada	Eventos Adversos Relaciona	Queda do paciente		
06/10/2021	CE	SOBRAL	HOSPITA L REGIONA	-	Enviada	Eventos Adversos Relaciona	Lesão por pressão		
06/10/2021	CE	SOBRAL	HOSPITA L REGIONA	-	Enviada	Eventos Adversos Relaciona	Lesão por pressão		

06/10/2021	CE	SOBRAL	HOSPITA L REGIONA	-	Enviada	Eventos Adversos Relaciona	Falhas envolvend o cateter		
06/10/2021	CE	SOBRAL	HOSPITA L REGIONA	-	Enviada	Eventos Adversos Relaciona	Lesão por pressão		
06/10/2021	CE	SOBRAL	HOSPITA L REGIONA	-	Enviada	Eventos Adversos Relaciona	Lesão por pressão		
06/10/2021	CE	SOBRAL	HOSPITA L REGIONA	-	Enviada	Eventos Adversos Relaciona	Queda do paciente		
06/10/2021	CE	SOBRAL	HOSPITA L REGIONA	-	Enviada	Eventos Adversos Relaciona	Queda do paciente		
06/10/2021	CE	SOBRAL	HOSPITA L REGIONA	-	Enviada	Eventos Adversos Relaciona	Queda do paciente		
06/10/2021	CE	SOBRAL	HOSPITA L REGIONA	-	Enviada	Eventos Adversos Relaciona	Queda do paciente		
06/10/2021	CE	SOBRAL	HOSPITA L REGIONA	-	Enviada	Eventos Adversos Relaciona	Lesão por pressão		
06/10/2021	CE	SOBRAL	HOSPITA L REGIONA	-	Enviada	Eventos Adversos Relaciona	Queda do paciente		
06/10/2021	CE	SOBRAL	HOSPITA L REGIONA	-	Enviada	Eventos Adversos Relaciona	Falhas durante a assistênci		
06/10/2021	CE	SOBRAL	HOSPITA L REGIONA	-	Enviada	Eventos Adversos Relaciona	Falhas envolvend o cateter		
06/10/2021	CE	SOBRAL	HOSPITA L REGIONA	-	Enviada	Eventos Adversos Relaciona	Queda do paciente		
07/10/2021	CE	SOBRAL	HOSPITA L REGIONA	-	Enviada	Eventos Adversos Relaciona	Lesão por pressão		
07/10/2021	CE	SOBRAL	HOSPITA L REGIONA	-	Enviada	Eventos Adversos Relaciona	Falhas envolvend o cateter		
07/10/2021	CE	SOBRAL	HOSPITA L REGIONA	-	Enviada	Eventos Adversos Relaciona	Falhas na administra ção de		Enteral (oral, cateter
07/10/2021	CE	SOBRAL	HOSPITA L REGIONA	-	Enviada	Eventos Adversos Relaciona	Falhas envolvend o cateter		
07/10/2021	CE	SOBRAL	HOSPITA L REGIONA	-	Enviada	Eventos Adversos Relaciona	Falhas envolvend o cateter		
07/10/2021	CE	SOBRAL	HOSPITA L REGIONA	-	Enviada	Eventos Adversos Relaciona	Queda do paciente		
07/10/2021	CE	SOBRAL	HOSPITA L REGIONA	-	Enviada	Eventos Adversos Relaciona	Falhas envolvend o sondas		

07/10/2021	CE	SOBRAL	HOSPITA L REGIONA	-	Enviada	Eventos Adversos Relaciona	Lesão por pressão		
07/10/2021	CE	SOBRAL	HOSPITA L REGIONA	-	Enviada	Eventos Adversos Relaciona	Queda do paciente		
07/10/2021	CE	SOBRAL	HOSPITA L REGIONA	-	Enviada	Eventos Adversos Relaciona	Lesão por pressão		
07/10/2021	CE	SOBRAL	HOSPITA L REGIONA	-	Enviada	Eventos Adversos Relaciona	Lesão por pressão		
07/10/2021	CE	SOBRAL	HOSPITA L REGIONA	-	Enviada	Eventos Adversos Relaciona	Falhas na administra ção de		Enteral (oral, cateter
07/10/2021	CE	SOBRAL	HOSPITA L REGIONA	-	Enviada	Eventos Adversos Relaciona	Lesão por pressão		
07/10/2021	CE	SOBRAL	HOSPITA L REGIONA	-	Enviada	Eventos Adversos Relaciona	Lesão por pressão		
07/10/2021	CE	SOBRAL	HOSPITA L REGIONA	-	Enviada	Eventos Adversos Relaciona	Falhas envolvend o cateter		
07/10/2021	CE	SOBRAL	HOSPITA L REGIONA	-	Enviada	Eventos Adversos Relaciona	Falhas durante a assistênci		
21/10/2021	CE	SOBRAL	HOSPITA L REGIONA	-	Enviada	Eventos Adversos Relaciona	Falhas envolvend o cateter		
21/10/2021	CE	SOBRAL	HOSPITA L REGIONA	-	Enviada	Eventos Adversos Relaciona	Falhas envolvend o cateter		
21/10/2021	CE	SOBRAL	HOSPITA L REGIONA	-	Enviada	Eventos Adversos Relaciona	Falhas envolvend o cateter		
21/10/2021	CE	SOBRAL	HOSPITA L REGIONA	-	Enviada	Eventos Adversos Relaciona	Falhas envolvend o cateter		
21/10/2021	CE	SOBRAL	HOSPITA L REGIONA	-	Enviada	Eventos Adversos Relaciona	Falhas envolvend o cateter		
21/10/2021	CE	SOBRAL	HOSPITA L REGIONA	-	Enviada	Eventos Adversos Relaciona	Falhas envolvend o cateter		
21/10/2021	CE	SOBRAL	HOSPITA L REGIONA	-	Enviada	Eventos Adversos Relaciona	Falhas envolvend o cateter		
21/10/2021	CE	SOBRAL	HOSPITA L REGIONA	-	Enviada	Eventos Adversos Relaciona	Falhas envolvend o cateter		
22/10/2021	CE	SOBRAL	HOSPITA L REGIONA	-	Enviada	Eventos Adversos Relaciona	Falhas envolvend o cateter		
22/10/2021	CE	SOBRAL	HOSPITA L REGIONA	-	Enviada	Eventos Adversos Relaciona	Falhas envolvend o cateter		

22/10/2021	CE	SOBRAL	HOSPITA L REGIONA	-	Enviada	Eventos Adversos Relaciona	Falhas envolvend o cateter		
22/10/2021	CE	SOBRAL	HOSPITA L REGIONA	-	Enviada	Eventos Adversos Relaciona	Falhas envolvend o cateter		
28/10/2021	CE	SOBRAL	HOSPITA L REGIONA	-	Enviada	Eventos Adversos Relaciona	Lesão por pressão		
28/10/2021	CE	SOBRAL	HOSPITA L REGIONA	-	Enviada	Eventos Adversos Relaciona	Lesão por pressão		
28/10/2021	CE	SOBRAL	HOSPITA L REGIONA	-	Enviada	Eventos Adversos Relaciona	Falha na document ação		
28/10/2021	CE	SOBRAL	HOSPITA L REGIONA	-	Enviada	Eventos Adversos Relaciona	Lesão por pressão		
28/10/2021	CE	SOBRAL	HOSPITA L REGIONA	-	Enviada	Eventos Adversos Relaciona	Lesão por pressão		
28/10/2021	CE	SOBRAL	HOSPITA L REGIONA	-	Enviada	Eventos Adversos Relaciona	Lesão por pressão		
28/10/2021	CE	SOBRAL	HOSPITA L REGIONA	-	Enviada	Eventos Adversos Relaciona	Lesão por pressão		
29/10/2021	CE	SOBRAL	HOSPITA L REGIONA	-	Enviada	Eventos Adversos Relaciona	Falhas na administra ção de		Enteral (oral, cateter
29/10/2021	CE	SOBRAL	HOSPITA L REGIONA	-	Enviada	Eventos Adversos Relaciona	Falhas envolvend o cateter		
29/10/2021	CE	SOBRAL	HOSPITA L REGIONA	-	Enviada	Eventos Adversos Relaciona	Falhas durante a assistênci		
29/10/2021	CE	SOBRAL	HOSPITA L REGIONA	-	Enviada	Eventos Adversos Relaciona	Lesão por pressão		
29/10/2021	CE	SOBRAL	HOSPITA L REGIONA	-	Enviada	Eventos Adversos Relaciona	Lesão por pressão		
29/10/2021	CE	SOBRAL	HOSPITA L REGIONA	-	Enviada	Eventos Adversos Relaciona	Falhas durante a assistênci		
29/10/2021	CE	SOBRAL	HOSPITA L REGIONA	-	Enviada	Eventos Adversos Relaciona	Falhas envolvend o cateter		
29/10/2021	CE	SOBRAL	HOSPITA L REGIONA	-	Enviada	Eventos Adversos Relaciona	Falhas durante a assistênci		
29/10/2021	CE	SOBRAL	HOSPITA L REGIONA	-	Enviada	Eventos Adversos Relaciona	Queimadu ra de paciente		
24/11/2021	CE	SOBRAL	HOSPITA L REGIONA	-	Enviada	Eventos Adversos Relaciona	Falhas na administra ção de		Enteral (oral, cateter

24/11/2021	CE	SOBRAL	HOSPITA L REGIONA	-	Enviada	Eventos Adversos Relaciona	Falhas durante a assistênci		
24/11/2021	CE	SOBRAL	HOSPITA L REGIONA	-	Enviada	Eventos Adversos Relaciona	Falhas na administra ção de		Enteral (oral, cateter
24/11/2021	CE	SOBRAL	HOSPITA L REGIONA	-	Enviada	Eventos Adversos Relaciona	Lesão por pressão		
24/11/2021	CE	SOBRAL	HOSPITA L REGIONA	-	Enviada	Eventos Adversos Relaciona	Falhas durante a assistênci		
24/11/2021	CE	SOBRAL	HOSPITA L REGIONA	-	Enviada	Eventos Adversos Relaciona	Falhas durante a assistênci		
24/11/2021	CE	SOBRAL	HOSPITA L REGIONA	-	Enviada	Eventos Adversos Relaciona	Falhas na administra ção de		Enteral (oral, cateter
24/11/2021	CE	SOBRAL	HOSPITA L REGIONA	-	Enviada	Eventos Adversos Relaciona	Falhas durante a assistênci		
24/11/2021	CE	SOBRAL	HOSPITA L REGIONA	-	Enviada	Eventos Adversos Relaciona	Falhas durante a assistênci		
24/11/2021	CE	SOBRAL	HOSPITA L REGIONA	-	Enviada	Eventos Adversos Relaciona	Falhas durante a assistênci		
24/11/2021	CE	SOBRAL	HOSPITA L REGIONA	-	Enviada	Eventos Adversos Relaciona	Falhas durante a assistênci		
24/11/2021	CE	SOBRAL	HOSPITA L REGIONA	-	Enviada	Eventos Adversos Relaciona	Falhas durante a assistênci		
24/11/2021	CE	SOBRAL	HOSPITA L REGIONA	-	Enviada	Eventos Adversos Relaciona	Queda do paciente		
24/11/2021	CE	SOBRAL	HOSPITA L REGIONA	-	Enviada	Eventos Adversos Relaciona	Falhas ocorridas em		
24/11/2021	CE	SOBRAL	HOSPITA L REGIONA	-	Enviada	Eventos Adversos Relaciona	Falhas ocorridas em		
24/11/2021	CE	SOBRAL	HOSPITA L REGIONA	-	Enviada	Eventos Adversos Relaciona	Falhas durante a assistênci		
24/11/2021	CE	SOBRAL	HOSPITA L REGIONA	-	Enviada	Eventos Adversos Relaciona	Falhas durante a assistênci		
24/11/2021	CE	SOBRAL	HOSPITA L REGIONA	-	Enviada	Eventos Adversos Relaciona	Falhas na administra ção de		Enteral (oral, cateter
24/11/2021	CE	SOBRAL	HOSPITA L REGIONA	-	Enviada	Eventos Adversos Relaciona	Falhas durante a assistênci		
24/11/2021	CE	SOBRAL	HOSPITA L REGIONA	-	Enviada	Eventos Adversos Relaciona	Falhas na administra ção de		Enteral (oral, cateter

24/11/2021	CE	SOBRAL	HOSPITA L REGIONA	-	Enviada	Eventos Adversos Relaciona	Falhas na administra ção de		Enteral (oral, cateter
24/11/2021	CE	SOBRAL	HOSPITA L REGIONA	-	Enviada	Eventos Adversos Relaciona	Falhas na administra ção de		Enteral (oral, cateter
24/11/2021	CE	SOBRAL	HOSPITA L REGIONA	-	Enviada	Eventos Adversos Relaciona	Falhas na administra ção de		Enteral (oral, cateter
24/11/2021	CE	SOBRAL	HOSPITA L REGIONA	-	Enviada	Eventos Adversos Relaciona	Falhas ocorridas em		
24/11/2021	CE	SOBRAL	HOSPITA L REGIONA	-	Enviada	Eventos Adversos Relaciona	Falhas durante a assistênci		
24/11/2021	CE	SOBRAL	HOSPITA L REGIONA	-	Enviada	Eventos Adversos Relaciona	Queda do paciente		
24/11/2021	CE	SOBRAL	HOSPITA L REGIONA	-	Enviada	Eventos Adversos Relaciona	Queda do paciente		
24/11/2021	CE	SOBRAL	HOSPITA L REGIONA	-	Enviada	Eventos Adversos Relaciona	Falhas envolvend o cateter		
25/11/2021	CE	SOBRAL	HOSPITA L REGIONA	-	Enviada	Eventos Adversos Relaciona	Falhas durante a assistênci		
25/11/2021	CE	SOBRAL	HOSPITA L REGIONA	-	Enviada	Eventos Adversos Relaciona	Falhas durante a assistênci		
25/11/2021	CE	SOBRAL	HOSPITA L REGIONA	-	Enviada	Eventos Adversos Relaciona	Falhas durante a assistênci		
25/11/2021	CE	SOBRAL	HOSPITA L REGIONA	-	Enviada	Eventos Adversos Relaciona	Falhas durante a assistênci		
25/11/2021	CE	SOBRAL	HOSPITA L REGIONA	-	Enviada	Eventos Adversos Relaciona	Falhas na administra ção de		Enteral (oral, cateter
25/11/2021	CE	SOBRAL	HOSPITA L REGIONA	-	Enviada	Eventos Adversos Relaciona	Falhas ocorridas em		
25/11/2021	CE	SOBRAL	HOSPITA L REGIONA	-	Enviada	Eventos Adversos Relaciona	Lesão por pressão		
25/11/2021	CE	SOBRAL	HOSPITA L REGIONA	-	Enviada	Eventos Adversos Relaciona	Lesão por pressão		
25/11/2021	CE	SOBRAL	HOSPITA L REGIONA	-	Enviada	Eventos Adversos Relaciona	Falhas na administra ção de		Enteral (oral, cateter
25/11/2021	CE	SOBRAL	HOSPITA L REGIONA	-	Enviada	Eventos Adversos Relaciona	Falhas durante a assistênci		
25/11/2021	CE	SOBRAL	HOSPITA L REGIONA	-	Enviada	Eventos Adversos Relaciona	Lesão por pressão		

25/11/2021	CE	SOBRAL	HOSPITA L REGIONA	-	Enviada	Eventos Adversos Relaciona	Falhas durante a assistênci		
25/11/2021	CE	SOBRAL	HOSPITA L REGIONA	-	Enviada	Eventos Adversos Relaciona	Falhas durante a assistênci		
25/11/2021	CE	SOBRAL	HOSPITA L REGIONA	-	Enviada	Eventos Adversos Relaciona	Falhas na administra ção de		Enteral (oral, cateter
25/11/2021	CE	SOBRAL	HOSPITA L REGIONA	-	Enviada	Eventos Adversos Relaciona	Falhas durante a assistênci		
25/11/2021	CE	SOBRAL	HOSPITA L REGIONA	-	Enviada	Eventos Adversos Relaciona	Lesão por pressão		
25/11/2021	CE	SOBRAL	HOSPITA L REGIONA	-	Enviada	Eventos Adversos Relaciona	Falhas na administra ção de		Enteral (oral, cateter
25/11/2021	CE	SOBRAL	HOSPITA L REGIONA	-	Enviada	Eventos Adversos Relaciona	Falhas na administra ção de		Enteral (oral, cateter
25/11/2021	CE	SOBRAL	HOSPITA L REGIONA	-	Enviada	Eventos Adversos Relaciona	Lesão por pressão		
25/11/2021	CE	SOBRAL	HOSPITA L REGIONA	-	Enviada	Eventos Adversos Relaciona	Falhas durante a assistênci		
25/11/2021	CE	SOBRAL	HOSPITA L REGIONA	-	Enviada	Eventos Adversos Relaciona	Falhas durante a assistênci		
25/11/2021	CE	SOBRAL	HOSPITA L REGIONA	-	Enviada	Eventos Adversos Relaciona	Falhas durante a assistênci		
25/11/2021	CE	SOBRAL	HOSPITA L REGIONA	-	Enviada	Eventos Adversos Relaciona	Falhas durante a assistênci		
25/11/2021	CE	SOBRAL	HOSPITA L REGIONA	-	Enviada	Eventos Adversos Relaciona	Falhas na administra ção de		Enteral (oral, cateter
25/11/2021	CE	SOBRAL	HOSPITA L REGIONA	-	Enviada	Eventos Adversos Relaciona	Falhas durante a assistênci		
25/11/2021	CE	SOBRAL	HOSPITA L REGIONA	-	Enviada	Eventos Adversos Relaciona	Falhas envolvend o cateter		
25/11/2021	CE	SOBRAL	HOSPITA L REGIONA	-	Enviada	Eventos Adversos Relaciona	Falhas envolvend o cateter		
25/11/2021	CE	SOBRAL	HOSPITA L REGIONA	-	Enviada	Eventos Adversos Relaciona	Falhas durante a assistênci		
26/11/2021	CE	SOBRAL	HOSPITA L REGIONA	-	Enviada	Eventos Adversos Relaciona	Lesão por pressão		
29/11/2021	CE	SOBRAL	HOSPITA L REGIONA	INSERÇÃ O DE CHICOTE	Concluída	Eventos Adversos Relaciona	Falhas no cuidado / proteção		

07/12/2021	CE	SOBRAL	HOSPITA L REGIONA	-	Enviada	Eventos Adversos Relaciona	Lesão por pressão		
07/12/2021	CE	SOBRAL	HOSPITA L REGIONA	-	Enviada	Eventos Adversos Relaciona	Lesão por pressão		
10/12/2021	CE	SOBRAL	HOSPITA L REGIONA	-	Enviada	Eventos Adversos Relaciona	Lesão por pressão		
10/12/2021	CE	SOBRAL	HOSPITA L REGIONA	-	Enviada	Eventos Adversos Relaciona	Lesão por pressão		
10/12/2021	CE	SOBRAL	HOSPITA L REGIONA	-	Enviada	Eventos Adversos Relaciona	Lesão por pressão		
10/12/2021	CE	SOBRAL	HOSPITA L REGIONA	-	Enviada	Eventos Adversos Relaciona	Lesão por pressão		
10/12/2021	CE	SOBRAL	HOSPITA L REGIONA	-	Enviada	Eventos Adversos Relaciona	Lesão por pressão		
10/12/2021	CE	SOBRAL	HOSPITA L REGIONA	-	Enviada	Eventos Adversos Relaciona	Lesão por pressão		
10/12/2021	CE	SOBRAL	HOSPITA L REGIONA	-	Enviada	Eventos Adversos Relaciona	Lesão por pressão		
10/12/2021	CE	SOBRAL	HOSPITA L REGIONA	-	Enviada	Eventos Adversos Relaciona	Lesão por pressão		
10/12/2021	CE	SOBRAL	HOSPITA L REGIONA	-	Enviada	Eventos Adversos Relaciona	Lesão por pressão		
10/12/2021	CE	SOBRAL	HOSPITA L REGIONA	-	Enviada	Eventos Adversos Relaciona	Lesão por pressão		
10/12/2021	CE	SOBRAL	HOSPITA L REGIONA	-	Enviada	Eventos Adversos Relaciona	Lesão por pressão		
10/12/2021	CE	SOBRAL	HOSPITA L REGIONA	-	Enviada	Eventos Adversos Relaciona	Lesão por pressão		
10/12/2021	CE	SOBRAL	HOSPITA L REGIONA	-	Enviada	Eventos Adversos Relaciona	Lesão por pressão		
15/12/2021	CE	SOBRAL	HOSPITA L REGIONA	-	Enviada	Eventos Adversos Relaciona	Lesão por pressão		
15/12/2021	CE	SOBRAL	HOSPITA L REGIONA	-	Enviada	Eventos Adversos Relaciona	Lesão por pressão		
15/12/2021	CE	SOBRAL	HOSPITA L REGIONA	-	Enviada	Eventos Adversos Relaciona	Lesão por pressão		
15/12/2021	CE	SOBRAL	HOSPITA L REGIONA	-	Enviada	Eventos Adversos Relaciona	Falhas durante a assistênci		

15/12/2021	CE	SOBRAL	HOSPITA L REGIONA	-	Enviada	Eventos Adversos Relaciona	Falhas durante a assistênci		
15/12/2021	CE	SOBRAL	HOSPITA L REGIONA	-	Enviada	Eventos Adversos Relaciona	Falhas durante a assistênci		
15/12/2021	CE	SOBRAL	HOSPITA L REGIONA	-	Enviada	Eventos Adversos Relaciona	Queda do paciente		
15/12/2021	CE	SOBRAL	HOSPITA L REGIONA	-	Enviada	Eventos Adversos Relaciona	Falhas envolvend o cateter		
15/12/2021	CE	SOBRAL	HOSPITA L REGIONA	-	Enviada	Eventos Adversos Relaciona	Falhas durante a assistênci		
15/12/2021	CE	SOBRAL	HOSPITA L REGIONA	-	Enviada	Eventos Adversos Relaciona	Falhas na administra ção de		Enteral (oral, cateter
15/12/2021	CE	SOBRAL	HOSPITA L REGIONA	-	Enviada	Eventos Adversos Relaciona	Falhas durante a assistênci		
15/12/2021	CE	SOBRAL	HOSPITA L REGIONA	-	Enviada	Eventos Adversos Relaciona	Falhas durante a assistênci		
15/12/2021	CE	SOBRAL	HOSPITA L REGIONA	-	Enviada	Eventos Adversos Relaciona	Lesão por pressão		
15/12/2021	CE	SOBRAL	HOSPITA L REGIONA	-	Enviada	Eventos Adversos Relaciona	Falhas durante a assistênci		
16/12/2021	CE	SOBRAL	HOSPITA L REGIONA	-	Enviada	Eventos Adversos Relaciona	Falhas durante a assistênci		
16/12/2021	CE	SOBRAL	HOSPITA L REGIONA	-	Enviada	Eventos Adversos Relaciona	Falhas nas atividades		
16/12/2021	CE	SOBRAL	HOSPITA L REGIONA	-	Enviada	Eventos Adversos Relaciona	Falhas durante a assistênci		
16/12/2021	CE	SOBRAL	HOSPITA L REGIONA	-	Enviada	Eventos Adversos Relaciona	Falhas durante a assistênci		
16/12/2021	CE	SOBRAL	HOSPITA L REGIONA	-	Enviada	Eventos Adversos Relaciona	Queda do paciente		
16/12/2021	CE	SOBRAL	HOSPITA L REGIONA	-	Enviada	Eventos Adversos Relaciona	Falhas durante a assistênci		
16/12/2021	CE	SOBRAL	HOSPITA L REGIONA	-	Enviada	Eventos Adversos Relaciona	Falhas envolvend o cateter		
16/12/2021	CE	SOBRAL	HOSPITA L REGIONA	-	Enviada	Eventos Adversos Relaciona	Falhas durante a assistênci		
16/12/2021	CE	SOBRAL	HOSPITA L REGIONA	-	Enviada	Eventos Adversos Relaciona	Falhas durante a assistênci		

16/12/2021	CE	SOBRAL	HOSPITA L REGIONA	-	Enviada	Eventos Adversos Relaciona	Falhas durante a assistênci		
16/12/2021	CE	SOBRAL	HOSPITA L REGIONA	-	Enviada	Eventos Adversos Relaciona	Queda do paciente		
16/12/2021	CE	SOBRAL	HOSPITA L REGIONA	-	Enviada	Eventos Adversos Relaciona	Falhas durante a assistênci		
16/12/2021	CE	SOBRAL	HOSPITA L REGIONA	-	Enviada	Eventos Adversos Relaciona	Falhas durante a assistênci		
16/12/2021	CE	SOBRAL	HOSPITA L REGIONA	-	Enviada	Eventos Adversos Relaciona	Falhas durante a assistênci		
16/12/2021	CE	SOBRAL	HOSPITA L REGIONA	-	Enviada	Eventos Adversos Relaciona	Falhas durante a assistênci		
16/12/2021	CE	SOBRAL	HOSPITA L REGIONA	-	Enviada	Eventos Adversos Relaciona	Falhas durante a assistênci		
16/12/2021	CE	SOBRAL	HOSPITA L REGIONA	-	Enviada	Eventos Adversos Relaciona	Falhas durante a assistênci		
16/12/2021	CE	SOBRAL	HOSPITA L REGIONA	-	Enviada	Eventos Adversos Relaciona	Lesão por pressão		
17/12/2021	CE	SOBRAL	HOSPITA L REGIONA	-	Enviada	Eventos Adversos Relaciona	Falhas durante a assistênci		
17/12/2021	CE	SOBRAL	HOSPITA L REGIONA	-	Enviada	Eventos Adversos Relaciona	Falhas na administra ção de		Enteral (oral, cateter
17/12/2021	CE	SOBRAL	HOSPITA L REGIONA	-	Enviada	Eventos Adversos Relaciona	Falhas na administra ção de		Enteral (oral, cateter
17/12/2021	CE	SOBRAL	HOSPITA L REGIONA	-	Enviada	Eventos Adversos Relaciona	Lesão por pressão		
17/12/2021	CE	SOBRAL	HOSPITA L REGIONA	-	Enviada	Eventos Adversos Relaciona	Lesão por pressão		
17/12/2021	CE	SOBRAL	HOSPITA L REGIONA	-	Enviada	Eventos Adversos Relaciona	Queda do paciente		
17/12/2021	CE	SOBRAL	HOSPITA L REGIONA	-	Enviada	Eventos Adversos Relaciona	Lesão por pressão		
18/12/2021	CE	SOBRAL	HOSPITA L REGIONA	-	Enviada	Eventos Adversos Relaciona	Falhas na administra ção de		Enteral (oral, cateter
18/12/2021	CE	SOBRAL	HOSPITA L REGIONA	-	Enviada	Eventos Adversos Relaciona	Falhas na administra ção de		Enteral (oral, cateter
18/12/2021	CE	SOBRAL	HOSPITA L REGIONA	-	Enviada	Eventos Adversos Relaciona	Lesão por pressão		

18/12/2021	CE	SOBRAL	HOSPITA L REGIONA	-	Enviada	Eventos Adversos Relaciona	Falhas envolvend o cateter		
18/12/2021	CE	SOBRAL	HOSPITA L REGIONA	-	Enviada	Eventos Adversos Relaciona	Lesão por pressão		
18/12/2021	CE	SOBRAL	HOSPITA L REGIONA	-	Enviada	Eventos Adversos Relaciona	Queda do paciente		
18/12/2021	CE	SOBRAL	HOSPITA L REGIONA	-	Enviada	Eventos Adversos Relaciona	Falhas durante a assistênci		
18/12/2021	CE	SOBRAL	HOSPITA L REGIONA	-	Enviada	Eventos Adversos Relaciona	Falhas durante a assistênci		
18/12/2021	CE	SOBRAL	HOSPITA L REGIONA	-	Enviada	Eventos Adversos Relaciona	Falhas durante a assistênci		
18/12/2021	CE	SOBRAL	HOSPITA L REGIONA	-	Enviada	Eventos Adversos Relaciona	Queda do paciente		
18/12/2021	CE	SOBRAL	HOSPITA L REGIONA	-	Enviada	Eventos Adversos Relaciona	Falhas na administra ção de		Enteral (oral, cateter
18/12/2021	CE	SOBRAL	HOSPITA L REGIONA	-	Enviada	Eventos Adversos Relaciona	Falhas durante a assistênci		
18/12/2021	CE	SOBRAL	HOSPITA L REGIONA	-	Enviada	Eventos Adversos Relaciona	Queda do paciente		
18/12/2021	CE	SOBRAL	HOSPITA L REGIONA	-	Enviada	Eventos Adversos Relaciona	Falhas na administra ção de		Enteral (oral, cateter
18/12/2021	CE	SOBRAL	HOSPITA L REGIONA	-	Enviada	Eventos Adversos Relaciona	Falhas durante a assistênci		
18/12/2021	CE	SOBRAL	HOSPITA L REGIONA	-	Enviada	Eventos Adversos Relaciona	Falhas na administra ção de		Enteral (oral, cateter
18/12/2021	CE	SOBRAL	HOSPITA L REGIONA	-	Enviada	Eventos Adversos Relaciona	Falhas envolvend o cateter		
18/12/2021	CE	SOBRAL	HOSPITA L REGIONA	-	Enviada	Eventos Adversos Relaciona	Falhas na administra ção de		Enteral (oral, cateter
18/12/2021	CE	SOBRAL	HOSPITA L REGIONA	-	Enviada	Eventos Adversos Relaciona	Falhas durante a assistênci		
18/12/2021	CE	SOBRAL	HOSPITA L REGIONA	-	Enviada	Eventos Adversos Relaciona	Lesão por pressão		
18/12/2021	CE	SOBRAL	HOSPITA L REGIONA	-	Enviada	Eventos Adversos Relaciona	Falhas na administra ção de		Enteral (oral, cateter
18/12/2021	CE	SOBRAL	HOSPITA L REGIONA	-	Enviada	Eventos Adversos Relaciona	Falhas durante a assistênci		

18/12/2021	CE	SOBRAL	HOSPITA L REGIONA	-	Enviada	Eventos Adversos Relaciona	Lesão por pressão		
18/12/2021	CE	SOBRAL	HOSPITA L REGIONA	-	Enviada	Eventos Adversos Relaciona	Falhas durante a assistênci		
18/12/2021	CE	SOBRAL	HOSPITA L REGIONA	-	Enviada	Eventos Adversos Relaciona	Falhas durante a assistênci		
18/12/2021	CE	SOBRAL	HOSPITA L REGIONA	-	Enviada	Eventos Adversos Relaciona	Queda do paciente		
18/12/2021	CE	SOBRAL	HOSPITA L REGIONA	-	Enviada	Eventos Adversos Relaciona	Falhas na administra ção de		Enteral (oral, cateter
18/12/2021	CE	SOBRAL	HOSPITA L REGIONA	-	Enviada	Eventos Adversos Relaciona	Falhas envolvend o cateter		
18/12/2021	CE	SOBRAL	HOSPITA L REGIONA	-	Enviada	Eventos Adversos Relaciona	Falhas durante a assistênci		
18/12/2021	CE	SOBRAL	HOSPITA L REGIONA	-	Enviada	Eventos Adversos Relaciona	Falhas durante a assistênci		
18/12/2021	CE	SOBRAL	HOSPITA L REGIONA	-	Enviada	Eventos Adversos Relaciona	Lesão por pressão		
18/12/2021	CE	SOBRAL	HOSPITA L REGIONA	-	Enviada	Eventos Adversos Relaciona	Queda do paciente		
18/12/2021	CE	SOBRAL	HOSPITA L REGIONA	-	Enviada	Eventos Adversos Relaciona	Falhas durante a assistênci		
18/12/2021	CE	SOBRAL	HOSPITA L REGIONA	-	Enviada	Eventos Adversos Relaciona	Lesão por pressão		
18/12/2021	CE	SOBRAL	HOSPITA L REGIONA	-	Enviada	Eventos Adversos Relaciona	Lesão por pressão		
18/12/2021	CE	SOBRAL	HOSPITA L REGIONA	-	Enviada	Eventos Adversos Relaciona	Falhas durante a assistênci		
18/12/2021	CE	SOBRAL	HOSPITA L REGIONA	-	Enviada	Eventos Adversos Relaciona	Falhas durante a assistênci		
18/12/2021	CE	SOBRAL	HOSPITA L REGIONA	-	Enviada	Eventos Adversos Relaciona	Lesão por pressão		
18/12/2021	CE	SOBRAL	HOSPITA L REGIONA	-	Enviada	Eventos Adversos Relaciona	Falhas na administra ção de		Enteral (oral, cateter
18/12/2021	CE	SOBRAL	HOSPITA L REGIONA	-	Enviada	Eventos Adversos Relaciona	Lesão por pressão		
18/12/2021	CE	SOBRAL	HOSPITA L REGIONA	-	Enviada	Eventos Adversos Relaciona	Falhas envolvend o cateter		

18/12/2021	CE	SOBRAL	HOSPITA L REGIONA	-	Enviada	Eventos Adversos Relaciona	Lesão por pressão		
18/12/2021	CE	SOBRAL	HOSPITA L REGIONA	-	Enviada	Eventos Adversos Relaciona	Lesão por pressão		
20/12/2021	CE	SOBRAL	HOSPITA L REGIONA	-	Enviada	Eventos Adversos Relaciona	Lesão por pressão		
20/12/2021	CE	SOBRAL	HOSPITA L REGIONA	-	Enviada	Eventos Adversos Relaciona	Lesão por pressão		
20/12/2021	CE	SOBRAL	HOSPITA L REGIONA	-	Enviada	Eventos Adversos Relaciona	Lesão por pressão		
20/12/2021	CE	SOBRAL	HOSPITA L REGIONA	-	Enviada	Eventos Adversos Relaciona	Lesão por pressão		
20/12/2021	CE	SOBRAL	HOSPITA L REGIONA	-	Enviada	Eventos Adversos Relaciona	Lesão por pressão		
20/12/2021	CE	SOBRAL	HOSPITA L REGIONA	-	Enviada	Eventos Adversos Relaciona	Lesão por pressão		
20/12/2021	CE	SOBRAL	HOSPITA L REGIONA	-	Enviada	Eventos Adversos Relaciona	Lesão por pressão		
20/12/2021	CE	SOBRAL	HOSPITA L REGIONA	-	Enviada	Eventos Adversos Relaciona	Lesão por pressão		
22/12/2021	CE	SOBRAL	HOSPITA L REGIONA	-	Enviada	Eventos Adversos Relaciona	Lesão por pressão		
22/12/2021	CE	SOBRAL	HOSPITA L REGIONA	-	Enviada	Eventos Adversos Relaciona	Lesão por pressão		
22/12/2021	CE	SOBRAL	HOSPITA L REGIONA	-	Enviada	Eventos Adversos Relaciona	Lesão por pressão		
22/12/2021	CE	SOBRAL	HOSPITA L REGIONA	-	Enviada	Eventos Adversos Relaciona	Lesão por pressão		
22/12/2021	CE	SOBRAL	HOSPITA L REGIONA	-	Enviada	Eventos Adversos Relaciona	Lesão por pressão		
22/12/2021	CE	SOBRAL	HOSPITA L REGIONA	-	Enviada	Eventos Adversos Relaciona	Lesão por pressão		
22/12/2021	CE	SOBRAL	HOSPITA L REGIONA	-	Enviada	Eventos Adversos Relaciona	Lesão por pressão		
22/12/2021	CE	SOBRAL	HOSPITA L REGIONA	-	Enviada	Eventos Adversos Relaciona	Lesão por pressão		
22/12/2021	CE	SOBRAL	HOSPITA L REGIONA	-	Enviada	Eventos Adversos Relaciona	Lesão por pressão		

22/12/2021	CE	SOBRAL	HOSPITA L REGIONA	-	Enviada	Eventos Adversos Relaciona	Lesão por pressão		
22/12/2021	CE	SOBRAL	HOSPITA L REGIONA	-	Enviada	Eventos Adversos Relaciona	Lesão por pressão		
22/12/2021	CE	SOBRAL	HOSPITA L REGIONA	-	Enviada	Eventos Adversos Relaciona	Falhas envolvend o cateter		
22/12/2021	CE	SOBRAL	HOSPITA L REGIONA	-	Enviada	Eventos Adversos Relaciona	Falhas envolvend o cateter		
22/12/2021	CE	SOBRAL	HOSPITA L REGIONA	-	Enviada	Eventos Adversos Relaciona	Falhas envolvend o cateter		
22/12/2021	CE	SOBRAL	HOSPITA L REGIONA	-	Enviada	Eventos Adversos Relaciona	Falhas envolvend o cateter		
22/12/2021	CE	SOBRAL	HOSPITA L REGIONA	-	Enviada	Eventos Adversos Relaciona	Falhas durante a assistênci		
22/12/2021	CE	SOBRAL	HOSPITA L REGIONA	-	Enviada	Eventos Adversos Relaciona	Falhas durante a assistênci		
22/12/2021	CE	SOBRAL	HOSPITA L REGIONA	-	Enviada	Eventos Adversos Relaciona	Falhas envolvend o cateter		
22/12/2021	CE	SOBRAL	HOSPITA L REGIONA	-	Enviada	Eventos Adversos Relaciona	Falhas envolvend o cateter		
22/12/2021	CE	SOBRAL	HOSPITA L REGIONA	-	Enviada	Eventos Adversos Relaciona	Falhas envolvend o cateter		
22/12/2021	CE	SOBRAL	HOSPITA L REGIONA	-	Enviada	Eventos Adversos Relaciona	Falhas envolvend o cateter		
22/12/2021	CE	SOBRAL	HOSPITA L REGIONA	-	Enviada	Eventos Adversos Relaciona	Falhas envolvend o cateter		
22/12/2021	CE	SOBRAL	HOSPITA L REGIONA	-	Enviada	Eventos Adversos Relaciona	Falhas envolvend o cateter		
22/12/2021	CE	SOBRAL	HOSPITA L REGIONA	-	Enviada	Eventos Adversos Relaciona	Queda do paciente		
22/12/2021	CE	SOBRAL	HOSPITA L REGIONA	-	Enviada	Eventos Adversos Relaciona	Queda do paciente		
22/12/2021	CE	SOBRAL	HOSPITA L REGIONA	-	Enviada	Eventos Adversos Relaciona	Queda do paciente		
22/12/2021	CE	SOBRAL	HOSPITA L REGIONA	-	Enviada	Eventos Adversos Relaciona	Queda do paciente		
22/12/2021	CE	SOBRAL	HOSPITA L REGIONA	-	Enviada	Eventos Adversos Relaciona	Queda do paciente		

22/12/2021	CE	SOBRAL	HOSPITA L REGIONA	-	Enviada	Eventos Adversos Relaciona	Queda do paciente		
22/12/2021	CE	SOBRAL	HOSPITA L REGIONA	-	Enviada	Eventos Adversos Relaciona	Falhas na administra ção de		Enteral (oral, cateter
22/12/2021	CE	SOBRAL	HOSPITA L REGIONA	-	Enviada	Eventos Adversos Relaciona	Falhas na administra ção de		Enteral (oral, cateter
22/12/2021	CE	SOBRAL	HOSPITA L REGIONA	-	Enviada	Eventos Adversos Relaciona	Falhas na administra ção de		Enteral (oral, cateter
22/12/2021	CE	SOBRAL	HOSPITA L REGIONA	-	Enviada	Eventos Adversos Relaciona	Falhas na administra ção de		Enteral (oral, cateter
22/12/2021	CE	SOBRAL	HOSPITA L REGIONA	-	Enviada	Eventos Adversos Relaciona	Falhas na administra ção de		Enteral (oral, cateter
22/12/2021	CE	SOBRAL	HOSPITA L REGIONA	-	Enviada	Eventos Adversos Relaciona	Falhas na administra ção de		Enteral (oral, cateter
22/12/2021	CE	SOBRAL	HOSPITA L REGIONA	-	Enviada	Eventos Adversos Relaciona	Falhas durante a assistênci		
22/12/2021	CE	SOBRAL	HOSPITA L REGIONA	-	Enviada	Eventos Adversos Relaciona	Falhas na administra ção de		Enteral (oral, cateter
22/12/2021	CE	SOBRAL	HOSPITA L REGIONA	-	Enviada	Eventos Adversos Relaciona	Falhas durante a assistênci		
22/12/2021	CE	SOBRAL	HOSPITA L REGIONA	-	Enviada	Eventos Adversos Relaciona	Falhas na administra ção de		Enteral (oral, cateter
22/12/2021	CE	SOBRAL	HOSPITA L REGIONA	-	Enviada	Eventos Adversos Relaciona	Falhas envolvend o cateter		
28/12/2021	CE	SOBRAL	HOSPITA L REGIONA	-	Enviada	Eventos Adversos Relaciona	Lesão por pressão		
28/12/2021	CE	SOBRAL	HOSPITA L REGIONA	-	Enviada	Eventos Adversos Relaciona	Lesão por pressão		
28/12/2021	CE	SOBRAL	HOSPITA L REGIONA	-	Enviada	Eventos Adversos Relaciona	Falhas durante a assistênci		
18/01/2022	CE	SOBRAL	HOSPITA L REGIONA	-	Enviada	Eventos Adversos Relaciona	Falhas envolvend o cateter		
18/01/2022	CE	SOBRAL	HOSPITA L REGIONA	-	Enviada	Eventos Adversos Relaciona	Falhas durante a assistênci		
18/01/2022	CE	SOBRAL	HOSPITA L REGIONA	-	Enviada	Eventos Adversos Relaciona	Falhas durante a assistênci		
18/01/2022	CE	SOBRAL	HOSPITA L REGIONA	-	Enviada	Eventos Adversos Relaciona	Falhas durante a assistênci		

18/01/2022	CE	SOBRAL	HOSPITA L REGIONA	-	Enviada	Eventos Adversos Relaciona	Falhas envolvend o cateter		
18/01/2022	CE	SOBRAL	HOSPITA L REGIONA	-	Enviada	Eventos Adversos Relaciona	Falhas durante a assistênci		
18/01/2022	CE	SOBRAL	HOSPITA L REGIONA	-	Enviada	Eventos Adversos Relaciona	Lesão por pressão		
18/01/2022	CE	SOBRAL	HOSPITA L REGIONA	-	Enviada	Eventos Adversos Relaciona	Lesão por pressão		
19/01/2022	CE	SOBRAL	HOSPITA L REGIONA	-	Enviada	Eventos Adversos Relaciona	Falhas envolvend o cateter		
19/01/2022	CE	SOBRAL	HOSPITA L REGIONA	-	Enviada	Eventos Adversos Relaciona	Falhas durante a assistênci		
19/01/2022	CE	SOBRAL	HOSPITA L REGIONA	-	Enviada	Eventos Adversos Relaciona	Falhas envolvend o cateter		
19/01/2022	CE	SOBRAL	HOSPITA L REGIONA	-	Enviada	Eventos Adversos Relaciona	Falhas durante a assistênci		
19/01/2022	CE	SOBRAL	HOSPITA L REGIONA	-	Enviada	Eventos Adversos Relaciona	Falhas durante a assistênci		
19/01/2022	CE	SOBRAL	HOSPITA L REGIONA	-	Enviada	Eventos Adversos Relaciona	Lesão por pressão		
19/01/2022	CE	SOBRAL	HOSPITA L REGIONA	-	Enviada	Eventos Adversos Relaciona	Lesão por pressão		
19/01/2022	CE	SOBRAL	HOSPITA L REGIONA	-	Enviada	Eventos Adversos Relaciona	Lesão por pressão		
19/01/2022	CE	SOBRAL	HOSPITA L REGIONA	-	Enviada	Eventos Adversos Relaciona	Lesão por pressão		
19/01/2022	CE	SOBRAL	HOSPITA L REGIONA	-	Enviada	Eventos Adversos Relaciona	Lesão por pressão		
19/01/2022	CE	SOBRAL	HOSPITA L REGIONA	-	Enviada	Eventos Adversos Relaciona	Lesão por pressão		
19/01/2022	CE	SOBRAL	HOSPITA L REGIONA	-	Enviada	Eventos Adversos Relaciona	Falhas durante a assistênci		
19/01/2022	CE	SOBRAL	HOSPITA L REGIONA	-	Enviada	Eventos Adversos Relaciona	Falhas durante a assistênci		
19/01/2022	CE	SOBRAL	HOSPITA L REGIONA	-	Enviada	Eventos Adversos Relaciona	Falhas ocorridas em		
19/01/2022	CE	SOBRAL	HOSPITA L REGIONA	-	Enviada	Eventos Adversos Relaciona	Falhas ocorridas em		

19/01/2022	CE	SOBRAL	HOSPITA L REGIONA	-	Enviada	Eventos Adversos Relaciona	Falhas na administra ção de		Enteral (oral, cateter
19/01/2022	CE	SOBRAL	HOSPITA L REGIONA	-	Enviada	Eventos Adversos Relaciona	Falhas envolvend o cateter		
19/01/2022	CE	SOBRAL	HOSPITA L REGIONA	-	Enviada	Eventos Adversos Relaciona	Falhas envolvend o cateter		
19/01/2022	CE	SOBRAL	HOSPITA L REGIONA	-	Enviada	Eventos Adversos Relaciona	Falhas envolvend o cateter		
19/01/2022	CE	SOBRAL	HOSPITA L REGIONA	-	Enviada	Eventos Adversos Relaciona	Falhas envolvend o cateter		
19/01/2022	CE	SOBRAL	HOSPITA L REGIONA	-	Enviada	Eventos Adversos Relaciona	Queda do paciente		
04/02/2022	CE	SOBRAL	HOSPITA L REGIONA	-	Enviada	Eventos Adversos Relaciona	Lesão por pressão		
07/02/2022	CE	SOBRAL	HOSPITA L REGIONA	-	Enviada	Eventos Adversos Relaciona	Lesão por pressão		
07/02/2022	CE	SOBRAL	HOSPITA L REGIONA	-	Enviada	Eventos Adversos Relaciona	Queda do paciente		
07/02/2022	CE	SOBRAL	HOSPITA L REGIONA	-	Enviada	Eventos Adversos Relaciona	Lesão por pressão		
07/02/2022	CE	SOBRAL	HOSPITA L REGIONA	-	Enviada	Eventos Adversos Relaciona	Lesão por pressão		
07/02/2022	CE	SOBRAL	HOSPITA L REGIONA	-	Enviada	Eventos Adversos Relaciona	Lesão por pressão		
07/02/2022	CE	SOBRAL	HOSPITA L REGIONA	-	Enviada	Eventos Adversos Relaciona	Falhas envolvend o cateter		
07/02/2022	CE	SOBRAL	HOSPITA L REGIONA	-	Enviada	Eventos Adversos Relaciona	Lesão por pressão		
07/02/2022	CE	SOBRAL	HOSPITA L REGIONA	-	Enviada	Eventos Adversos Relaciona	Queda do paciente		
07/02/2022	CE	SOBRAL	HOSPITA L REGIONA	-	Enviada	Eventos Adversos Relaciona	Lesão por pressão		
07/02/2022	CE	SOBRAL	HOSPITA L REGIONA	-	Enviada	Eventos Adversos Relaciona	Falhas durante a assistênci		
07/02/2022	CE	SOBRAL	HOSPITA L REGIONA	-	Enviada	Eventos Adversos Relaciona	Falhas durante a assistênci		
07/02/2022	CE	SOBRAL	HOSPITA L REGIONA	-	Enviada	Eventos Adversos Relaciona	Lesão por pressão		

07/02/2022	CE	SOBRAL	HOSPITA L REGIONA	-	Enviada	Eventos Adversos Relaciona	Lesão por pressão		
07/02/2022	CE	SOBRAL	HOSPITA L REGIONA	-	Enviada	Eventos Adversos Relaciona	Lesão por pressão		
07/02/2022	CE	SOBRAL	HOSPITA L REGIONA	-	Enviada	Eventos Adversos Relaciona	Lesão por pressão		
07/02/2022	CE	SOBRAL	HOSPITA L REGIONA	-	Enviada	Eventos Adversos Relaciona	Lesão por pressão		
07/02/2022	CE	SOBRAL	HOSPITA L REGIONA	-	Enviada	Eventos Adversos Relaciona	Lesão por pressão		
07/02/2022	CE	SOBRAL	HOSPITA L REGIONA	-	Enviada	Eventos Adversos Relaciona	Lesão por pressão		
07/02/2022	CE	SOBRAL	HOSPITA L REGIONA	-	Enviada	Eventos Adversos Relaciona	Lesão por pressão		
07/02/2022	CE	SOBRAL	HOSPITA L REGIONA	-	Enviada	Eventos Adversos Relaciona	Lesão por pressão		
08/02/2022	CE	SOBRAL	HOSPITA L REGIONA	-	Enviada	Eventos Adversos Relaciona	Falhas durante a assistênci		
08/02/2022	CE	SOBRAL	HOSPITA L REGIONA	-	Enviada	Eventos Adversos Relaciona	Falhas durante a assistênci		
08/02/2022	CE	SOBRAL	HOSPITA L REGIONA	-	Enviada	Eventos Adversos Relaciona	Falhas durante a assistênci		
08/02/2022	CE	SOBRAL	HOSPITA L REGIONA	-	Enviada	Eventos Adversos Relaciona	Falhas durante a assistênci		
08/02/2022	CE	SOBRAL	HOSPITA L REGIONA	-	Enviada	Eventos Adversos Relaciona	Falhas durante a assistênci		
08/02/2022	CE	SOBRAL	HOSPITA L REGIONA	-	Enviada	Eventos Adversos Relaciona	Falhas durante a assistênci		
09/02/2022	CE	SOBRAL	HOSPITA L REGIONA	-	Enviada	Eventos Adversos Relaciona	Queda do paciente		
10/02/2022	CE	SOBRAL	HOSPITA L REGIONA	-	Enviada	Eventos Adversos Relaciona	Queda do paciente		
10/02/2022	CE	SOBRAL	HOSPITA L REGIONA	-	Enviada	Eventos Adversos Relaciona	Queda do paciente		
10/02/2022	CE	SOBRAL	HOSPITA L REGIONA	-	Enviada	Eventos Adversos Relaciona	Falhas durante a assistênci		
10/02/2022	CE	SOBRAL	HOSPITA L REGIONA	-	Enviada	Eventos Adversos Relaciona	Lesão por pressão		

10/02/2022	CE	SOBRAL	HOSPITA L REGIONA	-	Enviada	Eventos Adversos Relaciona	Queda do paciente		
10/02/2022	CE	SOBRAL	HOSPITA L REGIONA	-	Enviada	Eventos Adversos Relaciona	Falhas durante a assistênci		
10/02/2022	CE	SOBRAL	HOSPITA L REGIONA	-	Enviada	Eventos Adversos Relaciona	Falhas durante a assistênci		
10/02/2022	CE	SOBRAL	HOSPITA L REGIONA	-	Enviada	Eventos Adversos Relaciona	Queda do paciente		
10/02/2022	CE	SOBRAL	HOSPITA L REGIONA	-	Enviada	Eventos Adversos Relaciona	Falhas durante a assistênci		
10/02/2022	CE	SOBRAL	HOSPITA L REGIONA	-	Enviada	Eventos Adversos Relaciona	Queda do paciente		
10/02/2022	CE	SOBRAL	HOSPITA L REGIONA	-	Enviada	Eventos Adversos Relaciona	Falhas durante a assistênci		
10/02/2022	CE	SOBRAL	HOSPITA L REGIONA	-	Enviada	Eventos Adversos Relaciona	Lesão por pressão		
10/02/2022	CE	SOBRAL	HOSPITA L REGIONA	-	Enviada	Eventos Adversos Relaciona	Queda do paciente		
10/02/2022	CE	SOBRAL	HOSPITA L REGIONA	-	Enviada	Eventos Adversos Relaciona	Falhas na administra ção de		Enteral (oral, cateter
10/02/2022	CE	SOBRAL	HOSPITA L REGIONA	-	Enviada	Eventos Adversos Relaciona	Lesão por pressão		
11/02/2022	CE	SOBRAL	HOSPITA L REGIONA	-	Enviada	Eventos Adversos Relaciona	Lesão por pressão		
14/02/2022	CE	SOBRAL	HOSPITA L REGIONA	-	Enviada	Eventos Adversos Relaciona	Falhas na administra ção de		Enteral (oral, cateter
14/02/2022	CE	SOBRAL	HOSPITA L REGIONA	-	Enviada	Eventos Adversos Relaciona	Falhas durante a assistênci		
14/02/2022	CE	SOBRAL	HOSPITA L REGIONA	-	Enviada	Eventos Adversos Relaciona	Lesão por pressão		
15/02/2022	CE	SOBRAL	HOSPITA L REGIONA	-	Enviada	Eventos Adversos Relaciona	Lesão por pressão		
15/02/2022	CE	SOBRAL	HOSPITA L REGIONA	-	Enviada	Eventos Adversos Relaciona	Lesão por pressão		
16/02/2022	CE	SOBRAL	HOSPITA L REGIONA	-	Enviada	Eventos Adversos Relaciona	Lesão por pressão		
16/02/2022	CE	SOBRAL	HOSPITA L REGIONA	-	Enviada	Eventos Adversos Relaciona	Falhas durante a assistênci		

16/02/2022	CE	SOBRAL	HOSPITA L REGIONA	-	Enviada	Eventos Adversos Relaciona	Falhas durante a assistênci		
17/02/2022	CE	SOBRAL	HOSPITA L REGIONA	-	Enviada	Eventos Adversos Relaciona	Lesão por pressão		
18/02/2022	CE	SOBRAL	HOSPITA L REGIONA	-	Enviada	Eventos Adversos Relaciona	Lesão por pressão		
18/02/2022	CE	SOBRAL	HOSPITA L REGIONA	-	Enviada	Eventos Adversos Relaciona	Falhas durante a assistênci		
18/02/2022	CE	SOBRAL	HOSPITA L REGIONA	-	Enviada	Eventos Adversos Relaciona	Falhas durante a assistênci		
18/02/2022	CE	SOBRAL	HOSPITA L REGIONA	-	Enviada	Eventos Adversos Relaciona	Falhas na administra ção de		Enteral (oral, cateter
21/02/2022	CE	SOBRAL	HOSPITA L REGIONA	-	Enviada	Eventos Adversos Relaciona	Lesão por pressão		
21/02/2022	CE	SOBRAL	HOSPITA L REGIONA	-	Enviada	Eventos Adversos Relaciona	Lesão por pressão		
22/02/2022	CE	SOBRAL	HOSPITA L REGIONA	-	Enviada	Eventos Adversos Relaciona	Queda do paciente		
22/02/2022	CE	SOBRAL	HOSPITA L REGIONA	-	Enviada	Eventos Adversos Relaciona	Falhas durante a assistênci		
22/02/2022	CE	SOBRAL	HOSPITA L REGIONA	-	Enviada	Eventos Adversos Relaciona	Queda do paciente		
23/02/2022	CE	SOBRAL	HOSPITA L REGIONA	-	Enviada	Eventos Adversos Relaciona	Lesão por pressão		
23/02/2022	CE	SOBRAL	HOSPITA L REGIONA	-	Enviada	Eventos Adversos Relaciona	Lesão por pressão		
24/02/2022	CE	SOBRAL	HOSPITA L REGIONA	-	Enviada	Eventos Adversos Relaciona	Lesão por pressão		
01/03/2022	CE	SOBRAL	HOSPITA L REGIONA	-	Enviada	Eventos Adversos Relaciona	Lesão por pressão		
01/03/2022	CE	SOBRAL	HOSPITA L REGIONA	-	Enviada	Eventos Adversos Relaciona	Lesão por pressão		
01/03/2022	CE	SOBRAL	HOSPITA L REGIONA	-	Enviada	Eventos Adversos Relaciona	Lesão por pressão		
01/03/2022	CE	SOBRAL	HOSPITA L REGIONA	-	Enviada	Eventos Adversos Relaciona	Lesão por pressão		
01/03/2022	CE	SOBRAL	HOSPITA L REGIONA	-	Enviada	Eventos Adversos Relaciona	Falhas durante a assistênci		

01/03/2022	CE	SOBRAL	HOSPITA L REGIONA	-	Enviada	Eventos Adversos Relaciona	Falhas envolvend o cateter		
01/03/2022	CE	SOBRAL	HOSPITA L REGIONA	-	Enviada	Eventos Adversos Relaciona	Falhas durante a assistênci		
03/03/2022	CE	SOBRAL	HOSPITA L REGIONA	-	Enviada	Eventos Adversos Relaciona	Lesão por pressão		
03/03/2022	CE	SOBRAL	HOSPITA L REGIONA	-	Enviada	Eventos Adversos Relaciona	Lesão por pressão		
03/03/2022	CE	SOBRAL	HOSPITA L REGIONA	-	Enviada	Eventos Adversos Relaciona	Lesão por pressão		
03/03/2022	CE	SOBRAL	HOSPITA L REGIONA	-	Enviada	Eventos Adversos Relaciona	Lesão por pressão		
04/03/2022	CE	SOBRAL	HOSPITA L REGIONA	-	Enviada	Eventos Adversos Relaciona	Falhas durante a assistênci		
04/03/2022	CE	SOBRAL	HOSPITA L REGIONA	-	Enviada	Eventos Adversos Relaciona	Falhas na administra ção de		Enteral (oral, cateter
04/03/2022	CE	SOBRAL	HOSPITA L REGIONA	-	Enviada	Eventos Adversos Relaciona	Falhas na administra ção de		Enteral (oral, cateter
04/03/2022	CE	SOBRAL	HOSPITA L REGIONA	-	Enviada	Eventos Adversos Relaciona	Falhas na administra ção de		Enteral (oral, cateter
08/03/2022	CE	SOBRAL	HOSPITA L REGIONA	-	Enviada	Eventos Adversos Relaciona	Queda do paciente		
08/03/2022	CE	SOBRAL	HOSPITA L REGIONA	-	Enviada	Eventos Adversos Relaciona	Queda do paciente		
08/03/2022	CE	SOBRAL	HOSPITA L REGIONA	-	Enviada	Eventos Adversos Relaciona	Lesão por pressão		
08/03/2022	CE	SOBRAL	HOSPITA L REGIONA	-	Enviada	Eventos Adversos Relaciona	Queda do paciente		
08/03/2022	CE	SOBRAL	HOSPITA L REGIONA	-	Enviada	Eventos Adversos Relaciona	Lesão por pressão		
09/03/2022	CE	SOBRAL	HOSPITA L REGIONA	-	Enviada	Eventos Adversos Relaciona	Lesão por pressão		
09/03/2022	CE	SOBRAL	HOSPITA L REGIONA	-	Enviada	Eventos Adversos Relaciona	Lesão por pressão		
09/03/2022	CE	SOBRAL	HOSPITA L REGIONA	-	Enviada	Eventos Adversos Relaciona	Lesão por pressão		
09/03/2022	CE	SOBRAL	HOSPITA L REGIONA	-	Enviada	Eventos Adversos Relaciona	Lesão por pressão		

09/03/2022	CE	SOBRAL	HOSPITA L REGIONA	-	Enviada	Eventos Adversos Relaciona	Falha na identificaç ão do		
09/03/2022	CE	SOBRAL	HOSPITA L REGIONA	-	Enviada	Eventos Adversos Relaciona	Falhas na administra ção de		Enteral (oral, cateter
09/03/2022	CE	SOBRAL	HOSPITA L REGIONA	-	Enviada	Eventos Adversos Relaciona	Falhas na administra ção de		Enteral (oral, cateter
09/03/2022	CE	SOBRAL	HOSPITA L REGIONA	-	Enviada	Eventos Adversos Relaciona	Falhas na administra ção de		Enteral (oral, cateter
11/03/2022	CE	SOBRAL	HOSPITA L REGIONA	-	Enviada	Eventos Adversos Relaciona	Falhas durante a assistênci		
11/03/2022	CE	SOBRAL	HOSPITA L REGIONA	-	Enviada	Eventos Adversos Relaciona	Lesão por pressão		
11/03/2022	CE	SOBRAL	HOSPITA L REGIONA	-	Enviada	Eventos Adversos Relaciona	Lesão por pressão		
11/03/2022	CE	SOBRAL	HOSPITA L REGIONA	-	Enviada	Eventos Adversos Relaciona	Lesão por pressão		
11/03/2022	CE	SOBRAL	HOSPITA L REGIONA	-	Enviada	Eventos Adversos Relaciona	Falhas na administra ção de		Enteral (oral, cateter
11/03/2022	CE	SOBRAL	HOSPITA L REGIONA	-	Enviada	Eventos Adversos Relaciona	Falhas na administra ção de		Enteral (oral, cateter
11/03/2022	CE	SOBRAL	HOSPITA L REGIONA	-	Enviada	Eventos Adversos Relaciona	Falhas na administra ção de		Enteral (oral, cateter
14/03/2022	CE	SOBRAL	HOSPITA L REGIONA	-	Enviada	Eventos Adversos Relaciona	Falhas durante a assistênci		
14/03/2022	CE	SOBRAL	HOSPITA L REGIONA	-	Enviada	Eventos Adversos Relaciona	Lesão por pressão		
14/03/2022	CE	SOBRAL	HOSPITA L REGIONA	-	Enviada	Eventos Adversos Relaciona	Lesão por pressão		
14/03/2022	CE	SOBRAL	HOSPITA L REGIONA	-	Enviada	Eventos Adversos Relaciona	Falhas durante a assistênci		
18/03/2022	CE	SOBRAL	HOSPITA L REGIONA	-	Enviada	Eventos Adversos Relaciona	Lesão por pressão		
18/03/2022	CE	SOBRAL	HOSPITA L REGIONA	-	Enviada	Eventos Adversos Relaciona	Falhas durante a assistênci		
18/03/2022	CE	SOBRAL	HOSPITA L REGIONA	-	Enviada	Eventos Adversos Relaciona	Falhas durante a assistênci		
18/03/2022	CE	SOBRAL	HOSPITA L REGIONA	-	Enviada	Eventos Adversos Relaciona	Queda do paciente		

23/03/2022	CE	SOBRAL	HOSPITA L REGIONA	-	Enviada	Eventos Adversos Relaciona	Falhas durante a assistênci		
23/03/2022	CE	SOBRAL	HOSPITA L REGIONA	-	Enviada	Eventos Adversos Relaciona	Lesão por pressão		
23/03/2022	CE	SOBRAL	HOSPITA L REGIONA	-	Enviada	Eventos Adversos Relaciona	Lesão por pressão		
23/03/2022	CE	SOBRAL	HOSPITA L REGIONA	-	Enviada	Eventos Adversos Relaciona	Falhas envolvend o cateter		
23/03/2022	CE	SOBRAL	HOSPITA L REGIONA	-	Enviada	Eventos Adversos Relaciona	Falhas durante a assistênci		
23/03/2022	CE	SOBRAL	HOSPITA L REGIONA	-	Enviada	Eventos Adversos Relaciona	Falhas envolvend o cateter		
04/04/2022	CE	SOBRAL	HOSPITA L REGIONA	-	Enviada	Eventos Adversos Relaciona	Falhas durante a assistênci		
04/04/2022	CE	SOBRAL	HOSPITA L REGIONA	-	Enviada	Eventos Adversos Relaciona	Lesão por pressão		
04/04/2022	CE	SOBRAL	HOSPITA L REGIONA	-	Enviada	Eventos Adversos Relaciona	Lesão por pressão		
04/04/2022	CE	SOBRAL	HOSPITA L REGIONA	-	Enviada	Eventos Adversos Relaciona	Lesão por pressão		
04/04/2022	CE	SOBRAL	HOSPITA L REGIONA	-	Enviada	Eventos Adversos Relaciona	Lesão por pressão		
04/04/2022	CE	SOBRAL	HOSPITA L REGIONA	-	Enviada	Eventos Adversos Relaciona	Lesão por pressão		
04/04/2022	CE	SOBRAL	HOSPITA L REGIONA	-	Enviada	Eventos Adversos Relaciona	Lesão por pressão		
04/04/2022	CE	SOBRAL	HOSPITA L REGIONA	-	Enviada	Eventos Adversos Relaciona	Falhas envolvend o cateter		
04/04/2022	CE	SOBRAL	HOSPITA L REGIONA	-	Enviada	Eventos Adversos Relaciona	Falhas durante a assistênci		
04/04/2022	CE	SOBRAL	HOSPITA L REGIONA	-	Enviada	Eventos Adversos Relaciona	Falhas na administra ção de		Enteral (oral, cateter
04/04/2022	CE	SOBRAL	HOSPITA L REGIONA	-	Enviada	Eventos Adversos Relaciona	Falhas durante a assistênci		
05/04/2022	CE	SOBRAL	HOSPITA L REGIONA	-	Enviada	Eventos Adversos Relaciona	Falhas durante a assistênci		
05/04/2022	CE	SOBRAL	HOSPITA L REGIONA	-	Enviada	Eventos Adversos Relaciona	Falhas durante a assistênci		

05/04/2022	CE	SOBRAL	HOSPITA L REGIONA	-	Enviada	Eventos Adversos Relaciona	Falhas durante a assistênci		
05/04/2022	CE	SOBRAL	HOSPITA L REGIONA	-	Enviada	Eventos Adversos Relaciona	Falhas durante a assistênci		
12/04/2022	CE	SOBRAL	HOSPITA L REGIONA	-	Enviada	Eventos Adversos Relaciona	Lesão por pressão		
12/04/2022	CE	SOBRAL	HOSPITA L REGIONA	-	Enviada	Eventos Adversos Relaciona	Lesão por pressão		
12/04/2022	CE	SOBRAL	HOSPITA L REGIONA	-	Enviada	Eventos Adversos Relaciona	Lesão por pressão		
12/04/2022	CE	SOBRAL	HOSPITA L REGIONA	-	Enviada	Eventos Adversos Relaciona	Lesão por pressão		
12/04/2022	CE	SOBRAL	HOSPITA L REGIONA	-	Enviada	Eventos Adversos Relaciona	Lesão por pressão		
12/04/2022	CE	SOBRAL	HOSPITA L REGIONA	-	Enviada	Eventos Adversos Relaciona	Falhas envolvend o cateter		
12/04/2022	CE	SOBRAL	HOSPITA L REGIONA	-	Enviada	Eventos Adversos Relaciona	Queda do paciente		
12/04/2022	CE	SOBRAL	HOSPITA L REGIONA	-	Enviada	Eventos Adversos Relaciona	Queda do paciente		
12/04/2022	CE	SOBRAL	HOSPITA L REGIONA	-	Enviada	Eventos Adversos Relaciona	Queda do paciente		
12/04/2022	CE	SOBRAL	HOSPITA L REGIONA	-	Enviada	Eventos Adversos Relaciona	Falhas durante a assistênci		
12/04/2022	CE	SOBRAL	HOSPITA L REGIONA	-	Enviada	Eventos Adversos Relaciona	Falhas durante a assistênci		
12/04/2022	CE	SOBRAL	HOSPITA L REGIONA	-	Enviada	Eventos Adversos Relaciona	Falhas durante a assistênci		
12/04/2022	CE	SOBRAL	HOSPITA L REGIONA	-	Enviada	Eventos Adversos Relaciona	Falhas durante a assistênci		
12/04/2022	CE	SOBRAL	HOSPITA L REGIONA	-	Enviada	Eventos Adversos Relaciona	Falhas durante a assistênci		
18/04/2022	CE	SOBRAL	HOSPITA L REGIONA	-	Enviada	Eventos Adversos Relaciona	Lesão por pressão		
18/04/2022	CE	SOBRAL	HOSPITA L REGIONA	-	Enviada	Eventos Adversos Relaciona	Lesão por pressão		
18/04/2022	CE	SOBRAL	HOSPITA L REGIONA	-	Enviada	Eventos Adversos Relaciona	Lesão por pressão		

18/04/2022	CE	SOBRAL	HOSPITA L REGIONA	-	Enviada	Eventos Adversos Relaciona	Lesão por pressão		
18/04/2022	CE	SOBRAL	HOSPITA L REGIONA	-	Enviada	Eventos Adversos Relaciona	Falhas durante a assistênci		
18/04/2022	CE	SOBRAL	HOSPITA L REGIONA	-	Enviada	Eventos Adversos Relaciona	Falhas durante a assistênci		
18/04/2022	CE	SOBRAL	HOSPITA L REGIONA	-	Enviada	Eventos Adversos Relaciona	Falhas durante a assistênci		
18/04/2022	CE	SOBRAL	HOSPITA L REGIONA	-	Enviada	Eventos Adversos Relaciona	Falhas durante a assistênci		
18/04/2022	CE	SOBRAL	HOSPITA L REGIONA	-	Enviada	Eventos Adversos Relaciona	Falhas durante a assistênci		
18/04/2022	CE	SOBRAL	HOSPITA L REGIONA	-	Enviada	Eventos Adversos Relaciona	Falhas durante a assistênci		
18/04/2022	CE	SOBRAL	HOSPITA L REGIONA	-	Enviada	Eventos Adversos Relaciona	Falhas durante a assistênci		
18/04/2022	CE	SOBRAL	HOSPITA L REGIONA	-	Enviada	Eventos Adversos Relaciona	Falhas na administra ção de		Enteral (oral, cateter
18/04/2022	CE	SOBRAL	HOSPITA L REGIONA	-	Enviada	Eventos Adversos Relaciona	Falhas durante a assistênci		
18/04/2022	CE	SOBRAL	HOSPITA L REGIONA	-	Enviada	Eventos Adversos Relaciona	Falhas durante a assistênci		
18/04/2022	CE	SOBRAL	HOSPITA L REGIONA	-	Enviada	Eventos Adversos Relaciona	Falhas durante a assistênci		
18/04/2022	CE	SOBRAL	HOSPITA L REGIONA	-	Enviada	Eventos Adversos Relaciona	Falhas durante a assistênci		
18/04/2022	CE	SOBRAL	HOSPITA L REGIONA	-	Enviada	Eventos Adversos Relaciona	Falhas durante a assistênci		
18/04/2022	CE	SOBRAL	HOSPITA L REGIONA	-	Enviada	Eventos Adversos Relaciona	Falhas na administra ção de		Enteral (oral, cateter
19/04/2022	CE	SOBRAL	HOSPITA L REGIONA	-	Enviada	Eventos Adversos Relaciona	Falhas durante a assistênci		
19/04/2022	CE	SOBRAL	HOSPITA L REGIONA	-	Enviada	Eventos Adversos Relaciona	Falhas durante a assistênci		
19/04/2022	CE	SOBRAL	HOSPITA L REGIONA	-	Enviada	Eventos Adversos Relaciona	Falhas na administra ção de		Enteral (oral, cateter
22/04/2022	CE	SOBRAL	HOSPITA L REGIONA	-	Enviada	Eventos Adversos Relaciona	Lesão por pressão		

22/04/2022	CE	SOBRAL	HOSPITA L REGIONA	-	Enviada	Eventos Adversos Relaciona	Lesão por pressão		
22/04/2022	CE	SOBRAL	HOSPITA L REGIONA	-	Enviada	Eventos Adversos Relaciona	Falhas envolvend o cateter		
27/04/2022	CE	SOBRAL	HOSPITA L REGIONA	-	Enviada	Eventos Adversos Relaciona	Falhas envolvend o cateter		
27/04/2022	CE	SOBRAL	HOSPITA L REGIONA	-	Enviada	Eventos Adversos Relaciona	Lesão por pressão		
27/04/2022	CE	SOBRAL	HOSPITA L REGIONA	-	Enviada	Eventos Adversos Relaciona	Lesão por pressão		
27/04/2022	CE	SOBRAL	HOSPITA L REGIONA	-	Enviada	Eventos Adversos Relaciona	Lesão por pressão		
27/04/2022	CE	SOBRAL	HOSPITA L REGIONA	-	Enviada	Eventos Adversos Relaciona	Lesão por pressão		
27/04/2022	CE	SOBRAL	HOSPITA L REGIONA	-	Enviada	Eventos Adversos Relaciona	Lesão por pressão		
27/04/2022	CE	SOBRAL	HOSPITA L REGIONA	-	Enviada	Eventos Adversos Relaciona	Lesão por pressão		
18/05/2022	CE	SOBRAL	HOSPITA L REGIONA	-	Enviada	Eventos Adversos Relaciona	Falhas durante a assistênci		
18/05/2022	CE	SOBRAL	HOSPITA L REGIONA	-	Enviada	Eventos Adversos Relaciona	Falhas na administra ção de		Enteral (oral, cateter
18/05/2022	CE	SOBRAL	HOSPITA L REGIONA	-	Enviada	Eventos Adversos Relaciona	Queda do paciente		
18/05/2022	CE	SOBRAL	HOSPITA L REGIONA	-	Enviada	Eventos Adversos Relaciona	Falhas durante a assistênci		
18/05/2022	CE	SOBRAL	HOSPITA L REGIONA	-	Enviada	Eventos Adversos Relaciona	Queda do paciente		
18/05/2022	CE	SOBRAL	HOSPITA L REGIONA	-	Enviada	Eventos Adversos Relaciona	Falhas durante a assistênci		
18/05/2022	CE	SOBRAL	HOSPITA L REGIONA	-	Enviada	Eventos Adversos Relaciona	Falhas durante a assistênci		
18/05/2022	CE	SOBRAL	HOSPITA L REGIONA	-	Enviada	Eventos Adversos Relaciona	Falhas na administra ção de		Enteral (oral, cateter
18/05/2022	CE	SOBRAL	HOSPITA L REGIONA	-	Enviada	Eventos Adversos Relaciona	Falhas na administra ção de		Enteral (oral, cateter
18/05/2022	CE	SOBRAL	HOSPITA L REGIONA	-	Enviada	Eventos Adversos Relaciona	Falhas durante a assistênci		

18/05/2022	CE	SOBRAL	HOSPITA L REGIONA	-	Enviada	Eventos Adversos Relaciona	Falhas na administra ção de		Enteral (oral, cateter
18/05/2022	CE	SOBRAL	HOSPITA L REGIONA	-	Enviada	Eventos Adversos Relaciona	Falhas durante a assistênci		
18/05/2022	CE	SOBRAL	HOSPITA L REGIONA	-	Enviada	Eventos Adversos Relaciona	Falhas na administra ção de		Enteral (oral, cateter
18/05/2022	CE	SOBRAL	HOSPITA L REGIONA	-	Enviada	Eventos Adversos Relaciona	Lesão por pressão		
18/05/2022	CE	SOBRAL	HOSPITA L REGIONA	-	Enviada	Eventos Adversos Relaciona	Lesão por pressão		
18/05/2022	CE	SOBRAL	HOSPITA L REGIONA	-	Enviada	Eventos Adversos Relaciona	Lesão por pressão		
18/05/2022	CE	SOBRAL	HOSPITA L REGIONA	-	Enviada	Eventos Adversos Relaciona	Lesão por pressão		
18/05/2022	CE	SOBRAL	HOSPITA L REGIONA	-	Enviada	Eventos Adversos Relaciona	Lesão por pressão		
18/05/2022	CE	SOBRAL	HOSPITA L REGIONA	-	Enviada	Eventos Adversos Relaciona	Lesão por pressão		
18/05/2022	CE	SOBRAL	HOSPITA L REGIONA	-	Enviada	Eventos Adversos Relaciona	Lesão por pressão		
18/05/2022	CE	SOBRAL	HOSPITA L REGIONA	-	Enviada	Eventos Adversos Relaciona	Lesão por pressão		
18/05/2022	CE	SOBRAL	HOSPITA L REGIONA	-	Enviada	Eventos Adversos Relaciona	Lesão por pressão		
18/05/2022	CE	SOBRAL	HOSPITA L REGIONA	-	Enviada	Eventos Adversos Relaciona	Lesão por pressão		
18/05/2022	CE	SOBRAL	HOSPITA L REGIONA	-	Enviada	Eventos Adversos Relaciona	Lesão por pressão		
18/05/2022	CE	SOBRAL	HOSPITA L REGIONA	-	Enviada	Eventos Adversos Relaciona	Lesão por pressão		
18/05/2022	CE	SOBRAL	HOSPITA L REGIONA	-	Enviada	Eventos Adversos Relaciona	Lesão por pressão		
18/05/2022	CE	SOBRAL	HOSPITA L REGIONA	-	Enviada	Eventos Adversos Relaciona	Lesão por pressão		
18/05/2022	CE	SOBRAL	HOSPITA L REGIONA	-	Enviada	Eventos Adversos Relaciona	Falhas envolvend o cateter		
24/05/2022	CE	SOBRAL	HOSPITA L REGIONA	-	Enviada	Eventos Adversos Relaciona	Falhas na administra ção de		Enteral (oral, cateter

24/05/2022	CE	SOBRAL	HOSPITA L REGIONA	-	Enviada	Eventos Adversos Relaciona	Falhas na administra ção de		Enteral (oral, cateter
24/05/2022	CE	SOBRAL	HOSPITA L REGIONA	-	Enviada	Eventos Adversos Relaciona	Falhas na administra ção de		Enteral (oral, cateter
24/05/2022	CE	SOBRAL	HOSPITA L REGIONA	-	Enviada	Eventos Adversos Relaciona	Falhas na administra ção de		Enteral (oral, cateter
24/05/2022	CE	SOBRAL	HOSPITA L REGIONA	-	Enviada	Eventos Adversos Relaciona	Falhas na administra ção de		Enteral (oral, cateter
24/05/2022	CE	SOBRAL	HOSPITA L REGIONA	-	Enviada	Eventos Adversos Relaciona	Falhas na administra ção de		Enteral (oral, cateter
24/05/2022	CE	SOBRAL	HOSPITA L REGIONA	-	Enviada	Eventos Adversos Relaciona	Falhas durante a assistênci		
24/05/2022	CE	SOBRAL	HOSPITA L REGIONA	-	Enviada	Eventos Adversos Relaciona	Falhas durante a assistênci		
24/05/2022	CE	SOBRAL	HOSPITA L REGIONA	-	Enviada	Eventos Adversos Relaciona	Falhas durante a assistênci		
24/05/2022	CE	SOBRAL	HOSPITA L REGIONA	-	Enviada	Eventos Adversos Relaciona	Falhas durante a assistênci		
24/05/2022	CE	SOBRAL	HOSPITA L REGIONA	-	Enviada	Eventos Adversos Relaciona	Falhas durante a assistênci		
24/05/2022	CE	SOBRAL	HOSPITA L REGIONA	-	Enviada	Eventos Adversos Relaciona	Falhas durante a assistênci		
24/05/2022	CE	SOBRAL	HOSPITA L REGIONA	-	Enviada	Eventos Adversos Relaciona	Falhas durante a assistênci		
24/05/2022	CE	SOBRAL	HOSPITA L REGIONA	-	Enviada	Eventos Adversos Relaciona	Falhas durante a assistênci		
24/05/2022	CE	SOBRAL	HOSPITA L REGIONA	-	Enviada	Eventos Adversos Relaciona	Falhas durante a assistênci		
24/05/2022	CE	SOBRAL	HOSPITA L REGIONA	-	Enviada	Eventos Adversos Relaciona	Falhas durante a assistênci		
24/05/2022	CE	SOBRAL	HOSPITA L REGIONA	-	Enviada	Eventos Adversos Relaciona	Falhas durante a assistênci		
24/05/2022	CE	SOBRAL	HOSPITA L REGIONA	-	Enviada	Eventos Adversos Relaciona	Falhas durante a assistênci		
24/05/2022	CE	SOBRAL	HOSPITA L REGIONA	-	Enviada	Eventos Adversos Relaciona	Falhas durante a assistênci		
24/05/2022	CE	SOBRAL	HOSPITA L REGIONA	-	Enviada	Eventos Adversos Relaciona	Queda do paciente		

24/05/2022	CE	SOBRAL	HOSPITA L REGIONA	-	Enviada	Eventos Adversos Relaciona	Falhas durante a assistênci		
24/05/2022	CE	SOBRAL	HOSPITA L REGIONA	-	Enviada	Eventos Adversos Relaciona	Falhas durante a assistênci		
24/05/2022	CE	SOBRAL	HOSPITA L REGIONA	-	Enviada	Eventos Adversos Relaciona	Falhas durante a assistênci		
24/05/2022	CE	SOBRAL	HOSPITA L REGIONA	-	Enviada	Eventos Adversos Relaciona	Falhas durante a assistênci		
24/05/2022	CE	SOBRAL	HOSPITA L REGIONA	-	Enviada	Eventos Adversos Relaciona	Falhas durante a assistênci		
24/05/2022	CE	SOBRAL	HOSPITA L REGIONA	-	Enviada	Eventos Adversos Relaciona	Falhas na administra ção de		Enteral (oral, cateter
24/05/2022	CE	SOBRAL	HOSPITA L REGIONA	-	Enviada	Eventos Adversos Relaciona	Falhas durante a assistênci		
24/05/2022	CE	SOBRAL	HOSPITA L REGIONA	-	Enviada	Eventos Adversos Relaciona	Falhas envolvend o cateter		
24/05/2022	CE	SOBRAL	HOSPITA L REGIONA	-	Enviada	Eventos Adversos Relaciona	Falhas envolvend o cateter		
24/05/2022	CE	SOBRAL	HOSPITA L REGIONA	-	Enviada	Eventos Adversos Relaciona	Falhas durante a assistênci		
24/05/2022	CE	SOBRAL	HOSPITA L REGIONA	-	Enviada	Eventos Adversos Relaciona	Falhas envolvend o cateter		
24/05/2022	CE	SOBRAL	HOSPITA L REGIONA	-	Enviada	Eventos Adversos Relaciona	Queda do paciente		
24/05/2022	CE	SOBRAL	HOSPITA L REGIONA	-	Enviada	Eventos Adversos Relaciona	Falhas envolvend o cateter		
24/05/2022	CE	SOBRAL	HOSPITA L REGIONA	-	Enviada	Eventos Adversos Relaciona	Falhas durante a assistênci		
24/05/2022	CE	SOBRAL	HOSPITA L REGIONA	-	Enviada	Eventos Adversos Relaciona	Falhas envolvend o cateter		
24/05/2022	CE	SOBRAL	HOSPITA L REGIONA	-	Enviada	Eventos Adversos Relaciona	Falhas envolvend o cateter		
24/05/2022	CE	SOBRAL	HOSPITA L REGIONA	-	Enviada	Eventos Adversos Relaciona	Lesão por pressão		
24/05/2022	CE	SOBRAL	HOSPITA L REGIONA	-	Enviada	Eventos Adversos Relaciona	Falhas envolvend o cateter		
25/05/2022	CE	SOBRAL	HOSPITA L REGIONA	-	Enviada	Eventos Adversos Relaciona	Lesão por pressão		

25/05/2022	CE	SOBRAL	HOSPITA L REGIONA	-	Enviada	Eventos Adversos Relaciona	Lesão por pressão		
25/05/2022	CE	SOBRAL	HOSPITA L REGIONA	-	Enviada	Eventos Adversos Relaciona	Lesão por pressão		
25/05/2022	CE	SOBRAL	HOSPITA L REGIONA	-	Enviada	Eventos Adversos Relaciona	Lesão por pressão		
25/05/2022	CE	SOBRAL	HOSPITA L REGIONA	-	Enviada	Eventos Adversos Relaciona	Lesão por pressão		
25/05/2022	CE	SOBRAL	HOSPITA L REGIONA	-	Enviada	Eventos Adversos Relaciona	Lesão por pressão		
25/05/2022	CE	SOBRAL	HOSPITA L REGIONA	-	Enviada	Eventos Adversos Relaciona	Lesão por pressão		
25/05/2022	CE	SOBRAL	HOSPITA L REGIONA	-	Enviada	Eventos Adversos Relaciona	Lesão por pressão		
25/05/2022	CE	SOBRAL	HOSPITA L REGIONA	-	Enviada	Eventos Adversos Relaciona	Lesão por pressão		
25/05/2022	CE	SOBRAL	HOSPITA L REGIONA	-	Enviada	Eventos Adversos Relaciona	Lesão por pressão		
25/05/2022	CE	SOBRAL	HOSPITA L REGIONA	-	Enviada	Eventos Adversos Relaciona	Lesão por pressão		
25/05/2022	CE	SOBRAL	HOSPITA L REGIONA	-	Enviada	Eventos Adversos Relaciona	Lesão por pressão		
25/05/2022	CE	SOBRAL	HOSPITA L REGIONA	-	Enviada	Eventos Adversos Relaciona	Lesão por pressão		
25/05/2022	CE	SOBRAL	HOSPITA L REGIONA	-	Enviada	Eventos Adversos Relaciona	Lesão por pressão		
25/05/2022	CE	SOBRAL	HOSPITA L REGIONA	-	Enviada	Eventos Adversos Relaciona	Lesão por pressão		
25/05/2022	CE	SOBRAL	HOSPITA L REGIONA	-	Enviada	Eventos Adversos Relaciona	Lesão por pressão		
25/05/2022	CE	SOBRAL	HOSPITA L REGIONA	-	Enviada	Eventos Adversos Relaciona	Lesão por pressão		
26/05/2022	CE	SOBRAL	HOSPITA L REGIONA	-	Enviada	Eventos Adversos Relaciona	Falhas envolvend o cateter		
26/05/2022	CE	SOBRAL	HOSPITA L REGIONA	-	Enviada	Eventos Adversos Relaciona	Falhas durante a assistênci		
26/05/2022	CE	SOBRAL	HOSPITA L REGIONA	-	Enviada	Eventos Adversos Relaciona	Falhas durante a assistênci		

26/05/2022	CE	SOBRAL	HOSPITA L REGIONA	-	Enviada	Eventos Adversos Relaciona	Falhas envolvend o cateter		
26/05/2022	CE	SOBRAL	HOSPITA L REGIONA	-	Enviada	Eventos Adversos Relaciona	Falhas durante a assistênci		
26/05/2022	CE	SOBRAL	HOSPITA L REGIONA	-	Enviada	Eventos Adversos Relaciona	Falhas durante a assistênci		
26/05/2022	CE	SOBRAL	HOSPITA L REGIONA	-	Enviada	Eventos Adversos Relaciona	Queda do paciente		
26/05/2022	CE	SOBRAL	HOSPITA L REGIONA	-	Enviada	Eventos Adversos Relaciona	Falhas envolvend o cateter		
26/05/2022	CE	SOBRAL	HOSPITA L REGIONA	-	Enviada	Eventos Adversos Relaciona	Falhas durante a assistênci		
26/05/2022	CE	SOBRAL	HOSPITA L REGIONA	-	Enviada	Eventos Adversos Relaciona	Falhas envolvend o cateter		
26/05/2022	CE	SOBRAL	HOSPITA L REGIONA	-	Enviada	Eventos Adversos Relaciona	Falhas durante a assistênci		
26/05/2022	CE	SOBRAL	HOSPITA L REGIONA	-	Enviada	Eventos Adversos Relaciona	Falhas durante a assistênci		
26/05/2022	CE	SOBRAL	HOSPITA L REGIONA	-	Enviada	Eventos Adversos Relaciona	Falhas durante a assistênci		
26/05/2022	CE	SOBRAL	HOSPITA L REGIONA	-	Enviada	Eventos Adversos Relaciona	Falhas durante a assistênci		
26/05/2022	CE	SOBRAL	HOSPITA L REGIONA	-	Enviada	Eventos Adversos Relaciona	Falhas envolvend o cateter		
26/05/2022	CE	SOBRAL	HOSPITA L REGIONA	-	Enviada	Eventos Adversos Relaciona	Falhas durante a assistênci		
26/05/2022	CE	SOBRAL	HOSPITA L REGIONA	-	Enviada	Eventos Adversos Relaciona	Falhas durante a assistênci		
26/05/2022	CE	SOBRAL	HOSPITA L REGIONA	-	Enviada	Eventos Adversos Relaciona	Falhas durante a assistênci		
26/05/2022	CE	SOBRAL	HOSPITA L REGIONA	-	Enviada	Eventos Adversos Relaciona	Falhas durante a assistênci		
26/05/2022	CE	SOBRAL	HOSPITA L REGIONA	-	Enviada	Eventos Adversos Relaciona	Falhas durante a assistênci		
27/05/2022	CE	SOBRAL	HOSPITA L REGIONA	-	Enviada	Eventos Adversos Relaciona	Falhas durante a assistênci		
27/05/2022	CE	SOBRAL	HOSPITA L REGIONA	-	Enviada	Eventos Adversos Relaciona	Falhas durante a assistênci		

27/05/2022	CE	SOBRAL	HOSPITA L REGIONA	-	Enviada	Eventos Adversos Relaciona	Falhas durante a assistênci		
27/05/2022	CE	SOBRAL	HOSPITA L REGIONA	-	Enviada	Eventos Adversos Relaciona	Falhas durante a assistênci		
27/05/2022	CE	SOBRAL	HOSPITA L REGIONA	-	Enviada	Eventos Adversos Relaciona	Falhas durante a assistênci		
27/05/2022	CE	SOBRAL	HOSPITA L REGIONA	-	Enviada	Eventos Adversos Relaciona	Falhas durante a assistênci		
27/05/2022	CE	SOBRAL	HOSPITA L REGIONA	-	Enviada	Eventos Adversos Relaciona	Falhas durante a assistênci		
27/05/2022	CE	SOBRAL	HOSPITA L REGIONA	-	Enviada	Eventos Adversos Relaciona	Lesão por pressão		
27/05/2022	CE	SOBRAL	HOSPITA L REGIONA	-	Enviada	Eventos Adversos Relaciona	Falhas durante a assistênci		
27/05/2022	CE	SOBRAL	HOSPITA L REGIONA	-	Enviada	Eventos Adversos Relaciona	Falhas durante a assistênci		
27/05/2022	CE	SOBRAL	HOSPITA L REGIONA	-	Enviada	Eventos Adversos Relaciona	Falhas durante a assistênci		
27/05/2022	CE	SOBRAL	HOSPITA L REGIONA	-	Enviada	Eventos Adversos Relaciona	Falhas envolvend o cateter		
27/05/2022	CE	SOBRAL	HOSPITA L REGIONA	-	Enviada	Eventos Adversos Relaciona	Falhas envolvend o cateter		
27/05/2022	CE	SOBRAL	HOSPITA L REGIONA	-	Enviada	Eventos Adversos Relaciona	Falhas durante a assistênci		
31/05/2022	CE	SOBRAL	HOSPITA L REGIONA	-	Enviada	Eventos Adversos Relaciona	Falhas durante a assistênci		
31/05/2022	CE	SOBRAL	HOSPITA L REGIONA	-	Enviada	Eventos Adversos Relaciona	Falhas durante a assistênci		
31/05/2022	CE	SOBRAL	HOSPITA L REGIONA	-	Enviada	Eventos Adversos Relaciona	Falhas durante a assistênci		
31/05/2022	CE	SOBRAL	HOSPITA L REGIONA	-	Enviada	Eventos Adversos Relaciona	Falhas durante a assistênci		
31/05/2022	CE	SOBRAL	HOSPITA L REGIONA	-	Enviada	Eventos Adversos Relaciona	Lesão por pressão		
31/05/2022	CE	SOBRAL	HOSPITA L REGIONA	-	Enviada	Eventos Adversos Relaciona	Falhas durante a assistênci		
01/06/2022	CE	SOBRAL	HOSPITA L REGIONA	-	Enviada	Eventos Adversos Relaciona	Lesão por pressão		

01/06/2022	CE	SOBRAL	HOSPITA L REGIONA	-	Enviada	Eventos Adversos Relaciona	Queda do paciente		
01/06/2022	CE	SOBRAL	HOSPITA L REGIONA	-	Enviada	Eventos Adversos Relaciona	Lesão por pressão		
01/06/2022	CE	SOBRAL	HOSPITA L REGIONA	-	Enviada	Eventos Adversos Relaciona	Lesão por pressão		
01/06/2022	CE	SOBRAL	HOSPITA L REGIONA	-	Enviada	Eventos Adversos Relaciona	Lesão por pressão		
01/06/2022	CE	SOBRAL	HOSPITA L REGIONA	-	Enviada	Eventos Adversos Relaciona	Falhas durante a assistênci		
01/06/2022	CE	SOBRAL	HOSPITA L REGIONA	-	Enviada	Eventos Adversos Relaciona	Falhas durante a assistênci		
02/06/2022	CE	SOBRAL	HOSPITA L REGIONA	-	Enviada	Eventos Adversos Relaciona	Lesão por pressão		
02/06/2022	CE	SOBRAL	HOSPITA L REGIONA	-	Enviada	Eventos Adversos Relaciona	Falhas na administra ção de		Enteral (oral, cateter
02/06/2022	CE	SOBRAL	HOSPITA L REGIONA	-	Enviada	Eventos Adversos Relaciona	Falhas durante a assistênci		
02/06/2022	CE	SOBRAL	HOSPITA L REGIONA	-	Enviada	Eventos Adversos Relaciona	Lesão por pressão		
02/06/2022	CE	SOBRAL	HOSPITA L REGIONA	-	Enviada	Eventos Adversos Relaciona	Lesão por pressão		
02/06/2022	CE	SOBRAL	HOSPITA L REGIONA	-	Enviada	Eventos Adversos Relaciona	Lesão por pressão		
02/06/2022	CE	SOBRAL	HOSPITA L REGIONA	-	Enviada	Eventos Adversos Relaciona	Lesão por pressão		
02/06/2022	CE	SOBRAL	HOSPITA L REGIONA	-	Enviada	Eventos Adversos Relaciona	Lesão por pressão		
02/06/2022	CE	SOBRAL	HOSPITA L REGIONA	-	Enviada	Eventos Adversos Relaciona	Lesão por pressão		
02/06/2022	CE	SOBRAL	HOSPITA L REGIONA	-	Enviada	Eventos Adversos Relaciona	Falhas durante a assistênci		
03/06/2022	CE	SOBRAL	HOSPITA L REGIONA	-	Enviada	Eventos Adversos Relaciona	Falhas durante a assistênci		
06/06/2022	CE	SOBRAL	HOSPITA L REGIONA	-	Enviada	Eventos Adversos Relaciona	Falhas durante a assistênci		
06/06/2022	CE	SOBRAL	HOSPITA L REGIONA	-	Enviada	Eventos Adversos Relaciona	Lesão por pressão		

06/06/2022	CE	SOBRAL	HOSPITA L REGIONA	-	Enviada	Eventos Adversos Relaciona	Queimadu ra de paciente		
06/06/2022	CE	SOBRAL	HOSPITA L REGIONA	-	Enviada	Eventos Adversos Relaciona	Lesão por pressão		
06/06/2022	CE	SOBRAL	HOSPITA L REGIONA	-	Enviada	Eventos Adversos Relaciona	Falhas durante a assistênci		
06/06/2022	CE	SOBRAL	HOSPITA L REGIONA	-	Enviada	Eventos Adversos Relaciona	Falhas durante a assistênci		
07/06/2022	CE	SOBRAL	HOSPITA L REGIONA	-	Enviada	Eventos Adversos Relaciona	Lesão por pressão		
07/06/2022	CE	SOBRAL	HOSPITA L REGIONA	-	Enviada	Eventos Adversos Relaciona	Falhas durante a assistênci		
07/06/2022	CE	SOBRAL	HOSPITA L REGIONA	-	Enviada	Eventos Adversos Relaciona	Falhas durante a assistênci		
07/06/2022	CE	SOBRAL	HOSPITA L REGIONA	-	Enviada	Eventos Adversos Relaciona	Lesão por pressão		
07/06/2022	CE	SOBRAL	HOSPITA L REGIONA	-	Enviada	Eventos Adversos Relaciona	Lesão por pressão		
07/06/2022	CE	SOBRAL	HOSPITA L REGIONA	-	Enviada	Eventos Adversos Relaciona	Lesão por pressão		
07/06/2022	CE	SOBRAL	HOSPITA L REGIONA	-	Enviada	Eventos Adversos Relaciona	Lesão por pressão		
07/06/2022	CE	SOBRAL	HOSPITA L REGIONA	-	Enviada	Eventos Adversos Relaciona	Falhas durante a assistênci		
07/06/2022	CE	SOBRAL	HOSPITA L REGIONA	-	Enviada	Eventos Adversos Relaciona	Falhas na administra ção de		Enteral (oral, cateter
07/06/2022	CE	SOBRAL	HOSPITA L REGIONA	-	Enviada	Eventos Adversos Relaciona	Falhas durante a assistênci		
07/06/2022	CE	SOBRAL	HOSPITA L REGIONA	-	Enviada	Eventos Adversos Relaciona	Lesão por pressão		
07/06/2022	CE	SOBRAL	HOSPITA L REGIONA	-	Enviada	Eventos Adversos Relaciona	Lesão por pressão		
07/06/2022	CE	SOBRAL	HOSPITA L REGIONA	-	Enviada	Eventos Adversos Relaciona	Lesão por pressão		
09/06/2022	CE	SOBRAL	HOSPITA L REGIONA	-	Enviada	Eventos Adversos Relaciona	Lesão por pressão		
09/06/2022	CE	SOBRAL	HOSPITA L REGIONA	-	Enviada	Eventos Adversos Relaciona	Falhas na administra ção de		Enteral (oral, cateter

10/06/2022	CE	SOBRAL	HOSPITAL REGIONA	-	Enviada	Eventos Adversos Relaciona	Falhas durante a assistênci		
14/06/2022	CE	SOBRAL	HOSPITAL REGIONA	-	Enviada	Eventos Adversos Relaciona	Lesão por pressão		
14/06/2022	CE	SOBRAL	HOSPITAL REGIONA	-	Enviada	Eventos Adversos Relaciona	Lesão por pressão		
14/06/2022	CE	SOBRAL	HOSPITAL REGIONA	-	Enviada	Eventos Adversos Relaciona	Lesão por pressão		
14/06/2022	CE	SOBRAL	HOSPITAL REGIONA	-	Enviada	Eventos Adversos Relaciona	Lesão por pressão		
14/06/2022	CE	SOBRAL	HOSPITAL REGIONA	-	Enviada	Eventos Adversos Relaciona	Falhas durante a assistênci		
14/06/2022	CE	SOBRAL	HOSPITAL REGIONA	-	Enviada	Eventos Adversos Relaciona	Falhas durante a assistênci		
15/06/2022	CE	SOBRAL	HOSPITAL REGIONA	-	Enviada	Eventos Adversos Relaciona	Falhas durante a assistênci		
15/06/2022	CE	SOBRAL	HOSPITAL REGIONA	-	Enviada	Eventos Adversos Relaciona	Falhas durante a assistênci		
15/06/2022	CE	SOBRAL	HOSPITAL REGIONA	-	Enviada	Eventos Adversos Relaciona	Falhas durante a assistênci		
15/06/2022	CE	SOBRAL	HOSPITAL REGIONA	-	Enviada	Eventos Adversos Relaciona	Falhas durante a assistênci		
15/06/2022	CE	SOBRAL	HOSPITAL REGIONA	-	Enviada	Eventos Adversos Relaciona	Queda do paciente		
15/06/2022	CE	SOBRAL	HOSPITAL REGIONA	-	Enviada	Eventos Adversos Relaciona	Queda do paciente		
17/06/2022	CE	SOBRAL	HOSPITAL REGIONA	-	Enviada	Eventos Adversos Relaciona	Falhas na administração de		Enteral (oral, cateter
17/06/2022	CE	SOBRAL	HOSPITAL REGIONA	-	Enviada	Eventos Adversos Relaciona	Falhas durante a assistênci		
17/06/2022	CE	SOBRAL	HOSPITAL REGIONA	-	Enviada	Eventos Adversos Relaciona	Lesão por pressão		
22/06/2022	CE	SOBRAL	HOSPITAL REGIONA	-	Enviada	Eventos Adversos Relaciona	Lesão por pressão		
22/06/2022	CE	SOBRAL	HOSPITAL REGIONA	-	Enviada	Eventos Adversos Relaciona	Lesão por pressão		
22/06/2022	CE	SOBRAL	HOSPITAL REGIONA	-	Enviada	Eventos Adversos Relaciona	Lesão por pressão		

23/06/2022	CE	SOBRAL	HOSPITA L REGIONA	-	Enviada	Eventos Adversos Relaciona	Falhas envolvend o cateter		
23/06/2022	CE	SOBRAL	HOSPITA L REGIONA	-	Enviada	Eventos Adversos Relaciona	Falhas durante a assistênci		
23/06/2022	CE	SOBRAL	HOSPITA L REGIONA	-	Enviada	Eventos Adversos Relaciona	Falhas durante a assistênci		
23/06/2022	CE	SOBRAL	HOSPITA L REGIONA	-	Enviada	Eventos Adversos Relaciona	Falhas durante a assistênci		
23/06/2022	CE	SOBRAL	HOSPITA L REGIONA	-	Enviada	Eventos Adversos Relaciona	Falhas durante a assistênci		
23/06/2022	CE	SOBRAL	HOSPITA L REGIONA	-	Enviada	Eventos Adversos Relaciona	Queda do paciente		
23/06/2022	CE	SOBRAL	HOSPITA L REGIONA	-	Enviada	Eventos Adversos Relaciona	Falhas durante a assistênci		
23/06/2022	CE	SOBRAL	HOSPITA L REGIONA	-	Enviada	Eventos Adversos Relaciona	Falhas envolvend o cateter		
23/06/2022	CE	SOBRAL	HOSPITA L REGIONA	-	Enviada	Eventos Adversos Relaciona	Falhas durante a assistênci		
23/06/2022	CE	SOBRAL	HOSPITA L REGIONA	-	Enviada	Eventos Adversos Relaciona	Queda do paciente		
23/06/2022	CE	SOBRAL	HOSPITA L REGIONA	-	Enviada	Eventos Adversos Relaciona	Falhas durante a assistênci		
23/06/2022	CE	SOBRAL	HOSPITA L REGIONA	-	Enviada	Eventos Adversos Relaciona	Queda do paciente		
23/06/2022	CE	SOBRAL	HOSPITA L REGIONA	-	Enviada	Eventos Adversos Relaciona	Queda do paciente		
06/07/2022	CE	SOBRAL	HOSPITA L REGIONA	BACTERI MIA ASSOCIA	Concluída	Eventos Adversos Relaciona	Falhas durante a assistênci		
06/07/2022	CE	SOBRAL	HOSPITA L REGIONA	BACTERI MIA ASSOCIA	Concluída	Eventos Adversos Relaciona	Falhas durante a assistênci		
06/07/2022	CE	SOBRAL	HOSPITA L REGIONA	BACTERI MIA ASSOCIA	Concluída	Eventos Adversos Relaciona	Falhas durante a assistênci		
06/07/2022	CE	SOBRAL	HOSPITA L REGIONA	BACTER EMIA ASSOCIA	Concluída	Eventos Adversos Relaciona	Falhas durante a assistênci		
06/07/2022	CE	SOBRAL	HOSPITA L REGIONA	BACTER MIA ASSOCIA	Concluída	Eventos Adversos Relaciona	Falhas durante a assistênci		
06/07/2022	CE	SOBRAL	HOSPITA L REGIONA	BACTER EMIA ASSOCIA	Concluída	Eventos Adversos Relaciona	Falhas durante a assistênci		

06/07/2022	CE	SOBRAL	HOSPITA L REGIONA	BACTERI MIA ASSOCIA	Concluída	Eventos Adversos Relaciona	Falhas durante a assistênci		
12/07/2022	CE	SOBRAL	HOSPITA L REGIONA	BACTERI MIA ASSOCIA	Concluída	Eventos Adversos Relaciona	Falhas durante a assistênci		
12/07/2022	CE	SOBRAL	HOSPITA L REGIONA	BACTERI MIA ASSOCIA	Concluída	Eventos Adversos Relaciona	Falhas durante a assistênci		
12/07/2022	CE	SOBRAL	HOSPITA L REGIONA	BACTER MIA ASSOCIA	Concluída	Eventos Adversos Relaciona	Falhas durante a assistênci		
28/07/2022	CE	SOBRAL	HOSPITA L REGIONA	BACTERI MIA ASSOCIA	Concluída	Eventos Adversos Relaciona	Falhas durante a assistênci		
28/07/2022	CE	SOBRAL	HOSPITA L REGIONA	BACTERI MIA ASSOCIA	Concluída	Eventos Adversos Relaciona	Falhas durante a assistênci		
29/07/2022	CE	SOBRAL	HOSPITA L REGIONA	-	Enviada	Eventos Adversos Relaciona	Queda do paciente		
29/07/2022	CE	SOBRAL	HOSPITA L REGIONA	-	Enviada	Eventos Adversos Relaciona	Queda do paciente		
29/07/2022	CE	SOBRAL	HOSPITA L REGIONA	-	Enviada	Eventos Adversos Relaciona	Lesão por pressão		
29/07/2022	CE	SOBRAL	HOSPITA L REGIONA	-	Enviada	Eventos Adversos Relaciona	Lesão por pressão		
29/07/2022	CE	SOBRAL	HOSPITA L REGIONA	-	Enviada	Eventos Adversos Relaciona	Lesão por pressão		
29/07/2022	CE	SOBRAL	HOSPITA L REGIONA	-	Enviada	Eventos Adversos Relaciona	Lesão por pressão		
29/07/2022	CE	SOBRAL	HOSPITA L REGIONA	-	Enviada	Eventos Adversos Relaciona	Lesão por pressão		
29/07/2022	CE	SOBRAL	HOSPITA L REGIONA	-	Enviada	Eventos Adversos Relaciona	Lesão por pressão		
29/07/2022	CE	SOBRAL	HOSPITA L REGIONA	-	Enviada	Eventos Adversos Relaciona	Lesão por pressão		
29/07/2022	CE	SOBRAL	HOSPITA L REGIONA	-	Enviada	Eventos Adversos Relaciona	Lesão por pressão		
29/07/2022	CE	SOBRAL	HOSPITA L REGIONA	-	Enviada	Eventos Adversos Relaciona	Lesão por pressão		
29/07/2022	CE	SOBRAL	HOSPITA L REGIONA	-	Enviada	Eventos Adversos Relaciona	Lesão por pressão		
29/07/2022	CE	SOBRAL	HOSPITA L REGIONA	-	Enviada	Eventos Adversos Relaciona	Lesão por pressão		

29/07/2022	CE	SOBRAL	HOSPITA L REGIONA	-	Enviada	Eventos Adversos Relaciona	Lesão por pressão		
01/08/2022	CE	SOBRAL	HOSPITA L REGIONA	-	Enviada	Eventos Adversos Relaciona	Lesão por pressão		
01/08/2022	CE	SOBRAL	HOSPITA L REGIONA	-	Enviada	Eventos Adversos Relaciona	Lesão por pressão		
01/08/2022	CE	SOBRAL	HOSPITA L REGIONA	-	Enviada	Eventos Adversos Relaciona	Lesão por pressão		
01/08/2022	CE	SOBRAL	HOSPITA L REGIONA	-	Enviada	Eventos Adversos Relaciona	Lesão por pressão		
01/08/2022	CE	SOBRAL	HOSPITA L REGIONA	-	Enviada	Eventos Adversos Relaciona	Lesão por pressão		
01/08/2022	CE	SOBRAL	HOSPITA L REGIONA	-	Enviada	Eventos Adversos Relaciona	Lesão por pressão		
01/08/2022	CE	SOBRAL	HOSPITA L REGIONA	-	Enviada	Eventos Adversos Relaciona	Lesão por pressão		
01/08/2022	CE	SOBRAL	HOSPITA L REGIONA	-	Enviada	Eventos Adversos Relaciona	Lesão por pressão		
01/08/2022	CE	SOBRAL	HOSPITA L REGIONA	-	Enviada	Eventos Adversos Relaciona	Lesão por pressão		
01/08/2022	CE	SOBRAL	HOSPITA L REGIONA	-	Enviada	Eventos Adversos Relaciona	Lesão por pressão		
01/08/2022	CE	SOBRAL	HOSPITA L REGIONA	-	Enviada	Eventos Adversos Relaciona	Falhas envolvend o cateter		
01/08/2022	CE	SOBRAL	HOSPITA L REGIONA	-	Enviada	Eventos Adversos Relaciona	Falhas envolvend o cateter		
01/08/2022	CE	SOBRAL	HOSPITA L REGIONA	-	Enviada	Eventos Adversos Relaciona	Falhas envolvend o cateter		
01/08/2022	CE	SOBRAL	HOSPITA L REGIONA	-	Enviada	Eventos Adversos Relaciona	Falhas envolvend o cateter		
01/08/2022	CE	SOBRAL	HOSPITA L REGIONA	-	Enviada	Eventos Adversos Relaciona	Falhas envolvend o cateter		
02/08/2022	CE	SOBRAL	HOSPITA L REGIONA	-	Enviada	Eventos Adversos Relaciona	Falhas envolvend o cateter		
02/08/2022	CE	SOBRAL	HOSPITA L REGIONA	-	Enviada	Eventos Adversos Relaciona	Falhas na administra ção de		Enteral (oral, cateter
02/08/2022	CE	SOBRAL	HOSPITA L REGIONA	-	Enviada	Eventos Adversos Relaciona	Falhas durante a assistênci		

02/08/2022	CE	SOBRAL	HOSPITA L REGIONA	-	Enviada	Eventos Adversos Relaciona	Falhas durante a assistênci		
02/08/2022	CE	SOBRAL	HOSPITA L REGIONA	-	Enviada	Eventos Adversos Relaciona	Falhas na administra ção de		Enteral (oral, cateter
02/08/2022	CE	SOBRAL	HOSPITA L REGIONA	-	Enviada	Eventos Adversos Relaciona	Falhas envolvend o cateter		
02/08/2022	CE	SOBRAL	HOSPITA L REGIONA	-	Enviada	Eventos Adversos Relaciona	Falhas durante a assistênci		
02/08/2022	CE	SOBRAL	HOSPITA L REGIONA	-	Enviada	Eventos Adversos Relaciona	Falhas envolvend o cateter		
02/08/2022	CE	SOBRAL	HOSPITA L REGIONA	-	Em retificação	Eventos Adversos Relaciona	Falha na identificaç ão do		
02/08/2022	CE	SOBRAL	HOSPITA L REGIONA	-	Enviada	Eventos Adversos Relaciona	Falhas durante a assistênci		
02/08/2022	CE	SOBRAL	HOSPITA L REGIONA	-	Enviada	Eventos Adversos Relaciona	Falhas durante a assistênci		
02/08/2022	CE	SOBRAL	HOSPITA L REGIONA	-	Enviada	Eventos Adversos Relaciona	Falhas durante a assistênci		
02/08/2022	CE	SOBRAL	HOSPITA L REGIONA	-	Enviada	Eventos Adversos Relaciona	Falhas durante a assistênci		
02/08/2022	CE	SOBRAL	HOSPITA L REGIONA	-	Enviada	Eventos Adversos Relaciona	Falhas na administra ção de		Enteral (oral, cateter
02/08/2022	CE	SOBRAL	HOSPITA L REGIONA	-	Enviada	Eventos Adversos Relaciona	Falhas durante a assistênci		
02/08/2022	CE	SOBRAL	HOSPITA L REGIONA	-	Enviada	Eventos Adversos Relaciona	Falhas durante a assistênci		
02/08/2022	CE	SOBRAL	HOSPITA L REGIONA	-	Enviada	Eventos Adversos Relaciona	Falhas na administra ção de		Enteral (oral, cateter
02/08/2022	CE	SOBRAL	HOSPITA L REGIONA	-	Enviada	Eventos Adversos Relaciona	Falhas na administra ção de		Enteral (oral, cateter
02/08/2022	CE	SOBRAL	HOSPITA L REGIONA	-	Enviada	Eventos Adversos Relaciona	Falhas durante a assistênci		
02/08/2022	CE	SOBRAL	HOSPITA L REGIONA	-	Enviada	Eventos Adversos Relaciona	Falhas durante a assistênci		
02/08/2022	CE	SOBRAL	HOSPITA L REGIONA	-	Enviada	Eventos Adversos Relaciona	Falhas durante a assistênci		
02/08/2022	CE	SOBRAL	HOSPITA L REGIONA	-	Enviada	Eventos Adversos Relaciona	Falhas durante a assistênci		

02/08/2022	CE	SOBRAL	HOSPITA L REGIONA	-	Enviada	Eventos Adversos Relaciona	Falhas envolvend o cateter		
02/08/2022	CE	SOBRAL	HOSPITA L REGIONA	-	Enviada	Eventos Adversos Relaciona	Falhas durante a assistênci		
02/08/2022	CE	SOBRAL	HOSPITA L REGIONA	-	Enviada	Eventos Adversos Relaciona	Falhas durante a assistênci		
02/08/2022	CE	SOBRAL	HOSPITA L REGIONA	-	Enviada	Eventos Adversos Relaciona	Falhas envolvend o cateter		
02/08/2022	CE	SOBRAL	HOSPITA L REGIONA	-	Enviada	Eventos Adversos Relaciona	Lesão por pressão		
02/08/2022	CE	SOBRAL	HOSPITA L REGIONA	-	Enviada	Eventos Adversos Relaciona	Lesão por pressão		
02/08/2022	CE	SOBRAL	HOSPITA L REGIONA	-	Enviada	Eventos Adversos Relaciona	Falhas durante a assistênci		
02/08/2022	CE	SOBRAL	HOSPITA L REGIONA	-	Enviada	Eventos Adversos Relaciona	Lesão por pressão		
02/08/2022	CE	SOBRAL	HOSPITA L REGIONA	-	Enviada	Eventos Adversos Relaciona	Lesão por pressão		
02/08/2022	CE	SOBRAL	HOSPITA L REGIONA	-	Enviada	Eventos Adversos Relaciona	Falhas durante a assistênci		
02/08/2022	CE	SOBRAL	HOSPITA L REGIONA	-	Enviada	Eventos Adversos Relaciona	Falhas na administra ção de		Enteral (oral, cateter
02/08/2022	CE	SOBRAL	HOSPITA L REGIONA	-	Enviada	Eventos Adversos Relaciona	Falhas ocorridas em		
03/08/2022	CE	SOBRAL	HOSPITA L REGIONA	-	Enviada	Eventos Adversos Relaciona	Lesão por pressão		
03/08/2022	CE	SOBRAL	HOSPITA L REGIONA	-	Enviada	Eventos Adversos Relaciona	Falhas durante a assistênci		
03/08/2022	CE	SOBRAL	HOSPITA L REGIONA	-	Enviada	Eventos Adversos Relaciona	Falhas durante a assistênci		
03/08/2022	CE	SOBRAL	HOSPITA L REGIONA	-	Enviada	Eventos Adversos Relaciona	Falhas durante a assistênci		
08/08/2022	CE	SOBRAL	HOSPITA L REGIONA	-	Enviada	Eventos Adversos Relaciona	Lesão por pressão		
08/08/2022	CE	SOBRAL	HOSPITA L REGIONA	-	Enviada	Eventos Adversos Relaciona	Lesão por pressão		
08/08/2022	CE	SOBRAL	HOSPITA L REGIONA	-	Enviada	Eventos Adversos Relaciona	Lesão por pressão		

08/08/2022	CE	SOBRAL	HOSPITA L REGIONA	-	Enviada	Eventos Adversos Relaciona	Lesão por pressão		
08/08/2022	CE	SOBRAL	HOSPITA L REGIONA	-	Enviada	Eventos Adversos Relaciona	Lesão por pressão		
08/08/2022	CE	SOBRAL	HOSPITA L REGIONA	-	Enviada	Eventos Adversos Relaciona	Lesão por pressão		
08/08/2022	CE	SOBRAL	HOSPITA L REGIONA	-	Enviada	Eventos Adversos Relaciona	Lesão por pressão		
08/08/2022	CE	SOBRAL	HOSPITA L REGIONA	-	Enviada	Eventos Adversos Relaciona	Lesão por pressão		
08/08/2022	CE	SOBRAL	HOSPITA L REGIONA	-	Enviada	Eventos Adversos Relaciona	Lesão por pressão		
08/08/2022	CE	SOBRAL	HOSPITA L REGIONA	-	Enviada	Eventos Adversos Relaciona	Lesão por pressão		
08/08/2022	CE	SOBRAL	HOSPITA L REGIONA	-	Enviada	Eventos Adversos Relaciona	Lesão por pressão		
08/08/2022	CE	SOBRAL	HOSPITA L REGIONA	-	Enviada	Eventos Adversos Relaciona	Lesão por pressão		
08/08/2022	CE	SOBRAL	HOSPITA L REGIONA	-	Enviada	Eventos Adversos Relaciona	Lesão por pressão		
08/08/2022	CE	SOBRAL	HOSPITA L REGIONA	-	Enviada	Eventos Adversos Relaciona	Lesão por pressão		
08/08/2022	CE	SOBRAL	HOSPITA L REGIONA	-	Enviada	Eventos Adversos Relaciona	Lesão por pressão		
08/08/2022	CE	SOBRAL	HOSPITA L REGIONA	-	Enviada	Eventos Adversos Relaciona	Lesão por pressão		
08/08/2022	CE	SOBRAL	HOSPITA L REGIONA	-	Enviada	Eventos Adversos Relaciona	Lesão por pressão		
08/08/2022	CE	SOBRAL	HOSPITA L REGIONA	-	Enviada	Eventos Adversos Relaciona	Lesão por pressão		
08/08/2022	CE	SOBRAL	HOSPITA L REGIONA	-	Enviada	Eventos Adversos Relaciona	Lesão por pressão		
09/08/2022	CE	SOBRAL	HOSPITA L REGIONA	-	Enviada	Eventos Adversos Relaciona	Lesão por pressão		
09/08/2022	CE	SOBRAL	HOSPITA L REGIONA	-	Enviada	Eventos Adversos Relaciona	Lesão por pressão		
09/08/2022	CE	SOBRAL	HOSPITA L REGIONA	-	Enviada	Eventos Adversos Relaciona	Lesão por pressão		

09/08/2022	CE	SOBRAL	HOSPITA L REGIONA	-	Enviada	Eventos Adversos Relaciona	Lesão por pressão		
09/08/2022	CE	SOBRAL	HOSPITA L REGIONA	-	Enviada	Eventos Adversos Relaciona	Lesão por pressão		
09/08/2022	CE	SOBRAL	HOSPITA L REGIONA	-	Enviada	Eventos Adversos Relaciona	Lesão por pressão		
09/08/2022	CE	SOBRAL	HOSPITA L REGIONA	-	Enviada	Eventos Adversos Relaciona	Lesão por pressão		
09/08/2022	CE	SOBRAL	HOSPITA L REGIONA	-	Enviada	Eventos Adversos Relaciona	Falhas envolvend o cateter		
09/08/2022	CE	SOBRAL	HOSPITA L REGIONA	-	Enviada	Eventos Adversos Relaciona	Falhas envolvend o cateter		
09/08/2022	CE	SOBRAL	HOSPITA L REGIONA	-	Enviada	Eventos Adversos Relaciona	Falhas envolvend o cateter		
09/08/2022	CE	SOBRAL	HOSPITA L REGIONA	-	Enviada	Eventos Adversos Relaciona	Falhas envolvend o cateter		
09/08/2022	CE	SOBRAL	HOSPITA L REGIONA	-	Enviada	Eventos Adversos Relaciona	Falhas envolvend o cateter		
09/08/2022	CE	SOBRAL	HOSPITA L REGIONA	-	Enviada	Eventos Adversos Relaciona	Falhas envolvend o cateter		
09/08/2022	CE	SOBRAL	HOSPITA L REGIONA	-	Enviada	Eventos Adversos Relaciona	Falhas envolvend o cateter		
09/08/2022	CE	SOBRAL	HOSPITA L REGIONA	-	Enviada	Eventos Adversos Relaciona	Falhas envolvend o cateter		
09/08/2022	CE	SOBRAL	HOSPITA L REGIONA	-	Enviada	Eventos Adversos Relaciona	Falhas envolvend o cateter		
09/08/2022	CE	SOBRAL	HOSPITA L REGIONA	-	Enviada	Eventos Adversos Relaciona	Falhas envolvend o cateter		
09/08/2022	CE	SOBRAL	HOSPITA L REGIONA	-	Enviada	Eventos Adversos Relaciona	Falhas envolvend o cateter		
09/08/2022	CE	SOBRAL	HOSPITA L REGIONA	-	Enviada	Eventos Adversos Relaciona	Falhas envolvend o cateter		
09/08/2022	CE	SOBRAL	HOSPITA L REGIONA	-	Enviada	Eventos Adversos Relaciona	Falhas durante a assistênci		
09/08/2022	CE	SOBRAL	HOSPITA L REGIONA	-	Enviada	Eventos Adversos Relaciona	Falhas durante a assistênci		
09/08/2022	CE	SOBRAL	HOSPITA L REGIONA	-	Enviada	Eventos Adversos Relaciona	Falhas durante a assistênci		

09/08/2022	CE	SOBRAL	HOSPITA L REGIONA	-	Enviada	Eventos Adversos Relaciona	Falhas durante a assistênci		
09/08/2022	CE	SOBRAL	HOSPITA L REGIONA	-	Enviada	Eventos Adversos Relaciona	Falhas durante a assistênci		
09/08/2022	CE	SOBRAL	HOSPITA L REGIONA	-	Enviada	Eventos Adversos Relaciona	Falhas durante a assistênci		
09/08/2022	CE	SOBRAL	HOSPITA L REGIONA	-	Enviada	Eventos Adversos Relaciona	Falhas durante a assistênci		
09/08/2022	CE	SOBRAL	HOSPITA L REGIONA	-	Enviada	Eventos Adversos Relaciona	Falhas durante a assistênci		
09/08/2022	CE	SOBRAL	HOSPITA L REGIONA	-	Enviada	Eventos Adversos Relaciona	Falhas durante a assistênci		
09/08/2022	CE	SOBRAL	HOSPITA L REGIONA	-	Enviada	Eventos Adversos Relaciona	Falhas durante a assistênci		
09/08/2022	CE	SOBRAL	HOSPITA L REGIONA	-	Enviada	Eventos Adversos Relaciona	Falhas durante a assistênci		
09/08/2022	CE	SOBRAL	HOSPITA L REGIONA	-	Enviada	Eventos Adversos Relaciona	Falhas durante a assistênci		
09/08/2022	CE	SOBRAL	HOSPITA L REGIONA	-	Enviada	Eventos Adversos Relaciona	Falhas durante a assistênci		
09/08/2022	CE	SOBRAL	HOSPITA L REGIONA	-	Enviada	Eventos Adversos Relaciona	Queda do paciente		
09/08/2022	CE	SOBRAL	HOSPITA L REGIONA	-	Enviada	Eventos Adversos Relaciona	Queda do paciente		
09/08/2022	CE	SOBRAL	HOSPITA L REGIONA	-	Enviada	Eventos Adversos Relaciona	Queda do paciente		
09/08/2022	CE	SOBRAL	HOSPITA L REGIONA	-	Enviada	Eventos Adversos Relaciona	Queda do paciente		
09/08/2022	CE	SOBRAL	HOSPITA L REGIONA	-	Enviada	Eventos Adversos Relaciona	Queda do paciente		
09/08/2022	CE	SOBRAL	HOSPITA L REGIONA	-	Enviada	Eventos Adversos Relaciona	Falhas durante a assistênci		
09/08/2022	CE	SOBRAL	HOSPITA L REGIONA	-	Enviada	Eventos Adversos Relaciona	Falhas durante a assistênci		
09/08/2022	CE	SOBRAL	HOSPITA L REGIONA	-	Enviada	Eventos Adversos Relaciona	Falhas envolvend o cateter		
09/08/2022	CE	SOBRAL	HOSPITA L REGIONA	-	Enviada	Eventos Adversos Relaciona	Falhas na administra ção de		Enteral (oral, cateter

09/08/2022	CE	SOBRAL	HOSPITA L REGIONA	-	Enviada	Eventos Adversos Relaciona	Falhas durante a assistênci		
09/08/2022	CE	SOBRAL	HOSPITA L REGIONA	-	Enviada	Eventos Adversos Relaciona	Falhas durante a assistênci		
09/08/2022	CE	SOBRAL	HOSPITA L REGIONA	-	Enviada	Eventos Adversos Relaciona	Lesão por pressão		
10/08/2022	CE	SOBRAL	HOSPITA L REGIONA	-	Enviada	Eventos Adversos Relaciona	Falhas envolvend o cateter		
10/08/2022	CE	SOBRAL	HOSPITA L REGIONA	-	Enviada	Eventos Adversos Relaciona	Lesão por pressão		
10/08/2022	CE	SOBRAL	HOSPITA L REGIONA	-	Enviada	Eventos Adversos Relaciona	Queda do paciente		
10/08/2022	CE	SOBRAL	HOSPITA L REGIONA	-	Enviada	Eventos Adversos Relaciona	Falhas durante a assistênci		
10/08/2022	CE	SOBRAL	HOSPITA L REGIONA	-	Enviada	Eventos Adversos Relaciona	Falhas envolvend o cateter		
12/08/2022	CE	SOBRAL	HOSPITA L REGIONA	-	Enviada	Eventos Adversos Relaciona	Falhas na administra ção de		Enteral (oral, cateter
12/08/2022	CE	SOBRAL	HOSPITA L REGIONA	-	Enviada	Eventos Adversos Relaciona	Falhas envolvend o cateter		
12/08/2022	CE	SOBRAL	HOSPITA L REGIONA	-	Enviada	Eventos Adversos Relaciona	Queda do paciente		
12/08/2022	CE	SOBRAL	HOSPITA L REGIONA	-	Enviada	Eventos Adversos Relaciona	Lesão por pressão		
12/08/2022	CE	SOBRAL	HOSPITA L REGIONA	-	Enviada	Eventos Adversos Relaciona	Queda do paciente		
12/08/2022	CE	SOBRAL	HOSPITA L REGIONA	-	Enviada	Eventos Adversos Relaciona	Falhas envolvend o cateter		
12/08/2022	CE	SOBRAL	HOSPITA L REGIONA	-	Enviada	Eventos Adversos Relaciona	Falhas na administra ção de		Enteral (oral, cateter
12/08/2022	CE	SOBRAL	HOSPITA L REGIONA	-	Enviada	Eventos Adversos Relaciona	Falhas durante a assistênci		
12/08/2022	CE	SOBRAL	HOSPITA L REGIONA	-	Enviada	Eventos Adversos Relaciona	Falhas durante a assistênci		
15/08/2022	CE	SOBRAL	HOSPITA L REGIONA	-	Enviada	Eventos Adversos Relaciona	Lesão por pressão		
15/08/2022	CE	SOBRAL	HOSPITA L REGIONA	-	Enviada	Eventos Adversos Relaciona	Falhas envolvend o cateter		

15/08/2022	CE	SOBRAL	HOSPITA L REGIONA	-	Enviada	Eventos Adversos Relaciona	Queda do paciente		
15/08/2022	CE	SOBRAL	HOSPITA L REGIONA	-	Enviada	Eventos Adversos Relaciona	Falhas envolvend o cateter		
15/08/2022	CE	SOBRAL	HOSPITA L REGIONA	-	Enviada	Eventos Adversos Relaciona	Falhas envolvend o cateter		
15/08/2022	CE	SOBRAL	HOSPITA L REGIONA	-	Enviada	Eventos Adversos Relaciona	Falhas durante a assistênci		
15/08/2022	CE	SOBRAL	HOSPITA L REGIONA	-	Enviada	Eventos Adversos Relaciona	Falhas durante a assistênci		
15/08/2022	CE	SOBRAL	HOSPITA L REGIONA	-	Enviada	Eventos Adversos Relaciona	Falhas na administra ção de		Enteral (oral, cateter
16/08/2022	CE	SOBRAL	HOSPITA L REGIONA	-	Enviada	Eventos Adversos Relaciona	Falhas envolvend o cateter		
16/08/2022	CE	SOBRAL	HOSPITA L REGIONA	-	Enviada	Eventos Adversos Relaciona	Falhas durante a assistênci		
16/08/2022	CE	SOBRAL	HOSPITA L REGIONA	-	Enviada	Eventos Adversos Relaciona	Lesão por pressão		
17/08/2022	CE	SOBRAL	HOSPITA L REGIONA	-	Enviada	Eventos Adversos Relaciona	Falhas durante a assistênci		
22/08/2022	CE	SOBRAL	HOSPITA L REGIONA	-	Enviada	Eventos Adversos Relaciona	Falhas durante a assistênci		
22/08/2022	CE	SOBRAL	HOSPITA L REGIONA	-	Enviada	Eventos Adversos Relaciona	Falhas durante a assistênci		
22/08/2022	CE	SOBRAL	HOSPITA L REGIONA	-	Enviada	Eventos Adversos Relaciona	Falhas durante a assistênci		
22/08/2022	CE	SOBRAL	HOSPITA L REGIONA	BACTERI MIA ASSOCIA	Concluída	Eventos Adversos Relaciona	Falhas durante a assistênci		
22/08/2022	CE	SOBRAL	HOSPITA L REGIONA	BACTERI MIA ASSOCIA	Concluída	Eventos Adversos Relaciona	Falhas durante a assistênci		
22/08/2022	CE	SOBRAL	HOSPITA L REGIONA	BACTER EMIA ASSOCIA	Em retificação	Eventos Adversos Relaciona	Falhas durante a assistênci		
22/08/2022	CE	SOBRAL	HOSPITA L REGIONA	BACTERI MIA ASSOCIA	Em retificação	Eventos Adversos Relaciona	Falhas durante a assistênci		
23/08/2022	CE	SOBRAL	HOSPITA L REGIONA	-	Enviada	Eventos Adversos Relaciona	Falhas envolvend o cateter		
23/08/2022	CE	SOBRAL	HOSPITA L REGIONA	-	Enviada	Eventos Adversos Relaciona	Lesão por pressão		

23/08/2022	CE	SOBRAL	HOSPITA L REGIONA	-	Enviada	Eventos Adversos Relaciona	Lesão por pressão		
23/08/2022	CE	SOBRAL	HOSPITA L REGIONA	-	Enviada	Eventos Adversos Relaciona	Lesão por pressão		
23/08/2022	CE	SOBRAL	HOSPITA L REGIONA	-	Enviada	Eventos Adversos Relaciona	Lesão por pressão		
23/08/2022	CE	SOBRAL	HOSPITA L REGIONA	-	Enviada	Eventos Adversos Relaciona	Falhas durante a assistênci		
23/08/2022	CE	SOBRAL	HOSPITA L REGIONA	-	Enviada	Eventos Adversos Relaciona	Queda do paciente		
23/08/2022	CE	SOBRAL	HOSPITA L REGIONA	-	Enviada	Eventos Adversos Relaciona	Falhas durante a assistênci		
23/08/2022	CE	SOBRAL	HOSPITA L REGIONA	-	Enviada	Eventos Adversos Relaciona	Falhas durante a assistênci		
24/08/2022	CE	SOBRAL	HOSPITA L REGIONA	-	Enviada	Eventos Adversos Relaciona	Lesão por pressão		
24/08/2022	CE	SOBRAL	HOSPITA L REGIONA	-	Enviada	Eventos Adversos Relaciona	Falhas durante a assistênci		
24/08/2022	CE	SOBRAL	HOSPITA L REGIONA	-	Enviada	Eventos Adversos Relaciona	Falhas na administra ção de		Enteral (oral, cateter
24/08/2022	CE	SOBRAL	HOSPITA L REGIONA	-	Enviada	Eventos Adversos Relaciona	Falhas durante a assistênci		
25/08/2022	CE	SOBRAL	HOSPITA L REGIONA	-	Enviada	Eventos Adversos Relaciona	Falhas durante a assistênci		
25/08/2022	CE	SOBRAL	HOSPITA L REGIONA	-	Enviada	Eventos Adversos Relaciona	Falhas durante a assistênci		
26/08/2022	CE	SOBRAL	HOSPITA L REGIONA	-	Enviada	Eventos Adversos Relaciona	Lesão por pressão		
26/08/2022	CE	SOBRAL	HOSPITA L REGIONA	-	Enviada	Eventos Adversos Relaciona	Lesão por pressão		
29/08/2022	CE	SOBRAL	HOSPITA L REGIONA	-	Enviada	Eventos Adversos Relaciona	Lesão por pressão		
29/08/2022	CE	SOBRAL	HOSPITA L REGIONA	-	Enviada	Eventos Adversos Relaciona	Lesão por pressão		
29/08/2022	CE	SOBRAL	HOSPITA L REGIONA	-	Enviada	Eventos Adversos Relaciona	Lesão por pressão		
29/08/2022	CE	SOBRAL	HOSPITA L REGIONA	-	Enviada	Eventos Adversos Relaciona	Lesão por pressão		

29/08/2022	CE	SOBRAL	HOSPITA L REGIONA	-	Enviada	Eventos Adversos Relaciona	Lesão por pressão		
29/08/2022	CE	SOBRAL	HOSPITA L REGIONA	-	Enviada	Eventos Adversos Relaciona	Lesão por pressão		
29/08/2022	CE	SOBRAL	HOSPITA L REGIONA	-	Enviada	Eventos Adversos Relaciona	Lesão por pressão		
29/08/2022	CE	SOBRAL	HOSPITA L REGIONA	-	Enviada	Eventos Adversos Relaciona	Lesão por pressão		
29/08/2022	CE	SOBRAL	HOSPITA L REGIONA	-	Enviada	Eventos Adversos Relaciona	Queda do paciente		
29/08/2022	CE	SOBRAL	HOSPITA L REGIONA	-	Enviada	Eventos Adversos Relaciona	Queda do paciente		
29/08/2022	CE	SOBRAL	HOSPITA L REGIONA	-	Enviada	Eventos Adversos Relaciona	Falhas envolvend o cateter		
29/08/2022	CE	SOBRAL	HOSPITA L REGIONA	-	Enviada	Eventos Adversos Relaciona	Queda do paciente		
30/08/2022	CE	SOBRAL	HOSPITA L REGIONA	-	Enviada	Eventos Adversos Relaciona	Queda do paciente		
30/08/2022	CE	SOBRAL	HOSPITA L REGIONA	-	Enviada	Eventos Adversos Relaciona	Lesão por pressão		
30/08/2022	CE	SOBRAL	HOSPITA L REGIONA	-	Enviada	Eventos Adversos Relaciona	Lesão por pressão		
05/09/2022	CE	SOBRAL	HOSPITA L REGIONA	-	Enviada	Eventos Adversos Relaciona	Falhas durante a assistênci		
05/09/2022	CE	SOBRAL	HOSPITA L REGIONA	-	Enviada	Eventos Adversos Relaciona	Falhas durante a assistênci		
05/09/2022	CE	SOBRAL	HOSPITA L REGIONA	-	Enviada	Eventos Adversos Relaciona	Falhas durante a assistênci		
05/09/2022	CE	SOBRAL	HOSPITA L REGIONA	-	Enviada	Eventos Adversos Relaciona	Queda do paciente		
05/09/2022	CE	SOBRAL	HOSPITA L REGIONA	-	Enviada	Eventos Adversos Relaciona	Falhas durante a assistênci		
05/09/2022	CE	SOBRAL	HOSPITA L REGIONA	-	Enviada	Eventos Adversos Relaciona	Falhas durante a assistênci		
05/09/2022	CE	SOBRAL	HOSPITA L REGIONA	-	Enviada	Eventos Adversos Relaciona	Lesão por pressão		
05/09/2022	CE	SOBRAL	HOSPITA L REGIONA	-	Enviada	Eventos Adversos Relaciona	Lesão por pressão		

05/09/2022	CE	SOBRAL	HOSPITA L REGIONA	-	Enviada	Eventos Adversos Relaciona	Lesão por pressão		
05/09/2022	CE	SOBRAL	HOSPITA L REGIONA	-	Enviada	Eventos Adversos Relaciona	Lesão por pressão		
05/09/2022	CE	SOBRAL	HOSPITA L REGIONA	-	Enviada	Eventos Adversos Relaciona	Lesão por pressão		
05/09/2022	CE	SOBRAL	HOSPITA L REGIONA	-	Enviada	Eventos Adversos Relaciona	Lesão por pressão		
06/09/2022	CE	SOBRAL	HOSPITA L REGIONA	-	Enviada	Eventos Adversos Relaciona	Lesão por pressão		
06/09/2022	CE	SOBRAL	HOSPITA L REGIONA	-	Enviada	Eventos Adversos Relaciona	Lesão por pressão		
06/09/2022	CE	SOBRAL	HOSPITA L REGIONA	-	Enviada	Eventos Adversos Relaciona	Lesão por pressão		
06/09/2022	CE	SOBRAL	HOSPITA L REGIONA	-	Enviada	Eventos Adversos Relaciona	Lesão por pressão		
06/09/2022	CE	SOBRAL	HOSPITA L REGIONA	-	Enviada	Eventos Adversos Relaciona	Falhas envolvend o cateter		
06/09/2022	CE	SOBRAL	HOSPITA L REGIONA	-	Enviada	Eventos Adversos Relaciona	Falhas durante a assistênci		
06/09/2022	CE	SOBRAL	HOSPITA L REGIONA	-	Enviada	Eventos Adversos Relaciona	Falhas durante a assistênci		
06/09/2022	CE	SOBRAL	HOSPITA L REGIONA	-	Enviada	Eventos Adversos Relaciona	Queda do paciente		
06/09/2022	CE	SOBRAL	HOSPITA L REGIONA	-	Enviada	Eventos Adversos Relaciona	Falhas envolvend o cateter		
06/09/2022	CE	SOBRAL	HOSPITA L REGIONA	-	Enviada	Eventos Adversos Relaciona	Falhas envolvend o cateter		
06/09/2022	CE	SOBRAL	HOSPITA L REGIONA	-	Enviada	Eventos Adversos Relaciona	Falhas durante a assistênci		
06/09/2022	CE	SOBRAL	HOSPITA L REGIONA	-	Enviada	Eventos Adversos Relaciona	Falhas envolvend o cateter		
08/09/2022	CE	SOBRAL	HOSPITA L REGIONA	-	Enviada	Eventos Adversos Relaciona	Falhas envolvend o cateter		
08/09/2022	CE	SOBRAL	HOSPITA L REGIONA	-	Enviada	Eventos Adversos Relaciona	Falhas durante a assistênci		
08/09/2022	CE	SOBRAL	HOSPITA L REGIONA	-	Enviada	Eventos Adversos Relaciona	Falhas envolvend o cateter		

08/09/2022	CE	SOBRAL	HOSPITA L REGIONA	-	Enviada	Eventos Adversos Relaciona	Falhas envolvend o cateter		
08/09/2022	CE	SOBRAL	HOSPITA L REGIONA	-	Enviada	Eventos Adversos Relaciona	Queda do paciente		
08/09/2022	CE	SOBRAL	HOSPITA L REGIONA	-	Enviada	Eventos Adversos Relaciona	Lesão por pressão		
12/09/2022	CE	SOBRAL	HOSPITA L REGIONA	-	Enviada	Eventos Adversos Relaciona	Lesão por pressão		
12/09/2022	CE	SOBRAL	HOSPITA L REGIONA	-	Enviada	Eventos Adversos Relaciona	Falhas na administra ção de		Enteral (oral, cateter
12/09/2022	CE	SOBRAL	HOSPITA L REGIONA	-	Enviada	Eventos Adversos Relaciona	Lesão por pressão		
12/09/2022	CE	SOBRAL	HOSPITA L REGIONA	-	Enviada	Eventos Adversos Relaciona	Falhas durante a assistênci		
12/09/2022	CE	SOBRAL	HOSPITA L REGIONA	-	Enviada	Eventos Adversos Relaciona	Falhas durante a assistênci		
13/09/2022	CE	SOBRAL	HOSPITA L REGIONA	-	Enviada	Eventos Adversos Relaciona	Lesão por pressão		
13/09/2022	CE	SOBRAL	HOSPITA L REGIONA	-	Enviada	Eventos Adversos Relaciona	Falhas durante a assistênci		
13/09/2022	CE	SOBRAL	HOSPITA L REGIONA	-	Enviada	Eventos Adversos Relaciona	Falhas durante a assistênci		
13/09/2022	CE	SOBRAL	HOSPITA L REGIONA	-	Enviada	Eventos Adversos Relaciona	Falhas na administra ção de		Enteral (oral, cateter
13/09/2022	CE	SOBRAL	HOSPITA L REGIONA	-	Enviada	Eventos Adversos Relaciona	Falhas durante a assistênci		
14/09/2022	CE	SOBRAL	HOSPITA L REGIONA	-	Enviada	Eventos Adversos Relaciona	Lesão por pressão		
14/09/2022	CE	SOBRAL	HOSPITA L REGIONA	-	Enviada	Eventos Adversos Relaciona	Falhas durante a assistênci		
14/09/2022	CE	SOBRAL	HOSPITA L REGIONA	-	Enviada	Eventos Adversos Relaciona	Lesão por pressão		
14/09/2022	CE	SOBRAL	HOSPITA L REGIONA	-	Enviada	Eventos Adversos Relaciona	Lesão por pressão		
16/09/2022	CE	SOBRAL	HOSPITA L REGIONA	-	Enviada	Eventos Adversos Relaciona	Falhas durante a assistênci		
16/09/2022	CE	SOBRAL	HOSPITA L REGIONA	-	Enviada	Eventos Adversos Relaciona	Lesão por pressão		

16/09/2022	CE	SOBRAL	HOSPITAL REGIONA	-	Enviada	Eventos Adversos Relaciona	Lesão por pressão		
16/09/2022	CE	SOBRAL	HOSPITAL REGIONA	-	Enviada	Eventos Adversos Relaciona	Lesão por pressão		
16/09/2022	CE	SOBRAL	HOSPITAL REGIONA	-	Enviada	Eventos Adversos Relaciona	Lesão por pressão		
16/09/2022	CE	SOBRAL	HOSPITAL REGIONA	-	Enviada	Eventos Adversos Relaciona	Falhas durante a assistênci		
16/09/2022	CE	SOBRAL	HOSPITAL REGIONA	-	Enviada	Eventos Adversos Relaciona	Lesão por pressão		
16/09/2022	CE	SOBRAL	HOSPITAL REGIONA	-	Enviada	Eventos Adversos Relaciona	Lesão por pressão		
16/09/2022	CE	SOBRAL	HOSPITAL REGIONA	-	Enviada	Eventos Adversos Relaciona	Falhas na administração de		Enteral (oral, cateter
16/09/2022	CE	SOBRAL	HOSPITAL REGIONA	-	Enviada	Eventos Adversos Relaciona	Falhas durante a assistênci		
16/09/2022	CE	SOBRAL	HOSPITAL REGIONA	-	Enviada	Eventos Adversos Relaciona	Lesão por pressão		
16/09/2022	CE	SOBRAL	HOSPITAL REGIONA	-	Enviada	Eventos Adversos Relaciona	Falhas durante a assistênci		
17/09/2022	CE	SOBRAL	HOSPITAL REGIONA	-	Enviada	Eventos Adversos Relaciona	Lesão por pressão		
17/09/2022	CE	SOBRAL	HOSPITAL REGIONA	-	Enviada	Eventos Adversos Relaciona	Lesão por pressão		
17/09/2022	CE	SOBRAL	HOSPITAL REGIONA	-	Enviada	Eventos Adversos Relaciona	Lesão por pressão		
20/09/2022	CE	SOBRAL	HOSPITAL REGIONA	-	Enviada	Eventos Adversos Relaciona	Falhas envolvendo cateter		
20/09/2022	CE	SOBRAL	HOSPITAL REGIONA	-	Enviada	Eventos Adversos Relaciona	Falhas envolvendo cateter		
20/09/2022	CE	SOBRAL	HOSPITAL REGIONA	-	Enviada	Eventos Adversos Relaciona	Lesão por pressão		
21/09/2022	CE	SOBRAL	HOSPITAL REGIONA	-	Enviada	Eventos Adversos Relaciona	Falhas na administração de		Enteral (oral, cateter
21/09/2022	CE	SOBRAL	HOSPITAL REGIONA	-	Enviada	Eventos Adversos Relaciona	Falhas durante a assistênci		
21/09/2022	CE	SOBRAL	HOSPITAL REGIONA	IPCS ASSOCIADA À	Concluída	Eventos Adversos Relaciona	Falhas durante a assistênci		

21/09/2022	CE	SOBRAL	HOSPITA L REGIONA	BACTERI MIA ASSOCIA	Concluída	Eventos Adversos Relaciona	Falhas durante a assistênci		
21/09/2022	CE	SOBRAL	HOSPITA L REGIONA	IPCS ASSOCIA DA À	Concluída	Eventos Adversos Relaciona	Falhas durante a assistênci		
21/09/2022	CE	SOBRAL	HOSPITA L REGIONA	BACTERI MIA ASSOCIA	Concluída	Eventos Adversos Relaciona	Falhas durante a assistênci		
21/09/2022	CE	SOBRAL	HOSPITA L REGIONA	BACTERI MIA ASSOCIA	Concluída	Eventos Adversos Relaciona	Falhas durante a assistênci		
21/09/2022	CE	SOBRAL	HOSPITA L REGIONA	IPCS RELACIO NADA À	Concluída	Eventos Adversos Relaciona	Falhas durante a assistênci		
21/09/2022	CE	SOBRAL	HOSPITA L REGIONA	IPCS ASSOCIA DA À	Concluída	Eventos Adversos Relaciona	Falhas durante a assistênci		
21/09/2022	CE	SOBRAL	HOSPITA L REGIONA	BACTERI MIA ASSOCIA	Concluída	Eventos Adversos Relaciona	Falhas durante a assistênci		
23/09/2022	CE	SOBRAL	HOSPITA L REGIONA	-	Enviada	Eventos Adversos Relaciona	Falhas durante a assistênci		
23/09/2022	CE	SOBRAL	HOSPITA L REGIONA	-	Enviada	Eventos Adversos Relaciona	Lesão por pressão		
26/09/2022	CE	SOBRAL	HOSPITA L REGIONA	-	Enviada	Eventos Adversos Relaciona	Lesão por pressão		
26/09/2022	CE	SOBRAL	HOSPITA L REGIONA	-	Enviada	Eventos Adversos Relaciona	Falhas durante a assistênci		
26/09/2022	CE	SOBRAL	HOSPITA L REGIONA	-	Enviada	Eventos Adversos Relaciona	Falhas durante a assistênci		
26/09/2022	CE	SOBRAL	HOSPITA L REGIONA	-	Enviada	Eventos Adversos Relaciona	Falhas nas atividades		
26/09/2022	CE	SOBRAL	HOSPITA L REGIONA	-	Enviada	Eventos Adversos Relaciona	Lesão por pressão		
27/09/2022	CE	SOBRAL	HOSPITA L REGIONA	-	Enviada	Eventos Adversos Relaciona	Falhas durante a assistênci		
27/09/2022	CE	SOBRAL	HOSPITA L REGIONA	-	Enviada	Eventos Adversos Relaciona	Falhas durante a assistênci		
27/09/2022	CE	SOBRAL	HOSPITA L REGIONA	-	Enviada	Eventos Adversos Relaciona	Falhas durante a assistênci		
27/09/2022	CE	SOBRAL	HOSPITA L REGIONA	-	Enviada	Eventos Adversos Relaciona	Falhas durante a assistênci		
27/09/2022	CE	SOBRAL	HOSPITA L REGIONA	-	Enviada	Eventos Adversos Relaciona	Falhas durante a assistênci		

27/09/2022	CE	SOBRAL	HOSPITA L REGIONA	-	Enviada	Eventos Adversos Relaciona	Falhas durante a assistênci		
27/09/2022	CE	SOBRAL	HOSPITA L REGIONA	-	Enviada	Eventos Adversos Relaciona	Falhas durante a assistênci		
27/09/2022	CE	SOBRAL	HOSPITA L REGIONA	-	Enviada	Eventos Adversos Relaciona	Lesão por pressão		
27/09/2022	CE	SOBRAL	HOSPITA L REGIONA	-	Enviada	Eventos Adversos Relaciona	Falhas envolvend o sondas		
27/09/2022	CE	SOBRAL	HOSPITA L REGIONA	-	Enviada	Eventos Adversos Relaciona	Falhas envolvend o sondas		
27/09/2022	CE	SOBRAL	HOSPITA L REGIONA	-	Enviada	Eventos Adversos Relaciona	Falhas envolvend o sondas		
27/09/2022	CE	SOBRAL	HOSPITA L REGIONA	EVOLUIU COM TVP	Enviada	Eventos Adversos Relaciona	Tromboe mbolismo venoso		
27/09/2022	CE	SOBRAL	HOSPITA L REGIONA	EVOLUIU COM TVP	Enviada	Eventos Adversos Relaciona	Tromboe mbolismo venoso		
27/09/2022	CE	SOBRAL	HOSPITA L REGIONA	-	Enviada	Eventos Adversos Relaciona	Falhas durante a assistênci		
27/09/2022	CE	SOBRAL	HOSPITA L REGIONA	-	Enviada	Eventos Adversos Relaciona	Falhas durante a assistênci		
27/09/2022	CE	SOBRAL	HOSPITA L REGIONA	AVP NO MSE COM	Enviada	Eventos Adversos Relaciona	Falhas envolvend o cateter		
27/09/2022	CE	SOBRAL	HOSPITA L REGIONA	SETOR DE NUTRIÇÃ	Enviada	Eventos Adversos Relaciona	Falhas na administra ção de		Enteral (oral, cateter
27/09/2022	CE	SOBRAL	HOSPITA L REGIONA	TERMO DE CONSEN	Enviada	Eventos Adversos Relaciona	Falhas durante a assistênci		
27/09/2022	CE	SOBRAL	HOSPITA L REGIONA	TERMO DE CONSEN	Enviada	Eventos Adversos Relaciona	Falhas durante a assistênci		
27/09/2022	CE	SOBRAL	HOSPITA L REGIONA	FALTAND O PROTOC	Enviada	Eventos Adversos Relaciona	Falhas durante a assistênci		
27/09/2022	CE	SOBRAL	HOSPITA L REGIONA	FLUXO DE ALTA A	Enviada	Eventos Adversos Relaciona	Falhas nas atividades		
27/09/2022	CE	SOBRAL	HOSPITA L REGIONA	IPCS ASSOCIA DA À	Concluída	Eventos Adversos Relaciona	Falhas durante a assistênci		
27/09/2022	CE	SOBRAL	HOSPITA L REGIONA	BACTER EMIA ASSOCIA	Concluída	Eventos Adversos Relaciona	Falhas durante a assistênci		
27/09/2022	CE	SOBRAL	HOSPITA L REGIONA	IPCS ASSOCIA DA À	Concluída	Eventos Adversos Relaciona	Falhas durante a assistênci		

27/09/2022	CE	SOBRAL	HOSPITA L REGIONA	IPCS RELACIO NADA À	Concluída	Eventos Adversos Relaciona	Falhas durante a assistênci		
28/09/2022	CE	SOBRAL	HOSPITA L REGIONA	-	Enviada	Eventos Adversos Relaciona	Falhas durante a assistênci		
28/09/2022	CE	SOBRAL	HOSPITA L REGIONA	-	Enviada	Eventos Adversos Relaciona	Falhas nas atividades		
28/09/2022	CE	SOBRAL	HOSPITA L REGIONA	-	Enviada	Eventos Adversos Relaciona	Falhas nas atividades		
28/09/2022	CE	SOBRAL	HOSPITA L REGIONA	-	Enviada	Eventos Adversos Relaciona	Lesão por pressão		
28/09/2022	CE	SOBRAL	HOSPITA L REGIONA	-	Enviada	Eventos Adversos Relaciona	Falhas durante a assistênci		
30/09/2022	CE	SOBRAL	HOSPITA L REGIONA	-	Enviada	Eventos Adversos Relaciona	Falhas durante a assistênci		
30/09/2022	CE	SOBRAL	HOSPITA L REGIONA	-	Enviada	Eventos Adversos Relaciona	Falhas durante a assistênci		
30/09/2022	CE	SOBRAL	HOSPITA L REGIONA	-	Enviada	Eventos Adversos Relaciona	Falhas durante a assistênci		
30/09/2022	CE	SOBRAL	HOSPITA L REGIONA	-	Enviada	Eventos Adversos Relaciona	Falhas durante a assistênci		
30/09/2022	CE	SOBRAL	HOSPITA L REGIONA	-	Enviada	Eventos Adversos Relaciona	Falhas durante a assistênci		
30/09/2022	CE	SOBRAL	HOSPITA L REGIONA	-	Enviada	Eventos Adversos Relaciona	Falhas durante a assistênci		
30/09/2022	CE	SOBRAL	HOSPITA L REGIONA	FLEBITE EM MIE. AVP	Enviada	Eventos Adversos Relaciona	Falhas envolvend o cateter		
30/09/2022	CE	SOBRAL	HOSPITA L REGIONA	-	Enviada	Eventos Adversos Relaciona	Lesão por pressão		
30/09/2022	CE	SOBRAL	HOSPITA L REGIONA	Não foi informado que	Enviada	Eventos Adversos Relaciona	Falhas durante a assistênci		
30/09/2022	CE	SOBRAL	HOSPITA L REGIONA	-	Enviada	Eventos Adversos Relaciona	Falhas durante a assistênci		
30/09/2022	CE	SOBRAL	HOSPITA L REGIONA	COLONO SCOPIA DE DOIS	Enviada	Eventos Adversos Relaciona	Falhas nas atividades		
30/09/2022	CE	SOBRAL	HOSPITA L REGIONA	-	Enviada	Eventos Adversos Relaciona	Falhas durante a assistênci		
30/09/2022	CE	SOBRAL	HOSPITA L REGIONA	-	Enviada	Eventos Adversos Relaciona	Falhas durante a assistênci		

11/10/2022	CE	SOBRAL	HOSPITA L REGIONA	FOI PARA CENTRO	Enviada	Eventos Adversos Relaciona	Falhas durante a assistênci		
11/10/2022	CE	SOBRAL	HOSPITA L REGIONA	FOI PARA CENTRO	Enviada	Eventos Adversos Relaciona	Falhas durante a assistênci		
11/10/2022	CE	SOBRAL	HOSPITA L REGIONA	FOI PARA O CENTRO	Enviada	Eventos Adversos Relaciona	Falhas durante a assistênci		
11/10/2022	CE	SOBRAL	HOSPITA L REGIONA	PACIENT E FOI PARA	Enviada	Eventos Adversos Relaciona	Falhas durante a assistênci		
11/10/2022	CE	SOBRAL	HOSPITA L REGIONA	FOI PARA CENTRO	Enviada	Eventos Adversos Relaciona	Falhas durante a assistênci		
11/10/2022	CE	SOBRAL	HOSPITA L REGIONA	LP ESTAGIO 2 EM	Enviada	Eventos Adversos Relaciona	Lesão por pressão		
11/10/2022	CE	SOBRAL	HOSPITA L REGIONA	-	Enviada	Eventos Adversos Relaciona	Lesão por pressão		
11/10/2022	CE	SOBRAL	HOSPITA L REGIONA	-	Enviada	Eventos Adversos Relaciona	Lesão por pressão		
11/10/2022	CE	SOBRAL	HOSPITA L REGIONA	-	Enviada	Eventos Adversos Relaciona	Lesão por pressão		
11/10/2022	CE	SOBRAL	HOSPITA L REGIONA	-	Enviada	Eventos Adversos Relaciona	Lesão por pressão		
11/10/2022	CE	SOBRAL	HOSPITA L REGIONA	-	Enviada	Eventos Adversos Relaciona	Lesão por pressão		
11/10/2022	CE	SOBRAL	HOSPITA L REGIONA	-	Enviada	Eventos Adversos Relaciona	Lesão por pressão		
11/10/2022	CE	SOBRAL	HOSPITA L REGIONA	-	Enviada	Eventos Adversos Relaciona	Lesão por pressão		
11/10/2022	CE	SOBRAL	HOSPITA L REGIONA	-	Enviada	Eventos Adversos Relaciona	Lesão por pressão		
11/10/2022	CE	SOBRAL	HOSPITA L REGIONA	-	Enviada	Eventos Adversos Relaciona	Lesão por pressão		
11/10/2022	CE	SOBRAL	HOSPITA L REGIONA	-	Enviada	Eventos Adversos Relaciona	Lesão por pressão		
13/10/2022	CE	SOBRAL	HOSPITA L REGIONA	PACIENT E SOFREU	Enviada	Eventos Adversos Relaciona	Queda do paciente		
13/10/2022	CE	SOBRAL	HOSPITA L REGIONA	PACIENT E RECEBID	Enviada	Eventos Adversos Relaciona	Falhas envolvend o cateter		
13/10/2022	CE	SOBRAL	HOSPITA L REGIONA	-	Enviada	Eventos Adversos Relaciona	Falhas na administra ção de		Enteral (oral, cateter

13/10/2022	CE	SOBRAL	HOSPITA L REGIONA	-	Enviada	Eventos Adversos Relaciona	Falhas na administra ção de		Enteral (oral, cateter
13/10/2022	CE	SOBRAL	HOSPITA L REGIONA	-	Enviada	Eventos Adversos Relaciona	Falhas nas atividades		
13/10/2022	CE	SOBRAL	HOSPITA L REGIONA	-	Enviada	Eventos Adversos Relaciona	Lesão por pressão		
13/10/2022	CE	SOBRAL	HOSPITA L REGIONA	-	Enviada	Eventos Adversos Relaciona	Falhas durante a assistênci		
13/10/2022	CE	SOBRAL	HOSPITA L REGIONA	PACIENT E SACOU	Enviada	Eventos Adversos Relaciona	Falhas envolvend o sondas		
14/10/2022	CE	SOBRAL	HOSPITA L REGIONA	-	Enviada	Eventos Adversos Relaciona	Falhas nas atividades		
14/10/2022	CE	SOBRAL	HOSPITA L REGIONA	-	Enviada	Eventos Adversos Relaciona	Lesão por pressão		
14/10/2022	CE	SOBRAL	HOSPITA L REGIONA	-	Enviada	Eventos Adversos Relaciona	Lesão por pressão		
14/10/2022	CE	SOBRAL	HOSPITA L REGIONA	-	Enviada	Eventos Adversos Relaciona	Lesão por pressão		
14/10/2022	CE	SOBRAL	HOSPITA L REGIONA	-	Enviada	Eventos Adversos Relaciona	Lesão por pressão		
14/10/2022	CE	SOBRAL	HOSPITA L REGIONA	-	Enviada	Eventos Adversos Relaciona	Falhas durante a assistênci		
14/10/2022	CE	SOBRAL	HOSPITA L REGIONA	-	Enviada	Eventos Adversos Relaciona	Falhas durante a assistênci		
14/10/2022	CE	SOBRAL	HOSPITA L REGIONA	-	Enviada	Eventos Adversos Relaciona	Falhas durante a assistênci		
14/10/2022	CE	SOBRAL	HOSPITA L REGIONA	-	Enviada	Eventos Adversos Relaciona	Falhas durante a assistênci		
14/10/2022	CE	SOBRAL	HOSPITA L REGIONA	-	Enviada	Eventos Adversos Relaciona	Falhas durante a assistênci		
14/10/2022	CE	SOBRAL	HOSPITA L REGIONA	-	Enviada	Eventos Adversos Relaciona	Falhas durante a assistênci		
14/10/2022	CE	SOBRAL	HOSPITA L REGIONA	-	Enviada	Eventos Adversos Relaciona	Falhas durante a assistênci		
14/10/2022	CE	SOBRAL	HOSPITA L REGIONA	-	Enviada	Eventos Adversos Relaciona	Falhas ocorridas em		
14/10/2022	CE	SOBRAL	HOSPITA L REGIONA	-	Enviada	Eventos Adversos Relaciona	Falha na document ação		

14/10/2022	CE	SOBRAL	HOSPITA L REGIONA	-	Enviada	Eventos Adversos Relaciona	Falhas nas atividades		
14/10/2022	CE	SOBRAL	HOSPITA L REGIONA	-	Enviada	Eventos Adversos Relaciona	Falhas nas atividades		
14/10/2022	CE	SOBRAL	HOSPITA L REGIONA	-	Enviada	Eventos Adversos Relaciona	Falhas na administra ção de		Enteral (oral, cateter
14/10/2022	CE	SOBRAL	HOSPITA L REGIONA	-	Enviada	Eventos Adversos Relaciona	Falhas durante a assistênci		
14/10/2022	CE	SOBRAL	HOSPITA L REGIONA	-	Enviada	Eventos Adversos Relaciona	Falhas durante a assistênci		
14/10/2022	CE	SOBRAL	HOSPITA L REGIONA	-	Enviada	Eventos Adversos Relaciona	Falhas durante a assistênci		
14/10/2022	CE	SOBRAL	HOSPITA L REGIONA	-	Enviada	Eventos Adversos Relaciona	Falhas envolvend o cateter		
14/10/2022	CE	SOBRAL	HOSPITA L REGIONA	-	Enviada	Eventos Adversos Relaciona	Falhas envolvend o sondas		
14/10/2022	CE	SOBRAL	HOSPITA L REGIONA	-	Enviada	Eventos Adversos Relaciona	Falhas durante a assistênci		
14/10/2022	CE	SOBRAL	HOSPITA L REGIONA	-	Enviada	Eventos Adversos Relaciona	Falhas envolvend o cateter		
17/10/2022	CE	SOBRAL	HOSPITA L REGIONA	-	Enviada	Eventos Adversos Relaciona	Falhas envolvend o sondas		
17/10/2022	CE	SOBRAL	HOSPITA L REGIONA	-	Enviada	Eventos Adversos Relaciona	Extubaçã o endotraqu		
17/10/2022	CE	SOBRAL	HOSPITA L REGIONA	-	Enviada	Eventos Adversos Relaciona	Broncoas piração		
17/10/2022	CE	SOBRAL	HOSPITA L REGIONA	-	Enviada	Eventos Adversos Relaciona	Falhas nas atividades		
18/10/2022	CE	SOBRAL	HOSPITA L REGIONA	-	Enviada	Eventos Adversos Relaciona	Falhas envolvend o sondas		
18/10/2022	CE	SOBRAL	HOSPITA L REGIONA	-	Enviada	Eventos Adversos Relaciona	Falhas durante a assistênci		
18/10/2022	CE	SOBRAL	HOSPITA L REGIONA	-	Enviada	Eventos Adversos Relaciona	Falhas durante a assistênci		
20/10/2022	CE	SOBRAL	HOSPITA L REGIONA	-	Enviada	Eventos Adversos Relaciona	Falha na identificaç ão do		
20/10/2022	CE	SOBRAL	HOSPITA L REGIONA	-	Enviada	Eventos Adversos Relaciona	Falhas durante a assistênci		

20/10/2022	CE	SOBRAL	HOSPITA L REGIONA	-	Enviada	Eventos Adversos Relaciona	Falha na identificaç ão do		
20/10/2022	CE	SOBRAL	HOSPITA L REGIONA	-	Enviada	Eventos Adversos Relaciona	Falha na document ação		
20/10/2022	CE	SOBRAL	HOSPITA L REGIONA	-	Enviada	Eventos Adversos Relaciona	Falhas durante a assistênci		
20/10/2022	CE	SOBRAL	HOSPITA L REGIONA	-	Enviada	Eventos Adversos Relaciona	Falhas nas atividades		
20/10/2022	CE	SOBRAL	HOSPITA L REGIONA	-	Enviada	Eventos Adversos Relaciona	Broncoas piração		
20/10/2022	CE	SOBRAL	HOSPITA L REGIONA	-	Enviada	Eventos Adversos Relaciona	Queda do paciente		
20/10/2022	CE	SOBRAL	HOSPITA L REGIONA	-	Enviada	Eventos Adversos Relaciona	Extubaçã o endotraqu		
20/10/2022	CE	SOBRAL	HOSPITA L REGIONA	-	Enviada	Eventos Adversos Relaciona	Falhas durante procedim		
20/10/2022	CE	SOBRAL	HOSPITA L REGIONA	-	Enviada	Eventos Adversos Relaciona	Falha na document ação		
20/10/2022	CE	SOBRAL	HOSPITA L REGIONA	Pulseira de identificaç	Enviada	Eventos Adversos Relaciona	Falha na identificaç ão do		
20/10/2022	CE	SOBRAL	HOSPITA L REGIONA	-	Enviada	Eventos Adversos Relaciona	Falhas durante a assistênci		
20/10/2022	CE	SOBRAL	HOSPITA L REGIONA	-	Enviada	Eventos Adversos Relaciona	Falhas durante a assistênci		
20/10/2022	CE	SOBRAL	HOSPITA L REGIONA	-	Enviada	Eventos Adversos Relaciona	Falhas nas atividades		
20/10/2022	CE	SOBRAL	HOSPITA L REGIONA	-	Enviada	Eventos Adversos Relaciona	Falhas durante a assistênci		
21/10/2022	CE	SOBRAL	HOSPITA L REGIONA	-	Enviada	Eventos Adversos Relaciona	Falhas durante a assistênci		
21/10/2022	CE	SOBRAL	HOSPITA L REGIONA	-	Enviada	Eventos Adversos Relaciona	Falhas durante a assistênci		
21/10/2022	CE	SOBRAL	HOSPITA L REGIONA	-	Enviada	Eventos Adversos Relaciona	Falhas durante a assistênci		
21/10/2022	CE	SOBRAL	HOSPITA L REGIONA	-	Enviada	Eventos Adversos Relaciona	Falhas durante a assistênci		
21/10/2022	CE	SOBRAL	HOSPITA L REGIONA	-	Enviada	Eventos Adversos Relaciona	Falhas durante a assistênci		

21/10/2022	CE	SOBRAL	HOSPITA L REGIONA	-	Enviada	Eventos Adversos Relaciona	Falhas durante a assistênci		
21/10/2022	CE	SOBRAL	HOSPITA L REGIONA	-	Enviada	Eventos Adversos Relaciona	Falhas durante a assistênci		
21/10/2022	CE	SOBRAL	HOSPITA L REGIONA	-	Enviada	Eventos Adversos Relaciona	Queda do paciente		
24/10/2022	CE	SOBRAL	HOSPITA L REGIONA	-	Enviada	Eventos Adversos Relaciona	Lesão por pressão		
24/10/2022	CE	SOBRAL	HOSPITA L REGIONA	-	Enviada	Eventos Adversos Relaciona	Lesão por pressão		
24/10/2022	CE	SOBRAL	HOSPITA L REGIONA	-	Enviada	Eventos Adversos Relaciona	Falhas durante a assistênci		
24/10/2022	CE	SOBRAL	HOSPITA L REGIONA	-	Enviada	Eventos Adversos Relaciona	Falha na document ação		
24/10/2022	CE	SOBRAL	HOSPITA L REGIONA	-	Enviada	Eventos Adversos Relaciona	Falhas durante a assistênci		
24/10/2022	CE	SOBRAL	HOSPITA L REGIONA	-	Enviada	Eventos Adversos Relaciona	Falhas nas atividades		
24/10/2022	CE	SOBRAL	HOSPITA L REGIONA	-	Enviada	Eventos Adversos Relaciona	Falhas durante a assistênci		
24/10/2022	CE	SOBRAL	HOSPITA L REGIONA	-	Enviada	Eventos Adversos Relaciona	Falhas envolvend o sondas		
24/10/2022	CE	SOBRAL	HOSPITA L REGIONA	-	Enviada	Eventos Adversos Relaciona	Falhas nas atividades		
24/10/2022	CE	SOBRAL	HOSPITA L REGIONA	-	Enviada	Eventos Adversos Relaciona	Lesão por pressão		
24/10/2022	CE	SOBRAL	HOSPITA L REGIONA	-	Enviada	Eventos Adversos Relaciona	Falhas durante a assistênci		
24/10/2022	CE	SOBRAL	HOSPITA L REGIONA	-	Enviada	Eventos Adversos Relaciona	Falhas durante a assistênci		
24/10/2022	CE	SOBRAL	HOSPITA L REGIONA	-	Enviada	Eventos Adversos Relaciona	Falhas durante a assistênci		
24/10/2022	CE	SOBRAL	HOSPITA L REGIONA	-	Enviada	Eventos Adversos Relaciona	Falhas nas atividades		
24/10/2022	CE	SOBRAL	HOSPITA L REGIONA	-	Enviada	Eventos Adversos Relaciona	Lesão por pressão		
24/10/2022	CE	SOBRAL	HOSPITA L REGIONA	-	Enviada	Eventos Adversos Relaciona	Falha na document ação		

24/10/2022	CE	SOBRAL	HOSPITA L REGIONA	-	Enviada	Eventos Adversos Relaciona	Falha na document ação		
24/10/2022	CE	SOBRAL	HOSPITA L REGIONA	-	Enviada	Eventos Adversos Relaciona	Falhas durante a assistênci		
24/10/2022	CE	SOBRAL	HOSPITA L REGIONA	-	Enviada	Eventos Adversos Relaciona	Falhas durante a assistênci		
24/10/2022	CE	SOBRAL	HOSPITA L REGIONA	-	Enviada	Eventos Adversos Relaciona	Falha na identificaç ão do		
24/10/2022	CE	SOBRAL	HOSPITA L REGIONA	-	Enviada	Eventos Adversos Relaciona	Falhas na administra ção de		Enteral (oral, cateter
24/10/2022	CE	SOBRAL	HOSPITA L REGIONA	-	Enviada	Eventos Adversos Relaciona	Queda do paciente		
01/11/2022	CE	SOBRAL	HOSPITA L REGIONA	-	Enviada	Eventos Adversos Relaciona	Falhas durante a assistênci		
01/11/2022	CE	SOBRAL	HOSPITA L REGIONA	-	Enviada	Eventos Adversos Relaciona	Falhas nas atividades		
01/11/2022	CE	SOBRAL	HOSPITA L REGIONA	-	Enviada	Eventos Adversos Relaciona	Falha na document ação		
01/11/2022	CE	SOBRAL	HOSPITA L REGIONA	-	Enviada	Eventos Adversos Relaciona	Falhas envolvend o cateter		
01/11/2022	CE	SOBRAL	HOSPITA L REGIONA	-	Enviada	Eventos Adversos Relaciona	Falha na document ação		
01/11/2022	CE	SOBRAL	HOSPITA L REGIONA	-	Enviada	Eventos Adversos Relaciona	Falhas durante a assistênci		
01/11/2022	CE	SOBRAL	HOSPITA L REGIONA	-	Enviada	Eventos Adversos Relaciona	Lesão por pressão		
01/11/2022	CE	SOBRAL	HOSPITA L REGIONA	-	Enviada	Eventos Adversos Relaciona	Falhas durante a assistênci		
01/11/2022	CE	SOBRAL	HOSPITA L REGIONA	-	Enviada	Eventos Adversos Relaciona	Falhas durante a assistênci		
01/11/2022	CE	SOBRAL	HOSPITA L REGIONA	-	Enviada	Eventos Adversos Relaciona	Falhas durante a assistênci		
01/11/2022	CE	SOBRAL	HOSPITA L REGIONA	-	Enviada	Eventos Adversos Relaciona	Falhas durante a assistênci		
04/11/2022	CE	SOBRAL	HOSPITA L REGIONA	-	Enviada	Eventos Adversos Relaciona	Lesão por pressão		
04/11/2022	CE	SOBRAL	HOSPITA L REGIONA	-	Enviada	Eventos Adversos Relaciona	Lesão por pressão		

04/11/2022	CE	SOBRAL	HOSPITA L REGIONA	-	Enviada	Eventos Adversos Relaciona	Lesão por pressão		
04/11/2022	CE	SOBRAL	HOSPITA L REGIONA	-	Enviada	Eventos Adversos Relaciona	Lesão por pressão		
07/11/2022	CE	SOBRAL	HOSPITA L REGIONA	-	Enviada	Eventos Adversos Relaciona	Falhas durante a assistênci		
07/11/2022	CE	SOBRAL	HOSPITA L REGIONA	-	Enviada	Eventos Adversos Relaciona	Falhas durante a assistênci		
07/11/2022	CE	SOBRAL	HOSPITA L REGIONA	-	Enviada	Eventos Adversos Relaciona	Falhas durante a assistênci		
07/11/2022	CE	SOBRAL	HOSPITA L REGIONA	-	Enviada	Eventos Adversos Relaciona	Falhas durante a assistênci		
07/11/2022	CE	SOBRAL	HOSPITA L REGIONA	-	Enviada	Eventos Adversos Relaciona	Falhas durante a assistênci		
08/11/2022	CE	SOBRAL	HOSPITA L REGIONA	-	Enviada	Eventos Adversos Relaciona	Falhas durante a assistênci		
08/11/2022	CE	SOBRAL	HOSPITA L REGIONA	-	Enviada	Eventos Adversos Relaciona	Falhas durante a assistênci		
08/11/2022	CE	SOBRAL	HOSPITA L REGIONA	-	Enviada	Eventos Adversos Relaciona	Falhas durante a assistênci		
08/11/2022	CE	SOBRAL	HOSPITA L REGIONA	-	Enviada	Eventos Adversos Relaciona	Falhas durante a assistênci		
08/11/2022	CE	SOBRAL	HOSPITA L REGIONA	-	Enviada	Eventos Adversos Relaciona	Falhas durante a assistênci		
08/11/2022	CE	SOBRAL	HOSPITA L REGIONA	-	Enviada	Eventos Adversos Relaciona	Falhas durante a assistênci		
08/11/2022	CE	SOBRAL	HOSPITA L REGIONA	-	Enviada	Eventos Adversos Relaciona	Falhas durante a assistênci		
08/11/2022	CE	SOBRAL	HOSPITA L REGIONA	-	Enviada	Eventos Adversos Relaciona	Falhas durante a assistênci		
08/11/2022	CE	SOBRAL	HOSPITA L REGIONA	-	Enviada	Eventos Adversos Relaciona	Falhas durante a assistênci		
09/11/2022	CE	SOBRAL	HOSPITA L REGIONA	-	Enviada	Eventos Adversos Relaciona	Falhas durante a assistênci		
09/11/2022	CE	SOBRAL	HOSPITA L REGIONA	-	Enviada	Eventos Adversos Relaciona	Falhas ocorridas em		
09/11/2022	CE	SOBRAL	HOSPITA L REGIONA	-	Enviada	Eventos Adversos Relaciona	Falha na document ação		

09/11/2022	CE	SOBRAL	HOSPITA L REGIONA	-	Enviada	Eventos Adversos Relaciona	Falha na document ação		
09/11/2022	CE	SOBRAL	HOSPITA L REGIONA	-	Enviada	Eventos Adversos Relaciona	Falhas durante a assistênci		
09/11/2022	CE	SOBRAL	HOSPITA L REGIONA	-	Enviada	Eventos Adversos Relaciona	Falhas nas atividades		
09/11/2022	CE	SOBRAL	HOSPITA L REGIONA	-	Enviada	Eventos Adversos Relaciona	Falhas na administra ção de		Enteral (oral, cateter
09/11/2022	CE	SOBRAL	HOSPITA L REGIONA	-	Enviada	Eventos Adversos Relaciona	Queda do paciente		
09/11/2022	CE	SOBRAL	HOSPITA L REGIONA	-	Enviada	Eventos Adversos Relaciona	Queda do paciente		
09/11/2022	CE	SOBRAL	HOSPITA L REGIONA	-	Enviada	Eventos Adversos Relaciona	Falhas durante a assistênci		
09/11/2022	CE	SOBRAL	HOSPITA L REGIONA	-	Enviada	Eventos Adversos Relaciona	Falhas durante a assistênci		
09/11/2022	CE	SOBRAL	HOSPITA L REGIONA	-	Enviada	Eventos Adversos Relaciona	Falhas na administra ção de		Enteral (oral, cateter
11/11/2022	CE	SOBRAL	HOSPITA L REGIONA	-	Enviada	Eventos Adversos Relaciona	Falha na document ação		
11/11/2022	CE	SOBRAL	HOSPITA L REGIONA	-	Enviada	Eventos Adversos Relaciona	Falhas nas atividades		
11/11/2022	CE	SOBRAL	HOSPITA L REGIONA	-	Enviada	Eventos Adversos Relaciona	Falhas durante a assistênci		
11/11/2022	CE	SOBRAL	HOSPITA L REGIONA	-	Enviada	Eventos Adversos Relaciona	Falhas nas atividades		
11/11/2022	CE	SOBRAL	HOSPITA L REGIONA	-	Enviada	Eventos Adversos Relaciona	Falhas nas atividades		
11/11/2022	CE	SOBRAL	HOSPITA L REGIONA	-	Enviada	Eventos Adversos Relaciona	Queda do paciente		
11/11/2022	CE	SOBRAL	HOSPITA L REGIONA	-	Enviada	Eventos Adversos Relaciona	Falhas durante a assistênci		
11/11/2022	CE	SOBRAL	HOSPITA L REGIONA	-	Enviada	Eventos Adversos Relaciona	Falhas durante a assistênci		
11/11/2022	CE	SOBRAL	HOSPITA L REGIONA	-	Enviada	Eventos Adversos Relaciona	Falhas durante a assistênci		
11/11/2022	CE	SOBRAL	HOSPITA L REGIONA	-	Enviada	Eventos Adversos Relaciona	Falhas durante a assistênci		

11/11/2022	CE	SOBRAL	HOSPITA L REGIONA	-	Enviada	Eventos Adversos Relaciona	Falhas durante a assistênci		
11/11/2022	CE	SOBRAL	HOSPITA L REGIONA	-	Enviada	Eventos Adversos Relaciona	Falhas nas atividades		
11/11/2022	CE	SOBRAL	HOSPITA L REGIONA	-	Enviada	Eventos Adversos Relaciona	Falhas envolvend o cateter		
24/11/2022	CE	SOBRAL	HOSPITA L REGIONA	-	Enviada	Eventos Adversos Relaciona	Lesão por pressão		
24/11/2022	CE	SOBRAL	HOSPITA L REGIONA	-	Enviada	Eventos Adversos Relaciona	Lesão por pressão		
24/11/2022	CE	SOBRAL	HOSPITA L REGIONA	-	Enviada	Eventos Adversos Relaciona	Lesão por pressão		
24/11/2022	CE	SOBRAL	HOSPITA L REGIONA	-	Enviada	Eventos Adversos Relaciona	Falhas durante a assistênci		
24/11/2022	CE	SOBRAL	HOSPITA L REGIONA	-	Enviada	Eventos Adversos Relaciona	Falhas durante a assistênci		
24/11/2022	CE	SOBRAL	HOSPITA L REGIONA	-	Enviada	Eventos Adversos Relaciona	Falhas durante a assistênci		
24/11/2022	CE	SOBRAL	HOSPITA L REGIONA	-	Enviada	Eventos Adversos Relaciona	Falhas durante a assistênci		
24/11/2022	CE	SOBRAL	HOSPITA L REGIONA	-	Enviada	Eventos Adversos Relaciona	Falhas durante a assistênci		
24/11/2022	CE	SOBRAL	HOSPITA L REGIONA	-	Enviada	Eventos Adversos Relaciona	Falhas durante a assistênci		
24/11/2022	CE	SOBRAL	HOSPITA L REGIONA	-	Enviada	Eventos Adversos Relaciona	Falhas durante a assistênci		
24/11/2022	CE	SOBRAL	HOSPITA L REGIONA	-	Enviada	Eventos Adversos Relaciona	Falhas durante a assistênci		
24/11/2022	CE	SOBRAL	HOSPITA L REGIONA	-	Enviada	Eventos Adversos Relaciona	Falhas durante a assistênci		
24/11/2022	CE	SOBRAL	HOSPITA L REGIONA	-	Enviada	Eventos Adversos Relaciona	Falhas durante a assistênci		
24/11/2022	CE	SOBRAL	HOSPITA L REGIONA	-	Enviada	Eventos Adversos Relaciona	Falhas durante a assistênci		
24/11/2022	CE	SOBRAL	HOSPITA L REGIONA	-	Enviada	Eventos Adversos Relaciona	Falhas envolvend o cateter		
24/11/2022	CE	SOBRAL	HOSPITA L REGIONA	-	Enviada	Eventos Adversos Relaciona	Falha na identificaç ão do		

24/11/2022	CE	SOBRAL	HOSPITAL REGIONA	-	Enviada	Eventos Adversos Relaciona	Falhas nas atividades		
24/11/2022	CE	SOBRAL	HOSPITAL REGIONA	-	Enviada	Eventos Adversos Relaciona	Lesão por pressão		
24/11/2022	CE	SOBRAL	HOSPITAL REGIONA	-	Enviada	Eventos Adversos Relaciona	Queda do paciente		
24/11/2022	CE	SOBRAL	HOSPITAL REGIONA	-	Enviada	Eventos Adversos Relaciona	Falhas durante a assistênci		
24/11/2022	CE	SOBRAL	HOSPITAL REGIONA	-	Enviada	Eventos Adversos Relaciona	Tromboembolismo venoso		
24/11/2022	CE	SOBRAL	HOSPITAL REGIONA	-	Enviada	Eventos Adversos Relaciona	Tromboembolismo venoso		
24/11/2022	CE	SOBRAL	HOSPITAL REGIONA	-	Enviada	Eventos Adversos Relaciona	Falhas nas atividades		
24/11/2022	CE	SOBRAL	HOSPITAL REGIONA	-	Enviada	Eventos Adversos Relaciona	Queda do paciente		
24/11/2022	CE	SOBRAL	HOSPITAL REGIONA	-	Enviada	Eventos Adversos Relaciona	Falhas durante a assistênci		
24/11/2022	CE	SOBRAL	HOSPITAL REGIONA	-	Enviada	Eventos Adversos Relaciona	Falhas durante a assistênci		
24/11/2022	CE	SOBRAL	HOSPITAL REGIONA	-	Enviada	Eventos Adversos Relaciona	Falhas nas atividades		
24/11/2022	CE	SOBRAL	HOSPITAL REGIONA	-	Enviada	Eventos Adversos Relaciona	Falhas durante a assistênci		
24/11/2022	CE	SOBRAL	HOSPITAL REGIONA	-	Enviada	Eventos Adversos Relaciona	Falhas nas atividades		
24/11/2022	CE	SOBRAL	HOSPITAL REGIONA	-	Enviada	Eventos Adversos Relaciona	Falhas durante a assistênci		
24/11/2022	CE	SOBRAL	HOSPITAL REGIONA	-	Enviada	Eventos Adversos Relaciona	Falhas durante a assistênci		
24/11/2022	CE	SOBRAL	HOSPITAL REGIONA	-	Enviada	Eventos Adversos Relaciona	Falhas nas atividades		
24/11/2022	CE	SOBRAL	HOSPITAL REGIONA	-	Enviada	Eventos Adversos Relaciona	Falhas nas atividades		
24/11/2022	CE	SOBRAL	HOSPITAL REGIONA	-	Enviada	Eventos Adversos Relaciona	Queda do paciente		
24/11/2022	CE	SOBRAL	HOSPITAL REGIONA	-	Enviada	Eventos Adversos Relaciona	Lesão por pressão		

24/11/2022	CE	SOBRAL	HOSPITA L REGIONA	-	Enviada	Eventos Adversos Relaciona	Falhas na administra ção de		Enteral (oral, cateter
24/11/2022	CE	SOBRAL	HOSPITA L REGIONA	-	Enviada	Eventos Adversos Relaciona	Falhas na administra ção de		Enteral (oral, cateter
24/11/2022	CE	SOBRAL	HOSPITA L REGIONA	-	Enviada	Eventos Adversos Relaciona	Falhas na administra ção de		Enteral (oral, cateter
24/11/2022	CE	SOBRAL	HOSPITA L REGIONA	-	Enviada	Eventos Adversos Relaciona	Falhas nas atividades		
05/12/2022	CE	SOBRAL	HOSPITA L REGIONA	-	Enviada	Eventos Adversos Relaciona	Falhas durante a assistênci		
05/12/2022	CE	SOBRAL	HOSPITA L REGIONA	-	Enviada	Eventos Adversos Relaciona	Falhas durante a assistênci		
05/12/2022	CE	SOBRAL	HOSPITA L REGIONA	-	Enviada	Eventos Adversos Relaciona	Falhas durante a assistênci		
05/12/2022	CE	SOBRAL	HOSPITA L REGIONA	-	Enviada	Eventos Adversos Relaciona	Falhas durante a assistênci		
05/12/2022	CE	SOBRAL	HOSPITA L REGIONA	-	Enviada	Eventos Adversos Relaciona	Falhas durante a assistênci		
05/12/2022	CE	SOBRAL	HOSPITA L REGIONA	-	Enviada	Eventos Adversos Relaciona	Falhas durante a assistênci		
05/12/2022	CE	SOBRAL	HOSPITA L REGIONA	-	Enviada	Eventos Adversos Relaciona	Falhas durante a assistênci		
05/12/2022	CE	SOBRAL	HOSPITA L REGIONA	-	Enviada	Eventos Adversos Relaciona	Falhas durante a assistênci		
05/12/2022	CE	SOBRAL	HOSPITA L REGIONA	-	Enviada	Eventos Adversos Relaciona	Falhas durante a assistênci		
05/12/2022	CE	SOBRAL	HOSPITA L REGIONA	-	Enviada	Eventos Adversos Relaciona	Falhas durante a assistênci		
05/12/2022	CE	SOBRAL	HOSPITA L REGIONA	-	Enviada	Eventos Adversos Relaciona	Falhas durante a assistênci		
06/12/2022	CE	SOBRAL	HOSPITA L REGIONA	-	Enviada	Eventos Adversos Relaciona	Falhas na administra ção de		Enteral (oral, cateter
06/12/2022	CE	SOBRAL	HOSPITA L REGIONA	-	Enviada	Eventos Adversos Relaciona	Lesão por pressão		
06/12/2022	CE	SOBRAL	HOSPITA L REGIONA	-	Enviada	Eventos Adversos Relaciona	Lesão por pressão		
06/12/2022	CE	SOBRAL	HOSPITA L REGIONA	-	Enviada	Eventos Adversos Relaciona	Falhas durante a assistênci		

06/12/2022	CE	SOBRAL	HOSPITA L REGIONA	-	Enviada	Eventos Adversos Relaciona	Falhas durante a assistênci		
06/12/2022	CE	SOBRAL	HOSPITA L REGIONA	-	Enviada	Eventos Adversos Relaciona	Lesão por pressão		
06/12/2022	CE	SOBRAL	HOSPITA L REGIONA	-	Enviada	Eventos Adversos Relaciona	Queda do paciente		
06/12/2022	CE	SOBRAL	HOSPITA L REGIONA	-	Enviada	Eventos Adversos Relaciona	Queda do paciente		
06/12/2022	CE	SOBRAL	HOSPITA L REGIONA	-	Enviada	Eventos Adversos Relaciona	Falhas durante a assistênci		
06/12/2022	CE	SOBRAL	HOSPITA L REGIONA	-	Enviada	Eventos Adversos Relaciona	Falhas durante a assistênci		
06/12/2022	CE	SOBRAL	HOSPITA L REGIONA	-	Enviada	Eventos Adversos Relaciona	Falhas durante a assistênci		
06/12/2022	CE	SOBRAL	HOSPITA L REGIONA	-	Enviada	Eventos Adversos Relaciona	Falhas durante a assistênci		
06/12/2022	CE	SOBRAL	HOSPITA L REGIONA	-	Enviada	Eventos Adversos Relaciona	Falhas durante a assistênci		
06/12/2022	CE	SOBRAL	HOSPITA L REGIONA	-	Enviada	Eventos Adversos Relaciona	Falhas na administra ção de		Enteral (oral, cateter
06/12/2022	CE	SOBRAL	HOSPITA L REGIONA	-	Enviada	Eventos Adversos Relaciona	Falhas na administra ção de		Enteral (oral, cateter
06/12/2022	CE	SOBRAL	HOSPITA L REGIONA	-	Enviada	Eventos Adversos Relaciona	Lesão por pressão		
06/12/2022	CE	SOBRAL	HOSPITA L REGIONA	-	Enviada	Eventos Adversos Relaciona	Falhas na administra ção de		Enteral (oral, cateter
06/12/2022	CE	SOBRAL	HOSPITA L REGIONA	-	Enviada	Eventos Adversos Relaciona	Falhas na administra ção de		Enteral (oral, cateter
06/12/2022	CE	SOBRAL	HOSPITA L REGIONA	-	Enviada	Eventos Adversos Relaciona	Falhas durante a assistênci		
06/12/2022	CE	SOBRAL	HOSPITA L REGIONA	-	Enviada	Eventos Adversos Relaciona	Queda do paciente		
06/12/2022	CE	SOBRAL	HOSPITA L REGIONA	-	Enviada	Eventos Adversos Relaciona	Falha na identificaç ão do		
06/12/2022	CE	SOBRAL	HOSPITA L REGIONA	-	Enviada	Eventos Adversos Relaciona	Queda do paciente		
06/12/2022	CE	SOBRAL	HOSPITA L REGIONA	-	Enviada	Eventos Adversos Relaciona	Queda do paciente		

06/12/2022	CE	SOBRAL	HOSPITA L REGIONA	-	Enviada	Eventos Adversos Relaciona	Lesão por pressão		
06/12/2022	CE	SOBRAL	HOSPITA L REGIONA	-	Enviada	Eventos Adversos Relaciona	Falhas durante a assistênci		
06/12/2022	CE	SOBRAL	HOSPITA L REGIONA	-	Enviada	Eventos Adversos Relaciona	Falhas nas atividades		
06/12/2022	CE	SOBRAL	HOSPITA L REGIONA	-	Enviada	Eventos Adversos Relaciona	Falhas durante a assistênci		
06/12/2022	CE	SOBRAL	HOSPITA L REGIONA	-	Enviada	Eventos Adversos Relaciona	Falhas durante a assistênci		
06/12/2022	CE	SOBRAL	HOSPITA L REGIONA	-	Enviada	Eventos Adversos Relaciona	Falhas envolvend o cateter		
06/12/2022	CE	SOBRAL	HOSPITA L REGIONA	-	Enviada	Eventos Adversos Relaciona	Falhas envolvend o sondas		
06/12/2022	CE	SOBRAL	HOSPITA L REGIONA	-	Enviada	Eventos Adversos Relaciona	Falhas durante a assistênci		
06/12/2022	CE	SOBRAL	HOSPITA L REGIONA	-	Enviada	Eventos Adversos Relaciona	Falhas durante a assistênci		
06/12/2022	CE	SOBRAL	HOSPITA L REGIONA	-	Enviada	Eventos Adversos Relaciona	Lesão por pressão		
06/12/2022	CE	SOBRAL	HOSPITA L REGIONA	-	Enviada	Eventos Adversos Relaciona	Falhas durante a assistênci		
06/12/2022	CE	SOBRAL	HOSPITA L REGIONA	-	Enviada	Eventos Adversos Relaciona	Falhas envolvend o sondas		
06/12/2022	CE	SOBRAL	HOSPITA L REGIONA	-	Enviada	Eventos Adversos Relaciona	Falhas durante a assistênci		
06/12/2022	CE	SOBRAL	HOSPITA L REGIONA	-	Enviada	Eventos Adversos Relaciona	Falhas durante a assistênci		
06/12/2022	CE	SOBRAL	HOSPITA L REGIONA	-	Enviada	Eventos Adversos Relaciona	Falhas durante a assistênci		
06/12/2022	CE	SOBRAL	HOSPITA L REGIONA	-	Enviada	Eventos Adversos Relaciona	Lesão por pressão		
06/12/2022	CE	SOBRAL	HOSPITA L REGIONA	-	Enviada	Eventos Adversos Relaciona	Lesão por pressão		
07/12/2022	CE	SOBRAL	HOSPITA L REGIONA	-	Enviada	Eventos Adversos Relaciona	Lesão por pressão		
07/12/2022	CE	SOBRAL	HOSPITA L REGIONA	-	Enviada	Eventos Adversos Relaciona	Lesão por pressão		

07/12/2022	CE	SOBRAL	HOSPITAL REGIONA	-	Enviada	Eventos Adversos Relaciona	Falhas durante a assistênci		
07/12/2022	CE	SOBRAL	HOSPITAL REGIONA	-	Enviada	Eventos Adversos Relaciona	Lesão por pressão		
07/12/2022	CE	SOBRAL	HOSPITAL REGIONA	-	Enviada	Eventos Adversos Relaciona	Falhas durante a assistênci		
07/12/2022	CE	SOBRAL	HOSPITAL REGIONA	-	Enviada	Eventos Adversos Relaciona	Falhas durante a assistênci		
07/12/2022	CE	SOBRAL	HOSPITAL REGIONA	-	Enviada	Eventos Adversos Relaciona	Falhas durante a assistênci		
07/12/2022	CE	SOBRAL	HOSPITAL REGIONA	-	Enviada	Eventos Adversos Relaciona	Falhas durante a assistênci		
07/12/2022	CE	SOBRAL	HOSPITAL REGIONA	PACIENTE APRESE	Enviada	Eventos Adversos Relaciona	Tromboembolismo venoso		
07/12/2022	CE	SOBRAL	HOSPITAL REGIONA	-	Enviada	Eventos Adversos Relaciona	Falhas envolvendo o cateter		
07/12/2022	CE	SOBRAL	HOSPITAL REGIONA	-	Enviada	Eventos Adversos Relaciona	Falhas durante a assistênci		
07/12/2022	CE	SOBRAL	HOSPITAL REGIONA	-	Enviada	Eventos Adversos Relaciona	Falhas durante a assistênci		
07/12/2022	CE	SOBRAL	HOSPITAL REGIONA	-	Enviada	Eventos Adversos Relaciona	Lesão por pressão		
07/12/2022	CE	SOBRAL	HOSPITAL REGIONA	-	Enviada	Eventos Adversos Relaciona	Falhas durante a assistênci		
07/12/2022	CE	SOBRAL	HOSPITAL REGIONA	-	Enviada	Eventos Adversos Relaciona	Falhas envolvendo sondas		
07/12/2022	CE	SOBRAL	HOSPITAL REGIONA	-	Enviada	Eventos Adversos Relaciona	Falhas durante a assistênci		
07/12/2022	CE	SOBRAL	HOSPITAL REGIONA	-	Enviada	Eventos Adversos Relaciona	Falhas durante a assistênci		
07/12/2022	CE	SOBRAL	HOSPITAL REGIONA	-	Enviada	Eventos Adversos Relaciona	Falhas durante a assistênci		
07/12/2022	CE	SOBRAL	HOSPITAL REGIONA	-	Enviada	Eventos Adversos Relaciona	Falhas envolvendo o cateter		
07/12/2022	CE	SOBRAL	HOSPITAL REGIONA	-	Enviada	Eventos Adversos Relaciona	Falhas durante a assistênci		
07/12/2022	CE	SOBRAL	HOSPITAL REGIONA	-	Enviada	Eventos Adversos Relaciona	Falhas envolvendo o cateter		

07/12/2022	CE	SOBRAL	HOSPITA L REGIONA	-	Enviada	Eventos Adversos Relaciona	Falhas durante a assistênci		
07/12/2022	CE	SOBRAL	HOSPITA L REGIONA	-	Enviada	Eventos Adversos Relaciona	Falhas nas atividades		
07/12/2022	CE	SOBRAL	HOSPITA L REGIONA	-	Enviada	Eventos Adversos Relaciona	Falhas durante a assistênci		
07/12/2022	CE	SOBRAL	HOSPITA L REGIONA	-	Enviada	Eventos Adversos Relaciona	Falhas durante a assistênci		
07/12/2022	CE	SOBRAL	HOSPITA L REGIONA	-	Enviada	Eventos Adversos Relaciona	Falhas durante a assistênci		
07/12/2022	CE	SOBRAL	HOSPITA L REGIONA	-	Enviada	Eventos Adversos Relaciona	Falhas nas atividades		
07/12/2022	CE	SOBRAL	HOSPITA L REGIONA	-	Enviada	Eventos Adversos Relaciona	Falhas nas atividades		
07/12/2022	CE	SOBRAL	HOSPITA L REGIONA	-	Enviada	Eventos Adversos Relaciona	Falha na document ação		
07/12/2022	CE	SOBRAL	HOSPITA L REGIONA	-	Enviada	Eventos Adversos Relaciona	Falhas durante a assistênci		
07/12/2022	CE	SOBRAL	HOSPITA L REGIONA	-	Enviada	Eventos Adversos Relaciona	Falha na document ação		
07/12/2022	CE	SOBRAL	HOSPITA L REGIONA	-	Enviada	Eventos Adversos Relaciona	Lesão por pressão		
07/12/2022	CE	SOBRAL	HOSPITA L REGIONA	-	Enviada	Eventos Adversos Relaciona	Lesão por pressão		
07/12/2022	CE	SOBRAL	HOSPITA L REGIONA	-	Enviada	Eventos Adversos Relaciona	Falhas envolvend o sondas		
12/12/2022	CE	SOBRAL	HOSPITA L REGIONA	-	Enviada	Eventos Adversos Relaciona	Tromboe mbolismo venoso		
12/12/2022	CE	SOBRAL	HOSPITA L REGIONA	-	Enviada	Eventos Adversos Relaciona	Lesão por pressão		
12/12/2022	CE	SOBRAL	HOSPITA L REGIONA	-	Enviada	Eventos Adversos Relaciona	Lesão por pressão		
12/12/2022	CE	SOBRAL	HOSPITA L REGIONA	-	Enviada	Eventos Adversos Relaciona	Falhas durante a assistênci		
12/12/2022	CE	SOBRAL	HOSPITA L REGIONA	-	Enviada	Eventos Adversos Relaciona	Queda do paciente		
12/12/2022	CE	SOBRAL	HOSPITA L REGIONA	-	Enviada	Eventos Adversos Relaciona	Falhas durante a assistênci		

12/12/2022	CE	SOBRAL	HOSPITA L REGIONA	-	Enviada	Eventos Adversos Relaciona	Queda do paciente		
12/12/2022	CE	SOBRAL	HOSPITA L REGIONA	-	Enviada	Eventos Adversos Relaciona	Queda do paciente		
12/12/2022	CE	SOBRAL	HOSPITA L REGIONA	-	Enviada	Eventos Adversos Relaciona	Falha na document ação		
12/12/2022	CE	SOBRAL	HOSPITA L REGIONA	-	Enviada	Eventos Adversos Relaciona	Falhas durante a assistênci		
12/12/2022	CE	SOBRAL	HOSPITA L REGIONA	-	Enviada	Eventos Adversos Relaciona	Falhas durante a assistênci		
12/12/2022	CE	SOBRAL	HOSPITA L REGIONA	-	Enviada	Eventos Adversos Relaciona	Queda do paciente		
12/12/2022	CE	SOBRAL	HOSPITA L REGIONA	-	Enviada	Eventos Adversos Relaciona	Falhas durante a assistênci		
12/12/2022	CE	SOBRAL	HOSPITA L REGIONA	-	Enviada	Eventos Adversos Relaciona	Falhas durante a assistênci		
12/12/2022	CE	SOBRAL	HOSPITA L REGIONA	-	Enviada	Eventos Adversos Relaciona	Falhas durante a assistênci		
12/12/2022	CE	SOBRAL	HOSPITA L REGIONA	-	Enviada	Eventos Adversos Relaciona	Broncoas piração		
12/12/2022	CE	SOBRAL	HOSPITA L REGIONA	-	Enviada	Eventos Adversos Relaciona	Falha na document ação		
12/12/2022	CE	SOBRAL	HOSPITA L REGIONA	-	Enviada	Eventos Adversos Relaciona	Falhas na administra ção de		Enteral (oral, cateter
12/12/2022	CE	SOBRAL	HOSPITA L REGIONA	-	Enviada	Eventos Adversos Relaciona	Tromboe mbolismo venoso		
12/12/2022	CE	SOBRAL	HOSPITA L REGIONA	-	Enviada	Eventos Adversos Relaciona	Lesão por pressão		
12/12/2022	CE	SOBRAL	HOSPITA L REGIONA	-	Enviada	Eventos Adversos Relaciona	Lesão por pressão		
12/12/2022	CE	SOBRAL	HOSPITA L REGIONA	-	Enviada	Eventos Adversos Relaciona	Falhas nas atividades		
12/12/2022	CE	SOBRAL	HOSPITA L REGIONA	-	Enviada	Eventos Adversos Relaciona	Falhas envolvend o cateter		
12/12/2022	CE	SOBRAL	HOSPITA L REGIONA	-	Enviada	Eventos Adversos Relaciona	Lesão por pressão		
12/12/2022	CE	SOBRAL	HOSPITA L REGIONA	-	Enviada	Eventos Adversos Relaciona	Falhas ocorridas em		

12/12/2022	CE	SOBRAL	HOSPITAL REGIONA	-	Enviada	Eventos Adversos Relaciona	Falhas durante a assistênci		
12/12/2022	CE	SOBRAL	HOSPITAL REGIONA	-	Enviada	Eventos Adversos Relaciona	Lesão por pressão		
12/12/2022	CE	SOBRAL	HOSPITAL REGIONA	-	Enviada	Eventos Adversos Relaciona	Falhas durante a assistênci		
12/12/2022	CE	SOBRAL	HOSPITAL REGIONA	-	Enviada	Eventos Adversos Relaciona	Falhas durante a assistênci		
12/12/2022	CE	SOBRAL	HOSPITAL REGIONA	-	Enviada	Eventos Adversos Relaciona	Falhas durante a assistênci		
12/12/2022	CE	SOBRAL	HOSPITAL REGIONA	-	Enviada	Eventos Adversos Relaciona	Queda do paciente		
12/12/2022	CE	SOBRAL	HOSPITAL REGIONA	-	Enviada	Eventos Adversos Relaciona	Falhas durante a assistênci		
12/12/2022	CE	SOBRAL	HOSPITAL REGIONA	-	Enviada	Eventos Adversos Relaciona	Falhas ocorridas em		
12/12/2022	CE	SOBRAL	HOSPITAL REGIONA	-	Enviada	Eventos Adversos Relaciona	Queda do paciente		
12/12/2022	CE	SOBRAL	HOSPITAL REGIONA	-	Enviada	Eventos Adversos Relaciona	Falhas envolvendo o cateter		
12/12/2022	CE	SOBRAL	HOSPITAL REGIONA	-	Enviada	Eventos Adversos Relaciona	Queda do paciente		
12/12/2022	CE	SOBRAL	HOSPITAL REGIONA	-	Enviada	Eventos Adversos Relaciona	Falha na identificação do		
12/12/2022	CE	SOBRAL	HOSPITAL REGIONA	-	Enviada	Eventos Adversos Relaciona	Falhas durante a assistênci		
12/12/2022	CE	SOBRAL	HOSPITAL REGIONA	-	Enviada	Eventos Adversos Relaciona	Lesão por pressão		
12/12/2022	CE	SOBRAL	HOSPITAL REGIONA	-	Enviada	Eventos Adversos Relaciona	Lesão por pressão		
12/12/2022	CE	SOBRAL	HOSPITAL REGIONA	-	Enviada	Eventos Adversos Relaciona	Falhas envolvendo sondas		
12/12/2022	CE	SOBRAL	HOSPITAL REGIONA	-	Enviada	Eventos Adversos Relaciona	Lesão por pressão		
12/12/2022	CE	SOBRAL	HOSPITAL REGIONA	-	Enviada	Eventos Adversos Relaciona	Falhas durante a assistênci		
13/12/2022	CE	SOBRAL	HOSPITAL REGIONA	-	Enviada	Eventos Adversos Relaciona	Falhas durante a assistênci		

13/12/2022	CE	SOBRAL	HOSPITAL REGIONA	-	Enviada	Eventos Adversos Relaciona	Falhas durante a assistênci		
13/12/2022	CE	SOBRAL	HOSPITAL REGIONA	-	Enviada	Eventos Adversos Relaciona	Falhas envolvendo sondas		
13/12/2022	CE	SOBRAL	HOSPITAL REGIONA	-	Enviada	Eventos Adversos Relaciona	Falhas envolvendo cateter		
13/12/2022	CE	SOBRAL	HOSPITAL REGIONA	-	Enviada	Eventos Adversos Relaciona	Lesão por pressão		
13/12/2022	CE	SOBRAL	HOSPITAL REGIONA	-	Enviada	Eventos Adversos Relaciona	Lesão por pressão		
13/12/2022	CE	SOBRAL	HOSPITAL REGIONA	-	Enviada	Eventos Adversos Relaciona	Falhas nas atividades		
28/12/2022	CE	SOBRAL	HOSPITAL REGIONA	dermatite	Enviada	Eventos Adversos Relaciona	Falhas durante a assistênci		
29/12/2022	CE	SOBRAL	HOSPITAL REGIONA	-	Enviada	Eventos Adversos Relaciona	Falhas envolvendo cateter		
29/12/2022	CE	SOBRAL	HOSPITAL REGIONA	DERMATITE	Enviada	Eventos Adversos Relaciona	Falhas durante a assistênci		
29/12/2022	CE	SOBRAL	HOSPITAL REGIONA	-	Enviada	Eventos Adversos Relaciona	Falhas envolvendo cateter		
29/12/2022	CE	SOBRAL	HOSPITAL REGIONA	-	Enviada	Eventos Adversos Relaciona	Falhas envolvendo cateter		
29/12/2022	CE	SOBRAL	HOSPITAL REGIONA	-	Enviada	Eventos Adversos Relaciona	Falhas durante a assistênci		
29/12/2022	CE	SOBRAL	HOSPITAL REGIONA	PACIENTE CHEGOU	Enviada	Eventos Adversos Relaciona	Falhas durante a assistênci		
29/12/2022	CE	SOBRAL	HOSPITAL REGIONA	-	Enviada	Eventos Adversos Relaciona	Queda do paciente		
29/12/2022	CE	SOBRAL	HOSPITAL REGIONA	-	Enviada	Eventos Adversos Relaciona	Falhas durante a assistênci		
29/12/2022	CE	SOBRAL	HOSPITAL REGIONA	-	Enviada	Eventos Adversos Relaciona	Falhas envolvendo cateter		
29/12/2022	CE	SOBRAL	HOSPITAL REGIONA	-	Enviada	Eventos Adversos Relaciona	Falhas durante a assistênci		
29/12/2022	CE	SOBRAL	HOSPITAL REGIONA	-	Enviada	Eventos Adversos Relaciona	Falha na identificação do		
29/12/2022	CE	SOBRAL	HOSPITAL REGIONA	-	Enviada	Eventos Adversos Relaciona	Queda do paciente		

30/12/2022	CE	SOBRAL	HOSPITA L REGIONA	-	Enviada	Eventos Adversos Relaciona	Falha na identificaç ão do		
30/12/2022	CE	SOBRAL	HOSPITA L REGIONA	-	Enviada	Eventos Adversos Relaciona	Falhas durante a assistênci		
30/12/2022	CE	SOBRAL	HOSPITA L REGIONA	-	Enviada	Eventos Adversos Relaciona	Falhas nas atividades		
30/12/2022	CE	SOBRAL	HOSPITA L REGIONA	-	Enviada	Eventos Adversos Relaciona	Falhas durante a assistênci		
30/12/2022	CE	SOBRAL	HOSPITA L REGIONA	-	Enviada	Eventos Adversos Relaciona	Falhas nas atividades		
30/12/2022	CE	SOBRAL	HOSPITA L REGIONA	-	Enviada	Eventos Adversos Relaciona	Falhas durante a assistênci		
30/12/2022	CE	SOBRAL	HOSPITA L REGIONA	-	Enviada	Eventos Adversos Relaciona	Falhas envolvend o cateter		
30/12/2022	CE	SOBRAL	HOSPITA L REGIONA	DERMATI TE	Enviada	Eventos Adversos Relaciona	Falhas durante a assistênci		
30/12/2022	CE	SOBRAL	HOSPITA L REGIONA	-	Enviada	Eventos Adversos Relaciona	Falhas nas atividades		
30/12/2022	CE	SOBRAL	HOSPITA L REGIONA	-	Enviada	Eventos Adversos Relaciona	Falhas na administra ção de		Enteral (oral, cateter
30/12/2022	CE	SOBRAL	HOSPITA L REGIONA	-	Enviada	Eventos Adversos Relaciona	Falhas durante a assistênci		
30/12/2022	CE	SOBRAL	HOSPITA L REGIONA	-	Enviada	Eventos Adversos Relaciona	Falhas nas atividades		
30/12/2022	CE	SOBRAL	HOSPITA L REGIONA	-	Enviada	Eventos Adversos Relaciona	Falhas envolvend o cateter		
30/12/2022	CE	SOBRAL	HOSPITA L REGIONA	-	Enviada	Eventos Adversos Relaciona	Falhas durante a assistênci		
30/12/2022	CE	SOBRAL	HOSPITA L REGIONA	-	Enviada	Eventos Adversos Relaciona	Falhas durante a assistênci		
30/12/2022	CE	SOBRAL	HOSPITA L REGIONA	-	Enviada	Eventos Adversos Relaciona	Falhas durante a assistênci		
30/12/2022	CE	SOBRAL	HOSPITA L REGIONA	-	Enviada	Eventos Adversos Relaciona	Queda do paciente		
30/12/2022	CE	SOBRAL	HOSPITA L REGIONA	-	Enviada	Eventos Adversos Relaciona	Falhas nas atividades		
30/12/2022	CE	SOBRAL	HOSPITA L REGIONA	-	Enviada	Eventos Adversos Relaciona	Queda do paciente		

30/12/2022	CE	SOBRAL	HOSPITA L REGIONA	-	Enviada	Eventos Adversos Relaciona	Falhas durante a assistênci		
30/12/2022	CE	SOBRAL	HOSPITA L REGIONA	-	Enviada	Eventos Adversos Relaciona	Falhas durante a assistênci		
30/12/2022	CE	SOBRAL	HOSPITA L REGIONA	-	Enviada	Eventos Adversos Relaciona	Falhas durante a assistênci		
30/12/2022	CE	SOBRAL	HOSPITA L REGIONA	-	Enviada	Eventos Adversos Relaciona	Falhas nas atividades		
30/12/2022	CE	SOBRAL	HOSPITA L REGIONA	-	Enviada	Eventos Adversos Relaciona	Falhas durante a assistênci		
30/12/2022	CE	SOBRAL	HOSPITA L REGIONA	-	Enviada	Eventos Adversos Relaciona	Queda do paciente		
30/12/2022	CE	SOBRAL	HOSPITA L REGIONA	-	Enviada	Eventos Adversos Relaciona	Falhas nas atividades		
30/12/2022	CE	SOBRAL	HOSPITA L REGIONA	-	Enviada	Eventos Adversos Relaciona	Falhas durante a assistênci		
02/01/2023	CE	SOBRAL	HOSPITA L REGIONA	-	Enviada	Eventos Adversos Relaciona	Falhas durante a assistênci		
02/01/2023	CE	SOBRAL	HOSPITA L REGIONA	-	Enviada	Eventos Adversos Relaciona	Falhas durante a assistênci		
02/01/2023	CE	SOBRAL	HOSPITA L REGIONA	-	Enviada	Eventos Adversos Relaciona	Falhas durante a assistênci		
02/01/2023	CE	SOBRAL	HOSPITA L REGIONA	-	Enviada	Eventos Adversos Relaciona	Falhas durante a assistênci		
02/01/2023	CE	SOBRAL	HOSPITA L REGIONA	-	Enviada	Eventos Adversos Relaciona	Extubaçã o endotraqu		
02/01/2023	CE	SOBRAL	HOSPITA L REGIONA	-	Enviada	Eventos Adversos Relaciona	Falhas envolvend o sondas		
02/01/2023	CE	SOBRAL	HOSPITA L REGIONA	-	Enviada	Eventos Adversos Relaciona	Falhas durante a assistênci		
02/01/2023	CE	SOBRAL	HOSPITA L REGIONA	-	Enviada	Eventos Adversos Relaciona	Falhas durante a assistênci		
02/01/2023	CE	SOBRAL	HOSPITA L REGIONA	-	Enviada	Eventos Adversos Relaciona	Falhas nas atividades		
03/01/2023	CE	SOBRAL	HOSPITA L REGIONA	-	Enviada	Eventos Adversos Relaciona	Lesão por pressão		
03/01/2023	CE	SOBRAL	HOSPITA L REGIONA	-	Enviada	Eventos Adversos Relaciona	Falhas durante a assistênci		

03/01/2023	CE	SOBRAL	HOSPITA L REGIONA	-	Enviada	Eventos Adversos Relaciona	Falha na document ação		
05/01/2023	CE	SOBRAL	HOSPITA L REGIONA	-	Enviada	Eventos Adversos Relaciona	Lesão por pressão		
09/01/2023	CE	SOBRAL	HOSPITA L REGIONA	-	Enviada	Eventos Adversos Relaciona	Queda do paciente		
09/01/2023	CE	SOBRAL	HOSPITA L REGIONA	-	Enviada	Eventos Adversos Relaciona	Lesão por pressão		
09/01/2023	CE	SOBRAL	HOSPITA L REGIONA	-	Enviada	Eventos Adversos Relaciona	Falhas durante a assistênci		
09/01/2023	CE	SOBRAL	HOSPITA L REGIONA	-	Enviada	Eventos Adversos Relaciona	Queda do paciente		
19/01/2023	CE	SOBRAL	HOSPITA L REGIONA	-	Enviada	Eventos Adversos Relaciona	Lesão por pressão		
26/01/2023	CE	SOBRAL	HOSPITA L REGIONA	-	Enviada	Eventos Adversos Relaciona	Falhas durante a assistênci		
26/01/2023	CE	SOBRAL	HOSPITA L REGIONA	-	Enviada	Eventos Adversos Relaciona	Lesão por pressão		
26/01/2023	CE	SOBRAL	HOSPITA L REGIONA	-	Enviada	Eventos Adversos Relaciona	Falhas durante a assistênci		
26/01/2023	CE	SOBRAL	HOSPITA L REGIONA	-	Enviada	Eventos Adversos Relaciona	Falhas nas atividades		
26/01/2023	CE	SOBRAL	HOSPITA L REGIONA	-	Enviada	Eventos Adversos Relaciona	Falhas envolvend o sondas		
26/01/2023	CE	SOBRAL	HOSPITA L REGIONA	-	Enviada	Eventos Adversos Relaciona	Extubaçã o endotraqu		
27/01/2023	CE	SOBRAL	HOSPITA L REGIONA	-	Enviada	Eventos Adversos Relaciona	Falhas durante a assistênci		
27/01/2023	CE	SOBRAL	HOSPITA L REGIONA	-	Enviada	Eventos Adversos Relaciona	Falhas durante a assistênci		
27/01/2023	CE	SOBRAL	HOSPITA L REGIONA	-	Enviada	Eventos Adversos Relaciona	Falhas durante a assistênci		
27/01/2023	CE	SOBRAL	HOSPITA L REGIONA	-	Enviada	Eventos Adversos Relaciona	Falha na document ação		
27/01/2023	CE	SOBRAL	HOSPITA L REGIONA	-	Enviada	Eventos Adversos Relaciona	Lesão por pressão		
27/01/2023	CE	SOBRAL	HOSPITA L REGIONA	-	Enviada	Eventos Adversos Relaciona	Lesão por pressão		

27/01/2023	CE	SOBRAL	HOSPITA L REGIONA	-	Enviada	Eventos Adversos Relaciona	Falhas durante a assistênci		
27/01/2023	CE	SOBRAL	HOSPITA L REGIONA	-	Enviada	Eventos Adversos Relaciona	Falhas durante a assistênci		
08/02/2023	CE	SOBRAL	HOSPITA L REGIONA	FALHA NA COMUNI	Enviada	Eventos Adversos Relaciona	Falhas nas atividades		
08/02/2023	CE	SOBRAL	HOSPITA L REGIONA	CURATIV O SEM IDENTIFI	Enviada	Eventos Adversos Relaciona	Falhas durante a assistênci		
08/02/2023	CE	SOBRAL	HOSPITA L REGIONA	-	Enviada	Eventos Adversos Relaciona	Lesão por pressão		
08/02/2023	CE	SOBRAL	HOSPITA L REGIONA	-	Enviada	Eventos Adversos Relaciona	Lesão por pressão		
08/02/2023	CE	SOBRAL	HOSPITA L REGIONA	-	Enviada	Eventos Adversos Relaciona	Falhas durante a assistênci		
08/02/2023	CE	SOBRAL	HOSPITA L REGIONA	-	Enviada	Eventos Adversos Relaciona	Falhas durante a assistênci		
08/02/2023	CE	SOBRAL	HOSPITA L REGIONA	-	Enviada	Eventos Adversos Relaciona	Falhas durante a assistênci		
08/02/2023	CE	SOBRAL	HOSPITA L REGIONA	-	Enviada	Eventos Adversos Relaciona	Tromboe mbolismo venoso		
09/02/2023	CE	SOBRAL	HOSPITA L REGIONA	-	Enviada	Eventos Adversos Relaciona	Falhas nas atividades		
09/02/2023	CE	SOBRAL	HOSPITA L REGIONA	-	Enviada	Eventos Adversos Relaciona	Lesão por pressão		
09/02/2023	CE	SOBRAL	HOSPITA L REGIONA	-	Enviada	Eventos Adversos Relaciona	Lesão por pressão		
09/02/2023	CE	SOBRAL	HOSPITA L REGIONA	-	Enviada	Eventos Adversos Relaciona	Lesão por pressão		
09/02/2023	CE	SOBRAL	HOSPITA L REGIONA	-	Enviada	Eventos Adversos Relaciona	Lesão por pressão		
09/02/2023	CE	SOBRAL	HOSPITA L REGIONA	-	Enviada	Eventos Adversos Relaciona	Lesão por pressão		
09/02/2023	CE	SOBRAL	HOSPITA L REGIONA	-	Enviada	Eventos Adversos Relaciona	Lesão por pressão		
09/02/2023	CE	SOBRAL	HOSPITA L REGIONA	-	Enviada	Eventos Adversos Relaciona	Lesão por pressão		
09/02/2023	CE	SOBRAL	HOSPITA L REGIONA	-	Enviada	Eventos Adversos Relaciona	Lesão por pressão		

09/02/2023	CE	SOBRAL	HOSPITA L REGIONA	-	Enviada	Eventos Adversos Relaciona	Falhas durante a assistênci		
09/02/2023	CE	SOBRAL	HOSPITA L REGIONA	-	Enviada	Eventos Adversos Relaciona	Lesão por pressão		
09/02/2023	CE	SOBRAL	HOSPITA L REGIONA	-	Enviada	Eventos Adversos Relaciona	Lesão por pressão		
09/02/2023	CE	SOBRAL	HOSPITA L REGIONA	-	Enviada	Eventos Adversos Relaciona	Lesão por pressão		
09/02/2023	CE	SOBRAL	HOSPITA L REGIONA	-	Enviada	Eventos Adversos Relaciona	Lesão por pressão		
09/02/2023	CE	SOBRAL	HOSPITA L REGIONA	-	Enviada	Eventos Adversos Relaciona	Lesão por pressão		
09/02/2023	CE	SOBRAL	HOSPITA L REGIONA	-	Enviada	Eventos Adversos Relaciona	Lesão por pressão		
09/02/2023	CE	SOBRAL	HOSPITA L REGIONA	-	Enviada	Eventos Adversos Relaciona	Lesão por pressão		
09/02/2023	CE	SOBRAL	HOSPITA L REGIONA	-	Enviada	Eventos Adversos Relaciona	Lesão por pressão		
09/02/2023	CE	SOBRAL	HOSPITA L REGIONA	-	Enviada	Eventos Adversos Relaciona	Lesão por pressão		
13/02/2023	CE	SOBRAL	HOSPITA L REGIONA	PACIENT E EM POS	Enviada	Eventos Adversos Relaciona	Falhas durante procedim		
13/02/2023	CE	SOBRAL	HOSPITA L REGIONA	PERDA DE AVP EM MSD,	Enviada	Eventos Adversos Relaciona	Falhas envolvend o cateter		
13/02/2023	CE	SOBRAL	HOSPITA L REGIONA	DERMATI TE	Enviada	Eventos Adversos Relaciona	Falhas durante a assistênci		
13/02/2023	CE	SOBRAL	HOSPITA L REGIONA	-	Enviada	Eventos Adversos Relaciona	Lesão por pressão		
13/02/2023	CE	SOBRAL	HOSPITA L REGIONA	APRESE NTANDO HIPEREM	Enviada	Eventos Adversos Relaciona	Falhas envolvend o cateter		
13/02/2023	CE	SOBRAL	HOSPITA L REGIONA	-	Enviada	Eventos Adversos Relaciona	Lesão por pressão		
13/02/2023	CE	SOBRAL	HOSPITA L REGIONA	A AUSENCI A DA	Enviada	Eventos Adversos Relaciona	Falhas durante a assistênci		
13/02/2023	CE	SOBRAL	HOSPITA L REGIONA	PACIENT E ESTAVA	Enviada	Eventos Adversos Relaciona	Queda do paciente		
13/02/2023	CE	SOBRAL	HOSPITA L REGIONA	-	Enviada	Eventos Adversos Relaciona	Lesão por pressão		

13/02/2023	CE	SOBRAL	HOSPITA L REGIONA	-	Enviada	Eventos Adversos Relaciona	Lesão por pressão		
13/02/2023	CE	SOBRAL	HOSPITA L REGIONA	-	Enviada	Eventos Adversos Relaciona	Extubaçã o endotraqu		
13/02/2023	CE	SOBRAL	HOSPITA L REGIONA	-	Enviada	Eventos Adversos Relaciona	Lesão por pressão		
13/02/2023	CE	SOBRAL	HOSPITA L REGIONA	PERDA DE SNG IDENTIFI	Enviada	Eventos Adversos Relaciona	Falhas envolvend o sondas		
13/02/2023	CE	SOBRAL	HOSPITA L REGIONA	-	Enviada	Eventos Adversos Relaciona	Lesão por pressão		
13/02/2023	CE	SOBRAL	HOSPITA L REGIONA	-	Enviada	Eventos Adversos Relaciona	Falhas envolvend o sondas		
13/02/2023	CE	SOBRAL	HOSPITA L REGIONA	-	Enviada	Eventos Adversos Relaciona	Extubaçã o endotraqu		
13/02/2023	CE	SOBRAL	HOSPITA L REGIONA	SEM COMUNI CAÇÃO	Enviada	Eventos Adversos Relaciona	Falhas nas atividades		
16/02/2023	CE	SOBRAL	HOSPITA L REGIONA	-	Enviada	Eventos Adversos Relaciona	Lesão por pressão		
16/02/2023	CE	SOBRAL	HOSPITA L REGIONA	-	Enviada	Eventos Adversos Relaciona	Lesão por pressão		
16/02/2023	CE	SOBRAL	HOSPITA L REGIONA	-	Enviada	Eventos Adversos Relaciona	Lesão por pressão		
16/02/2023	CE	SOBRAL	HOSPITA L REGIONA	-	Enviada	Eventos Adversos Relaciona	Lesão por pressão		
16/02/2023	CE	SOBRAL	HOSPITA L REGIONA	-	Enviada	Eventos Adversos Relaciona	Lesão por pressão		
16/02/2023	CE	SOBRAL	HOSPITA L REGIONA	-	Enviada	Eventos Adversos Relaciona	Lesão por pressão		
16/02/2023	CE	SOBRAL	HOSPITA L REGIONA	-	Enviada	Eventos Adversos Relaciona	Lesão por pressão		
16/02/2023	CE	SOBRAL	HOSPITA L REGIONA	-	Enviada	Eventos Adversos Relaciona	Lesão por pressão		
16/02/2023	CE	SOBRAL	HOSPITA L REGIONA	-	Enviada	Eventos Adversos Relaciona	Lesão por pressão		
16/02/2023	CE	SOBRAL	HOSPITA L REGIONA	DEVIDO A RECOME	Enviada	Eventos Adversos Relaciona	Lesão por pressão		
16/02/2023	CE	SOBRAL	HOSPITA L REGIONA	-	Enviada	Eventos Adversos Relaciona	Lesão por pressão		

16/02/2023	CE	SOBRAL	HOSPITA L REGIONA	PACIENT E COM LESÃO	Enviada	Eventos Adversos Relaciona	Lesão por pressão		
16/02/2023	CE	SOBRAL	HOSPITA L REGIONA	-	Enviada	Eventos Adversos Relaciona	Lesão por pressão		
16/02/2023	CE	SOBRAL	HOSPITA L REGIONA	-	Enviada	Eventos Adversos Relaciona	Lesão por pressão		
16/02/2023	CE	SOBRAL	HOSPITA L REGIONA	-	Enviada	Eventos Adversos Relaciona	Lesão por pressão		
16/02/2023	CE	SOBRAL	HOSPITA L REGIONA	-	Enviada	Eventos Adversos Relaciona	Lesão por pressão		
16/02/2023	CE	SOBRAL	HOSPITA L REGIONA	-	Enviada	Eventos Adversos Relaciona	Lesão por pressão		
16/02/2023	CE	SOBRAL	HOSPITA L REGIONA	-	Enviada	Eventos Adversos Relaciona	Lesão por pressão		
16/02/2023	CE	SOBRAL	HOSPITA L REGIONA	-	Enviada	Eventos Adversos Relaciona	Lesão por pressão		
16/02/2023	CE	SOBRAL	HOSPITA L REGIONA	-	Enviada	Eventos Adversos Relaciona	Lesão por pressão		
16/02/2023	CE	SOBRAL	HOSPITA L REGIONA	-	Enviada	Eventos Adversos Relaciona	Lesão por pressão		
16/02/2023	CE	SOBRAL	HOSPITA L REGIONA	-	Enviada	Eventos Adversos Relaciona	Lesão por pressão		
16/02/2023	CE	SOBRAL	HOSPITA L REGIONA	-	Enviada	Eventos Adversos Relaciona	Lesão por pressão		
16/02/2023	CE	SOBRAL	HOSPITA L REGIONA	-	Enviada	Eventos Adversos Relaciona	Lesão por pressão		
16/02/2023	CE	SOBRAL	HOSPITA L REGIONA	-	Enviada	Eventos Adversos Relaciona	Lesão por pressão		
16/02/2023	CE	SOBRAL	HOSPITA L REGIONA	-	Enviada	Eventos Adversos Relaciona	Lesão por pressão		
16/02/2023	CE	SOBRAL	HOSPITA L REGIONA	-	Enviada	Eventos Adversos Relaciona	Lesão por pressão		
16/02/2023	CE	SOBRAL	HOSPITA L REGIONA	-	Enviada	Eventos Adversos Relaciona	Falhas envolvend o cateter		
16/02/2023	CE	SOBRAL	HOSPITA L REGIONA	-	Enviada	Eventos Adversos Relaciona	Falhas envolvend o cateter		
16/02/2023	CE	SOBRAL	HOSPITA L REGIONA	-	Enviada	Eventos Adversos Relaciona	Falhas envolvend o cateter		

16/02/2023	CE	SOBRAL	HOSPITA L REGIONA	-	Enviada	Eventos Adversos Relaciona	Falhas envolvend o cateter		
16/02/2023	CE	SOBRAL	HOSPITA L REGIONA	-	Enviada	Eventos Adversos Relaciona	Falhas envolvend o cateter		
16/02/2023	CE	SOBRAL	HOSPITA L REGIONA	-	Enviada	Eventos Adversos Relaciona	Falhas envolvend o cateter		
16/02/2023	CE	SOBRAL	HOSPITA L REGIONA	-	Enviada	Eventos Adversos Relaciona	Falhas envolvend o cateter		
16/02/2023	CE	SOBRAL	HOSPITA L REGIONA	-	Enviada	Eventos Adversos Relaciona	Falhas envolvend o cateter		
16/02/2023	CE	SOBRAL	HOSPITA L REGIONA	-	Enviada	Eventos Adversos Relaciona	Falhas envolvend o cateter		
16/02/2023	CE	SOBRAL	HOSPITA L REGIONA	-	Enviada	Eventos Adversos Relaciona	Falhas envolvend o cateter		
16/02/2023	CE	SOBRAL	HOSPITA L REGIONA	-	Enviada	Eventos Adversos Relaciona	Falhas envolvend o cateter		
16/02/2023	CE	SOBRAL	HOSPITA L REGIONA	-	Enviada	Eventos Adversos Relaciona	Falhas envolvend o cateter		
16/02/2023	CE	SOBRAL	HOSPITA L REGIONA	DERMATI TE	Enviada	Eventos Adversos Relaciona	Falhas durante a assistênci		
16/02/2023	CE	SOBRAL	HOSPITA L REGIONA	-	Enviada	Eventos Adversos Relaciona	Falhas durante a assistênci		
16/02/2023	CE	SOBRAL	HOSPITA L REGIONA	-	Enviada	Eventos Adversos Relaciona	Falhas durante a assistênci		
16/02/2023	CE	SOBRAL	HOSPITA L REGIONA	-	Enviada	Eventos Adversos Relaciona	Falhas durante a assistênci		
16/02/2023	CE	SOBRAL	HOSPITA L REGIONA	-	Enviada	Eventos Adversos Relaciona	Falhas durante a assistênci		
16/02/2023	CE	SOBRAL	HOSPITA L REGIONA	-	Enviada	Eventos Adversos Relaciona	Falhas durante a assistênci		
16/02/2023	CE	SOBRAL	HOSPITA L REGIONA	-	Enviada	Eventos Adversos Relaciona	Falhas durante a assistênci		
16/02/2023	CE	SOBRAL	HOSPITA L REGIONA	-	Enviada	Eventos Adversos Relaciona	Falhas durante a assistênci		
16/02/2023	CE	SOBRAL	HOSPITA L REGIONA	-	Enviada	Eventos Adversos Relaciona	Falhas durante a assistênci		
16/02/2023	CE	SOBRAL	HOSPITA L REGIONA	-	Enviada	Eventos Adversos Relaciona	Falhas durante a assistênci		

16/02/2023	CE	SOBRAL	HOSPITA L REGIONA	-	Enviada	Eventos Adversos Relaciona	Falhas durante a assistênci		
16/02/2023	CE	SOBRAL	HOSPITA L REGIONA	-	Enviada	Eventos Adversos Relaciona	Falhas durante a assistênci		
16/02/2023	CE	SOBRAL	HOSPITA L REGIONA	-	Enviada	Eventos Adversos Relaciona	Falhas durante a assistênci		
16/02/2023	CE	SOBRAL	HOSPITA L REGIONA	-	Enviada	Eventos Adversos Relaciona	Falhas durante a assistênci		
16/02/2023	CE	SOBRAL	HOSPITA L REGIONA	-	Enviada	Eventos Adversos Relaciona	Falhas durante a assistênci		
16/02/2023	CE	SOBRAL	HOSPITA L REGIONA	-	Enviada	Eventos Adversos Relaciona	Falhas durante a assistênci		
16/02/2023	CE	SOBRAL	HOSPITA L REGIONA	-	Enviada	Eventos Adversos Relaciona	Falhas durante a assistênci		
17/02/2023	CE	SOBRAL	HOSPITA L REGIONA	-	Enviada	Eventos Adversos Relaciona	Falhas durante a assistênci		
17/02/2023	CE	SOBRAL	HOSPITA L REGIONA	-	Enviada	Eventos Adversos Relaciona	Falhas durante a assistênci		
17/02/2023	CE	SOBRAL	HOSPITA L REGIONA	-	Enviada	Eventos Adversos Relaciona	Falhas durante a assistênci		
17/02/2023	CE	SOBRAL	HOSPITA L REGIONA	-	Enviada	Eventos Adversos Relaciona	Falhas durante a assistênci		
17/02/2023	CE	SOBRAL	HOSPITA L REGIONA	-	Enviada	Eventos Adversos Relaciona	Falhas durante a assistênci		
17/02/2023	CE	SOBRAL	HOSPITA L REGIONA	-	Enviada	Eventos Adversos Relaciona	Falhas durante a assistênci		
17/02/2023	CE	SOBRAL	HOSPITA L REGIONA	-	Enviada	Eventos Adversos Relaciona	Falhas durante a assistênci		
17/02/2023	CE	SOBRAL	HOSPITA L REGIONA	-	Enviada	Eventos Adversos Relaciona	Falhas envolvend o cateter		
17/02/2023	CE	SOBRAL	HOSPITA L REGIONA	-	Enviada	Eventos Adversos Relaciona	Falhas envolvend o cateter		
17/02/2023	CE	SOBRAL	HOSPITA L REGIONA	-	Enviada	Eventos Adversos Relaciona	Falhas envolvend o cateter		
17/02/2023	CE	SOBRAL	HOSPITA L REGIONA	-	Enviada	Eventos Adversos Relaciona	Falhas envolvend o cateter		
17/02/2023	CE	SOBRAL	HOSPITA L REGIONA	-	Enviada	Eventos Adversos Relaciona	Falhas envolvend o cateter		

17/02/2023	CE	SOBRAL	HOSPITA L REGIONA	-	Enviada	Eventos Adversos Relaciona	Falhas envolvend o cateter		
17/02/2023	CE	SOBRAL	HOSPITA L REGIONA	-	Enviada	Eventos Adversos Relaciona	Falhas envolvend o cateter		
17/02/2023	CE	SOBRAL	HOSPITA L REGIONA	-	Enviada	Eventos Adversos Relaciona	Falhas envolvend o cateter		
17/02/2023	CE	SOBRAL	HOSPITA L REGIONA	-	Enviada	Eventos Adversos Relaciona	Falhas envolvend o cateter		
17/02/2023	CE	SOBRAL	HOSPITA L REGIONA	-	Enviada	Eventos Adversos Relaciona	Falhas envolvend o cateter		
17/02/2023	CE	SOBRAL	HOSPITA L REGIONA	-	Enviada	Eventos Adversos Relaciona	Falhas durante a assistênci		
17/02/2023	CE	SOBRAL	HOSPITA L REGIONA	-	Enviada	Eventos Adversos Relaciona	Falhas durante a assistênci		
17/02/2023	CE	SOBRAL	HOSPITA L REGIONA	-	Enviada	Eventos Adversos Relaciona	Falhas durante a assistênci		
17/02/2023	CE	SOBRAL	HOSPITA L REGIONA	-	Enviada	Eventos Adversos Relaciona	Falhas durante a assistênci		
17/02/2023	CE	SOBRAL	HOSPITA L REGIONA	-	Enviada	Eventos Adversos Relaciona	Lesão por pressão		
17/02/2023	CE	SOBRAL	HOSPITA L REGIONA	-	Enviada	Eventos Adversos Relaciona	Lesão por pressão		
17/02/2023	CE	SOBRAL	HOSPITA L REGIONA	-	Enviada	Eventos Adversos Relaciona	Lesão por pressão		
17/02/2023	CE	SOBRAL	HOSPITA L REGIONA	-	Enviada	Eventos Adversos Relaciona	Lesão por pressão		
17/02/2023	CE	SOBRAL	HOSPITA L REGIONA	-	Enviada	Eventos Adversos Relaciona	Lesão por pressão		
17/02/2023	CE	SOBRAL	HOSPITA L REGIONA	-	Enviada	Eventos Adversos Relaciona	Lesão por pressão		
17/02/2023	CE	SOBRAL	HOSPITA L REGIONA	-	Enviada	Eventos Adversos Relaciona	Lesão por pressão		
17/02/2023	CE	SOBRAL	HOSPITA L REGIONA	-	Enviada	Eventos Adversos Relaciona	Lesão por pressão		
17/02/2023	CE	SOBRAL	HOSPITA L REGIONA	-	Enviada	Eventos Adversos Relaciona	Lesão por pressão		
17/02/2023	CE	SOBRAL	HOSPITA L REGIONA	NOTIFIC O INTENSO	Enviada	Eventos Adversos Relaciona	Falhas durante procedim		

17/02/2023	CE	SOBRAL	HOSPITA L REGIONA	APÓS EXPULSÃ O DO	Enviada	Eventos Adversos Relaciona	Falhas durante procedim		
17/02/2023	CE	SOBRAL	HOSPITA L REGIONA	-	Enviada	Eventos Adversos Relaciona	Falhas durante procedim		
17/02/2023	CE	SOBRAL	HOSPITA L REGIONA	-	Enviada	Eventos Adversos Relaciona	Falhas durante procedim		
17/02/2023	CE	SOBRAL	HOSPITA L REGIONA	-	Enviada	Eventos Adversos Relaciona	Falhas durante procedim		
17/02/2023	CE	SOBRAL	HOSPITA L REGIONA	-	Enviada	Eventos Adversos Relaciona	Falhas durante procedim		
17/02/2023	CE	SOBRAL	HOSPITA L REGIONA	-	Enviada	Eventos Adversos Relaciona	Lesão por pressão		
17/02/2023	CE	SOBRAL	HOSPITA L REGIONA	-	Enviada	Eventos Adversos Relaciona	Falhas durante procedim		
17/02/2023	CE	SOBRAL	HOSPITA L REGIONA	-	Enviada	Eventos Adversos Relaciona	Falhas durante procedim		
17/02/2023	CE	SOBRAL	HOSPITA L REGIONA	-	Enviada	Eventos Adversos Relaciona	Falhas durante procedim		
17/02/2023	CE	SOBRAL	HOSPITA L REGIONA	-	Enviada	Eventos Adversos Relaciona	Falhas durante procedim		
17/02/2023	CE	SOBRAL	HOSPITA L REGIONA	-	Enviada	Eventos Adversos Relaciona	Falhas durante procedim		
28/02/2023	CE	SOBRAL	HOSPITA L REGIONA	-	Enviada	Eventos Adversos Relaciona	Falhas envolvend o sondas		
28/02/2023	CE	SOBRAL	HOSPITA L REGIONA	Paciente com distúrbio	Enviada	Eventos Adversos Relaciona	Falhas envolvend o sondas		
28/02/2023	CE	SOBRAL	HOSPITA L REGIONA	CRIANÇA RETIROU	Enviada	Eventos Adversos Relaciona	Falhas envolvend o sondas		
28/02/2023	CE	SOBRAL	HOSPITA L REGIONA	-	Enviada	Eventos Adversos Relaciona	Falhas envolvend o sondas		
28/02/2023	CE	SOBRAL	HOSPITA L REGIONA	-	Enviada	Eventos Adversos Relaciona	Falhas envolvend o sondas		
28/02/2023	CE	SOBRAL	HOSPITA L REGIONA	-	Enviada	Eventos Adversos Relaciona	Falhas envolvend o sondas		
28/02/2023	CE	SOBRAL	HOSPITA L REGIONA	-	Enviada	Eventos Adversos Relaciona	Falhas envolvend o sondas		
28/02/2023	CE	SOBRAL	HOSPITA L REGIONA	-	Enviada	Eventos Adversos Relaciona	Falhas envolvend o sondas		

28/02/2023	CE	SOBRAL	HOSPITA L REGIONA	-	Enviada	Eventos Adversos Relaciona	Falhas envolvend o sondas		
28/02/2023	CE	SOBRAL	HOSPITA L REGIONA	-	Enviada	Eventos Adversos Relaciona	Falhas envolvend o sondas		
28/02/2023	CE	SOBRAL	HOSPITA L REGIONA	-	Enviada	Eventos Adversos Relaciona	Falhas envolvend o sondas		
28/02/2023	CE	SOBRAL	HOSPITA L REGIONA	-	Enviada	Eventos Adversos Relaciona	Falhas envolvend o sondas		
28/02/2023	CE	SOBRAL	HOSPITA L REGIONA	-	Enviada	Eventos Adversos Relaciona	Falhas envolvend o sondas		
02/03/2023	CE	SOBRAL	HOSPITA L REGIONA	PACIENT E PARIU VIA	Enviada	Eventos Adversos Relaciona	Falhas durante procedim		
02/03/2023	CE	SOBRAL	HOSPITA L REGIONA	PACIENT E	Enviada	Eventos Adversos Relaciona	Falhas durante procedim		
02/03/2023	CE	SOBRAL	HOSPITA L REGIONA	PACIENT E APRESE	Enviada	Eventos Adversos Relaciona	Falhas durante procedim		
02/03/2023	CE	SOBRAL	HOSPITA L REGIONA	-	Enviada	Eventos Adversos Relaciona	Falhas envolvend o sondas		
02/03/2023	CE	SOBRAL	HOSPITA L REGIONA	-	Enviada	Eventos Adversos Relaciona	Falhas envolvend o sondas		
02/03/2023	CE	SOBRAL	HOSPITA L REGIONA	-	Enviada	Eventos Adversos Relaciona	Lesão por pressão		
02/03/2023	CE	SOBRAL	HOSPITA L REGIONA	-	Enviada	Eventos Adversos Relaciona	Falhas nas atividades		
02/03/2023	CE	SOBRAL	HOSPITA L REGIONA	RN ENCAMIN HADO	Enviada	Eventos Adversos Relaciona	Falhas durante a assistênci		
03/03/2023	CE	SOBRAL	HOSPITA L REGIONA	-	Enviada	Eventos Adversos Relaciona	Tromboe mbolismo venoso		
03/03/2023	CE	SOBRAL	HOSPITA L REGIONA	-	Enviada	Eventos Adversos Relaciona	Tromboe mbolismo venoso		
03/03/2023	CE	SOBRAL	HOSPITA L REGIONA	-	Enviada	Eventos Adversos Relaciona	Tromboe mbolismo venoso		
03/03/2023	CE	SOBRAL	HOSPITA L REGIONA	-	Enviada	Eventos Adversos Relaciona	Tromboe mbolismo venoso		
03/03/2023	CE	SOBRAL	HOSPITA L REGIONA	-	Enviada	Eventos Adversos Relaciona	Falha na document ação		
03/03/2023	CE	SOBRAL	HOSPITA L REGIONA	-	Enviada	Eventos Adversos Relaciona	Falha na document ação		

03/03/2023	CE	SOBRAL	HOSPITAL REGIONA	-	Enviada	Eventos Adversos Relaciona	Falha na documentação		
03/03/2023	CE	SOBRAL	HOSPITAL REGIONA	-	Enviada	Eventos Adversos Relaciona	Falha na documentação		
03/03/2023	CE	SOBRAL	HOSPITAL REGIONA	-	Enviada	Eventos Adversos Relaciona	Falha na documentação		
03/03/2023	CE	SOBRAL	HOSPITAL REGIONA	-	Enviada	Eventos Adversos Relaciona	Falhas durante a assistênci		
03/03/2023	CE	SOBRAL	HOSPITAL REGIONA	-	Enviada	Eventos Adversos Relaciona	Falhas na administração de		Enteral (oral, cateter
03/03/2023	CE	SOBRAL	HOSPITAL REGIONA	-	Enviada	Eventos Adversos Relaciona	Falhas envolvendo cateter		
03/03/2023	CE	SOBRAL	HOSPITAL REGIONA	-	Enviada	Eventos Adversos Relaciona	Falhas envolvendo cateter		
03/03/2023	CE	SOBRAL	HOSPITAL REGIONA	-	Enviada	Eventos Adversos Relaciona	Falhas envolvendo cateter		
03/03/2023	CE	SOBRAL	HOSPITAL REGIONA	-	Enviada	Eventos Adversos Relaciona	Falhas envolvendo cateter		
03/03/2023	CE	SOBRAL	HOSPITAL REGIONA	-	Enviada	Eventos Adversos Relaciona	Falhas envolvendo cateter		
03/03/2023	CE	SOBRAL	HOSPITAL REGIONA	-	Enviada	Eventos Adversos Relaciona	Falha na documentação		
03/03/2023	CE	SOBRAL	HOSPITAL REGIONA	-	Enviada	Eventos Adversos Relaciona	Falha na documentação		
03/03/2023	CE	SOBRAL	HOSPITAL REGIONA	-	Enviada	Eventos Adversos Relaciona	Falha na documentação		
04/03/2023	CE	SOBRAL	HOSPITAL REGIONA	-	Enviada	Eventos Adversos Relaciona	Falhas durante a assistênci		
04/03/2023	CE	SOBRAL	HOSPITAL REGIONA	-	Enviada	Eventos Adversos Relaciona	Broncoaspiração		
04/03/2023	CE	SOBRAL	HOSPITAL REGIONA	-	Enviada	Eventos Adversos Relaciona	Falhas durante a assistênci		
04/03/2023	CE	SOBRAL	HOSPITAL REGIONA	-	Enviada	Eventos Adversos Relaciona	Queda do paciente		
04/03/2023	CE	SOBRAL	HOSPITAL REGIONA	-	Enviada	Eventos Adversos Relaciona	Lesão por pressão		
04/03/2023	CE	SOBRAL	HOSPITAL REGIONA	-	Enviada	Eventos Adversos Relaciona	Falhas ocorridas em		

06/03/2023	CE	SOBRAL	HOSPITA L REGIONA	PACIENT E VEIO POR VIA	Enviada	Eventos Adversos Relaciona	Falhas na assistênci a		
06/03/2023	CE	SOBRAL	HOSPITA L REGIONA	NÃO FORAM ENTREG	Enviada	Eventos Adversos Relaciona	Falhas na administra ção de		Enteral (oral, cateter
07/03/2023	CE	SOBRAL	HOSPITA L REGIONA	-	Enviada	Eventos Adversos Relaciona	Falhas durante a assistênci		
07/03/2023	CE	SOBRAL	HOSPITA L REGIONA	-	Enviada	Eventos Adversos Relaciona	Falha na document ação		
07/03/2023	CE	SOBRAL	HOSPITA L REGIONA	-	Enviada	Eventos Adversos Relaciona	Falhas durante a assistênci		
07/03/2023	CE	SOBRAL	HOSPITA L REGIONA	ADMITID A PACIENT	Enviada	Eventos Adversos Relaciona	Falhas nas atividades		
07/03/2023	CE	SOBRAL	HOSPITA L REGIONA	-	Enviada	Eventos Adversos Relaciona	Falhas durante a assistênci		
07/03/2023	CE	SOBRAL	HOSPITA L REGIONA	-	Enviada	Eventos Adversos Relaciona	Falhas na administra ção de		Enteral (oral, cateter
07/03/2023	CE	SOBRAL	HOSPITA L REGIONA	-	Enviada	Eventos Adversos Relaciona	Falhas na administra ção de		Enteral (oral, cateter
07/03/2023	CE	SOBRAL	HOSPITA L REGIONA	-	Enviada	Eventos Adversos Relaciona	Falhas na administra ção de		Enteral (oral, cateter
07/03/2023	CE	SOBRAL	HOSPITA L REGIONA	-	Enviada	Eventos Adversos Relaciona	Falhas na administra ção de		Enteral (oral, cateter
07/03/2023	CE	SOBRAL	HOSPITA L REGIONA	-	Enviada	Eventos Adversos Relaciona	Falhas ocorridas em		
07/03/2023	CE	SOBRAL	HOSPITA L REGIONA	-	Enviada	Eventos Adversos Relaciona	Falhas durante a assistênci		
07/03/2023	CE	SOBRAL	HOSPITA L REGIONA	-	Enviada	Eventos Adversos Relaciona	Falhas na administra ção de		Enteral (oral, cateter
07/03/2023	CE	SOBRAL	HOSPITA L REGIONA	CRIANÇA DEITADA	Enviada	Eventos Adversos Relaciona	Queda do paciente		
07/03/2023	CE	SOBRAL	HOSPITA L REGIONA	-	Enviada	Eventos Adversos Relaciona	Falhas na assistênci a		
07/03/2023	CE	SOBRAL	HOSPITA L REGIONA	-	Enviada	Eventos Adversos Relaciona	Queda do paciente		
07/03/2023	CE	SOBRAL	HOSPITA L REGIONA	-	Enviada	Eventos Adversos Relaciona	Falhas ocorridas em		
07/03/2023	CE	SOBRAL	HOSPITA L REGIONA	ATRASO NO APRAZA	Enviada	Eventos Adversos Relaciona	Falhas durante a assistênci		

07/03/2023	CE	SOBRAL	HOSPITA L REGIONA	-	Enviada	Eventos Adversos Relaciona	Falhas nas atividades		
07/03/2023	CE	SOBRAL	HOSPITA L REGIONA	-	Enviada	Eventos Adversos Relaciona	Falhas durante a assistênci		
07/03/2023	CE	SOBRAL	HOSPITA L REGIONA	-	Enviada	Eventos Adversos Relaciona	Queda do paciente		
07/03/2023	CE	SOBRAL	HOSPITA L REGIONA	-	Enviada	Eventos Adversos Relaciona	Falhas envolvend o cateter		
07/03/2023	CE	SOBRAL	HOSPITA L REGIONA	-	Enviada	Eventos Adversos Relaciona	Falhas envolvend o cateter		
07/03/2023	CE	SOBRAL	HOSPITA L REGIONA	-	Enviada	Eventos Adversos Relaciona	Falhas envolvend o cateter		
08/03/2023	CE	SOBRAL	HOSPITA L REGIONA	-	Enviada	Eventos Adversos Relaciona	Falhas nas atividades		
08/03/2023	CE	SOBRAL	HOSPITA L REGIONA	-	Enviada	Eventos Adversos Relaciona	Falhas nas atividades		
08/03/2023	CE	SOBRAL	HOSPITA L REGIONA	-	Enviada	Eventos Adversos Relaciona	Falhas nas atividades		
08/03/2023	CE	SOBRAL	HOSPITA L REGIONA	-	Enviada	Eventos Adversos Relaciona	Falhas nas atividades		
08/03/2023	CE	SOBRAL	HOSPITA L REGIONA	-	Enviada	Eventos Adversos Relaciona	Falhas nas atividades		
08/03/2023	CE	SOBRAL	HOSPITA L REGIONA	-	Enviada	Eventos Adversos Relaciona	Falhas durante a assistênci		
08/03/2023	CE	SOBRAL	HOSPITA L REGIONA	-	Enviada	Eventos Adversos Relaciona	Falhas durante a assistênci		
08/03/2023	CE	SOBRAL	HOSPITA L REGIONA	-	Enviada	Eventos Adversos Relaciona	Queda do paciente		
08/03/2023	CE	SOBRAL	HOSPITA L REGIONA	-	Em análise	Eventos Adversos Relaciona	Queda do paciente		
08/03/2023	CE	SOBRAL	HOSPITA L REGIONA	-	Em análise	Eventos Adversos Relaciona	Queda do paciente		
08/03/2023	CE	SOBRAL	HOSPITA L REGIONA	-	Enviada	Eventos Adversos Relaciona	Queda do paciente		
08/03/2023	CE	SOBRAL	HOSPITA L REGIONA	-	Em análise	Eventos Adversos Relaciona	Queda do paciente		
08/03/2023	CE	SOBRAL	HOSPITA L REGIONA	-	Em análise	Eventos Adversos Relaciona	Queda do paciente		

08/03/2023	CE	SOBRAL	HOSPITA L REGIONA	-	Em análise	Eventos Adversos Relaciona	Queda do paciente		
09/03/2023	CE	SOBRAL	HOSPITA L REGIONA	-	Enviada	Eventos Adversos Relaciona	Falhas durante a assistênci		
09/03/2023	CE	SOBRAL	HOSPITA L REGIONA	-	Enviada	Eventos Adversos Relaciona	Falhas durante a assistênci		
09/03/2023	CE	SOBRAL	HOSPITA L REGIONA	-	Enviada	Eventos Adversos Relaciona	Falhas durante a assistênci		
09/03/2023	CE	SOBRAL	HOSPITA L REGIONA	-	Enviada	Eventos Adversos Relaciona	Falhas durante a assistênci		
09/03/2023	CE	SOBRAL	HOSPITA L REGIONA	-	Enviada	Eventos Adversos Relaciona	Falhas durante a assistênci		
09/03/2023	CE	SOBRAL	HOSPITA L REGIONA	-	Enviada	Eventos Adversos Relaciona	Falhas durante a assistênci		
09/03/2023	CE	SOBRAL	HOSPITA L REGIONA	-	Enviada	Eventos Adversos Relaciona	Falhas durante a assistênci		
09/03/2023	CE	SOBRAL	HOSPITA L REGIONA	-	Enviada	Eventos Adversos Relaciona	Falhas durante a assistênci		
09/03/2023	CE	SOBRAL	HOSPITA L REGIONA	-	Enviada	Eventos Adversos Relaciona	Falhas durante a assistênci		
09/03/2023	CE	SOBRAL	HOSPITA L REGIONA	-	Enviada	Eventos Adversos Relaciona	Falhas durante a assistênci		
09/03/2023	CE	SOBRAL	HOSPITA L REGIONA	-	Enviada	Eventos Adversos Relaciona	Falhas durante a assistênci		
09/03/2023	CE	SOBRAL	HOSPITA L REGIONA	-	Enviada	Eventos Adversos Relaciona	Falhas ocorridas em		
09/03/2023	CE	SOBRAL	HOSPITA L REGIONA	-	Enviada	Eventos Adversos Relaciona	Falhas ocorridas em		
09/03/2023	CE	SOBRAL	HOSPITA L REGIONA	-	Enviada	Eventos Adversos Relaciona	Broncoas piração		
09/03/2023	CE	SOBRAL	HOSPITA L REGIONA	-	Enviada	Eventos Adversos Relaciona	Broncoas piração		
09/03/2023	CE	SOBRAL	HOSPITA L REGIONA	-	Concluída	Eventos Adversos Relaciona	Lesão por pressão		
09/03/2023	CE	SOBRAL	HOSPITA L REGIONA	-	Enviada	Eventos Adversos Relaciona	Lesão por pressão		
09/03/2023	CE	SOBRAL	HOSPITA L REGIONA	-	Enviada	Eventos Adversos Relaciona	Lesão por pressão		

09/03/2023	CE	SOBRAL	HOSPITA L REGIONA	-	Enviada	Eventos Adversos Relaciona	Lesão por pressão		
09/03/2023	CE	SOBRAL	HOSPITA L REGIONA	-	Enviada	Eventos Adversos Relaciona	Falhas durante a assistênci		
09/03/2023	CE	SOBRAL	HOSPITA L REGIONA	-	Enviada	Eventos Adversos Relaciona	Falhas durante a assistênci		
09/03/2023	CE	SOBRAL	HOSPITA L REGIONA	-	Enviada	Eventos Adversos Relaciona	Falhas durante a assistênci		
09/03/2023	CE	SOBRAL	HOSPITA L REGIONA	-	Enviada	Eventos Adversos Relaciona	Falhas durante a assistênci		
09/03/2023	CE	SOBRAL	HOSPITA L REGIONA	-	Enviada	Eventos Adversos Relaciona	Falhas durante a assistênci		
09/03/2023	CE	SOBRAL	HOSPITA L REGIONA	-	Enviada	Eventos Adversos Relaciona	Falhas durante a assistênci		
09/03/2023	CE	SOBRAL	HOSPITA L REGIONA	-	Enviada	Eventos Adversos Relaciona	Falhas durante a assistênci		
09/03/2023	CE	SOBRAL	HOSPITA L REGIONA	-	Enviada	Eventos Adversos Relaciona	Falhas durante a assistênci		
09/03/2023	CE	SOBRAL	HOSPITA L REGIONA	-	Enviada	Eventos Adversos Relaciona	Falhas durante a assistênci		
09/03/2023	CE	SOBRAL	HOSPITA L REGIONA	-	Enviada	Eventos Adversos Relaciona	Falhas durante a assistênci		
09/03/2023	CE	SOBRAL	HOSPITA L REGIONA	-	Enviada	Eventos Adversos Relaciona	Falhas durante a assistênci		
09/03/2023	CE	SOBRAL	HOSPITA L REGIONA	-	Enviada	Eventos Adversos Relaciona	Falhas durante a assistênci		
09/03/2023	CE	SOBRAL	HOSPITA L REGIONA	-	Enviada	Eventos Adversos Relaciona	Falhas durante a assistênci		
09/03/2023	CE	SOBRAL	HOSPITA L REGIONA	-	Enviada	Eventos Adversos Relaciona	Falhas durante a assistênci		
09/03/2023	CE	SOBRAL	HOSPITA L REGIONA	-	Enviada	Eventos Adversos Relaciona	Falhas durante a assistênci		
09/03/2023	CE	SOBRAL	HOSPITA L REGIONA	-	Enviada	Eventos Adversos Relaciona	Falhas durante a assistênci		
09/03/2023	CE	SOBRAL	HOSPITA L REGIONA	-	Enviada	Eventos Adversos Relaciona	Falhas durante a assistênci		
09/03/2023	CE	SOBRAL	HOSPITA L REGIONA	-	Enviada	Eventos Adversos Relaciona	Falhas durante a assistênci		

09/03/2023	CE	SOBRAL	HOSPITA L REGIONA	-	Enviada	Eventos Adversos Relaciona	Falhas durante a assistênci		
09/03/2023	CE	SOBRAL	HOSPITA L REGIONA	-	Enviada	Eventos Adversos Relaciona	Falhas durante a assistênci		
09/03/2023	CE	SOBRAL	HOSPITA L REGIONA	-	Enviada	Eventos Adversos Relaciona	Falhas durante a assistênci		
10/03/2023	CE	SOBRAL	HOSPITA L REGIONA	-	Enviada	Eventos Adversos Relaciona	Falhas durante a assistênci		
10/03/2023	CE	SOBRAL	HOSPITA L REGIONA	-	Enviada	Eventos Adversos Relaciona	Falhas envolvend o cateter		
10/03/2023	CE	SOBRAL	HOSPITA L REGIONA	-	Enviada	Eventos Adversos Relaciona	Falhas envolvend o sondas		
10/03/2023	CE	SOBRAL	HOSPITA L REGIONA	-	Enviada	Eventos Adversos Relaciona	Falhas envolvend o sondas		
10/03/2023	CE	SOBRAL	HOSPITA L REGIONA	-	Enviada	Eventos Adversos Relaciona	Falhas envolvend o sondas		
10/03/2023	CE	SOBRAL	HOSPITA L REGIONA	-	Enviada	Eventos Adversos Relaciona	Falhas envolvend o sondas		
10/03/2023	CE	SOBRAL	HOSPITA L REGIONA	-	Enviada	Eventos Adversos Relaciona	Falhas envolvend o cateter		
10/03/2023	CE	SOBRAL	HOSPITA L REGIONA	DERMATI TE	Enviada	Eventos Adversos Relaciona	Falhas durante a assistênci		
10/03/2023	CE	SOBRAL	HOSPITA L REGIONA	DERMATI TE	Enviada	Eventos Adversos Relaciona	Falhas durante a assistênci		
10/03/2023	CE	SOBRAL	HOSPITA L REGIONA	-	Enviada	Eventos Adversos Relaciona	Falhas envolvend o sondas		
10/03/2023	CE	SOBRAL	HOSPITA L REGIONA	-	Enviada	Eventos Adversos Relaciona	Falha na identificaç ão do		
10/03/2023	CE	SOBRAL	HOSPITA L REGIONA	EXAME DE VDRL SOLICITA	Enviada	Eventos Adversos Relaciona	Falhas ocorridas em		
10/03/2023	CE	SOBRAL	HOSPITA L REGIONA	-	Enviada	Eventos Adversos Relaciona	Falhas envolvend o sondas		
10/03/2023	CE	SOBRAL	HOSPITA L REGIONA	-	Enviada	Eventos Adversos Relaciona	Falhas durante a assistênci		
10/03/2023	CE	SOBRAL	HOSPITA L REGIONA	-	Enviada	Eventos Adversos Relaciona	Falhas na administra ção de		Parenteral (via intravenos
10/03/2023	CE	SOBRAL	HOSPITA L REGIONA	-	Enviada	Eventos Adversos Relaciona	Falhas envolvend o sondas		

10/03/2023	CE	SOBRAL	HOSPITA L REGIONA	-	Enviada	Eventos Adversos Relaciona	Falhas envolvend o sondas		
10/03/2023	CE	SOBRAL	HOSPITA L REGIONA	-	Enviada	Eventos Adversos Relaciona	Falhas durante a assistênci		
10/03/2023	CE	SOBRAL	HOSPITA L REGIONA	-	Enviada	Eventos Adversos Relaciona	Falhas envolvend o sondas		
10/03/2023	CE	SOBRAL	HOSPITA L REGIONA	-	Enviada	Eventos Adversos Relaciona	Falhas envolvend o cateter		
10/03/2023	CE	SOBRAL	HOSPITA L REGIONA	-	Enviada	Eventos Adversos Relaciona	Falha na document ação		
10/03/2023	CE	SOBRAL	HOSPITA L REGIONA	-	Enviada	Eventos Adversos Relaciona	Falhas ocorridas em		
10/03/2023	CE	SOBRAL	HOSPITA L REGIONA	-	Enviada	Eventos Adversos Relaciona	Falhas envolvend o sondas		
10/03/2023	CE	SOBRAL	HOSPITA L REGIONA	-	Enviada	Eventos Adversos Relaciona	Falhas na assistênci a		
10/03/2023	CE	SOBRAL	HOSPITA L REGIONA	-	Enviada	Eventos Adversos Relaciona	Lesão por pressão		
10/03/2023	CE	SOBRAL	HOSPITA L REGIONA	-	Enviada	Eventos Adversos Relaciona	Falhas envolvend o cateter		
10/03/2023	CE	SOBRAL	HOSPITA L REGIONA	-	Enviada	Eventos Adversos Relaciona	Falhas envolvend o cateter		
10/03/2023	CE	SOBRAL	HOSPITA L REGIONA	-	Enviada	Eventos Adversos Relaciona	Falhas envolvend o cateter		
10/03/2023	CE	SOBRAL	HOSPITA L REGIONA	-	Enviada	Eventos Adversos Relaciona	Falhas envolvend o cateter		
10/03/2023	CE	SOBRAL	HOSPITA L REGIONA	-	Enviada	Eventos Adversos Relaciona	Falhas envolvend o cateter		
10/03/2023	CE	SOBRAL	HOSPITA L REGIONA	-	Enviada	Eventos Adversos Relaciona	Falhas envolvend o cateter		
10/03/2023	CE	SOBRAL	HOSPITA L REGIONA	-	Enviada	Eventos Adversos Relaciona	Falhas envolvend o cateter		
10/03/2023	CE	SOBRAL	HOSPITA L REGIONA	-	Enviada	Eventos Adversos Relaciona	Falhas envolvend o cateter		
10/03/2023	CE	SOBRAL	HOSPITA L REGIONA	-	Enviada	Eventos Adversos Relaciona	Lesão por pressão		
10/03/2023	CE	SOBRAL	HOSPITA L REGIONA	-	Concluída	Eventos Adversos Relaciona	Lesão por pressão		

10/03/2023	CE	SOBRAL	HOSPITA L REGIONA	-	Enviada	Eventos Adversos Relaciona	Lesão por pressão		
10/03/2023	CE	SOBRAL	HOSPITA L REGIONA	-	Enviada	Eventos Adversos Relaciona	Lesão por pressão		
10/03/2023	CE	SOBRAL	HOSPITA L REGIONA	-	Enviada	Eventos Adversos Relaciona	Lesão por pressão		
10/03/2023	CE	SOBRAL	HOSPITA L REGIONA	-	Enviada	Eventos Adversos Relaciona	Lesão por pressão		
10/03/2023	CE	SOBRAL	HOSPITA L REGIONA	-	Enviada	Eventos Adversos Relaciona	Lesão por pressão		
10/03/2023	CE	SOBRAL	HOSPITA L REGIONA	-	Enviada	Eventos Adversos Relaciona	Lesão por pressão		
10/03/2023	CE	SOBRAL	HOSPITA L REGIONA	-	Enviada	Eventos Adversos Relaciona	Lesão por pressão		
10/03/2023	CE	SOBRAL	HOSPITA L REGIONA	-	Enviada	Eventos Adversos Relaciona	Lesão por pressão		
10/03/2023	CE	SOBRAL	HOSPITA L REGIONA	-	Enviada	Eventos Adversos Relaciona	Lesão por pressão		
10/03/2023	CE	SOBRAL	HOSPITA L REGIONA	-	Enviada	Eventos Adversos Relaciona	Lesão por pressão		
13/03/2023	CE	SOBRAL	HOSPITA L REGIONA	-	Enviada	Eventos Adversos Relaciona	Lesão por pressão		
13/03/2023	CE	SOBRAL	HOSPITA L REGIONA	-	Enviada	Eventos Adversos Relaciona	Lesão por pressão		
13/03/2023	CE	SOBRAL	HOSPITA L REGIONA	-	Enviada	Eventos Adversos Relaciona	Lesão por pressão		
13/03/2023	CE	SOBRAL	HOSPITA L REGIONA	-	Enviada	Eventos Adversos Relaciona	Lesão por pressão		
13/03/2023	CE	SOBRAL	HOSPITA L REGIONA	-	Enviada	Eventos Adversos Relaciona	Lesão por pressão		
13/03/2023	CE	SOBRAL	HOSPITA L REGIONA	-	Enviada	Eventos Adversos Relaciona	Lesão por pressão		
13/03/2023	CE	SOBRAL	HOSPITA L REGIONA	-	Enviada	Eventos Adversos Relaciona	Falhas durante a assistênci		
13/03/2023	CE	SOBRAL	HOSPITA L REGIONA	-	Enviada	Eventos Adversos Relaciona	Falhas durante a assistênci		
13/03/2023	CE	SOBRAL	HOSPITA L REGIONA	-	Enviada	Eventos Adversos Relaciona	Falhas durante a assistênci		

13/03/2023	CE	SOBRAL	HOSPITA L REGIONA	-	Enviada	Eventos Adversos Relaciona	Falhas durante a assistênci		
14/03/2023	CE	SOBRAL	HOSPITA L REGIONA	-	Enviada	Eventos Adversos Relaciona	Falhas nas atividades		
14/03/2023	CE	SOBRAL	HOSPITA L REGIONA	-	Enviada	Eventos Adversos Relaciona	Falhas envolvend o sondas		
14/03/2023	CE	SOBRAL	HOSPITA L REGIONA	-	Enviada	Eventos Adversos Relaciona	Falhas envolvend o sondas		
14/03/2023	CE	SOBRAL	HOSPITA L REGIONA	-	Enviada	Eventos Adversos Relaciona	Falhas envolvend o sondas		
14/03/2023	CE	SOBRAL	HOSPITA L REGIONA	-	Enviada	Eventos Adversos Relaciona	Falhas envolvend o sondas		
14/03/2023	CE	SOBRAL	HOSPITA L REGIONA	-	Enviada	Eventos Adversos Relaciona	Falhas envolvend o sondas		
14/03/2023	CE	SOBRAL	HOSPITA L REGIONA	-	Enviada	Eventos Adversos Relaciona	Falhas envolvend o sondas		
14/03/2023	CE	SOBRAL	HOSPITA L REGIONA	-	Enviada	Eventos Adversos Relaciona	Falhas envolvend o sondas		
14/03/2023	CE	SOBRAL	HOSPITA L REGIONA	-	Enviada	Eventos Adversos Relaciona	Falhas envolvend o sondas		
14/03/2023	CE	SOBRAL	HOSPITA L REGIONA	-	Enviada	Eventos Adversos Relaciona	Falhas envolvend o sondas		
14/03/2023	CE	SOBRAL	HOSPITA L REGIONA	-	Enviada	Eventos Adversos Relaciona	Falhas envolvend o sondas		
14/03/2023	CE	SOBRAL	HOSPITA L REGIONA	-	Enviada	Eventos Adversos Relaciona	Falhas envolvend o sondas		
14/03/2023	CE	SOBRAL	HOSPITA L REGIONA	-	Enviada	Eventos Adversos Relaciona	Falhas envolvend o sondas		
14/03/2023	CE	SOBRAL	HOSPITA L REGIONA	-	Enviada	Eventos Adversos Relaciona	Falhas envolvend o sondas		
14/03/2023	CE	SOBRAL	HOSPITA L REGIONA	-	Enviada	Eventos Adversos Relaciona	Falhas envolvend o sondas		
14/03/2023	CE	SOBRAL	HOSPITA L REGIONA	-	Enviada	Eventos Adversos Relaciona	Falhas envolvend o sondas		
14/03/2023	CE	SOBRAL	HOSPITA L REGIONA	-	Enviada	Eventos Adversos Relaciona	Falhas envolvend o sondas		
15/03/2023	CE	SOBRAL	HOSPITA L REGIONA	-	Enviada	Eventos Adversos Relaciona	Falhas envolvend o sondas		

15/03/2023	CE	SOBRAL	HOSPITA L REGIONA	-	Enviada	Eventos Adversos Relaciona	Falhas envolvend o sondas		
15/03/2023	CE	SOBRAL	HOSPITA L REGIONA	-	Enviada	Eventos Adversos Relaciona	Falhas envolvend o sondas		
15/03/2023	CE	SOBRAL	HOSPITA L REGIONA	-	Enviada	Eventos Adversos Relaciona	Falhas envolvend o sondas		
15/03/2023	CE	SOBRAL	HOSPITA L REGIONA	-	Enviada	Eventos Adversos Relaciona	Falhas envolvend o sondas		
15/03/2023	CE	SOBRAL	HOSPITA L REGIONA	-	Enviada	Eventos Adversos Relaciona	Falhas envolvend o sondas		
15/03/2023	CE	SOBRAL	HOSPITA L REGIONA	-	Enviada	Eventos Adversos Relaciona	Falhas envolvend o sondas		
15/03/2023	CE	SOBRAL	HOSPITA L REGIONA	-	Enviada	Eventos Adversos Relaciona	Falhas envolvend o sondas		
15/03/2023	CE	SOBRAL	HOSPITA L REGIONA	-	Enviada	Eventos Adversos Relaciona	Falha na document ação		
15/03/2023	CE	SOBRAL	HOSPITA L REGIONA	-	Enviada	Eventos Adversos Relaciona	Queda do paciente		
15/03/2023	CE	SOBRAL	HOSPITA L REGIONA	-	Enviada	Eventos Adversos Relaciona	Falhas durante a assistênci		
15/03/2023	CE	SOBRAL	HOSPITA L REGIONA	-	Enviada	Eventos Adversos Relaciona	Falhas durante a assistênci		
15/03/2023	CE	SOBRAL	HOSPITA L REGIONA	-	Enviada	Eventos Adversos Relaciona	Falhas nas atividades		
15/03/2023	CE	SOBRAL	HOSPITA L REGIONA	-	Enviada	Eventos Adversos Relaciona	Falhas durante a assistênci		
15/03/2023	CE	SOBRAL	HOSPITA L REGIONA	-	Enviada	Eventos Adversos Relaciona	Falha na document ação		
15/03/2023	CE	SOBRAL	HOSPITA L REGIONA	-	Enviada	Eventos Adversos Relaciona	Falhas na administra ção de		Enteral (oral, cateter
15/03/2023	CE	SOBRAL	HOSPITA L REGIONA	DERMATI TE	Enviada	Eventos Adversos Relaciona	Falhas durante a assistênci		
15/03/2023	CE	SOBRAL	HOSPITA L REGIONA	-	Enviada	Eventos Adversos Relaciona	Extubaçã o endotraqu		
15/03/2023	CE	SOBRAL	HOSPITA L REGIONA	-	Enviada	Eventos Adversos Relaciona	Falhas durante a assistênci		
15/03/2023	CE	SOBRAL	HOSPITA L REGIONA	-	Enviada	Eventos Adversos Relaciona	Falhas durante a assistênci		

15/03/2023	CE	SOBRAL	HOSPITA L REGIONA	-	Enviada	Eventos Adversos Relaciona	Falhas durante a assistênci		
15/03/2023	CE	SOBRAL	HOSPITA L REGIONA	DERMATI TE	Enviada	Eventos Adversos Relaciona	Falhas durante a assistênci		
15/03/2023	CE	SOBRAL	HOSPITA L REGIONA	-	Enviada	Eventos Adversos Relaciona	Falhas durante a assistênci		
15/03/2023	CE	SOBRAL	HOSPITA L REGIONA	-	Enviada	Eventos Adversos Relaciona	Falhas durante a assistênci		
15/03/2023	CE	SOBRAL	HOSPITA L REGIONA	-	Enviada	Eventos Adversos Relaciona	Falhas durante a assistênci		
15/03/2023	CE	SOBRAL	HOSPITA L REGIONA	-	Enviada	Eventos Adversos Relaciona	Falhas durante a assistênci		
16/03/2023	CE	SOBRAL	HOSPITA L REGIONA	-	Enviada	Eventos Adversos Relaciona	Falhas na administra ção de		Enteral (oral, cateter
16/03/2023	CE	SOBRAL	HOSPITA L REGIONA	-	Enviada	Eventos Adversos Relaciona	Falhas na administra ção de		Enteral (oral, cateter
16/03/2023	CE	SOBRAL	HOSPITA L REGIONA	-	Enviada	Eventos Adversos Relaciona	Tromboe mbolismo venoso		
17/03/2023	CE	SOBRAL	HOSPITA L REGIONA	-	Enviada	Eventos Adversos Relaciona	Falhas durante a assistênci		
17/03/2023	CE	SOBRAL	HOSPITA L REGIONA	-	Enviada	Eventos Adversos Relaciona	Queda do paciente		
17/03/2023	CE	SOBRAL	HOSPITA L REGIONA	EXTUBA ÇÃO ACIDENT	Enviada	Eventos Adversos Relaciona	Extubaçã o endotraqu		
17/03/2023	CE	SOBRAL	HOSPITA L REGIONA	-	Enviada	Eventos Adversos Relaciona	Falhas durante a assistênci		
17/03/2023	CE	SOBRAL	HOSPITA L REGIONA	-	Enviada	Eventos Adversos Relaciona	Falhas na administra ção de		Enteral (oral, cateter
17/03/2023	CE	SOBRAL	HOSPITA L REGIONA	-	Enviada	Eventos Adversos Relaciona	Queda do paciente		
17/03/2023	CE	SOBRAL	HOSPITA L REGIONA	-	Enviada	Eventos Adversos Relaciona	Falhas na assistênci a		
17/03/2023	CE	SOBRAL	HOSPITA L REGIONA	-	Enviada	Eventos Adversos Relaciona	Falhas durante a assistênci		
17/03/2023	CE	SOBRAL	HOSPITA L REGIONA	-	Enviada	Eventos Adversos Relaciona	Falhas durante a assistênci		
17/03/2023	CE	SOBRAL	HOSPITA L REGIONA	-	Enviada	Eventos Adversos Relaciona	Falhas durante a assistênci		

17/03/2023	CE	SOBRAL	HOSPITA L REGIONA	-	Enviada	Eventos Adversos Relaciona	Lesão por pressão		
17/03/2023	CE	SOBRAL	HOSPITA L REGIONA	-	Enviada	Eventos Adversos Relaciona	Falhas envolvend o cateter		
17/03/2023	CE	SOBRAL	HOSPITA L REGIONA	-	Enviada	Eventos Adversos Relaciona	Falhas envolvend o cateter		
17/03/2023	CE	SOBRAL	HOSPITA L REGIONA	-	Enviada	Eventos Adversos Relaciona	Falhas nas atividades		
17/03/2023	CE	SOBRAL	HOSPITA L REGIONA	-	Enviada	Eventos Adversos Relaciona	Lesão por pressão		
17/03/2023	CE	SOBRAL	HOSPITA L REGIONA	-	Enviada	Eventos Adversos Relaciona	Falhas durante a assistênci		
17/03/2023	CE	SOBRAL	HOSPITA L REGIONA	-	Enviada	Eventos Adversos Relaciona	Falhas envolvend o cateter		
17/03/2023	CE	SOBRAL	HOSPITA L REGIONA	-	Enviada	Eventos Adversos Relaciona	Falhas durante a assistênci		
17/03/2023	CE	SOBRAL	HOSPITA L REGIONA	-	Enviada	Eventos Adversos Relaciona	Queda do paciente		
17/03/2023	CE	SOBRAL	HOSPITA L REGIONA	-	Enviada	Eventos Adversos Relaciona	Queda do paciente		
17/03/2023	CE	SOBRAL	HOSPITA L REGIONA	-	Enviada	Eventos Adversos Relaciona	Falhas ocorridas em		
17/03/2023	CE	SOBRAL	HOSPITA L REGIONA	-	Enviada	Eventos Adversos Relaciona	Falhas durante a assistênci		
17/03/2023	CE	SOBRAL	HOSPITA L REGIONA	-	Enviada	Eventos Adversos Relaciona	Falhas nas atividades		
17/03/2023	CE	SOBRAL	HOSPITA L REGIONA	-	Enviada	Eventos Adversos Relaciona	Queda do paciente		
17/03/2023	CE	SOBRAL	HOSPITA L REGIONA	-	Enviada	Eventos Adversos Relaciona	Extubaçã o endotraqu		
17/03/2023	CE	SOBRAL	HOSPITA L REGIONA	-	Enviada	Eventos Adversos Relaciona	Falhas envolvend o cateter		
17/03/2023	CE	SOBRAL	HOSPITA L REGIONA	-	Enviada	Eventos Adversos Relaciona	Falha na document ação		
17/03/2023	CE	SOBRAL	HOSPITA L REGIONA	-	Enviada	Eventos Adversos Relaciona	Falhas envolvend o cateter		
17/03/2023	CE	SOBRAL	HOSPITA L REGIONA	-	Enviada	Eventos Adversos Relaciona	Falhas nas atividades		

17/03/2023	CE	SOBRAL	HOSPITA L REGIONA	-	Enviada	Eventos Adversos Relaciona	Falhas nas atividades		
17/03/2023	CE	SOBRAL	HOSPITA L REGIONA	-	Enviada	Eventos Adversos Relaciona	Falhas durante a assistênci		
17/03/2023	CE	SOBRAL	HOSPITA L REGIONA	-	Enviada	Eventos Adversos Relaciona	Queda do paciente		
17/03/2023	CE	SOBRAL	HOSPITA L REGIONA	-	Enviada	Eventos Adversos Relaciona	Falha na document ação		
17/03/2023	CE	SOBRAL	HOSPITA L REGIONA	-	Enviada	Eventos Adversos Relaciona	Falhas durante a assistênci		
17/03/2023	CE	SOBRAL	HOSPITA L REGIONA	-	Enviada	Eventos Adversos Relaciona	Falha na document ação		
17/03/2023	CE	SOBRAL	HOSPITA L REGIONA	-	Enviada	Eventos Adversos Relaciona	Falhas ocorridas em		
17/03/2023	CE	SOBRAL	HOSPITA L REGIONA	-	Enviada	Eventos Adversos Relaciona	Queda do paciente		
17/03/2023	CE	SOBRAL	HOSPITA L REGIONA	-	Enviada	Eventos Adversos Relaciona	Lesão por pressão		
17/03/2023	CE	SOBRAL	HOSPITA L REGIONA	-	Enviada	Eventos Adversos Relaciona	Lesão por pressão		
17/03/2023	CE	SOBRAL	HOSPITA L REGIONA	-	Enviada	Eventos Adversos Relaciona	Lesão por pressão		
17/03/2023	CE	SOBRAL	HOSPITA L REGIONA	-	Enviada	Eventos Adversos Relaciona	Lesão por pressão		
17/03/2023	CE	SOBRAL	HOSPITA L REGIONA	-	Enviada	Eventos Adversos Relaciona	Lesão por pressão		
17/03/2023	CE	SOBRAL	HOSPITA L REGIONA	-	Enviada	Eventos Adversos Relaciona	Lesão por pressão		
17/03/2023	CE	SOBRAL	HOSPITA L REGIONA	-	Enviada	Eventos Adversos Relaciona	Lesão por pressão		
17/03/2023	CE	SOBRAL	HOSPITA L REGIONA	-	Enviada	Eventos Adversos Relaciona	Lesão por pressão		
17/03/2023	CE	SOBRAL	HOSPITA L REGIONA	-	Enviada	Eventos Adversos Relaciona	Lesão por pressão		
17/03/2023	CE	SOBRAL	HOSPITA L REGIONA	-	Enviada	Eventos Adversos Relaciona	Lesão por pressão		
17/03/2023	CE	SOBRAL	HOSPITA L REGIONA	-	Enviada	Eventos Adversos Relaciona	Lesão por pressão		

21/03/2023	CE	SOBRAL	HOSPITA L REGIONA	-	Enviada	Eventos Adversos Relaciona	Falhas envolvend o sondas		
21/03/2023	CE	SOBRAL	HOSPITA L REGIONA	-	Enviada	Eventos Adversos Relaciona	Falhas durante a assistênci		
21/03/2023	CE	SOBRAL	HOSPITA L REGIONA	-	Enviada	Eventos Adversos Relaciona	Falhas nas atividades		
21/03/2023	CE	SOBRAL	HOSPITA L REGIONA	-	Enviada	Eventos Adversos Relaciona	Falhas nas atividades		
21/03/2023	CE	SOBRAL	HOSPITA L REGIONA	-	Enviada	Eventos Adversos Relaciona	Falhas envolvend o cateter		
21/03/2023	CE	SOBRAL	HOSPITA L REGIONA	-	Enviada	Eventos Adversos Relaciona	Falhas durante a assistênci		
21/03/2023	CE	SOBRAL	HOSPITA L REGIONA	-	Enviada	Eventos Adversos Relaciona	Broncoas piração		
22/03/2023	CE	SOBRAL	HOSPITA L REGIONA	-	Enviada	Eventos Adversos Relaciona	Falhas envolvend o cateter		
22/03/2023	CE	SOBRAL	HOSPITA L REGIONA	-	Enviada	Eventos Adversos Relaciona	Falhas envolvend o cateter		
22/03/2023	CE	SOBRAL	HOSPITA L REGIONA	-	Enviada	Eventos Adversos Relaciona	Falhas durante a assistênci		
22/03/2023	CE	SOBRAL	HOSPITA L REGIONA	-	Enviada	Eventos Adversos Relaciona	Falhas durante a assistênci		
22/03/2023	CE	SOBRAL	HOSPITA L REGIONA	-	Enviada	Eventos Adversos Relaciona	Falhas durante a assistênci		
22/03/2023	CE	SOBRAL	HOSPITA L REGIONA	-	Enviada	Eventos Adversos Relaciona	Falhas durante a assistênci		
22/03/2023	CE	SOBRAL	HOSPITA L REGIONA	-	Enviada	Eventos Adversos Relaciona	Falhas durante a assistênci		
22/03/2023	CE	SOBRAL	HOSPITA L REGIONA	-	Enviada	Eventos Adversos Relaciona	Falhas durante a assistênci		
22/03/2023	CE	SOBRAL	HOSPITA L REGIONA	-	Enviada	Eventos Adversos Relaciona	Falhas durante a assistênci		
22/03/2023	CE	SOBRAL	HOSPITA L REGIONA	-	Enviada	Eventos Adversos Relaciona	Falhas durante a assistênci		
22/03/2023	CE	SOBRAL	HOSPITA L REGIONA	-	Enviada	Eventos Adversos Relaciona	Falhas nas atividades		
22/03/2023	CE	SOBRAL	HOSPITA L REGIONA	-	Enviada	Eventos Adversos Relaciona	Falhas ocorridas em		

22/03/2023	CE	SOBRAL	HOSPITA L REGIONA	-	Enviada	Eventos Adversos Relaciona	Lesão por pressão		
22/03/2023	CE	SOBRAL	HOSPITA L REGIONA	-	Enviada	Eventos Adversos Relaciona	Falhas durante a assistênci		
22/03/2023	CE	SOBRAL	HOSPITA L REGIONA	DERMATI TE	Enviada	Eventos Adversos Relaciona	Falhas durante a assistênci		
22/03/2023	CE	SOBRAL	HOSPITA L REGIONA	-	Enviada	Eventos Adversos Relaciona	Falhas envolvend o sondas		
22/03/2023	CE	SOBRAL	HOSPITA L REGIONA	-	Enviada	Eventos Adversos Relaciona	Falha na document ação		
22/03/2023	CE	SOBRAL	HOSPITA L REGIONA	-	Enviada	Eventos Adversos Relaciona	Falhas durante a assistênci		
22/03/2023	CE	SOBRAL	HOSPITA L REGIONA	-	Concluída	Eventos Adversos Relaciona	Lesão por pressão		
22/03/2023	CE	SOBRAL	HOSPITA L REGIONA	-	Enviada	Eventos Adversos Relaciona	Falhas na assistênci a		
22/03/2023	CE	SOBRAL	HOSPITA L REGIONA	-	Enviada	Eventos Adversos Relaciona	Falhas durante a assistênci		
24/03/2023	CE	SOBRAL	HOSPITA L REGIONA	-	Enviada	Eventos Adversos Relaciona	Lesão por pressão		
24/03/2023	CE	SOBRAL	HOSPITA L REGIONA	-	Enviada	Eventos Adversos Relaciona	Lesão por pressão		
24/03/2023	CE	SOBRAL	HOSPITA L REGIONA	-	Enviada	Eventos Adversos Relaciona	Lesão por pressão		
24/03/2023	CE	SOBRAL	HOSPITA L REGIONA	-	Enviada	Eventos Adversos Relaciona	Lesão por pressão		
24/03/2023	CE	SOBRAL	HOSPITA L REGIONA	-	Enviada	Eventos Adversos Relaciona	Lesão por pressão		
24/03/2023	CE	SOBRAL	HOSPITA L REGIONA	-	Enviada	Eventos Adversos Relaciona	Lesão por pressão		
24/03/2023	CE	SOBRAL	HOSPITA L REGIONA	-	Enviada	Eventos Adversos Relaciona	Lesão por pressão		
24/03/2023	CE	SOBRAL	HOSPITA L REGIONA	-	Enviada	Eventos Adversos Relaciona	Lesão por pressão		
24/03/2023	CE	SOBRAL	HOSPITA L REGIONA	-	Concluída	Eventos Adversos Relaciona	Lesão por pressão		
24/03/2023	CE	SOBRAL	HOSPITA L REGIONA	-	Enviada	Eventos Adversos Relaciona	Lesão por pressão		

24/03/2023	CE	SOBRAL	HOSPITA L REGIONA	-	Enviada	Eventos Adversos Relaciona	Falhas nas atividades		
24/03/2023	CE	SOBRAL	HOSPITA L REGIONA	-	Enviada	Eventos Adversos Relaciona	Falhas nas atividades		
24/03/2023	CE	SOBRAL	HOSPITA L REGIONA	-	Enviada	Eventos Adversos Relaciona	Falhas durante a assistênci		
24/03/2023	CE	SOBRAL	HOSPITA L REGIONA	-	Enviada	Eventos Adversos Relaciona	Tromboe mbolismo venoso		
24/03/2023	CE	SOBRAL	HOSPITA L REGIONA	-	Enviada	Eventos Adversos Relaciona	Queda do paciente		
24/03/2023	CE	SOBRAL	HOSPITA L REGIONA	-	Enviada	Eventos Adversos Relaciona	Falhas nas atividades		
24/03/2023	CE	SOBRAL	HOSPITA L REGIONA	-	Enviada	Eventos Adversos Relaciona	Queda do paciente		
24/03/2023	CE	SOBRAL	HOSPITA L REGIONA	-	Enviada	Eventos Adversos Relaciona	Lesão por pressão		
24/03/2023	CE	SOBRAL	HOSPITA L REGIONA	-	Enviada	Eventos Adversos Relaciona	Falhas envolvend o cateter		
24/03/2023	CE	SOBRAL	HOSPITA L REGIONA	-	Enviada	Eventos Adversos Relaciona	Falha na document ação		
24/03/2023	CE	SOBRAL	HOSPITA L REGIONA	-	Enviada	Eventos Adversos Relaciona	Falhas envolvend o cateter		
24/03/2023	CE	SOBRAL	HOSPITA L REGIONA	-	Enviada	Eventos Adversos Relaciona	Falhas envolvend o cateter		
24/03/2023	CE	SOBRAL	HOSPITA L REGIONA	-	Enviada	Eventos Adversos Relaciona	Falhas durante a assistênci		
24/03/2023	CE	SOBRAL	HOSPITA L REGIONA	-	Enviada	Eventos Adversos Relaciona	Falhas nas atividades		
24/03/2023	CE	SOBRAL	HOSPITA L REGIONA	-	Enviada	Eventos Adversos Relaciona	Falhas durante a assistênci		
24/03/2023	CE	SOBRAL	HOSPITA L REGIONA	-	Enviada	Eventos Adversos Relaciona	Falhas envolvend o cateter		
24/03/2023	CE	SOBRAL	HOSPITA L REGIONA	-	Enviada	Eventos Adversos Relaciona	Falhas envolvend o cateter		
24/03/2023	CE	SOBRAL	HOSPITA L REGIONA	-	Enviada	Eventos Adversos Relaciona	Falhas durante a assistênci		
24/03/2023	CE	SOBRAL	HOSPITA L REGIONA	-	Enviada	Eventos Adversos Relaciona	Falhas ocorridas em		

24/03/2023	CE	SOBRAL	HOSPITA L REGIONA	-	Enviada	Eventos Adversos Relaciona	Falha na document ação		
24/03/2023	CE	SOBRAL	HOSPITA L REGIONA	-	Enviada	Eventos Adversos Relaciona	Falhas durante procedim		
24/03/2023	CE	SOBRAL	HOSPITA L REGIONA	-	Enviada	Eventos Adversos Relaciona	Falhas nas atividades		
24/03/2023	CE	SOBRAL	HOSPITA L REGIONA	-	Enviada	Eventos Adversos Relaciona	Falhas durante a assistênci		
27/03/2023	CE	SOBRAL	HOSPITA L REGIONA	-	Enviada	Eventos Adversos Relaciona	Lesão por pressão		
27/03/2023	CE	SOBRAL	HOSPITA L REGIONA	-	Enviada	Eventos Adversos Relaciona	Lesão por pressão		
27/03/2023	CE	SOBRAL	HOSPITA L REGIONA	-	Enviada	Eventos Adversos Relaciona	Lesão por pressão		
27/03/2023	CE	SOBRAL	HOSPITA L REGIONA	-	Enviada	Eventos Adversos Relaciona	Falhas durante a assistênci		
27/03/2023	CE	SOBRAL	HOSPITA L REGIONA	-	Enviada	Eventos Adversos Relaciona	Falhas durante a assistênci		
27/03/2023	CE	SOBRAL	HOSPITA L REGIONA	-	Enviada	Eventos Adversos Relaciona	Falhas envolvend o cateter		
27/03/2023	CE	SOBRAL	HOSPITA L REGIONA	-	Enviada	Eventos Adversos Relaciona	Queda do paciente		
27/03/2023	CE	SOBRAL	HOSPITA L REGIONA	-	Enviada	Eventos Adversos Relaciona	Queda do paciente		
27/03/2023	CE	SOBRAL	HOSPITA L REGIONA	-	Enviada	Eventos Adversos Relaciona	Falhas durante procedim		
27/03/2023	CE	SOBRAL	HOSPITA L REGIONA	-	Enviada	Eventos Adversos Relaciona	Falhas na assistênci a		
27/03/2023	CE	SOBRAL	HOSPITA L REGIONA	-	Enviada	Eventos Adversos Relaciona	Falhas na assistênci a		
28/03/2023	CE	SOBRAL	HOSPITA L REGIONA	-	Enviada	Eventos Adversos Relaciona	Falhas durante a assistênci		
28/03/2023	CE	SOBRAL	HOSPITA L REGIONA	-	Enviada	Eventos Adversos Relaciona	Falhas durante a assistênci		
28/03/2023	CE	SOBRAL	HOSPITA L REGIONA	-	Enviada	Eventos Adversos Relaciona	Lesão por pressão		
28/03/2023	CE	SOBRAL	HOSPITA L REGIONA	-	Enviada	Eventos Adversos Relaciona	Falhas envolvend o cateter		

28/03/2023	CE	SOBRAL	HOSPITA L REGIONA	-	Enviada	Eventos Adversos Relaciona	Falha na document ação		
28/03/2023	CE	SOBRAL	HOSPITA L REGIONA	-	Enviada	Eventos Adversos Relaciona	Falhas durante a assistênci		
28/03/2023	CE	SOBRAL	HOSPITA L REGIONA	-	Enviada	Eventos Adversos Relaciona	Falhas envolvend o sondas		
28/03/2023	CE	SOBRAL	HOSPITA L REGIONA	-	Enviada	Eventos Adversos Relaciona	Lesão por pressão		
28/03/2023	CE	SOBRAL	HOSPITA L REGIONA	-	Enviada	Eventos Adversos Relaciona	Lesão por pressão		
28/03/2023	CE	SOBRAL	HOSPITA L REGIONA	-	Enviada	Eventos Adversos Relaciona	Lesão por pressão		
30/03/2023	CE	SOBRAL	HOSPITA L REGIONA	-	Enviada	Eventos Adversos Relaciona	Lesão por pressão		
30/03/2023	CE	SOBRAL	HOSPITA L REGIONA	-	Enviada	Eventos Adversos Relaciona	Lesão por pressão		
30/03/2023	CE	SOBRAL	HOSPITA L REGIONA	-	Enviada	Eventos Adversos Relaciona	Falhas envolvend o sondas		
30/03/2023	CE	SOBRAL	HOSPITA L REGIONA	-	Enviada	Eventos Adversos Relaciona	Queda do paciente		
30/03/2023	CE	SOBRAL	HOSPITA L REGIONA	-	Enviada	Eventos Adversos Relaciona	Falhas envolvend o sondas		
30/03/2023	CE	SOBRAL	HOSPITA L REGIONA	-	Enviada	Eventos Adversos Relaciona	Falhas envolvend o cateter		
30/03/2023	CE	SOBRAL	HOSPITA L REGIONA	-	Enviada	Eventos Adversos Relaciona	Lesão por pressão		
30/03/2023	CE	SOBRAL	HOSPITA L REGIONA	-	Enviada	Eventos Adversos Relaciona	Lesão por pressão		
30/03/2023	CE	SOBRAL	HOSPITA L REGIONA	-	Enviada	Eventos Adversos Relaciona	Lesão por pressão		
30/03/2023	CE	SOBRAL	HOSPITA L REGIONA	-	Enviada	Eventos Adversos Relaciona	Lesão por pressão		
30/03/2023	CE	SOBRAL	HOSPITA L REGIONA	-	Enviada	Eventos Adversos Relaciona	Falhas durante a assistênci		
30/03/2023	CE	SOBRAL	HOSPITA L REGIONA	-	Enviada	Eventos Adversos Relaciona	Lesão por pressão		
30/03/2023	CE	SOBRAL	HOSPITA L REGIONA	-	Enviada	Eventos Adversos Relaciona	Lesão por pressão		

31/03/2023	CE	SOBRAL	HOSPITA L REGIONA	-	Enviada	Eventos Adversos Relaciona	Falhas durante a assistênci		
31/03/2023	CE	SOBRAL	HOSPITA L REGIONA	-	Enviada	Eventos Adversos Relaciona	Falhas durante a assistênci		
31/03/2023	CE	SOBRAL	HOSPITA L REGIONA	-	Enviada	Eventos Adversos Relaciona	Falhas durante a assistênci		
31/03/2023	CE	SOBRAL	HOSPITA L REGIONA	-	Enviada	Eventos Adversos Relaciona	Falhas durante a assistênci		
31/03/2023	CE	SOBRAL	HOSPITA L REGIONA	-	Enviada	Eventos Adversos Relaciona	Lesão por pressão		
31/03/2023	CE	SOBRAL	HOSPITA L REGIONA	-	Enviada	Eventos Adversos Relaciona	Lesão por pressão		
31/03/2023	CE	SOBRAL	HOSPITA L REGIONA	-	Enviada	Eventos Adversos Relaciona	Falhas envolvend o cateter		
31/03/2023	CE	SOBRAL	HOSPITA L REGIONA	-	Enviada	Eventos Adversos Relaciona	Falha na document ação		
31/03/2023	CE	SOBRAL	HOSPITA L REGIONA	-	Enviada	Eventos Adversos Relaciona	Lesão por pressão		
31/03/2023	CE	SOBRAL	HOSPITA L REGIONA	-	Enviada	Eventos Adversos Relaciona	Lesão por pressão		
31/03/2023	CE	SOBRAL	HOSPITA L REGIONA	-	Enviada	Eventos Adversos Relaciona	Falhas durante a assistênci		
31/03/2023	CE	SOBRAL	HOSPITA L REGIONA	-	Enviada	Eventos Adversos Relaciona	Falhas durante a assistênci		
31/03/2023	CE	SOBRAL	HOSPITA L REGIONA	-	Enviada	Eventos Adversos Relaciona	Falhas nas atividades		
31/03/2023	CE	SOBRAL	HOSPITA L REGIONA	-	Enviada	Eventos Adversos Relaciona	Lesão por pressão		
05/02/2019	CE	QUIXERA MOBIM	HOSPITA L REGIONA L DO SERTÃO	-	Enviada	Eventos Adversos Relaciona dos à Assistênci	Lesão por pressão		
15/02/2019	CE	QUIXERA MOBIM	HOSPITA L REGIONA L DO SERTÃO	-	Enviada	Eventos Adversos Relaciona dos à Assistênci	Queda do paciente		
15/02/2019	CE	QUIXERA MOBIM	HOSPITA L REGIONA L DO SERTÃO	Broncoas piração	Enviada	Eventos Adversos Relaciona dos à Assistênci	Outro		

15/02/2019	CE	QUIXERAMOBIM	HOSPITAL REGIONAL DO SERTÃO	Saída não planejada de cânula endotraqu	Enviada	Eventos Adversos Relacionados à Assistênci	Outro		
15/02/2019	CE	QUIXERAMOBIM	HOSPITAL REGIONAL DO SERTÃO	Saída não planejada de cânula endotraqu	Enviada	Eventos Adversos Relacionados à Assistênci	Outro		
15/02/2019	CE	QUIXERAMOBIM	HOSPITAL REGIONAL DO SERTÃO	-	Enviada	Eventos Adversos Relacionados à Assistênci	Lesão por pressão		
15/02/2019	CE	QUIXERAMOBIM	HOSPITAL REGIONAL DO SERTÃO	-	Enviada	Eventos Adversos Relacionados à Assistênci	Lesão por pressão		
15/02/2019	CE	QUIXERAMOBIM	HOSPITAL REGIONAL DO SERTÃO	-	Enviada	Eventos Adversos Relacionados à Assistênci	Lesão por pressão		
17/05/2019	CE	QUIXERAMOBIM	HOSPITAL REGIONAL DO SERTÃO	-	Enviada	Eventos Adversos Relacionados à Assistênci	Lesão por pressão		
17/05/2019	CE	QUIXERAMOBIM	HOSPITAL REGIONAL DO SERTÃO	-	Enviada	Eventos Adversos Relacionados à Assistênci	Lesão por pressão		
17/05/2019	CE	QUIXERAMOBIM	HOSPITAL REGIONAL DO SERTÃO	-	Enviada	Eventos Adversos Relacionados à Assistênci	Lesão por pressão		
17/05/2019	CE	QUIXERAMOBIM	HOSPITAL REGIONAL DO SERTÃO	Broncoaspiração	Enviada	Eventos Adversos Relacionados à Assistênci	Outro		
17/05/2019	CE	QUIXERAMOBIM	HOSPITAL REGIONAL DO SERTÃO	-	Enviada	Eventos Adversos Relacionados à Assistênci	Queda do paciente		
17/05/2019	CE	QUIXERAMOBIM	HOSPITAL REGIONAL DO SERTÃO	-	Enviada	Eventos Adversos Relacionados à Assistênci	Queda do paciente		
17/05/2019	CE	QUIXERAMOBIM	HOSPITAL REGIONAL DO SERTÃO	-	Enviada	Eventos Adversos Relacionados à Assistênci	Lesão por pressão		

23/07/2019	CE	QUIXERA MOBIM	HOSPITA L REGIONA L DO SERTÃO	-	Enviada	Eventos Adversos Relaciona dos à Assistênci	Lesão por pressão		
23/07/2019	CE	QUIXERA MOBIM	HOSPITA L REGIONA L DO SERTÃO	-	Enviada	Eventos Adversos Relaciona dos à Assistênci	Queda do paciente		
23/07/2019	CE	QUIXERA MOBIM	HOSPITA L REGIONA L DO SERTÃO	-	Enviada	Eventos Adversos Relaciona dos à Assistênci	Lesão por pressão		
23/07/2019	CE	QUIXERA MOBIM	HOSPITA L REGIONA L DO SERTÃO	-	Enviada	Eventos Adversos Relaciona dos à Assistênci	Queda do paciente		
23/07/2019	CE	QUIXERA MOBIM	HOSPITA L REGIONA L DO SERTÃO	-	Enviada	Eventos Adversos Relaciona dos à Assistênci	Queda do paciente		
23/07/2019	CE	QUIXERA MOBIM	HOSPITA L REGIONA L DO SERTÃO	-	Enviada	Eventos Adversos Relaciona dos à Assistênci	Lesão por pressão		
23/07/2019	CE	QUIXERA MOBIM	HOSPITA L REGIONA L DO SERTÃO	-	Enviada	Eventos Adversos Relaciona dos à Assistênci	Queda do paciente		
23/07/2019	CE	QUIXERA MOBIM	HOSPITA L REGIONA L DO SERTÃO	-	Enviada	Eventos Adversos Relaciona dos à Assistênci	Lesão por pressão		
23/07/2019	CE	QUIXERA MOBIM	HOSPITA L REGIONA L DO SERTÃO	-	Enviada	Eventos Adversos Relaciona dos à Assistênci	Broncoas piração		
25/11/2019	CE	QUIXERA MOBIM	HOSPITA L REGIONA L DO SERTÃO	Extubaçã o	Enviada	Eventos Adversos Relaciona dos à Assistênci	Extubaça o endotraqu eal acidental		
25/11/2019	CE	QUIXERA MOBIM	HOSPITA L REGIONA L DO SERTÃO	-	Enviada	Eventos Adversos Relaciona dos à Assistênci	Falhas envolvend o sondas		
25/11/2019	CE	QUIXERA MOBIM	HOSPITA L REGIONA L DO SERTÃO	-	Enviada	Eventos Adversos Relaciona dos à Assistênci	Extubaça o endotraqu eal acidental		

17/12/2019	CE	QUIXERAMOBIM	HOSPITAL REGIONAL DO SERTÃO	-	Enviada	Eventos Adversos Relacionados à Assistênci	Broncoaspiração		
17/12/2019	CE	QUIXERAMOBIM	HOSPITAL REGIONAL DO SERTÃO	-	Enviada	Eventos Adversos Relacionados à Assistênci	Broncoaspiração		
18/05/2021	CE	QUIXERAMOBIM	HOSPITAL REGIONAL DO SERTÃO	-	Enviada	Eventos Adversos Relacionados à Assistênci	Extubaçao endotraqueal acidental		
18/05/2021	CE	QUIXERAMOBIM	HOSPITAL REGIONAL DO SERTÃO	-	Enviada	Eventos Adversos Relacionados à Assistênci	Queda do paciente		
18/05/2021	CE	QUIXERAMOBIM	HOSPITAL REGIONAL DO SERTÃO	Dano: fratura de membro superior	Enviada	Eventos Adversos Relacionados à Assistênci	Queda do paciente		
18/05/2021	CE	QUIXERAMOBIM	HOSPITAL REGIONAL DO SERTÃO	-	Enviada	Eventos Adversos Relacionados à Assistênci	Extubaçao endotraqueal acidental		
30/06/2021	CE	QUIXERAMOBIM	HOSPITAL REGIONAL DO SERTÃO	-	Enviada	Eventos Adversos Relacionados à Assistênci	Extubaçao endotraqueal acidental		
02/09/2021	CE	QUIXERAMOBIM	HOSPITAL REGIONAL DO SERTÃO	-	Enviada	Eventos Adversos Relacionados à Assistênci	Queda do paciente		
02/09/2021	CE	QUIXERAMOBIM	HOSPITAL REGIONAL DO SERTÃO	-	Enviada	Eventos Adversos Relacionados à Assistênci	Broncoaspiração		
06/09/2022	CE	QUIXERAMOBIM	HOSPITAL REGIONAL DO SERTÃO	-	Enviada	Eventos Adversos Relacionados à Assistênci	Lesão por pressão		
06/09/2022	CE	QUIXERAMOBIM	HOSPITAL REGIONAL DO SERTÃO	-	Enviada	Eventos Adversos Relacionados à Assistênci	Lesão por pressão		
06/09/2022	CE	QUIXERAMOBIM	HOSPITAL REGIONAL DO SERTÃO	-	Enviada	Eventos Adversos Relacionados à Assistênci	Lesão por pressão		

06/09/2022	CE	QUIXERA MOBIM	HOSPITA L REGIONA L DO SERTÃO	-	Enviada	Eventos Adversos Relaciona dos à Assistênci	Lesão por pressão		
06/09/2022	CE	QUIXERA MOBIM	HOSPITA L REGIONA L DO SERTÃO	-	Enviada	Eventos Adversos Relaciona dos à Assistênci	Lesão por pressão		
06/09/2022	CE	QUIXERA MOBIM	HOSPITA L REGIONA L DO SERTÃO	-	Enviada	Eventos Adversos Relaciona dos à Assistênci	Lesão por pressão		
06/09/2022	CE	QUIXERA MOBIM	HOSPITA L REGIONA L DO SERTÃO	-	Enviada	Eventos Adversos Relaciona dos à Assistênci	Lesão por pressão		
06/09/2022	CE	QUIXERA MOBIM	HOSPITA L REGIONA L DO SERTÃO	-	Enviada	Eventos Adversos Relaciona dos à Assistênci	Lesão por pressão		
06/09/2022	CE	QUIXERA MOBIM	HOSPITA L REGIONA L DO SERTÃO	Tissular profunda	Enviada	Eventos Adversos Relaciona dos à Assistênci	Lesão por pressão		
06/09/2022	CE	QUIXERA MOBIM	HOSPITA L REGIONA L DO SERTÃO	Tissular profunda	Enviada	Eventos Adversos Relaciona dos à Assistênci	Lesão por pressão		
06/09/2022	CE	QUIXERA MOBIM	HOSPITA L REGIONA L DO SERTÃO	-	Enviada	Eventos Adversos Relaciona dos à Assistênci	Lesão por pressão		
06/09/2022	CE	QUIXERA MOBIM	HOSPITA L REGIONA L DO SERTÃO	-	Enviada	Eventos Adversos Relaciona dos à Assistênci	Lesão por pressão		
12/09/2022	CE	QUIXERA MOBIM	HOSPITA L REGIONA L DO SERTÃO	-	Enviada	Eventos Adversos Relaciona dos à Assistênci	Queda do paciente		
12/09/2022	CE	QUIXERA MOBIM	HOSPITA L REGIONA L DO SERTÃO	-	Enviada	Eventos Adversos Relaciona dos à Assistênci	Queda do paciente		
12/09/2022	CE	QUIXERA MOBIM	HOSPITA L REGIONA L DO SERTÃO	-	Enviada	Eventos Adversos Relaciona dos à Assistênci	Queda do paciente		

12/09/2022	CE	QUIXERA MOBIM	HOSPITA L REGIONA L DO SERTÃO	-	Enviada	Eventos Adversos Relaciona dos à Assistênci	Queda do paciente		
13/09/2022	CE	QUIXERA MOBIM	HOSPITA L REGIONA L DO SERTÃO	Maca do SAMU durante espera do	Enviada	Eventos Adversos Relaciona dos à Assistênci	Queda do paciente		
15/09/2022	CE	QUIXERA MOBIM	HOSPITA L REGIONA L DO SERTÃO	-	Enviada	Eventos Adversos Relaciona dos à Assistênci	Extubaça o endotraqu eal acidental		
19/09/2022	CE	QUIXERA MOBIM	HOSPITA L REGIONA L DO SERTÃO	-	Enviada	Eventos Adversos Relaciona dos à Assistênci	Queda do paciente		
21/09/2022	CE	QUIXERA MOBIM	HOSPITA L REGIONA L DO SERTÃO	-	Enviada	Eventos Adversos Relaciona dos à Assistênci	Queda do paciente		
26/09/2022	CE	QUIXERA MOBIM	HOSPITA L REGIONA L DO SERTÃO	-	Enviada	Eventos Adversos Relaciona dos à Assistênci	Queda do paciente		
04/10/2022	CE	QUIXERA MOBIM	HOSPITA L REGIONA L DO SERTÃO	-	Enviada	Eventos Adversos Relaciona dos à Assistênci	Queda do paciente		
04/10/2022	CE	QUIXERA MOBIM	HOSPITA L REGIONA L DO SERTÃO	Maléolo lateral de MID.	Enviada	Eventos Adversos Relaciona dos à Assistênci	Lesão por pressão		
04/10/2022	CE	QUIXERA MOBIM	HOSPITA L REGIONA L DO SERTÃO	Região sacra.	Enviada	Eventos Adversos Relaciona dos à Assistênci	Lesão por pressão		
04/10/2022	CE	QUIXERA MOBIM	HOSPITA L REGIONA L DO SERTÃO	Região occipital.	Enviada	Eventos Adversos Relaciona dos à Assistênci	Lesão por pressão		
04/10/2022	CE	QUIXERA MOBIM	HOSPITA L REGIONA L DO SERTÃO	Nádegas.	Enviada	Eventos Adversos Relaciona dos à Assistênci	Lesão por pressão		
04/10/2022	CE	QUIXERA MOBIM	HOSPITA L REGIONA L DO SERTÃO	Região sacra.	Enviada	Eventos Adversos Relaciona dos à Assistênci	Lesão por pressão		

04/10/2022	CE	QUIXERA MOBIM	HOSPITA L REGIONA L DO SERTÃO	Região sacra.	Enviada	Eventos Adversos Relaciona dos à Assistênci	Lesão por pressão		
04/10/2022	CE	QUIXERA MOBIM	HOSPITA L REGIONA L DO SERTÃO	Região sacra.	Enviada	Eventos Adversos Relaciona dos à Assistênci	Lesão por pressão		
04/10/2022	CE	QUIXERA MOBIM	HOSPITA L REGIONA L DO SERTÃO	Calcâneo direito.	Enviada	Eventos Adversos Relaciona dos à Assistênci	Lesão por pressão		
04/10/2022	CE	QUIXERA MOBIM	HOSPITA L REGIONA L DO SERTÃO	Trocanter direito.	Enviada	Eventos Adversos Relaciona dos à Assistênci	Lesão por pressão		
04/10/2022	CE	QUIXERA MOBIM	HOSPITA L REGIONA L DO SERTÃO	Região sacra.	Enviada	Eventos Adversos Relaciona dos à Assistênci	Lesão por pressão		
04/10/2022	CE	QUIXERA MOBIM	HOSPITA L REGIONA L DO SERTÃO	Região sacra.	Enviada	Eventos Adversos Relaciona dos à Assistênci	Lesão por pressão		
04/10/2022	CE	QUIXERA MOBIM	HOSPITA L REGIONA L DO SERTÃO	Trocanter esquerdo.	Enviada	Eventos Adversos Relaciona dos à Assistênci	Lesão por pressão		
04/10/2022	CE	QUIXERA MOBIM	HOSPITA L REGIONA L DO SERTÃO	Região sacra.	Enviada	Eventos Adversos Relaciona dos à Assistênci	Lesão por pressão		
11/10/2022	CE	QUIXERA MOBIM	HOSPITA L REGIONA L DO SERTÃO	-	Enviada	Eventos Adversos Relaciona dos à Assistênci	Queda do paciente		
11/10/2022	CE	QUIXERA MOBIM	HOSPITA L REGIONA L DO SERTÃO	-	Enviada	Eventos Adversos Relaciona dos à Assistênci	Queda do paciente		
08/11/2022	CE	QUIXERA MOBIM	HOSPITA L REGIONA L DO SERTÃO	-	Enviada	Eventos Adversos Relaciona dos à Assistênci	Queda do paciente		
08/11/2022	CE	QUIXERA MOBIM	HOSPITA L REGIONA L DO SERTÃO	-	Enviada	Eventos Adversos Relaciona dos à Assistênci	Queda do paciente		

08/11/2022	CE	QUIXERA MOBIM	HOSPITA L REGIONA L DO SERTÃO	-	Enviada	Eventos Adversos Relaciona dos à Assistênci	Queda do paciente		
08/11/2022	CE	QUIXERA MOBIM	HOSPITA L REGIONA L DO SERTÃO	-	Enviada	Eventos Adversos Relaciona dos à Assistênci	Queda do paciente		
08/11/2022	CE	QUIXERA MOBIM	HOSPITA L REGIONA L DO SERTÃO	-	Enviada	Eventos Adversos Relaciona dos à Assistênci	Queda do paciente		
08/11/2022	CE	QUIXERA MOBIM	HOSPITA L REGIONA L DO SERTÃO	-	Enviada	Eventos Adversos Relaciona dos à Assistênci	Queda do paciente		
08/11/2022	CE	QUIXERA MOBIM	HOSPITA L REGIONA L DO SERTÃO	-	Enviada	Eventos Adversos Relaciona dos à Assistênci	Queda do paciente		
08/11/2022	CE	QUIXERA MOBIM	HOSPITA L REGIONA L DO SERTÃO	-	Enviada	Eventos Adversos Relaciona dos à Assistênci	Queda do paciente		
08/11/2022	CE	QUIXERA MOBIM	HOSPITA L REGIONA L DO SERTÃO	-	Enviada	Eventos Adversos Relaciona dos à Assistênci	Queda do paciente		
16/11/2022	CE	QUIXERA MOBIM	HOSPITA L REGIONA L DO SERTÃO	-	Enviada	Eventos Adversos Relaciona dos à Assistênci	Lesão por pressão		
16/11/2022	CE	QUIXERA MOBIM	HOSPITA L REGIONA L DO SERTÃO	-	Enviada	Eventos Adversos Relaciona dos à Assistênci	Lesão por pressão		
16/11/2022	CE	QUIXERA MOBIM	HOSPITA L REGIONA L DO SERTÃO	-	Enviada	Eventos Adversos Relaciona dos à Assistênci	Lesão por pressão		
16/11/2022	CE	QUIXERA MOBIM	HOSPITA L REGIONA L DO SERTÃO	-	Enviada	Eventos Adversos Relaciona dos à Assistênci	Lesão por pressão		
16/11/2022	CE	QUIXERA MOBIM	HOSPITA L REGIONA L DO SERTÃO	-	Enviada	Eventos Adversos Relaciona dos à Assistênci	Lesão por pressão		

16/11/2022	CE	QUIXERA MOBIM	HOSPITA L REGIONA L DO SERTÃO	-	Enviada	Eventos Adversos Relaciona dos à Assistênci	Lesão por pressão		
16/11/2022	CE	QUIXERA MOBIM	HOSPITA L REGIONA L DO SERTÃO	-	Enviada	Eventos Adversos Relaciona dos à Assistênci	Lesão por pressão		
16/11/2022	CE	QUIXERA MOBIM	HOSPITA L REGIONA L DO SERTÃO	-	Enviada	Eventos Adversos Relaciona dos à Assistênci	Lesão por pressão		
16/11/2022	CE	QUIXERA MOBIM	HOSPITA L REGIONA L DO SERTÃO	-	Enviada	Eventos Adversos Relaciona dos à Assistênci	Lesão por pressão		
16/11/2022	CE	QUIXERA MOBIM	HOSPITA L REGIONA L DO SERTÃO	-	Enviada	Eventos Adversos Relaciona dos à Assistênci	Queda do paciente		
16/11/2022	CE	QUIXERA MOBIM	HOSPITA L REGIONA L DO SERTÃO	-	Enviada	Eventos Adversos Relaciona dos à Assistênci	Queda do paciente		
16/11/2022	CE	QUIXERA MOBIM	HOSPITA L REGIONA L DO SERTÃO	-	Enviada	Eventos Adversos Relaciona dos à Assistênci	Queda do paciente		
23/11/2022	CE	QUIXERA MOBIM	HOSPITA L REGIONA L DO SERTÃO	-	Enviada	Eventos Adversos Relaciona dos à Assistênci	Queda do paciente		
25/11/2022	CE	QUIXERA MOBIM	HOSPITA L REGIONA L DO SERTÃO	-	Enviada	Eventos Adversos Relaciona dos à Assistênci	Queda do paciente		
29/11/2022	CE	QUIXERA MOBIM	HOSPITA L REGIONA L DO SERTÃO	-	Enviada	Eventos Adversos Relaciona dos à Assistênci	Queda do paciente		
01/12/2022	CE	QUIXERA MOBIM	HOSPITA L REGIONA L DO SERTÃO	-	Enviada	Eventos Adversos Relaciona dos à Assistênci	Queda do paciente		
12/12/2022	CE	QUIXERA MOBIM	HOSPITA L REGIONA L DO SERTÃO	-	Enviada	Eventos Adversos Relaciona dos à Assistênci	Queda do paciente		

12/12/2022	CE	QUIXERAMOBIM	HOSPITAL REGIONAL DO SERTÃO	-	Enviada	Eventos Adversos Relacionados à Assistênci	Queda do paciente		
13/12/2022	CE	QUIXERAMOBIM	HOSPITAL REGIONAL DO SERTÃO	Maléolo direito.	Enviada	Eventos Adversos Relacionados à Assistênci	Lesão por pressão		
13/12/2022	CE	QUIXERAMOBIM	HOSPITAL REGIONAL DO SERTÃO	Região sacra.	Enviada	Eventos Adversos Relacionados à Assistênci	Lesão por pressão		
13/12/2022	CE	QUIXERAMOBIM	HOSPITAL REGIONAL DO SERTÃO	Região sacra.	Enviada	Eventos Adversos Relacionados à Assistênci	Lesão por pressão		
13/12/2022	CE	QUIXERAMOBIM	HOSPITAL REGIONAL DO SERTÃO	5º pododáctilo esquerdo.	Enviada	Eventos Adversos Relacionados à Assistênci	Lesão por pressão		
13/12/2022	CE	QUIXERAMOBIM	HOSPITAL REGIONAL DO SERTÃO	Região sacra.	Enviada	Eventos Adversos Relacionados à Assistênci	Lesão por pressão		
13/12/2022	CE	QUIXERAMOBIM	HOSPITAL REGIONAL DO SERTÃO	Região sacra.	Enviada	Eventos Adversos Relacionados à Assistênci	Lesão por pressão		
13/12/2022	CE	QUIXERAMOBIM	HOSPITAL REGIONAL DO SERTÃO	Região sacra.	Enviada	Eventos Adversos Relacionados à Assistênci	Lesão por pressão		
14/12/2022	CE	QUIXERAMOBIM	HOSPITAL REGIONAL DO SERTÃO	-	Enviada	Eventos Adversos Relacionados à Assistênci	Queda do paciente		
26/12/2022	CE	QUIXERAMOBIM	HOSPITAL REGIONAL DO SERTÃO	-	Enviada	Eventos Adversos Relacionados à Assistênci	Queda do paciente		
27/12/2022	CE	QUIXERAMOBIM	HOSPITAL REGIONAL DO SERTÃO	-	Enviada	Eventos Adversos Relacionados à Assistênci	Queda do paciente		
05/01/2023	CE	QUIXERAMOBIM	HOSPITAL REGIONAL DO SERTÃO	Maléolo esquerdo.	Enviada	Eventos Adversos Relacionados à Assistênci	Lesão por pressão		

05/01/2023	CE	QUIXERAMOBIM	HOSPITAL REGIONAL DO SERTÃO	Região sacra.	Enviada	Eventos Adversos Relacionados à Assistênci	Lesão por pressão		
05/01/2023	CE	QUIXERAMOBIM	HOSPITAL REGIONAL DO SERTÃO	Trocanter direito.	Enviada	Eventos Adversos Relacionados à Assistênci	Lesão por pressão		
05/01/2023	CE	QUIXERAMOBIM	HOSPITAL REGIONAL DO SERTÃO	Calcâneo esquerdo.	Enviada	Eventos Adversos Relacionados à Assistênci	Lesão por pressão		
05/01/2023	CE	QUIXERAMOBIM	HOSPITAL REGIONAL DO SERTÃO	Região sacra.	Enviada	Eventos Adversos Relacionados à Assistênci	Lesão por pressão		
05/01/2023	CE	QUIXERAMOBIM	HOSPITAL REGIONAL DO SERTÃO	Trocanter direito.	Enviada	Eventos Adversos Relacionados à Assistênci	Lesão por pressão		
12/01/2023	CE	QUIXERAMOBIM	HOSPITAL REGIONAL DO SERTÃO	-	Enviada	Eventos Adversos Relacionados à Assistênci	Queda do paciente		
07/02/2023	CE	QUIXERAMOBIM	HOSPITAL REGIONAL DO SERTÃO	Região sacra.	Enviada	Eventos Adversos Relacionados à Assistênci	Lesão por pressão		
07/02/2023	CE	QUIXERAMOBIM	HOSPITAL REGIONAL DO SERTÃO	Interglúteo.	Enviada	Eventos Adversos Relacionados à Assistênci	Lesão por pressão		
07/02/2023	CE	QUIXERAMOBIM	HOSPITAL REGIONAL DO SERTÃO	Região sacra.	Enviada	Eventos Adversos Relacionados à Assistênci	Lesão por pressão		
07/02/2023	CE	QUIXERAMOBIM	HOSPITAL REGIONAL DO SERTÃO	Interglúteo.	Enviada	Eventos Adversos Relacionados à Assistênci	Lesão por pressão		
07/02/2023	CE	QUIXERAMOBIM	HOSPITAL REGIONAL DO SERTÃO	Região sacra.	Enviada	Eventos Adversos Relacionados à Assistênci	Lesão por pressão		
07/02/2023	CE	QUIXERAMOBIM	HOSPITAL REGIONAL DO SERTÃO	Região sacra.	Enviada	Eventos Adversos Relacionados à Assistênci	Lesão por pressão		

07/02/2023	CE	QUIXERAMOBIM	HOSPITAL REGIONAL DO SERTÃO	Região escapular direita.	Enviada	Eventos Adversos Relacionados à Assistênci	Lesão por pressão		
08/02/2023	CE	QUIXERAMOBIM	HOSPITAL REGIONAL DO SERTÃO	-	Enviada	Eventos Adversos Relacionados à Assistênci	Queda do paciente		
08/02/2023	CE	QUIXERAMOBIM	HOSPITAL REGIONAL DO SERTÃO	-	Enviada	Eventos Adversos Relacionados à Assistênci	Queda do paciente		
08/02/2023	CE	QUIXERAMOBIM	HOSPITAL REGIONAL DO SERTÃO	-	Enviada	Eventos Adversos Relacionados à Assistênci	Queda do paciente		
08/02/2023	CE	QUIXERAMOBIM	HOSPITAL REGIONAL DO SERTÃO	-	Enviada	Eventos Adversos Relacionados à Assistênci	Queda do paciente		
08/02/2023	CE	QUIXERAMOBIM	HOSPITAL REGIONAL DO SERTÃO	-	Enviada	Eventos Adversos Relacionados à Assistênci	Queda do paciente		
08/02/2023	CE	QUIXERAMOBIM	HOSPITAL REGIONAL DO SERTÃO	-	Enviada	Eventos Adversos Relacionados à Assistênci	Queda do paciente		
08/02/2023	CE	QUIXERAMOBIM	HOSPITAL REGIONAL DO SERTÃO	-	Enviada	Eventos Adversos Relacionados à Assistênci	Queda do paciente		
10/02/2023	CE	QUIXERAMOBIM	HOSPITAL REGIONAL DO SERTÃO	-	Enviada	Eventos Adversos Relacionados à Assistênci	Queda do paciente		
13/02/2023	CE	QUIXERAMOBIM	HOSPITAL REGIONAL DO SERTÃO	Perda de cateter de hemodiálise	Enviada	Eventos Adversos Relacionados à Assistênci	Falhas envolvendo o cateter venoso		
22/02/2023	CE	QUIXERAMOBIM	HOSPITAL REGIONAL DO SERTÃO	-	Enviada	Eventos Adversos Relacionados à Assistênci	Queda do paciente		
02/03/2023	CE	QUIXERAMOBIM	HOSPITAL REGIONAL DO SERTÃO	-	Enviada	Eventos Adversos Relacionados à Assistênci	Queda do paciente		

02/03/2023	CE	QUIXERA MOBIM	HOSPITA L REGIONA L DO SERTÃO	-	Enviada	Eventos Adversos Relaciona dos à Assistênci	Queda do paciente		
06/03/2023	CE	QUIXERA MOBIM	HOSPITA L REGIONA L DO SERTÃO	-	Enviada	Eventos Adversos Relaciona dos à Assistênci	Queda do paciente		
20/03/2023	CE	QUIXERA MOBIM	HOSPITA L REGIONA L DO SERTÃO	-	Enviada	Eventos Adversos Relaciona dos à Assistênci	Queda do paciente		
20/03/2023	CE	QUIXERA MOBIM	HOSPITA L REGIONA L DO SERTÃO	-	Enviada	Eventos Adversos Relaciona dos à Assistênci	Queda do paciente		
20/03/2023	CE	QUIXERA MOBIM	HOSPITA L REGIONA L DO SERTÃO	-	Enviada	Eventos Adversos Relaciona dos à Assistênci	Queda do paciente		
31/03/2023	CE	QUIXERA MOBIM	HOSPITA L REGIONA L DO SERTÃO	Calcâneo esquerdo.	Enviada	Eventos Adversos Relaciona dos à Assistênci	Lesão por pressão		
31/03/2023	CE	QUIXERA MOBIM	HOSPITA L REGIONA L DO SERTÃO	Calcâneo direito.	Enviada	Eventos Adversos Relaciona dos à Assistênci	Lesão por pressão		
31/03/2023	CE	QUIXERA MOBIM	HOSPITA L REGIONA L DO SERTÃO	-	Enviada	Eventos Adversos Relaciona dos à Assistênci	Lesão por pressão		
31/03/2023	CE	QUIXERA MOBIM	HOSPITA L REGIONA L DO SERTÃO	-	Enviada	Eventos Adversos Relaciona dos à Assistênci	Lesão por pressão		
31/03/2023	CE	QUIXERA MOBIM	HOSPITA L REGIONA L DO SERTÃO	-	Enviada	Eventos Adversos Relaciona dos à Assistênci	Lesão por pressão		
31/03/2023	CE	QUIXERA MOBIM	HOSPITA L REGIONA L DO SERTÃO	Região sacra.	Enviada	Eventos Adversos Relaciona dos à Assistênci	Lesão por pressão		
31/03/2023	CE	QUIXERA MOBIM	HOSPITA L REGIONA L DO SERTÃO	Região sacra.	Enviada	Eventos Adversos Relaciona dos à Assistênci	Lesão por pressão		

31/03/2023	CE	QUIXERAMOBIM	HOSPITAL REGIONAL DO SERTÃO	Região sacra.	Enviada	Eventos Adversos Relacionados à Assistênci	Lesão por pressão		

Processo envolvid	Documento envolvid	Problema ocorrido	Troca de nome dos	Falta de pulseiras de	Falta de identificação no	Que tipo de problema	Em que fase do processo	Tipo de queda	Queda envolvendo
								Queda da própria altura	Cama
			X						
Assistência geral		Incompleto / inadequad							
Administração		Paciente errado							
			X						
Administração		Paciente errado							
								Queda da própria altura	Cama
			X						
Administração		Paciente errado							
Procedimento / tratament		Procedimento / tratament							
Procedimento / tratament		Procedimento / tratament							
Assistência geral		Incompleto / inadequad							
Administração		Paciente errado							
								Queda da própria altura	Cama
								Queda da própria altura	Banheiro

								Desmaio	Quarto
								Queda da própria altura	Quarto
								Desmaio	Banheiro
								Queda da própria altura	Banheiro
								Escorregar	Banheiro
								Queda da própria altura	Banheiro
Assistência geral		Incompleto / inadequad							
Distribuição		Não administração							
Distribuição		Dieta errada							
Administração		Não administração							
Procedimento / tratament		Incompleto / inadequad							
								Queda da própria altura	Quarto
Assistência geral		Incompleto / inadequad							

Entrega		Quantidade errada							
Entrega		Não administração							
Entrega		Dieta errada							
		Lesão de órgão durante a							
								Queda da própria altura	Quarto
								Escorregar	Cama
Distribuição		Dieta errada							
		Abertura involuntária da							
				X					
								Queda da própria altura	Cama
Procedimento / tratament		Incompleto / inadequad							
Distribuição		Dieta errada							
Administração		Não administração							
Entrega		Dieta errada							

Administr ação		Não administra ção							
Administr ação		Não administra ção							
Assistênci a geral		Incomplet o / inadequad							
Assistênci a geral		Incomplet o / inadequad							
Diagnóstic o / Meios compleme		Incomplet o / inadequad							
Administr ação		Quantidad e errada							
Administr ação		Não administra ção							
Administr ação		Não administra ção							
Entrega		Não administra ção							
Distribuiç ão		Dieta errada							
Assistênci a geral		Incomplet o / inadequad							
Administr ação		Não administra ção							
Assistênci a geral		Incomplet o / inadequad							
Assistênci a geral		Incomplet o / inadequad							
Assistênci a geral		Incomplet o / inadequad							
Assistênci a geral		Incomplet o / inadequad							

Administração		Não administração							
Administração		Não administração							
								Queda da própria altura	Banheiro
								Queda da própria altura	Banheiro
Assistência geral		Incompleto / inadequad							
								Queda da própria altura	Quarto
								Perda do equilíbrio	Cama
								Queda da própria altura	Quarto
Administração		Paciente errado							
								Queda da própria altura	Quarto
								Queda da própria altura	Banheiro

								Escorregar	Banheiro
								Perda do equilíbrio	Quarto
								Queda da própria altura	Escadas / degraus
								Queda da própria altura	Cama
Administração		Paciente errado							

								Escorregar	Banheiro
								Escorregar	Cama
								Escorregar	Cama
								Escorregar	Quarto

								Escorregar	Cama
								Perda do equilíbrio	Cama
								Perda do equilíbrio	Quarto
								Escorregar	Enquanto transportado / apoiado
								Escorregar	Quarto
								Escorregar	Enquanto transportado / apoiado
								Escorregar	Quarto
								Tropeçar	Enquanto transportado / apoiado

								Escorregar	Cama
								Perda do equilíbrio	Quarto
								Desmaio	Banheiro
								Queda da própria altura	Enquanto transportado / apoiado
						Coleta errada	Fase pre-analítica (fase que se inicia		

Procedimento / tratamento /		Procedimento / tratamento /							
Assistência geral		Procedimento / tratamento /							
						Coleta errada	Fase pre-analítica (fase que se inicia		
Procedimento / tratamento /		Procedimento / tratamento /							
	Formulários	Informações ambígua / ilegível /							
							Perda do equilíbrio	Cama

Assistência geral		Procedimento / tratamento /							
		Hemorragia após a cirurgia							
Procedimento / tratamento /		Procedimento / tratamento /							
			X						
Procedimento / tratamento /		Não efetuado quando indicado							
Procedimento / tratamento /		Indisponível							
Procedimento / tratamento /		Procedimento / tratamento /							
Procedimento / tratamento /		Paciente errado							
Procedimento / tratamento /		Procedimento / tratamento /							

Administração		Não administração							
Administração		Não administração							
Assistência geral		Procedimento / tratamento /							
Assistência geral		Procedimento / tratamento /							
Assistência geral		Procedimento / tratamento /							
Assistência geral		Procedimento / tratamento /							
Assistência geral		Procedimento / tratamento /							
Administração		Não administração							
Diagnostico / Meios complementares de		Não efetuado quando indicado							
Assistência geral		Procedimento / tratamento /							

			X						
Procedimento / tratamento /		Incompleto / inadequado							
								Escorregar	Banheiro
			X						
			X						
						Óbito ou lesão grave de paciente	Fase pré-analítica (fase que se inicia		
								Escorregar	Quarto
		Lesão de órgão durante a cirurgia							
								Perda do equilíbrio	Banheiro
Prescrição		Não administração							

Distribuição		Frequência errada							
Distribuição		Frequência errada							
Procedimento / tratamento /		Incompleto / inadequado							
			X						

	Formulários	Documento em falta ou indisponív							
Diagnostico / Meios complementares de		Não efetuado quando indicado							
Procedimento / tratamento /		Incompleto / inadequado							
	Formulários	Documento em falta ou indisponív							

Procedimento / tratamento /		Incompleto / inadequado							
transferência de paciente		Incompleto / inadequado							
Procedimento / tratamento /		Procedimento / tratamento /							
			X						
			X						
								Perda do equilíbrio	Banheiro
Procedimento / tratamento /		Procedimento / tratamento /							
								Desmaio	Banheiro
Procedimento / tratamento /		Incompleto / inadequado							
transferência de paciente		Incompleto / inadequado							
	Requisições / pedidos	Atraso no acesso ao document							

Assistência geral		Procedimento / tratamento /							
		Lesão de órgão durante a cirurgia							
Triagem / check up		Incompleto / inadequado							
								Escorregar	Cama
Administração		Quantidade errada							
Procedimento / tratamento /		Procedimento / tratamento /							

Procedimento / tratamento /		Incompleto / inadequado							
Procedimento / tratamento /		Não efetuado quando indicado							
			X						
								Perda do equilíbrio	Cama
Prescrição		Não administração							
transferência de paciente		Incompleto / inadequado							
Regulação / referenciamento		Processo errado							
Distribuição		Não administração							
Assistência geral		Procedimento / tratamento /							
Procedimento / tratamento /		Procedimento / tratamento /							

Fornecimento		Quantidade errada							
Procedimento / tratamento /		Procedimento / tratamento /							
		Procedimento cirúrgico realizado							
Entrega		Não administração							
Procedimento / tratamento /		Incompleto / inadequado							
Diagnostico / Meios complementares de		Indisponível							
Alta		Processo errado							
Alta		Processo errado							
Resposta à emergência		Incompleto / inadequado							
Entrega		Frequência errada							
	Requisições / pedidos	Atraso no acesso ao document							

			X						
Procedimento / tratamento /		Não efetuado quando indicado							
Procedimento / tratamento /		Não efetuado quando indicado							
Assistência geral		Incompleto / inadequado							
transferência de paciente		Incompleto / inadequado							
								Escorregar	Cama
Procedimento / tratamento /		Incompleto / inadequado							
Administração		Não administração							
						Coleta errada	Fase pre-analítica (fase que se inicia		
Procedimento / tratamento /		Indisponível							
								Queda da própria altura	Cama

Procedimento / tratamento /		Incompleto / inadequado							
Procedimento / tratamento /		Incompleto / inadequado							
Procedimento / tratamento /		Incompleto / inadequado							
Procedimento / tratamento /		Incompleto / inadequado							

								Perda do equilíbrio	Quarto
Procedimento / tratamento /		Procedimento / tratamento /							
Procedimento / tratamento /		Procedimento / tratamento /							
Procedimento / tratamento /		Procedimento / tratamento /							
Fornecimento		Dieta errada							
Diagnostico / Meios complementares de		Incompleto / inadequado							
Assistência geral		Incompleto / inadequado							

Procedimento / tratamento /		Procedimento / tratamento /							
Procedimento / tratamento /		Nao efetuado quando indicado							
						Processamento errado	Fase pre-analítica (fase que se inicia		
Procedimento / tratamento /		Procedimento / tratamento /							
Procedimento / tratamento /		Incompleto / inadequado							
Procedimento / tratamento /		Incompleto / inadequado							
Procedimento / tratamento /		Incompleto / inadequado							

								Queda da própria altura	Cama
Procedimento / tratamento /		Não efetuado quando indicado							
								Perda do equilíbrio	Banheiro
Preparação		Não administração							

Procedimento / tratamento /		Procedimento / tratamento /							
Procedimento / tratamento /		Procedimento / tratamento /							
								Desmaio	Escadas / degraus
		Óbito intra-operatório ou							
Assistência geral		Incompleto / inadequado							

			X						
Assistênci a geral		Incomplet o / inadequad o							
Procedim ento / tratament o /		Incomplet o / inadequad o							
Procedim ento / tratament o /		Incomplet o / inadequad o							
Procedim ento / tratament o /		Procedim ento / tratament o /							

								Perda do equilíbrio	Banheiro
		Lesão de órgão durante a cirurgia							
		Lesão de órgão durante a cirurgia							
		Retenção não intencional de							

								Escorregar	Banheiro
					X				
Procedimento / tratamento /		Procedimento / tratamento /							
Assistência geral		Incompleto / inadequado							
			X						
Procedimento / tratamento /		Incompleto / inadequado							

								Desmaio	Cama
Assistência geral		Incompleto / inadequado							
Assistência geral		Procedimento / tratamento /							
Assistência geral		Procedimento / tratamento /							

	Instruções / informaçõ es /	Informaço es ambígua / ilegível /							
		Lesão de órgão durante a cirurgia							
								Queda da própria altura	Cama
		Hemorragia após a cirurgia							
Admissão		Não efetuado quando indicado							

Procedimento / tratamento /		Nao efetuado quando indicado							

					X				
Resposta à emergência		Não efetuado quando indicado							
								Queda da própria altura	Cama
								Perda do equilíbrio	Cama
			X						
Assistência geral		Incompleto / inadequado							
		Retenção não intencional de							
								Queda da própria altura	Cama

Procedimento / tratamento /		Procedimento / tratamento /							
Procedimento / tratamento /		Incompleto / inadequado							
Assistência geral		Incompleto / inadequado							

								Perda do equilíbrio	Cama
								Perda do equilíbrio	Banheiro
						Processa mento errado	Fase pre- analítica (fase que se inicia		
		Lesão de órgão durante a cirurgia							

								Perda do equilíbrio	Cama
Procedimento / tratamento / [illegible]		Incompleto / inadequado							
Assistência geral		Incompleto / inadequado							
								Escorregar	Banheiro
								Queda da própria altura	Quarto

Procedim ento / tratament o /		Não efetuado quando indicado							
Diagnostic o / Meios compleme ntares de		Não efetuado quando indicado							
						Processa mento errado	Fase pre-analítica (fase que se inicia		
Assistênci a geral		Incomplet o / inadequad o							
Assistênci a geral		Procedim ento / tratament o /							
Procedim ento / tratament o /		Incomplet o / inadequad o							
Procedim ento / tratament o /		Incomplet o / inadequad o							
Procedim ento / tratament o /		Incomplet o / inadequad o							

Procedimento / tratamento /		Incompleto / inadequado							
								Queda da própria altura	Cama
Procedimento / tratamento /		Paciente errado							
								Queda da própria altura	Escadas / degraus
								Perda do equilíbrio	Cama
								Desmaio	Banheiro
								Perda do equilíbrio	Banheiro
					X				

Administração		Quantidade errada							
Assistência geral		Incompleto / inadequado							
Diagnostico / Meios complementares de		Incompleto / inadequado							
Procedimento / tratamento /		Não efetuado quando indicado							
Assistência geral		Incompleto / inadequado							
Administração		Paciente errado							
Procedimento / tratamento /		Incompleto / inadequado							
								Queda da própria altura	Cama

Distribuição		Quantidade errada							
Procedimento / tratamento /		Incompleto / inadequado							

								Queda da própria altura	Cama
								Queda da própria altura	Equipamento terapêutico /
								Queda da própria altura	Cama
			X						

								Escorregar	Cama
								Perda do equilíbrio	Banheiro
Procedimento / tratamento / [illegible]		Incompleto / inadequado							
			X						

		Lesão de órgão durante a cirurgia							
								Perda do equilíbrio	Banheiro

Procedimento / tratamento /		Procedimento / tratamento /							
						Resultado errado	Fase pre-analítica (fase que se inicia		
Diagnostico / Meios complementares de		Incompleto / inadequado							
Prescrição		Não administração							

Prescrição		Não administração							
								Queda da própria altura	Quarto
								Escorregar	Maca
								Escorregar	Banheiro
Procedimento / tratamento /		Incompleto / inadequado							
Procedimento / tratamento /		Incompleto / inadequado							
								Escorregar	Maca
								Escorregar	Quarto
								Queda da própria altura	Quarto
								Queda da própria altura	Banheiro
								Queda da própria altura	Quarto
								Escorregar	Maca
								Perda do equilíbrio	Banheiro

Procedim ento / tratament o /		Incomplet o / inadequad o							
								Escorrega r	Banheiro
								Desmaio	Enquanto transporta do / apoiado
								Queda da própria altura	Quarto
Procedim ento / tratament o /		Procedim ento / tratament o /							
Procedim ento / tratament o /		Incomplet o / inadequad o							
								Escorrega r	Maca
								Queda da própria altura	Banheiro
								Queda da própria altura	Cama
								Queda da própria altura	Cadeira
		Lesão de órgão durante a cirurgia							
								Queda da própria altura	Banheiro
								Perda do equilíbrio	Cama
		Procedim ento cirúrgico realizado							

Procedim ento / tratament o /		Incomplet o / inadequad o							
		Lesão de órgão durante a cirurgia							
Procedim ento / tratament o /		Paciente errado							
								Queda da própria altura	Quarto
								Perda do equilíbrio	Cadeira
								Queda da própria altura	Banheiro
								Escorrega r	Banheiro
								Perda do equilíbrio	Banheiro
								Queda da própria altura	Banheiro
								Escorrega r	Cama
								Escorrega r	Cama
								Escorrega r	Maca
								Tropeçar	Banheiro
								Escorrega r	Cama

								Escorregar	Maca
								Escorregar	Cadeira
								Escorregar	Maca
								Queda da própria altura	Banheiro
								Perda do equilíbrio	Cama
								Perda do equilíbrio	Cama
								Perda do equilíbrio	Cama
								Queda da própria altura	Cadeira
								Desmaio	Cadeira
								Escorregar	Cama
								Perda do equilíbrio	Banheiro
								Escorregar	Cama
								Escorregar	Quarto
								Desmaio	Maca
								Perda do equilíbrio	Maca

								Desmaio	Cadeira
								Escorregar	Maca
Procedimento / tratamento /		Procedimento / tratamento /							
								Escorregar	Maca
	Listas de verificação	Documento entregue para							
								Queda da própria altura	Banheiro
	Formulários	Documento entregue para							
								Escorregar	Banheiro
								Escorregar	Cama
								Escorregar	Banheiro
Procedimento / tratamento /		Procedimento / tratamento /							
								Escorregar	Cadeira
								Escorregar	Cama
								Queda da própria altura	Banheiro

								Queda da própria altura	Maca
								Queda da própria altura	Maca
								Queda da própria altura	Maca
								Tropeçar	Banheiro
								Queda da própria altura	Cadeira
								Queda da própria altura	Cama
								Queda da própria altura	Banheiro

								Queda da própria altura	Maca
								Escorregar	Cama
								Escorregar	Cama
								Queda da própria altura	Cadeira
								Queda da própria altura	Cama
								Escorregar	Banheiro
								Queda da própria altura	Maca
								Queda da própria altura	Cadeira

								Perda do equilíbrio	Banheiro
								Queda da própria altura	Banheiro
Procedimento / tratamento /		Procedimento / tratamento /							
								Desmaio	Banheiro
Procedimento / tratamento /		Incompleto / inadequado							
Procedimento / tratamento /		Incompleto / inadequado							
Procedimento / tratamento /		Procedimento / tratamento /							
								Queda da própria altura	Banheiro

	Instruções / informaçõ es /	Document o entregue para							
Procedim ento / tratament o /		Não efetuado quando indicado							
			X						
								Escorregar	Banheiro
								Perda do equilíbrio	Cadeira
								Escorregar	Cadeira
								Queda da própria altura	Banheiro
								Escorregar	Maca
								Escorregar	Maca
								Escorregar	Banheiro
								Escorregar	Banheiro
								Queda da própria altura	Banheiro
								Perda do equilíbrio	Cama
								Queda da própria altura	Maca
								Queda da própria altura	Banheiro

								Escorregar	Cama
								Queda da própria altura	Banheiro
								Queda da própria altura	Cadeira
				X					
Assistência geral		Procedimento / tratamento /							
								Queda da própria altura	Banheiro
								Escorregar	Banheiro
								Perda do equilíbrio	Cama
Procedimento / tratamento /		Procedimento / tratamento /							
								Perda do equilíbrio	Banheiro
								Queda da própria altura	Cama
								Perda do equilíbrio	Cama
Assistência geral		Procedimento / tratamento /							

								Perda do equilíbrio	Cama
								Perda do equilíbrio	Maca
								Perda do equilíbrio	Cama
								Escorregar	Banheiro
Assistência geral		Procedimento / tratamento /							

								Perda do equilíbrio	Maca
								Perda do equilíbrio	Cadeira

								Escorregar	Banheiro

								Escorregar	Cama
								Perda do equilíbrio	Cama
								Queda da própria altura	Quarto
								Queda da própria altura	Cama
Procedimento / tratamento /		Procedimento / tratamento /							

								Escorregar	Banheiro
								Desmaio	Banheiro
Assistência geral		Paciente errado							
								Perda do equilíbrio	Banheiro
								Perda do equilíbrio	Cadeira
								Escorregar	Maca
								Escorregar	Maca

								Escorregar	Maca

								Escorregar	Cama
								Desmaio	Banheiro

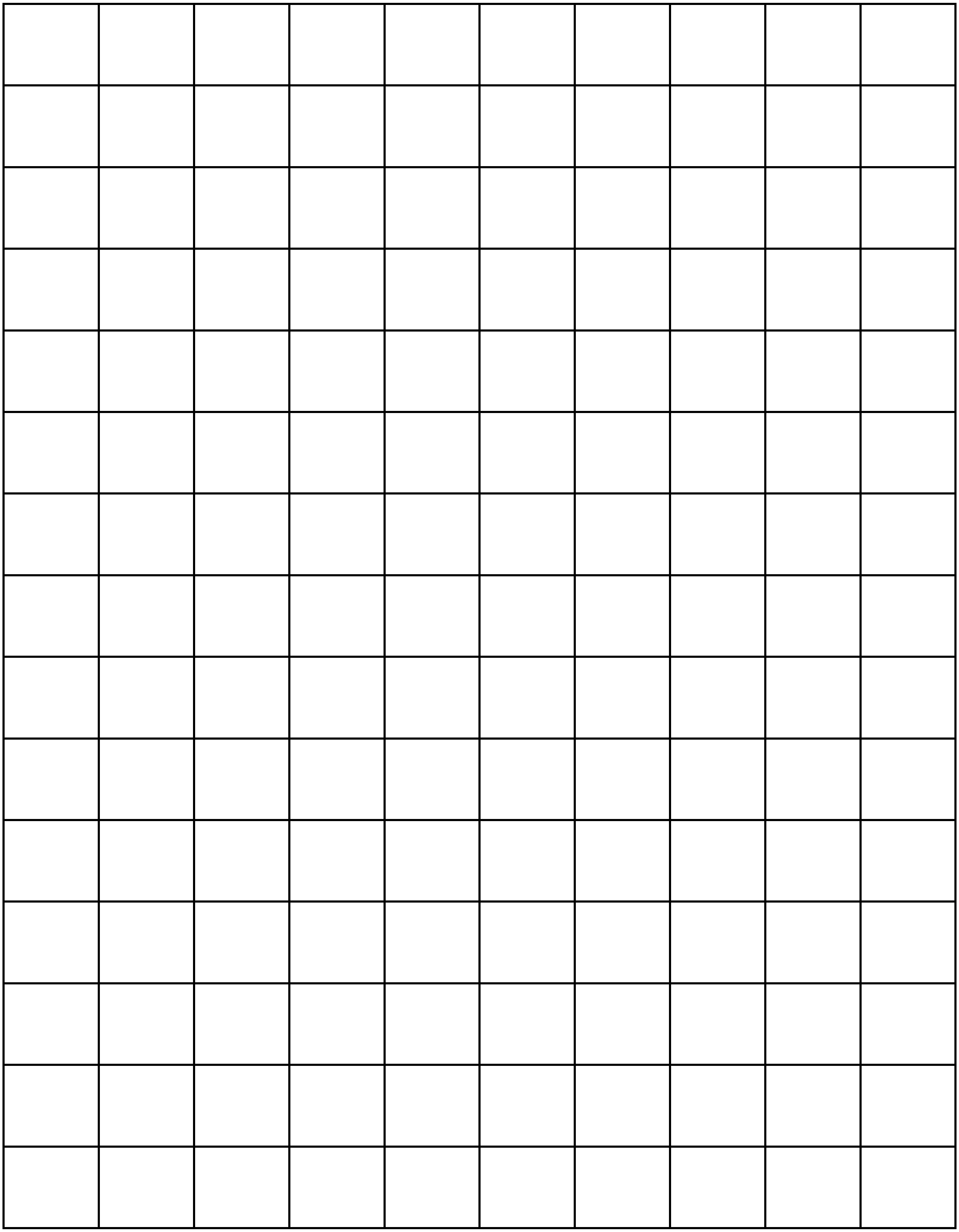

								Queda da própria altura	Banheiro
								Escorregar	Banheiro
								Perda do equilíbrio	Banheiro
								Escorregar	Banheiro
								Escorregar	Banheiro
								Escorregar	Cadeira
								Escorregar	Banheiro
								Escorregar	Cadeira
Procedimento / tratamento /		Não efetuado quando indicado							
								Escorregar	Cama

								Escorregar	Cama

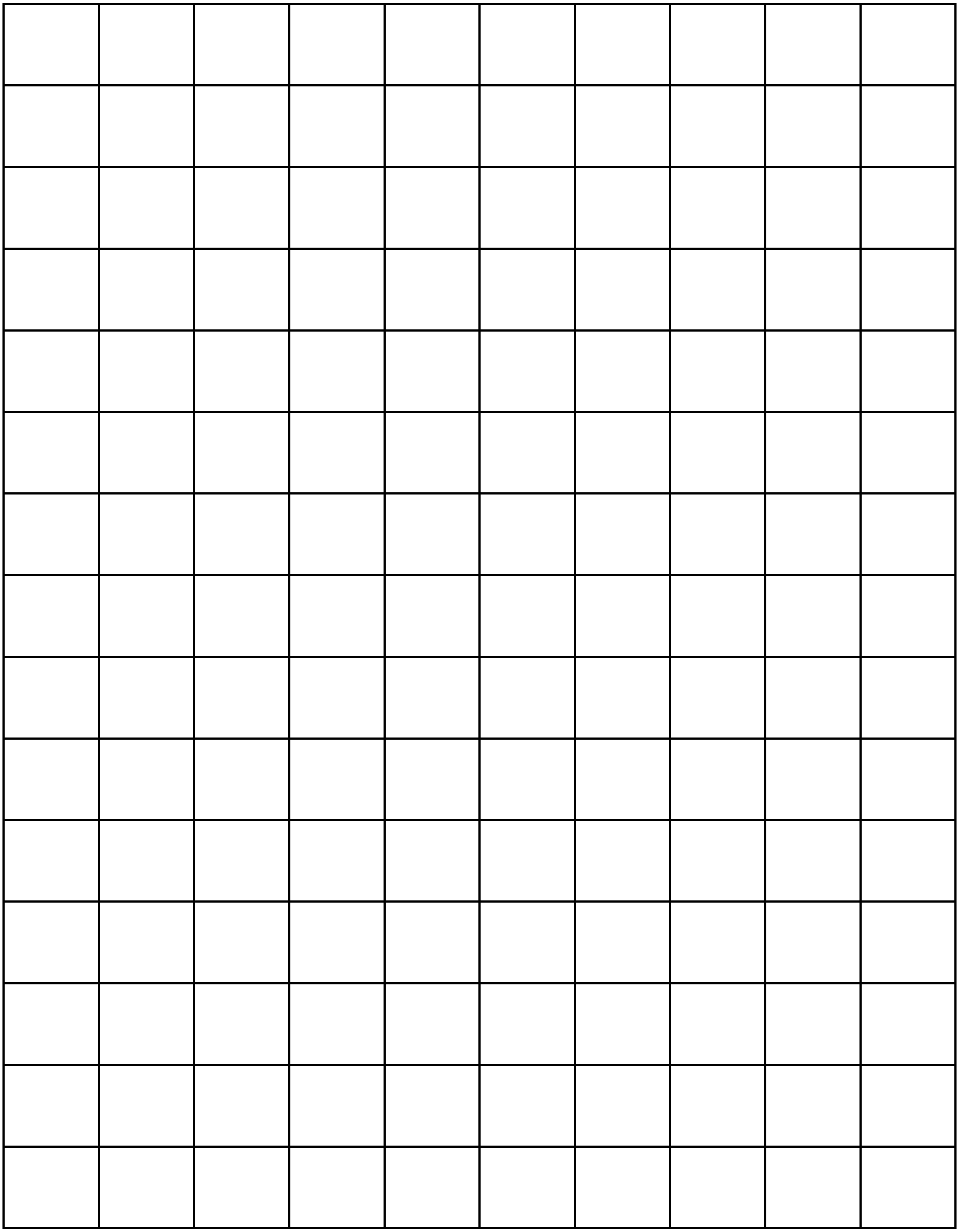

								Queda da própria altura	Maca
								Queda da própria altura	Maca
								Queda da própria altura	Banheiro
Assistência geral		Procedim ento / tratament o /							
transferência de paciente		Processo errado							
Procedim ento / tratament o /		Procedim ento / tratament o /							
								Perda do equilíbrio	Cadeira

Assistência geral		Paciente errado							
								Escorregar	Cama
								Escorregar	Cama
								Perda do equilíbrio	Banheiro
Assistência geral		Procedimento / tratamento /							
								Escorregar	Maca

Diagnostico / Meios complementares de		Não efetuado quando indicado							
Procedimento / tratamento /		Paciente errado							
Procedimento / tratamento /		Incompleto / inadequado							
Procedimento / tratamento /		Incompleto / inadequado							
Procedimento / tratamento /		Incompleto / inadequado							
Procedimento / tratamento /		Incompleto / inadequado							
Procedimento / tratamento /		Incompleto / inadequado							
								Perda do equilíbrio	Cama
								Escorregar	Cama
								Escorregar	Cadeira
								Escorregar	Equipamento terapêutico /
								Perda do equilíbrio	Cama

Procedimento / tratamento /		Paciente errado							
								Queda da própria altura	Cama
Procedimento / tratamento /		Procedimento / tratamento /							

								Queda da própria altura	Maca
								Desmaio	Banheiro
Procedimento / tratamento /		Paciente errado							
Procedimento / tratamento /		Não efetuado quando indicado							
Assistência geral		Procedimento / tratamento /							
Assistência geral		Procedimento / tratamento /							

Procedimento / tratamento /		Incompleto / inadequado							
								Perda do equilíbrio	Cadeira
Assistência geral		Paciente errado							
								Escorregar	Quarto
								Perda do equilíbrio	Banheiro
Procedimento / tratamento /		Procedimento / tratamento /							
								Escorregar	Cama
Procedimento / tratamento /		Procedimento / tratamento /							

								Queda da própria altura	Banheiro
								Queda da própria altura	Banheiro
								Perda do equilíbrio	Banheiro
Procedimento / tratamento /		Procedimento / tratamento /							
								Perda do equilíbrio	Cama
Assistência geral		Incompleto / inadequado							
								Queda da própria altura	Banheiro
								Escorregar	Cama

Procedimento / tratamento /		Incompleto / inadequado							
Procedimento / tratamento /		Incompleto / inadequado							
Assistência geral		Incompleto / inadequado							
								Perda do equilíbrio	Cama
Procedimento / tratamento /		Incompleto / inadequado							
Procedimento / tratamento /		Incompleto / inadequado							
Procedimento / tratamento /		Incompleto / inadequado							
Assistência geral		Incompleto / inadequado							

Procedim ento / tratament o /		Nao efetuado quando indicado							
Assistênci a geral		Incomplet o / inadequad o							
Procedim ento / tratament o /		Incomplet o / inadequad o							
								Perda do equilíbrio	Cama
Procedim ento / tratament o /		Nao efetuado quando indicado							

Assistência geral		Incompleto / inadequado							

Diagnostico / Meios complementares de		Incompleto / inadequado							
Diagnostico / Meios complementares de		Procedimento / tratamento /							
Procedimento / tratamento /		Incompleto / inadequado							
								Tropeçar	Quarto
								Perda do equilíbrio	Quarto
Procedimento / tratamento /		Incompleto / inadequado							
Procedimento / tratamento /		Incompleto / inadequado							

Procedimento / tratamento /		Incompleto / inadequado							
Procedimento / tratamento /		Paciente errado							
Procedimento / tratamento /		Incompleto / inadequado							
								Tropeçar	Quarto

								Perda do equilíbrio	Enquanto transportado / apoiado

								Perda do equilíbrio	Cama
								Escorregar	Banheiro
Procedimento / tratamento /		Nao efetuado quando indicado							
Administração		Paciente errado							
			X						

Procedim ento / tratament o /		Incomplet o / inadequad o							
								Perda do equilíbrio	Banheiro
Diagnostic o / Meios compleme ntares de		Incomplet o / inadequad o							
								Perda do equilíbrio	Maca
								Perda do equilíbrio	Maca
								Perda do equilíbrio	Maca

								Tropeçar	Quarto

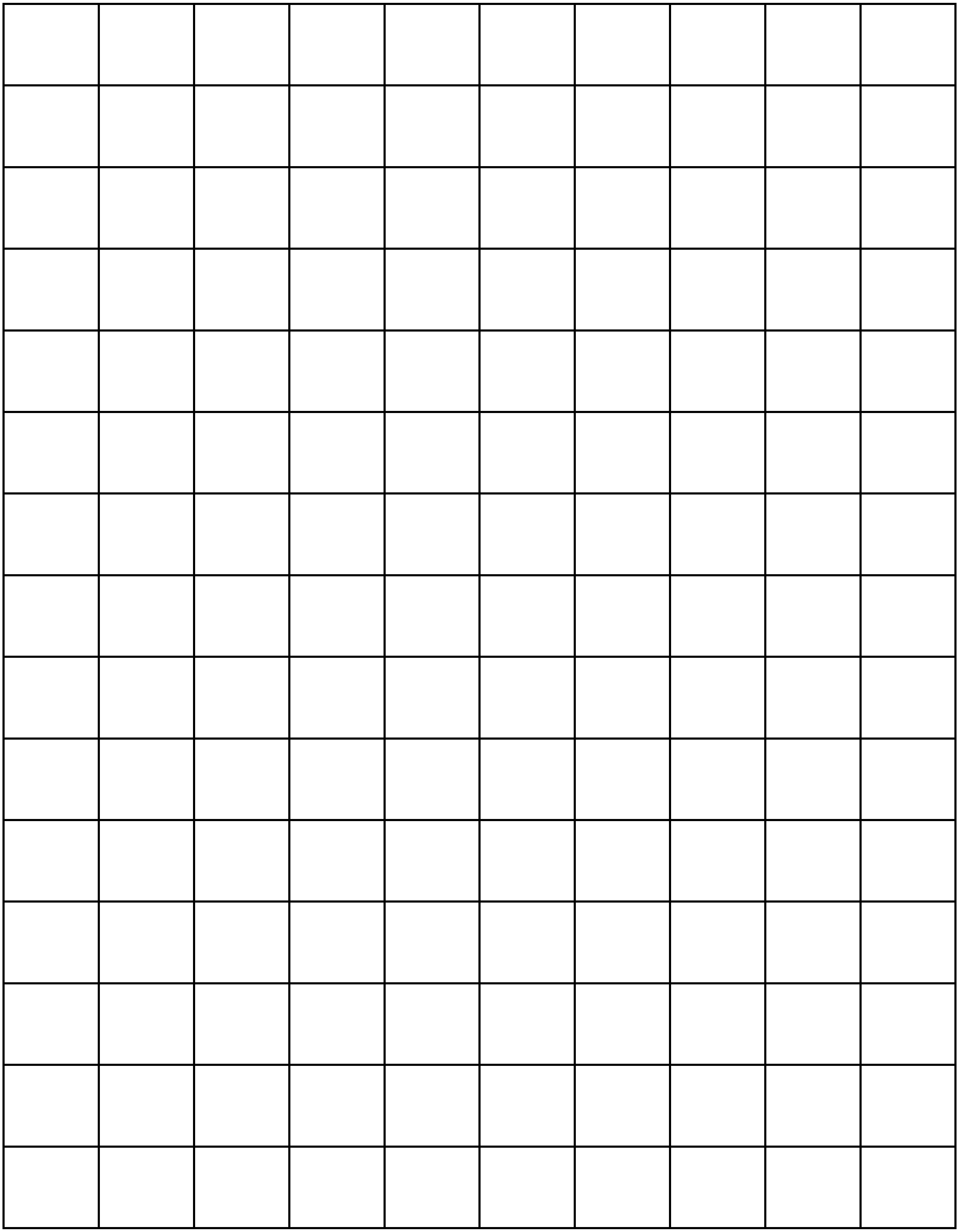

								Queda da própria altura	Cama
Procedimento / tratamento /		Incompleto / inadequado							
Procedimento / tratamento /		Paciente errado							
								Queda da própria altura	Quarto

								Perda do equilíbrio	Maca
								Perda do equilíbrio	Banheiro
Procedimento / tratamento / [illegible]		Incompleto / inadequado							
								Perda do equilíbrio	Cama
	Relatorios / resultados / imagens [illegible]	Informaçoes ambígua / ilegível / [illegible]							
								Perda do equilíbrio	Cama

			X						
								Perda do equilíbrio	Cama
Procedimento / tratamento /		Procedimento / tratamento /							
								Perda do equilíbrio	Cama

Procedimento / tratamento /		Procedimento / tratamento /							

								Perda do equilíbrio	Cama
								Perda do equilíbrio	Cama
								Perda do equilíbrio	Cama
								Queda da própria altura	Cama

Procedimento / tratamento /		Procedimento / tratamento /							

								Perda do equilíbrio	Cama
Procedimento / tratamento /		Incompleto / inadequado							
Procedimento / tratamento /		Incompleto / inadequado							
Procedimento / tratamento /		Procedimento / tratamento /							
								Perda do equilíbrio	Maca
Procedimento / tratamento /		Incompleto / inadequado							
Procedimento / tratamento /		Incompleto / inadequado							
Procedimento / tratamento /		Incompleto / inadequado							

Procedimento / tratamento /		Procedimento / tratamento /							
Assistência geral		Não efetuado quando indicado							
								Perda do equilíbrio	Maca
								Escorregar	Cama
Assistência geral		Incompleto / inadequado							
Procedimento / tratamento /		Não efetuado quando indicado							
								Perda do equilíbrio	Cama
								Perda do equilíbrio	Cama
								Perda do equilíbrio	Quarto
Procedimento / tratamento /		Incompleto / inadequado							

								Queda da própria altura	Cama
Procedimento / tratamento /		Incompleto / inadequado							

								Escorregar	Banheiro
								Perda do equilíbrio	Quarto
								Escorregar	Banheiro
								Escorregar	Maca
								Perda do equilíbrio	Banheiro
Procedimento / tratamento /		Não efetuado quando indicado							
Procedimento / tratamento /		Paciente errado							
								Perda do equilíbrio	Cama

Procedimento / tratamento /		Não efetuado quando indicado							
Procedimento / tratamento /		Incompleto / inadequado							
								Perda do equilíbrio	Cama
								Perda do equilíbrio	Cama
								Perda do equilíbrio	Enquanto transportado / apoiado

Procedimento / tratamento /		Não efetuado quando indicado							
								Perda do equilíbrio	Quarto
Assistência geral		Incompleto / inadequado							

								Perda do equilíbrio	Cama
								Perda do equilíbrio	Quarto
Procedimento / tratamento / [illegible]		Não efetuado quando indicado							
								Perda do equilíbrio	Cama
								Queda da própria altura	Cama
Procedimento / tratamento / [illegible]		Paciente errado							
								Queda da própria altura	Quarto
								Perda do equilíbrio	Quarto
								Perda do equilíbrio	Cama
								Perda do equilíbrio	Quarto

								Perda do equilíbrio	Cama
Assistência geral		Incompleto / inadequado							
								Queda da própria altura	Cadeira
								Escorregar	Berço
								Perda do equilíbrio	Quarto
								Perda do equilíbrio	Cama
								Queda da própria altura	Banheiro
Assistência geral		Incompleto / inadequado							

Assistência geral		Incompleto / inadequado							
		Hemorragia após a cirurgia							

Assistência geral		Não efetuado quando indicado							
Procedimento / tratamento /		Incompleto / inadequado							
								Escorregar	Cama

								Perda do equilíbrio	Enquanto transportado / apoiado
Procedimento / tratamento /		Não efetuado quando indicado							
Diagnostico / Meios complementares de		Incompleto / inadequado							
								Queda da própria altura	Banheiro
								Perda do equilíbrio	Banheiro
Procedimento / tratamento /		Incompleto / inadequado							

								Queda da própria altura	Cama
Procedimento / tratamento /		Procedimento / tratamento /							
Assistência geral		Procedimento / tratamento /							
								Queda da própria altura	Banheiro
								Queda da própria altura	Banheiro
								Escorregar	Cama
Procedimento / tratamento /		Procedimento / tratamento /							
								Queda da própria altura	Banheiro
Assistência geral		Procedimento / tratamento /							
								Queda da própria altura	Quarto
								Escorregar	Banheiro

			X						
Procedim ento / tratament o /		Procedim ento / tratament o /							
Assistênci a geral		Procedim ento / tratament o /							
								Escorrega r	Berço
								Escorrega r	Banheiro
								Perda do equilíbrio	Banheiro
								Queda da própria altura	Quarto
								Queda da própria altura	Quarto
Procedim ento / tratament o /		Nao efetuado quando indicado							

								Escorregar	Banheiro
								Escorregar	Banheiro
Procedimento / tratamento /		Procedimento / tratamento /							
Procedimento / tratamento /		Nao efetuado quando indicado							

								Escorregar	Maca
								Escorregar	Maca
								Perda do equilíbrio	Cama

Procedimento / tratamento /		Incompleto / inadequado							
Procedimento / tratamento /		Procedimento / tratamento /							
								Perda do equilíbrio	Cama
transferência de paciente		Incompleto / inadequado							
Distribuição		Não administração							
			X						

								Queda da própria altura	Quarto

								Escorregar	Banheiro

Procedim ento / tratament o /		Nao efetuado quando indicado							

								Escorregar	Cama
								Perda do equilíbrio	Cama
								Escorregar	Banheiro

								Queda da própria altura	Banheiro
Assistência geral		Incompleto / inadequado							
								Escorregar	Banheiro
Assistência geral		Incompleto / inadequado							
								Escorregar	Cadeira
Assistência geral		Nao efetuado quando indicado							

								Escorrega r	Cama
Assistênci a geral		Incomplet o / inadequad o							
Assistênci a geral		Incomplet o / inadequad o							
Assistênci a geral		Incomplet o / inadequad o							
Assistênci a geral		Incomplet o / inadequad o							

								Queda da própria altura	Banheiro
								Escorregar	Cama
Procedimento / tratamento /		Incompleto / inadequado							

								Escorrega r	Cama
								Queda da própria altura	Quarto

								Perda do equilíbrio	Quarto

								Escorregar	Banheiro
			X						
								Escorregar	Banheiro
								Queda da própria altura	Quarto
								Escorregar	Cama
								Escorregar	Cama
								Desmaio	Banheiro
								Desmaio	Banheiro

								Queda da própria altura	Cama
								Escorrega r	Banheiro
								Perda do equilíbrio	Banheiro

								Escorregar	Banheiro
								Escorregar	Maca
								Perda do equilíbrio	Banheiro
								Perda do equilíbrio	Banheiro
								Queda da própria altura	Quarto

Procedimento / tratamento /		Procedimento / tratamento /							
								Escorregar	Cama
Assistência geral		Paciente errado							
Procedimento / tratamento /		Incompleto / inadequado							

								Desmaio	Banheiro

								Queda da própria altura	Banheiro
								Queda da própria altura	Cama
								Queda da própria altura	Maca

								Queda da própria altura	Cama

								Queda da própria altura	Banheiro
								Queda da própria altura	Banheiro
								Queda da própria altura	Cadeira
								Queda da própria altura	Cama

Assistência geral		Incompleto / inadequado							

Procedimento / tratamento /		Procedimento / tratamento /							
Marcação		Nao efetuado quando indicado							

transferência de paciente		Processo errado							
Procedimento / tratamento /		Procedimento / tratamento /							
Procedimento / tratamento /		Incompleto / inadequado							
Procedimento / tratamento /		Incompleto / inadequado							
Procedimento / tratamento /		Incompleto / inadequado							

Procedimento / tratamento / [illegible]		Nao efetuado quando indicado							

Triagem / check up		Incomplet o / inadequad o							

Assistência geral		Nao efetuado quando indicado							

								Queda da própria altura	Cama
Administração		Local errado							

								Queda da própria altura	Cama
Assistência geral		Incompleto / inadequado							
								Queda da própria altura	Cadeira
								Queda da própria altura	Banheiro
								Escorregar	Banheiro
Procedimento / tratamento /		Procedimento / tratamento /							

			X						
Assistência geral		Indisponível							
							Queda da própria altura	Quarto

Assistência geral		Procedimento / tratamento /							
								Perda do equilíbrio	Cama
								Perda do equilíbrio	Cadeira

								Escorregar	Quarto

								Queda da própria altura	Cama
								Perda do equilíbrio	Banheiro
								Perda do equilíbrio	Cama
								Perda do equilíbrio	Cama

								Queda da própria altura	Quarto
								Perda do equilíbrio	Berço
								Queda da própria altura	Cama

Assistência geral		Não efetuado quando indicado							

								Perda do equilíbrio	Cama
								Tropeçar	Banheiro

								Perda do equilíbrio	Berço
								Perda do equilíbrio	Cama
								Tropeçar	Cama

								Perda do equilíbrio	Cama
								Queda da própria altura	Quarto
								Queda da própria altura	Quarto
								Perda do equilíbrio	Cama

								Escorregar	Banheiro

								Perda do equilíbrio	Escadas / degraus
								Escorregar	Banheiro
								Perda do equilíbrio	Cama

								Perda do equilíbrio	Cama

								Escorregar	Banheiro
								Escorregar	Banheiro
								Escorregar	Banheiro
						Coleta errada	Fase analítica (conjunto de		

								Perda do equilíbrio	Banheiro
								Escorrega r	Banheiro
								Queda da própria altura	Quarto
								Queda da própria altura	Banheiro

Procedim ento / tratament o /		Procedim ento / tratament o /							
								Escorrega r	Cama
Procedim ento / tratament o /		Procedim ento / tratament o /							
Diagnostic o / Meios compleme ntares de		Incomplet o / inadequad o							
			X						
			X						

		Procedimento cirúrgico realizado							
						Paciente errado	Fase pre-analítica (fase que se inicia		
						Paciente errado	Fase pre-analítica (fase que se inicia		
Identificação do paciente		Incompleto / inadequado							
								Queda da própria altura	Cama
	Relatorios / resultados / imagens	Informaçoes ambígua / ilegível /							
Diagnostico / Meios complementares de		Nao efetuado quando indicado							

		Alta ou liberação de paciente							
transferência de paciente		Incompleto / inadequado							
Procedimento / tratamento /		Incompleto / inadequado							
Assistência geral		Não efetuado quando indicado							
Preparação		Dieta errada							
Procedimento / tratamento /		Procedimento / tratamento /							
						Processamento errado	Fase pre-analítica (fase que se inicia		
Admissão		Não efetuado quando indicado							

Assistência geral		Incompleto / inadequado							
transferência de paciente		Processo errado							
Procedimento / tratamento /		Nao efetuado quando indicado							
						Processamento errado	Fase pre-analítica (fase que se inicia		
						Processamento errado	Fase pre-analítica (fase que se inicia		
Procedimento / tratamento /		Procedimento / tratamento /							
Regulaçao / referenciamento		Processo errado							
Identificação do paciente		Nao efetuado quando indicado							
Procedimento / tratamento /		Nao efetuado quando indicado							
						Processamento errado	Fase pre-analítica (fase que se inicia		
			X						

Procedimento / tratamento /		Nao efetuado quando indicado							
Assistência geral		Procedimento / tratamento /							
Identificação do paciente		Processo errado							
Identificação do paciente		Incompleto / inadequado							
Procedimento / tratamento /		Incompleto / inadequado							
						Resultado errado	Fase pos-analítica (fase que se inicia		
Procedimento / tratamento /		Procedimento / tratamento /							
Identificação do paciente		Processo errado							
Identificação do paciente		Paciente errado							
Assistência geral		Incompleto / inadequado							
				X					
Diagnostico / Meios complementares de		Incompleto / inadequado							

Procedimento / tratamento /		Incompleto / inadequado							
Assistência geral		Incompleto / inadequado							
Procedimento / tratamento /		Procedimento / tratamento /							
								Escorregar	Cama
		Retenção não intencional de							
								Perda do equilíbrio	Cama
						Coleta errada	Fase pré-analítica (fase que se inicia com a solicitaçã		
Procedimento / tratamento / intervençã o		Procedimento / tratamento / intervenção errada							
								Desmaio	Maca
								Queda da própria altura	Quarto

								Perda do equilíbrio	Maca
								Perda do equilíbrio	Quarto
								Desmaio	Cadeira
								Queda da própria altura	Cadeira
								Queda da própria altura	Banheiro
								Perda do equilíbrio	Maca
								Queda da própria altura	Banheiro
								Queda da própria altura	Cama
								Queda da própria altura	Maca
								Queda da própria altura	Maca

								Queda da própria altura	Maca
								Queda da própria altura	Maca
								Queda da própria altura	Cama
								Queda da própria altura	Banheiro
								Queda da própria altura	Cama
								Escorregar	Banheiro

Assistênci a geral		Incomplet o / inadequad o							
								Tropeçar	Cama
								Perda do equilíbrio	Cama
								Perda do equilíbrio	Cadeira
Identificaç ão do paciente		Indisponív el							
Assistênci a geral		Incomplet o / inadequad o							

			X						
			X						
			X						
			X						
			X						
			X						
Administração		Não administração							

								Queda da própria altura	Maca
								Queda da própria altura	Banheiro
								Queda da própria altura	Banheiro
								Queda da própria altura	Maca
								Queda da própria altura	Cama

								Perda do equilíbrio	Cama
								Queda da própria altura	Cama
								Queda da própria altura	Enquanto transportado / apoiado por outro

Assistência geral		Incompleto / inadequado							
Assistência geral		Incompleto / inadequado							
Assistência geral		Incompleto / inadequado							
Procedimento / tratamento / intervençã		Incompleto / inadequado							
								Queda da própria altura	Cama
								Escorregar	Maca
								Queda da própria altura	Cama

								Queda da própria altura	Cama
								Queda da própria altura	Cama
								Desmaio	Banheiro
								Escorregar	Cadeira
								Queda da própria altura	Cadeira
Procedimento / tratamento / intervençã		Incompleto / inadequado							
Procedimento / tratamento / intervençã		Incompleto / inadequado							

Preparação		Dieta errada							
								Tropeçar	Cama
								Escorregar	Banheiro
								Queda da própria altura	Berço
								Escorregar	Banheiro
								Escorregar	Cama
Assistência geral		Procedimento / tratamento / intervençã[illegible]							
								Perda do equilíbrio	Banheiro
								Perda do equilíbrio	Cama

								Perda do equilíbrio	Cama

								Queda da própria altura	Banheiro
								Escorregar	Cama
								Escorregar	Banheiro

								Queda da própria altura	Cama
								Perda do equilíbrio	Cama
								Tropeçar	Banheiro

								Escorrega r	Cadeira
								Perda do equilíbrio	Cama
								Queda da própria altura	Cama
								Perda do equilíbrio	Banheiro
								Perda do equilíbrio	Cama

								Desmaio	Cadeira
								Perda do equilíbrio	Escadas / degraus

								Desmaio	Cadeira

								Perda do equilíbrio	Cama
								Queda da própria altura	Cama
Administração		Paciente errado							
								Queda da própria altura	Cama
								Queda da própria altura	Cama
								Queda da própria altura	Banheiro
								Queda da própria altura	Cama
								Queda da própria altura	Banheiro
								Perda do equilíbrio	Cadeira
			X						
				X					
			X						

								Queda da própria altura	Banheiro
								Queda da própria altura	Cama
								Queda da própria altura	Cama
								Perda do equilíbrio	Quarto
								Queda da própria altura	Cama
								Queda da própria altura	Cama
								Queda da própria altura	Cama
								Queda da própria altura	Cama
								Tropeçar	Banheiro
Procedimento / tratamento / intervençã		Incompleto / inadequado							
								Tropeçar	Banheiro
			X						

								Queda da própria altura	Cama
								Queda da própria altura	Banheiro
								Queda da própria altura	Cama
								Queda da própria altura	Cama
								Escorregar	Banheiro
								Perda do equilíbrio	Cadeira
								Desmaio	Banheiro
								Perda do equilíbrio	Cama
								Tropeçar	Quarto
								Perda do equilíbrio	Banheiro
				X					

								Perda do equilíbrio	Enquanto transportado / apoiado por outro
								Perda do equilíbrio	Banheiro
				X					
Procedimento / tratamento / intervençã		Incompleto / inadequado							
								Perda do equilíbrio	Maca
								Queda da própria altura	Cama
								Perda do equilíbrio	Equipamento terapêutico / diagnóstic
				X					
			X						
								Perda do equilíbrio	Cadeira
Procedimento / tratamento / intervençã		Incompleto / inadequado							

								Perda do equilíbrio	Cadeira
								Queda da própria altura	Cadeira
				X					
				X					
				X					

transferência de paciente		Indisponível							
								Perda do equilíbrio	Banheiro
								Queda da própria altura	Quarto
								Queda da própria altura	Cama
								Queda da própria altura	Quarto
								Perda do equilíbrio	Banheiro
								Perda do equilíbrio	Cama
								Perda do equilíbrio	Cama

								Desmaio	Quarto
								Queda da própria altura	Banheiro
								Queda da própria altura	Banheiro

								Queda da própria altura	Cadeira
								Perda do equilíbrio	Cama
								Queda da própria altura	Cama
								Queda da própria altura	Banheiro
								Escorregar	Banheiro
								Escorregar	Cadeira
								Perda do equilíbrio	Cama
								Desmaio	Cama
								Escorregar	Banheiro
								Perda do equilíbrio	Cadeira
								Queda da própria altura	Cama
								Escorregar	Cama

								Escorregar	Cama
								Escorregar	Cadeira
								Escorregar	Maca
								Escorregar	Maca
								Perda do equilíbrio	Cama
								Queda da própria altura	Cadeira
								Escorregar	Cama
								Perda do equilíbrio	Cadeira
								Perda do equilíbrio	Cama
								Perda do equilíbrio	Banheiro

								Perda do equilíbrio	Cama
								Queda da própria altura	Banheiro
								Queda da própria altura	Cama
								Queda da própria altura	Banheiro
Procedimento / tratamento / intervençã		Não efetuado quando indicado							

								Perda do equilíbrio	Cama
								Queda da própria altura	Banheiro
								Escorregar	Escadas / degraus
								Queda da própria altura	Banheiro
								Queda da própria altura	Cama
								Escorregar	Cama
								Queda da própria altura	Cama
								Perda do equilíbrio	Banheiro

								Queda da própria altura	Cama
								Queda da própria altura	Cadeira
								Escorregar	Cama
								Perda do equilíbrio	Cadeira
								Escorregar	Cama
								Escorregar	Cama
								Queda da própria altura	Cama
								Escorregar	Banheiro

								Tropeçar	Cadeira
								Escorregar	Cadeira
								Queda da própria altura	Cama
								Queda da própria altura	Cadeira

								Queda da própria altura	Maca

								Queda da própria altura	Banheiro
						Paciente errado	Fase pre-analítica (fase que		
Administração		Paciente errado							
								Queda da própria altura	Banheiro
								Perda do equilíbrio	Quarto
								Queda da própria altura	Quarto
Requisição		Não administração							

								Queda da própria altura	Quarto
Fornecimento		Não administração							
Assistência geral		Incompleto / inadequad							
Diagnostico / Meios compleme		Não efetuado quando							
Requisição		Não administração							
Administração		Não administração							
Assistência geral		Incompleto / inadequad							
Distribuição		Frequência errada							
Distribuição		Frequência errada							
								Perda do equilíbrio	Banheiro
								Queda da própria altura	Cama
								Perda do equilíbrio	Maca

								Perda do equilíbrio	Banheiro
								Queda da própria altura	Quarto
								Queda da própria altura	Banheiro
						Resultado errado	Fase analítica (conjunto		
						Coleta errada	Fase pos-analítica (fase que		
								Escorregar	Cama

								Perda do equilíbrio	Cama
								Queda da própria altura	Cadeira
Diagnostico / Meios compleme		Incompleto / inadequad							
Procedimento / tratament		Procedimento / tratament							
Alta		Processo errado							
Procedimento / tratament		Procedimento / tratament							
Marcação		Não efetuado quando							
Marcação		Não efetuado quando							
Assistência geral		Não efetuado quando							

Procedimento / tratament		Incompleto / inadequad							
Procedimento / tratament		Incompleto / inadequad							
								Escorregar	Cadeira
Assistência geral		Incompleto / inadequad							
Procedimento / tratament		Incompleto / inadequad							
Marcação		Não efetuado quando							
Procedimento / tratament		Indisponível							
Procedimento / tratament		Não efetuado quando							
Procedimento / tratament		Não efetuado quando							
Procedimento / tratament		Incompleto / inadequad							
Procedimento / tratament		Incompleto / inadequad							

Procedimento / tratament		Indisponível							
Procedimento / tratament		Indisponível							
Procedimento / tratament		Indisponível							
Diagnostico / Meios compleme		Incompleto / inadequad							
Procedimento / tratament		Incompleto / inadequad							
Procedimento / tratament		Paciente errado							
Procedimento / tratament		Indisponível							
Procedimento / tratament		Indisponível							
Procedimento / tratament		Indisponível							
Procedimento / tratament		Indisponível							

Procedim ento / tratament		Incomplet o / inadequad							
Lista de espera		Não efetuado quando							
Procedim ento / tratament		Não efetuado quando							
Procedim ento / tratament		Incomplet o / inadequad							
Assistênci a geral		Incomplet o / inadequad							
Assistênci a geral		Indisponív el							
Assistênci a geral		Indisponív el							
Fornecim ento		Não administra ção							
Procedim ento / tratament		Não efetuado quando							
Procedim ento / tratament		Incomplet o / inadequad							
						Coleta errada	Fase pós-analítica (fase que		
Procedim ento / tratament		Procedim ento / tratament							
Procedim ento / tratament		Não efetuado quando							
Diagnóstic o / Meios compleme		Incomplet o / inadequad							

Assistência geral		Incompleto / inadequad							
Procedimento / tratament		Não efetuado quando							
Procedimento / tratament		Não efetuado quando							
Procedimento / tratament		Procedimento / tratament							
Diagnostico / Meios compleme		Indisponível							
Diagnostico / Meios compleme		Não efetuado quando							
Procedimento / tratament		Paciente errado							
Diagnostico / Meios compleme		Não efetuado quando							
Procedimento / tratament		Incompleto / inadequad							
Assistência geral		Não efetuado quando							
Assistência geral		Procedimento / tratament							
Assistência geral		Procedimento / tratament							
Diagnostico / Meios compleme		Incompleto / inadequad							
Diagnostico / Meios compleme		Não efetuado quando							

								Queda da própria altura	Cama
Assistência geral		Incompleto / inadequad							
Assistência geral		Não efetuado quando							
Assistência geral		Não efetuado quando							
Procedimento / tratament		Não efetuado quando							
Assistência geral		Não efetuado quando							
Procedimento / tratament		Incompleto / inadequad							
Administração		Frequência errada							
Diagnostico / Meios compleme		Não efetuado quando							
Procedimento / tratament		Não efetuado quando							

Diagnostico / Meios compleme		Incompleto / inadequad							
Assistência geral		Procedimento / tratament							
Procedimento / tratament		Incompleto / inadequad							
Diagnostico / Meios compleme		Não efetuado quando							
Assistência geral		Não efetuado quando							
Procedimento / tratament		Procedimento / tratament							
								Perda do equilíbrio	Cama
Fornecimento		Frequência errada							
Procedimento / tratament		Não efetuado quando							
Assistência geral		Não efetuado quando							
Fornecimento		Dieta errada							
Procedimento / tratament		Não efetuado quando							
Procedimento / tratament		Não efetuado quando							
Assistência geral		Indisponível							
Assistência geral		Indisponível							

Procedim ento / tratament		Não efetuado quando							
Procedim ento / tratament		Incomplet o / inadequad							
Assistênci a geral		Não efetuado quando							
transferen cia de paciente		Processo errado							
Diagnostic o / Meios compleme		Incomplet o / inadequad							
Procedim ento / tratament		Não efetuado quando							
Lista de espera		Não efetuado quando							
Diagnostic o / Meios compleme		Incomplet o / inadequad							
Procedim ento / tratament		Procedim ento / tratament							
Assistênci a geral		Incomplet o / inadequad							
Procedim ento / tratament		Não efetuado quando							
Assistênci a geral		Procedim ento / tratament							
Assistênci a geral		Procedim ento / tratament							
								Queda da própria altura	Cama
Procedim ento / tratament		Não efetuado quando							
Diagnostic o / Meios compleme		Indisponív el							
Diagnostic o / Meios compleme		Não efetuado quando							
Assistênci a geral		Incomplet o / inadequad							
Procedim ento / tratament		Procedim ento / tratament							

Regulaça o / referencia		Não efetuado quando							
Assistênci a geral		Incomplet o / inadequad							
Assistênci a geral		Incomplet o / inadequad							
Assistênci a geral		Incomplet o / inadequad							
Assistênci a geral		Incomplet o / inadequad							
Procedim ento / tratament		Incomplet o / inadequad							
Assistênci a geral		Incomplet o / inadequad							
Assistênci a geral		Incomplet o / inadequad							
Assistênci a geral		Incomplet o / inadequad							
Assistênci a geral		Incomplet o / inadequad							
Procedim ento / tratament		Incomplet o / inadequad							
Assistênci a geral		Incomplet o / inadequad							
Assistênci a geral		Incomplet o / inadequad							
Assistênci a geral		Incomplet o / inadequad							
Procedim ento / tratament		Incomplet o / inadequad							
Procedim ento / tratament		Incomplet o / inadequad							
Assistênci a geral		Incomplet o / inadequad							
Assistênci a geral		Incomplet o / inadequad							
Assistênci a geral		Incomplet o / inadequad							
Procedim ento / tratament		Incomplet o / inadequad							
Procedim ento / tratament		Incomplet o / inadequad							
Assistênci a geral		Incomplet o / inadequad							

Assistência geral		Incompleto / inadequad							
		Outras falhas na assistênci							
Assistência geral		Incompleto / inadequad							
		Outras falhas na assistênci							
Assistência geral		Não efetuado quando							
Assistência geral		Incompleto / inadequad							
Procedimento / tratament		Não efetuado quando							
Assistência geral		Incompleto / inadequad							
Procedimento / tratament		Procedimento / tratament							
transferencia de paciente		Não efetuado quando							
Admissão		Não efetuado quando							
Administração		Frequência errada							
Alta		Incompleto / inadequad							
Marcação		Não efetuado quando							
Lista de espera		Não efetuado quando							
Assistência geral		Não efetuado quando							
Procedimento / tratament		Indisponível							

Contenção física		Procedimento / tratament							
Assistência geral		Não efetuado quando							
transferencia de paciente		Não efetuado quando							
Assistência geral		Incompleto / inadequad							
Diagnostico / Meios compleme		Não efetuado quando							
Assistência geral		Procedimento / tratament							
Diagnostico / Meios compleme		Não efetuado quando							
Procedimento / tratament		Procedimento / tratament							
Assistência geral		Incompleto / inadequad							
Procedimento / tratament		Não efetuado quando							
Assistência geral		Não efetuado quando							
		Outras falhas na assistênci							
Assistência geral		Não efetuado quando							
Procedimento / tratament		Procedimento / tratament							

Procedimento / tratament		Não efetuado quando							
Assistência geral		Incompleto / inadequad							
		Outras falhas na assistênci							
Procedimento / tratament		Incompleto / inadequad							
Procedimento / tratament		Incompleto / inadequad							
Assistência geral		Indisponível							
Administração		Frequência errada							
Procedimento / tratament		Não efetuado quando							
Procedimento / tratament		Incompleto / inadequad							

Assistênci a geral		Incomplet o / inadequad							

Assistênci a geral		Incomplet o / inadequad							
Assistênci a geral		Incomplet o / inadequad							
Assistênci a geral		Incomplet o / inadequad							
Assistênci a geral		Incomplet o / inadequad							
Assistênci a geral		Incomplet o / inadequad							
Assistênci a geral		Incomplet o / inadequad							
Assistênci a geral		Incomplet o / inadequad							
Assistênci a geral		Incomplet o / inadequad							
Assistênci a geral		Incomplet o / inadequad							
Assistênci a geral		Incomplet o / inadequad							
Assistênci a geral		Incomplet o / inadequad							
Assistênci a geral		Incomplet o / inadequad							
Assistênci a geral		Incomplet o / inadequad							
Assistênci a geral		Incomplet o / inadequad							
Assistênci a geral		Incomplet o / inadequad							
Assistênci a geral		Incomplet o / inadequad							
Assistênci a geral		Incomplet o / inadequad							
Assistênci a geral		Incomplet o / inadequad							
Assistênci a geral		Incomplet o / inadequad							

Assistência geral		Incompleto / inadequad							
Assistência geral		Incompleto / inadequad							
Assistência geral		Incompleto / inadequad							
Assistência geral		Incompleto / inadequad							
Assistência geral		Incompleto / inadequad							
Assistência geral		Incompleto / inadequad							
Assistência geral		Incompleto / inadequad							
Assistência geral		Incompleto / inadequad							
Assistência geral		Incompleto / inadequad							
Assistência geral		Incompleto / inadequad							
Assistência geral		Incompleto / inadequad							
Assistência geral		Incompleto / inadequad							
Assistência geral		Incompleto / inadequad							
Assistência geral		Incompleto / inadequad							
Assistência geral		Incompleto / inadequad							
Assistência geral		Incompleto / inadequad							
Assistência geral		Incompleto / inadequad							
Assistência geral		Incompleto / inadequad							
Assistência geral		Incompleto / inadequad							

Assistência geral		Incompleto / inadequad							
Procedimento / tratament		Não efetuado quando							
Procedimento / tratament		Não efetuado quando							
Procedimento / tratament		Não efetuado quando							
Diagnóstico / Meios compleme		Não efetuado quando							
Assistência geral		Incompleto / inadequad							
Assistência geral		Incompleto / inadequad							
Assistência geral		Incompleto / inadequad							
Assistência geral		Incompleto / inadequad							
Assistência geral		Incompleto / inadequad							
Assistência geral		Incompleto / inadequad							
Assistência geral		Incompleto / inadequad							
Assistência geral		Incompleto / inadequad							
Assistência geral		Incompleto / inadequad							
						Resultado errado	Fase pós-analítica (fase que		
Diagnóstico / Meios compleme		Paciente errado							
Diagnóstico / Meios compleme		Não efetuado quando							
Diagnóstico / Meios compleme		Incompleto / inadequad							
Diagnóstico / Meios compleme		Não efetuado quando							

Entrega		Dieta errada							
Preparaçã o		Consistên cia errada							
Preparaçã o		Consistên cia errada							
Administr ação		Paciente errado							
Administr ação		Paciente errado							
Entrega		Frequênci a errada							
Requisiçã o		Não administra ção							
Preparaçã o		Armazena mento errado							
Preparaçã o		Dieta errada							
Entrega		Frequênci a errada							
Entrega		Frequênci a errada							
Entrega		Dieta errada							
Administr ação		Paciente errado							
Distribuiç ão		Não administra ção							
Entrega		Quantidad e errada							
Administr ação		Não administra ção							
Entrega		Quantidad e errada							
Entrega		Quantidad e errada							
Distribuiç ão		Não administra ção							

Preparação		Dieta errada							
Preparação		Armazenamento errado							
Distribuição		Não administração							
Distribuição		Não administração							
Administração		Consistência errada							
Entrega		Não administração							
Entrega		Dieta errada							
Entrega		Dieta errada							
Entrega		Dieta errada							
Administração		Dieta errada							
Preparação		Quantidade errada							
Entrega		Dieta errada							
Entrega		Dieta errada							
Apresentação		Não administração							
Distribuição		Consistência errada							
Administração		Dieta errada							
Apresentação		Quantidade errada							
Armazenamento		Armazenamento errado							
Preparação		Consistência errada							

Administr ação		Paciente errado							
Administr ação		Paciente errado							
Entrega		Paciente errado							
Preparaçã o		Consistên cia errada							
Entrega		Frequênci a errada							
								Queda da própria altura	Quarto
								Escorrega r	Cadeira
								Escorrega r	Cama
								Escorrega r	Cama
								Escorrega r	Enquanto transporta do /
								Queda da própria altura	Banheiro
								Escorrega r	Banheiro
								Escorrega r	Cama
								Escorrega r	Cama
								Desmaio	Banheiro
								Queda da própria altura	Quarto

Assistência geral		Incompleto / inadequad							
Assistência geral		Incompleto / inadequad							
Assistência geral		Incompleto / inadequad							
Assistência geral		Incompleto / inadequad							
Assistência geral		Incompleto / inadequad							
Assistência geral		Incompleto / inadequad							
Assistência geral		Incompleto / inadequad							
Assistência geral		Incompleto / inadequad							
Assistência geral		Incompleto / inadequad							
Assistência geral		Incompleto / inadequad							
Assistência geral		Incompleto / inadequad							
Preparação		Dieta errada							

Assistência geral		Incompleto / inadequad							
Assistência geral		Incompleto / inadequad							
Assistência geral		Incompleto / inadequad							
Assistência geral		Incompleto / inadequad							
Assistência geral		Incompleto / inadequad							
Distribuição		Não administração							
Distribuição		Não administração							
Administração		Dieta errada							
Assistência geral		Incompleto / inadequad							
Assistência geral		Incompleto / inadequad							
Assistência geral		Incompleto / inadequad							
Assistência geral		Incompleto / inadequad							
Assistência geral		Incompleto / inadequad							
Assistência geral		Incompleto / inadequad							
Assistência geral		Incompleto / inadequad							
Assistência geral		Incompleto / inadequad							
Assistência geral		Incompleto / inadequad							
Assistência geral		Incompleto / inadequad							
Assistência geral		Incompleto / inadequad							

Assistência geral		Incompleto / inadequad							
Assistência geral		Incompleto / inadequad							
Assistência geral		Incompleto / inadequad							
Assistência geral		Incompleto / inadequad							
Assistência geral		Incompleto / inadequad							
Assistência geral		Incompleto / inadequad							
Assistência geral		Incompleto / inadequad							
Assistência geral		Incompleto / inadequad							
Assistência geral		Incompleto / inadequad							
Assistência geral		Incompleto / inadequad							
Assistência geral		Incompleto / inadequad							
Assistência geral		Incompleto / inadequad							
Assistência geral		Incompleto / inadequad							
Assistência geral		Incompleto / inadequad							
Assistência geral		Incompleto / inadequad							
Assistência geral		Incompleto / inadequad							
Assistência geral		Incompleto / inadequad							
Assistência geral		Incompleto / inadequad							
Assistência geral		Incompleto / inadequad							

Assistênci a geral		Incomplet o / inadequad							
Assistênci a geral		Incomplet o / inadequad							
Entrega		Não administra ção							
Entrega		Não administra ção							
Entrega		Não administra ção							
Diagnóstic o / Meios compleme		Não efetuado quando							
Diagnóstic o / Meios compleme		Incomplet o / inadequad							
Procedim ento / tratament		Não efetuado quando							
Procedim ento / tratament		Não efetuado quando							
Diagnóstic o / Meios compleme		Incomplet o / inadequad							
Diagnóstic o / Meios compleme		Não efetuado quando							
Entrega		Não administra ção							
Procedim ento / tratament		Não efetuado quando							
Preparaçã o		Quantidad e errada							
Preparaçã o		Dieta errada							
Entrega		Frequênci a errada							
Entrega		Não administra ção							
Entrega		Não administra ção							
Entrega		Não administra ção							

Administr ação		Dieta errada							
Entrega		Não administra ção							
Entrega		Não administra ção							
Preparaçã o		Consistên cia errada							
Preparaçã o		Consistên cia errada							
								Escorrega r	Cama
								Escorrega r	Cama
								Escorrega r	Cadeira
								Queda da própria altura	Quarto
								Escorrega r	Banheiro
								Queda da própria altura	Quarto
								Queda da própria altura	Quarto
								Escorrega r	Cadeira
								Escorrega r	Cama
								Perda do equilíbrio	Quarto
								Escorrega r	Berço
								Escorrega r	Maca
								Escorrega r	Berço

								Escorrega r	Maca
								Escorrega r	Enquanto transporta do /
								Escorrega r	Cama
Administr ação		Frequênci a errada							
Administr ação		Não administra ção							
Administr ação		Frequênci a errada							
Administr ação		Frequênci a errada							
Administr ação		Paciente errado							
Administr ação		Paciente errado							
Administr ação		Paciente errado							
Administr ação		Paciente errado							
Assistênci a geral		Incomplet o / inadequad							
Assistênci a geral		Incomplet o / inadequad							
Assistênci a geral		Incomplet o / inadequad							
Assistênci a geral		Incomplet o / inadequad							
			X						
						Resultado errado	Fase analítica (conjunto		
Administr ação		Não administra ção							

Diagnóstic o / Meios compleme		Não efetuado quando							

Assistência geral		Incompleto / inadequad							
Assistência geral		Incompleto / inadequad							
Assistência geral		Incompleto / inadequad							
Distribuição		Local errado							
Procedimento / tratament		Não efetuado quando							
Diagnóstico / Meios compleme		Incompleto / inadequad							

Procedim ento / tratament		Não efetuado quando							
Diagnóstic o / Meios compleme		Incomplet o / inadequad							
Diagnóstic o / Meios compleme		Incomplet o / inadequad							
								Queda da própria altura	Quarto
								Queda da própria altura	Banheiro
Distribuiç ão		Não administra ção							
Diagnóstic o / Meios compleme		Incomplet o / inadequad							
Diagnóstic o / Meios compleme		Incomplet o / inadequad							
								Queda da própria altura	Banheiro
Administr ação		Não administra ção							
Administr ação		Não administra ção							
	Mecanism os de identificaç	Document o em falta ou							
								Queda da própria altura	Banheiro
Diagnóstic o / Meios compleme		Incomplet o / inadequad							
Diagnóstic o / Meios compleme		Incomplet o / inadequad							

Procedim ento / tratament		Não efetuado quando							
						Paciente errado	Fase pré-analítica (fase que		
Distribuiç ão		Não administra ção							
Distribuiç ão		Não administra ção							
Diagnóstic o / Meios compleme		Incomplet o / inadequad							
								Escorrega r	Cama
								Queda da própria altura	Quarto
	Tabelas / registros médicos /	Informaçõ es ambígua							
					X				
					X				
								Queda da própria altura	Banheiro
Diagnóstic o / Meios compleme		Incomplet o / inadequad							
Diagnóstic o / Meios compleme		Incomplet o / inadequad							

Diagnóstico / Meios compleme		Incompleto / inadequad							
								Escorregar	Cama
Diagnóstico / Meios compleme		Incompleto / inadequad							
Diagnóstico / Meios compleme		Incompleto / inadequad							
Diagnóstico / Meios compleme		Incompleto / inadequad							
Diagnóstico / Meios compleme		Incompleto / inadequad							
Diagnóstico / Meios compleme		Incompleto / inadequad							
Diagnóstico / Meios compleme		Incompleto / inadequad							
								Queda da própria altura	Banheiro
Diagnóstico / Meios compleme		Incompleto / inadequad							
Diagnóstico / Meios compleme		Incompleto / inadequad							
Diagnóstico / Meios compleme		Incompleto / inadequad							
								Queda da própria altura	Banheiro
Diagnóstico / Meios compleme		Incompleto / inadequad							
Diagnóstico / Meios compleme		Incompleto / inadequad							

Assistência geral		Incompleto / inadequad							
Distribuição		Frequência errada							
Distribuição		Frequência errada							
								Escorregar	Berço
Diagnóstico / Meios compleme		Incompleto / inadequad							
Diagnóstico / Meios compleme		Incompleto / inadequad							
Diagnóstico / Meios compleme		Incompleto / inadequad							
Administração		Não administração							
Diagnóstico / Meios compleme		Incompleto / inadequad							
Diagnóstico / Meios compleme		Incompleto / inadequad							

Diagnóstico / Meios compleme		Incompleto / inadequad							
Diagnóstico / Meios compleme		Incompleto / inadequad							
Diagnóstico / Meios compleme		Incompleto / inadequad							
Diagnóstico / Meios compleme		Incompleto / inadequad							

Diagnóstico / Meios compleme		Incompleto / inadequad							
Diagnóstico / Meios compleme		Incompleto / inadequad							
Assistência geral		Procedimento / tratament							
Assistência geral		Procedimento / tratament							
Diagnóstico / Meios compleme		Incompleto / inadequad							
Assistência geral		Procedimento / tratament							
Diagnóstico / Meios compleme		Incompleto / inadequad							
Assistência geral		Procedimento / tratament							
Assistência geral		Procedimento / tratament							
Diagnóstico / Meios compleme		Incompleto / inadequad							

Diagnóstico / Meios compleme		Incompleto / inadequad							

Assistência geral		Incompleto / inadequad							
Assistência geral		Incompleto / inadequad							
Assistência geral		Incompleto / inadequad							
Assistência geral		Incompleto / inadequad							
Assistência geral		Incompleto / inadequad							
Assistência geral		Incompleto / inadequad							
Assistência geral		Incompleto / inadequad							
Assistência geral		Incompleto / inadequad							
Assistência geral		Incompleto / inadequad							
Assistência geral		Incompleto / inadequad							
Assistência geral		Incompleto / inadequad							
Assistência geral		Incompleto / inadequad							
Assistência geral		Incompleto / inadequad							

Assistência geral		Incompleto / inadequad							
Assistência geral		Incompleto / inadequad							
Assistência geral		Incompleto / inadequad							
Assistência geral		Procedimento / tratament							
Procedimento / tratament		Procedimento / tratament							
Procedimento / tratament		Procedimento / tratament							
Procedimento / tratament		Incompleto / inadequad							
Assistência geral		Incompleto / inadequad							
Assistência geral		Incompleto / inadequad							
Procedimento / tratament		Incompleto / inadequad							
Assistência geral		Incompleto / inadequad							
Procedimento / tratament		Incompleto / inadequad							
Assistência geral		Incompleto / inadequad							
Assistência geral		Incompleto / inadequad							
Procedimento / tratament		Incompleto / inadequad							
Armazenamento		Armazenamento errado							
Distribuição		Não administração							
Distribuição		Não administração							
Administração		Armazenamento errado							

Distribuição		Não administração							
Preparação		Dieta errada							
Prescrição		Paciente errado							

Administração		Não administração							

Administração		Não administração							
Administração		Não administração							
Administração		Não administração							
Administração		Não administração							
Administração		Não administração							
Administração		Não administração							
Administração		Não administração							
Administração		Não administração							
Administração		Não administração							
Administração		Não administração							
Administração		Não administração							
Administração		Não administração							
Preparação		Consistência errada							
			X						
					X				
					X				
			X						
						Resultado errado	Fase analítica (conjunto		
						Coleta errada	Fase pré-analítica (fase que		

						Coleta errada	Fase pré-analítica (fase que		
						Coleta errada	Fase pré-analítica (fase que		
						Coleta errada	Fase pré-analítica (fase que		
						Coleta errada	Fase pré-analítica (fase que		
						Coleta errada	Fase pré-analítica (fase que		
						Coleta errada	Fase pré-analítica (fase que		
						Coleta errada	Fase pré-analítica (fase que		
						Coleta errada	Fase pré-analítica (fase que		
						Coleta errada	Fase pré-analítica (fase que		
						Coleta errada	Fase pré-analítica (fase que		
						Coleta errada	Fase pré-analítica (fase que		

Assistênci a geral		Indisponív el							
Procedim ento / tratament		Não efetuado quando							
Procedim ento / tratament		Procedim ento / tratament							
transferên cia de paciente		Processo errado							
transferên cia de paciente		Processo errado							
transferên cia de paciente		Processo errado							
Procedim ento / tratament		Procedim ento / tratament							
Procedim ento / tratament		Procedim ento / tratament							
Procedim ento / tratament		Procedim ento / tratament							
Procedim ento / tratament		Procedim ento / tratament							
Contençã o física		Procedim ento / tratament							
Diagnóstic o / Meios compleme		Procedim ento / tratament							
Procedim ento / tratament		Procedim ento / tratament							
Procedim ento / tratament		Procedim ento / tratament							

Procedimento / tratament		Procedimento / tratament							
Procedimento / tratament		Procedimento / tratament							
Procedimento / tratament		Procedimento / tratament							
Procedimento / tratament		Procedimento / tratament							

								Queda da própria altura	Banheiro
								Queda da própria altura	Cama
								Perda do equilíbrio	Cama
						Coleta errada	Fase pré-analítica (fase que		
								Queda da própria altura	Cama
						Coleta errada	Fase pré-analítica (fase que		
								Escorregar	Banheiro
						Coleta errada	Fase pré-analítica (fase que		
								Perda do equilíbrio	Cama
						Coleta errada	Fase pré-analítica (fase que		
								Escorregar	Cama
								Escorregar	Cama
						Processamento errado	Fase pré-analítica (fase que		
						Processamento errado	Fase pré-analítica (fase que		
								Queda da própria altura	Banheiro

						Processa mento errado	Fase pré-analítica (fase que		
						Processa mento errado	Fase pré-analítica (fase que		
								Escorrega r	Banheiro
						Processa mento errado	Fase pré-analítica (fase que		
								Perda do equilíbrio	Cama
Consenti mento informado		Não efetuado quando							
	Formulári os	Informaçõ es ambígua							
								Queda da própria altura	Quarto
								Escorrega r	Cama
transferên cia de paciente		Processo errado							
								Perda do equilíbrio	Cama
transferên cia de paciente		Processo errado							
Consenti mento informado		Não efetuado quando							
Consenti mento informado		Não efetuado quando							
								Tropeçar	Cama
Assistênci a geral		Procedim ento / tratament							
transferên cia de paciente		Processo errado							
transferên cia de paciente		Processo errado							
								Tropeçar	Cama

Procedimento / tratament		Procedimento / tratament							
								Escorregar	Cama
Assistência geral		Não efetuado quando							
						Coleta errada	Fase pré-analítica (fase que		
								Queda da própria altura	Quarto
Procedimento / tratament		Procedimento / tratament							
								Queda da própria altura	Cama
Procedimento / tratament		Procedimento / tratament							
								Queda da própria altura	Quarto
								Tropeçar	Cama
								Tropeçar	Cama
								Escorregar	Banheiro
								Queda da própria altura	Quarto
								Tropeçar	Cama
								Escorregar	Banheiro
								Perda do equilíbrio	Cama
								Perda do equilíbrio	Cama
Assistência geral		Não efetuado quando							
Procedimento / tratament		Procedimento / tratament							

Procedimento / tratament		Procedimento / tratament							
Procedimento / tratament		Procedimento / tratament							
Procedimento / tratament		Procedimento / tratament							
Assistência geral		Procedimento / tratament							
Procedimento / tratament		Procedimento / tratament							
Procedimento / tratament		Procedimento / tratament							
Procedimento / tratament		Não efetuado quando							
								Escorregar	Banheiro
Procedimento / tratament		Procedimento / tratament							
Procedimento / tratament		Não efetuado quando							
								Perda do equilíbrio	Cama
								Escorregar	Cama
								Desmaio	Quarto
								Queda da própria altura	Banheiro
								Perda do equilíbrio	Banheiro
								Escorregar	Banheiro
								Escorregar	Cama
								Queda da própria altura	Quarto
								Escorregar	Cama

								Queda da própria altura	Cama
Procedimento / tratament		Procedimento / tratament							
Entrega		Não administração							
Distribuição		Armazenamento errado							
Procedimento / tratament		Procedimento / tratament							
Assistência geral		Procedimento / tratament							
Distribuição		Frequência errada							
Assistência geral		Procedimento / tratament							
Administração		Não administração							
Fornecimento		Local errado							
Distribuição		Frequência errada							
Distribuição		Dieta errada							
Distribuição		Local errado							
Distribuição		Dieta errada							
Distribuição		Dieta errada							
Distribuição		Frequência errada							
Distribuição		Dieta errada							
Distribuição		Frequência errada							
Distribuição		Frequência errada							

Distribuição		Frequência errada							
Distribuição		Frequência errada							
Distribuição		Dieta errada							
Distribuição		Não administração							
Distribuição		Frequência errada							
Distribuição		Paciente errado							
Apresentação		Dieta errada							
Entrega		Dieta errada							
Administração		Frequência errada							
Entrega		Paciente errado							
Distribuição		Dieta errada							
			X						
Administração		Frequência errada							
Administração		Não administração							
Administração		Não administração							
Administração		Não administração							
Administração		Não administração							
Administração		Não administração							
Administração		Não administração							

transferência de paciente		Processo errado							
transferência de paciente		Processo errado							
transferência de paciente		Processo errado							
transferência de paciente		Processo errado							
transferência de paciente		Processo errado							
transferência de paciente		Processo errado							
transferência de paciente		Paciente errado							
transferência de paciente		Paciente errado							
transferência de paciente		Paciente errado							
transferência de paciente		Paciente errado							
transferência de paciente		Processo errado							
transferência de paciente		Processo errado							
transferência de paciente		Processo errado							
transferência de paciente		Paciente errado							
transferência de paciente		Processo errado							
transferência de paciente		Processo errado							
Alta		Processo errado							
transferência de paciente		Processo errado							
transferência de paciente		Processo errado							

transferência de paciente		Paciente errado							
transferência de paciente		Processo errado							
transferência de paciente		Processo errado							
transferência de paciente		Processo errado							
transferência de paciente		Processo errado							
transferência de paciente		Processo errado							
transferência de paciente		Processo errado							
transferência de paciente		Processo errado							
transferência de paciente		Processo errado							
						Processamento errado	Fase pré-analítica (fase que		
transferência de paciente		Incompleto / inadequad							
Consentimento informado		Incompleto / inadequad							
Procedimento / tratament		Incompleto / inadequad							
Diagnóstico / Meios compleme		Procedimento / tratament							
transferência de paciente		Processo errado							

Lista de espera		Não efetuado quando							
Procedimento / tratament		Incompleto / inadequad							
Procedimento / tratament		Procedimento / tratament							
transferência de paciente		Processo errado							

								Queda da própria altura	Banheiro
								Escorregar	Maca
								Queda da própria altura	Enquanto transportado /
								Escorregar	Banheiro
								Escorregar	Banheiro
								Escorregar	Banheiro
								Perda do equilíbrio	Cama
								Queda da própria altura	Berço
Procedimento / tratament		Procedimento / tratament							
Distribuição		Dieta errada							
Preparação		Quantidade errada							
Distribuição		Não administração							
Distribuição		Frequência errada							
Distribuição		Não administração							
Distribuição		Dieta errada							
Distribuição		Frequência errada							

Entrega		Frequência errada							
Armazenamento		Frequência errada							
Distribuição		Frequência errada							
Preparação		Dieta errada							
Resposta à emergênc		Processo errado							
Identificação do paciente		Processo errado							
Identificação do paciente		Processo errado							
Identificação do paciente		Processo errado							
Diagnóstico / Meios compleme		Paciente errado							
Diagnóstico / Meios compleme		Não efetuado quando							
Procedimento / tratament		Incompleto / inadequad							
Procedimento / tratament		Incompleto / inadequad							
Procedimento / tratament		Não efetuado quando							
Diagnóstico / Meios compleme		Procedimento / tratament							
Procedimento / tratament		Paciente errado							
Procedimento / tratament		Paciente errado							
transferência de paciente		Incompleto / inadequad							
Procedimento / tratament		Incompleto / inadequad							
Procedimento / tratament		Incompleto / inadequad							

Procedim ento / tratament		Incomplet o / inadequad							
Procedim ento / tratament		Incomplet o / inadequad							
Procedim ento / tratament		Incomplet o / inadequad							
Procedim ento / tratament		Incomplet o / inadequad							
Diagnóstic o / Meios compleme		Incomplet o / inadequad							
Diagnóstic o / Meios compleme		Incomplet o / inadequad							
Diagnóstic o / Meios compleme		Incomplet o / inadequad							
Diagnóstic o / Meios compleme		Incomplet o / inadequad							
Diagnóstic o / Meios compleme		Incomplet o / inadequad							
Diagnóstic o / Meios compleme		Incomplet o / inadequad							
Diagnóstic o / Meios compleme		Incomplet o / inadequad							
Diagnóstic o / Meios compleme		Incomplet o / inadequad							
Diagnóstic o / Meios compleme		Incomplet o / inadequad							
Diagnóstic o / Meios compleme		Incomplet o / inadequad							
Diagnóstic o / Meios compleme		Incomplet o / inadequad							
Assistênci a geral		Incomplet o / inadequad							
Diagnóstic o / Meios compleme		Incomplet o / inadequad							
Procedim ento / tratament		Incomplet o / inadequad							
Diagnóstic o / Meios compleme		Incomplet o / inadequad							

Diagnóstico / Meios compleme		Incompleto / inadequad							
Diagnóstico / Meios compleme		Paciente errado							
Diagnóstico / Meios compleme		Incompleto / inadequad							
Diagnóstico / Meios compleme		Incompleto / inadequad							
Diagnóstico / Meios compleme		Incompleto / inadequad							
Diagnóstico / Meios compleme		Incompleto / inadequad							
Diagnóstico / Meios compleme		Paciente errado							
Diagnóstico / Meios compleme		Incompleto / inadequad							
Diagnóstico / Meios compleme		Incompleto / inadequad							
Procedimento / tratament		Indisponível							
Procedimento / tratament		Não efetuado quando							
Procedimento / tratament		Não efetuado quando							
Procedimento / tratament		Não efetuado quando							

Procedimento / tratament		Indisponível							
								Escorregar	Cama
								Perda do equilíbrio	Cama
Administração		Paciente errado							
Administração		Frequência errada							
Distribuição		Frequência errada							

Distribuição		Frequência errada							
Distribuição		Frequência errada							
Distribuição		Frequência errada							
Identificação do paciente		Paciente errado							
Identificação do paciente		Paciente errado							
transferência de paciente		Paciente errado							
Identificação do paciente		Paciente errado							
transferência de paciente		Não efetuado quando							
transferência de paciente		Processo errado							
transferência de paciente		Processo errado							
transferência de paciente		Processo errado							
transferência de paciente		Paciente errado							
transferência de paciente		Processo errado							
Procedimento / tratament		Incompleto / inadequad							
Procedimento / tratament		Incompleto / inadequad							
Procedimento / tratament		Incompleto / inadequad							
Procedimento / tratament		Incompleto / inadequad							
Procedimento / tratament		Incompleto / inadequad							
Procedimento / tratament		Incompleto / inadequad							

Procedim ento / tratament		Incomplet o / inadequad							
Procedim ento / tratament		Incomplet o / inadequad							
Procedim ento / tratament		Incomplet o / inadequad							
Procedim ento / tratament		Incomplet o / inadequad							
Diagnóstic o / Meios compleme		Procedim ento / tratament							
						Coleta errada	Fase pré-analítica (fase que		
Procedim ento / tratament		Incomplet o / inadequad							
Procedim ento / tratament		Incomplet o / inadequad							
Procedim ento / tratament		Procedim ento / tratament							
						Processa mento errado	Fase analítica (conjunto		
Procedim ento / tratament		Incomplet o / inadequad							
Procedim ento / tratament		Incomplet o / inadequad							
						Coleta errada	Fase analítica (conjunto		
Procedim ento / tratament		Indisponív el							
Assistênci a geral		Incomplet o / inadequad							
Procedim ento / tratament		Incomplet o / inadequad							
Identificaç ão do paciente		Processo errado							
Procedim ento / tratament		Incomplet o / inadequad							
Procedim ento / tratament		Procedim ento / tratament							

Diagnóstico / Meios compleme		Procedimento / tratament							
transferência de paciente		Processo errado							
Procedimento / tratament		Não efetuado quando							
Diagnóstico / Meios compleme		Não efetuado quando							
Identificação do paciente		Paciente errado							
Preparação		Consistência errada							
Administração		Quantidade errada							
Distribuição		Quantidade errada							
Distribuição		Quantidade errada							
Distribuição		Quantidade errada							
Distribuição		Frequência errada							
								Queda da própria altura	Cadeira

Procedim ento / tratament		Não efetuado quando							

Prescrição		Não administração							
Distribuição		Frequência errada							

Preparação		Dieta errada							
Prescrição		Quantidade errada							
Procedimento / tratament		Incompleto / inadequad							
Distribuição		Frequência errada							
transferência de paciente		Processo errado							
transferência de paciente		Incompleto / inadequad							
transferência de paciente		Processo errado							
Procedimento / tratament		Não efetuado quando							
Procedimento / tratament		Não efetuado quando							
Procedimento / tratament		Não efetuado quando							
Distribuição		Frequência errada							
Identificação do paciente		Paciente errado							

Distribuição		Frequência errada							
Distribuição		Frequência errada							
	Formulários	Documento em falta ou							
	Formulários	Documento em falta ou							
	Formulários	Documento em falta ou							
Procedimento / tratament		Paciente errado							
								Queda da própria altura	Cama
Distribuição		Quantidade errada							
Assistência geral		Procedimento / tratament							
								Queda da própria altura	Cama
								Queda da própria altura	Cama
Distribuição		Dieta errada							
Assistência geral		Incompleto / inadequad							

Assistência geral		Incompleto / inadequad							
Distribuição		Frequência errada							
Distribuição		Frequência errada							
Entrega		Frequência errada							
Requisição		Frequência errada							
Requisição		Frequência errada							
								Queda da própria altura	Cadeira
								Queda da própria altura	Cadeira
Distribuição		Frequência errada							
Distribuição		Frequência errada							
								Queda da própria altura	Cama
Assistência geral		Não efetuado quando							

								Queda da própria altura	Cama
Assistênci a geral		Não efetuado quando							

								Escorregar	Banheiro
								Queda da própria altura	Cama
								Perda do equilíbrio	Cama
								Escorregar	Maca
								Escorregar	Cama
Prescrição		Local errado							
Entrega		Não administração							
Consentimento informado		Processo errado							
	Mecanismos de identificaç	Informações ambígua							
					X				
					X				
	Formulários	Informações ambígua							
Procedimento / tratament		Não efetuado quando							

Procedim ento / tratament		Procedim ento / tratament							
Diagnóstic o / Meios compleme		Não efetuado quando							
Procedim ento / tratament		Não efetuado quando							
						Coleta errada	Fase pré-analítica (fase que		
Diagnóstic o / Meios compleme		Procedim ento / tratament							

								Queda da própria altura	Quarto
								Escorregar	Cama
Distribuição		Frequência errada							
Distribuição		Frequência errada							

Distribuição		Frequência errada							
Distribuição		Frequência errada							
Administração		Frequência errada							
	Formulários	Documento em falta ou							
Diagnóstico / Meios compleme		Incompleto / inadequad							
Diagnóstico / Meios compleme		Incompleto / inadequad							
Diagnóstico / Meios compleme		Incompleto / inadequad							
Diagnóstico / Meios compleme		Incompleto / inadequad							
Diagnóstico / Meios compleme		Não efetuado quando							
Diagnóstico / Meios compleme		Indisponível							

Distribuição		Frequência errada							
Assistência geral		Incompleto / inadequad							
			X						
Assistência geral		Incompleto / inadequad							
Procedimento / tratament		Incompleto / inadequad							
Procedimento / tratament		Incompleto / inadequad							

								Queda da própria altura	Quarto
								Queda da própria altura	Quarto
Distribuição		Não administração							

Identificação do paciente		Processo errado							
Assistência geral		Não efetuado quando							
Diagnóstico / Meios compleme		Procedimento / tratament							

Diagnóstico / Meios compleme		Não efetuado quando							
Diagnóstico / Meios compleme		Não efetuado quando							
Diagnóstico / Meios compleme		Não efetuado quando							
Diagnóstico / Meios compleme		Não efetuado quando							
Procedimento / tratament		Não efetuado quando							
Consentimento informado		Não efetuado quando							
Administração		Frequência errada							
Consentimento informado		Não efetuado quando							
Diagnóstico / Meios compleme		Incompleto / inadequad							
Diagnóstico / Meios compleme		Não efetuado quando							
Diagnóstico / Meios compleme		Paciente errado							

Assistênci a geral		Não efetuado quando							
Administr ação		Frequênci a errada							
Assistênci a geral		Não efetuado quando							
Distribuiç ão		Dieta errada							
Diagnóstic o / Meios compleme		Não efetuado quando							
Distribuiç ão		Dieta errada							
								Queda da própria altura	Cadeira
								Queda da própria altura	Banheiro
Distribuiç ão		Quantidad e errada							
Prescriçã o		Dieta errada							
Distribuiç ão		Frequênci a errada							
Diagnóstic o / Meios compleme		Não efetuado quando							
								Queda da própria altura	Cama
								Queda da própria altura	Berço

Assistência geral		Procedimento / tratament							
Diagnóstico / Meios compleme		Não efetuado quando							
Distribuição		Frequência errada							
Identificação do paciente		Processo errado							
Assistência geral		Não efetuado quando							
Diagnóstico / Meios compleme		Não efetuado quando							
Armazenamento		Armazenamento errado							
								Queda da própria altura	Cama

Procedim ento / tratament		Incomplet o / inadequad							
Assistênci a geral		Incomplet o / inadequad							
Assistênci a geral		Incomplet o / inadequad							
			X						
Procedim ento / tratament		Não efetuado quando							
Diagnóstic o / Meios compleme		Não efetuado quando							
Entrega		Dieta errada							
								Queda da própria altura	Berço
				X					

								Queda da própria altura	Berço
Assistência geral		Incompleto / inadequad							
Prescrição		Dieta errada							
Diagnóstico / Meios compleme		Não efetuado quando							

Distribuição		Frequência errada							
Procedimento / tratament		Não efetuado quando							
Distribuição		Dieta errada							

Procedimento / tratament		Não efetuado quando							
Distribuição		Frequência errada							
Distribuição		Frequência errada							
Procedimento / tratament		Não efetuado quando							
Administração		Consistência errada							
Administração		Não administração							
Distribuição		Dieta errada							
Assistência geral		Incompleto / inadequad							
Assistência geral		Não efetuado quando							
Procedimento / tratament		Procedimento / tratament							

Procedim ento / tratament		Não efetuado quando							
								Escorrega r	Enquanto transporta do /
								Queda da própria altura	Quarto
Distribuiç ão		Frequênci a errada							
Procedim ento / tratament		Não efetuado quando							
Assistênci a geral		Incomplet o / inadequad							
Administr ação		Não administra ção							
Assistênci a geral		Não efetuado quando							
		Lesão de órgão durante a							
Administr ação		Frequênci a errada							
Administr ação		Dieta errada							
Assistênci a geral		Não efetuado quando							
Assistênci a geral		Não efetuado quando							
Distribuiç ão		Frequênci a errada							
Assistênci a geral		Incomplet o / inadequad							
Assistênci a geral		Incomplet o / inadequad							
								Queda da própria altura	Cama

Assistência geral		Não efetuado quando							
Assistência geral		Incompleto / inadequad							
Assistência geral		Incompleto / inadequad							
Administração		Não administração							
								Queda da própria altura	Cama
								Queda da própria altura	Cama
Assistência geral		Incompleto / inadequad							
Identificação do paciente		Incompleto / inadequad							
Distribuição		Não administração							
Assistência geral		Incompleto / inadequad							
Entrega		Não administração							
Administração		Quantidade errada							
Assistência geral		Incompleto / inadequad							
								Queda da própria altura	Cama
								Queda da própria altura	Banheiro
								Queda da própria altura	Berço
Assistência geral		Incompleto / inadequad							

Distribuição		Quantidade errada							
Diagnóstico / Meios compleme		Não efetuado quando							
Diagnóstico / Meios compleme		Paciente errado							
								Queda da própria altura	Cama
Diagnóstico / Meios compleme		Não efetuado quando							
Requisição		Frequência errada							
								Perda do equilíbrio	Cama
Administração		Não administração							
Administração		Paciente errado							
								Queda da própria altura	Banheiro
								Perda do equilíbrio	Cama

Procedimento / tratament		Incompleto / inadequad							
Assistência geral		Não efetuado quando							
Procedimento / tratament		Não efetuado quando							
Assistência geral		Incompleto / inadequad							
Administração		Não administração							
								Queda da própria altura	Cadeira
Assistência geral		Incompleto / inadequad							

Assistênci a geral		Não efetuado quando							
								Queda da própria altura	Enquanto transporta do /
Assistênci a geral		Incomplet o / inadequad							
Assistênci a geral		Incomplet o / inadequad							
Assistênci a geral		Incomplet o / inadequad							
Assistênci a geral		Incomplet o / inadequad							
Assistênci a geral		Incomplet o / inadequad							
Assistênci a geral		Incomplet o / inadequad							
Assistênci a geral		Incomplet o / inadequad							
Assistênci a geral		Incomplet o / inadequad							
Assistênci a geral		Incomplet o / inadequad							
								Queda da própria altura	Cama

Assistência geral		Incompleto / inadequad							
Assistência geral		Não efetuado quando							
								Escorregar	Berço
								Queda da própria altura	Cama
Assistência geral		Incompleto / inadequad							
Assistência geral		Incompleto / inadequad							
Assistência geral		Incompleto / inadequad							

								Queda da própria altura	Berço
								Perda do equilíbrio	Maca
								Perda do equilíbrio	Cama
Procedim ento / tratament		Não efetuado quando							
Procedim ento / tratament		Não efetuado quando							
								Queda da própria altura	Cadeira
								Queda da própria altura	Berço

								Queda da própria altura	Cama
								Perda do equilíbrio	Cama
								Perda do equilíbrio	Cama
								Perda do equilíbrio	Cama
								Perda do equilíbrio	Cama
Procedimento / tratament		Não efetuado quando							
								Queda da própria altura	Cama
Preparação		Consistência errada							
								Queda da própria altura	Berço

								Perda do equilíbrio	Cama
Requisição		Consistência errada							
Assistência geral		Incompleto / inadequad							

	Formulários	Informações ambígua							
Administração		Frequência errada							
Assistência geral		Não efetuado quando							
Assistência geral		Não efetuado quando							
Assistência geral		Incompleto / inadequad							
Administração		Paciente errado							

Assistência geral		Não efetuado quando							
Distribuição		Quantidade errada							
Assistência geral		Não efetuado quando							
Assistência geral		Não efetuado quando							
Distribuição		Quantidade errada							
Assistência geral		Incompleto / inadequad							
Assistência geral		Incompleto / inadequad							
Assistência geral		Incompleto / inadequad							
Assistência geral		Não efetuado quando							
Assistência geral		Incompleto / inadequad							
								Queda da própria altura	Quarto
						Resultado errado	Fase pós-analítica (fase que		
						Coleta errada	Fase pré-analítica (fase que		
Assistência geral		Não efetuado quando							
Assistência geral		Incompleto / inadequad							
Distribuição		Paciente errado							
Assistência geral		Incompleto / inadequad							
Preparação		Quantidade errada							

Distribuição		Quantidade errada							
Preparação		Quantidade errada							
Preparação		Dieta errada							
						Processamento errado	Fase pré-analítica (fase que		
Procedimento / tratament		Não efetuado quando							
								Queda da própria altura	Quarto
								Queda da própria altura	Quarto
Assistência geral		Incompleto / inadequad							
Assistência geral		Incompleto / inadequad							
Assistência geral		Não efetuado quando							
Assistência geral		Incompleto / inadequad							
Requisição		Dieta errada							
						Coleta errada	Fase pré-analítica (fase que		
Preparação		Dieta errada							
Assistência geral		Incompleto / inadequad							

Assistência geral		Não efetuado quando							
Assistência geral		Incompleto / inadequad							
Administração		Paciente errado							
Assistência geral		Não efetuado quando							
Distribuição		Frequência errada							
Distribuição		Frequência errada							
Assistência geral		Não efetuado quando							
Assistência geral		Não efetuado quando							
Assistência geral		Incompleto / inadequad							
Assistência geral		Não efetuado quando							
Distribuição		Dieta errada							
Assistência geral		Incompleto / inadequad							
Assistência geral		Não efetuado quando							
		Suicídio de paciente,							

Assistência geral		Incompleto / inadequad							

Assistência geral		Incompleto / inadequad							
Assistência geral		Incompleto / inadequad							
								Queda da própria altura	Banheiro
Assistência geral		Incompleto / inadequad							
Preparação		Quantidade errada							
Assistência geral		Não efetuado quando							
Assistência geral		Não efetuado quando							
Assistência geral		Não efetuado quando							
Assistência geral		Incompleto / inadequad							
Identificação do paciente		Incompleto / inadequad							
Assistência geral		Incompleto / inadequad							
Assistência geral		Não efetuado quando							
								Perda do equilíbrio	Cama
Assistência geral		Incompleto / inadequad							
Assistência geral		Não efetuado quando							
Assistência geral		Não efetuado quando							

Assistência geral		Incompleto / inadequad							
								Queda da própria altura	Cama
Assistência geral		Não efetuado quando							
Assistência geral		Incompleto / inadequad							
Assistência geral		Incompleto / inadequad							
Assistência geral		Não efetuado quando							
Assistência geral		Não efetuado quando							
Assistência geral		Não efetuado quando							
Assistência geral		Incompleto / inadequad							
Distribuição		Frequência errada							
Preparação		Consistência errada							
								Queda da própria altura	Banheiro
Administração		Não administração							
Administração		Não administração							

								Queda da própria altura	Banheiro
Assistência geral		Não efetuado quando							
Assistência geral		Não efetuado quando							
Assistência geral		Não efetuado quando							
								Queda da própria altura	Banheiro
Preparação		Dieta errada							
Diagnóstico / Meios compleme		Não efetuado quando							
								Queda da própria altura	Cama
Administração		Consistência errada							
Assistência geral		Não efetuado quando							
Preparação		Consistência errada							
Preparação		Consistência errada							
Assistência geral		Incompleto / inadequad							
Administração		Consistência errada							
Assistência geral		Incompleto / inadequad							

Assistênci a geral		Não efetuado quando							
Assistênci a geral		Não efetuado quando							
								Queda da própria altura	Quarto
Preparaçã o		Consistên cia errada							
Assistênci a geral		Incomplet o / inadequad							
Assistênci a geral		Incomplet o / inadequad							
								Desmaio	Banheiro
Assistênci a geral		Não efetuado quando							
Diagnóstic o / Meios compleme		Incomplet o / inadequad							
Assistênci a geral		Incomplet o / inadequad							
Preparaçã o		Dieta errada							

Assistência geral		Incompleto / inadequad							
Assistência geral		Incompleto / inadequad							
								Escorregar	Berço
								Queda da própria altura	Cama
								Queda da própria altura	Cadeira
								Queda da própria altura	Cama
								Queda da própria altura	Berço

								Queda da própria altura	Cama
Distribuição		Dieta errada							
Distribuição		Não administração							
Distribuição		Dieta errada							
Distribuição		Dieta errada							
Distribuição		Dieta errada							
Distribuição		Dieta errada							
Assistência geral		Não efetuado quando							
Preparação		Consistência errada							
Assistência geral		Não efetuado quando							
Preparação		Dieta errada							
Assistência geral		Não efetuado quando							
Assistência geral		Incompleto / inadequad							
Assistência geral		Não efetuado quando							
Assistência geral		Incompleto / inadequad							

Procedim ento / tratament		Não efetuado quando							
Assistênci a geral		Não efetuado quando							
Assistênci a geral		Incomplet o / inadequad							
Assistênci a geral		Incomplet o / inadequad							
Assistênci a geral		Não efetuado quando							
						Resultado errado	Fase pós-analítica (fase que		
						Processa mento errado	Fase analítica (conjunto		

Distribuição		Paciente errado							
								Queda da própria altura	Banheiro
								Perda do equilíbrio	Cama
								Queda da própria altura	Banheiro
Assistência geral		Não efetuado quando							
Assistência geral		Incompleto / inadequad							

Assistência geral		Não efetuado quando							
Assistência geral		Não efetuado quando							
Assistência geral		Paciente errado							
Assistência geral		Incompleto / inadequad							
Assistência geral		Incompleto / inadequad							
Assistência geral		Não efetuado quando							
								Queda da própria altura	Banheiro
								Queda da própria altura	Banheiro
								Escorregar	Cama
Assistência geral		Não efetuado quando							

								Escorregar	Berço
Assistência geral		Não efetuado quando							
Assistência geral		Incompleto / inadequad							
								Queda da própria altura	Cama
Assistência geral		Não efetuado quando							
								Perda do equilíbrio	Cama
Assistência geral		Incompleto / inadequad							
								Escorregar	Berço
Distribuição		Frequência errada							
Distribuição		Frequência errada							
Assistência geral		Incompleto / inadequad							
Assistência geral		Incompleto / inadequad							

Assistênci a geral		Incomplet o / inadequad							
Distribuiç ão		Frequênci a errada							
								Escorrega r	Cama
Assistênci a geral		Não efetuado quando							
								Perda do equilíbrio	Banheiro
Assistênci a geral		Incomplet o / inadequad							

Assistênci a geral		Não efetuado quando							
Assistênci a geral		Incomplet o / inadequad							
Preparaçã o		Consistên cia errada							
Distribuiç ão		Não administra ção							
Distribuiç ão		Não administra ção							
								Queda da própria altura	Quarto
								Escorrega r	Cama
								Escorrega r	Cama

			X						
Distribuição		Dieta errada							
Distribuição		Dieta errada							
Distribuição		Frequência errada							
Assistência geral		Incompleto / inadequad							
Fornecimento		Não administração							
Administração		Quantidade errada							
Preparação		Quantidade errada							
Assistência geral		Não efetuado quando							
Assistência geral		Não efetuado quando							
Assistência geral		Incompleto / inadequad							
								Escorregar	Cama

Assistência geral		Não efetuado quando							
Assistência geral		Incompleto / inadequad							
Assistência geral		Não efetuado quando							
Assistência geral		Não efetuado quando							
Distribuição		Quantidade errada							
Assistência geral		Incompleto / inadequad							
Assistência geral		Incompleto / inadequad							
Assistência geral		Incompleto / inadequad							

Assistência geral		Incompleto / inadequad							
Assistência geral		Incompleto / inadequad							
								Escorregar	Berço
								Escorregar	Cama
								Escorregar	Cadeira
Assistência geral		Incompleto / inadequad							
Assistência geral		Incompleto / inadequad							
Assistência geral		Não efetuado quando							
Assistência geral		Não efetuado quando							
Assistência geral		Incompleto / inadequad							

Assistência geral		Incompleto / inadequad							
Assistência geral		Incompleto / inadequad							
Assistência geral		Incompleto / inadequad							
Assistência geral		Incompleto / inadequad							
Assistência geral		Incompleto / inadequad							
Assistência geral		Incompleto / inadequad							
Assistência geral		Incompleto / inadequad							
Preparação		Dieta errada							
Assistência geral		Incompleto / inadequad							
Assistência geral		Não efetuado quando							
Assistência geral		Incompleto / inadequad							
Assistência geral		Paciente errado							
Preparação		Armazenamento errado							
Assistência geral		Não efetuado quando							
Assistência geral		Incompleto / inadequad							
Distribuição		Frequência errada							

Assistência geral		Incompleto / inadequad							
Requisição		Frequência errada							
								Perda do equilíbrio	Cadeira
Assistência geral		Incompleto / inadequad							
								Escorregar	Cama
Assistência geral		Incompleto / inadequad							
Assistência geral		Procedimento / tratament							
Distribuição		Frequência errada							
Distribuição		Frequência errada							
Assistência geral		Incompleto / inadequad							

Distribuição		Dieta errada							
Assistência geral		Incompleto / inadequad							
Distribuição		Frequência errada							
Prescrição		Frequência errada							

Distribuição		Frequência errada							
Distribuição		Frequência errada							
Distribuição		Frequência errada							
Distribuição		Dieta errada							
Distribuição		Frequência errada							
Assistência geral		Incompleto / inadequad							
Assistência geral		Incompleto / inadequad							
Assistência geral		Incompleto / inadequad							
Assistência geral		Incompleto / inadequad							
Assistência geral		Incompleto / inadequad							
Assistência geral		Incompleto / inadequad							
Assistência geral		Incompleto / inadequad							
Assistência geral		Incompleto / inadequad							
Assistência geral		Incompleto / inadequad							
Assistência geral		Incompleto / inadequad							
Assistência geral		Incompleto / inadequad							
Assistência geral		Incompleto / inadequad							
Assistência geral		Paciente errado							
								Perda do equilíbrio	Cama

Assistênci a geral		Paciente errado							
Assistênci a geral		Incomplet o / inadequad							
Assistênci a geral		Paciente errado							
Assistênci a geral		Não efetuado quando							
Assistênci a geral		Não efetuado quando							
Preparaçã o		Quantidad e errada							
Assistênci a geral		Incomplet o / inadequad							
Assistênci a geral		Indisponív el							
								Perda do equilíbrio	Cama
Assistênci a geral		Não efetuado quando							

Assistênci a geral		Incomplet o / inadequad							
Assistênci a geral		Incomplet o / inadequad							

Assistênci a geral		Incomplet o / inadequad							
Procedim ento / tratament		Não efetuado quando							
								Escorrega r	Banheiro
Assistênci a geral		Não efetuado quando							
Assistênci a geral		Não efetuado quando							
Assistênci a geral		Incomplet o / inadequad							
Assistênci a geral		Incomplet o / inadequad							
Assistênci a geral		Incomplet o / inadequad							
Assistênci a geral		Incomplet o / inadequad							
Assistênci a geral		Incomplet o / inadequad							
Assistênci a geral		Indisponív el							
Assistênci a geral		Incomplet o / inadequad							
Assistênci a geral		Incomplet o / inadequad							
Assistênci a geral		Não efetuado quando							
Assistênci a geral		Incomplet o / inadequad							

Assistência geral		Incompleto / inadequad							
Diagnóstico / Meios compleme		Paciente errado							
Assistência geral		Não efetuado quando							
Assistência geral		Incompleto / inadequad							
Assistência geral		Não efetuado quando							
Assistência geral		Não efetuado quando							
Assistência geral		Incompleto / inadequad							
Diagnóstico / Meios compleme		Incompleto / inadequad							
Assistência geral		Incompleto / inadequad							
Assistência geral		Incompleto / inadequad							
Assistência geral		Incompleto / inadequad							
Assistência geral		Incompleto / inadequad							
Assistência geral		Incompleto / inadequad							
Assistência geral		Paciente errado							

								Queda da própria altura	Banheiro
Assistência geral		Incompleto / inadequad							
Assistência geral		Incompleto / inadequad							
Distribuição		Frequência errada							
Assistência geral		Incompleto / inadequad							
Assistência geral		Incompleto / inadequad							
Assistência geral		Não efetuado quando							
Assistência geral		Não efetuado quando							

Assistência geral		Não efetuado quando							
Assistência geral		Não efetuado quando							
Assistência geral		Não efetuado quando							
Assistência geral		Não efetuado quando							
Assistência geral		Incompleto / inadequad							
Distribuição		Frequência errada							
Assistência geral		Incompleto / inadequad							
Distribuição		Frequência errada							

Assistênci a geral		Incomplet o / inadequad							
Assistênci a geral		Não efetuado quando							
Assistênci a geral		Incomplet o / inadequad							
Assistênci a geral		Incomplet o / inadequad							
Assistênci a geral		Incomplet o / inadequad							
Assistênci a geral		Incomplet o / inadequad							
Assistênci a geral		Paciente errado							
								Escorrega r	Cama
								Perda do equilíbrio	Cama
Administr ação		Dieta errada							
Assistênci a geral		Não efetuado quando							

Assistência geral		Incompleto / inadequad							
Assistência geral		Incompleto / inadequad							
Assistência geral		Incompleto / inadequad							
Assistência geral		Incompleto / inadequad							
								Escorregar	Cama
Assistência geral		Incompleto / inadequad							
Assistência geral		Incompleto / inadequad							
								Escorregar	Enquanto transportado /
Assistência geral		Incompleto / inadequad							
								Escorregar	Cadeira
								Queda da própria altura	Cama
Procedimento / tratament		Procedimento / tratament							
Procedimento / tratament		Procedimento / tratament							
Procedimento / tratament		Procedimento / tratament							
Procedimento / tratament		Procedimento / tratament							
Procedimento / tratament		Procedimento / tratament							
Procedimento / tratament		Procedimento / tratament							

Procedimento / tratament		Procedimento / tratament							
Procedimento / tratament		Procedimento / tratament							
Procedimento / tratament		Procedimento / tratament							
Procedimento / tratament		Procedimento / tratament							
Procedimento / tratament		Procedimento / tratament							
Procedimento / tratament		Procedimento / tratament							
								Escorregar	Enquanto transportado /
								Escorregar	Banheiro

Administr ação		Frequênci a errada							
Assistênci a geral		Incomplet o / inadequad							

Assistênci a geral		Incomplet o / inadequad							
Distribuiç ão		Frequênci a errada							
Assistênci a geral		Incomplet o / inadequad							
					X				
Assistênci a geral		Incomplet o / inadequad							
Assistênci a geral		Incomplet o / inadequad							
Assistênci a geral		Incomplet o / inadequad							
Assistênci a geral		Paciente errado							
Administr ação		Não administra ção							
Assistênci a geral		Incomplet o / inadequad							
Assistênci a geral		Não efetuado quando							
Distribuiç ão		Não administra ção							
Preparaçã o		Não administra ção							
Assistênci a geral		Não efetuado quando							
Assistênci a geral		Incomplet o / inadequad							
Assistênci a geral		Incomplet o / inadequad							
Assistênci a geral		Incomplet o / inadequad							

Diagnóstico / Meios compleme		Procedimento / tratament							
Assistência geral		Incompleto / inadequad							
Assistência geral		Incompleto / inadequad							
Assistência geral		Incompleto / inadequad							
Preparação		Quantidade errada							
						Processamento errado	Fase pré-analítica (fase que		
Assistência geral		Incompleto / inadequad							
Assistência geral		Incompleto / inadequad							
Assistência geral		Incompleto / inadequad							

Assistência geral		Incompleto / inadequad							
Assistência geral		Incompleto / inadequad							
Assistência geral		Incompleto / inadequad							

Assistência geral		Incompleto / inadequad							
Assistência geral		Incompleto / inadequad							
Assistência geral		Incompleto / inadequad							
Assistência geral		Incompleto / inadequad							
Assistência geral		Incompleto / inadequad							
Assistência geral		Incompleto / inadequad							
Assistência geral		Incompleto / inadequad							
Assistência geral		Incompleto / inadequad							
Assistência geral		Incompleto / inadequad							
Assistência geral		Incompleto / inadequad							
								Queda da própria altura	Cama
								Escorregar	Maca
								Queda da própria altura	Banheiro
								Queda da própria altura	Cama
								Queda da própria altura	Cama
Assistência geral		Não efetuado quando							
Assistência geral		Não efetuado quando							
Distribuição		Frequência errada							

Assistência geral		Incompleto / inadequad							
Assistência geral		Não efetuado quando							
								Queda da própria altura	Cama
Assistência geral		Incompleto / inadequad							
Preparação		Dieta errada							
								Perda do equilíbrio	Banheiro
								Perda do equilíbrio	Banheiro
Distribuição		Quantidade errada							
Assistência geral		Incompleto / inadequad							
Assistência geral		Incompleto / inadequad							

								Queda da própria altura	Berço
Assistência geral		Incompleto / inadequad							
Assistência geral		Incompleto / inadequad							
Preparação		Armazenamento errado							
Assistência geral		Incompleto / inadequad							
Assistência geral		Incompleto / inadequad							
Assistência geral		Não efetuado quando							
Assistência geral		Incompleto / inadequad							
Assistência geral		Incompleto / inadequad							
Procedimento / tratament		Procedimento / tratament							
Procedimento / tratament		Procedimento / tratament							
Procedimento / tratament		Procedimento / tratament							
Procedimento / tratament		Procedimento / tratament							

Assistência geral		Não efetuado quando							
								Perda do equilíbrio	Cama
Assistência geral		Não efetuado quando							
Assistência geral		Não efetuado quando							
Assistência geral		Não efetuado quando							
Preparação		Quantidade errada							
Assistência geral		Incompleto / inadequad							
Assistência geral		Incompleto / inadequad							
Assistência geral		Incompleto / inadequad							

								Queda da própria altura	Banheiro
								Perda do equilíbrio	Cama
								Perda do equilíbrio	Cama
								Queda da própria altura	Banheiro
Assistência geral		Incompleto / inadequad							
Assistência geral		Incompleto / inadequad							
								Queda da própria altura	Cadeira
Assistência geral		Incompleto / inadequad							
Assistência geral		Paciente errado							

Assistência geral		Não efetuado quando							
Assistência geral		Não efetuado quando							
								Queda da própria altura	Quarto
Assistência geral		Incompleto / inadequad							
Assistência geral		Incompleto / inadequad							

								Escorregar	Cama
Distribuição		Consistência errada							
Assistência geral		Não efetuado quando							
Assistência geral		Não efetuado quando							
Assistência geral		Incompleto / inadequad							
Assistência geral		Não efetuado quando							
Distribuição		Consistência errada							
Assistência geral		Incompleto / inadequad							
Assistência geral		Incompleto / inadequad							
Assistência geral		Incompleto / inadequad							

Assistênci a geral		Não efetuado quando							
Distribuiç ão		Não administra ção							
Assistênci a geral		Incomplet o / inadequad							
Assistênci a geral		Incomplet o / inadequad							
Distribuiç ão		Quantidad e errada							
Assistênci a geral		Incomplet o / inadequad							
Assistênci a geral		Incomplet o / inadequad							

Procedimento / tratament		Incompleto / inadequad							
Procedimento / tratament		Incompleto / inadequad							
Procedimento / tratament		Incompleto / inadequad							
Procedimento / tratament		Incompleto / inadequad							
Procedimento / tratament		Incompleto / inadequad							
Procedimento / tratament		Incompleto / inadequad							
Procedimento / tratament		Incompleto / inadequad							
Assistência geral		Não efetuado quando							
Procedimento / tratament		Não efetuado quando							
Procedimento / tratament		Não efetuado quando							
transferência de paciente		Incompleto / inadequad							
Assistência geral		Incompleto / inadequad							
Assistência geral		Não efetuado quando							
Assistência geral		Não efetuado quando							
Assistência geral		Não efetuado quando							
Assistência geral		Não efetuado quando							

Assistência geral		Não efetuado quando							
Assistência geral		Não efetuado quando							
Assistência geral		Paciente errado							
Assistência geral		Incompleto / inadequad							
Distribuição		Frequência errada							
Procedimento / tratament		Incompleto / inadequad							
Procedimento / tratament		Incompleto / inadequad							
Procedimento / tratament		Não efetuado quando							
Alta		Incompleto / inadequad							
Procedimento / tratament		Incompleto / inadequad							
Assistência geral		Incompleto / inadequad							
Procedimento / tratament		Incompleto / inadequad							

Assistência geral		Incompleto / inadequad							
Diagnóstico / Meios compleme		Incompleto / inadequad							
Marcação		Não efetuado quando							
Marcação		Não efetuado quando							
Assistência geral		Não efetuado quando							
Assistência geral		Não efetuado quando							
Assistência geral		Não efetuado quando							
Assistência geral		Não efetuado quando							
Assistência geral		Não efetuado quando							
Assistência geral		Não efetuado quando							
Assistência geral		Não efetuado quando							
Assistência geral		Não efetuado quando							
Assistência geral		Incompleto / inadequad							
Marcação		Não efetuado quando							
Assistência geral		Não efetuado quando							
Diagnóstico / Meios compleme		Não efetuado quando							

Assistênci a geral		Incomplet o / inadequad							
Assistênci a geral		Incomplet o / inadequad							
Assistênci a geral		Incomplet o / inadequad							
Assistênci a geral		Incomplet o / inadequad							
Assistênci a geral		Incomplet o / inadequad							
								Queda da própria altura	Cama
Requisiçã o		Dieta errada							

Distribuição		Dieta errada							
transferência de paciente		Incompleto / inadequad							
Assistência geral		Não efetuado quando							
Regulação / referencia		Paciente errado							
Assistência geral		Não efetuado quando							
Assistência geral		Não efetuado quando							
Assistência geral		Não efetuado quando							
Assistência geral		Não efetuado quando							
Assistência geral		Incompleto / inadequad							
Assistência geral		Incompleto / inadequad							
Assistência geral		Incompleto / inadequad							
						Coleta errada	Fase pré-analítica (fase que		
	Mecanismos de identificaç	Documento entregue							

Regulação / referencia		Não efetuado quando							
transferência de paciente		Não efetuado quando							
Distribuição		Frequência errada							
Assistência geral		Não efetuado quando							
Assistência geral		Incompleto / inadequad							
Assistência geral		Incompleto / inadequad							
Assistência geral		Incompleto / inadequad							
transferência de paciente		Incompleto / inadequad							
Assistência geral		Incompleto / inadequad							
Assistência geral		Incompleto / inadequad							
				X					
Assistência geral		Incompleto / inadequad							

				X					
	Requisições / pedidos	Documento em falta ou							
Procedimento / tratament		Paciente errado							
Regulação / referencia		Não efetuado quando							
								Queda da própria altura	Cama
		Hemorragia após a cirurgia							
	Relatórios / resultados	Atraso no acesso ao							
				X					
Assistência geral		Incompleto / inadequad							
Assistência geral		Não efetuado quando							
Regulação / referencia		Incompleto / inadequad							
Assistência geral		Incompleto / inadequad							
Assistência geral		Incompleto / inadequad							
Assistência geral		Incompleto / inadequad							
Assistência geral		Não efetuado quando							
Assistência geral		Não efetuado quando							
Assistência geral		Não efetuado quando							

Assistência geral		Não efetuado quando							
Assistência geral		Não efetuado quando							
								Escorregar	Cama
Procedimento / tratament		Incompleto / inadequad							
	Mecanismos de identificaç	Documento entregue							
Assistência geral		Incompleto / inadequad							
Regulação / referencia		Incompleto / inadequad							
Diagnóstico / Meios compleme		Incompleto / inadequad							
Regulação / referencia		Processo errado							
Assistência geral		Incompleto / inadequad							
Assistência geral		Incompleto / inadequad							
Assistência geral		Incompleto / inadequad							
Regulação / referencia		Processo errado							
	Requisições / pedidos	Documento em falta ou							

	Requisiçõ es / pedidos	Document o em falta ou							
Assistênci a geral		Incomplet o / inadequad							
Assistênci a geral		Incomplet o / inadequad							
				X					
Distribuiç ão		Frequênci a errada							
								Perda do equilíbrio	Cama
Assistênci a geral		Incomplet o / inadequad							
Identificaç ão do paciente		Incomplet o / inadequad							
	Requisiçõ es / pedidos	Document o em falta ou							
	Requisiçõ es / pedidos	Document o em falta ou							
Assistênci a geral		Não efetuado quando							
Assistênci a geral		Incomplet o / inadequad							
Assistênci a geral		Incomplet o / inadequad							
Assistênci a geral		Incomplet o / inadequad							
Assistênci a geral		Incomplet o / inadequad							

Assistênci a geral		Incomplet o / inadequad							
Assistênci a geral		Incomplet o / inadequad							
Assistênci a geral		Incomplet o / inadequad							
Assistênci a geral		Incomplet o / inadequad							
Assistênci a geral		Incomplet o / inadequad							
Assistênci a geral		Incomplet o / inadequad							
Assistênci a geral		Incomplet o / inadequad							
Assistênci a geral		Incomplet o / inadequad							
Assistênci a geral		Incomplet o / inadequad							
Assistênci a geral		Incomplet o / inadequad							
Assistênci a geral		Incomplet o / inadequad							
Assistênci a geral		Incomplet o / inadequad							
Assistênci a geral		Incomplet o / inadequad							
Assistênci a geral		Incomplet o / inadequad							
						Coleta errada	Fase pré-analítica (fase que		
	Tabelas / registros médicos /	Informaçõ es ambígua							

	Requisições / pedidos	Documento em falta ou							
Assistência geral		Incompleto / inadequad							
Alta		Incompleto / inadequad							
Entrega		Quantidade errada							
								Escorregar	Banheiro
								Queda da própria altura	Cama
Assistência geral		Paciente errado							
Entrega		Frequência errada							
	Relatórios / resultados	Documento em falta ou							
Regulação / referencia		Não efetuado quando							
Assistência geral		Não efetuado quando							
Marcação		Não efetuado quando							
Marcação		Não efetuado quando							
								Queda da própria altura	Enquanto transportado /
Assistência geral		Não efetuado quando							
Assistência geral		Não efetuado quando							
Assistência geral		Incompleto / inadequad							
Assistência geral		Não efetuado quando							

Assistênci a geral		Não efetuado quando							
Regulaçã o / referencia		Não efetuado quando							
Assistênci a geral		Não efetuado quando							
Assistênci a geral		Não efetuado quando							
Assistênci a geral		Não efetuado quando							
Assistênci a geral		Não efetuado quando							
Assistênci a geral		Incomplet o / inadequad							
Assistênci a geral		Incomplet o / inadequad							
Assistênci a geral		Incomplet o / inadequad							
Assistênci a geral		Incomplet o / inadequad							
Procedim ento / tratament		Não efetuado quando							
Assistênci a geral		Incomplet o / inadequad							
Assistênci a geral		Incomplet o / inadequad							
				X					

transferência de paciente		Não efetuado quando							
								Escorregar	Cama
Assistência geral		Não efetuado quando							
Admissão		Não efetuado quando							
								Escorregar	Cama
Procedimento / tratament		Procedimento / tratament							
Diagnóstico / Meios compleme		Não efetuado quando							
Marcação		Não efetuado quando							
Assistência geral		Incompleto / inadequad							
Regulação / referencia		Não efetuado quando							
Assistência geral		Incompleto / inadequad							
Assistência geral		Não efetuado quando							
Regulação / referencia		Incompleto / inadequad							
Alta		Processo errado							
								Queda da própria altura	Quarto

Distribuição		Dieta errada							
Distribuição		Dieta errada							
Distribuição		Frequência errada							
transferência de paciente		Incompleto / inadequad							
Assistência geral		Incompleto / inadequad							
Procedimento / tratament		Incompleto / inadequad							
Assistência geral		Não efetuado quando							
Procedimento / tratament		Procedimento / tratament							
Assistência geral		Não efetuado quando							
Assistência geral		Não efetuado quando							
Assistência geral		Não efetuado quando							
Assistência geral		Não efetuado quando							
Assistência geral		Não efetuado quando							
Assistência geral		Não efetuado quando							
Assistência geral		Não efetuado quando							
Distribuição		Frequência errada							
Assistência geral		Incompleto / inadequad							

Assistência geral		Incompleto / inadequad							
								Queda da própria altura	Banheiro
								Queda da própria altura	Banheiro
Assistência geral		Incompleto / inadequad							
Assistência geral		Não efetuado quando							
Assistência geral		Incompleto / inadequad							
Assistência geral		Incompleto / inadequad							
Assistência geral		Incompleto / inadequad							
Distribuição		Frequência errada							
Distribuição		Frequência errada							
Distribuição		Dieta errada							
Distribuição		Dieta errada							
Assistência geral		Não efetuado quando							
								Perda do equilíbrio	Enquanto transportado /
				X					
								Queda da própria altura	Enquanto transportado /
								Queda da própria altura	Cama

Assistência geral		Não efetuado quando							
Marcação		Incompleto / inadequad							
Assistência geral		Incompleto / inadequad							
Assistência geral		Não efetuado quando							
Assistência geral		Incompleto / inadequad							
Assistência geral		Não efetuado quando							
Assistência geral		Procedimento / tratament							
Assistência geral		Incompleto / inadequad							
Assistência geral		Incompleto / inadequad							
Assistência geral		Incompleto / inadequad							

Assistênci a geral		Não efetuado quando							
Assistênci a geral		Não efetuado quando							
Assistênci a geral		Incomplet o / inadequad							
Assistênci a geral		Não efetuado quando							
Assistênci a geral		Não efetuado quando							
Diagnóstic o / Meios compleme		Incomplet o / inadequad							
Procedim ento / tratament		Procedim ento / tratament							
Assistênci a geral		Incomplet o / inadequad							
Assistênci a geral		Não efetuado quando							
Procedim ento / tratament		Procedim ento / tratament							
Assistênci a geral		Não efetuado quando							
Assistênci a geral		Não efetuado quando							

Assistência geral		Não efetuado quando							
transferência de paciente		Não efetuado quando							
Assistência geral		Incompleto / inadequad							
Procedimento / tratament		Procedimento / tratament							
Procedimento / tratament		Procedimento / tratament							
Regulação / referencia		Processo errado							
Regulação / referencia		Processo errado							
	Rótulos / etiquetas	Documento entregue							
Assistência geral		Não efetuado quando							
	Mecanismos de identificaç	Documento em falta ou							
Assistência geral		Incompleto / inadequad							
								Escorregar	Cama
Assistência geral		Incompleto / inadequad							

								Escorrega r	Cama
								Perda do equilíbrio	Quarto
	Tabelas / registros médicos /	Document o entregue							
Procedim ento / tratament		Não efetuado quando							
Assistênci a geral		Incomplet o / inadequad							
								Queda da própria altura	Banheiro
Assistênci a geral		Incomplet o / inadequad							
Assistênci a geral		Incomplet o / inadequad							
Assistênci a geral		Não efetuado quando							
	Relatórios / resultados	Document o em falta ou							
Distribuiç ão		Frequênci a errada							
transferên cia de paciente		Não efetuado quando							
						Coleta errada	Fase pré- analítica (fase que		

Assistência geral		Incompleto / inadequad							
Procedimento / tratament		Não efetuado quando							
Assistência geral		Incompleto / inadequad							
Assistência geral		Incompleto / inadequad							
								Queda da própria altura	Banheiro
Assistência geral		Não efetuado quando							
						Coleta errada	Fase pré-analítica (fase que		
								Queda da própria altura	Cama
								Queda da própria altura	Banheiro
				X					
Procedimento / tratament		Incompleto / inadequad							
Assistência geral		Incompleto / inadequad							
Assistência geral		Incompleto / inadequad							

Assistência geral		Incompleto / inadequad							
Regulação / referencia		Não efetuado quando							
Assistência geral		Não efetuado quando							
Assistência geral		Não efetuado quando							
Assistência geral		Incompleto / inadequad							
Procedimento / tratament		Não efetuado quando							
								Queda da própria altura	Banheiro
Assistência geral		Procedimento / tratament							
Assistência geral		Não efetuado quando							
				X					
								Queda da própria altura	Banheiro

				X					
Assistência geral		Incompleto / inadequad							
transferência de paciente		Processo errado							
Assistência geral		Não efetuado quando							
transferência de paciente		Processo errado							
Assistência geral		Incompleto / inadequad							
Assistência geral		Não efetuado quando							
Alta		Processo errado							
Entrega		Consistência errada							
Assistência geral		Incompleto / inadequad							
Consentimento informado		Incompleto / inadequad							
Assistência geral		Incompleto / inadequad							
Assistência geral		Incompleto / inadequad							
Procedimento / tratament		Não efetuado quando							
								Escorregar	Berço
transferência de paciente		Não efetuado quando							
								Queda da própria altura	Cama

Procedim ento / tratament		Não efetuado quando							
Procedim ento / tratament		Não efetuado quando							
Procedim ento / tratament		Não efetuado quando							
transferên cia de paciente		Não efetuado quando							
Procedim ento / tratament		Não efetuado quando							
								Queda da própria altura	Cama
transferên cia de paciente		Não efetuado quando							
Assistênci a geral		Incomplet o / inadequad							
Procedim ento / tratament		Não efetuado quando							
Procedim ento / tratament		Não efetuado quando							
Procedim ento / tratament		Não efetuado quando							
Procedim ento / tratament		Não efetuado quando							
Procedim ento / tratament		Não efetuado quando							
Procedim ento / tratament		Não efetuado quando							
Marcação		Não efetuado quando							
Assistênci a geral		Incomplet o / inadequad							

	Requisições / pedidos	Documento em falta ou							
								Queda da própria altura	Cama
Procedimento / tratament		Incompleto / inadequad							
								Queda da própria altura	Banheiro
Procedimento / tratament		Não efetuado quando							
Diagnóstico / Meios compleme		Não efetuado quando							
Marcação		Não efetuado quando							
Procedimento / tratament		Não efetuado quando							
Assistência geral		Incompleto / inadequad							
Procedimento / tratament		Não efetuado quando							
	Listas de verificação	Informações ambígua							

Procedim ento / tratament		Não efetuado quando							
Procedim ento / tratament		Não efetuado quando							
transferên cia de paciente		Incomplet o / inadequad							
Assistênci a geral		Não efetuado quando							
Assistênci a geral		Não efetuado quando							
Assistênci a geral		Não efetuado quando							
Assistênci a geral		Não efetuado quando							
transferên cia de paciente		Não efetuado quando							

Procedimento / tratament		Incompleto / inadequad							
		Hemorragia após a cirurgia							
Assistência geral		Não efetuado quando							
Assistência geral		Não efetuado quando							
								Queda da própria altura	Banheiro

transferência de paciente		Não efetuado quando							

Assistênci a geral		Incomplet o / inadequad							
Assistênci a geral		Incomplet o / inadequad							
Assistênci a geral		Incomplet o / inadequad							
Assistênci a geral		Incomplet o / inadequad							
Assistênci a geral		Incomplet o / inadequad							
Assistênci a geral		Incomplet o / inadequad							
Assistênci a geral		Incomplet o / inadequad							
Assistênci a geral		Incomplet o / inadequad							
Assistênci a geral		Incomplet o / inadequad							
Assistênci a geral		Não efetuado quando							

Assistênci a geral		Incomplet o / inadequad							
Assistênci a geral		Não efetuado quando							
Assistênci a geral		Incomplet o / inadequad							
Assistênci a geral		Não efetuado quando							
Assistênci a geral		Não efetuado quando							
Assistênci a geral		Não efetuado quando							
Assistênci a geral		Não efetuado quando							
Assistênci a geral		Não efetuado quando							
Assistênci a geral		Não efetuado quando							
Assistênci a geral		Não efetuado quando							
Assistênci a geral		Incomplet o / inadequad							
Assistênci a geral		Incomplet o / inadequad							
Assistênci a geral		Não efetuado quando							
Assistênci a geral		Não efetuado quando							

Assistência geral		Incompleto / inadequad							
Assistência geral		Não efetuado quando							
Assistência geral		Incompleto / inadequad							
Assistência geral		Incompleto / inadequad							
		Hemorragia após a cirurgia							

		Hemorragia após a cirurgia							
		Hemorragia após a cirurgia							
		Hemorragia após a cirurgia							
		Hemorragia após a cirurgia							
		Hemorragia após a cirurgia							
		Hemorragia após a cirurgia							
		Hemorragia após a cirurgia							
		Hemorragia após a cirurgia							
		Hemorragia após a cirurgia							

		Hemorragia após a cirurgia							
		Hemorragia após a cirurgia							
		Hemorragia após a cirurgia							
Alta		Não efetuado quando							
Assistência geral		Não efetuado quando							
	Requisições / pedidos	Documento em falta ou							

	Tabelas / registros médicos /	Documento em falta ou							
	Relatórios / resultados	Documento em falta ou							
	Formulários	Documento em falta ou							
Assistência geral		Não efetuado quando							
Administração		Paciente errado							
	Requisições / pedidos	Informações ambígua							
	Requisições / pedidos	Atraso no acesso ao							
	Requisições / pedidos	Documento em falta ou							
Assistência geral		Incompleto / inadequad							
Assistência geral		Não efetuado quando							
								Queda da própria altura	Cama
						Coleta errada	Fase pré-analítica (fase que		

		Outras falhas na assistênci							
Distribuição		Frequência errada							
Assistência geral		Não efetuado quando							
	Mecanismos de identificaç	Informações ambígua							
Assistência geral		Incompleto / inadequad							
Regulação / referencia		Processo errado							
Assistência geral		Incompleto / inadequad							
Distribuição		Dieta errada							
Distribuição		Quantidade errada							
Distribuição		Quantidade errada							
Distribuição		Quantidade errada							
						Coleta errada	Fase pré-analítica (fase que		
Assistência geral		Não efetuado quando							
Preparação		Dieta errada							
								Escorregar	Enquanto transportado /
		Outras falhas na assistênci							
								Escorregar	Berço
						Coleta errada	Fase pré-analítica (fase que		
Procedimento / tratament		Não efetuado quando							

transferência de paciente		Incompleto / inadequad							
Assistência geral		Incompleto / inadequad							
								Tropeçar	Banheiro
Regulação / referencia		Incompleto / inadequad							
Regulação / referencia		Incompleto / inadequad							
Regulação / referencia		Processo errado							
Regulação / referencia		Processo errado							
Regulação / referencia		Processo errado							
Assistência geral		Não efetuado quando							
Assistência geral		Não efetuado quando							
								Escorregar	Banheiro
								Queda da própria altura	Cama
								Escorregar	Banheiro
								Escorregar	Banheiro
								Escorregar	Escadas / degraus
								Escorregar	Banheiro

								Escorregar	Banheiro
Procedimento / tratament		Incompleto / inadequad							
Procedimento / tratament		Incompleto / inadequad							
Procedimento / tratament		Incompleto / inadequad							
Procedimento / tratament		Incompleto / inadequad							
Procedimento / tratament		Incompleto / inadequad							
Procedimento / tratament		Incompleto / inadequad							
Procedimento / tratament		Incompleto / inadequad							
Procedimento / tratament		Incompleto / inadequad							
Procedimento / tratament		Incompleto / inadequad							
Procedimento / tratament		Incompleto / inadequad							
						Coleta errada	Fase pré-analítica (fase que		
						Coleta errada	Fase pré-analítica (fase que		

Assistência geral		Não efetuado quando							
Procedimento / tratament		Não efetuado quando							
Assistência geral		Não efetuado quando							
Assistência geral		Não efetuado quando							
Assistência geral		Incompleto / inadequad							
Assistência geral		Incompleto / inadequad							
Assistência geral		Não efetuado quando							
Assistência geral		Não efetuado quando							
Assistência geral		Não efetuado quando							
Assistência geral		Incompleto / inadequad							
Assistência geral		Não efetuado quando							
Assistência geral		Não efetuado quando							
Assistência geral		Não efetuado quando							
Procedimento / tratament		Não efetuado quando							
Assistência geral		Incompleto / inadequad							
Assistência geral		Não efetuado quando							

Assistênci a geral		Não efetuado quando							
Assistênci a geral		Não efetuado quando							
Procedim ento / tratament		Não efetuado quando							
Assistênci a geral		Não efetuado quando							
Assistênci a geral		Não efetuado quando							
				X					
						Outras falhas ocorridas	Fase pré-analítica (fase que		
Assistênci a geral		Não efetuado quando							
Administr ação		Frequênci a errada							

Assistência geral		Não efetuado quando							
	Formulários	Documento entregue							
						Outras falhas ocorridas	Fase pré-analítica (fase que		
		Outras falhas na assistênci							

Assistência geral		Incompleto / inadequad							
Assistência geral		Incompleto / inadequad							

Assistência geral		Incompleto / inadequad							
transferência de paciente		Incompleto / inadequad							

	Tabelas / registros médicos /	Informações ambígua							
								Queda da própria altura	Banheiro
Assistência geral		Não efetuado quando							
Diagnóstico / Meios compleme		Não efetuado quando							
Identificação do paciente		Incompleto / inadequad							
Assistência geral		Não efetuado quando							
	Relatórios / resultados	Documento em falta ou							
Distribuição		Frequência errada							
Assistência geral		Não efetuado quando							
Procedimento / tratament		Incompleto / inadequad							
Procedimento / tratament		Incompleto / inadequad							

Diagnóstico / Meios compleme		Incompleto / inadequad							
Assistência geral		Não efetuado quando							
Assistência geral		Não efetuado quando							
Assistência geral		Não efetuado quando							
Assistência geral		Incompleto / inadequad							
Assistência geral		Não efetuado quando							
Distribuição		Quantidade errada							
Entrega		Frequência errada							
Assistência geral		Procedimento / tratament							
								Perda do equilíbrio	Quarto
Diagnóstico / Meios compleme		Incompleto / inadequad							
Administração		Paciente errado							
								Escorregar	Banheiro
		Outras falhas na assistênci							
Assistência geral		Não efetuado quando							
Procedimento / tratament		Não efetuado quando							

Marcação		Processo errado							
Diagnóstico / Meios compleme		Incompleto / inadequad							
Assistência geral		Incompleto / inadequad							
								Queda da própria altura	Cama
								Desmaio	Banheiro
						Coleta errada	Fase pré-analítica (fase que		
Assistência geral		Incompleto / inadequad							
Marcação		Não efetuado quando							
								Perda do equilíbrio	Cama
	Relatórios / resultados	Documento entregue							
Marcação		Processo errado							

Marcação		Não efetuado quando							
Assistênci a geral		Incomplet o / inadequad							
								Queda da própria altura	Berço
	Relatórios / resultados	Document o entregue							
Procedim ento / tratament		Não efetuado quando							
	Relatórios / resultados	Document o entregue							
						Coleta errada	Fase pré-analítica (fase que		
								Escorrega r	Cama

Assistênci a geral		Incomplet o / inadequad							
Regulaçã o / referencia		Processo errado							
Regulaçã o / referencia		Processo errado							
Assistênci a geral		Não efetuado quando							
Assistênci a geral		Não efetuado quando							
Assistênci a geral		Não efetuado quando							
Assistênci a geral		Não efetuado quando							
Assistênci a geral		Não efetuado quando							
Assistênci a geral		Não efetuado quando							
Assistênci a geral		Não efetuado quando							
Assistênci a geral		Não efetuado quando							
Assistênci a geral		Não efetuado quando							
Marcação		Não efetuado quando							
						Coleta errada	Fase pré-analítica (fase que		

Assistência geral		Não efetuado quando							
Assistência geral		Não efetuado quando							
	Tabelas / registros médicos /	Informações ambígua							
Assistência geral		Não efetuado quando							
		Outras falhas na assistênci							
Assistência geral		Incompleto / inadequad							

Marcação		Não efetuado quando							
Regulação / referencia		Não efetuado quando							
Procedimento / tratament		Não efetuado quando							
								Perda do equilíbrio	Banheiro
transferência de paciente		Processo errado							
								Escorregar	Cadeira
	Mecanismos de identificaç	Informações ambígua							
Assistência geral		Incompleto / inadequad							
Regulação / referencia		Processo errado							
Procedimento / tratament		Não efetuado quando							
Assistência geral		Não efetuado quando							
						Coleta errada	Fase pré-analítica (fase que		

	Tabelas / registros médicos /	Document o entregue							
		Hemorrag ia após a cirurgia							
transferên cia de paciente		Processo errado							
Assistênci a geral		Incomplet o / inadequad							
Assistênci a geral		Não efetuado quando							
Assistênci a geral		Não efetuado quando							
								Perda do equilíbrio	Banheiro
								Escorrega r	Banheiro
		Hemorrag ia após a cirurgia							
		Outras falhas na assistênci							
		Outras falhas na assistênci							
Assistênci a geral		Incomplet o / inadequad							
Assistênci a geral		Procedim ento / tratament							

	Tabelas / registros médicos /	Informações ambígua							
Assistência geral		Incompleto / inadequad							
								Escorregar	Banheiro
Assistência geral		Não efetuado quando							

Procedimento / tratament		Não efetuado quando							
Procedimento / tratament		Não efetuado quando							
Procedimento / tratament		Não efetuado quando							
Assistência geral		Não efetuado quando							
	Tabelas / registros médicos /	Documento entregue							
Assistência geral		Incompleto / inadequad							
Assistência geral		Incompleto / inadequad							
Regulação / referencia		Incompleto / inadequad							
								Queda da própria altura	Quarto

								Queda da própria altura	Quarto
								Perda do equilíbrio	Cama

								Queda da própria altura	Cama
								Queda da própria altura	Quarto
								Queda da própria altura	Cama
								Queda da própria altura	Cadeira

								Queda da própria altura	Banheiro
								Queda da própria altura	Quarto
								Escorregar	Cama

								Escorregar	Banheiro
								Escorregar	Banheiro
								Queda da própria altura	Cama

								Queda da própria altura	Cama
								Escorregar	Maca
								Tropeçar	Cadeira
								Perda do equilíbrio	Banheiro
								Escorregar	Banheiro
								Escorregar	Banheiro

								Perda do equilíbrio	Enquanto transportado / apoiado por outro
								Desmaio	Cama
								Perda do equilíbrio	Cama
								Perda do equilíbrio	Banheiro

								Perda do equilíbrio	Cama
								Escorrega r	Cama
								Perda do equilíbrio	Quarto
								Escorrega r	Banheiro
								Perda do equilíbrio	Banheiro
								Queda da própria altura	Quarto
								Perda do equilíbrio	Cadeira

								Queda da própria altura	Maca
								Perda do equilíbrio	Cama
								Perda do equilíbrio	Banheiro
								Desmaio	Banheiro
								Escorregar	Banheiro
								Perda do equilíbrio	Banheiro
								Escorregar	Cama
								Perda do equilíbrio	Banheiro

								Escorregar	Cama
								Perda do equilíbrio	Banheiro
								Queda da própria altura	Banheiro
								Queda da própria altura	Maca

								Desmaio	Banheiro

								Desmaio	Quarto
								Perda do equilíbrio	Quarto
								Escorregar	Cama
								Perda do equilíbrio	Cadeira
								Perda do equilíbrio	Quarto
								Perda do equilíbrio	Enquanto transportado / apoiado por outro
								Perda do equilíbrio	Cama
								Queda da própria altura	Banheiro
								Perda do equilíbrio	Cama
								Escorregar	Banheiro

								Desmaio	Quarto
								Queda da própria altura	Cama
								Queda da própria altura	Cama
								Escorregar	Banheiro
								Tropeçar	Cadeira

Queimaduras	Estágio	Transmissão de doença	Transmissão de doença	Rejeição	Transmissão de tumor	Tipo de incidente / evento	Grau do dano	Gênero	Idade
							Moderado	Masculino	de 76 a 85 anos
							Nenhum	Masculino	de 66 a 75 anos
							Moderado	Masculino	mais de 85 anos
							Nenhum	Masculino	de 66 a 75 anos
							Moderado	Masculino	de 66 a 75 anos
							Leve	Feminino	de 46 a 55 anos
							Nenhum	Masculino	de 66 a 75 anos
							Leve	Masculino	mais de 85 anos
							Nenhum	Masculino	de 66 a 75 anos
							Nenhum	Feminino	de 76 a 85 anos
							Leve	Masculino	de 66 a 75 anos
							Moderado	Masculino	de 66 a 75 anos
							Moderado	Masculino	de 36 a 45 anos
							Leve	Feminino	mais de 85 anos
							Nenhum	Feminino	de 76 a 85 anos
							Leve	Masculino	de 76 a 85 anos
							Moderado	Feminino	de 76 a 85 anos

							Moderado	Masculino	de 56 a 65 anos
							Moderado	Masculino	de 66 a 75 anos
Queimaduras.							Moderado	Masculino	de 76 a 85 anos
							Nenhum	Masculino	de 56 a 65 anos
							Nenhum	Feminino	de 56 a 65 anos
							Moderado	Feminino	de 56 a 65 anos
							Nenhum	Masculino	de 76 a 85 anos
							Leve	Feminino	de 46 a 55 anos
							Leve	Masculino	de 76 a 85 anos
Queimaduras.							Moderado	Masculino	de 26 a 35 anos
							Leve	Masculino	de 66 a 75 anos
							Nenhum	Masculino	de 36 a 45 anos
							Nenhum	Feminino	de 76 a 85 anos
							Nenhum	Masculino	de 66 a 75 anos
							Moderado	Masculino	de 66 a 75 anos
							Nenhum	Masculino	de 26 a 35 anos
							Leve	Masculino	de 56 a 65 anos
							Moderado	Masculino	de 26 a 35 anos

							Nenhum	Feminino	de 76 a 85 anos
							Nenhum	Feminino	de 18 a 25 anos
							Nenhum	Feminino	de 56 a 65 anos
							Moderado	Masculino	de 76 a 85 anos
							Moderado	Masculino	de 66 a 75 anos
							Leve	Feminino	de 66 a 75 anos
							Nenhum	Masculino	de 66 a 75 anos
							Moderado	Masculino	de 76 a 85 anos
							Moderado	Masculino	de 76 a 85 anos
							Nenhum	Masculino	de 76 a 85 anos
							Moderado	Feminino	de 56 a 65 anos
							Leve	Feminino	de 18 a 25 anos
							Moderado	Masculino	de 66 a 75 anos
							Nenhum	Feminino	de 46 a 55 anos
							Nenhum	Feminino	de 76 a 85 anos
							Leve	Feminino	de 56 a 65 anos
							Leve	Masculino	de 46 a 55 anos
							Moderado	Masculino	de 66 a 75 anos

							Leve	Masculino	de 56 a 65 anos
							Nenhum	Masculino	de 76 a 85 anos
							Leve	Feminino	de 66 a 75 anos
							Grave	Feminino	de 56 a 65 anos
							Moderado	Masculino	de 66 a 75 anos
							Nenhum	Feminino	de 76 a 85 anos
							Nenhum	Feminino	de 76 a 85 anos
							Nenhum	Feminino	de 56 a 65 anos
							Nenhum	Masculino	de 76 a 85 anos
							Grave	Masculino	de 56 a 65 anos
							Moderado	Masculino	de 36 a 45 anos
							Nenhum	Masculino	de 76 a 85 anos
							Nenhum	Feminino	de 36 a 45 anos
							Leve	Masculino	de 66 a 75 anos
							Nenhum	Masculino	de 66 a 75 anos
							Moderado	Feminino	de 66 a 75 anos
							Leve	Feminino	de 26 a 35 anos
							Nenhum	Feminino	de 66 a 75 anos

							Moderado	Masculino	de 36 a 45 anos
							Nenhum	Feminino	de 76 a 85 anos
							Nenhum	Masculino	mais de 85 anos
							Leve	Masculino	de 76 a 85 anos
							Leve	Feminino	de 66 a 75 anos
							Leve	Feminino	de 66 a 75 anos
							Moderado	Feminino	de 36 a 45 anos
							Leve	Feminino	de 66 a 75 anos
							Leve	Masculino	de 76 a 85 anos
							Leve	Feminino	de 66 a 75 anos
							Nenhum	Masculino	de 5 a 11 anos
							Nenhum	Feminino	de 56 a 65 anos
							Moderado	Masculino	de 46 a 55 anos
							Leve	Feminino	de 76 a 85 anos
							Moderado	Feminino	mais de 85 anos
							Nenhum	Masculino	de 56 a 65 anos
							Leve	Feminino	de 66 a 75 anos
							Leve	Masculino	de 66 a 75 anos

							Moderado	Masculino	de 66 a 75 anos
							Leve	Masculino	de 76 a 85 anos
							Leve	Feminino	mais de 85 anos
							Leve	Feminino	de 56 a 65 anos
							Leve	Masculino	de 66 a 75 anos
							Leve	Masculino	de 76 a 85 anos
							Leve	Feminino	de 56 a 65 anos
							Nenhum	Feminino	de 76 a 85 anos
							Nenhum	Masculino	de 66 a 75 anos
	Estágio 2 (perda parcial da						Moderado	Feminino	de 66 a 75 anos
							Leve	Feminino	de 76 a 85 anos
							Nenhum	Masculino	de 56 a 65 anos
Queimaduras.							Leve	Feminino	de 36 a 45 anos
							Leve	Masculino	de 46 a 55 anos
Queimaduras.							Moderado	Masculino	de 46 a 55 anos
							Leve	Masculino	de 56 a 65 anos
							Leve	Feminino	mais de 85 anos
	Estágio 1 (pele íntegra						Leve	Masculino	de 66 a 75 anos

	Estágio 1 (pele íntegra						Leve	Feminino	mais de 85 anos
							Leve	Masculino	de 46 a 55 anos
							Moderado	Feminino	de 26 a 35 anos
							Grave	Feminino	de 26 a 35 anos
	Estágio 4 (perda total da espessura						Grave	Masculino	de 76 a 85 anos
	Estágio 3 (perda total da espessura						Moderado	Feminino	de 56 a 65 anos
	Estágio 3 (perda total da espessura						Moderado	Masculino	de 66 a 75 anos
	Estágio 3 (perda total da espessura						Moderado	Masculino	de 76 a 85 anos
							Nenhum	Feminino	menos de 28 dias
							Nenhum	Feminino	menos de 28 dias
							Nenhum	Feminino	de 26 a 35 anos
	Estágio 3 (perda total da espessura						Moderado	Feminino	de 56 a 65 anos
	Estágio 3 (perda total da espessura						Moderado	Masculino	de 76 a 85 anos
	Estágio 3 (perda total da espessura						Moderado	Feminino	de 56 a 65 anos
	Estágio 2 (perda parcial da espessura						Moderado	Feminino	de 56 a 65 anos
	Estágio 3 (perda total da espessura						Moderado	Masculino	de 66 a 75 anos

							Leve	Masculino	de 76 a 85 anos
							Leve	Feminino	de 56 a 65 anos
							Leve	Feminino	de 56 a 65 anos
							Leve	Feminino	de 36 a 45 anos
	Estágio 3 (perda total da espessura						Moderado	Feminino	de 46 a 55 anos
	Estágio 4 (perda total da espessura						Grave	Feminino	mais de 85 anos
	Estágio 3 (perda total da espessura						Moderado	Feminino	de 66 a 75 anos
	Estágio 2 (perda parcial da espessura						Moderado	Masculino	de 46 a 55 anos
	Estágio 3 (perda total da espessura						Moderado	Masculino	de 66 a 75 anos
							Leve	Feminino	de 26 a 35 anos
							Leve	Feminino	de 46 a 55 anos
							Leve	Feminino	de 46 a 55 anos
							Leve	Feminino	de 46 a 55 anos
							Leve	Feminino	de 46 a 55 anos
							Moderado	Feminino	de 46 a 55 anos

	Estágio 3 (perda total da espessura						Moderado	Masculino	de 66 a 75 anos
	Estágio 4 (perda total da espessura						Moderado	Feminino	de 76 a 85 anos
							Moderado	Feminino	de 76 a 85 anos
	Estágio 3 (perda total da espessura						Moderado	Feminino	de 56 a 65 anos
							Leve	Feminino	de 46 a 55 anos
							Moderado	Feminino	de 46 a 55 anos
							Leve	Feminino	de 56 a 65 anos
							Leve	Feminino	menos de 28 dias
							Moderado	Feminino	de 56 a 65 anos
							Moderado	Feminino	de 46 a 55 anos
							Leve	Masculino	de 46 a 55 anos
							Moderado	Feminino	menos de 28 dias
							Moderado	Feminino	menos de 28 dias
							Leve	Masculino	de 46 a 55 anos
							Leve	Feminino	de 46 a 55 anos

							Leve	Feminino	de 56 a 65 anos
							Leve	Feminino	de 56 a 65 anos
							Leve	Feminino	de 56 a 65 anos
							Leve	Feminino	de 46 a 55 anos
							Leve	Feminino	de 46 a 55 anos
							Leve	Feminino	de 76 a 85 anos
							Leve	Feminino	menos de 28 dias
							Leve	Feminino	de 56 a 65 anos
							Leve	Masculino	de 56 a 65 anos
							Leve	Feminino	de 76 a 85 anos
							Nenhum	Feminino	de 26 a 35 anos
							Leve	Masculino	de 66 a 75 anos
							Leve	Masculino	de 56 a 65 anos
	Estágio 2 (perda parcial da espessura						Moderado	Masculino	de 76 a 85 anos
							Moderado	Masculino	de 76 a 85 anos

	Estágio 2 (perda parcial da espessura						Leve	Masculino	de 66 a 75 anos
	Estágio 3 (perda total da espessura						Moderado	Feminino	de 46 a 55 anos
							Leve	Feminino	menos de 28 dias
							Leve	Masculino	de 56 a 65 anos
							Leve	Feminino	menos de 28 dias
							Óbito	Masculino	de 18 a 25 anos
							Leve	Feminino	de 46 a 55 anos
							Moderado	Masculino	de 36 a 45 anos
							Leve	Feminino	menos de 28 dias
							Leve	Feminino	menos de 28 dias
							Leve	Feminino	menos de 28 dias
							Moderado	Feminino	de 46 a 55 anos
							Nenhum	Feminino	de 56 a 65 anos
							Moderado	Feminino	de 56 a 65 anos
							Leve	Masculino	de 66 a 75 anos

							Leve	Masculino	de 66 a 75 anos
Queimaduras.							Leve	Masculino	de 56 a 65 anos
							Nenhum	Feminino	de 36 a 45 anos
							Nenhum	Feminino	menos de 28 dias
							Óbito	Masculino	de 56 a 65 anos
							Moderado	Feminino	de 36 a 45 anos
							Leve	Masculino	de 56 a 65 anos
							Leve	Feminino	menos de 28 dias
							Leve	Feminino	de 36 a 45 anos
							Leve	Feminino	de 56 a 65 anos
							Leve	Masculino	de 56 a 65 anos
							Moderado	Masculino	de 18 a 25 anos
							Leve	Feminino	menos de 28 dias
							Leve	Masculino	de 46 a 55 anos
							Leve	Feminino	de 18 a 25 anos

							Moderado	Masculino	de 29 dias a 1 ano
							Leve	Feminino	de 18 a 25 anos
							Leve	Masculino	de 56 a 65 anos
	Estágio 4 (perda total da espessura						Moderado	Masculino	de 36 a 45 anos
							Leve	Masculino	de 36 a 45 anos
	Estágio 2 (perda parcial da espessura						Leve	Masculino	de 46 a 55 anos
							Leve	Feminino	de 46 a 55 anos
							Leve	Feminino	de 36 a 45 anos
							Leve	Feminino	de 26 a 35 anos
							Leve	Masculino	de 76 a 85 anos
							Moderado	Feminino	de 56 a 65 anos
							Moderado	Masculino	de 66 a 75 anos
	Estágio 3 (perda total da espessura						Moderado	Feminino	de 56 a 65 anos
							Leve	Feminino	de 46 a 55 anos
	Estágio 2 (perda parcial da espessura						Leve	Masculino	de 56 a 65 anos

							Leve	Feminino	de 29 dias a 1 ano
	Estágio 2 (perda parcial da espessura						Moderado	Feminino	de 66 a 75 anos
							Leve	Feminino	menos de 28 dias
	Estágio 2 (perda parcial da espessura						Moderado	Masculino	de 46 a 55 anos
							Leve	Masculino	de 66 a 75 anos
	Estágio 1 (pele íntegra com						Leve	Feminino	de 66 a 75 anos
	Estágio 3 (perda total da espessura						Moderado	Masculino	de 46 a 55 anos
							Moderado	Feminino	de 46 a 55 anos
	Estágio 2 (perda parcial da espessura						Moderado	Feminino	de 36 a 45 anos
	Estágio 3 (perda total da espessura						Grave	Masculino	de 36 a 45 anos
							Leve	Masculino	de 56 a 65 anos
	Estágio 3 (perda total da espessura						Moderado	Masculino	de 66 a 75 anos
	Estágio 2 (perda parcial da espessura						Moderado	Masculino	de 46 a 55 anos
	Estágio 3 (perda total da espessura						Moderado	Masculino	de 56 a 65 anos
	Estágio 3 (perda total da espessura						Moderado	Feminino	de 76 a 85 anos

	Estágio 2 (perda parcial da espessura						Leve	Feminino	de 76 a 85 anos
	Estágio 3 (perda total da espessura						Moderado	Masculino	de 46 a 55 anos
	Estágio 3 (perda total da espessura						Moderado	Feminino	de 76 a 85 anos
	Estágio 2 (perda parcial da espessura						Leve	Feminino	de 56 a 65 anos
	Estágio 3 (perda total da espessura						Moderado	Feminino	de 56 a 65 anos
	Estágio 3 (perda total da espessura						Moderado	Feminino	de 76 a 85 anos
	Estágio 3 (perda total da espessura						Moderado	Feminino	de 46 a 55 anos
	Estágio 2 (perda parcial da espessura						Moderado	Feminino	de 66 a 75 anos
	Estágio 3 (perda total da espessura						Moderado	Feminino	de 36 a 45 anos
	Estágio 2 (perda parcial da espessura						Moderado	Feminino	de 46 a 55 anos
							Nenhum	Masculino	de 18 a 25 anos
							Leve	Feminino	de 36 a 45 anos
							Leve	Feminino	menos de 28 dias
							Leve	Feminino	de 46 a 55 anos
							Leve	Feminino	mais de 85 anos

							Leve	Feminino	menos de 28 dias
							Leve	Masculino	de 26 a 35 anos
							Moderado	Feminino	de 26 a 35 anos
							Leve	Feminino	de 26 a 35 anos
							Leve	Feminino	de 26 a 35 anos
							Nenhum	Masculino	de 26 a 35 anos
							Nenhum	Masculino	de 26 a 35 anos
							Leve	Masculino	de 66 a 75 anos
							Leve	Masculino	de 66 a 75 anos
							Leve	Feminino	de 36 a 45 anos
							Leve	Feminino	de 46 a 55 anos
							Moderado	Feminino	de 18 a 25 anos
							Moderado	Feminino	de 18 a 25 anos
							Nenhum	Feminino	menos de 28 dias
							Leve	Feminino	de 46 a 55 anos

							Leve	Feminino	menos de 28 dias
							Leve	Feminino	menos de 28 dias
							Moderado	Masculino	de 76 a 85 anos
							Moderado	Masculino	de 76 a 85 anos
							Moderado	Masculino	de 76 a 85 anos
							Moderado	Feminino	de 46 a 55 anos
							Moderado	Feminino	mais de 85 anos
							Leve	Feminino	menos de 28 dias
	Estágio 3 (perda total da espessura						Leve	Feminino	de 36 a 45 anos
							Nenhum	Feminino	de 36 a 45 anos
	Estágio 3 (perda total da espessura						Leve	Feminino	de 29 dias a 1 ano
							Moderado	Masculino	de 26 a 35 anos
							Leve	Feminino	menos de 28 dias
							Moderado	Feminino	menos de 28 dias
	Estágio 2 (perda parcial da espessura						Leve	Feminino	menos de 28 dias

							Leve	Feminino	de 46 a 55 anos
							Moderado	Feminino	de 26 a 35 anos
							Moderado	Feminino	menos de 28 dias
							Nenhum	Feminino	de 46 a 55 anos
							Leve	Feminino	de 56 a 65 anos
							Leve	Feminino	menos de 28 dias
	Estagio 3 (perda total da espessura						Moderado	Feminino	menos de 28 dias
							Moderado	Feminino	menos de 28 dias
							Leve	Feminino	menos de 28 dias
	Estagio 3 (perda total da espessura						Moderado	Feminino	menos de 28 dias
							Moderado	Feminino	menos de 28 dias
							Leve	Feminino	menos de 28 dias
							Leve	Feminino	menos de 28 dias
							Leve	Masculino	de 46 a 55 anos
							Moderado	Feminino	menos de 28 dias

	Estágio 2 (perda parcial da espessura						Leve	Masculino	de 56 a 65 anos
							Leve	Feminino	de 29 dias a 1 ano
	Estágio 3 (perda total da espessura						Moderado	Masculino	de 36 a 45 anos
	Estágio 2 (perda parcial da espessura						Leve	Feminino	menos de 28 dias
							Moderado	Feminino	menos de 28 dias
							Leve	Feminino	de 46 a 55 anos
	Estágio 3 (perda total da espessura						Moderado	Masculino	de 56 a 65 anos
							Leve	Masculino	de 66 a 75 anos
							Leve	Masculino	mais de 85 anos
							Leve	Feminino	de 46 a 55 anos
							Leve	Feminino	de 26 a 35 anos
							Leve	Feminino	de 26 a 35 anos
							Leve	Masculino	de 76 a 85 anos
							Leve	Feminino	menos de 28 dias
							Leve	Feminino	de 46 a 55 anos

							Nenhum	Feminino	menos de 28 dias
	Estagio 1 (pele íntegra com						Leve	Feminino	menos de 28 dias
							Leve	Feminino	de 56 a 65 anos
							Leve	Masculino	de 56 a 65 anos
							Moderado	Feminino	de 56 a 65 anos
							Leve	Masculino	de 66 a 75 anos
							Moderado	Feminino	de 66 a 75 anos
	Estagio 3 (perda total da espessura						Moderado	Masculino	de 76 a 85 anos
							Moderado	Feminino	de 56 a 65 anos
	Estagio 2 (perda parcial da espessura						Moderado	Feminino	menos de 28 dias
							Leve	Masculino	de 56 a 65 anos
							Leve	Feminino	menos de 28 dias
							Leve	Feminino	menos de 28 dias
	Estagio 3 (perda total da espessura						Moderado	Feminino	de 46 a 55 anos
							Óbito	Masculino	de 76 a 85 anos

	Estágio 2 (perda parcial da espessura						Leve	Feminino	de 46 a 55 anos
	Estágio 3 (perda total da espessura						Moderado	Feminino	de 36 a 45 anos
	Estágio 2 (perda parcial da espessura						Moderado	Feminino	menos de 28 dias
	Estágio 3 (perda total da espessura						Moderado	Feminino	de 56 a 65 anos
							Leve	Feminino	de 26 a 35 anos
	Estágio 3 (perda total da espessura						Moderado	Feminino	de 56 a 65 anos
							Moderado	Masculino	menos de 28 dias
	Estágio 4 (perda total da espessura						Moderado	Feminino	de 46 a 55 anos
	Estágio 4 (perda total da espessura						Moderado	Feminino	de 76 a 85 anos
	Estágio 4 (perda total da espessura						Moderado	Feminino	de 66 a 75 anos
							Leve	Feminino	de 18 a 25 anos
	Estágio 2 (perda parcial da espessura						Moderado	Feminino	de 56 a 65 anos
	Estágio 2 (perda parcial da espessura						Moderado	Feminino	de 56 a 65 anos
							Leve	Masculino	de 66 a 75 anos
							Leve	Feminino	de 18 a 25 anos

	Estágio 4 (perda total da espessura						Moderado	Masculino	menos de 28 dias
	Estágio 3 (perda total da espessura						Moderado	Feminino	menos de 28 dias
							Moderado	Feminino	menos de 28 dias
							Nenhum	Feminino	de 36 a 45 anos
							Nenhum	Feminino	de 36 a 45 anos
							Leve	Feminino	menos de 28 dias
							Leve	Feminino	menos de 28 dias
							Leve	Feminino	menos de 28 dias
							Leve	Feminino	menos de 28 dias
							Moderado	Feminino	de 66 a 75 anos
							Leve	Feminino	menos de 28 dias
	Estágio 2 (perda parcial da espessura						Moderado	Feminino	menos de 28 dias
	Estágio 2 (perda parcial da espessura						Leve	Feminino	menos de 28 dias
							Leve	Feminino	menos de 28 dias
							Moderado	Masculino	de 56 a 65 anos

							Moderado	Masculino	de 56 a 65 anos
							Leve	Feminino	de 46 a 55 anos
	Estagio 4 (perda total da espessura						Grave	Feminino	de 66 a 75 anos
							Moderado	Masculino	de 66 a 75 anos
							Leve	Feminino	menos de 28 dias
							Moderado	Feminino	de 46 a 55 anos
	Estagio 2 (perda parcial da espessura						Moderado	Masculino	de 66 a 75 anos
							Leve	Feminino	de 36 a 45 anos
	Estagio 3 (perda total da espessura						Moderado	Masculino	de 66 a 75 anos
	Estagio 2 (perda parcial da espessura						Moderado	Masculino	de 66 a 75 anos
							Leve	Feminino	de 26 a 35 anos
	Estagio 2 (perda parcial da espessura						Moderado	Masculino	de 66 a 75 anos
	Estagio 4 (perda total da espessura						Grave	Feminino	de 66 a 75 anos
							Leve	Feminino	menos de 28 dias
							Leve	Feminino	menos de 28 dias

	Estágio 4 (perda total da espessura						Grave	Feminino	menos de 28 dias
	Estágio 4 (perda total da espessura						Grave	Feminino	de 26 a 35 anos
	Estágio 1 (pele íntegra com						Leve	Feminino	de 66 a 75 anos
							Leve	Feminino	de 66 a 75 anos
	Estágio 3 (perda total da espessura						Moderado	Feminino	de 66 a 75 anos
	Estágio 4 (perda total da espessura						Grave	Feminino	de 66 a 75 anos
	Estágio 2 (perda parcial da espessura						Moderado	Feminino	de 76 a 85 anos
	Estágio 2 (perda parcial da espessura						Moderado	Feminino	de 56 a 65 anos
	Estágio 4 (perda total da espessura						Grave	Masculino	de 66 a 75 anos
Queimaduras.							Leve	Feminino	menos de 28 dias
							Leve	Feminino	de 76 a 85 anos
	Estágio 2 (perda parcial da espessura						Leve	Masculino	de 36 a 45 anos
							Leve	Feminino	de 66 a 75 anos
	Estágio 3 (perda total da espessura						Moderado	Feminino	de 56 a 65 anos
							Moderado	Feminino	menos de 28 dias

							Leve	Masculino	de 66 a 75 anos
Queimaduras.							Moderado	Feminino	menos de 28 dias
							Leve	Feminino	de 56 a 65 anos
							Leve	Feminino	de 18 a 25 anos
							Moderado	Masculino	de 46 a 55 anos
							Óbito	Feminino	de 66 a 75 anos
							Óbito	Feminino	de 36 a 45 anos
							Óbito	Feminino	de 36 a 45 anos
	Estágio 2 (perda parcial da espessura						Moderado	Feminino	de 56 a 65 anos
							Moderado	Masculino	menos de 28 dias
	Estágio 3 (perda total da espessura						Moderado	Feminino	de 56 a 65 anos
	Estágio 2 (perda parcial da espessura						Moderado	Feminino	de 66 a 75 anos
	Estágio 2 (perda parcial da espessura						Moderado	Feminino	de 66 a 75 anos
	Estágio 2 (perda parcial da espessura						Moderado	Feminino	de 66 a 75 anos
	Estágio 2 (perda parcial da espessura						Moderado	Masculino	menos de 28 dias

							Nenhum	Feminino	de 46 a 55 anos
	Estágio 2 (perda parcial da espessura						Leve	Feminino	de 66 a 75 anos
							Leve	Masculino	de 36 a 45 anos
	Estágio 2 (perda parcial da espessura						Moderado	Masculino	de 36 a 45 anos
							Leve	Masculino	de 36 a 45 anos
							Moderado	Feminino	menos de 28 dias
							Moderado	Masculino	menos de 28 dias
	Estágio 2 (perda parcial da espessura						Moderado	Feminino	de 36 a 45 anos
	Estágio 2 (perda parcial da espessura						Moderado	Feminino	de 36 a 45 anos
							Moderado	Feminino	de 46 a 55 anos
	Estágio 3 (perda total da espessura						Moderado	Feminino	menos de 28 dias
							Moderado	Masculino	de 46 a 55 anos
							Moderado	Feminino	de 18 a 25 anos
							Óbito	Feminino	de 46 a 55 anos
	Estágio 3 (perda total da espessura						Moderado	Feminino	de 56 a 65 anos

	Estágio 1 (pele íntegra com						Leve	Feminino	de 29 dias a 1 ano
	Estágio 3 (perda total da espessura						Moderado	Masculino	menos de 28 dias
	Estágio 3 (perda total da espessura						Moderado	Feminino	menos de 28 dias
	Estágio 2 (perda parcial da espessura						Moderado	Feminino	de 66 a 75 anos
	Estágio 2 (perda parcial da espessura						Moderado	Feminino	de 26 a 35 anos
							Moderado	Feminino	de 26 a 35 anos
	Estágio 3 (perda total da espessura						Moderado	Masculino	de 66 a 75 anos
Queimaduras.							Moderado	Feminino	de 66 a 75 anos
	Estágio 3 (perda total da espessura						Moderado	Masculino	de 56 a 65 anos
							Leve	Feminino	de 56 a 65 anos
	Estágio 4 (perda total da espessura						Grave	Feminino	de 66 a 75 anos
							Grave	Feminino	de 36 a 45 anos
							Grave	Feminino	de 36 a 45 anos
							Grave	Masculino	de 46 a 55 anos
							Grave	Feminino	de 18 a 25 anos

	Estágio 3 (perda total da espessura						Moderado	Masculino	de 26 a 35 anos
	Estágio 2 (perda parcial da espessura						Moderado	Feminino	de 18 a 25 anos
							Leve	Feminino	de 26 a 35 anos
							Leve	Masculino	de 46 a 55 anos
							Nenhum	Masculino	de 56 a 65 anos
							Leve	Masculino	de 56 a 65 anos
							Nenhum	Feminino	de 26 a 35 anos
	Estágio 4 (perda total da espessura						Grave	Feminino	menos de 28 dias
	Estágio 2 (perda parcial da espessura						Leve	Feminino	de 56 a 65 anos
							Moderado	Feminino	menos de 28 dias
	Estágio 3 (perda total da espessura						Moderado	Feminino	de 26 a 35 anos
	Estágio 1 (pele íntegra com						Leve	Masculino	de 56 a 65 anos
	Estágio 1 (pele íntegra com						Moderado	Masculino	menos de 28 dias
	Estágio 4 (perda total da espessura						Moderado	Feminino	de 76 a 85 anos
	Estágio 3 (perda total da espessura						Moderado	Masculino	de 18 a 25 anos

	Estágio 3 (perda total da espessura						Moderado	Masculino	de 56 a 65 anos
	Estágio 1 (pele íntegra com						Leve	Feminino	de 56 a 65 anos
							Leve	Feminino	de 18 a 25 anos
							Leve	Masculino	de 46 a 55 anos
							Leve	Masculino	menos de 28 dias
							Leve	Masculino	menos de 28 dias
	Estágio 3 (perda total da espessura						Moderado	Masculino	de 56 a 65 anos
	Estágio 2 (perda parcial da espessura						Moderado	Masculino	de 76 a 85 anos
	Estágio 2 (perda parcial da espessura						Moderado	Masculino	de 56 a 65 anos
	Estágio 2 (perda parcial da espessura						Moderado	Feminino	de 66 a 75 anos
							Grave	Feminino	de 66 a 75 anos
							Grave	Feminino	de 56 a 65 anos
	Estágio 3 (perda total da espessura						Moderado	Feminino	de 56 a 65 anos
	Estágio 4 (perda total da espessura						Grave	Feminino	de 18 a 25 anos
	Estágio 2 (perda parcial da espessura						Moderado	Masculino	de 56 a 65 anos

	Estágio 1 (pele íntegra com						Leve	Masculino	de 46 a 55 anos
	Estágio 3 (perda total da espessura						Grave	Feminino	menos de 28 dias
	Estágio 3 (perda total da espessura						Moderado	Feminino	de 66 a 75 anos
	Estágio 2 (perda parcial da espessura						Moderado	Feminino	de 36 a 45 anos
	Estágio 2 (perda parcial da espessura						Moderado	Feminino	de 56 a 65 anos
	Estágio 2 (perda parcial da espessura						Moderado	Masculino	de 46 a 55 anos
							Leve	Masculino	de 46 a 55 anos
							Grave	Feminino	de 76 a 85 anos
							Grave	Feminino	de 76 a 85 anos
							Grave	Feminino	de 76 a 85 anos
							Grave	Masculino	de 66 a 75 anos
Queimaduras.							Leve	Masculino	de 46 a 55 anos
Queimaduras.							Leve	Masculino	de 46 a 55 anos
							Óbito	Feminino	de 26 a 35 anos
							Leve	Masculino	de 46 a 55 anos

							Leve	Masculino	menos de 28 dias
							Moderado	Feminino	menos de 28 dias
							Moderado	Feminino	menos de 28 dias
	Estágio 2 (perda parcial da espessura						Moderado	Feminino	de 56 a 65 anos
	Estágio 4 (perda total da espessura						Grave	Masculino	de 56 a 65 anos
	Estágio 2 (perda parcial da espessura						Moderado	Masculino	de 18 a 25 anos
	Estágio 4 (perda total da espessura						Moderado	Feminino	menos de 28 dias
	Estágio 1 (pele íntegra com						Leve	Feminino	de 66 a 75 anos
							Moderado	Feminino	de 46 a 55 anos
							Leve	Masculino	de 66 a 75 anos
	Estágio 1 (pele íntegra com						Leve	Masculino	de 46 a 55 anos
	Estágio 3 (perda total da espessura						Moderado	Masculino	de 26 a 35 anos
	Estágio 2 (perda parcial da espessura						Leve	Feminino	de 36 a 45 anos
	Estágio 2 (perda parcial da espessura						Moderado	Feminino	de 46 a 55 anos
	Estágio 2 (perda parcial da espessura						Moderado	Masculino	de 46 a 55 anos

							Leve	Feminino	menos de 28 dias
							Moderado	Feminino	de 46 a 55 anos
							Moderado	Feminino	de 18 a 25 anos
							Moderado	Feminino	de 18 a 25 anos
							Moderado	Feminino	de 56 a 65 anos
	Estágio 3 (perda total da espessura						Grave	Masculino	de 18 a 25 anos
	Estágio 2 (perda parcial da espessura						Moderado	Feminino	de 56 a 65 anos
							Nenhum	Feminino	de 26 a 35 anos
							Leve	Feminino	de 36 a 45 anos
							Grave	Feminino	de 26 a 35 anos
	Estágio 4 (perda total da espessura						Grave	Masculino	de 56 a 65 anos
	Estágio 4 (perda total da espessura						Grave	Feminino	menos de 28 dias
							Moderado	Masculino	de 56 a 65 anos
	Estágio 1 (pele íntegra com						Moderado	Masculino	de 26 a 35 anos
	Estágio 2 (perda parcial da espessura						Moderado	Feminino	menos de 28 dias

	Estágio 3 (perda total da espessura						Moderado	Masculino	de 76 a 85 anos
	Estágio 2 (perda parcial da espessura						Moderado	Feminino	de 46 a 55 anos
							Leve	Feminino	de 26 a 35 anos
	Estágio 3 (perda total da espessura						Moderado	Masculino	de 46 a 55 anos
							Moderado	Feminino	de 56 a 65 anos
	Estágio 2 (perda parcial da espessura						Grave	Feminino	de 76 a 85 anos
	Estágio 3 (perda total da espessura						Grave	Feminino	de 29 dias a 1 ano
	Estágio 2 (perda parcial da espessura						Moderado	Feminino	de 76 a 85 anos
							Óbito	Masculino	de 66 a 75 anos
							Moderado	Feminino	de 36 a 45 anos
							Moderado	Feminino	menos de 28 dias
							Moderado	Feminino	menos de 28 dias
							Moderado	Masculino	menos de 28 dias
							Moderado	Feminino	de 29 dias a 1 ano
	Estágio 3 (perda total da espessura						Moderado	Masculino	de 26 a 35 anos

	Estágio 2 (perda parcial da espessura						Moderado	Feminino	de 46 a 55 anos
							Moderado	Feminino	de 26 a 35 anos
							Moderado	Masculino	de 56 a 65 anos
	Estágio 2 (perda parcial da espessura						Leve	Masculino	de 66 a 75 anos
							Leve	Feminino	de 12 a 17 anos
							Moderado	Feminino	menos de 28 dias
							Moderado	Feminino	menos de 28 dias
							Leve	Masculino	de 56 a 65 anos
							Moderado	Feminino	menos de 28 dias
							Grave	Masculino	menos de 28 dias
	Estágio 2 (perda parcial da espessura						Moderado	Masculino	de 46 a 55 anos
	Estágio 2 (perda parcial da espessura						Moderado	Feminino	de 66 a 75 anos
	Estágio 2 (perda parcial da espessura						Moderado	Masculino	de 76 a 85 anos
	Estágio 2 (perda parcial da espessura						Moderado	Masculino	de 76 a 85 anos
	Estágio 2 (perda parcial da espessura						Moderado	Masculino	de 76 a 85 anos

	Estágio 2 (perda parcial da espessura						Moderado	Masculino	de 76 a 85 anos
							Moderado	Feminino	de 46 a 55 anos
							Moderado	Feminino	de 36 a 45 anos
							Moderado	Masculino	menos de 28 dias
							Moderado	Feminino	menos de 28 dias
							Moderado	Masculino	de 29 dias a 1 ano
							Moderado	Masculino	de 56 a 65 anos
							Moderado	Feminino	de 46 a 55 anos
	Estágio 4 (perda total da espessura						Grave	Feminino	de 56 a 65 anos
	Estágio 3 (perda total da espessura						Moderado	Masculino	de 29 dias a 1 ano
							Moderado	Masculino	de 36 a 45 anos
	Estágio 3 (perda total da espessura						Moderado	Masculino	de 29 dias a 1 ano
	Estágio 3 (perda total da espessura						Moderado	Feminino	de 76 a 85 anos
	Estágio 3 (perda total da espessura						Moderado	Masculino	mais de 85 anos
	Estágio 3 (perda total da espessura						Moderado	Feminino	menos de 28 dias

	Estágio 2 (perda parcial da espessura						Moderado	Feminino	de 66 a 75 anos
							Leve	Masculino	menos de 28 dias
							Leve	Masculino	menos de 28 dias
							Leve	Feminino	menos de 28 dias
							Leve	Masculino	menos de 28 dias
							Grave	Feminino	de 26 a 35 anos
							Grave	Feminino	de 12 a 17 anos
							Grave	Feminino	de 12 a 17 anos
	Estágio 2 (perda parcial da espessura						Moderado	Masculino	mais de 85 anos
	Estágio 4 (perda total da espessura						Grave	Feminino	menos de 28 dias
							Leve	Feminino	de 18 a 25 anos
							Moderado	Feminino	menos de 28 dias
							Leve	Masculino	de 46 a 55 anos
Queimaduras.							Grave	Feminino	de 29 dias a 1 ano
							Moderado	Masculino	menos de 28 dias

							Leve	Masculino	de 46 a 55 anos
							Leve	Feminino	de 76 a 85 anos
							Leve	Feminino	de 66 a 75 anos
	Estágio 2 (perda parcial da espessura						Moderado	Feminino	de 76 a 85 anos
							Nenhum	Feminino	de 26 a 35 anos
	Estágio 2 (perda parcial da espessura						Moderado	Masculino	menos de 28 dias
							Moderado	Masculino	menos de 28 dias
							Moderado	Feminino	de 56 a 65 anos
							Leve	Masculino	de 46 a 55 anos
							Moderado	Feminino	de 66 a 75 anos
							Leve	Masculino	de 26 a 35 anos
							Nenhum	Masculino	de 36 a 45 anos
	Estágio 1 (pele íntegra com						Leve	Feminino	menos de 28 dias
	Estágio 3 (perda total da espessura						Grave	Masculino	de 76 a 85 anos
	Estágio 2 (perda parcial da espessura						Moderado	Masculino	de 46 a 55 anos

	Estágio 2 (perda parcial da [illegible]						Moderado	Feminino	menos de 28 dias
	Estágio 2 (perda parcial da [illegible]						Moderado	Masculino	de 76 a 85 anos
	Estágio 3 (perda total da [illegible]						Grave	Feminino	mais de 85 anos
	Estágio 2 (perda parcial da [illegible]						Moderado	Masculino	de 46 a 55 anos
	Estágio 2 (perda parcial da [illegible]						Moderado	Masculino	mais de 85 anos
	Estágio 2 (perda parcial da [illegible]						Moderado	Masculino	de 76 a 85 anos
	Estágio 2 (perda parcial da [illegible]						Moderado	Feminino	de 76 a 85 anos
							Grave	Masculino	menos de 28 dias
							Moderado	Masculino	de 46 a 55 anos
							Nenhum	Masculino	de 46 a 55 anos
							Leve	Masculino	menos de 28 dias
							Leve	Feminino	de 46 a 55 anos
							Moderado	Feminino	menos de 28 dias
							Moderado	Masculino	de 36 a 45 anos
							Leve	Feminino	de 36 a 45 anos

							Moderado	Feminino	menos de 28 dias
							Leve	Feminino	de 36 a 45 anos
	Estágio 3 (perda total da espessura						Grave	Masculino	de 56 a 65 anos
	Estágio 3 (perda total da espessura						Grave	Feminino	de 36 a 45 anos
	Estágio 2 (perda parcial da espessura						Moderado	Masculino	de 66 a 75 anos
	Estágio 2 (perda parcial da espessura						Moderado	Masculino	de 56 a 65 anos
	Estágio 3 (perda total da espessura						Grave	Feminino	de 29 dias a 1 ano
	Estágio 2 (perda parcial da espessura						Moderado	Feminino	de 29 dias a 1 ano
	Estágio 1 (pele íntegra com						Leve	Feminino	de 36 a 45 anos
	Estágio 1 (pele íntegra com						Leve	Feminino	menos de 28 dias
	Estágio 1 (pele íntegra com						Moderado	Masculino	de 46 a 55 anos
	Estágio 2 (perda parcial da espessura						Moderado	Feminino	de 76 a 85 anos
	Estágio 1 (pele íntegra com						Leve	Masculino	de 76 a 85 anos
	Estágio 2 (perda parcial da espessura						Moderado	Masculino	menos de 28 dias
	Estágio 3 (perda total da espessura						Moderado	Masculino	de 76 a 85 anos

	Estágio 2 (perda parcial da espessura						Moderado	Masculino	de 56 a 65 anos
	Estágio 2 (perda parcial da espessura						Moderado	Masculino	de 66 a 75 anos
	Estágio 2 (perda parcial da espessura						Moderado	Feminino	de 66 a 75 anos
	Estágio 2 (perda parcial da espessura						Moderado	Feminino	de 66 a 75 anos
							Moderado	Masculino	de 56 a 65 anos
	Estágio 2 (perda parcial da espessura						Moderado	Feminino	de 66 a 75 anos
	Estágio 4 (perda total da espessura						Grave	Masculino	de 56 a 65 anos
	Estágio 2 (perda parcial da espessura						Moderado	Feminino	menos de 28 dias
							Moderado	Feminino	de 56 a 65 anos
							Leve	Feminino	de 18 a 25 anos
	Estágio 3 (perda total da espessura						Grave	Feminino	de 76 a 85 anos
							Leve	Feminino	de 26 a 35 anos
							Grave	Feminino	de 29 dias a 1 ano
							Grave	Feminino	de 29 dias a 1 ano
							Moderado	Feminino	de 29 dias a 1 ano

	Estágio 2 (perda parcial da espessura						Moderado	Masculino	de 36 a 45 anos
							Moderado	Feminino	de 56 a 65 anos
							Grave	Masculino	menos de 28 dias
							Leve	Masculino	de 66 a 75 anos
							Moderado	Feminino	de 26 a 35 anos
	Estágio 3 (perda total da espessura						Moderado	Masculino	de 26 a 35 anos
	Estágio 4 (perda total da espessura						Grave	Masculino	de 36 a 45 anos
	Estágio 2 (perda parcial da espessura						Moderado	Feminino	de 36 a 45 anos
	Estágio 2 (perda parcial da espessura						Moderado	Feminino	de 56 a 65 anos
	Estágio 2 (perda parcial da espessura						Moderado	Feminino	de 36 a 45 anos
	Estágio 3 (perda total da espessura						Moderado	Feminino	de 66 a 75 anos
	Estágio 2 (perda parcial da espessura						Moderado	Masculino	de 29 dias a 1 ano
	Estágio 2 (perda parcial da espessura						Moderado	Masculino	de 56 a 65 anos
							Grave	Feminino	de 18 a 25 anos
							Moderado	Masculino	menos de 28 dias

	Estágio 3 (perda total da espessura						Moderado	Feminino	de 26 a 35 anos
							Moderado	Feminino	de 46 a 55 anos
	Estágio 2 (perda parcial da espessura						Moderado	Masculino	de 36 a 45 anos
	Estágio 2 (perda parcial da espessura						Moderado	Feminino	de 26 a 35 anos
							Moderado	Masculino	de 29 dias a 1 ano
							Moderado	Feminino	de 29 dias a 1 ano
							Moderado	Masculino	menos de 28 dias
							Grave	Feminino	de 26 a 35 anos
							Moderado	Masculino	de 56 a 65 anos
	Estágio 3 (perda total da espessura						Moderado	Masculino	menos de 28 dias
	Relacionada a dispositivo para a						Moderado	Feminino	menos de 28 dias
	Estágio 3 (perda total da espessura						Moderado	Feminino	mais de 85 anos
	Estágio 2 (perda parcial da espessura						Moderado	Feminino	de 66 a 75 anos
	Não Classificável (perda						Grave	Masculino	de 66 a 75 anos
	Estágio 2 (perda parcial da espessura						Moderado	Feminino	de 66 a 75 anos

	Estágio 2 (perda parcial da espessura						Moderado	Masculino	mais de 85 anos
	Estágio 3 (perda total da espessura						Moderado	Feminino	de 46 a 55 anos
							Moderado	Feminino	de 29 dias a 1 ano
							Moderado	Feminino	de 29 dias a 1 ano
							Moderado	Feminino	de 66 a 75 anos
							Moderado	Masculino	de 46 a 55 anos
							Leve	Feminino	de 36 a 45 anos
	Não Classificável (perda						Grave	Masculino	mais de 85 anos
	Estágio 2 (perda parcial da espessura						Moderado	Masculino	de 26 a 35 anos
	Estágio 3 (perda total da espessura						Grave	Masculino	de 26 a 35 anos
	Não Classificável (perda						Grave	Feminino	mais de 85 anos
	Estágio 2 (perda parcial da espessura						Moderado	Masculino	de 76 a 85 anos
	Estágio 2 (perda parcial da espessura						Moderado	Feminino	de 26 a 35 anos
	Estágio 2 (perda parcial da espessura						Moderado	Feminino	de 76 a 85 anos
							Moderado	Feminino	menos de 28 dias

							Moderado	Masculino	menos de 28 dias
							Leve	Masculino	de 76 a 85 anos
							Leve	Feminino	de 56 a 65 anos
							Leve	Masculino	de 56 a 65 anos
							Leve	Masculino	de 36 a 45 anos
Queimaduras.							Leve	Feminino	de 46 a 55 anos
							Leve	Feminino	de 56 a 65 anos
							Moderado	Masculino	de 56 a 65 anos
							Leve	Masculino	de 66 a 75 anos
							Leve	Masculino	de 56 a 65 anos
							Leve	Masculino	de 56 a 65 anos
							Leve	Masculino	de 56 a 65 anos
							Leve	Masculino	de 56 a 65 anos
							Leve	Masculino	de 66 a 75 anos
							Moderado	Masculino	de 56 a 65 anos

							Leve	Feminino	de 36 a 45 anos
							Leve	Masculino	de 66 a 75 anos
							Leve	Masculino	de 66 a 75 anos
							Leve	Masculino	de 46 a 55 anos
							Leve	Masculino	de 76 a 85 anos
							Leve	Feminino	de 26 a 35 anos
							Leve	Feminino	de 26 a 35 anos
							Leve	Feminino	de 46 a 55 anos
							Leve	Masculino	de 46 a 55 anos
							Leve	Feminino	de 76 a 85 anos
							Leve	Feminino	de 56 a 65 anos
							Leve	Masculino	de 18 a 25 anos
							Leve	Feminino	de 66 a 75 anos
							Leve	Feminino	de 26 a 35 anos
							Leve	Feminino	de 46 a 55 anos

Queimaduras.							Leve	Masculino	de 36 a 45 anos
							Leve	Feminino	de 56 a 65 anos
							Leve	Feminino	de 66 a 75 anos
							Leve	Masculino	de 26 a 35 anos
							Leve	Feminino	de 36 a 45 anos
							Leve	Feminino	de 46 a 55 anos
							Leve	Feminino	de 18 a 25 anos
							Moderado	Masculino	de 66 a 75 anos
							Leve	Masculino	de 56 a 65 anos
							Leve	Masculino	de 26 a 35 anos
							Leve	Masculino	de 66 a 75 anos
							Leve	Feminino	de 56 a 65 anos
							Leve	Masculino	de 56 a 65 anos
							Leve	Masculino	de 56 a 65 anos
							Leve	Masculino	de 56 a 65 anos

							Leve	Masculino	de 76 a 85 anos
							Leve	Masculino	de 18 a 25 anos
							Leve	Masculino	de 56 a 65 anos
							Leve	Feminino	de 36 a 45 anos
							Leve	Masculino	de 36 a 45 anos
							Leve	Masculino	de 36 a 45 anos
							Leve	Masculino	de 76 a 85 anos
							Leve	Masculino	de 56 a 65 anos
							Leve	Masculino	de 66 a 75 anos
							Leve	Masculino	de 66 a 75 anos
							Leve	Masculino	de 56 a 65 anos
							Leve	Masculino	de 66 a 75 anos
							Leve	Masculino	de 36 a 45 anos
							Leve	Masculino	de 26 a 35 anos
							Leve	Feminino	de 66 a 75 anos

							Leve	Masculino	de 36 a 45 anos
							Leve	Masculino	de 18 a 25 anos
							Grave	Masculino	de 76 a 85 anos
							Leve	Masculino	de 56 a 65 anos
							Nenhum	Feminino	de 56 a 65 anos
							Moderado	Masculino	de 66 a 75 anos
							Nenhum	Masculino	de 46 a 55 anos
							Leve	Feminino	de 56 a 65 anos
							Leve	Feminino	de 66 a 75 anos
							Leve	Masculino	de 46 a 55 anos
							Nenhum	Feminino	de 66 a 75 anos
Obito ou lesão grave de paciente							Leve	Feminino	de 36 a 45 anos
							Leve	Feminino	de 56 a 65 anos
							Leve	Masculino	mais de 85 anos
							Leve	Masculino	de 76 a 85 anos

							Leve	Masculino	de 56 a 65 anos
							Leve	Feminino	de 18 a 25 anos
							Leve	Masculino	de 66 a 75 anos
							Leve	Feminino	de 56 a 65 anos
							Leve	Feminino	de 66 a 75 anos
							Leve	Feminino	de 36 a 45 anos
							Leve	Feminino	de 36 a 45 anos
	Estágio 3 (perda total da espessura						Leve	Feminino	de 76 a 85 anos
	Estágio 3 (perda total da espessura						Leve	Feminino	de 66 a 75 anos
	Estágio 3 (perda total da espessura						Leve	Feminino	de 18 a 25 anos
	Estágio 3 (perda total da espessura						Leve	Feminino	de 76 a 85 anos
	Estágio 3 (perda total da espessura						Leve	Feminino	de 56 a 65 anos
	Estágio 4 (perda total da espessura						Leve	Masculino	de 56 a 65 anos
	Estágio 3 (perda total da espessura						Leve	Feminino	de 56 a 65 anos
	Estágio 3 (perda total da espessura						Leve	Masculino	de 66 a 75 anos

	Estágio 3 (perda total da espessura						Leve	Feminino	de 76 a 85 anos
	Estágio 3 (perda total da espessura						Leve	Masculino	de 26 a 35 anos
	Estágio 3 (perda total da espessura						Leve	Feminino	de 36 a 45 anos
	Estágio 3 (perda total da espessura						Leve	Masculino	de 76 a 85 anos
	Estágio 3 (perda total da espessura						Leve	Feminino	de 46 a 55 anos
	Estágio 3 (perda total da espessura						Leve	Feminino	de 29 dias a 1 ano
							Leve	Feminino	de 36 a 45 anos
							Leve	Feminino	de 36 a 45 anos
							Leve	Feminino	de 66 a 75 anos
							Leve	Feminino	de 66 a 75 anos
							Leve	Feminino	de 76 a 85 anos
							Leve	Feminino	de 36 a 45 anos
							Leve	Feminino	de 18 a 25 anos
							Leve	Masculino	de 46 a 55 anos
							Leve	Masculino	de 56 a 65 anos

							Leve	Feminino	de 18 a 25 anos
							Leve	Feminino	de 46 a 55 anos
	Estágio 4 (perda total da espessura						Leve	Masculino	de 76 a 85 anos
	Estágio 3 (perda total da espessura						Leve	Masculino	de 76 a 85 anos
	Estágio 3 (perda total da espessura						Leve	Masculino	de 12 a 17 anos
	Estágio 4 (perda total da espessura						Leve	Masculino	de 76 a 85 anos
	Estágio 4 (perda total da espessura						Leve	Masculino	de 66 a 75 anos
	Estágio 4 (perda total da espessura						Leve	Masculino	de 66 a 75 anos
							Leve	Feminino	mais de 85 anos
							Leve	Feminino	de 36 a 45 anos
							Leve	Feminino	de 56 a 65 anos
							Óbito	Feminino	de 18 a 25 anos
							Nenhum	Feminino	de 26 a 35 anos
							Nenhum	Feminino	de 36 a 45 anos
							Leve	Feminino	de 18 a 25 anos

							Nenhum	Feminino	de 18 a 25 anos
							Nenhum	Masculino	de 56 a 65 anos
							Nenhum	Feminino	de 26 a 35 anos
							Leve	Masculino	de 46 a 55 anos
							Leve	Feminino	de 26 a 35 anos
							Leve	Masculino	de 56 a 65 anos
							Leve	Masculino	de 26 a 35 anos
							Leve	Feminino	de 56 a 65 anos
							Leve	Feminino	de 26 a 35 anos
							Leve	Masculino	de 66 a 75 anos
							Leve	Masculino	de 46 a 55 anos
							Leve	Masculino	de 46 a 55 anos
							Leve	Feminino	de 46 a 55 anos
							Leve	Masculino	de 46 a 55 anos
							Leve	Masculino	de 46 a 55 anos

							Leve	Masculino	de 56 a 65 anos
							Leve	Feminino	de 36 a 45 anos
							Leve	Masculino	de 66 a 75 anos
	Estágio 3 (perda total da espessura						Leve	Masculino	de 46 a 55 anos
							Leve	Masculino	de 56 a 65 anos
							Leve	Feminino	de 66 a 75 anos
							Leve	Masculino	de 56 a 65 anos
							Leve	Feminino	de 66 a 75 anos
							Leve	Masculino	de 66 a 75 anos
							Leve	Masculino	de 66 a 75 anos
	Estágio 4 (perda total da espessura						Leve	Masculino	de 66 a 75 anos
							Leve	Masculino	de 36 a 45 anos
							Leve	Feminino	de 56 a 65 anos
							Leve	Masculino	de 56 a 65 anos
							Leve	Masculino	de 26 a 35 anos

	Estágio 2 (perda parcial da espessura						Leve	Masculino	de 66 a 75 anos
							Leve	Masculino	de 66 a 75 anos
	Estágio 2 (perda parcial da espessura						Leve	Masculino	de 56 a 65 anos
							Leve	Masculino	de 56 a 65 anos
							Leve	Masculino	de 46 a 55 anos
	Estágio 1 (pele íntegra com						Leve	Masculino	de 76 a 85 anos
							Leve	Masculino	de 46 a 55 anos
							Nenhum	Feminino	de 56 a 65 anos
	Estágio 3 (perda total da espessura						Leve	Masculino	de 18 a 25 anos
	Estágio 3 (perda total da espessura						Leve	Masculino	de 46 a 55 anos
	Estágio 3 (perda total da espessura						Leve	Feminino	de 66 a 75 anos
	Estágio 3 (perda total da espessura						Leve	Masculino	de 46 a 55 anos
	Estágio 3 (perda total da espessura						Leve	Feminino	de 76 a 85 anos
	Estágio 3 (perda total da espessura						Leve	Masculino	de 46 a 55 anos
	Estágio 3 (perda total da espessura						Leve	Feminino	de 66 a 75 anos

	Estágio 3 (perda total da espessura						Leve	Feminino	de 66 a 75 anos
	Estágio 3 (perda total da espessura						Leve	Masculino	de 26 a 35 anos
	Estágio 4 (perda total da espessura						Leve	Masculino	de 76 a 85 anos
	Estágio 3 (perda total da espessura						Leve	Feminino	de 56 a 65 anos
	Estágio 3 (perda total da espessura						Leve	Feminino	de 56 a 65 anos
	Estágio 3 (perda total da espessura						Leve	Masculino	de 66 a 75 anos
	Estágio 3 (perda total da espessura						Leve	Masculino	de 56 a 65 anos
	Estágio 3 (perda total da espessura						Leve	Masculino	de 46 a 55 anos
	Estágio 4 (perda total da espessura						Leve	Masculino	de 76 a 85 anos
	Estágio 4 (perda total da espessura						Leve	Masculino	de 76 a 85 anos
	Estágio 3 (perda total da espessura						Leve	Masculino	de 76 a 85 anos
	Estágio 3 (perda total da espessura						Leve	Masculino	de 66 a 75 anos
							Leve	Masculino	de 56 a 65 anos
							Leve	Feminino	de 66 a 75 anos
	Estágio 4 (perda total da espessura						Leve	Masculino	de 56 a 65 anos

	Estágio 4 (perda total da espessura						Leve	Masculino	de 66 a 75 anos
	Estágio 4 (perda total da espessura						Leve	Masculino	de 36 a 45 anos
	Estágio 3 (perda total da espessura						Leve	Masculino	de 66 a 75 anos
	Estágio 3 (perda total da espessura						Leve	Feminino	de 56 a 65 anos
	Estágio 4 (perda total da espessura						Leve	Masculino	de 76 a 85 anos
	Estágio 4 (perda total da espessura						Leve	Masculino	de 66 a 75 anos
	Estágio 4 (perda total da espessura						Leve	Masculino	de 66 a 75 anos
	Estágio 4 (perda total da espessura						Leve	Masculino	de 56 a 65 anos
	Estágio 4 (perda total da espessura						Leve	Feminino	de 36 a 45 anos
	Estágio 4 (perda total da espessura						Leve	Feminino	de 36 a 45 anos
	Estágio 4 (perda total da espessura						Leve	Masculino	de 56 a 65 anos
	Estágio 4 (perda total da espessura						Leve	Feminino	de 46 a 55 anos
	Estágio 4 (perda total da espessura						Leve	Masculino	de 46 a 55 anos
	Estágio 4 (perda total da espessura						Leve	Masculino	de 18 a 25 anos
	Estágio 4 (perda total da espessura						Leve	Feminino	de 76 a 85 anos

	Estágio 3 (perda total da espessura						Leve	Masculino	de 46 a 55 anos
	Estágio 3 (perda total da espessura						Leve	Feminino	de 18 a 25 anos
	Estágio 3 (perda total da espessura						Leve	Masculino	de 46 a 55 anos
	Estágio 3 (perda total da espessura						Leve	Masculino	de 46 a 55 anos
	Estágio 3 (perda total da espessura						Leve	Feminino	de 56 a 65 anos
	Estágio 3 (perda total da espessura						Leve	Feminino	de 18 a 25 anos
	Estágio 3 (perda total da espessura						Leve	Masculino	de 46 a 55 anos
	Estágio 3 (perda total da espessura						Leve	Masculino	de 66 a 75 anos
	Estágio 3 (perda total da espessura						Leve	Feminino	de 36 a 45 anos
	Estágio 4 (perda total da espessura						Leve	Masculino	de 18 a 25 anos
	Estágio 4 (perda total da espessura						Leve	Feminino	de 66 a 75 anos
	Estágio 4 (perda total da espessura						Leve	Masculino	mais de 85 anos
	Estágio 4 (perda total da espessura						Leve	Feminino	de 66 a 75 anos
	Estágio 4 (perda total da espessura						Leve	Masculino	de 46 a 55 anos
	Estágio 4 (perda total da espessura						Leve	Masculino	de 46 a 55 anos

	Estágio 3 (perda total da espessura						Leve	Masculino	de 76 a 85 anos
	Estágio 3 (perda total da espessura						Leve	Feminino	de 66 a 75 anos
	Estágio 3 (perda total da espessura						Leve	Masculino	de 18 a 25 anos
	Estágio 3 (perda total da espessura						Leve	Masculino	de 46 a 55 anos
	Estágio 3 (perda total da espessura						Leve	Feminino	de 56 a 65 anos
	Estágio 3 (perda total da espessura						Leve	Feminino	de 66 a 75 anos
	Estágio 3 (perda total da espessura						Leve	Feminino	de 56 a 65 anos
	Estágio 3 (perda total da espessura						Leve	Feminino	de 18 a 25 anos
	Estágio 3 (perda total da espessura						Leve	Masculino	de 66 a 75 anos
	Estágio 3 (perda total da espessura						Leve	Feminino	de 66 a 75 anos
	Estágio 4 (perda total da espessura						Leve	Masculino	mais de 85 anos
	Estágio 4 (perda total da espessura						Leve	Masculino	mais de 85 anos
	Estágio 3 (perda total da espessura						Leve	Feminino	de 66 a 75 anos
	Estágio 3 (perda total da espessura						Leve	Masculino	de 76 a 85 anos
	Estágio 3 (perda total da espessura						Leve	Feminino	de 66 a 75 anos

	Estágio 4 (perda total da espessura						Leve	Masculino	de 66 a 75 anos
	Estágio 3 (perda total da espessura						Leve	Feminino	de 18 a 25 anos
	Estágio 3 (perda total da espessura						Leve	Masculino	de 66 a 75 anos
	Estágio 3 (perda total da espessura						Leve	Masculino	de 66 a 75 anos
	Estágio 3 (perda total da espessura						Leve	Masculino	de 46 a 55 anos
	Estágio 3 (perda total da espessura						Leve	Masculino	de 56 a 65 anos
	Estágio 3 (perda total da espessura						Leve	Masculino	de 46 a 55 anos
	Estágio 3 (perda total da espessura						Leve	Masculino	de 46 a 55 anos
	Estágio 4 (perda total da espessura						Leve	Masculino	de 46 a 55 anos
	Estágio 4 (perda total da espessura						Leve	Masculino	de 46 a 55 anos
	Estágio 4 (perda total da espessura						Leve	Masculino	mais de 85 anos
	Estágio 4 (perda total da espessura						Leve	Feminino	mais de 85 anos
	Estágio 4 (perda total da espessura						Leve	Masculino	de 76 a 85 anos
	Estágio 4 (perda total da espessura						Leve	Masculino	de 46 a 55 anos
	Estágio 4 (perda total da espessura						Leve	Masculino	de 46 a 55 anos

	Estágio 4 (perda total da espessura						Leve	Masculino	de 46 a 55 anos
	Estágio 4 (perda total da espessura						Leve	Masculino	de 46 a 55 anos
	Estágio 4 (perda total da espessura						Leve	Masculino	de 56 a 65 anos
	Estágio 4 (perda total da espessura						Leve	Feminino	de 66 a 75 anos
	Estágio 4 (perda total da espessura						Leve	Masculino	de 46 a 55 anos
	Estágio 3 (perda total da espessura						Leve	Masculino	de 66 a 75 anos
	Estágio 3 (perda total da espessura						Leve	Feminino	de 26 a 35 anos
	Estágio 3 (perda total da espessura						Leve	Masculino	de 56 a 65 anos
	Estágio 3 (perda total da espessura						Leve	Masculino	de 26 a 35 anos
	Estágio 3 (perda total da espessura						Leve	Masculino	de 66 a 75 anos
	Estágio 3 (perda total da espessura						Leve	Masculino	de 26 a 35 anos
	Estágio 3 (perda total da espessura						Leve	Feminino	de 56 a 65 anos
	Estágio 3 (perda total da espessura						Leve	Masculino	de 66 a 75 anos
	Estágio 3 (perda total da espessura						Leve	Masculino	de 18 a 25 anos
	Estágio 3 (perda total da espessura						Leve	Masculino	de 46 a 55 anos

	Estágio 3 (perda total da espessura						Leve	Masculino	de 18 a 25 anos
	Estágio 3 (perda total da espessura						Leve	Masculino	de 56 a 65 anos
	Estágio 3 (perda total da espessura						Leve	Masculino	de 46 a 55 anos
	Estágio 3 (perda total da espessura						Leve	Feminino	de 36 a 45 anos
	Estágio 3 (perda total da espessura						Leve	Feminino	de 56 a 65 anos
	Estágio 3 (perda total da espessura						Leve	Masculino	de 66 a 75 anos
	Estágio 3 (perda total da espessura						Leve	Masculino	de 66 a 75 anos
	Estágio 3 (perda total da espessura						Leve	Masculino	mais de 85 anos
	Estágio 3 (perda total da espessura						Leve	Masculino	de 76 a 85 anos
	Estágio 4 (perda total da espessura						Leve	Masculino	mais de 85 anos
	Estágio 3 (perda total da espessura						Leve	Masculino	de 66 a 75 anos
	Estágio 3 (perda total da espessura						Leve	Masculino	de 46 a 55 anos
	Estágio 3 (perda total da espessura						Leve	Feminino	de 18 a 25 anos
	Estágio 3 (perda total da espessura						Leve	Masculino	de 56 a 65 anos
	Estágio 4 (perda total da espessura						Leve	Feminino	menos de 28 dias

	Estágio 4 (perda total da espessura						Leve	Masculino	de 46 a 55 anos
	Estágio 3 (perda total da espessura						Leve	Masculino	de 56 a 65 anos
	Estágio 3 (perda total da espessura						Leve	Masculino	de 56 a 65 anos
	Estágio 3 (perda total da espessura						Leve	Masculino	de 66 a 75 anos
	Estágio 4 (perda total da espessura						Leve	Masculino	de 46 a 55 anos
	Estágio 3 (perda total da espessura						Leve	Feminino	de 36 a 45 anos
	Estágio 4 (perda total da espessura						Leve	Masculino	de 56 a 65 anos
	Estágio 4 (perda total da espessura						Leve	Feminino	de 66 a 75 anos
	Estágio 4 (perda total da espessura						Leve	Masculino	de 66 a 75 anos
	Estágio 4 (perda total da espessura						Leve	Masculino	de 36 a 45 anos
	Estágio 3 (perda total da espessura						Leve	Masculino	de 66 a 75 anos
	Estágio 3 (perda total da espessura						Leve	Masculino	de 66 a 75 anos
	Estágio 3 (perda total da espessura						Leve	Masculino	de 46 a 55 anos
	Estágio 3 (perda total da espessura						Leve	Masculino	de 46 a 55 anos
							Leve	Masculino	de 56 a 65 anos

							Leve	Masculino	de 56 a 65 anos
							Leve	Masculino	de 76 a 85 anos
							Leve	Masculino	de 66 a 75 anos
							Leve	Feminino	de 76 a 85 anos
							Moderado	Masculino	de 56 a 65 anos
	Estágio 3 (perda total da espessura						Leve	Feminino	de 56 a 65 anos
	Estágio 3 (perda total da espessura						Leve	Feminino	de 56 a 65 anos
	Estágio 4 (perda total da espessura						Leve	Feminino	de 56 a 65 anos
	Estágio 3 (perda total da espessura						Leve	Feminino	de 76 a 85 anos
	Estágio 3 (perda total da espessura						Leve	Feminino	de 76 a 85 anos
	Estágio 3 (perda total da espessura						Leve	Masculino	de 76 a 85 anos
	Estágio 3 (perda total da espessura						Leve	Masculino	de 76 a 85 anos
	Estágio 3 (perda total da espessura						Leve	Masculino	de 76 a 85 anos
	Estágio 4 (perda total da espessura						Leve	Masculino	de 66 a 75 anos
	Estágio 3 (perda total da espessura						Leve	Masculino	de 66 a 75 anos

	Estágio 3 (perda total da espessura						Leve	Masculino	de 46 a 55 anos
	Estágio 3 (perda total da espessura						Leve	Feminino	de 18 a 25 anos
	Estágio 4 (perda total da espessura						Leve	Feminino	de 76 a 85 anos
	Estágio 3 (perda total da espessura						Leve	Feminino	de 18 a 25 anos
	Estágio 3 (perda total da espessura						Leve	Feminino	de 18 a 25 anos
	Estágio 3 (perda total da espessura						Leve	Masculino	de 66 a 75 anos
	Estágio 3 (perda total da espessura						Leve	Masculino	de 56 a 65 anos
	Estágio 3 (perda total da espessura						Leve	Feminino	mais de 85 anos
	Estágio 4 (perda total da espessura						Leve	Feminino	de 66 a 75 anos
	Estágio 4 (perda total da espessura						Leve	Masculino	de 56 a 65 anos
	Estágio 3 (perda total da espessura						Leve	Feminino	de 56 a 65 anos
	Estágio 4 (perda total da espessura						Leve	Masculino	de 36 a 45 anos
	Estágio 3 (perda total da espessura						Leve	Masculino	de 66 a 75 anos
	Estágio 3 (perda total da espessura						Leve	Masculino	de 66 a 75 anos
	Estágio 3 (perda total da espessura						Leve	Masculino	de 66 a 75 anos

	Estágio 3 (perda total da espessura						Leve	Masculino	de 66 a 75 anos
	Estágio 3 (perda total da espessura						Leve	Masculino	de 36 a 45 anos
	Estágio 4 (perda total da espessura						Leve	Masculino	de 56 a 65 anos
	Estágio 4 (perda total da espessura						Leve	Masculino	de 26 a 35 anos
	Estágio 3 (perda total da espessura						Leve	Masculino	de 26 a 35 anos
	Estágio 3 (perda total da espessura						Leve	Feminino	de 56 a 65 anos
	Estágio 3 (perda total da espessura						Leve	Masculino	de 56 a 65 anos
	Estágio 3 (perda total da espessura						Leve	Masculino	de 66 a 75 anos
	Estágio 4 (perda total da espessura						Leve	Masculino	de 46 a 55 anos
	Estágio 3 (perda total da espessura						Leve	Masculino	de 66 a 75 anos
	Estágio 3 (perda total da espessura						Leve	Feminino	de 66 a 75 anos
	Estágio 3 (perda total da espessura						Leve	Feminino	de 56 a 65 anos
	Estágio 4 (perda total da espessura						Leve	Feminino	de 56 a 65 anos
	Estágio 4 (perda total da espessura						Leve	Feminino	de 56 a 65 anos
	Estágio 4 (perda total da espessura						Leve	Feminino	de 56 a 65 anos

	Estágio 4 (perda total da espessura						Leve	Feminino	de 36 a 45 anos
	Estágio 3 (perda total da espessura						Leve	Masculino	de 66 a 75 anos
	Estágio 3 (perda total da espessura						Leve	Masculino	de 66 a 75 anos
	Estágio 4 (perda total da espessura						Leve	Masculino	de 66 a 75 anos
	Estágio 3 (perda total da espessura						Leve	Feminino	de 26 a 35 anos
	Estágio 3 (perda total da espessura						Leve	Feminino	de 36 a 45 anos
	Estágio 3 (perda total da espessura						Leve	Feminino	mais de 85 anos
	Estágio 3 (perda total da espessura						Leve	Feminino	mais de 85 anos
	Estágio 4 (perda total da espessura						Leve	Masculino	de 46 a 55 anos
	Estágio 4 (perda total da espessura						Leve	Masculino	de 46 a 55 anos
	Estágio 4 (perda total da espessura						Leve	Masculino	de 46 a 55 anos
	Estágio 4 (perda total da espessura						Leve	Masculino	de 18 a 25 anos
	Estágio 3 (perda total da espessura						Leve	Masculino	de 26 a 35 anos
	Estágio 3 (perda total da espessura						Leve	Masculino	de 66 a 75 anos
	Estágio 3 (perda total da espessura						Leve	Masculino	de 66 a 75 anos

	Estágio 3 (perda total da espessura						Leve	Feminino	de 76 a 85 anos
	Estágio 4 (perda total da espessura						Leve	Feminino	de 66 a 75 anos
	Estágio 3 (perda total da espessura						Leve	Feminino	de 66 a 75 anos
	Estágio 3 (perda total da espessura						Leve	Feminino	de 18 a 25 anos
	Estágio 3 (perda total da espessura						Leve	Feminino	de 18 a 25 anos
	Estágio 3 (perda total da espessura						Leve	Feminino	de 18 a 25 anos
	Estágio 4 (perda total da espessura						Leve	Masculino	de 66 a 75 anos
	Estágio 3 (perda total da espessura						Leve	Feminino	de 76 a 85 anos
	Estágio 3 (perda total da espessura						Leve	Feminino	de 46 a 55 anos
	Estágio 3 (perda total da espessura						Leve	Feminino	de 46 a 55 anos
	Estágio 4 (perda total da espessura						Leve	Feminino	de 26 a 35 anos
	Estágio 4 (perda total da espessura						Leve	Masculino	de 66 a 75 anos
	Estágio 4 (perda total da espessura						Leve	Masculino	de 46 a 55 anos
	Estágio 3 (perda total da espessura						Leve	Masculino	de 46 a 55 anos
	Estágio 3 (perda total da espessura						Leve	Masculino	de 46 a 55 anos

	Estágio 4 (perda total da espessura						Leve	Feminino	de 56 a 65 anos
	Estágio 4 (perda total da espessura						Leve	Feminino	de 56 a 65 anos
	Estágio 4 (perda total da espessura						Leve	Feminino	de 56 a 65 anos
	Estágio 3 (perda total da espessura						Leve	Masculino	de 46 a 55 anos
	Estágio 3 (perda total da espessura						Leve	Masculino	de 46 a 55 anos
	Estágio 3 (perda total da espessura						Leve	Feminino	de 56 a 65 anos
	Estágio 4 (perda total da espessura						Leve	Feminino	de 76 a 85 anos
	Estágio 3 (perda total da espessura						Leve	Masculino	de 46 a 55 anos
	Estágio 4 (perda total da espessura						Leve	Masculino	de 56 a 65 anos
	Estágio 3 (perda total da espessura						Leve	Masculino	de 56 a 65 anos
	Estágio 3 (perda total da espessura						Leve	Feminino	de 66 a 75 anos
	Estágio 3 (perda total da espessura						Leve	Feminino	de 56 a 65 anos
	Estágio 3 (perda total da espessura						Leve	Masculino	de 66 a 75 anos
	Estágio 3 (perda total da espessura						Leve	Feminino	de 56 a 65 anos
	Estágio 3 (perda total da espessura						Leve	Feminino	de 56 a 65 anos

	Estágio 3 (perda total da espessura						Leve	Feminino	de 66 a 75 anos
	Estágio 3 (perda total da espessura						Leve	Feminino	de 66 a 75 anos
	Estágio 3 (perda total da espessura						Leve	Masculino	de 66 a 75 anos
	Estágio 4 (perda total da espessura						Leve	Masculino	de 18 a 25 anos
	Estágio 3 (perda total da espessura						Leve	Feminino	de 46 a 55 anos
	Estágio 3 (perda total da espessura						Leve	Masculino	de 46 a 55 anos
	Estágio 3 (perda total da espessura						Leve	Feminino	de 46 a 55 anos
	Estágio 3 (perda total da espessura						Leve	Masculino	de 76 a 85 anos
	Estágio 3 (perda total da espessura						Leve	Feminino	de 76 a 85 anos
	Estágio 4 (perda total da espessura						Leve	Masculino	de 66 a 75 anos
	Estágio 3 (perda total da espessura						Leve	Masculino	de 18 a 25 anos
	Estágio 3 (perda total da espessura						Leve	Masculino	de 18 a 25 anos
	Estágio 3 (perda total da espessura						Leve	Masculino	de 66 a 75 anos
	Estágio 3 (perda total da espessura						Leve	Feminino	de 66 a 75 anos
	Estágio 3 (perda total da espessura						Leve	Feminino	de 46 a 55 anos

	Estágio 3 (perda total da espessura						Leve	Feminino	de 46 a 55 anos
	Estágio 4 (perda total da espessura						Leve	Masculino	de 26 a 35 anos
							Leve	Masculino	de 26 a 35 anos
	Estágio 3 (perda total da espessura						Leve	Masculino	de 26 a 35 anos
	Estágio 3 (perda total da espessura						Leve	Feminino	de 56 a 65 anos
	Estágio 3 (perda total da espessura						Leve	Masculino	de 56 a 65 anos
	Estágio 4 (perda total da espessura						Leve	Masculino	de 46 a 55 anos
	Estágio 4 (perda total da espessura						Leve	Masculino	de 26 a 35 anos
	Estágio 3 (perda total da espessura						Leve	Masculino	de 66 a 75 anos
	Estágio 3 (perda total da espessura						Leve	Masculino	de 76 a 85 anos
	Estágio 3 (perda total da espessura						Leve	Masculino	de 76 a 85 anos
	Estágio 4 (perda total da espessura						Leve	Feminino	de 46 a 55 anos
	Estágio 4 (perda total da espessura						Leve	Masculino	de 56 a 65 anos
	Estágio 3 (perda total da espessura						Leve	Masculino	de 56 a 65 anos
	Estágio 4 (perda total da espessura						Leve	Masculino	de 56 a 65 anos

	Estágio 3 (perda total da espessura						Leve	Masculino	de 66 a 75 anos
	Estágio 3 (perda total da espessura						Leve	Feminino	de 18 a 25 anos
							Leve	Feminino	de 76 a 85 anos
							Leve	Masculino	de 46 a 55 anos
							Leve	Masculino	de 56 a 65 anos
							Leve	Masculino	de 46 a 55 anos
							Leve	Feminino	de 46 a 55 anos
							Leve	Masculino	de 36 a 45 anos
							Leve	Masculino	de 66 a 75 anos
	Estágio 3 (perda total da espessura						Leve	Feminino	de 46 a 55 anos
	Estágio 3 (perda total da espessura						Leve	Feminino	de 66 a 75 anos
	Estágio 4 (perda total da espessura						Leve	Masculino	de 56 a 65 anos
	Estágio 4 (perda total da espessura						Leve	Masculino	de 56 a 65 anos
	Estágio 3 (perda total da espessura						Leve	Masculino	de 56 a 65 anos
	Estágio 3 (perda total da espessura						Leve	Masculino	de 76 a 85 anos

	Estágio 3 (perda total da espessura						Leve	Masculino	de 46 a 55 anos
	Estágio 4 (perda total da espessura						Leve	Masculino	de 76 a 85 anos
	Estágio 4 (perda total da espessura						Leve	Feminino	de 76 a 85 anos
	Estágio 3 (perda total da espessura						Leve	Feminino	de 76 a 85 anos
	Estágio 3 (perda total da espessura						Leve	Masculino	de 56 a 65 anos
	Estágio 3 (perda total da espessura						Leve	Masculino	de 56 a 65 anos
	Estágio 4 (perda total da espessura						Leve	Feminino	de 76 a 85 anos
	Estágio 4 (perda total da espessura						Leve	Feminino	de 76 a 85 anos
	Estágio 4 (perda total da espessura						Leve	Feminino	de 76 a 85 anos
	Estágio 3 (perda total da espessura						Leve	Masculino	mais de 85 anos
	Estágio 3 (perda total da espessura						Leve	Feminino	de 56 a 65 anos
	Estágio 4 (perda total da espessura						Leve	Feminino	de 56 a 65 anos
	Estágio 3 (perda total da espessura						Leve	Masculino	de 66 a 75 anos
	Estágio 3 (perda total da espessura						Leve	Feminino	de 26 a 35 anos
	Estágio 4 (perda total da espessura						Leve	Masculino	de 56 a 65 anos

	Estagio 3 (perda total da espessura						Leve	Masculino	de 56 a 65 anos
	Estagio 3 (perda total da espessura						Leve	Feminino	de 46 a 55 anos
	Estagio 3 (perda total da espessura						Leve	Feminino	de 46 a 55 anos
	Estagio 4 (perda total da espessura						Leve	Masculino	de 66 a 75 anos
	Estagio 4 (perda total da espessura						Leve	Masculino	de 66 a 75 anos
	Estagio 3 (perda total da espessura						Leve	Masculino	de 66 a 75 anos
	Estagio 3 (perda total da espessura						Leve	Masculino	de 66 a 75 anos
	Estagio 3 (perda total da espessura						Leve	Feminino	de 18 a 25 anos
	Estagio 3 (perda total da espessura						Leve	Masculino	de 76 a 85 anos
	Estagio 4 (perda total da espessura						Leve	Feminino	de 56 a 65 anos
	Estagio 4 (perda total da espessura						Leve	Feminino	de 56 a 65 anos
	Estagio 3 (perda total da espessura						Leve	Feminino	de 76 a 85 anos
	Estagio 3 (perda total da espessura						Leve	Feminino	de 76 a 85 anos
	Estagio 3 (perda total da espessura						Leve	Masculino	de 56 a 65 anos
	Estagio 3 (perda total da espessura						Leve	Feminino	de 66 a 75 anos

	Estágio 3 (perda total da espessura						Leve	Feminino	de 56 a 65 anos
	Estágio 3 (perda total da espessura						Leve	Feminino	de 56 a 65 anos
	Estágio 3 (perda total da espessura						Leve	Feminino	de 56 a 65 anos
	Estágio 3 (perda total da espessura						Leve	Feminino	de 56 a 65 anos
	Estágio 3 (perda total da espessura						Leve	Masculino	mais de 85 anos
							Leve	Masculino	de 76 a 85 anos
	Estágio 3 (perda total da espessura						Leve	Feminino	de 46 a 55 anos
	Estágio 4 (perda total da espessura						Leve	Feminino	de 66 a 75 anos
	Estágio 3 (perda total da espessura						Leve	Feminino	de 46 a 55 anos
	Estágio 4 (perda total da espessura						Leve	Masculino	de 26 a 35 anos
	Estágio 4 (perda total da espessura						Leve	Masculino	de 26 a 35 anos
	Estágio 3 (perda total da espessura						Leve	Masculino	de 76 a 85 anos
	Estágio 3 (perda total da espessura						Leve	Masculino	de 56 a 65 anos
	Estágio 4 (perda total da espessura						Leve	Masculino	de 76 a 85 anos
	Estágio 3 (perda total da espessura						Leve	Masculino	de 76 a 85 anos

	Estágio 3 (perda total da espessura						Leve	Masculino	de 76 a 85 anos
	Estágio 3 (perda total da espessura						Leve	Masculino	de 76 a 85 anos
	Estágio 4 (perda total da espessura						Leve	Masculino	de 76 a 85 anos
	Estágio 3 (perda total da espessura						Leve	Masculino	de 46 a 55 anos
	Estágio 4 (perda total da espessura						Leve	Masculino	de 56 a 65 anos
	Estágio 3 (perda total da espessura						Leve	Masculino	de 56 a 65 anos
	Estágio 3 (perda total da espessura						Leve	Masculino	de 56 a 65 anos
	Estágio 4 (perda total da espessura						Leve	Feminino	de 26 a 35 anos
	Estágio 4 (perda total da espessura						Leve	Feminino	de 26 a 35 anos
	Estágio 4 (perda total da espessura						Leve	Feminino	de 56 a 65 anos
	Estágio 3 (perda total da espessura						Leve	Feminino	de 36 a 45 anos
	Estágio 3 (perda total da espessura						Leve	Feminino	de 36 a 45 anos
	Estágio 3 (perda total da espessura						Leve	Masculino	de 66 a 75 anos
	Estágio 3 (perda total da espessura						Leve	Masculino	de 56 a 65 anos
	Estágio 3 (perda total da espessura						Leve	Feminino	de 36 a 45 anos

	Estágio 3 (perda total da espessura						Leve	Feminino	de 18 a 25 anos
	Estágio 3 (perda total da espessura						Leve	Feminino	de 18 a 25 anos
	Estágio 4 (perda total da espessura						Leve	Masculino	de 66 a 75 anos
	Estágio 4 (perda total da espessura						Leve	Masculino	de 18 a 25 anos
	Estágio 4 (perda total da espessura						Leve	Masculino	de 18 a 25 anos
	Estágio 4 (perda total da espessura						Leve	Masculino	de 18 a 25 anos
	Estágio 3 (perda total da espessura						Leve	Feminino	de 18 a 25 anos
	Estágio 3 (perda total da espessura						Leve	Feminino	de 18 a 25 anos
	Estágio 3 (perda total da espessura						Leve	Masculino	de 76 a 85 anos
	Estágio 3 (perda total da espessura						Leve	Masculino	de 66 a 75 anos
	Estágio 3 (perda total da espessura						Leve	Masculino	de 26 a 35 anos
	Estágio 3 (perda total da espessura						Leve	Feminino	de 56 a 65 anos
	Estágio 3 (perda total da espessura						Leve	Feminino	de 76 a 85 anos
	Estágio 3 (perda total da espessura						Leve	Feminino	de 76 a 85 anos
	Estágio 3 (perda total da espessura						Leve	Masculino	de 36 a 45 anos

	Estágio 3 (perda total da espessura						Leve	Masculino	de 36 a 45 anos
	Estágio 4 (perda total da espessura						Leve	Masculino	de 76 a 85 anos
	Estágio 3 (perda total da espessura						Leve	Masculino	de 76 a 85 anos
	Estágio 3 (perda total da espessura						Leve	Masculino	de 76 a 85 anos
	Estágio 3 (perda total da espessura						Leve	Masculino	de 76 a 85 anos
	Estágio 4 (perda total da espessura						Leve	Masculino	de 76 a 85 anos
	Estágio 4 (perda total da espessura						Leve	Feminino	de 26 a 35 anos
	Estágio 3 (perda total da espessura						Leve	Masculino	de 76 a 85 anos
	Estágio 4 (perda total da espessura						Leve	Masculino	de 36 a 45 anos
	Estágio 4 (perda total da espessura						Leve	Masculino	de 66 a 75 anos
	Estágio 3 (perda total da espessura						Leve	Masculino	de 56 a 65 anos
	Estágio 4 (perda total da espessura						Leve	Masculino	de 56 a 65 anos
	Estágio 3 (perda total da espessura						Leve	Feminino	de 66 a 75 anos
	Estágio 3 (perda total da espessura						Leve	Masculino	de 26 a 35 anos
	Estágio 3 (perda total da espessura						Leve	Masculino	de 26 a 35 anos

	Estágio 3 (perda total da espessura						Leve	Masculino	de 26 a 35 anos
	Estágio 4 (perda total da espessura						Leve	Masculino	de 66 a 75 anos
	Estágio 4 (perda total da espessura						Leve	Masculino	de 66 a 75 anos
	Estágio 3 (perda total da espessura						Leve	Masculino	de 66 a 75 anos
	Estágio 3 (perda total da espessura						Leve	Masculino	de 66 a 75 anos
	Estágio 3 (perda total da espessura						Leve	Masculino	de 36 a 45 anos
	Estágio 3 (perda total da espessura						Leve	Masculino	de 56 a 65 anos
	Estágio 4 (perda total da espessura						Leve	Masculino	de 36 a 45 anos
	Estágio 3 (perda total da espessura						Leve	Masculino	de 66 a 75 anos
	Estágio 3 (perda total da espessura						Leve	Feminino	de 36 a 45 anos
	Estágio 3 (perda total da espessura						Leve	Feminino	de 36 a 45 anos
	Estágio 3 (perda total da espessura						Leve	Masculino	de 66 a 75 anos
	Estágio 3 (perda total da espessura						Leve	Feminino	de 56 a 65 anos
	Estágio 4 (perda total da espessura						Leve	Masculino	de 26 a 35 anos
	Estágio 3 (perda total da espessura						Leve	Masculino	de 18 a 25 anos

	Estagio 4 (perda total da espessura						Leve	Masculino	de 36 a 45 anos
	Estagio 3 (perda total da espessura						Leve	Feminino	de 56 a 65 anos
	Estagio 3 (perda total da espessura						Leve	Feminino	de 56 a 65 anos
	Estagio 4 (perda total da espessura						Leve	Masculino	de 66 a 75 anos
	Estagio 4 (perda total da espessura						Leve	Masculino	de 66 a 75 anos
	Estagio 3 (perda total da espessura						Leve	Masculino	de 66 a 75 anos
	Estagio 4 (perda total da espessura						Leve	Masculino	de 26 a 35 anos
	Estagio 4 (perda total da espessura						Leve	Masculino	de 26 a 35 anos
	Estagio 3 (perda total da espessura						Leve	Feminino	de 56 a 65 anos
	Estagio 4 (perda total da espessura						Leve	Masculino	de 36 a 45 anos
	Estagio 4 (perda total da espessura						Leve	Feminino	de 46 a 55 anos
	Estagio 3 (perda total da espessura						Leve	Masculino	de 66 a 75 anos
	Estagio 4 (perda total da espessura						Leve	Feminino	de 66 a 75 anos
	Estagio 3 (perda total da espessura						Leve	Feminino	de 76 a 85 anos
	Estagio 3 (perda total da espessura						Leve	Feminino	de 18 a 25 anos

	Estágio 3 (perda total da espessura						Leve	Masculino	de 76 a 85 anos
	Estágio 3 (perda total da espessura						Leve	Masculino	de 36 a 45 anos
	Estágio 3 (perda total da espessura						Leve	Masculino	de 26 a 35 anos
	Estágio 4 (perda total da espessura						Leve	Masculino	de 46 a 55 anos
							Leve	Masculino	de 46 a 55 anos
	Estágio 3 (perda total da espessura						Leve	Masculino	de 46 a 55 anos
	Estágio 4 (perda total da espessura						Leve	Masculino	de 66 a 75 anos
	Estágio 3 (perda total da espessura						Leve	Feminino	de 66 a 75 anos
	Estágio 3 (perda total da espessura						Leve	Feminino	de 36 a 45 anos
	Estágio 3 (perda total da espessura						Leve	Feminino	de 36 a 45 anos
	Estágio 3 (perda total da espessura						Leve	Feminino	de 66 a 75 anos
							Leve	Feminino	de 76 a 85 anos
							Leve	Feminino	de 56 a 65 anos
	Estágio 4 (perda total da espessura						Leve	Feminino	de 56 a 65 anos
	Estágio 3 (perda total da espessura						Leve	Masculino	de 26 a 35 anos

	Estágio 3 (perda total da espessura						Leve	Masculino	de 56 a 65 anos
	Estágio 3 (perda total da espessura						Leve	Feminino	de 46 a 55 anos
	Estágio 3 (perda total da espessura						Leve	Feminino	de 66 a 75 anos
	Estágio 3 (perda total da espessura						Leve	Feminino	de 66 a 75 anos
	Estágio 3 (perda total da espessura						Leve	Feminino	de 18 a 25 anos
	Estágio 2 (perda parcial da espessura						Leve	Feminino	de 66 a 75 anos
	Estágio 3 (perda total da espessura						Leve	Feminino	de 46 a 55 anos
	Estágio 3 (perda total da espessura						Leve	Feminino	de 18 a 25 anos
	Estágio 3 (perda total da espessura						Leve	Masculino	de 76 a 85 anos
	Estágio 3 (perda total da espessura						Leve	Feminino	de 56 a 65 anos
	Estágio 4 (perda total da espessura						Leve	Feminino	de 56 a 65 anos
	Estágio 3 (perda total da espessura						Leve	Feminino	de 46 a 55 anos
	Estágio 3 (perda total da espessura						Leve	Masculino	de 36 a 45 anos
	Estágio 3 (perda total da espessura						Leve	Masculino	de 36 a 45 anos
	Estágio 3 (perda total da espessura						Leve	Masculino	de 46 a 55 anos

	Estágio 3 (perda total da espessura						Leve	Feminino	de 46 a 55 anos
	Estágio 3 (perda total da espessura						Leve	Feminino	de 46 a 55 anos
	Estágio 3 (perda total da espessura						Leve	Feminino	de 66 a 75 anos
	Estágio 3 (perda total da espessura						Leve	Feminino	de 46 a 55 anos
	Estágio 3 (perda total da espessura						Leve	Feminino	de 36 a 45 anos
	Estágio 3 (perda total da espessura						Leve	Feminino	de 76 a 85 anos
	Estágio 3 (perda total da espessura						Leve	Masculino	mais de 85 anos
	Estágio 3 (perda total da espessura						Leve	Feminino	de 56 a 65 anos
	Estágio 3 (perda total da espessura						Leve	Feminino	de 66 a 75 anos
	Estágio 4 (perda total da espessura						Leve	Masculino	de 36 a 45 anos
	Estágio 3 (perda total da espessura						Leve	Masculino	de 26 a 35 anos
	Estágio 4 (perda total da espessura						Leve	Masculino	de 26 a 35 anos
	Estágio 3 (perda total da espessura						Leve	Feminino	de 66 a 75 anos
	Estágio 3 (perda total da espessura						Leve	Masculino	de 26 a 35 anos
	Estágio 4 (perda total da espessura						Leve	Masculino	de 66 a 75 anos

	Estágio 4 (perda total da espessura						Leve	Masculino	de 66 a 75 anos
	Estágio 3 (perda total da espessura						Leve	Feminino	mais de 85 anos
	Estágio 3 (perda total da espessura						Leve	Feminino	mais de 85 anos
	Estágio 3 (perda total da espessura						Leve	Feminino	mais de 85 anos
	Estágio 3 (perda total da espessura						Leve	Masculino	mais de 85 anos
							Leve	Feminino	de 46 a 55 anos
							Leve	Feminino	de 46 a 55 anos
							Leve	Feminino	de 56 a 65 anos
							Leve	Feminino	de 46 a 55 anos
							Leve	Masculino	de 36 a 45 anos
							Leve	Masculino	de 46 a 55 anos
							Leve	Masculino	de 66 a 75 anos
							Leve	Masculino	de 66 a 75 anos
							Leve	Feminino	de 36 a 45 anos
							Moderado	Feminino	de 76 a 85 anos

							Leve	Masculino	de 36 a 45 anos
	Estágio 3 (perda total da espessura						Leve	Masculino	de 18 a 25 anos
	Estágio 3 (perda total da espessura						Leve	Masculino	de 66 a 75 anos
	Estágio 3 (perda total da espessura						Leve	Masculino	de 56 a 65 anos
	Estágio 3 (perda total da espessura						Leve	Masculino	de 66 a 75 anos
	Estágio 4 (perda total da espessura						Leve	Masculino	de 56 a 65 anos
	Estágio 3 (perda total da espessura						Leve	Masculino	de 56 a 65 anos
	Estágio 2 (perda parcial da espessura						Leve	Feminino	de 66 a 75 anos
	Estágio 3 (perda total da espessura						Leve	Feminino	de 46 a 55 anos
	Estágio 3 (perda total da espessura						Leve	Feminino	de 46 a 55 anos
	Estágio 3 (perda total da espessura						Leve	Masculino	de 36 a 45 anos
	Estágio 3 (perda total da espessura						Leve	Masculino	de 76 a 85 anos
	Estágio 3 (perda total da espessura						Leve	Masculino	de 76 a 85 anos
	Estágio 3 (perda total da espessura						Leve	Masculino	de 76 a 85 anos
	Estágio 3 (perda total da espessura						Leve	Feminino	de 76 a 85 anos

	Estágio 3 (perda total da espessura						Leve	Feminino	de 46 a 55 anos
	Estágio 3 (perda total da espessura						Leve	Feminino	de 36 a 45 anos
	Estágio 4 (perda total da espessura						Leve	Masculino	de 56 a 65 anos
	Estágio 4 (perda total da espessura						Leve	Masculino	de 56 a 65 anos
	Estágio 3 (perda total da espessura						Leve	Masculino	de 66 a 75 anos
	Estágio 3 (perda total da espessura						Leve	Masculino	de 46 a 55 anos
	Estágio 3 (perda total da espessura						Leve	Masculino	de 56 a 65 anos
	Estágio 3 (perda total da espessura						Leve	Feminino	mais de 85 anos
	Estágio 2 (perda parcial da espessura						Leve	Feminino	de 76 a 85 anos
	Estágio 3 (perda total da espessura						Leve	Masculino	mais de 85 anos
	Estágio 4 (perda total da espessura						Leve	Masculino	de 76 a 85 anos
	Estágio 3 (perda total da espessura						Leve	Masculino	de 56 a 65 anos
	Estágio 3 (perda total da espessura						Leve	Feminino	mais de 85 anos
	Estágio 3 (perda total da espessura						Leve	Masculino	de 66 a 75 anos
	Estágio 3 (perda total da espessura						Leve	Feminino	de 56 a 65 anos

	Estágio 3 (perda total da espessura						Leve	Feminino	de 76 a 85 anos
	Estágio 3 (perda total da espessura						Leve	Feminino	de 76 a 85 anos
	Estágio 3 (perda total da espessura						Leve	Feminino	de 76 a 85 anos
	Estágio 3 (perda total da espessura						Leve	Masculino	de 26 a 35 anos
	Estágio 3 (perda total da espessura						Leve	Masculino	de 26 a 35 anos
	Estágio 3 (perda total da espessura						Leve	Masculino	de 26 a 35 anos
	Estágio 4 (perda total da espessura						Leve	Masculino	de 26 a 35 anos
	Estágio 4 (perda total da espessura						Leve	Masculino	de 26 a 35 anos
	Estágio 4 (perda total da espessura						Leve	Masculino	de 26 a 35 anos
	Estágio 3 (perda total da espessura						Leve	Masculino	de 26 a 35 anos
	Estágio 4 (perda total da espessura						Leve	Masculino	de 26 a 35 anos
	Estágio 4 (perda total da espessura						Leve	Masculino	de 56 a 65 anos
	Estágio 3 (perda total da espessura						Leve	Feminino	de 76 a 85 anos
	Estágio 3 (perda total da espessura						Leve	Masculino	de 76 a 85 anos
	Estágio 3 (perda total da espessura						Leve	Masculino	de 66 a 75 anos

	Estágio 3 (perda total da espessura						Leve	Masculino	de 66 a 75 anos
	Estágio 4 (perda total da espessura						Leve	Masculino	de 56 a 65 anos
	Estágio 4 (perda total da espessura						Leve	Feminino	mais de 85 anos
	Estágio 4 (perda total da espessura						Leve	Feminino	de 76 a 85 anos
							Leve	Masculino	de 66 a 75 anos
							Leve	Masculino	de 56 a 65 anos
							Leve	Masculino	de 36 a 45 anos
							Leve	Feminino	de 46 a 55 anos
							Leve	Feminino	de 12 a 17 anos
							Moderado	Masculino	de 66 a 75 anos
							Leve	Feminino	de 66 a 75 anos
							Leve	Masculino	de 46 a 55 anos
	Estágio 3 (perda total da espessura						Leve	Masculino	de 76 a 85 anos
	Estágio 3 (perda total da espessura						Leve	Feminino	de 66 a 75 anos
	Estágio 3 (perda total da espessura						Leve	Masculino	de 46 a 55 anos

	Estágio 3 (perda total da espessura						Leve	Feminino	de 12 a 17 anos
	Estágio 3 (perda total da espessura						Leve	Feminino	de 66 a 75 anos
	Estágio 4 (perda total da espessura						Leve	Feminino	de 66 a 75 anos
	Estágio 3 (perda total da espessura						Leve	Feminino	de 66 a 75 anos
	Estágio 3 (perda total da espessura						Leve	Feminino	de 66 a 75 anos
	Estágio 3 (perda total da espessura						Leve	Feminino	de 56 a 65 anos
	Estágio 3 (perda total da espessura						Leve	Masculino	de 56 a 65 anos
	Estágio 3 (perda total da espessura						Leve	Feminino	de 56 a 65 anos
	Estágio 3 (perda total da espessura						Leve	Masculino	de 36 a 45 anos
	Estágio 3 (perda total da espessura						Leve	Masculino	de 36 a 45 anos
	Estágio 4 (perda total da espessura						Leve	Masculino	de 26 a 35 anos
	Estágio 3 (perda total da espessura						Leve	Masculino	de 66 a 75 anos
	Estágio 3 (perda total da espessura						Leve	Feminino	de 66 a 75 anos
	Estágio 3 (perda total da espessura						Leve	Masculino	de 56 a 65 anos
	Estágio 3 (perda total da espessura						Leve	Masculino	de 56 a 65 anos

	Estágio 3 (perda total da espessura						Leve	Masculino	de 56 a 65 anos
	Estágio 4 (perda total da espessura						Leve	Feminino	de 18 a 25 anos
	Estágio 3 (perda total da espessura						Leve	Feminino	de 56 a 65 anos
	Estágio 3 (perda total da espessura						Leve	Feminino	de 66 a 75 anos
	Estágio 3 (perda total da espessura						Leve	Masculino	de 36 a 45 anos
	Estágio 3 (perda total da espessura						Leve	Masculino	de 66 a 75 anos
	Estágio 4 (perda total da espessura						Leve	Masculino	de 56 a 65 anos
	Estágio 4 (perda total da espessura						Leve	Masculino	de 56 a 65 anos
	Estágio 3 (perda total da espessura						Leve	Feminino	de 66 a 75 anos
	Estágio 3 (perda total da espessura						Leve	Masculino	de 66 a 75 anos
	Estágio 3 (perda total da espessura						Leve	Masculino	de 56 a 65 anos
	Estágio 3 (perda total da espessura						Leve	Feminino	de 76 a 85 anos
	Estágio 3 (perda total da espessura						Leve	Masculino	de 56 a 65 anos
	Estágio 3 (perda total da espessura						Leve	Masculino	de 56 a 65 anos
	Estágio 3 (perda total da espessura						Leve	Feminino	de 76 a 85 anos

	Estágio 3 (perda total da espessura						Leve	Masculino	de 56 a 65 anos
	Estágio 4 (perda total da espessura						Leve	Masculino	de 26 a 35 anos
	Estágio 4 (perda total da espessura						Leve	Masculino	de 26 a 35 anos
	Estágio 3 (perda total da espessura						Leve	Masculino	de 26 a 35 anos
	Estágio 3 (perda total da espessura						Leve	Masculino	de 26 a 35 anos
	Estágio 3 (perda total da espessura						Leve	Feminino	de 56 a 65 anos
	Estágio 3 (perda total da espessura						Leve	Feminino	de 56 a 65 anos
	Estágio 3 (perda total da espessura						Leve	Feminino	de 46 a 55 anos
	Estágio 2 (perda parcial da espessura						Leve	Masculino	de 56 a 65 anos
	Estágio 3 (perda total da espessura						Leve	Masculino	de 56 a 65 anos
	Estágio 3 (perda total da espessura						Leve	Masculino	de 56 a 65 anos
	Estágio 3 (perda total da espessura						Leve	Feminino	de 66 a 75 anos
	Estágio 4 (perda total da espessura						Leve	Feminino	de 66 a 75 anos
	Estágio 3 (perda total da espessura						Leve	Feminino	de 56 a 65 anos
	Estágio 3 (perda total da espessura						Leve	Feminino	de 56 a 65 anos

	Estágio 2 (perda parcial da espessura						Leve	Masculino	de 56 a 65 anos
	Estágio 3 (perda total da espessura						Leve	Masculino	de 56 a 65 anos
	Estágio 3 (perda total da espessura						Leve	Feminino	de 18 a 25 anos
	Estágio 4 (perda total da espessura						Leve	Masculino	de 26 a 35 anos
	Estágio 4 (perda total da espessura						Leve	Masculino	de 26 a 35 anos
	Estágio 4 (perda total da espessura						Leve	Masculino	de 76 a 85 anos
	Estágio 4 (perda total da espessura						Leve	Masculino	de 76 a 85 anos
	Estágio 4 (perda total da espessura						Leve	Masculino	de 76 a 85 anos
	Estágio 4 (perda total da espessura						Leve	Masculino	de 76 a 85 anos
	Estágio 4 (perda total da espessura						Leve	Masculino	de 56 a 65 anos
	Estágio 3 (perda total da espessura						Leve	Masculino	de 56 a 65 anos
	Estágio 3 (perda total da espessura						Leve	Masculino	de 46 a 55 anos
	Estágio 3 (perda total da espessura						Leve	Feminino	de 56 a 65 anos
	Estágio 3 (perda total da espessura						Leve	Masculino	de 66 a 75 anos
	Estágio 4 (perda total da espessura						Leve	Masculino	de 56 a 65 anos

	Estágio 4 (perda total da espessura						Leve	Masculino	de 56 a 65 anos
	Estágio 4 (perda total da espessura						Leve	Feminino	de 66 a 75 anos
	Estágio 3 (perda total da espessura						Leve	Masculino	de 76 a 85 anos
	Estágio 3 (perda total da espessura						Leve	Feminino	de 66 a 75 anos
	Estágio 3 (perda total da espessura						Leve	Feminino	de 66 a 75 anos
	Estágio 3 (perda total da espessura						Leve	Feminino	de 66 a 75 anos
	Estágio 3 (perda total da espessura						Leve	Masculino	de 46 a 55 anos
	Estágio 3 (perda total da espessura						Leve	Masculino	de 76 a 85 anos
	Estágio 3 (perda total da espessura						Leve	Feminino	de 56 a 65 anos
							Leve	Feminino	de 56 a 65 anos
							Leve	Masculino	de 76 a 85 anos
							Leve	Feminino	de 66 a 75 anos
							Leve	Feminino	de 26 a 35 anos
							Leve	Feminino	de 66 a 75 anos
							Leve	Masculino	de 66 a 75 anos

							Leve	Masculino	de 46 a 55 anos
							Nenhum	Masculino	de 36 a 45 anos
							Moderado	Masculino	de 46 a 55 anos
	Estágio 2 (perda parcial da espessura						Leve	Feminino	de 66 a 75 anos
							Moderado	Feminino	de 56 a 65 anos
							Moderado	Masculino	de 76 a 85 anos
							Moderado	Masculino	mais de 85 anos
							Moderado	Feminino	de 66 a 75 anos
							Leve	Feminino	de 46 a 55 anos
							Leve	Feminino	de 76 a 85 anos
							Leve	Feminino	de 46 a 55 anos
							Leve	Feminino	de 46 a 55 anos
							Leve	Masculino	de 26 a 35 anos
	Estágio 3 (perda total da espessura						Leve	Masculino	de 66 a 75 anos
	Estágio 3 (perda total da espessura						Leve	Feminino	de 56 a 65 anos

	Estágio 3 (perda total da espessura						Leve	Feminino	de 56 a 65 anos
	Estágio 4 (perda total da espessura						Leve	Feminino	mais de 85 anos
	Estágio 4 (perda total da espessura						Leve	Feminino	de 56 a 65 anos
	Estágio 4 (perda total da espessura						Leve	Masculino	de 56 a 65 anos
	Estágio 4 (perda total da espessura						Leve	Masculino	de 56 a 65 anos
	Estágio 4 (perda total da espessura						Leve	Masculino	de 56 a 65 anos
	Estágio 3 (perda total da espessura						Leve	Masculino	de 56 a 65 anos
	Estágio 3 (perda total da espessura						Leve	Feminino	de 56 a 65 anos
	Estágio 4 (perda total da espessura						Leve	Feminino	de 26 a 35 anos
	Estágio 3 (perda total da espessura						Leve	Masculino	de 46 a 55 anos
	Estágio 4 (perda total da espessura						Leve	Masculino	de 56 a 65 anos
	Estágio 4 (perda total da espessura						Leve	Masculino	de 56 a 65 anos
	Estágio 4 (perda total da espessura						Leve	Masculino	de 56 a 65 anos
	Estágio 3 (perda total da espessura						Leve	Masculino	de 46 a 55 anos
	Estágio 3 (perda total da espessura						Leve	Feminino	de 66 a 75 anos

	Estágio 3 (perda total da espessura						Leve	Masculino	de 76 a 85 anos
	Estágio 3 (perda total da espessura						Leve	Feminino	de 66 a 75 anos
	Estágio 3 (perda total da espessura						Leve	Masculino	de 76 a 85 anos
	Estágio 3 (perda total da espessura						Leve	Feminino	de 66 a 75 anos
	Estágio 3 (perda total da espessura						Leve	Masculino	de 66 a 75 anos
	Estágio 3 (perda total da espessura						Leve	Feminino	de 56 a 65 anos
	Estágio 3 (perda total da espessura						Leve	Masculino	de 76 a 85 anos
	Estágio 4 (perda total da espessura						Leve	Masculino	de 76 a 85 anos
	Estágio 3 (perda total da espessura						Leve	Masculino	de 66 a 75 anos
	Estágio 3 (perda total da espessura						Leve	Masculino	de 26 a 35 anos
	Estágio 3 (perda total da espessura						Leve	Masculino	de 66 a 75 anos
	Estágio 3 (perda total da espessura						Leve	Feminino	de 56 a 65 anos
	Estágio 3 (perda total da espessura						Leve	Feminino	de 56 a 65 anos
	Estágio 3 (perda total da espessura						Leve	Feminino	de 26 a 35 anos
	Estágio 3 (perda total da espessura						Leve	Feminino	de 26 a 35 anos

	Estágio 3 (perda total da espessura						Leve	Feminino	de 26 a 35 anos
	Estágio 3 (perda total da espessura						Leve	Feminino	de 26 a 35 anos
	Estágio 3 (perda total da espessura						Leve	Feminino	de 76 a 85 anos
	Estágio 3 (perda total da espessura						Leve	Feminino	de 76 a 85 anos
	Estágio 3 (perda total da espessura						Leve	Masculino	de 66 a 75 anos
	Estágio 3 (perda total da espessura						Leve	Feminino	mais de 85 anos
	Estágio 4 (perda total da espessura						Leve	Masculino	de 26 a 35 anos
	Estágio 3 (perda total da espessura						Leve	Masculino	de 76 a 85 anos
	Estágio 4 (perda total da espessura						Leve	Masculino	de 46 a 55 anos
	Estágio 4 (perda total da espessura						Leve	Masculino	de 76 a 85 anos
	Estágio 4 (perda total da espessura						Leve	Feminino	de 46 a 55 anos
	Estágio 3 (perda total da espessura						Leve	Masculino	de 56 a 65 anos
	Estágio 4 (perda total da espessura						Leve	Masculino	de 56 a 65 anos
	Estágio 3 (perda total da espessura						Leve	Masculino	de 76 a 85 anos
	Estágio 4 (perda total da espessura						Leve	Masculino	de 46 a 55 anos

	Estágio 4 (perda total da espessura						Leve	Masculino	de 46 a 55 anos
	Estágio 4 (perda total da espessura						Leve	Feminino	de 56 a 65 anos
	Estágio 3 (perda total da espessura						Leve	Masculino	de 76 a 85 anos
	Estágio 3 (perda total da espessura						Leve	Masculino	de 46 a 55 anos
	Estágio 3 (perda total da espessura						Leve	Feminino	de 66 a 75 anos
	Estágio 3 (perda total da espessura						Leve	Masculino	de 46 a 55 anos
	Estágio 3 (perda total da espessura						Leve	Masculino	de 46 a 55 anos
	Estágio 4 (perda total da espessura						Leve	Feminino	de 76 a 85 anos
	Estágio 4 (perda total da espessura						Leve	Masculino	de 46 a 55 anos
	Estágio 4 (perda total da espessura						Leve	Masculino	de 46 a 55 anos
							Leve	Feminino	de 26 a 35 anos
							Leve	Feminino	de 46 a 55 anos
	Estágio 4 (perda total da espessura						Leve	Feminino	de 56 a 65 anos
	Estágio 3 (perda total da espessura						Leve	Masculino	de 18 a 25 anos
							Leve	Masculino	de 56 a 65 anos

							Leve	Masculino	de 76 a 85 anos
	Estagio 3 (perda total da espessura						Leve	Feminino	de 66 a 75 anos
							Leve	Masculino	de 56 a 65 anos
	Estagio 3 (perda total da espessura						Leve	Masculino	de 56 a 65 anos
							Leve	Masculino	de 56 a 65 anos
							Leve	Feminino	de 56 a 65 anos
							Leve	Feminino	de 76 a 85 anos
	Estagio 3 (perda total da espessura						Leve	Feminino	de 56 a 65 anos
	Estagio 3 (perda total da espessura						Leve	Masculino	de 76 a 85 anos
	Estagio 3 (perda total da espessura						Leve	Masculino	de 46 a 55 anos
	Estagio 3 (perda total da espessura						Leve	Feminino	de 76 a 85 anos
	Estagio 3 (perda total da espessura						Leve	Masculino	de 56 a 65 anos
							Moderado	Masculino	de 36 a 45 anos
	Estagio 4 (perda total da espessura						Leve	Feminino	de 76 a 85 anos
	Estagio 4 (perda total da espessura						Leve	Feminino	de 56 a 65 anos

	Estágio 3 (perda total da espessura						Leve	Masculino	de 26 a 35 anos
	Estágio 3 (perda total da espessura						Leve	Masculino	de 46 a 55 anos
	Estágio 3 (perda total da espessura						Leve	Masculino	de 46 a 55 anos
	Estágio 3 (perda total da espessura						Leve	Feminino	de 56 a 65 anos
	Estágio 3 (perda total da espessura						Leve	Feminino	de 56 a 65 anos
	Estágio 3 (perda total da espessura						Leve	Masculino	de 56 a 65 anos
	Estágio 3 (perda total da espessura						Leve	Masculino	de 56 a 65 anos
	Estágio 3 (perda total da espessura						Leve	Feminino	de 56 a 65 anos
	Estágio 3 (perda total da espessura						Leve	Feminino	de 76 a 85 anos
	Estágio 4 (perda total da espessura						Leve	Masculino	de 46 a 55 anos
	Estágio 3 (perda total da espessura						Leve	Feminino	de 76 a 85 anos
	Estágio 3 (perda total da espessura						Leve	Masculino	de 26 a 35 anos
	Estágio 4 (perda total da espessura						Leve	Masculino	de 66 a 75 anos
	Estágio 3 (perda total da espessura						Leve	Masculino	de 66 a 75 anos
	Estágio 4 (perda total da espessura						Leve	Masculino	de 46 a 55 anos

	Estágio 4 (perda total da espessura						Leve	Feminino	de 66 a 75 anos
	Estágio 3 (perda total da espessura						Leve	Masculino	de 56 a 65 anos
	Estágio 3 (perda total da espessura						Leve	Feminino	de 56 a 65 anos
	Estágio 4 (perda total da espessura						Leve	Masculino	de 76 a 85 anos
	Estágio 4 (perda total da espessura						Leve	Masculino	de 66 a 75 anos
	Estágio 4 (perda total da espessura						Leve	Masculino	de 36 a 45 anos
	Estágio 3 (perda total da espessura						Leve	Masculino	de 26 a 35 anos
	Estágio 3 (perda total da espessura						Leve	Feminino	de 36 a 45 anos
	Estágio 3 (perda total da espessura						Leve	Masculino	de 36 a 45 anos
	Estágio 3 (perda total da espessura						Leve	Masculino	de 76 a 85 anos
	Estágio 3 (perda total da espessura						Leve	Feminino	de 56 a 65 anos
	Estágio 4 (perda total da espessura						Leve	Masculino	de 56 a 65 anos
	Estágio 4 (perda total da espessura						Leve	Feminino	de 46 a 55 anos
	Estágio 3 (perda total da espessura						Leve	Feminino	de 76 a 85 anos
	Estágio 3 (perda total da espessura						Leve	Feminino	de 56 a 65 anos

	Estágio 3 (perda total da espessura						Leve	Feminino	de 66 a 75 anos
	Estágio 3 (perda total da espessura						Leve	Feminino	de 66 a 75 anos
	Estágio 3 (perda total da espessura						Leve	Masculino	de 66 a 75 anos
	Estágio 4 (perda total da espessura						Leve	Masculino	de 56 a 65 anos
							Leve	Masculino	de 36 a 45 anos
							Nenhum	Feminino	de 56 a 65 anos
							Leve	Feminino	de 76 a 85 anos
							Nenhum	Masculino	de 56 a 65 anos
							Leve	Masculino	de 46 a 55 anos
							Leve	Feminino	de 36 a 45 anos
							Leve	Masculino	de 56 a 65 anos
							Leve	Masculino	de 56 a 65 anos
							Leve	Feminino	de 26 a 35 anos
							Leve	Feminino	de 46 a 55 anos
							Leve	Feminino	de 36 a 45 anos

							Leve	Feminino	de 46 a 55 anos
							Leve	Feminino	de 46 a 55 anos
							Leve	Feminino	de 66 a 75 anos
							Leve	Feminino	de 56 a 65 anos
							Moderado	Masculino	de 56 a 65 anos
							Leve	Masculino	de 36 a 45 anos
							Leve	Feminino	de 66 a 75 anos
							Moderado	Feminino	de 26 a 35 anos
							Leve	Masculino	de 56 a 65 anos
							Moderado	Masculino	de 56 a 65 anos
							Leve	Feminino	de 18 a 25 anos
							Moderado	Masculino	de 66 a 75 anos
							Leve	Masculino	de 26 a 35 anos
							Leve	Masculino	de 36 a 45 anos
							Leve	Feminino	de 76 a 85 anos

							Leve	Feminino	de 66 a 75 anos
							Leve	Feminino	de 46 a 55 anos
							Leve	Masculino	de 26 a 35 anos
							Leve	Masculino	de 18 a 25 anos
							Leve	Masculino	de 36 a 45 anos
							Leve	Feminino	de 66 a 75 anos
							Leve	Feminino	de 66 a 75 anos
							Leve	Masculino	de 66 a 75 anos
							Grave	Feminino	de 76 a 85 anos
							Nenhum	Masculino	de 36 a 45 anos
							Leve	Masculino	de 56 a 65 anos
							Leve	Masculino	de 36 a 45 anos
							Leve	Masculino	de 36 a 45 anos
							Moderado	Feminino	de 26 a 35 anos
							Moderado	Masculino	de 66 a 75 anos

							Leve	Masculino	de 46 a 55 anos
							Nenhum	Feminino	de 46 a 55 anos
							Nenhum	Feminino	de 36 a 45 anos
							Nenhum	Feminino	de 46 a 55 anos
							Leve	Masculino	de 46 a 55 anos
							Nenhum	Masculino	de 46 a 55 anos
	Estágio 3 (perda total da espessura						Moderado	Masculino	de 66 a 75 anos
	Estágio 3 (perda total da espessura						Moderado	Feminino	de 46 a 55 anos
	Estágio 3 (perda total da espessura						Moderado	Feminino	mais de 85 anos
	Estágio 3 (perda total da espessura						Moderado	Masculino	de 76 a 85 anos
	Estágio 3 (perda total da espessura						Grave	Feminino	de 46 a 55 anos
							Nenhum	Feminino	de 56 a 65 anos
	Estágio 3 (perda total da espessura						Moderado	Feminino	de 66 a 75 anos
	Estágio 3 (perda total da espessura						Moderado	Feminino	de 66 a 75 anos
	Estágio 3 (perda total da espessura						Moderado	Feminino	de 56 a 65 anos

	Estágio 4 (perda total da espessura						Moderado	Feminino	de 36 a 45 anos
	Estágio 4 (perda total da espessura						Moderado	Masculino	de 46 a 55 anos
	Estágio 3 (perda total da espessura						Moderado	Feminino	de 56 a 65 anos
	Estágio 3 (perda total da espessura						Moderado	Masculino	de 76 a 85 anos
	Estágio 3 (perda total da espessura						Moderado	Masculino	de 56 a 65 anos
	Estágio 3 (perda total da espessura						Moderado	Masculino	de 36 a 45 anos
	Estágio 4 (perda total da espessura						Moderado	Masculino	de 56 a 65 anos
	Estágio 3 (perda total da espessura						Moderado	Masculino	de 36 a 45 anos
	Estágio 2 (perda parcial da espessura						Leve	Feminino	de 56 a 65 anos
	Estágio 1 (pele íntegra com						Nenhum	Feminino	de 46 a 55 anos
	Estágio 2 (perda parcial da espessura						Leve	Masculino	de 46 a 55 anos
	Estágio 2 (perda parcial da espessura						Leve	Masculino	de 56 a 65 anos
	Estágio 1 (pele íntegra com						Leve	Feminino	de 46 a 55 anos
	Estágio 2 (perda parcial da espessura						Leve	Masculino	de 46 a 55 anos
	Estágio 2 (perda parcial da espessura						Leve	Masculino	de 56 a 65 anos

	Estágio 2 (perda parcial da espessura						Leve	Feminino	de 76 a 85 anos
	Estágio 2 (perda parcial da espessura						Leve	Feminino	de 76 a 85 anos
	Estágio 2 (perda parcial da espessura						Leve	Feminino	de 76 a 85 anos
	Estágio 2 (perda parcial da espessura						Leve	Masculino	de 56 a 65 anos
	Estágio 2 (perda parcial da espessura						Leve	Masculino	de 56 a 65 anos
	Estágio 2 (perda parcial da espessura						Leve	Masculino	de 46 a 55 anos
							Leve	Masculino	de 46 a 55 anos
	Estágio 2 (perda parcial da espessura						Leve	Feminino	mais de 85 anos
	Estágio 2 (perda parcial da espessura						Leve	Feminino	mais de 85 anos
	Estágio 2 (perda parcial da espessura						Leve	Masculino	de 76 a 85 anos
	Estágio 2 (perda parcial da espessura						Leve	Masculino	de 46 a 55 anos
	Estágio 2 (perda parcial da espessura						Leve	Feminino	de 66 a 75 anos
	Estágio 2 (perda parcial da espessura						Leve	Feminino	de 46 a 55 anos
	Estágio 1 (pele íntegra com						Leve	Feminino	de 76 a 85 anos
	Estágio 2 (perda parcial da espessura						Leve	Feminino	de 56 a 65 anos

	Estágio 2 (perda parcial da espessura						Leve	Masculino	de 56 a 65 anos
	Estágio 2 (perda parcial da espessura						Leve	Feminino	de 66 a 75 anos
	Estágio 1 (pele íntegra com						Leve	Masculino	de 36 a 45 anos
	Estágio 2 (perda parcial da espessura						Leve	Feminino	de 46 a 55 anos
	Estágio 1 (pele íntegra com						Leve	Feminino	de 46 a 55 anos
	Estágio 1 (pele íntegra com						Leve	Masculino	de 66 a 75 anos
	Estágio 1 (pele íntegra com						Leve	Masculino	de 46 a 55 anos
	Estágio 2 (perda parcial da espessura						Leve	Masculino	de 66 a 75 anos
	Estágio 2 (perda parcial da espessura						Leve	Masculino	de 56 a 65 anos
	Estágio 2 (perda parcial da espessura						Leve	Feminino	de 76 a 85 anos
	Estágio 2 (perda parcial da espessura						Leve	Feminino	de 36 a 45 anos
	Estágio 2 (perda parcial da espessura						Leve	Masculino	de 46 a 55 anos
	Estágio 2 (perda parcial da espessura						Leve	Feminino	de 56 a 65 anos
	Estágio 2 (perda parcial da espessura						Leve	Feminino	de 56 a 65 anos
	Estágio 1 (pele íntegra com						Leve	Feminino	de 46 a 55 anos

	Estágio 2 (perda parcial da espessura						Leve	Masculino	de 46 a 55 anos
	Estágio 2 (perda parcial da espessura						Leve	Masculino	de 46 a 55 anos
	Estágio 2 (perda parcial da espessura						Leve	Masculino	de 36 a 45 anos
	Estágio 1 (pele íntegra com						Leve	Masculino	de 76 a 85 anos
	Estágio 2 (perda parcial da espessura						Leve	Feminino	de 46 a 55 anos
	Estágio 2 (perda parcial da espessura						Leve	Feminino	de 66 a 75 anos
	Estágio 2 (perda parcial da espessura						Leve	Feminino	de 66 a 75 anos
	Estágio 2 (perda parcial da espessura						Leve	Feminino	de 56 a 65 anos
	Estágio 2 (perda parcial da espessura						Leve	Masculino	de 36 a 45 anos
	Estágio 2 (perda parcial da espessura						Leve	Masculino	de 66 a 75 anos
	Estágio 2 (perda parcial da espessura						Leve	Masculino	de 56 a 65 anos
	Estágio 2 (perda parcial da espessura						Leve	Feminino	de 66 a 75 anos
	Estágio 2 (perda parcial da espessura						Leve	Feminino	de 46 a 55 anos
	Estágio 2 (perda parcial da espessura						Leve	Feminino	de 46 a 55 anos
	Estágio 2 (perda parcial da espessura						Leve	Feminino	de 46 a 55 anos

	Estágio 2 (perda parcial da espessura						Leve	Feminino	de 56 a 65 anos
	Estágio 2 (perda parcial da espessura						Leve	Feminino	de 66 a 75 anos
	Estágio 2 (perda parcial da espessura						Leve	Masculino	de 56 a 65 anos
							Leve	Masculino	de 56 a 65 anos
	Estágio 2 (perda parcial da espessura						Leve	Feminino	de 66 a 75 anos
	Estágio 2 (perda parcial da espessura						Leve	Feminino	de 66 a 75 anos
							Leve	Feminino	de 66 a 75 anos
	Estágio 2 (perda parcial da espessura						Leve	Feminino	de 66 a 75 anos
	Estágio 2 (perda parcial da espessura						Leve	Feminino	de 66 a 75 anos
	Estágio 2 (perda parcial da espessura						Leve	Feminino	de 76 a 85 anos
	Estágio 2 (perda parcial da espessura						Leve	Feminino	de 76 a 85 anos
	Estágio 2 (perda parcial da espessura						Leve	Feminino	de 76 a 85 anos
	Estágio 2 (perda parcial da espessura						Leve	Feminino	de 56 a 65 anos
	Estágio 2 (perda parcial da espessura						Leve	Masculino	mais de 85 anos
	Estágio 2 (perda parcial da espessura						Leve	Feminino	de 36 a 45 anos

							Leve	Feminino	de 36 a 45 anos
							Leve	Feminino	de 36 a 45 anos
	Estágio 2 (perda parcial da espessura						Leve	Feminino	mais de 85 anos
	Estágio 2 (perda parcial da espessura						Leve	Masculino	de 66 a 75 anos
	Estágio 1 (pele íntegra com						Leve	Masculino	de 46 a 55 anos
	Estágio 2 (perda parcial da espessura						Leve	Feminino	de 66 a 75 anos
	Estágio 2 (perda parcial da espessura						Leve	Feminino	de 76 a 85 anos
	Estágio 2 (perda parcial da espessura						Leve	Masculino	de 76 a 85 anos
	Estágio 1 (pele íntegra com						Leve	Feminino	de 18 a 25 anos
	Estágio 1 (pele íntegra com						Leve	Masculino	de 66 a 75 anos
	Estágio 2 (perda parcial da espessura						Leve	Masculino	de 26 a 35 anos
	Estágio 2 (perda parcial da espessura						Leve	Feminino	de 66 a 75 anos
	Estágio 1 (pele íntegra com						Leve	Feminino	de 66 a 75 anos
	Estágio 1 (pele íntegra com						Leve	Feminino	de 66 a 75 anos
	Estágio 2 (perda parcial da espessura						Leve	Feminino	de 66 a 75 anos

							Grave	Masculino	de 66 a 75 anos
	Estágio 1 (pele íntegra com						Leve	Masculino	de 46 a 55 anos
	Estágio 1 (pele íntegra com						Leve	Masculino	de 46 a 55 anos
	Estágio 2 (perda parcial da espessura						Leve	Masculino	de 46 a 55 anos
	Estágio 2 (perda parcial da espessura						Leve	Feminino	de 26 a 35 anos
	Estágio 2 (perda parcial da espessura						Leve	Feminino	de 66 a 75 anos
	Estágio 2 (perda parcial da espessura						Leve	Feminino	de 66 a 75 anos
	Estágio 2 (perda parcial da espessura						Leve	Feminino	de 66 a 75 anos
	Estágio 2 (perda parcial da espessura						Leve	Feminino	de 66 a 75 anos
	Estágio 2 (perda parcial da espessura						Leve	Masculino	de 56 a 65 anos
	Estágio 2 (perda parcial da espessura						Leve	Feminino	de 46 a 55 anos
	Estágio 2 (perda parcial da espessura						Leve	Masculino	de 46 a 55 anos
	Estágio 2 (perda parcial da espessura						Leve	Masculino	de 56 a 65 anos
	Estágio 2 (perda parcial da espessura						Leve	Feminino	de 66 a 75 anos
	Estágio 1 (pele íntegra com						Leve	Feminino	de 18 a 25 anos

	Estágio 1 (pele íntegra com						Leve	Feminino	de 36 a 45 anos
	Estágio 2 (perda parcial da espessura						Leve	Feminino	de 56 a 65 anos
							Leve	Masculino	de 56 a 65 anos
							Leve	Masculino	de 36 a 45 anos
							Leve	Masculino	de 56 a 65 anos
	Estágio 1 (pele íntegra com						Leve	Masculino	de 76 a 85 anos
							Leve	Feminino	de 66 a 75 anos
							Leve	Feminino	de 66 a 75 anos
							Leve	Masculino	de 36 a 45 anos
							Leve	Feminino	de 66 a 75 anos
							Leve	Masculino	de 26 a 35 anos
	Estágio 2 (perda parcial da espessura						Leve	Feminino	de 76 a 85 anos
	Estágio 2 (perda parcial da espessura						Leve	Masculino	de 36 a 45 anos
	Estágio 1 (pele íntegra com						Leve	Feminino	de 76 a 85 anos
	Estágio 2 (perda parcial da espessura						Leve	Feminino	de 36 a 45 anos

	Estágio 2 (perda parcial da espessura						Leve	Masculino	de 46 a 55 anos
	Estágio 2 (perda parcial da espessura						Leve	Masculino	de 56 a 65 anos
	Estágio 2 (perda parcial da espessura						Leve	Masculino	de 56 a 65 anos
	Estágio 2 (perda parcial da espessura						Leve	Masculino	de 76 a 85 anos
	Estágio 2 (perda parcial da espessura						Leve	Masculino	de 66 a 75 anos
	Estágio 1 (pele íntegra com						Leve	Feminino	de 66 a 75 anos
	Estágio 2 (perda parcial da espessura						Leve	Masculino	de 76 a 85 anos
	Estágio 2 (perda parcial da espessura						Leve	Masculino	de 76 a 85 anos
	Estágio 2 (perda parcial da espessura						Leve	Masculino	de 66 a 75 anos
	Estágio 2 (perda parcial da espessura						Leve	Masculino	de 76 a 85 anos
	Estágio 2 (perda parcial da espessura						Leve	Feminino	de 76 a 85 anos
	Estágio 2 (perda parcial da espessura						Leve	Feminino	de 36 a 45 anos
	Estágio 2 (perda parcial da espessura						Leve	Masculino	de 56 a 65 anos
	Estágio 2 (perda parcial da espessura						Leve	Masculino	de 56 a 65 anos
	Estágio 2 (perda parcial da espessura						Leve	Feminino	de 46 a 55 anos

	Estágio 2 (perda parcial da espessura						Leve	Masculino	de 56 a 65 anos
	Estágio 1 (pele íntegra com						Leve	Masculino	de 56 a 65 anos
	Estágio 1 (pele íntegra com						Leve	Feminino	de 66 a 75 anos
	Estágio 3 (perda total da espessura						Moderado	Masculino	de 66 a 75 anos
	Estágio 2 (perda parcial da espessura						Leve	Masculino	de 56 a 65 anos
	Estágio 2 (perda parcial da espessura						Leve	Masculino	de 46 a 55 anos
	Estágio 1 (pele íntegra com						Leve	Masculino	de 46 a 55 anos
	Estágio 2 (perda parcial da espessura						Leve	Feminino	de 76 a 85 anos
	Estágio 1 (pele íntegra com						Leve	Feminino	de 76 a 85 anos
	Estágio 2 (perda parcial da espessura						Leve	Feminino	de 56 a 65 anos
	Estágio 1 (pele íntegra com						Leve	Masculino	de 66 a 75 anos
	Estágio 2 (perda parcial da espessura						Leve	Masculino	mais de 85 anos
	Estágio 4 (perda total da espessura						Moderado	Masculino	de 56 a 65 anos
	Estágio 2 (perda parcial da espessura						Leve	Masculino	de 26 a 35 anos
	Estágio 2 (perda parcial da espessura						Leve	Feminino	de 66 a 75 anos

	Estágio 2 (perda parcial da espessura						Leve	Feminino	de 46 a 55 anos
	Estágio 2 (perda parcial da espessura						Leve	Feminino	de 56 a 65 anos
	Estágio 2 (perda parcial da espessura						Leve	Masculino	de 56 a 65 anos
	Estágio 2 (perda parcial da espessura						Leve	Feminino	de 46 a 55 anos
	Estágio 1 (pele íntegra com						Leve	Masculino	de 46 a 55 anos
	Estágio 1 (pele íntegra com						Leve	Masculino	de 56 a 65 anos
	Estágio 2 (perda parcial da espessura						Leve	Masculino	de 56 a 65 anos
	Estágio 2 (perda parcial da espessura						Leve	Masculino	de 46 a 55 anos
	Estágio 1 (pele íntegra com						Leve	Masculino	de 46 a 55 anos
	Estágio 2 (perda parcial da espessura						Leve	Masculino	mais de 85 anos
	Estágio 2 (perda parcial da espessura						Leve	Masculino	de 66 a 75 anos
	Estágio 2 (perda parcial da espessura						Leve	Masculino	de 56 a 65 anos
	Estágio 2 (perda parcial da espessura						Leve	Masculino	de 26 a 35 anos
	Estágio 2 (perda parcial da espessura						Leve	Masculino	de 66 a 75 anos
	Estágio 2 (perda parcial da espessura						Leve	Masculino	de 26 a 35 anos

	Estágio 2 (perda parcial da espessura						Leve	Masculino	de 46 a 55 anos
	Estágio 2 (perda parcial da espessura						Leve	Masculino	de 46 a 55 anos
							Leve	Masculino	de 46 a 55 anos
	Estágio 2 (perda parcial da espessura						Leve	Feminino	de 56 a 65 anos
	Estágio 2 (perda parcial da espessura						Leve	Masculino	mais de 85 anos
	Estágio 2 (perda parcial da espessura						Leve	Masculino	de 66 a 75 anos
							Leve	Masculino	de 66 a 75 anos
							Leve	Masculino	de 66 a 75 anos
	Estágio 2 (perda parcial da espessura						Leve	Masculino	de 76 a 85 anos
	Estágio 2 (perda parcial da espessura						Leve	Masculino	de 46 a 55 anos
	Estágio 2 (perda parcial da espessura						Leve	Feminino	de 66 a 75 anos
	Estágio 2 (perda parcial da espessura						Leve	Feminino	de 76 a 85 anos
	Estágio 1 (pele íntegra com						Leve	Masculino	de 56 a 65 anos
	Estágio 2 (perda parcial da espessura						Leve	Masculino	de 36 a 45 anos
	Estágio 1 (pele íntegra com						Leve	Feminino	de 56 a 65 anos

	Estágio 1 (pele íntegra com						Leve	Masculino	de 46 a 55 anos
	Estágio 1 (pele íntegra com						Leve	Feminino	de 76 a 85 anos
	Estágio 2 (perda parcial da espessura						Leve	Masculino	de 66 a 75 anos
	Estágio 2 (perda parcial da espessura						Leve	Masculino	de 76 a 85 anos
	Estágio 1 (pele íntegra com						Leve	Feminino	de 76 a 85 anos
	Estágio 2 (perda parcial da espessura						Leve	Feminino	de 56 a 65 anos
	Estágio 1 (pele íntegra com						Leve	Feminino	de 76 a 85 anos
	Estágio 1 (pele íntegra com						Leve	Masculino	de 36 a 45 anos
	Estágio 2 (perda parcial da espessura						Leve	Masculino	de 56 a 65 anos
	Estágio 2 (perda parcial da espessura						Leve	Feminino	de 56 a 65 anos
	Estágio 2 (perda parcial da espessura						Leve	Masculino	de 56 a 65 anos
	Estágio 2 (perda parcial da espessura						Leve	Masculino	de 36 a 45 anos
	Estágio 2 (perda parcial da espessura						Leve	Masculino	de 18 a 25 anos
	Estágio 1 (pele íntegra com						Leve	Feminino	de 36 a 45 anos
	Estágio 1 (pele íntegra com						Leve	Masculino	de 46 a 55 anos

	Estágio 2 (perda parcial da espessura						Leve	Feminino	de 66 a 75 anos
	Estágio 2 (perda parcial da espessura						Leve	Feminino	de 76 a 85 anos
	Estágio 2 (perda parcial da espessura						Leve	Masculino	de 46 a 55 anos
	Estágio 1 (pele íntegra com						Leve	Masculino	de 76 a 85 anos
	Estágio 2 (perda parcial da espessura						Leve	Masculino	de 46 a 55 anos
	Estágio 2 (perda parcial da espessura						Leve	Masculino	de 46 a 55 anos
	Estágio 1 (pele íntegra com						Leve	Masculino	de 26 a 35 anos
	Estágio 2 (perda parcial da espessura						Leve	Masculino	de 76 a 85 anos
	Estágio 1 (pele íntegra com						Leve	Feminino	de 26 a 35 anos
	Estágio 2 (perda parcial da espessura						Leve	Masculino	de 76 a 85 anos
	Estágio 2 (perda parcial da espessura						Leve	Feminino	de 46 a 55 anos
	Estágio 2 (perda parcial da espessura						Leve	Masculino	de 76 a 85 anos
	Estágio 1 (pele íntegra com						Leve	Masculino	de 76 a 85 anos
	Estágio 2 (perda parcial da espessura						Leve	Masculino	de 76 a 85 anos
	Estágio 2 (perda parcial da espessura						Leve	Feminino	de 56 a 65 anos

	Estágio 3 (perda total da espessura						Moderado	Masculino	de 56 a 65 anos
	Estágio 2 (perda parcial da espessura						Leve	Masculino	mais de 85 anos
	Estágio 2 (perda parcial da espessura						Leve	Masculino	de 56 a 65 anos
	Estágio 2 (perda parcial da espessura						Leve	Masculino	de 66 a 75 anos
	Estágio 1 (pele íntegra com						Leve	Masculino	de 36 a 45 anos
	Estágio 1 (pele íntegra com						Leve	Feminino	de 56 a 65 anos
	Estágio 3 (perda total da espessura						Moderado	Masculino	de 36 a 45 anos
	Estágio 2 (perda parcial da espessura						Leve	Feminino	de 56 a 65 anos
	Estágio 1 (pele íntegra com						Leve	Masculino	de 66 a 75 anos
	Estágio 2 (perda parcial da espessura						Leve	Masculino	de 56 a 65 anos
	Estágio 2 (perda parcial da espessura						Leve	Masculino	de 36 a 45 anos
	Estágio 2 (perda parcial da espessura						Leve	Masculino	de 66 a 75 anos
	Estágio 1 (pele íntegra com						Leve	Masculino	de 46 a 55 anos
	Estágio 2 (perda parcial da espessura						Leve	Feminino	de 56 a 65 anos
	Estágio 2 (perda parcial da espessura						Leve	Feminino	de 66 a 75 anos

	Estágio 1 (pele íntegra com						Leve	Feminino	de 18 a 25 anos
	Estágio 2 (perda parcial da espessura						Leve	Feminino	de 66 a 75 anos
							Leve	Feminino	de 66 a 75 anos
	Estágio 2 (perda parcial da espessura						Leve	Feminino	de 76 a 85 anos
	Estágio 2 (perda parcial da espessura						Leve	Feminino	de 46 a 55 anos
	Estágio 2 (perda parcial da espessura						Leve	Feminino	de 76 a 85 anos
	Estágio 1 (pele íntegra com						Leve	Feminino	de 66 a 75 anos
	Estágio 4 (perda total da espessura						Leve	Feminino	de 46 a 55 anos
	Estágio 2 (perda parcial da espessura						Leve	Feminino	de 66 a 75 anos
							Leve	Feminino	de 66 a 75 anos
	Estágio 2 (perda parcial da espessura						Leve	Feminino	de 56 a 65 anos
	Estágio 2 (perda parcial da espessura						Leve	Masculino	de 46 a 55 anos
	Estágio 2 (perda parcial da espessura						Leve	Feminino	de 46 a 55 anos
	Estágio 2 (perda parcial da espessura						Leve	Feminino	mais de 85 anos
	Estágio 2 (perda parcial da espessura						Leve	Feminino	de 66 a 75 anos

	Estágio 2 (perda parcial da espessura						Leve	Feminino	de 66 a 75 anos
	Estágio 2 (perda parcial da espessura						Leve	Masculino	de 46 a 55 anos
	Estágio 2 (perda parcial da espessura						Leve	Masculino	de 56 a 65 anos
	Estágio 2 (perda parcial da espessura						Leve	Feminino	mais de 85 anos
	Estágio 2 (perda parcial da espessura						Leve	Masculino	de 56 a 65 anos
	Estágio 1 (pele íntegra com						Leve	Masculino	de 76 a 85 anos
	Estágio 1 (pele íntegra com						Leve	Feminino	de 76 a 85 anos
	Estágio 2 (perda parcial da espessura						Leve	Feminino	de 56 a 65 anos
	Estágio 2 (perda parcial da espessura						Leve	Feminino	de 56 a 65 anos
	Estágio 1 (pele íntegra com						Leve	Feminino	de 66 a 75 anos
	Estágio 2 (perda parcial da espessura						Leve	Feminino	de 56 a 65 anos
	Estágio 1 (pele íntegra com						Leve	Feminino	de 56 a 65 anos
	Estágio 2 (perda parcial da espessura						Leve	Masculino	de 56 a 65 anos
	Estágio 1 (pele íntegra com						Leve	Masculino	de 56 a 65 anos
	Estágio 2 (perda parcial da espessura						Leve	Feminino	de 36 a 45 anos

	Estágio 1 (pele íntegra com						Leve	Feminino	de 66 a 75 anos
	Estágio 2 (perda parcial da espessura						Leve	Masculino	de 36 a 45 anos
	Estágio 2 (perda parcial da espessura						Leve	Feminino	de 66 a 75 anos
	Estágio 1 (pele íntegra com						Leve	Feminino	de 66 a 75 anos
	Estágio 1 (pele íntegra com						Leve	Masculino	de 46 a 55 anos
	Estágio 2 (perda parcial da espessura						Leve	Masculino	de 26 a 35 anos
	Estágio 1 (pele íntegra com						Leve	Masculino	de 66 a 75 anos
	Estágio 1 (pele íntegra com						Leve	Masculino	de 46 a 55 anos
	Estágio 1 (pele íntegra com						Leve	Masculino	de 26 a 35 anos
	Estágio 2 (perda parcial da espessura						Leve	Masculino	de 56 a 65 anos
	Estágio 2 (perda parcial da espessura						Leve	Masculino	de 76 a 85 anos
	Estágio 2 (perda parcial da espessura						Leve	Masculino	de 56 a 65 anos
	Estágio 1 (pele íntegra com						Leve	Feminino	mais de 85 anos
	Estágio 2 (perda parcial da espessura						Leve	Feminino	de 76 a 85 anos
							Leve	Feminino	de 76 a 85 anos

	Estágio 2 (perda parcial da espessura						Leve	Feminino	de 66 a 75 anos
	Estágio 3 (perda total da espessura						Moderado	Masculino	de 46 a 55 anos
	Estágio 1 (pele íntegra com						Leve	Masculino	de 46 a 55 anos
	Estágio 3 (perda total da espessura						Moderado	Feminino	de 46 a 55 anos
	Estágio 4 (perda total da espessura						Moderado	Masculino	de 76 a 85 anos
							Moderado	Masculino	de 76 a 85 anos
	Estágio 3 (perda total da espessura						Moderado	Feminino	de 46 a 55 anos
	Estágio 3 (perda total da espessura						Moderado	Feminino	de 46 a 55 anos
	Estágio 2 (perda parcial da espessura						Leve	Masculino	de 76 a 85 anos
	Estágio 2 (perda parcial da espessura						Leve	Masculino	de 56 a 65 anos
	Estágio 2 (perda parcial da espessura						Leve	Feminino	de 76 a 85 anos
	Estágio 2 (perda parcial da espessura						Leve	Feminino	de 46 a 55 anos
	Estágio 3 (perda total da espessura						Leve	Feminino	de 36 a 45 anos
	Estágio 2 (perda parcial da espessura						Leve	Masculino	de 36 a 45 anos
	Estágio 2 (perda parcial da espessura						Leve	Masculino	de 76 a 85 anos

	Estágio 2 (perda parcial da espessura						Leve	Feminino	mais de 85 anos
	Estágio 1 (pele íntegra com						Leve	Masculino	de 56 a 65 anos
	Estágio 1 (pele íntegra com						Leve	Feminino	de 46 a 55 anos
	Estágio 2 (perda parcial da espessura						Leve	Masculino	de 66 a 75 anos
	Estágio 2 (perda parcial da espessura						Leve	Feminino	de 46 a 55 anos
	Estágio 2 (perda parcial da espessura						Leve	Feminino	de 46 a 55 anos
	Estágio 3 (perda total da espessura						Moderado	Masculino	de 66 a 75 anos
	Estágio 2 (perda parcial da espessura						Leve	Feminino	de 56 a 65 anos
	Estágio 2 (perda parcial da espessura						Leve	Masculino	de 76 a 85 anos
	Estágio 2 (perda parcial da espessura						Leve	Feminino	de 76 a 85 anos
	Estágio 2 (perda parcial da espessura						Leve	Masculino	de 76 a 85 anos
	Estágio 1 (pele íntegra com						Leve	Feminino	mais de 85 anos
	Estágio 1 (pele íntegra com						Leve	Feminino	de 56 a 65 anos
	Estágio 2 (perda parcial da espessura						Leve	Feminino	de 56 a 65 anos
	Estágio 2 (perda parcial da espessura						Leve	Masculino	mais de 85 anos

	Estágio 2 (perda parcial da espessura						Leve	Masculino	de 56 a 65 anos
	Estágio 2 (perda parcial da espessura						Leve	Masculino	de 76 a 85 anos
	Estágio 2 (perda parcial da espessura						Leve	Feminino	de 56 a 65 anos
	Estágio 1 (pele íntegra com						Leve	Masculino	de 76 a 85 anos
	Estágio 1 (pele íntegra com						Leve	Masculino	de 66 a 75 anos
	Estágio 2 (perda parcial da espessura						Leve	Masculino	de 26 a 35 anos
	Estágio 2 (perda parcial da espessura						Leve	Masculino	de 66 a 75 anos
	Estágio 3 (perda total da espessura						Moderado	Feminino	de 76 a 85 anos
	Estágio 2 (perda parcial da espessura						Leve	Masculino	de 56 a 65 anos
	Estágio 2 (perda parcial da espessura						Leve	Masculino	de 76 a 85 anos
	Estágio 3 (perda total da espessura						Moderado	Masculino	de 66 a 75 anos
	Estágio 2 (perda parcial da espessura						Leve	Feminino	de 56 a 65 anos
	Estágio 1 (pele íntegra com						Leve	Masculino	mais de 85 anos
	Estágio 1 (pele íntegra com						Leve	Masculino	de 56 a 65 anos
	Estágio 2 (perda parcial da espessura						Leve	Masculino	de 76 a 85 anos

	Estágio 2 (perda parcial da espessura						Leve	Masculino	de 66 a 75 anos
	Estágio 1 (pele íntegra com						Leve	Feminino	de 36 a 45 anos
	Estágio 2 (perda parcial da espessura						Leve	Feminino	de 46 a 55 anos
	Estágio 2 (perda parcial da espessura						Leve	Feminino	de 46 a 55 anos
	Estágio 1 (pele íntegra com						Leve	Masculino	de 46 a 55 anos
	Estágio 1 (pele íntegra com						Leve	Feminino	de 56 a 65 anos
	Estágio 2 (perda parcial da espessura						Leve	Feminino	de 76 a 85 anos
	Estágio 2 (perda parcial da espessura						Leve	Feminino	de 76 a 85 anos
	Estágio 2 (perda parcial da espessura						Leve	Masculino	de 66 a 75 anos
	Estágio 3 (perda total da espessura						Moderado	Masculino	de 56 a 65 anos
	Estágio 2 (perda parcial da espessura						Leve	Masculino	de 46 a 55 anos
	Estágio 1 (pele íntegra com						Leve	Masculino	de 66 a 75 anos
	Estágio 1 (pele íntegra com						Leve	Feminino	de 76 a 85 anos
	Estágio 2 (perda parcial da espessura						Leve	Feminino	de 56 a 65 anos
	Estágio 2 (perda parcial da espessura						Leve	Feminino	de 76 a 85 anos

	Estágio 2 (perda parcial da espessura						Leve	Masculino	de 46 a 55 anos
	Estágio 2 (perda parcial da espessura						Leve	Masculino	de 56 a 65 anos
	Estágio 2 (perda parcial da espessura						Leve	Feminino	de 76 a 85 anos
	Estágio 2 (perda parcial da espessura						Leve	Feminino	de 66 a 75 anos
	Estágio 2 (perda parcial da espessura						Leve	Feminino	de 66 a 75 anos
	Estágio 2 (perda parcial da espessura						Leve	Feminino	de 46 a 55 anos
	Estágio 2 (perda parcial da espessura						Leve	Masculino	de 56 a 65 anos
	Estágio 2 (perda parcial da espessura						Leve	Feminino	de 46 a 55 anos
	Estágio 1 (pele íntegra com						Leve	Feminino	de 66 a 75 anos
	Estágio 2 (perda parcial da espessura						Leve	Masculino	de 26 a 35 anos
	Estágio 2 (perda parcial da espessura						Leve	Feminino	de 26 a 35 anos
	Estágio 2 (perda parcial da espessura						Leve	Feminino	mais de 85 anos
	Estágio 1 (pele íntegra com						Leve	Feminino	de 26 a 35 anos
	Estágio 3 (perda total da espessura						Leve	Masculino	de 46 a 55 anos
	Estágio 1 (pele íntegra com						Leve	Masculino	de 76 a 85 anos

	Estágio 2 (perda parcial da espessura						Leve	Masculino	de 46 a 55 anos
	Estágio 2 (perda parcial da espessura						Leve	Masculino	de 76 a 85 anos
	Estágio 1 (pele íntegra com						Leve	Feminino	de 56 a 65 anos
	Estágio 2 (perda parcial da espessura						Leve	Masculino	de 76 a 85 anos
	Estágio 2 (perda parcial da espessura						Leve	Masculino	de 36 a 45 anos
	Estágio 2 (perda parcial da espessura						Leve	Feminino	mais de 85 anos
	Estágio 1 (pele íntegra com						Leve	Masculino	de 56 a 65 anos
	Estágio 2 (perda parcial da espessura						Leve	Feminino	de 66 a 75 anos
	Estágio 1 (pele íntegra com						Leve	Feminino	de 66 a 75 anos
	Estágio 4 (perda total da espessura						Moderado	Masculino	de 46 a 55 anos
	Estágio 3 (perda total da espessura						Moderado	Feminino	de 76 a 85 anos
	Estágio 3 (perda total da espessura						Moderado	Feminino	de 46 a 55 anos
	Estágio 3 (perda total da espessura						Moderado	Masculino	de 66 a 75 anos
	Estágio 1 (pele íntegra com						Leve	Masculino	de 66 a 75 anos
	Estágio 1 (pele íntegra com						Leve	Masculino	de 76 a 85 anos

	Estágio 1 (pele íntegra com						Leve	Feminino	de 18 a 25 anos
	Estágio 2 (perda parcial da espessura						Leve	Masculino	de 56 a 65 anos
	Estágio 2 (perda parcial da espessura						Leve	Masculino	de 66 a 75 anos
	Estágio 3 (perda total da espessura						Moderado	Feminino	de 46 a 55 anos
	Estágio 3 (perda total da espessura						Moderado	Feminino	de 66 a 75 anos
	Estágio 2 (perda parcial da espessura						Leve	Masculino	de 56 a 65 anos
	Estágio 2 (perda parcial da espessura						Leve	Feminino	de 36 a 45 anos
	Estágio 2 (perda parcial da espessura						Leve	Feminino	de 66 a 75 anos
	Estágio 2 (perda parcial da espessura						Leve	Feminino	mais de 85 anos
	Estágio 2 (perda parcial da espessura						Leve	Feminino	de 66 a 75 anos
	Estágio 2 (perda parcial da espessura						Leve	Feminino	de 66 a 75 anos
	Estágio 2 (perda parcial da espessura						Leve	Masculino	mais de 85 anos
	Estágio 2 (perda parcial da espessura						Leve	Masculino	mais de 85 anos
	Estágio 2 (perda parcial da espessura						Leve	Masculino	mais de 85 anos
	Estágio 3 (perda total da espessura						Moderado	Masculino	de 76 a 85 anos

	Estágio 2 (perda parcial da espessura						Leve	Feminino	de 76 a 85 anos
	Estágio 2 (perda parcial da espessura						Leve	Masculino	de 46 a 55 anos
							Leve	Masculino	de 76 a 85 anos
	Estágio 2 (perda parcial da espessura						Leve	Masculino	de 46 a 55 anos
	Estágio 2 (perda parcial da espessura						Leve	Masculino	de 76 a 85 anos
	Estágio 2 (perda parcial da espessura						Leve	Masculino	de 76 a 85 anos
	Estágio 2 (perda parcial da espessura						Leve	Masculino	de 56 a 65 anos
							Leve	Masculino	de 56 a 65 anos
							Leve	Feminino	de 26 a 35 anos
							Nenhum	Masculino	de 18 a 25 anos
							Nenhum	Feminino	de 76 a 85 anos
							Leve	Masculino	de 46 a 55 anos
	Estágio 2 (perda parcial da espessura						Leve	Masculino	de 56 a 65 anos
							Nenhum	Feminino	de 66 a 75 anos
							Leve	Masculino	de 46 a 55 anos

							Leve	Masculino	de 56 a 65 anos
	Estágio 4 (perda total da espessura						Moderado	Masculino	de 56 a 65 anos
	Estágio 3 (perda total da espessura						Moderado	Masculino	de 66 a 75 anos
							Nenhum	Feminino	de 56 a 65 anos
							Leve	Masculino	de 66 a 75 anos
							Nenhum	Feminino	de 56 a 65 anos
							Nenhum	Masculino	de 46 a 55 anos
							Leve	Masculino	de 56 a 65 anos
							Leve	Feminino	de 66 a 75 anos
							Leve	Masculino	de 76 a 85 anos
							Leve	Feminino	de 66 a 75 anos
							Nenhum	Masculino	de 18 a 25 anos
							Nenhum	Masculino	de 56 a 65 anos
							Nenhum	Masculino	de 56 a 65 anos
							Nenhum	Masculino	de 26 a 35 anos

							Leve	Masculino	de 56 a 65 anos
							Moderado	Masculino	de 76 a 85 anos
							Leve	Masculino	de 66 a 75 anos
							Leve	Masculino	de 46 a 55 anos
							Óbito	Feminino	de 36 a 45 anos
							Grave	Feminino	de 56 a 65 anos
							Nenhum	Feminino	de 66 a 75 anos
							Nenhum	Masculino	de 46 a 55 anos
							Nenhum	Masculino	de 46 a 55 anos
							Nenhum	Feminino	de 76 a 85 anos
	Estágio 2 (perda parcial da espessura						Leve	Feminino	de 66 a 75 anos
							Nenhum	Feminino	de 46 a 55 anos
	Estágio 2 (perda parcial da espessura						Leve	Masculino	de 36 a 45 anos
	Estágio 2 (perda parcial da espessura						Leve	Feminino	de 76 a 85 anos
	Estágio 1 (pele íntegra com						Leve	Feminino	de 76 a 85 anos

	Estágio 1 (pele íntegra com						Leve	Feminino	mais de 85 anos
	Estágio 1 (pele íntegra com						Leve	Feminino	de 76 a 85 anos
	Estágio 1 (pele íntegra com						Leve	Feminino	de 76 a 85 anos
							Leve	Feminino	de 76 a 85 anos
	Estágio 1 (pele íntegra com						Leve	Feminino	de 66 a 75 anos
	Estágio 2 (perda parcial da espessura						Leve	Feminino	de 76 a 85 anos
	Estágio 2 (perda parcial da espessura						Leve	Feminino	de 66 a 75 anos
	Estágio 1 (pele íntegra com						Leve	Feminino	mais de 85 anos
	Estágio 2 (perda parcial da espessura						Leve	Masculino	de 66 a 75 anos
	Estágio 2 (perda parcial da espessura						Leve	Masculino	de 56 a 65 anos
	Estágio 2 (perda parcial da espessura						Leve	Feminino	de 76 a 85 anos
	Estágio 1 (pele íntegra com						Leve	Feminino	de 76 a 85 anos
							Leve	Feminino	de 76 a 85 anos
	Estágio 3 (perda total da espessura						Leve	Masculino	de 66 a 75 anos
	Estágio 1 (pele íntegra com						Leve	Masculino	de 46 a 55 anos

	Estágio 2 (perda parcial da espessura						Leve	Feminino	de 66 a 75 anos
							Leve	Feminino	de 66 a 75 anos
	Estágio 2 (perda parcial da espessura						Leve	Masculino	de 56 a 65 anos
	Estágio 2 (perda parcial da espessura						Leve	Feminino	mais de 85 anos
	Estágio 2 (perda parcial da espessura						Leve	Masculino	de 46 a 55 anos
	Estágio 2 (perda parcial da espessura						Leve	Masculino	de 36 a 45 anos
	Estágio 2 (perda parcial da espessura						Leve	Masculino	de 66 a 75 anos
	Estágio 1 (pele íntegra com						Leve	Feminino	de 76 a 85 anos
	Estágio 3 (perda total da espessura						Moderado	Feminino	de 56 a 65 anos
	Estágio 2 (perda parcial da espessura						Leve	Feminino	mais de 85 anos
	Estágio 2 (perda parcial da espessura						Leve	Masculino	de 66 a 75 anos
	Estágio 3 (perda total da espessura						Moderado	Masculino	de 76 a 85 anos
	Estágio 2 (perda parcial da espessura						Leve	Masculino	de 76 a 85 anos
	Estágio 2 (perda parcial da espessura						Leve	Masculino	de 46 a 55 anos
	Estágio 2 (perda parcial da espessura						Leve	Masculino	de 76 a 85 anos

	Estágio 2 (perda parcial da espessura						Leve	Masculino	de 76 a 85 anos
	Estágio 2 (perda parcial da espessura						Leve	Feminino	de 46 a 55 anos
	Estágio 3 (perda total da espessura						Moderado	Masculino	de 36 a 45 anos
	Estágio 2 (perda parcial da espessura						Leve	Feminino	de 66 a 75 anos
	Estágio 2 (perda parcial da espessura						Leve	Feminino	de 36 a 45 anos
	Estágio 2 (perda parcial da espessura						Leve	Feminino	de 66 a 75 anos
	Estágio 1 (pele íntegra com						Leve	Feminino	de 66 a 75 anos
	Estágio 3 (perda total da espessura						Moderado	Masculino	de 76 a 85 anos
	Estágio 2 (perda parcial da espessura						Leve	Masculino	de 76 a 85 anos
	Estágio 2 (perda parcial da espessura						Leve	Masculino	de 76 a 85 anos
	Estágio 2 (perda parcial da espessura						Leve	Feminino	de 76 a 85 anos
	Estágio 2 (perda parcial da espessura						Leve	Masculino	de 76 a 85 anos
	Estágio 2 (perda parcial da espessura						Leve	Masculino	de 76 a 85 anos
	Estágio 1 (pele íntegra com						Leve	Feminino	de 66 a 75 anos
	Estágio 2 (perda parcial da espessura						Leve	Masculino	de 46 a 55 anos

	Estágio 3 (perda total da espessura						Leve	Masculino	de 46 a 55 anos
	Estágio 2 (perda parcial da espessura						Leve	Feminino	de 76 a 85 anos
	Estágio 3 (perda total da espessura						Leve	Masculino	de 76 a 85 anos
	Estágio 2 (perda parcial da espessura						Leve	Feminino	de 36 a 45 anos
	Estágio 3 (perda total da espessura						Moderado	Masculino	de 56 a 65 anos
	Estágio 2 (perda parcial da espessura						Leve	Masculino	de 46 a 55 anos
	Estágio 2 (perda parcial da espessura						Leve	Feminino	de 46 a 55 anos
	Estágio 2 (perda parcial da espessura						Leve	Feminino	de 46 a 55 anos
	Estágio 2 (perda parcial da espessura						Leve	Masculino	de 76 a 85 anos
	Estágio 2 (perda parcial da espessura						Leve	Masculino	de 66 a 75 anos
	Estágio 3 (perda total da espessura						Moderado	Feminino	de 66 a 75 anos
	Estágio 4 (perda total da espessura						Leve	Feminino	de 66 a 75 anos
	Estágio 2 (perda parcial da espessura						Leve	Feminino	de 66 a 75 anos
	Estágio 2 (perda parcial da espessura						Leve	Masculino	de 66 a 75 anos
	Estágio 2 (perda parcial da espessura						Leve	Masculino	de 66 a 75 anos

	Estágio 2 (perda parcial da espessura						Leve	Masculino	de 66 a 75 anos
	Estágio 2 (perda parcial da espessura						Leve	Masculino	de 76 a 85 anos
	Estágio 2 (perda parcial da espessura						Leve	Feminino	de 46 a 55 anos
	Estágio 2 (perda parcial da espessura						Leve	Masculino	de 46 a 55 anos
	Estágio 2 (perda parcial da espessura						Leve	Masculino	de 66 a 75 anos
	Estágio 3 (perda total da espessura						Moderado	Masculino	de 66 a 75 anos
	Estágio 2 (perda parcial da espessura						Leve	Feminino	de 76 a 85 anos
	Estágio 2 (perda parcial da espessura						Leve	Feminino	de 66 a 75 anos
	Estágio 2 (perda parcial da espessura						Leve	Feminino	de 76 a 85 anos
	Estágio 2 (perda parcial da espessura						Leve	Feminino	de 26 a 35 anos
	Estágio 2 (perda parcial da espessura						Leve	Feminino	de 26 a 35 anos
	Estágio 3 (perda total da espessura						Moderado	Feminino	de 36 a 45 anos
	Estágio 2 (perda parcial da espessura						Leve	Feminino	de 26 a 35 anos
	Estágio 2 (perda parcial da espessura						Leve	Feminino	de 56 a 65 anos
	Estágio 3 (perda total da espessura						Moderado	Masculino	de 56 a 65 anos

	Estágio 1 (pele íntegra com						Leve	Masculino	de 36 a 45 anos
	Estágio 1 (pele íntegra com						Leve	Masculino	de 66 a 75 anos
	Estágio 3 (perda total da espessura						Moderado	Feminino	de 36 a 45 anos
							Moderado	Feminino	de 36 a 45 anos
	Estágio 2 (perda parcial da espessura						Leve	Masculino	de 56 a 65 anos
	Estágio 2 (perda parcial da espessura						Leve	Feminino	de 56 a 65 anos
	Estágio 2 (perda parcial da espessura						Leve	Masculino	de 76 a 85 anos
	Estágio 3 (perda total da espessura						Moderado	Feminino	de 26 a 35 anos
	Estágio 1 (pele íntegra com						Leve	Feminino	de 66 a 75 anos
	Estágio 2 (perda parcial da espessura						Leve	Masculino	de 56 a 65 anos
	Estágio 2 (perda parcial da espessura						Leve	Masculino	de 46 a 55 anos
	Estágio 2 (perda parcial da espessura						Leve	Feminino	de 66 a 75 anos
	Estágio 2 (perda parcial da espessura						Leve	Feminino	de 46 a 55 anos
	Estágio 3 (perda total da espessura						Moderado	Feminino	de 36 a 45 anos
	Estágio 2 (perda parcial da espessura						Leve	Masculino	de 66 a 75 anos

	Estágio 1 (pele íntegra com						Leve	Masculino	de 26 a 35 anos
	Estágio 1 (pele íntegra com						Leve	Masculino	de 26 a 35 anos
	Estágio 1 (pele íntegra com						Leve	Masculino	de 26 a 35 anos
	Estágio 1 (pele íntegra com						Leve	Feminino	de 36 a 45 anos
	Estágio 2 (perda parcial da espessura						Leve	Feminino	de 26 a 35 anos
	Estágio 3 (perda total da espessura						Moderado	Masculino	de 46 a 55 anos
	Estágio 3 (perda total da espessura						Leve	Masculino	de 46 a 55 anos
	Estágio 2 (perda parcial da espessura						Leve	Feminino	de 18 a 25 anos
	Estágio 1 (pele íntegra com						Leve	Masculino	de 18 a 25 anos
	Estágio 2 (perda parcial da espessura						Leve	Feminino	de 26 a 35 anos
	Estágio 2 (perda parcial da espessura						Leve	Masculino	de 18 a 25 anos
	Estágio 2 (perda parcial da espessura						Leve	Masculino	de 18 a 25 anos
	Estágio 2 (perda parcial da espessura						Leve	Masculino	de 46 a 55 anos
	Estágio 1 (pele íntegra com						Leve	Feminino	de 76 a 85 anos
	Estágio 2 (perda parcial da espessura						Leve	Feminino	de 66 a 75 anos

	Estágio 2 (perda parcial da espessura						Leve	Feminino	de 66 a 75 anos
	Estágio 1 (pele íntegra com						Leve	Masculino	de 56 a 65 anos
	Estágio 3 (perda total da espessura						Moderado	Feminino	de 36 a 45 anos
	Estágio 2 (perda parcial da espessura						Leve	Feminino	de 76 a 85 anos
	Estágio 3 (perda total da espessura						Moderado	Feminino	de 56 a 65 anos
	Estágio 3 (perda total da espessura						Leve	Feminino	de 56 a 65 anos
							Leve	Feminino	de 56 a 65 anos
	Estágio 2 (perda parcial da espessura						Leve	Feminino	de 56 a 65 anos
	Estágio 2 (perda parcial da espessura						Leve	Feminino	de 46 a 55 anos
	Estágio 2 (perda parcial da espessura						Leve	Feminino	de 66 a 75 anos
	Estágio 2 (perda parcial da espessura						Leve	Feminino	de 36 a 45 anos
	Estágio 2 (perda parcial da espessura						Leve	Feminino	de 36 a 45 anos
	Estágio 2 (perda parcial da espessura						Leve	Masculino	de 66 a 75 anos
	Estágio 4 (perda total da espessura						Moderado	Feminino	de 36 a 45 anos
	Estágio 2 (perda parcial da espessura						Leve	Feminino	de 36 a 45 anos

	Estágio 4 (perda total da espessura						Moderado	Feminino	de 36 a 45 anos
	Estágio 1 (pele íntegra com						Leve	Masculino	de 66 a 75 anos
	Estágio 2 (perda parcial da espessura						Leve	Feminino	de 46 a 55 anos
	Estágio 4 (perda total da espessura						Moderado	Feminino	de 36 a 45 anos
	Estágio 2 (perda parcial da espessura						Leve	Masculino	de 76 a 85 anos
	Estágio 3 (perda total da espessura						Leve	Feminino	de 56 a 65 anos
	Estágio 2 (perda parcial da espessura						Leve	Feminino	de 66 a 75 anos
							Leve	Feminino	de 66 a 75 anos
	Estágio 1 (pele íntegra com						Leve	Feminino	de 66 a 75 anos
	Estágio 1 (pele íntegra com						Leve	Masculino	de 46 a 55 anos
	Estágio 2 (perda parcial da espessura						Leve	Feminino	de 76 a 85 anos
	Estágio 1 (pele íntegra com						Leve	Masculino	de 46 a 55 anos
	Estágio 1 (pele íntegra com						Leve	Feminino	de 18 a 25 anos
	Estágio 2 (perda parcial da espessura						Leve	Feminino	de 56 a 65 anos
	Estágio 2 (perda parcial da espessura						Leve	Feminino	de 56 a 65 anos

	Estágio 2 (perda parcial da espessura						Leve	Feminino	de 46 a 55 anos
	Estágio 2 (perda parcial da espessura						Leve	Masculino	de 26 a 35 anos
	Estágio 2 (perda parcial da espessura						Leve	Feminino	de 56 a 65 anos
	Estágio 2 (perda parcial da espessura						Leve	Feminino	de 46 a 55 anos
	Estágio 3 (perda total da espessura						Moderado	Feminino	de 46 a 55 anos
	Estágio 2 (perda parcial da espessura						Leve	Masculino	de 76 a 85 anos
	Estágio 2 (perda parcial da espessura						Leve	Masculino	de 76 a 85 anos
	Estágio 2 (perda parcial da espessura						Leve	Feminino	de 46 a 55 anos
	Estágio 2 (perda parcial da espessura						Leve	Masculino	mais de 85 anos
	Estágio 2 (perda parcial da espessura						Leve	Masculino	de 76 a 85 anos
	Estágio 2 (perda parcial da espessura						Leve	Feminino	de 26 a 35 anos
	Estágio 2 (perda parcial da espessura						Leve	Masculino	de 46 a 55 anos
	Estágio 2 (perda parcial da espessura						Leve	Masculino	de 46 a 55 anos
	Estágio 2 (perda parcial da espessura						Leve	Feminino	de 76 a 85 anos
	Estágio 2 (perda parcial da espessura						Leve	Masculino	de 66 a 75 anos

	Estágio 2 (perda parcial da espessura						Leve	Masculino	de 66 a 75 anos
	Estágio 1 (pele íntegra com						Leve	Feminino	de 36 a 45 anos
	Estágio 2 (perda parcial da espessura						Leve	Masculino	de 56 a 65 anos
							Leve	Feminino	de 56 a 65 anos
	Estágio 1 (pele íntegra com						Leve	Masculino	de 56 a 65 anos
							Leve	Masculino	de 56 a 65 anos
							Leve	Masculino	de 66 a 75 anos
							Nenhum	Feminino	de 56 a 65 anos
							Leve	Masculino	de 66 a 75 anos
							Leve	Feminino	de 66 a 75 anos
							Leve	Masculino	de 66 a 75 anos
							Leve	Feminino	de 26 a 35 anos
							Leve	Feminino	de 76 a 85 anos
							Leve	Feminino	de 66 a 75 anos
							Leve	Feminino	de 76 a 85 anos

							Leve	Masculino	de 76 a 85 anos
							Leve	Masculino	de 56 a 65 anos
							Leve	Feminino	de 76 a 85 anos
	Estágio 1 (pele íntegra com						Leve	Feminino	de 76 a 85 anos
	Estágio 2 (perda parcial da espessura						Leve	Feminino	de 36 a 45 anos
	Estágio 2 (perda parcial da espessura						Leve	Feminino	de 56 a 65 anos
	Estágio 2 (perda parcial da espessura						Leve	Masculino	de 46 a 55 anos
	Estágio 2 (perda parcial da espessura						Leve	Feminino	de 56 a 65 anos
	Estágio 2 (perda parcial da espessura						Leve	Masculino	de 46 a 55 anos
	Estágio 1 (pele íntegra com						Leve	Masculino	de 46 a 55 anos
	Estágio 2 (perda parcial da espessura						Leve	Masculino	de 46 a 55 anos
	Estágio 3 (perda total da espessura						Moderado	Masculino	de 66 a 75 anos
	Estágio 2 (perda parcial da espessura						Leve	Feminino	de 66 a 75 anos
	Estágio 3 (perda total da espessura						Leve	Feminino	de 46 a 55 anos
	Estágio 1 (pele íntegra com						Leve	Masculino	mais de 85 anos

	Estágio 2 (perda parcial da espessura						Leve	Feminino	de 76 a 85 anos
	Estágio 2 (perda parcial da espessura						Leve	Feminino	de 66 a 75 anos
	Estágio 2 (perda parcial da espessura						Leve	Feminino	de 66 a 75 anos
	Estágio 2 (perda parcial da espessura						Leve	Masculino	de 76 a 85 anos
	Estágio 3 (perda total da espessura						Leve	Feminino	de 26 a 35 anos
	Estágio 2 (perda parcial da espessura						Leve	Feminino	mais de 85 anos
	Estágio 1 (pele íntegra com						Leve	Feminino	de 46 a 55 anos
	Estágio 2 (perda parcial da espessura						Leve	Masculino	de 56 a 65 anos
	Estágio 2 (perda parcial da espessura						Leve	Feminino	de 66 a 75 anos
	Estágio 2 (perda parcial da espessura						Leve	Masculino	de 56 a 65 anos
	Estágio 3 (perda total da espessura						Moderado	Masculino	de 56 a 65 anos
	Estágio 3 (perda total da espessura						Moderado	Masculino	de 56 a 65 anos
	Estágio 2 (perda parcial da espessura						Leve	Feminino	de 76 a 85 anos
	Estágio 2 (perda parcial da espessura						Leve	Feminino	de 76 a 85 anos
	Estágio 2 (perda parcial da espessura						Leve	Feminino	de 76 a 85 anos

	Estágio 1 (pele íntegra com						Leve	Masculino	de 56 a 65 anos
	Estágio 2 (perda parcial da espessura						Leve	Masculino	de 76 a 85 anos
	Estágio 3 (perda total da espessura						Moderado	Feminino	de 46 a 55 anos
	Estágio 2 (perda parcial da espessura						Leve	Masculino	de 46 a 55 anos
	Estágio 2 (perda parcial da espessura						Leve	Masculino	de 46 a 55 anos
	Estágio 2 (perda parcial da espessura						Leve	Masculino	de 66 a 75 anos
	Estágio 2 (perda parcial da espessura						Leve	Feminino	de 56 a 65 anos
	Estágio 2 (perda parcial da espessura						Leve	Masculino	de 76 a 85 anos
	Estágio 2 (perda parcial da espessura						Leve	Feminino	de 66 a 75 anos
	Estágio 2 (perda parcial da espessura						Leve	Feminino	de 66 a 75 anos
	Estágio 3 (perda total da espessura						Leve	Feminino	de 66 a 75 anos
							Leve	Feminino	de 66 a 75 anos
	Estágio 4 (perda total da espessura						Moderado	Masculino	de 18 a 25 anos
	Estágio 2 (perda parcial da espessura						Leve	Masculino	de 18 a 25 anos
	Estágio 2 (perda parcial da espessura						Leve	Masculino	de 18 a 25 anos

	Estágio 2 (perda parcial da espessura						Leve	Masculino	de 18 a 25 anos
	Estágio 2 (perda parcial da espessura						Leve	Feminino	mais de 85 anos
	Estágio 1 (pele íntegra com						Leve	Feminino	de 66 a 75 anos
	Estágio 2 (perda parcial da espessura						Leve	Feminino	de 56 a 65 anos
	Estágio 3 (perda total da espessura						Leve	Masculino	de 76 a 85 anos
	Estágio 2 (perda parcial da espessura						Leve	Masculino	de 66 a 75 anos
	Estágio 3 (perda total da espessura						Moderado	Feminino	de 56 a 65 anos
	Estágio 2 (perda parcial da espessura						Leve	Feminino	de 76 a 85 anos
	Estágio 2 (perda parcial da espessura						Leve	Masculino	de 76 a 85 anos
	Estágio 2 (perda parcial da espessura						Leve	Masculino	de 76 a 85 anos
	Estágio 1 (pele íntegra com						Leve	Masculino	de 46 a 55 anos
	Estágio 2 (perda parcial da espessura						Leve	Feminino	de 76 a 85 anos
	Estágio 2 (perda parcial da espessura						Leve	Masculino	de 56 a 65 anos
	Estágio 1 (pele íntegra com						Leve	Masculino	de 46 a 55 anos
	Estágio 3 (perda total da espessura						Moderado	Masculino	de 46 a 55 anos

	Estágio 2 (perda parcial da espessura						Leve	Masculino	mais de 85 anos
	Estágio 2 (perda parcial da espessura						Leve	Masculino	de 46 a 55 anos
	Estágio 2 (perda parcial da espessura						Leve	Masculino	de 46 a 55 anos
	Estágio 2 (perda parcial da espessura						Leve	Masculino	de 56 a 65 anos
	Estágio 3 (perda total da espessura						Moderado	Masculino	de 56 a 65 anos
	Estágio 2 (perda parcial da espessura						Leve	Masculino	de 66 a 75 anos
	Estágio 3 (perda total da espessura						Moderado	Masculino	de 36 a 45 anos
	Estágio 1 (pele íntegra com						Leve	Feminino	mais de 85 anos
	Estágio 2 (perda parcial da espessura						Leve	Feminino	de 56 a 65 anos
	Estágio 2 (perda parcial da espessura						Leve	Feminino	de 66 a 75 anos
	Estágio 2 (perda parcial da espessura						Leve	Feminino	de 66 a 75 anos
	Estágio 1 (pele íntegra com						Leve	Feminino	mais de 85 anos
	Estágio 2 (perda parcial da espessura						Leve	Feminino	de 46 a 55 anos
	Estágio 2 (perda parcial da espessura						Leve	Masculino	de 18 a 25 anos
	Estágio 2 (perda parcial da espessura						Leve	Masculino	de 26 a 35 anos

	Estágio 3 (perda total da espessura						Leve	Feminino	mais de 85 anos
	Estágio 1 (pele íntegra com						Leve	Feminino	de 66 a 75 anos
	Estágio 1 (pele íntegra com						Leve	Masculino	de 36 a 45 anos
	Estágio 2 (perda parcial da espessura						Leve	Masculino	de 56 a 65 anos
	Estágio 1 (pele íntegra com						Leve	Feminino	de 66 a 75 anos
	Estágio 2 (perda parcial da espessura						Leve	Masculino	de 56 a 65 anos
	Estágio 2 (perda parcial da espessura						Leve	Masculino	de 56 a 65 anos
	Estágio 2 (perda parcial da espessura						Leve	Feminino	de 76 a 85 anos
	Estágio 2 (perda parcial da espessura						Leve	Feminino	de 56 a 65 anos
	Estágio 2 (perda parcial da espessura						Leve	Feminino	de 56 a 65 anos
	Estágio 3 (perda total da espessura						Moderado	Feminino	de 56 a 65 anos
	Estágio 2 (perda parcial da espessura						Leve	Masculino	de 46 a 55 anos
	Estágio 2 (perda parcial da espessura						Leve	Masculino	de 46 a 55 anos
	Estágio 2 (perda parcial da espessura						Leve	Feminino	de 56 a 65 anos
	Estágio 1 (pele íntegra com						Leve	Masculino	de 76 a 85 anos

	Estágio 1 (pele íntegra com						Leve	Feminino	de 56 a 65 anos
	Estágio 2 (perda parcial da espessura						Leve	Feminino	de 66 a 75 anos
	Estágio 2 (perda parcial da espessura						Leve	Masculino	de 76 a 85 anos
	Estágio 2 (perda parcial da espessura						Leve	Masculino	de 66 a 75 anos
	Estágio 3 (perda total da espessura						Moderado	Masculino	de 46 a 55 anos
	Estágio 2 (perda parcial da espessura						Leve	Masculino	de 46 a 55 anos
	Estágio 2 (perda parcial da espessura						Leve	Feminino	de 18 a 25 anos
	Estágio 2 (perda parcial da espessura						Leve	Feminino	de 36 a 45 anos
	Estágio 2 (perda parcial da espessura						Leve	Masculino	de 46 a 55 anos
	Estágio 1 (pele íntegra com						Leve	Feminino	de 36 a 45 anos
	Estágio 2 (perda parcial da espessura						Leve	Feminino	de 46 a 55 anos
	Estágio 2 (perda parcial da espessura						Leve	Masculino	de 56 a 65 anos
	Estágio 2 (perda parcial da espessura						Leve	Feminino	de 66 a 75 anos
	Estágio 1 (pele íntegra com						Leve	Feminino	de 66 a 75 anos
	Estágio 2 (perda parcial da espessura						Leve	Masculino	de 46 a 55 anos

	Estágio 2 (perda parcial da espessura						Leve	Feminino	de 56 a 65 anos
	Estágio 2 (perda parcial da espessura						Leve	Feminino	de 36 a 45 anos
	Estágio 2 (perda parcial da espessura						Leve	Feminino	de 56 a 65 anos
	Estágio 2 (perda parcial da espessura						Leve	Masculino	de 46 a 55 anos
	Estágio 2 (perda parcial da espessura						Leve	Masculino	de 46 a 55 anos
	Estágio 2 (perda parcial da espessura						Leve	Masculino	de 66 a 75 anos
	Estágio 2 (perda parcial da espessura						Leve	Masculino	de 66 a 75 anos
	Estágio 2 (perda parcial da espessura						Leve	Masculino	de 46 a 55 anos
	Estágio 2 (perda parcial da espessura						Leve	Feminino	de 26 a 35 anos
							Leve	Feminino	de 26 a 35 anos
	Estágio 2 (perda parcial da espessura						Leve	Masculino	de 56 a 65 anos
	Estágio 2 (perda parcial da espessura						Leve	Masculino	de 56 a 65 anos
							Leve	Masculino	de 56 a 65 anos
	Estágio 2 (perda parcial da espessura						Leve	Feminino	de 66 a 75 anos
	Estágio 2 (perda parcial da espessura						Leve	Feminino	de 26 a 35 anos

	Estágio 3 (perda total da espessura						Moderado	Feminino	de 56 a 65 anos
	Estágio 3 (perda total da espessura						Moderado	Masculino	de 26 a 35 anos
	Estágio 2 (perda parcial da espessura						Leve	Feminino	de 66 a 75 anos
	Estágio 2 (perda parcial da espessura						Leve	Feminino	mais de 85 anos
	Estágio 3 (perda total da espessura						Leve	Masculino	de 66 a 75 anos
							Leve	Masculino	de 66 a 75 anos
	Estágio 2 (perda parcial da espessura						Leve	Feminino	de 76 a 85 anos
	Estágio 2 (perda parcial da espessura						Leve	Feminino	de 66 a 75 anos
	Estágio 2 (perda parcial da espessura						Leve	Feminino	de 66 a 75 anos
	Estágio 2 (perda parcial da espessura						Leve	Masculino	de 46 a 55 anos
	Estágio 2 (perda parcial da espessura						Leve	Feminino	mais de 85 anos
	Estágio 2 (perda parcial da espessura						Leve	Feminino	de 56 a 65 anos
	Estágio 2 (perda parcial da espessura						Leve	Masculino	de 76 a 85 anos
	Estágio 1 (pele íntegra com						Leve	Feminino	de 66 a 75 anos
	Estágio 2 (perda parcial da espessura						Leve	Masculino	de 66 a 75 anos

	Estágio 2 (perda parcial da espessura						Leve	Masculino	de 46 a 55 anos
	Estágio 2 (perda parcial da espessura						Leve	Masculino	de 66 a 75 anos
	Estágio 2 (perda parcial da espessura						Leve	Masculino	de 66 a 75 anos
	Estágio 2 (perda parcial da espessura						Leve	Masculino	de 66 a 75 anos
	Estágio 2 (perda parcial da espessura						Leve	Masculino	de 46 a 55 anos
	Estágio 2 (perda parcial da espessura						Leve	Masculino	de 46 a 55 anos
	Estágio 2 (perda parcial da espessura						Leve	Feminino	de 66 a 75 anos
	Estágio 1 (pele íntegra com						Leve	Feminino	de 18 a 25 anos
	Estágio 1 (pele íntegra com						Leve	Masculino	de 36 a 45 anos
	Estágio 1 (pele íntegra com						Leve	Masculino	de 46 a 55 anos
	Estágio 1 (pele íntegra com						Leve	Masculino	de 36 a 45 anos
	Estágio 3 (perda total da espessura						Leve	Masculino	de 36 a 45 anos
	Estágio 3 (perda total da espessura						Leve	Masculino	de 36 a 45 anos
	Estágio 2 (perda parcial da espessura						Leve	Masculino	de 66 a 75 anos
	Estágio 1 (pele íntegra com						Leve	Masculino	de 76 a 85 anos

	Estágio 2 (perda parcial da espessura						Leve	Masculino	de 46 a 55 anos
	Estágio 2 (perda parcial da espessura						Leve	Masculino	de 66 a 75 anos
	Estágio 2 (perda parcial da espessura						Leve	Feminino	de 56 a 65 anos
	Estágio 2 (perda parcial da espessura						Leve	Feminino	de 66 a 75 anos
							Leve	Feminino	de 66 a 75 anos
	Estágio 1 (pele íntegra com						Leve	Masculino	de 76 a 85 anos
	Estágio 2 (perda parcial da espessura						Leve	Masculino	de 56 a 65 anos
	Estágio 1 (pele íntegra com						Leve	Masculino	de 46 a 55 anos
	Estágio 2 (perda parcial da espessura						Leve	Feminino	de 56 a 65 anos
	Estágio 2 (perda parcial da espessura						Leve	Masculino	de 26 a 35 anos
	Estágio 2 (perda parcial da espessura						Leve	Masculino	de 26 a 35 anos
	Estágio 2 (perda parcial da espessura						Leve	Feminino	de 66 a 75 anos
	Estágio 2 (perda parcial da espessura						Leve	Feminino	de 26 a 35 anos
	Estágio 2 (perda parcial da espessura						Leve	Masculino	de 26 a 35 anos
	Estágio 2 (perda parcial da espessura						Leve	Feminino	de 26 a 35 anos

	Estágio 2 (perda parcial da espessura						Leve	Feminino	de 76 a 85 anos
	Estágio 2 (perda parcial da espessura						Leve	Masculino	de 46 a 55 anos
	Estágio 2 (perda parcial da espessura						Leve	Feminino	de 46 a 55 anos
	Estágio 2 (perda parcial da espessura						Leve	Feminino	de 66 a 75 anos
	Estágio 4 (perda total da espessura						Moderado	Feminino	de 26 a 35 anos
	Estágio 1 (pele íntegra com						Leve	Feminino	de 26 a 35 anos
	Estágio 2 (perda parcial da espessura						Leve	Masculino	de 66 a 75 anos
	Estágio 2 (perda parcial da espessura						Leve	Masculino	de 36 a 45 anos
	Estágio 2 (perda parcial da espessura						Leve	Masculino	de 76 a 85 anos
	Estágio 1 (pele íntegra com						Leve	Masculino	de 66 a 75 anos
	Estágio 2 (perda parcial da espessura						Leve	Masculino	mais de 85 anos
	Estágio 1 (pele íntegra com						Leve	Masculino	de 66 a 75 anos
	Estágio 1 (pele íntegra com						Leve	Feminino	de 36 a 45 anos
	Estágio 1 (pele íntegra com						Leve	Masculino	de 46 a 55 anos
	Estágio 2 (perda parcial da espessura						Leve	Feminino	de 56 a 65 anos

	Estágio 1 (pele íntegra com						Leve	Feminino	de 66 a 75 anos
	Estágio 2 (perda parcial da espessura						Leve	Masculino	de 76 a 85 anos
	Estágio 2 (perda parcial da espessura						Leve	Masculino	de 76 a 85 anos
	Estágio 1 (pele íntegra com						Leve	Feminino	de 36 a 45 anos
	Estágio 1 (pele íntegra com						Leve	Feminino	de 36 a 45 anos
	Estágio 2 (perda parcial da espessura						Leve	Masculino	de 76 a 85 anos
	Estágio 1 (pele íntegra com						Leve	Feminino	de 56 a 65 anos
	Estágio 2 (perda parcial da espessura						Leve	Feminino	de 26 a 35 anos
	Estágio 1 (pele íntegra com						Leve	Feminino	de 36 a 45 anos
	Estágio 2 (perda parcial da espessura						Leve	Masculino	de 66 a 75 anos
	Estágio 2 (perda parcial da espessura						Leve	Masculino	de 76 a 85 anos
	Estágio 2 (perda parcial da espessura						Leve	Masculino	de 66 a 75 anos
							Leve	Masculino	de 66 a 75 anos
	Estágio 2 (perda parcial da espessura						Leve	Feminino	de 76 a 85 anos
	Estágio 2 (perda parcial da espessura						Leve	Masculino	de 76 a 85 anos

	Estágio 2 (perda parcial da espessura						Leve	Feminino	de 56 a 65 anos
	Estágio 2 (perda parcial da espessura						Leve	Feminino	de 76 a 85 anos
	Estágio 2 (perda parcial da espessura						Leve	Feminino	de 46 a 55 anos
	Estágio 3 (perda total da espessura						Moderado	Feminino	de 46 a 55 anos
	Estágio 4 (perda total da espessura						Leve	Feminino	de 46 a 55 anos
	Estágio 1 (pele íntegra com						Leve	Masculino	de 56 a 65 anos
	Estágio 3 (perda total da espessura						Moderado	Feminino	de 36 a 45 anos
	Estágio 4 (perda total da espessura						Moderado	Feminino	de 76 a 85 anos
	Estágio 2 (perda parcial da espessura						Leve	Feminino	de 56 a 65 anos
	Estágio 1 (pele íntegra com						Leve	Masculino	de 56 a 65 anos
	Estágio 1 (pele íntegra com						Leve	Feminino	de 76 a 85 anos
	Estágio 2 (perda parcial da espessura						Leve	Feminino	de 66 a 75 anos
	Estágio 2 (perda parcial da espessura						Leve	Feminino	de 56 a 65 anos
	Estágio 2 (perda parcial da espessura						Leve	Feminino	de 76 a 85 anos
	Estágio 1 (pele íntegra com						Leve	Masculino	de 66 a 75 anos

	Estágio 4 (perda total da espessura						Moderado	Feminino	de 56 a 65 anos
	Estágio 2 (perda parcial da espessura						Leve	Feminino	de 56 a 65 anos
	Estágio 2 (perda parcial da espessura						Leve	Masculino	de 56 a 65 anos
	Estágio 3 (perda total da espessura						Moderado	Masculino	de 76 a 85 anos
							Moderado	Masculino	de 76 a 85 anos
	Estágio 2 (perda parcial da espessura						Leve	Masculino	de 76 a 85 anos
	Estágio 2 (perda parcial da espessura						Leve	Feminino	de 56 a 65 anos
	Estágio 2 (perda parcial da espessura						Leve	Masculino	de 46 a 55 anos
	Estágio 1 (pele íntegra com						Leve	Masculino	de 46 a 55 anos
							Leve	Masculino	de 46 a 55 anos
	Estágio 1 (pele íntegra com						Leve	Masculino	de 66 a 75 anos
	Estágio 2 (perda parcial da espessura						Leve	Masculino	de 46 a 55 anos
	Estágio 2 (perda parcial da espessura						Leve	Masculino	de 66 a 75 anos
	Estágio 4 (perda total da espessura						Moderado	Masculino	de 36 a 45 anos
	Estágio 3 (perda total da espessura						Leve	Masculino	de 76 a 85 anos

	Estágio 3 (perda total da espessura						Leve	Masculino	de 56 a 65 anos
	Estágio 2 (perda parcial da espessura						Leve	Masculino	de 76 a 85 anos
	Estágio 1 (pele íntegra com						Leve	Masculino	de 66 a 75 anos
	Estágio 2 (perda parcial da espessura						Leve	Masculino	de 66 a 75 anos
	Estágio 2 (perda parcial da espessura						Leve	Masculino	de 66 a 75 anos
	Estágio 2 (perda parcial da espessura						Leve	Feminino	de 36 a 45 anos
	Estágio 2 (perda parcial da espessura						Leve	Masculino	de 66 a 75 anos
	Estágio 2 (perda parcial da espessura						Leve	Masculino	de 26 a 35 anos
	Estágio 2 (perda parcial da espessura						Leve	Feminino	de 66 a 75 anos
	Estágio 1 (pele íntegra com						Leve	Masculino	de 46 a 55 anos
							Leve	Masculino	de 46 a 55 anos
	Estágio 1 (pele íntegra com						Leve	Feminino	de 56 a 65 anos
							Leve	Feminino	de 56 a 65 anos
	Estágio 1 (pele íntegra com						Leve	Masculino	de 76 a 85 anos
							Leve	Masculino	de 76 a 85 anos

	Estágio 2 (perda parcial da espessura						Leve	Feminino	de 76 a 85 anos
	Estágio 1 (pele íntegra com						Leve	Masculino	de 46 a 55 anos
	Estágio 2 (perda parcial da espessura						Leve	Feminino	de 76 a 85 anos
	Estágio 2 (perda parcial da espessura						Leve	Masculino	de 46 a 55 anos
	Estágio 2 (perda parcial da espessura						Leve	Feminino	de 46 a 55 anos
	Estágio 2 (perda parcial da espessura						Leve	Feminino	de 26 a 35 anos
	Estágio 2 (perda parcial da espessura						Leve	Masculino	de 26 a 35 anos
							Leve	Feminino	de 76 a 85 anos
							Leve	Masculino	de 66 a 75 anos
							Leve	Masculino	de 56 a 65 anos
							Leve	Feminino	de 26 a 35 anos
	Estágio 3 (perda total da espessura						Moderado	Feminino	de 46 a 55 anos
	Estágio 2 (perda parcial da espessura						Leve	Feminino	de 66 a 75 anos
	Estágio 2 (perda parcial da espessura						Leve	Masculino	mais de 85 anos
	Estágio 2 (perda parcial da espessura						Leve	Masculino	de 66 a 75 anos

	Estágio 2 (perda parcial da espessura						Leve	Masculino	de 66 a 75 anos
	Estágio 1 (pele íntegra com						Leve	Feminino	de 66 a 75 anos
	Estágio 2 (perda parcial da espessura						Leve	Feminino	de 46 a 55 anos
	Estágio 2 (perda parcial da espessura						Leve	Masculino	de 76 a 85 anos
	Estágio 3 (perda total da espessura						Moderado	Masculino	de 36 a 45 anos
	Estágio 1 (pele íntegra com						Leve	Feminino	de 56 a 65 anos
	Estágio 1 (pele íntegra com						Leve	Feminino	de 66 a 75 anos
	Estágio 2 (perda parcial da espessura						Leve	Feminino	de 66 a 75 anos
	Estágio 2 (perda parcial da espessura						Leve	Feminino	de 66 a 75 anos
	Estágio 2 (perda parcial da espessura						Leve	Feminino	de 56 a 65 anos
	Estágio 3 (perda total da espessura						Moderado	Masculino	de 46 a 55 anos
	Estágio 2 (perda parcial da espessura						Leve	Masculino	de 46 a 55 anos
	Estágio 2 (perda parcial da espessura						Leve	Feminino	de 56 a 65 anos
	Estágio 1 (pele íntegra com						Leve	Masculino	de 46 a 55 anos
	Estágio 2 (perda parcial da espessura						Leve	Feminino	de 56 a 65 anos

	Estágio 2 (perda parcial da espessura						Leve	Feminino	de 76 a 85 anos
	Estágio 2 (perda parcial da espessura						Leve	Feminino	de 56 a 65 anos
	Estágio 2 (perda parcial da espessura						Leve	Masculino	de 46 a 55 anos
	Estágio 3 (perda total da espessura						Moderado	Feminino	de 56 a 65 anos
	Estágio 2 (perda parcial da espessura						Leve	Feminino	de 56 a 65 anos
	Estágio 3 (perda total da espessura						Moderado	Feminino	de 56 a 65 anos
	Estágio 2 (perda parcial da espessura						Leve	Feminino	de 66 a 75 anos
	Estágio 1 (pele íntegra com						Leve	Feminino	de 46 a 55 anos
	Estágio 1 (pele íntegra com						Leve	Feminino	de 76 a 85 anos
	Estágio 2 (perda parcial da espessura						Leve	Masculino	de 46 a 55 anos
	Estágio 1 (pele íntegra com						Leve	Masculino	de 76 a 85 anos
	Estágio 3 (perda total da espessura						Moderado	Feminino	de 66 a 75 anos
	Estágio 2 (perda parcial da espessura						Leve	Feminino	de 66 a 75 anos
	Estágio 3 (perda total da espessura						Moderado	Feminino	de 46 a 55 anos
	Estágio 2 (perda parcial da espessura						Leve	Feminino	de 56 a 65 anos

	Estágio 2 (perda parcial da espessura						Leve	Feminino	de 46 a 55 anos
	Estágio 2 (perda parcial da espessura						Leve	Feminino	de 66 a 75 anos
	Estágio 2 (perda parcial da espessura						Leve	Feminino	de 56 a 65 anos
	Estágio 1 (pele íntegra com						Leve	Feminino	de 56 a 65 anos
	Estágio 3 (perda total da espessura						Moderado	Masculino	mais de 85 anos
	Estágio 2 (perda parcial da espessura						Leve	Masculino	de 46 a 55 anos
	Estágio 2 (perda parcial da espessura						Leve	Masculino	de 26 a 35 anos
	Estágio 2 (perda parcial da espessura						Leve	Masculino	de 26 a 35 anos
	Estágio 1 (pele íntegra com						Leve	Feminino	de 66 a 75 anos
	Estágio 1 (pele íntegra com						Leve	Feminino	de 36 a 45 anos
	Estágio 2 (perda parcial da espessura						Leve	Masculino	de 76 a 85 anos
	Estágio 2 (perda parcial da espessura						Leve	Feminino	de 56 a 65 anos
	Estágio 2 (perda parcial da espessura						Leve	Masculino	de 76 a 85 anos
	Estágio 2 (perda parcial da espessura						Leve	Masculino	de 76 a 85 anos
	Estágio 1 (pele íntegra com						Leve	Masculino	de 46 a 55 anos

	Estágio 1 (pele íntegra com						Leve	Masculino	de 46 a 55 anos
	Estágio 2 (perda parcial da espessura						Leve	Feminino	de 46 a 55 anos
	Estágio 2 (perda parcial da espessura						Leve	Masculino	de 56 a 65 anos
	Estágio 2 (perda parcial da espessura						Leve	Masculino	de 56 a 65 anos
	Estágio 2 (perda parcial da espessura						Leve	Masculino	de 46 a 55 anos
	Estágio 2 (perda parcial da espessura						Leve	Feminino	de 66 a 75 anos
	Estágio 2 (perda parcial da espessura						Leve	Masculino	de 46 a 55 anos
	Estágio 1 (pele íntegra com						Leve	Feminino	de 66 a 75 anos
	Estágio 2 (perda parcial da espessura						Leve	Feminino	de 56 a 65 anos
	Estágio 2 (perda parcial da espessura						Leve	Feminino	mais de 85 anos
	Estágio 2 (perda parcial da espessura						Leve	Masculino	de 46 a 55 anos
	Estágio 2 (perda parcial da espessura						Leve	Feminino	de 76 a 85 anos
	Estágio 2 (perda parcial da espessura						Leve	Feminino	de 66 a 75 anos
	Estágio 3 (perda total da espessura						Moderado	Masculino	mais de 85 anos
	Estágio 3 (perda total da espessura						Moderado	Masculino	mais de 85 anos

	Estágio 2 (perda parcial da espessura						Leve	Feminino	de 26 a 35 anos
	Estágio 1 (pele íntegra com						Leve	Masculino	de 36 a 45 anos
	Estágio 2 (perda parcial da espessura						Leve	Masculino	de 46 a 55 anos
	Estágio 2 (perda parcial da espessura						Leve	Feminino	de 76 a 85 anos
	Estágio 2 (perda parcial da espessura						Leve	Masculino	de 36 a 45 anos
	Estágio 2 (perda parcial da espessura						Leve	Feminino	de 76 a 85 anos
	Estágio 2 (perda parcial da espessura						Leve	Feminino	de 66 a 75 anos
	Estágio 2 (perda parcial da espessura						Leve	Masculino	de 66 a 75 anos
	Estágio 2 (perda parcial da espessura						Leve	Masculino	de 66 a 75 anos
	Estágio 1 (pele íntegra com						Leve	Feminino	de 56 a 65 anos
	Estágio 2 (perda parcial da espessura						Leve	Feminino	de 76 a 85 anos
	Estágio 1 (pele íntegra com						Leve	Masculino	de 66 a 75 anos
	Estágio 2 (perda parcial da espessura						Leve	Feminino	de 66 a 75 anos
	Estágio 2 (perda parcial da espessura						Leve	Feminino	de 56 a 65 anos
	Estágio 2 (perda parcial da espessura						Leve	Masculino	de 56 a 65 anos

							Leve	Masculino	de 76 a 85 anos
							Leve	Masculino	de 66 a 75 anos
							Leve	Feminino	de 46 a 55 anos
	Estágio 3 (perda total da espessura						Moderado	Feminino	de 76 a 85 anos
	Estágio 2 (perda parcial da espessura						Leve	Masculino	de 66 a 75 anos
	Estágio 3 (perda total da espessura						Moderado	Masculino	de 66 a 75 anos
							Moderado	Feminino	de 36 a 45 anos
							Leve	Feminino	de 46 a 55 anos
							Leve	Feminino	de 26 a 35 anos
							Leve	Masculino	de 46 a 55 anos
	Estágio 1 (pele íntegra com						Leve	Masculino	de 66 a 75 anos
							Nenhum	Masculino	de 56 a 65 anos
	Estágio 2 (perda parcial da espessura						Leve	Masculino	de 46 a 55 anos
	Estágio 2 (perda parcial da espessura						Leve	Feminino	de 56 a 65 anos
							Leve	Feminino	de 76 a 85 anos

	Estágio 1 (pele íntegra com						Leve	Masculino	de 36 a 45 anos
	Estágio 2 (perda parcial da espessura						Leve	Masculino	de 46 a 55 anos
	Estágio 2 (perda parcial da espessura						Leve	Feminino	de 56 a 65 anos
	Estágio 3 (perda total da espessura						Moderado	Masculino	de 66 a 75 anos
	Estágio 2 (perda parcial da espessura						Leve	Masculino	de 66 a 75 anos
	Estágio 1 (pele íntegra com						Leve	Masculino	de 36 a 45 anos
	Estágio 1 (pele íntegra com						Leve	Feminino	de 56 a 65 anos
	Estágio 1 (pele íntegra com						Leve	Feminino	de 76 a 85 anos
	Estágio 1 (pele íntegra com						Leve	Masculino	de 46 a 55 anos
	Estágio 1 (pele íntegra com						Leve	Masculino	de 66 a 75 anos
	Estágio 2 (perda parcial da espessura						Leve	Masculino	de 76 a 85 anos
	Estágio 1 (pele íntegra com						Leve	Masculino	de 56 a 65 anos
	Estágio 2 (perda parcial da espessura						Leve	Feminino	de 56 a 65 anos
	Estágio 2 (perda parcial da espessura						Leve	Feminino	mais de 85 anos
	Estágio 1 (pele íntegra com						Leve	Masculino	de 66 a 75 anos

	Estágio 2 (perda parcial da espessura						Leve	Feminino	de 46 a 55 anos
	Estágio 3 (perda total da espessura						Moderado	Feminino	de 76 a 85 anos
	Estágio 2 (perda parcial da espessura						Leve	Masculino	de 76 a 85 anos
	Estágio 2 (perda parcial da espessura						Leve	Feminino	de 56 a 65 anos
	Estágio 2 (perda parcial da espessura						Leve	Masculino	de 56 a 65 anos
	Estágio 2 (perda parcial da espessura						Leve	Feminino	de 56 a 65 anos
	Estágio 2 (perda parcial da espessura						Leve	Feminino	de 66 a 75 anos
	Estágio 2 (perda parcial da espessura						Leve	Feminino	de 36 a 45 anos
	Estágio 2 (perda parcial da espessura						Leve	Masculino	de 66 a 75 anos
	Estágio 3 (perda total da espessura						Moderado	Feminino	de 76 a 85 anos
	Estágio 2 (perda parcial da espessura						Leve	Masculino	de 46 a 55 anos
	Estágio 2 (perda parcial da espessura						Leve	Feminino	de 76 a 85 anos
	Estágio 3 (perda total da espessura						Moderado	Masculino	de 26 a 35 anos
	Estágio 1 (pele íntegra com						Leve	Masculino	de 56 a 65 anos
	Estágio 2 (perda parcial da espessura						Leve	Masculino	de 66 a 75 anos

	Estágio 2 (perda parcial da espessura						Leve	Masculino	de 56 a 65 anos
	Estágio 2 (perda parcial da espessura						Leve	Masculino	de 66 a 75 anos
	Estágio 2 (perda parcial da espessura						Leve	Feminino	de 26 a 35 anos
							Leve	Feminino	de 26 a 35 anos
	Estágio 3 (perda total da espessura						Moderado	Masculino	de 66 a 75 anos
	Estágio 2 (perda parcial da espessura						Leve	Masculino	mais de 85 anos
	Estágio 2 (perda parcial da espessura						Leve	Feminino	de 26 a 35 anos
	Estágio 2 (perda parcial da espessura						Leve	Masculino	de 56 a 65 anos
	Estágio 3 (perda total da espessura						Moderado	Masculino	de 76 a 85 anos
	Estágio 2 (perda parcial da espessura						Leve	Feminino	de 76 a 85 anos
	Estágio 2 (perda parcial da espessura						Leve	Masculino	de 46 a 55 anos
	Estágio 2 (perda parcial da espessura						Leve	Feminino	de 18 a 25 anos
	Estágio 2 (perda parcial da espessura						Leve	Feminino	de 46 a 55 anos
	Estágio 2 (perda parcial da espessura						Leve	Feminino	de 46 a 55 anos
							Leve	Feminino	de 46 a 55 anos

	Estágio 2 (perda parcial da espessura						Leve	Feminino	de 66 a 75 anos
	Estágio 2 (perda parcial da espessura						Leve	Feminino	de 76 a 85 anos
							Leve	Feminino	de 76 a 85 anos
	Estágio 2 (perda parcial da espessura						Leve	Feminino	mais de 85 anos
	Estágio 1 (pele íntegra com						Leve	Feminino	de 76 a 85 anos
	Estágio 2 (perda parcial da espessura						Leve	Masculino	mais de 85 anos
	Estágio 1 (pele íntegra com						Leve	Masculino	mais de 85 anos
	Estágio 1 (pele íntegra com						Leve	Feminino	de 66 a 75 anos
	Estágio 1 (pele íntegra com						Leve	Feminino	de 66 a 75 anos
	Estágio 1 (pele íntegra com						Leve	Feminino	de 66 a 75 anos
	Estágio 2 (perda parcial da espessura						Leve	Feminino	mais de 85 anos
	Estágio 3 (perda total da espessura						Leve	Masculino	de 46 a 55 anos
	Estágio 3 (perda total da espessura						Moderado	Masculino	de 46 a 55 anos
	Estágio 2 (perda parcial da espessura						Leve	Feminino	de 66 a 75 anos
	Estágio 2 (perda parcial da espessura						Leve	Masculino	mais de 85 anos

	Estágio 2 (perda parcial da espessura						Leve	Feminino	de 66 a 75 anos
	Estágio 2 (perda parcial da espessura						Leve	Feminino	mais de 85 anos
	Estágio 2 (perda parcial da espessura						Leve	Feminino	de 76 a 85 anos
	Estágio 2 (perda parcial da espessura						Leve	Masculino	de 56 a 65 anos
	Estágio 2 (perda parcial da espessura						Leve	Masculino	mais de 85 anos
	Estágio 2 (perda parcial da espessura						Leve	Feminino	de 56 a 65 anos
	Estágio 2 (perda parcial da espessura						Leve	Feminino	de 76 a 85 anos
	Estágio 2 (perda parcial da espessura						Leve	Masculino	de 46 a 55 anos
	Estágio 2 (perda parcial da espessura						Leve	Feminino	de 56 a 65 anos
	Estágio 2 (perda parcial da espessura						Leve	Masculino	mais de 85 anos
	Estágio 2 (perda parcial da espessura						Leve	Feminino	de 66 a 75 anos
	Estágio 1 (pele íntegra com						Leve	Feminino	de 66 a 75 anos
	Estágio 1 (pele íntegra com						Leve	Feminino	de 66 a 75 anos
	Estágio 2 (perda parcial da espessura						Leve	Feminino	mais de 85 anos
	Estágio 2 (perda parcial da espessura						Leve	Masculino	de 66 a 75 anos

	Estágio 2 (perda parcial da espessura						Leve	Masculino	de 76 a 85 anos
	Estágio 3 (perda total da espessura						Moderado	Masculino	de 76 a 85 anos
	Estágio 1 (pele íntegra com						Leve	Masculino	de 66 a 75 anos
	Estágio 2 (perda parcial da espessura						Leve	Masculino	de 56 a 65 anos
	Estágio 1 (pele íntegra com						Leve	Masculino	mais de 85 anos
	Estágio 3 (perda total da espessura						Moderado	Feminino	de 26 a 35 anos
	Estágio 3 (perda total da espessura						Moderado	Masculino	de 66 a 75 anos
	Estágio 2 (perda parcial da espessura						Leve	Masculino	de 76 a 85 anos
	Estágio 3 (perda total da espessura						Moderado	Masculino	de 76 a 85 anos
	Estágio 2 (perda parcial da espessura						Leve	Masculino	de 46 a 55 anos
	Estágio 3 (perda total da espessura						Moderado	Feminino	de 26 a 35 anos
	Estágio 2 (perda parcial da espessura						Leve	Feminino	de 46 a 55 anos
	Estágio 2 (perda parcial da espessura						Leve	Feminino	de 66 a 75 anos
	Estágio 2 (perda parcial da espessura						Leve	Masculino	mais de 85 anos
	Estágio 1 (pele íntegra com						Leve	Masculino	de 56 a 65 anos

	Estágio 3 (perda total da espessura						Moderado	Masculino	de 66 a 75 anos
	Estágio 2 (perda parcial da espessura						Leve	Masculino	de 66 a 75 anos
	Estágio 2 (perda parcial da espessura						Leve	Masculino	de 66 a 75 anos
	Estágio 2 (perda parcial da espessura						Leve	Masculino	de 66 a 75 anos
	Estágio 1 (pele íntegra com						Leve	Feminino	de 66 a 75 anos
	Estágio 1 (pele íntegra com						Leve	Feminino	de 66 a 75 anos
	Estágio 3 (perda total da espessura						Leve	Feminino	de 18 a 25 anos
	Estágio 2 (perda parcial da espessura						Leve	Masculino	de 76 a 85 anos
	Estágio 2 (perda parcial da espessura						Leve	Masculino	de 66 a 75 anos
	Estágio 3 (perda total da espessura						Moderado	Masculino	de 76 a 85 anos
	Estágio 1 (pele íntegra com						Leve	Masculino	de 66 a 75 anos
	Estágio 2 (perda parcial da espessura						Leve	Masculino	de 66 a 75 anos
	Estágio 2 (perda parcial da espessura						Leve	Masculino	de 26 a 35 anos
	Estágio 2 (perda parcial da espessura						Leve	Masculino	de 66 a 75 anos
	Estágio 2 (perda parcial da espessura						Leve	Masculino	de 76 a 85 anos

	Estágio 2 (perda parcial da espessura						Leve	Feminino	de 66 a 75 anos
	Estágio 2 (perda parcial da espessura						Leve	Feminino	de 76 a 85 anos
	Estágio 1 (pele íntegra com						Leve	Masculino	de 76 a 85 anos
	Estágio 1 (pele íntegra com						Leve	Masculino	de 76 a 85 anos
	Estágio 2 (perda parcial da espessura						Leve	Masculino	de 36 a 45 anos
	Estágio 2 (perda parcial da espessura						Leve	Feminino	de 46 a 55 anos
	Estágio 1 (pele íntegra com						Leve	Feminino	de 56 a 65 anos
	Estágio 3 (perda total da espessura						Leve	Masculino	de 66 a 75 anos
							Leve	Masculino	de 66 a 75 anos
	Estágio 3 (perda total da espessura						Moderado	Masculino	de 66 a 75 anos
	Estágio 1 (pele íntegra com						Leve	Feminino	de 56 a 65 anos
	Estágio 3 (perda total da espessura						Moderado	Masculino	de 66 a 75 anos
	Estágio 2 (perda parcial da espessura						Leve	Feminino	de 18 a 25 anos
	Estágio 3 (perda total da espessura						Moderado	Masculino	de 66 a 75 anos
	Estágio 1 (pele íntegra com						Leve	Feminino	de 56 a 65 anos

	Estágio 3 (perda total da espessura						Moderado	Masculino	de 66 a 75 anos
	Estágio 2 (perda parcial da espessura						Leve	Masculino	de 66 a 75 anos
	Estágio 2 (perda parcial da espessura						Leve	Masculino	de 56 a 65 anos
	Estágio 2 (perda parcial da espessura						Leve	Feminino	de 46 a 55 anos
	Estágio 2 (perda parcial da espessura						Leve	Masculino	de 46 a 55 anos
	Estágio 3 (perda total da espessura						Moderado	Feminino	de 56 a 65 anos
	Estágio 2 (perda parcial da espessura						Leve	Feminino	de 46 a 55 anos
	Estágio 2 (perda parcial da espessura						Leve	Feminino	de 56 a 65 anos
	Estágio 2 (perda parcial da espessura						Leve	Feminino	de 46 a 55 anos
	Estágio 2 (perda parcial da espessura						Leve	Feminino	de 56 a 65 anos
	Estágio 1 (pele íntegra com						Leve	Masculino	de 56 a 65 anos
	Estágio 2 (perda parcial da espessura						Leve	Feminino	de 66 a 75 anos
	Estágio 2 (perda parcial da espessura						Leve	Masculino	de 56 a 65 anos
	Estágio 2 (perda parcial da espessura						Leve	Feminino	de 76 a 85 anos
	Estágio 3 (perda total da espessura						Moderado	Masculino	de 56 a 65 anos

	Estágio 1 (pele íntegra com						Leve	Masculino	de 46 a 55 anos
	Estágio 2 (perda parcial da espessura						Leve	Masculino	de 66 a 75 anos
	Estágio 1 (pele íntegra com						Leve	Feminino	de 76 a 85 anos
	Estágio 2 (perda parcial da espessura						Leve	Feminino	de 46 a 55 anos
	Estágio 2 (perda parcial da espessura						Leve	Feminino	de 66 a 75 anos
	Estágio 2 (perda parcial da espessura						Leve	Masculino	de 56 a 65 anos
	Estágio 2 (perda parcial da espessura						Leve	Masculino	de 36 a 45 anos
	Estágio 1 (pele íntegra com						Leve	Masculino	de 46 a 55 anos
	Estágio 2 (perda parcial da espessura						Leve	Feminino	de 66 a 75 anos
	Estágio 1 (pele íntegra com						Leve	Masculino	de 56 a 65 anos
	Estágio 2 (perda parcial da espessura						Leve	Masculino	de 66 a 75 anos
	Estágio 2 (perda parcial da espessura						Leve	Feminino	de 56 a 65 anos
	Estágio 1 (pele íntegra com						Leve	Feminino	de 46 a 55 anos
	Estágio 1 (pele íntegra com						Leve	Feminino	de 66 a 75 anos
	Estágio 2 (perda parcial da espessura						Leve	Feminino	mais de 85 anos

	Estágio 2 (perda parcial da espessura						Leve	Feminino	de 56 a 65 anos
	Estágio 1 (pele íntegra com						Leve	Feminino	de 46 a 55 anos
	Estágio 2 (perda parcial da espessura						Leve	Feminino	de 66 a 75 anos
	Estágio 2 (perda parcial da espessura						Leve	Masculino	de 46 a 55 anos
	Estágio 2 (perda parcial da espessura						Leve	Masculino	de 56 a 65 anos
	Estágio 2 (perda parcial da espessura						Leve	Masculino	de 76 a 85 anos
	Estágio 3 (perda total da espessura						Leve	Masculino	de 56 a 65 anos
	Estágio 2 (perda parcial da espessura						Leve	Feminino	de 66 a 75 anos
	Estágio 2 (perda parcial da espessura						Leve	Masculino	de 46 a 55 anos
	Estágio 2 (perda parcial da espessura						Leve	Masculino	de 46 a 55 anos
	Estágio 2 (perda parcial da espessura						Leve	Feminino	de 46 a 55 anos
	Estágio 2 (perda parcial da espessura						Leve	Feminino	de 66 a 75 anos
	Estágio 2 (perda parcial da espessura						Leve	Feminino	de 66 a 75 anos
	Estágio 2 (perda parcial da espessura						Leve	Feminino	de 46 a 55 anos
	Estágio 2 (perda parcial da espessura						Leve	Masculino	de 66 a 75 anos

	Estágio 3 (perda total da espessura						Moderado	Masculino	de 56 a 65 anos
	Estágio 2 (perda parcial da espessura						Leve	Masculino	de 56 a 65 anos
	Estágio 3 (perda total da espessura						Moderado	Masculino	de 56 a 65 anos
	Estágio 2 (perda parcial da espessura						Leve	Masculino	de 56 a 65 anos
	Estágio 1 (pele íntegra com						Leve	Feminino	de 56 a 65 anos
	Estágio 2 (perda parcial da espessura						Leve	Feminino	de 76 a 85 anos
	Estágio 3 (perda total da espessura						Moderado	Feminino	de 76 a 85 anos
	Estágio 3 (perda total da espessura						Moderado	Feminino	de 76 a 85 anos
	Estágio 2 (perda parcial da espessura						Leve	Feminino	de 76 a 85 anos
	Estágio 1 (pele íntegra com						Leve	Feminino	de 36 a 45 anos
	Estágio 2 (perda parcial da espessura						Leve	Masculino	de 56 a 65 anos
	Estágio 2 (perda parcial da espessura						Leve	Feminino	de 46 a 55 anos
	Estágio 2 (perda parcial da espessura						Leve	Masculino	de 46 a 55 anos
	Estágio 2 (perda parcial da espessura						Leve	Feminino	de 56 a 65 anos
	Estágio 1 (pele íntegra com						Leve	Feminino	de 56 a 65 anos

	Estágio 2 (perda parcial da espessura						Leve	Feminino	de 76 a 85 anos
	Estágio 2 (perda parcial da espessura						Leve	Masculino	de 66 a 75 anos
	Estágio 2 (perda parcial da espessura						Leve	Feminino	de 76 a 85 anos
	Estágio 2 (perda parcial da espessura						Leve	Feminino	de 76 a 85 anos
	Estágio 1 (pele íntegra com						Leve	Feminino	de 56 a 65 anos
	Estágio 2 (perda parcial da espessura						Leve	Masculino	de 26 a 35 anos
	Estágio 2 (perda parcial da espessura						Leve	Feminino	de 46 a 55 anos
	Estágio 2 (perda parcial da espessura						Leve	Masculino	de 26 a 35 anos
	Estágio 3 (perda total da espessura						Moderado	Masculino	de 46 a 55 anos
	Estágio 2 (perda parcial da espessura						Leve	Feminino	de 36 a 45 anos
	Estágio 2 (perda parcial da espessura						Leve	Feminino	de 56 a 65 anos
	Estágio 3 (perda total da espessura						Moderado	Feminino	de 56 a 65 anos
	Estágio 3 (perda total da espessura						Moderado	Masculino	de 46 a 55 anos
	Estágio 1 (pele íntegra com						Leve	Masculino	de 36 a 45 anos
	Estágio 2 (perda parcial da espessura						Leve	Masculino	de 26 a 35 anos

	Estágio 2 (perda parcial da espessura						Leve	Masculino	de 56 a 65 anos
	Estágio 2 (perda parcial da espessura						Leve	Feminino	de 66 a 75 anos
	Estágio 3 (perda total da espessura						Moderado	Feminino	de 56 a 65 anos
	Estágio 3 (perda total da espessura						Moderado	Feminino	de 36 a 45 anos
	Estágio 3 (perda total da espessura						Moderado	Feminino	de 66 a 75 anos
	Estágio 3 (perda total da espessura						Moderado	Feminino	de 46 a 55 anos
	Estágio 2 (perda parcial da espessura						Leve	Feminino	de 46 a 55 anos
	Estágio 1 (pele íntegra com						Leve	Masculino	de 56 a 65 anos
	Estágio 2 (perda parcial da espessura						Leve	Feminino	de 56 a 65 anos
	Estágio 1 (pele íntegra com						Leve	Masculino	de 76 a 85 anos
	Estágio 1 (pele íntegra com						Leve	Feminino	de 66 a 75 anos
	Estágio 2 (perda parcial da espessura						Leve	Feminino	de 36 a 45 anos
	Estágio 3 (perda total da espessura						Moderado	Masculino	de 56 a 65 anos
	Estágio 2 (perda parcial da espessura						Leve	Masculino	de 56 a 65 anos
	Estágio 2 (perda parcial da espessura						Leve	Masculino	de 56 a 65 anos

	Estágio 2 (perda parcial da espessura						Leve	Feminino	de 76 a 85 anos
	Estágio 2 (perda parcial da espessura						Leve	Feminino	de 66 a 75 anos
	Estágio 2 (perda parcial da espessura						Leve	Masculino	de 36 a 45 anos
	Estágio 1 (pele íntegra com						Leve	Masculino	de 56 a 65 anos
	Estágio 2 (perda parcial da espessura						Leve	Masculino	de 46 a 55 anos
	Estágio 3 (perda total da espessura						Moderado	Feminino	de 56 a 65 anos
	Estágio 1 (pele íntegra com						Leve	Feminino	de 76 a 85 anos
	Estágio 2 (perda parcial da espessura						Leve	Feminino	de 56 a 65 anos
	Estágio 2 (perda parcial da espessura						Leve	Masculino	de 26 a 35 anos
							Nenhum	Masculino	de 56 a 65 anos
							Leve	Feminino	de 36 a 45 anos
							Leve	Masculino	de 26 a 35 anos
							Nenhum	Masculino	de 18 a 25 anos
	Estágio 1 (pele íntegra com						Leve	Feminino	mais de 85 anos
	Estágio 1 (pele íntegra com						Leve	Feminino	mais de 85 anos

	Estágio 1 (pele íntegra com						Leve	Masculino	de 66 a 75 anos
	Estágio 1 (pele íntegra com						Leve	Masculino	de 66 a 75 anos
	Estágio 1 (pele íntegra com						Leve	Feminino	de 56 a 65 anos
	Estágio 1 (pele íntegra com						Leve	Feminino	de 56 a 65 anos
	Estágio 2 (perda parcial da espessura						Leve	Feminino	de 46 a 55 anos
	Estágio 1 (pele íntegra com						Leve	Masculino	de 56 a 65 anos
	Estágio 2 (perda parcial da espessura						Leve	Masculino	de 56 a 65 anos
	Estágio 1 (pele íntegra com						Leve	Feminino	mais de 85 anos
	Estágio 3 (perda total da espessura						Moderado	Masculino	de 56 a 65 anos
	Estágio 3 (perda total da espessura						Moderado	Masculino	de 56 a 65 anos
	Estágio 2 (perda parcial da espessura						Leve	Masculino	de 66 a 75 anos
	Estágio 1 (pele íntegra com						Leve	Masculino	de 76 a 85 anos
	Estágio 1 (pele íntegra com						Leve	Masculino	de 76 a 85 anos
	Estágio 3 (perda total da espessura						Moderado	Masculino	de 36 a 45 anos
	Estágio 3 (perda total da espessura						Moderado	Feminino	de 46 a 55 anos

	Estágio 2 (perda parcial da espessura						Leve	Masculino	de 36 a 45 anos
	Estágio 2 (perda parcial da espessura						Leve	Feminino	de 56 a 65 anos
	Estágio 2 (perda parcial da espessura						Leve	Masculino	de 56 a 65 anos
	Estágio 3 (perda total da espessura						Moderado	Masculino	de 56 a 65 anos
	Estágio 2 (perda parcial da espessura						Leve	Feminino	de 76 a 85 anos
	Estágio 1 (pele íntegra com						Leve	Masculino	de 46 a 55 anos
	Estágio 2 (perda parcial da espessura						Leve	Feminino	de 56 a 65 anos
	Estágio 1 (pele íntegra com						Leve	Masculino	de 36 a 45 anos
	Estágio 2 (perda parcial da espessura						Leve	Feminino	de 46 a 55 anos
	Estágio 2 (perda parcial da espessura						Leve	Feminino	de 46 a 55 anos
	Estágio 2 (perda parcial da espessura						Leve	Feminino	de 36 a 45 anos
	Estágio 4 (perda total da espessura						Moderado	Feminino	mais de 85 anos
	Estágio 3 (perda total da espessura						Moderado	Masculino	de 26 a 35 anos
	Estágio 2 (perda parcial da espessura						Leve	Feminino	de 56 a 65 anos
	Estágio 3 (perda total da espessura						Moderado	Masculino	de 26 a 35 anos

	Estágio 2 (perda parcial da espessura						Leve	Masculino	de 26 a 35 anos
	Estágio 2 (perda parcial da espessura						Leve	Masculino	de 36 a 45 anos
	Estágio 2 (perda parcial da espessura						Leve	Feminino	de 76 a 85 anos
	Estágio 2 (perda parcial da espessura						Leve	Feminino	de 56 a 65 anos
	Estágio 2 (perda parcial da espessura						Leve	Feminino	de 36 a 45 anos
	Estágio 1 (pele íntegra com						Leve	Masculino	de 46 a 55 anos
	Estágio 2 (perda parcial da espessura						Leve	Masculino	de 66 a 75 anos
	Estágio 2 (perda parcial da espessura						Leve	Feminino	de 46 a 55 anos
	Estágio 2 (perda parcial da espessura						Leve	Masculino	mais de 85 anos
	Estágio 3 (perda total da espessura						Moderado	Masculino	de 66 a 75 anos
	Estágio 2 (perda parcial da espessura						Leve	Masculino	de 46 a 55 anos
	Estágio 1 (pele íntegra com						Leve	Masculino	de 46 a 55 anos
	Estágio 2 (perda parcial da espessura						Leve	Feminino	mais de 85 anos
	Estágio 2 (perda parcial da espessura						Leve	Feminino	de 46 a 55 anos
	Estágio 2 (perda parcial da espessura						Leve	Masculino	de 46 a 55 anos

	Estágio 2 (perda parcial da espessura						Leve	Masculino	de 46 a 55 anos
	Estágio 3 (perda total da espessura						Moderado	Masculino	de 66 a 75 anos
	Estágio 2 (perda parcial da espessura						Leve	Masculino	de 46 a 55 anos
	Estágio 4 (perda total da espessura						Moderado	Feminino	de 76 a 85 anos
	Estágio 2 (perda parcial da espessura						Leve	Masculino	de 56 a 65 anos
	Estágio 1 (pele íntegra com						Leve	Masculino	de 46 a 55 anos
	Estágio 2 (perda parcial da espessura						Leve	Feminino	de 36 a 45 anos
	Estágio 2 (perda parcial da espessura						Leve	Masculino	de 36 a 45 anos
	Estágio 2 (perda parcial da espessura						Leve	Masculino	de 66 a 75 anos
	Estágio 2 (perda parcial da espessura						Leve	Feminino	de 46 a 55 anos
	Estágio 2 (perda parcial da espessura						Leve	Feminino	de 46 a 55 anos
	Estágio 2 (perda parcial da espessura						Leve	Feminino	de 46 a 55 anos
	Estágio 2 (perda parcial da espessura						Leve	Masculino	de 56 a 65 anos
	Estágio 2 (perda parcial da espessura						Leve	Feminino	de 66 a 75 anos
	Estágio 2 (perda parcial da espessura						Leve	Feminino	de 76 a 85 anos

	Estágio 3 (perda total da espessura						Moderado	Feminino	de 36 a 45 anos
	Estágio 4 (perda total da espessura						Moderado	Feminino	de 36 a 45 anos
	Estágio 3 (perda total da espessura						Moderado	Masculino	de 26 a 35 anos
	Estágio 3 (perda total da espessura						Moderado	Masculino	de 26 a 35 anos
	Estágio 2 (perda parcial da espessura						Leve	Feminino	de 56 a 65 anos
	Estágio 2 (perda parcial da espessura						Leve	Feminino	de 12 a 17 anos
	Estágio 2 (perda parcial da espessura						Leve	Feminino	de 46 a 55 anos
	Estágio 1 (pele íntegra com						Leve	Feminino	de 76 a 85 anos
	Estágio 2 (perda parcial da espessura						Leve	Feminino	de 56 a 65 anos
	Estágio 1 (pele íntegra com						Leve	Masculino	de 66 a 75 anos
	Estágio 2 (perda parcial da espessura						Leve	Masculino	de 56 a 65 anos
	Estágio 2 (perda parcial da espessura						Leve	Feminino	de 76 a 85 anos
	Estágio 3 (perda total da espessura						Moderado	Feminino	de 66 a 75 anos
	Estágio 1 (pele íntegra com						Leve	Feminino	de 66 a 75 anos
	Estágio 1 (pele íntegra com						Leve	Masculino	de 76 a 85 anos

	Estágio 1 (pele íntegra com						Leve	Feminino	de 46 a 55 anos
	Estágio 2 (perda parcial da espessura						Leve	Feminino	de 46 a 55 anos
	Estágio 3 (perda total da espessura						Moderado	Feminino	de 76 a 85 anos
	Estágio 2 (perda parcial da espessura						Leve	Feminino	de 56 a 65 anos
	Estágio 2 (perda parcial da espessura						Leve	Feminino	de 66 a 75 anos
	Estágio 2 (perda parcial da espessura						Leve	Masculino	de 46 a 55 anos
	Estágio 2 (perda parcial da espessura						Leve	Masculino	de 46 a 55 anos
	Estágio 1 (pele íntegra com						Leve	Masculino	de 76 a 85 anos
	Estágio 2 (perda parcial da espessura						Leve	Masculino	de 46 a 55 anos
	Estágio 2 (perda parcial da espessura						Leve	Masculino	de 56 a 65 anos
	Estágio 2 (perda parcial da espessura						Leve	Masculino	de 66 a 75 anos
	Estágio 2 (perda parcial da espessura						Leve	Feminino	de 56 a 65 anos
	Estágio 2 (perda parcial da espessura						Leve	Masculino	de 66 a 75 anos
	Estágio 2 (perda parcial da espessura						Leve	Masculino	mais de 85 anos
	Estágio 1 (pele íntegra com						Leve	Masculino	de 36 a 45 anos

	Estágio 2 (perda parcial da espessura						Leve	Masculino	de 76 a 85 anos
							Leve	Masculino	de 76 a 85 anos
	Estágio 2 (perda parcial da espessura						Leve	Masculino	de 76 a 85 anos
	Estágio 3 (perda total da espessura						Moderado	Feminino	de 56 a 65 anos
	Estágio 2 (perda parcial da espessura						Leve	Masculino	de 36 a 45 anos
	Estágio 2 (perda parcial da espessura						Leve	Masculino	de 76 a 85 anos
	Estágio 2 (perda parcial da espessura						Leve	Masculino	de 46 a 55 anos
	Estágio 1 (pele íntegra com						Leve	Feminino	de 66 a 75 anos
	Estágio 2 (perda parcial da espessura						Leve	Feminino	de 36 a 45 anos
	Estágio 2 (perda parcial da espessura						Leve	Feminino	de 66 a 75 anos
	Estágio 2 (perda parcial da espessura						Leve	Masculino	de 56 a 65 anos
	Estágio 2 (perda parcial da espessura						Leve	Feminino	de 46 a 55 anos
	Estágio 2 (perda parcial da espessura						Leve	Masculino	de 76 a 85 anos
	Estágio 2 (perda parcial da espessura						Leve	Feminino	de 66 a 75 anos
	Estágio 1 (pele íntegra com						Leve	Masculino	de 66 a 75 anos

	Estagio 2 (perda parcial da espessura						Leve	Feminino	de 46 a 55 anos
	Estagio 2 (perda parcial da espessura						Leve	Masculino	mais de 85 anos
	Estagio 2 (perda parcial da espessura						Leve	Masculino	de 66 a 75 anos
	Estagio 2 (perda parcial da espessura						Leve	Feminino	de 66 a 75 anos
	Estagio 3 (perda total da espessura						Leve	Feminino	de 66 a 75 anos
	Estagio 1 (pele íntegra com						Leve	Feminino	menos de 28 dias
	Estagio 2 (perda parcial da espessura						Leve	Masculino	de 36 a 45 anos
	Estagio 2 (perda parcial da espessura						Leve	Masculino	de 66 a 75 anos
	Estagio 2 (perda parcial da espessura						Leve	Masculino	de 66 a 75 anos
	Estagio 2 (perda parcial da espessura						Leve	Masculino	de 66 a 75 anos
	Estagio 2 (perda parcial da espessura						Leve	Feminino	de 76 a 85 anos
	Estagio 2 (perda parcial da espessura						Leve	Masculino	de 46 a 55 anos
	Estagio 2 (perda parcial da espessura						Leve	Masculino	de 56 a 65 anos
	Estagio 2 (perda parcial da espessura						Leve	Feminino	de 36 a 45 anos
	Estagio 3 (perda total da espessura						Moderado	Masculino	de 76 a 85 anos

	Estágio 3 (perda total da espessura						Leve	Feminino	de 76 a 85 anos
	Estágio 2 (perda parcial da espessura						Leve	Masculino	de 76 a 85 anos
	Estágio 1 (pele íntegra com						Leve	Feminino	mais de 85 anos
	Estágio 1 (pele íntegra com						Leve	Feminino	mais de 85 anos
	Estágio 1 (pele íntegra com						Leve	Feminino	mais de 85 anos
	Estágio 2 (perda parcial da espessura						Leve	Feminino	de 66 a 75 anos
	Estágio 1 (pele íntegra com						Leve	Masculino	de 76 a 85 anos
	Estágio 1 (pele íntegra com						Leve	Masculino	de 66 a 75 anos
	Estágio 1 (pele íntegra com						Leve	Masculino	mais de 85 anos
	Estágio 2 (perda parcial da espessura						Leve	Masculino	de 46 a 55 anos
	Estágio 2 (perda parcial da espessura						Leve	Masculino	de 66 a 75 anos
	Estágio 2 (perda parcial da espessura						Leve	Feminino	de 56 a 65 anos
	Estágio 2 (perda parcial da espessura						Leve	Masculino	de 56 a 65 anos
							Leve	Masculino	de 66 a 75 anos
							Leve	Feminino	de 76 a 85 anos

							Leve	Masculino	de 66 a 75 anos
							Leve	Feminino	de 76 a 85 anos
							Leve	Masculino	de 56 a 65 anos
							Leve	Masculino	de 66 a 75 anos
							Leve	Masculino	de 76 a 85 anos
							Leve	Masculino	de 66 a 75 anos
							Leve	Masculino	de 76 a 85 anos
							Leve	Masculino	de 76 a 85 anos
	Estágio 2 (perda parcial da espessura						Leve	Feminino	de 56 a 65 anos
	Estágio 2 (perda parcial da espessura						Leve	Feminino	de 46 a 55 anos
	Estágio 2 (perda parcial da espessura						Leve	Feminino	de 46 a 55 anos
	Estágio 2 (perda parcial da espessura						Leve	Masculino	de 46 a 55 anos
	Estágio 2 (perda parcial da espessura						Leve	Masculino	de 56 a 65 anos
	Estágio 2 (perda parcial da espessura						Leve	Masculino	de 26 a 35 anos
	Estágio 2 (perda parcial da espessura						Leve	Masculino	de 76 a 85 anos

	Estágio 4 (perda total da espessura						Moderado	Masculino	de 66 a 75 anos
	Estágio 2 (perda parcial da espessura						Leve	Masculino	de 76 a 85 anos
	Estágio 1 (pele íntegra com						Leve	Masculino	de 56 a 65 anos
	Estágio 1 (pele íntegra com						Leve	Masculino	de 66 a 75 anos
Queimaduras.							Leve	Masculino	de 66 a 75 anos
	Estágio 2 (perda parcial da espessura						Leve	Masculino	de 46 a 55 anos
	Estágio 2 (perda parcial da espessura						Leve	Masculino	de 56 a 65 anos
	Estágio 3 (perda total da espessura						Moderado	Masculino	de 46 a 55 anos
	Estágio 1 (pele íntegra com						Leve	Feminino	de 56 a 65 anos
	Estágio 2 (perda parcial da espessura						Leve	Masculino	de 36 a 45 anos
							Leve	Masculino	de 36 a 45 anos
	Estágio 2 (perda parcial da espessura						Leve	Feminino	de 76 a 85 anos
	Estágio 2 (perda parcial da espessura						Leve	Masculino	de 36 a 45 anos
							Leve	Masculino	de 36 a 45 anos
	Estágio 2 (perda parcial da espessura						Leve	Masculino	de 66 a 75 anos

	Estágio 2 (perda parcial da espessura						Leve	Feminino	de 76 a 85 anos
	Estágio 1 (pele íntegra com						Leve	Masculino	de 56 a 65 anos
							Leve	Feminino	de 46 a 55 anos
							Leve	Feminino	de 46 a 55 anos
	Estágio 2 (perda parcial da espessura						Leve	Masculino	de 26 a 35 anos
	Estágio 2 (perda parcial da espessura						Leve	Feminino	de 26 a 35 anos
	Estágio 1 (pele íntegra com						Leve	Masculino	de 76 a 85 anos
	Estágio 2 (perda parcial da espessura						Leve	Masculino	de 66 a 75 anos
	Estágio 2 (perda parcial da espessura						Leve	Feminino	de 76 a 85 anos
	Estágio 2 (perda parcial da espessura						Leve	Feminino	de 66 a 75 anos
	Estágio 2 (perda parcial da espessura						Leve	Masculino	de 66 a 75 anos
	Estágio 2 (perda parcial da espessura						Leve	Feminino	de 66 a 75 anos
	Estágio 2 (perda parcial da espessura						Leve	Feminino	de 46 a 55 anos
	Estágio 2 (perda parcial da espessura						Leve	Feminino	de 56 a 65 anos
	Estágio 2 (perda parcial da espessura						Leve	Masculino	de 76 a 85 anos

	Estágio 2 (perda parcial da espessura						Leve	Masculino	de 66 a 75 anos
	Estágio 2 (perda parcial da espessura						Leve	Feminino	de 36 a 45 anos
	Estágio 2 (perda parcial da espessura						Leve	Feminino	de 76 a 85 anos
	Estágio 1 (pele íntegra com						Leve	Masculino	de 56 a 65 anos
	Estágio 2 (perda parcial da espessura						Leve	Masculino	de 76 a 85 anos
	Estágio 2 (perda parcial da espessura						Leve	Feminino	de 46 a 55 anos
	Estágio 2 (perda parcial da espessura						Leve	Masculino	de 56 a 65 anos
	Estágio 2 (perda parcial da espessura						Leve	Feminino	de 56 a 65 anos
	Estágio 2 (perda parcial da espessura						Leve	Masculino	de 46 a 55 anos
	Estágio 2 (perda parcial da espessura						Leve	Masculino	de 76 a 85 anos
	Estágio 2 (perda parcial da espessura						Leve	Masculino	de 56 a 65 anos
	Estágio 1 (pele íntegra com						Leve	Feminino	de 66 a 75 anos
	Estágio 1 (pele íntegra com						Leve	Feminino	mais de 85 anos
	Estágio 1 (pele íntegra com						Leve	Masculino	de 66 a 75 anos
	Estágio 1 (pele íntegra com						Leve	Masculino	de 76 a 85 anos

	Estágio 2 (perda parcial da espessura						Leve	Feminino	mais de 85 anos
	Estágio 2 (perda parcial da espessura						Leve	Feminino	de 56 a 65 anos
	Estágio 2 (perda parcial da espessura						Leve	Masculino	de 46 a 55 anos
	Estágio 1 (pele íntegra com						Leve	Feminino	de 76 a 85 anos
	Estágio 2 (perda parcial da espessura						Leve	Masculino	de 76 a 85 anos
	Estágio 1 (pele íntegra com						Leve	Masculino	de 76 a 85 anos
	Estágio 2 (perda parcial da espessura						Leve	Feminino	de 76 a 85 anos
	Estágio 2 (perda parcial da espessura						Leve	Feminino	de 76 a 85 anos
	Estágio 2 (perda parcial da espessura						Leve	Masculino	de 76 a 85 anos
	Estágio 1 (pele íntegra com						Leve	Masculino	de 76 a 85 anos
	Estágio 2 (perda parcial da espessura						Leve	Masculino	de 76 a 85 anos
	Estágio 2 (perda parcial da espessura						Leve	Masculino	de 56 a 65 anos
	Estágio 2 (perda parcial da espessura						Leve	Masculino	de 46 a 55 anos
	Estágio 2 (perda parcial da espessura						Leve	Feminino	de 76 a 85 anos
	Estágio 1 (pele íntegra com						Leve	Feminino	de 66 a 75 anos

	Estágio 2 (perda parcial da espessura						Leve	Masculino	de 66 a 75 anos
	Estágio 2 (perda parcial da espessura						Leve	Masculino	de 76 a 85 anos
	Estágio 2 (perda parcial da espessura						Leve	Feminino	de 66 a 75 anos
	Estágio 2 (perda parcial da espessura						Leve	Feminino	de 18 a 25 anos
	Estágio 1 (pele íntegra com						Leve	Masculino	de 76 a 85 anos
	Estágio 2 (perda parcial da espessura						Leve	Feminino	mais de 85 anos
	Estágio 2 (perda parcial da espessura						Leve	Feminino	de 76 a 85 anos
	Estágio 2 (perda parcial da espessura						Leve	Feminino	de 56 a 65 anos
	Estágio 2 (perda parcial da espessura						Leve	Masculino	de 46 a 55 anos
	Estágio 2 (perda parcial da espessura						Leve	Masculino	mais de 85 anos
	Estágio 2 (perda parcial da espessura						Leve	Masculino	de 76 a 85 anos
	Estágio 2 (perda parcial da espessura						Leve	Masculino	de 46 a 55 anos
	Estágio 3 (perda total da espessura						Moderado	Feminino	de 36 a 45 anos
	Estágio 2 (perda parcial da espessura						Leve	Masculino	de 66 a 75 anos
	Estágio 2 (perda parcial da espessura						Leve	Masculino	de 56 a 65 anos

	Estágio 1 (pele íntegra com						Leve	Feminino	de 76 a 85 anos
	Estágio 2 (perda parcial da espessura						Leve	Feminino	de 76 a 85 anos
	Estágio 2 (perda parcial da espessura						Leve	Feminino	mais de 85 anos
	Estágio 1 (pele íntegra com						Leve	Masculino	de 46 a 55 anos
	Estágio 2 (perda parcial da espessura						Leve	Feminino	de 56 a 65 anos
	Estágio 2 (perda parcial da espessura						Leve	Feminino	de 66 a 75 anos
	Estágio 2 (perda parcial da espessura						Leve	Masculino	de 66 a 75 anos
	Estágio 2 (perda parcial da espessura						Leve	Feminino	de 18 a 25 anos
	Estágio 2 (perda parcial da espessura						Leve	Masculino	de 46 a 55 anos
	Estágio 1 (pele íntegra com						Leve	Feminino	de 66 a 75 anos
	Estágio 2 (perda parcial da espessura						Leve	Masculino	de 76 a 85 anos
	Estágio 2 (perda parcial da espessura						Leve	Feminino	de 76 a 85 anos
	Estágio 2 (perda parcial da espessura						Leve	Feminino	de 56 a 65 anos
	Estágio 1 (pele íntegra com						Leve	Masculino	de 56 a 65 anos
	Estágio 2 (perda parcial da espessura						Leve	Feminino	de 76 a 85 anos

	Estágio 2 (perda parcial da espessura						Leve	Masculino	de 76 a 85 anos
	Estágio 2 (perda parcial da espessura						Leve	Masculino	de 76 a 85 anos
	Estágio 2 (perda parcial da espessura						Leve	Masculino	de 66 a 75 anos
	Estágio 3 (perda total da espessura						Moderado	Feminino	de 18 a 25 anos
	Estágio 2 (perda parcial da espessura						Leve	Feminino	de 56 a 65 anos
	Estágio 2 (perda parcial da espessura						Leve	Masculino	de 56 a 65 anos
	Estágio 1 (pele íntegra com						Leve	Feminino	de 46 a 55 anos
	Estágio 2 (perda parcial da espessura						Leve	Masculino	de 66 a 75 anos
	Estágio 2 (perda parcial da espessura						Leve	Feminino	de 56 a 65 anos
	Estágio 3 (perda total da espessura						Moderado	Feminino	de 46 a 55 anos
	Estágio 2 (perda parcial da espessura						Leve	Masculino	de 36 a 45 anos
	Estágio 2 (perda parcial da espessura						Leve	Feminino	de 56 a 65 anos
	Estágio 1 (pele íntegra com						Leve	Feminino	de 66 a 75 anos
	Estágio 2 (perda parcial da espessura						Leve	Feminino	de 56 a 65 anos
							Nenhum	Masculino	de 66 a 75 anos

							Leve	Masculino	de 76 a 85 anos
							Leve	Feminino	de 26 a 35 anos
							Leve	Masculino	de 56 a 65 anos
							Leve	Masculino	de 76 a 85 anos
							Leve	Masculino	de 76 a 85 anos
							Nenhum	Masculino	de 46 a 55 anos
							Leve	Masculino	de 66 a 75 anos
							Moderado	Feminino	de 18 a 25 anos
							Nenhum	Masculino	de 76 a 85 anos
							Nenhum	Feminino	de 56 a 65 anos
							Leve	Feminino	de 76 a 85 anos
							Leve	Feminino	de 76 a 85 anos
							Nenhum	Feminino	mais de 85 anos
							Leve	Feminino	de 18 a 25 anos
							Nenhum	Masculino	de 36 a 45 anos

							Leve	Masculino	de 76 a 85 anos
							Grave	Masculino	mais de 85 anos
							Óbito	Masculino	de 18 a 25 anos
							Leve	Masculino	mais de 85 anos
							Leve	Feminino	de 46 a 55 anos
							Leve	Masculino	de 36 a 45 anos
							Leve	Masculino	de 66 a 75 anos
							Moderado	Feminino	mais de 85 anos
							Leve	Masculino	de 36 a 45 anos
							Leve	Masculino	de 56 a 65 anos
							Leve	Masculino	de 46 a 55 anos
							Leve	Feminino	de 36 a 45 anos
							Leve	Feminino	de 76 a 85 anos
							Leve	Masculino	de 66 a 75 anos
							Moderado	Feminino	de 26 a 35 anos

							Leve	Masculino	de 56 a 65 anos
							Leve	Masculino	de 56 a 65 anos
	Estágio 2 (perda parcial da espessura						Leve	Feminino	de 36 a 45 anos
	Estágio 1 (pele íntegra com						Leve	Feminino	de 66 a 75 anos
	Estágio 4 (perda total da espessura						Moderado	Masculino	de 76 a 85 anos
	Estágio 2 (perda parcial da espessura						Leve	Masculino	de 76 a 85 anos
	Estágio 4 (perda total da espessura						Moderado	Feminino	de 36 a 45 anos
	Estágio 2 (perda parcial da espessura						Leve	Feminino	de 46 a 55 anos
	Estágio 2 (perda parcial da espessura						Leve	Feminino	de 46 a 55 anos
	Estágio 2 (perda parcial da espessura						Leve	Feminino	de 46 a 55 anos
	Estágio 2 (perda parcial da espessura						Leve	Masculino	de 56 a 65 anos
	Estágio 2 (perda parcial da espessura						Leve	Masculino	de 56 a 65 anos
	Estágio 2 (perda parcial da espessura						Leve	Masculino	de 56 a 65 anos
	Estágio 2 (perda parcial da espessura						Leve	Feminino	de 56 a 65 anos
	Estágio 2 (perda parcial da espessura						Leve	Masculino	de 76 a 85 anos

	Estágio 3 (perda total da espessura						Moderado	Masculino	de 46 a 55 anos
	Estágio 2 (perda parcial da espessura						Leve	Masculino	de 46 a 55 anos
	Estágio 1 (pele íntegra com						Leve	Masculino	de 46 a 55 anos
							Leve	Masculino	de 66 a 75 anos
	Estágio 3 (perda total da espessura						Moderado	Masculino	de 26 a 35 anos
	Estágio 3 (perda total da espessura						Moderado	Masculino	de 76 a 85 anos
	Estágio 2 (perda parcial da espessura						Leve	Masculino	de 76 a 85 anos
							Leve	Masculino	de 66 a 75 anos
	Estágio 2 (perda parcial da espessura						Leve	Feminino	de 56 a 65 anos
	Estágio 2 (perda parcial da espessura						Leve	Masculino	de 12 a 17 anos
	Estágio 1 (pele íntegra com						Leve	Masculino	de 56 a 65 anos
	Estágio 1 (pele íntegra com						Leve	Feminino	de 56 a 65 anos
	Estágio 3 (perda total da espessura						Moderado	Masculino	de 66 a 75 anos
	Estágio 2 (perda parcial da espessura						Leve	Feminino	de 46 a 55 anos
	Estágio 1 (pele íntegra com						Leve	Masculino	de 56 a 65 anos

	Estágio 1 (pele íntegra com						Leve	Masculino	de 56 a 65 anos
	Estágio 1 (pele íntegra com						Leve	Masculino	mais de 85 anos
	Estágio 1 (pele íntegra com						Leve	Masculino	de 76 a 85 anos
	Estágio 3 (perda total da espessura						Moderado	Masculino	de 76 a 85 anos
	Estágio 1 (pele íntegra com						Leve	Masculino	de 56 a 65 anos
	Estágio 1 (pele íntegra com						Leve	Feminino	de 76 a 85 anos
	Estágio 1 (pele íntegra com						Leve	Masculino	de 76 a 85 anos
	Estágio 2 (perda parcial da espessura						Leve	Feminino	de 56 a 65 anos
	Estágio 2 (perda parcial da espessura						Leve	Masculino	de 56 a 65 anos
	Estágio 4 (perda total da espessura						Moderado	Masculino	de 66 a 75 anos
	Estágio 4 (perda total da espessura						Moderado	Masculino	de 66 a 75 anos
	Estágio 3 (perda total da espessura						Moderado	Masculino	de 66 a 75 anos
	Estágio 4 (perda total da espessura						Moderado	Masculino	de 66 a 75 anos
	Estágio 2 (perda parcial da espessura						Leve	Masculino	de 66 a 75 anos
							Leve	Masculino	de 46 a 55 anos

							Leve	Masculino	de 76 a 85 anos
	Estágio 3 (perda total da espessura						Moderado	Masculino	de 56 a 65 anos
	Estágio 3 (perda total da espessura						Moderado	Feminino	de 56 a 65 anos
	Estágio 4 (perda total da espessura						Moderado	Feminino	de 46 a 55 anos
	Estágio 4 (perda total da espessura						Moderado	Masculino	de 46 a 55 anos
	Estágio 2 (perda parcial da espessura						Leve	Masculino	de 56 a 65 anos
	Estágio 2 (perda parcial da espessura						Leve	Masculino	de 56 a 65 anos
	Estágio 2 (perda parcial da espessura						Leve	Masculino	de 66 a 75 anos
	Estágio 1 (pele íntegra com						Leve	Masculino	de 76 a 85 anos
	Estágio 1 (pele íntegra com						Leve	Masculino	de 76 a 85 anos
	Estágio 2 (perda parcial da espessura						Leve	Masculino	de 46 a 55 anos
	Estágio 2 (perda parcial da espessura						Leve	Masculino	mais de 85 anos
	Estágio 1 (pele íntegra com						Leve	Feminino	menos de 28 dias
	Estágio 2 (perda parcial da espessura						Leve	Feminino	de 56 a 65 anos
	Estágio 1 (pele íntegra com						Leve	Masculino	de 76 a 85 anos

	Estágio 2 (perda parcial da espessura						Leve	Masculino	de 76 a 85 anos
	Estágio 1 (pele íntegra com						Leve	Masculino	de 56 a 65 anos
	Estágio 2 (perda parcial da espessura						Leve	Masculino	de 56 a 65 anos
	Estágio 2 (perda parcial da espessura						Leve	Masculino	de 56 a 65 anos
	Estágio 2 (perda parcial da espessura						Leve	Masculino	de 56 a 65 anos
	Estágio 2 (perda parcial da espessura						Leve	Masculino	de 66 a 75 anos
	Estágio 2 (perda parcial da espessura						Leve	Feminino	de 46 a 55 anos
	Estágio 2 (perda parcial da espessura						Leve	Feminino	de 46 a 55 anos
	Estágio 1 (pele íntegra com						Leve	Masculino	de 56 a 65 anos
	Estágio 2 (perda parcial da espessura						Leve	Masculino	de 66 a 75 anos
	Estágio 2 (perda parcial da espessura						Leve	Masculino	de 26 a 35 anos
	Estágio 1 (pele íntegra com						Leve	Masculino	de 56 a 65 anos
	Estágio 2 (perda parcial da espessura						Leve	Masculino	de 56 a 65 anos
	Estágio 3 (perda total da espessura						Moderado	Feminino	de 56 a 65 anos
	Estágio 3 (perda total da espessura						Moderado	Feminino	de 56 a 65 anos

	Estágio 2 (perda parcial da espessura						Leve	Masculino	de 46 a 55 anos
	Estágio 2 (perda parcial da espessura						Leve	Feminino	de 36 a 45 anos
	Estágio 1 (pele íntegra com						Leve	Feminino	de 36 a 45 anos
	Estágio 2 (perda parcial da espessura						Leve	Feminino	de 36 a 45 anos
	Estágio 3 (perda total da espessura						Moderado	Feminino	de 36 a 45 anos
	Estágio 1 (pele íntegra com						Leve	Masculino	de 36 a 45 anos
	Estágio 2 (perda parcial da espessura						Leve	Masculino	de 76 a 85 anos
	Estágio 2 (perda parcial da espessura						Leve	Masculino	de 36 a 45 anos
	Estágio 2 (perda parcial da espessura						Leve	Masculino	de 46 a 55 anos
	Estágio 2 (perda parcial da espessura						Leve	Masculino	de 76 a 85 anos
	Estágio 2 (perda parcial da espessura						Leve	Feminino	de 76 a 85 anos
	Estágio 1 (pele íntegra com						Leve	Masculino	de 56 a 65 anos
	Estágio 3 (perda total da espessura						Moderado	Masculino	de 56 a 65 anos
	Estágio 2 (perda parcial da espessura						Leve	Masculino	de 66 a 75 anos
	Estágio 2 (perda parcial da espessura						Leve	Masculino	de 66 a 75 anos

	Estágio 2 (perda parcial da espessura						Leve	Masculino	de 76 a 85 anos
	Estágio 2 (perda parcial da espessura						Leve	Masculino	de 76 a 85 anos
	Estágio 3 (perda total da espessura						Moderado	Masculino	de 56 a 65 anos
	Estágio 2 (perda parcial da espessura						Leve	Feminino	de 66 a 75 anos
	Estágio 2 (perda parcial da espessura						Leve	Feminino	de 66 a 75 anos
	Estágio 2 (perda parcial da espessura						Leve	Feminino	de 66 a 75 anos
	Estágio 2 (perda parcial da espessura						Leve	Masculino	de 66 a 75 anos
	Estágio 2 (perda parcial da espessura						Leve	Feminino	de 36 a 45 anos
	Estágio 2 (perda parcial da espessura						Leve	Feminino	de 46 a 55 anos
	Estágio 2 (perda parcial da espessura						Leve	Feminino	de 66 a 75 anos
	Estágio 2 (perda parcial da espessura						Leve	Masculino	de 56 a 65 anos
	Estágio 2 (perda parcial da espessura						Leve	Masculino	de 66 a 75 anos
	Estágio 2 (perda parcial da espessura						Leve	Feminino	de 66 a 75 anos
	Estágio 2 (perda parcial da espessura						Leve	Feminino	de 56 a 65 anos
	Estágio 3 (perda total da espessura						Moderado	Feminino	de 66 a 75 anos

	Estágio 2 (perda parcial da espessura						Leve	Masculino	de 26 a 35 anos
	Estágio 2 (perda parcial da espessura						Leve	Masculino	de 26 a 35 anos
	Estágio 2 (perda parcial da espessura						Leve	Masculino	de 26 a 35 anos
	Estágio 3 (perda total da espessura						Moderado	Masculino	de 66 a 75 anos
	Estágio 2 (perda parcial da espessura						Leve	Masculino	de 76 a 85 anos
	Estágio 2 (perda parcial da espessura						Leve	Masculino	de 56 a 65 anos
	Estágio 2 (perda parcial da espessura						Leve	Masculino	de 56 a 65 anos
	Estágio 1 (pele íntegra com						Leve	Masculino	mais de 85 anos
	Estágio 2 (perda parcial da espessura						Leve	Masculino	de 66 a 75 anos
	Estágio 2 (perda parcial da espessura						Leve	Masculino	de 66 a 75 anos
	Estágio 2 (perda parcial da espessura						Leve	Masculino	de 56 a 65 anos
	Estágio 1 (pele íntegra com						Leve	Masculino	de 66 a 75 anos
	Estágio 2 (perda parcial da espessura						Leve	Masculino	de 76 a 85 anos
	Estágio 2 (perda parcial da espessura						Leve	Feminino	de 66 a 75 anos
	Estágio 2 (perda parcial da espessura						Leve	Masculino	de 56 a 65 anos

	Estágio 1 (pele íntegra com						Leve	Masculino	mais de 85 anos
	Estágio 1 (pele íntegra com						Leve	Masculino	mais de 85 anos
	Estágio 2 (perda parcial da espessura						Leve	Feminino	de 46 a 55 anos
	Estágio 3 (perda total da espessura						Moderado	Masculino	de 46 a 55 anos
	Estágio 2 (perda parcial da espessura						Leve	Masculino	de 46 a 55 anos
	Estágio 2 (perda parcial da espessura						Leve	Masculino	de 46 a 55 anos
	Estágio 2 (perda parcial da espessura						Leve	Feminino	de 66 a 75 anos
	Estágio 2 (perda parcial da espessura						Leve	Feminino	de 46 a 55 anos
	Estágio 3 (perda total da espessura						Moderado	Feminino	de 76 a 85 anos
	Estágio 3 (perda total da espessura						Moderado	Feminino	de 76 a 85 anos
	Estágio 3 (perda total da espessura						Moderado	Feminino	de 76 a 85 anos
	Estágio 2 (perda parcial da espessura						Leve	Feminino	de 46 a 55 anos
	Estágio 3 (perda total da espessura						Moderado	Masculino	de 56 a 65 anos
	Estágio 2 (perda parcial da espessura						Leve	Feminino	de 76 a 85 anos
	Estágio 2 (perda parcial da espessura						Leve	Masculino	de 36 a 45 anos

	Estágio 1 (pele íntegra com						Leve	Masculino	de 66 a 75 anos
	Estágio 2 (perda parcial da espessura						Leve	Masculino	de 66 a 75 anos
	Estágio 2 (perda parcial da espessura						Leve	Feminino	de 76 a 85 anos
	Estágio 2 (perda parcial da espessura						Leve	Masculino	de 36 a 45 anos
	Estágio 2 (perda parcial da espessura						Leve	Feminino	de 36 a 45 anos
	Estágio 3 (perda total da espessura						Moderado	Feminino	de 36 a 45 anos
	Estágio 2 (perda parcial da espessura						Leve	Feminino	de 36 a 45 anos
	Estágio 2 (perda parcial da espessura						Leve	Masculino	de 56 a 65 anos
	Estágio 2 (perda parcial da espessura						Leve	Masculino	de 56 a 65 anos
	Estágio 2 (perda parcial da espessura						Leve	Feminino	de 66 a 75 anos
	Estágio 2 (perda parcial da espessura						Leve	Masculino	de 76 a 85 anos
	Estágio 2 (perda parcial da espessura						Leve	Masculino	de 56 a 65 anos
	Estágio 2 (perda parcial da espessura						Leve	Masculino	de 56 a 65 anos
	Estágio 4 (perda total da espessura						Moderado	Feminino	de 76 a 85 anos
	Estágio 2 (perda parcial da espessura						Leve	Masculino	de 26 a 35 anos

	Estágio 3 (perda total da espessura						Moderado	Masculino	de 46 a 55 anos
	Estágio 4 (perda total da espessura						Moderado	Masculino	de 66 a 75 anos
	Estágio 2 (perda parcial da espessura						Leve	Masculino	de 56 a 65 anos
	Estágio 2 (perda parcial da espessura						Leve	Masculino	de 66 a 75 anos
	Estágio 2 (perda parcial da espessura						Leve	Masculino	de 66 a 75 anos
	Estágio 2 (perda parcial da espessura						Leve	Masculino	de 66 a 75 anos
	Estágio 2 (perda parcial da espessura						Leve	Masculino	de 66 a 75 anos
	Estágio 2 (perda parcial da espessura						Leve	Feminino	de 66 a 75 anos
	Estágio 3 (perda total da espessura						Moderado	Masculino	de 18 a 25 anos
	Estágio 2 (perda parcial da espessura						Leve	Masculino	de 18 a 25 anos
	Estágio 2 (perda parcial da espessura						Leve	Feminino	de 76 a 85 anos
	Estágio 1 (pele íntegra com						Leve	Masculino	de 76 a 85 anos
	Estágio 1 (pele íntegra com						Leve	Feminino	de 56 a 65 anos
	Estágio 4 (perda total da espessura						Moderado	Masculino	de 76 a 85 anos
	Estágio 2 (perda parcial da espessura						Leve	Masculino	de 76 a 85 anos

	Estágio 2 (perda parcial da espessura						Leve	Masculino	de 76 a 85 anos
	Estágio 2 (perda parcial da espessura						Leve	Masculino	de 76 a 85 anos
	Estágio 2 (perda parcial da espessura						Leve	Masculino	de 66 a 75 anos
	Estágio 2 (perda parcial da espessura						Leve	Feminino	de 76 a 85 anos
	Estágio 2 (perda parcial da espessura						Leve	Feminino	de 76 a 85 anos
	Estágio 2 (perda parcial da espessura						Leve	Masculino	de 36 a 45 anos
	Estágio 2 (perda parcial da espessura						Leve	Masculino	de 46 a 55 anos
	Estágio 2 (perda parcial da espessura						Leve	Masculino	de 66 a 75 anos
	Estágio 2 (perda parcial da espessura						Leve	Masculino	de 66 a 75 anos
	Estágio 2 (perda parcial da espessura						Leve	Masculino	de 46 a 55 anos
	Estágio 2 (perda parcial da espessura						Leve	Masculino	de 76 a 85 anos
	Estágio 1 (pele íntegra com						Leve	Masculino	de 66 a 75 anos
	Estágio 2 (perda parcial da espessura						Leve	Feminino	de 66 a 75 anos
	Estágio 1 (pele íntegra com						Leve	Feminino	de 66 a 75 anos
	Estágio 1 (pele íntegra com						Leve	Masculino	de 66 a 75 anos

	Estágio 4 (perda total da espessura						Moderado	Masculino	de 46 a 55 anos
	Estágio 2 (perda parcial da espessura						Leve	Feminino	de 66 a 75 anos
	Estágio 2 (perda parcial da espessura						Leve	Feminino	de 46 a 55 anos
	Estágio 2 (perda parcial da espessura						Leve	Masculino	de 46 a 55 anos
	Estágio 2 (perda parcial da espessura						Leve	Masculino	mais de 85 anos
	Estágio 1 (pele íntegra com						Leve	Feminino	de 76 a 85 anos
	Estágio 3 (perda total da espessura						Moderado	Masculino	de 76 a 85 anos
	Estágio 4 (perda total da espessura						Moderado	Masculino	de 56 a 65 anos
	Estágio 4 (perda total da espessura						Moderado	Masculino	de 56 a 65 anos
	Estágio 3 (perda total da espessura						Moderado	Masculino	de 56 a 65 anos
	Estágio 2 (perda parcial da espessura						Leve	Masculino	de 46 a 55 anos
	Estágio 4 (perda total da espessura						Moderado	Feminino	de 56 a 65 anos
	Estágio 2 (perda parcial da espessura						Leve	Feminino	de 66 a 75 anos
	Estágio 2 (perda parcial da espessura						Leve	Feminino	de 46 a 55 anos
	Estágio 1 (pele íntegra com						Leve	Masculino	de 66 a 75 anos

	Estágio 2 (perda parcial da espessura						Leve	Masculino	de 66 a 75 anos
	Estágio 2 (perda parcial da espessura						Leve	Masculino	de 66 a 75 anos
	Estágio 2 (perda parcial da espessura						Leve	Feminino	de 66 a 75 anos
	Estágio 2 (perda parcial da espessura						Leve	Masculino	de 46 a 55 anos
	Estágio 1 (pele íntegra com						Leve	Masculino	de 66 a 75 anos
	Estágio 2 (perda parcial da espessura						Leve	Feminino	de 76 a 85 anos
	Estágio 2 (perda parcial da espessura						Leve	Feminino	de 76 a 85 anos
	Estágio 1 (pele íntegra com						Leve	Feminino	de 76 a 85 anos
	Estágio 1 (pele íntegra com						Leve	Masculino	de 66 a 75 anos
	Estágio 1 (pele íntegra com						Leve	Masculino	de 46 a 55 anos
	Estágio 4 (perda total da espessura						Moderado	Masculino	de 56 a 65 anos
	Estágio 2 (perda parcial da espessura						Leve	Masculino	mais de 85 anos
	Estágio 1 (pele íntegra com						Leve	Masculino	de 46 a 55 anos
	Estágio 2 (perda parcial da espessura						Leve	Masculino	de 46 a 55 anos
	Estágio 2 (perda parcial da espessura						Leve	Feminino	de 26 a 35 anos

	Estágio 4 (perda total da espessura						Moderado	Feminino	de 56 a 65 anos
	Estágio 2 (perda parcial da espessura						Leve	Feminino	de 36 a 45 anos
	Estágio 2 (perda parcial da espessura						Leve	Masculino	de 56 a 65 anos
	Estágio 2 (perda parcial da espessura						Leve	Masculino	mais de 85 anos
	Estágio 1 (pele íntegra com						Leve	Masculino	de 56 a 65 anos
	Estágio 2 (perda parcial da espessura						Leve	Masculino	de 56 a 65 anos
	Estágio 2 (perda parcial da espessura						Leve	Feminino	de 56 a 65 anos
	Estágio 3 (perda total da espessura						Moderado	Feminino	de 66 a 75 anos
	Estágio 1 (pele íntegra com						Leve	Masculino	de 76 a 85 anos
	Estágio 2 (perda parcial da espessura						Leve	Feminino	de 26 a 35 anos
	Estágio 2 (perda parcial da espessura						Leve	Masculino	de 56 a 65 anos
	Estágio 1 (pele íntegra com						Leve	Feminino	de 36 a 45 anos
	Estágio 2 (perda parcial da espessura						Leve	Feminino	de 36 a 45 anos
	Estágio 1 (pele íntegra com						Leve	Masculino	de 76 a 85 anos
	Estágio 2 (perda parcial da espessura						Leve	Masculino	de 76 a 85 anos

	Estágio 2 (perda parcial da espessura						Leve	Masculino	de 46 a 55 anos
	Estágio 2 (perda parcial da espessura						Leve	Masculino	de 56 a 65 anos
	Estágio 2 (perda parcial da espessura						Leve	Feminino	de 76 a 85 anos
	Estágio 2 (perda parcial da espessura						Leve	Feminino	de 76 a 85 anos
	Estágio 2 (perda parcial da espessura						Leve	Masculino	de 66 a 75 anos
	Estágio 2 (perda parcial da espessura						Leve	Masculino	de 66 a 75 anos
	Estágio 3 (perda total da espessura						Moderado	Feminino	de 76 a 85 anos
	Estágio 2 (perda parcial da espessura						Leve	Masculino	de 36 a 45 anos
	Estágio 2 (perda parcial da espessura						Leve	Masculino	de 36 a 45 anos
	Estágio 3 (perda total da espessura						Moderado	Masculino	de 76 a 85 anos
	Estágio 3 (perda total da espessura						Moderado	Masculino	de 56 a 65 anos
	Estágio 2 (perda parcial da espessura						Leve	Feminino	de 76 a 85 anos
	Estágio 2 (perda parcial da espessura						Leve	Masculino	de 56 a 65 anos
	Estágio 1 (pele íntegra com						Leve	Masculino	de 56 a 65 anos
	Estágio 2 (perda parcial da espessura						Leve	Masculino	de 18 a 25 anos

	Estágio 2 (perda parcial da espessura						Leve	Feminino	de 76 a 85 anos
	Estágio 2 (perda parcial da espessura						Leve	Masculino	de 76 a 85 anos
	Estágio 2 (perda parcial da espessura						Leve	Feminino	de 46 a 55 anos
	Estágio 2 (perda parcial da espessura						Leve	Masculino	de 36 a 45 anos
	Estágio 1 (pele íntegra com						Leve	Masculino	de 66 a 75 anos
	Estágio 1 (pele íntegra com						Leve	Feminino	mais de 85 anos
	Estágio 2 (perda parcial da espessura						Leve	Masculino	de 36 a 45 anos
	Estágio 2 (perda parcial da espessura						Leve	Feminino	de 76 a 85 anos
	Estágio 2 (perda parcial da espessura						Leve	Masculino	mais de 85 anos
	Estágio 1 (pele íntegra com						Leve	Feminino	de 56 a 65 anos
	Estágio 1 (pele íntegra com						Leve	Masculino	de 66 a 75 anos
	Estágio 3 (perda total da espessura						Moderado	Masculino	de 18 a 25 anos
	Estágio 3 (perda total da espessura						Moderado	Masculino	de 18 a 25 anos
	Estágio 2 (perda parcial da espessura						Leve	Masculino	de 76 a 85 anos
	Estágio 2 (perda parcial da espessura						Leve	Feminino	de 36 a 45 anos

	Estágio 2 (perda parcial da espessura						Leve	Masculino	de 66 a 75 anos
	Estágio 3 (perda total da espessura						Moderado	Masculino	de 56 a 65 anos
	Estágio 2 (perda parcial da espessura						Leve	Masculino	de 56 a 65 anos
	Estágio 2 (perda parcial da espessura						Leve	Masculino	de 76 a 85 anos
	Estágio 2 (perda parcial da espessura						Leve	Feminino	de 46 a 55 anos
	Estágio 2 (perda parcial da espessura						Leve	Masculino	de 36 a 45 anos
	Estágio 2 (perda parcial da espessura						Leve	Feminino	de 76 a 85 anos
	Estágio 2 (perda parcial da espessura						Leve	Feminino	de 18 a 25 anos
	Estágio 2 (perda parcial da espessura						Leve	Masculino	de 36 a 45 anos
	Estágio 2 (perda parcial da espessura						Leve	Feminino	de 46 a 55 anos
	Estágio 2 (perda parcial da espessura						Leve	Masculino	de 46 a 55 anos
	Estágio 2 (perda parcial da espessura						Leve	Feminino	de 26 a 35 anos
	Estágio 2 (perda parcial da espessura						Leve	Feminino	de 36 a 45 anos
	Estágio 2 (perda parcial da espessura						Leve	Feminino	de 36 a 45 anos
	Estágio 2 (perda parcial da espessura						Leve	Feminino	de 66 a 75 anos

	Estágio 2 (perda parcial da espessura						Leve	Feminino	de 36 a 45 anos
	Estágio 2 (perda parcial da espessura						Leve	Masculino	de 76 a 85 anos
	Estágio 3 (perda total da espessura						Moderado	Feminino	de 46 a 55 anos
	Estágio 1 (pele íntegra com						Leve	Masculino	de 26 a 35 anos
	Estágio 1 (pele íntegra com						Leve	Feminino	de 66 a 75 anos
	Estágio 2 (perda parcial da espessura						Leve	Feminino	de 46 a 55 anos
	Estágio 2 (perda parcial da espessura						Leve	Feminino	menos de 28 dias
	Estágio 1 (pele íntegra com						Leve	Feminino	menos de 28 dias
	Estágio 1 (pele íntegra com						Leve	Feminino	de 66 a 75 anos
	Estágio 4 (perda total da espessura						Moderado	Masculino	de 66 a 75 anos
	Estágio 4 (perda total da espessura						Moderado	Masculino	de 66 a 75 anos
	Estágio 2 (perda parcial da espessura						Leve	Feminino	de 76 a 85 anos
	Estágio 3 (perda total da espessura						Moderado	Feminino	de 56 a 65 anos
	Estágio 1 (pele íntegra com						Leve	Feminino	de 36 a 45 anos
	Estágio 1 (pele íntegra com						Leve	Feminino	de 66 a 75 anos

							Leve	Masculino	de 46 a 55 anos
							Leve	Masculino	de 76 a 85 anos
							Leve	Feminino	de 36 a 45 anos
							Leve	Feminino	de 36 a 45 anos
							Leve	Masculino	de 46 a 55 anos
							Leve	Masculino	de 26 a 35 anos
							Leve	Masculino	de 36 a 45 anos
							Leve	Feminino	de 46 a 55 anos
							Leve	Feminino	de 18 a 25 anos
							Nenhum	Feminino	de 36 a 45 anos
							Leve	Feminino	de 36 a 45 anos
							Leve	Masculino	de 66 a 75 anos
							Leve	Masculino	de 36 a 45 anos
							Moderado	Feminino	de 26 a 35 anos
							Leve	Feminino	de 66 a 75 anos

							Leve	Feminino	de 46 a 55 anos
							Leve	Feminino	de 56 a 65 anos
							Moderado	Feminino	de 46 a 55 anos
							Leve	Masculino	de 66 a 75 anos
							Leve	Masculino	de 56 a 65 anos
							Moderado	Feminino	de 46 a 55 anos
							Leve	Masculino	de 66 a 75 anos
							Leve	Feminino	de 66 a 75 anos
							Leve	Feminino	de 56 a 65 anos
							Leve	Masculino	de 66 a 75 anos
							Moderado	Feminino	de 56 a 65 anos
	Estágio 2 (perda parcial da espessura						Leve	Feminino	mais de 85 anos
	Estágio 2 (perda parcial da espessura						Leve	Feminino	de 18 a 25 anos
	Estágio 1 (pele íntegra com						Leve	Feminino	de 66 a 75 anos
	Estágio 2 (perda parcial da espessura						Leve	Feminino	de 76 a 85 anos

	Estágio 2 (perda parcial da espessura						Leve	Feminino	de 66 a 75 anos
	Estágio 2 (perda parcial da espessura						Leve	Feminino	de 76 a 85 anos
	Estágio 1 (pele íntegra com						Leve	Masculino	de 36 a 45 anos
	Estágio 2 (perda parcial da espessura						Leve	Feminino	de 36 a 45 anos
	Estágio 2 (perda parcial da espessura						Leve	Feminino	de 66 a 75 anos
	Estágio 2 (perda parcial da espessura						Leve	Feminino	mais de 85 anos
	Estágio 2 (perda parcial da espessura						Leve	Feminino	de 36 a 45 anos
	Estágio 3 (perda total da espessura						Leve	Feminino	de 56 a 65 anos
	Estágio 2 (perda parcial da espessura						Leve	Feminino	de 56 a 65 anos
							Grave	Masculino	de 66 a 75 anos
							Leve	Feminino	de 66 a 75 anos
							Leve	Masculino	de 36 a 45 anos
							Leve	Feminino	de 36 a 45 anos
							Leve	Feminino	de 66 a 75 anos
							Leve	Feminino	de 66 a 75 anos

							Leve	Feminino	de 66 a 75 anos
							Leve	Feminino	de 26 a 35 anos
							Leve	Masculino	de 56 a 65 anos
							Leve	Masculino	de 46 a 55 anos
							Leve	Feminino	de 46 a 55 anos
							Leve	Feminino	de 46 a 55 anos
							Leve	Masculino	de 76 a 85 anos
							Leve	Feminino	de 76 a 85 anos
							Moderado	Masculino	de 56 a 65 anos
							Leve	Feminino	de 26 a 35 anos
							Leve	Masculino	de 66 a 75 anos
							Leve	Masculino	mais de 85 anos
							Leve	Masculino	de 18 a 25 anos
							Leve	Feminino	de 26 a 35 anos
							Leve	Feminino	de 46 a 55 anos

							Leve	Masculino	de 46 a 55 anos
							Leve	Feminino	de 66 a 75 anos
							Leve	Feminino	de 46 a 55 anos
							Leve	Feminino	de 29 dias a 1 ano
							Leve	Feminino	de 2 a 4 anos
							Leve	Feminino	de 46 a 55 anos
							Leve	Masculino	de 66 a 75 anos
							Leve	Masculino	de 18 a 25 anos
							Leve	Feminino	de 56 a 65 anos
	Estágio 2 (perda parcial da espessura						Leve	Feminino	de 56 a 65 anos
	Estágio 2 (perda parcial da espessura						Leve	Feminino	de 46 a 55 anos
	Estágio 2 (perda parcial da espessura						Leve	Masculino	de 56 a 65 anos
	Estágio 1 (pele íntegra com						Leve	Masculino	de 56 a 65 anos
	Estágio 2 (perda parcial da espessura						Leve	Masculino	de 76 a 85 anos
	Estágio 4 (perda total da espessura						Moderado	Masculino	de 76 a 85 anos

	Estágio 1 (pele íntegra com						Leve	Masculino	de 66 a 75 anos
	Estágio 2 (perda parcial da espessura						Leve	Feminino	de 56 a 65 anos
	Estágio 2 (perda parcial da espessura						Leve	Feminino	de 56 a 65 anos
	Estágio 2 (perda parcial da espessura						Leve	Masculino	de 66 a 75 anos
	Estágio 2 (perda parcial da espessura						Leve	Masculino	de 46 a 55 anos
	Estágio 2 (perda parcial da espessura						Leve	Feminino	de 56 a 65 anos
	Estágio 1 (pele íntegra com						Leve	Feminino	de 46 a 55 anos
	Estágio 3 (perda total da espessura						Moderado	Feminino	de 76 a 85 anos
	Estágio 2 (perda parcial da espessura						Leve	Feminino	de 66 a 75 anos
	Estágio 2 (perda parcial da espessura						Leve	Masculino	de 26 a 35 anos
	Estágio 2 (perda parcial da espessura						Leve	Masculino	de 66 a 75 anos
	Estágio 2 (perda parcial da espessura						Leve	Masculino	de 66 a 75 anos
	Estágio 2 (perda parcial da espessura						Leve	Feminino	de 46 a 55 anos
	Estágio 3 (perda total da espessura						Moderado	Masculino	de 56 a 65 anos
	Estágio 3 (perda total da espessura						Moderado	Feminino	de 5 a 11 anos

	Estágio 2 (perda parcial da espessura						Leve	Feminino	de 18 a 25 anos
	Estágio 2 (perda parcial da espessura						Leve	Masculino	de 56 a 65 anos
	Estágio 1 (pele íntegra com						Leve	Feminino	de 46 a 55 anos
	Estágio 2 (perda parcial da espessura						Leve	Masculino	de 36 a 45 anos
	Estágio 2 (perda parcial da espessura						Leve	Masculino	de 26 a 35 anos
	Estágio 1 (pele íntegra com						Leve	Feminino	de 46 a 55 anos
	Estágio 1 (pele íntegra com						Leve	Feminino	de 36 a 45 anos
	Estágio 2 (perda parcial da espessura						Leve	Masculino	de 36 a 45 anos
	Estágio 3 (perda total da espessura						Moderado	Masculino	de 46 a 55 anos
	Estágio 2 (perda parcial da espessura						Leve	Masculino	de 26 a 35 anos
	Estágio 2 (perda parcial da espessura						Leve	Masculino	de 66 a 75 anos
	Estágio 1 (pele íntegra com						Leve	Masculino	de 66 a 75 anos
	Estágio 2 (perda parcial da espessura						Leve	Feminino	de 46 a 55 anos
	Estágio 2 (perda parcial da espessura						Leve	Feminino	de 66 a 75 anos
	Estágio 2 (perda parcial da espessura						Leve	Masculino	de 26 a 35 anos

	Estágio 1 (pele íntegra com						Leve	Feminino	de 66 a 75 anos
	Estágio 2 (perda parcial da espessura						Leve	Feminino	de 36 a 45 anos
	Estágio 1 (pele íntegra com						Leve	Masculino	de 46 a 55 anos
	Estágio 2 (perda parcial da espessura						Leve	Feminino	de 36 a 45 anos
	Estágio 1 (pele íntegra com						Leve	Feminino	de 56 a 65 anos
	Estágio 3 (perda total da espessura						Moderado	Feminino	de 56 a 65 anos
	Estágio 1 (pele íntegra com						Leve	Masculino	de 66 a 75 anos
	Estágio 2 (perda parcial da espessura						Leve	Masculino	de 36 a 45 anos
	Estágio 2 (perda parcial da espessura						Leve	Masculino	de 46 a 55 anos
	Estágio 2 (perda parcial da espessura						Leve	Feminino	de 56 a 65 anos
	Estágio 2 (perda parcial da espessura						Leve	Feminino	de 66 a 75 anos
	Estágio 1 (pele íntegra com						Leve	Feminino	de 56 a 65 anos
	Estágio 2 (perda parcial da espessura						Leve	Feminino	de 56 a 65 anos
	Estágio 1 (pele íntegra com						Leve	Feminino	mais de 85 anos
	Estágio 2 (perda parcial da espessura						Leve	Feminino	de 46 a 55 anos

	Estágio 2 (perda parcial da espessura						Leve	Feminino	de 66 a 75 anos
	Estágio 3 (perda total da espessura						Leve	Masculino	de 66 a 75 anos
	Estágio 2 (perda parcial da espessura						Leve	Masculino	de 26 a 35 anos
	Estágio 2 (perda parcial da espessura						Leve	Masculino	de 26 a 35 anos
	Estágio 3 (perda total da espessura						Leve	Masculino	de 66 a 75 anos
	Estágio 2 (perda parcial da espessura						Leve	Masculino	de 56 a 65 anos
	Estágio 2 (perda parcial da espessura						Leve	Feminino	de 36 a 45 anos
	Estágio 1 (pele íntegra com						Leve	Feminino	de 36 a 45 anos
	Estágio 1 (pele íntegra com						Leve	Feminino	de 46 a 55 anos
	Estágio 2 (perda parcial da espessura						Leve	Feminino	de 66 a 75 anos
	Estágio 2 (perda parcial da espessura						Leve	Masculino	de 56 a 65 anos
	Estágio 2 (perda parcial da espessura						Leve	Feminino	de 66 a 75 anos
	Estágio 2 (perda parcial da espessura						Leve	Masculino	de 56 a 65 anos
	Estágio 3 (perda total da espessura						Moderado	Masculino	de 26 a 35 anos
	Estágio 3 (perda total da espessura						Moderado	Masculino	de 56 a 65 anos

	Estágio 2 (perda parcial da espessura						Leve	Masculino	de 66 a 75 anos
	Estágio 2 (perda parcial da espessura						Leve	Masculino	de 66 a 75 anos
	Estágio 3 (perda total da espessura						Moderado	Masculino	de 76 a 85 anos
	Estágio 3 (perda total da espessura						Moderado	Masculino	de 36 a 45 anos
	Estágio 1 (pele íntegra com						Leve	Masculino	mais de 85 anos
	Estágio 2 (perda parcial da espessura						Leve	Masculino	mais de 85 anos
	Estágio 3 (perda total da espessura						Leve	Masculino	de 66 a 75 anos
	Estágio 2 (perda parcial da espessura						Leve	Masculino	de 46 a 55 anos
	Estágio 1 (pele íntegra com						Leve	Masculino	de 66 a 75 anos
	Estágio 2 (perda parcial da espessura						Leve	Masculino	de 56 a 65 anos
	Estágio 2 (perda parcial da espessura						Leve	Feminino	de 66 a 75 anos
	Estágio 3 (perda total da espessura						Moderado	Feminino	de 18 a 25 anos
	Estágio 2 (perda parcial da espessura						Leve	Feminino	de 46 a 55 anos
	Estágio 3 (perda total da espessura						Leve	Feminino	de 56 a 65 anos
	Estágio 2 (perda parcial da espessura						Leve	Feminino	de 66 a 75 anos

	Estagio 1 (pele íntegra com						Leve	Masculino	de 36 a 45 anos
	Estagio 1 (pele íntegra com						Leve	Feminino	mais de 85 anos
	Estagio 1 (pele íntegra com						Leve	Masculino	de 56 a 65 anos
	Estagio 3 (perda total da espessura						Moderado	Feminino	de 46 a 55 anos
	Estagio 2 (perda parcial da espessura						Leve	Feminino	de 66 a 75 anos
	Estagio 2 (perda parcial da espessura						Leve	Masculino	de 56 a 65 anos
	Estagio 2 (perda parcial da espessura						Leve	Masculino	de 36 a 45 anos
	Estagio 2 (perda parcial da espessura						Leve	Masculino	de 56 a 65 anos
	Estagio 2 (perda parcial da espessura						Leve	Feminino	de 36 a 45 anos
	Estagio 2 (perda parcial da espessura						Leve	Masculino	de 66 a 75 anos
	Estagio 1 (pele íntegra com						Leve	Masculino	de 66 a 75 anos
	Estagio 2 (perda parcial da espessura						Leve	Masculino	de 46 a 55 anos
	Estagio 3 (perda total da espessura						Moderado	Feminino	de 36 a 45 anos
	Estagio 1 (pele íntegra com						Leve	Feminino	de 76 a 85 anos
	Estagio 2 (perda parcial da espessura						Leve	Masculino	mais de 85 anos

	Estagio 2 (perda parcial da espessura						Leve	Masculino	mais de 85 anos
	Estagio 3 (perda total da espessura						Moderado	Masculino	mais de 85 anos
	Estagio 2 (perda parcial da espessura						Leve	Feminino	de 66 a 75 anos
	Estagio 2 (perda parcial da espessura						Leve	Feminino	de 56 a 65 anos
	Estagio 2 (perda parcial da espessura						Leve	Masculino	de 36 a 45 anos
	Estagio 2 (perda parcial da espessura						Leve	Masculino	de 36 a 45 anos
	Estagio 2 (perda parcial da espessura						Leve	Masculino	de 66 a 75 anos
	Estagio 1 (pele íntegra com						Leve	Masculino	de 56 a 65 anos
	Estagio 1 (pele íntegra com						Leve	Feminino	de 66 a 75 anos
	Estagio 2 (perda parcial da espessura						Leve	Feminino	de 26 a 35 anos
	Estagio 2 (perda parcial da espessura						Leve	Feminino	de 46 a 55 anos
	Estagio 1 (pele íntegra com						Leve	Feminino	de 46 a 55 anos
	Estagio 2 (perda parcial da espessura						Leve	Feminino	de 26 a 35 anos
	Estagio 2 (perda parcial da espessura						Leve	Feminino	de 56 a 65 anos
	Estagio 2 (perda parcial da espessura						Leve	Feminino	de 56 a 65 anos

	Estágio 2 (perda parcial da espessura						Leve	Masculino	de 76 a 85 anos
	Estágio 1 (pele íntegra com						Leve	Masculino	de 76 a 85 anos
	Estágio 2 (perda parcial da espessura						Leve	Masculino	de 46 a 55 anos
	Estágio 2 (perda parcial da espessura						Leve	Feminino	de 26 a 35 anos
	Estágio 2 (perda parcial da espessura						Leve	Feminino	de 76 a 85 anos
	Estágio 1 (pele íntegra com						Leve	Feminino	de 46 a 55 anos
	Estágio 2 (perda parcial da espessura						Leve	Masculino	de 56 a 65 anos
	Estágio 2 (perda parcial da espessura						Leve	Masculino	de 56 a 65 anos
	Estágio 1 (pele íntegra com						Leve	Feminino	de 56 a 65 anos
	Estágio 4 (perda total da espessura						Moderado	Feminino	de 56 a 65 anos
	Estágio 1 (pele íntegra com						Leve	Feminino	de 76 a 85 anos
	Estágio 1 (pele íntegra com						Leve	Feminino	de 46 a 55 anos
	Estágio 3 (perda total da espessura						Moderado	Feminino	de 26 a 35 anos
	Estágio 2 (perda parcial da espessura						Leve	Feminino	de 36 a 45 anos
	Estágio 2 (perda parcial da espessura						Leve	Masculino	de 66 a 75 anos

	Estágio 1 (pele íntegra com						Leve	Masculino	de 56 a 65 anos
	Estágio 1 (pele íntegra com						Leve	Feminino	de 56 a 65 anos
	Estágio 2 (perda parcial da espessura						Leve	Feminino	de 56 a 65 anos
	Estágio 2 (perda parcial da espessura						Leve	Feminino	de 66 a 75 anos
	Estágio 2 (perda parcial da espessura						Leve	Masculino	de 56 a 65 anos
	Estágio 1 (pele íntegra com						Leve	Masculino	de 46 a 55 anos
	Estágio 1 (pele íntegra com						Leve	Masculino	de 26 a 35 anos
	Estágio 2 (perda parcial da espessura						Leve	Feminino	de 66 a 75 anos
	Estágio 3 (perda total da espessura						Moderado	Feminino	de 46 a 55 anos
	Estágio 2 (perda parcial da espessura						Leve	Masculino	de 56 a 65 anos
	Estágio 1 (pele íntegra com						Leve	Masculino	de 66 a 75 anos
	Estágio 1 (pele íntegra com						Leve	Masculino	de 56 a 65 anos
	Estágio 1 (pele íntegra com						Leve	Masculino	de 46 a 55 anos
	Estágio 4 (perda total da espessura						Moderado	Masculino	de 76 a 85 anos
	Estágio 1 (pele íntegra com						Leve	Masculino	de 66 a 75 anos

	Estágio 2 (perda parcial da espessura						Leve	Masculino	de 46 a 55 anos
	Estágio 1 (pele íntegra com						Leve	Masculino	de 46 a 55 anos
	Estágio 2 (perda parcial da espessura						Leve	Masculino	de 56 a 65 anos
	Estágio 2 (perda parcial da espessura						Leve	Masculino	de 66 a 75 anos
	Estágio 2 (perda parcial da espessura						Leve	Masculino	de 76 a 85 anos
	Estágio 1 (pele íntegra com						Leve	Masculino	de 56 a 65 anos
	Estágio 2 (perda parcial da espessura						Leve	Masculino	de 76 a 85 anos
	Estágio 2 (perda parcial da espessura						Leve	Feminino	de 56 a 65 anos
	Estágio 2 (perda parcial da espessura						Leve	Masculino	de 66 a 75 anos
	Estágio 1 (pele íntegra com						Leve	Masculino	de 56 a 65 anos
	Estágio 2 (perda parcial da espessura						Leve	Feminino	de 76 a 85 anos
	Estágio 2 (perda parcial da espessura						Leve	Feminino	de 56 a 65 anos
	Estágio 2 (perda parcial da espessura						Leve	Feminino	de 56 a 65 anos
	Estágio 1 (pele íntegra com						Leve	Feminino	de 46 a 55 anos
	Estágio 1 (pele íntegra com						Leve	Feminino	de 46 a 55 anos

	Estágio 3 (perda total da espessura						Moderado	Feminino	de 56 a 65 anos
	Estágio 2 (perda parcial da espessura						Leve	Feminino	de 66 a 75 anos
	Estágio 4 (perda total da espessura						Moderado	Feminino	de 36 a 45 anos
	Estágio 2 (perda parcial da espessura						Leve	Feminino	de 66 a 75 anos
	Estágio 1 (pele íntegra com						Leve	Feminino	de 36 a 45 anos
	Estágio 3 (perda total da espessura						Moderado	Feminino	de 56 a 65 anos
	Estágio 1 (pele íntegra com						Leve	Feminino	de 76 a 85 anos
	Estágio 4 (perda total da espessura						Moderado	Feminino	de 76 a 85 anos
	Estágio 1 (pele íntegra com						Leve	Masculino	de 76 a 85 anos
	Estágio 2 (perda parcial da espessura						Leve	Feminino	de 76 a 85 anos
	Estágio 2 (perda parcial da espessura						Leve	Feminino	de 26 a 35 anos
	Estágio 1 (pele íntegra com						Leve	Masculino	de 56 a 65 anos
	Estágio 2 (perda parcial da espessura						Leve	Masculino	de 56 a 65 anos
	Estágio 1 (pele íntegra com						Leve	Masculino	de 66 a 75 anos
	Estágio 2 (perda parcial da espessura						Leve	Feminino	de 56 a 65 anos

	Estágio 2 (perda parcial da espessura						Leve	Feminino	de 46 a 55 anos
	Estágio 2 (perda parcial da espessura						Leve	Masculino	de 56 a 65 anos
	Estágio 1 (pele íntegra com						Leve	Masculino	de 46 a 55 anos
	Estágio 2 (perda parcial da espessura						Leve	Masculino	de 66 a 75 anos
	Estágio 1 (pele íntegra com						Leve	Feminino	de 46 a 55 anos
	Estágio 2 (perda parcial da espessura						Leve	Feminino	de 46 a 55 anos
	Estágio 2 (perda parcial da espessura						Leve	Feminino	de 56 a 65 anos
	Estágio 1 (pele íntegra com						Leve	Feminino	de 56 a 65 anos
	Estágio 4 (perda total da espessura						Moderado	Masculino	de 56 a 65 anos
	Estágio 2 (perda parcial da espessura						Leve	Masculino	de 56 a 65 anos
	Estágio 2 (perda parcial da espessura						Leve	Masculino	de 36 a 45 anos
	Estágio 2 (perda parcial da espessura						Leve	Masculino	de 66 a 75 anos
	Estágio 2 (perda parcial da espessura						Leve	Masculino	mais de 85 anos
	Estágio 1 (pele íntegra com						Leve	Masculino	de 56 a 65 anos
	Estágio 2 (perda parcial da espessura						Leve	Feminino	de 56 a 65 anos

	Estágio 1 (pele íntegra com						Leve	Masculino	de 56 a 65 anos
	Estágio 2 (perda parcial da espessura						Leve	Feminino	de 76 a 85 anos
	Estágio 1 (pele íntegra com						Leve	Masculino	de 46 a 55 anos
	Estágio 2 (perda parcial da espessura						Leve	Masculino	de 36 a 45 anos
	Estágio 1 (pele íntegra com						Leve	Masculino	de 66 a 75 anos
	Estágio 1 (pele íntegra com						Leve	Feminino	de 66 a 75 anos
	Estágio 2 (perda parcial da espessura						Leve	Feminino	de 56 a 65 anos
	Estágio 2 (perda parcial da espessura						Leve	Feminino	de 66 a 75 anos
	Estágio 3 (perda total da espessura						Moderado	Feminino	de 76 a 85 anos
	Estágio 2 (perda parcial da espessura						Leve	Masculino	de 56 a 65 anos
	Estágio 2 (perda parcial da espessura						Leve	Masculino	de 66 a 75 anos
	Estágio 1 (pele íntegra com						Leve	Masculino	de 56 a 65 anos
	Estágio 1 (pele íntegra com						Leve	Masculino	de 66 a 75 anos
	Estágio 2 (perda parcial da espessura						Leve	Masculino	de 66 a 75 anos
	Estágio 1 (pele íntegra com						Leve	Masculino	de 46 a 55 anos

	Estágio 2 (perda parcial da espessura						Leve	Masculino	de 26 a 35 anos
	Estágio 2 (perda parcial da espessura						Leve	Masculino	de 76 a 85 anos
	Estágio 2 (perda parcial da espessura						Leve	Masculino	de 66 a 75 anos
	Estágio 1 (pele íntegra com						Leve	Masculino	de 76 a 85 anos
	Estágio 2 (perda parcial da espessura						Leve	Masculino	de 46 a 55 anos
	Estágio 1 (pele íntegra com						Leve	Masculino	de 46 a 55 anos
	Estágio 2 (perda parcial da espessura						Leve	Masculino	de 46 a 55 anos
	Estágio 3 (perda total da espessura						Moderado	Masculino	de 26 a 35 anos
	Estágio 1 (pele íntegra com						Leve	Masculino	de 66 a 75 anos
	Estágio 2 (perda parcial da espessura						Leve	Feminino	de 26 a 35 anos
	Estágio 2 (perda parcial da espessura						Leve	Feminino	de 36 a 45 anos
	Estágio 2 (perda parcial da espessura						Leve	Masculino	de 66 a 75 anos
	Estágio 3 (perda total da espessura						Moderado	Masculino	de 26 a 35 anos
	Estágio 2 (perda parcial da espessura						Leve	Masculino	de 36 a 45 anos
	Estágio 2 (perda parcial da espessura						Leve	Masculino	de 36 a 45 anos

	Estágio 2 (perda parcial da espessura						Leve	Masculino	de 76 a 85 anos
	Estágio 1 (pele íntegra com						Leve	Masculino	de 76 a 85 anos
	Estágio 2 (perda parcial da espessura						Leve	Masculino	de 46 a 55 anos
	Estágio 1 (pele íntegra com						Leve	Masculino	de 46 a 55 anos
	Estágio 2 (perda parcial da espessura						Leve	Masculino	de 56 a 65 anos
	Estágio 2 (perda parcial da espessura						Leve	Masculino	de 56 a 65 anos
	Estágio 2 (perda parcial da espessura						Leve	Feminino	de 56 a 65 anos
	Estágio 2 (perda parcial da espessura						Leve	Feminino	de 56 a 65 anos
	Estágio 2 (perda parcial da espessura						Leve	Feminino	de 56 a 65 anos
	Estágio 2 (perda parcial da espessura						Leve	Masculino	de 66 a 75 anos
	Estágio 4 (perda total da espessura						Moderado	Feminino	de 36 a 45 anos
	Estágio 1 (pele íntegra com						Leve	Masculino	de 66 a 75 anos
	Estágio 2 (perda parcial da espessura						Leve	Feminino	de 56 a 65 anos
	Estágio 2 (perda parcial da espessura						Leve	Feminino	de 66 a 75 anos
	Estágio 1 (pele íntegra com						Leve	Feminino	de 36 a 45 anos

	Estágio 1 (pele íntegra com						Leve	Feminino	mais de 85 anos
	Estágio 1 (pele íntegra com						Leve	Feminino	de 76 a 85 anos
	Estágio 1 (pele íntegra com						Leve	Feminino	mais de 85 anos
	Estágio 2 (perda parcial da espessura						Leve	Feminino	de 66 a 75 anos
	Estágio 2 (perda parcial da espessura						Leve	Feminino	de 46 a 55 anos
	Estágio 1 (pele íntegra com						Leve	Feminino	de 76 a 85 anos
	Estágio 2 (perda parcial da espessura						Leve	Feminino	de 56 a 65 anos
	Estágio 2 (perda parcial da espessura						Leve	Feminino	de 56 a 65 anos
	Estágio 1 (pele íntegra com						Leve	Feminino	de 76 a 85 anos
	Estágio 1 (pele íntegra com						Leve	Masculino	de 76 a 85 anos
	Estágio 1 (pele íntegra com						Leve	Masculino	de 66 a 75 anos
	Estágio 2 (perda parcial da espessura						Leve	Masculino	de 36 a 45 anos
	Estágio 2 (perda parcial da espessura						Leve	Feminino	de 66 a 75 anos
	Estágio 2 (perda parcial da espessura						Leve	Masculino	de 56 a 65 anos
	Estágio 2 (perda parcial da espessura						Leve	Masculino	mais de 85 anos

	Estágio 1 (pele íntegra com						Leve	Masculino	mais de 85 anos
	Estágio 1 (pele íntegra com						Leve	Feminino	de 66 a 75 anos
	Estágio 2 (perda parcial da espessura						Leve	Feminino	de 66 a 75 anos
	Estágio 2 (perda parcial da espessura						Leve	Masculino	de 36 a 45 anos
	Estágio 2 (perda parcial da espessura						Leve	Feminino	de 46 a 55 anos
	Estágio 2 (perda parcial da espessura						Leve	Masculino	mais de 85 anos
	Estágio 1 (pele íntegra com						Leve	Masculino	de 56 a 65 anos
	Estágio 2 (perda parcial da espessura						Leve	Feminino	de 76 a 85 anos
	Estágio 2 (perda parcial da espessura						Leve	Feminino	de 56 a 65 anos
	Estágio 2 (perda parcial da espessura						Leve	Masculino	de 46 a 55 anos
	Estágio 2 (perda parcial da espessura						Leve	Masculino	de 76 a 85 anos
	Estágio 2 (perda parcial da espessura						Leve	Feminino	de 66 a 75 anos
							Grave	Masculino	de 76 a 85 anos
							Moderado	Masculino	de 56 a 65 anos
							Moderado	Masculino	de 56 a 65 anos

							Leve	Feminino	de 76 a 85 anos
							Leve	Masculino	de 56 a 65 anos
							Leve	Feminino	de 66 a 75 anos
							Nenhum	Masculino	de 36 a 45 anos
	Estágio 1 (pele íntegra com						Leve	Masculino	de 46 a 55 anos
	Estágio 2 (perda parcial da espessura						Leve	Masculino	de 76 a 85 anos
	Estágio 2 (perda parcial da espessura						Leve	Feminino	de 76 a 85 anos
	Estágio 1 (pele íntegra com						Leve	Feminino	de 26 a 35 anos
	Estágio 2 (perda parcial da espessura						Leve	Feminino	de 36 a 45 anos
	Estágio 2 (perda parcial da espessura						Leve	Feminino	de 66 a 75 anos
	Estágio 2 (perda parcial da espessura						Leve	Feminino	de 46 a 55 anos
	Estágio 2 (perda parcial da espessura						Leve	Masculino	de 36 a 45 anos
	Estágio 2 (perda parcial da espessura						Leve	Masculino	de 76 a 85 anos
	Estágio 2 (perda parcial da espessura						Leve	Masculino	de 56 a 65 anos
	Estágio 4 (perda total da espessura						Moderado	Masculino	de 76 a 85 anos

	Estágio 2 (perda parcial da espessura						Leve	Masculino	de 56 a 65 anos
	Estágio 1 (pele íntegra com						Leve	Masculino	de 66 a 75 anos
	Estágio 2 (perda parcial da espessura						Leve	Masculino	de 76 a 85 anos
	Estágio 2 (perda parcial da espessura						Leve	Feminino	de 76 a 85 anos
	Estágio 2 (perda parcial da espessura						Leve	Feminino	de 46 a 55 anos
	Estágio 2 (perda parcial da espessura						Leve	Masculino	de 56 a 65 anos
	Estágio 2 (perda parcial da espessura						Leve	Masculino	de 56 a 65 anos
	Estágio 2 (perda parcial da espessura						Leve	Feminino	de 56 a 65 anos
	Estágio 2 (perda parcial da espessura						Leve	Masculino	de 46 a 55 anos
	Estágio 2 (perda parcial da espessura						Leve	Masculino	de 56 a 65 anos
	Estágio 2 (perda parcial da espessura						Leve	Masculino	de 66 a 75 anos
	Estágio 2 (perda parcial da espessura						Leve	Masculino	de 66 a 75 anos
	Estágio 1 (pele íntegra com						Leve	Masculino	de 66 a 75 anos
	Estágio 2 (perda parcial da espessura						Leve	Masculino	de 66 a 75 anos
	Estágio 1 (pele íntegra com						Leve	Masculino	de 56 a 65 anos

	Estágio 2 (perda parcial da espessura						Leve	Masculino	de 46 a 55 anos
	Estágio 2 (perda parcial da espessura						Leve	Masculino	de 66 a 75 anos
	Estágio 2 (perda parcial da espessura						Leve	Masculino	de 46 a 55 anos
	Estágio 2 (perda parcial da espessura						Leve	Masculino	de 56 a 65 anos
	Estágio 2 (perda parcial da espessura						Leve	Masculino	de 56 a 65 anos
	Estágio 2 (perda parcial da espessura						Leve	Masculino	de 76 a 85 anos
	Estágio 1 (pele íntegra com						Leve	Masculino	de 76 a 85 anos
	Estágio 2 (perda parcial da espessura						Leve	Feminino	de 66 a 75 anos
	Estágio 1 (pele íntegra com						Leve	Masculino	de 66 a 75 anos
	Estágio 2 (perda parcial da espessura						Leve	Masculino	de 56 a 65 anos
	Estágio 2 (perda parcial da espessura						Leve	Masculino	de 76 a 85 anos
	Estágio 1 (pele íntegra com						Leve	Masculino	mais de 85 anos
	Estágio 2 (perda parcial da espessura						Leve	Masculino	de 66 a 75 anos
	Estágio 2 (perda parcial da espessura						Leve	Feminino	de 36 a 45 anos
	Estágio 1 (pele íntegra com						Leve	Masculino	de 66 a 75 anos

	Estágio 1 (pele íntegra com						Leve	Masculino	de 66 a 75 anos
	Estágio 2 (perda parcial da espessura						Leve	Feminino	de 66 a 75 anos
	Estágio 2 (perda parcial da espessura						Leve	Masculino	de 76 a 85 anos
	Estágio 3 (perda total da espessura						Moderado	Feminino	de 36 a 45 anos
	Estágio 2 (perda parcial da espessura						Leve	Feminino	de 36 a 45 anos
	Estágio 1 (pele íntegra com						Leve	Feminino	de 76 a 85 anos
	Estágio 2 (perda parcial da espessura						Leve	Feminino	de 76 a 85 anos
	Estágio 2 (perda parcial da espessura						Leve	Feminino	de 66 a 75 anos
	Estágio 2 (perda parcial da espessura						Leve	Feminino	de 66 a 75 anos
	Estágio 2 (perda parcial da espessura						Leve	Feminino	de 76 a 85 anos
	Estágio 2 (perda parcial da espessura						Leve	Feminino	de 66 a 75 anos
	Estágio 2 (perda parcial da espessura						Leve	Feminino	de 66 a 75 anos
	Estágio 2 (perda parcial da espessura						Leve	Feminino	de 66 a 75 anos
	Estágio 1 (pele íntegra com						Leve	Feminino	de 46 a 55 anos
	Estágio 2 (perda parcial da espessura						Leve	Feminino	de 56 a 65 anos

	Estágio 1 (pele íntegra com						Leve	Feminino	de 56 a 65 anos
	Estágio 1 (pele íntegra com						Leve	Masculino	de 36 a 45 anos
	Estágio 2 (perda parcial da espessura						Leve	Feminino	de 46 a 55 anos
	Estágio 1 (pele íntegra com						Leve	Feminino	de 56 a 65 anos
	Estágio 4 (perda total da espessura						Moderado	Masculino	de 66 a 75 anos
	Estágio 1 (pele íntegra com						Leve	Feminino	mais de 85 anos
	Estágio 1 (pele íntegra com						Leve	Masculino	de 26 a 35 anos
	Estágio 2 (perda parcial da espessura						Leve	Masculino	de 46 a 55 anos
	Estágio 3 (perda total da espessura						Moderado	Feminino	de 66 a 75 anos
							Leve	Feminino	de 46 a 55 anos
							Leve	Masculino	de 66 a 75 anos
							Moderado	Feminino	de 76 a 85 anos
							Leve	Masculino	de 56 a 65 anos
							Leve	Masculino	de 66 a 75 anos
							Nenhum	Masculino	de 66 a 75 anos

							Nenhum	Masculino	de 66 a 75 anos
							Leve	Masculino	de 26 a 35 anos
							Nenhum	Masculino	menos de 28 dias
							Leve	Masculino	menos de 28 dias
							Leve	Masculino	de 76 a 85 anos
							Moderado	Masculino	de 26 a 35 anos
							Moderado	Feminino	de 36 a 45 anos
							Moderado	Masculino	de 36 a 45 anos
							Leve	Feminino	de 26 a 35 anos
							Leve	Feminino	de 18 a 25 anos
							Leve	Feminino	de 36 a 45 anos
							Leve	Feminino	de 76 a 85 anos
							Leve	Feminino	de 66 a 75 anos
							Leve	Feminino	de 56 a 65 anos
							Leve	Feminino	de 36 a 45 anos

							Leve	Feminino	de 36 a 45 anos
							Leve	Feminino	de 66 a 75 anos
							Leve	Feminino	de 66 a 75 anos
							Leve	Feminino	de 56 a 65 anos
							Nenhum	Feminino	de 26 a 35 anos
							Leve	Masculino	de 56 a 65 anos
							Leve	Feminino	de 29 dias a 1 ano
							Leve	Feminino	de 66 a 75 anos
							Leve	Masculino	de 36 a 45 anos
							Leve	Masculino	de 76 a 85 anos
							Leve	Feminino	de 26 a 35 anos
							Leve	Feminino	de 26 a 35 anos
							Leve	Feminino	de 66 a 75 anos
							Leve	Feminino	de 56 a 65 anos
	Estágio 2 (perda parcial da espessura						Leve	Feminino	de 56 a 65 anos

							Leve	Masculino	de 46 a 55 anos
	Estágio 2 (perda parcial da espessura						Leve	Masculino	de 56 a 65 anos
							Leve	Masculino	de 18 a 25 anos
	Estágio 2 (perda parcial da espessura						Leve	Masculino	de 76 a 85 anos
	Estágio 2 (perda parcial da espessura						Leve	Feminino	de 76 a 85 anos
							Leve	Masculino	de 18 a 25 anos
	Estágio 2 (perda parcial da espessura						Leve	Feminino	de 66 a 75 anos
	Estágio 2 (perda parcial da espessura						Leve	Masculino	de 36 a 45 anos
	Estágio 2 (perda parcial da espessura						Leve	Masculino	de 36 a 45 anos
							Moderado	Feminino	de 76 a 85 anos
	Estágio 2 (perda parcial da espessura						Leve	Masculino	de 36 a 45 anos
	Estágio 2 (perda parcial da espessura						Leve	Feminino	de 66 a 75 anos
							Leve	Feminino	de 66 a 75 anos
	Estágio 2 (perda parcial da espessura						Leve	Feminino	de 56 a 65 anos
	Estágio 2 (perda parcial da espessura						Leve	Masculino	de 56 a 65 anos

	Estágio 2 (perda parcial da espessura						Leve	Masculino	de 56 a 65 anos
	Estágio 1 (pele íntegra com						Leve	Masculino	de 56 a 65 anos
							Leve	Masculino	de 56 a 65 anos
	Estágio 2 (perda parcial da espessura						Leve	Masculino	de 56 a 65 anos
	Estágio 2 (perda parcial da espessura						Leve	Feminino	de 36 a 45 anos
							Leve	Feminino	de 56 a 65 anos
	Estágio 2 (perda parcial da espessura						Leve	Feminino	de 56 a 65 anos
	Estágio 2 (perda parcial da espessura						Leve	Masculino	de 56 a 65 anos
							Moderado	Masculino	de 66 a 75 anos
	Estágio 1 (pele íntegra com						Leve	Masculino	de 26 a 35 anos
	Estágio 2 (perda parcial da espessura						Leve	Feminino	mais de 85 anos
	Estágio 2 (perda parcial da espessura						Leve	Feminino	de 66 a 75 anos
	Estágio 3 (perda total da espessura						Moderado	Masculino	mais de 85 anos
							Leve	Feminino	de 56 a 65 anos
							Leve	Feminino	de 56 a 65 anos

							Moderado	Masculino	de 56 a 65 anos
							Leve	Feminino	de 56 a 65 anos
							Nenhum	Feminino	de 26 a 35 anos
							Leve	Feminino	mais de 85 anos
							Leve	Feminino	de 18 a 25 anos
	Estágio 2 (perda parcial da espessura						Leve	Feminino	de 76 a 85 anos
							Leve	Masculino	de 56 a 65 anos
	Estágio 2 (perda parcial da espessura						Leve	Feminino	de 56 a 65 anos
	Estágio 2 (perda parcial da espessura						Leve	Masculino	de 56 a 65 anos
							Leve	Masculino	de 56 a 65 anos
	Estágio 2 (perda parcial da espessura						Leve	Feminino	de 56 a 65 anos
	Estágio 3 (perda total da espessura						Moderado	Feminino	de 56 a 65 anos
	Estágio 2 (perda parcial da espessura						Leve	Masculino	de 56 a 65 anos
							Nenhum	Feminino	de 66 a 75 anos
	Estágio 2 (perda parcial da espessura						Leve	Masculino	de 46 a 55 anos

	Estágio 2 (perda parcial da espessura						Leve	Masculino	de 46 a 55 anos
	Estágio 2 (perda parcial da espessura						Leve	Feminino	de 76 a 85 anos
	Estágio 2 (perda parcial da espessura						Leve	Feminino	de 26 a 35 anos
	Estágio 1 (pele íntegra com						Leve	Masculino	de 46 a 55 anos
	Estágio 2 (perda parcial da espessura						Leve	Masculino	de 46 a 55 anos
	Estágio 2 (perda parcial da espessura						Leve	Masculino	de 46 a 55 anos
	Estágio 1 (pele íntegra com						Leve	Feminino	de 66 a 75 anos
	Estágio 2 (perda parcial da espessura						Leve	Feminino	de 36 a 45 anos
							Leve	Masculino	de 46 a 55 anos
							Leve	Feminino	de 46 a 55 anos
							Leve	Masculino	de 56 a 65 anos
	Estágio 2 (perda parcial da espessura						Leve	Masculino	de 66 a 75 anos
	Estágio 2 (perda parcial da espessura						Leve	Masculino	de 66 a 75 anos
	Estágio 2 (perda parcial da espessura						Leve	Masculino	de 76 a 85 anos
	Estágio 2 (perda parcial da espessura						Leve	Masculino	de 76 a 85 anos

	Estágio 2 (perda parcial da espessura						Leve	Masculino	de 76 a 85 anos
	Estágio 2 (perda parcial da espessura						Leve	Masculino	de 46 a 55 anos
	Estágio 2 (perda parcial da espessura						Leve	Feminino	de 36 a 45 anos
	Estágio 2 (perda parcial da espessura						Leve	Feminino	de 36 a 45 anos
							Leve	Feminino	de 46 a 55 anos
	Estágio 2 (perda parcial da espessura						Leve	Masculino	de 26 a 35 anos
	Estágio 2 (perda parcial da espessura						Leve	Feminino	de 56 a 65 anos
	Estágio 1 (pele íntegra com						Leve	Feminino	de 56 a 65 anos
	Estágio 2 (perda parcial da espessura						Leve	Feminino	de 56 a 65 anos
	Estágio 1 (pele íntegra com						Leve	Masculino	de 56 a 65 anos
	Estágio 1 (pele íntegra com						Leve	Masculino	de 66 a 75 anos
	Estágio 2 (perda parcial da espessura						Leve	Feminino	de 76 a 85 anos
	Estágio 1 (pele íntegra com						Leve	Feminino	de 66 a 75 anos
	Estágio 1 (pele íntegra com						Leve	Masculino	de 66 a 75 anos
	Estágio 1 (pele íntegra com						Leve	Feminino	de 36 a 45 anos

	Estagio 1 (pele íntegra com						Leve	Masculino	de 66 a 75 anos
	Estagio 2 (perda parcial da espessura						Leve	Feminino	de 66 a 75 anos
							Leve	Masculino	de 66 a 75 anos
	Estagio 2 (perda parcial da espessura						Leve	Masculino	de 46 a 55 anos
	Estagio 1 (pele íntegra com						Leve	Feminino	de 76 a 85 anos
	Estagio 2 (perda parcial da espessura						Leve	Feminino	de 66 a 75 anos
							Leve	Masculino	de 76 a 85 anos
	Estagio 2 (perda parcial da espessura						Leve	Masculino	de 36 a 45 anos
	Estagio 2 (perda parcial da espessura						Leve	Feminino	de 46 a 55 anos
	Estagio 1 (pele íntegra com						Leve	Feminino	de 76 a 85 anos
	Estagio 2 (perda parcial da espessura						Leve	Feminino	de 36 a 45 anos
	Estagio 2 (perda parcial da espessura						Leve	Feminino	de 46 a 55 anos
	Estagio 2 (perda parcial da espessura						Leve	Feminino	de 66 a 75 anos
	Estagio 2 (perda parcial da espessura						Leve	Feminino	de 66 a 75 anos
	Estagio 2 (perda parcial da espessura						Leve	Feminino	de 26 a 35 anos

	Estágio 2 (perda parcial da espessura						Leve	Feminino	de 36 a 45 anos
	Estágio 2 (perda parcial da espessura						Leve	Feminino	de 18 a 25 anos
	Estágio 2 (perda parcial da espessura						Leve	Masculino	de 46 a 55 anos
	Estágio 1 (pele íntegra com						Leve	Masculino	de 46 a 55 anos
	Estágio 1 (pele íntegra com						Leve	Masculino	de 46 a 55 anos
	Estágio 2 (perda parcial da espessura						Leve	Masculino	de 76 a 85 anos
	Estágio 2 (perda parcial da espessura						Leve	Masculino	de 76 a 85 anos
	Estágio 2 (perda parcial da espessura						Leve	Feminino	de 56 a 65 anos
							Leve	Masculino	de 36 a 45 anos
							Leve	Feminino	de 56 a 65 anos
							Leve	Feminino	de 18 a 25 anos
							Leve	Feminino	de 56 a 65 anos
							Leve	Feminino	de 18 a 25 anos
							Leve	Masculino	de 56 a 65 anos
							Leve	Masculino	de 56 a 65 anos

							Leve	Feminino	de 56 a 65 anos
							Moderado	Masculino	de 56 a 65 anos
							Moderado	Masculino	de 56 a 65 anos
							Leve	Masculino	de 56 a 65 anos
							Nenhum	Feminino	de 36 a 45 anos
							Moderado	Masculino	de 66 a 75 anos
							Leve	Feminino	de 26 a 35 anos
							Leve	Feminino	de 66 a 75 anos
							Leve	Masculino	de 2 a 4 anos
							Leve	Masculino	de 56 a 65 anos
							Leve	Masculino	de 26 a 35 anos
	Estagio 2 (perda parcial da espessura						Leve	Feminino	de 66 a 75 anos
							Leve	Feminino	de 56 a 65 anos
							Leve	Feminino	de 66 a 75 anos
							Leve	Feminino	de 36 a 45 anos

							Leve	Masculino	de 46 a 55 anos
							Leve	Feminino	de 46 a 55 anos
							Leve	Masculino	de 26 a 35 anos
	Estágio 2 (perda parcial da espessura						Leve	Masculino	de 26 a 35 anos
							Moderado	Feminino	de 46 a 55 anos
							Moderado	Masculino	de 66 a 75 anos
							Moderado	Feminino	de 66 a 75 anos
							Moderado	Masculino	de 46 a 55 anos
	Estágio 1 (pele íntegra com						Leve	Masculino	de 66 a 75 anos
	Estágio 2 (perda parcial da espessura						Leve	Masculino	de 66 a 75 anos
	Estágio 2 (perda parcial da espessura						Leve	Feminino	de 76 a 85 anos
	Estágio 2 (perda parcial da espessura						Leve	Masculino	de 56 a 65 anos
	Estágio 3 (perda total da espessura						Leve	Masculino	de 36 a 45 anos
	Estágio 1 (pele íntegra com						Leve	Masculino	de 46 a 55 anos
	Estágio 2 (perda parcial da espessura						Leve	Masculino	de 56 a 65 anos

	Estágio 2 (perda parcial da espessura						Leve	Masculino	de 66 a 75 anos
	Estágio 2 (perda parcial da espessura						Leve	Feminino	de 46 a 55 anos
	Estágio 1 (pele íntegra com						Leve	Masculino	de 56 a 65 anos
	Estágio 2 (perda parcial da espessura						Leve	Masculino	de 56 a 65 anos
	Estágio 1 (pele íntegra com						Leve	Masculino	de 76 a 85 anos
	Estágio 1 (pele íntegra com						Leve	Feminino	de 66 a 75 anos
	Estágio 2 (perda parcial da espessura						Leve	Masculino	de 56 a 65 anos
	Estágio 2 (perda parcial da espessura						Leve	Masculino	de 36 a 45 anos
	Estágio 4 (perda total da espessura						Leve	Masculino	de 36 a 45 anos
	Estágio 1 (pele íntegra com						Leve	Feminino	de 56 a 65 anos
	Estágio 1 (pele íntegra com						Leve	Masculino	de 56 a 65 anos
	Estágio 1 (pele íntegra com						Leve	Masculino	de 76 a 85 anos
	Estágio 2 (perda parcial da espessura						Leve	Masculino	de 66 a 75 anos
	Estágio 1 (pele íntegra com						Leve	Masculino	de 66 a 75 anos
	Estágio 1 (pele íntegra com						Leve	Masculino	de 66 a 75 anos

	Estágio 2 (perda parcial da espessura						Leve	Masculino	de 66 a 75 anos
	Estágio 2 (perda parcial da espessura						Leve	Feminino	de 56 a 65 anos
	Estágio 2 (perda parcial da espessura						Leve	Masculino	de 46 a 55 anos
	Estágio 2 (perda parcial da espessura						Leve	Masculino	de 56 a 65 anos
	Estágio 2 (perda parcial da espessura						Leve	Feminino	de 56 a 65 anos
	Estágio 1 (pele íntegra com						Leve	Feminino	de 26 a 35 anos
	Estágio 2 (perda parcial da espessura						Leve	Feminino	mais de 85 anos
	Estágio 3 (perda total da espessura						Moderado	Feminino	mais de 85 anos
	Estágio 2 (perda parcial da espessura						Leve	Feminino	mais de 85 anos
	Estágio 2 (perda parcial da espessura						Leve	Feminino	mais de 85 anos
	Estágio 2 (perda parcial da espessura						Leve	Feminino	mais de 85 anos
	Estágio 2 (perda parcial da espessura						Leve	Feminino	mais de 85 anos
	Estágio 2 (perda parcial da espessura						Leve	Masculino	de 66 a 75 anos
	Estágio 2 (perda parcial da espessura						Leve	Masculino	de 66 a 75 anos
	Estágio 2 (perda parcial da espessura						Leve	Feminino	de 46 a 55 anos

	Estágio 2 (perda parcial da espessura						Leve	Masculino	de 26 a 35 anos
	Estágio 2 (perda parcial da espessura						Leve	Feminino	de 76 a 85 anos
	Estágio 2 (perda parcial da espessura						Leve	Masculino	de 36 a 45 anos
	Estágio 2 (perda parcial da espessura						Leve	Masculino	de 76 a 85 anos
	Estágio 2 (perda parcial da espessura						Leve	Masculino	de 66 a 75 anos
	Estágio 2 (perda parcial da espessura						Leve	Masculino	de 66 a 75 anos
	Estágio 1 (pele íntegra com						Leve	Feminino	de 66 a 75 anos
	Estágio 2 (perda parcial da espessura						Leve	Masculino	de 36 a 45 anos
	Estágio 2 (perda parcial da espessura						Leve	Feminino	de 66 a 75 anos
	Estágio 2 (perda parcial da espessura						Leve	Feminino	de 66 a 75 anos
	Estágio 2 (perda parcial da espessura						Leve	Masculino	de 66 a 75 anos
	Estágio 2 (perda parcial da espessura						Leve	Feminino	de 66 a 75 anos
	Estágio 2 (perda parcial da espessura						Leve	Masculino	de 76 a 85 anos
	Estágio 2 (perda parcial da espessura						Leve	Masculino	de 76 a 85 anos
	Estágio 2 (perda parcial da espessura						Leve	Masculino	de 66 a 75 anos

	Estágio 2 (perda parcial da espessura						Leve	Masculino	de 66 a 75 anos
	Estágio 2 (perda parcial da espessura						Leve	Masculino	de 66 a 75 anos
	Estágio 1 (pele íntegra com						Leve	Feminino	de 66 a 75 anos
	Estágio 2 (perda parcial da espessura						Leve	Feminino	de 76 a 85 anos
	Estágio 2 (perda parcial da espessura						Leve	Feminino	de 66 a 75 anos
	Estágio 3 (perda total da espessura						Moderado	Masculino	de 66 a 75 anos
	Estágio 1 (pele íntegra com						Leve	Masculino	de 66 a 75 anos
	Estágio 2 (perda parcial da espessura						Leve	Feminino	de 36 a 45 anos
	Estágio 1 (pele íntegra com						Leve	Feminino	de 56 a 65 anos
	Estágio 2 (perda parcial da espessura						Leve	Feminino	de 46 a 55 anos
	Estágio 2 (perda parcial da espessura						Leve	Feminino	de 66 a 75 anos
	Estágio 2 (perda parcial da espessura						Leve	Masculino	de 46 a 55 anos
	Estágio 2 (perda parcial da espessura						Leve	Masculino	de 66 a 75 anos
	Estágio 3 (perda total da espessura						Moderado	Masculino	de 26 a 35 anos
	Estágio 2 (perda parcial da espessura						Leve	Feminino	de 66 a 75 anos

	Estágio 2 (perda parcial da espessura						Leve	Masculino	de 66 a 75 anos
	Estágio 2 (perda parcial da espessura						Leve	Masculino	de 56 a 65 anos
	Estágio 2 (perda parcial da espessura						Leve	Feminino	mais de 85 anos
	Estágio 2 (perda parcial da espessura						Leve	Masculino	de 66 a 75 anos
	Estágio 2 (perda parcial da espessura						Leve	Masculino	de 56 a 65 anos
	Estágio 2 (perda parcial da espessura						Leve	Feminino	de 56 a 65 anos
	Estágio 2 (perda parcial da espessura						Leve	Feminino	de 46 a 55 anos
	Estágio 2 (perda parcial da espessura						Leve	Feminino	de 56 a 65 anos
	Estágio 2 (perda parcial da espessura						Leve	Masculino	de 66 a 75 anos
	Estágio 2 (perda parcial da espessura						Leve	Feminino	de 66 a 75 anos
	Estágio 2 (perda parcial da espessura						Leve	Feminino	de 56 a 65 anos
	Estágio 2 (perda parcial da espessura						Leve	Feminino	de 76 a 85 anos
	Estágio 2 (perda parcial da espessura						Leve	Feminino	de 76 a 85 anos
	Estágio 2 (perda parcial da espessura						Leve	Feminino	de 76 a 85 anos
	Estágio 1 (pele íntegra com						Leve	Feminino	de 36 a 45 anos

	Estágio 1 (pele íntegra com						Leve	Feminino	de 66 a 75 anos
	Estágio 1 (pele íntegra com						Leve	Feminino	de 66 a 75 anos
	Estágio 1 (pele íntegra com						Leve	Feminino	de 66 a 75 anos
	Estágio 1 (pele íntegra com						Leve	Feminino	de 66 a 75 anos
	Estágio 1 (pele íntegra com						Leve	Masculino	de 46 a 55 anos
							Leve	Masculino	de 46 a 55 anos
	Estágio 1 (pele íntegra com						Leve	Feminino	de 66 a 75 anos
	Estágio 1 (pele íntegra com						Leve	Feminino	de 46 a 55 anos
	Estágio 1 (pele íntegra com						Leve	Feminino	de 66 a 75 anos
	Estágio 1 (pele íntegra com						Leve	Feminino	de 76 a 85 anos
	Estágio 1 (pele íntegra com						Leve	Masculino	de 56 a 65 anos
	Estágio 2 (perda parcial da espessura						Leve	Masculino	de 56 a 65 anos
	Estágio 2 (perda parcial da espessura						Leve	Feminino	de 66 a 75 anos
	Estágio 2 (perda parcial da espessura						Leve	Masculino	de 56 a 65 anos
	Estágio 2 (perda parcial da espessura						Leve	Feminino	de 66 a 75 anos

	Estágio 2 (perda parcial da espessura						Leve	Feminino	de 56 a 65 anos
	Estágio 2 (perda parcial da espessura						Leve	Feminino	de 66 a 75 anos
	Estágio 1 (pele íntegra com						Leve	Masculino	de 46 a 55 anos
	Estágio 2 (perda parcial da espessura						Leve	Masculino	de 46 a 55 anos
	Estágio 1 (pele íntegra com						Leve	Masculino	de 56 a 65 anos
	Estágio 2 (perda parcial da espessura						Leve	Feminino	de 66 a 75 anos
	Estágio 1 (pele íntegra com						Leve	Masculino	de 46 a 55 anos
	Estágio 2 (perda parcial da espessura						Leve	Feminino	de 56 a 65 anos
	Estágio 1 (pele íntegra com						Leve	Feminino	de 56 a 65 anos
	Estágio 1 (pele íntegra com						Leve	Feminino	de 56 a 65 anos
	Estágio 2 (perda parcial da espessura						Leve	Feminino	de 66 a 75 anos
	Estágio 2 (perda parcial da espessura						Leve	Masculino	de 46 a 55 anos
	Estágio 2 (perda parcial da espessura						Leve	Masculino	de 66 a 75 anos
	Estágio 1 (pele íntegra com						Leve	Feminino	de 76 a 85 anos
	Estágio 1 (pele íntegra com						Leve	Masculino	de 36 a 45 anos

	Estágio 2 (perda parcial da espessura						Leve	Feminino	de 56 a 65 anos
	Estágio 3 (perda total da espessura						Moderado	Masculino	de 36 a 45 anos
	Estágio 1 (pele íntegra com						Leve	Masculino	de 66 a 75 anos
	Estágio 2 (perda parcial da espessura						Leve	Masculino	de 36 a 45 anos
	Estágio 1 (pele íntegra com						Leve	Feminino	de 66 a 75 anos
	Estágio 2 (perda parcial da espessura						Leve	Masculino	de 56 a 65 anos
	Estágio 3 (perda total da espessura						Moderado	Feminino	de 66 a 75 anos
	Estágio 2 (perda parcial da espessura						Leve	Masculino	de 56 a 65 anos
	Estágio 2 (perda parcial da espessura						Leve	Feminino	de 66 a 75 anos
	Estágio 2 (perda parcial da espessura						Leve	Masculino	de 46 a 55 anos
	Estágio 2 (perda parcial da espessura						Leve	Masculino	mais de 85 anos
	Estágio 2 (perda parcial da espessura						Leve	Feminino	de 76 a 85 anos
	Estágio 2 (perda parcial da espessura						Leve	Feminino	de 56 a 65 anos
	Estágio 3 (perda total da espessura						Moderado	Feminino	de 66 a 75 anos
	Estágio 2 (perda parcial da espessura						Leve	Feminino	de 66 a 75 anos

	Estágio 2 (perda parcial da espessura						Leve	Feminino	de 56 a 65 anos
	Estágio 2 (perda parcial da espessura						Leve	Masculino	de 18 a 25 anos
	Estágio 2 (perda parcial da espessura						Leve	Feminino	de 46 a 55 anos
	Estágio 2 (perda parcial da espessura						Leve	Masculino	de 56 a 65 anos
	Estágio 1 (pele íntegra com						Leve	Feminino	de 46 a 55 anos
	Estágio 1 (pele íntegra com						Leve	Feminino	de 66 a 75 anos
	Estágio 3 (perda total da espessura						Moderado	Feminino	de 46 a 55 anos
	Estágio 1 (pele íntegra com						Leve	Masculino	de 76 a 85 anos
	Estágio 1 (pele íntegra com						Leve	Feminino	de 36 a 45 anos
	Estágio 1 (pele íntegra com						Nenhum	Feminino	de 26 a 35 anos
	Estágio 2 (perda parcial da espessura						Leve	Feminino	de 56 a 65 anos
	Estágio 1 (pele íntegra com						Leve	Feminino	de 76 a 85 anos
	Estágio 1 (pele íntegra com						Leve	Masculino	de 76 a 85 anos
	Estágio 2 (perda parcial da espessura						Leve	Feminino	de 46 a 55 anos
	Estágio 2 (perda parcial da espessura						Leve	Masculino	de 66 a 75 anos

	Estágio 1 (pele íntegra com						Leve	Masculino	de 56 a 65 anos
	Estágio 2 (perda parcial da espessura						Leve	Masculino	de 66 a 75 anos
	Estágio 2 (perda parcial da espessura						Leve	Masculino	de 46 a 55 anos
	Estágio 1 (pele íntegra com						Leve	Feminino	de 76 a 85 anos
	Estágio 2 (perda parcial da espessura						Leve	Masculino	de 36 a 45 anos
	Estágio 2 (perda parcial da espessura						Leve	Masculino	de 46 a 55 anos
	Estágio 1 (pele íntegra com						Leve	Masculino	de 76 a 85 anos
	Estágio 2 (perda parcial da espessura						Leve	Masculino	de 66 a 75 anos
	Estágio 2 (perda parcial da espessura						Leve	Masculino	de 76 a 85 anos
	Estágio 2 (perda parcial da espessura						Leve	Masculino	de 66 a 75 anos
	Estágio 1 (pele íntegra com						Leve	Feminino	de 56 a 65 anos
	Estágio 2 (perda parcial da espessura						Leve	Feminino	de 46 a 55 anos
	Estágio 2 (perda parcial da espessura						Leve	Feminino	de 36 a 45 anos
	Estágio 2 (perda parcial da espessura						Leve	Feminino	de 46 a 55 anos
	Estágio 2 (perda parcial da espessura						Leve	Masculino	de 36 a 45 anos

	Estágio 2 (perda parcial da espessura						Leve	Masculino	de 46 a 55 anos
	Estágio 1 (pele íntegra com						Leve	Masculino	de 18 a 25 anos
	Estágio 2 (perda parcial da espessura						Leve	Masculino	de 56 a 65 anos
	Estágio 2 (perda parcial da espessura						Leve	Masculino	de 56 a 65 anos
	Estágio 2 (perda parcial da espessura						Leve	Masculino	de 56 a 65 anos
							Moderado	Masculino	de 18 a 25 anos
							Leve	Feminino	de 76 a 85 anos
							Leve	Masculino	de 66 a 75 anos
							Leve	Feminino	de 66 a 75 anos
							Leve	Masculino	de 56 a 65 anos
							Leve	Masculino	de 46 a 55 anos
							Leve	Feminino	de 56 a 65 anos
							Leve	Feminino	de 66 a 75 anos
							Leve	Feminino	de 76 a 85 anos
							Leve	Feminino	de 76 a 85 anos

							Leve	Masculino	de 76 a 85 anos
							Leve	Masculino	de 36 a 45 anos
							Leve	Masculino	de 56 a 65 anos
							Leve	Masculino	de 46 a 55 anos
							Leve	Feminino	de 56 a 65 anos
							Leve	Feminino	de 46 a 55 anos
							Leve	Feminino	de 56 a 65 anos
							Leve	Feminino	de 76 a 85 anos
							Leve	Masculino	de 36 a 45 anos
							Leve	Feminino	de 76 a 85 anos
	Estagio 1 (pele íntegra com						Leve	Feminino	de 56 a 65 anos
	Estagio 1 (pele íntegra com						Leve	Masculino	de 76 a 85 anos
							Leve	Masculino	de 36 a 45 anos
							Leve	Feminino	de 66 a 75 anos
							Leve	Feminino	de 18 a 25 anos

							Leve	Feminino	de 66 a 75 anos
							Leve	Masculino	de 46 a 55 anos
							Leve	Feminino	de 66 a 75 anos
							Leve	Feminino	de 46 a 55 anos
							Leve	Feminino	mais de 85 anos
							Leve	Masculino	de 46 a 55 anos
							Leve	Masculino	de 26 a 35 anos
							Leve	Masculino	de 26 a 35 anos
							Leve	Masculino	de 46 a 55 anos
							Leve	Masculino	de 56 a 65 anos
							Leve	Feminino	de 46 a 55 anos
							Leve	Masculino	de 76 a 85 anos
							Grave	Feminino	de 66 a 75 anos
							Leve	Feminino	de 76 a 85 anos
							Leve	Masculino	de 66 a 75 anos

							Leve	Masculino	de 66 a 75 anos
							Leve	Masculino	de 56 a 65 anos
							Leve	Masculino	de 56 a 65 anos
							Nenhum	Feminino	de 26 a 35 anos
							Leve	Masculino	de 66 a 75 anos
							Leve	Masculino	de 66 a 75 anos
							Leve	Masculino	de 66 a 75 anos
							Leve	Masculino	de 76 a 85 anos
							Leve	Feminino	de 66 a 75 anos
	Estagio 2 (perda parcial da espessura						Leve	Feminino	menos de 28 dias
							Leve	Masculino	de 76 a 85 anos
							Leve	Feminino	de 56 a 65 anos
							Leve	Masculino	de 76 a 85 anos
	Estagio 1 (pele íntegra com						Leve	Feminino	de 56 a 65 anos
							Leve	Masculino	de 26 a 35 anos

							Leve	Feminino	de 56 a 65 anos
	Estágio 2 (perda parcial da espessura						Leve	Masculino	de 26 a 35 anos
							Leve	Masculino	de 26 a 35 anos
							Leve	Feminino	de 12 a 17 anos
							Leve	Masculino	de 66 a 75 anos
							Leve	Masculino	de 56 a 65 anos
	Estágio 2 (perda parcial da espessura						Leve	Feminino	mais de 85 anos
							Leve	Feminino	de 36 a 45 anos
							Leve	Feminino	de 66 a 75 anos
							Leve	Feminino	de 46 a 55 anos
							Leve	Masculino	de 36 a 45 anos
	Estágio 1 (pele íntegra com						Leve	Feminino	de 76 a 85 anos
							Leve	Feminino	de 66 a 75 anos
							Leve	Feminino	de 56 a 65 anos
							Leve	Masculino	de 66 a 75 anos

							Leve	Masculino	menos de 28 dias
							Leve	Masculino	de 76 a 85 anos
							Leve	Feminino	de 46 a 55 anos
							Leve	Masculino	de 66 a 75 anos
							Leve	Feminino	de 18 a 25 anos
	Estagio 1 (pele íntegra com						Leve	Feminino	de 46 a 55 anos
	Estagio 1 (pele íntegra com						Leve	Feminino	de 66 a 75 anos
							Leve	Feminino	de 46 a 55 anos
							Leve	Masculino	de 56 a 65 anos
	Estagio 2 (perda parcial da espessura						Leve	Feminino	de 66 a 75 anos
							Leve	Masculino	de 26 a 35 anos
							Leve	Feminino	de 56 a 65 anos
	Estagio 1 (pele íntegra com						Nenhum	Feminino	de 36 a 45 anos
							Leve	Feminino	de 18 a 25 anos
							Leve	Masculino	de 26 a 35 anos

							Leve	Masculino	de 26 a 35 anos
							Leve	Masculino	de 46 a 55 anos
							Leve	Masculino	de 56 a 65 anos
							Leve	Masculino	de 56 a 65 anos
							Leve	Masculino	de 56 a 65 anos
	Estágio 2 (perda parcial da espessura						Leve	Masculino	de 46 a 55 anos
	Estágio 2 (perda parcial da espessura						Leve	Masculino	de 66 a 75 anos
	Estágio 2 (perda parcial da espessura						Leve	Masculino	de 18 a 25 anos
							Leve	Masculino	de 56 a 65 anos
							Leve	Feminino	de 66 a 75 anos
							Leve	Masculino	de 12 a 17 anos
							Leve	Masculino	de 36 a 45 anos
							Leve	Feminino	de 46 a 55 anos
							Leve	Feminino	de 18 a 25 anos
							Leve	Masculino	de 12 a 17 anos

							Óbito	Masculino	de 36 a 45 anos
							Leve	Masculino	de 46 a 55 anos
	Estágio 2 (perda parcial da espessura						Leve	Masculino	de 46 a 55 anos
	Estágio 2 (perda parcial da espessura						Leve	Masculino	de 36 a 45 anos
							Leve	Masculino	de 46 a 55 anos
							Leve	Feminino	de 66 a 75 anos
	Estágio 1 (pele íntegra com						Leve	Feminino	de 76 a 85 anos
							Leve	Feminino	de 46 a 55 anos
							Leve	Feminino	de 66 a 75 anos
							Leve	Masculino	de 66 a 75 anos
	Estágio 2 (perda parcial da espessura						Leve	Feminino	de 76 a 85 anos
							Leve	Feminino	de 76 a 85 anos
							Leve	Masculino	de 56 a 65 anos
							Leve	Masculino	de 46 a 55 anos
							Leve	Masculino	de 46 a 55 anos

							Leve	Masculino	de 66 a 75 anos
							Leve	Masculino	de 66 a 75 anos
	Estágio 3 (perda total da espessura						Leve	Masculino	de 36 a 45 anos
	Estágio 1 (pele íntegra com						Leve	Masculino	de 46 a 55 anos
	Estágio 2 (perda parcial da espessura						Leve	Masculino	de 56 a 65 anos
	Estágio 2 (perda parcial da espessura						Leve	Masculino	de 46 a 55 anos
	Estágio 1 (pele íntegra com						Leve	Feminino	de 46 a 55 anos
	Estágio 1 (pele íntegra com						Leve	Feminino	de 66 a 75 anos
							Leve	Masculino	de 56 a 65 anos
							Leve	Masculino	de 26 a 35 anos
	Estágio 2 (perda parcial da espessura						Leve	Masculino	de 56 a 65 anos
							Leve	Masculino	de 66 a 75 anos
							Leve	Masculino	de 66 a 75 anos
	Estágio 2 (perda parcial da espessura						Leve	Feminino	de 46 a 55 anos
							Leve	Feminino	de 46 a 55 anos

							Leve	Feminino	de 18 a 25 anos
							Leve	Feminino	de 36 a 45 anos
	Estagio 2 (perda parcial da espessura						Leve	Feminino	de 46 a 55 anos
							Grave	Masculino	de 76 a 85 anos
							Nenhum	Masculino	de 56 a 65 anos
							Leve	Feminino	de 46 a 55 anos
							Grave	Masculino	de 66 a 75 anos
							Leve	Feminino	de 66 a 75 anos
	Estagio 1 (pele íntegra com						Leve	Masculino	de 46 a 55 anos
							Leve	Feminino	de 66 a 75 anos
							Leve	Masculino	de 56 a 65 anos
	Nao Classificável (perda						Leve	Masculino	de 76 a 85 anos
	Estagio 2 (perda parcial da espessura						Leve	Feminino	de 56 a 65 anos
							Leve	Feminino	de 76 a 85 anos
							Leve	Feminino	de 18 a 25 anos

							Leve	Feminino	de 26 a 35 anos
							Leve	Feminino	de 36 a 45 anos
	Estágio 1 (pele íntegra com						Leve	Masculino	de 18 a 25 anos
	Relacionada a dispositivo para a						Moderado	Feminino	de 46 a 55 anos
	Não Classificável (perda						Leve	Masculino	de 66 a 75 anos
							Leve	Masculino	de 56 a 65 anos
							Leve	Masculino	de 76 a 85 anos
	Estágio 2 (perda parcial da espessura						Leve	Feminino	de 46 a 55 anos
							Leve	Feminino	de 56 a 65 anos
							Leve	Masculino	de 18 a 25 anos
							Leve	Masculino	de 36 a 45 anos
							Leve	Masculino	de 26 a 35 anos
							Leve	Masculino	de 56 a 65 anos
							Leve	Feminino	de 26 a 35 anos
							Leve	Feminino	de 76 a 85 anos

							Leve	Masculino	de 46 a 55 anos
	Nao Classificá vel (perda						Leve	Masculino	de 66 a 75 anos
							Leve	Feminino	de 36 a 45 anos
							Leve	Masculino	de 26 a 35 anos
							Leve	Feminino	de 36 a 45 anos
							Leve	Feminino	de 46 a 55 anos
							Leve	Masculino	de 66 a 75 anos
							Leve	Feminino	de 36 a 45 anos
							Leve	Feminino	de 36 a 45 anos
							Leve	Masculino	de 56 a 65 anos
	Estagio 2 (perda parcial da espessura						Leve	Masculino	de 76 a 85 anos
	Estagio 3 (perda total da espessura						Moderado	Feminino	de 66 a 75 anos
	Nao Classificá vel (perda						Moderado	Feminino	de 76 a 85 anos
	Estagio 2 (perda parcial da espessura						Leve	Masculino	de 56 a 65 anos
	Estagio 1 (pele íntegra com						Leve	Feminino	de 66 a 75 anos

	Não Classificável (perda						Moderado	Masculino	de 46 a 55 anos
	Não Classificável (perda						Moderado	Masculino	de 46 a 55 anos
	Estágio 3 (perda total da espessura						Moderado	Masculino	de 56 a 65 anos
	Estágio 2 (perda parcial da espessura						Leve	Masculino	de 76 a 85 anos
	Estágio 2 (perda parcial da espessura						Leve	Feminino	de 36 a 45 anos
	Estágio 2 (perda parcial da espessura						Leve	Masculino	de 56 a 65 anos
	Estágio 2 (perda parcial da espessura						Leve	Masculino	de 26 a 35 anos
	Não Classificável (perda						Moderado	Feminino	de 56 a 65 anos
	Estágio 2 (perda parcial da espessura						Leve	Feminino	de 36 a 45 anos
	Não Classificável (perda						Leve	Masculino	de 56 a 65 anos
	Não Classificável (perda						Moderado	Feminino	de 26 a 35 anos
	Estágio 2 (perda parcial da espessura						Leve	Feminino	de 46 a 55 anos
	Estágio 2 (perda parcial da espessura						Leve	Feminino	de 66 a 75 anos
	Não Classificável (perda						Moderado	Feminino	mais de 85 anos
	Estágio 2 (perda parcial da espessura						Leve	Masculino	de 46 a 55 anos

	Estágio 3 (perda total da espessura						Moderado	Feminino	de 56 a 65 anos
	Estágio 1 (pele íntegra com						Leve	Masculino	de 56 a 65 anos
	Estágio 3 (perda total da espessura						Moderado	Masculino	de 56 a 65 anos
[truncated: 350,313 more chars]
