# Supplementary material for: Frequency and content analysis of adverse event reports in surgical centers: a cross-sectional study
Source: Rev Bras Enferm. 2025 Mar 14;78(1):e20240082. doi: 10.1590/0034-7167-2024-0082 (PMC11913048; doi:10.1590/0034-7167-2024-0082)
Supplement: Translation

# Translation: Frequência e análise do conteúdo das notificações de eventos adversos em centros cirúrgicos: um estudo transversal

**DOI:** 10.1590/0034-7167-2024-0082pt

**Authors:** Ítalo Lennon Sales de Almeida, Ana Paula Agostinho Alencar, Vanessa de Brito Poveda, Rhanna Emanuela Fontenele Lima de Carvalho

**Affiliations:** I Universidade Estadual do Ceará. Fortaleza, Ceará, Brasil; II Universidade de São Paulo. São Paulo, São Paulo, Brasil

**Keywords:** Segurança do Paciente, Notificação, Eventos Adversos, Centros Cirúrgicos, Procedimentos Cirúrgicos Operatórios

## Abstract

**Objetivos:**

identificar a frequência de eventos adversos notificados em centros cirúrgicos e analisar o conteúdo das notificações realizadas.

**Métodos:**

estudo transversal analisou notificações de janeiro de 2019 a março de 2023 em oito hospitais, com uma amostra de 163 notificações. A análise quantitativa considerou variáveis como tipo e grau do dano, enquanto a qualitativa utilizou análise de similitude no Iramuteq.

**Resultados:**

foram identificados dezesseis *never events*, incluindo lesões por pressão (estágios três e quatro), perda de material para biópsia, procedimentos cirúrgicos incorretos e retenção não intencional de corpo estranho. A análise qualitativa destacou termos como “falhas na assistência”, “falha”, “procedimento cirúrgico” e “lesão por pressão”.

**Conclusões:**

lesões por pressão, queimaduras, lesão de órgão e infecção de sítio cirúrgico foram as notificações mais frequentes. Há subnotificação de eventos adversos em centros cirúrgicos e limitações na qualidade dos registros, incluindo eventos adversos não especificados e ausência de padronização no relato.

## INTRODUÇÃO

Danos causados por cuidados realizados de forma insegura e, portanto evitáveis, podem levar a morte, a incapacidade e causar importante impacto financeiro e econômico para instituições de saúde e sistemas de saúde, logo são um desafio global^(1)^. Os danos em saúde levam à redução da confiança das pessoas nos sistemas de saúde e no trabalho dos profissionais, que também podem sofrer danos psicológicos quando envolvidos em eventos graves com danos permanentes ou óbito dos seus pacientes^(1)^.

No Brasil, desde o lançamento do Programa Nacional de Segurança do Paciente (PNSP), em 2013, várias ações foram implementadas para melhorar o cuidado. Isso incluiu a criação de Núcleos de Segurança do Paciente (NSP) e protocolos de segurança em estabelecimentos de saúde, além de outras medidas que envolveram a participação de pacientes e de familiares nos cuidados, aumentar o acesso da sociedade a informações sobre segurança do paciente, produzir e disseminar conhecimentos sobre o tema e promover a inclusão do assunto nos cursos de formação na área da saúde^(2)^.

O segundo Desafio Global para a Segurança do Paciente concentra-se na cirurgia segura, integrando ações propostas pela Organização Mundial de Saúde a protocolos essenciais que foram incentivados pelo Ministério da Saúde do Brasil para gerenciamento de riscos em saúde. Essa abordagem é motivada pela incidência de eventos adversos que chegam à 16% em centros cirúrgicos, segundo a OMS, e podem atingir 20% dos procedimentos cirúrgicos realizados, segundo estudos brasileiros^(3-6)^.

Por conseguinte, a notificação de eventos adversos é o que torna possível identificar os momentos da prestação de cuidados em saúde nos quais ocorrem mais incidentes. Logo, as notificações dão subsídios para o desenvolvimento de estratégias de gerenciamento de riscos para prevenção da ocorrência de falhas nos cuidados^(7,8)^. Sendo o Notivisa (Sistema Nacional de Notificações para a Vigilância Sanitária), desenvolvido pela Agência Nacional de Vigilância Sanitária (Anvisa), é o sistema nacional que permite a notificação de eventos adversos na assistência à saúde. O sistema permite que os profissionais de saúde, os gestores e a população em geral possam acessá-lo para relatar a ocorrência de incidentes^(9)^.

Assim, eventos adversos são definidos como incidentes que geram danos aos pacientes durante a internação ou o atendimento em estabelecimentos assistenciais de saúde ou, ainda, durante o uso de tecnologias de saúde. E inclui efeitos indesejados de medicamentos em doses terapêuticas habituais, queixas técnicas de produtos de saúde e incidentes cirúrgicos, sendo que a maioria destes eventos poderia ser prevenida. Já os eventos adversos que causam danos graves e expõe pacientes a risco de óbito ou geram danos irreparáveis são classificados como *never events,* sendo eles: a cirurgia em um paciente errado ou em um membro errado, a perda de uma peça para biópsia, a retenção de corpo estranho em um paciente após uma cirurgia, entre outros^(9)^.

A cultura de notificação de eventos adversos no Brasil é um importante ponto de discussão, considerando que as instituições de saúde ainda encontram barreiras e limitações para o ato de notificar. A cultura punitiva e o medo de reconhecer o erro ainda são realidades que trazem desafios à gestores e aos líderes em instituições de saúde^(10,11)^.

Além do desafio de assegurar o acesso universal aos procedimentos cirúrgicos no sistema de saúde brasileiro^(12)^, é importante destacar que a adesão aos protocolos de segurança cirúrgica ainda é incipiente ou apresenta falhas significativas em sua implementação. Isso ressalta a necessidade de desenvolver estratégias de aprimoramento para promover uma cultura mais robusta de segurança cirúrgica em estabelecimentos de saúde^(13,14)^.

Logo, a análise de eventos adversos notificados pelos centros cirúrgicos deve ser utilizada como forma de dar subsídios à tomada de decisão no gerenciamento da assistência cirúrgica, com foco na melhoria da segurança e da qualidade do cuidado ofertado aos pacientes que necessitam de intervenções cirúrgicas.

No Brasil, as análises de eventos adversos em centros cirúrgicos têm se concentrado na revisão de prontuários^(5-7)^. Por conseguinte, o conteúdo das notificações realizadas no Notivisa é desconhecido e ainda requer a realização de análises. Assim, este estudo poderá indicar quais são os eventos adversos mais notificados, suas características e como os profissionais notificadores relatam esses eventos no processo de notificação, colaborando para o conhecimento da área no que diz respeito à forma como o sistema nacional de notificação de eventos adversos relacionados à assistência à saúde tem sido utilizado e quais as potencialidades e fragilidades do processo de notificação.

Em suma, as características dos eventos notificados devem apontar para a direção que os protocolos institucionais devem seguir para que sejam criadas estratégias de prevenção. Compreende-se que ao analisar o conteúdo das notificações será possível pensar estratégias de melhoria da sua qualidade nas instituições notificadoras.

## OBJETIVOS

Identificar a frequência de eventos adversos notificados em centros cirúrgicos e analisar o conteúdo das notificações realizadas.

## MÉTODOS

### Aspectos éticos

O projeto de pesquisa foi apreciado e aprovado pelo Comitê de Ética em Pesquisa da Universidade Estadual do Ceará. Após a aprovação ética foi obtida anuência de acesso aos dados do Notivisa pela Secretaria de Saúde do Estado do Ceará (SESA-CE) e realizada reunião de alinhamento junto da Coordenadoria de Vigilância Sanitária da SESA-CE (COVIS), para apresentação do projeto de pesquisa e alinhamento da forma de disponibilização do banco de dados do sistema pela equipe técnica responsável.

Assim, o banco com todos os dados e variáveis disponíveis no Notivisa foi enviado ao e-mail pessoal do pesquisador responsável pelo estudo. Contudo, com o propósito de garantir a confidencialidade dos dados, foi criada uma versão do banco para análise de dados que não possuía dados de identificação do profissional notificador e não permitia a identificação da instituição de ocorrência do evento, pois receberam codificações. Para a apresentação dos dados, o nome das instituições foi codificado por letras (Hospital A, Hospital B, Hospital C...).

### Desenho, período e local do estudo

Estudo transversal, realizado com os dados de eventos adversos notificados em centros cirúrgicos, no período de janeiro de 2019 a março de 2023, em oito hospitais terciários sob gestão estadual, localizados em um estado da região Nordeste do Brasil. O estudo seguiu as recomendações do *Strengthening the Reporting of Observational Studies in Epidemiology* (STROBE) para relatar estudos observacionais.

### População ou amostra; critérios de inclusão e de exclusão

As notificações foram extraídas do sistema brasileiro de notificação de eventos adversos na assistência à saúde (Notivisa). Compuseram a amostra do estudo as notificações de eventos adversos relacionados à assistência à saúde realizadas pelos centros cirúrgicos nas instituições. Não foram definidos critérios de exclusão, sendo utilizadas todas as notificações identificadas. Foram detectadas 163 notificações no período consultado (N = 163).

### Protocolo do estudo

No período analisado, o banco de dados de notificações do Notivisa possuía 9.253 registros de eventos adversos relacionados à assistência à saúde. Posto isto, foi realizada a filtragem dessas notificações pela variável “local de ocorrência”, sendo selecionadas as notificações que foram realizadas no centro cirúrgico. Das variáveis disponíveis no banco, foram selecionadas para o estudo: tipo de incidente, gênero, idade do paciente, grau do dano e diagnóstico de internação.

Para o cálculo da prevalência de eventos adversos em centros cirúrgicos, foi usado o quantitativo de cirurgias realizadas pelas instituições no período analisado pelo estudo, tendo o Sistema de Informação Hospitalar (SIH) como fonte, por meio do DATASUS do Ministério da Saúde, utilizando o conteúdo “internações”, grupo de procedimentos código 04 “procedimentos cirúrgicos” e o CNES de cada estabelecimento. As especialidades atendidas pelas instituições foram consultadas por meio de acesso ao Cadastro Nacional de Estabelecimentos de Saúde (CNES), via DATASUS e análise da produção no SIH.

### Análise dos resultados e da estatística

Os dados foram analisados por meio de cálculo das frequências simples e relativas das variáveis. Foi utilizado o programa estatístico JASP 0.17.3.0 de acesso gratuito. Para a classificação dos tipos de eventos adversos relatados nas notificações, foi utilizada como referência a Classificação Internacional para a Segurança do Paciente da Organização Mundial da Saúde (OMS)^(15)^. Os *never events*, eventos que nunca devem acontecer em serviços de saúde, foram classificados tendo como referências o Manual de Implantação do Núcleo de Segurança do Paciente em Serviços de Saúde da ANVISA^(9)^.

Já para a análise qualitativa foram utilizados os registros relatados nas notificações. No formulário de notificação do sistema Notivisa há uma área destinada para que o notificador faça um relato subjetivo do evento ocorrido, “informe o tipo de incidente ocorrido”. Das 163 notificações utilizadas no estudo, 44 deixaram este espaço sem preenchimento, consequentemente foram utilizados, para a análise qualitativa, 119 relatos que foram preenchidos.

Os dados foram organizados em um arquivo de texto e analisados no Software RStudio. R é uma linguagem de programação gratuita que oferece vários pacotes relacionados à avaliação da qualidade de dados para estudos observacionais de saúde. A análise dos domínios identificados nas pesquisas foi realizada por meio da interface R para análise multidimensional de textos e questionários (IRaMuTeQ) V.0.7 alpha 2.

Inicialmente, na análise do conteúdo das notificações, foram formadas figuras utilizando como base a teoria dos grafos (análise de similitude) e uma nuvem de palavras, por meio da qual é possível identificar as ocorrências textuais entre as palavras, auxiliando na identificação da estrutura das notificações a partir do conteúdo de um corpus textual. Também foram realizadas duas análises textuais: análise de similitude, que permite identificar as ocorrências entre as palavras e seu resultado e indica a ligação entre as palavras, considerando palavras com frequência igual ou superior a 10; nuvem de palavras, para agrupar as palavras e organizá-las graficamente de acordo com sua relevância, sendo as maiores aquelas com maior frequência, considerando palavras com qualquer frequência.

## RESULTADOS

No período analisado, os oito hospitais realizaram 151.293 procedimentos cirúrgicos e 9.253 notificações no NOTIVISA, sendo 163 (1,76%) notificações de eventos adversos realizadas pelo centro cirúrgico (Quadro 1), o que representa uma taxa de onze notificações para cada 10.000 cirurgias (0,0011%) e dezoito notificações cirúrgicas para cada mil notificações de eventos adversos realizadas (0,018%).

**Quadro 1:** Características dos hospitais participantes do estudo, quantidade de procedimentos cirúrgicos e notificações de eventos adversos (janeiro/2019 a março/2023)

	Especialidades cirúrgicas	Cirurgias realizadas^*^	Notificações no NOTIVISA	Notificações de eventos adversos cirúrgicos
Hospital A	Geral, bucomaxilofacial, neurocirurgia, endocrinologia, gastroenterologia, ginecologia, nefrologia, urologia, oftalmologia, oncologia, traumato-ortopedia, otorrinolaringologia, plástica e transplante	41.559	3.885	48
Hospital B	Geral, gastroenterologia, ginecologia, nefrologia, urologia, oncologia e torácica	23.345	553	23
Hospital C	Cardiologia, pneumologia, torácica e transplante (adultos e pediátrica)	20.818	327	1
Hospital D	Geral e neurocirurgia	18.612	3.708	39
Hospital E	Geral e traumato-ortopedia	17.385	362	43
Hospital F	Cirurgia pediátrica, cardiologia, gastroenterologia, nefrologia, urologia, neurocirurgia, oncologia, traumato-ortopedia, transplante	14.213	59	4
Hospital G	Geral, otorrinolaringologia e traumato-ortopedia (adultos e pediátrica)	7.952	111	4
Hospital H	Geral	7.409	248	1
Total		151.293	9.253	163

Fonte: CNES; SIH; NOTIVISA.

* SIH/DATASUS.

As características das instituições participantes do estudo são descritas no Quadro 1.

Quanto aos graus do dano sofrido, 57,1% das notificações apontaram para eventos com danos leves (Tabela 1), que acometeram principalmente o gênero masculino (95; 58,3%), adultos com idade entre 18 e 65 anos (92; 56,4%) ou com mais de 65 anos (58; 35,6%). As doenças do aparelho digestivo (22; 13,5%), lesões por causas externas (20; 12,3%), doenças do aparelho circulatório (19; 11,7%), respiratório (14;8,6%), neoplasias (13; 8%), afecções do aparelho geniturinário (12; 7,3%) e do sistema nervoso (10; 6,1%) são os principais diagnósticos dos pacientes que sofreram eventos adversos. Foi notificado um evento adverso que resultou em óbito (0,6%), sendo este relacionado a hemorragia pós-operatória.

**Tabela 1:** Distribuição dos eventos adversos segundo tipo de dano

Grau do dano	F	%
Nenhum	17	10,4
Leve	93	57,1
Moderado	45	27,6
Grave	7	4,3
Óbito	1	0,6

*Fonte: Notivisa/ Anvisa/ Ministério da Saúde.*

Quanto à identificação do tipo de evento adverso notificado, o mais frequente foi a lesão por pressão, seguida pelas queimaduras, infecção de sítio cirúrgico e lesão de órgão. Foram notificadas 21 (12,9%) suspensões cirúrgicas causadas por falhas no preparo do paciente para o procedimento, falta de profissionais ou insumos, além de lotação da Sala de Recuperação Pós-anestésica (SRPA). Cabe ressaltar que, quinze notificações não especificaram o tipo de evento adverso referido no formulário de notificação (Tabela 2).

**Tabela 2:** Classificação dos eventos adversos notificados pelos centros cirúrgicos nos hospitais analisados

Tipos de Incidentes	Especificação	F	%
Lesão por pressão (n = 59)	Lesão por pressão^+^	59	36,2
Incidente / evento adverso durante procedimento cirúrgico (n = 17)	Lesão de órgão	10	6,1
	Procedimento errado, paciente errado ou em local errado^#^	4	2,4
	Abertura involuntária da ferida operatória (deiscência)	1	0,6
	Hemorragia após a cirurgia	1	0,6
	Retenção de corpo estranho em paciente^#^	1	0,6
Acidentes com o paciente (n = 17)	Queimadura de paciente	11	6,7
	Extubação endotraqueal acidental	1	0,6
	Danos à cavidade oral durante a intubação	3	1,8
	Queda da maca	2	1,2
Infecções relacionadas à assistência à saúde (n = 10)	Infecção de sítio cirúrgico	10	6,1
Incidente / evento adverso nas atividades administrativas (n = 4)	Erro no resultado de exames	1	0,6
	Procedimento errado no mapa cirúrgico	2	1,2
	Troca de documentos do paciente	1	0,6
Incidente / evento adverso na identificação do paciente (n = 2)	Falta de pulseira de identificação	1	0,6
	Pulseira com nome de outro paciente	1	0,6
Problema / evento adverso relacionado ao uso de medicamentos (n = 1)	Administrado medicamento errado	1	0,6
Outros incidentes/ eventos adversos (n =9)	Falha na identificação de hipoglicemia	1	0,6
	Problemas com cateteres ou sondas	8	4,9
Qualidade do serviço (n = 29)	Suspensão Cirúrgica (incluindo falha no preparo do paciente, falta de profissional ou insumos e lotação na SRPA)	21	12,9
	Paciente sem exame intraoperatório por falta de insumo	1	0,6
	Protocolo de sepse descontinuado	1	0,6
	Perda de material para biópsia^#^	2	1,2
	SRPA lotada	1	0,6
	Tempo de cirurgia prolongado para desinfecção de equipamento usado em duas cirurgias	1	0,6
	Uso de equipamento com defeito em cirurgia (foco cirúrgico)	1	0,6
	Tempo de cirurgia prolongado por curto-circuito	1	0,6
Evento não especificado		15	9,2
Total		163	100

*Fonte: Notivisa/ Anvisa/ Ministério da Saúde. #Never event. +Never event: lesão por pressão estágio 3 e 4.*

Foram identificados 16 (9,8%) *never events*, sendo eles: lesões por pressão estágio três (6; 37,5%), lesões por pressão estágio quatro (3; 18,7%), perda de material para biópsia (2; 12,5%), procedimento cirúrgico em paciente errado (2; 12,5%), procedimento cirúrgico errado realizado no paciente (1; 6,2%), retenção não intencional de corpo estranho em paciente após cirurgia (1; 6,2%) e procedimento cirúrgico em local errado (1; 6,2%) (Tabela 2).

Das notificações de lesões por pressão, destacaram-se as de estágio dois (29; 49,2%), seguidas pelas classificadas como estágio um. O local de ocorrência mais frequente foi a região sacral (23; 39,0%) (Tabela 3).

**Tabela 3:** Características das lesões por pressão derivado de posicionamento perioperatório notificadas (n=59)

Estágio	F	%
1	21	35,6
2	29	49,2
3	6	10,1
4	3	5,1
Local		
Não informado	21	35,6
Sacral	23	39,0
Glúteo	5	8,4
Calcâneo	3	5,1
Trocânter	1	1,7
Maléolo	1	1,7
Pavilhão auricular	1	1,7
Joelho	1	1,7
Tórax	1	1,7
Occipital	1	1,7
Cotovelo	1	1,7
Total	59	100

*Fonte: Notivisa/ Anvisa/ Ministério da Saúde.*

Na análise de similitude conduzida a partir dos 119 relatos foi possível observar que há quatro termos proeminentes: “falhas na assistência”, “falha”, “procedimento cirúrgico” e “lesão por pressão”, com o qual todos as outras palavras ou termos estão conectados (Figura 1).

**Figura 1:** Análise de similitude

*Fonte: Notivisa/Anvisa/ Ministério da Saúde.*

Além do mais, o termo “falhas na assistência” está conectado aos termos “infecção associada à assistência”, “infecção de sítio cirúrgico”, “cirurgia primária” e “proteção do paciente”. Já a palavra “falha” se conecta aos termos “falha na assistência” e “procedimento cirúrgico”. E o termo “procedimento cirúrgico” está conectado as palavras “falha” e “pacientes”, além de estar conectado ao termo “suspensão de cirurgia”. Por fim, o termo “lesão por pressão” se conecta ao termo “procedimento cirúrgico” e a palavra “sacro”.

A nuvem de palavras permitiu que fossem confirmadas as palavras e os termos mais frequentemente utilizados nas notificações: lesão por pressão (f=39), paciente (f=33), sacro (f=23), cirurgia (f=16), falhas na assistência (f=15), proteção do paciente (f=15), falha (f=14), infecção de sítio cirúrgico (f=11), infecção associada à assistência (f=11), suspensão de cirurgia (f=10), causas evitáveis (f=8) e queimaduras (f=8).

## DISCUSSÃO

A notificação de eventos adversos é uma prática que permite que instituições de saúde identifiquem e reconheçam situações que geram risco e dano aos pacientes no momento da assistência, contudo, ainda enfrenta barreiras e limitações para sua incorporação plena na rotina de trabalho de profissionais da saúde^(16)^. Alguns tipos de eventos adversos enfrentam ainda mais barreiras para que sejam reportados, como sugerem os resultados do presente estudo ao apresentar apenas dezoito notificações cirúrgicas para cada mil notificações, considerando todas as realizadas pelos hospitais no período analisado. Esse dado alerta para um cenário de subnotificação que limita o reconhecimento do real cenário de ocorrência de eventos adversos em centros cirúrgicos.

Em comparação com os resultados de outros estudos brasileiros, a prevalência de eventos adversos notificados por centros cirúrgicos foi menor no presente estudo^(5,6)^. Cabe salientar que as análises aqui citadas foram realizadas levando em consideração investigação em prontuários que detectaram eventos adversos relatados pelos registros profissionais. Com isso, fica evidenciado que ainda há uma limitada cultura de notificação de eventos adversos de centros cirúrgicos em sistemas oficiais de notificação, sendo o prontuário do paciente a fonte mais segura para dimensionar a prevalência de incidentes que resultaram em dano ao paciente cirúrgico.

Por outro lado, as notificações identificadas permitiram verificar fragilidades em processos, como a adequada aplicação do Checklist de cirurgia segura, que resultaram em *never events*, como a realização de procedimentos errados e em pacientes errados, bem como falhas na identificação de pacientes, entre outros.

Estudos têm demonstrado que a equipe de saúde apresenta limitações quanto ao ato de notificar, que vão desde a compreensão do que são eventos adversos, identificação da sua ocorrência na prática clínica, detecção de quase-falha e quais as situações notificáveis^(17,18)^. No presente estudo essas limitações variam entre instituições e entre setores, considerando que algumas realizaram muito mais notificações que outras e que também pode ser identificada uma quantidade elevada de registros de outros tipos de eventos ocorridos no hospital, contudo poucos especificamente no Centro Cirúrgico, o que aponta para diferenças na cultura de notificação interinstitucional e entre os setores de uma mesma instituição.

Ademais, as lacunas no processo de notificação dos eventos adversos podem contribuir para o não reconhecimento dos problemas vivenciados no setor de saúde, impossibilitando que ações bem-sucedidas de prevenção possam ser adotadas e implementadas. Neste estudo, os eventos adversos mais frequentemente reportados estiveram relacionados às situações potencialmente preveníveis, por meio da implementação de protocolos bem definidos ou treinamento da equipe. Assim como ocorreu no único evento que resultou em óbito e esteve associado à ocorrência de hemorragia pós-operatória, que pode ter seu manejo beneficiado pela existência desses protocolos.

Nesse sentido, observou-se a incidência aumentada de lesões por pressão derivadas do posicionamento cirúrgico, especialmente relacionados às cirurgias com mais de duas horas de duração, uma vez que sucedem com frequência três vezes maior nesses casos, que além do efeito negativo em si da lesão, derivam problemas adicionais ao paciente e à instituição de saúde e são potencialmente preveníveis na assistência cirúrgica. Em concordância com os resultados encontrados, a literatura tem apontado que lesões em estágios iniciais (um e dois) são mais prevalentes, com predominância da região sacra como principal local de ocorrência, considerando que a posição supina em cirurgias é a mais prevalente^(19,20)^.

Outro evento adverso observado com frequência foi a ocorrência de queimaduras, que na maioria dos casos estão relacionadas aos aspectos relacionados à eletrocirurgia, derivados de falhas de manutenção, uso inadequado ou falta de treinamento. O treinamento regular da equipe cirúrgica na manipulação do bisturi elétrico, juntamente com a criação e a atualização de protocolos específicos, pode contribuir para a redução de danos. Sendo assim, a equipe deve permanecer alerta para alarmes sonoros, evitando acionamentos inadvertidos e garantindo o correto funcionamento dos sistemas de eletrocirurgia^(21)^.

Além do mais, outro aspecto que foi relacionado à ocorrência de eventos adversos foi a indicação adequada e técnica correta, pois mesmo procedimentos cirúrgicos eletivos estão sujeitos aos eventos associados ou não a condição clínica pré-existente do paciente e que podem levar a complicações relevantes e com impacto para a recuperação pós-operatória, como exemplo: acidente vascular cerebral, infarto do miocárdio, síndrome da angústia respiratória aguda, lesão renal aguda ou lesão intestinal aguda, que estão entre as causas mais comuns de morbilidade e mortalidade em pacientes cirúrgicos. Para sua prevenção, protocolos multidisciplinares de cuidados centrados no paciente têm efeitos benéficos na prevenção de estresse cirúrgico e na recuperação precoce no pós-operatório^(22)^.

Nesse sentido, a infecção de sítio cirúrgico é a complicação pós-operatória mais comum, além de ser o evento adverso relacionado à assistência mais frequentemente notificado. Sua ocorrência tem sido relacionada à cultura de segurança organizacional e suscita a necessidade de avaliação dos indicadores de assistência cirúrgica para que possam ser avaliados os riscos de sua ocorrência^(23)^.

No contexto de aprimoramento da segurança cirúrgica, a Lista de Verificação de Segurança Cirúrgica da OMS é a ferramenta de comunicação projetada para melhorar os processos e aprimorar o trabalho em equipe no centro cirúrgico, sendo apontada como o padrão-ouro para a prevenção de eventos adversos, como os relatados anteriormente^(24)^. Logo, sua adoção e adesão é altamente recomendada e fortemente associada à redução da ocorrência de danos na oferta de cuidados^(25)^. Contudo, sua adesão no contexto das instituições de saúde brasileiras ainda é incipiente^(14,26)^.

Na implementação, esta lacuna é particularmente relevante quando consideramos os achados do presente estudo, que revelam que os profissionais notificadores utilizam o sistema de notificação de eventos adversos como um canal para relatar problemas relacionados à qualidade do serviço ou às situações que geram riscos potenciais aos pacientes. Essas notificações abrangem desde notificações que relatam suspensão de cirurgias por falta de profissionais, insumos materiais ou oferta de leitos em SRPA, até outras situações que demandam atenção por parte da gestão.

Neste contexto, a comunicação segura e efetiva da equipe é um fator determinante para a redução de eventos adversos, a partir da valorização, percepção de atitudes e comportamento de todos os profissionais envolvidos no cuidado perioperatório do paciente, com vistas à promoção da cultura de segurança^(27)^.

Quanto ao conteúdo das notificações, foi evidente a variabilidade na forma como os profissionais descrevem os eventos adversos. Algumas notificações apresentavam relatos sucintos e informações limitadas sobre a ocorrência. Em contrapartida, outras notificações eram mais abrangentes, fornecendo detalhes minuciosos sobre o incidente, suas repercussões para o paciente e as medidas de intervenção implementadas pela equipe. Além do que, a literatura aponta para o desconhecimento dos profissionais sobre a existência de fichas de notificação ou instruções sobre como realizar a notificação e o uso de outros meios de registro dos eventos adversos ocorridos, como o caso de enfermeiros e técnicos de enfermagem que utilizam apenas as anotações de enfermagem para fazer esse tipo de relato^(28)^.

Destaca-se que dos registros analisados neste estudo, alguns não foram utilizados na análise qualitativa por não terem sido preenchidos no espaço destinado ao relato do notificador, o que alerta para uma grave falha no processo de notificação. O relato por escrito deve dar suporte e complementar o formulário com dados que poderão ser utilizados na análise de causa-raiz do evento, sendo o suporte necessário para que sejam elucidados os mecanismos de ocorrência e quais as medidas deverão ser adotadas para sua prevenção. Cabe ainda destacar que o presente estudo se diferencia de outras análises sobre o processo de notificação ao utilizar os dados do registro realizado.

Ao utilizar na descrição do evento e no momento da notificação termos como “falha na assistência” e “proteção do paciente”, os notificadores demonstram que compreendem que as ocorrências têm relação com erros no processo assistencial e estes comprometem a segurança das pessoas que recebem cuidados. Logo, há uma sensibilização prévia, ou seja, um entendimento sobre como eventos adversos afetam os processos de trabalho e a qualidade do cuidado cirúrgico. A análise de similitude comprova que esses termos se destacam nas notificações, sendo proeminentes e tendo relação com os termos que definem qual evento adverso está sendo relatado.

Vale ressaltar que, com a pandemia de covid-19, o ato da notificação voltou a ganhar notoriedade e ser discutido quanto a sua importância por permitir que sejam evidenciados o cenário epidemiológico e fatores associados com a ocorrência de doenças e agravos^(16)^.

Ademais, a qualidade da notificação, considerando o preenchimento completo e de maneira clara, deve ser um dos pontos a se trabalhar na criação de uma cultura institucional de notificação de eventos. Recomenda-se que as instituições, a partir da implantação da ficha de notificação de eventos, possam trabalhar este instrumento na educação permanente, treinando todos os profissionais para o preenchimento da ficha de notificação, esclarecendo sobre sua relevância e exercendo a avaliação e o monitoramento constante desta atividade pelos profissionais^(17,28)^.

Por fim, o desempenho do sistema de notificação brasileiro é um substancial fator de análise para a qualidade das notificações, isso vem sendo apontado em outros tipos de notificação, como a de farmacovigilância^(29)^. Logo, cabe propor melhorias para promoção da completude dos registros, fornecimento de mais dados que poderão ser utilizados para análise de causa-raiz e manejo correto do sistema pelos profissionais notificadores.

### Limitações do estudo

O estudo foi limitado pelo seu delineamento transversal e o acesso apenas aos registros documentados das notificações, sem ter sido realizada abordagem junto aos profissionais notificadores que poderiam apresentar sua visão sobre como compreendem os eventos notificados e o processo de registro no sistema. Por meio desta abordagem podem ser sugeridas melhorias e elaboradas estratégias de treinamento junto à equipe de saúde. Outro fator limitante foi a reduzida amostra de notificações detectadas. Isso limita a generalização dos dados para a população.

### Contribuições para a área da enfermagem, da saúde ou da política pública

O ato de notificar está intrinsecamente associado a uma cultura de segurança organizacional que dá subsídios aos profissionais por meio de medidas não punitivas, comunicação aberta, liderança presente e oferta de mecanismos próprios e de qualidade para a notificação. Os achados deste estudo indicam que a cultura de notificação de eventos adversos em centros cirúrgicos ainda sofre forte limitação representada pelo reduzido número de notificações identificadas, o que prejudica as análises e a gestão de riscos.

A principal contribuição dos resultados encontrados tem relação com a possibilidade de motivar gestores na construção de estratégias voltadas para o estímulo da notificação, do treinamento focado no ato de notificar e como fazê-lo com qualidade. É necessário que estudos adicionais proponham estratégias de treinamento e desenvolvam ferramentas que facilitem a notificação.

Além do mais, o desenvolvimento de sistemas que atendam pré-requisitos compatíveis com a necessidade dos serviços de saúde é uma demanda urgente. Definir um conjunto mínimo de variáveis obrigatórias que devem constar em todas as notificações e nas variáveis específicas para notificações cirúrgicas, como a checagem do uso do checklist de segurança cirúrgica, o tipo de anestesia utilizada no procedimento, quais medidas foram adotadas pela equipe cirúrgica ao detectar o evento adverso, quais as ações agravantes e atenuantes, que necessitam constar na notificação para que um panorama mais amplo seja traçado acerca da ocorrência do evento notificado.

## CONCLUSÕES

Lesões por pressão resultantes do posicionamento cirúrgico, queimaduras causadas pelo bisturi elétrico, lesão de órgão e infecção de sítio cirúrgico destacaram-se como os principais eventos adversos cirúrgicos notificados. Já os pacientes que mais sofreram eventos, eles tiveram danos classificados como leves, eram homens e estavam na faixa etária adulta de 18 a 65 anos. Os diagnósticos registrados na internação foram, principalmente, relacionados a doenças crônicas não-infecciosas ligadas aos aparelhos digestivo, circulatório, respiratório e neoplasias.

A prevalência de notificações cirúrgicas registradas em sistema, quando comparadas ao volume de notificações gerais e o número de cirurgias realizadas, aponta para um importante quadro de subnotificação dos incidentes que ocorrem durante a assistência cirúrgica. Logo, as notificações de eventos adversos apresentaram limitações em seus registros, como eventos adverso não especificado, dados não informados e descrição não preenchida por mais de um quarto das notificações analisadas. Além disso, há diferenças entre os relatos registrados, não havendo padronização na forma de relatar os eventos, que variaram entre descrições excessivamente breves a detalhadas.

Em suma, os resultados indicam quais eventos adversos notificados por centros cirúrgicos são mais prevalentes e, portanto, merecem atenção da equipe e serviços de saúde para a adoção de medidas específicas e protocolos clínicos para sua prevenção. Alguns eventos adversos identificados demonstram inconsistência na aplicação do checklist de segurança cirúrgica.

Por fim, é preciso pensar em estratégias de estímulo e de melhoria da notificação. Notificar mais e melhor deve ser a meta das instituições de saúde. Um modelo padrão de notificação deve ser pensado e replicado aos profissionais por meio de treinamento e processo contínuo de aprendizagem, para que então haja garantia de dados mínimos fornecidos pelas notificações. Com isso, será possível realizar investigações de eventos adversos que direcionam para os fatores associados e os pontos de melhoria necessários.

## Data Availability

https://doi.org/10.48331/scielodata.KUHHDE PDF
